# Genetic diversity and population structure of *Polygonatum cyrtonema* Hua in China using SSR markers

**DOI:** 10.1371/journal.pone.0290605

**Published:** 2023-08-31

**Authors:** Heng Liu, He Cheng, Jun Xu, Jiayi Hu, Chenchen Zhao, Lihua Xing, Mengjin Wang, Zhendong Wu, Daiyin Peng, Nianjun Yu, Junling Liu

**Affiliations:** 1 Department of Biopharmaceuticals, College of Pharmacy, Anhui University of Chinese Medicine, Hefei, Anhui Province, China; 2 MOE-Anhui Joint Collaborative Innovation Center for Quality Improvement of Anhui Genuine Chinese Medicinal Materials, Hefei, Anhui Province, China; 3 Anhui Qingyang County Jiuhua traditional Chinese Medicinal Materials Technology Co., Ltd, Chizhou City, Anhui Province, China; 4 Anhui Provincial Institutes for Food and Drug Control, Hefei, Anhui Province, China; Nuclear Science and Technology Research Institute, ISLAMIC REPUBLIC OF IRAN

## Abstract

*Polygonatum cyrtonema* Hua is a perennial herbaceous plant of the *Polygonatum* genus, belonging to the Liliaceae family, with significant medicinal and nutritional value. In China, this species is a traditional medicinal and edible herb with a long history of application and is widely appreciated by the people. However, as the demand for medicinal herbs continues to grow, excessive harvesting has led to the depletion of wild resources and the risk of genetic erosion. In addition, the chaotic cultivation of varieties and the lack of high quality germplasm resources have led to inconsistent quality of medical materials. Therefore, it is urgent to conduct genetic diversity evaluation of this species and establish a sound conservation plan. This study assessed the genetic diversity and population structure of 96 samples collected from seven regions in China using the simple sequence repeat (SSR) molecular marker technology. In this study, a total of 60 alleles (Na) were detected across the 10 polymorphic SSR markers used, with an average of 6.0 alleles generated per locus. The values of polymorphic information content (PIC) values ranged from 0.3396 to 0.8794, with an average value of 0.6430. The average value of the effective number of alleles (Ne) was 2.761, and the average value of the Shannon’s information index (I) was 1.196. The population structure analysis indicates that the *Polygonatum cyrtonema* Hua germplasm can be classified into three subpopulations (JZ, QY, JD) at the molecular level, which corresponds to the previous subgroups identified based on individual plant phenotypic traits. Analysis of Molecular Variance (AMOVA) showed that 74% of the genetic variation was between individuals within populations in different regions. The phylogenetic analysis of the 96 germplasm samples divided them into three main populations. The QY and JD subpopulations are largely clustered together, which could be attributed to their mountainous distribution and the local climate environment. The genetic differentiation coefficient (Fst) value was low at 0.065, indicating relatively low population differentiation. The ratio of the genetic differentiation coefficient (Fst) between the JZ population and the other two populations (QY and JD) is much higher than the ratio between the QY and JD populations. Based on the clustering results and the ratio of the genetic differentiation coefficient (Fst), it can be inferred that the genetic relationship between the QY and JD subpopulations is closer, with a certain degree of genetic differentiation from the JZ subpopulation. This study supports the conservation of germplasm resources of *Polygonatum cyrtonema* Hua in China and provides new parental material for germplasm genetic improvement and breeding programs.

## Introduction

There are more than 30 species of *Polygonatum* in China [1], which is the distribution center of *Polygonatum*. The *Polygonatum cyrtonema* Hua (*P*. *cyrtonema*) is widely distributed in the Yangtze River basin, and Anhui Province is one of the main production areas, of which Qingyang County is the Geo-Authentic Product Area.

According to the "Flora of Anhui" [2] the *P*. *cyrtonema* is distributed in Qimen, Shitai, Yixian, Xiuning, Shexian, Tongling, and Jinzhai, among others. These areas are all located in the southern and western mountainous regions of Anhui Province. Currently, the highest quality *P*. *cyrtonema* is produced in Jiuhua Mountain and its surrounding areas in Qingyang County, southern Anhui. The plant, with a medicinal history in China spanning over 2000 years, is recognized as one of the source plants for the medicinal herb rhizoma polygonati specified in the Pharmacopoeia of the People’s Republic of China [3]. With its diverse range of medicinal properties, this plant serves as a valuable medicinal herbal medicine, exhibiting remarkable effects in various areas. It showcases exceptional attributes such as anti-aging [4], immune modulation [5], blood lipid regulation [6], memory enhancement [7], anti-tumor [8], and antibacterial properties [9]. Moreover, it demonstrates immense potential for advancement in the realms of food and health products, positioning itself as a promising candidate for future development. At present, the species is a primary source of the herbal medicine Rhizoma Polygonati. A survey conducted in the Anhui market revealed that it is the most abundant variety, representing approximately 80% of the total resources available [10].

With the increased demand for the medicinal herbs of rhizoma polygonati and the investigation of its edible value, the related industry has seen rapidly increased growth [11]. Related food and health products are in demand in the market, and large-scale artificial cultivation of this plant is booming [12]. However, cultivated germplasm tends to be mixed and lacks superior traits, leading to uneven quality of medicinal herbs. The sources of germplasm resources include local wild species, cultivated species, and foreign germplasm. And breeding methods include seed breeding, rhizome breeding, and tissue culture, among others. Through field investigations, it has been found that due to uncontrolled harvesting, the wild *P*. *cyrtonema* has gradually become scarce, posing a risk of genetic erosion. Therefore, it is essential to evaluate the genetic diversity and kinship among populations to provide valuable references for the establishment of conservation programs.

Wild germplasm resources of the species are mainly found in the Dabie Mountain system in western Anhui, and the Jiuhua Mountain system and the Huangshan Mountain system in southern Anhui. The variations in plant traits of *P*. *cyrtonema* are closely associated with the geographical areas [13]. Through field surveys, it has been discovered that there are differences in plant traits such as rhizome shape, leaf shape, and pedicel length between populations in the Dabie Mountain region of western Anhui and the mountainous area of southern Anhui. Based on the differences in plant morphology, they can be divided into three main subspecies populations: the Dabie Mountain population (JZ) and the mountainous area of southern Anhui populations (QY and JD). Both Dabie Mountain and South Anhui Mountain are highly suitable areas for the species. The main ecological factors causing the difference in plant morphology between the two regions are eight factors such as rainfall in February and March, soil type, vegetation type and slope orientation [14]. The phenotypic variation of organisms is the result of different expression of genes under the influence of environmental factors. In this study, we further investigated the genetic variation of *P*. *cyrtonema* at the molecular level to validate the previous research on phenotypic traits and establish a correlation. The objective is to establish a solid foundation for the conservation of resources, breeding programs, and industrial development of this species.

As a result of gene interactions, multiple genotypes interact in a similar manner, giving rise to similar phenotypic phenomena [15, 16]. Some mutations in alleles do not lead to changes in phenotype and can also result in a reduction in phenotypic polymorphism [17]. Additionally, external environmental factors can have an impact on gene expression. In certain cases, environmental factors can exert similar effects on individuals with different genotypes, thereby reducing phenotypic polymorphism [18]. Therefore, in studies of plant morphological diversity, phenotypic polymorphism is often relatively low [19, 20], which in turn affects the accuracy of plant clustering using this approach. In contrast, molecular marker techniques have high polymorphism and therefore are increasingly common in the study of genetic diversity of germplasm resources [21, 22]. Molecular marker techniques can accurately identify and detect subtle genetic variations at the genomic level, thereby revealing hidden phenotypic diversity and allowing correlation with specific phenotypic traits [23, 24]. By selecting suitable parents, we can increase the genetic diversity of the gene pool and promote the generation and maintenance of phenotypic polymorphism, overcoming the challenge of relatively low phenotypic polymorphism [25]. At present, molecular marker technology has been widely and deeply applied in the fields of plant taxonomic identification [26–28], phylogeny[29], genetic diversity analysis [30–32] and breeding [33, 34]. Tamura [35] was the first to apply DNA barcoding techniques to the taxonomic study of the *Polygonatum*, and since then, molecular biology techniques such as RAPD [36], SRAP [37], SCoT [38], ISSR [39, 40] and SSR have been widely used. Among them, the more applied ISSR techniques are mostly used among different species [41, 42], while SSR techniques are more suitable for the study of the same species or closely related species. The germplasm utilized in this study exclusively consisted of *P*. *cyrtonema*, a study has confirmed the viability of employing transcriptome data from this species for the development of SSR markers [43].

Simple Sequence Repeats (SSR), also known as Microsatellites, are a class of short sequence repeat units that exist widely in the genome. SSRs exhibit high variability and genetic polymorphism in the genome. They are analyzed by PCR amplification of the SSR region in the target sequence and subsequent analysis of the size differences in the amplified products. SSR are favored for their advantages of co-dominant inheritance, abundance in the genome, high polymorphism, high repeatability among closely related species, and transferability [44]. SSR markers have been preferred in genetic characterization studies of different species, such as garlic [45], perilla [46], ginger [47].Molecular marker technologies such as SSR and SRAP have been applied to genetic diversity evaluation [48], DNA fingerprinting [49], Analysis of transcriptomic loci and development of molecular markers [50].

Therefore, in this study, the SSR molecular marker technology was selected for optimizing the analysis of genetic diversity in *P*. *cyrtonema* germplasm resources. The objectives are to elucidate the kinship and population genetic structure of germplasm resources from different sources and evaluate the diversity of germplasm resources more effectively. To be more specific, based on previous studies on traits, this research aims to provide genetic background information and novel materials for breeding high-quality germplasm resources with stable agronomic traits, high yield, and strong stress resistance. In summary, this study provides fundamental data for the conservation and utilization of the genetic resources of this species, offering valuable references for future resource management, conservation, and molecular marker-assisted breeding.

## Materials and methods

### Plant material and DNA extraction

In this study, 96 samples were collected from Jinzhai County, Huoshan County, Qingyang County, Qimen County, Shitai County, Huangshan City, and Jingde County areas of Anhui Province, which basically covered the main distribution areas of *P*. *cyrtonema*. The original plant was identified by Prof. Nianjun Yu and Prof. Helin Liu of Anhui University of Traditional Chinese Medicine as *P*. *cyrtonema*. More than 10 individuals were collected from each area (the interval between individuals was not less than 10m). Fresh young leaves were collected, dried with silica gel, and stored in an ultra-low temperature refrigerator at -70°C for backup. A voucher specimen of this plant was stored in the Center of Herbarium, Anhui University of Chinese Medicine, Hefei, China (AhtcmH, yxy.ahtcm.edu.cn/info/1006/6713.htm, ynj2005288@sina.com, under the voucher number 20210416). The sample collection information is shown in (Table 1).

**Table 1:** Information on 96 *Polygonatum cyrtonema* Hua germplasm collections from seven regions.

group	area	ID	Phenotypic traits
JZ	Jinzhai	DH1-16	The plant is tall overall, with a yellowish, plump, cylindrical, or ginger-shaped rootstock. The total pedicel is long, often more than twice as long as the pedicel, with large stem scars, which often account for more than half of the rhizome, and more winged buds.
	Huoshan	DH17-32	
QY	Qingyang	DH33-48	Plant stems mostly green, rhizomes yellowish, plump, mainly tessellated, with small stem scars.
	Shitai	DH49-64	
JD	Jingde	DH65-81	The plants are relatively small, with checkerboard-shaped rhizomes and more than two to three shoots per year.
	Huangshan	DH82-90	
	Qimen	DH91-96	

The 96 germplasm were divided into three subgroups (JZ, QY, JD) based on plant traits. The sampling site of JZ group is in the Dabie Mountain region in western Anhui, and the sampling sites of QY and JD group are in the Jiuhua Mountain system and the Huangshan Mountain system in southern Anhui, respectively.

The cetyltrimethylammonium bromide (CTAB) method, commonly used for extracting plant genomic DNA, yields high-quality and high-purity DNA suitable for molecular biology research, molecular marker analysis, gene cloning, and other applications [51]. The method mainly involves cell lysis, protein removal, DNA precipitation, DNA washing, and other steps. Therefore, in this study, the CTAB method [52, 53] was employed to extract total genomic DNA from leaf tissues. Using a 1% agarose gel, electrophoresis was performed at a constant voltage of 120 V. After approximately 30 minutes, when the DNA fragments had migrated approximately 2/3 of the way through the gel, the gel was removed and placed in an imaging analysis system for photography to assess the quality and concentration of the extracted DNA. The concentration was then adjusted to 50 ng/μL and stored at -20°C for further use.

### Primer screening and SSR-PCR amplification

Based on the relevant studies, 50 pairs of primers were screened in this paper [37, 39, 40, 42, 43, 54–58], and the primers were synthesized by Biotech Bioengineering (Shanghai) Co. A total of 14 samples were randomly selected from 2 samples in each of the 7 sample collection areas for PCR amplification pre-experiments. Different loci can be selected for pre-experiments for different samples to ensure coverage of all loci. 3 μL of amplification product was taken for electrophoresis to identify whether the PCR conditions were single and whether the fragment size was as expected.Bands with a single and consistent size can enter the experiment, and the concentration can be adjusted by adjusting the number of amplification cycles. The PCR reaction system consisted of 1 μL of template DNA, 0.5 μL each of forward and reverse primers (10 μmol.L^-1^), 5 μL of MIX enzyme(2×M5 HiPer plus Taq HiFi PCR mix (with blue dye)), and ddH_2_O added to achieve a total system volume of 10 μL. The amplification program included an initial denaturation step at 96°C for 3 minutes, followed by denaturation at 94°C for 1 minute, annealing at 56°C for 1 minute, extension at 72°C for 1 minute. This was repeated for a total of 20 cycles. After the cycling, there was a final extension step at 60°C for 10 minutes, followed by storage at 12°C. The amplified product was stored in a -20°C refrigerator.

The Fragment Analyzer^TM^ is a highly integrated and fully automated capillary electrophoresis system used for the analysis and quantification of biological molecules such as DNA, RNA, and proteins. It utilizes microfluidic technology and proprietary analysis software to measure the size, concentration, and quality of target molecules rapidly and accurately in the sample. The instrument is equipped with a proprietary analysis software called Prosize^TM^2.0, which allows for real-time monitoring and analysis of the electrophoretic data from the samples. It can generate high-quality electrophoresis profiles, quality scores, and quantitative data, and provides visual representation of the data. Therefore, in this study, the Fragment Analyzer^TM^ automatic capillary electrophoresis system was used for sequencing, and the Prosize^TM^2.0 software was applied to score the polymorphic DNA bands in the electrophoresis profiles [59].

The fragment length, effective allele number (Ne), major allele frequency (MAF), observed heterozygosity (Ho), expected heterozygosity (He) and polymorphic information content (PIC) were calculated using Power Marker software [60, 61], and finally 10 pairs of SSR primers with high polymorphism, clear bands and good reproducibility were selected for the population genetic study of *P*. *cyrtonema*, and the primer details are shown in (Table 2).

**Table 2:** Information of 10 pairs of polymorphic SSR primers screened.

primer	Forward Primer Sequences	Reverse Primer Sequences	Repeat motifs
DH11	GTCCGAGTTCTTTGACGAGC	AAAACCATCTCCATCCTCCC	(TG)_10_(AG)_6_
DH15	CGAAGAAGTCTCGATCCACC	TCGAGGAGTGCTTGATGATG	(TAACCC)_4_
DH18	TCTGTAAATCAGGAGGAGCGA	AATCGATGAGATCGACCTGG	(CT)_12_
DH19	CGAAACCCTCCTCAATCTCA	GCGACAAGTGTTGAAGGGTT	(ACC)_9_
DH21	CCAACAAGGGAAAAGCAAAG	AATATGGGGGTGACTCCGTT	(AGAA)_6_
DH24	ACCTAAAAGCCTCCAGCGAT	AGGAGGAGGAGGATAGGGGT	(CCGTAC)_4_
DH26	ACCCTCTCAATCGTCACCTG	TCAATCAATTCCCCTTCAGC	(CTG)_8_
DH34	GCTCGCAACAACAAACAAAA	GGGCTGAGAATTGAGAACCA	(TTAA)_6_
DH38	TGACCCTCCACTTCATCTCC	GACTCCTTCGAGTGGTACGC	(CGG)_8_
DH40	TACTTTCTCCCCGTTTCCCT	GCTCCCCATCTCCAGAAATA	(ACCG)_6_

### Data analysis

Genetic diversity was assessed using the allelic binary data set obtained from the ten polymorphic SSR markers. GenAlEx V.6.5 is a commonly used genetic data analysis software that provides a range of analysis tools and statistical methods for handling various types of genetic data, including microsatellite (SSR) markers, nucleotide sequence data, SNP data, and more. The Shannon’s information index (I) and the number of effective alleles(He) were determined with GenAlEx V.6.5 software [62]. PowerMarker is a software that encompasses various functionalities for genetic diversity analysis, genetic structure analysis, and more. Some of its features include calculating allele count, heterozygosity, performing AMOVA analysis, and conducting principal component analysis (PCA). The PowerMarker V3.25 software [61] was used to calculate indices of allele number (Na), expected heterozygosity (Ho), observed heterozygosity (He), major allele frequency (MAF) and polymorphic information content (PIC) per microsatellite locus, respectively. Analysis of molecular variance (AMOVA) and principal coordinate analysis (PCoA) were performed using GenALEx V.6.5 [62] to calculate genetic differentiation coefficients Fst and Φst [63] and to infer whether there was gene exchange and genetic differentiation between populations.

NTSYS-PC 2.1 is a software used for phylogenetic analysis and classification studies based on genetic marker data. In this study, the NJ phylogenetic tree was constructed using this software. The genetic structure of 96 germplasm was then analysed using STRUCTURE software [64] based on the Bayesian clustering algorithm. For the calculation, the grouping (K) was set according to the number of populations sampled [65, 66]. Due to the presence of gene flow and admixture among the samples in this study, we have opted to utilize the Admixture ancestry model and the independent allele frequency model. These models allow for variation and mixing in both genetic composition and allele frequencies across populations. This choice will facilitate a more accurate inference of the genetic relationships between populations [67–69]. After the operation is completed, the calculation results are uploaded to the STRUCTURE HARVESTER (http://taylor0.biology.ucla.edu/structureHarvester/) website [70]. The best segmentation K value is judged based on the LnP(D) value corresponding to the K value and the optimal partitioning K-value. It refers to the value of K that corresponds to the maximum LnP(D) and the relative maximum ΔK. It represents the best fitting number of populations for a given dataset, helping to reveal the genetic relationships and structure among populations, and providing a foundation for subsequent analysis and further research [68, 71].

## Results

### Genetic diversity estimated with SSR markers

A total of 10 pairs of SSR primers with good polymorphism were screened from 50 pairs of primers, and the detection information is shown in (Table 3). Based on the set frequency threshold, the PowerMarker software counts the frequency of each allele at each locus to calculate the number of alleles [61]. These 10 primer pairs detected a total of 60 alleles in 96 samples, with the number of alleles per locus (Na) ranging from 3.000 to 8.000, with a mean value of 6.000. The primer with the highest number of alleles was DH24 and the primer with the lowest number of alleles was DH11. The polymorphic information content (PIC) generated ranged from 0.3396 to 0.8794, with a mean value of 0.6430. The allele frequency (MAF) ranged from 0.1823–0.7083 with a mean value of 0.4370. The expected heterozygosity (He) ranged from 0.440 to 0.771, with a mean value of 0.604. The observed heterozygosity (Ho) ranged from 0.083 to 0.906, with a mean value of 0.458. The mean intrapopulation inbreeding coefficient (Fis) was 0.198. According to the relevant studies that have been reported [72], it is believed that all 10 pairs of SSR primer marker loci are highly polymorphic and possess abundant genetic diversity, making them suitable for genetic research on the *P*. *cyrtonema*.

**Table 3:** Detection information of 10 pairs of polymorphic SSR primers.

maker	Na	Ne	I	Ho	He	uHe	MAF	PIC	Fis
DH11	3.000	2.027	0.740	0.906	0.507	0.509	0.7083	0.3396	-0.792
DH15	7.000	3.805	1.491	0.740	0.737	0.741	0.4323	0.6316	-0.068
DH19	6.000	3.367	1.432	0.375	0.703	0.707	0.2813	0.7781	0.416
DH21	7.000	2.185	1.114	0.427	0.542	0.545	0.2344	0.8276	0.178
DH24	8.000	4.358	1.674	0.427	0.771	0.775	0.4063	0.6249	0.356
DH26	6.000	1.784	0.896	0.083	0.440	0.442	0.7083	0.4523	0.807
DH34	7.000	1.908	0.999	0.271	0.476	0.478	0.4323	0.6823	0.429
DH38	6.000	2.954	1.377	0.219	0.662	0.665	0.3750	0.7636	0.654
DH40	4.000	2.122	0.903	0.510	0.529	0.532	0.6094	0.4507	-0.041
DH18	6.000	3.102	1.335	0.625	0.678	0.681	0.1823	0.8794	0.038
Mean	6.000	2.761	1.196	0.458	0.604	0.607	0.4370	0.6430	0.198

Na(number of alleles), Ne(number of effective alleles), Ho(observed heterozygosity), I(Shannon’s information index), He(expected heterozygosity), uHe(unbiased expected heterozygosity), MAF(major allele frequency), PIC(Polymorphism information content), Fis(Intraspecific inbreeding coefficient)

### Diversity of *Polygonatum cyrtonema* Hua populations

**Fig 1:**
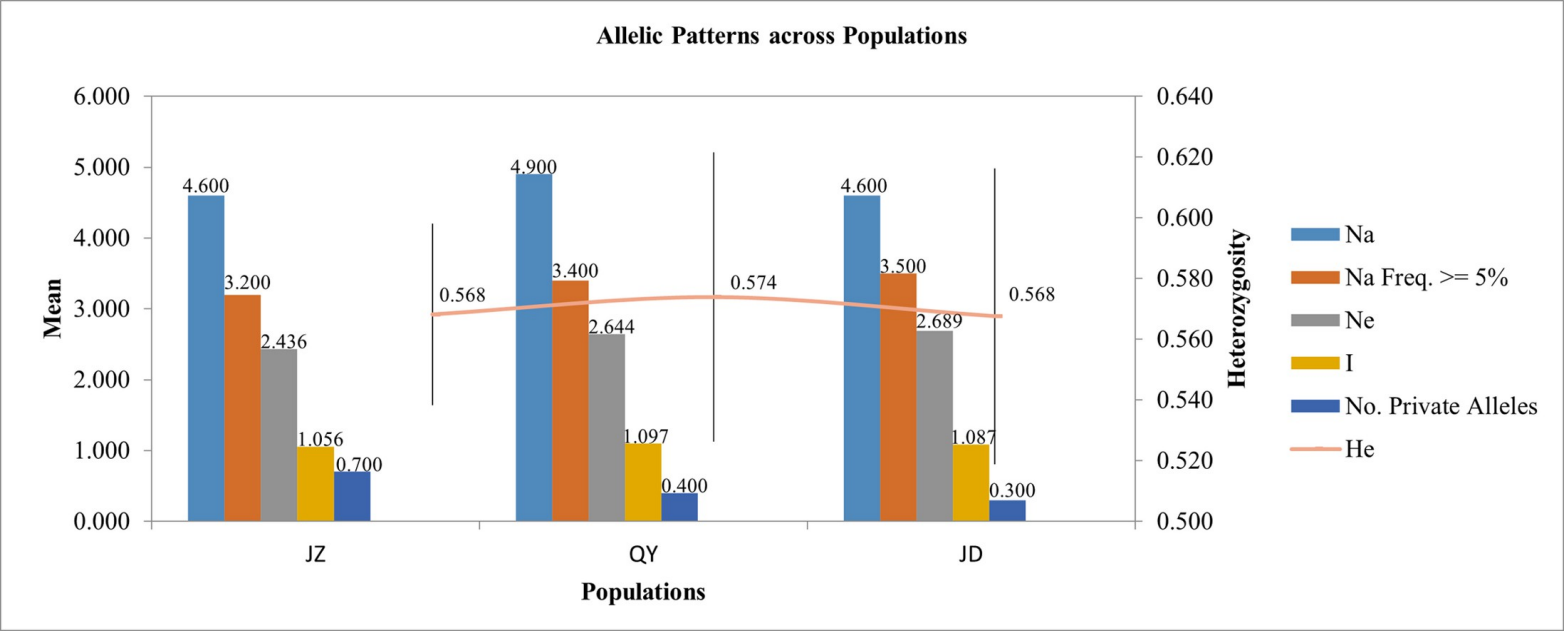
Diversity analysis of populations based on 10 pairs of SSR primers. Na = No. of Different Alleles Na (Freq ≥5%) = No. of Different Alleles with a Frequency≥5% Ne = No. of Effective Alleles = 1 / (Sum pi^2) I = Shannon’s Information Index = -1* Sum (pi * Ln (pi)) No. Private Alleles = No. of Alleles Unique to a Single Population He = Expected Heterozygosity = 1—Sum pi^2 .

Based on the geographical regions and plant morphology, the *P*. *cyrtonema* from seven regions were initially divided into three populations (JZ, JD, QY), and the data of genetic diversity analysis by SSR amplification are shown in (Table 4 and Fig 1). The mean number of alleles (Na) for the three populations (JZ, QY, JD) were 4.6, 4.9, and 4.6, respectively, and the mean effective allele numbers (Ne) were 2.436, 2.644, and 2.689, respectively. The Shannon’s Information Index (I) was 1.056, 1.097, and 1.096, respectively. Both the number of alleles (Na) and the Shannon’s Information Index (I) of the QY population were the highest, indicating that the QY population was more diverse. The JZ population had the lowest number of alleles (Na) and Shannon’s Information Index (I), indicating that the germplasm of the JZ population was more purified. The number of valid alleles in the JD population was closest to the number of alleles measured, indicating that the alleles were relatively evenly distributed in this population.

**Table 4:** Data table of genetic diversity analysis of three populations.

group	Na	Ne	I	Ho	He	uHe
JZ	4.600	2.436	1.056	0.428	0.568	0.577
QY	4.900	2.644	1.097	0.466	0.574	0.583
JD	4.600	2.689	1.087	0.481	0.568	0.577
Mean	4.700	2.589	1.080	0.458	0.570	0.579

JZ group: populations in Jinzhai and Huoshan areas, QY group: populations in Qingyang and Shitai areas, JD group: populations in Qimen, Huangshan, Jingde areas.

The mean observed heterozygosity (Ho) and mean expected heterozygosity (He) of the 96 germplasm were 0.458 and 0.570, respectively. The mean observed heterozygosity (Ho) was 0.428, 0.66, and 0.481 for the JZ, QY, and JD populations, respectively, and the mean expected heterozygosity (He) was 0.568, 0.574, and 0.568, respectively. The QY population had the highest observed heterozygosity and expected heterozygosity, and all three populations had lower observed heterozygosity than expected heterozygosity, indicating low intra-population gene exchange.

**Genetic differentiation between population and principal coordinate analysis.** Analysis of Molecular Variance (AMOVA) showed a highly significant difference (P<0.001), the results of AMOVA analysis are shown in (Table 5). The proportion of genetic variation between populations within regions was the lowest at 4%, the genetic variation between individuals within populations was the greatest (74%), and genetic variation between regions accounted for 20% of the total genetic variation. The results indicated that the diversity within regions was far greater than the diversity between regions. The genetic differentiation coefficient (Fst) of populations within the region is 0.065, and the gene flow (Nm) is 3.584. This indicates that there is a relatively low level of genetic differentiation among subgroups, while there is a higher level of genetic differentiation within subgroups (Table 6).

**Table 5:** Summary of AMOVA partitioning of *Polygonatum cyrtonema* Hua populations based on regions.

Source variation	df	SS	MS	Est. Var.	%
Among populations within regions	2	32.964	16.482	0.203	7%
Among regions	93	327.047	3.517	0.612	20%
Within population	96	220.000	2.292	2.292	74%
Total	191	580.010		3.107	100%

df = degree of freedom, SS = sum of squares, MS = mean squares, Est. var. = estimate of variance, % = percentage of total variation based on 999 permutations

**Table 6:** F-Statistics analysis of *Polygonatum cyrtonema* Hua populations in different regions based on allelic distance matrix.

F-Statistics	Value	P (rand ≥ data)
Fst	0.065	0.001
Fis	0.211	0.001
Fit	0.262	0.001
Nm	3.584	

Probability, P (rand ≥ data), for Fst, Fis and Fit is based on standard permutation across the full data set. Fst = AP / (WI + AI + AP) = AP / TOT Fis = AI / (WI + AI) Fit = (AI + AP) / (WI + AI + AP) = (AI + AP) / TOT Nm = [(1 / Fst) - 1] / 4

AP = Est. Var. Among Pops, AI = Est. Var. Among Individuals, WI = Est. Var. Within Individuals

**Fig 2:**
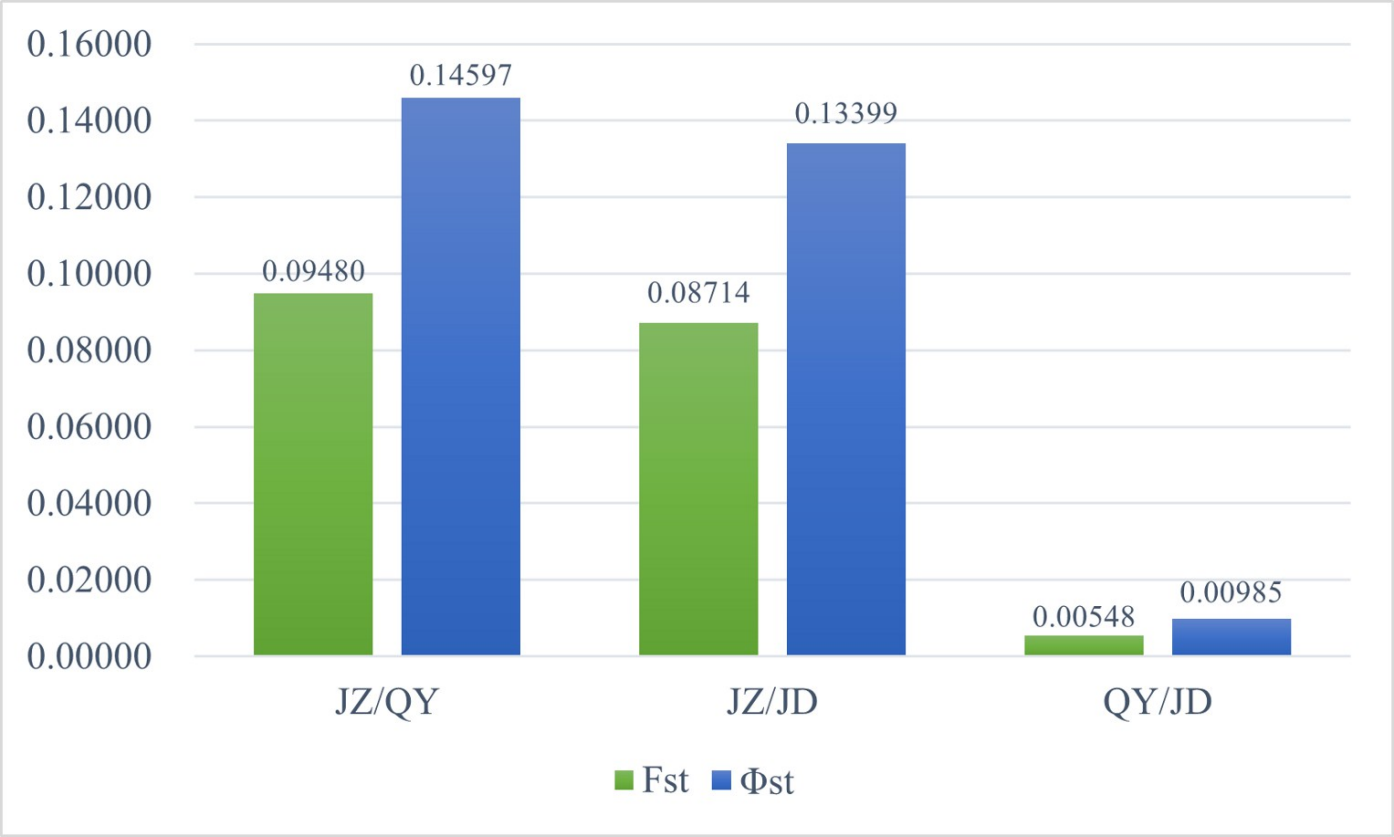
Ratio of Fst values and Φst values between each two populations. Fst: Genetic differentiation values based on allele frequency. Φst: Genetic differentiation values based on genetic distance.

**Fig 3:**
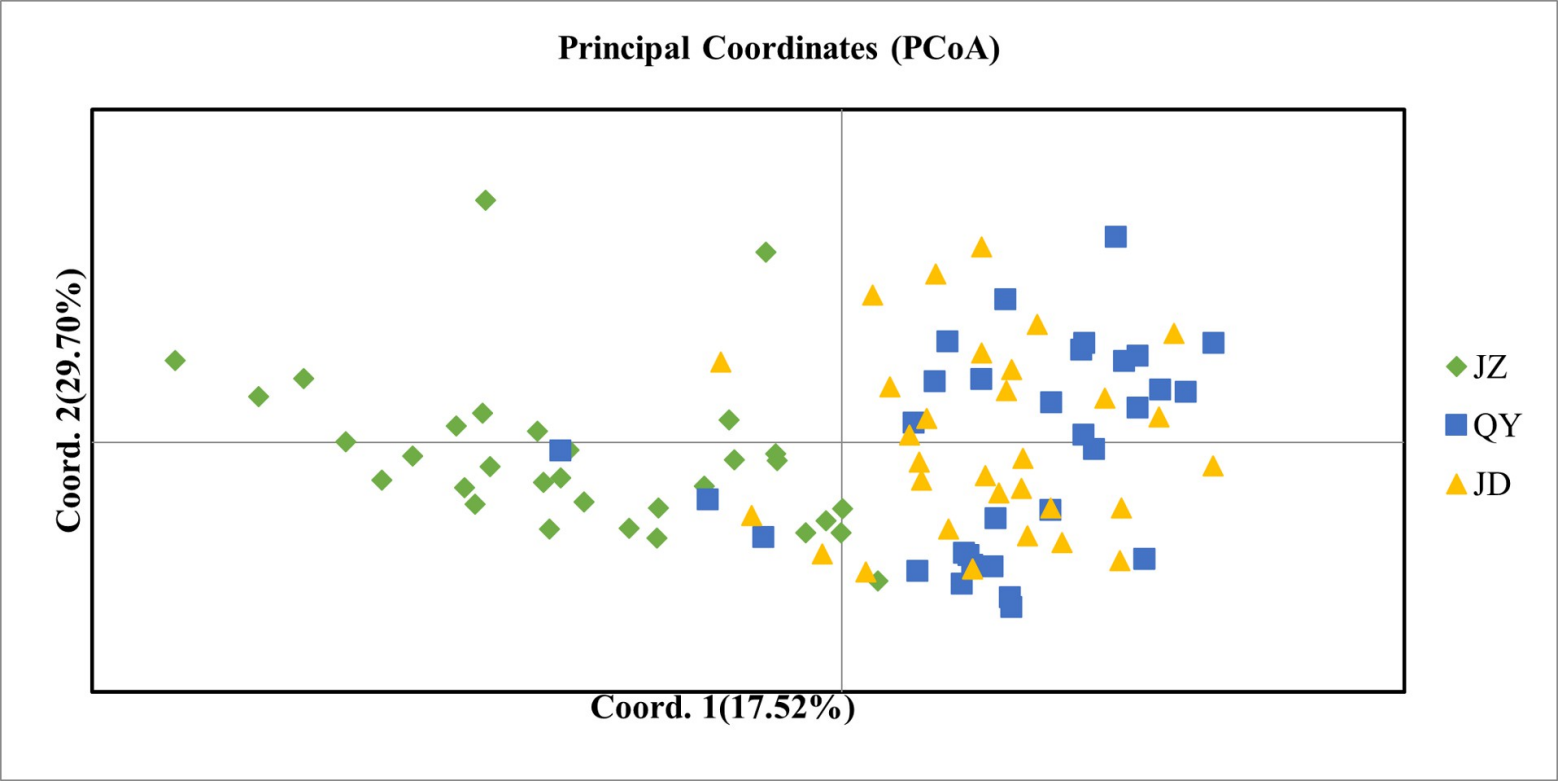
SSR-based principal coordinates analysis of *Polygonatum cyrtonema* Hua populations.

Fst values are used to calculate genetic differentiation among populations by means of allele frequencies, and Φst is a measure of genetic differentiation among populations using genetic distance. The values of Φst and Fst in the (Fig 2) show that there is a certain degree of genetic differentiation between the JZ population and the QY, JD populations, while there is no obvious genetic differentiation between the QY and JD populations. The kinship of the three populations of different origins was examined using principal coordinate analysis based on genetic distance matrix (Fig 3). The QY and JD populations are closely related and cannot be classified into two categories, while the JZ population is largely separable from the QY and JD populations and can be classified into a separate category. The contribution of the first principal coordinate and the second principal coordinate to the total genetic variation was 17.52% and 29.70%, respectively.

### Population structure analysis

**Fig 4:**
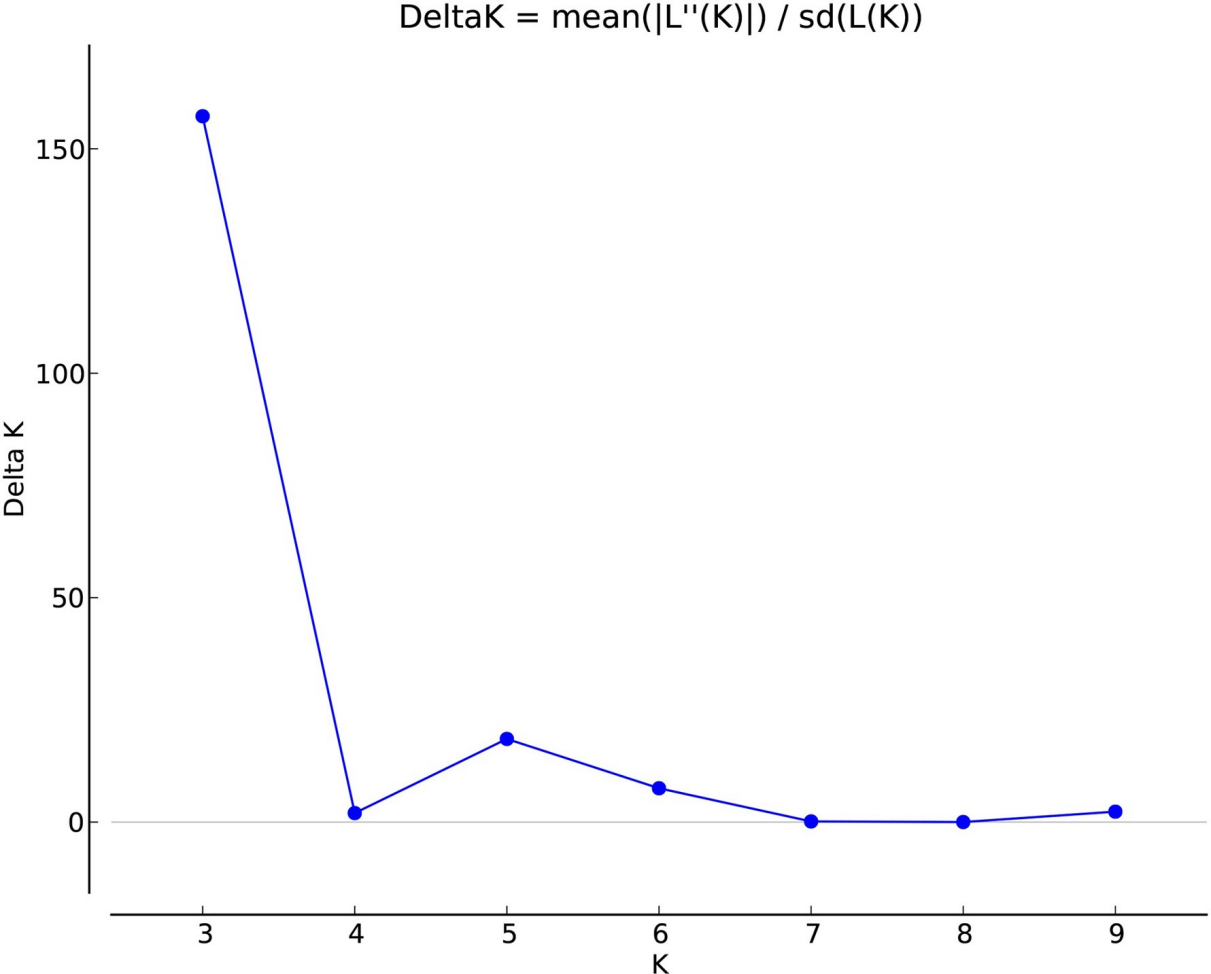
Determinations of subpopulations (K) of *Polygonatum cyrtonema* Hua population. The maximum of ad hoc measure ΔK determined by structure harvester was found to be K = 3, which indicated that these 96 germplasms could be grouped into two subgroups.

**Fig 5:**
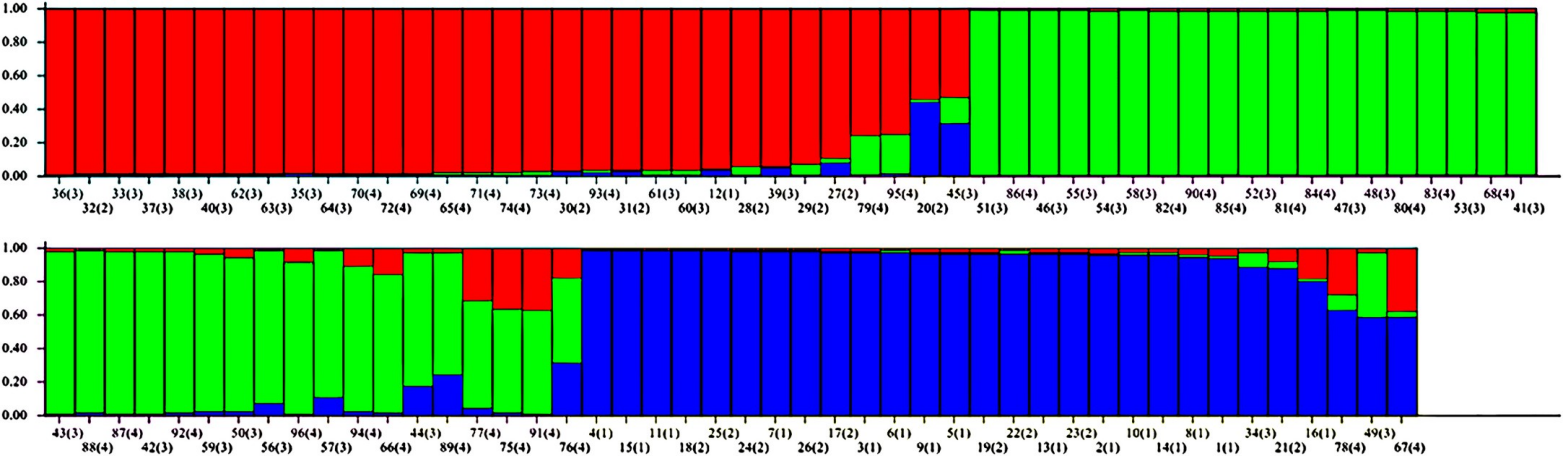
Population structure of 96 *Polygonatum cyrtonema* Hua accessions (K = 3).

The STRUCTURE software has the advantages of powerful statistical analysis, diversity analysis, visualization capabilities, data interpretation, and wide applicability. It is a commonly used tool in population genetic structure analysis. The Bayesian clustering method of STRUCTURE software was applied to analyze the genetic structure of 96 germplasm of three populations of *P*. *cyrtonema*, and the optimal grouping K values were determined by the posterior probability LnP(D) and ΔK maximum, setting the population size K as 2 to 10. The results showed that ΔK was maximum when K = 3 (Fig 4), indicating that the 96 germplasm could be divided into three subgroups. The three gene pools of red, blue and green were distributed in the JZ, QY and JD populations (Fig 5). According to the grouping results of STRUCTURE software, where red gene pools represent QY population, green gene pools represent JD population, and blue gene pools represent JZ population. The results of STRUCTURE analysis were the same as the clustering results of the study on the diversity of morphological characteristics of *P*. *cyrtonema*.

### Cluster analysis of *Polygonatum cyrtonema* Hua populations

**Fig 6:**
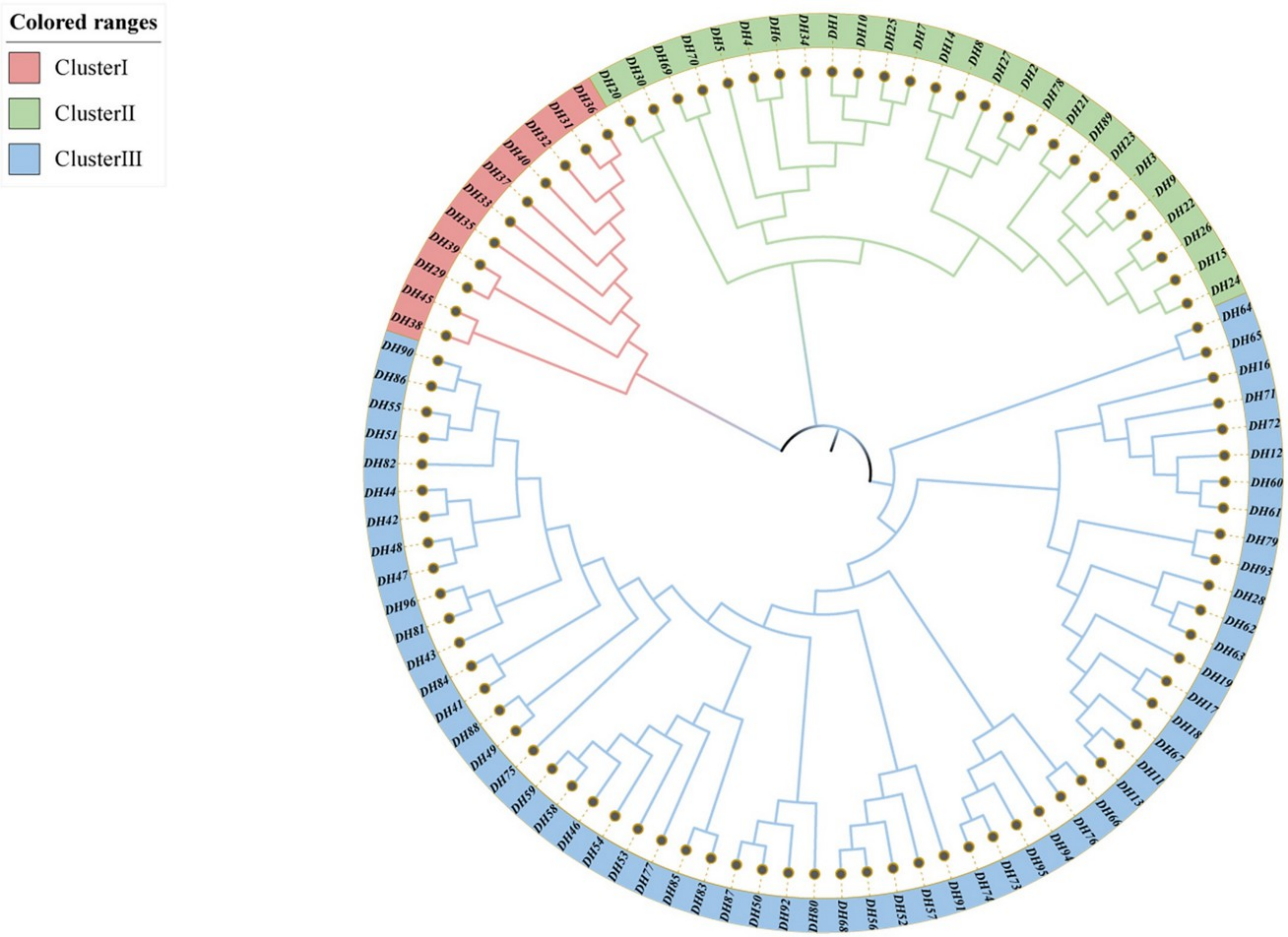
NJ Phylogenetic relationships among 96 *Polygonatum cyrtonema* Hua germplasm constructed based on Nei’s genetic distance. (Cluster I, red; Cluster II, green; Cluster III, blue).

The Neighbor-Joining (NJ) method is a clustering method based on a distance matrix used for constructing phylogenetic trees and inferring evolutionary relationships among species. It has the advantages of being simple, intuitive, computationally efficient, and widely applicable. In this study, an NJ evolutionary tree of 96 *P*. *cyrtonema* germplasm was constructed based on the genetic distance of Nei’s 1983, and three distinct clusters (clusters I, II, and III) were identified (Fig 6). Cluster I included eight germplasm of QY population and three germplasm of JZ population (DH29, DH31, DH32). Cluster II was mainly JZ population germplasm, but one QY population germplasm (DH34) and four JD population germplasm (DH69, DH70, DH78, DH89) were mixed in. This indicates that the three populations are not completely separated from each other according to the geographical distribution area of the germplasm collection, and there is some gene flow among the populations, and some germplasm relatives are close to each other. Cluster III consisted of all QY and JD germplasm except for one JZ population germplasm (DH16), indicating that the QY and JD populations were closely related and most of the germplasm was clustered into one group, while it was largely able to be clustered into different clusters from the JZ population.

## Discussion

### Method optimization

The present study employed fluorescence-labeled capillary electrophoresis technique for detection and analysis, which offers high sensitivity, high resolution, flexibility, and wide applicability. This technique allows for multi-color labeling, multi-channel analysis, and real-time monitoring [73, 74]. In contrast, traditional polyacrylamide gel electrophoresis and silver staining methods are complex to operate, time-consuming, and exhibit poor reproducibility in staining. Additionally, they have limited sensitivity and cannot accurately perform quantitative analysis [75, 76].

Fluorescence labeled capillary electrophoresis technology does not require gel making and silver staining, which saves time and effort and realizes automated data processing and reduces human errors. The size of amplified fragments can be accurately calculated, and data from different batches of reactions can be processed uniformly. High-throughput detection of samples and high-throughput detection of loci can be performed. Comparative studies on the detection of gene mutation sites and SSR sites have also demonstrated that capillary electrophoresis technique is superior [77, 78]. In general, fluorescence-labeled capillary electrophoresis is characterized by high throughput, high efficiency, high precision and good reproducibility [79, 80].

### SSR marker polymorphism analysis

In this study, we collected a total of 50 primer pairs from the previously published studies on *P*. *cyrtonema* and closely related species. And 10 primer pairs with high polymorphism were screened from the collected primers. The 10 primer pairs produced major allele frequencies(MAF) ranging from 0.1823 to 0.7083 with a mean value of 0.4370 on the 96 germplasm polymorphisms studied. A genetic differentiation analysis study on six species of *Polygonatum* based on 49 SSR markers found that the major allele frequencies (MAF) ranged from 0.40 to 0.98, with a mean value of 0.77 [48]. This result is higher than the findings of this study (MAF = 0.4370). The widespread distribution of germplasm resources, the abundant gene pool of populations, and the utilization of a substantial number of newly developed SSR markers may be the factors that have led to this outcome. The samples used in their study were obtained from 14 provinces across China, demonstrating a broad geographical distribution. Diverse environmental conditions and ecological system characteristics exist in different geographical regions, leading to genetic variations in species as they adapt to varying environmental conditions and respond to different selection pressures [81, 82]. Adaptive differences may lead to different genetic variation within different geographic regions, thereby increasing overall genetic diversity. Furthermore, geographic isolation can also restrict gene flow between species, leading to relatively independent evolution of populations in different geographical regions [83]. This has led to genetic differentiation and diversity of species within geographic regions. However, geographic diversity can also decrease the gene flow between different populations, affecting population size and distribution, thereby reducing genetic differentiation and diversity. It has been shown that geographic polymorphism is positively correlated with genetic diversity overall [84].

In this study, the number of alleles detected per core primer pair was 3.000–8.000, the average number of alleles (Na) and effective alleles (Ne) were 6.000, 2.761, respectively, and the polymorphic information content (PIC) ranged from 0.3396–0.8794 with a mean value of 0.6430. Some scholars used 12 primer pairs for SSR polymorphic locus analysis of 32 wild *Polygonatum sibiricum* Red. germplasm materials. The average number of alleles (Na) was 1.9843, the average effective allele number (Ne) was 1.5278, and the polymorphic information content (PIC) was 0.46 [85]. The small range of screening from 24 primer pairs and the fact that the germplasm resources were all wild origin may be the reason why the number of alleles (Na), the number of effective alleles (Ne), and the polymorphism information content (PIC) were lower than the results of this study. In addition, the results of an existing study (Na = 4.3061 PIC = 0.3069) [48] were lower than the results of the present study, probably due to the difference in the study material of six species of *Polygonatum*. In contrast, a related study using only *P*. *cyrtonema* as the study material reported that the number of alleles (Na) ranged from 2–11 with a mean value of 5.916, and the polymorphism information content (PIC) ranged from 0.232–0.851 with a mean value of 0.585 [55], which is basically consistent with the results of the present study. Our study showed that the expected heterozygosity (He) of SSR markers ranged from 0.440–0.771 with a mean value of 0.604, and the observed heterozygosity (Ho) ranged from 0.083–0.906 with a mean value of 0.458. Both were lower than those reported in similar studies (He = 0.772, Ho = 0.563) [56], and the expected heterozygosity obtained by Tinghui Feng (He = 0.887) [49] was significantly higher than that of the present study. These differences may be attributed to our use of *P*. *cyrtonema* germplasm from similar geographic regions, different primer types, and the amount of germplasm we used. And the results of Chen (He = 0.619 Ho = 0.458) [55] were closer to the present study. The Shannon’s Information Index (I) detected in this study ranged from 0.740 to 1.674, with a mean value of 1.196. This value is higher than the value reported by Wang (I = 0.4788) [85], which may be due to the lower number of germplasm and low population uncertainty, so the Shannon’s Information Index (I) is lower.

### Genetic relationship and population structure analysis among populations

The higher values of QY allele (Na), effective allele (Ne) and Shannon’s Information Index (I) in the three populations (JZ, QY and JD) indicate that the QY population has the highest genetic diversity and more complex genetic background. It may be caused by the fact that Qingyang is the main cultivation area with many exotic germplasm cultivars. The expected heterozygosity (He) was higher than the observed heterozygosity (Ho) in all three populations, suggesting a low intra-population gene exchange. Low levels of gene flow can lead to increased genetic drift, resulting in the loss of beneficial genes, accumulation of harmful genes, and reduced genetic diversity [86]. Additionally, it may result in gene loss within the species, leading to decreased adaptive and repair capabilities [87]. A low genetic differentiation coefficient (Fst = 0.065) and a high gene flow (Nm = 3.584) also indicate that there is a certain degree of gene exchange between populations and a relatively low level of genetic differentiation. To some extent, low levels of genetic differentiation can promote gene flow and reduce genetic divergence. Clearly, there seems to be a contradiction here. In the case of having a high gene flow value (Nm = 3.5840), the levels of gene exchange and genetic differentiation between populations are not high. The gene flow Nm value is an important indicator used to estimate gene exchange between populations, but it is not the sole determinant of gene flow between populations. Therefore, it is believed that this may be since the samples used in this study came from different mountain ranges, and there may be certain geographical barriers or ecological isolations that restrict the actual gene flow between populations. The introduction of cultivated resources has led to the exchange of seeds and seedlings between different populations, resulting in lower levels of genetic differentiation between populations. In conclusion, we believe that the two speculations may be the reasons behind this contradiction.

The differences in these correlation parameters among the three populations were generally not significant, with two populations, QY and JD, being closer to each other and relatively more different from the JZ population. The genetic differentiation coefficients Fst and ΦST indicated that there was some degree of genetic differentiation between the QY, JD populations and the JZ population, while no significant genetic differentiation was produced between the QY and JD populations. This is consistent with the analysis of molecular variation (AMOVA) showing the lowest percentage of genetic variation among populations (7%). The richness of genetic diversity in a population has significant impacts on its ability to adapt to environmental changes, resistance to diseases, population stability, and genetic health [88–90]. The QY population has a greater genetic diversity, which means that it would be a better source for selecting new germplasm when breeding. Genetic diversity can provide materials for natural selection to combat pathogens, which has the potential to help in addressing the widespread issue of root rot in the cultivation of *P*. *cyrtonema* [91]. Whereas genetic variation among individuals within populations is the main source of variation (74%), similar results have been reported [37, 58]. In the principal coordinates analysis (PCoA), the three populations could not be clearly divided into three populations based on germplasm origin, and the two populations QY and JD were mixed together, while the JZ population could be roughly separated from the other two populations, indicating that geographical distribution was the main influencing factor, which is consistent with the results of previous studies [56, 58].

The genetic structure analysis showed that the 96 germplasm could be divided into three subgroups, and the results were the same as the clustering results of the diversity study of morphological traits. However, there was a small amount of admixture in the three gene pools, indicating that there was some gene exchange between different populations and that these materials had the same or similar parents. The NJ cluster analysis did not show clear boundaries between germplasm from different geographical regions. However, the QY and JD populations in southern Anhui are relatively distantly related to the JZ population in western Anhui, and there are obvious geographical features. It suggests that geographic isolation and ecological differences due to different geographic distributions may be important factors contributing to this result. The genetic grouping was closely related to the mountain range, and the different origins of this species were genetically distant and did not cluster into one group. This indicates that intraspecific genetic diversity is related to geography, and these studies are consistent with the results of the present study. Dabie Mountain has a high altitude, relatively dry climate, relatively monotonous vegetation types, and is relatively barren. On the other hand, the southern Anhui mountainous region features complex topography, a more humid climate, diverse vegetation types, and soils that have good water retention capacity and fertility. Among the top eight ecological factors that have the most significant impact on the growth of this species, include rainfall, vegetation type, and soil type. These environmental differences may be important factors leading to a certain degree of genetic differentiation between the populations in southern Anhui (JD, QY) and western Anhui (JZ).

The QY and JD populations belong to the same mountainous area in southern Anhui, and the genetic differentiation between them is low, but there are obvious differences in plant morphology. This could be attributed to the fact that the QY population belongs to the Jiuhua Mountain range, while the JD population belongs to the Huangshan Mountain range, which exhibit variances in vegetation and ecological environments. Both Mount Jiuhua and Mount Huangshan are influenced by the monsoon climate, and they share many similarities in terms of their ecosystems and climatic environments. This could potentially lead to lower genetic differentiation between the two populations. Mount Jiuhua belongs to a subtropical humid climate and is primarily characterized by subtropical deciduous broad-leaved forests and mixed forests. On the other hand, Mount Huangshan belongs to a temperate humid climate, and its ecosystem includes subtropical evergreen broad-leaved forests and coniferous forests. Based on previous studies on ecological factors, the more abundant rainfall on Mount Jiuhua may be the main factor leading to the differences in plant traits between the two populations. The unique geographical landforms, climatic conditions, and vegetation types of Mounts Jiuhua create a shaded, cool, and humid environment. This environment aligns with the shade-loving and moisture-loving growth habits of *P*. *cyrtonema*. This may be one of the important reasons for the abundant genetic diversity and high medicinal quality of the QY population. In addition, the conversion of many wild resources into cultivation, the continuous use of rhizomes for asexual propagation and excessive fertilization may all be responsible for the differences in plant morphology.

## Conclusions

Our research study confirms that the Chinese *P*. *cyrtonema* population has a high genetic diversity. The population exhibits high genetic diversity, with most of the variation occurring within the population. The QY population has the highest genetic diversity and has great potential for use in resource conservation and breeding material selection. The genetic structure of populations tends to correlate with environmental factors such as geographical distribution and climatic conditions. The limited number of SSR primers restricts adequate coverage of the target species’ genome, thus compromising the analysis of diversity and accuracy of genetic analysis to some extent. Although the number of markers used in this study was small, but 10 pairs of SSR primers with highly polymorphic loci and abundant genetic variability in 96 germplasm were suitable for the study of genetic diversity of *P*. *cyrtonema*, which can still provide a reference for germplasm selection and resource conservation. In future studies, whole genome sequencing, population dynamics analysis, screening for adaptive genes and studying the interaction between environment and genetics will be of great importance for this species.

## Supporting information

S1 Appendix (DOCX)

S1 Data (ZIP)

S1 File (ZIP)

S2 File (XLSX)
