# Supplementary material for: Genetic diversity and population structure of Polygonatum cyrtonema Hua in China using SSR markers
Source: PLoS One. 2023 Aug 31;18(8):e0290605. doi: 10.1371/journal.pone.0290605 (PMC10470896; doi:10.1371/journal.pone.0290605)
Supplement: S1 Appendix — (DOCX) [file pone.0290605.s001.docx]

**Appendix:**

**Experimental procedure for DNA extraction using the CTAB method and operation of the Fragment Analyzer^TM^ capillary electrophoresis instrument**

**1. Experimental procedure for DNA extraction using the CTAB method**

1.1 Tissue sample pulverization: The plant sample is frozen using liquid nitrogen, then ground in a mortar to obtain finely crushed tissue. Take 25-50 mg of the finely crushed tissue and place it in a 2 mL centrifuge tube. Next, add one sterile magnetic bead to the centrifuge tube, and pulverize it in a ball mill at a frequency of 2000 for 30 seconds.

1.2 Cell lysis: In the 2 mL centrifuge tube containing the pulverized sample, add 900 μL of CTAB buffer preheated to 65℃, 10 μL of β-Mercaptoethanol and an appropriate amount of polyvinylpyrrolidone (PVP40). Then, place the centrifuge tube in a 65℃-water bath for 90 minutes, shaking it three times during this period to ensure thorough mixing.

1.3 Protein removal: After the water bath incubation, remove the centrifuge tube and allow it to cool to below 45℃. Then, add 910 μL of chloroform-isoamyl alcohol (at a 24:1 volume ratio) mixture and mix thoroughly. Centrifuge the mixture at room temperature and 12,000 rpm for 10 minutes. Transfer 700 μL of the supernatant to another 2 mL centrifuge tube and add an equal volume of chloroform-isoamyl alcohol mixture. Repeat this step. Then, take 500 μL of the supernatant and add 330 μL of isopropanol pre-cooled at -20℃ for 30 minutes. Centrifuge at 4℃ and 12,000 rpm for 10 minutes and discard the supernatant.

1.4 DNA precipitation: Add 500 μL of 70% ethanol into the centrifuge tube from the previous step. Centrifuge at 4℃ and 12,000 rpm for 5 minutes, then discard the supernatant. Repeat this step once more.

1.5 DNA washing: Add 500 μL of anhydrous ethanol into the centrifuge tube from the previous step. Centrifuge at 4℃ and 12,000 rpm for 5 minutes, then discard the supernatant. Repeat this step once more.

1.6 DNA dissolution: Place the centrifuge tube from the previous step in a fume hood to evaporate the solvent and let it air dry. Then, dissolve the DNA in sterile water and measure the DNA concentration and purity. Finally, store the DNA at -20℃.

**2. Operation of the Fragment Analyzer^TM^ capillary electrophoresis instrument**

2.1 Sample preparation: The purity and amount of the DNA sample should meet the experimental requirements, with a concentration not lower than 50ng/uL. The DNA fragments are chemically labelled at the 5' end with fluorescent dyes such as FAM, HEX or TARMER. After labelling with fluorescent dyes, the DNA fragments can be detected and confirmed. Different sample preparation methods may use different combinations of fluorescent dyes and reagents. Sample preparation is performed in a 96-well plate.

2.2 Set up the software: Select the appropriate run module and analysis module in the data acquisition software based on the labelling method used, capillary length and gel type for electrophoresis.

2.3 Prepare electrophoresis chip and buffer: Select the appropriate electrophoresis chip for the type of sample. In this study, a DNA Analysis Kit chip is used. Prepare the electrophoresis buffer according to the manufacturer's instructions and add it to the electrophoresis chip.

2.4 Load the samples: Add the diluted DNA samples to the sample wells on the chip. Avoid the formation of bubbles and ensure that the wells are completely filled with sample.

2.5 Run the electrophoresis: Insert the electrophoresis chip into the Fragment AnalyzerTM instrument and start the electrophoresis program. Set the appropriate run time and voltage according to the experimental requirements. DNA fragments of different sizes in the sample will begin to move from the negative electrode towards the positive electrode. The speed of movement is determined by the size of the fragment, with shorter fragments moving faster. Electrophoresis separates the charged DNA fragments according to their length and they pass through the detection window in order of length, generating signals. Shorter fragments reach the detection window first.

2.6 Data analysis: When electrophoresis is complete, the electrophoresis chip is removed from the instrument and the electrophoresis results are analysed using the appropriate software. Depending on the requirements, information such as fragment size distribution, concentration and sample integrity can be obtained.

**3. Laboratory Instruments**

| **Instrument Name** | **Factory Owners** | **Model Number** |
| --- | --- | --- |
| gel electrophoresis | Beijing LiuYi Biotechnology Co., Ltd | DYY-6C |
| PCR instrument | Beijing Donglin Changsheng Biotechnology Co. | DL9700 touch |
| centrifuges | Eppendorf | 5424R |
| capillary electrophoresis | Applied Biosystems(ABI) | 3730XL |
| Gel imaging analyser | Beijing Junyi Electrophoresis Co., Ltd | JY04S-3C |
| Vibration Ball Mill | Beijing Greiman Instrument Co. | GT100X |
| Ultra-low temperature -80 ℃ refrigerator | Haier | DW-S6L388A |
| -20℃ Low Temperature Refrigerator | Haier | BCD-527WDPC |
