# Supplementary material for: Genetic diversity and population structure of Polygonatum cyrtonema Hua in China using SSR markers
Source: PLoS One. 2023 Aug 31;18(8):e0290605. doi: 10.1371/journal.pone.0290605 (PMC10470896; doi:10.1371/journal.pone.0290605)
Supplement: S1 Data — (ZIP) [file pone.0290605.s002.zip › ╨┬╜¿╬─╝■╝╨ (2)/╥2╬∩11/╥2╬∩11.pdf]

Allele Report

Sample 1: Run date and time: 09/15/2020 - 15:47:40 -> 09/15/2020 - 16:45:54

Dye: Blue - 0 peaks - 1.fsa

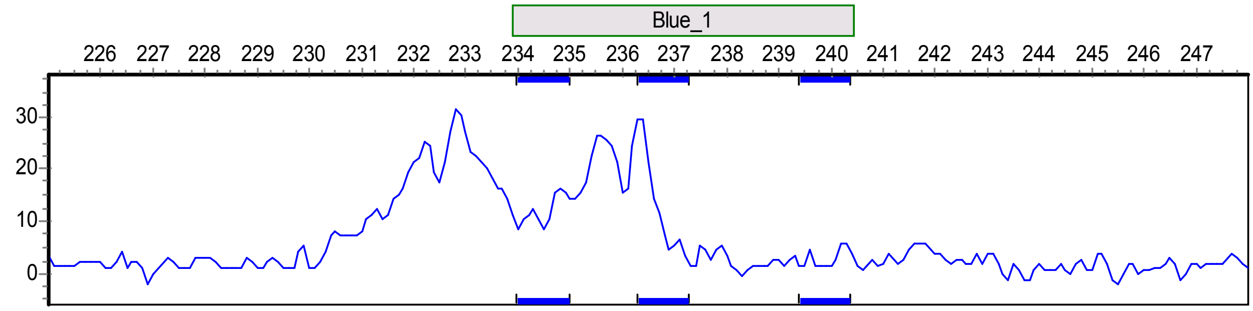

No	Size	Height	Area	Marker	Allele	Difference	Quality	Score	Allele Comments	Sample Comments

Sample 2: Run date and time: 09/15/2020 - 15:47:40 -> 09/15/2020 - 16:45:54

Dye: Blue - 0 peaks - 10.fsa

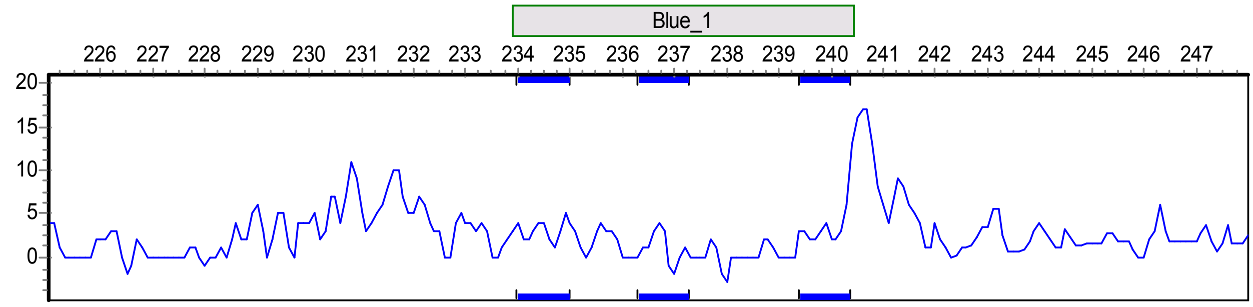

No	Size	Height	Area	Marker	Allele	Difference	Quality	Score	Allele Comments	Sample Comments

Sample 3: Run date and time: 09/15/2020 - 15:47:40 -> 09/15/2020 - 16:45:54

Dye: Blue - 0 peaks - 11.fsa

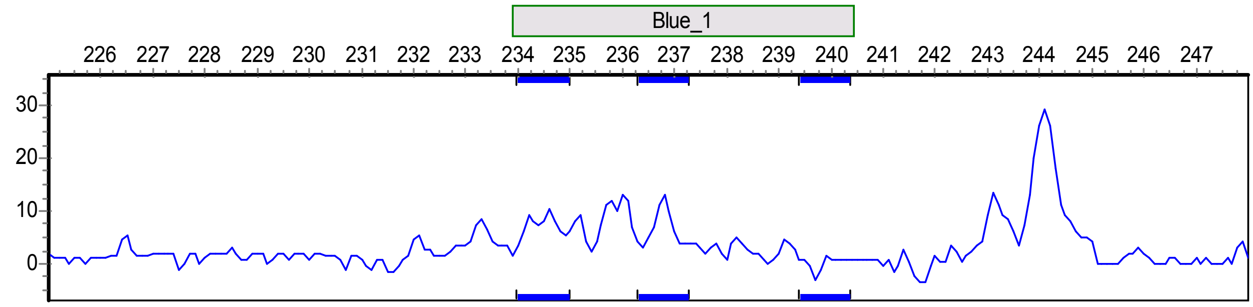

No	Size	Height	Area	Marker	Allele	Difference	Quality	Score	Allele Comments	Sample Comments

Sample 4: Run date and time: 09/15/2020 - 15:47:40 -> 09/15/2020 - 16:45:54

Dye: Blue - 0 peaks - 12.fsa

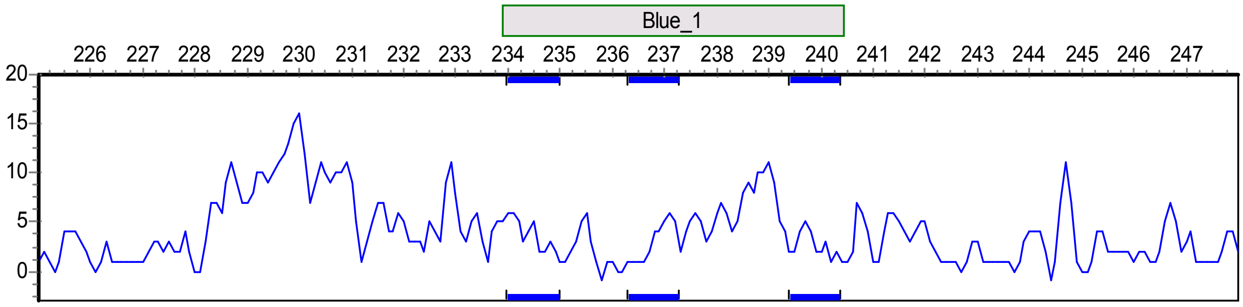

No	Size	Height	Area	Marker	Allele	Difference	Quality	Score	Allele Comments	Sample Comments

Sample 5: Run date and time: 09/15/2020 - 15:47:40 -> 09/15/2020 - 16:45:54

Dye: Blue - 0 peaks - 13.fsa

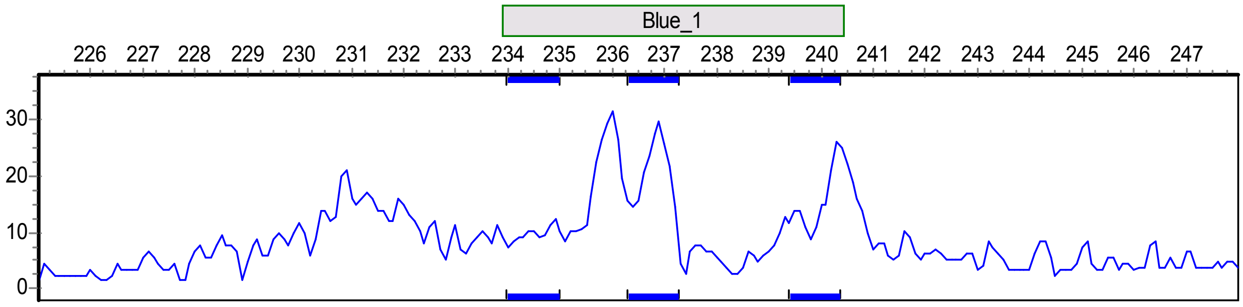

No	Size	Height	Area	Marker	Allele	Difference	Quality	Score	Allele Comments	Sample Comments

Sample 6: Run date and time: 09/15/2020 - 15:47:40 -> 09/15/2020 - 16:45:54

Dye: Blue - 0 peaks - 14.fsa

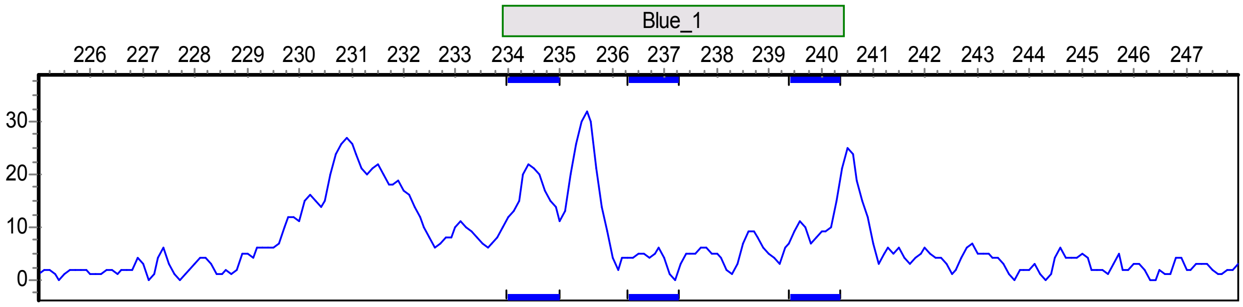

No	Size	Height	Area	Marker	Allele	Difference	Quality	Score	Allele Comments	Sample Comments

Allele Report

Sample 7: Run date and time: 09/15/2020 - 15:47:40 -> 09/15/2020 - 16:45:54

Dye: Blue - 1 peaks - 15.fsa

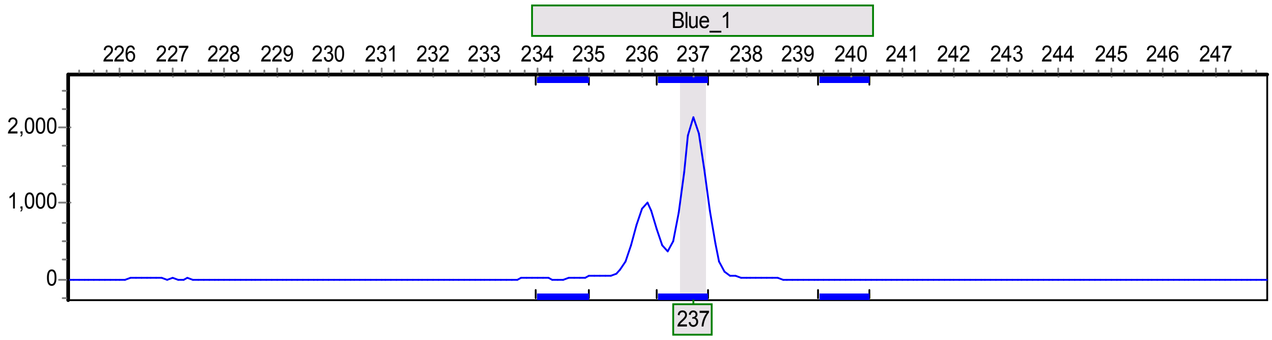

No	Size	Height	Area	Marker	Allele	Difference	Quality	Score	Allele Comments	Sample Comments
1	237.0	2109	11397	Blue_1	237	0.2	Pass	500.0		

Sample 8: Run date and time: 09/15/2020 - 15:47:40 -> 09/15/2020 - 16:45:54

Dye: Blue - 1 peaks - 16.fsa

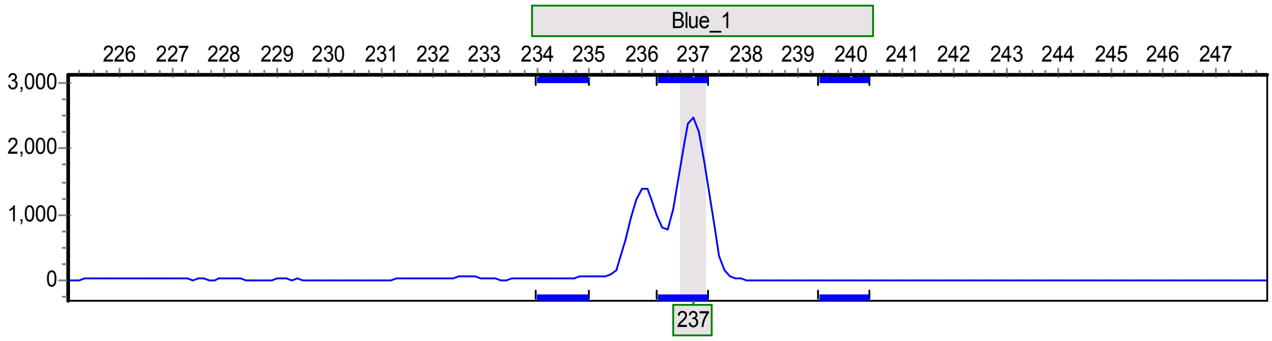

No	Size	Height	Area	Marker	Allele	Difference	Quality	Score	Allele Comments	Sample Comments
1	237.0	2456	16452	Blue_1	237	0.2	Pass	472.2		

Sample 9: Run date and time: 09/15/2020 - 15:47:40 -> 09/15/2020 - 16:45:54

Dye: Blue - 1 peaks - 17.fsa

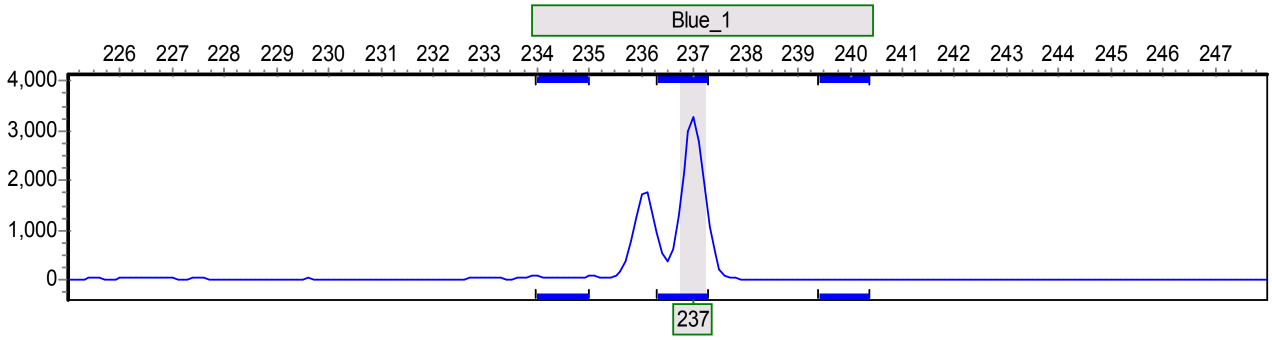

No	Size	Height	Area	Marker	Allele	Difference	Quality	Score	Allele Comments	Sample Comments
1	237.0	3258	15909	Blue_1	237	0.2	Pass	500.0		

Sample 10: Run date and time: 09/15/2020 - 15:47:40 -> 09/15/2020 - 16:45:54

Dye: Blue - 0 peaks - 18.fsa

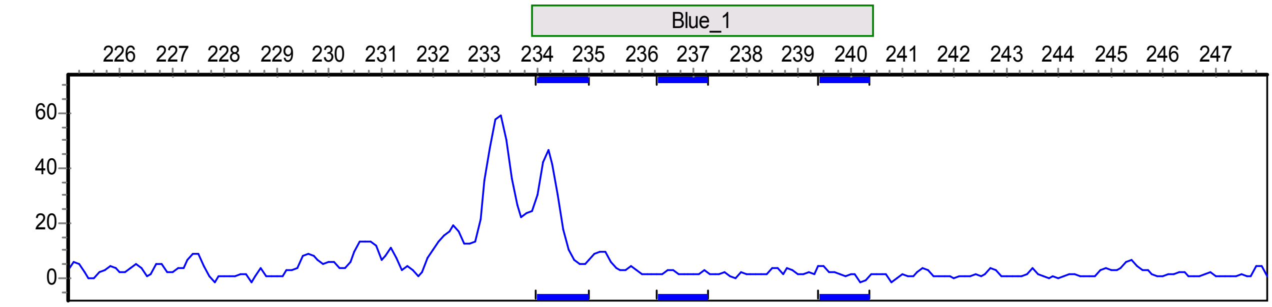

No	Size	Height	Area	Marker	Allele	Difference	Quality	Score	Allele Comments	Sample Comments

Sample 11: Run date and time: 09/15/2020 - 15:47:40 -> 09/15/2020 - 16:45:54

Dye: Blue - 0 peaks - 19.fsa

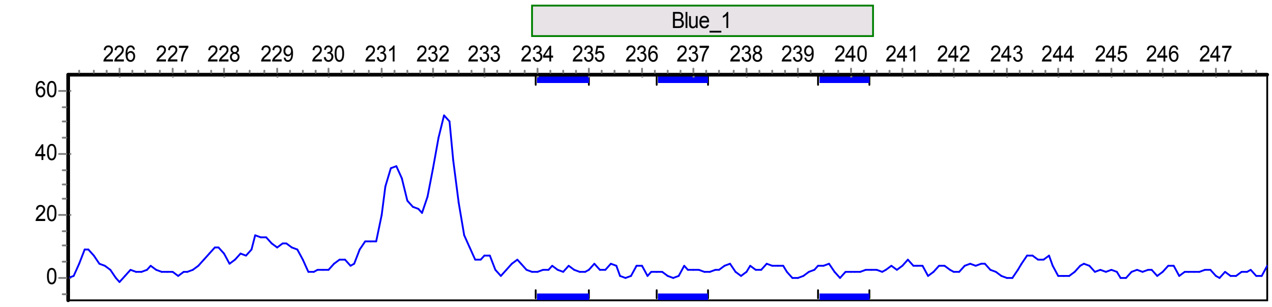

No	Size	Height	Area	Marker	Allele	Difference	Quality	Score	Allele Comments	Sample Comments

Sample 12: Run date and time: 09/15/2020 - 15:47:40 -> 09/15/2020 - 16:45:54

Dye: Blue - 1 peaks - 2.fsa

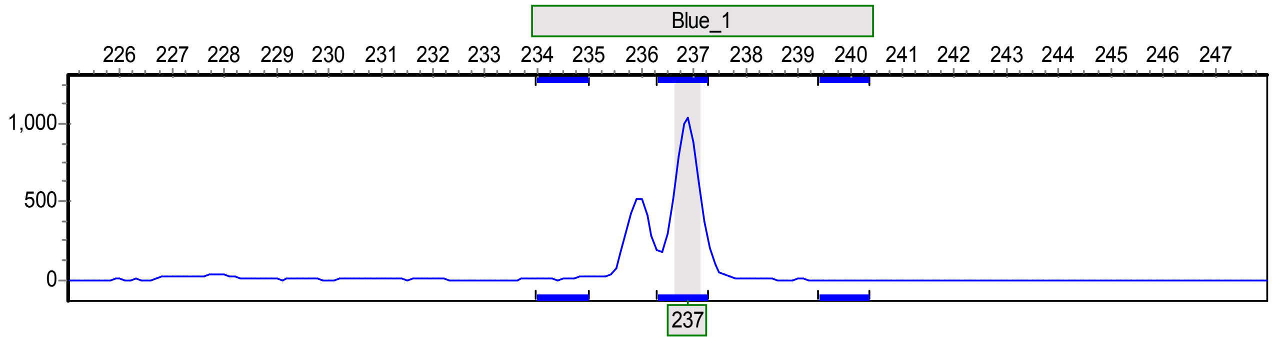

No	Size	Height	Area	Marker	Allele	Difference	Quality	Score	Allele Comments	Sample Comments
1	236.9	1030	5641	Blue_1	237	0.1	Pass	192.6		

Sample 13: Run date and time: 09/15/2020 - 15:47:40 -> 09/15/2020 - 16:45:54

Dye: Blue - 1 peaks - 20.fsa

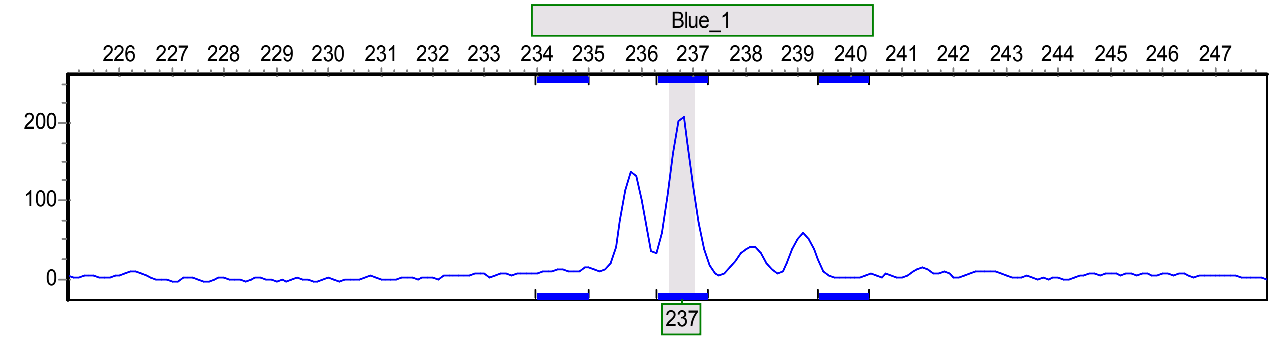

No	Size	Height	Area	Marker	Allele	Difference	Quality	Score	Allele Comments	Sample Comments
1	236.8	206	1118	Blue_1	237	0.0	Pass	14.7		

Sample 14: Run date and time: 09/15/2020 - 15:47:40 -> 09/15/2020 - 16:45:54

Dye: Blue - 1 peaks - 21.fsa

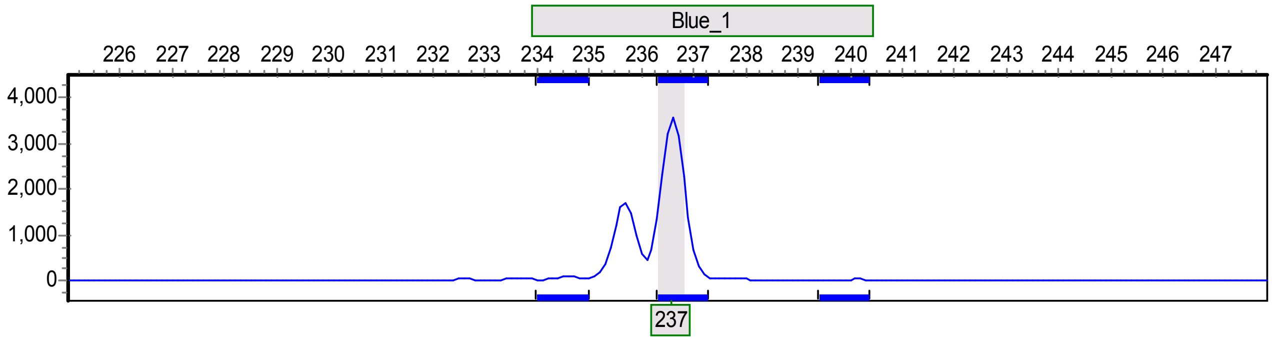

No	Size	Height	Area	Marker	Allele	Difference	Quality	Score	Allele Comments	Sample Comments
1	236.6	3537	17788	Blue_1	237	0.2	Pass	500.0		

Sample 15: Run date and time: 09/15/2020 - 15:47:40 -> 09/15/2020 - 16:45:54

Dye: Blue - 1 peaks - 22.fsa

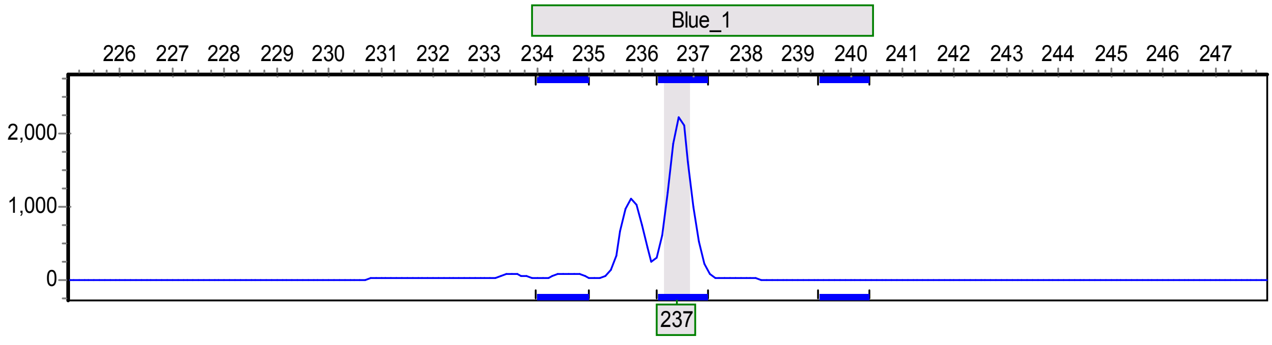

No	Size	Height	Area	Marker	Allele	Difference	Quality	Score	Allele Comments	Sample Comments
1	236.7	2207	10932	Blue_1	237	0.1	Pass	500.0		

Allele Report

Sample 16: Run date and time: 09/15/2020 - 15:47:40 -> 09/15/2020 - 16:45:54

Dye: Blue - 1 peaks - 23.fsa

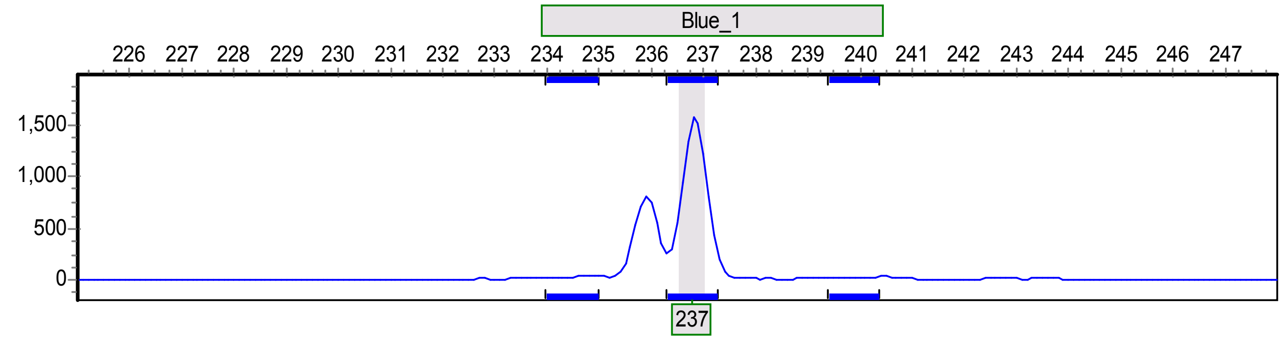

No	Size	Height	Area	Marker	Allele	Difference	Quality	Score	Allele Comments	Sample Comments
1	236.8	1568	8488	Blue_1	237	0.0	Pass	352.5		

Sample 17: Run date and time: 09/15/2020 - 15:47:40 -> 09/15/2020 - 16:45:54

Dye: Blue - 0 peaks - 24.fsa

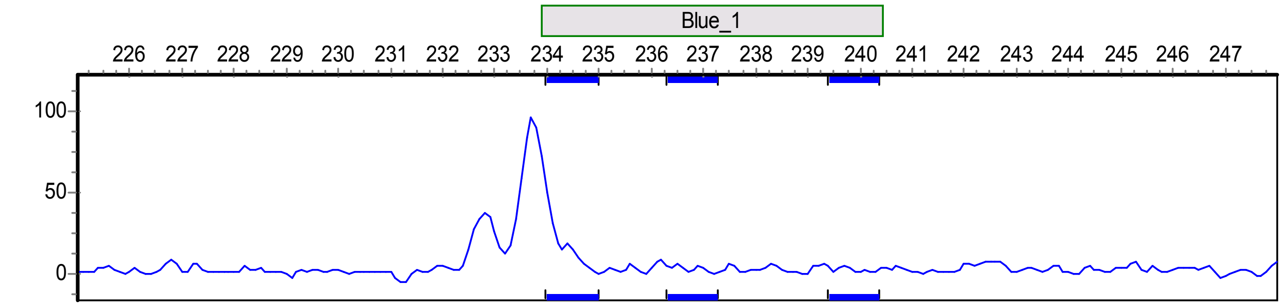

No	Size	Height	Area	Marker	Allele	Difference	Quality	Score	Allele Comments	Sample Comments

Sample 18: Run date and time: 09/15/2020 - 15:47:40 -> 09/15/2020 - 16:45:54

Dye: Blue - 0 peaks - 25.fsa

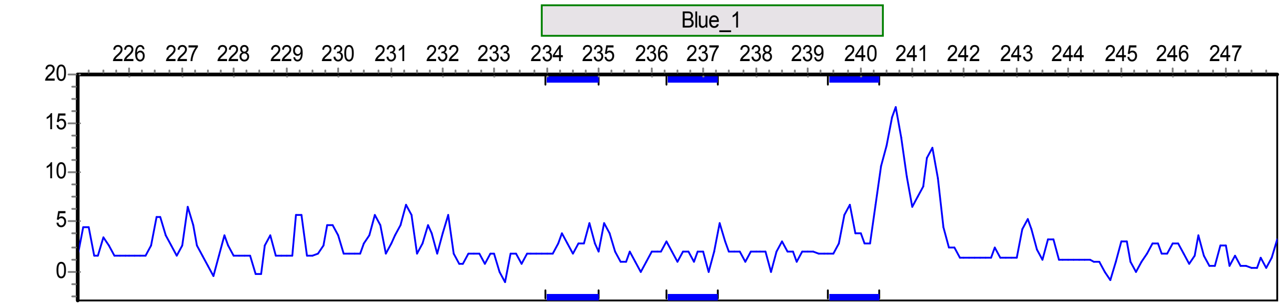

No	Size	Height	Area	Marker	Allele	Difference	Quality	Score	Allele Comments	Sample Comments

Sample 19: Run date and time: 09/15/2020 - 15:47:40 -> 09/15/2020 - 16:45:54

Dye: Blue - 0 peaks - 26.fsa

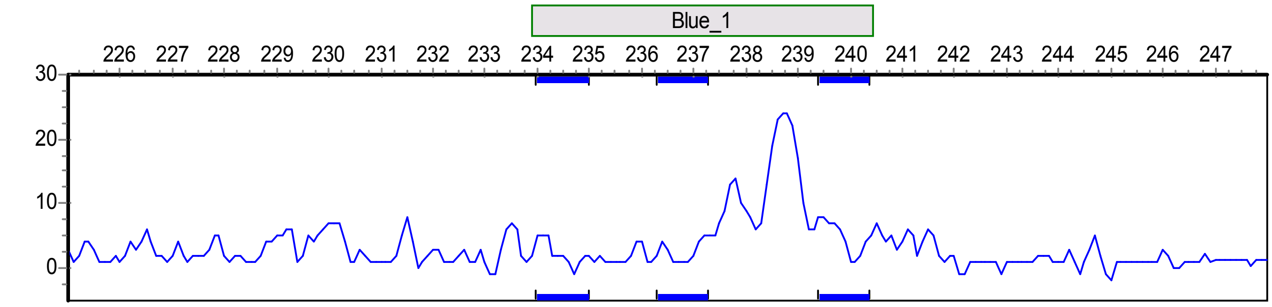

No	Size	Height	Area	Marker	Allele	Difference	Quality	Score	Allele Comments	Sample Comments

Sample 20: Run date and time: 09/15/2020 - 15:47:40 -> 09/15/2020 - 16:45:54

Dye: Blue - 1 peaks - 27.fsa

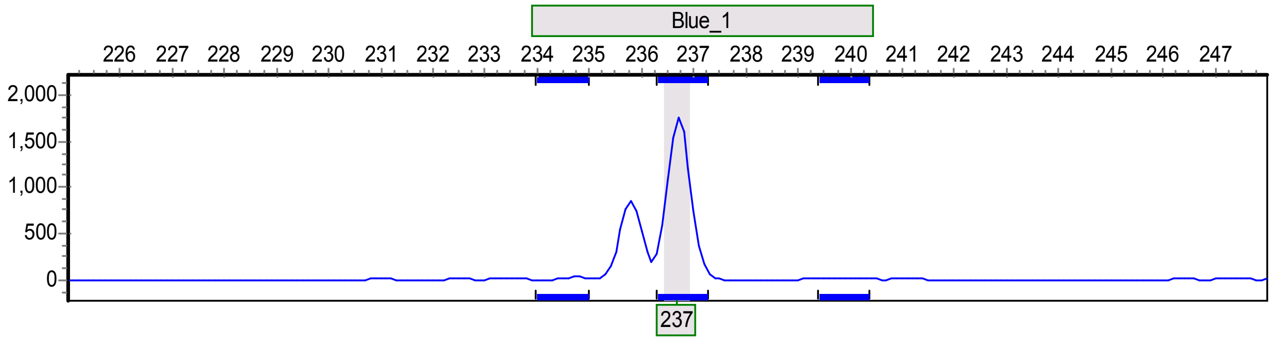

No	Size	Height	Area	Marker	Allele	Difference	Quality	Score	Allele Comments	Sample Comments
1	236.7	1742	8814	Blue_1	237	0.1	Pass	450.5		

Sample 21: Run date and time: 09/15/2020 - 15:47:40 -> 09/15/2020 - 16:45:54

Dye: Blue - 1 peaks - 28.fsa

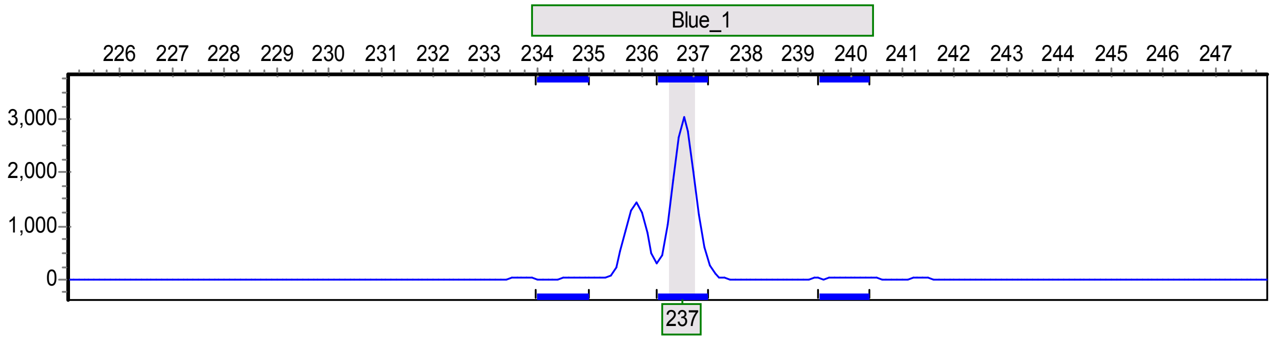

No	Size	Height	Area	Marker	Allele	Difference	Quality	Score	Allele Comments	Sample Comments
1	236.8	3005	14830	Blue_1	237	0.0	Pass	500.0		

Sample 22: Run date and time: 09/15/2020 - 15:47:40 -> 09/15/2020 - 16:45:54

Dye: Blue - 0 peaks - 29.fsa

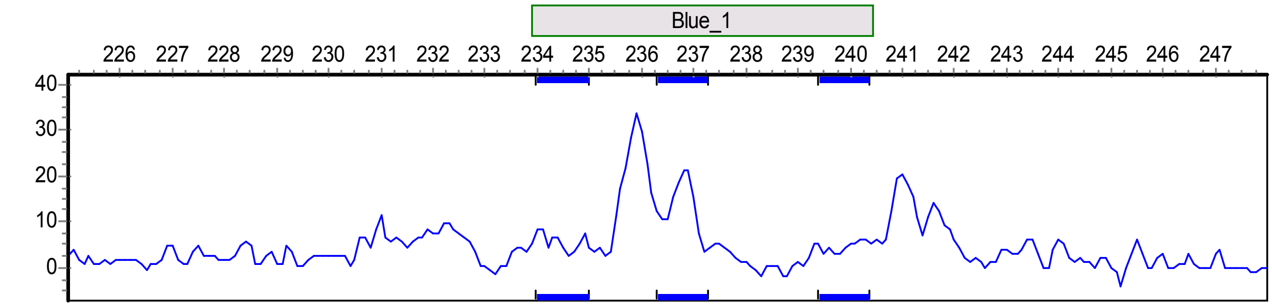

No	Size	Height	Area	Marker	Allele	Difference	Quality	Score	Allele Comments	Sample Comments

Sample 23: Run date and time: 09/15/2020 - 15:47:40 -> 09/15/2020 - 16:45:54

Dye: Blue - 1 peaks - 3.fsa

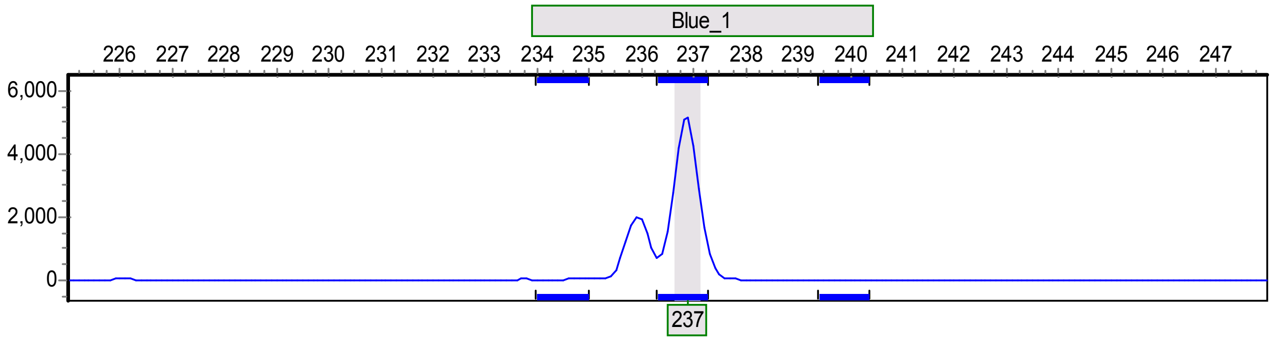

No	Size	Height	Area	Marker	Allele	Difference	Quality	Score	Allele Comments	Sample Comments
1	236.9	5112	27714	Blue_1	237	0.1	Pass	500.0		

Sample 24: Run date and time: 09/15/2020 - 15:47:40 -> 09/15/2020 - 16:45:54

Dye: Blue - 1 peaks - 30.fsa

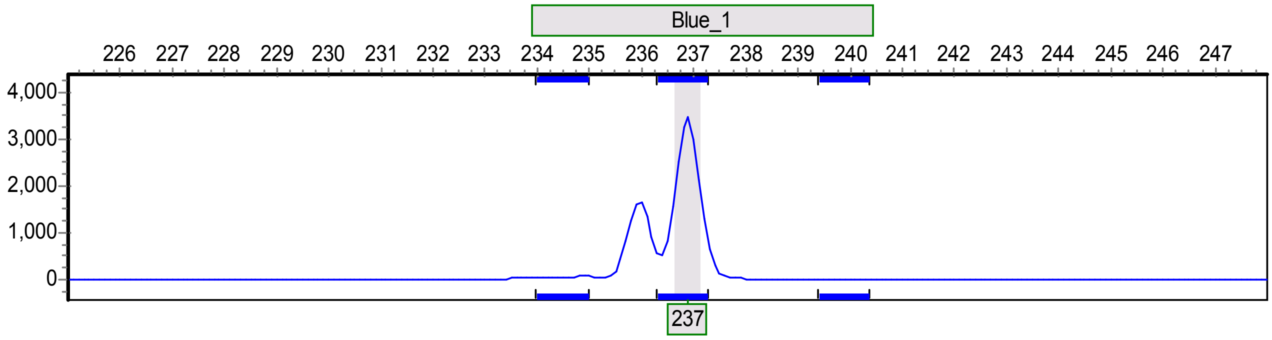

No	Size	Height	Area	Marker	Allele	Difference	Quality	Score	Allele Comments	Sample Comments
1	236.9	3476	18386	Blue_1	237	0.1	Pass	500.0		

Allele Report

Sample 25: Run date and time: 09/15/2020 - 15:47:40 -> 09/15/2020 - 16:45:54

Dye: Blue - 0 peaks - 31.fsa

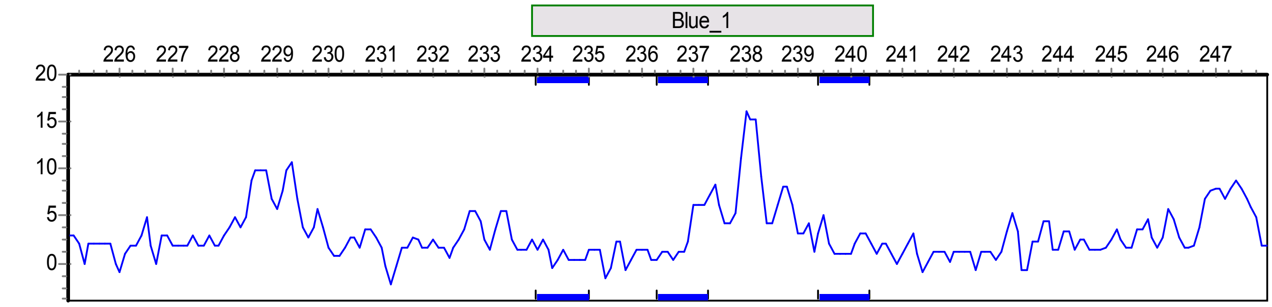

No	Size	Height	Area	Marker	Allele	Difference	Quality	Score	Allele Comments	Sample Comments

Sample 26: Run date and time: 09/15/2020 - 15:47:40 -> 09/15/2020 - 16:45:54

Dye: Blue - 0 peaks - 32.fsa

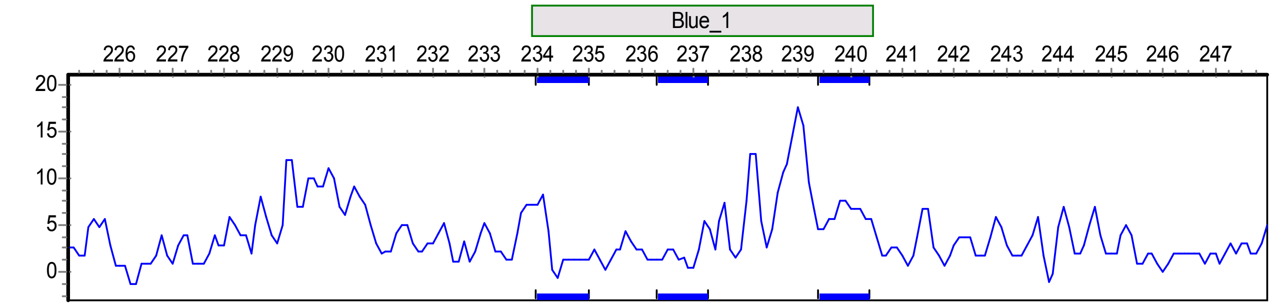

No	Size	Height	Area	Marker	Allele	Difference	Quality	Score	Allele Comments	Sample Comments

Sample 27: Run date and time: 09/15/2020 - 15:47:40 -> 09/15/2020 - 16:45:54

Dye: Blue - 1 peaks - 33.fsa

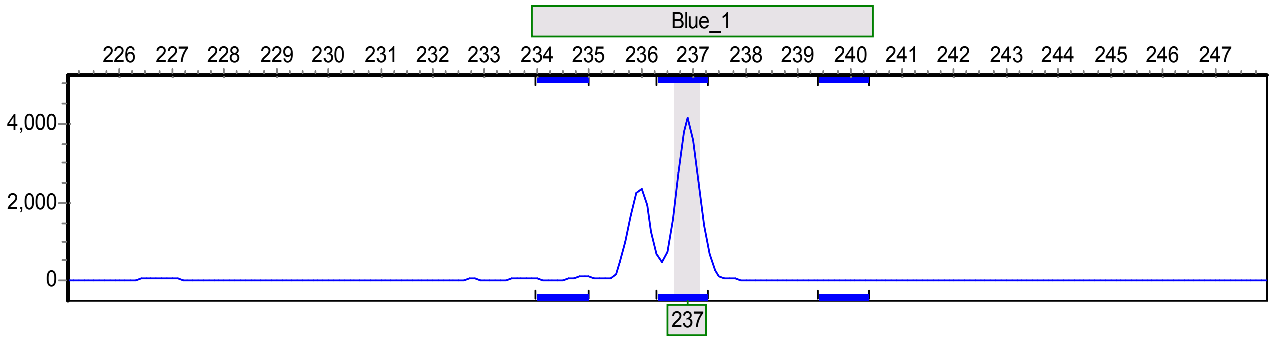

No	Size	Height	Area	Marker	Allele	Difference	Quality	Score	Allele Comments	Sample Comments
1	236.9	4153	20316	Blue_1	237	0.1	Pass	500.0		

Sample 28: Run date and time: 09/15/2020 - 15:47:40 -> 09/15/2020 - 16:45:54

Dye: Blue - 1 peaks - 34.fsa

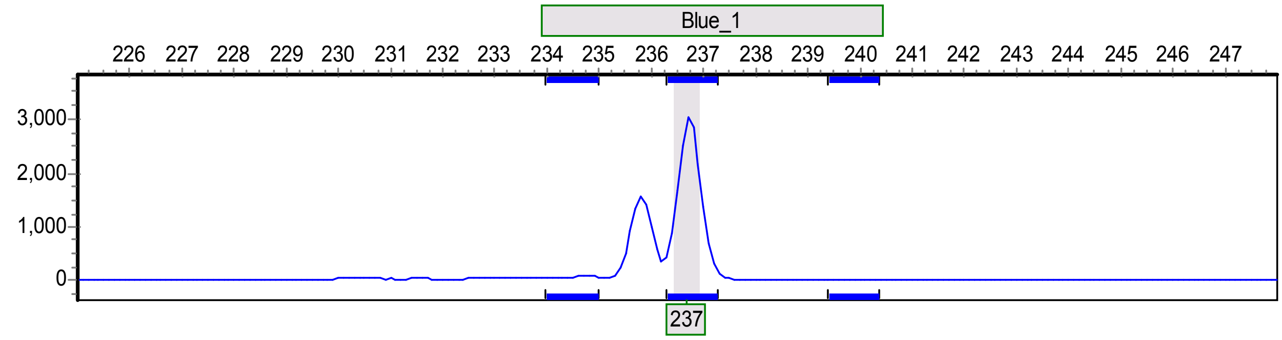

No	Size	Height	Area	Marker	Allele	Difference	Quality	Score	Allele Comments	Sample Comments
1	236.7	3031	15001	Blue_1	237	0.1	Pass	500.0		

Sample 29: Run date and time: 09/15/2020 - 15:47:40 -> 09/15/2020 - 16:45:54

Dye: Blue - 2 peaks - 35.fsa

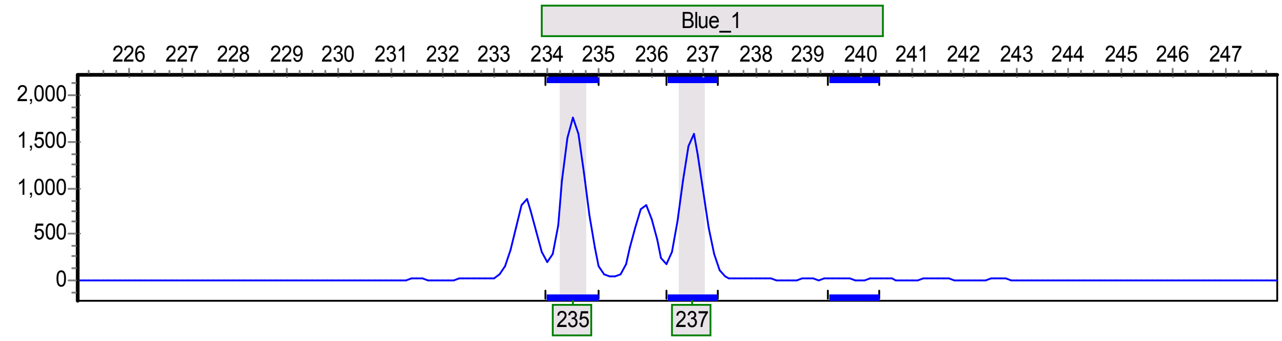

No	Size	Height	Area	Marker	Allele	Difference	Quality	Score	Allele Comments	Sample Comments
1	234.5	1754	8713	Blue_1	235	0.0	Pass	465.5		
2	236.8	1578	7884	Blue_1	237	0.0	Pass	397.7		

Sample 30: Run date and time: 09/15/2020 - 15:47:40 -> 09/15/2020 - 16:45:54

Dye: Blue - 1 peaks - 36.fsa

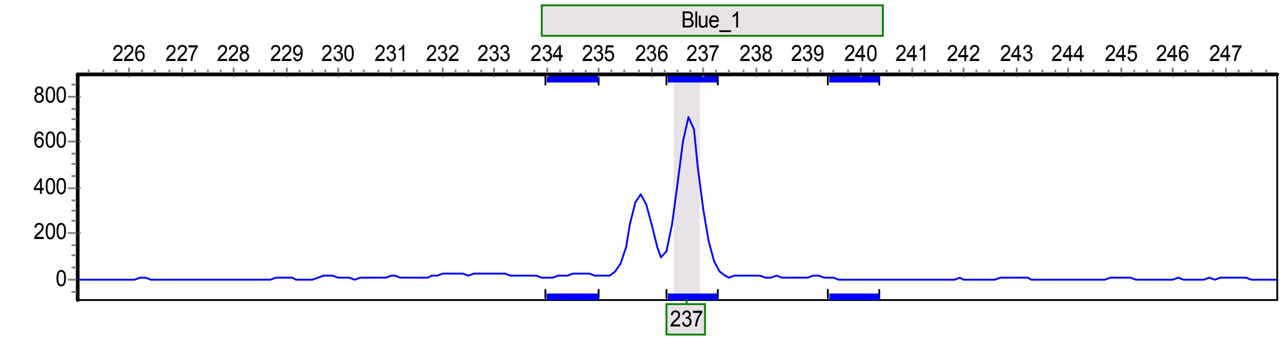

No	Size	Height	Area	Marker	Allele	Difference	Quality	Score	Allele Comments	Sample Comments
1	236.7	707	3627	Blue_1	237	0.1	Pass	119.6		

Allele Report

Sample 31: Run date and time: 09/15/2020 - 15:47:40 -> 09/15/2020 - 16:45:54

Dye: Blue - 1 peaks - 37.fsa

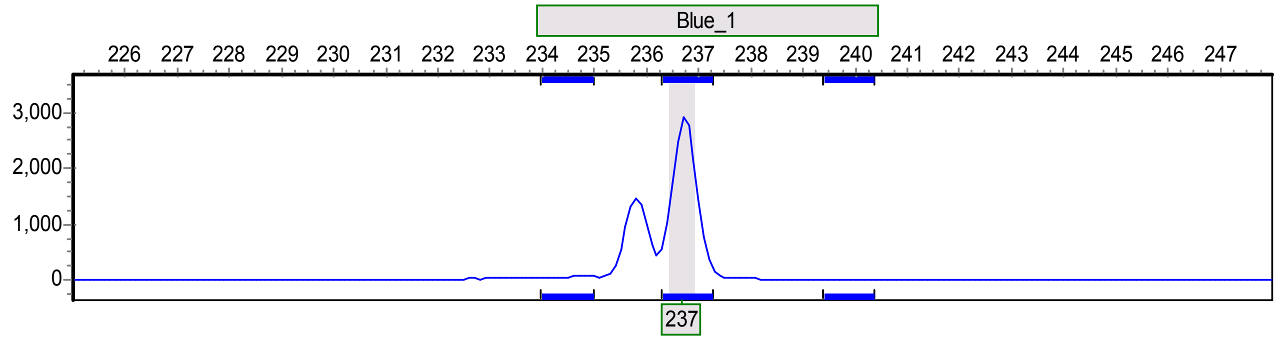

No	Size	Height	Area	Marker	Allele	Difference	Quality	Score	Allele Comments	Sample Comments
1	236.7	2902	15450	Blue_1	237	0.1	Pass	500.0		

Sample 32: Run date and time: 09/15/2020 - 15:47:40 -> 09/15/2020 - 16:45:54

Dye: Blue - 1 peaks - 38.fsa

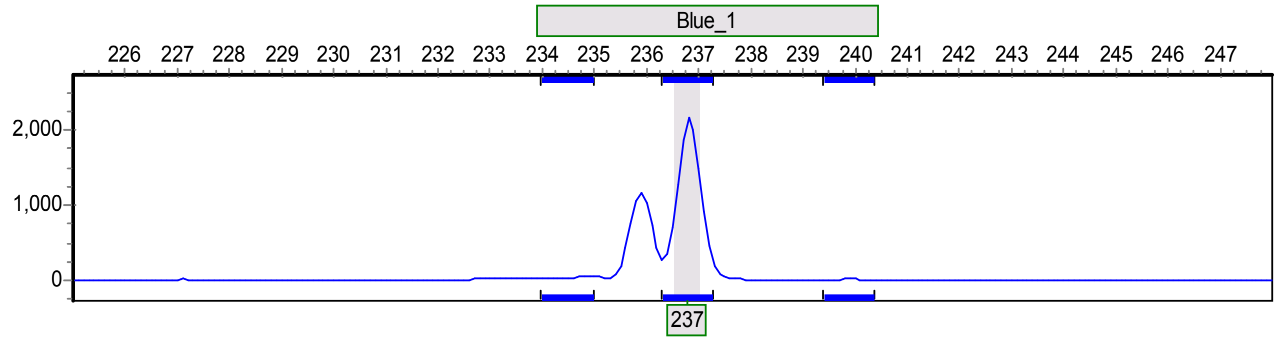

No	Size	Height	Area	Marker	Allele	Difference	Quality	Score	Allele Comments	Sample Comments
1	236.8	2142	10708	Blue_1	237	0.0	Pass	500.0		

Sample 33: Run date and time: 09/15/2020 - 15:47:40 -> 09/15/2020 - 16:45:54

Dye: Blue - 1 peaks - 39.fsa

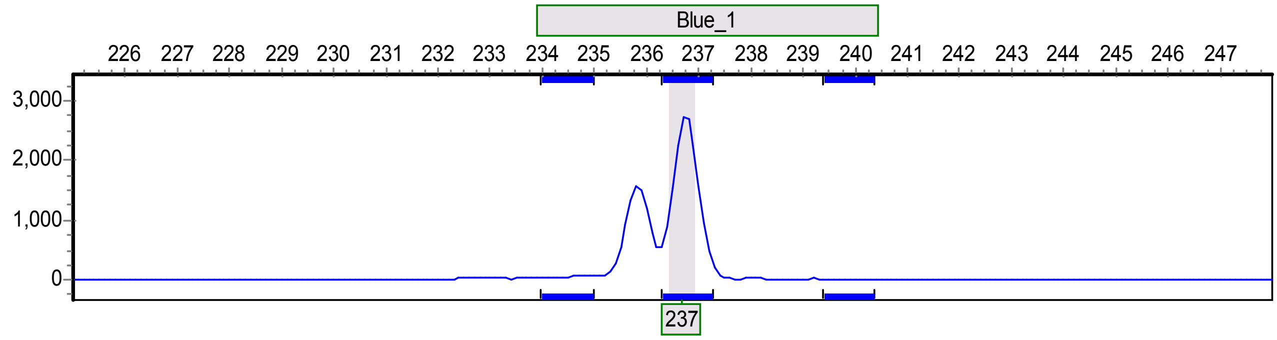

No	Size	Height	Area	Marker	Allele	Difference	Quality	Score	Allele Comments	Sample Comments
1	236.7	2696	14864	Blue_1	237	0.1	Pass	500.0		

Allele Report

Sample 34: Run date and time: 09/15/2020 - 15:47:40 -> 09/15/2020 - 16:45:54

Dye: Blue - 1 peaks - 4.fsa

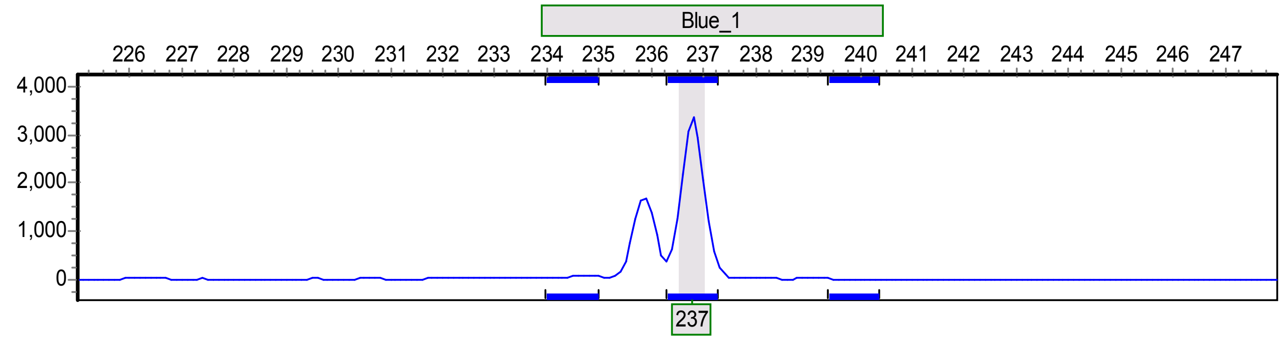

No	Size	Height	Area	Marker	Allele	Difference	Quality	Score	Allele Comments	Sample Comments
1	236.8	3345	16547	Blue_1	237	0.0	Pass	500.0		

Sample 35: Run date and time: 09/15/2020 - 15:47:40 -> 09/15/2020 - 16:45:54

Dye: Blue - 1 peaks - 40.fsa

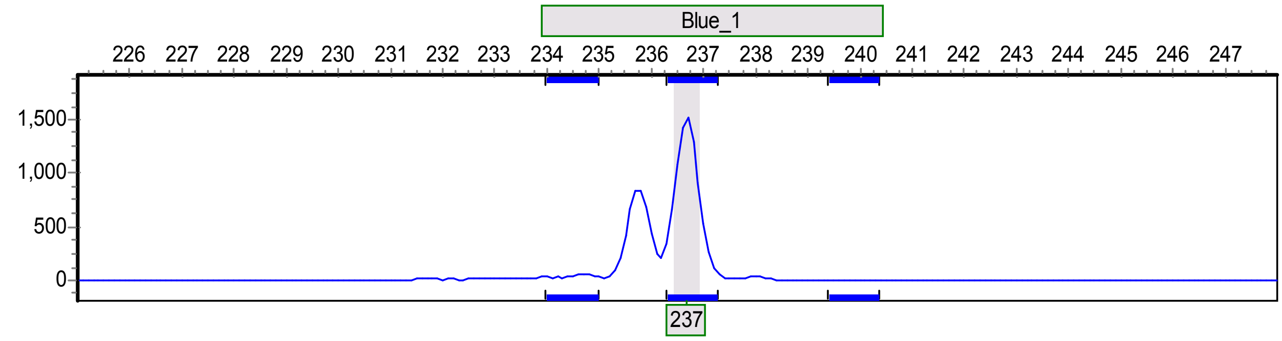

No	Size	Height	Area	Marker	Allele	Difference	Quality	Score	Allele Comments	Sample Comments
1	236.7	1505	7737	Blue_1	237	0.1	Pass	360.7		

Sample 36: Run date and time: 09/15/2020 - 15:47:40 -> 09/15/2020 - 16:45:54

Dye: Blue - 2 peaks - 41.fsa

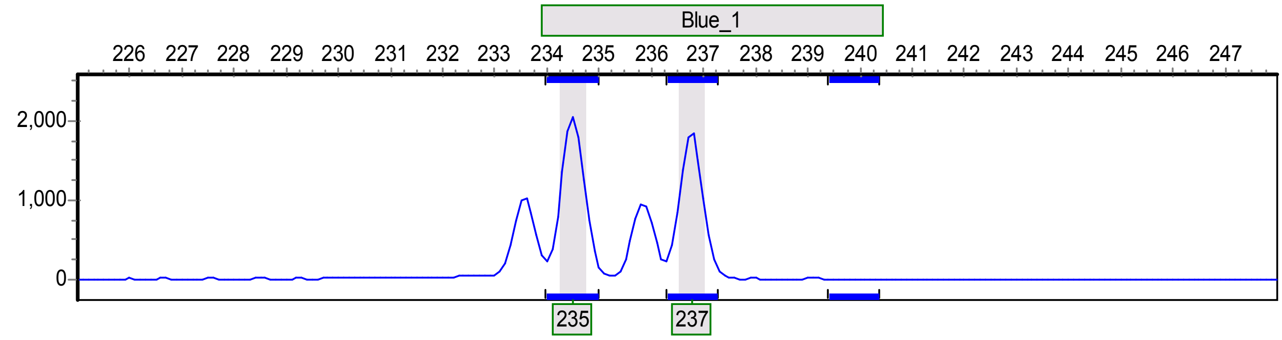

No	Size	Height	Area	Marker	Allele	Difference	Quality	Score	Allele Comments	Sample Comments
1	234.5	2040	10199	Blue_1	235	0.0	Pass	500.0		
2	236.8	1850	9361	Blue_1	237	0.0	Pass	494.1		

Sample 37: Run date and time: 09/15/2020 - 15:47:40 -> 09/15/2020 - 16:45:54

Dye: Blue - 1 peaks - 42.fsa

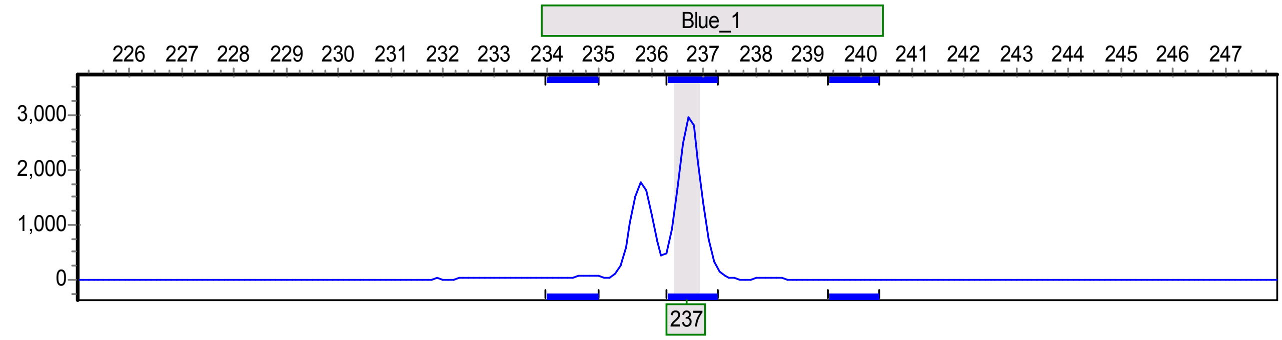

No	Size	Height	Area	Marker	Allele	Difference	Quality	Score	Allele Comments	Sample Comments
1	236.7	2947	15110	Blue_1	237	0.1	Pass	500.0		

Sample 38: Run date and time: 09/15/2020 - 15:47:40 -> 09/15/2020 - 16:45:54

Dye: Blue - 2 peaks - 43.fsa

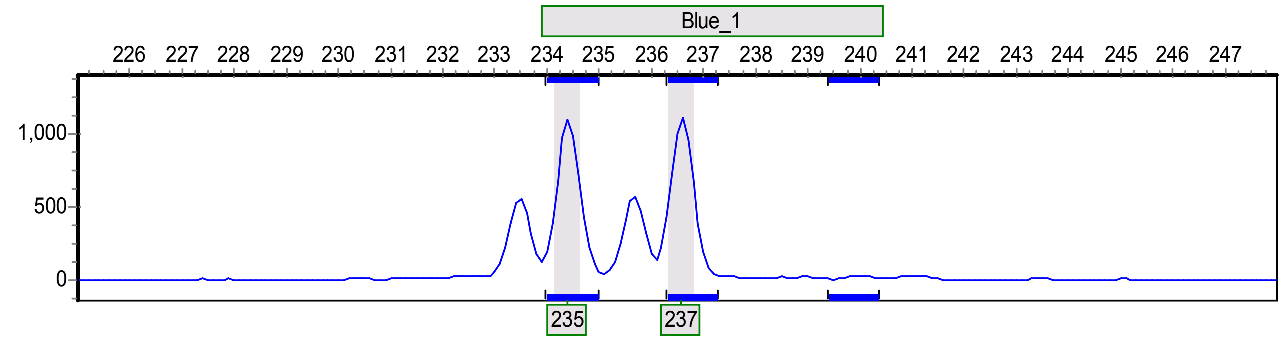

No	Size	Height	Area	Marker	Allele	Difference	Quality	Score	Allele Comments	Sample Comments
1	234.4	1088	5443	Blue_1	235	0.1	Pass	235.2		
2	236.6	1102	5412	Blue_1	237	0.2	Pass	245.0		

Sample 39: Run date and time: 09/15/2020 - 15:47:40 -> 09/15/2020 - 16:45:54

Dye: Blue - 2 peaks - 44.fsa

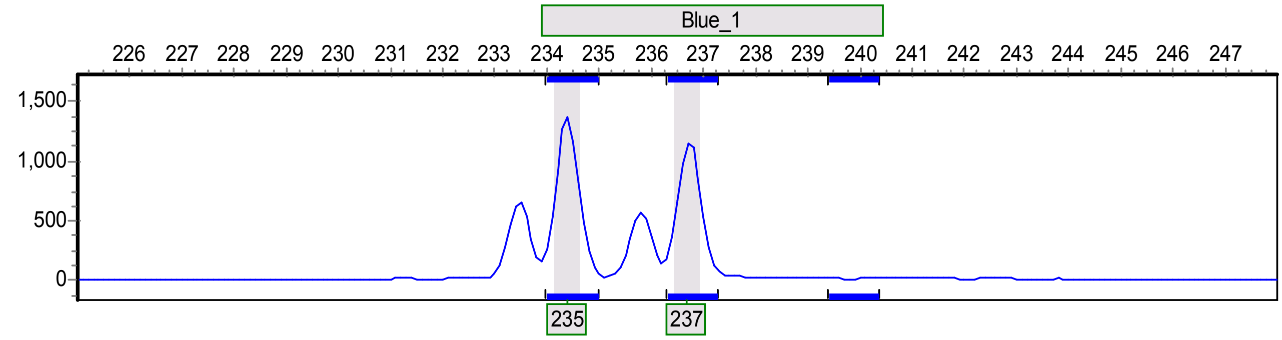

No	Size	Height	Area	Marker	Allele	Difference	Quality	Score	Allele Comments	Sample Comments
1	234.4	1360	6719	Blue_1	235	0.1	Pass	328.4		
2	236.7	1149	5861	Blue_1	237	0.1	Pass	247.5		

Allele Report

Sample 40: Run date and time: 09/15/2020 - 15:47:40 -> 09/15/2020 - 16:45:54

Dye: Blue - 1 peaks - 45.fsa

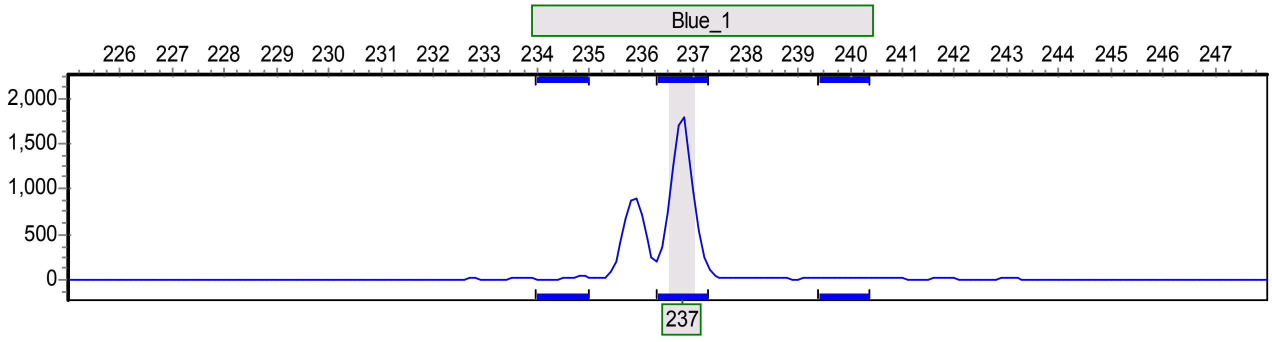

No	Size	Height	Area	Marker	Allele	Difference	Quality	Score	Allele Comments	Sample Comments
1	236.8	1789	8660	Blue_1	237	0.0	Pass	495.0		

Sample 41: Run date and time: 09/15/2020 - 15:47:40 -> 09/15/2020 - 16:45:54

Dye: Blue - 1 peaks - 46.fsa

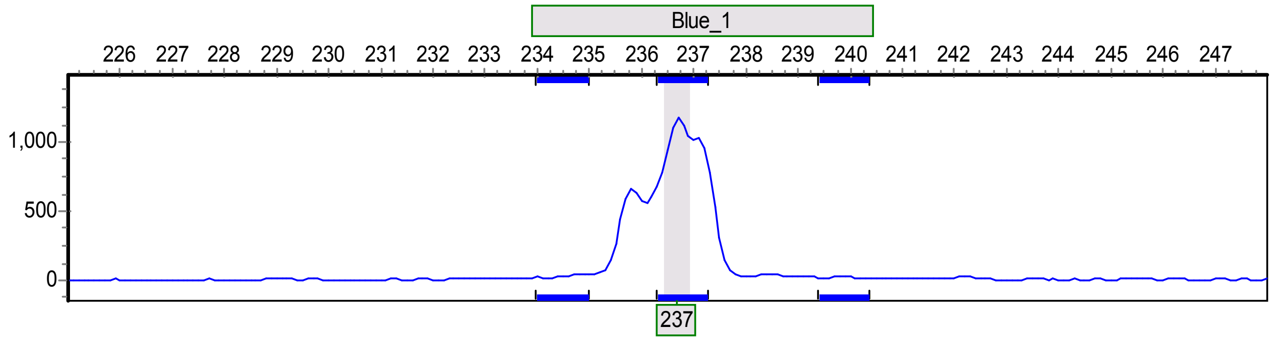

No	Size	Height	Area	Marker	Allele	Difference	Quality	Score	Allele Comments	Sample Comments
1	236.7	1165	11332	Blue_1	237	0.1	Pass	58.5		

Sample 42: Run date and time: 09/15/2020 - 15:47:40 -> 09/15/2020 - 16:45:54

Dye: Blue - 1 peaks - 47.fsa

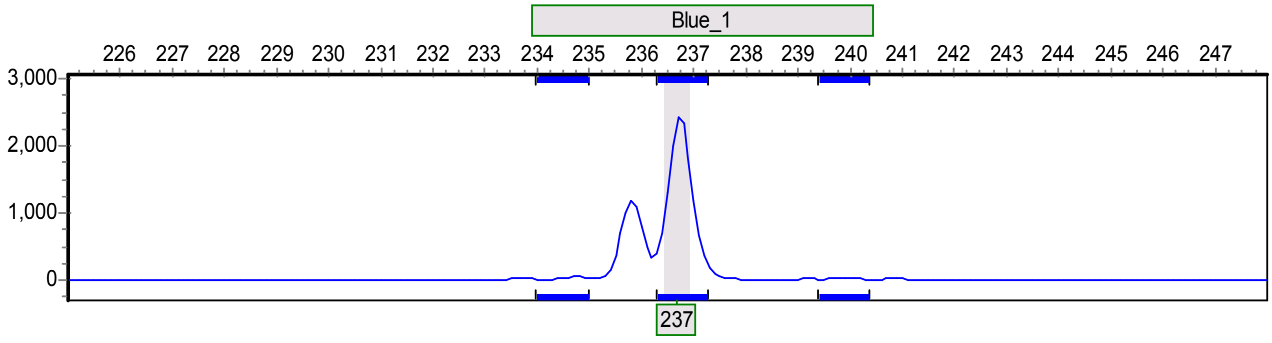

No	Size	Height	Area	Marker	Allele	Difference	Quality	Score	Allele Comments	Sample Comments
1	236.7	2404	12489	Blue_1	237	0.1	Pass	500.0		

Allele Report

Sample 43: Run date and time: 09/15/2020 - 15:47:40 -> 09/15/2020 - 16:45:54

Dye: Blue - 1 peaks - 48.fsa

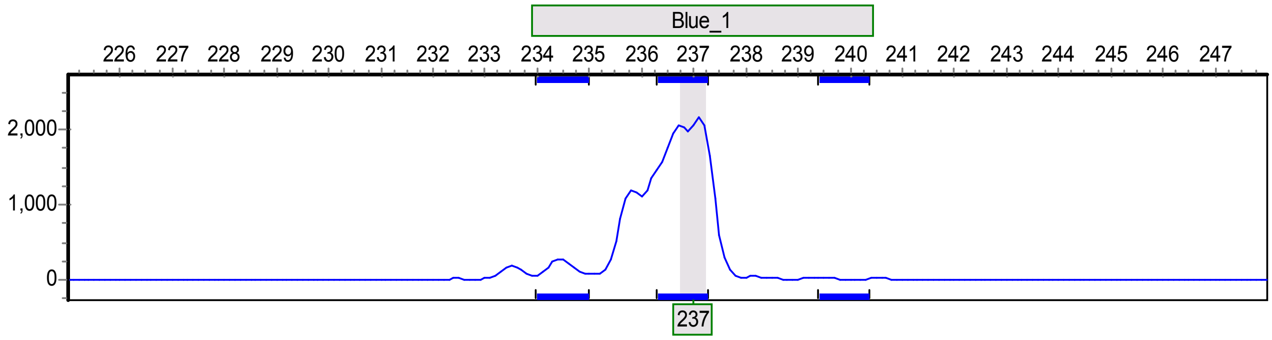

No	Size	Height	Area	Marker	Allele	Difference	Quality	Score	Allele Comments	Sample Comments
1	237.0	2050	30764	Blue_1	237	0.2	Pass	72.5		

Sample 44: Run date and time: 09/15/2020 - 15:47:40 -> 09/15/2020 - 16:45:54

Dye: Blue - 1 peaks - 49.fsa

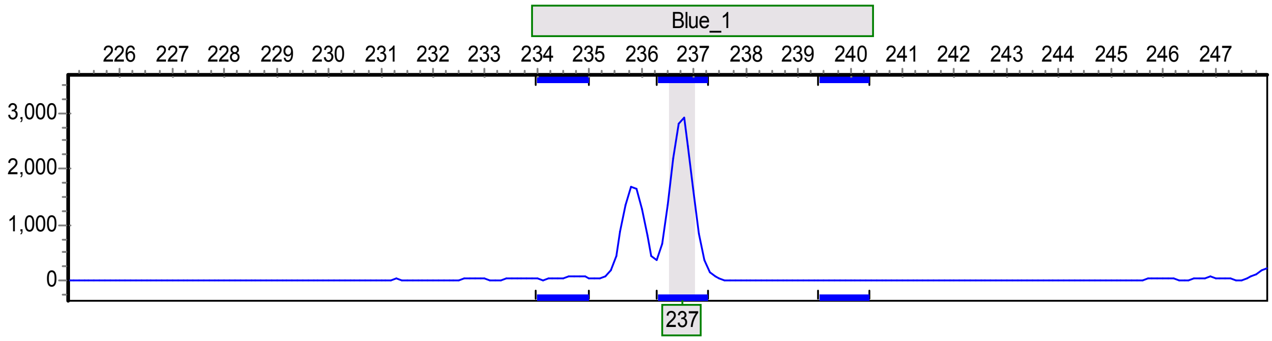

No	Size	Height	Area	Marker	Allele	Difference	Quality	Score	Allele Comments	Sample Comments
1	236.8	2899	14571	Blue_1	237	0.0	Pass	500.0		

Sample 45: Run date and time: 09/15/2020 - 15:47:40 -> 09/15/2020 - 16:45:54

Dye: Blue - 1 peaks - 5.fsa

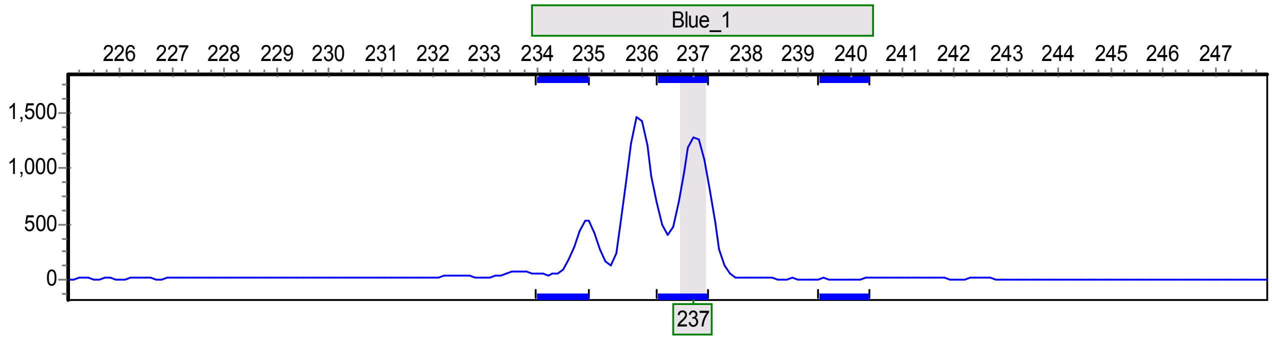

No	Size	Height	Area	Marker	Allele	Difference	Quality	Score	Allele Comments	Sample Comments
1	237.0	1286	8473	Blue_1	237	0.2	Pass	177.5		

Allele Report

Sample 46: Run date and time: 09/15/2020 - 15:47:40 -> 09/15/2020 - 16:45:54

Dye: Blue - 1 peaks - 50.fsa

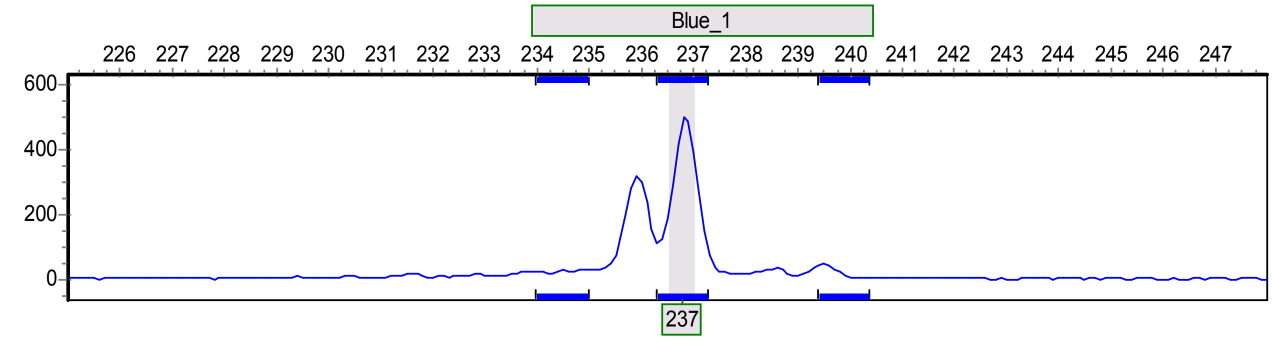

No	Size	Height	Area	Marker	Allele	Difference	Quality	Score	Allele Comments	Sample Comments
1	236.8	499	2689	Blue_1	237	0.0	Pass	59.6		

Sample 47: Run date and time: 09/15/2020 - 15:47:40 -> 09/15/2020 - 16:45:54

Dye: Blue - 1 peaks - 51.fsa

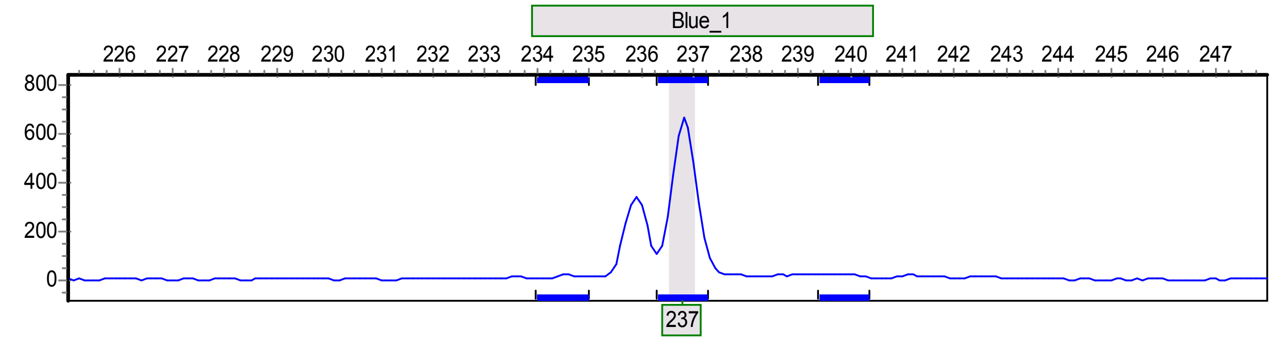

No	Size	Height	Area	Marker	Allele	Difference	Quality	Score	Allele Comments	Sample Comments
1	236.8	663	3611	Blue_1	237	0.0	Pass	99.6		

Sample 48: Run date and time: 09/15/2020 - 15:47:40 -> 09/15/2020 - 16:45:54

Dye: Blue - 1 peaks - 52.fsa

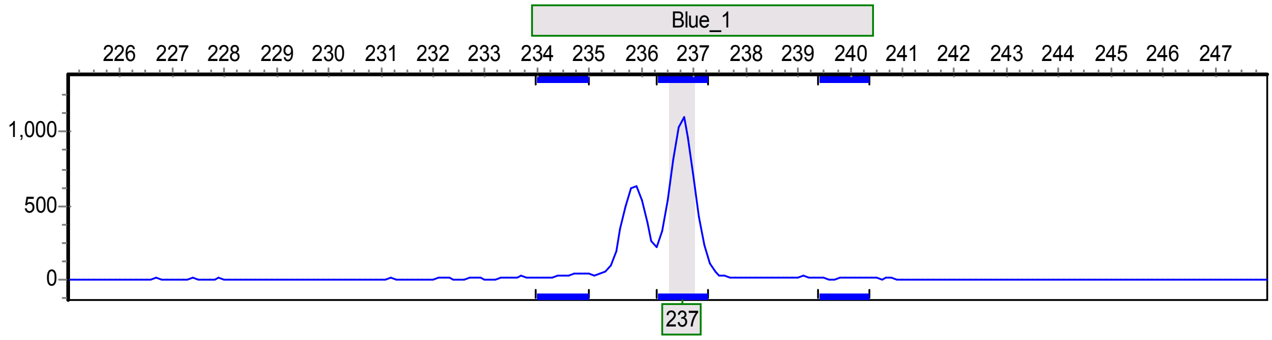

No	Size	Height	Area	Marker	Allele	Difference	Quality	Score	Allele Comments	Sample Comments
1	236.8	1096	5985	Blue_1	237	0.0	Pass	206.6		

Allele Report

Sample 49: Run date and time: 09/15/2020 - 15:47:40 -> 09/15/2020 - 16:45:54

Dye: Blue - 0 peaks - 53.fsa

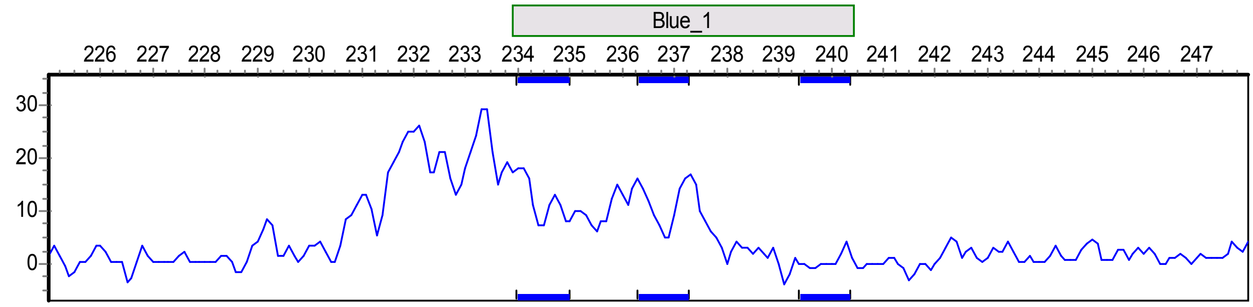

No	Size	Height	Area	Marker	Allele	Difference	Quality	Score	Allele Comments	Sample Comments

Sample 50: Run date and time: 09/15/2020 - 15:47:40 -> 09/15/2020 - 16:45:54

Dye: Blue - 1 peaks - 54.fsa

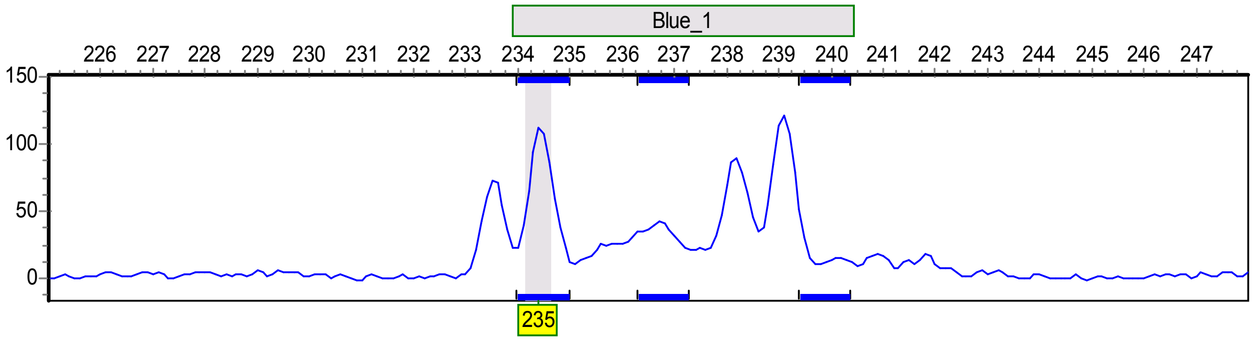

No	Size	Height	Area	Marker	Allele	Difference	Quality	Score	Allele Comments	Sample Comments
1	234.4	111	604	Blue_1	235	0.1	Check	4.6		

Sample 51: Run date and time: 09/15/2020 - 15:47:40 -> 09/15/2020 - 16:45:54

Dye: Blue - 1 peaks - 55.fsa

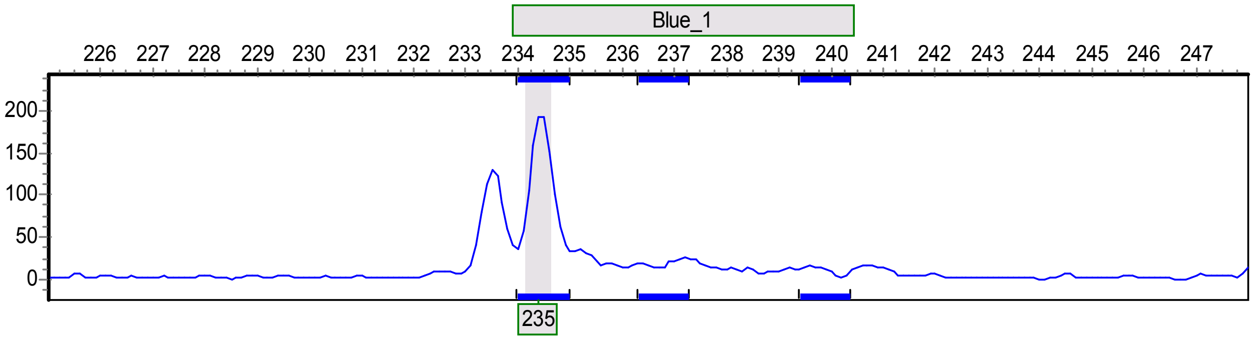

No	Size	Height	Area	Marker	Allele	Difference	Quality	Score	Allele Comments	Sample Comments
1	234.4	192	1060	Blue_1	235	0.1	Pass	12.8		

Sample 52: Run date and time: 09/15/2020 - 15:47:40 -> 09/15/2020 - 16:45:54

Dye: Blue - 1 peaks - 56.fsa

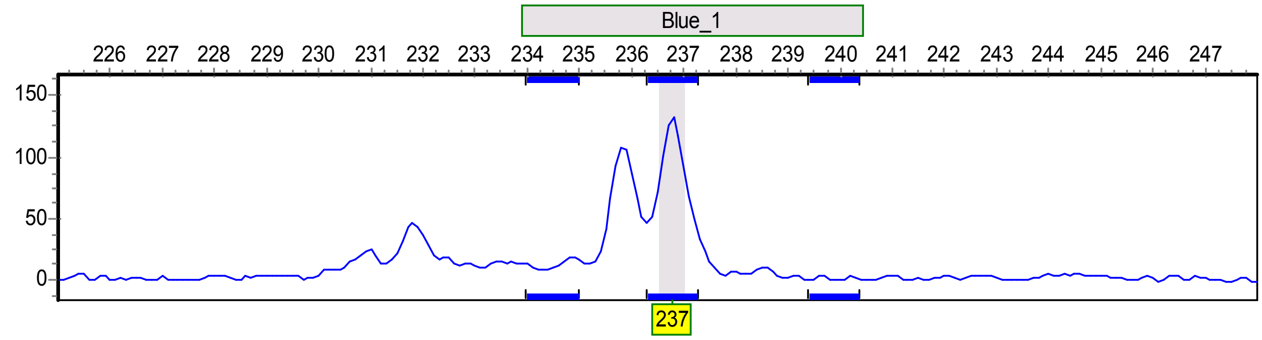

No	Size	Height	Area	Marker	Allele	Difference	Quality	Score	Allele Comments	Sample Comments
1	236.8	133	847	Blue_1	237	0.0	Check	4.8		

Sample 53: Run date and time: 09/15/2020 - 15:47:40 -> 09/15/2020 - 16:45:54

Dye: Blue - 1 peaks - 57.fsa

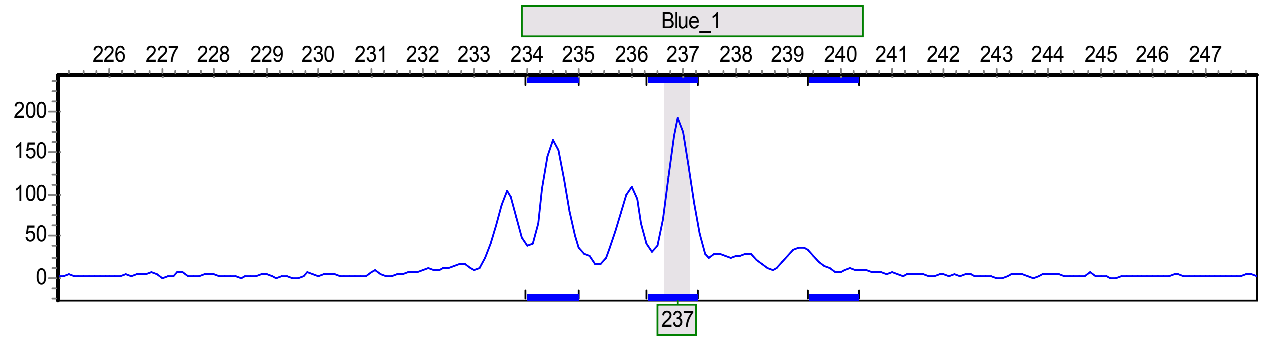

No	Size	Height	Area	Marker	Allele	Difference	Quality	Score	Allele Comments	Sample Comments
1	236.9	191	1018	Blue_1	237	0.1	Pass	13.2		

Sample 54: Run date and time: 09/15/2020 - 15:47:40 -> 09/15/2020 - 16:45:54

Dye: Blue - 1 peaks - 58.fsa

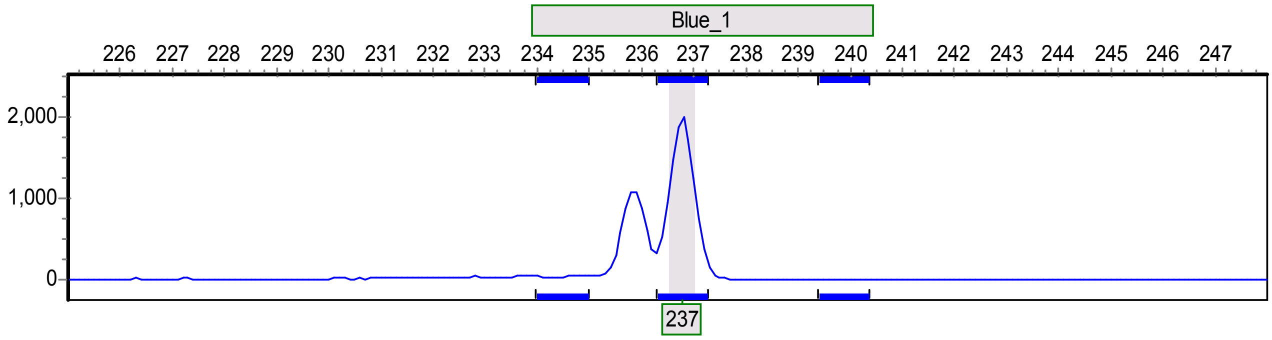

No	Size	Height	Area	Marker	Allele	Difference	Quality	Score	Allele Comments	Sample Comments
1	236.8	1980	10647	Blue_1	237	0.0	Pass	500.0		

Sample 55: Run date and time: 09/15/2020 - 15:47:40 -> 09/15/2020 - 16:45:54

Dye: Blue - 2 peaks - 59.fsa

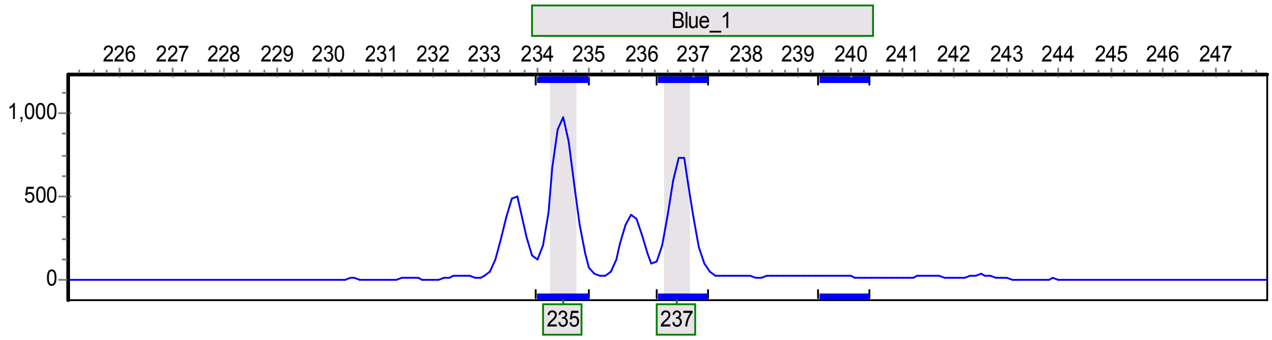

No	Size	Height	Area	Marker	Allele	Difference	Quality	Score	Allele Comments	Sample Comments
1	234.5	975	4873	Blue_1	235	0.0	Pass	201.2		
2	236.7	736	3847	Blue_1	237	0.1	Pass	123.6		

Sample 56: Run date and time: 09/15/2020 - 15:47:40 -> 09/15/2020 - 16:45:54

Dye: Blue - 1 peaks - 6.fsa

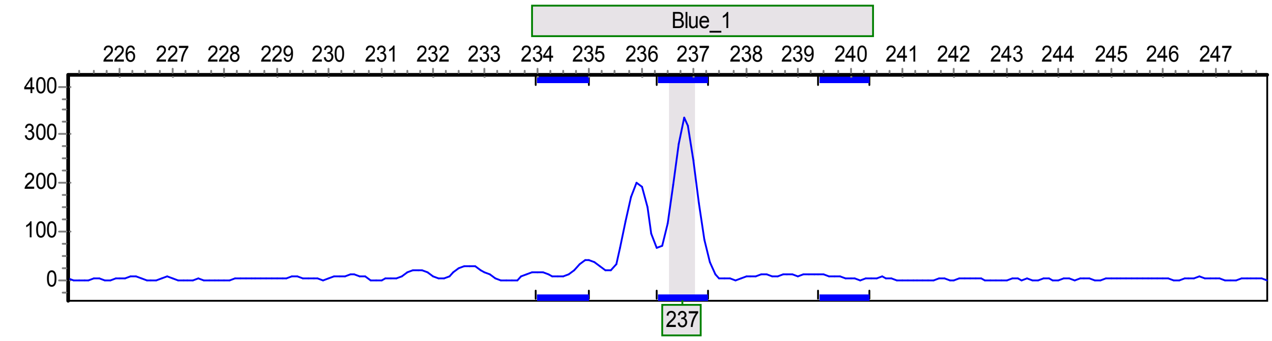

No	Size	Height	Area	Marker	Allele	Difference	Quality	Score	Allele Comments	Sample Comments
1	236.8	333	1712	Blue_1	237	0.0	Pass	34.0		

Sample 57: Run date and time: 09/15/2020 - 15:47:40 -> 09/15/2020 - 16:45:54

Dye: Blue - 0 peaks - 60.fsa

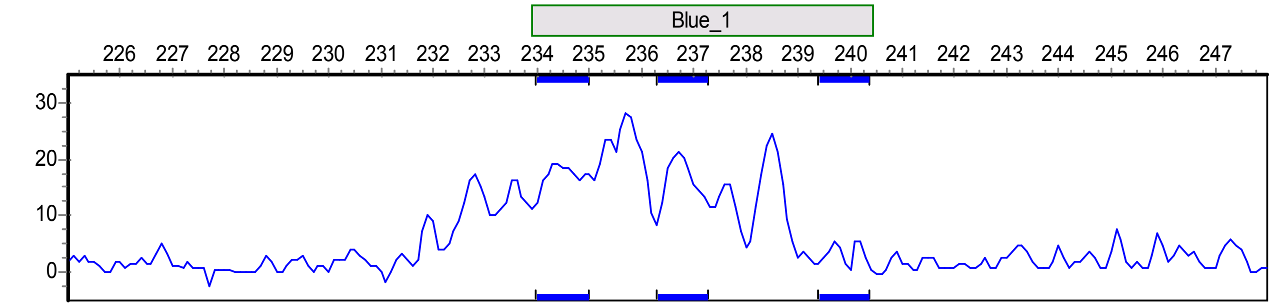

No	Size	Height	Area	Marker	Allele	Difference	Quality	Score	Allele Comments	Sample Comments

Sample 58: Run date and time: 09/15/2020 - 15:47:40 -> 09/15/2020 - 16:45:54

Dye: Blue - 1 peaks - 61.fsa

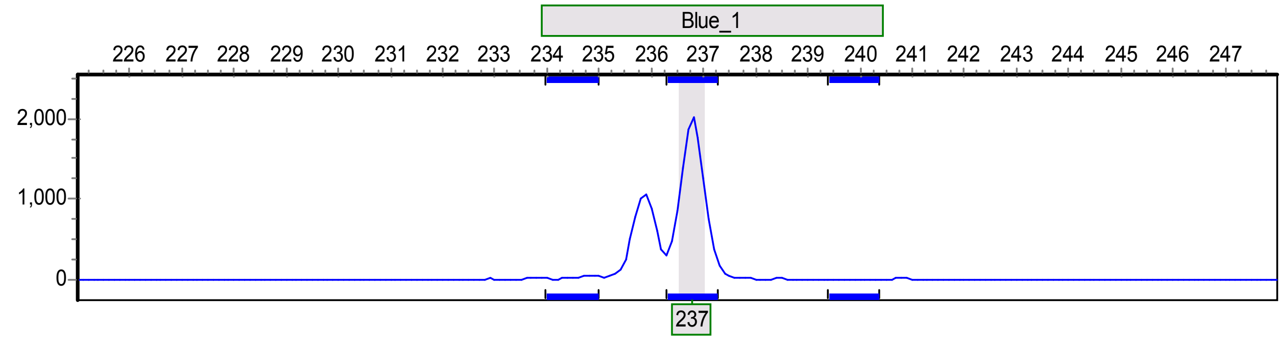

No	Size	Height	Area	Marker	Allele	Difference	Quality	Score	Allele Comments	Sample Comments
1	236.8	2012	10548	Blue_1	237	0.0	Pass	500.0		

Sample 59: Run date and time: 09/15/2020 - 15:47:40 -> 09/15/2020 - 16:45:54

Dye: Blue - 1 peaks - 62.fsa

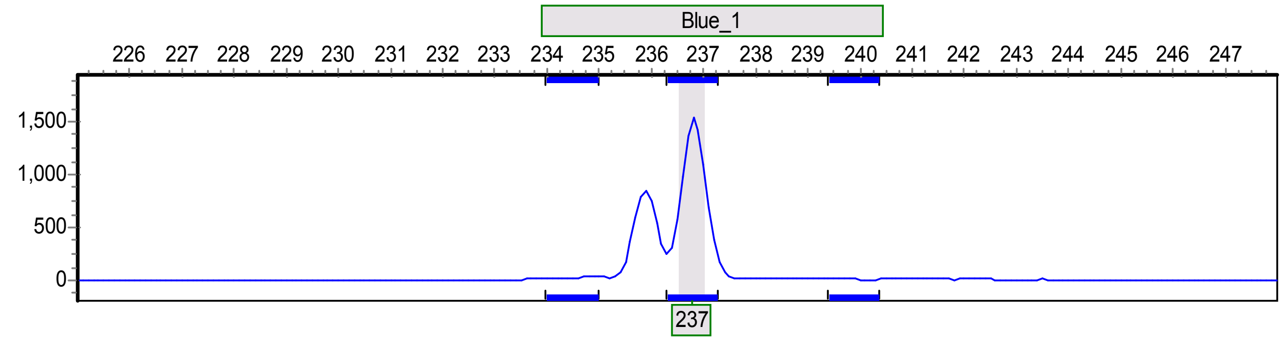

No	Size	Height	Area	Marker	Allele	Difference	Quality	Score	Allele Comments	Sample Comments
1	236.8	1527	8102	Blue_1	237	0.0	Pass	351.6		

Sample 60: Run date and time: 09/15/2020 - 15:47:40 -> 09/15/2020 - 16:45:54

Dye: Blue - 1 peaks - 63.fsa

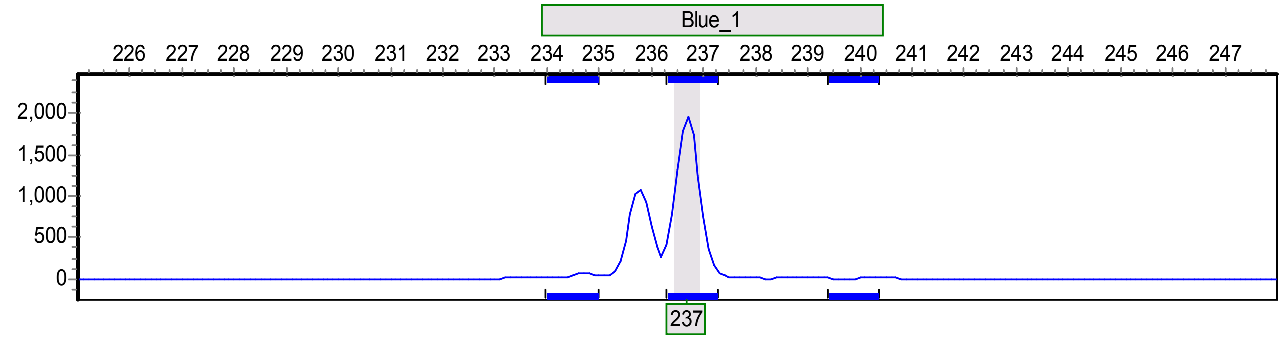

No	Size	Height	Area	Marker	Allele	Difference	Quality	Score	Allele Comments	Sample Comments
1	236.7	1950	9970	Blue_1	237	0.1	Pass	500.0		

Sample 61: Run date and time: 09/15/2020 - 15:47:40 -> 09/15/2020 - 16:45:54

Dye: Blue - 1 peaks - 64.fsa

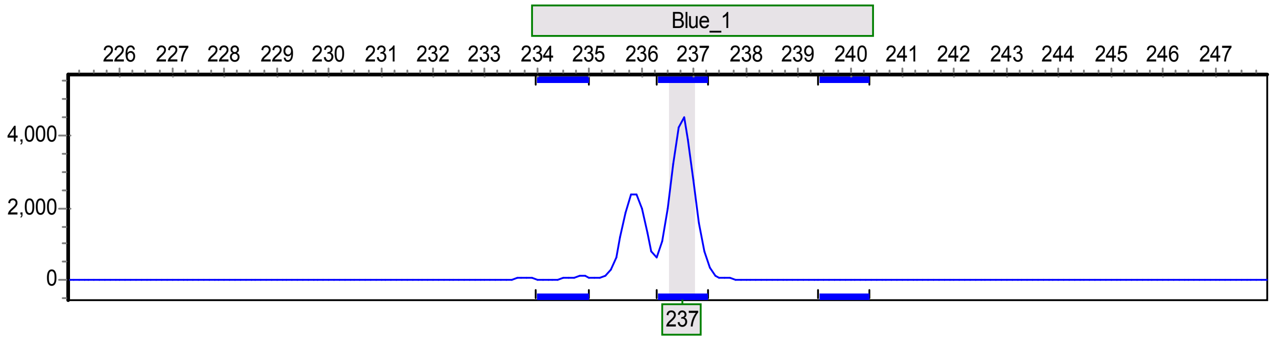

No	Size	Height	Area	Marker	Allele	Difference	Quality	Score	Allele Comments	Sample Comments
1	236.8	4497	23349	Blue_1	237	0.0	Pass	500.0		

Sample 62: Run date and time: 09/15/2020 - 15:47:40 -> 09/15/2020 - 16:45:54

Dye: Blue - 1 peaks - 65.fsa

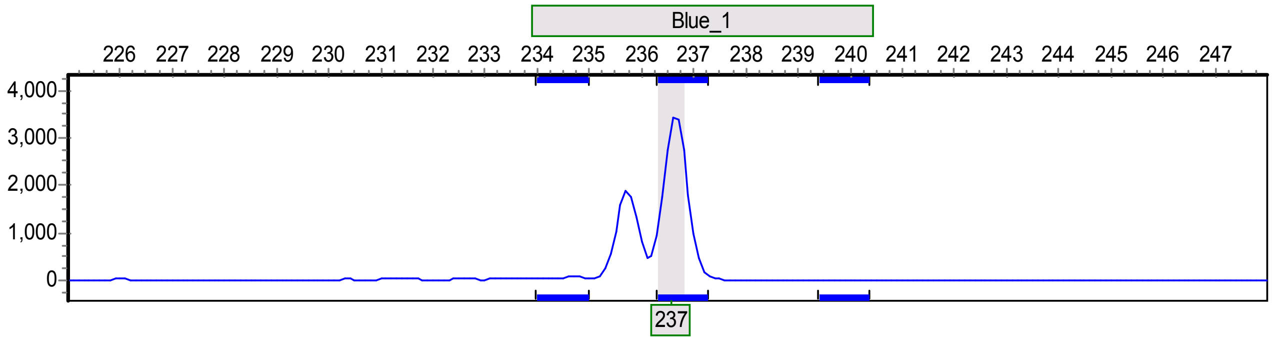

No	Size	Height	Area	Marker	Allele	Difference	Quality	Score	Allele Comments	Sample Comments
1	236.6	3407	17770	Blue_1	237	0.2	Pass	500.0		

Sample 63: Run date and time: 09/15/2020 - 15:47:40 -> 09/15/2020 - 16:45:54

Dye: Blue - 1 peaks - 66.fsa

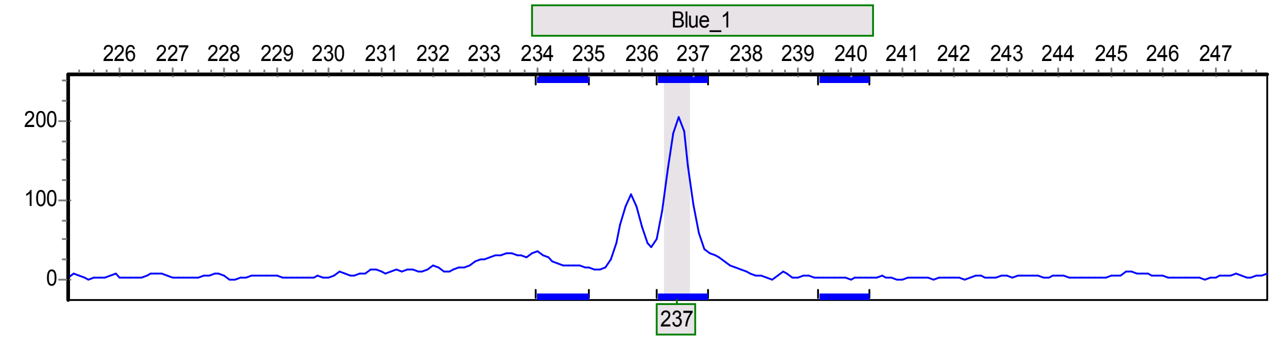

No	Size	Height	Area	Marker	Allele	Difference	Quality	Score	Allele Comments	Sample Comments
1	236.7	204	1162	Blue_1	237	0.1	Pass	13.8		

Sample 64: Run date and time: 09/15/2020 - 15:47:40 -> 09/15/2020 - 16:45:54

Dye: Blue - 1 peaks - 67.fsa

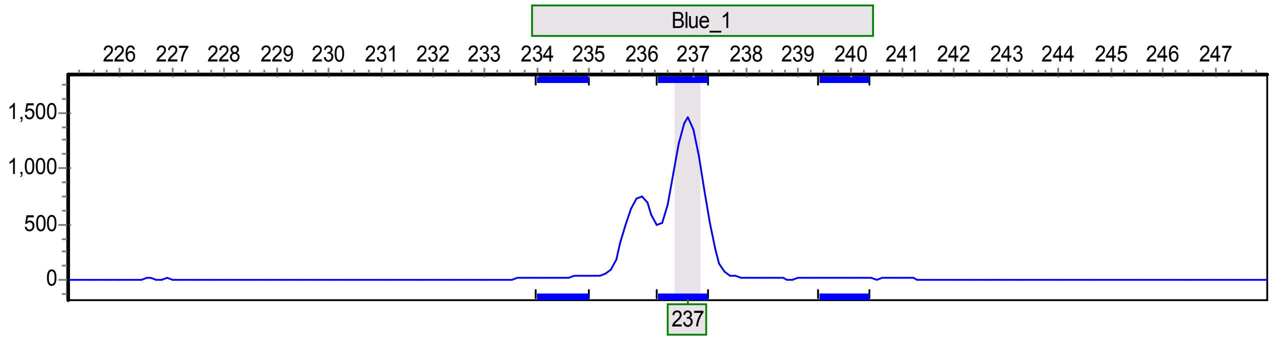

No	Size	Height	Area	Marker	Allele	Difference	Quality	Score	Allele Comments	Sample Comments
1	236.9	1448	10097	Blue_1	237	0.1	Pass	202.9		

Sample 65: Run date and time: 09/15/2020 - 15:47:40 -> 09/15/2020 - 16:45:54

Dye: Blue - 1 peaks - 68.fsa

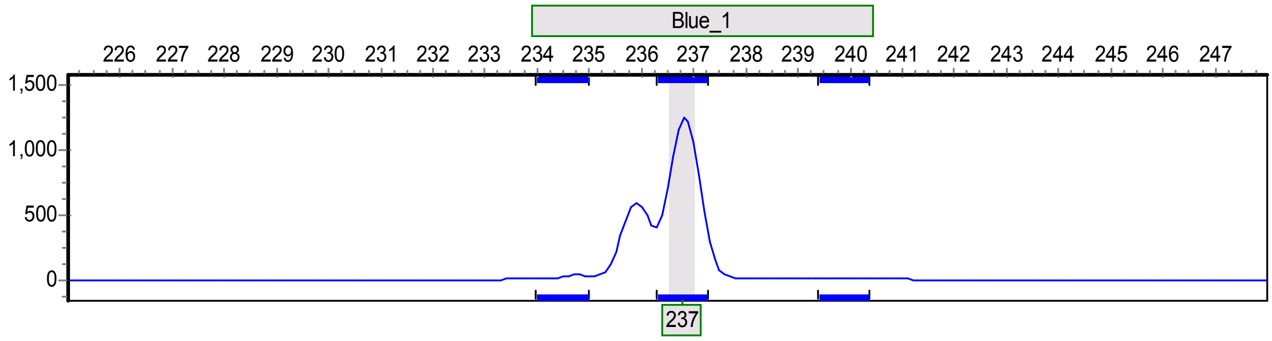

No	Size	Height	Area	Marker	Allele	Difference	Quality	Score	Allele Comments	Sample Comments
1	236.8	1241	8356	Blue_1	237	0.0	Pass	158.7		

Sample 66: Run date and time: 09/15/2020 - 15:47:40 -> 09/15/2020 - 16:45:54

Dye: Blue - 1 peaks - 69.fsa

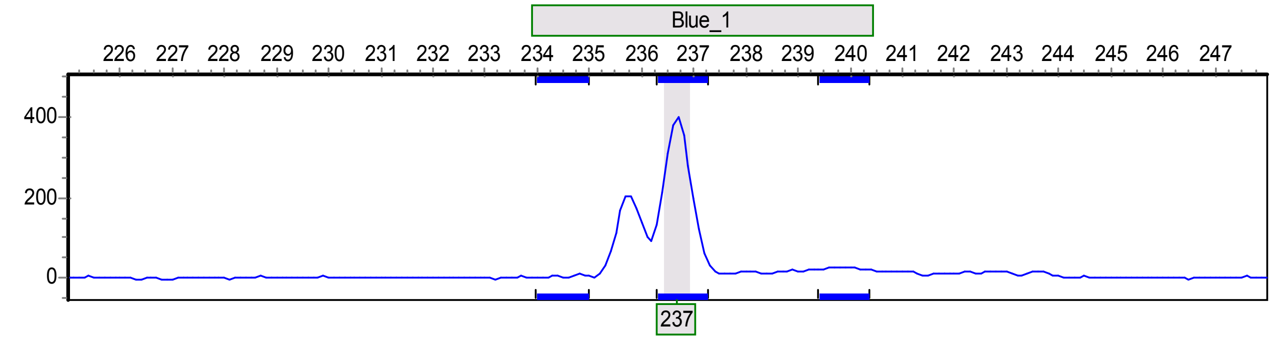

No	Size	Height	Area	Marker	Allele	Difference	Quality	Score	Allele Comments	Sample Comments
1	236.7	398	2393	Blue_1	237	0.1	Pass	35.6		

Sample 67: Run date and time: 09/15/2020 - 15:47:40 -> 09/15/2020 - 16:45:54

Dye: Blue - 0 peaks - 7.fsa

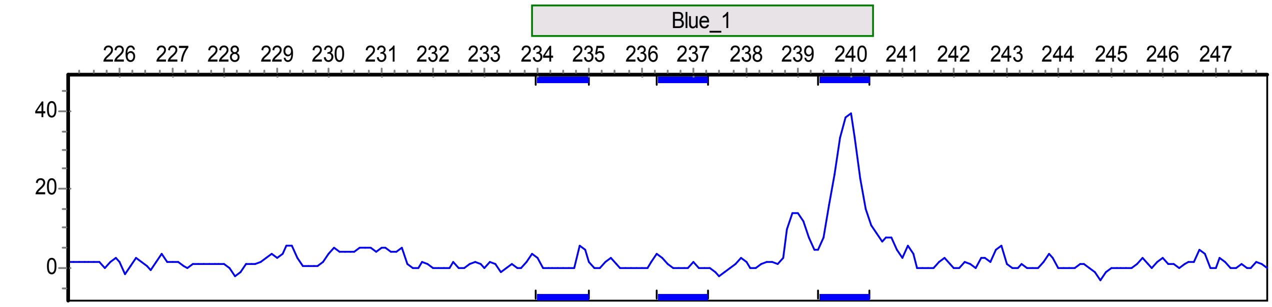

No	Size	Height	Area	Marker	Allele	Difference	Quality	Score	Allele Comments	Sample Comments

Sample 68: Run date and time: 09/15/2020 - 15:47:40 -> 09/15/2020 - 16:45:54

Dye: Blue - 1 peaks - 70.fsa

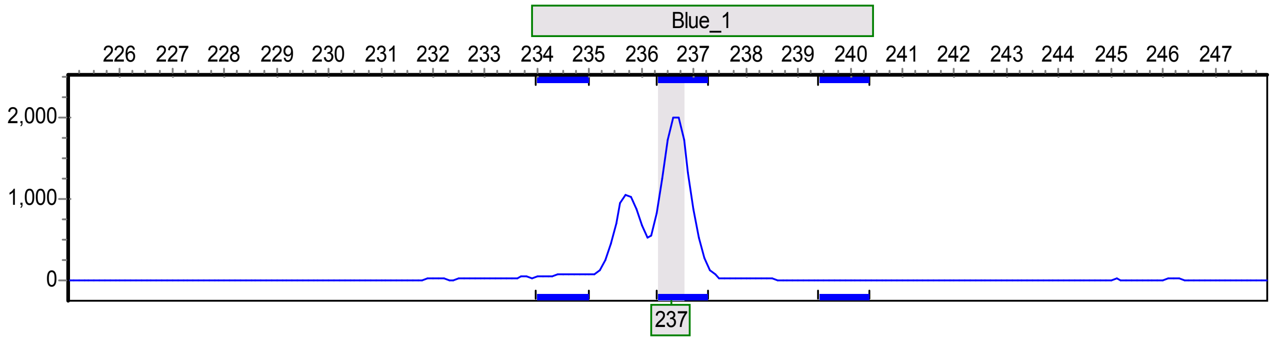

No	Size	Height	Area	Marker	Allele	Difference	Quality	Score	Allele Comments	Sample Comments
1	236.6	1982	12086	Blue_1	237	0.2	Pass	372.6		

Sample 69: Run date and time: 09/15/2020 - 15:47:40 -> 09/15/2020 - 16:45:54

Dye: Blue - 1 peaks - 71.fsa

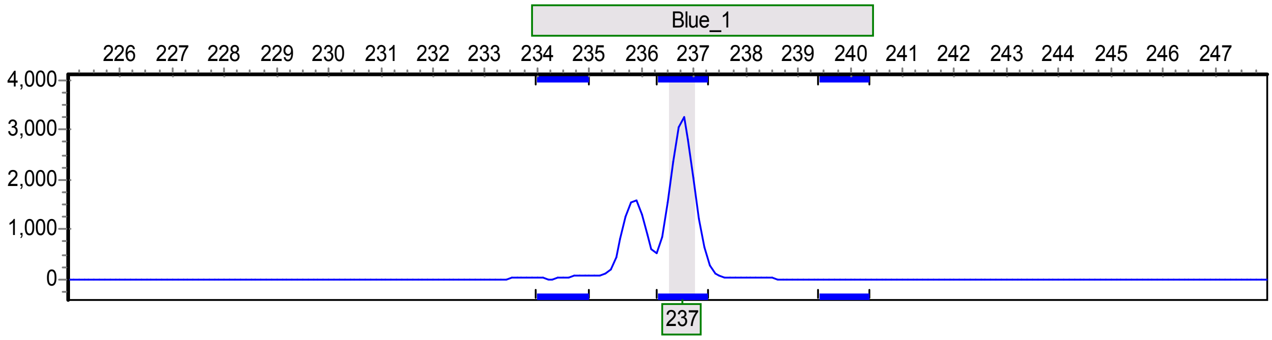

No	Size	Height	Area	Marker	Allele	Difference	Quality	Score	Allele Comments	Sample Comments
1	236.8	3240	17576	Blue_1	237	0.0	Pass	500.0		

Allele Report

Sample 70: Run date and time: 09/15/2020 - 15:47:40 -> 09/15/2020 - 16:45:54

Dye: Blue - 1 peaks - 72.fsa

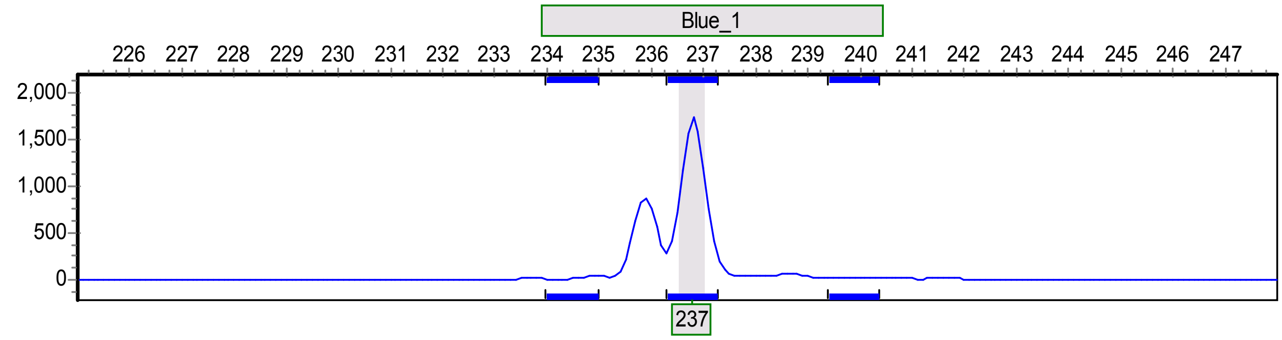

No	Size	Height	Area	Marker	Allele	Difference	Quality	Score	Allele Comments	Sample Comments
1	236.8	1722	9420	Blue_1	237	0.0	Pass	402.2		

Sample 71: Run date and time: 09/15/2020 - 15:47:40 -> 09/15/2020 - 16:45:54

Dye: Blue - 1 peaks - 73.fsa

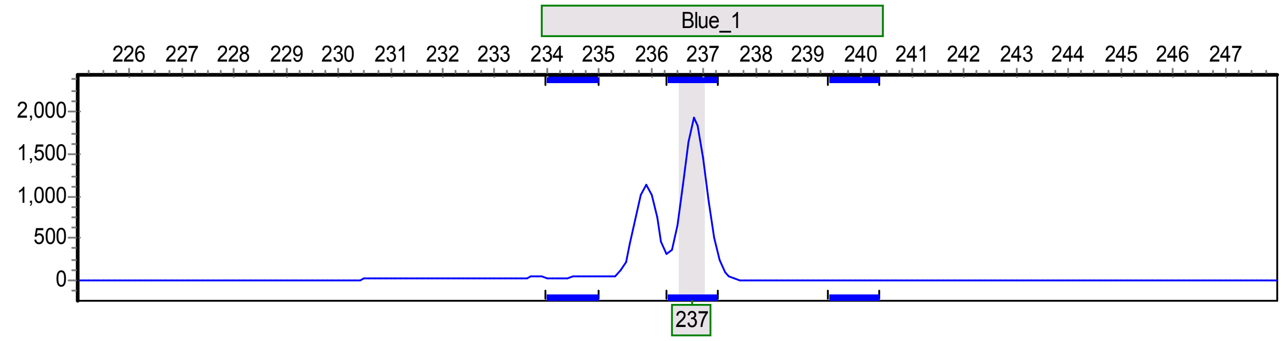

No	Size	Height	Area	Marker	Allele	Difference	Quality	Score	Allele Comments	Sample Comments
1	236.8	1921	10191	Blue_1	237	0.0	Pass	481.5		

Sample 72: Run date and time: 09/15/2020 - 15:47:40 -> 09/15/2020 - 16:45:54

Dye: Blue - 1 peaks - 74.fsa

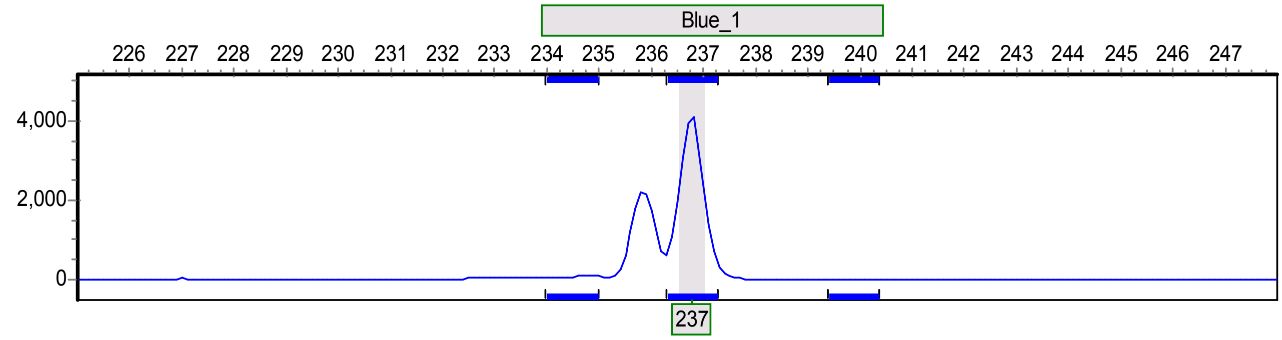

No	Size	Height	Area	Marker	Allele	Difference	Quality	Score	Allele Comments	Sample Comments
1	236.8	4055	21558	Blue_1	237	0.0	Pass	500.0		

Sample 73: Run date and time: 09/15/2020 - 15:47:40 -> 09/15/2020 - 16:45:54

Dye: Blue - 2 peaks - 75.fsa

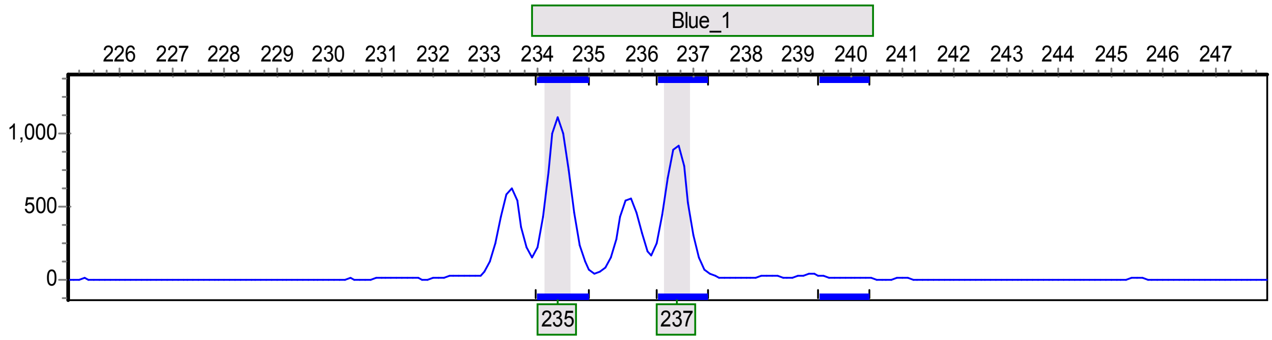

No	Size	Height	Area	Marker	Allele	Difference	Quality	Score	Allele Comments	Sample Comments
1	234.4	1109	5768	Blue_1	235	0.1	Pass	227.2		
2	236.7	916	4881	Blue_1	237	0.1	Pass	169.0		

Sample 74: Run date and time: 09/15/2020 - 15:47:40 -> 09/15/2020 - 16:45:54

Dye: Blue - 2 peaks - 76.fsa

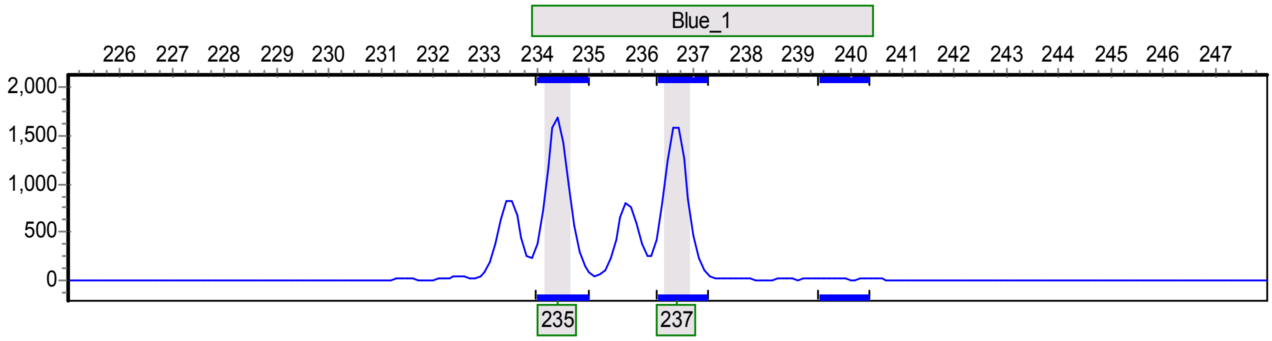

No	Size	Height	Area	Marker	Allele	Difference	Quality	Score	Allele Comments	Sample Comments
1	234.4	1680	8530	Blue_1	235	0.1	Pass	427.8		
2	236.7	1586	8240	Blue_1	237	0.1	Pass	385.3		

Sample 75: Run date and time: 09/15/2020 - 15:47:40 -> 09/15/2020 - 16:45:54

Dye: Blue - 1 peaks - 77.fsa

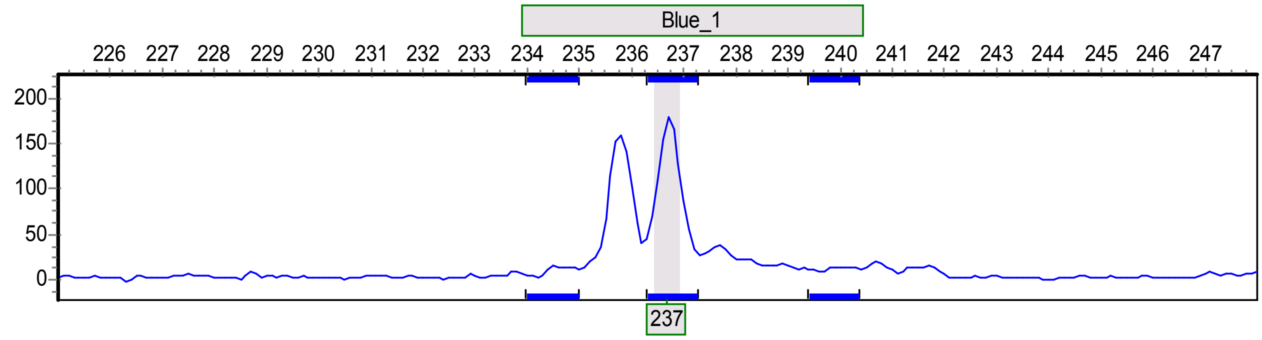

No	Size	Height	Area	Marker	Allele	Difference	Quality	Score	Allele Comments	Sample Comments
1	236.7	179	957	Blue_1	237	0.1	Pass	10.8		

Sample 76: Run date and time: 09/15/2020 - 15:47:40 -> 09/15/2020 - 16:45:54

Dye: Blue - 0 peaks - 78.fsa

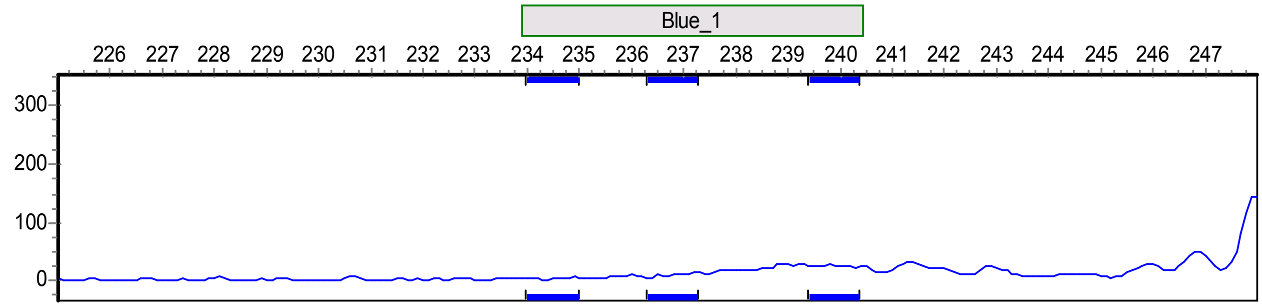

No	Size	Height	Area	Marker	Allele	Difference	Quality	Score	Allele Comments	Sample Comments

Sample 77: Run date and time: 09/15/2020 - 15:47:40 -> 09/15/2020 - 16:45:54

Dye: Blue - 1 peaks - 79.fsa

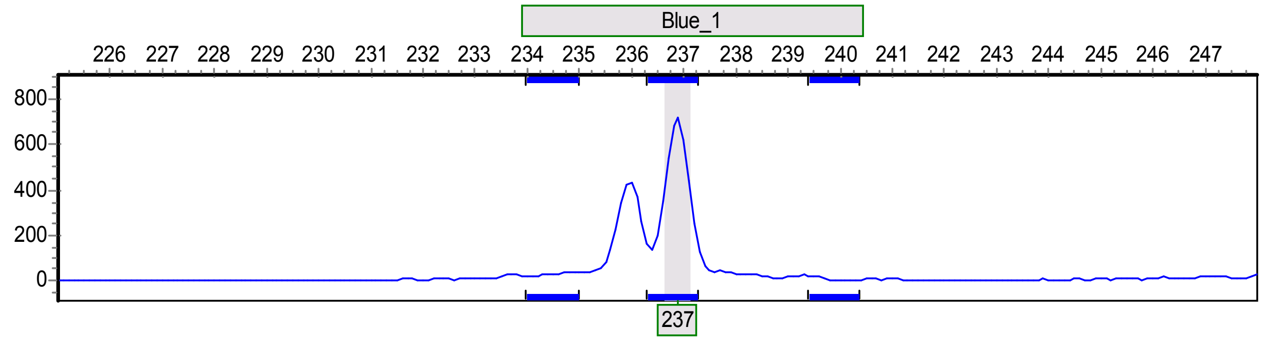

No	Size	Height	Area	Marker	Allele	Difference	Quality	Score	Allele Comments	Sample Comments
1	236.9	715	3903	Blue_1	237	0.1	Pass	113.1		

Sample 78: Run date and time: 09/15/2020 - 15:47:40 -> 09/15/2020 - 16:45:54

Dye: Blue - 0 peaks - 8.fsa

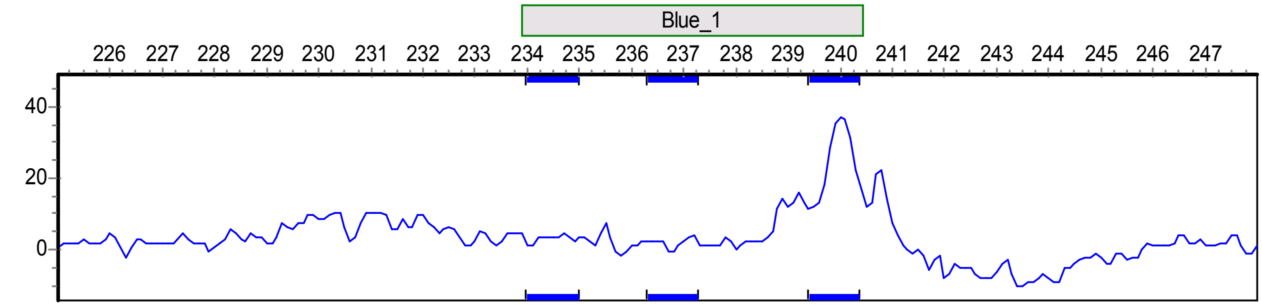

No	Size	Height	Area	Marker	Allele	Difference	Quality	Score	Allele Comments	Sample Comments

Sample 79: Run date and time: 09/15/2020 - 15:47:40 -> 09/15/2020 - 16:45:54

Dye: Blue - 1 peaks - 80.fsa

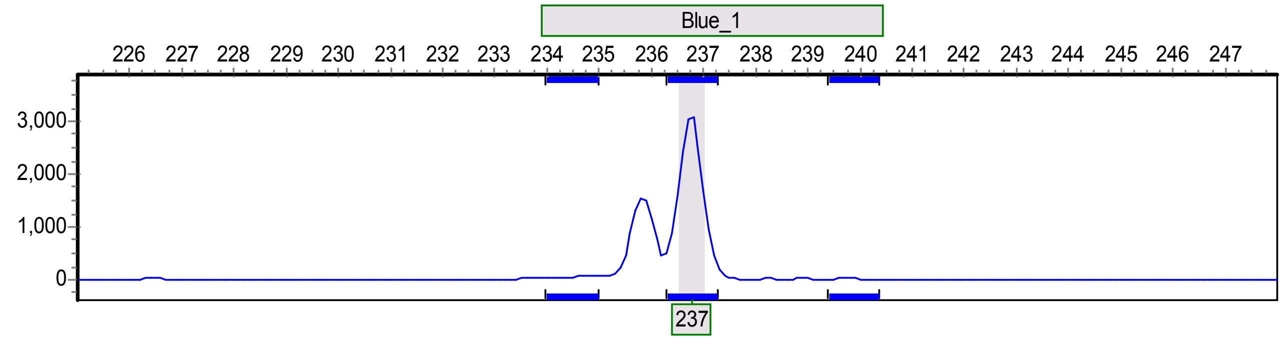

No	Size	Height	Area	Marker	Allele	Difference	Quality	Score	Allele Comments	Sample Comments
1	236.8	3045	16183	Blue_1	237	0.0	Pass	500.0		

Sample 80: Run date and time: 09/15/2020 - 15:47:40 -> 09/15/2020 - 16:45:54

Dye: Blue - 2 peaks - 81.fsa

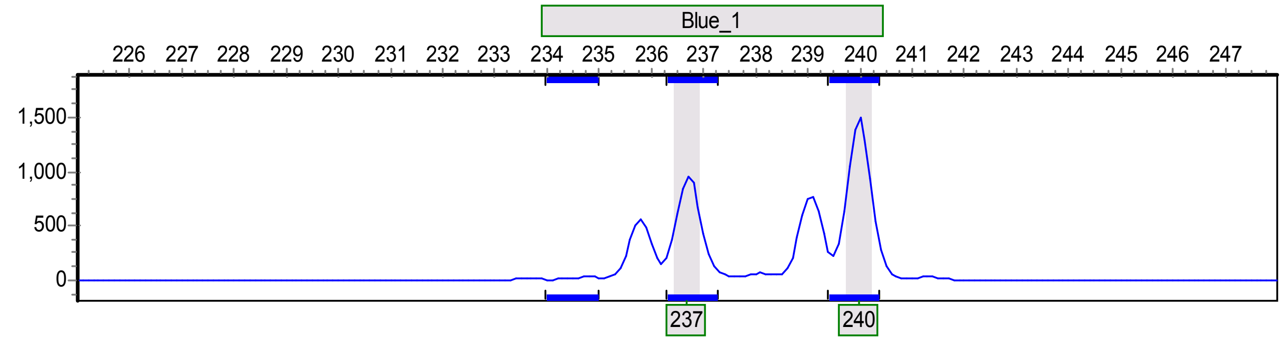

No	Size	Height	Area	Marker	Allele	Difference	Quality	Score	Allele Comments	Sample Comments
1	236.7	968	5192	Blue_1	237	0.1	Pass	179.9		
2	240.0	1498	7808	Blue_1	240	0.1	Pass	353.3		

Sample 81: Run date and time: 09/15/2020 - 15:47:40 -> 09/15/2020 - 16:45:54

Dye: Blue - 0 peaks - 82.fsa

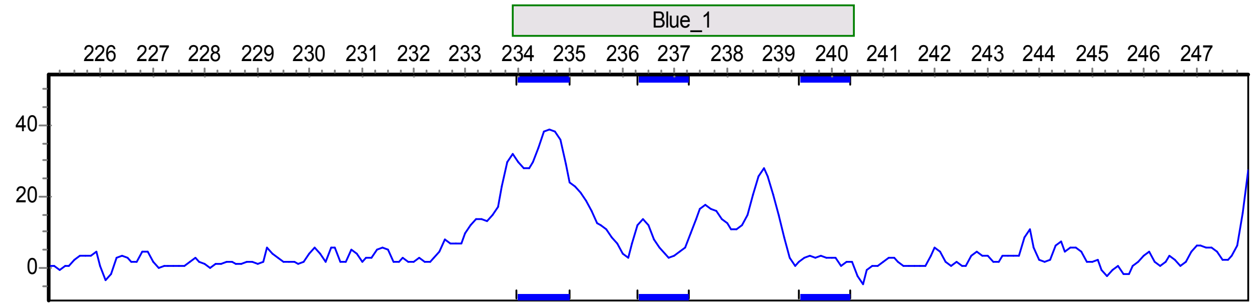

No	Size	Height	Area	Marker	Allele	Difference	Quality	Score	Allele Comments	Sample Comments

Sample 82: Run date and time: 09/15/2020 - 15:47:40 -> 09/15/2020 - 16:45:54

Dye: Blue - 1 peaks - 83.fsa

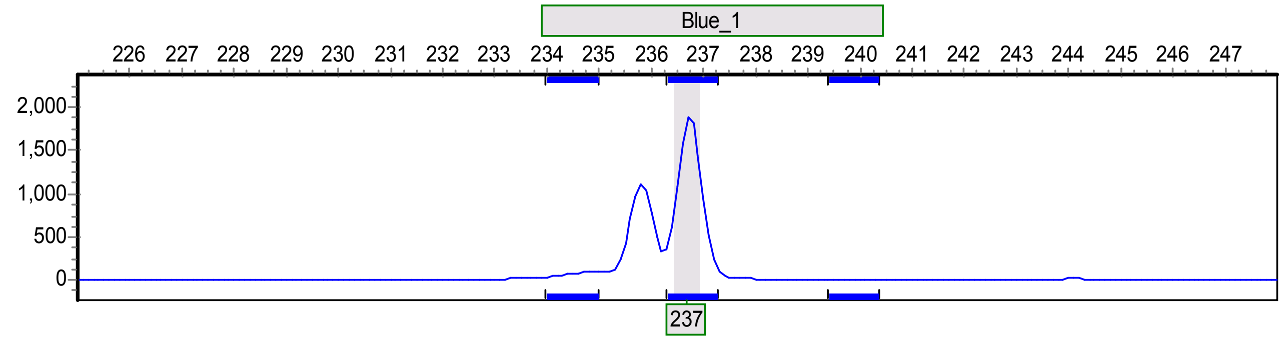

No	Size	Height	Area	Marker	Allele	Difference	Quality	Score	Allele Comments	Sample Comments
1	236.7	1873	9991	Blue_1	237	0.1	Pass	462.0		

Sample 83: Run date and time: 09/15/2020 - 15:47:40 -> 09/15/2020 - 16:45:54

Dye: Blue - 1 peaks - 84.fsa

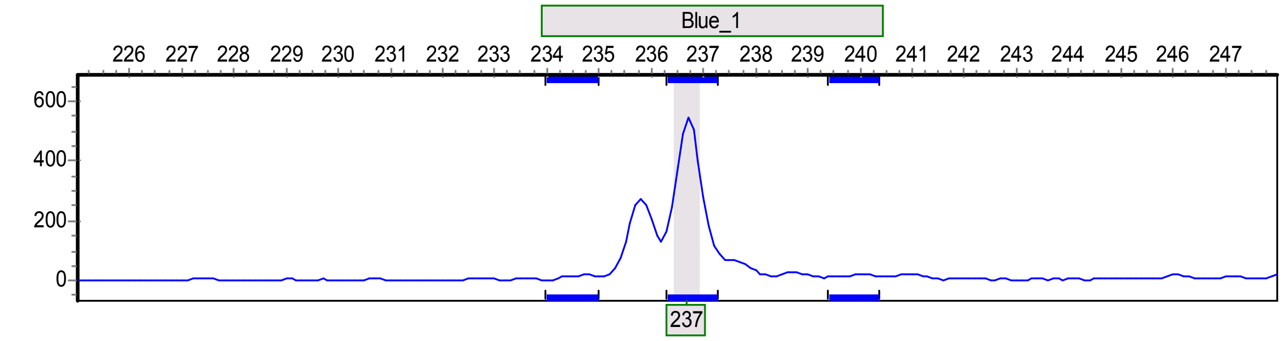

No	Size	Height	Area	Marker	Allele	Difference	Quality	Score	Allele Comments	Sample Comments
1	236.7	543	3272	Blue_1	237	0.1	Pass	61.7		

Sample 84: Run date and time: 09/15/2020 - 15:47:40 -> 09/15/2020 - 16:45:54

Dye: Blue - 1 peaks - 85.fsa

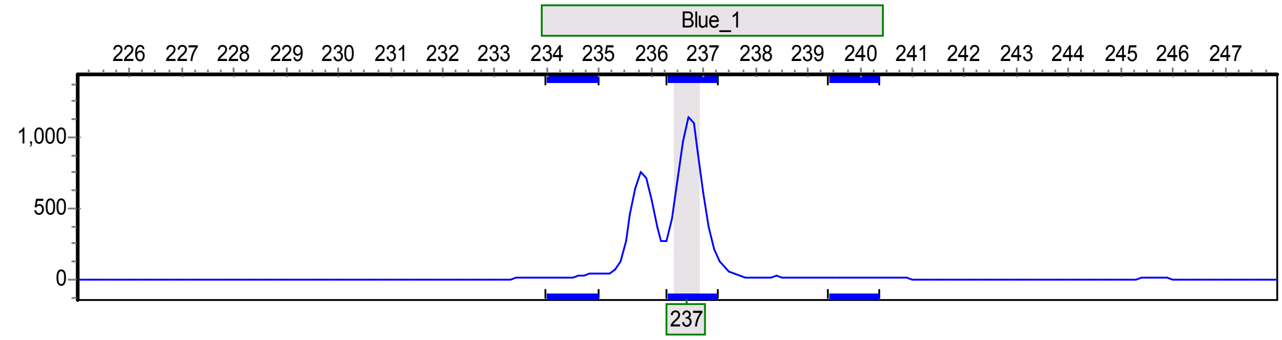

No	Size	Height	Area	Marker	Allele	Difference	Quality	Score	Allele Comments	Sample Comments
1	236.7	1129	6241	Blue_1	237	0.1	Pass	201.3		

Allele Report

Sample 85: Run date and time: 09/15/2020 - 15:47:40 -> 09/15/2020 - 16:45:54

Dye: Blue - 1 peaks - 86.fsa

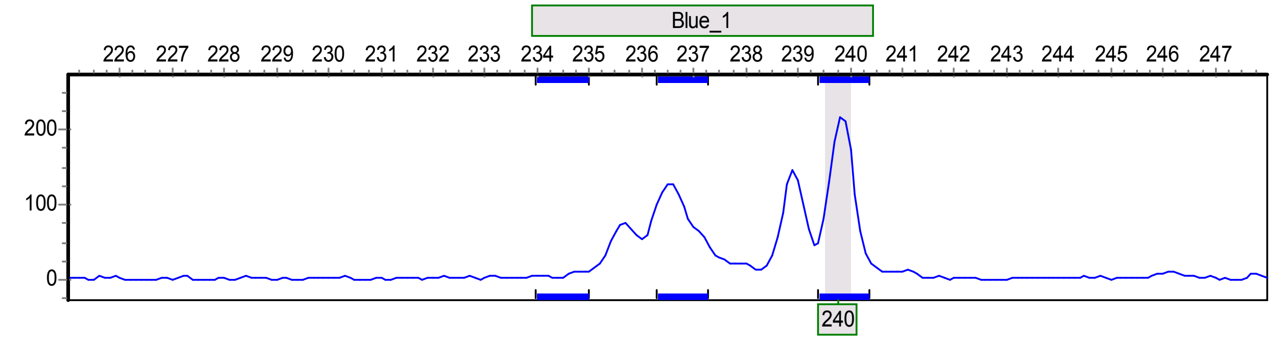

No	Size	Height	Area	Marker	Allele	Difference	Quality	Score	Allele Comments	Sample Comments
1	239.8	216	1165	Blue_1	240	0.1	Pass	14.9		

Sample 86: Run date and time: 09/15/2020 - 15:47:40 -> 09/15/2020 - 16:45:54

Dye: Blue - 1 peaks - 87.fsa

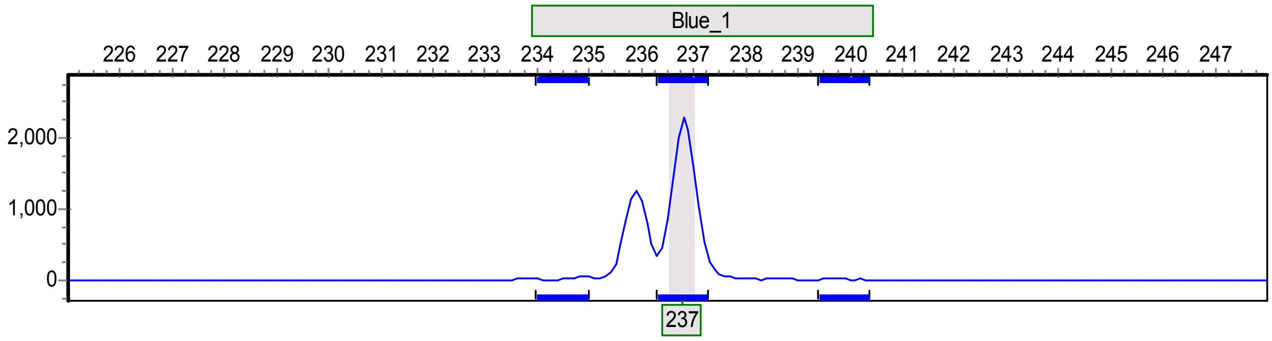

No	Size	Height	Area	Marker	Allele	Difference	Quality	Score	Allele Comments	Sample Comments
1	236.8	2286	12083	Blue_1	237	0.0	Pass	500.0		

Sample 87: Run date and time: 09/15/2020 - 15:47:40 -> 09/15/2020 - 16:45:54

Dye: Blue - 1 peaks - 88.fsa

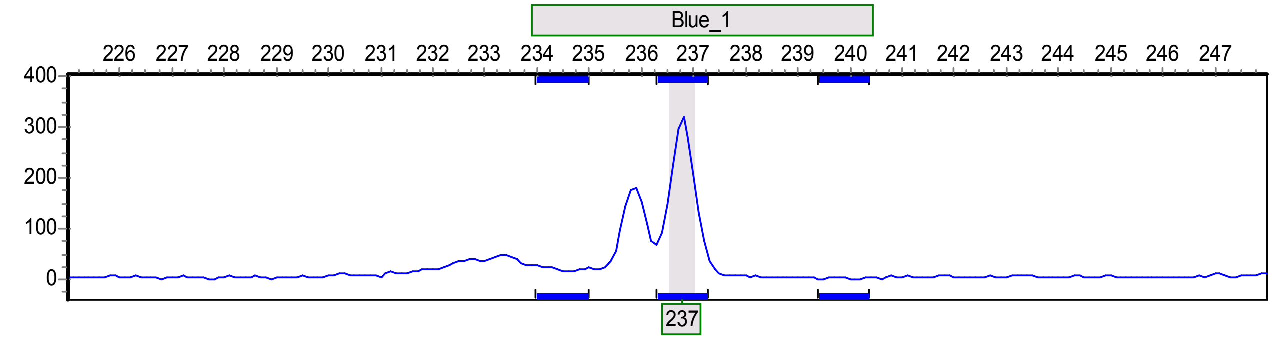

No	Size	Height	Area	Marker	Allele	Difference	Quality	Score	Allele Comments	Sample Comments
1	236.8	319	1742	Blue_1	237	0.0	Pass	30.3		

Sample 88: Run date and time: 09/15/2020 - 15:47:40 -> 09/15/2020 - 16:45:54

Dye: Blue - 1 peaks - 89.fsa

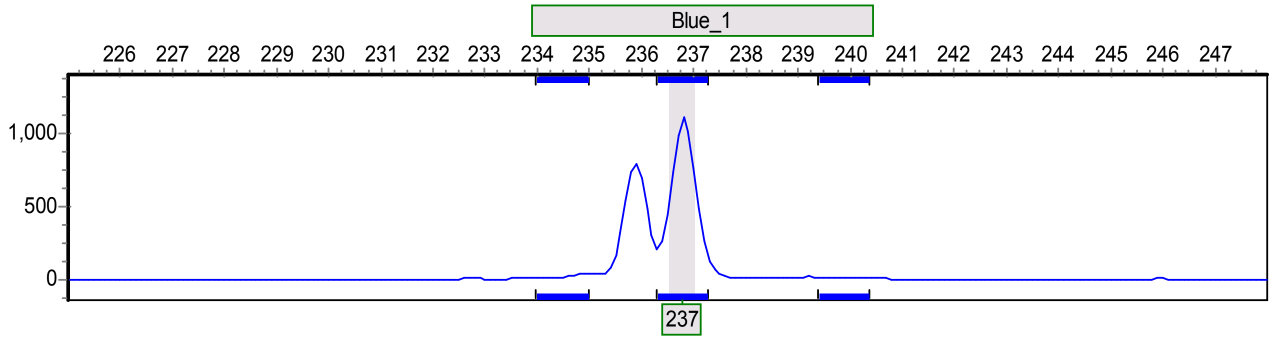

No	Size	Height	Area	Marker	Allele	Difference	Quality	Score	Allele Comments	Sample Comments
1	236.8	1101	6064	Blue_1	237	0.0	Pass	213.9		

Sample 89: Run date and time: 09/15/2020 - 15:47:40 -> 09/15/2020 - 16:45:54

Dye: Blue - 1 peaks - 9.fsa

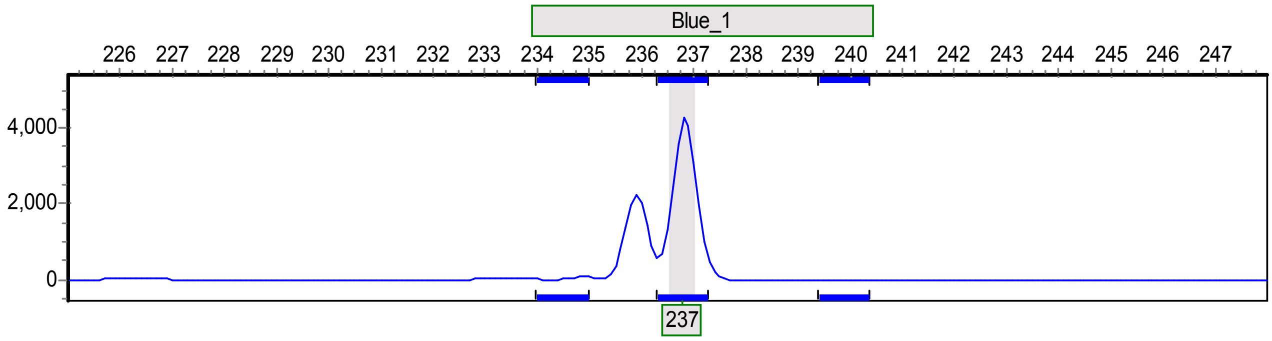

No	Size	Height	Area	Marker	Allele	Difference	Quality	Score	Allele Comments	Sample Comments
1	236.8	4228	21680	Blue_1	237	0.0	Pass	500.0		

Sample 90: Run date and time: 09/15/2020 - 15:47:40 -> 09/15/2020 - 16:45:54

Dye: Blue - 1 peaks - 90.fsa

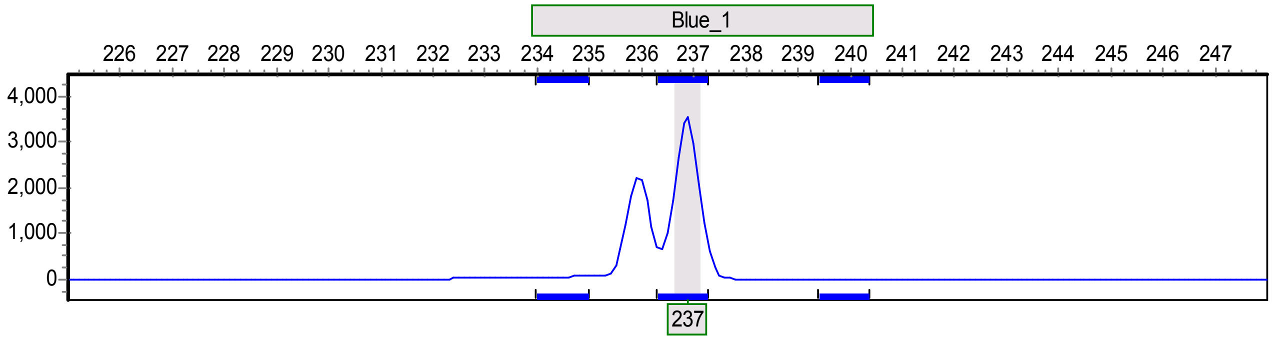

No	Size	Height	Area	Marker	Allele	Difference	Quality	Score	Allele Comments	Sample Comments
1	236.9	3517	19029	Blue_1	237	0.1	Pass	500.0		

Allele Report

Sample 91: Run date and time: 09/15/2020 - 15:47:40 -> 09/15/2020 - 16:45:54

Dye: Blue - 1 peaks - 91.fsa

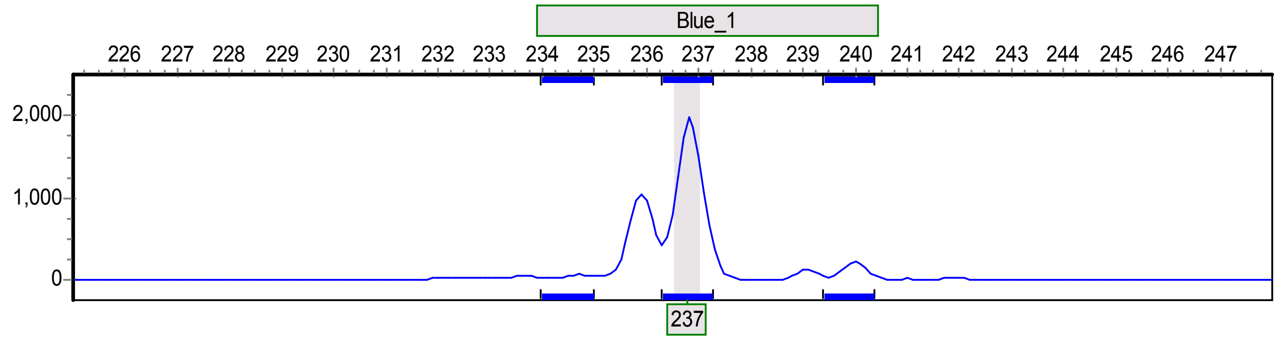

No	Size	Height	Area	Marker	Allele	Difference	Quality	Score	Allele Comments	Sample Comments
1	236.8	1969	11533	Blue_1	237	0.0	Pass	436.3		

Sample 92: Run date and time: 09/15/2020 - 15:47:40 -> 09/15/2020 - 16:45:54

Dye: Blue - 1 peaks - 92.fsa

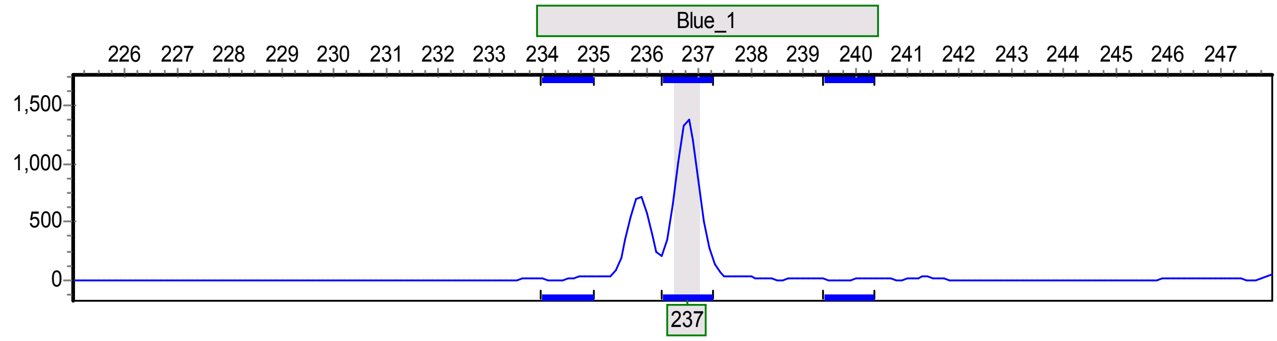

No	Size	Height	Area	Marker	Allele	Difference	Quality	Score	Allele Comments	Sample Comments
1	236.8	1387	7394	Blue_1	237	0.0	Pass	306.2		

Sample 93: Run date and time: 09/15/2020 - 15:47:40 -> 09/15/2020 - 16:45:54

Dye: Blue - 1 peaks - 93.fsa

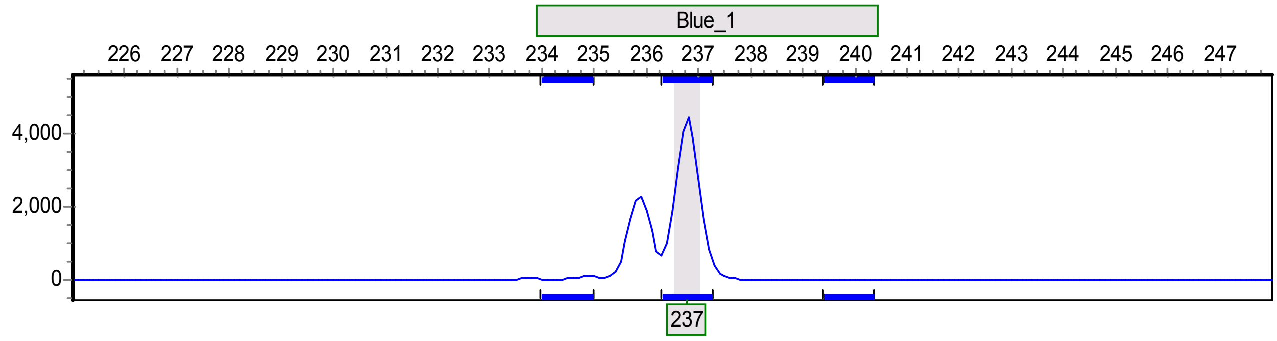

No	Size	Height	Area	Marker	Allele	Difference	Quality	Score	Allele Comments	Sample Comments
1	236.8	4392	22829	Blue_1	237	0.0	Pass	500.0		

Sample 94: Run date and time: 09/15/2020 - 15:47:40 -> 09/15/2020 - 16:45:54

Dye: Blue - 1 peaks - 94.fsa

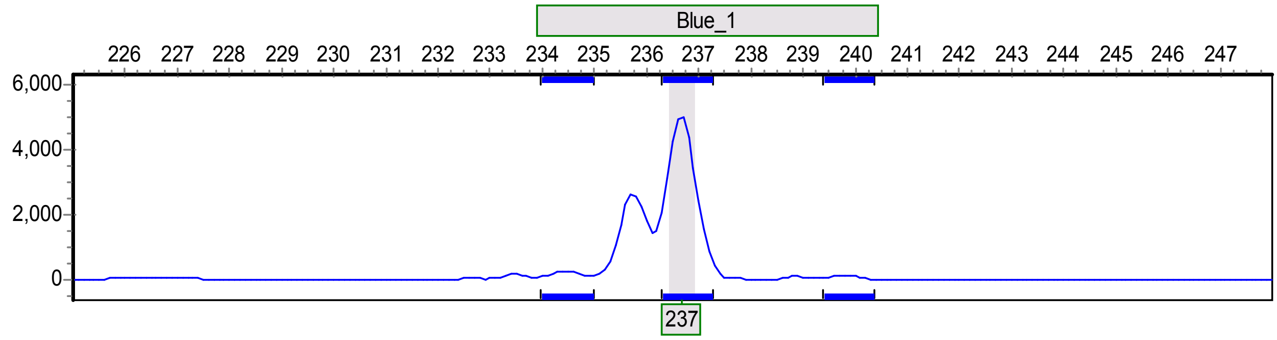

No	Size	Height	Area	Marker	Allele	Difference	Quality	Score	Allele Comments	Sample Comments
1	236.7	4970	32972	Blue_1	237	0.1	Pass	500.0		

Sample 95: Run date and time: 09/15/2020 - 15:47:40 -> 09/15/2020 - 16:45:54

Dye: Blue - 1 peaks - 95.fsa

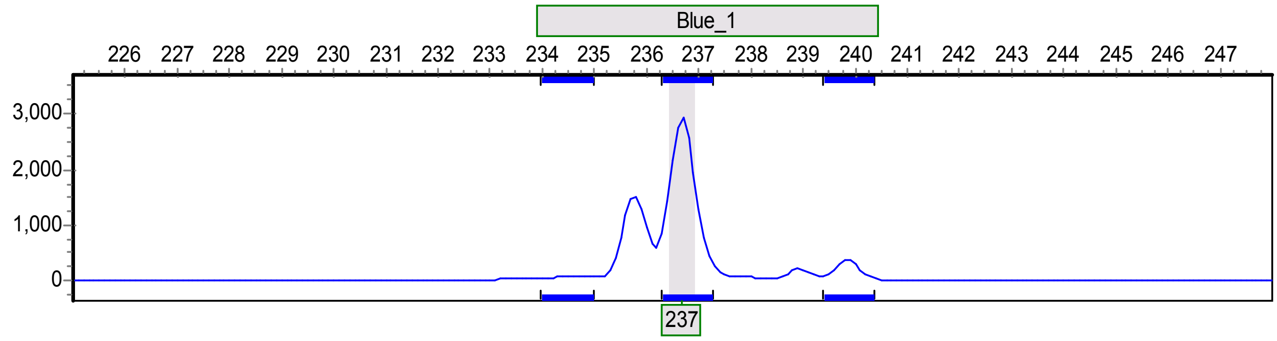

No	Size	Height	Area	Marker	Allele	Difference	Quality	Score	Allele Comments	Sample Comments
1	236.7	2919	16634	Blue_1	237	0.1	Pass	500.0		

Sample 96: Run date and time: 09/15/2020 - 15:47:40 -> 09/15/2020 - 16:45:54

Dye: Blue - 1 peaks - 96.fsa

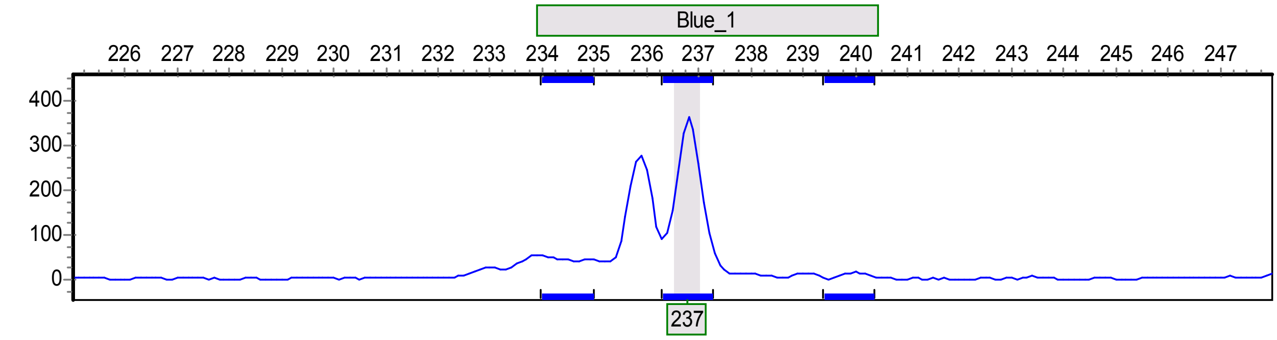

No	Size	Height	Area	Marker	Allele	Difference	Quality	Score	Allele Comments	Sample Comments
1	236.8	363	2077	Blue_1	237	0.0	Pass	34.8		
