# Supplementary material for: Genetic diversity and population structure of Polygonatum cyrtonema Hua in China using SSR markers
Source: PLoS One. 2023 Aug 31;18(8):e0290605. doi: 10.1371/journal.pone.0290605 (PMC10470896; doi:10.1371/journal.pone.0290605)
Supplement: S1 Data — (ZIP) [file pone.0290605.s002.zip › ╨┬╜¿╬─╝■╝╨ (2)/╥2╬∩15/yimwu15.pdf]

Allele Report

Sample 1: Run date and time: 09/11/2020 - 19:30:25 -> 09/11/2020 - 20:26:16

Dye: Blue - 0 peaks - 1.fsa

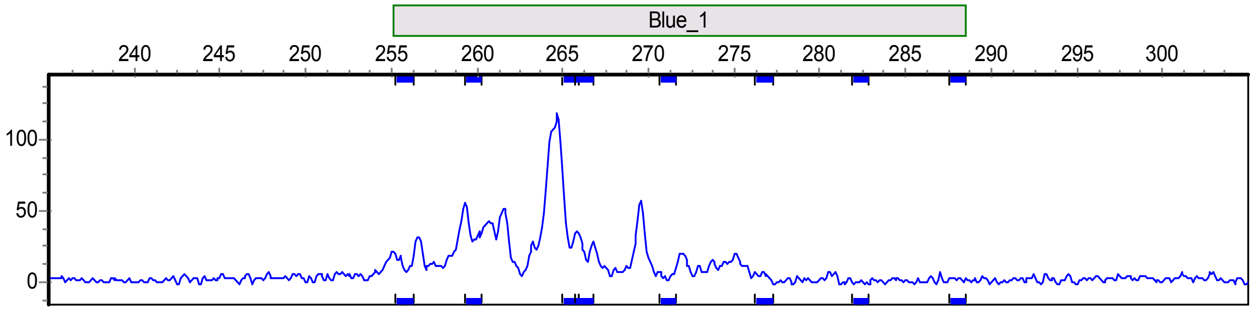

No	Size	Height	Area	Marker	Allele	Difference	Quality	Score	Allele Comments	Sample Comments

Sample 2: Run date and time: 09/11/2020 - 19:30:25 -> 09/11/2020 - 20:26:16

Dye: Blue - 0 peaks - 10.fsa

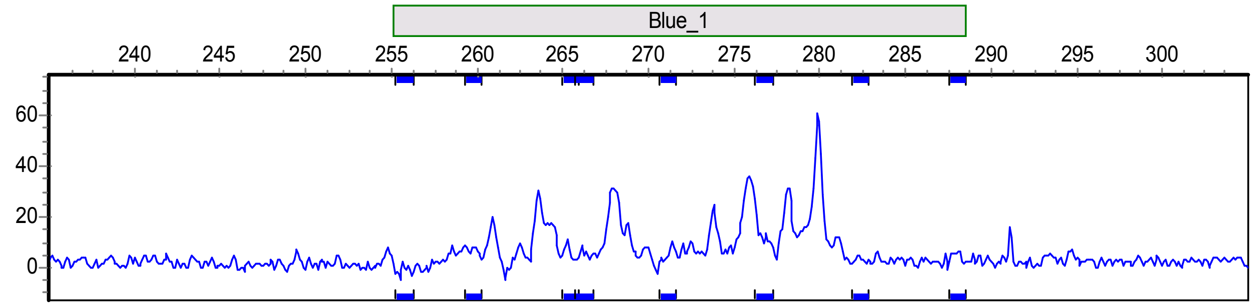

No	Size	Height	Area	Marker	Allele	Difference	Quality	Score	Allele Comments	Sample Comments

Sample 3: Run date and time: 09/11/2020 - 19:30:25 -> 09/11/2020 - 20:26:16

Dye: Blue - 0 peaks - 11.fsa

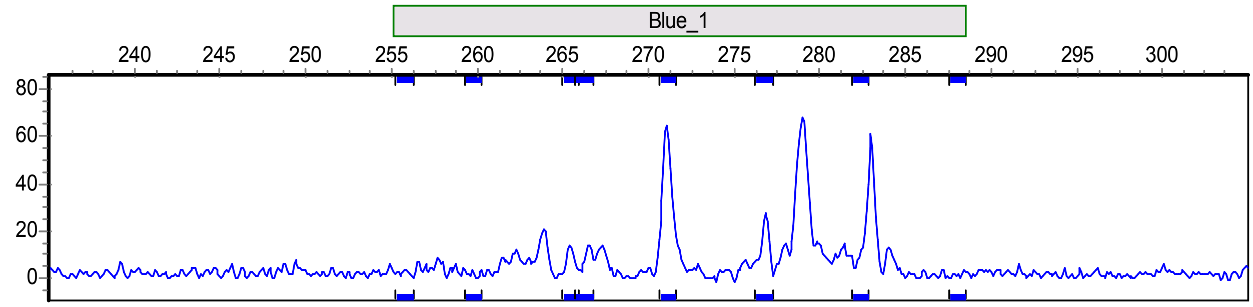

No	Size	Height	Area	Marker	Allele	Difference	Quality	Score	Allele Comments	Sample Comments

Sample 4: Run date and time: 09/11/2020 - 19:30:25 -> 09/11/2020 - 20:26:16

Dye: Blue - 0 peaks - 12.fsa

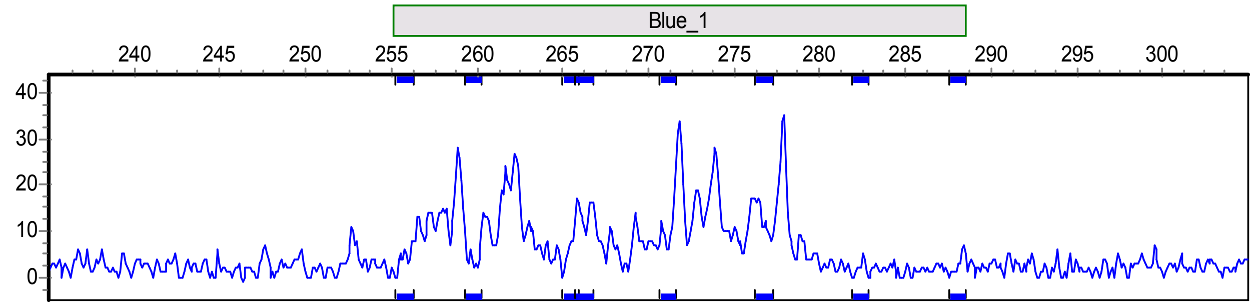

No	Size	Height	Area	Marker	Allele	Difference	Quality	Score	Allele Comments	Sample Comments

Sample 5: Run date and time: 09/11/2020 - 19:30:25 -> 09/11/2020 - 20:26:16

Dye: Blue - 2 peaks - 13.fsa

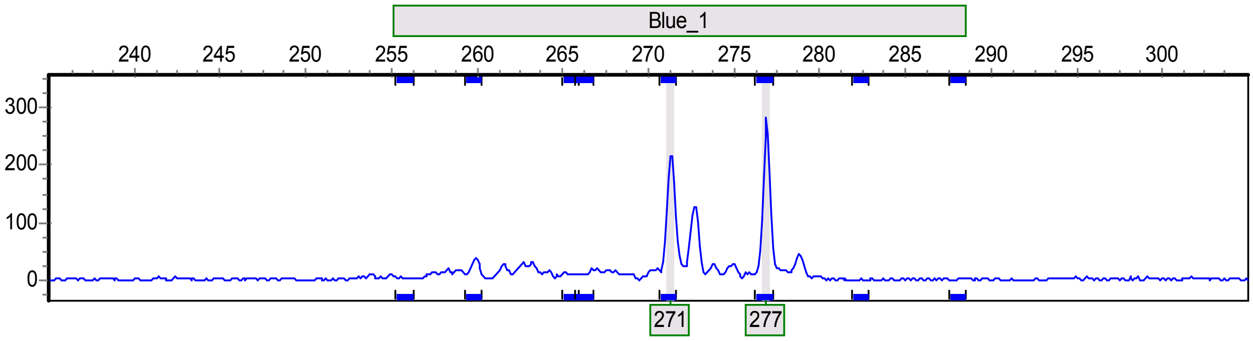

No	Size	Height	Area	Marker	Allele	Difference	Quality	Score	Allele Comments	Sample Comments
1	271.3	216	1157	Blue_1	271	0.1	Pass	16.5		
2	276.9	281	1415	Blue_1	277	0.1	Pass	28.0		

Sample 6: Run date and time: 09/11/2020 - 19:30:25 -> 09/11/2020 - 20:26:16

Dye: Blue - 2 peaks - 14.fsa

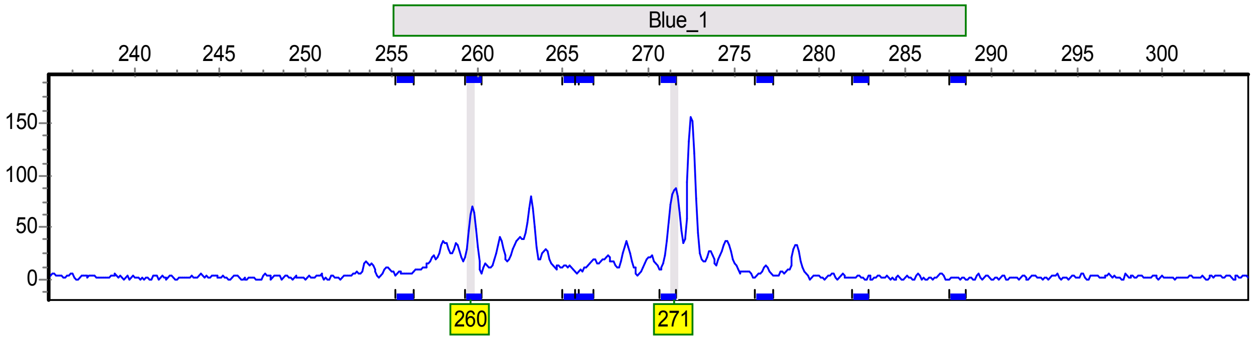

No	Size	Height	Area	Marker	Allele	Difference	Quality	Score	Allele Comments	Sample Comments
1	259.7	70	391	Blue_1	260	0.1	Check	2.0		
2	271.6	88	677	Blue_1	271	0.4	Check	1.3		

Allele Report

Sample 7: Run date and time: 09/11/2020 - 19:30:25 -> 09/11/2020 - 20:26:16

Dye: Blue - 1 peaks - 15.fsa

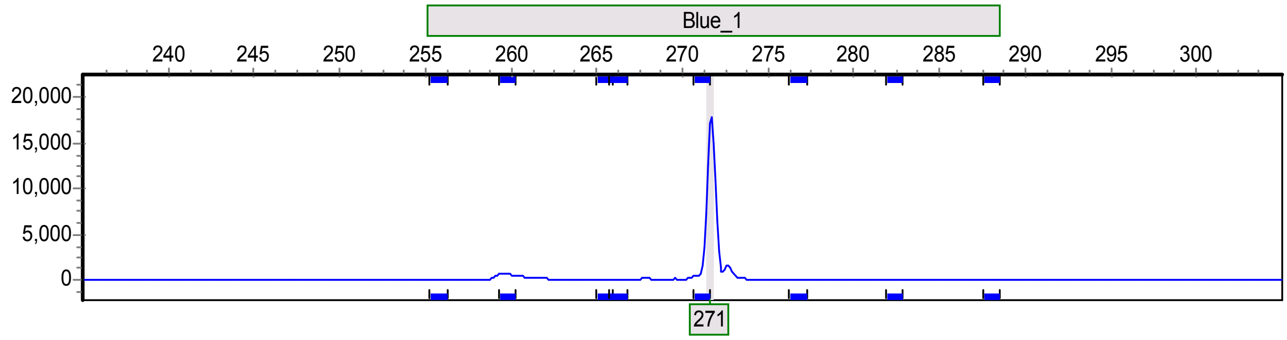

No	Size	Height	Area	Marker	Allele	Difference	Quality	Score	Allele Comments	Sample Comments
1	271.7	17717	90256	Blue_1	271	0.5	Pass	500.0		

Sample 8: Run date and time: 09/11/2020 - 19:30:25 -> 09/11/2020 - 20:26:16

Dye: Blue - 1 peaks - 16.fsa

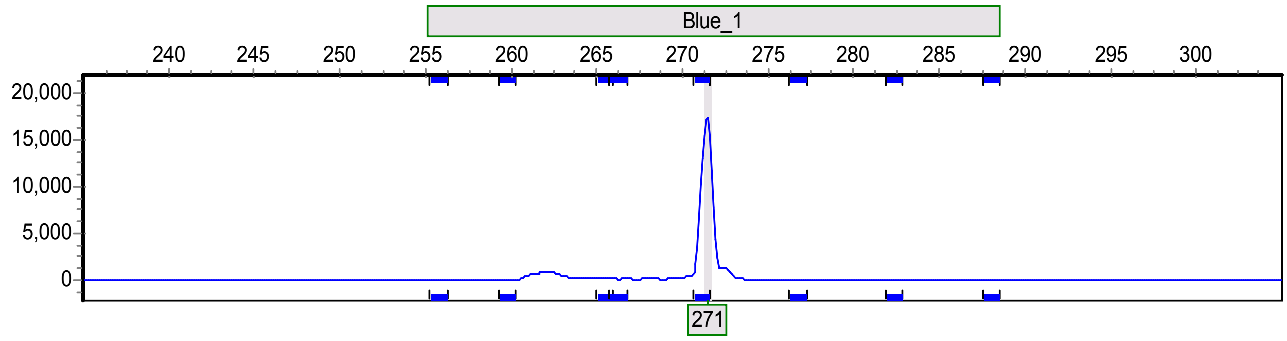

No	Size	Height	Area	Marker	Allele	Difference	Quality	Score	Allele Comments	Sample Comments
1	271.5	17269	120386	Blue_1	271	0.3	Pass	500.0		

Sample 9: Run date and time: 09/11/2020 - 19:30:25 -> 09/11/2020 - 20:26:16

Dye: Blue - 1 peaks - 17.fsa

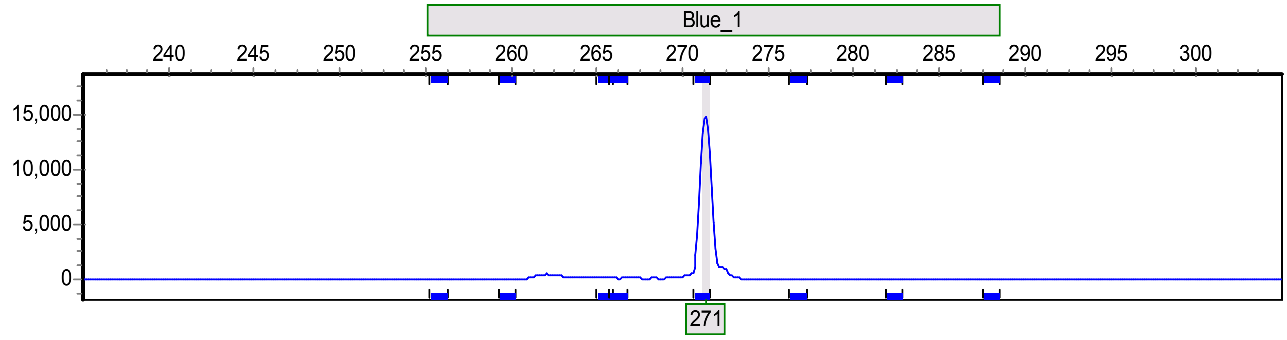

No	Size	Height	Area	Marker	Allele	Difference	Quality	Score	Allele Comments	Sample Comments
1	271.4	14677	103933	Blue_1	271	0.2	Pass	500.0		

Sample 10: Run date and time: 09/11/2020 - 19:30:25 -> 09/11/2020 - 20:26:16

Dye: Blue - 0 peaks - 18.fsa

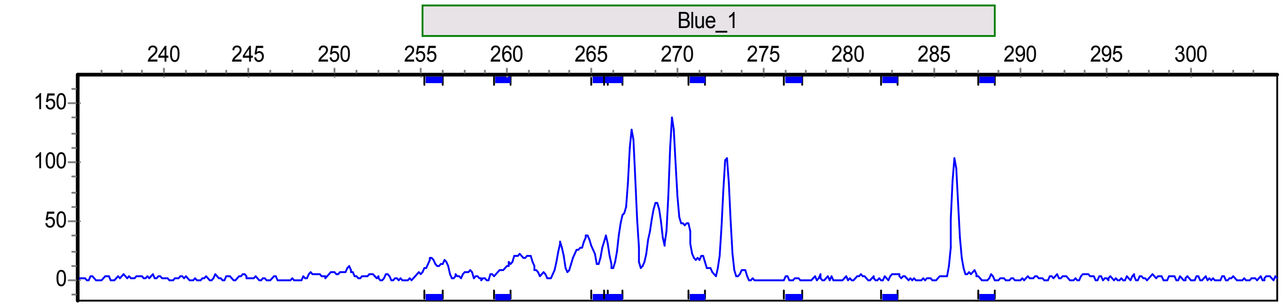

No	Size	Height	Area	Marker	Allele	Difference	Quality	Score	Allele Comments	Sample Comments

Sample 11: Run date and time: 09/11/2020 - 19:30:25 -> 09/11/2020 - 20:26:16

Dye: Blue - 1 peaks - 19.fsa

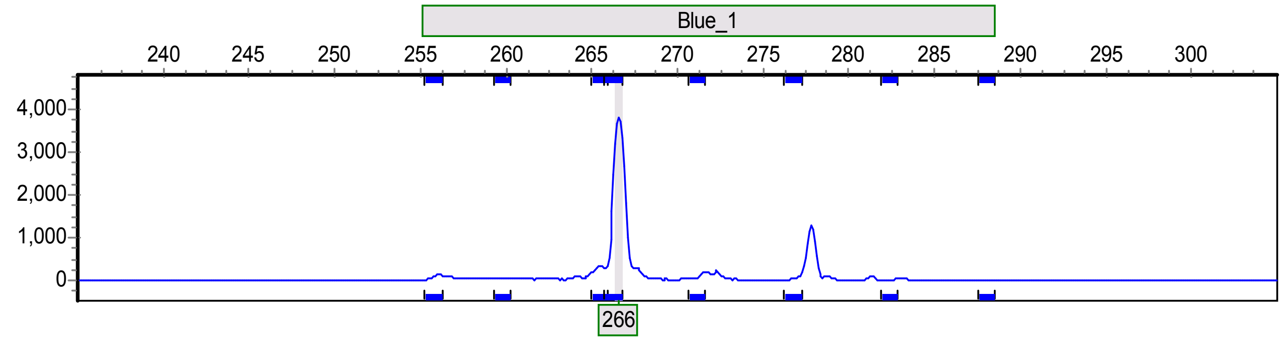

No	Size	Height	Area	Marker	Allele	Difference	Quality	Score	Allele Comments	Sample Comments
1	266.6	3771	27999	Blue_1	266	0.3	Pass	500.0		

Sample 12: Run date and time: 09/11/2020 - 19:30:25 -> 09/11/2020 - 20:26:16

Dye: Blue - 1 peaks - 2.fsa

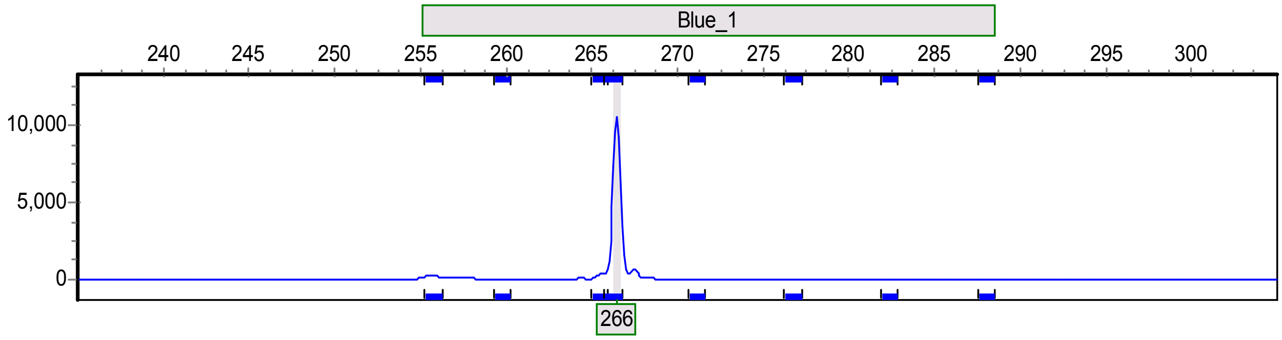

No	Size	Height	Area	Marker	Allele	Difference	Quality	Score	Allele Comments	Sample Comments
1	266.5	10398	53068	Blue_1	266	0.2	Pass	500.0		

Sample 13: Run date and time: 09/11/2020 - 19:30:25 -> 09/11/2020 - 20:26:16

Dye: Blue - 1 peaks - 20.fsa

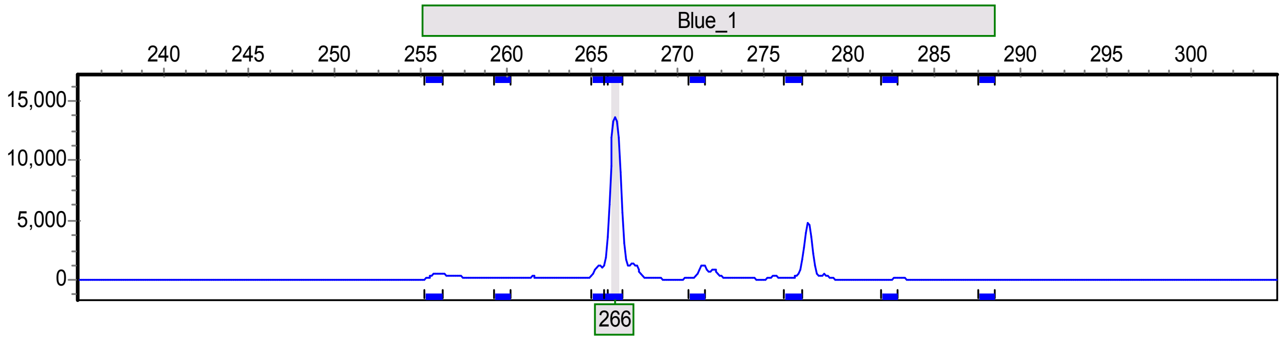

No	Size	Height	Area	Marker	Allele	Difference	Quality	Score	Allele Comments	Sample Comments
1	266.4	13539	100283	Blue_1	266	0.1	Pass	500.0		

Sample 14: Run date and time: 09/11/2020 - 19:30:25 -> 09/11/2020 - 20:26:16

Dye: Blue - 1 peaks - 21.fsa

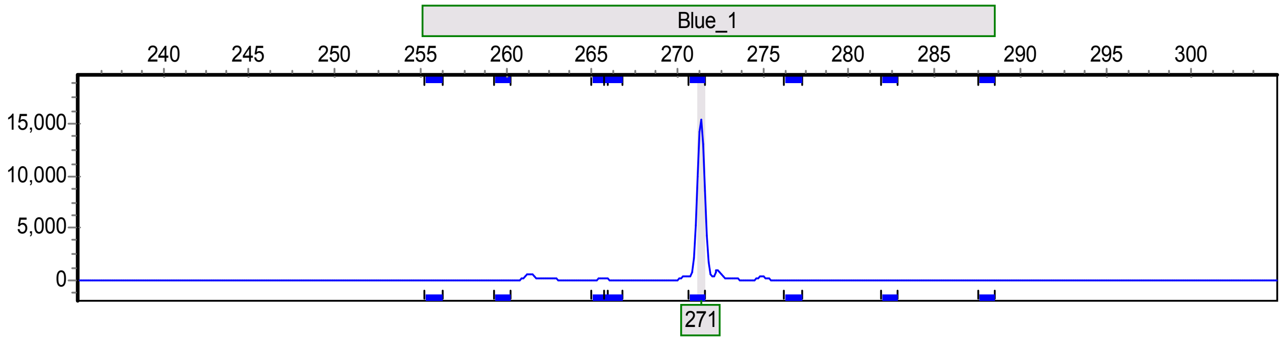

No	Size	Height	Area	Marker	Allele	Difference	Quality	Score	Allele Comments	Sample Comments
1	271.4	15496	70607	Blue_1	271	0.2	Pass	500.0		

Sample 15: Run date and time: 09/11/2020 - 19:30:25 -> 09/11/2020 - 20:26:16

Dye: Blue - 1 peaks - 22.fsa

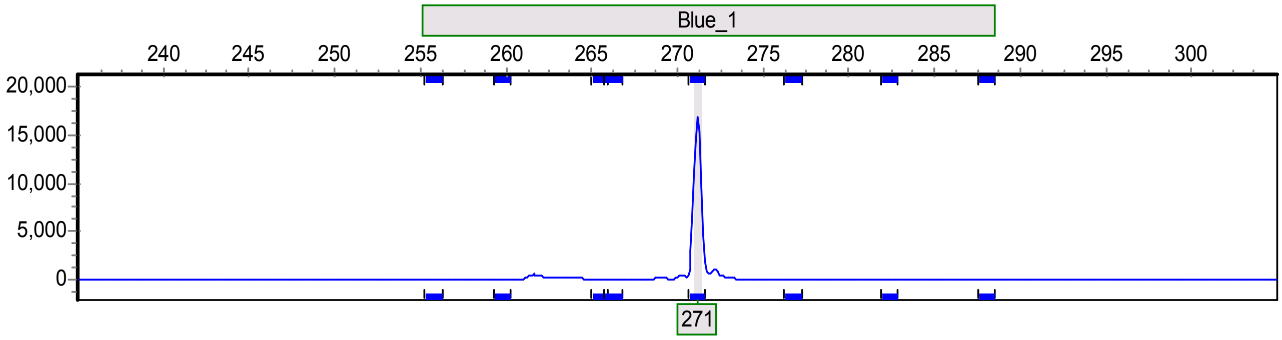

No	Size	Height	Area	Marker	Allele	Difference	Quality	Score	Allele Comments	Sample Comments
1	271.2	16823	79360	Blue_1	271	0.0	Pass	500.0		

Allele Report

Sample 16: Run date and time: 09/11/2020 - 19:30:25 -> 09/11/2020 - 20:26:16

Dye: Blue - 2 peaks - 23.fsa

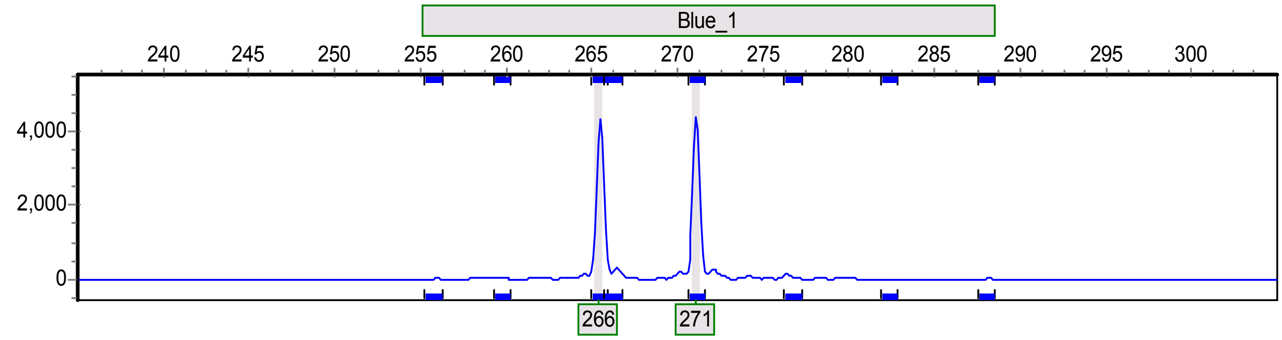

No	Size	Height	Area	Marker	Allele	Difference	Quality	Score	Allele Comments	Sample Comments
1	265.5	4316	19523	Blue_1	266	0.0	Pass	500.0		
2	271.1	4350	19995	Blue_1	271	0.1	Pass	500.0		

Sample 17: Run date and time: 09/11/2020 - 19:30:25 -> 09/11/2020 - 20:26:16

Dye: Blue - 1 peaks - 24.fsa

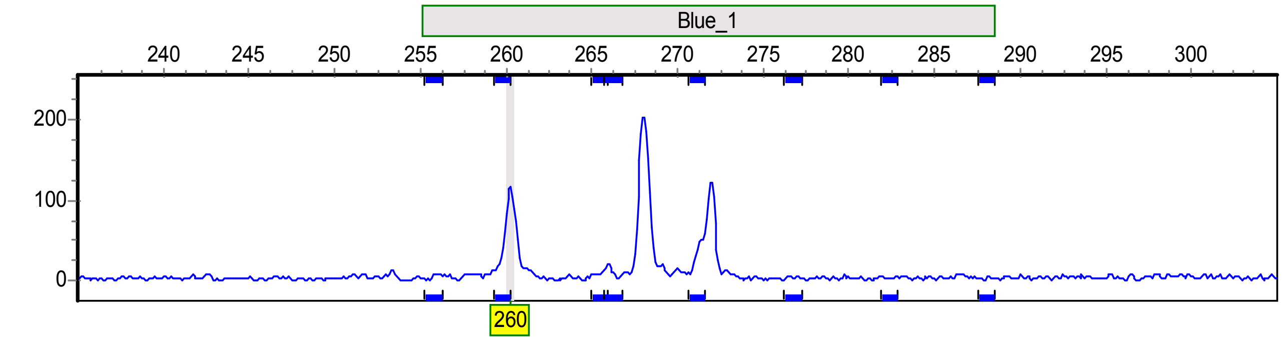

No	Size	Height	Area	Marker	Allele	Difference	Quality	Score	Allele Comments	Sample Comments
1	260.3	116	890	Blue_1	260	0.5	Check	2.4		

Sample 18: Run date and time: 09/11/2020 - 19:30:25 -> 09/11/2020 - 20:26:16

Dye: Blue - 0 peaks - 25.fsa

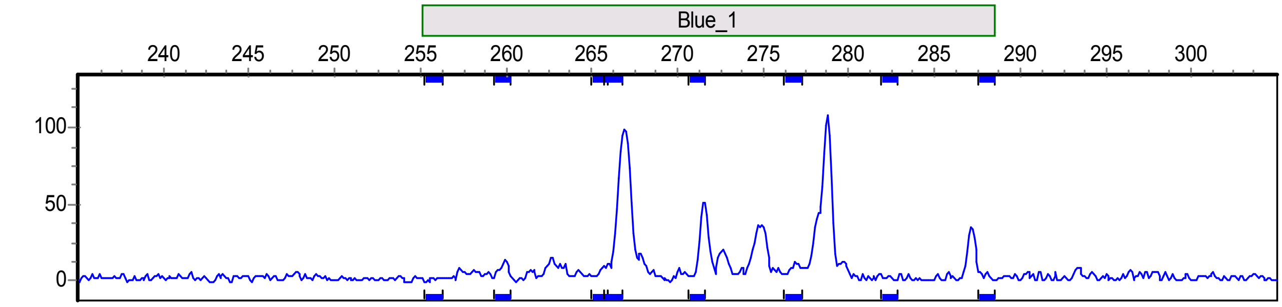

No	Size	Height	Area	Marker	Allele	Difference	Quality	Score	Allele Comments	Sample Comments

Sample 19: Run date and time: 09/11/2020 - 19:30:25 -> 09/11/2020 - 20:26:16

Dye: Blue - 1 peaks - 26.fsa

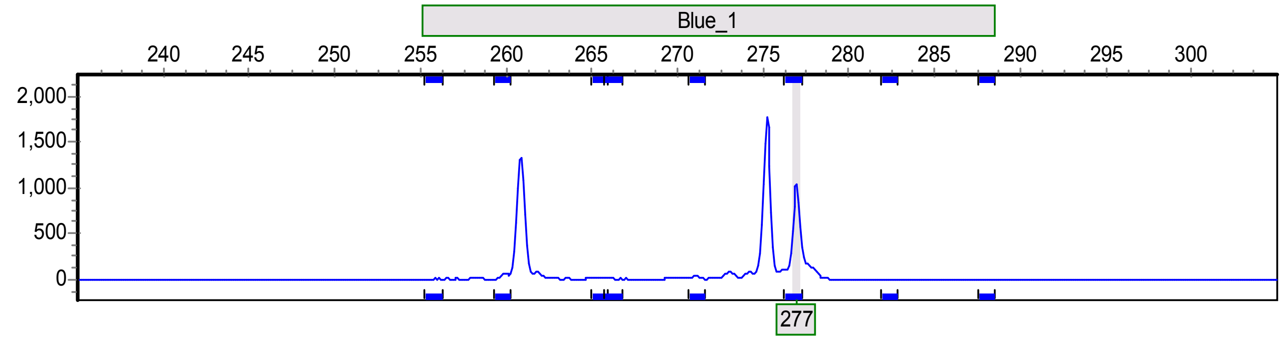

No	Size	Height	Area	Marker	Allele	Difference	Quality	Score	Allele Comments	Sample Comments
1	277.0	1030	5419	Blue_1	277	0.2	Pass	192.7		

Sample 20: Run date and time: 09/11/2020 - 19:30:25 -> 09/11/2020 - 20:26:16

Dye: Blue - 1 peaks - 27.fsa

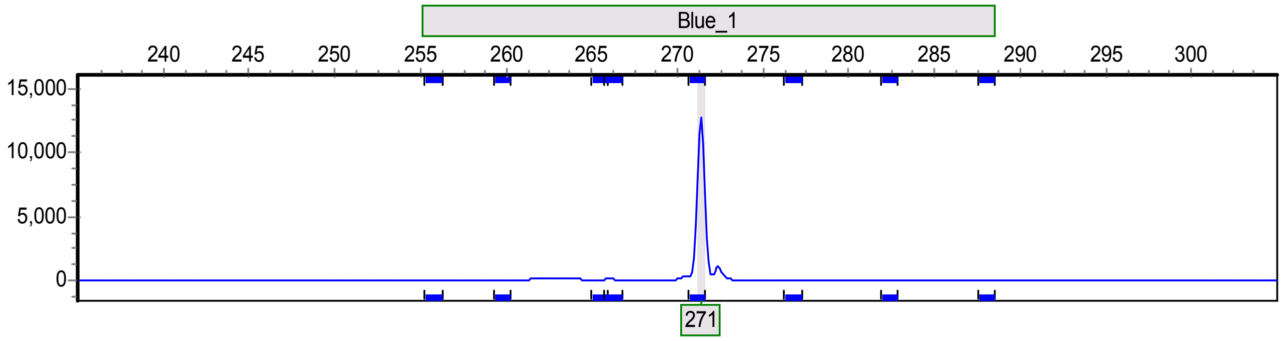

No	Size	Height	Area	Marker	Allele	Difference	Quality	Score	Allele Comments	Sample Comments
1	271.4	12656	57314	Blue_1	271	0.2	Pass	500.0		

Sample 21: Run date and time: 09/11/2020 - 19:30:25 -> 09/11/2020 - 20:26:16

Dye: Blue - 2 peaks - 28.fsa

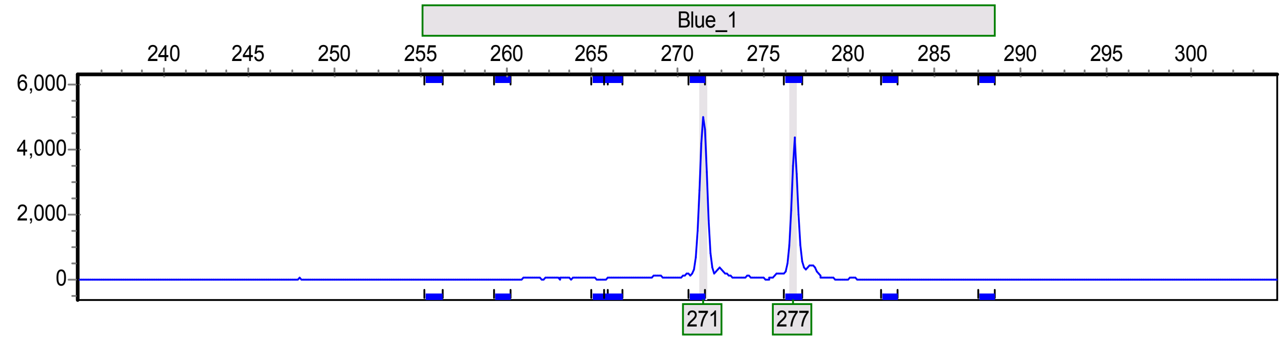

No	Size	Height	Area	Marker	Allele	Difference	Quality	Score	Allele Comments	Sample Comments
1	271.5	4959	23372	Blue_1	271	0.3	Pass	500.0		
2	276.8	4346	21151	Blue_1	277	0.0	Pass	500.0		

Sample 22: Run date and time: 09/11/2020 - 19:30:25 -> 09/11/2020 - 20:26:16

Dye: Blue - 1 peaks - 29.fsa

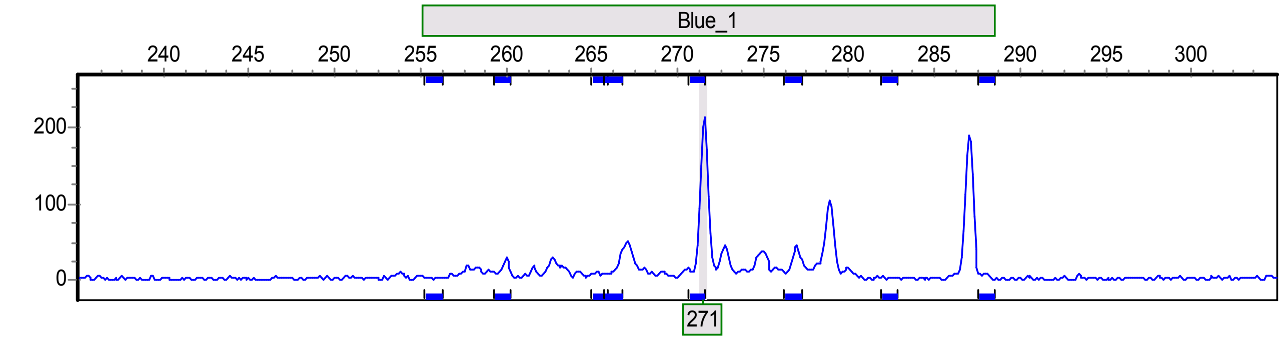

No	Size	Height	Area	Marker	Allele	Difference	Quality	Score	Allele Comments	Sample Comments
1	271.6	214	1026	Blue_1	271	0.4	Pass	19.2		

Sample 23: Run date and time: 09/11/2020 - 19:30:25 -> 09/11/2020 - 20:26:16

Dye: Blue - 1 peaks - 3.fsa

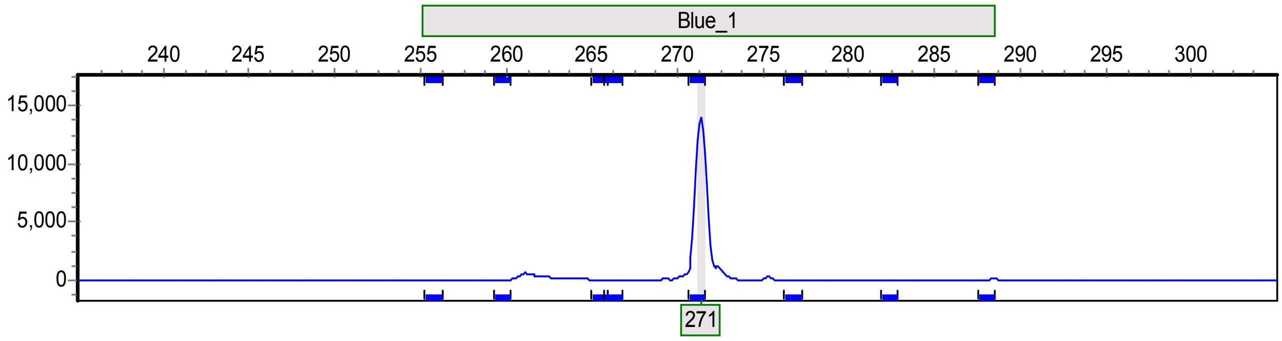

No	Size	Height	Area	Marker	Allele	Difference	Quality	Score	Allele Comments	Sample Comments
1	271.4	13890	98562	Blue_1	271	0.2	Pass	500.0		

Sample 24: Run date and time: 09/11/2020 - 19:30:25 -> 09/11/2020 - 20:26:16

Dye: Blue - 2 peaks - 30.fsa

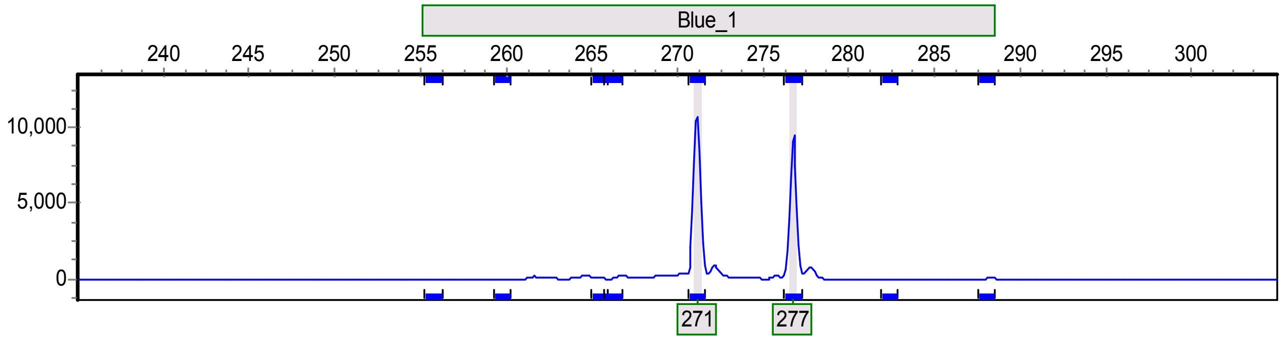

No	Size	Height	Area	Marker	Allele	Difference	Quality	Score	Allele Comments	Sample Comments
1	271.2	10537	49065	Blue_1	271	0.0	Pass	500.0		
2	276.8	9348	43668	Blue_1	277	0.0	Pass	500.0		

Allele Report

Sample 25: Run date and time: 09/11/2020 - 19:30:25 -> 09/11/2020 - 20:26:16

Dye: Blue - 0 peaks - 31.fsa

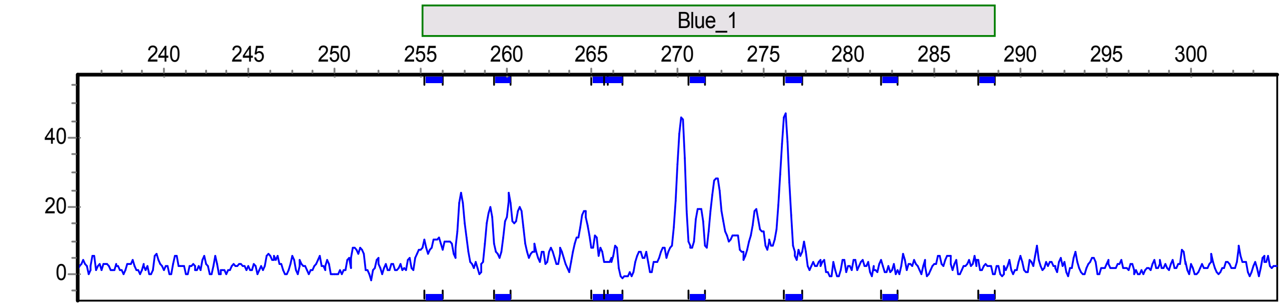

No	Size	Height	Area	Marker	Allele	Difference	Quality	Score	Allele Comments	Sample Comments

Sample 26: Run date and time: 09/11/2020 - 19:30:25 -> 09/11/2020 - 20:26:16

Dye: Blue - 0 peaks - 32.fsa

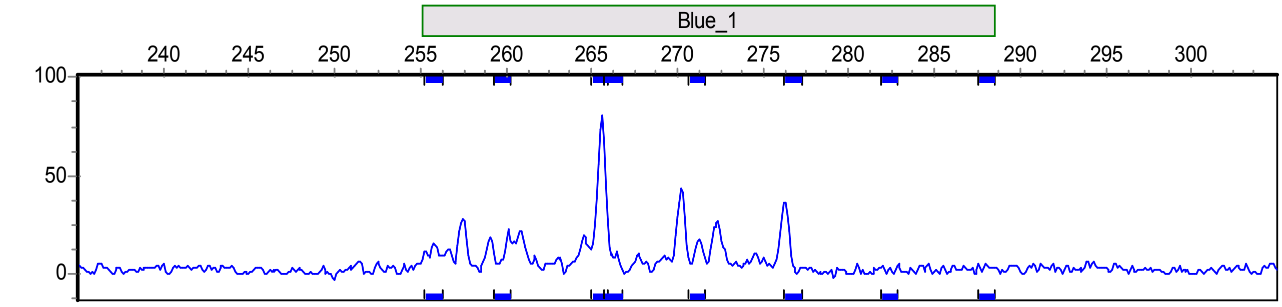

No	Size	Height	Area	Marker	Allele	Difference	Quality	Score	Allele Comments	Sample Comments

Sample 27: Run date and time: 09/11/2020 - 19:30:25 -> 09/11/2020 - 20:26:16

Dye: Blue - 2 peaks - 33.fsa

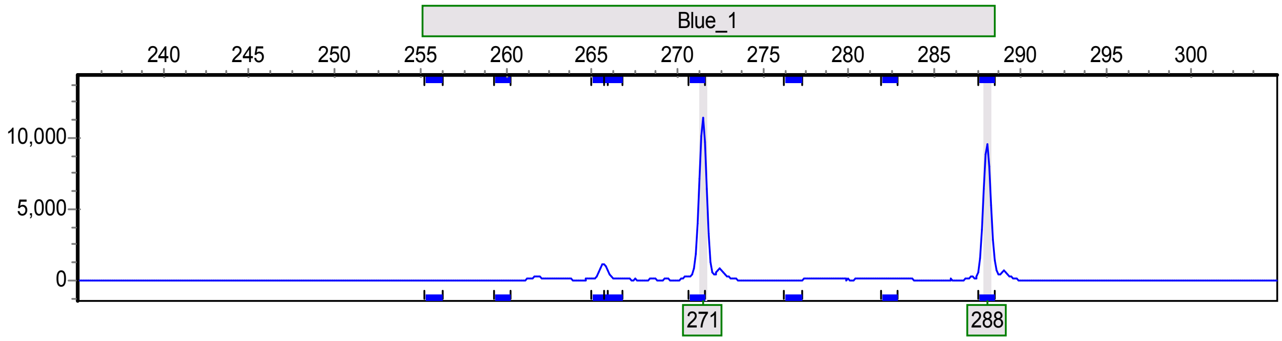

No	Size	Height	Area	Marker	Allele	Difference	Quality	Score	Allele Comments	Sample Comments
1	271.5	11280	51949	Blue_1	271	0.3	Pass	500.0		
2	288.1	9491	44940	Blue_1	288	0.0	Pass	500.0		

Sample 28: Run date and time: 09/11/2020 - 19:30:25 -> 09/11/2020 - 20:26:16

Dye: Blue - 2 peaks - 34.fsa

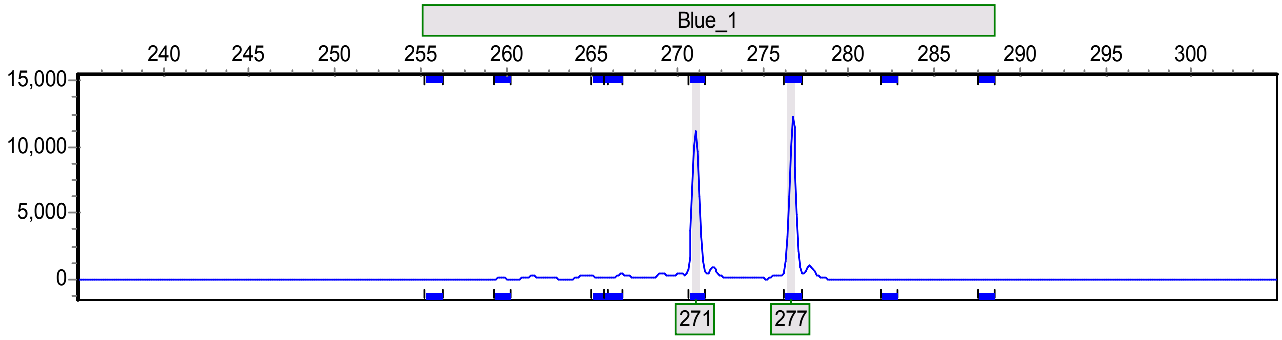

No	Size	Height	Area	Marker	Allele	Difference	Quality	Score	Allele Comments	Sample Comments
1	271.1	11208	51077	Blue_1	271	0.1	Pass	500.0		
2	276.7	12206	57226	Blue_1	277	0.1	Pass	500.0		

Sample 29: Run date and time: 09/11/2020 - 19:30:25 -> 09/11/2020 - 20:26:16

Dye: Blue - 2 peaks - 35.fsa

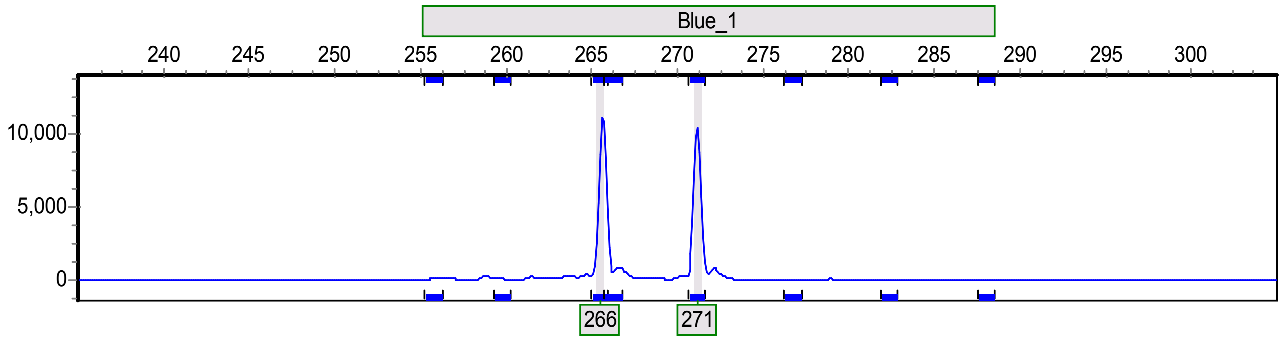

No	Size	Height	Area	Marker	Allele	Difference	Quality	Score	Allele Comments	Sample Comments
1	265.6	11110	52260	Blue_1	266	0.1	Pass	500.0		
2	271.2	10530	49561	Blue_1	271	0.0	Pass	500.0		

Sample 30: Run date and time: 09/11/2020 - 19:30:25 -> 09/11/2020 - 20:26:16

Dye: Blue - 2 peaks - 36.fsa

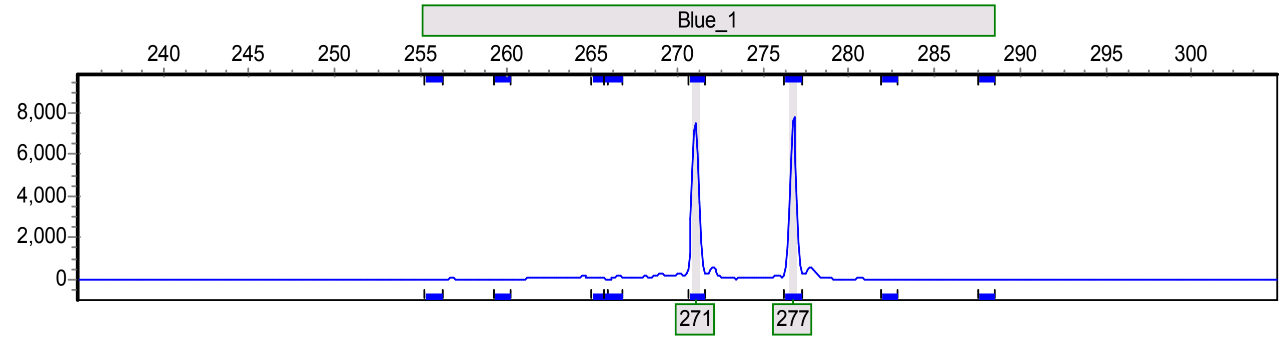

No	Size	Height	Area	Marker	Allele	Difference	Quality	Score	Allele Comments	Sample Comments
1	271.1	7452	34091	Blue_1	271	0.1	Pass	500.0		
2	276.8	7710	36367	Blue_1	277	0.0	Pass	500.0		

Sample 31: Run date and time: 09/11/2020 - 19:30:25 -> 09/11/2020 - 20:26:16

Dye: Blue - 1 peaks - 37.fsa

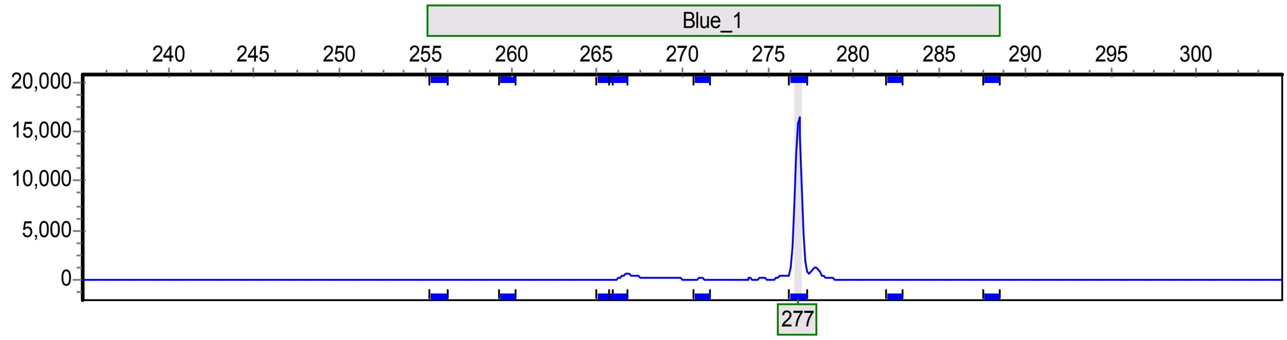

No	Size	Height	Area	Marker	Allele	Difference	Quality	Score	Allele Comments	Sample Comments
1	276.8	16359	83382	Blue_1	277	0.0	Pass	500.0		

Sample 32: Run date and time: 09/11/2020 - 19:30:25 -> 09/11/2020 - 20:26:16

Dye: Blue - 1 peaks - 38.fsa

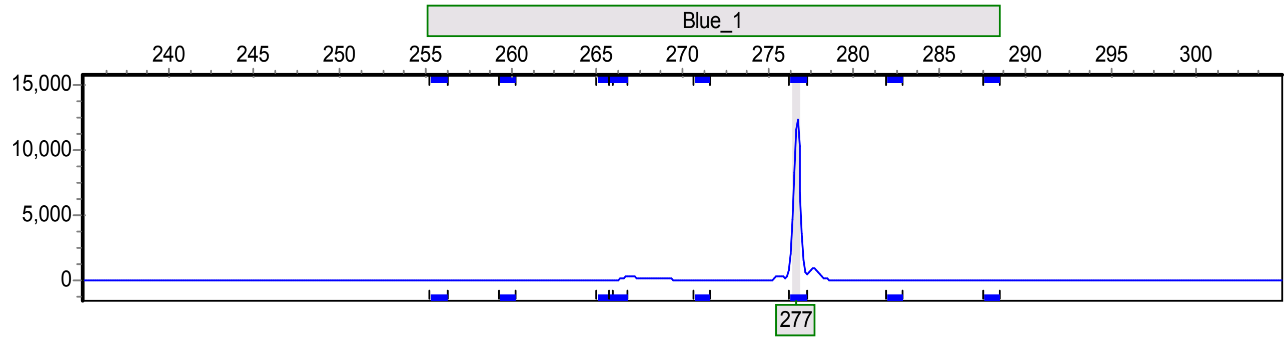

No	Size	Height	Area	Marker	Allele	Difference	Quality	Score	Allele Comments	Sample Comments
1	276.7	12399	57912	Blue_1	277	0.1	Pass	500.0		

Sample 33: Run date and time: 09/11/2020 - 19:30:25 -> 09/11/2020 - 20:26:16

Dye: Blue - 2 peaks - 39.fsa

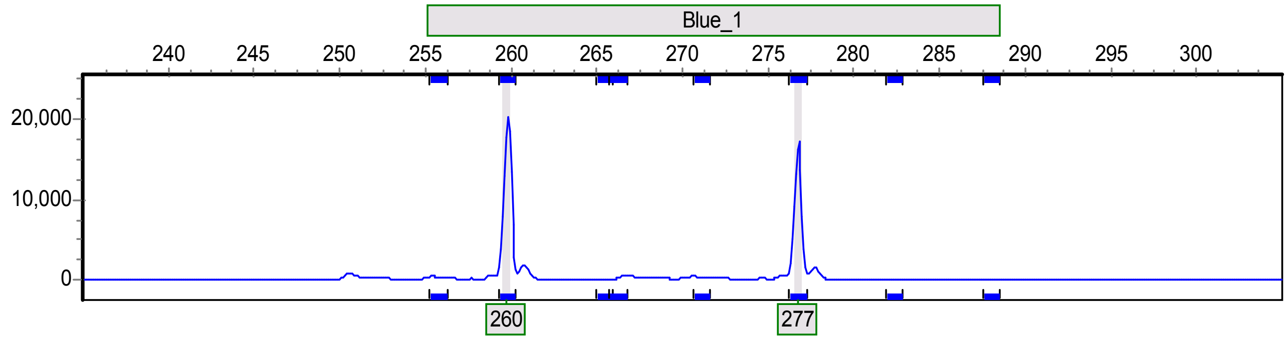

No	Size	Height	Area	Marker	Allele	Difference	Quality	Score	Allele Comments	Sample Comments
1	259.8	20240	100268	Blue_1	260	0.0	Pass	500.0		
2	276.8	17218	84883	Blue_1	277	0.0	Pass	500.0		

Sample 34: Run date and time: 09/11/2020 - 19:30:25 -> 09/11/2020 - 20:26:16

Dye: Blue - 1 peaks - 4.fsa

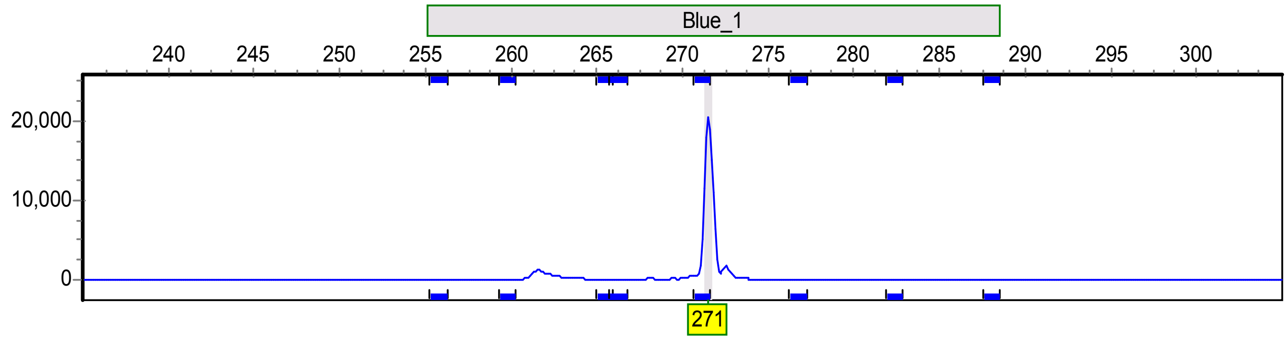

No	Size	Height	Area	Marker	Allele	Difference	Quality	Score	Allele Comments	Sample Comments
1	271.5	20391	105073	Blue_1	271	0.3	Check	500.0	[<SAT (Repaired)>]	

Sample 35: Run date and time: 09/11/2020 - 19:30:25 -> 09/11/2020 - 20:26:16

Dye: Blue - 2 peaks - 40.fsa

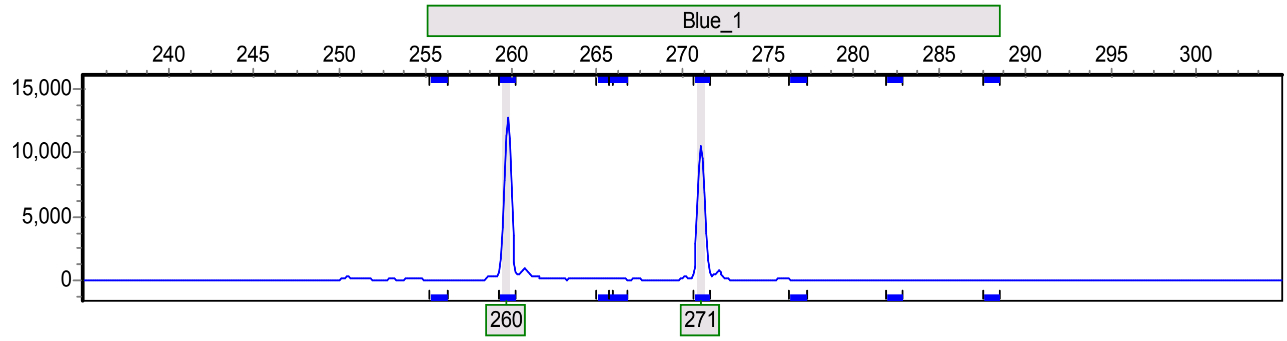

No	Size	Height	Area	Marker	Allele	Difference	Quality	Score	Allele Comments	Sample Comments
1	259.8	12660	57015	Blue_1	260	0.0	Pass	500.0		
2	271.1	10455	48070	Blue_1	271	0.1	Pass	500.0		

Sample 36: Run date and time: 09/11/2020 - 19:30:25 -> 09/11/2020 - 20:26:16

Dye: Blue - 2 peaks - 41.fsa

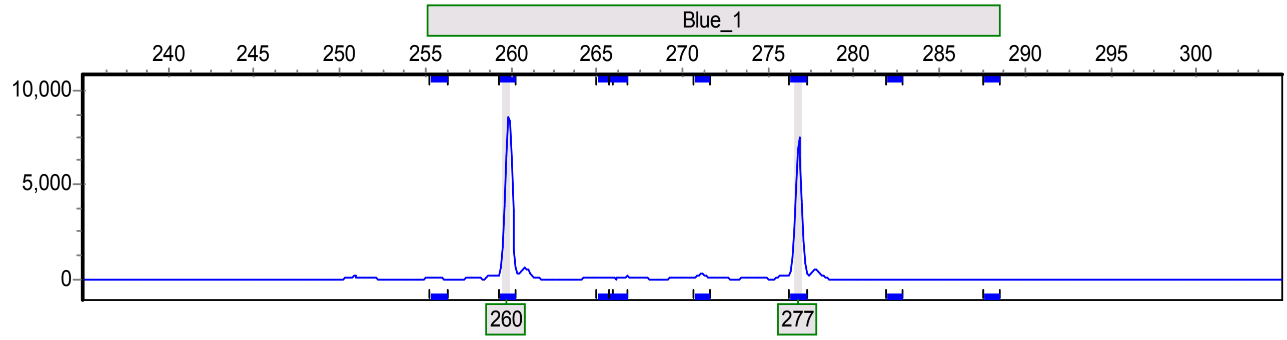

No	Size	Height	Area	Marker	Allele	Difference	Quality	Score	Allele Comments	Sample Comments
1	259.8	8522	38980	Blue_1	260	0.0	Pass	500.0		
2	276.8	7556	34790	Blue_1	277	0.0	Pass	500.0		

Allele Report

Sample 37: Run date and time: 09/11/2020 - 19:30:25 -> 09/11/2020 - 20:26:16

Dye: Blue - 2 peaks - 42.fsa

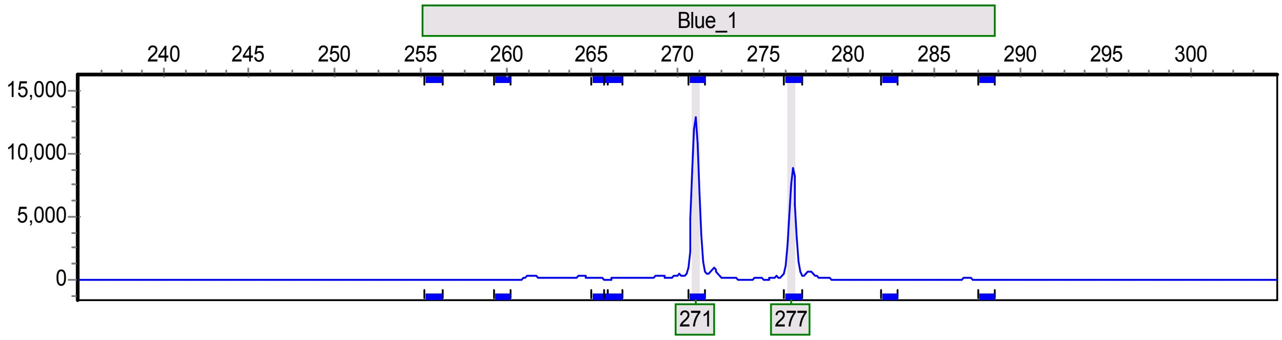

No	Size	Height	Area	Marker	Allele	Difference	Quality	Score	Allele Comments	Sample Comments
1	271.1	12782	59565	Blue_1	271	0.1	Pass	500.0		
2	276.7	8831	42335	Blue_1	277	0.1	Pass	500.0		

Sample 38: Run date and time: 09/11/2020 - 19:30:25 -> 09/11/2020 - 20:26:16

Dye: Blue - 2 peaks - 43.fsa

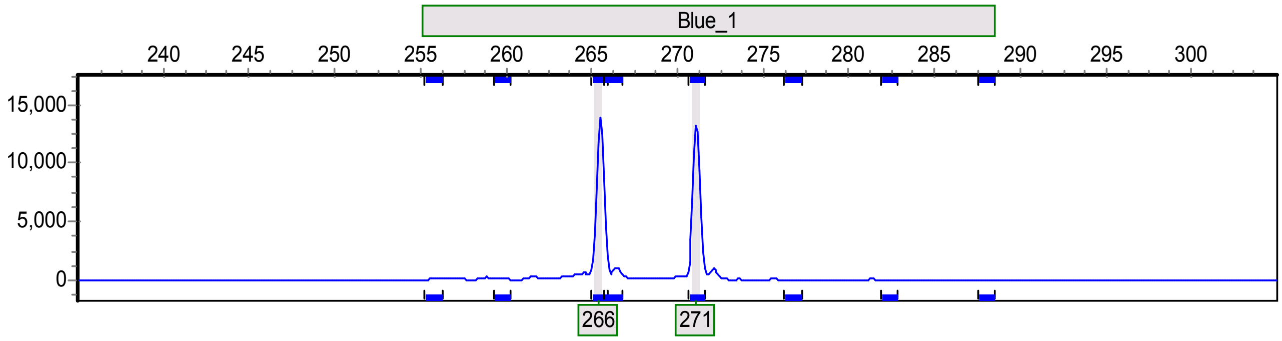

No	Size	Height	Area	Marker	Allele	Difference	Quality	Score	Allele Comments	Sample Comments
1	265.5	13817	63639	Blue_1	266	0.0	Pass	500.0		
2	271.1	13195	62550	Blue_1	271	0.1	Pass	500.0		

Sample 39: Run date and time: 09/11/2020 - 19:30:25 -> 09/11/2020 - 20:26:16

Dye: Blue - 2 peaks - 44.fsa

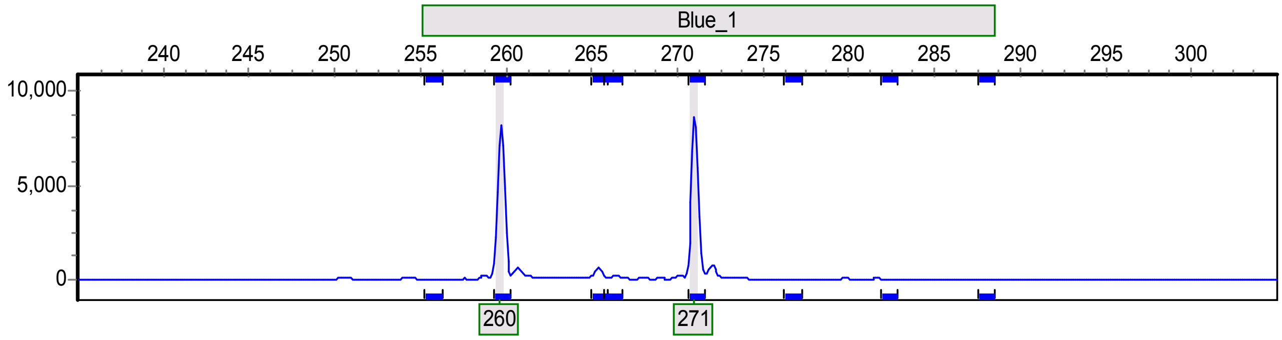

No	Size	Height	Area	Marker	Allele	Difference	Quality	Score	Allele Comments	Sample Comments
1	259.7	8158	36402	Blue_1	260	0.1	Pass	500.0		
2	271.0	8575	38808	Blue_1	271	0.2	Pass	500.0		

Sample 40: Run date and time: 09/11/2020 - 19:30:25 -> 09/11/2020 - 20:26:16

Dye: Blue - 2 peaks - 45.fsa

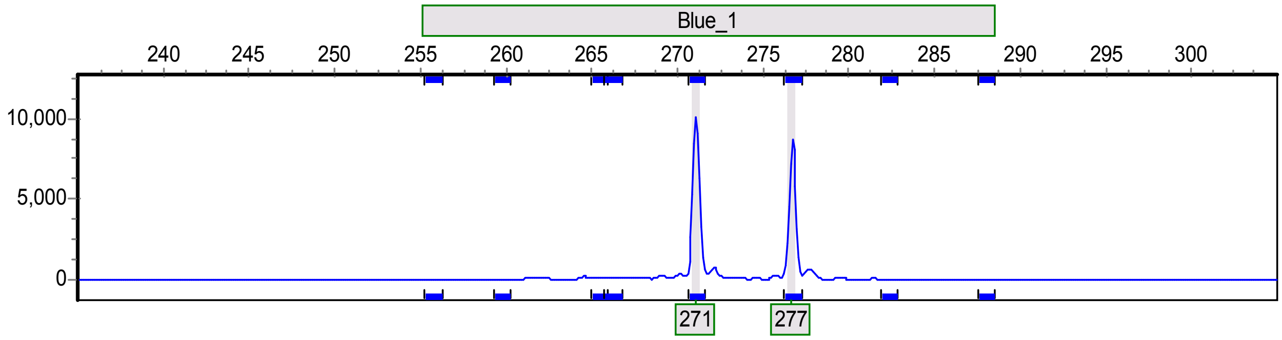

No	Size	Height	Area	Marker	Allele	Difference	Quality	Score	Allele Comments	Sample Comments
1	271.1	10019	44778	Blue_1	271	0.1	Pass	500.0		
2	276.7	8662	39780	Blue_1	277	0.1	Pass	500.0		

Sample 41: Run date and time: 09/11/2020 - 19:30:25 -> 09/11/2020 - 20:26:16

Dye: Blue - 2 peaks - 46.fsa

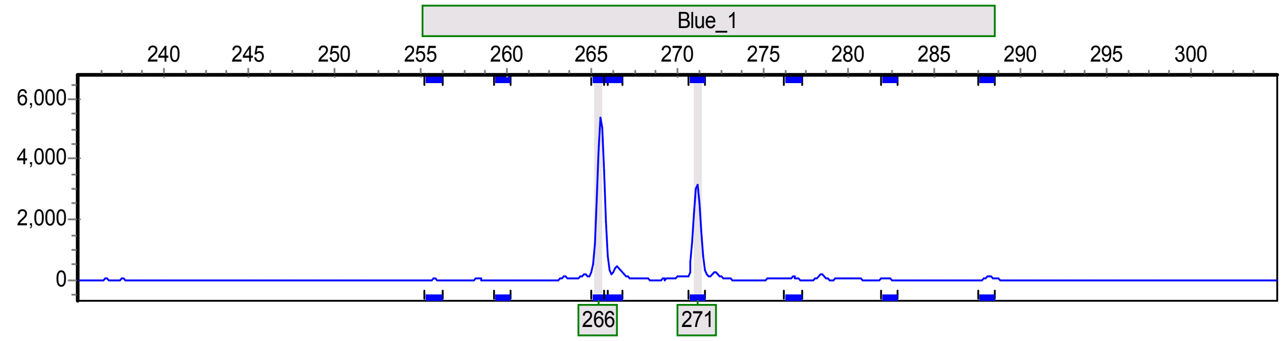

No	Size	Height	Area	Marker	Allele	Difference	Quality	Score	Allele Comments	Sample Comments
1	265.5	5341	24031	Blue_1	266	0.0	Pass	500.0		
2	271.2	3186	14685	Blue_1	271	0.0	Pass	500.0		

Sample 42: Run date and time: 09/11/2020 - 19:30:25 -> 09/11/2020 - 20:26:16

Dye: Blue - 1 peaks - 47.fsa

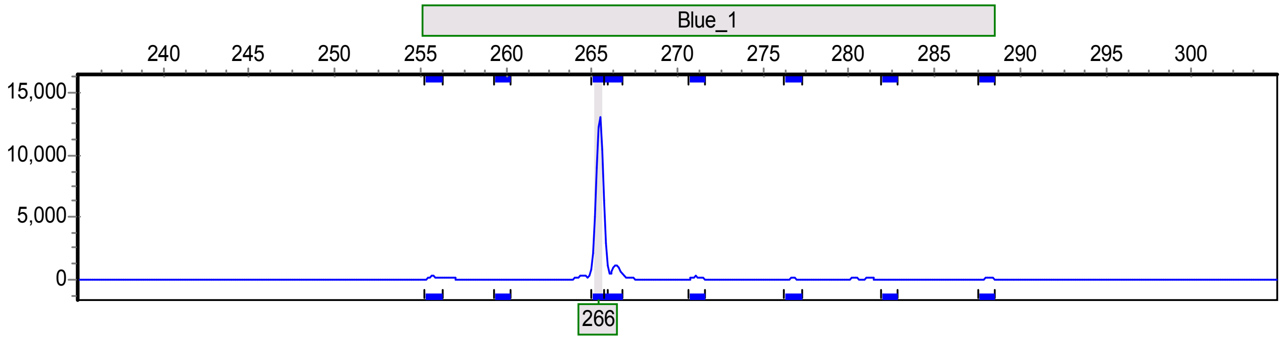

No	Size	Height	Area	Marker	Allele	Difference	Quality	Score	Allele Comments	Sample Comments
1	265.5	13016	58875	Blue_1	266	0.0	Pass	500.0		

Sample 43: Run date and time: 09/11/2020 - 19:30:25 -> 09/11/2020 - 20:26:16

Dye: Blue - 2 peaks - 48.fsa

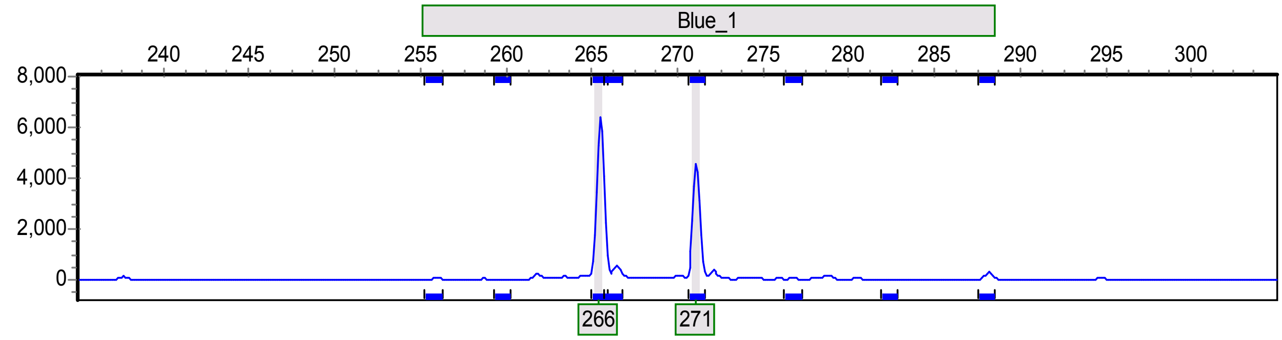

No	Size	Height	Area	Marker	Allele	Difference	Quality	Score	Allele Comments	Sample Comments
1	265.5	6361	28971	Blue_1	266	0.0	Pass	500.0		
2	271.1	4532	20873	Blue_1	271	0.1	Pass	500.0		

Sample 44: Run date and time: 09/11/2020 - 19:30:25 -> 09/11/2020 - 20:26:16

Dye: Blue - 1 peaks - 49.fsa

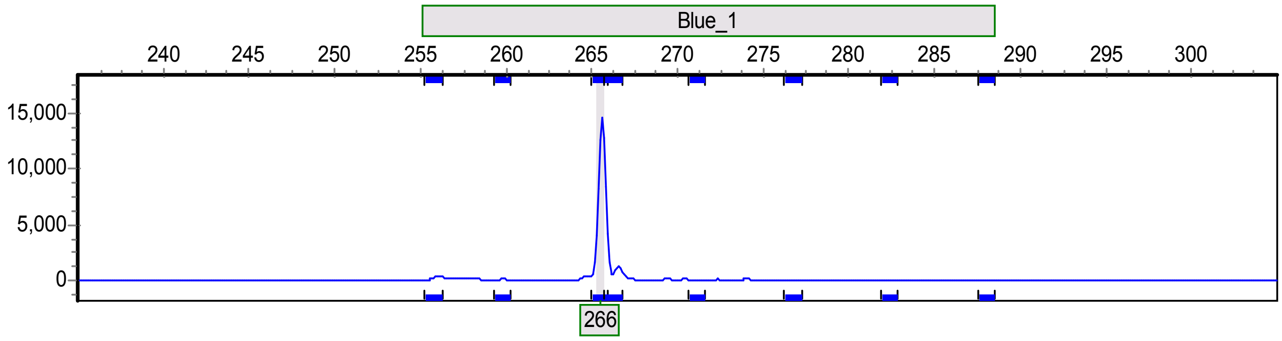

No	Size	Height	Area	Marker	Allele	Difference	Quality	Score	Allele Comments	Sample Comments
1	265.6	14471	64029	Blue_1	266	0.1	Pass	500.0		

Sample 45: Run date and time: 09/11/2020 - 19:30:25 -> 09/11/2020 - 20:26:16

Dye: Blue - 1 peaks - 5.fsa

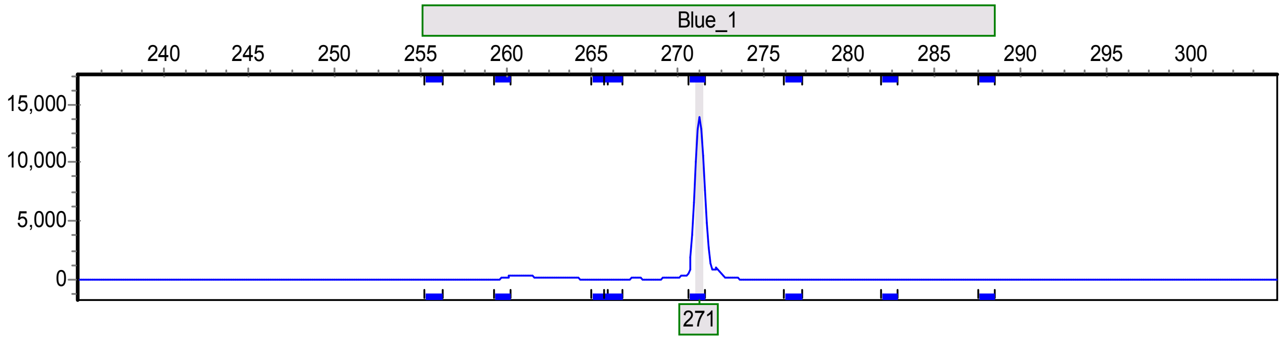

No	Size	Height	Area	Marker	Allele	Difference	Quality	Score	Allele Comments	Sample Comments
1	271.3	13822	84442	Blue_1	271	0.1	Pass	500.0		

Allele Report

Sample 46: Run date and time: 09/11/2020 - 19:30:25 -> 09/11/2020 - 20:26:16

Dye: Blue - 1 peaks - 50.fsa

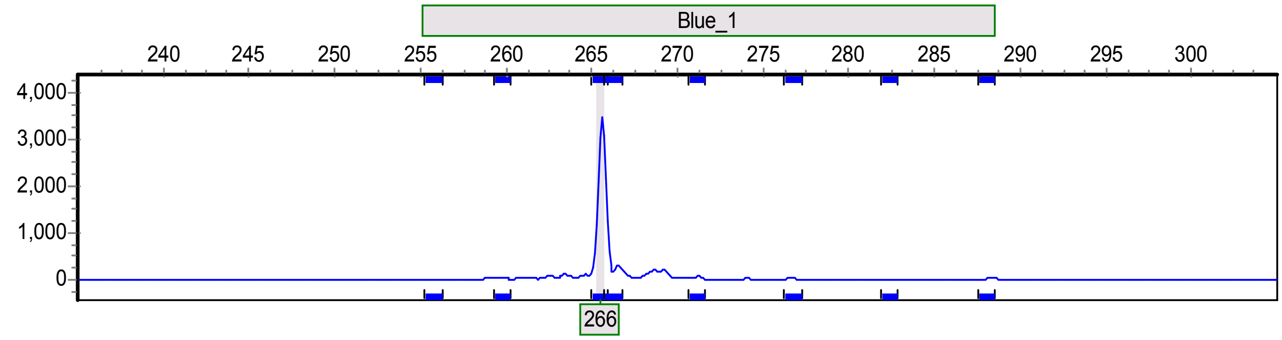

No	Size	Height	Area	Marker	Allele	Difference	Quality	Score	Allele Comments	Sample Comments
1	265.6	3459	16579	Blue_1	266	0.1	Pass	500.0		

Sample 47: Run date and time: 09/11/2020 - 19:30:25 -> 09/11/2020 - 20:26:16

Dye: Blue - 2 peaks - 51.fsa

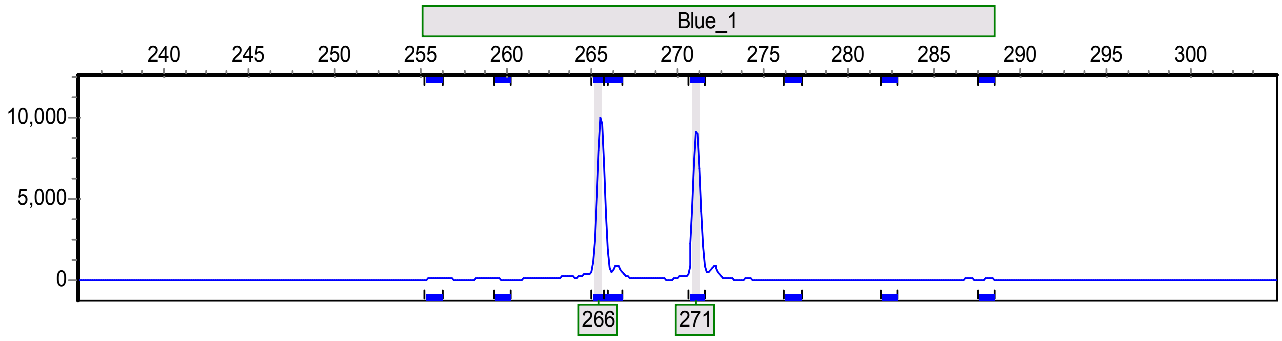

No	Size	Height	Area	Marker	Allele	Difference	Quality	Score	Allele Comments	Sample Comments
1	265.5	9948	47030	Blue_1	266	0.0	Pass	500.0		
2	271.1	9109	44086	Blue_1	271	0.1	Pass	500.0		

Sample 48: Run date and time: 09/11/2020 - 19:30:25 -> 09/11/2020 - 20:26:16

Dye: Blue - 2 peaks - 52.fsa

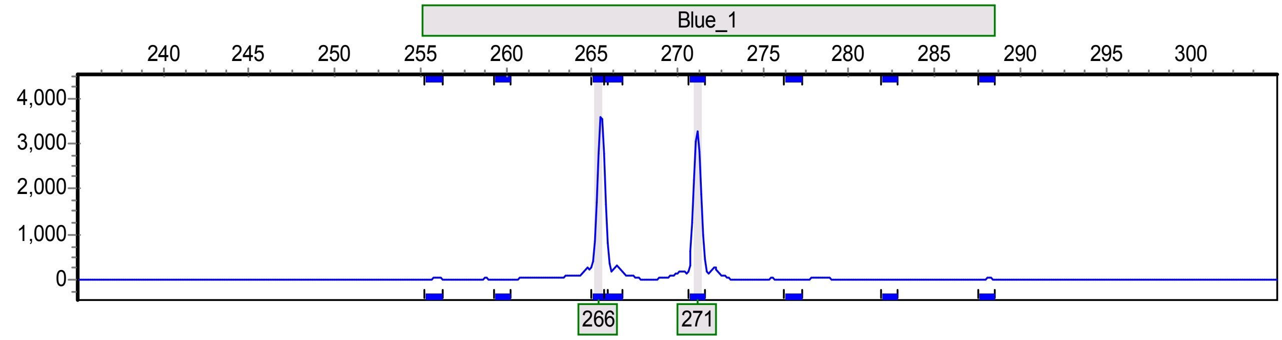

No	Size	Height	Area	Marker	Allele	Difference	Quality	Score	Allele Comments	Sample Comments
1	265.5	3562	17309	Blue_1	266	0.0	Pass	500.0		
2	271.2	3284	15784	Blue_1	271	0.0	Pass	500.0		

Allele Report

Sample 49: Run date and time: 09/11/2020 - 19:30:25 -> 09/11/2020 - 20:26:16

Dye: Blue - 0 peaks - 53.fsa

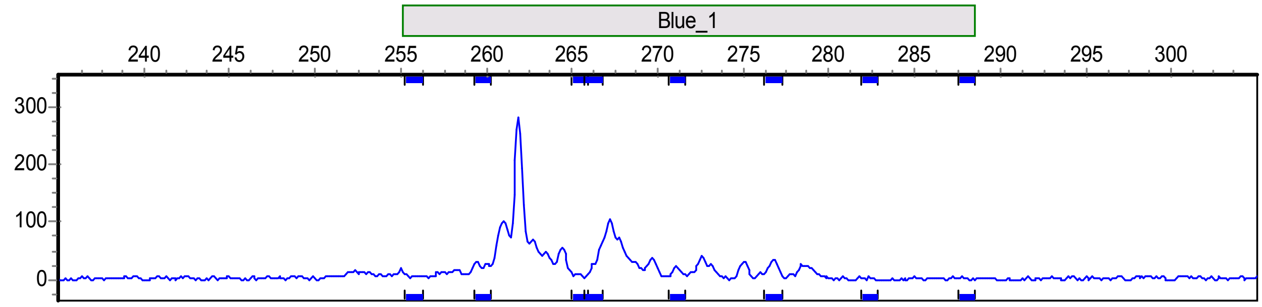

No	Size	Height	Area	Marker	Allele	Difference	Quality	Score	Allele Comments	Sample Comments

Sample 50: Run date and time: 09/11/2020 - 19:30:25 -> 09/11/2020 - 20:26:16

Dye: Blue - 1 peaks - 54.fsa

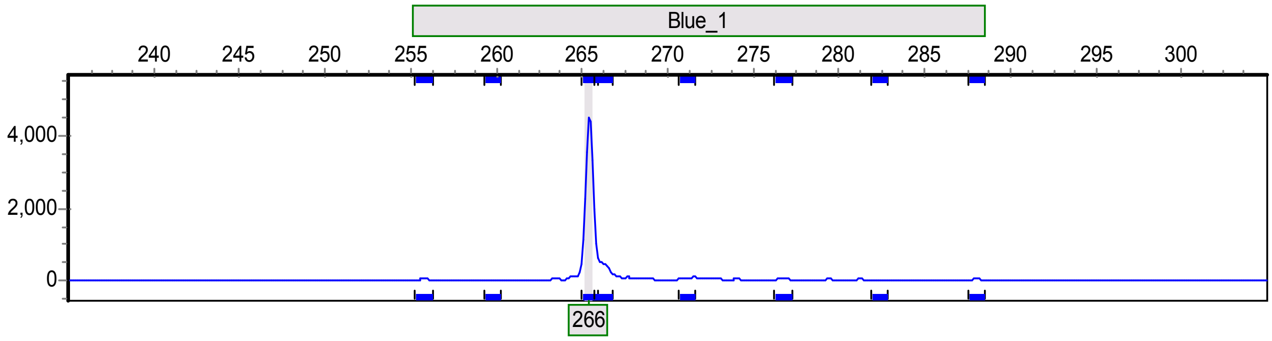

No	Size	Height	Area	Marker	Allele	Difference	Quality	Score	Allele Comments	Sample Comments
1	265.4	4499	21733	Blue_1	266	0.1	Pass	500.0		

Sample 51: Run date and time: 09/11/2020 - 19:30:25 -> 09/11/2020 - 20:26:16

Dye: Blue - 1 peaks - 55.fsa

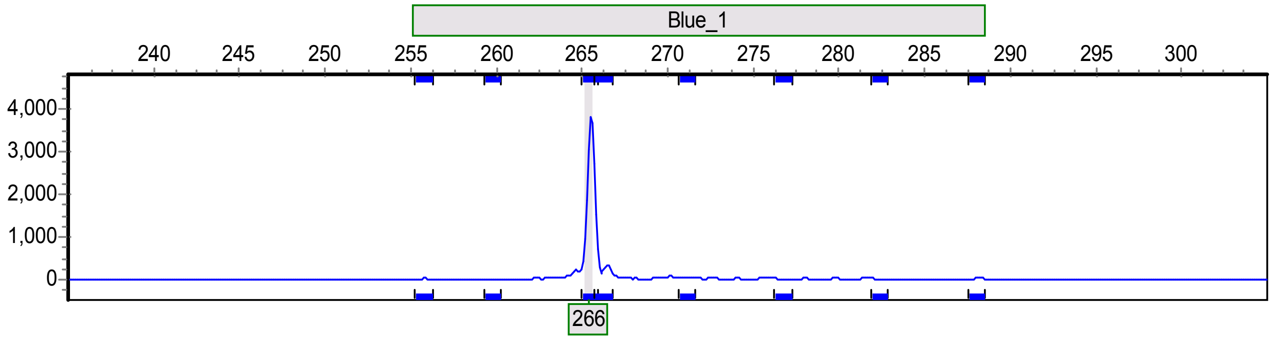

No	Size	Height	Area	Marker	Allele	Difference	Quality	Score	Allele Comments	Sample Comments
1	265.5	3808	17722	Blue_1	266	0.0	Pass	500.0		

Sample 52: Run date and time: 09/11/2020 - 19:30:25 -> 09/11/2020 - 20:26:16

Dye: Blue - 1 peaks - 56.fsa

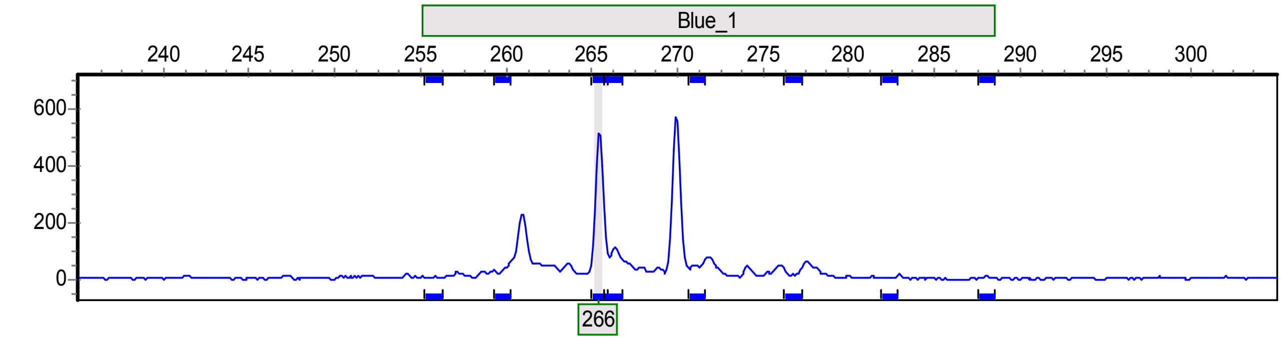

No	Size	Height	Area	Marker	Allele	Difference	Quality	Score	Allele Comments	Sample Comments
1	265.4	519	2605	Blue_1	266	0.1	Pass	78.7		

Sample 53: Run date and time: 09/11/2020 - 19:30:25 -> 09/11/2020 - 20:26:16

Dye: Blue - 1 peaks - 57.fsa

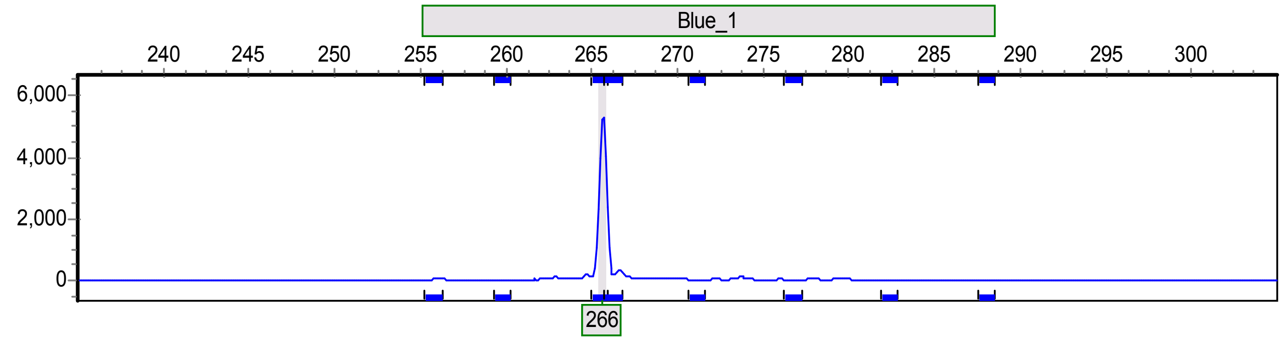

No	Size	Height	Area	Marker	Allele	Difference	Quality	Score	Allele Comments	Sample Comments
1	265.7	5264	24424	Blue_1	266	0.2	Pass	500.0		

Sample 54: Run date and time: 09/11/2020 - 19:30:25 -> 09/11/2020 - 20:26:16

Dye: Blue - 1 peaks - 58.fsa

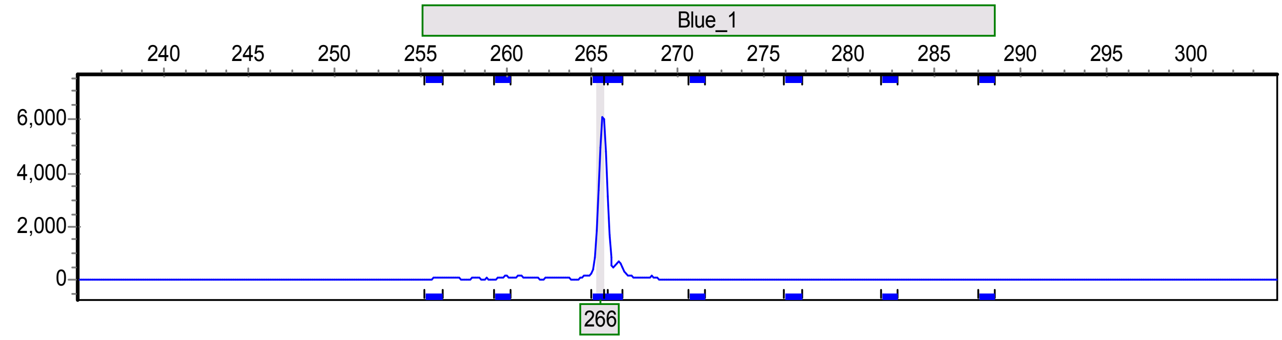

No	Size	Height	Area	Marker	Allele	Difference	Quality	Score	Allele Comments	Sample Comments
1	265.6	6067	31908	Blue_1	266	0.1	Pass	500.0		

Allele Report

Sample 55: Run date and time: 09/11/2020 - 19:30:25 -> 09/11/2020 - 20:26:16

Dye: Blue - 1 peaks - 59.fsa

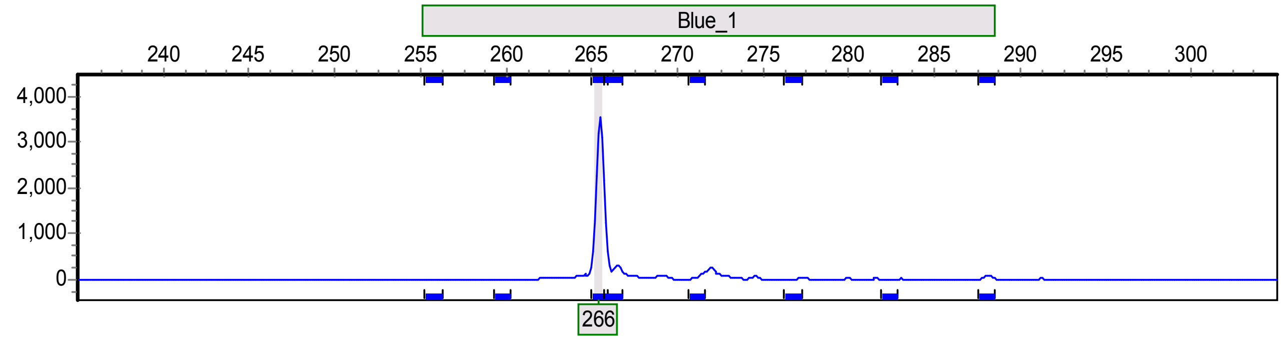

No	Size	Height	Area	Marker	Allele	Difference	Quality	Score	Allele Comments	Sample Comments
1	265.5	3516	17213	Blue_1	266	0.0	Pass	500.0		

Sample 56: Run date and time: 09/11/2020 - 19:30:25 -> 09/11/2020 - 20:26:16

Dye: Blue - 1 peaks - 6.fsa

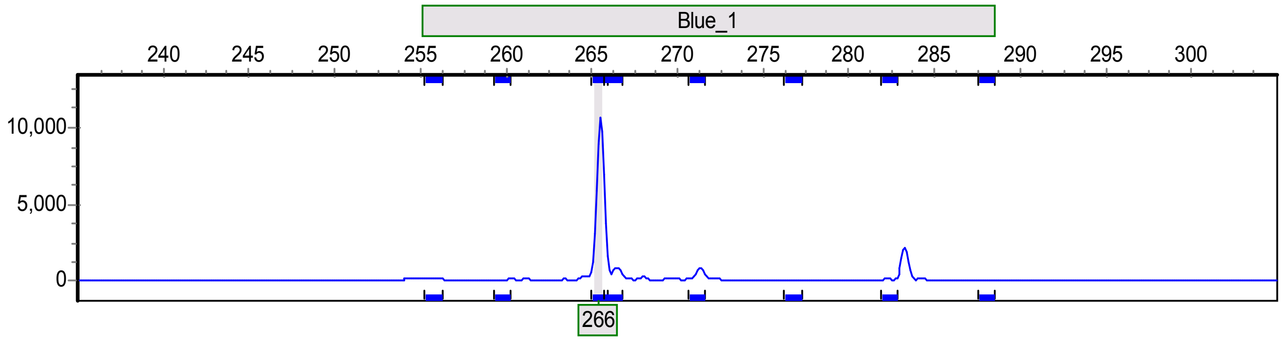

No	Size	Height	Area	Marker	Allele	Difference	Quality	Score	Allele Comments	Sample Comments
1	265.5	10644	49266	Blue_1	266	0.0	Pass	500.0		

Sample 57: Run date and time: 09/11/2020 - 19:30:25 -> 09/11/2020 - 20:26:16

Dye: Blue - 1 peaks - 60.fsa

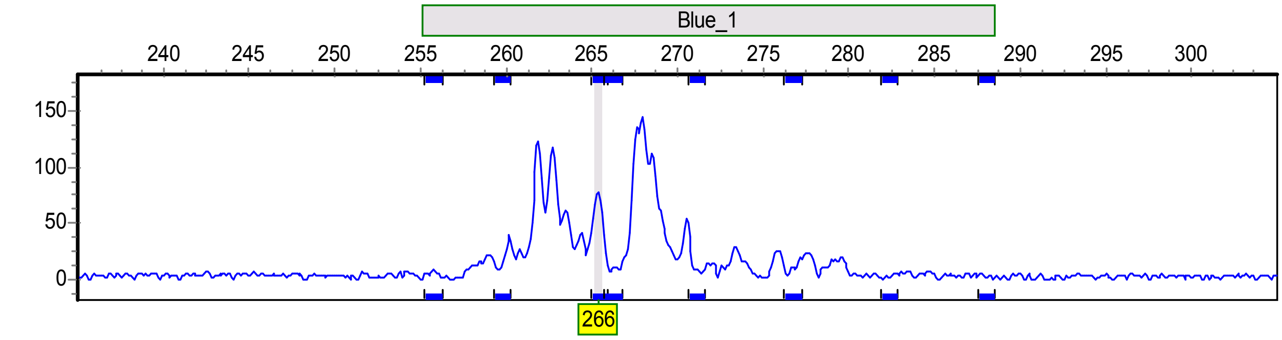

No	Size	Height	Area	Marker	Allele	Difference	Quality	Score	Allele Comments	Sample Comments
1	265.4	76	557	Blue_1	266	0.1	Check	1.3		

Allele Report

Sample 58: Run date and time: 09/11/2020 - 19:30:25 -> 09/11/2020 - 20:26:16

Dye: Blue - 1 peaks - 61.fsa

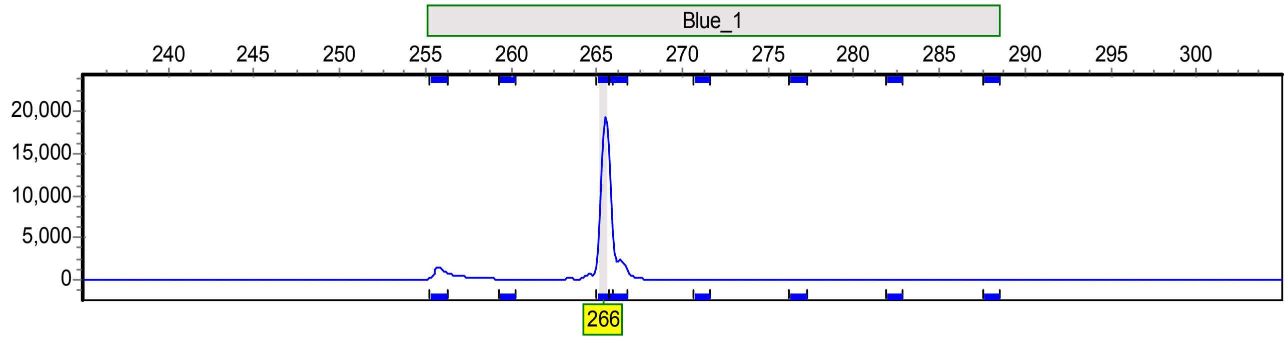

No	Size	Height	Area	Marker	Allele	Difference	Quality	Score	Allele Comments	Sample Comments
1	265.5	19262	111342	Blue_1	266	0.0	Check	500.0	[<SAT (Repaired)>]	

Sample 59: Run date and time: 09/11/2020 - 19:30:25 -> 09/11/2020 - 20:26:16

Dye: Blue - 1 peaks - 62.fsa

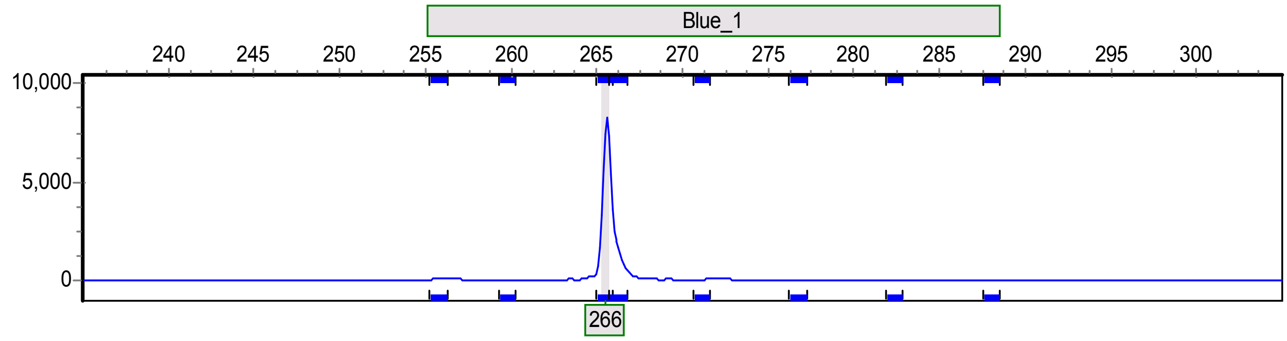

No	Size	Height	Area	Marker	Allele	Difference	Quality	Score	Allele Comments	Sample Comments
1	265.6	8205	44092	Blue_1	266	0.1	Pass	500.0		

Sample 60: Run date and time: 09/11/2020 - 19:30:25 -> 09/11/2020 - 20:26:16

Dye: Blue - 1 peaks - 63.fsa

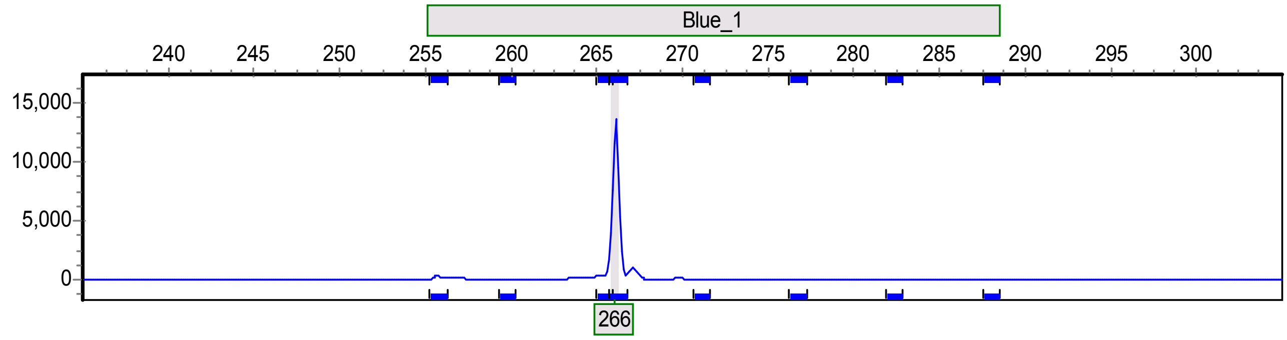

No	Size	Height	Area	Marker	Allele	Difference	Quality	Score	Allele Comments	Sample Comments
1	266.1	13652	64625	Blue_1	266	0.2	Pass	500.0		

Allele Report

Sample 61: Run date and time: 09/11/2020 - 19:30:25 -> 09/11/2020 - 20:26:16

Dye: Blue - 1 peaks - 64.fsa

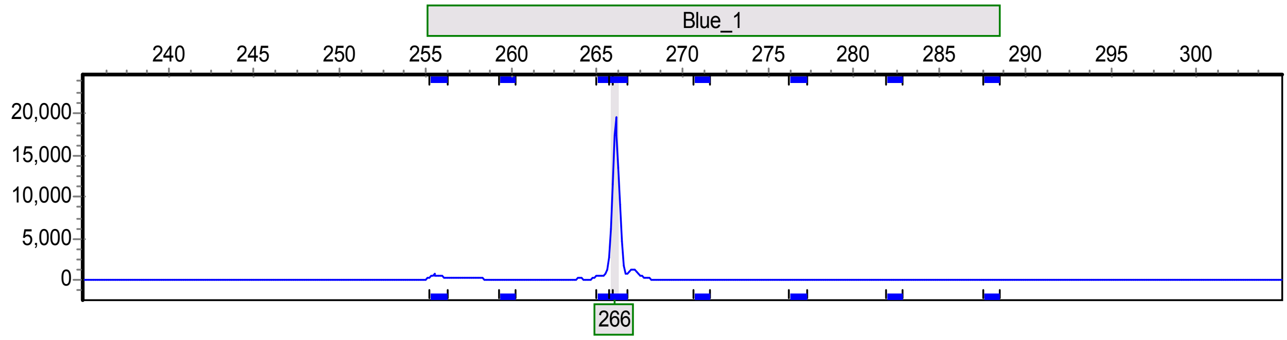

No	Size	Height	Area	Marker	Allele	Difference	Quality	Score	Allele Comments	Sample Comments
1	266.1	19443	98035	Blue_1	266	0.2	Pass	500.0		

Sample 62: Run date and time: 09/11/2020 - 19:30:25 -> 09/11/2020 - 20:26:16

Dye: Blue - 2 peaks - 65.fsa

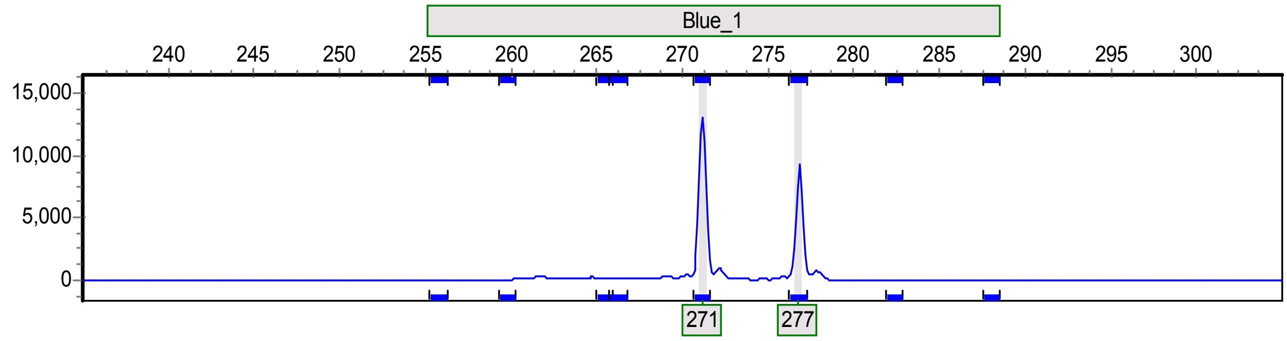

No	Size	Height	Area	Marker	Allele	Difference	Quality	Score	Allele Comments	Sample Comments
1	271.2	12978	60751	Blue_1	271	0.0	Pass	500.0		
2	276.8	9372	46478	Blue_1	277	0.0	Pass	500.0		

Sample 63: Run date and time: 09/11/2020 - 19:30:25 -> 09/11/2020 - 20:26:16

Dye: Blue - 2 peaks - 66.fsa

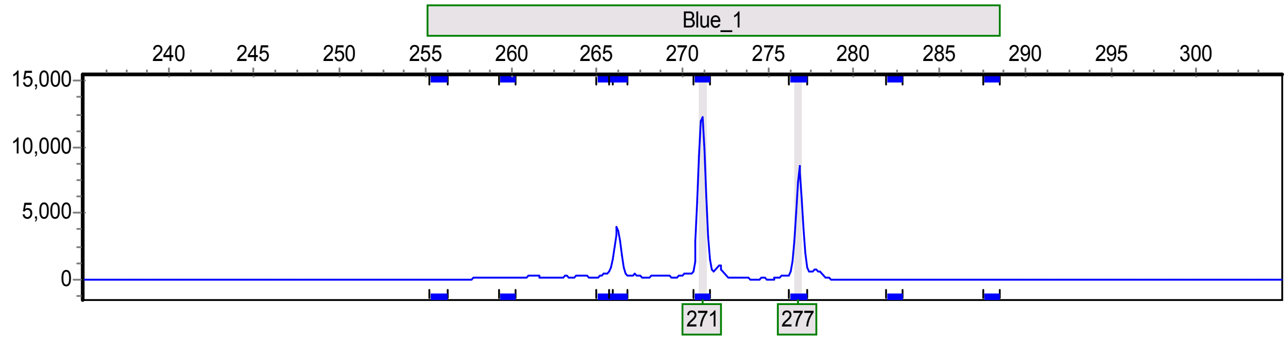

No	Size	Height	Area	Marker	Allele	Difference	Quality	Score	Allele Comments	Sample Comments
1	271.2	12192	60844	Blue_1	271	0.0	Pass	500.0		
2	276.8	8605	43866	Blue_1	277	0.0	Pass	500.0		

Allele Report

Sample 64: Run date and time: 09/11/2020 - 19:30:25 -> 09/11/2020 - 20:26:16

Dye: Blue - 2 peaks - 67.fsa

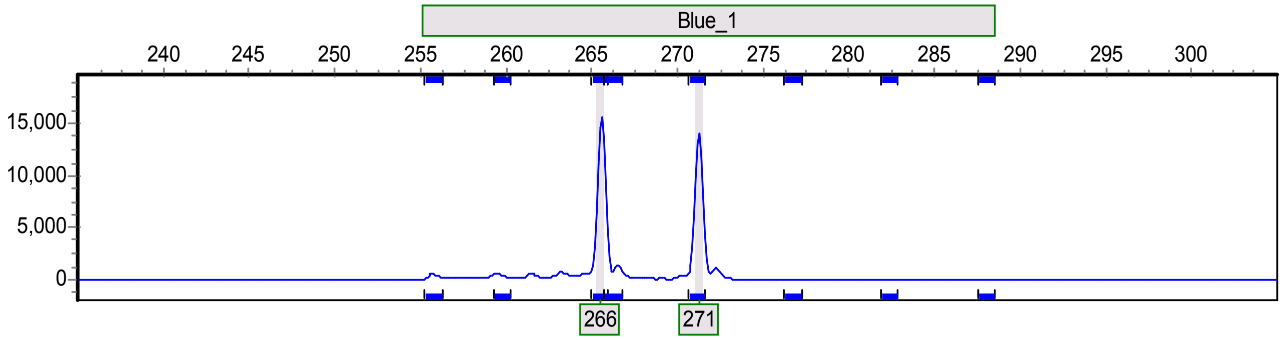

No	Size	Height	Area	Marker	Allele	Difference	Quality	Score	Allele Comments	Sample Comments
1	265.6	15449	76989	Blue_1	266	0.1	Pass	500.0		
2	271.3	13896	68919	Blue_1	271	0.1	Pass	500.0		

Sample 65: Run date and time: 09/11/2020 - 19:30:25 -> 09/11/2020 - 20:26:16

Dye: Blue - 2 peaks - 68.fsa

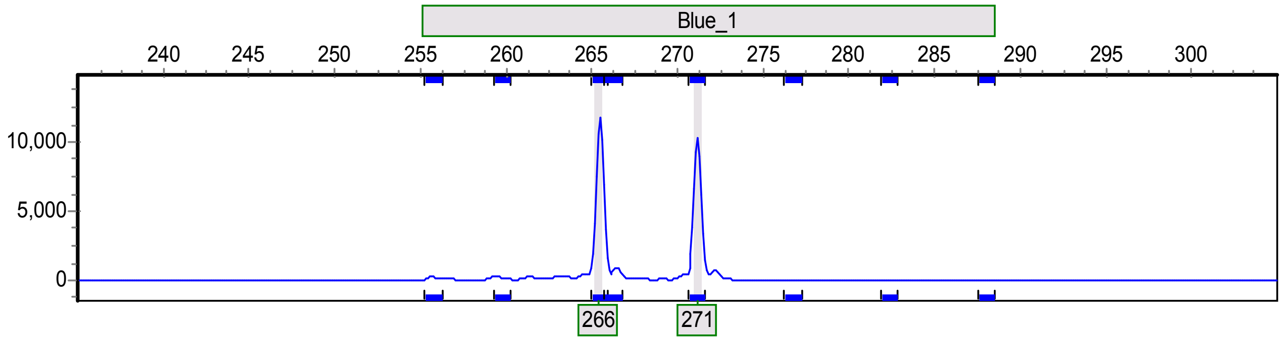

No	Size	Height	Area	Marker	Allele	Difference	Quality	Score	Allele Comments	Sample Comments
1	265.5	11648	55318	Blue_1	266	0.0	Pass	500.0		
2	271.2	10273	49599	Blue_1	271	0.0	Pass	500.0		

Sample 66: Run date and time: 09/11/2020 - 19:30:25 -> 09/11/2020 - 20:26:16

Dye: Blue - 1 peaks - 69.fsa

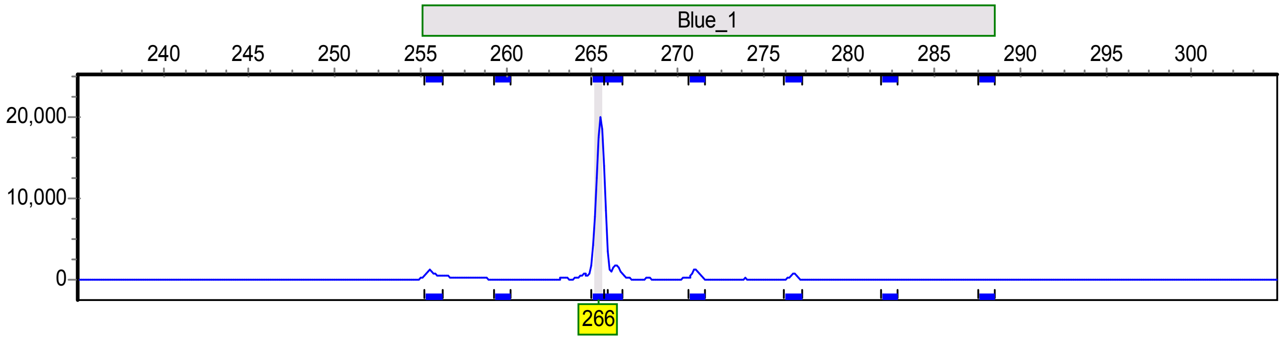

No	Size	Height	Area	Marker	Allele	Difference	Quality	Score	Allele Comments	Sample Comments
1	265.5	19775	101749	Blue_1	266	0.0	Check	500.0	[<SAT (Repaired)>]	

Sample 67: Run date and time: 09/11/2020 - 19:30:25 -> 09/11/2020 - 20:26:16

Dye: Blue - 1 peaks - 7.fsa

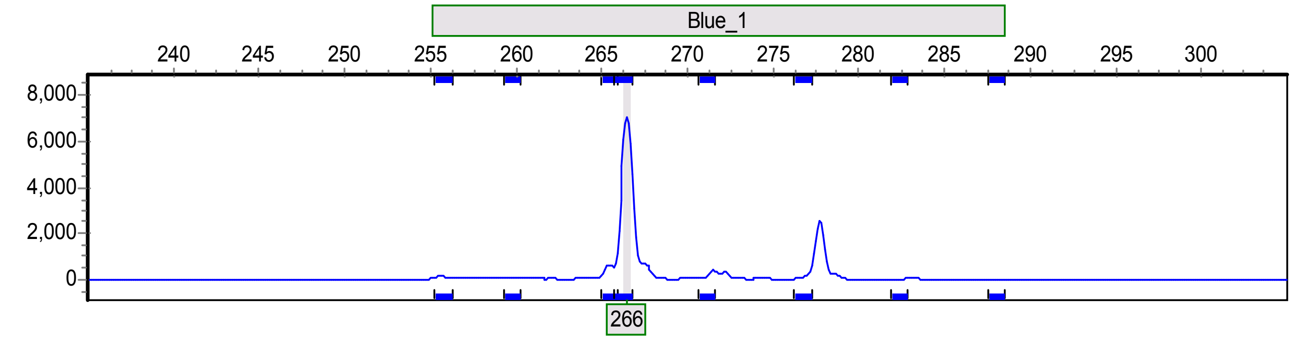

No	Size	Height	Area	Marker	Allele	Difference	Quality	Score	Allele Comments	Sample Comments
1	266.5	7000	52871	Blue_1	266	0.2	Pass	500.0		

Sample 68: Run date and time: 09/11/2020 - 19:30:25 -> 09/11/2020 - 20:26:16

Dye: Blue - 1 peaks - 70.fsa

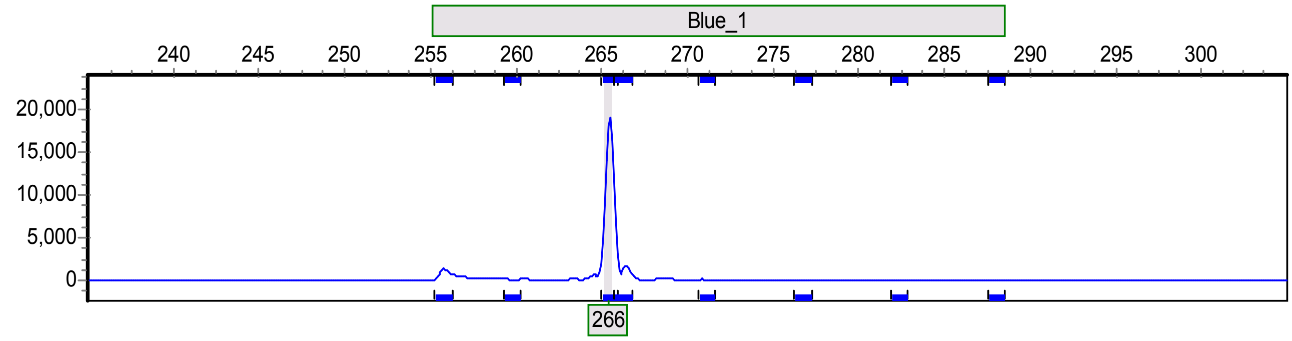

No	Size	Height	Area	Marker	Allele	Difference	Quality	Score	Allele Comments	Sample Comments
1	265.5	18992	100362	Blue_1	266	0.0	Pass	500.0		

Sample 69: Run date and time: 09/11/2020 - 19:30:25 -> 09/11/2020 - 20:26:16

Dye: Blue - 1 peaks - 71.fsa

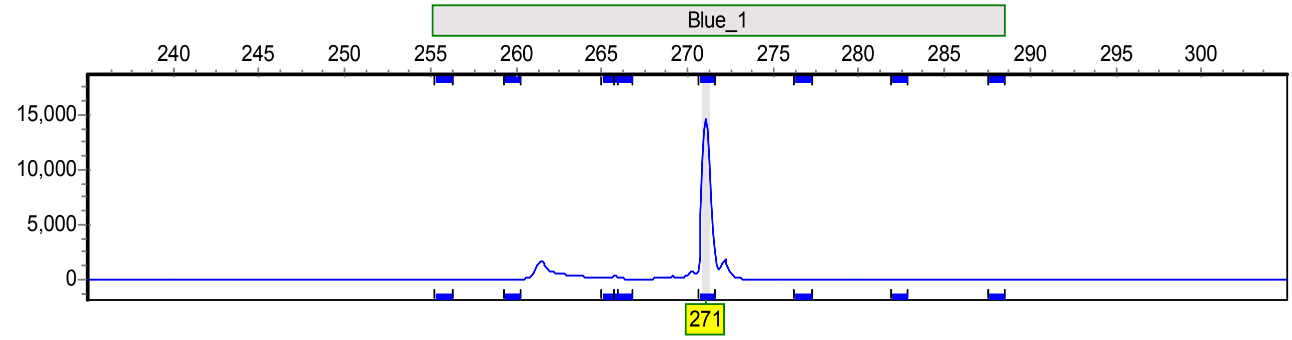

No	Size	Height	Area	Marker	Allele	Difference	Quality	Score	Allele Comments	Sample Comments
1	271.1	14730	81994	Blue_1	271	0.1	Check	500.0	[<SAT (Repaired)>]	

Allele Report

Sample 70: Run date and time: 09/11/2020 - 19:30:25 -> 09/11/2020 - 20:26:16

Dye: Blue - 1 peaks - 72.fsa

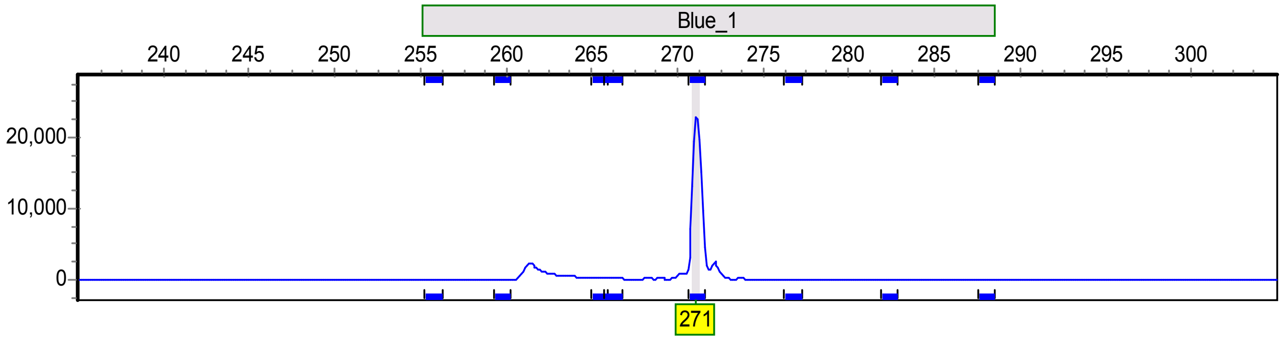

No	Size	Height	Area	Marker	Allele	Difference	Quality	Score	Allele Comments	Sample Comments
1	271.1	22761	132558	Blue_1	271	0.1	Check	500.0	[<SAT (Repaired)>]	

Sample 71: Run date and time: 09/11/2020 - 19:30:25 -> 09/11/2020 - 20:26:16

Dye: Blue - 1 peaks - 73.fsa

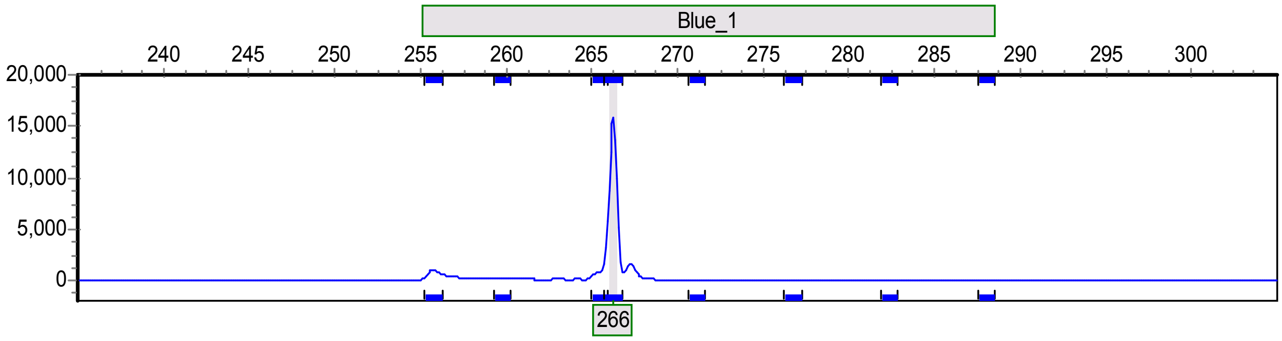

No	Size	Height	Area	Marker	Allele	Difference	Quality	Score	Allele Comments	Sample Comments
1	266.3	15785	88864	Blue_1	266	0.0	Pass	500.0		

Sample 72: Run date and time: 09/11/2020 - 19:30:25 -> 09/11/2020 - 20:26:16

Dye: Blue - 1 peaks - 74.fsa

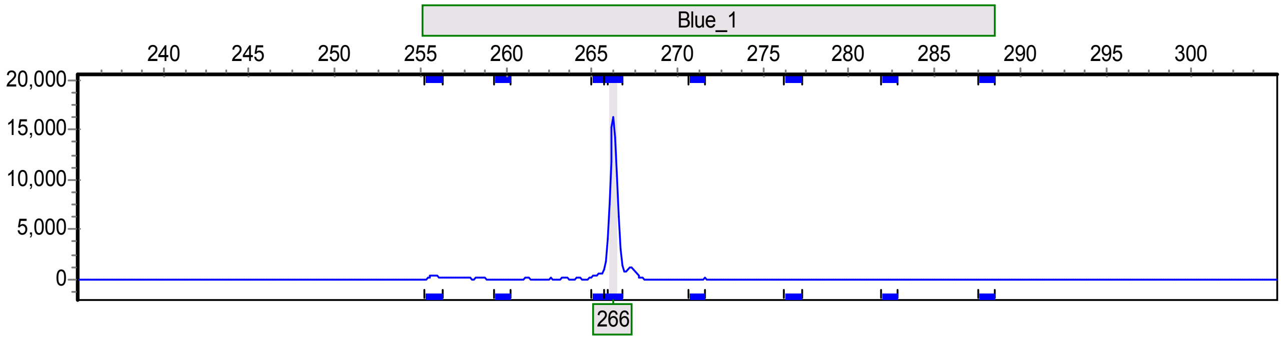

No	Size	Height	Area	Marker	Allele	Difference	Quality	Score	Allele Comments	Sample Comments
1	266.3	16180	86478	Blue_1	266	0.0	Pass	500.0		

Sample 73: Run date and time: 09/11/2020 - 19:30:25 -> 09/11/2020 - 20:26:16

Dye: Blue - 2 peaks - 75.fsa

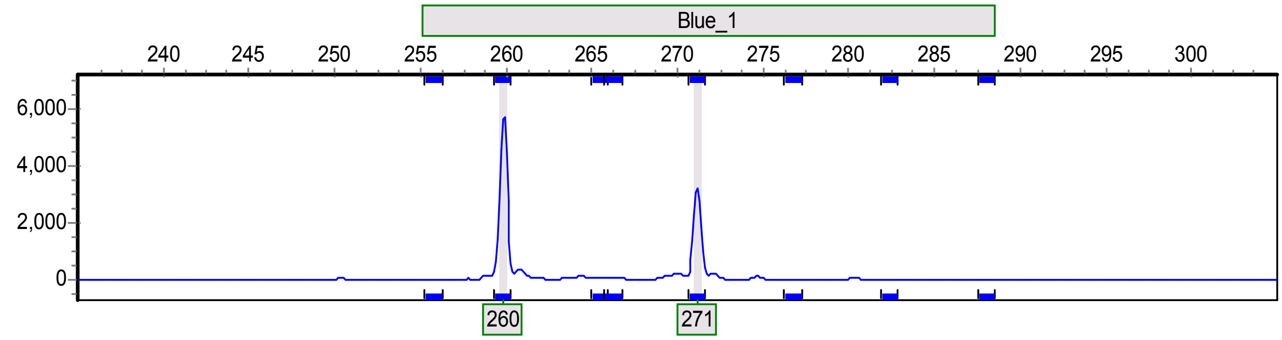

No	Size	Height	Area	Marker	Allele	Difference	Quality	Score	Allele Comments	Sample Comments
1	259.9	5703	28539	Blue_1	260	0.1	Pass	500.0		
2	271.2	3255	16075	Blue_1	271	0.0	Pass	500.0		

Sample 74: Run date and time: 09/11/2020 - 19:30:25 -> 09/11/2020 - 20:26:16

Dye: Blue - 2 peaks - 76.fsa

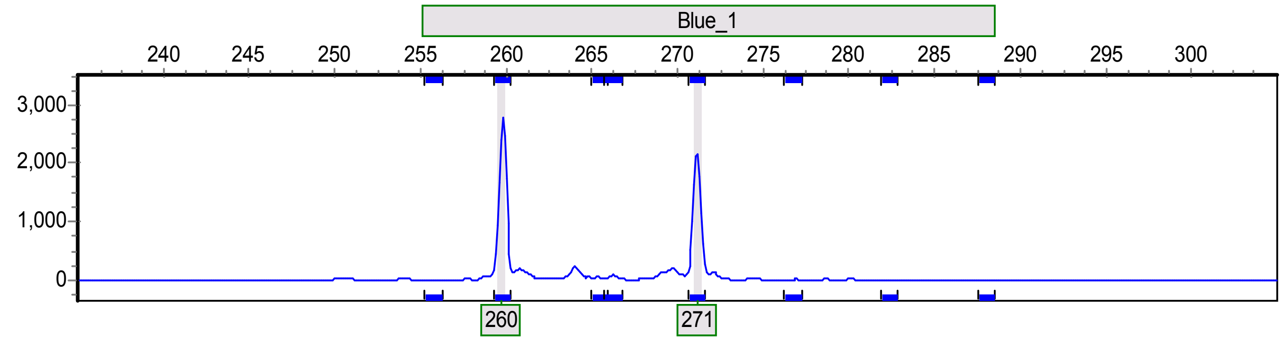

No	Size	Height	Area	Marker	Allele	Difference	Quality	Score	Allele Comments	Sample Comments
1	259.8	2765	13276	Blue_1	260	0.0	Pass	500.0		
2	271.2	2167	10860	Blue_1	271	0.0	Pass	500.0		

Sample 75: Run date and time: 09/11/2020 - 19:30:25 -> 09/11/2020 - 20:26:16

Dye: Blue - 1 peaks - 77.fsa

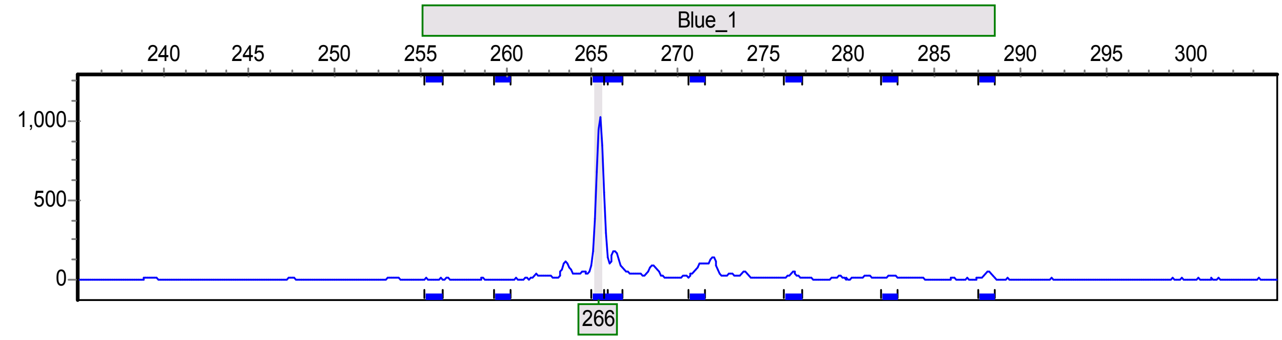

No	Size	Height	Area	Marker	Allele	Difference	Quality	Score	Allele Comments	Sample Comments
1	265.5	1025	4804	Blue_1	266	0.0	Pass	234.7		

Sample 76: Run date and time: 09/11/2020 - 19:30:25 -> 09/11/2020 - 20:26:16

Dye: Blue - 2 peaks - 78.fsa

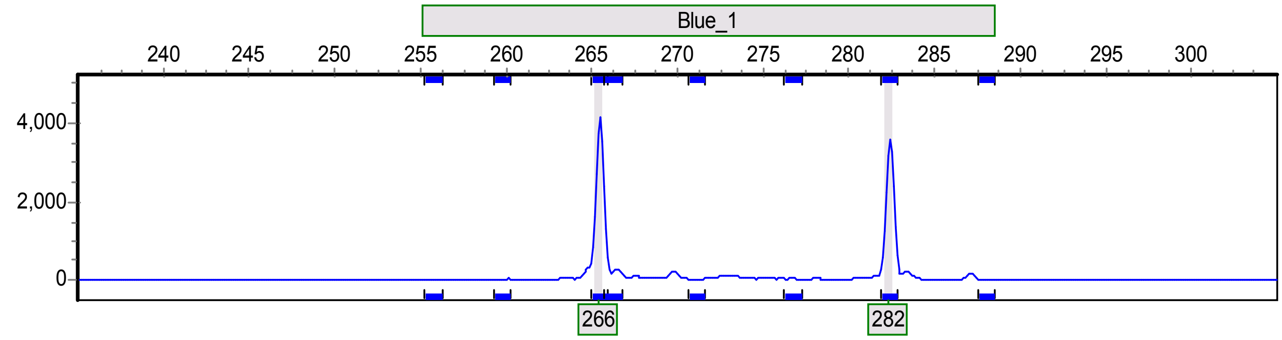

No	Size	Height	Area	Marker	Allele	Difference	Quality	Score	Allele Comments	Sample Comments
1	265.5	4135	19859	Blue_1	266	0.0	Pass	500.0		
2	282.4	3585	17631	Blue_1	282	0.0	Pass	500.0		

Sample 77: Run date and time: 09/11/2020 - 19:30:25 -> 09/11/2020 - 20:26:16

Dye: Blue - 1 peaks - 79.fsa

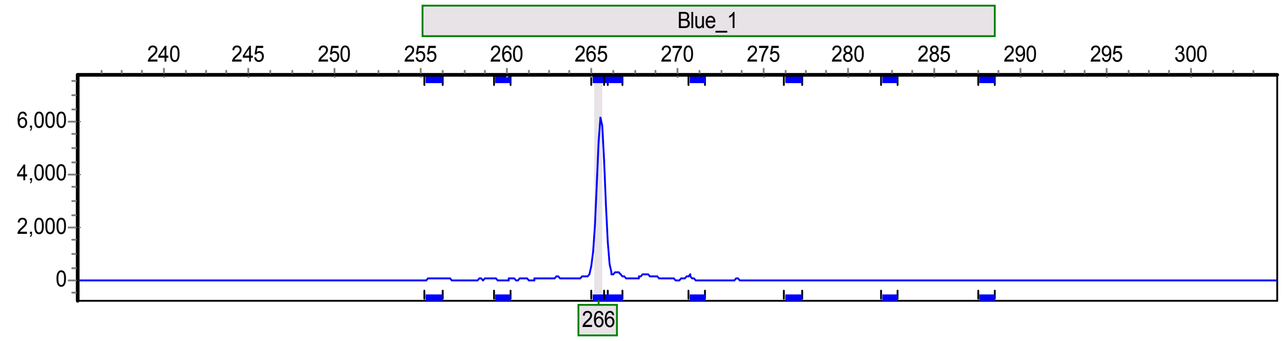

No	Size	Height	Area	Marker	Allele	Difference	Quality	Score	Allele Comments	Sample Comments
1	265.5	6140	31838	Blue_1	266	0.0	Pass	500.0		

Sample 78: Run date and time: 09/11/2020 - 19:30:25 -> 09/11/2020 - 20:26:16

Dye: Blue - 1 peaks - 8.fsa

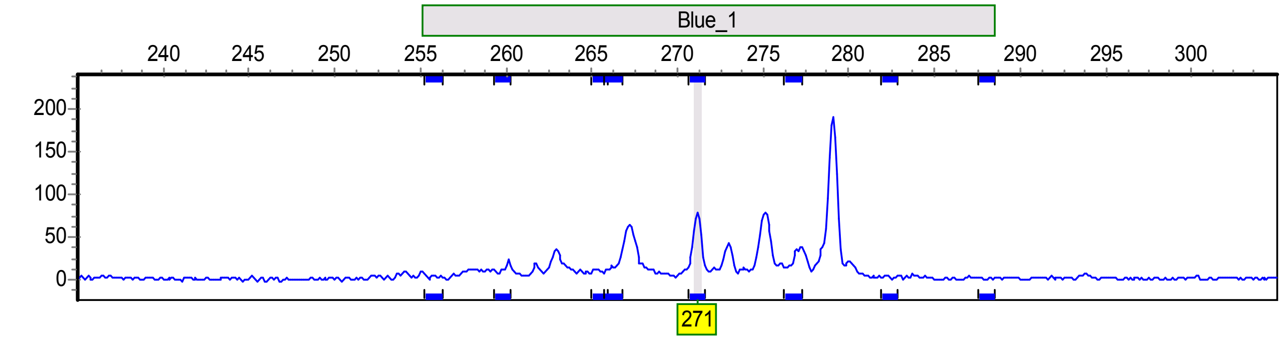

No	Size	Height	Area	Marker	Allele	Difference	Quality	Score	Allele Comments	Sample Comments
1	271.2	78	416	Blue_1	271	0.0	Check	2.4		

Sample 79: Run date and time: 09/11/2020 - 19:30:25 -> 09/11/2020 - 20:26:16

Dye: Blue - 2 peaks - 80.fsa

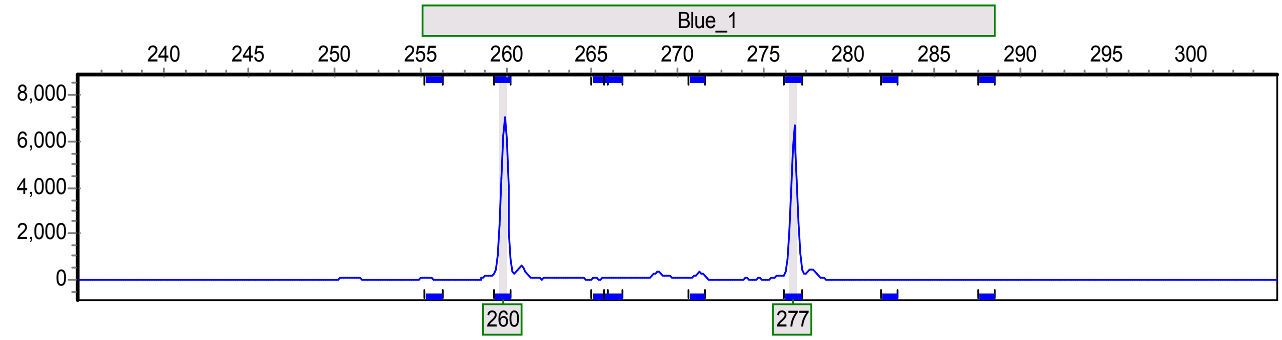

No	Size	Height	Area	Marker	Allele	Difference	Quality	Score	Allele Comments	Sample Comments
1	259.9	7027	32552	Blue_1	260	0.1	Pass	500.0		
2	276.8	6714	31727	Blue_1	277	0.0	Pass	500.0		

Sample 80: Run date and time: 09/11/2020 - 19:30:25 -> 09/11/2020 - 20:26:16

Dye: Blue - 2 peaks - 81.fsa

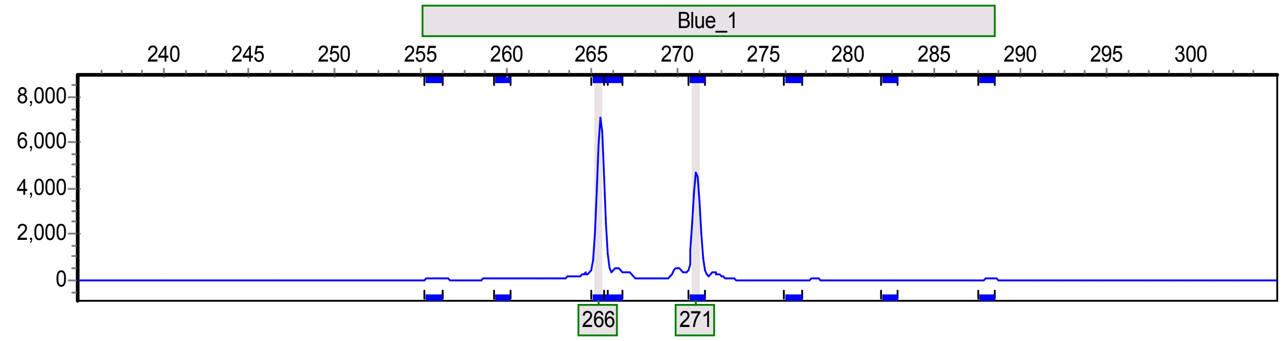

No	Size	Height	Area	Marker	Allele	Difference	Quality	Score	Allele Comments	Sample Comments
1	265.5	7036	32679	Blue_1	266	0.0	Pass	500.0		
2	271.1	4698	22980	Blue_1	271	0.1	Pass	500.0		

Sample 81: Run date and time: 09/11/2020 - 19:30:25 -> 09/11/2020 - 20:26:16

Dye: Blue - 1 peaks - 82.fsa

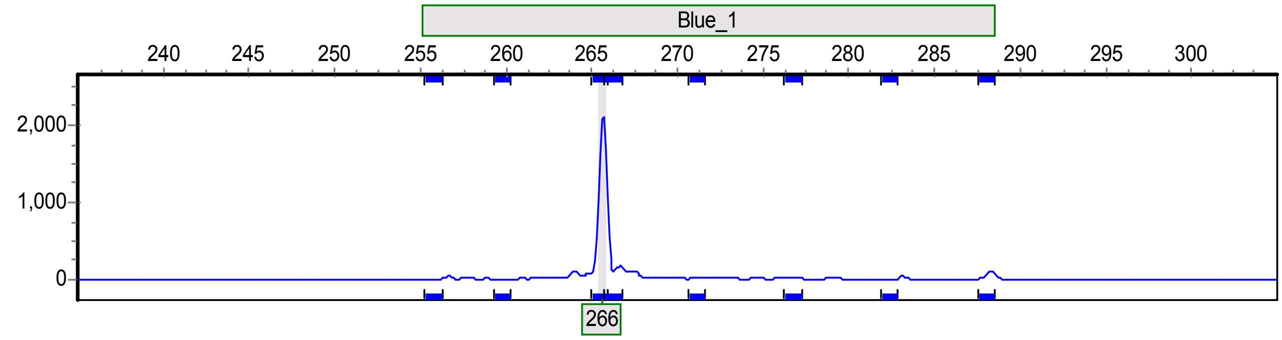

No	Size	Height	Area	Marker	Allele	Difference	Quality	Score	Allele Comments	Sample Comments
1	265.7	2100	10576	Blue_1	266	0.2	Pass	500.0		

Allele Report

Sample 82: Run date and time: 09/11/2020 - 19:30:25 -> 09/11/2020 - 20:26:16

Dye: Blue - 1 peaks - 83.fsa

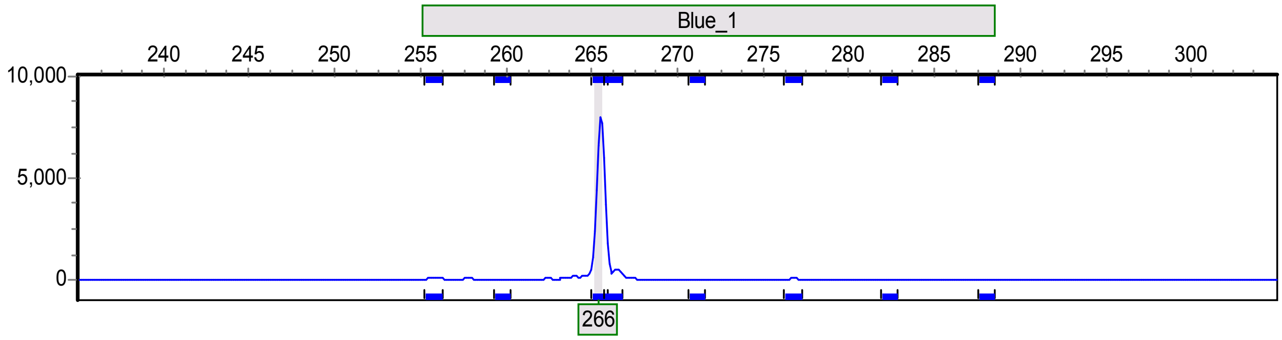

No	Size	Height	Area	Marker	Allele	Difference	Quality	Score	Allele Comments	Sample Comments
1	265.5	7926	39869	Blue_1	266	0.0	Pass	500.0		

Sample 83: Run date and time: 09/11/2020 - 19:30:25 -> 09/11/2020 - 20:26:16

Dye: Blue - 1 peaks - 84.fsa

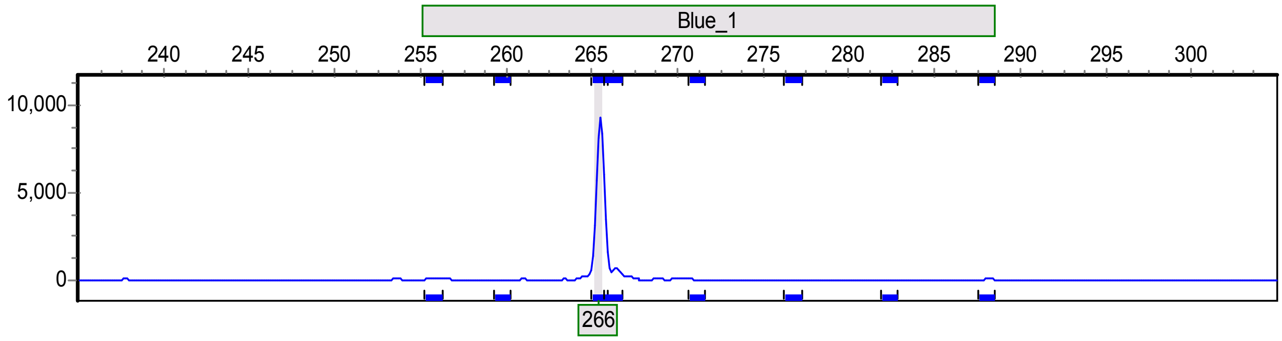

No	Size	Height	Area	Marker	Allele	Difference	Quality	Score	Allele Comments	Sample Comments
1	265.5	9219	44909	Blue_1	266	0.0	Pass	500.0		

Sample 84: Run date and time: 09/11/2020 - 19:30:25 -> 09/11/2020 - 20:26:16

Dye: Blue - 2 peaks - 85.fsa

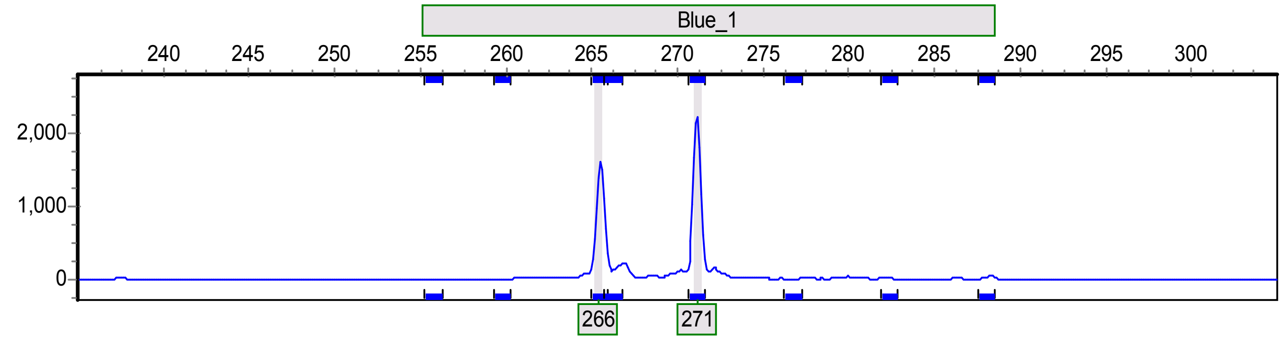

No	Size	Height	Area	Marker	Allele	Difference	Quality	Score	Allele Comments	Sample Comments
1	265.5	1623	8210	Blue_1	266	0.0	Pass	409.9		
2	271.2	2222	11095	Blue_1	271	0.0	Pass	500.0		

Allele Report

Sample 85: Run date and time: 09/11/2020 - 19:30:25 -> 09/11/2020 - 20:26:16

Dye: Blue - 2 peaks - 86.fsa

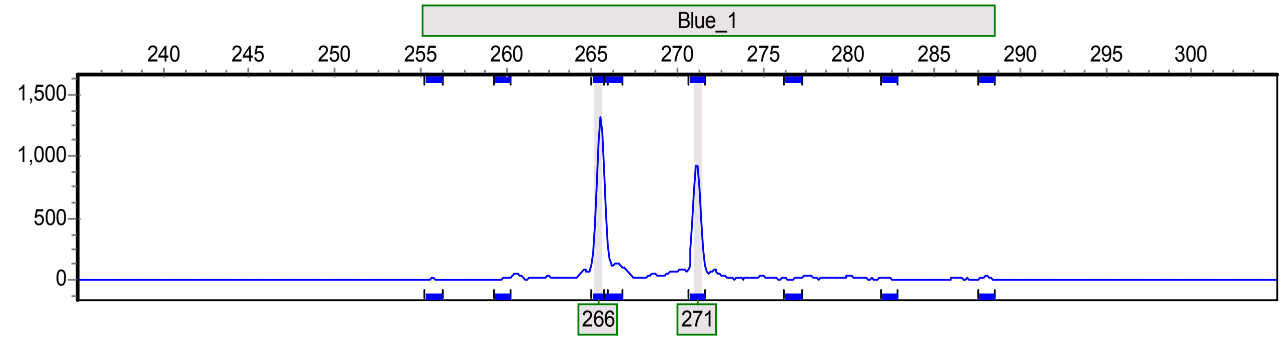

No	Size	Height	Area	Marker	Allele	Difference	Quality	Score	Allele Comments	Sample Comments
1	265.5	1305	6536	Blue_1	266	0.0	Pass	305.1		
2	271.2	920	4690	Blue_1	271	0.0	Pass	180.5		

Sample 86: Run date and time: 09/11/2020 - 19:30:25 -> 09/11/2020 - 20:26:16

Dye: Blue - 1 peaks - 87.fsa

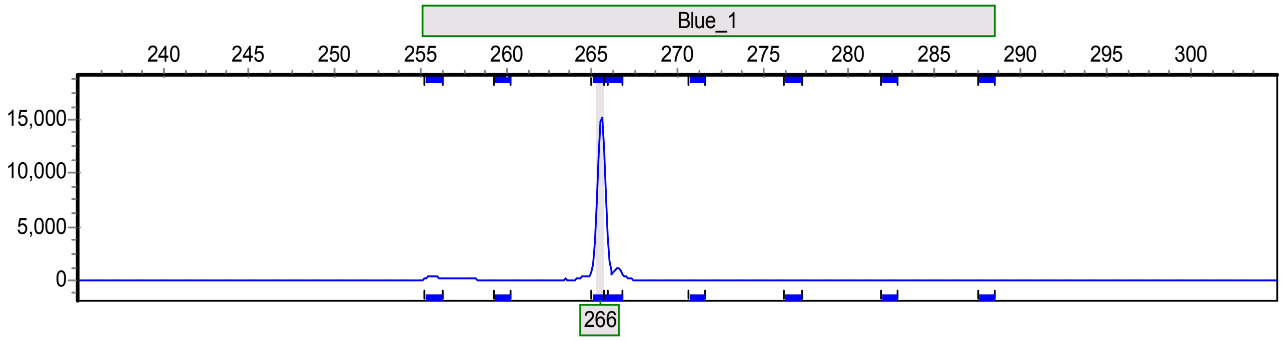

No	Size	Height	Area	Marker	Allele	Difference	Quality	Score	Allele Comments	Sample Comments
1	265.6	15038	74989	Blue_1	266	0.1	Pass	500.0		

Sample 87: Run date and time: 09/11/2020 - 19:30:25 -> 09/11/2020 - 20:26:16

Dye: Blue - 1 peaks - 88.fsa

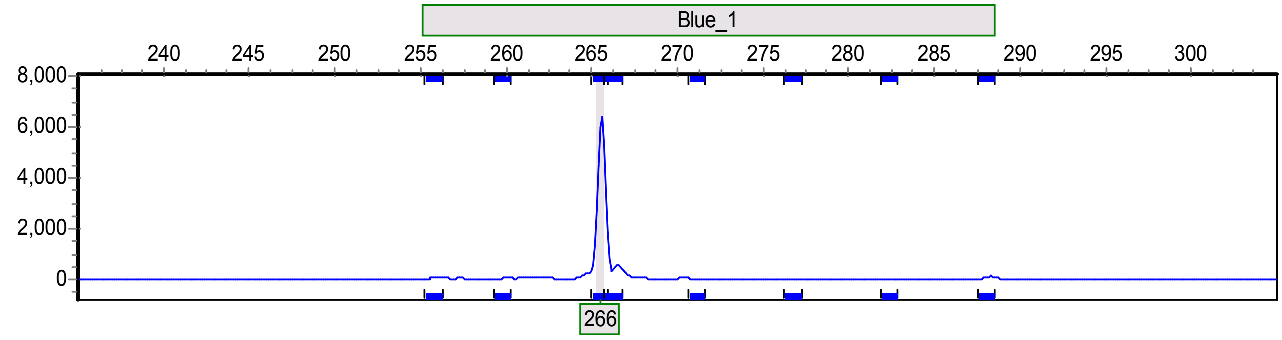

No	Size	Height	Area	Marker	Allele	Difference	Quality	Score	Allele Comments	Sample Comments
1	265.6	6347	30725	Blue_1	266	0.1	Pass	500.0		

Allele Report

Sample 88: Run date and time: 09/11/2020 - 19:30:25 -> 09/11/2020 - 20:26:16

Dye: Blue - 1 peaks - 89.fsa

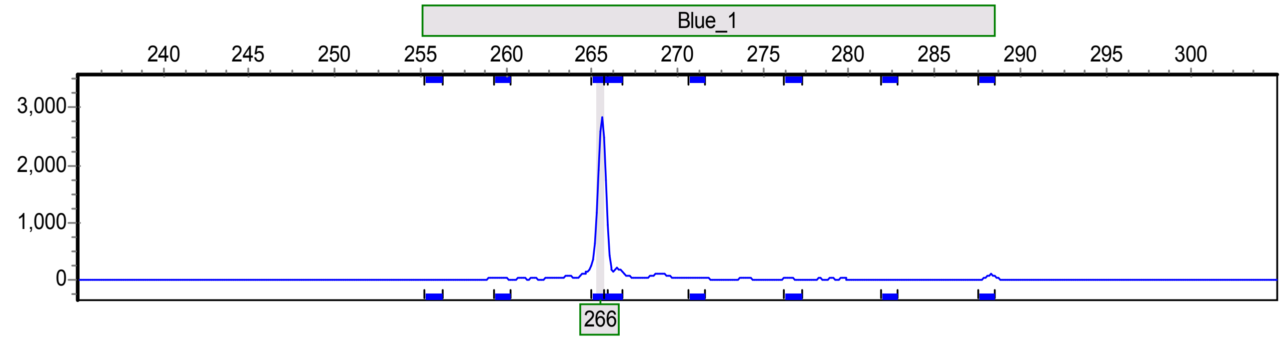

No	Size	Height	Area	Marker	Allele	Difference	Quality	Score	Allele Comments	Sample Comments
1	265.6	2816	14267	Blue_1	266	0.1	Pass	500.0		

Sample 89: Run date and time: 09/11/2020 - 19:30:25 -> 09/11/2020 - 20:26:16

Dye: Blue - 1 peaks - 9.fsa

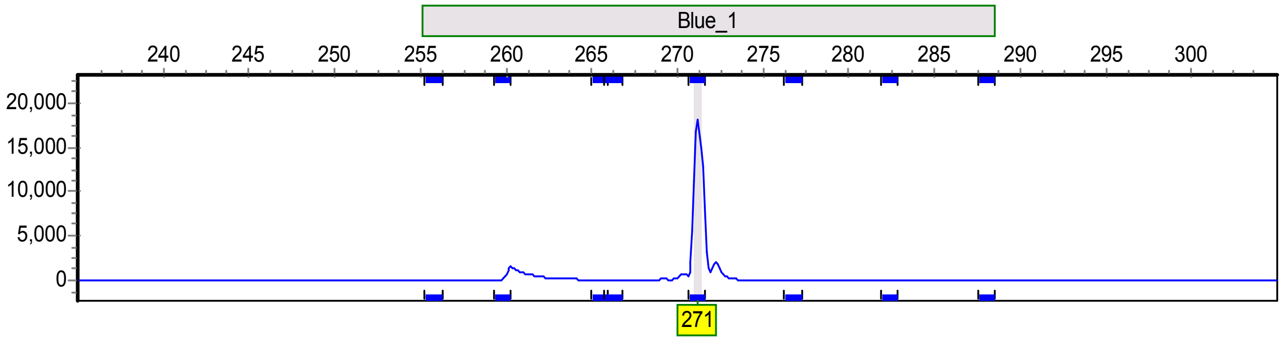

No	Size	Height	Area	Marker	Allele	Difference	Quality	Score	Allele Comments	Sample Comments
1	271.2	18246	105246	Blue_1	271	0.0	Check	500.0	[<SAT (Repaired)>]	

Sample 90: Run date and time: 09/11/2020 - 19:30:25 -> 09/11/2020 - 20:26:16

Dye: Blue - 2 peaks - 90.fsa

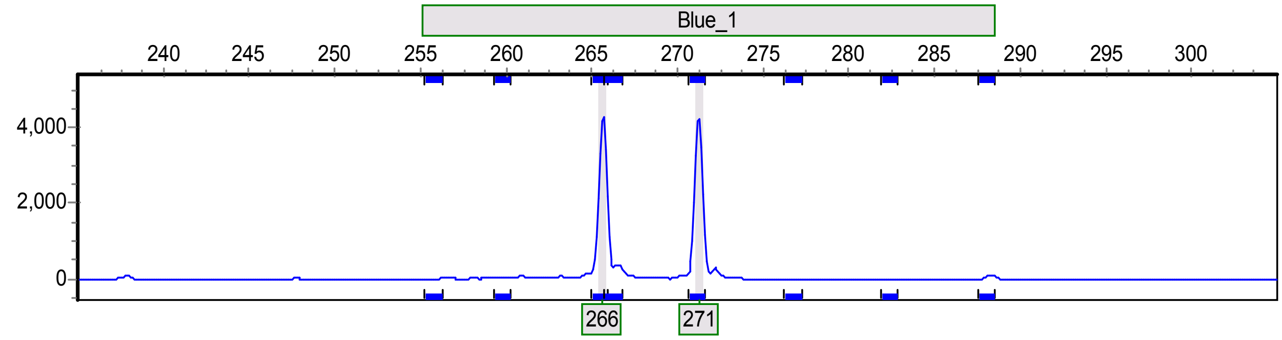

No	Size	Height	Area	Marker	Allele	Difference	Quality	Score	Allele Comments	Sample Comments
1	265.7	4229	21474	Blue_1	266	0.2	Pass	500.0		
2	271.3	4218	21138	Blue_1	271	0.1	Pass	500.0		

Sample 91: Run date and time: 09/11/2020 - 19:30:25 -> 09/11/2020 - 20:26:16

Dye: Blue - 2 peaks - 91.fsa

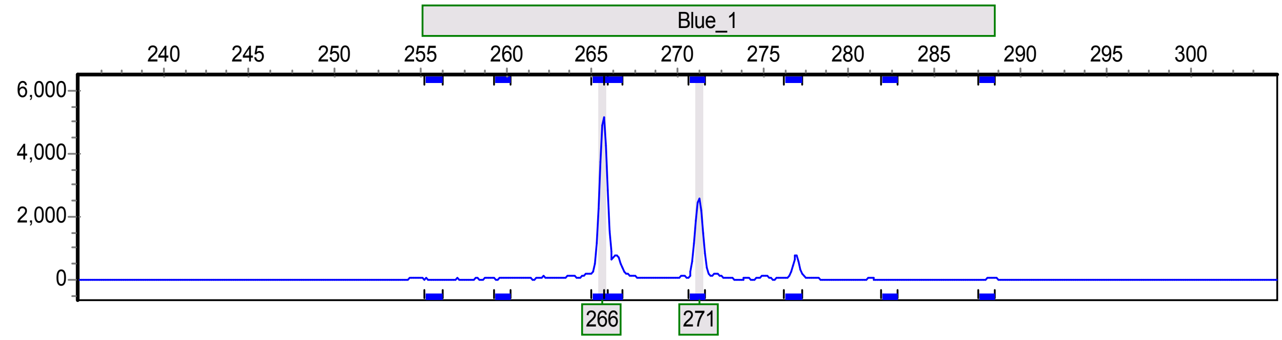

No	Size	Height	Area	Marker	Allele	Difference	Quality	Score	Allele Comments	Sample Comments
1	265.7	5156	25888	Blue_1	266	0.2	Pass	500.0		
2	271.3	2608	13172	Blue_1	271	0.1	Pass	500.0		

Sample 92: Run date and time: 09/11/2020 - 19:30:25 -> 09/11/2020 - 20:26:16

Dye: Blue - 1 peaks - 92.fsa

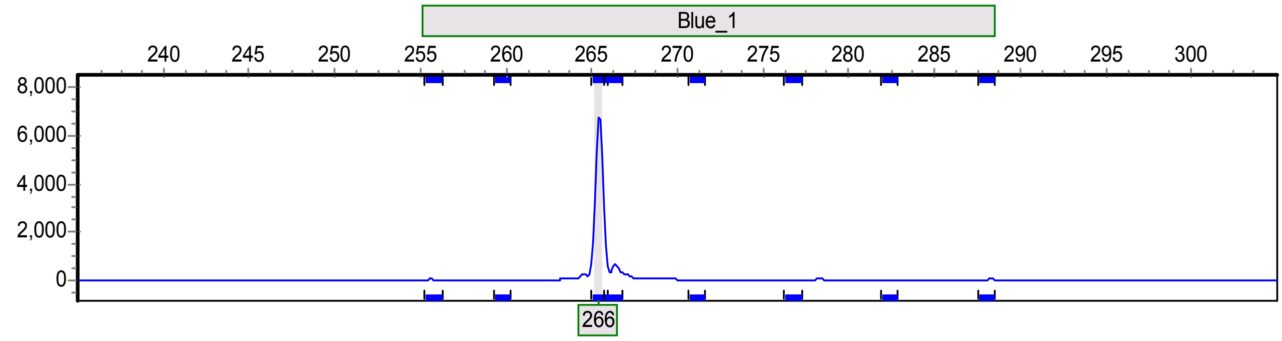

No	Size	Height	Area	Marker	Allele	Difference	Quality	Score	Allele Comments	Sample Comments
1	265.4	6697	32195	Blue_1	266	0.1	Pass	500.0		

Sample 93: Run date and time: 09/11/2020 - 19:30:25 -> 09/11/2020 - 20:26:16

Dye: Blue - 2 peaks - 93.fsa

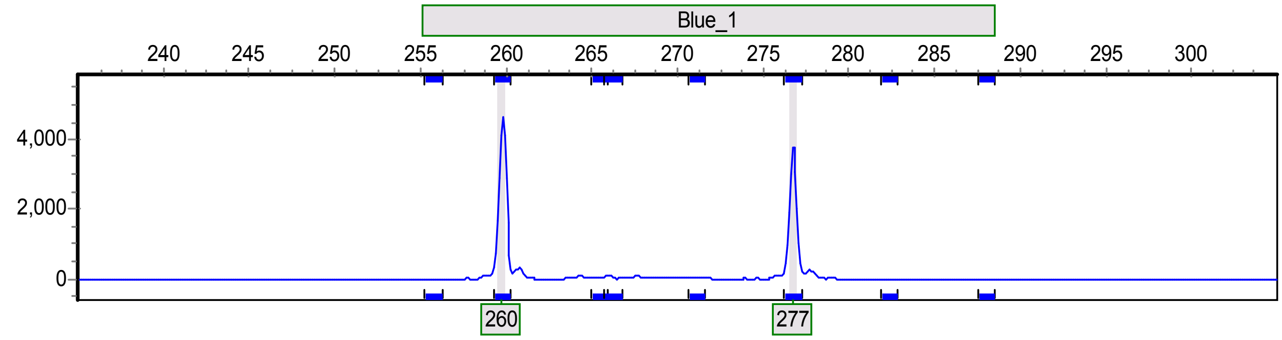

No	Size	Height	Area	Marker	Allele	Difference	Quality	Score	Allele Comments	Sample Comments
1	259.8	4597	22338	Blue_1	260	0.0	Pass	500.0		
2	276.8	3769	19421	Blue_1	277	0.0	Pass	500.0		

Sample 94: Run date and time: 09/11/2020 - 19:30:25 -> 09/11/2020 - 20:26:16

Dye: Blue - 2 peaks - 94.fsa

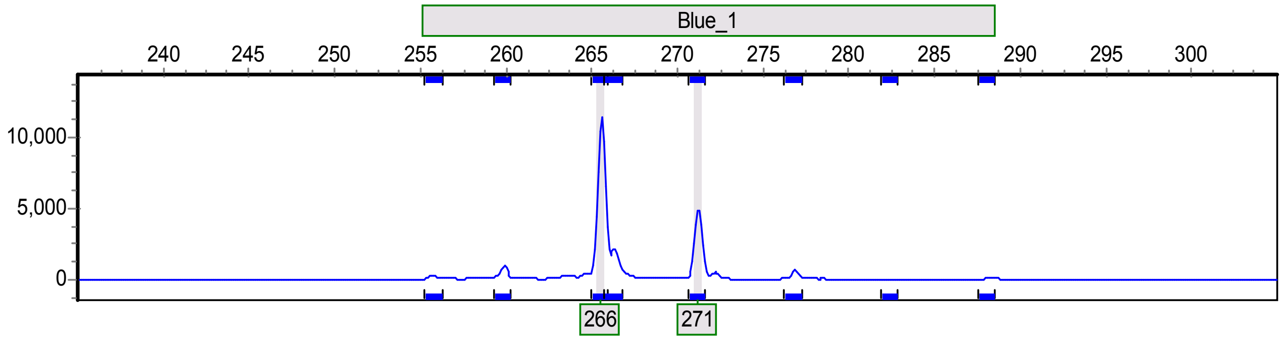

No	Size	Height	Area	Marker	Allele	Difference	Quality	Score	Allele Comments	Sample Comments
1	265.6	11305	55427	Blue_1	266	0.1	Pass	500.0		
2	271.2	4818	24844	Blue_1	271	0.0	Pass	500.0		

Sample 95: Run date and time: 09/11/2020 - 19:30:25 -> 09/11/2020 - 20:26:16

Dye: Blue - 3 peaks - 95.fsa

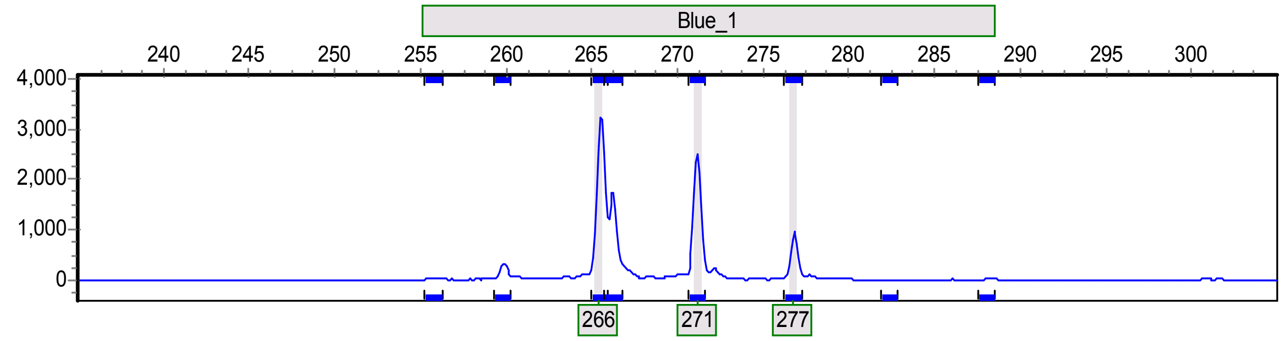

No	Size	Height	Area	Marker	Allele	Difference	Quality	Score	Allele Comments	Sample Comments
1	265.5	3205	17008	Blue_1	266	0.0	Pass	500.0		
2	271.2	2505	12451	Blue_1	271	0.0	Pass	500.0		
3	276.8	972	4916	Blue_1	277	0.0	Pass	198.3		

Sample 96: Run date and time: 09/11/2020 - 19:30:25 -> 09/11/2020 - 20:26:16

Dye: Blue - 2 peaks - 96.fsa

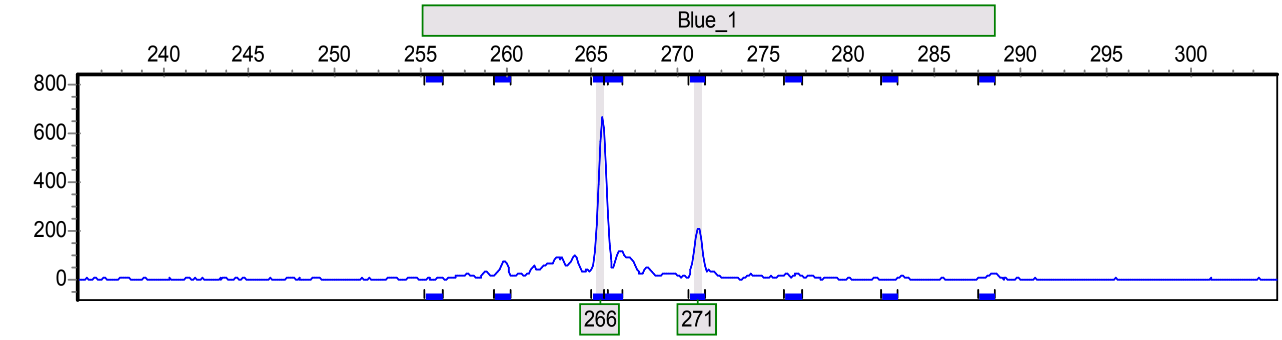

No	Size	Height	Area	Marker	Allele	Difference	Quality	Score	Allele Comments	Sample Comments
1	265.6	664	3364	Blue_1	266	0.1	Pass	112.1		
2	271.2	209	1123	Blue_1	271	0.0	Pass	15.3		
