# Supplementary material for: Genetic diversity and population structure of Polygonatum cyrtonema Hua in China using SSR markers
Source: PLoS One. 2023 Aug 31;18(8):e0290605. doi: 10.1371/journal.pone.0290605 (PMC10470896; doi:10.1371/journal.pone.0290605)
Supplement: S1 File — (ZIP) [file pone.0290605.s003.zip › Supporting Information/S1 Table. SSR markers that have been reported for use in related studies..docx]

**S1 Table.** **SSR markers that have been reported for use in related studies.**

| **Primer name** | **Repetition motif** | **Primer sequences** | **Temperature(℃)** | **Length (bp)** | **References** |
| --- | --- | --- | --- | --- | --- |
| Pc1 | G_8_(GA)_32_ | F: CTCTCCTATCGGCAGCAACT  R: ACTTCCTCCATCCTTACACCAT | 55.0 | 197–245 | Cheng W, Liu T, Wu H, et al. Isolation and characterization of twelve polymorphic microsatellite loci in Polygonatum cyrtonema and cross-species amplification[J]. Conservation Genetics Resources, 2010, 2: 105-107.  CHEN Y W. Analysis on SSR loci in transcriptome and development of molecular markers in Polygonatum cyrtonema[J]. Chinese Traditional and Herbal Drugs, 2020: 182-189.  Chen H, Qian H, Xu Z et al. SSR Loci Information Analysis in Polygonatum kingianum Transcriptome[J]. Northern Horticulture,2022,No.517(22):104-109.  籍蓉蓉、刘跃军，程文娟、刘新 |
| Pc11* | (AG)_48_ | F: TCTTGCTCTACCTCCTTGCTTCT  R: GTCACACCTTTCCCTCTACTTAAC | 53.0 | 164–240 |  |
| Pc14 | (AG)_21_G(GA)_22_ | F: AGGAAATGGAGATGAGTGATGC  R: CTGTTGATTCATCTGGTGCTTG | 53.0 | 225–273 |  |
| Pc17 | (AG)_40_ | F: GGACACCCGAAGAAATACAAG  R: CCAATTGCCTCCTTCACATC | 55.0 | 170–211 |  |
| Pc18* | (AG)_48_(AGAC)_5_ | F: CATGCTATCTCCCCTCACTTG  R: TGATTATCTGGTGCCGGAC | 55.0 | 150–283 |  |
| Pc25* | (CT)_8_CC(CT)_18_(TA)_5_ | F: CTCCCTTTCCCAATCCCGT  R: CCCAACATCTCGTAGTCGCAA | 58.0 | 205–260 |  |
| Pc26 | (TC)_23_T(TC)_5_ | F: GCCTTTGATTATCTGGTATCGGAC R: TCTTGGTTGCTAGGGAGGACA | 52.0 | 230–275 |  |
| Pc28 | (CT)_23_ | F: TTAAGTCTTCAGACCCGTCAACC  R: GATTCAATCAAAGTCCACCTCG | 54.0 | 206–265 |  |
| Pc30 | (GAA)_3_(AG)_34_ | F: AGCAAGAAAAGGGCACTCG  R: CCCTCCCCTTCATAATTGCT | 55.0 | 193–205 |  |
| Pc33 | (GA)_34_ | F: CGCACCCAGACCGAGAAA  R: GTAGGCAAGGAACACCCACAC | 59.0 | 225–275 |  |
| Pc34 | (AG)_8_G(GA)_41_ | F: GCTTAGAGTGGAAGAAATGGAGTAG R: GTTGATTCATCTGGTGCTTGTCTC | 59.0 | 218–241 |  |
| Pc48 | (GA)_46_ | F: TTTGGCAATGATTTGAGGG  R: CTGTGTGTTTATTCTGTAGAGTTGC | 55.0 | 190–195 |  |
| 0011643 | (AT)_16_ | F: CCATGTATGGCCATCCTCTA  R: GCAAGATGAATGAAATCG | 52.0 | 262 |  |
| 0096227 | (TG)_10_(AG)_6_ | F: GTCCGAGTTCTTTGACGAGC  R: AAAACCATCTCCATCCTCCC | 56.0 | 228 |  |
| 0123080 | (GA)_6_AC(AG)_9_ | F: ATGGAAGGGAGAGAGGGAGA  R: CCTTCCATCTACTGGAAACCC | 57.5 | 122 |  |
| 0098494 | (CCTCTC)_4_(CT)_6_ | F:CTGCTCTCTCACCAAACCCT  R: TAGAATCCTGGACCTCGTCG | 57.0 | 176 |  |
| 0016356 | (TACTGC)_5_ | F: GCTACATAAAGAGCCTGCGG  R: CATGGAGCACTCTAGACTCAAAG | 56.0 | 270 |  |
| 0073389 | (TAACCC)_4_ | F: CGAAGAAGTCTCGATCCACC  R:TCGAGGAGTGCTTGATGATG | 56.0 | 255 |  |
| 0085001 | (TCT)_5_(TGC)_5_ | F:ACCTCGAAAGGGTTGTCCTT  R: CGAAGACGAAGACCGAGTTC | 56.0 | 154 |  |
| 0016720 | (GA)_7_GGT(GA)_6_ | F: GACCAAAAAGCTCCCCTTTC  R: TTAACGCCTAACCCCAACTG | 55.0 | 243 |  |
| 0003599 | (CT)_2_ | F: TCTGTAAATCAGGAGGAGCGA  R: AATCGATGAGATCGACCTGG | 54.0 | 141 |  |
| 0092927 | (ACC)_9_ | F: CGAAACCCTCCTCAATCTCA  R: GCGACAAGTGTTGAAGGGTT | 54.5 | 193 |  |
| 0028164 | (AT)_6_ | F: TCTTTGGTCCCACATCAACA  R:GTGTCTGTCGTCACTGCGTC | 54.0 | 222 |  |
| 0028723 | (AGAA)_6_ | F: CCAACAAGGGAAAAGCAAAG  R:AATATGGGGGTGACTCCGTT | 54.0 | 277 |  |
| 0061334 | (AAG)_8_ | F: CCGCTCTCTTTCCCTCTCTT  R: TCGTCGGAGCTAGTGAATCC | 57.0 | 279 |  |
| 0029910 | (AT)9(AG)_10_ | F:TGGGAAAAAGCGAAATGAAC  R: GCGTGGGTTGTAATTGCTTC | 52.0 | 275 |  |
| 0053597 | (CCGTAC)_4_ | F: ACCTAAAAGCCTCCAGCGAT  R: AGGAGGAGGAGGATAGGGGT | 55.0 | 118 |  |
| 0101510 | (GCT)_8_ | F: GTTGCCAGAGATCGAAAAGC  R: TGCAATATCCATTACCTCGGA | 53.0 | 210 |  |
| 0101643 | (CTG)_8_ | F: ACCCTCTCAATCGTCACCTG  R:TCAATCAATTCcCCTTCAGC | 53.0 | 178 |  |
| 0109275 | (GC)_6_(AC)_6_ | F: TGCATGCTCAAAGTCACCTC  R: CATTGCTTCAAGTTGTGTTTGTT | 52.5 | 113 |  |
| 0109549 | (CCTCTG)_4_ | F: GAGACGAAGAGCTGGGATTG  R: GTGTGGGTAATGGGTATGGC | 57.0 | 143 |  |
| 0088740 | (CGGTGG)_4_ | F: ACGGTGTAGATTCGGTCCAG  R:TCTCCAATCCCCCTCTCTTT | 55.0 | 149 |  |
| 0095066 | (TG)_8_ | F: GTATCCAACGGTCCAACCAC  R: GCAGCAATAGGAATTTGACACC | 55.0 | 240 |  |
| 0005439 | (AAT)_14_ | F: TGGAGAGATTTGGTTACGGTC  R: GCATCCTAGTCTTCCGCTTG | 53.5 | 246 |  |
| 0006074 | (GGTGCT)_5_ | F: GCAATACTTGGGCTGGGTAA  R: GTGAGACACCCAACCTCGAT | 56.0 | 265 |  |
| 0096643 | (GAAGCA) _4_ | F: CCCATTTCTCTTCTCCCCTC  R: GGGAAGTGAGCGTTGGATTA | 56.0 | 188 |  |
| 0082043 | (TTAA)_6_ | F: GCTCGCAACAACAAACAAAA  R: GGGCTGAGAATTGAGAACCA | 51.0 | 126 |  |
| 0026671 | (TTA)_8_ | F: ATGATATGCCGTTGGGAAAA  R: GCTACCCTCGAACCTCTCCT | 50.5 | 279 |  |
| 0004036 | (AGGTGA)_5_ | F: AGCAAGAAGGAGAGCGTGAG  R: TTTACACAAAAGCCCATCCC | 52.5 | 185 |  |
| 0070231 | (ACCG)_6_ | F: TACTTTCTCCCCGTTTCCCT  R: GCTCCCCATCTCCAGAAATA | 53.5 | 206 |  |
| 0081828 | (CGG)_8_ | F: TGACCCTCCACTTCATCTCC  R: GACTCCTTCGAGTGGTACGC | 57.0 | 271 |  |
