# Supplementary material for: Genetic diversity and population structure of Polygonatum cyrtonema Hua in China using SSR markers
Source: PLoS One. 2023 Aug 31;18(8):e0290605. doi: 10.1371/journal.pone.0290605 (PMC10470896; doi:10.1371/journal.pone.0290605)
Supplement: S1 Data — (ZIP) [file pone.0290605.s002.zip › ╨┬╜¿╬─╝■╝╨ (2)/╥2╬∩18/yimwu18.pdf]

Allele Report

Sample 1: Run date and time: 09/16/2020 - 14:20:02 -> 09/16/2020 - 15:18:49

Dye: Blue - 0 peaks - 1.fsa

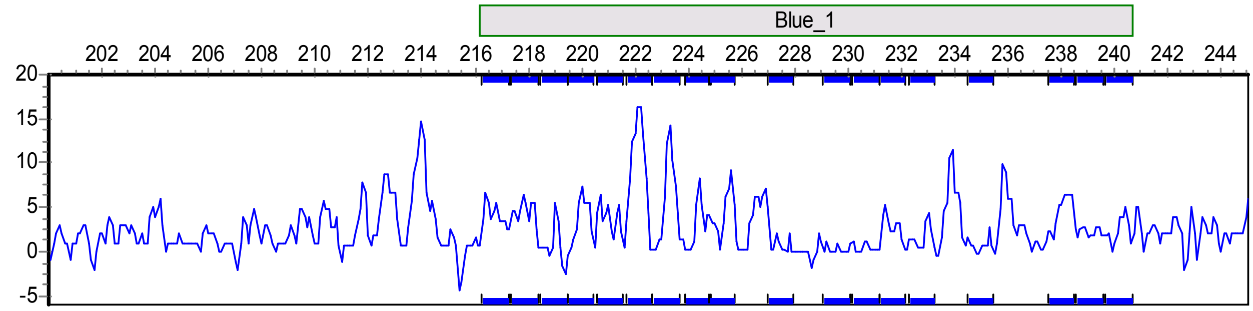

No	Size	Height	Area	Marker	Allele	Difference	Quality	Score	Allele Comments	Sample Comments

Sample 2: Run date and time: 09/16/2020 - 14:20:02 -> 09/16/2020 - 15:18:49

Dye: Blue - 2 peaks - 10.fsa

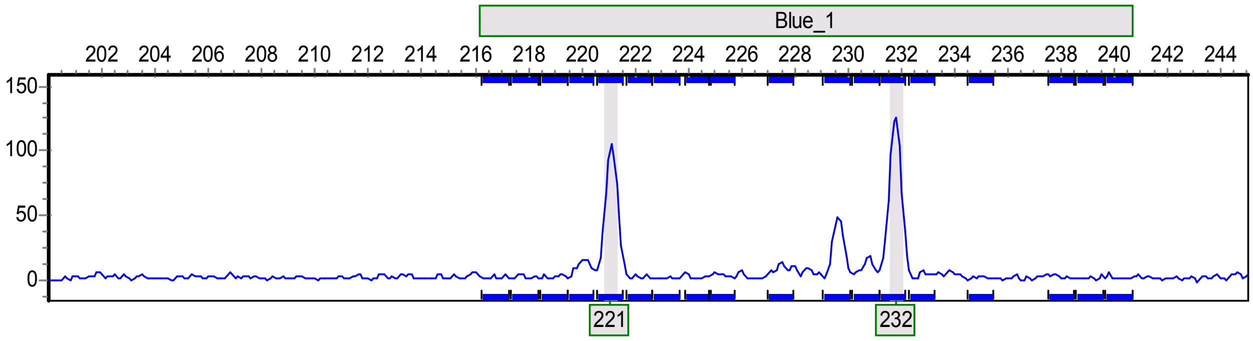

No	Size	Height	Area	Marker	Allele	Difference	Quality	Score	Allele Comments	Sample Comments
1	221.1	105	547	Blue_1	221	0.0	Pass	4.8	[<Confirmed>]	
2	231.8	126	645	Blue_1	232	0.1	Pass	6.6	[<Confirmed>]	

Sample 3: Run date and time: 09/16/2020 - 14:20:02 -> 09/16/2020 - 15:18:49

Dye: Blue - 0 peaks - 11.fsa

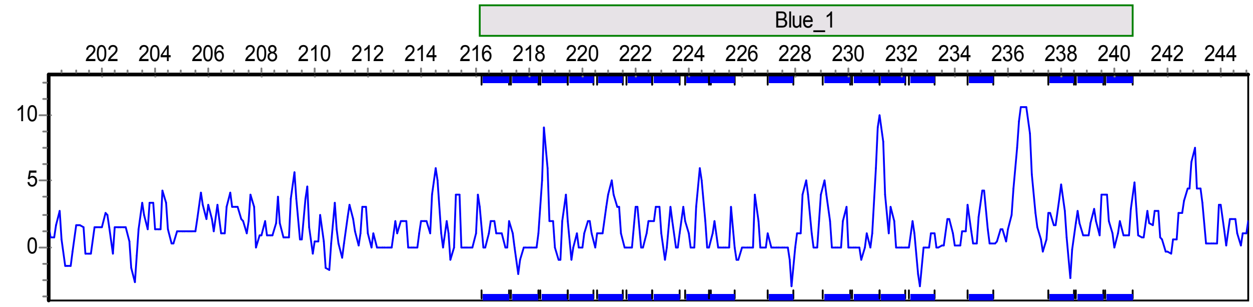

No	Size	Height	Area	Marker	Allele	Difference	Quality	Score	Allele Comments	Sample Comments

Allele Report

Sample 4: Run date and time: 09/16/2020 - 14:20:02 -> 09/16/2020 - 15:18:49

Dye: Blue - 0 peaks - 12.fsa

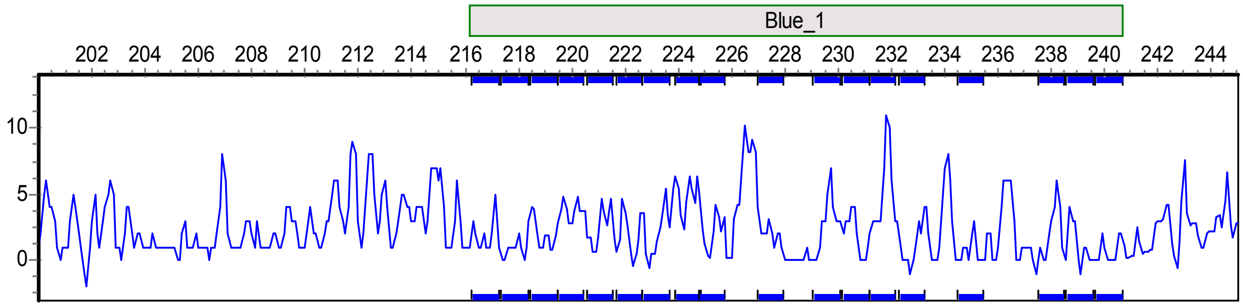

No	Size	Height	Area	Marker	Allele	Difference	Quality	Score	Allele Comments	Sample Comments

Sample 5: Run date and time: 09/16/2020 - 14:20:02 -> 09/16/2020 - 15:18:49

Dye: Blue - 0 peaks - 13.fsa

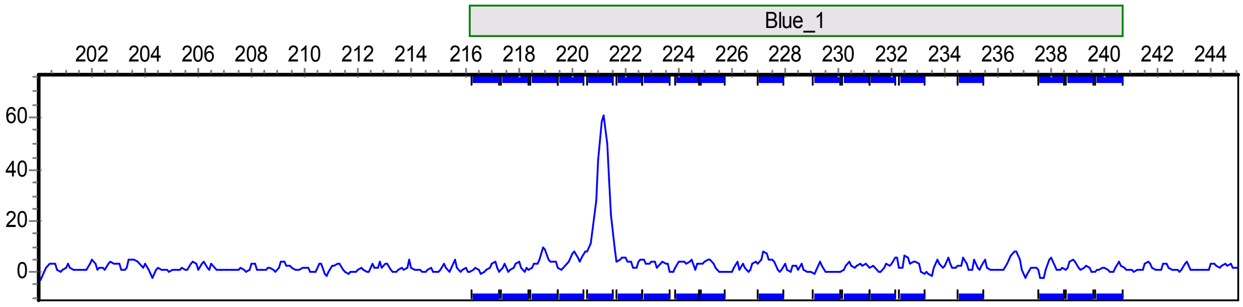

No	Size	Height	Area	Marker	Allele	Difference	Quality	Score	Allele Comments	Sample Comments

Sample 6: Run date and time: 09/16/2020 - 14:20:02 -> 09/16/2020 - 15:18:49

Dye: Blue - 0 peaks - 14.fsa

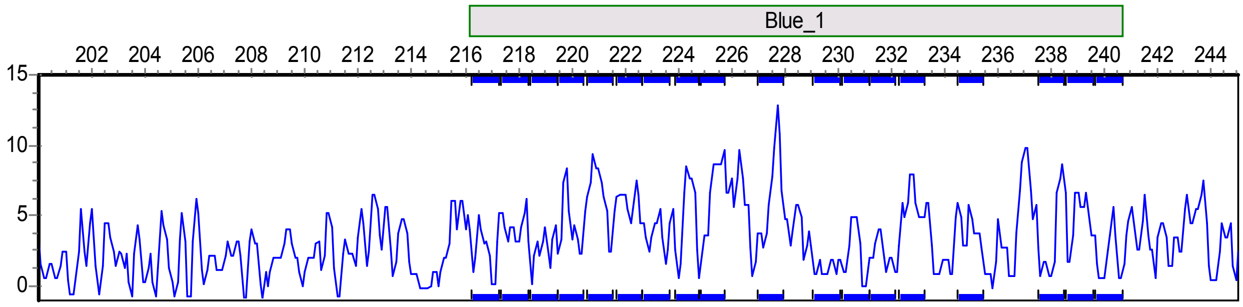

No	Size	Height	Area	Marker	Allele	Difference	Quality	Score	Allele Comments	Sample Comments

Allele Report

Sample 7: Run date and time: 09/16/2020 - 14:20:02 -> 09/16/2020 - 15:18:49

Dye: Blue - 2 peaks - 15.fsa

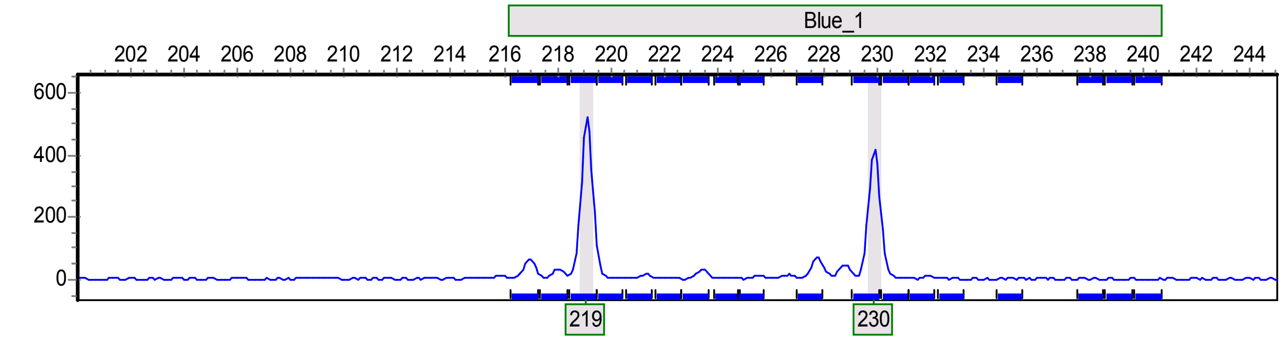

No	Size	Height	Area	Marker	Allele	Difference	Quality	Score	Allele Comments	Sample Comments
1	219.1	519	2582	Blue_1	219	0.1	Pass	78.2		
2	229.9	419	2157	Blue_1	230	0.3	Pass	52.6		

Sample 8: Run date and time: 09/16/2020 - 14:20:02 -> 09/16/2020 - 15:18:49

Dye: Blue - 2 peaks - 16.fsa

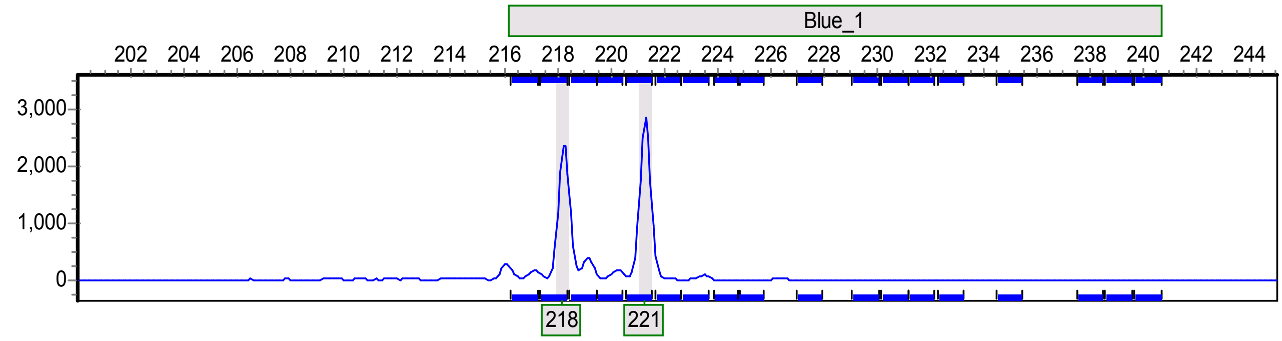

No	Size	Height	Area	Marker	Allele	Difference	Quality	Score	Allele Comments	Sample Comments
1	218.2	2371	11752	Blue_1	218	0.3	Pass	500.0		
2	221.3	2852	13449	Blue_1	221	0.2	Pass	500.0		

Sample 9: Run date and time: 09/16/2020 - 14:20:02 -> 09/16/2020 - 15:18:49

Dye: Blue - 2 peaks - 17.fsa

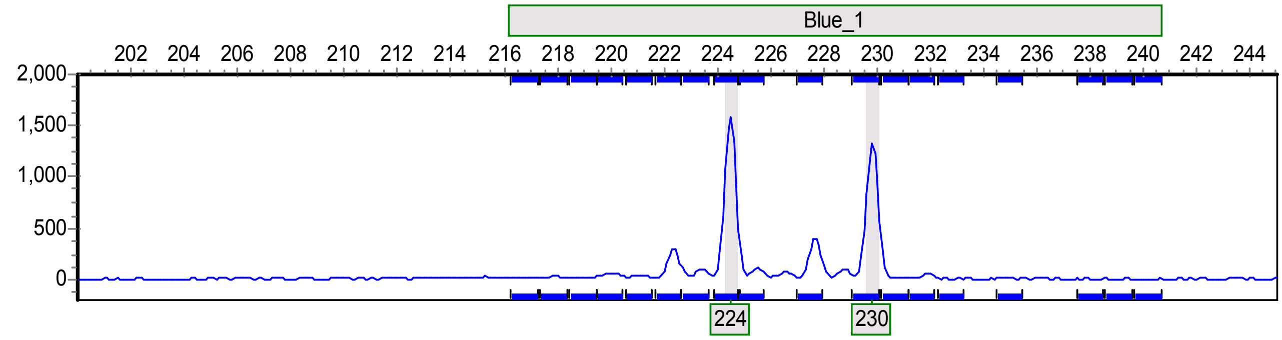

No	Size	Height	Area	Marker	Allele	Difference	Quality	Score	Allele Comments	Sample Comments
1	224.5	1574	7562	Blue_1	224	0.1	Pass	332.7		
2	229.8	1333	6732	Blue_1	230	0.2	Pass	245.2		

Allele Report

Sample 10: Run date and time: 09/16/2020 - 14:20:02 -> 09/16/2020 - 15:18:49

Dye: Blue - 0 peaks - 18.fsa

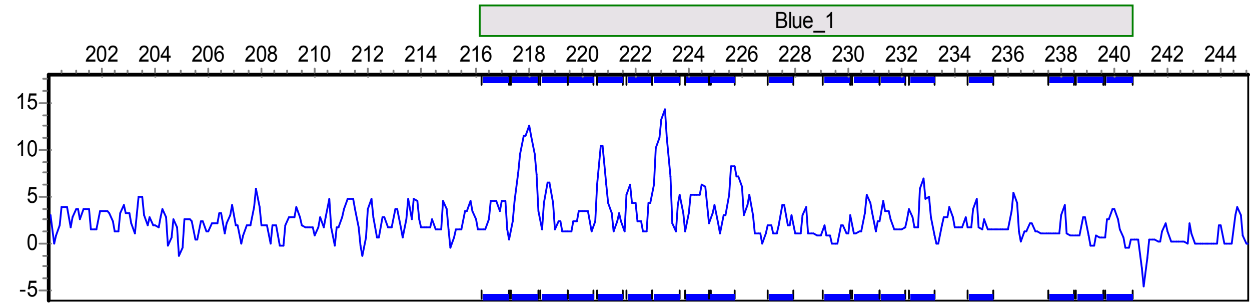

No	Size	Height	Area	Marker	Allele	Difference	Quality	Score	Allele Comments	Sample Comments

Sample 11: Run date and time: 09/16/2020 - 14:20:02 -> 09/16/2020 - 15:18:49

Dye: Blue - 0 peaks - 19.fsa

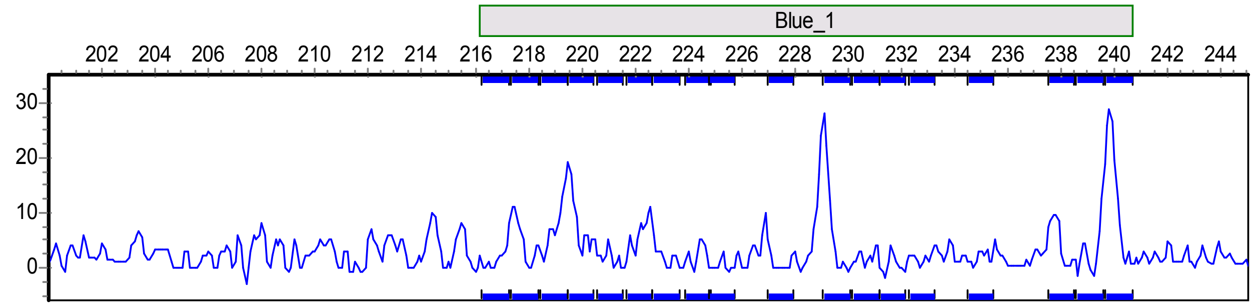

No	Size	Height	Area	Marker	Allele	Difference	Quality	Score	Allele Comments	Sample Comments

Sample 12: Run date and time: 09/16/2020 - 14:20:02 -> 09/16/2020 - 15:18:49

Dye: Blue - 2 peaks - 2.fsa

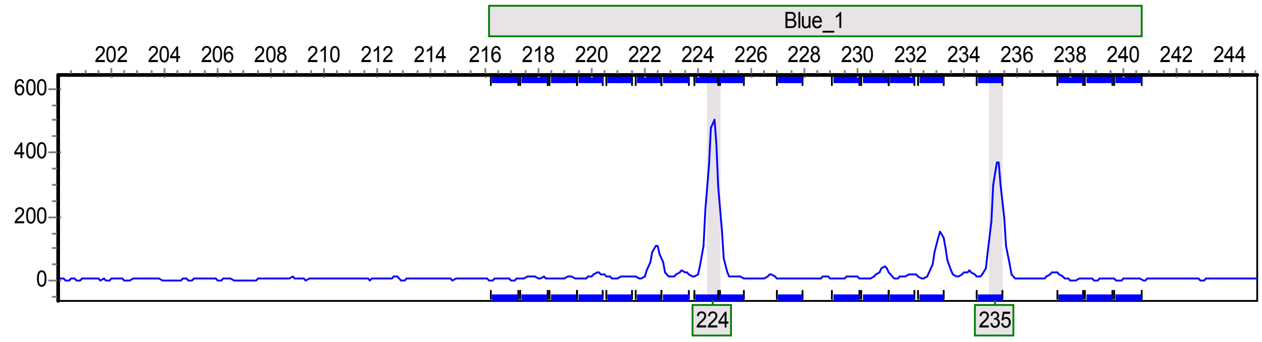

No	Size	Height	Area	Marker	Allele	Difference	Quality	Score	Allele Comments	Sample Comments
1	224.6	508	2523	Blue_1	224	0.2	Pass	75.7		
2	235.2	372	1887	Blue_1	235	0.2	Pass	44.9		

Allele Report

Sample 13: Run date and time: 09/16/2020 - 14:20:02 -> 09/16/2020 - 15:18:49

Dye: Blue - 0 peaks - 20.fsa

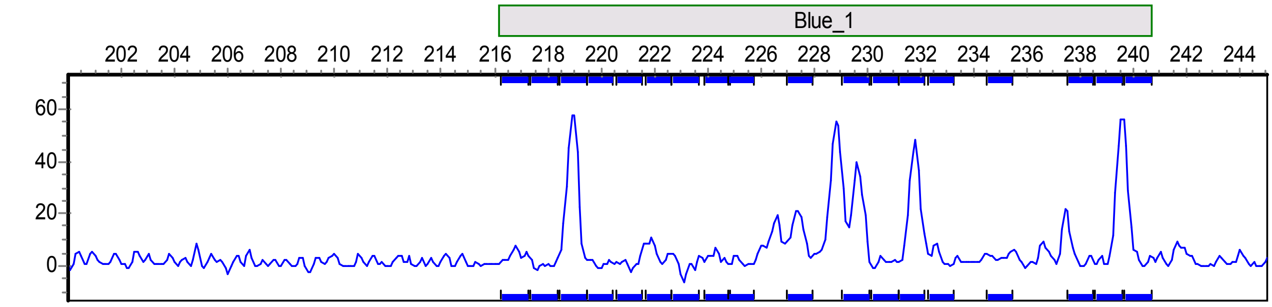

No	Size	Height	Area	Marker	Allele	Difference	Quality	Score	Allele Comments	Sample Comments

Sample 14: Run date and time: 09/16/2020 - 14:20:02 -> 09/16/2020 - 15:18:49

Dye: Blue - 0 peaks - 21.fsa

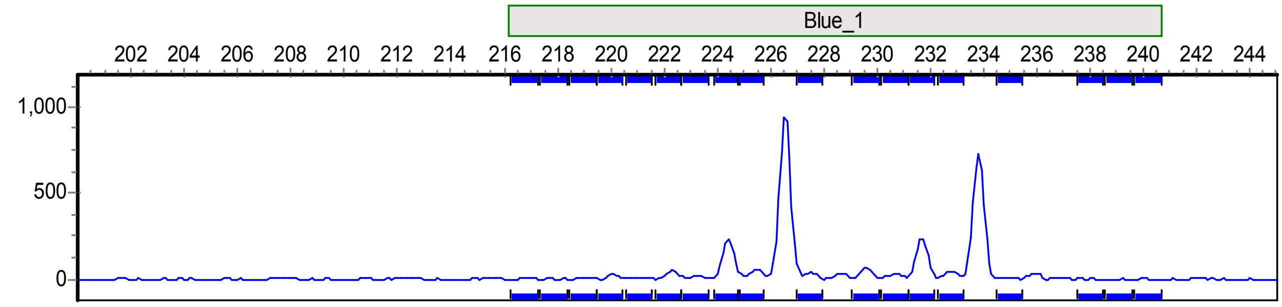

No	Size	Height	Area	Marker	Allele	Difference	Quality	Score	Allele Comments	Sample Comments

Sample 15: Run date and time: 09/16/2020 - 14:20:02 -> 09/16/2020 - 15:18:49

Dye: Blue - 2 peaks - 22.fsa

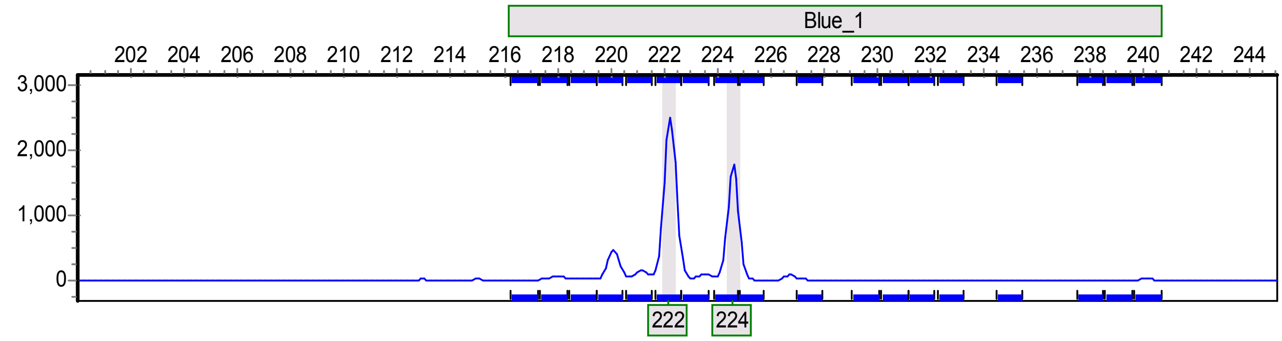

No	Size	Height	Area	Marker	Allele	Difference	Quality	Score	Allele Comments	Sample Comments
1	222.2	2486	13059	Blue_1	222	0.0	Pass	500.0		
2	224.6	1780	8503	Blue_1	224	0.2	Pass	500.0		

Sample 16: Run date and time: 09/16/2020 - 14:20:02 -> 09/16/2020 - 15:18:49

Dye: Blue - 1 peaks - 23.fsa

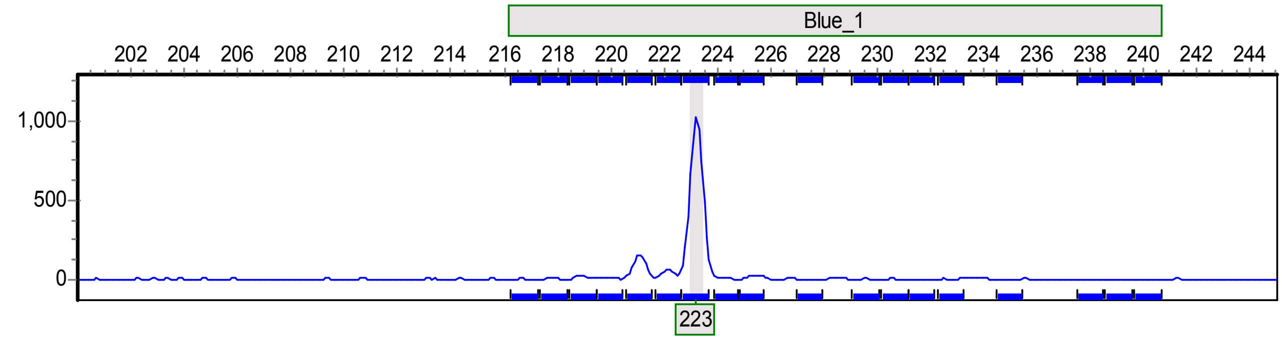

No	Size	Height	Area	Marker	Allele	Difference	Quality	Score	Allele Comments	Sample Comments
1	223.2	1014	5492	Blue_1	223	0.0	Pass	190.1		

Sample 17: Run date and time: 09/16/2020 - 14:20:02 -> 09/16/2020 - 15:18:49

Dye: Blue - 0 peaks - 24.fsa

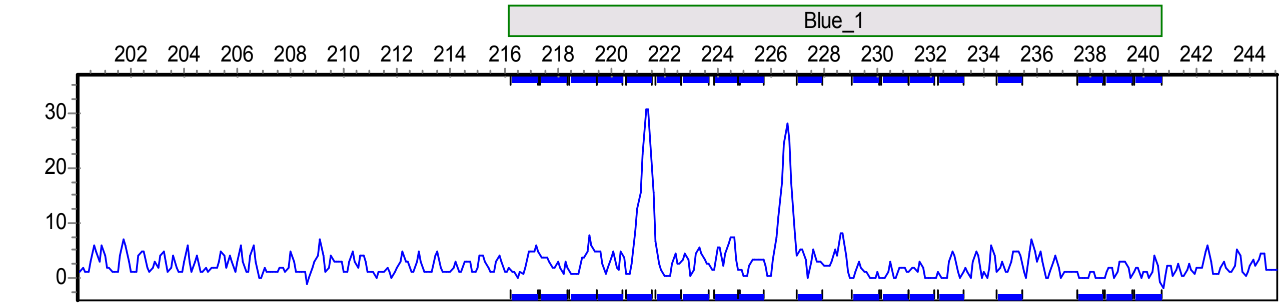

No	Size	Height	Area	Marker	Allele	Difference	Quality	Score	Allele Comments	Sample Comments

Sample 18: Run date and time: 09/16/2020 - 14:20:02 -> 09/16/2020 - 15:18:49

Dye: Blue - 0 peaks - 25.fsa

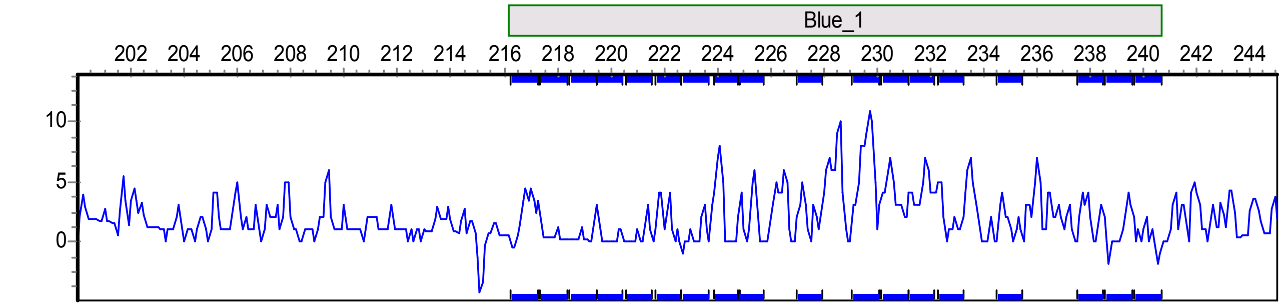

No	Size	Height	Area	Marker	Allele	Difference	Quality	Score	Allele Comments	Sample Comments

Sample 19: Run date and time: 09/16/2020 - 14:20:02 -> 09/16/2020 - 15:18:49

Dye: Blue - 0 peaks - 26.fsa

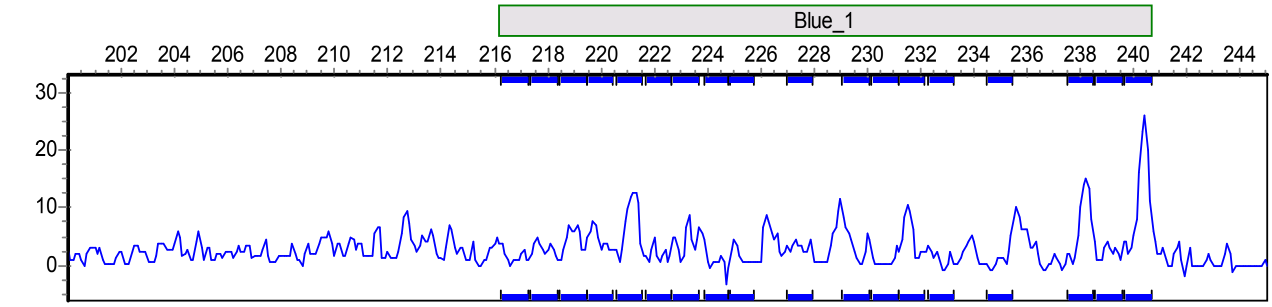

No	Size	Height	Area	Marker	Allele	Difference	Quality	Score	Allele Comments	Sample Comments

Sample 20: Run date and time: 09/16/2020 - 14:20:02 -> 09/16/2020 - 15:18:49

Dye: Blue - 2 peaks - 27.fsa

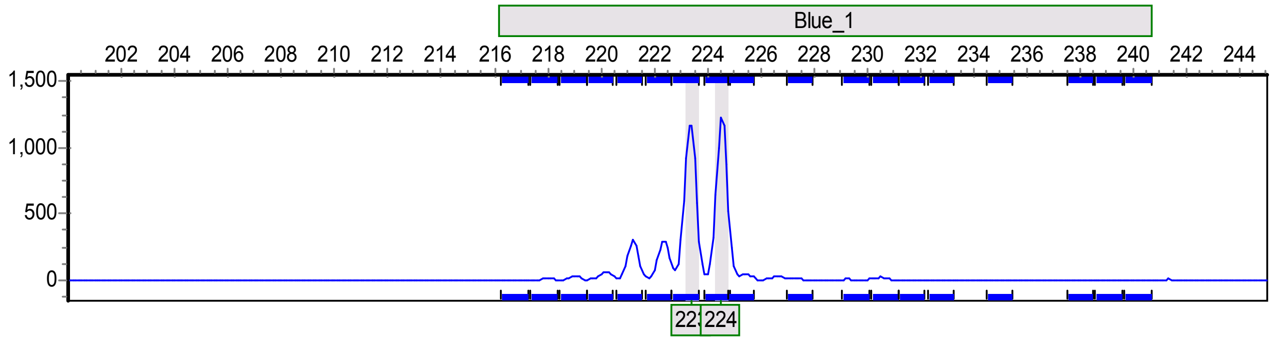

No	Size	Height	Area	Marker	Allele	Difference	Quality	Score	Allele Comments	Sample Comments
1	223.4	1170	5839	Blue_1	223	0.2	Pass	264.3		
2	224.5	1221	5824	Blue_1	224	0.1	Pass	298.6		

Sample 21: Run date and time: 09/16/2020 - 14:20:02 -> 09/16/2020 - 15:18:49

Dye: Blue - 1 peaks - 28.fsa

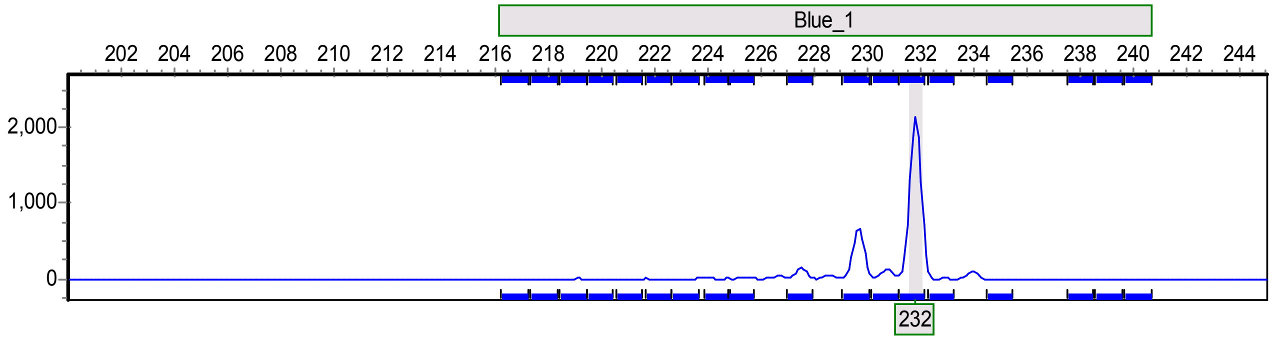

No	Size	Height	Area	Marker	Allele	Difference	Quality	Score	Allele Comments	Sample Comments
1	231.8	2113	9968	Blue_1	232	0.1	Pass	500.0		

Sample 22: Run date and time: 09/16/2020 - 14:20:02 -> 09/16/2020 - 15:18:49

Dye: Blue - 0 peaks - 29.fsa

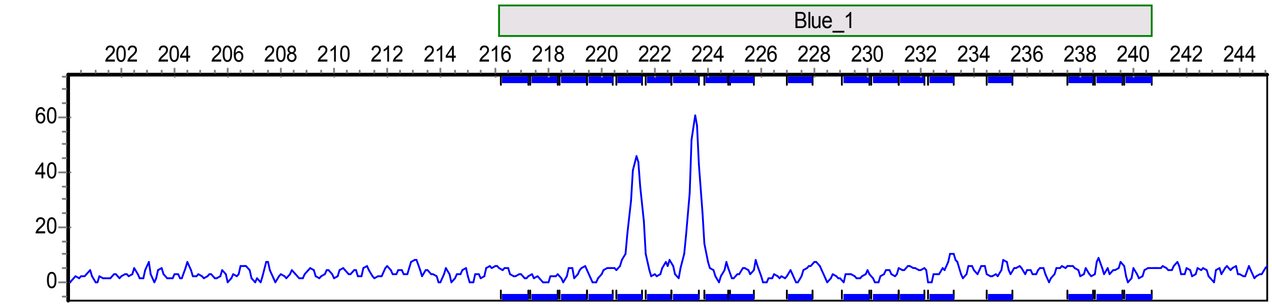

No	Size	Height	Area	Marker	Allele	Difference	Quality	Score	Allele Comments	Sample Comments

Sample 23: Run date and time: 09/16/2020 - 14:20:02 -> 09/16/2020 - 15:18:49

Dye: Blue - 2 peaks - 3.fsa

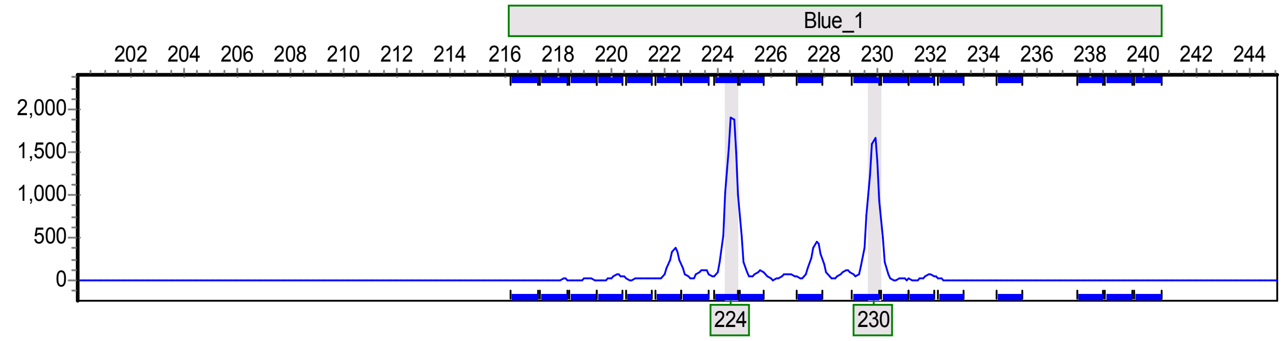

No	Size	Height	Area	Marker	Allele	Difference	Quality	Score	Allele Comments	Sample Comments
1	224.5	1903	9854	Blue_1	224	0.1	Pass	499.0		
2	229.9	1669	8410	Blue_1	230	0.3	Pass	430.3		

Sample 24: Run date and time: 09/16/2020 - 14:20:02 -> 09/16/2020 - 15:18:49

Dye: Blue - 2 peaks - 30.fsa

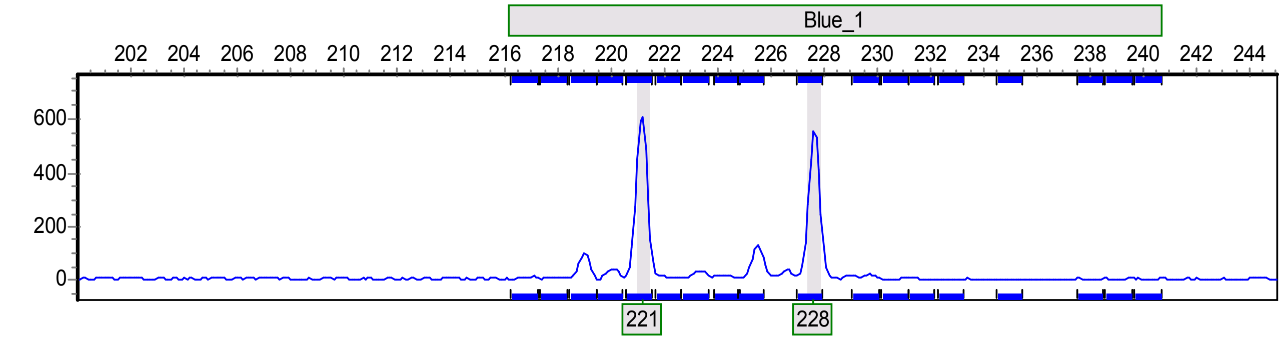

No	Size	Height	Area	Marker	Allele	Difference	Quality	Score	Allele Comments	Sample Comments
1	221.2	608	2892	Blue_1	221	0.1	Pass	106.4		
2	227.6	554	2633	Blue_1	228	0.1	Pass	93.1		

Sample 25: Run date and time: 09/16/2020 - 14:20:02 -> 09/16/2020 - 15:18:49

Dye: Blue - 0 peaks - 31.fsa

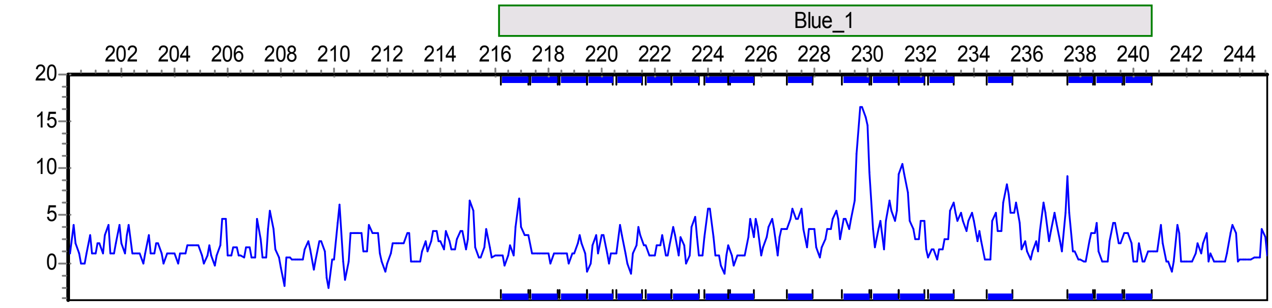

No	Size	Height	Area	Marker	Allele	Difference	Quality	Score	Allele Comments	Sample Comments

Sample 26: Run date and time: 09/16/2020 - 14:20:02 -> 09/16/2020 - 15:18:49

Dye: Blue - 0 peaks - 32.fsa

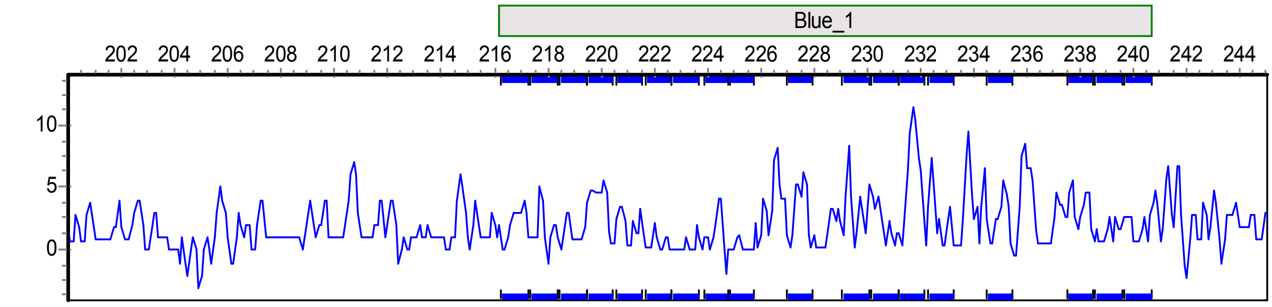

No	Size	Height	Area	Marker	Allele	Difference	Quality	Score	Allele Comments	Sample Comments

Sample 27: Run date and time: 09/16/2020 - 14:20:02 -> 09/16/2020 - 15:18:49

Dye: Blue - 1 peaks - 33.fsa

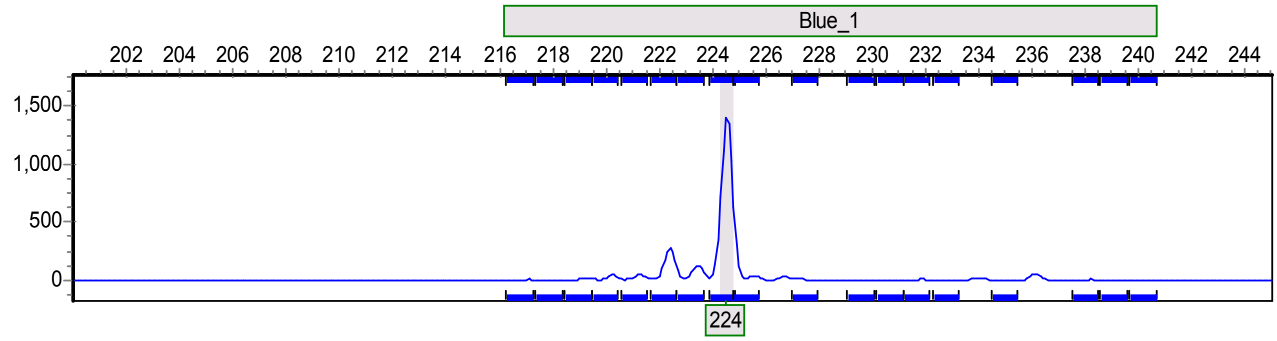

No	Size	Height	Area	Marker	Allele	Difference	Quality	Score	Allele Comments	Sample Comments
1	224.5	1387	6711	Blue_1	224	0.1	Pass	352.5		

Sample 28: Run date and time: 09/16/2020 - 14:20:02 -> 09/16/2020 - 15:18:49

Dye: Blue - 2 peaks - 34.fsa

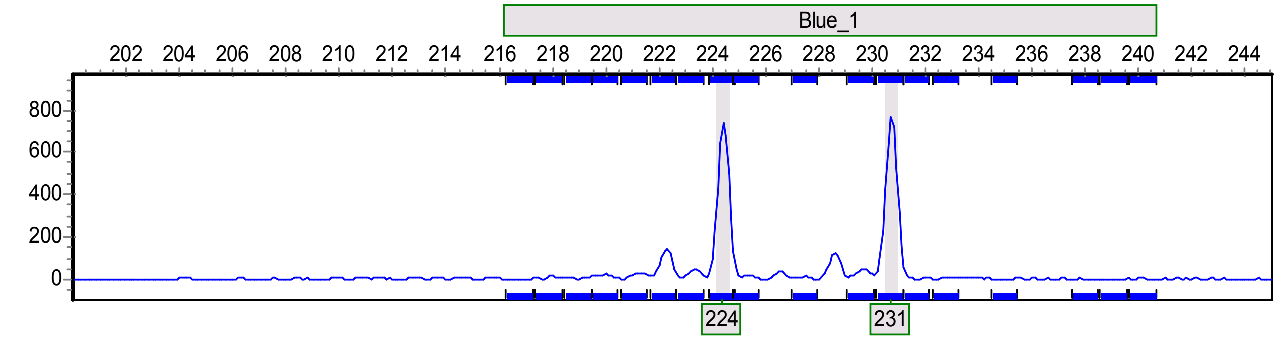

No	Size	Height	Area	Marker	Allele	Difference	Quality	Score	Allele Comments	Sample Comments
1	224.4	739	3548	Blue_1	224	0.0	Pass	142.4		
2	230.7	763	3691	Blue_1	231	0.0	Pass	146.8		

Sample 29: Run date and time: 09/16/2020 - 14:20:02 -> 09/16/2020 - 15:18:49

Dye: Blue - 1 peaks - 35.fsa

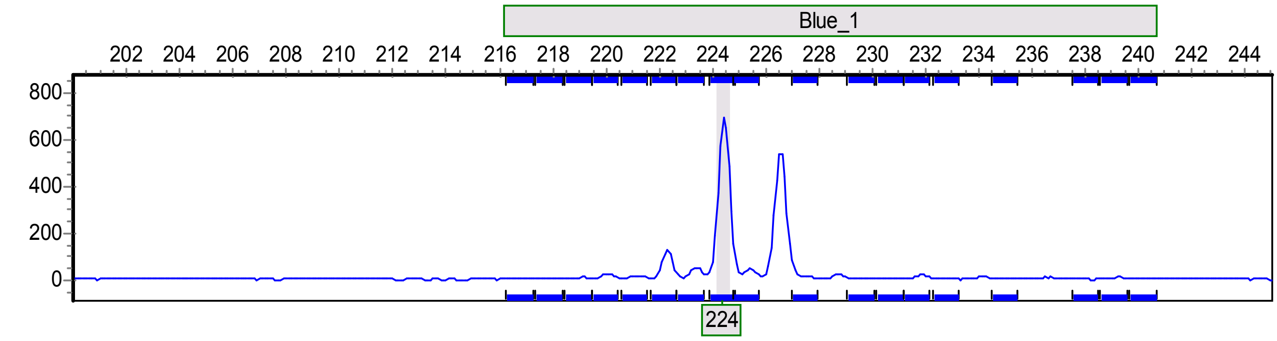

No	Size	Height	Area	Marker	Allele	Difference	Quality	Score	Allele Comments	Sample Comments
1	224.4	692	3332	Blue_1	224	0.0	Pass	127.7		

Sample 30: Run date and time: 09/16/2020 - 14:20:02 -> 09/16/2020 - 15:18:49

Dye: Blue - 2 peaks - 36.fsa

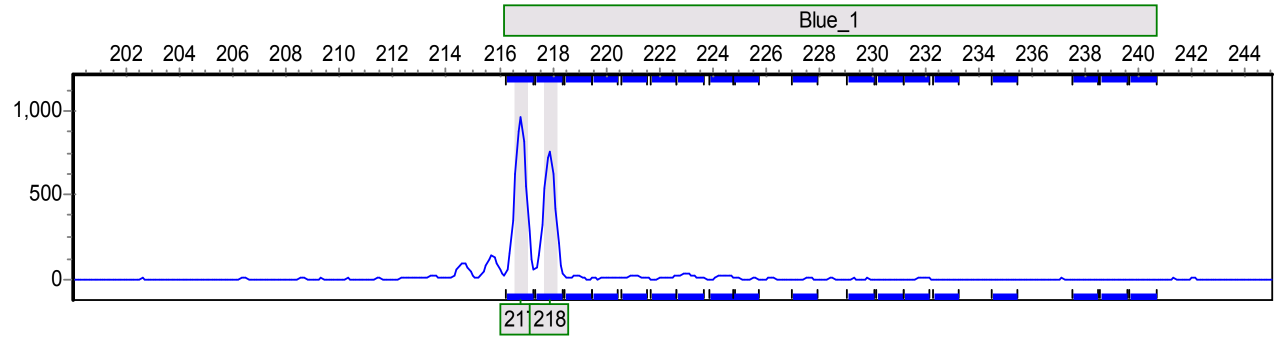

No	Size	Height	Area	Marker	Allele	Difference	Quality	Score	Allele Comments	Sample Comments
1	216.8	955	4487	Blue_1	217	0.0	Pass	213.4		
2	217.9	751	3648	Blue_1	218	0.0	Pass	142.8		

Sample 31: Run date and time: 09/16/2020 - 14:20:02 -> 09/16/2020 - 15:18:49

Dye: Blue - 2 peaks - 37.fsa

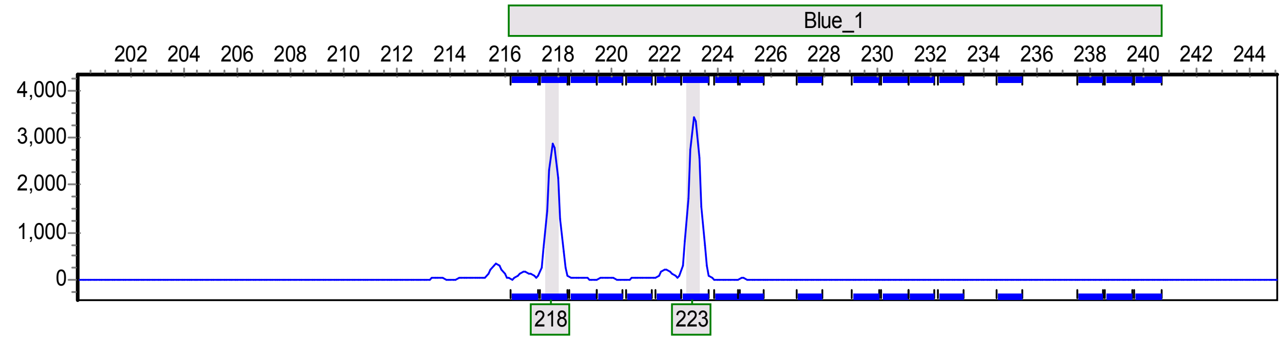

No	Size	Height	Area	Marker	Allele	Difference	Quality	Score	Allele Comments	Sample Comments
1	217.8	2872	13746	Blue_1	218	0.1	Pass	500.0		
2	223.1	3418	16382	Blue_1	223	0.1	Pass	500.0		

Sample 32: Run date and time: 09/16/2020 - 14:20:02 -> 09/16/2020 - 15:18:49

Dye: Blue - 1 peaks - 38.fsa

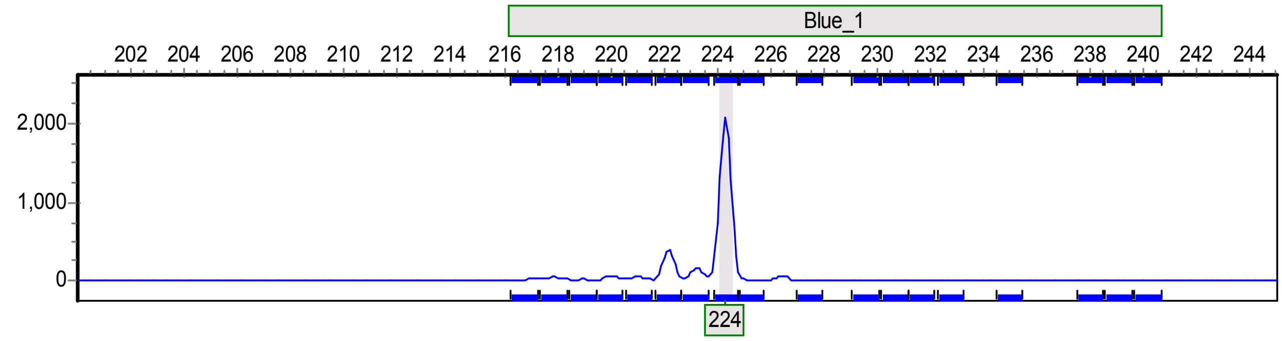

No	Size	Height	Area	Marker	Allele	Difference	Quality	Score	Allele Comments	Sample Comments
1	224.3	2068	9921	Blue_1	224	0.1	Pass	500.0		

Sample 33: Run date and time: 09/16/2020 - 14:20:02 -> 09/16/2020 - 15:18:49

Dye: Blue - 2 peaks - 39.fsa

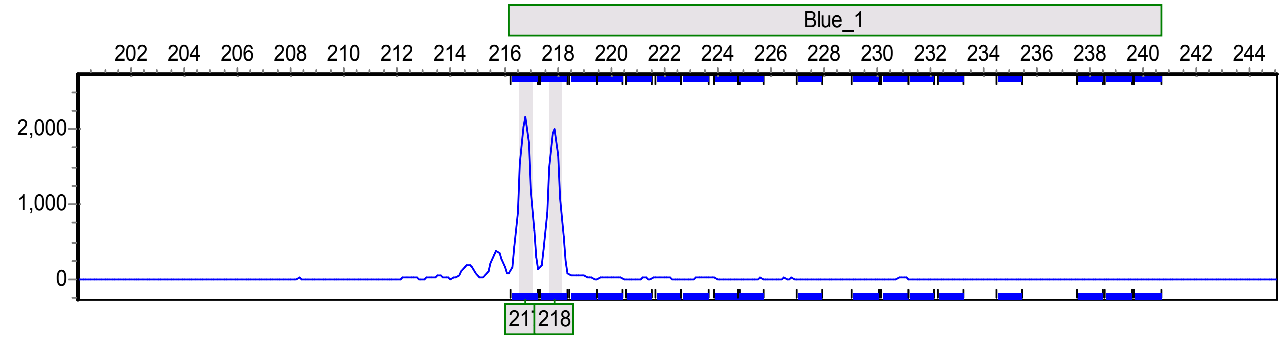

No	Size	Height	Area	Marker	Allele	Difference	Quality	Score	Allele Comments	Sample Comments
1	216.8	2162	10547	Blue_1	217	0.0	Pass	500.0		
2	217.9	2022	9867	Blue_1	218	0.0	Pass	500.0		

Allele Report

Sample 34: Run date and time: 09/16/2020 - 14:20:02 -> 09/16/2020 - 15:18:49

Dye: Blue - 2 peaks - 4.fsa

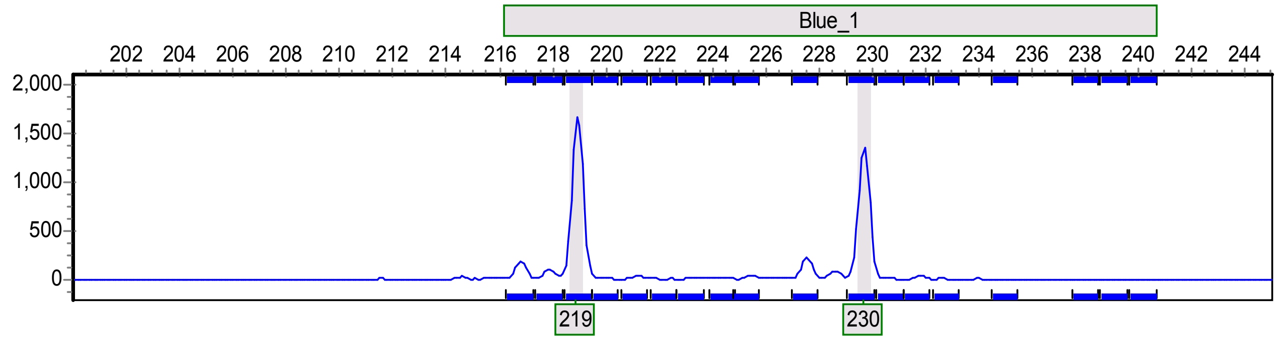

No	Size	Height	Area	Marker	Allele	Difference	Quality	Score	Allele Comments	Sample Comments
1	218.9	1652	7784	Blue_1	219	0.1	Pass	464.3		
2	229.7	1358	6526	Blue_1	230	0.1	Pass	342.9		

Sample 35: Run date and time: 09/16/2020 - 14:20:02 -> 09/16/2020 - 15:18:49

Dye: Blue - 2 peaks - 40.fsa

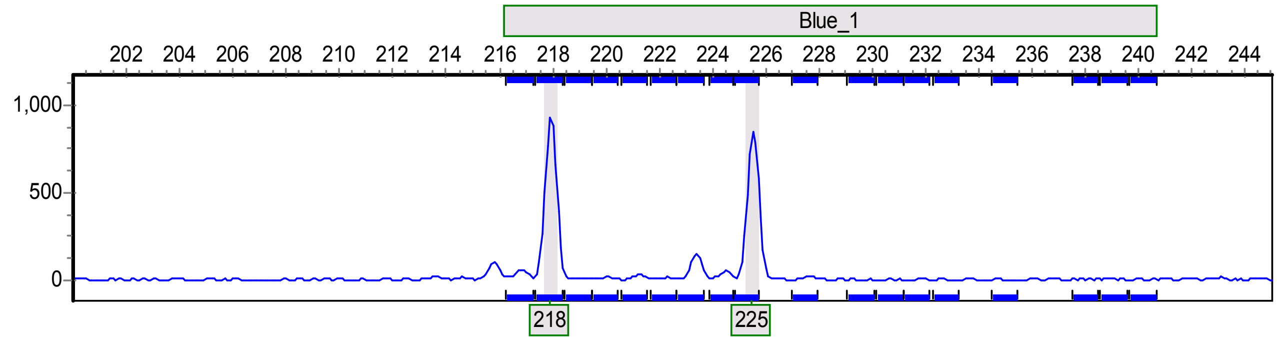

No	Size	Height	Area	Marker	Allele	Difference	Quality	Score	Allele Comments	Sample Comments
1	217.9	920	4424	Blue_1	218	0.0	Pass	195.3		
2	225.5	842	4088	Blue_1	225	0.2	Pass	169.5		

Sample 36: Run date and time: 09/16/2020 - 14:20:02 -> 09/16/2020 - 15:18:49

Dye: Blue - 1 peaks - 41.fsa

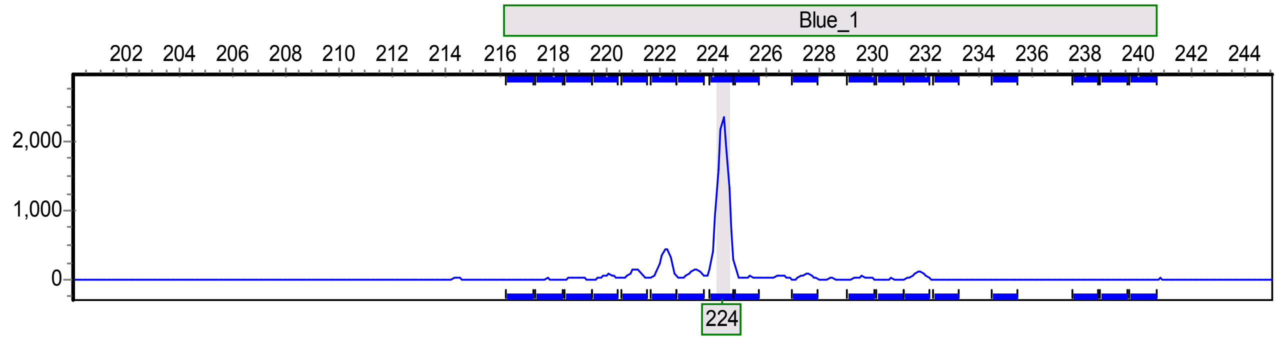

No	Size	Height	Area	Marker	Allele	Difference	Quality	Score	Allele Comments	Sample Comments
1	224.4	2329	11114	Blue_1	224	0.0	Pass	500.0		

Sample 37: Run date and time: 09/16/2020 - 14:20:02 -> 09/16/2020 - 15:18:49

Dye: Blue - 2 peaks - 42.fsa

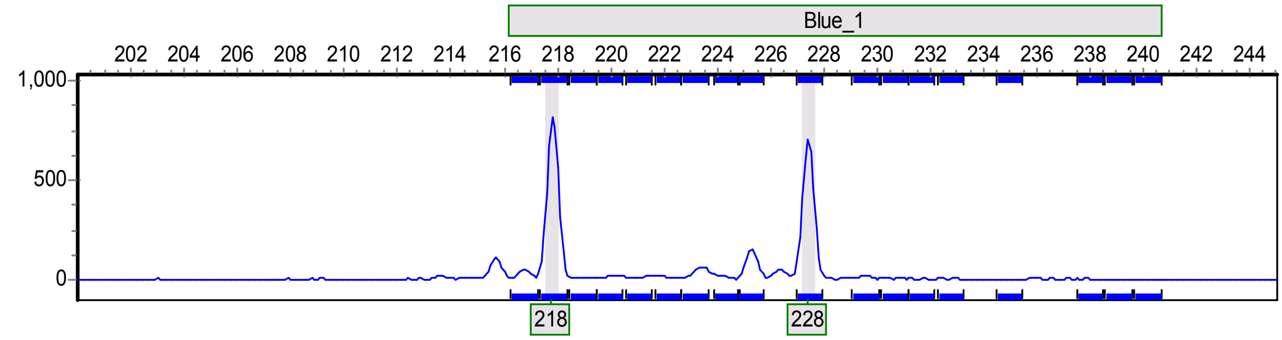

No	Size	Height	Area	Marker	Allele	Difference	Quality	Score	Allele Comments	Sample Comments
1	217.8	811	3824	Blue_1	218	0.1	Pass	167.4		
2	227.4	703	3334	Blue_1	228	0.1	Pass	134.0		

Sample 38: Run date and time: 09/16/2020 - 14:20:02 -> 09/16/2020 - 15:18:49

Dye: Blue - 2 peaks - 43.fsa

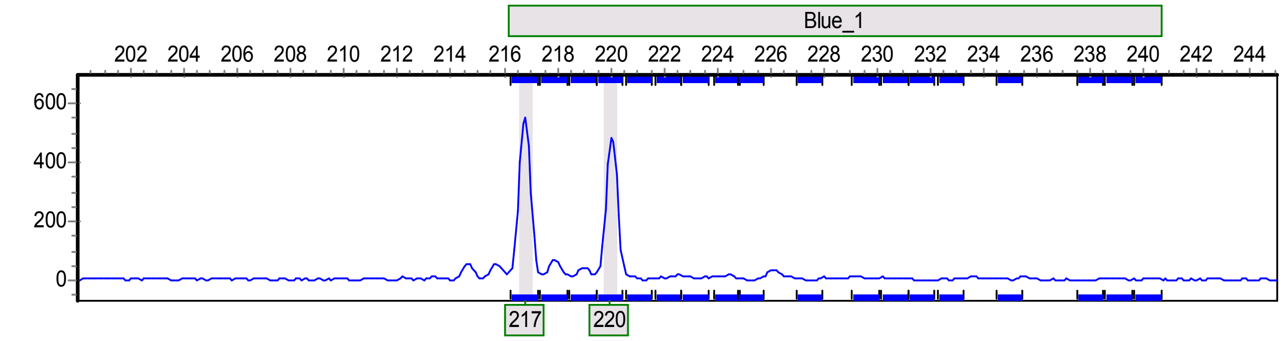

No	Size	Height	Area	Marker	Allele	Difference	Quality	Score	Allele Comments	Sample Comments
1	216.8	547	2643	Blue_1	217	0.0	Pass	88.4		
2	220.0	483	2315	Blue_1	220	0.0	Pass	73.9		

Sample 39: Run date and time: 09/16/2020 - 14:20:02 -> 09/16/2020 - 15:18:49

Dye: Blue - 2 peaks - 44.fsa

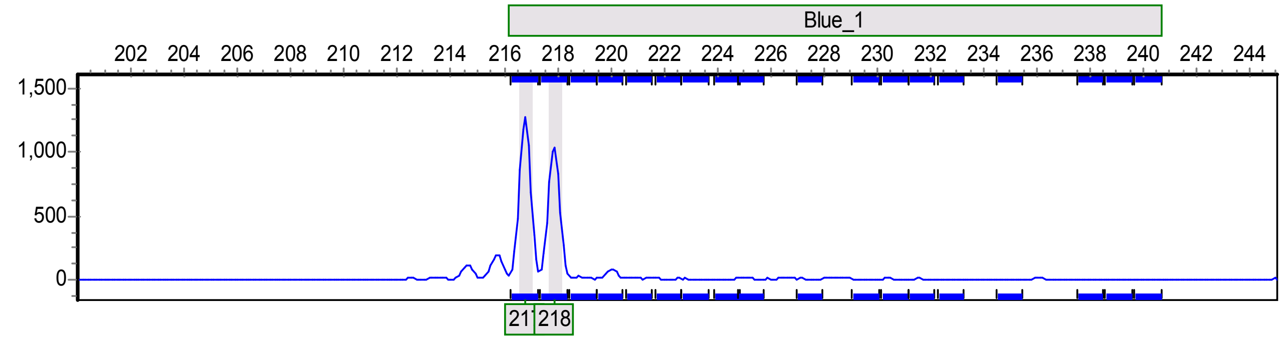

No	Size	Height	Area	Marker	Allele	Difference	Quality	Score	Allele Comments	Sample Comments
1	216.8	1266	5931	Blue_1	217	0.0	Pass	320.0		
2	217.9	1034	4939	Blue_1	218	0.0	Pass	233.3		

Sample 40: Run date and time: 09/16/2020 - 14:20:02 -> 09/16/2020 - 15:18:49

Dye: Blue - 2 peaks - 45.fsa

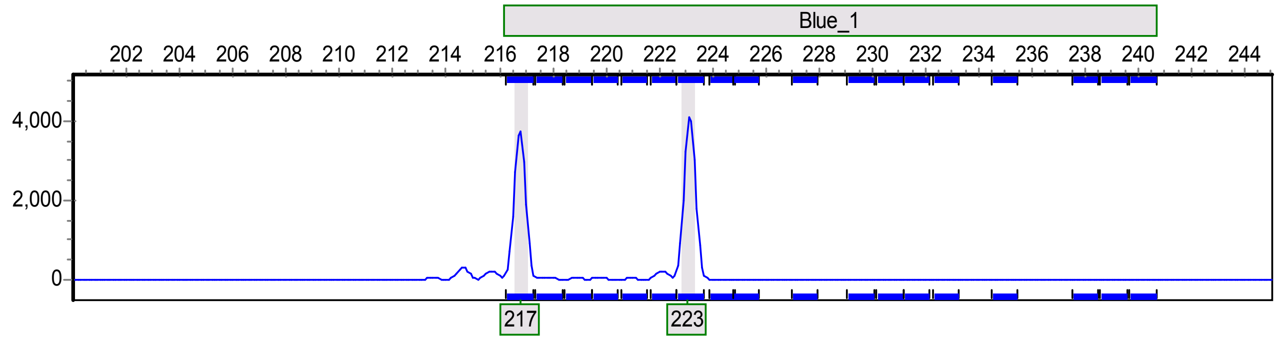

No	Size	Height	Area	Marker	Allele	Difference	Quality	Score	Allele Comments	Sample Comments
1	216.8	3736	17645	Blue_1	217	0.0	Pass	500.0		
2	223.1	4080	19332	Blue_1	223	0.1	Pass	500.0		

Sample 41: Run date and time: 09/16/2020 - 14:20:02 -> 09/16/2020 - 15:18:49

Dye: Blue - 2 peaks - 46.fsa

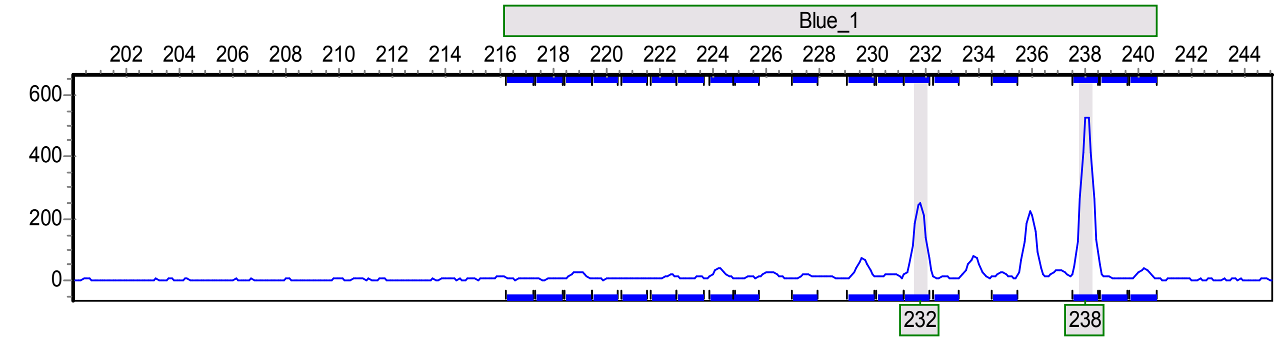

No	Size	Height	Area	Marker	Allele	Difference	Quality	Score	Allele Comments	Sample Comments
1	231.8	252	1249	Blue_1	232	0.1	Pass	24.3		
2	238.0	522	2605	Blue_1	238	0.0	Pass	79.0		

Sample 42: Run date and time: 09/16/2020 - 14:20:02 -> 09/16/2020 - 15:18:49

Dye: Blue - 2 peaks - 47.fsa

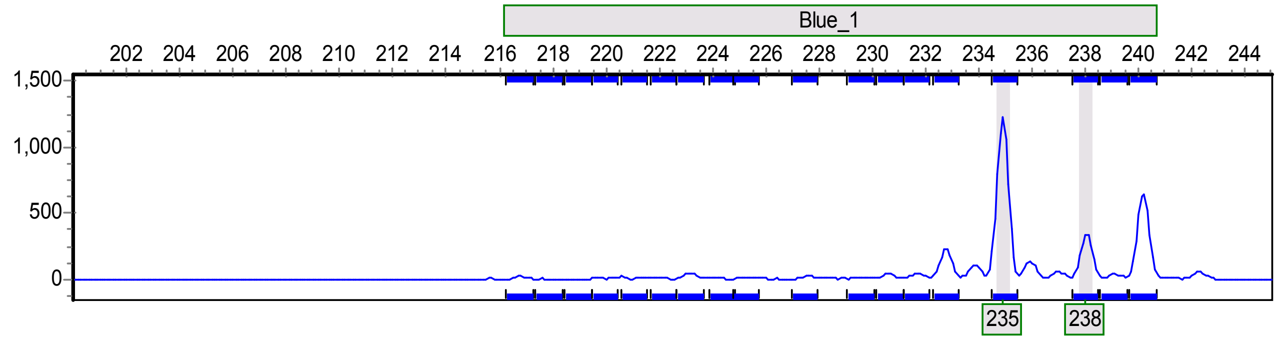

No	Size	Height	Area	Marker	Allele	Difference	Quality	Score	Allele Comments	Sample Comments
1	234.9	1220	5825	Blue_1	235	0.1	Pass	297.7		
2	238.0	337	1688	Blue_1	238	0.0	Pass	38.6		

Sample 43: Run date and time: 09/16/2020 - 14:20:02 -> 09/16/2020 - 15:18:49

Dye: Blue - 4 peaks - 48.fsa

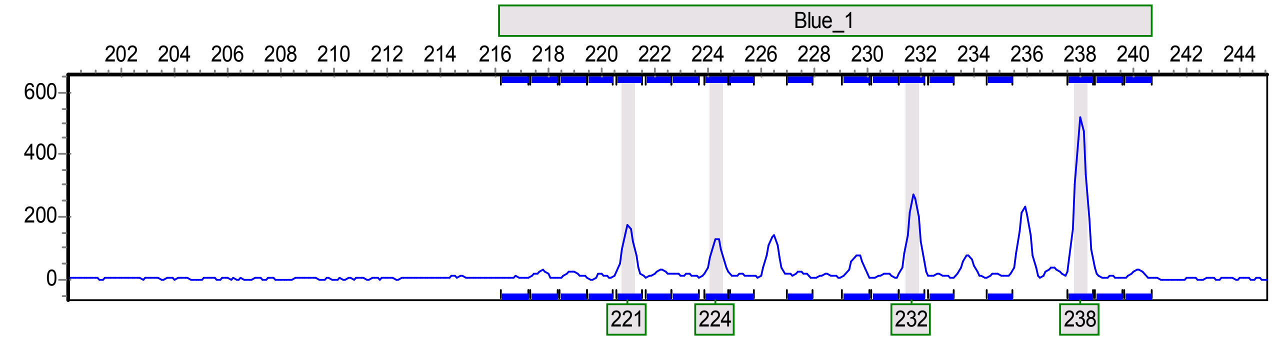

No	Size	Height	Area	Marker	Allele	Difference	Quality	Score	Allele Comments	Sample Comments
1	221.0	175	844	Blue_1	221	0.1	Pass	13.6		
2	224.3	130	654	Blue_1	224	0.1	Pass	7.5		
3	231.7	268	1316	Blue_1	232	0.0	Pass	27.2		
4	238.0	518	2465	Blue_1	238	0.0	Pass	83.8		

Sample 44: Run date and time: 09/16/2020 - 14:20:02 -> 09/16/2020 - 15:18:49

Dye: Blue - 2 peaks - 49.fsa

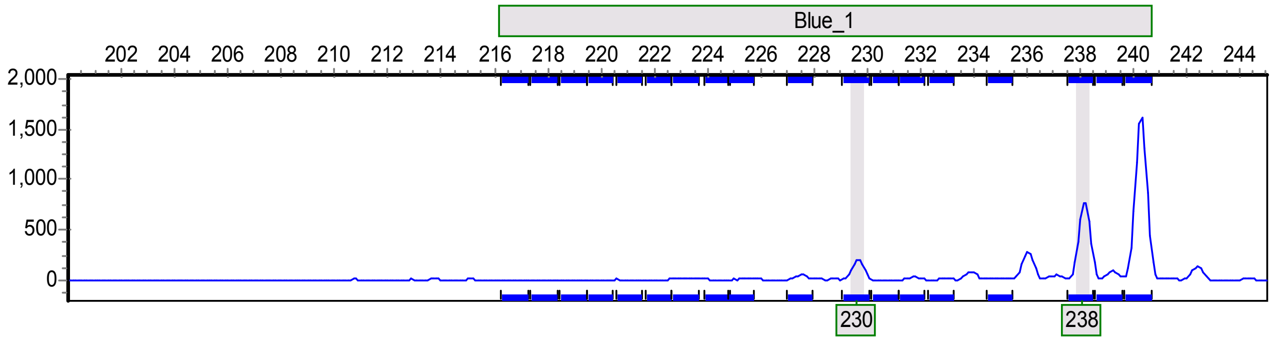

No	Size	Height	Area	Marker	Allele	Difference	Quality	Score	Allele Comments	Sample Comments
1	229.6	196	971	Blue_1	230	0.0	Pass	15.7		
2	238.1	767	3707	Blue_1	238	0.1	Pass	148.5		

Sample 45: Run date and time: 09/16/2020 - 14:20:02 -> 09/16/2020 - 15:18:49

Dye: Blue - 2 peaks - 5.fsa

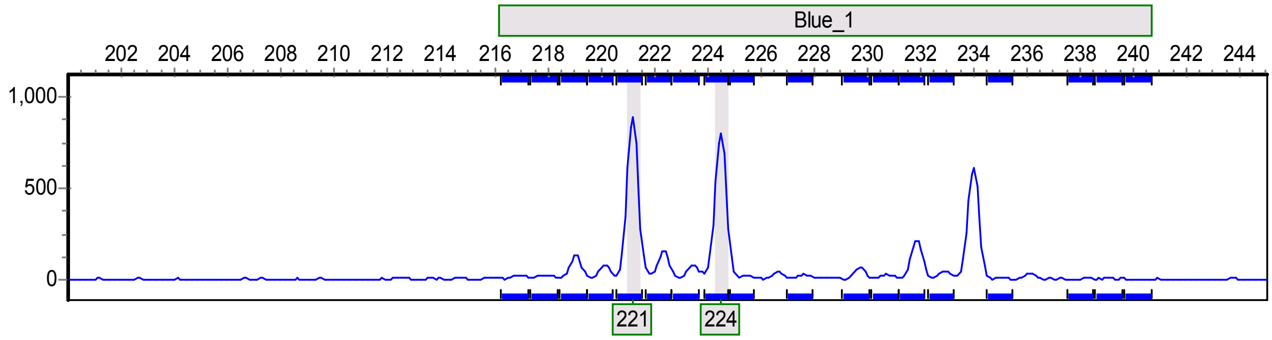

No	Size	Height	Area	Marker	Allele	Difference	Quality	Score	Allele Comments	Sample Comments
1	221.2	888	4275	Blue_1	221	0.1	Pass	184.9		
2	224.5	803	3897	Blue_1	224	0.1	Pass	157.7		

Allele Report

Sample 46: Run date and time: 09/16/2020 - 14:20:02 -> 09/16/2020 - 15:18:49

Dye: Blue - 2 peaks - 50.fsa

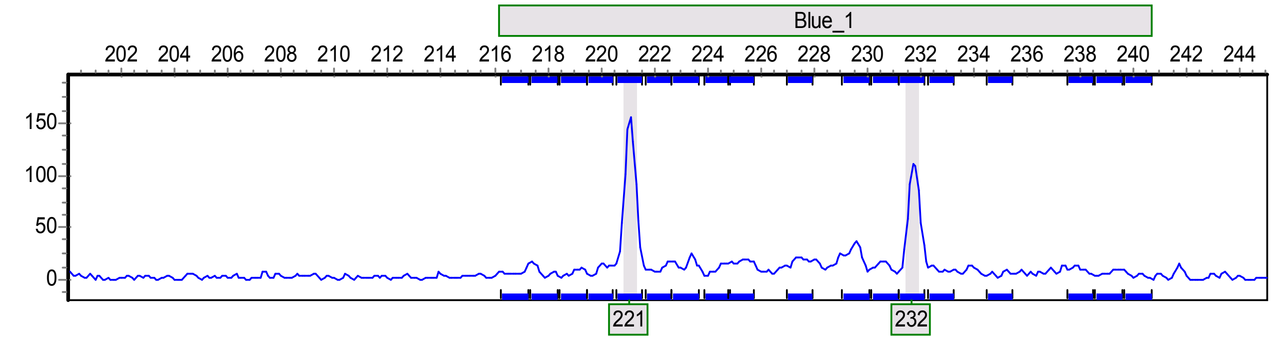

No	Size	Height	Area	Marker	Allele	Difference	Quality	Score	Allele Comments	Sample Comments
1	221.1	155	752	Blue_1	221	0.0	Pass	10.6		
2	231.7	111	561	Blue_1	232	0.0	Pass	5.7	[<Confirmed>]	

Sample 47: Run date and time: 09/16/2020 - 14:20:02 -> 09/16/2020 - 15:18:49

Dye: Blue - 2 peaks - 51.fsa

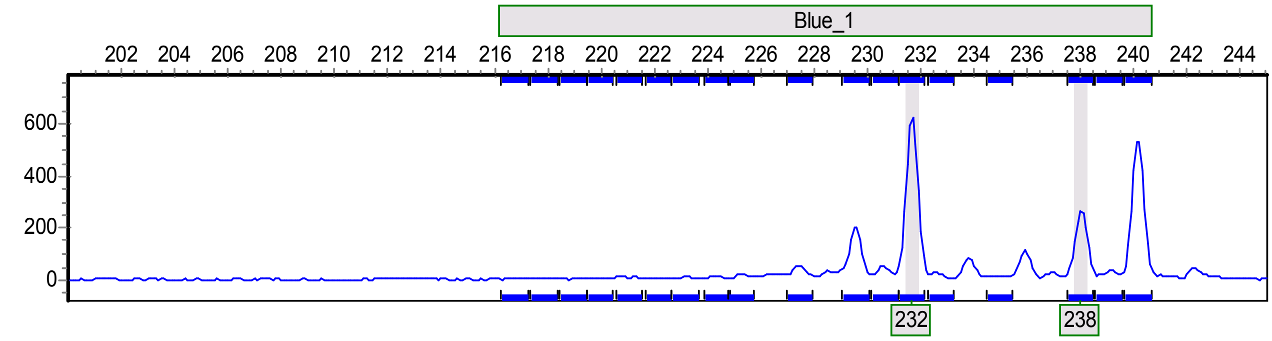

No	Size	Height	Area	Marker	Allele	Difference	Quality	Score	Allele Comments	Sample Comments
1	231.7	623	3042	Blue_1	232	0.0	Pass	105.9		
2	238.0	267	1358	Blue_1	238	0.0	Pass	25.5		

Sample 48: Run date and time: 09/16/2020 - 14:20:02 -> 09/16/2020 - 15:18:49

Dye: Blue - 2 peaks - 52.fsa

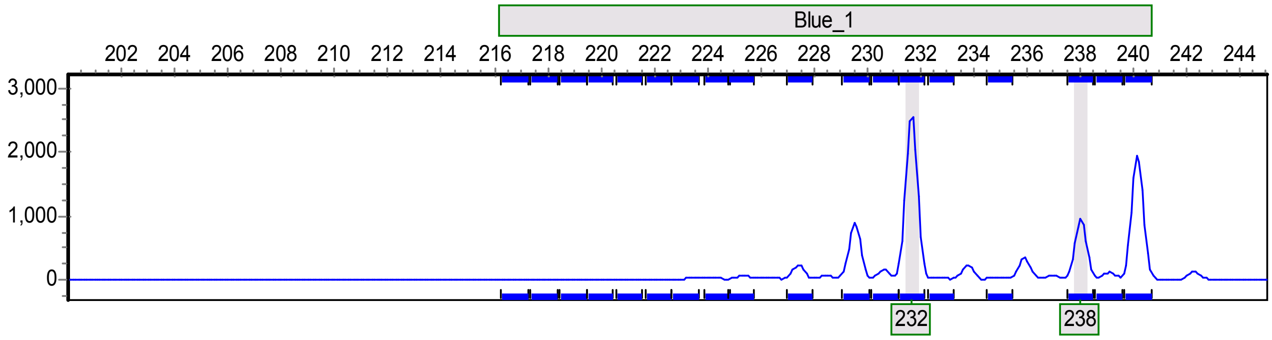

No	Size	Height	Area	Marker	Allele	Difference	Quality	Score	Allele Comments	Sample Comments
1	231.7	2534	12617	Blue_1	232	0.0	Pass	500.0		
2	238.0	946	4561	Blue_1	238	0.0	Pass	202.8		

Sample 49: Run date and time: 09/16/2020 - 14:20:02 -> 09/16/2020 - 15:18:49

Dye: Blue - 0 peaks - 53.fsa

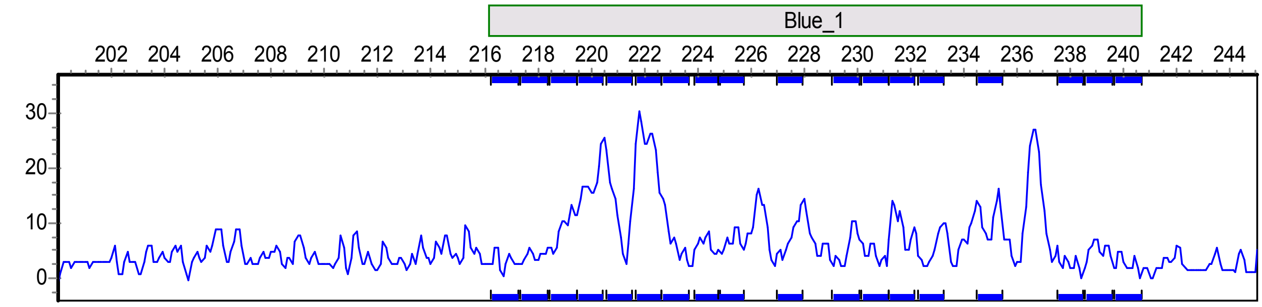

No	Size	Height	Area	Marker	Allele	Difference	Quality	Score	Allele Comments	Sample Comments

Sample 50: Run date and time: 09/16/2020 - 14:20:02 -> 09/16/2020 - 15:18:49

Dye: Blue - 1 peaks - 54.fsa

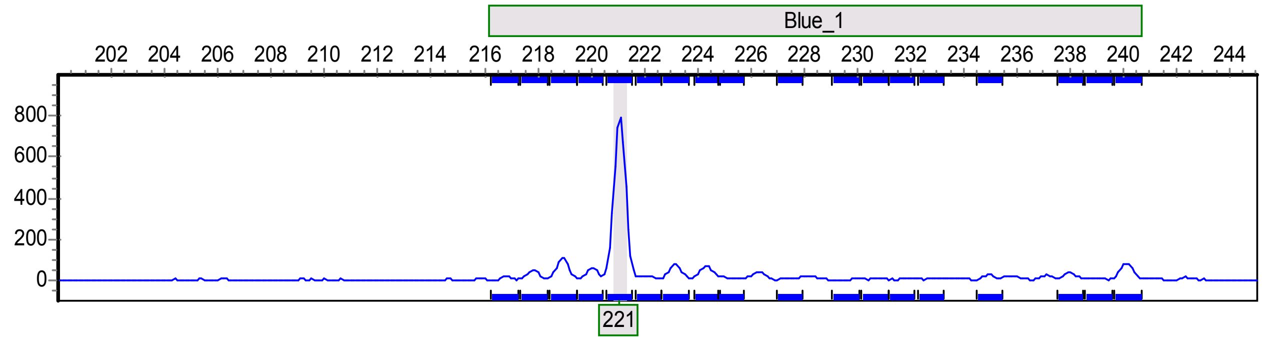

No	Size	Height	Area	Marker	Allele	Difference	Quality	Score	Allele Comments	Sample Comments
1	221.1	788	3865	Blue_1	221	0.0	Pass	150.9		

Sample 51: Run date and time: 09/16/2020 - 14:20:02 -> 09/16/2020 - 15:18:49

Dye: Blue - 1 peaks - 55.fsa

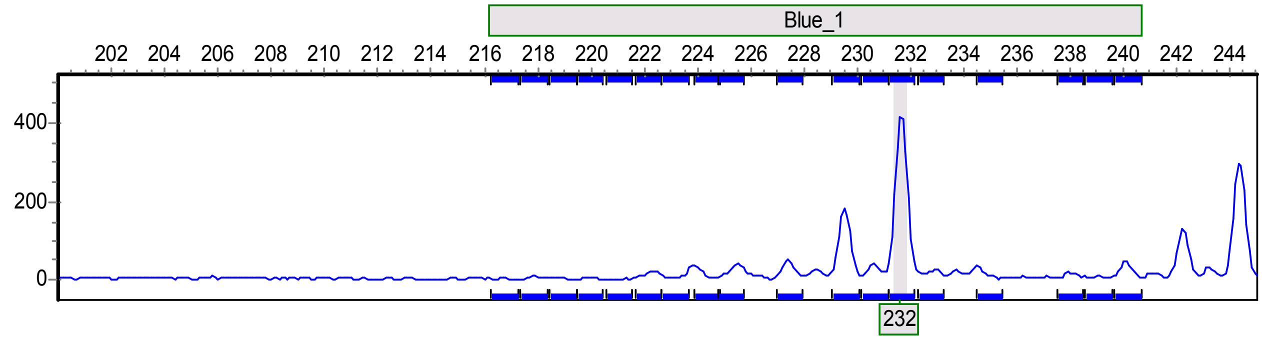

No	Size	Height	Area	Marker	Allele	Difference	Quality	Score	Allele Comments	Sample Comments
1	231.6	416	2093	Blue_1	232	0.1	Pass	54.7		

Sample 52: Run date and time: 09/16/2020 - 14:20:02 -> 09/16/2020 - 15:18:49

Dye: Blue - 2 peaks - 56.fsa

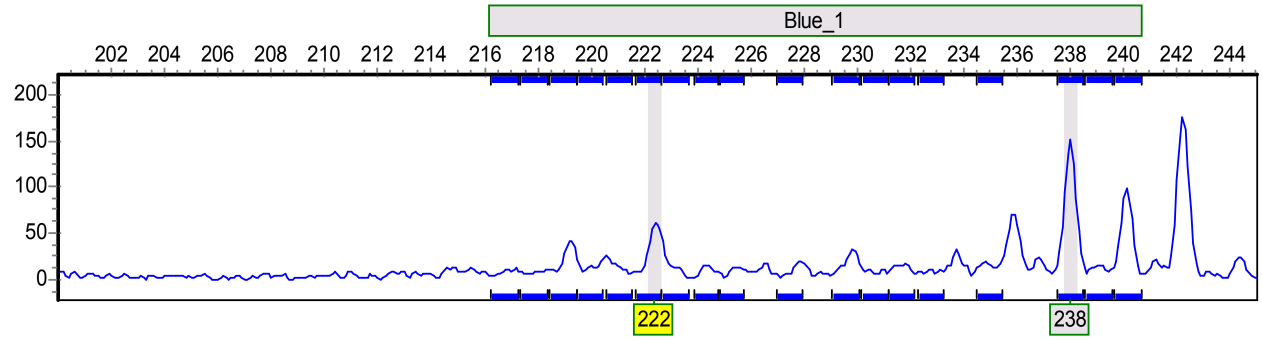

No	Size	Height	Area	Marker	Allele	Difference	Quality	Score	Allele Comments	Sample Comments
1	222.4	60	393	Blue_1	222	0.2	Check	1.1		
2	238.0	151	734	Blue_1	238	0.0	Pass	10.1		

Sample 53: Run date and time: 09/16/2020 - 14:20:02 -> 09/16/2020 - 15:18:49

Dye: Blue - 2 peaks - 57.fsa

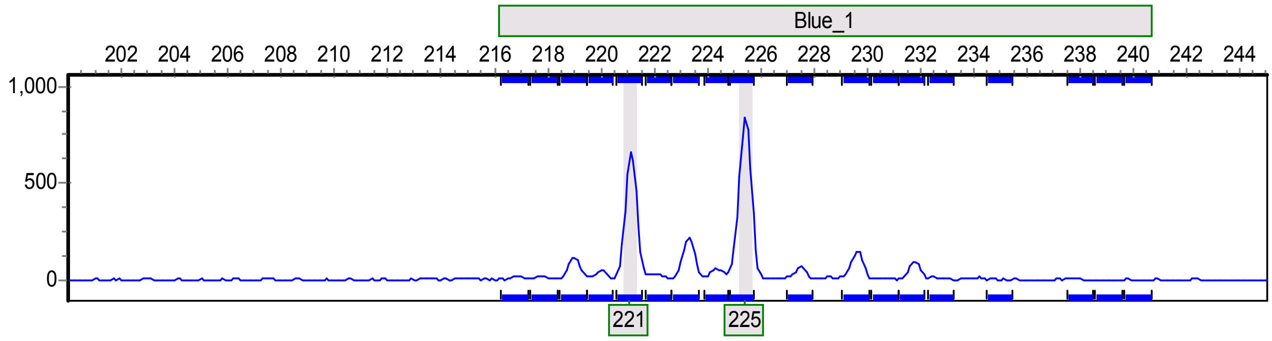

No	Size	Height	Area	Marker	Allele	Difference	Quality	Score	Allele Comments	Sample Comments
1	221.1	658	3202	Blue_1	221	0.0	Pass	116.7		
2	225.4	837	4301	Blue_1	225	0.1	Pass	154.9		

Sample 54: Run date and time: 09/16/2020 - 14:20:02 -> 09/16/2020 - 15:18:49

Dye: Blue - 3 peaks - 58.fsa

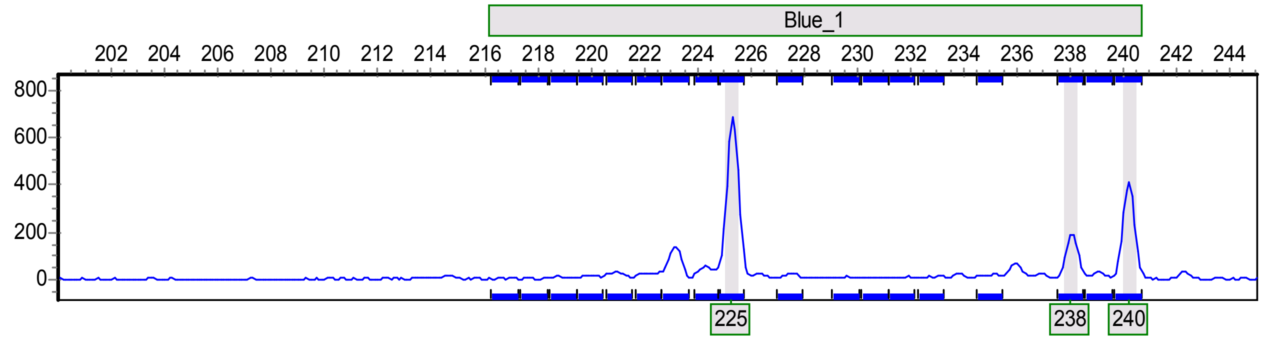

No	Size	Height	Area	Marker	Allele	Difference	Quality	Score	Allele Comments	Sample Comments
1	225.3	684	3319	Blue_1	225	0.0	Pass	123.9		
2	238.0	192	990	Blue_1	238	0.0	Pass	14.3		
3	240.2	412	1956	Blue_1	240	1.0	Pass	58.0	[<Confirmed><Inserted>]	

Sample 55: Run date and time: 09/16/2020 - 14:20:02 -> 09/16/2020 - 15:18:49

Dye: Blue - 1 peaks - 59.fsa

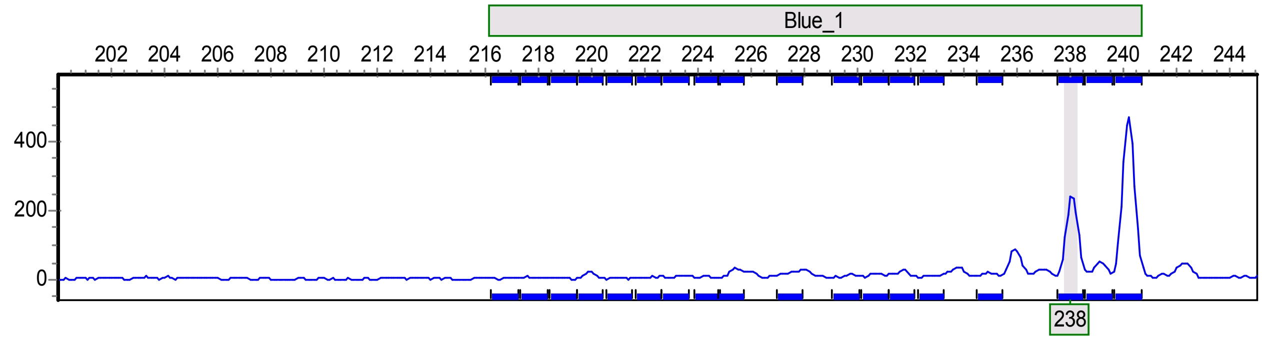

No	Size	Height	Area	Marker	Allele	Difference	Quality	Score	Allele Comments	Sample Comments
1	238.0	240	1210	Blue_1	238	0.0	Pass	21.6		

Sample 56: Run date and time: 09/16/2020 - 14:20:02 -> 09/16/2020 - 15:18:49

Dye: Blue - 2 peaks - 6.fsa

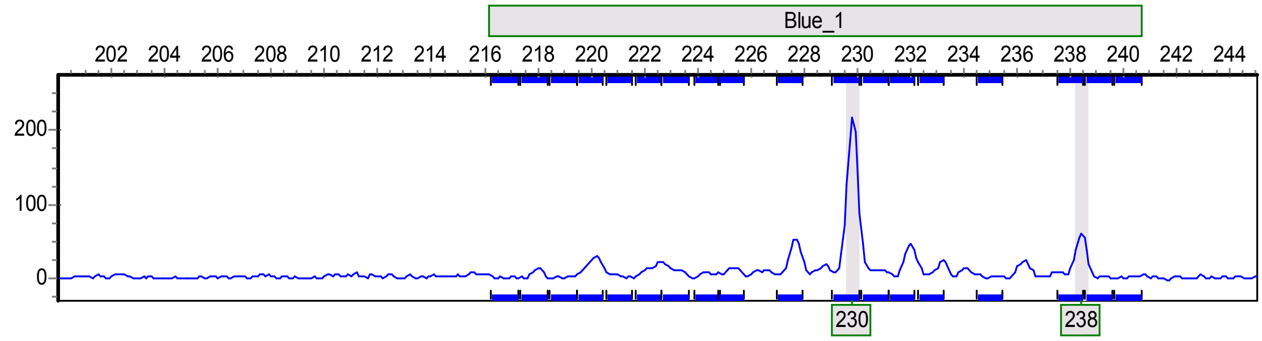

No	Size	Height	Area	Marker	Allele	Difference	Quality	Score	Allele Comments	Sample Comments
1	229.8	217	1068	Blue_1	230	0.2	Pass	19.0		
2	238.4	62	303	Blue_1	238	0.4	Pass	2.1	[<Confirmed>]	

Sample 57: Run date and time: 09/16/2020 - 14:20:02 -> 09/16/2020 - 15:18:49

Dye: Blue - 4 peaks - 60.fsa

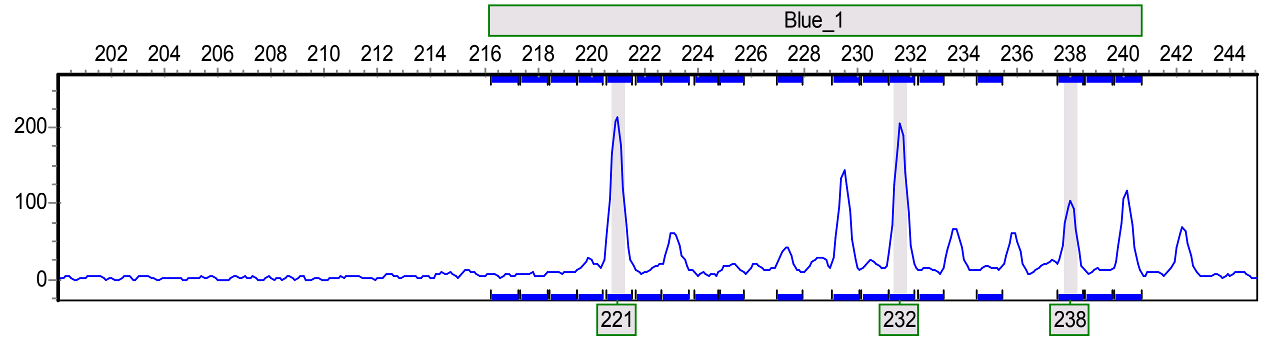

No	Size	Height	Area	Marker	Allele	Difference	Quality	Score	Allele Comments	Sample Comments
1	221.0	212	1128	Blue_1	221	0.1	Pass	15.7		
2	223.1	62	386	Blue_1	223	0.1	Check	1.1	[<Deleted>]	
3	231.6	205	1034	Blue_1	232	0.1	Pass	16.1		
4	238.0	104	557	Blue_1	238	0.0	Pass	4.6	[<Confirmed>]	

Allele Report

Sample 58: Run date and time: 09/16/2020 - 14:20:02 -> 09/16/2020 - 15:18:49

Dye: Blue - 2 peaks - 61.fsa

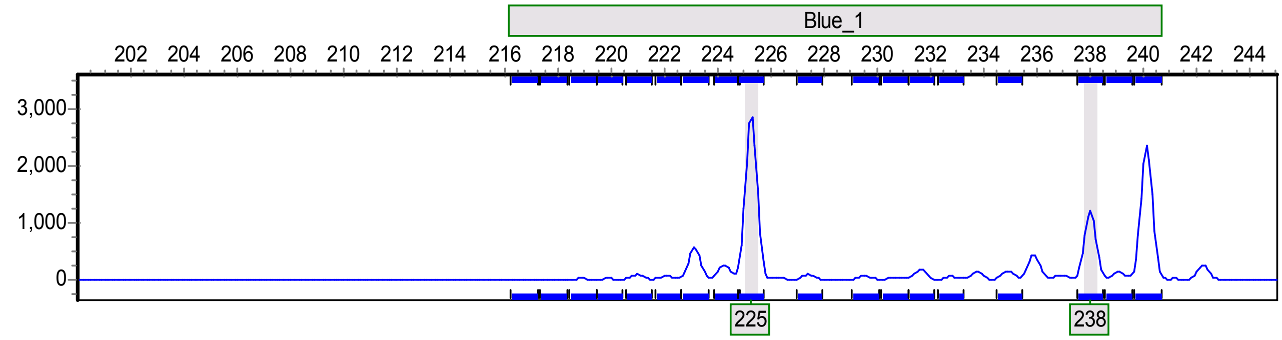

No	Size	Height	Area	Marker	Allele	Difference	Quality	Score	Allele Comments	Sample Comments
1	225.3	2835	13857	Blue_1	225	0.0	Pass	500.0		
2	238.0	1213	5813	Blue_1	238	0.0	Pass	292.2		

Sample 59: Run date and time: 09/16/2020 - 14:20:02 -> 09/16/2020 - 15:18:49

Dye: Blue - 3 peaks - 62.fsa

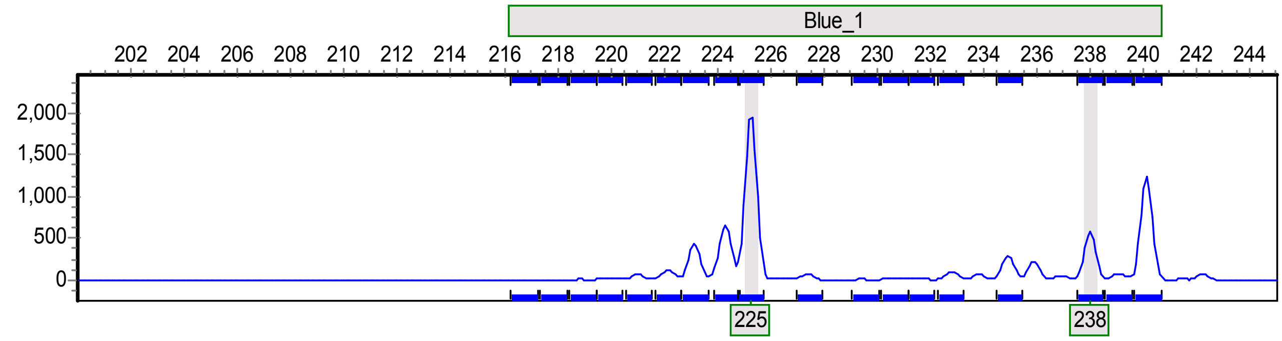

No	Size	Height	Area	Marker	Allele	Difference	Quality	Score	Allele Comments	Sample Comments
1	224.3	663	3524	Blue_1	224	0.1	Pass	102.3	[<Deleted>]	
2	225.3	1933	9561	Blue_1	225	0.0	Pass	500.0		
3	238.0	584	2812	Blue_1	238	0.0	Pass	98.4		

Sample 60: Run date and time: 09/16/2020 - 14:20:02 -> 09/16/2020 - 15:18:49

Dye: Blue - 2 peaks - 63.fsa

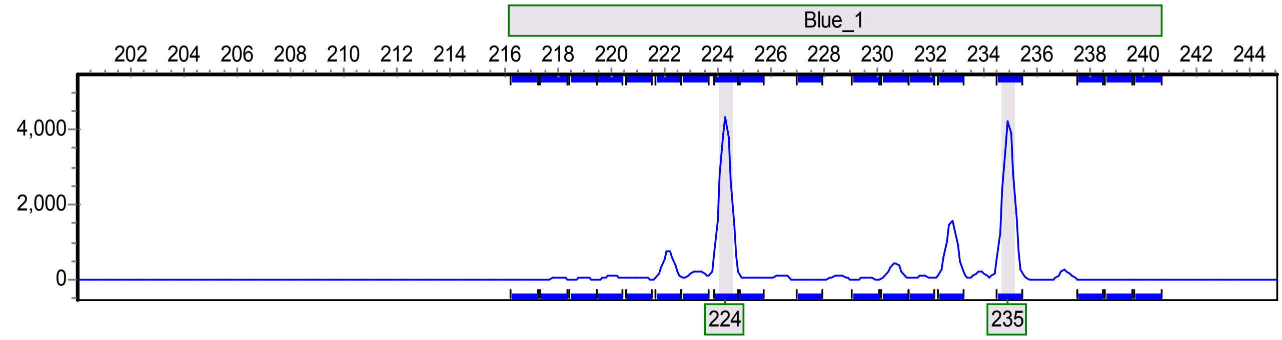

No	Size	Height	Area	Marker	Allele	Difference	Quality	Score	Allele Comments	Sample Comments
1	224.3	4298	20818	Blue_1	224	0.1	Pass	500.0		
2	234.9	4197	20031	Blue_1	235	0.1	Pass	500.0		

Allele Report

Sample 61: Run date and time: 09/16/2020 - 14:20:02 -> 09/16/2020 - 15:18:49

Dye: Blue - 2 peaks - 64.fsa

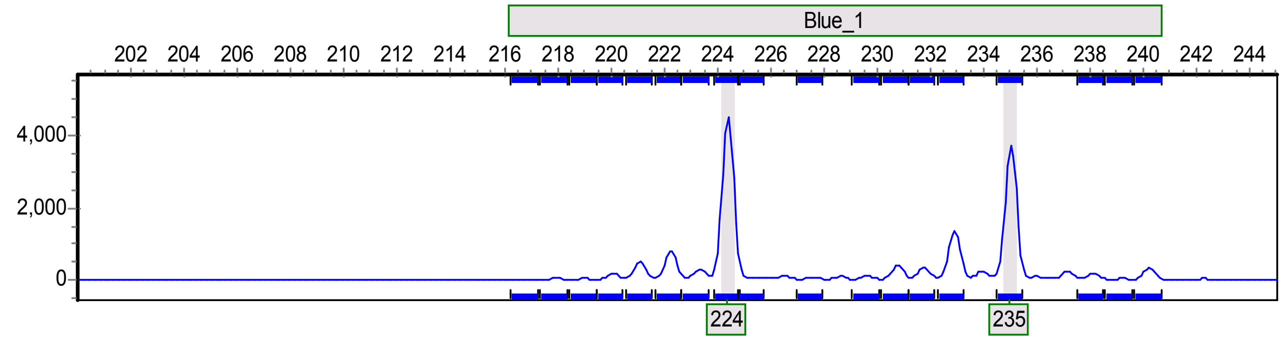

No	Size	Height	Area	Marker	Allele	Difference	Quality	Score	Allele Comments	Sample Comments
1	224.4	4483	21938	Blue_1	224	0.0	Pass	500.0		
2	235.0	3704	17856	Blue_1	235	0.0	Pass	500.0		

Sample 62: Run date and time: 09/16/2020 - 14:20:02 -> 09/16/2020 - 15:18:49

Dye: Blue - 1 peaks - 65.fsa

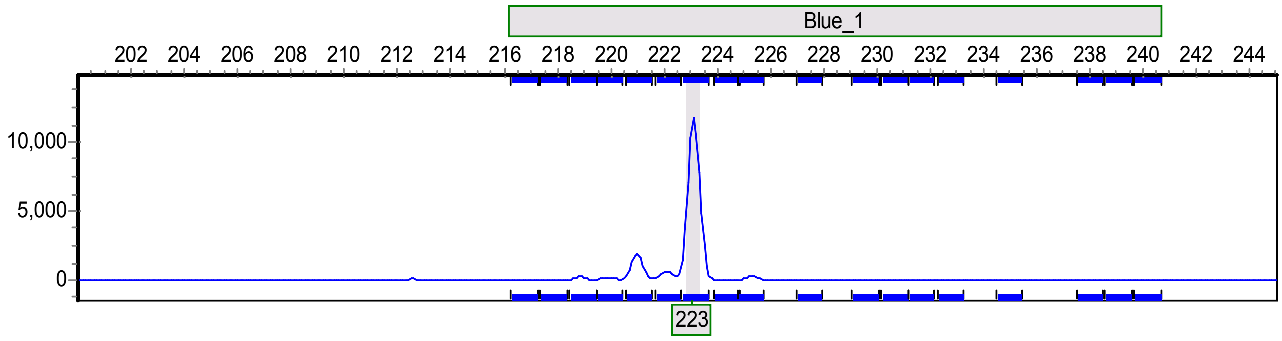

No	Size	Height	Area	Marker	Allele	Difference	Quality	Score	Allele Comments	Sample Comments
1	223.1	11688	57443	Blue_1	223	0.1	Pass	500.0		

Sample 63: Run date and time: 09/16/2020 - 14:20:02 -> 09/16/2020 - 15:18:49

Dye: Blue - 1 peaks - 66.fsa

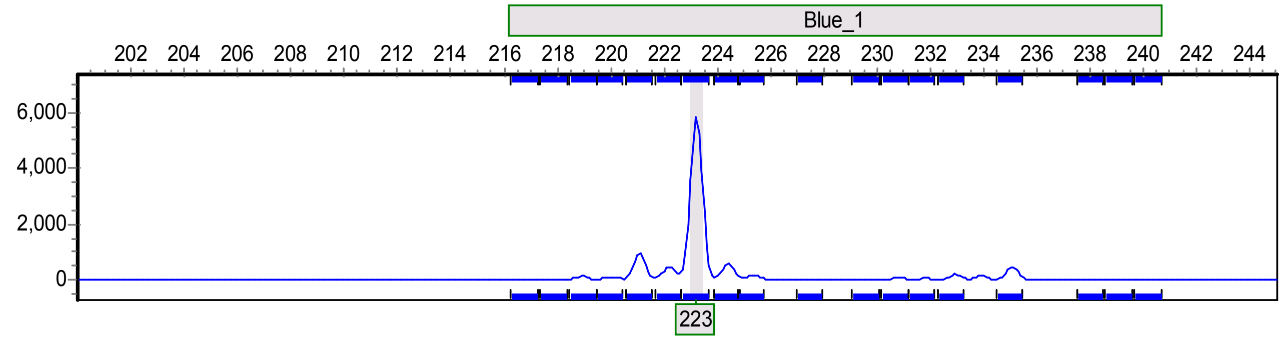

No	Size	Height	Area	Marker	Allele	Difference	Quality	Score	Allele Comments	Sample Comments
1	223.2	5830	29149	Blue_1	223	0.0	Pass	500.0		

Sample 64: Run date and time: 09/16/2020 - 14:20:02 -> 09/16/2020 - 15:18:49

Dye: Blue - 2 peaks - 67.fsa

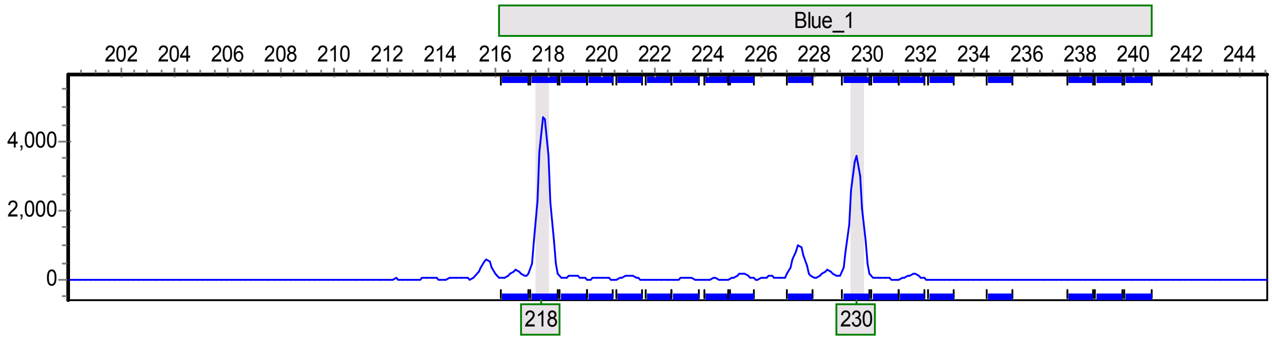

No	Size	Height	Area	Marker	Allele	Difference	Quality	Score	Allele Comments	Sample Comments
1	217.8	4694	23024	Blue_1	218	0.1	Pass	500.0		
2	229.6	3573	17986	Blue_1	230	0.0	Pass	500.0		

Sample 65: Run date and time: 09/16/2020 - 14:20:02 -> 09/16/2020 - 15:18:49

Dye: Blue - 2 peaks - 68.fsa

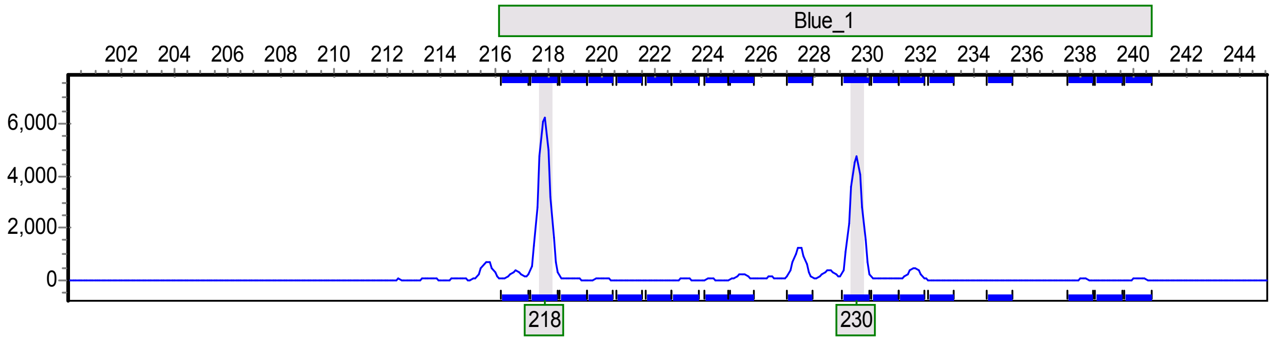

No	Size	Height	Area	Marker	Allele	Difference	Quality	Score	Allele Comments	Sample Comments
1	217.9	6213	30412	Blue_1	218	0.0	Pass	500.0		
2	229.6	4777	24323	Blue_1	230	0.0	Pass	500.0		

Sample 66: Run date and time: 09/16/2020 - 14:20:02 -> 09/16/2020 - 15:18:49

Dye: Blue - 2 peaks - 69.fsa

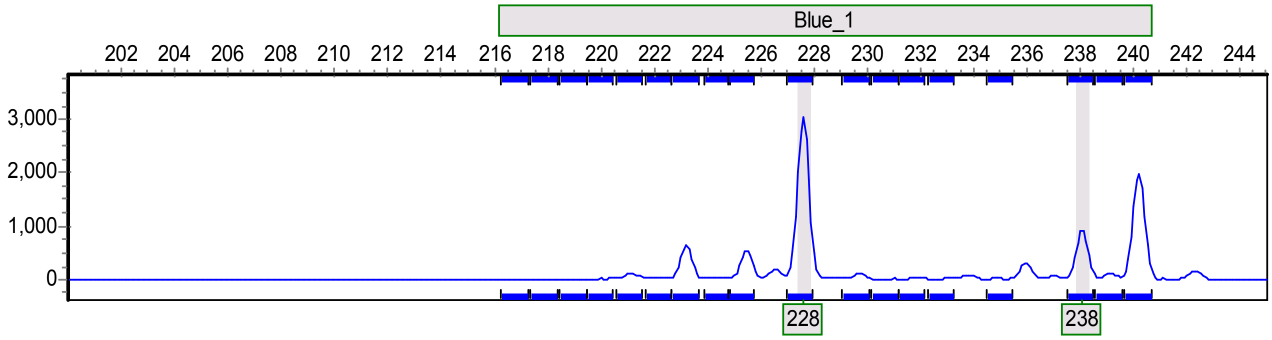

No	Size	Height	Area	Marker	Allele	Difference	Quality	Score	Allele Comments	Sample Comments
1	227.6	3017	14871	Blue_1	228	0.1	Pass	500.0		
2	238.1	912	4485	Blue_1	238	0.1	Pass	188.4		

Sample 67: Run date and time: 09/16/2020 - 14:20:02 -> 09/16/2020 - 15:18:49

Dye: Blue - 0 peaks - 7.fsa

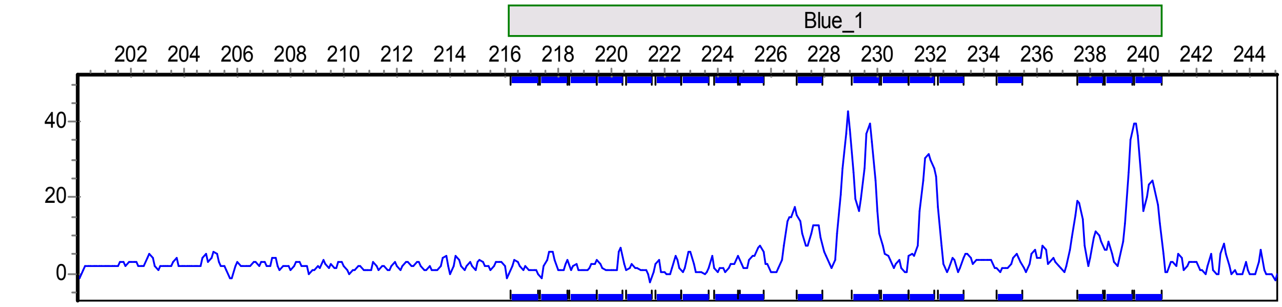

No	Size	Height	Area	Marker	Allele	Difference	Quality	Score	Allele Comments	Sample Comments

Sample 68: Run date and time: 09/16/2020 - 14:20:02 -> 09/16/2020 - 15:18:49

Dye: Blue - 2 peaks - 70.fsa

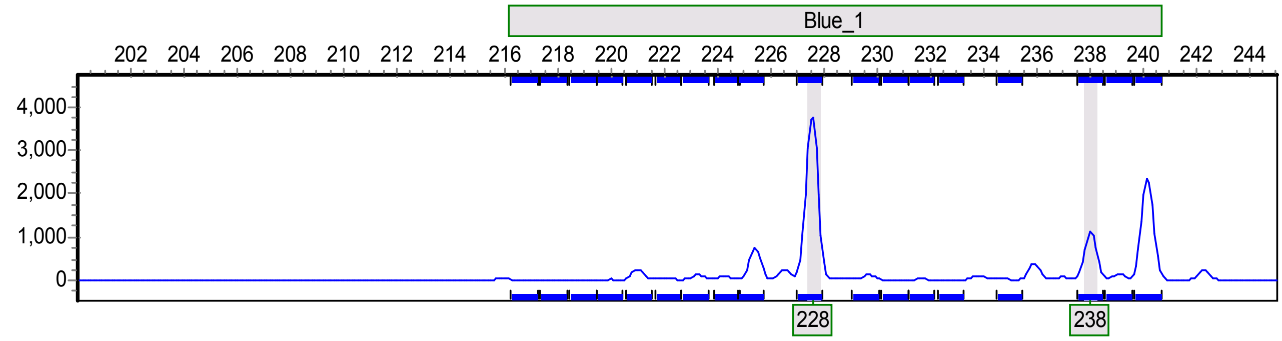

No	Size	Height	Area	Marker	Allele	Difference	Quality	Score	Allele Comments	Sample Comments
1	227.6	3734	19445	Blue_1	228	0.1	Pass	500.0		
2	238.0	1142	5594	Blue_1	238	0.0	Pass	261.3		

Sample 69: Run date and time: 09/16/2020 - 14:20:02 -> 09/16/2020 - 15:18:49

Dye: Blue - 1 peaks - 71.fsa

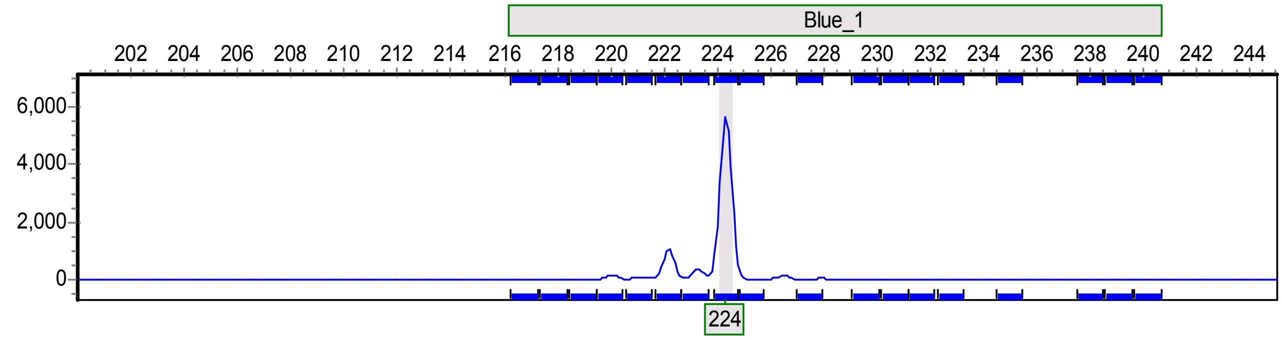

No	Size	Height	Area	Marker	Allele	Difference	Quality	Score	Allele Comments	Sample Comments
1	224.3	5606	27929	Blue_1	224	0.1	Pass	500.0		

Sample 70: Run date and time: 09/16/2020 - 14:20:02 -> 09/16/2020 - 15:18:49

Dye: Blue - 1 peaks - 72.fsa

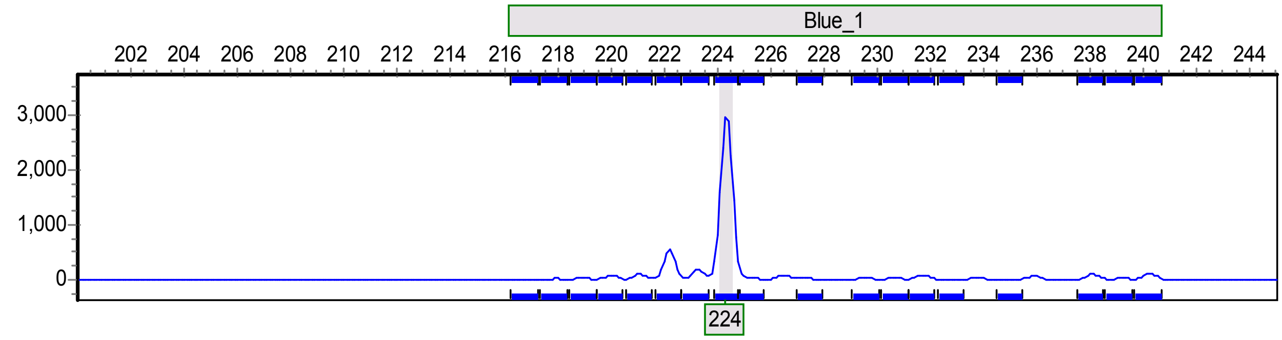

No	Size	Height	Area	Marker	Allele	Difference	Quality	Score	Allele Comments	Sample Comments
1	224.3	2947	14965	Blue_1	224	0.1	Pass	500.0		

Sample 71: Run date and time: 09/16/2020 - 14:20:02 -> 09/16/2020 - 15:18:49

Dye: Blue - 2 peaks - 73.fsa

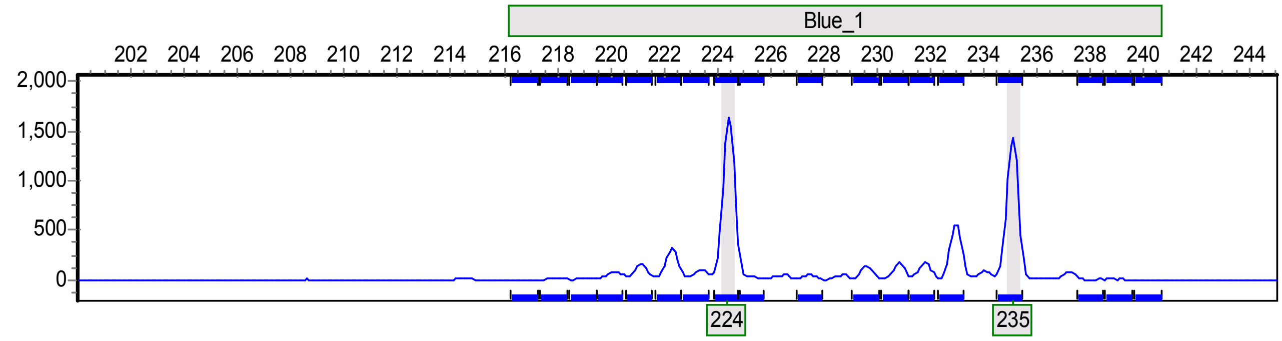

No	Size	Height	Area	Marker	Allele	Difference	Quality	Score	Allele Comments	Sample Comments
1	224.4	1629	8177	Blue_1	224	0.0	Pass	419.4		
2	235.1	1428	7061	Blue_1	235	0.1	Pass	354.6		

Sample 72: Run date and time: 09/16/2020 - 14:20:02 -> 09/16/2020 - 15:18:49

Dye: Blue - 2 peaks - 74.fsa

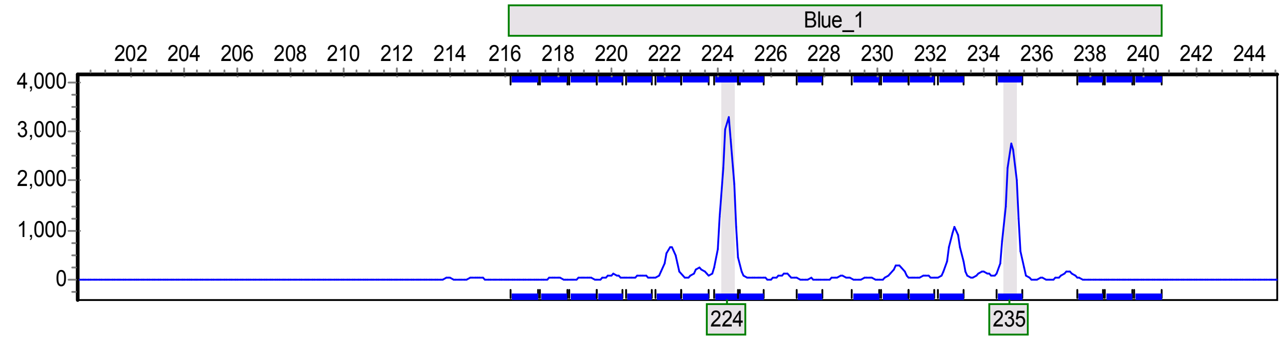

No	Size	Height	Area	Marker	Allele	Difference	Quality	Score	Allele Comments	Sample Comments
1	224.4	3264	15935	Blue_1	224	0.0	Pass	500.0		
2	235.0	2733	13390	Blue_1	235	0.0	Pass	500.0		

Allele Report

Sample 73: Run date and time: 09/16/2020 - 14:20:02 -> 09/16/2020 - 15:18:49

Dye: Blue - 2 peaks - 75.fsa

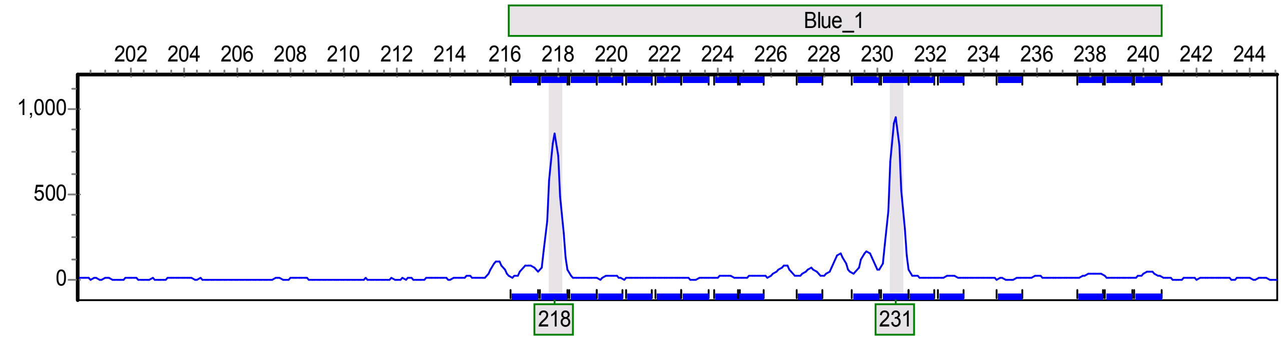

No	Size	Height	Area	Marker	Allele	Difference	Quality	Score	Allele Comments	Sample Comments
1	217.9	853	4167	Blue_1	218	0.0	Pass	170.2		
2	230.7	950	4679	Blue_1	231	0.0	Pass	198.3		

Sample 74: Run date and time: 09/16/2020 - 14:20:02 -> 09/16/2020 - 15:18:49

Dye: Blue - 2 peaks - 76.fsa

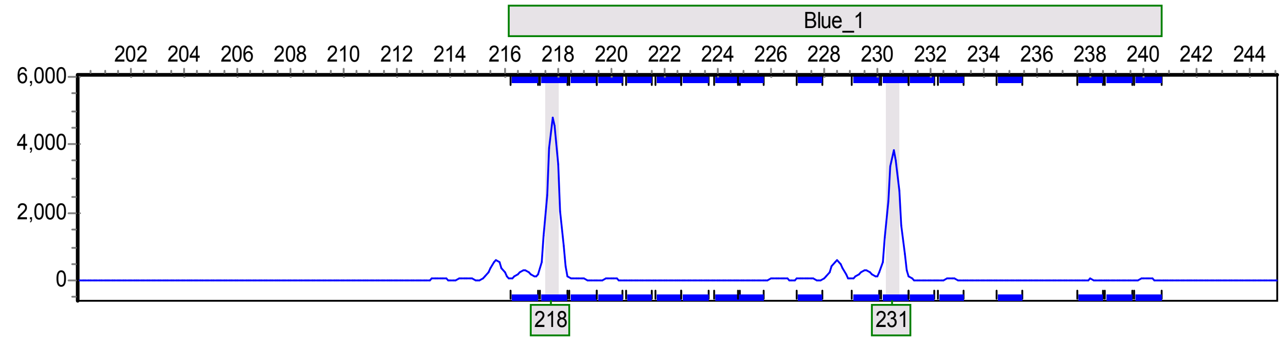

No	Size	Height	Area	Marker	Allele	Difference	Quality	Score	Allele Comments	Sample Comments
1	217.8	4777	23026	Blue_1	218	0.1	Pass	500.0		
2	230.6	3864	19161	Blue_1	231	0.1	Pass	500.0		

Sample 75: Run date and time: 09/16/2020 - 14:20:02 -> 09/16/2020 - 15:18:49

Dye: Blue - 2 peaks - 77.fsa

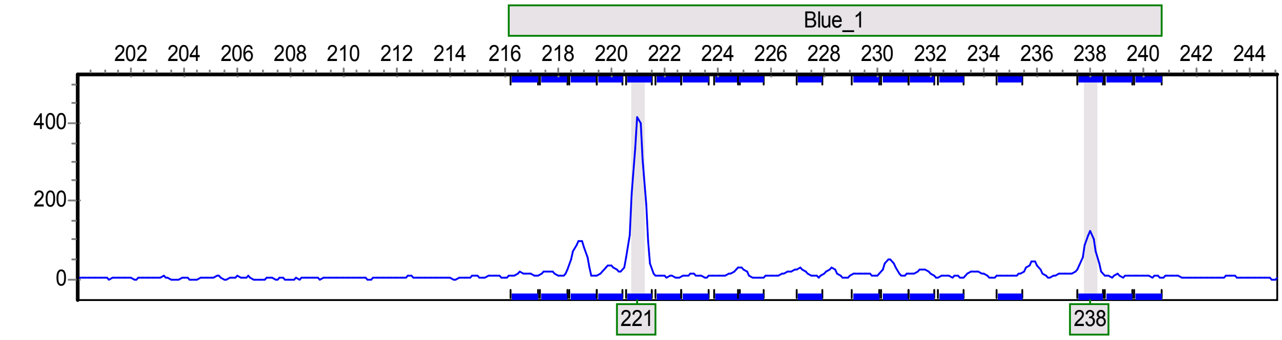

No	Size	Height	Area	Marker	Allele	Difference	Quality	Score	Allele Comments	Sample Comments
1	221.0	414	2052	Blue_1	221	0.1	Pass	55.1		
2	238.0	122	632	Blue_1	238	0.0	Pass	6.3	[<Confirmed>]	

Allele Report

Sample 76: Run date and time: 09/16/2020 - 14:20:02 -> 09/16/2020 - 15:18:49

Dye: Blue - 1 peaks - 78.fsa

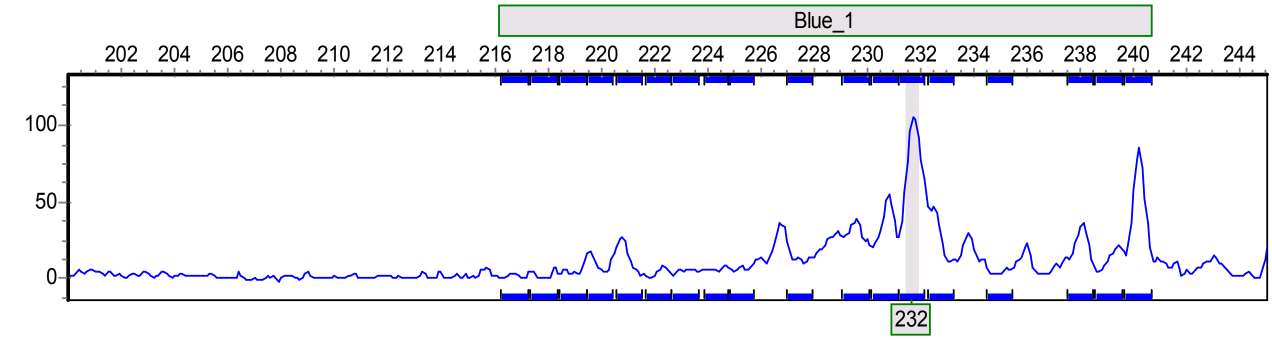

No	Size	Height	Area	Marker	Allele	Difference	Quality	Score	Allele Comments	Sample Comments
1	231.7	105	801	Blue_1	232	0.0	Pass	2.1	[<Confirmed>]	

Sample 77: Run date and time: 09/16/2020 - 14:20:02 -> 09/16/2020 - 15:18:49

Dye: Blue - 1 peaks - 79.fsa

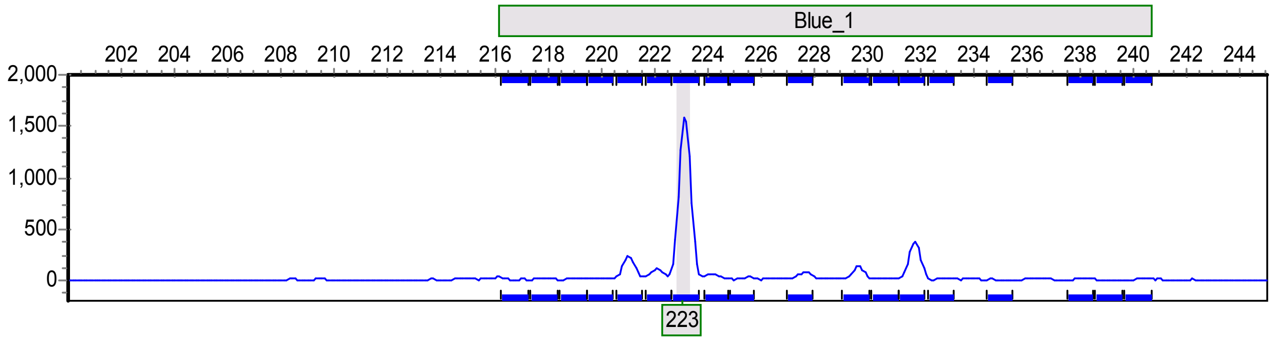

No	Size	Height	Area	Marker	Allele	Difference	Quality	Score	Allele Comments	Sample Comments
1	223.1	1577	7779	Blue_1	223	0.1	Pass	409.8		

Sample 78: Run date and time: 09/16/2020 - 14:20:02 -> 09/16/2020 - 15:18:49

Dye: Blue - 0 peaks - 8.fsa

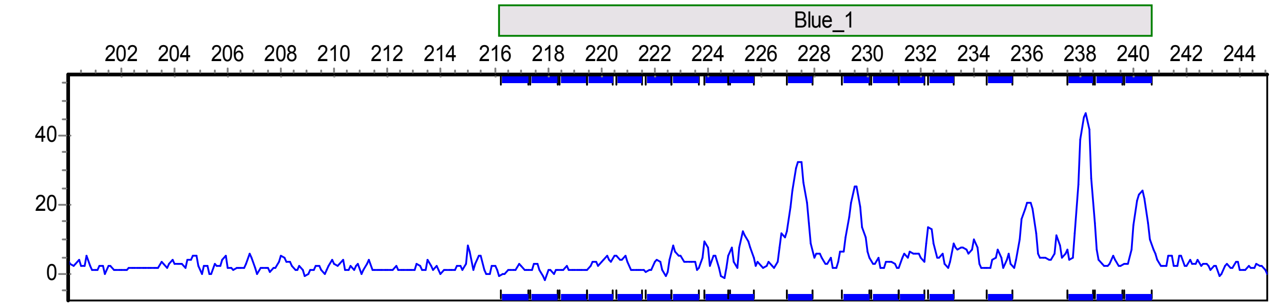

No	Size	Height	Area	Marker	Allele	Difference	Quality	Score	Allele Comments	Sample Comments

Allele Report

Sample 79: Run date and time: 09/16/2020 - 14:20:02 -> 09/16/2020 - 15:18:49

Dye: Blue - 2 peaks - 80.fsa

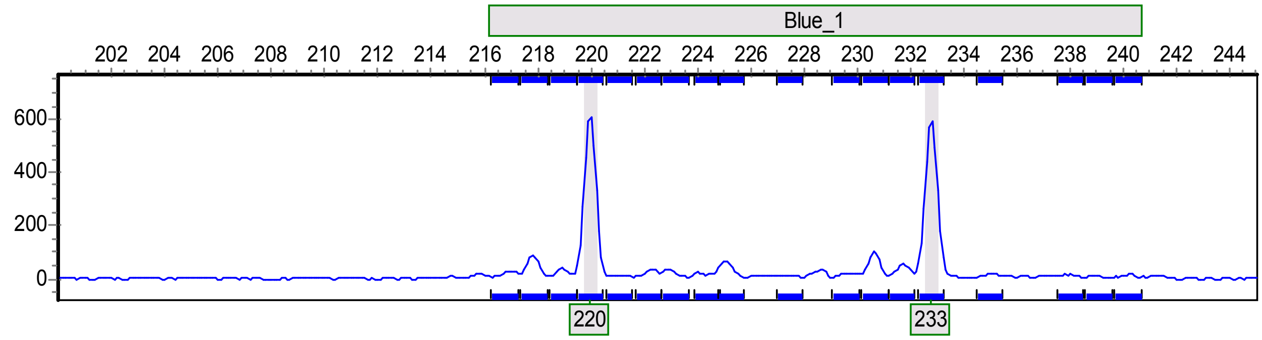

No	Size	Height	Area	Marker	Allele	Difference	Quality	Score	Allele Comments	Sample Comments
1	220.0	601	2995	Blue_1	220	0.0	Pass	98.0		
2	232.8	590	2959	Blue_1	233	0.0	Pass	94.5		

Sample 80: Run date and time: 09/16/2020 - 14:20:02 -> 09/16/2020 - 15:18:49

Dye: Blue - 1 peaks - 81.fsa

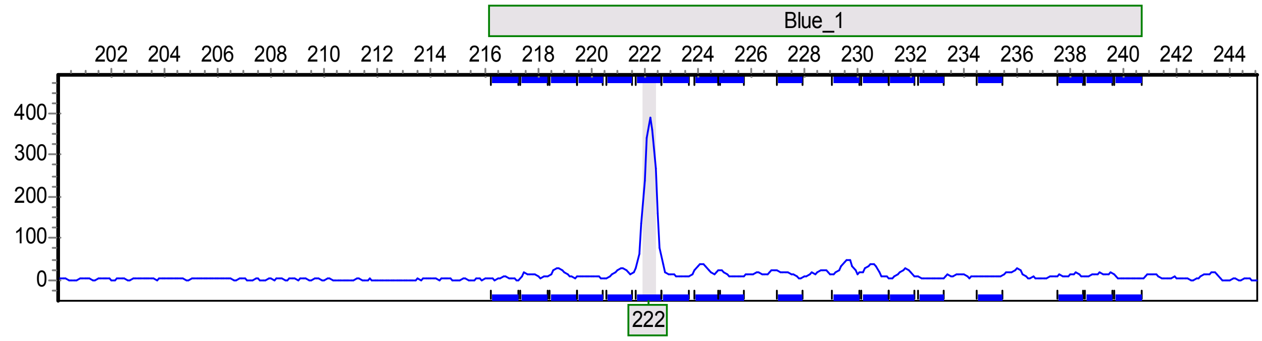

No	Size	Height	Area	Marker	Allele	Difference	Quality	Score	Allele Comments	Sample Comments
1	222.2	388	1947	Blue_1	222	0.0	Pass	48.5		

Sample 81: Run date and time: 09/16/2020 - 14:20:02 -> 09/16/2020 - 15:18:49

Dye: Blue - 1 peaks - 82.fsa

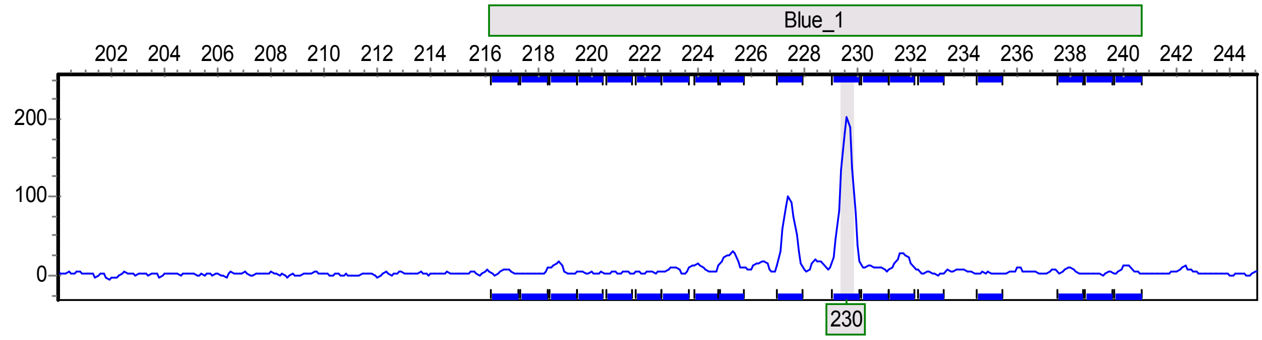

No	Size	Height	Area	Marker	Allele	Difference	Quality	Score	Allele Comments	Sample Comments
1	229.6	203	1052	Blue_1	230	0.0	Pass	15.4		

Allele Report

Sample 82: Run date and time: 09/16/2020 - 14:20:02 -> 09/16/2020 - 15:18:49

Dye: Blue - 1 peaks - 83.fsa

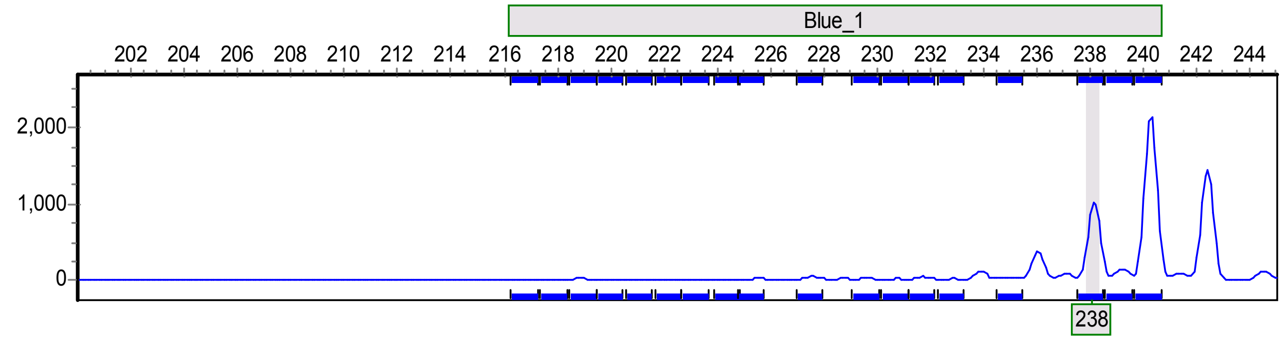

No	Size	Height	Area	Marker	Allele	Difference	Quality	Score	Allele Comments	Sample Comments
1	238.1	1025	5153	Blue_1	238	0.1	Pass	215.8		

Sample 83: Run date and time: 09/16/2020 - 14:20:02 -> 09/16/2020 - 15:18:49

Dye: Blue - 1 peaks - 84.fsa

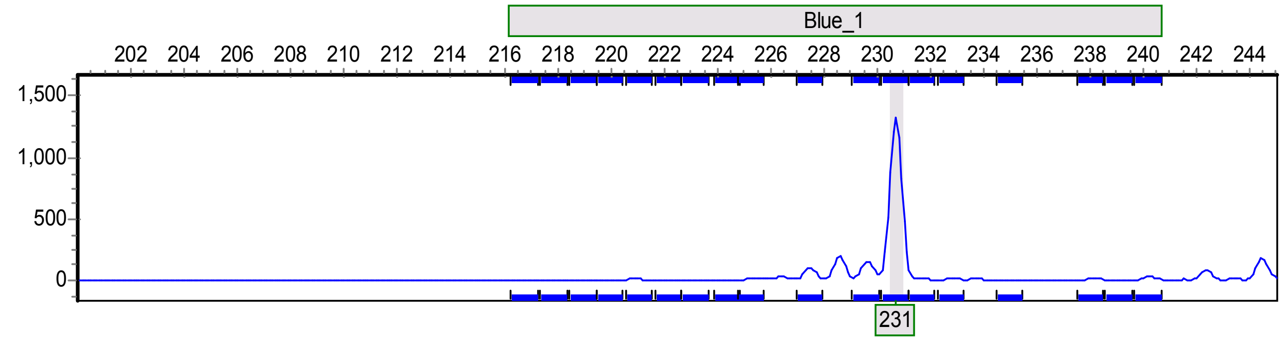

No	Size	Height	Area	Marker	Allele	Difference	Quality	Score	Allele Comments	Sample Comments
1	230.7	1315	6540	Blue_1	231	0.0	Pass	313.4		

Sample 84: Run date and time: 09/16/2020 - 14:20:02 -> 09/16/2020 - 15:18:49

Dye: Blue - 3 peaks - 85.fsa

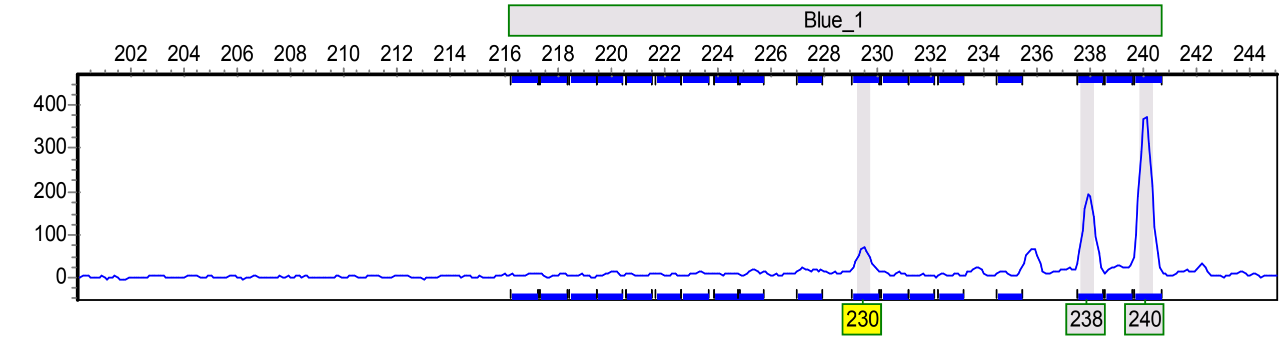

No	Size	Height	Area	Marker	Allele	Difference	Quality	Score	Allele Comments	Sample Comments
1	229.5	70	411	Blue_1	230	0.1	Check	1.7		
2	237.9	192	1011	Blue_1	238	0.1	Pass	13.9		
3	240.1	370	1954	Blue_1	240	1.0	Pass	41.4	[<Confirmed><Inserted>]	

Allele Report

Sample 85: Run date and time: 09/16/2020 - 14:20:02 -> 09/16/2020 - 15:18:49

Dye: Blue - 2 peaks - 86.fsa

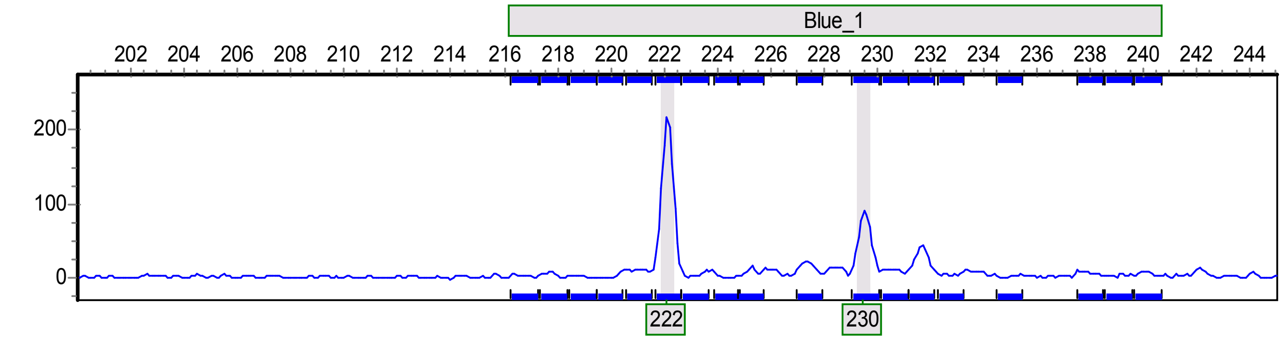

No	Size	Height	Area	Marker	Allele	Difference	Quality	Score	Allele Comments	Sample Comments
1	222.1	215	1060	Blue_1	222	0.1	Pass	18.6		
2	229.5	91	492	Blue_1	230	0.1	Pass	3.4	[<Confirmed>]	

Sample 86: Run date and time: 09/16/2020 - 14:20:02 -> 09/16/2020 - 15:18:49

Dye: Blue - 1 peaks - 87.fsa

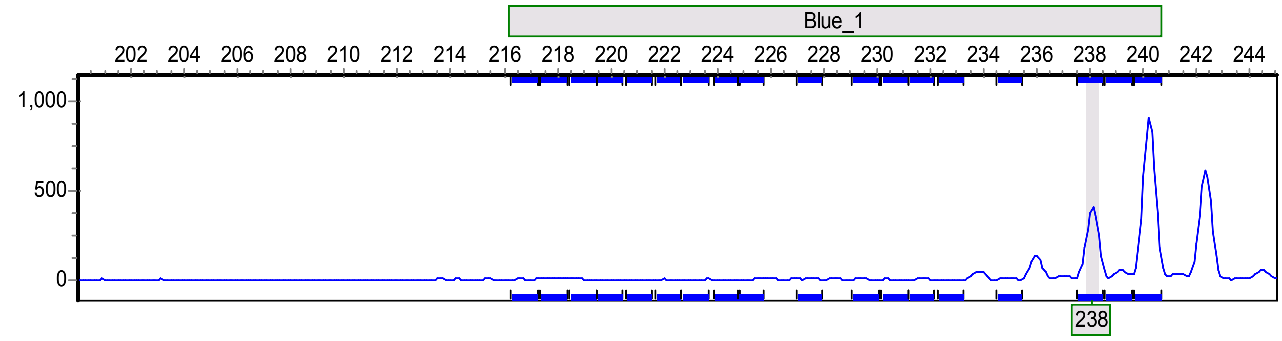

No	Size	Height	Area	Marker	Allele	Difference	Quality	Score	Allele Comments	Sample Comments
1	238.1	413	2084	Blue_1	238	0.1	Pass	53.5		

Sample 87: Run date and time: 09/16/2020 - 14:20:02 -> 09/16/2020 - 15:18:49

Dye: Blue - 1 peaks - 88.fsa

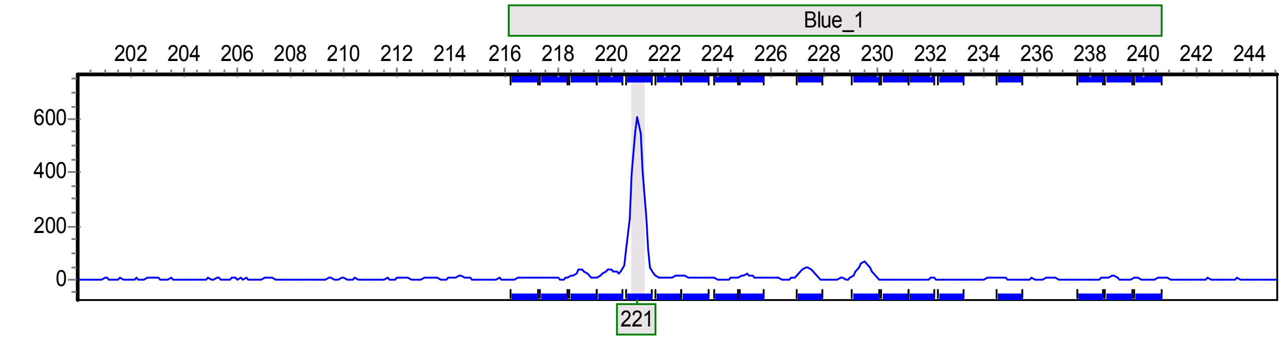

No	Size	Height	Area	Marker	Allele	Difference	Quality	Score	Allele Comments	Sample Comments
1	221.0	604	3059	Blue_1	221	0.1	Pass	95.5		

Sample 88: Run date and time: 09/16/2020 - 14:20:02 -> 09/16/2020 - 15:18:49

Dye: Blue - 0 peaks - 89.fsa

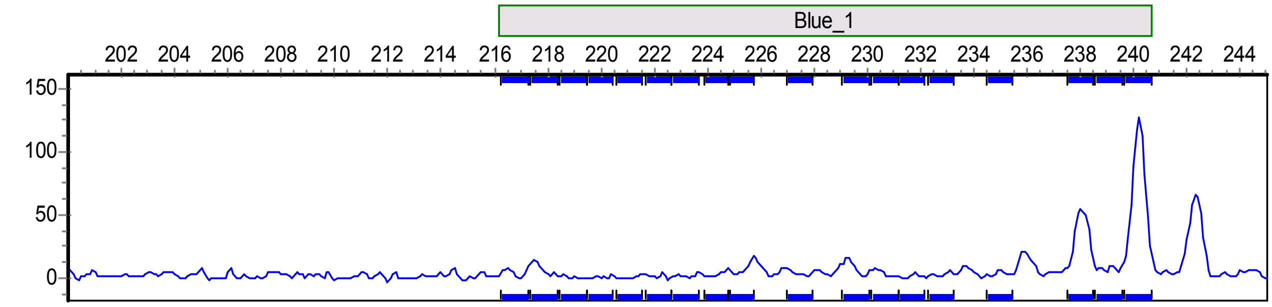

No	Size	Height	Area	Marker	Allele	Difference	Quality	Score	Allele Comments	Sample Comments

Sample 89: Run date and time: 09/16/2020 - 14:20:02 -> 09/16/2020 - 15:18:49

Dye: Blue - 1 peaks - 9.fsa

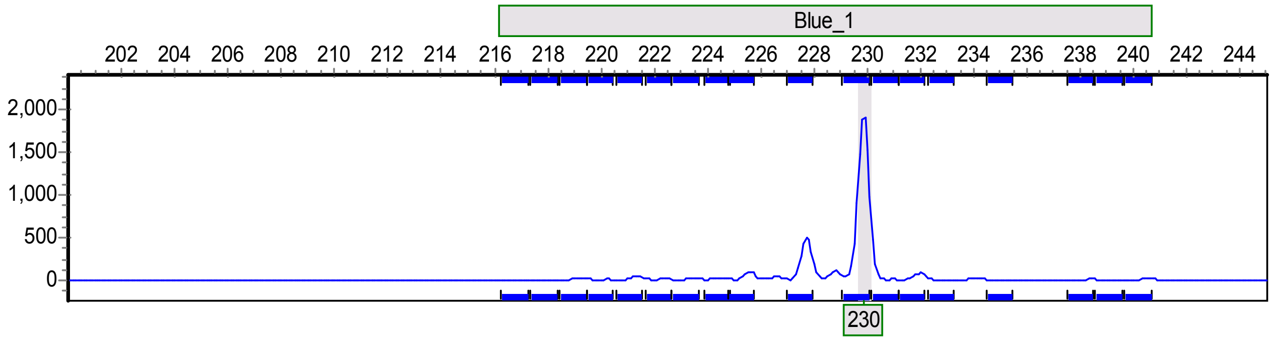

No	Size	Height	Area	Marker	Allele	Difference	Quality	Score	Allele Comments	Sample Comments
1	229.9	1885	9281	Blue_1	230	0.3	Pass	500.0		

Sample 90: Run date and time: 09/16/2020 - 14:20:02 -> 09/16/2020 - 15:18:49

Dye: Blue - 1 peaks - 90.fsa

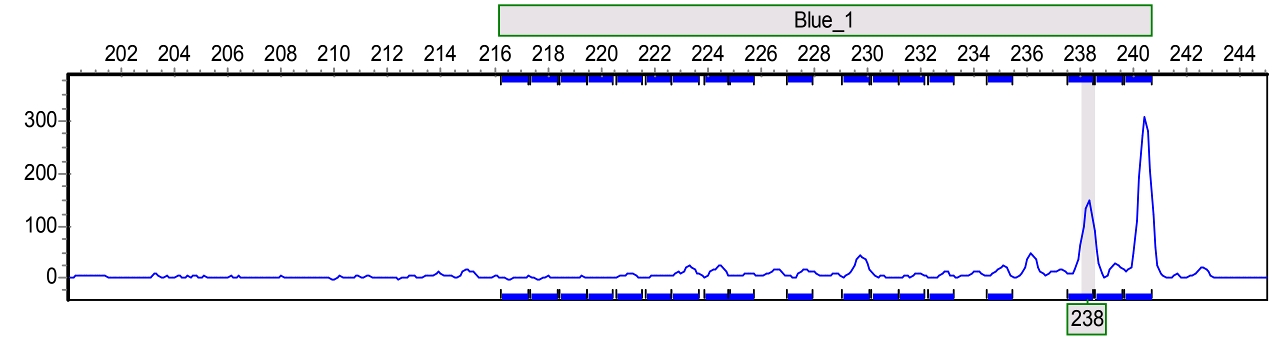

No	Size	Height	Area	Marker	Allele	Difference	Quality	Score	Allele Comments	Sample Comments
1	238.3	147	753	Blue_1	238	0.3	Pass	9.2		

Sample 91: Run date and time: 09/16/2020 - 14:20:02 -> 09/16/2020 - 15:18:49

Dye: Blue - 6 peaks - 91.fsa

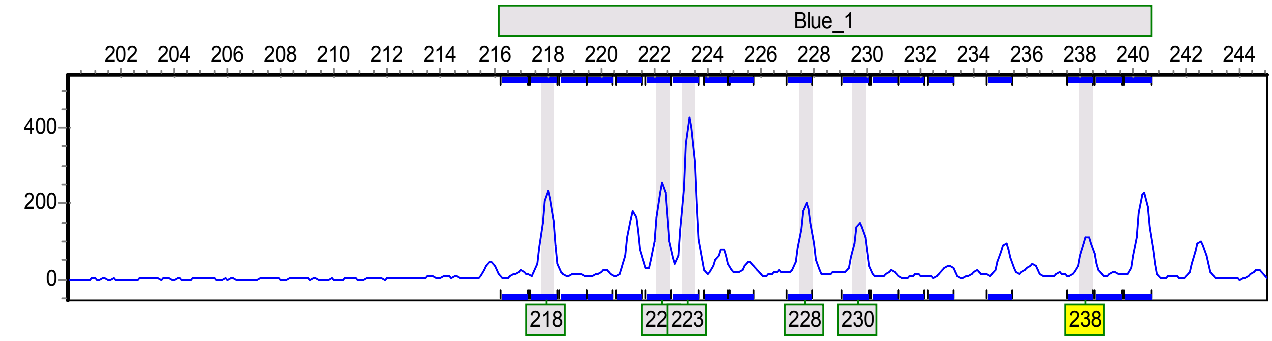

No	Size	Height	Area	Marker	Allele	Difference	Quality	Score	Allele Comments	Sample Comments
1	218.0	232	1163	Blue_1	218	0.1	Pass	20.4		
2	222.3	254	1331	Blue_1	222	0.1	Pass	22.5		
3	223.3	424	2160	Blue_1	223	0.1	Pass	54.7		
4	227.7	200	1118	Blue_1	228	0.2	Pass	13.3		
5	229.7	150	822	Blue_1	230	0.1	Pass	8.0		
6	238.2	114	660	Blue_1	238	0.2	Check	4.6		

Sample 92: Run date and time: 09/16/2020 - 14:20:02 -> 09/16/2020 - 15:18:49

Dye: Blue - 2 peaks - 92.fsa

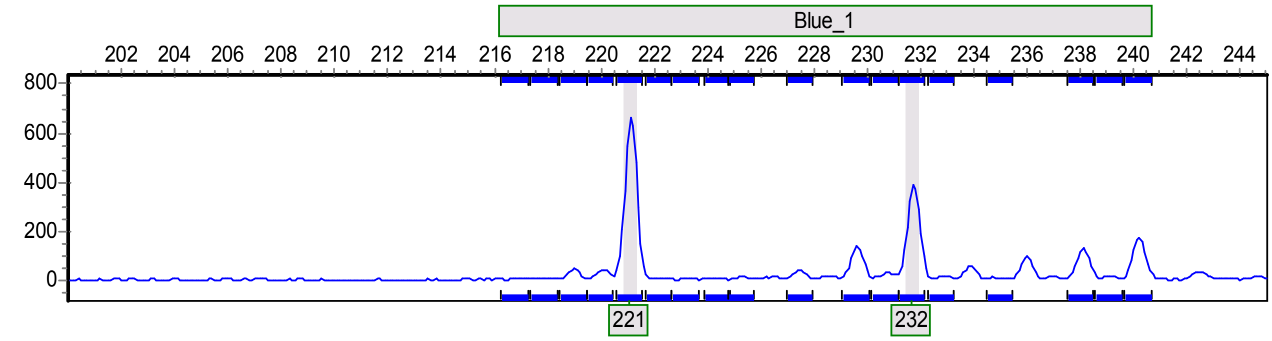

No	Size	Height	Area	Marker	Allele	Difference	Quality	Score	Allele Comments	Sample Comments
1	221.1	659	3304	Blue_1	221	0.0	Pass	112.7		
2	231.7	388	2006	Blue_1	232	0.0	Pass	46.4		

Sample 93: Run date and time: 09/16/2020 - 14:20:02 -> 09/16/2020 - 15:18:49

Dye: Blue - 2 peaks - 93.fsa

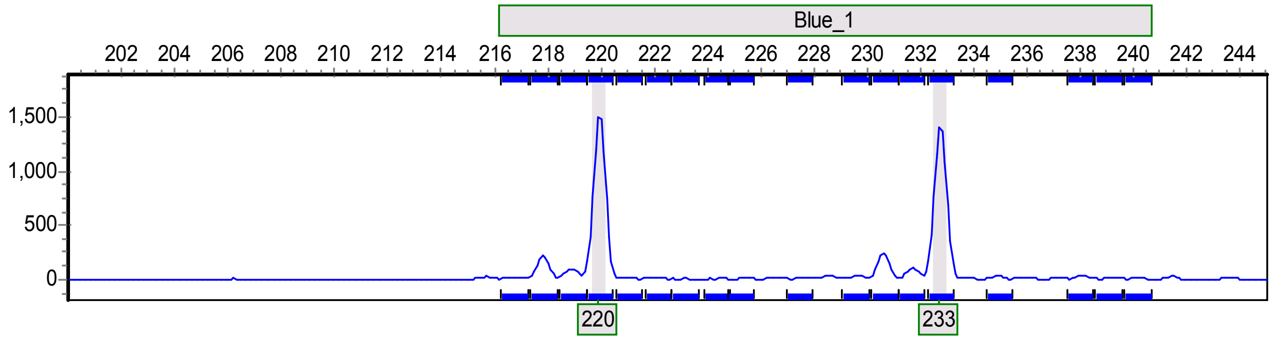

No	Size	Height	Area	Marker	Allele	Difference	Quality	Score	Allele Comments	Sample Comments
1	219.9	1488	7565	Blue_1	220	0.1	Pass	364.1		
2	232.7	1401	7180	Blue_1	233	0.1	Pass	329.7		

Allele Report

Sample 94: Run date and time: 09/16/2020 - 14:20:02 -> 09/16/2020 - 15:18:49

Dye: Blue - 7 peaks - 94.fsa

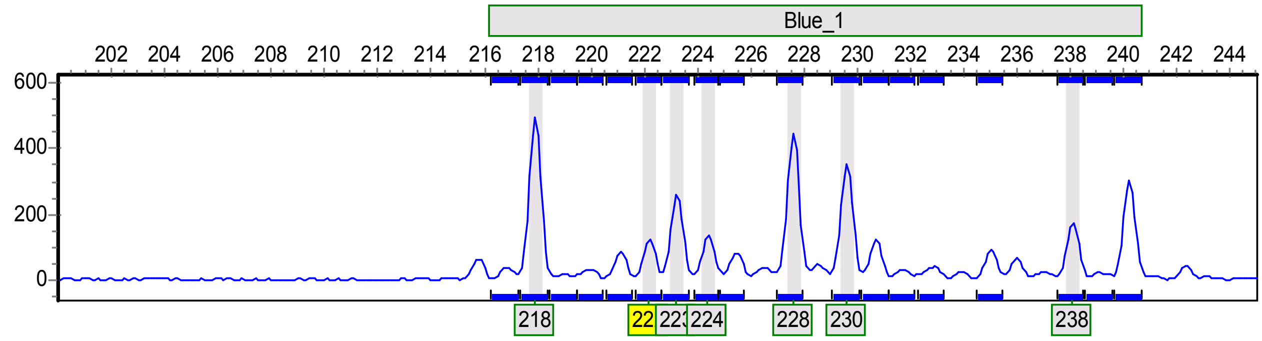

No	Size	Height	Area	Marker	Allele	Difference	Quality	Score	Allele Comments	Sample Comments
1	217.9	491	2408	Blue_1	218	0.0	Pass	73.6		
2	222.2	124	647	Blue_1	222	0.0	Check	6.1		
3	223.2	258	1317	Blue_1	223	0.0	Pass	23.8		
4	224.4	137	712	Blue_1	224	0.0	Pass	7.9		
5	227.6	441	2258	Blue_1	228	0.1	Pass	57.3		
6	229.6	349	1778	Blue_1	230	0.0	Pass	39.9		
7	238.1	175	890	Blue_1	238	0.1	Pass	12.3		

Sample 95: Run date and time: 09/16/2020 - 14:20:02 -> 09/16/2020 - 15:18:49

Dye: Blue - 5 peaks - 95.fsa

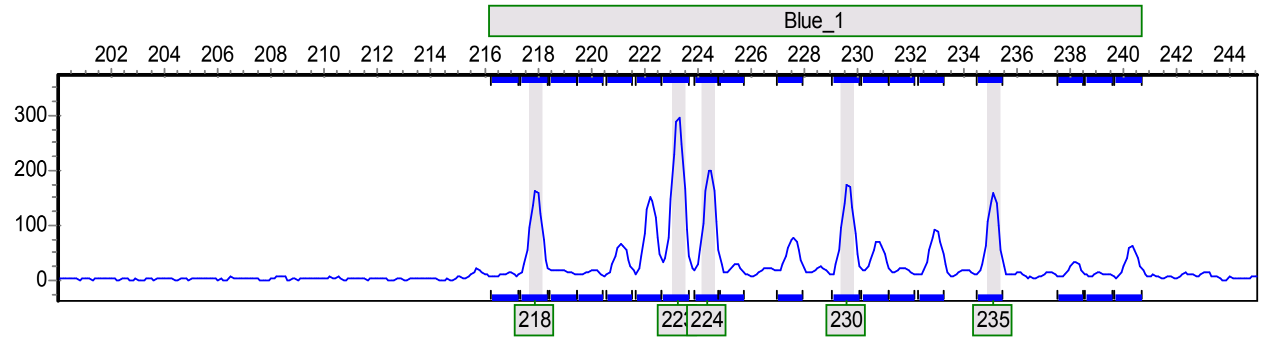

No	Size	Height	Area	Marker	Allele	Difference	Quality	Score	Allele Comments	Sample Comments
1	217.9	165	855	Blue_1	218	0.0	Pass	10.6		
2	223.3	296	1554	Blue_1	223	0.1	Pass	28.8		
3	224.4	202	1051	Blue_1	224	0.0	Pass	15.1		
4	229.6	173	914	Blue_1	230	0.0	Pass	11.4		
5	235.1	159	793	Blue_1	235	0.1	Pass	10.5		

Sample 96: Run date and time: 09/16/2020 - 14:20:02 -> 09/16/2020 - 15:18:49

Dye: Blue - 0 peaks - 96.fsa

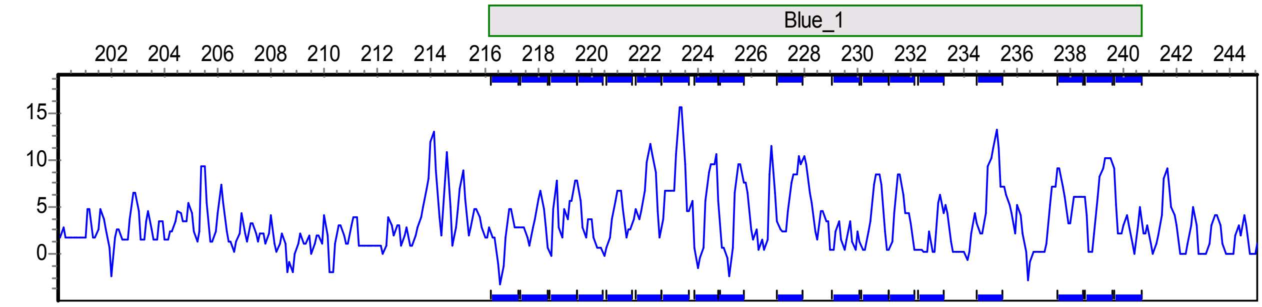

No	Size	Height	Area	Marker	Allele	Difference	Quality	Score	Allele Comments	Sample Comments
