# Supplementary material for: Genetic diversity and population structure of Polygonatum cyrtonema Hua in China using SSR markers
Source: PLoS One. 2023 Aug 31;18(8):e0290605. doi: 10.1371/journal.pone.0290605 (PMC10470896; doi:10.1371/journal.pone.0290605)
Supplement: S1 Data — (ZIP) [file pone.0290605.s002.zip › ╨┬╜¿╬─╝■╝╨ (2)/╥2╬∩19/yinwu19.pdf]

Allele Report

Sample 1: Run date and time: 09/15/2020 - 18:52:27 -> 09/15/2020 - 19:38:19

Dye: Blue - 0 peaks - 1.fsa

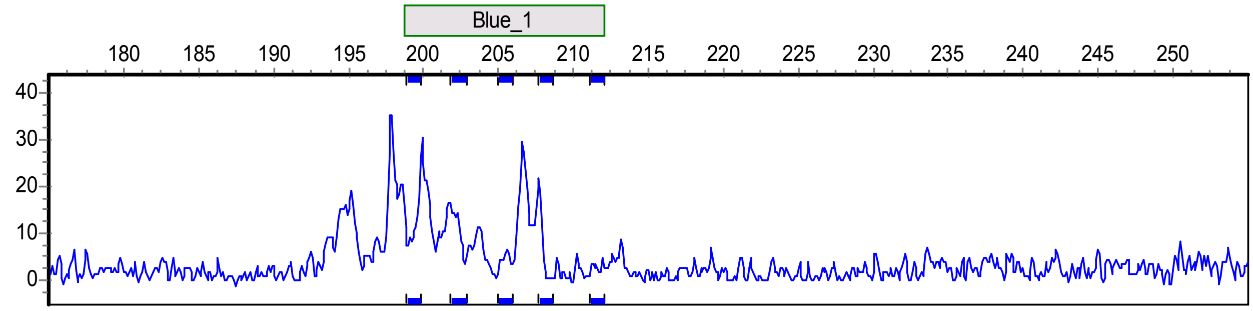

No	Size	Height	Area	Marker	Allele	Difference	Quality	Score	Allele Comments	Sample Comments

Sample 2: Run date and time: 09/15/2020 - 18:52:27 -> 09/15/2020 - 19:38:19

Dye: Blue - 0 peaks - 10.fsa

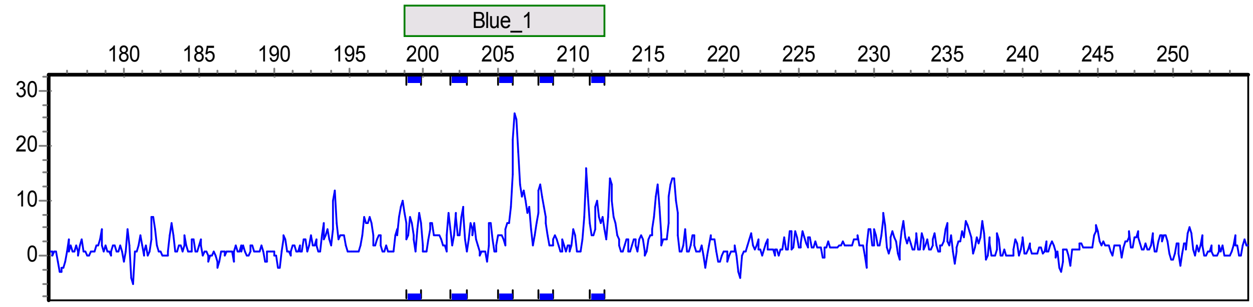

No	Size	Height	Area	Marker	Allele	Difference	Quality	Score	Allele Comments	Sample Comments

Sample 3: Run date and time: 09/15/2020 - 18:52:27 -> 09/15/2020 - 19:38:19

Dye: Blue - 0 peaks - 11.fsa

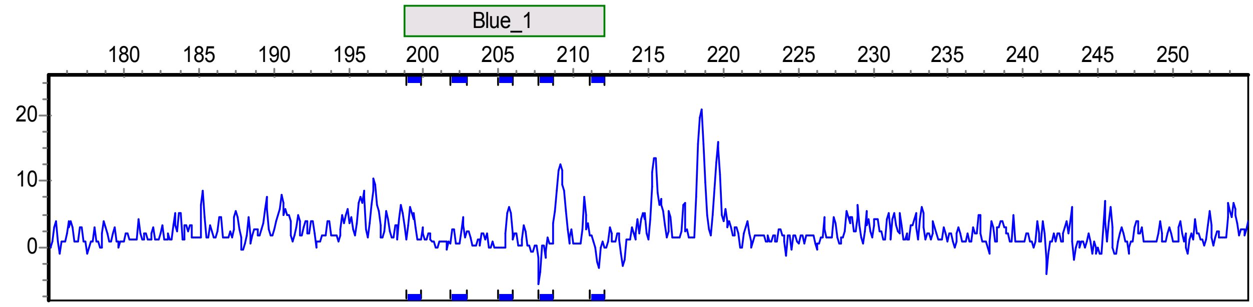

No	Size	Height	Area	Marker	Allele	Difference	Quality	Score	Allele Comments	Sample Comments

Sample 4: Run date and time: 09/15/2020 - 18:52:27 -> 09/15/2020 - 19:38:19

Dye: Blue - 0 peaks - 12.fsa

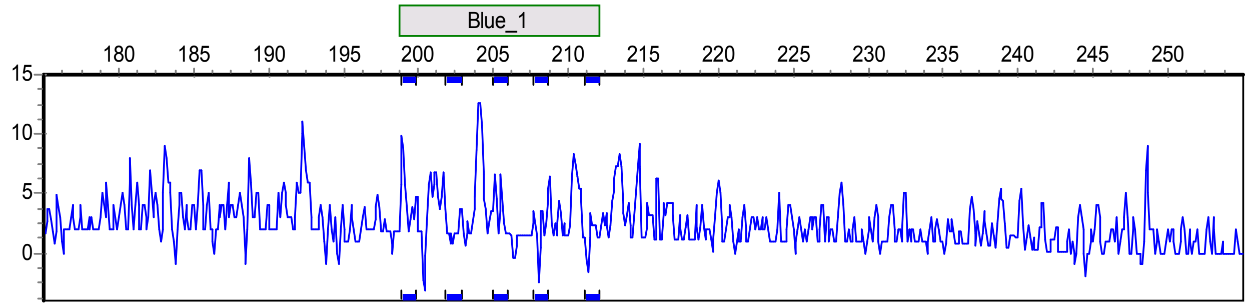

No	Size	Height	Area	Marker	Allele	Difference	Quality	Score	Allele Comments	Sample Comments

Sample 5: Run date and time: 09/15/2020 - 18:52:27 -> 09/15/2020 - 19:38:19

Dye: Blue - 0 peaks - 13.fsa

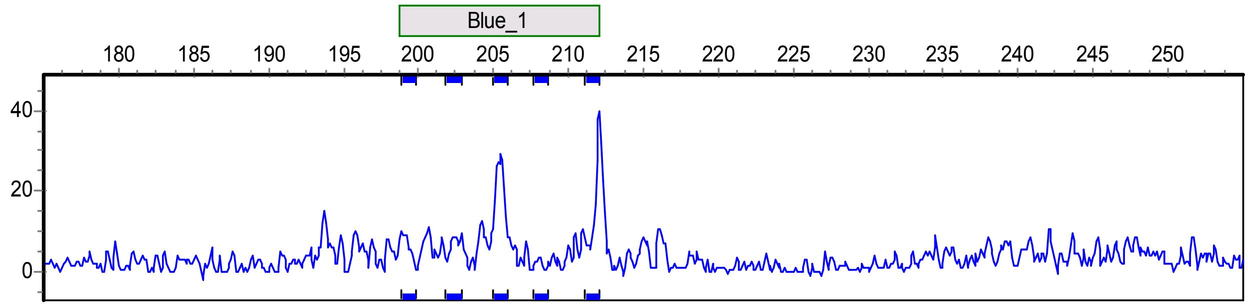

No	Size	Height	Area	Marker	Allele	Difference	Quality	Score	Allele Comments	Sample Comments

Sample 6: Run date and time: 09/15/2020 - 18:52:27 -> 09/15/2020 - 19:38:19

Dye: Blue - 0 peaks - 14.fsa

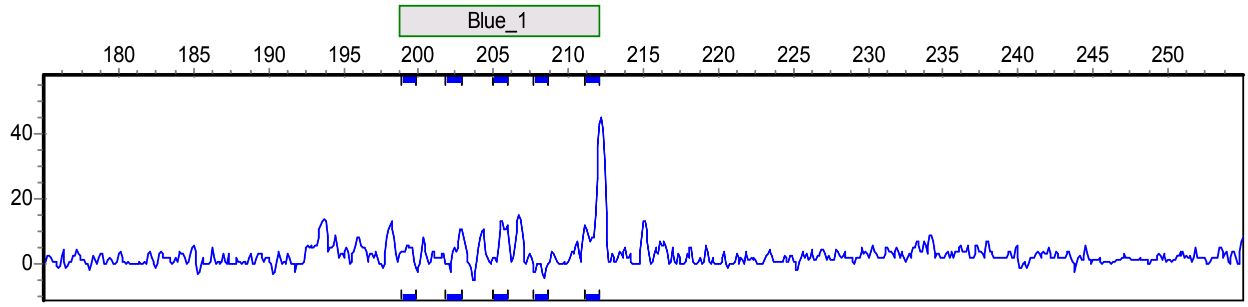

No	Size	Height	Area	Marker	Allele	Difference	Quality	Score	Allele Comments	Sample Comments

Sample 7: Run date and time: 09/15/2020 - 18:52:27 -> 09/15/2020 - 19:38:19

Dye: Blue - 1 peaks - 15.fsa

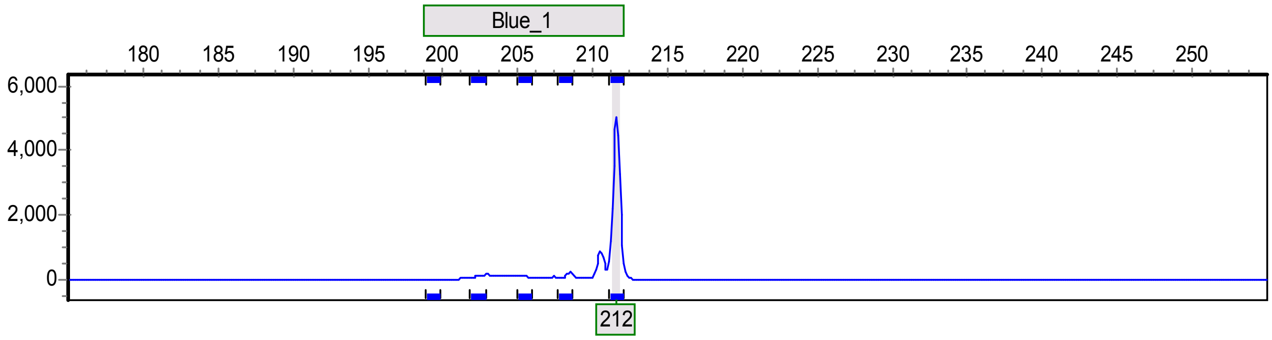

No	Size	Height	Area	Marker	Allele	Difference	Quality	Score	Allele Comments	Sample Comments
1	211.6	4997	26730	Blue_1	212	0.0	Pass	500.0		

Sample 8: Run date and time: 09/15/2020 - 18:52:27 -> 09/15/2020 - 19:38:19

Dye: Blue - 2 peaks - 16.fsa

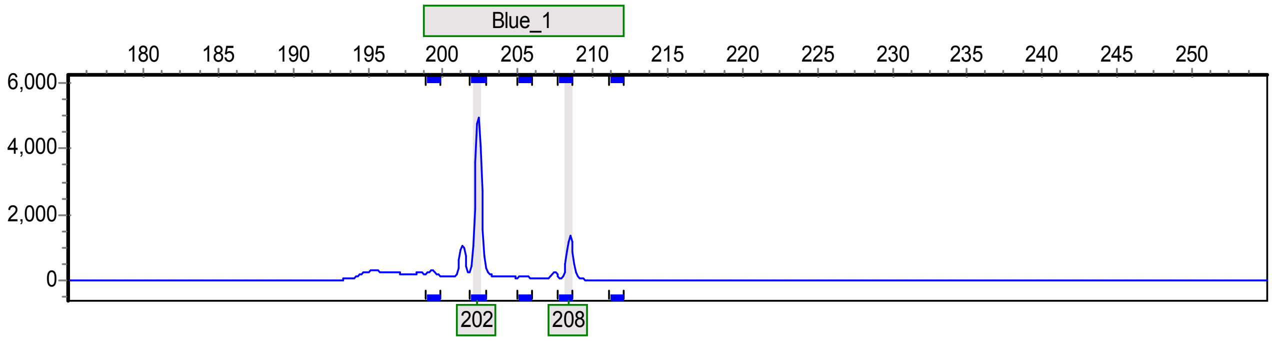

No	Size	Height	Area	Marker	Allele	Difference	Quality	Score	Allele Comments	Sample Comments
1	202.4	4903	24443	Blue_1	202	0.0	Pass	500.0		
2	208.5	1335	6585	Blue_1	208	0.3	Pass	321.6		

Sample 9: Run date and time: 09/15/2020 - 18:52:27 -> 09/15/2020 - 19:38:19

Dye: Blue - 1 peaks - 17.fsa

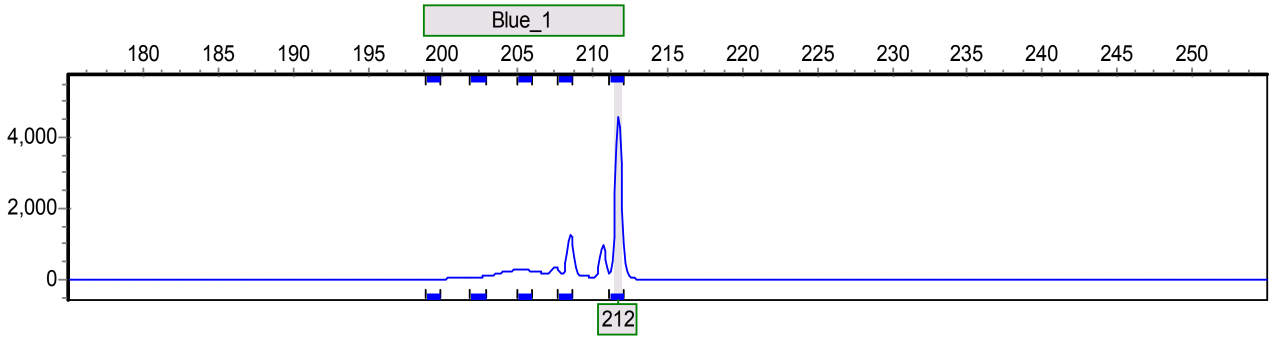

No	Size	Height	Area	Marker	Allele	Difference	Quality	Score	Allele Comments	Sample Comments
1	211.7	4528	22097	Blue_1	212	0.1	Pass	500.0		

Sample 10: Run date and time: 09/15/2020 - 18:52:27 -> 09/15/2020 - 19:38:19

Dye: Blue - 0 peaks - 18.fsa

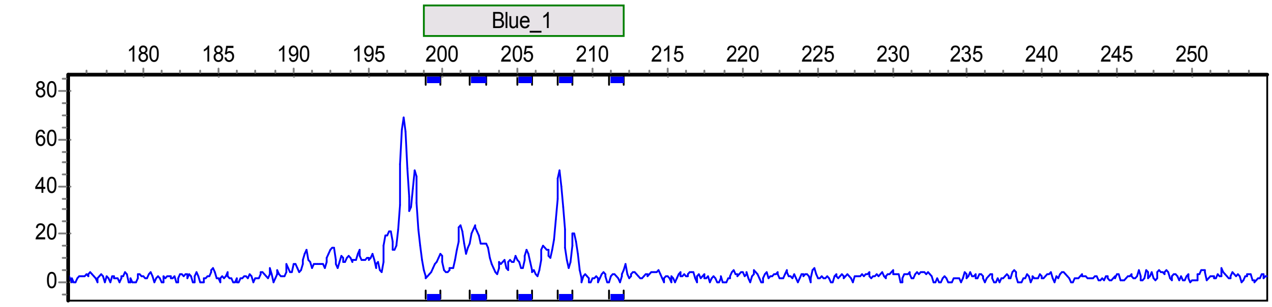

No	Size	Height	Area	Marker	Allele	Difference	Quality	Score	Allele Comments	Sample Comments

Sample 11: Run date and time: 09/15/2020 - 18:52:27 -> 09/15/2020 - 19:38:19

Dye: Blue - 1 peaks - 19.fsa

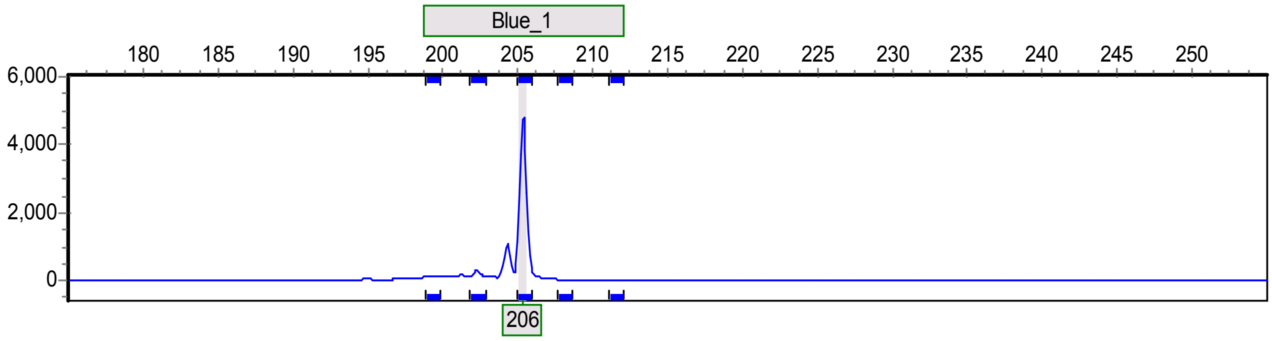

No	Size	Height	Area	Marker	Allele	Difference	Quality	Score	Allele Comments	Sample Comments
1	205.4	4755	24303	Blue_1	206	0.1	Pass	500.0		

Sample 12: Run date and time: 09/15/2020 - 18:52:27 -> 09/15/2020 - 19:38:19

Dye: Blue - 1 peaks - 2.fsa

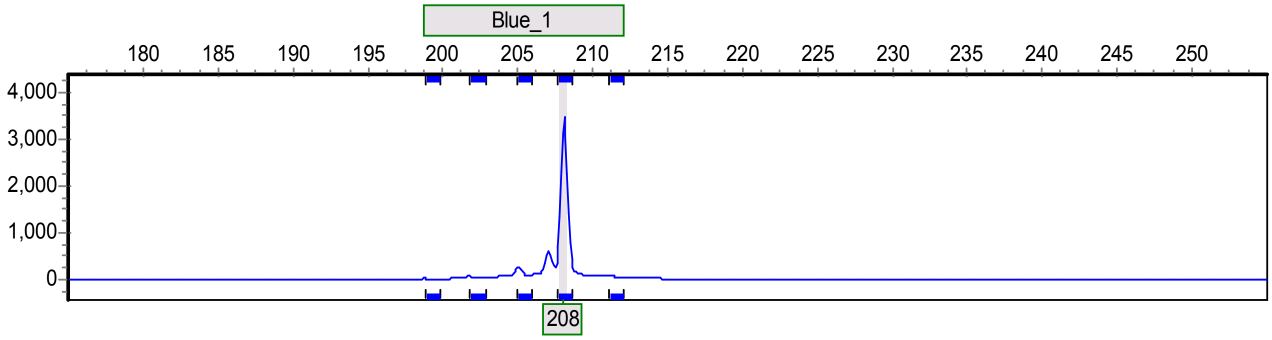

No	Size	Height	Area	Marker	Allele	Difference	Quality	Score	Allele Comments	Sample Comments
1	208.1	3465	18271	Blue_1	208	0.1	Pass	500.0		

Sample 13: Run date and time: 09/15/2020 - 18:52:27 -> 09/15/2020 - 19:38:19

Dye: Blue - 1 peaks - 20.fsa

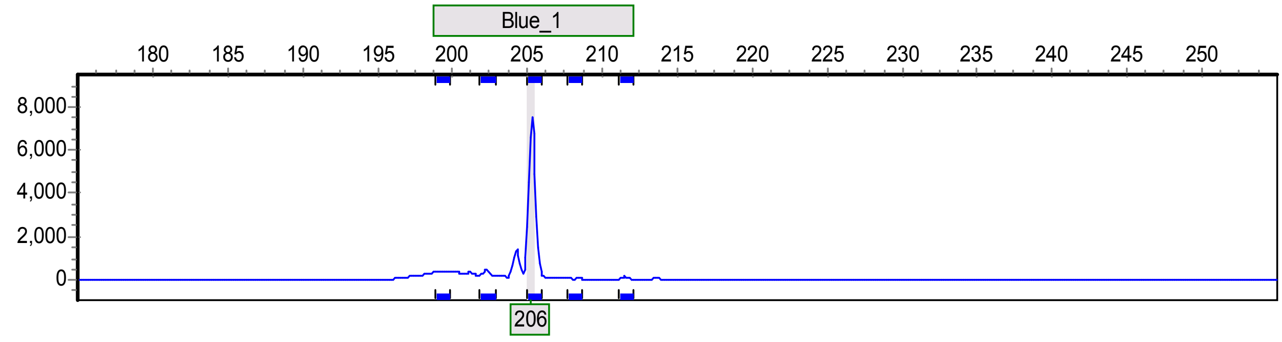

No	Size	Height	Area	Marker	Allele	Difference	Quality	Score	Allele Comments	Sample Comments
1	205.3	7473	36410	Blue_1	206	0.2	Pass	500.0		

Sample 14: Run date and time: 09/15/2020 - 18:52:27 -> 09/15/2020 - 19:38:19

Dye: Blue - 2 peaks - 21.fsa

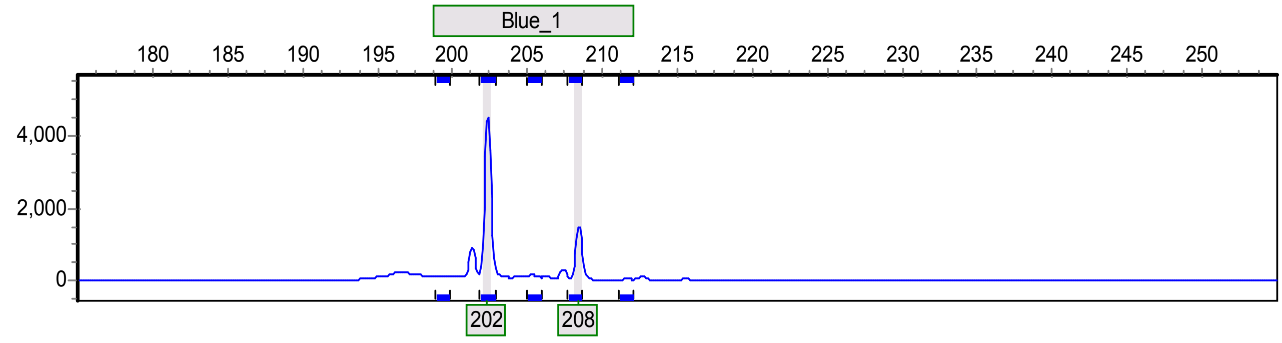

No	Size	Height	Area	Marker	Allele	Difference	Quality	Score	Allele Comments	Sample Comments
1	202.4	4484	22114	Blue_1	202	0.0	Pass	500.0		
2	208.4	1481	7396	Blue_1	208	0.2	Pass	369.5		

Sample 15: Run date and time: 09/15/2020 - 18:52:27 -> 09/15/2020 - 19:38:19

Dye: Blue - 1 peaks - 22.fsa

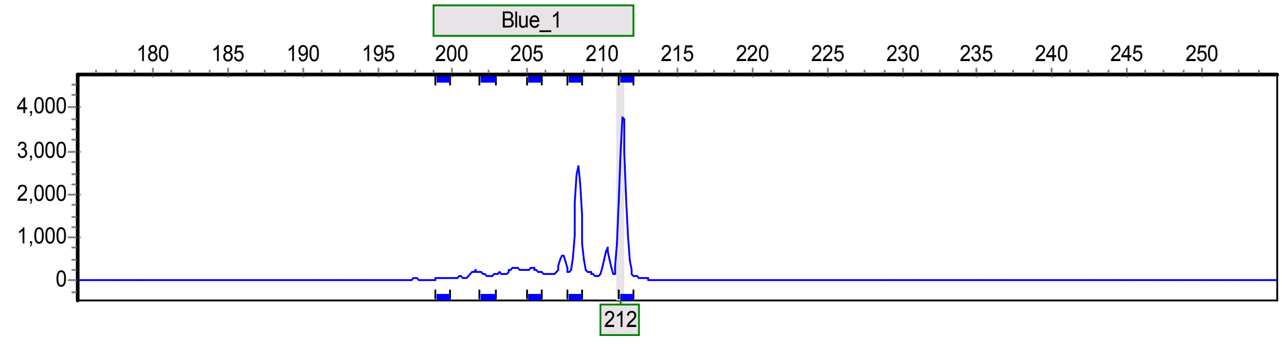

No	Size	Height	Area	Marker	Allele	Difference	Quality	Score	Allele Comments	Sample Comments
1	211.3	3757	18734	Blue_1	212	0.3	Pass	500.0		

Allele Report

Sample 16: Run date and time: 09/15/2020 - 18:52:27 -> 09/15/2020 - 19:38:19

Dye: Blue - 1 peaks - 23.fsa

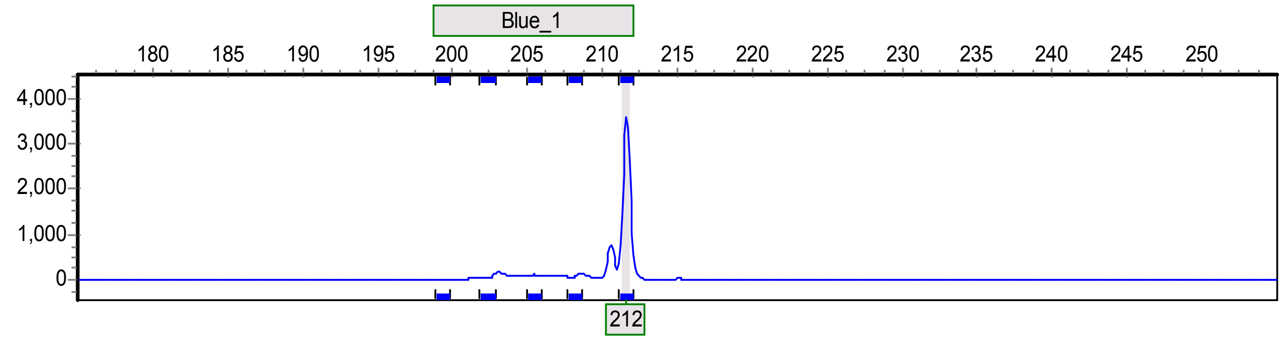

No	Size	Height	Area	Marker	Allele	Difference	Quality	Score	Allele Comments	Sample Comments
1	211.6	3559	19778	Blue_1	212	0.0	Pass	500.0		

Sample 17: Run date and time: 09/15/2020 - 18:52:27 -> 09/15/2020 - 19:38:19

Dye: Blue - 0 peaks - 24.fsa

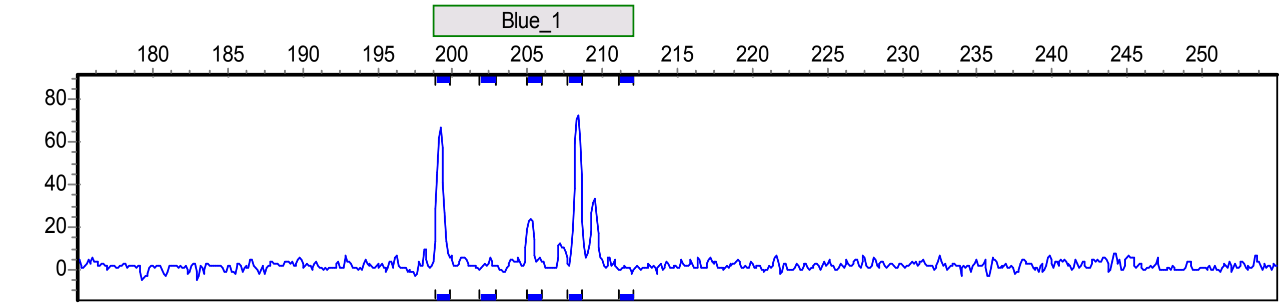

No	Size	Height	Area	Marker	Allele	Difference	Quality	Score	Allele Comments	Sample Comments

Sample 18: Run date and time: 09/15/2020 - 18:52:27 -> 09/15/2020 - 19:38:19

Dye: Blue - 0 peaks - 25.fsa

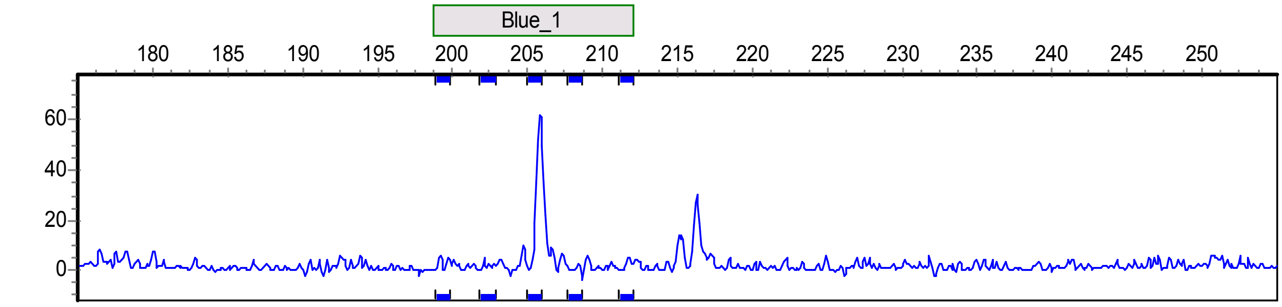

No	Size	Height	Area	Marker	Allele	Difference	Quality	Score	Allele Comments	Sample Comments

Allele Report

Sample 19: Run date and time: 09/15/2020 - 18:52:27 -> 09/15/2020 - 19:38:19

Dye: Blue - 2 peaks - 26.fsa

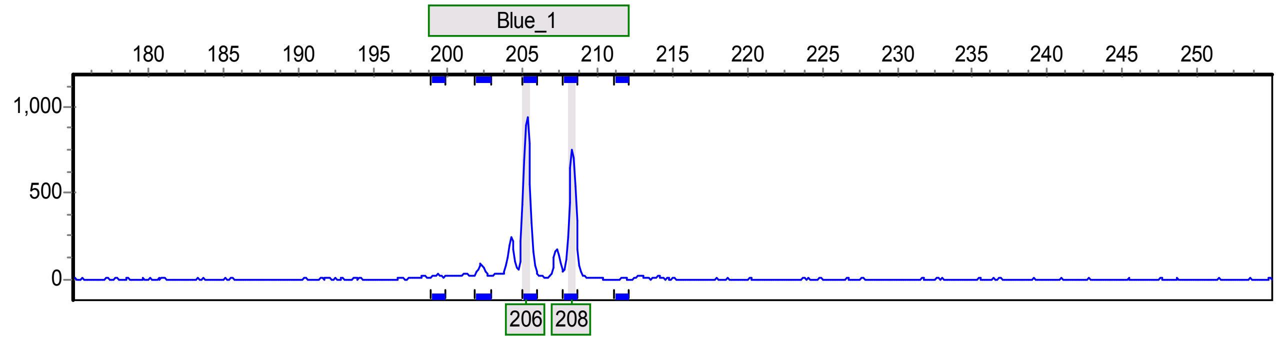

No	Size	Height	Area	Marker	Allele	Difference	Quality	Score	Allele Comments	Sample Comments
1	205.3	931	4832	Blue_1	206	0.2	Pass	178.7		
2	208.3	748	3811	Blue_1	208	0.1	Pass	133.3		

Sample 20: Run date and time: 09/15/2020 - 18:52:27 -> 09/15/2020 - 19:38:19

Dye: Blue - 1 peaks - 27.fsa

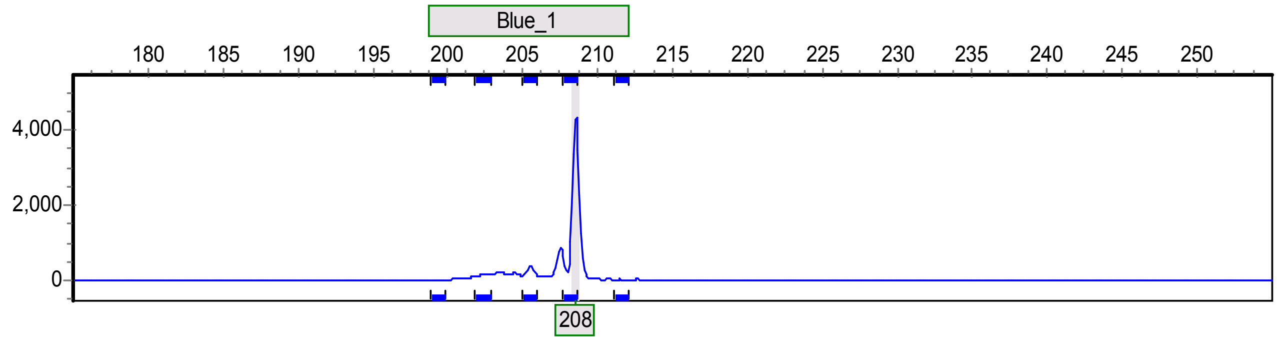

No	Size	Height	Area	Marker	Allele	Difference	Quality	Score	Allele Comments	Sample Comments
1	208.6	4302	21779	Blue_1	208	0.4	Pass	500.0		

Sample 21: Run date and time: 09/15/2020 - 18:52:27 -> 09/15/2020 - 19:38:19

Dye: Blue - 2 peaks - 28.fsa

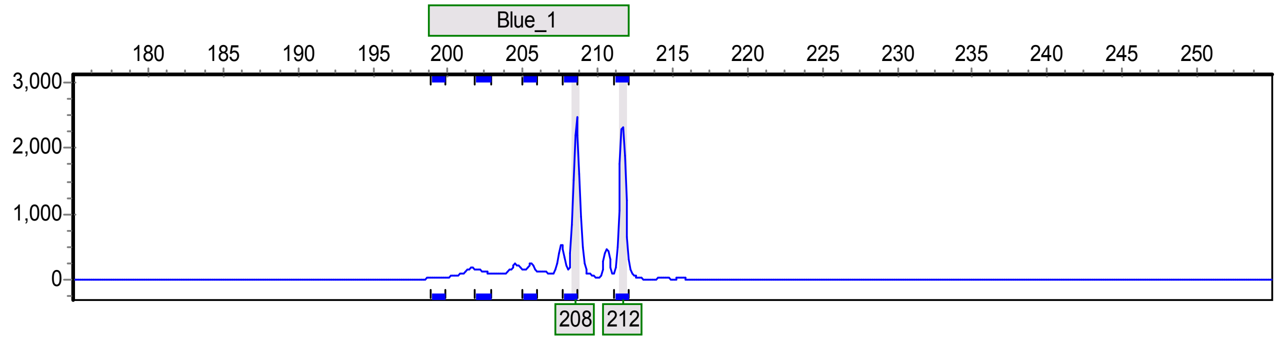

No	Size	Height	Area	Marker	Allele	Difference	Quality	Score	Allele Comments	Sample Comments
1	208.6	2458	12093	Blue_1	208	0.4	Pass	500.0		
2	211.7	2325	11368	Blue_1	212	0.1	Pass	500.0		

Sample 22: Run date and time: 09/15/2020 - 18:52:27 -> 09/15/2020 - 19:38:19

Dye: Blue - 2 peaks - 29.fsa

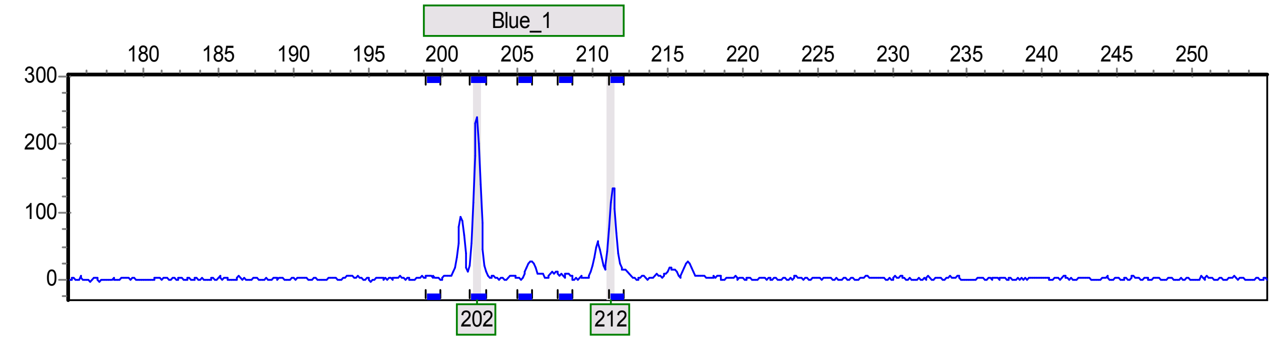

No	Size	Height	Area	Marker	Allele	Difference	Quality	Score	Allele Comments	Sample Comments
1	202.3	239	1259	Blue_1	202	0.1	Pass	19.9		
2	211.3	135	728	Blue_1	212	0.3	Pass	7.1		

Sample 23: Run date and time: 09/15/2020 - 18:52:27 -> 09/15/2020 - 19:38:19

Dye: Blue - 1 peaks - 3.fsa

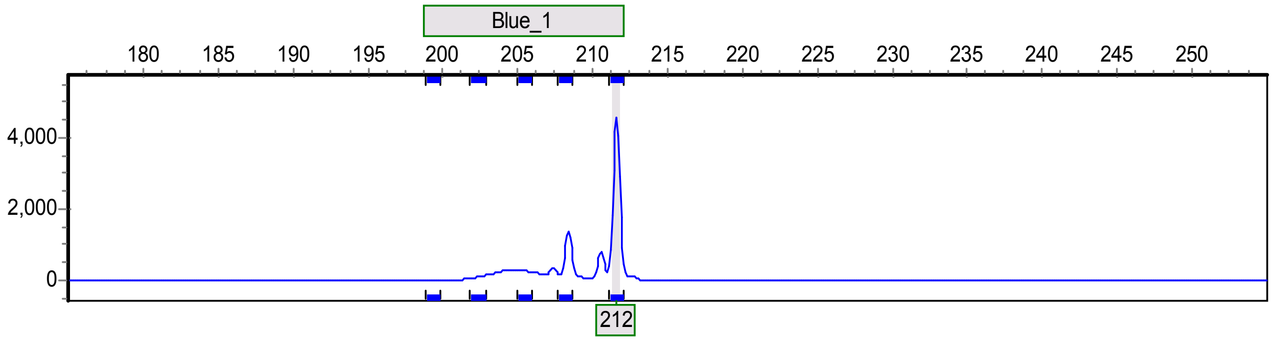

No	Size	Height	Area	Marker	Allele	Difference	Quality	Score	Allele Comments	Sample Comments
1	211.6	4536	23191	Blue_1	212	0.0	Pass	500.0		

Sample 24: Run date and time: 09/15/2020 - 18:52:27 -> 09/15/2020 - 19:38:19

Dye: Blue - 2 peaks - 30.fsa

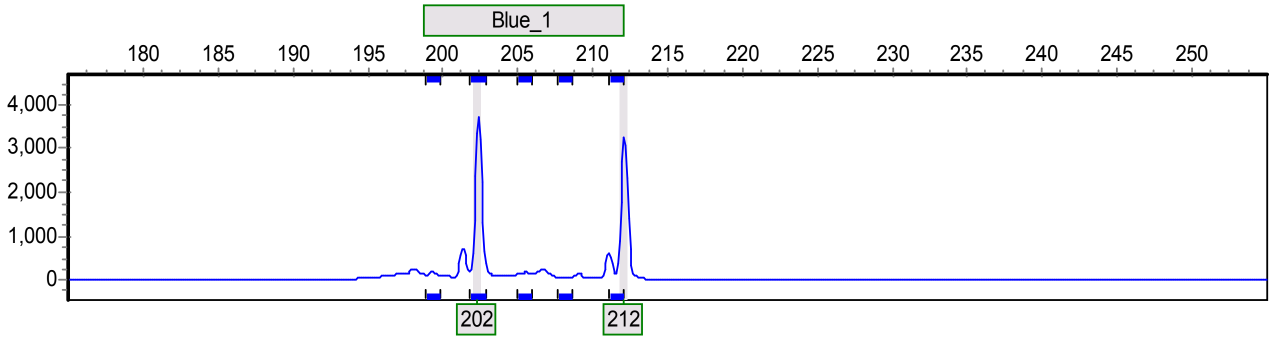

No	Size	Height	Area	Marker	Allele	Difference	Quality	Score	Allele Comments	Sample Comments
1	202.4	3698	17952	Blue_1	202	0.0	Pass	500.0		
2	212.1	3252	15730	Blue_1	212	0.5	Pass	500.0		

Allele Report

Sample 25: Run date and time: 09/15/2020 - 18:52:27 -> 09/15/2020 - 19:38:19

Dye: Blue - 0 peaks - 31.fsa

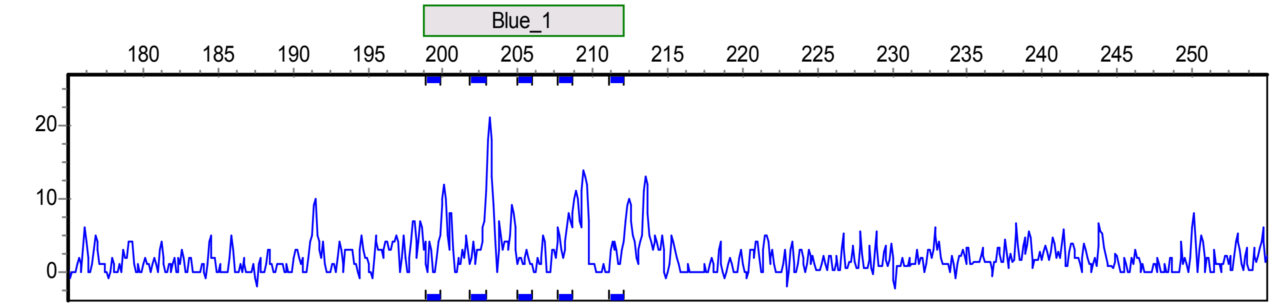

No	Size	Height	Area	Marker	Allele	Difference	Quality	Score	Allele Comments	Sample Comments

Sample 26: Run date and time: 09/15/2020 - 18:52:27 -> 09/15/2020 - 19:38:19

Dye: Blue - 0 peaks - 32.fsa

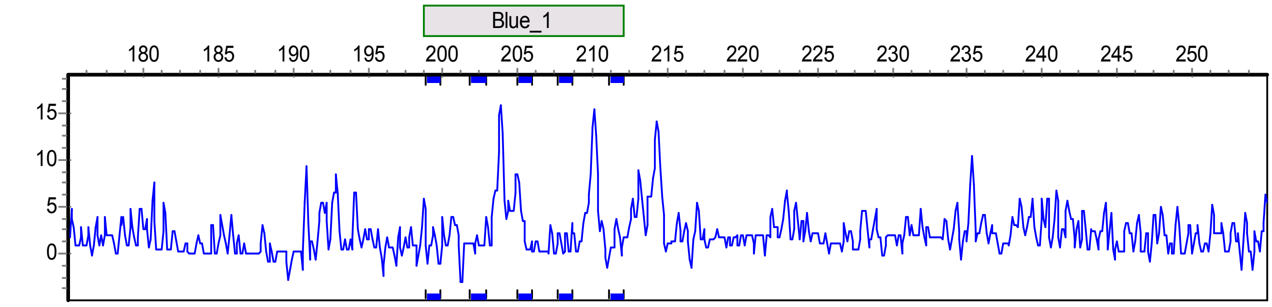

No	Size	Height	Area	Marker	Allele	Difference	Quality	Score	Allele Comments	Sample Comments

Sample 27: Run date and time: 09/15/2020 - 18:52:27 -> 09/15/2020 - 19:38:19

Dye: Blue - 1 peaks - 33.fsa

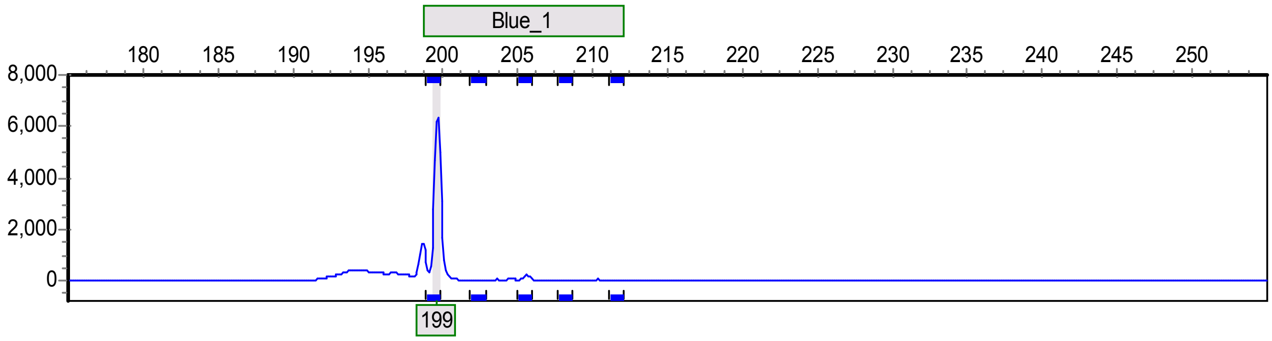

No	Size	Height	Area	Marker	Allele	Difference	Quality	Score	Allele Comments	Sample Comments
1	199.7	6312	30258	Blue_1	199	0.3	Pass	500.0		

Sample 28: Run date and time: 09/15/2020 - 18:52:27 -> 09/15/2020 - 19:38:19

Dye: Blue - 2 peaks - 34.fsa

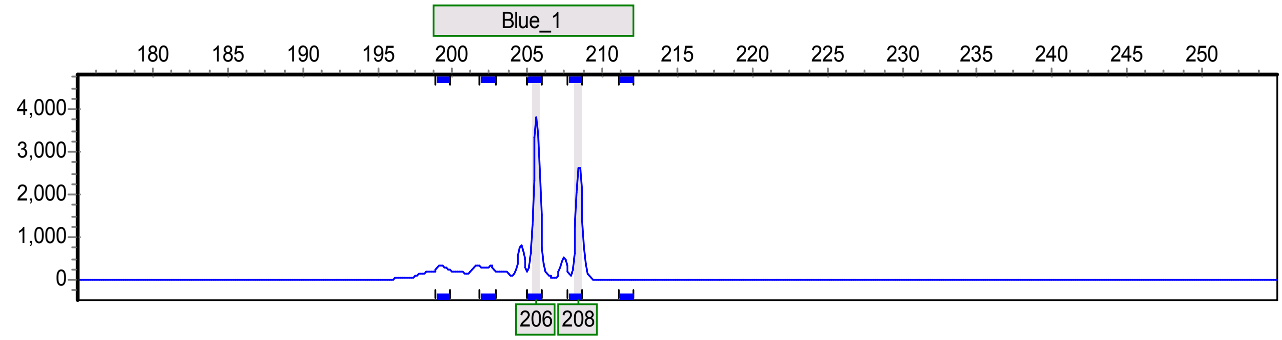

No	Size	Height	Area	Marker	Allele	Difference	Quality	Score	Allele Comments	Sample Comments
1	205.6	3771	18691	Blue_1	206	0.1	Pass	500.0		
2	208.5	2629	13136	Blue_1	208	0.3	Pass	500.0		

Sample 29: Run date and time: 09/15/2020 - 18:52:27 -> 09/15/2020 - 19:38:19

Dye: Blue - 1 peaks - 35.fsa

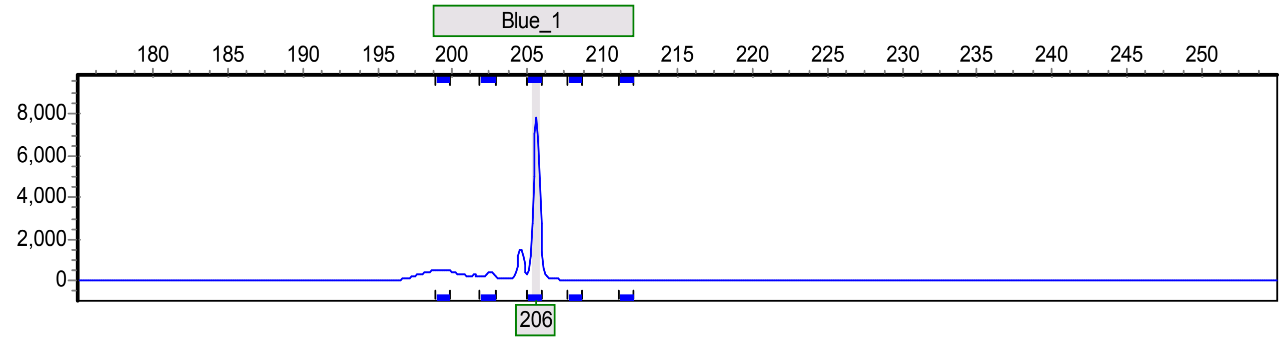

No	Size	Height	Area	Marker	Allele	Difference	Quality	Score	Allele Comments	Sample Comments
1	205.6	7797	37677	Blue_1	206	0.1	Pass	500.0		

Sample 30: Run date and time: 09/15/2020 - 18:52:27 -> 09/15/2020 - 19:38:19

Dye: Blue - 2 peaks - 36.fsa

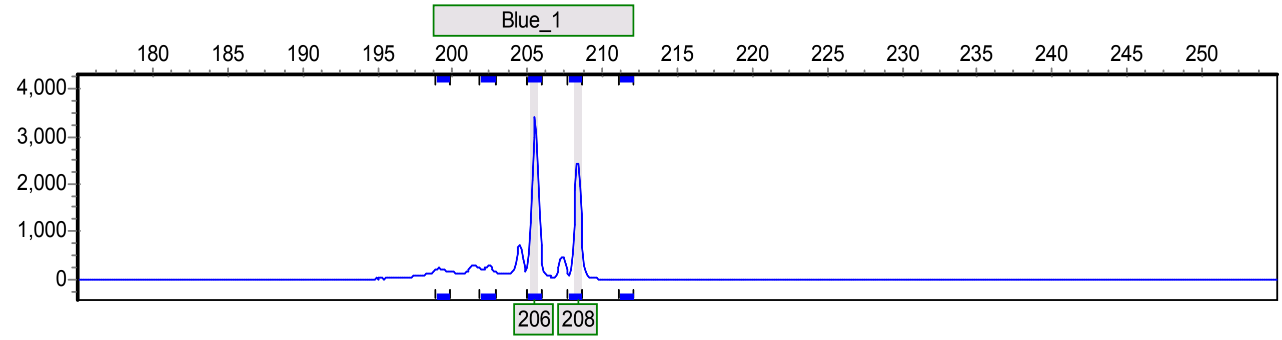

No	Size	Height	Area	Marker	Allele	Difference	Quality	Score	Allele Comments	Sample Comments
1	205.5	3384	16858	Blue_1	206	0.0	Pass	500.0		
2	208.4	2425	12052	Blue_1	208	0.2	Pass	500.0		

Sample 31: Run date and time: 09/15/2020 - 18:52:27 -> 09/15/2020 - 19:38:19

Dye: Blue - 1 peaks - 37.fsa

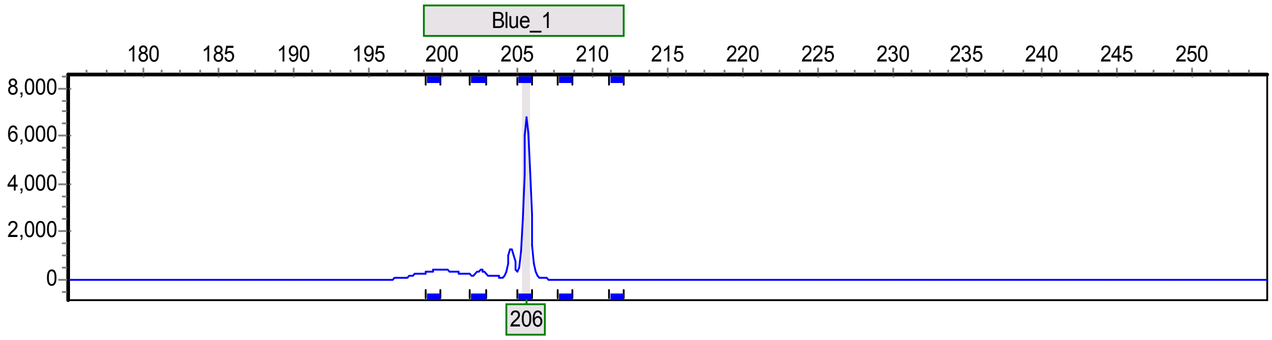

No	Size	Height	Area	Marker	Allele	Difference	Quality	Score	Allele Comments	Sample Comments
1	205.6	6734	34216	Blue_1	206	0.1	Pass	500.0		

Sample 32: Run date and time: 09/15/2020 - 18:52:27 -> 09/15/2020 - 19:38:19

Dye: Blue - 1 peaks - 38.fsa

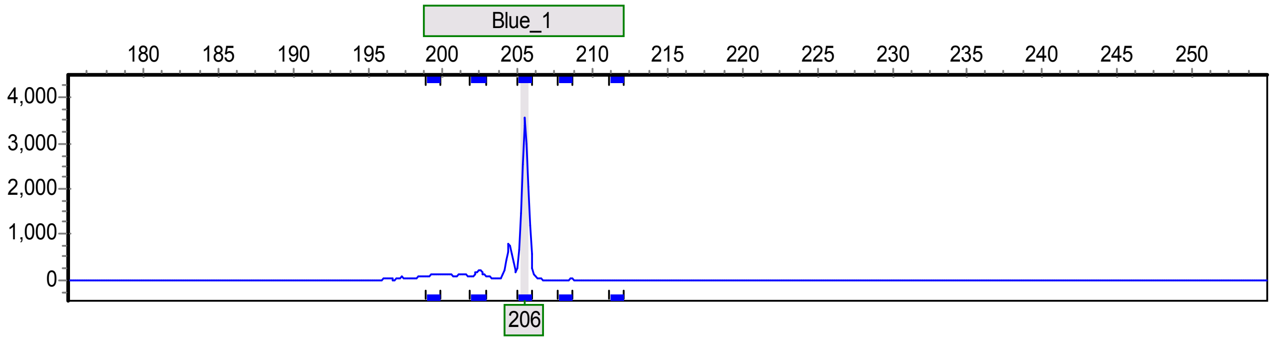

No	Size	Height	Area	Marker	Allele	Difference	Quality	Score	Allele Comments	Sample Comments
1	205.5	3540	17637	Blue_1	206	0.0	Pass	500.0		

Sample 33: Run date and time: 09/15/2020 - 18:52:27 -> 09/15/2020 - 19:38:19

Dye: Blue - 2 peaks - 39.fsa

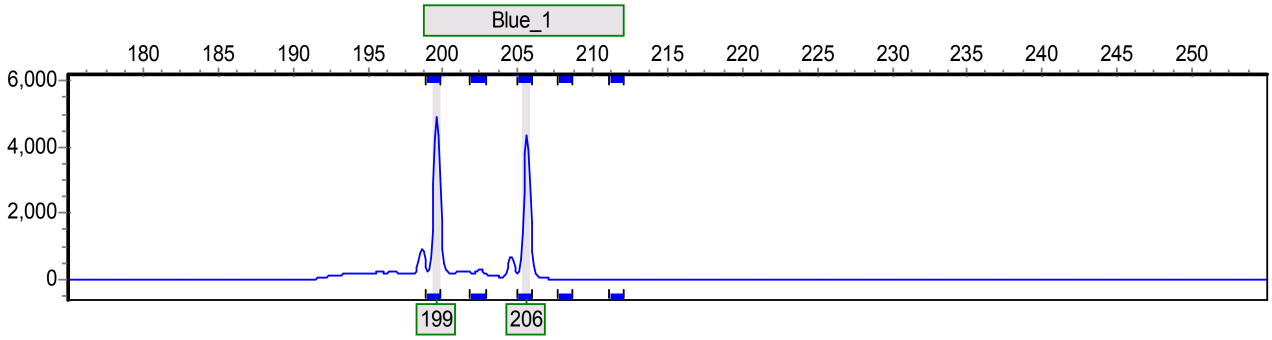

No	Size	Height	Area	Marker	Allele	Difference	Quality	Score	Allele Comments	Sample Comments
1	199.6	4892	23209	Blue_1	199	0.2	Pass	500.0		
2	205.6	4360	21397	Blue_1	206	0.1	Pass	500.0		

Sample 34: Run date and time: 09/15/2020 - 18:52:27 -> 09/15/2020 - 19:38:19

Dye: Blue - 1 peaks - 4.fsa

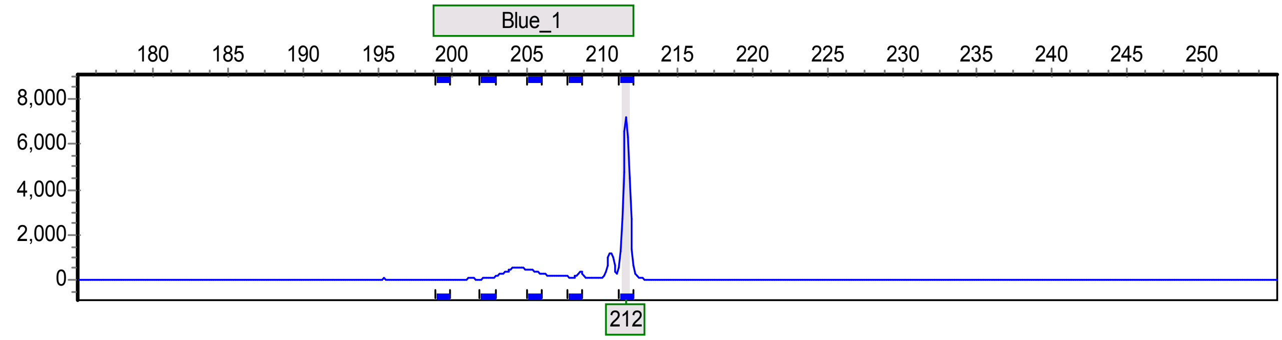

No	Size	Height	Area	Marker	Allele	Difference	Quality	Score	Allele Comments	Sample Comments
1	211.6	7171	35703	Blue_1	212	0.0	Pass	500.0		

Sample 35: Run date and time: 09/15/2020 - 18:52:27 -> 09/15/2020 - 19:38:19

Dye: Blue - 1 peaks - 40.fsa

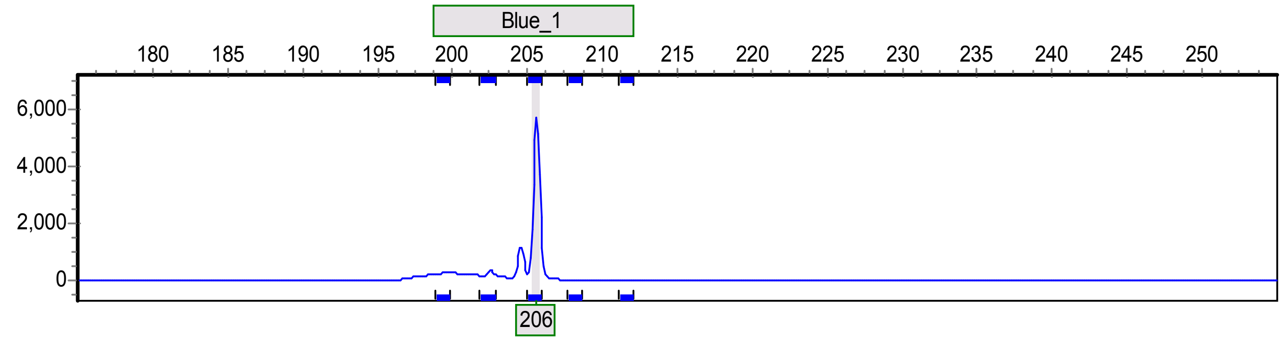

No	Size	Height	Area	Marker	Allele	Difference	Quality	Score	Allele Comments	Sample Comments
1	205.6	5675	27378	Blue_1	206	0.1	Pass	500.0		

Sample 36: Run date and time: 09/15/2020 - 18:52:27 -> 09/15/2020 - 19:38:19

Dye: Blue - 1 peaks - 41.fsa

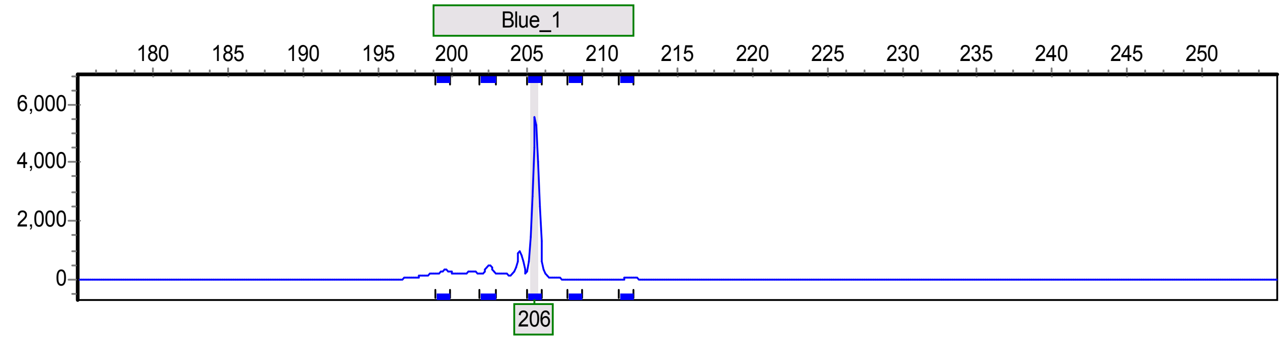

No	Size	Height	Area	Marker	Allele	Difference	Quality	Score	Allele Comments	Sample Comments
1	205.5	5523	27193	Blue_1	206	0.0	Pass	500.0		

Sample 37: Run date and time: 09/15/2020 - 18:52:27 -> 09/15/2020 - 19:38:19

Dye: Blue - 1 peaks - 42.fsa

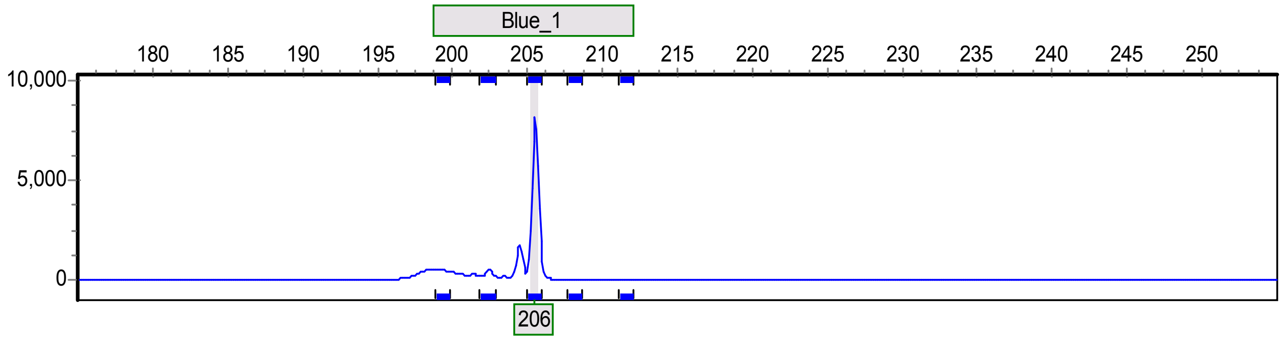

No	Size	Height	Area	Marker	Allele	Difference	Quality	Score	Allele Comments	Sample Comments
1	205.5	8107	40359	Blue_1	206	0.0	Pass	500.0		

Sample 38: Run date and time: 09/15/2020 - 18:52:27 -> 09/15/2020 - 19:38:19

Dye: Blue - 1 peaks - 43.fsa

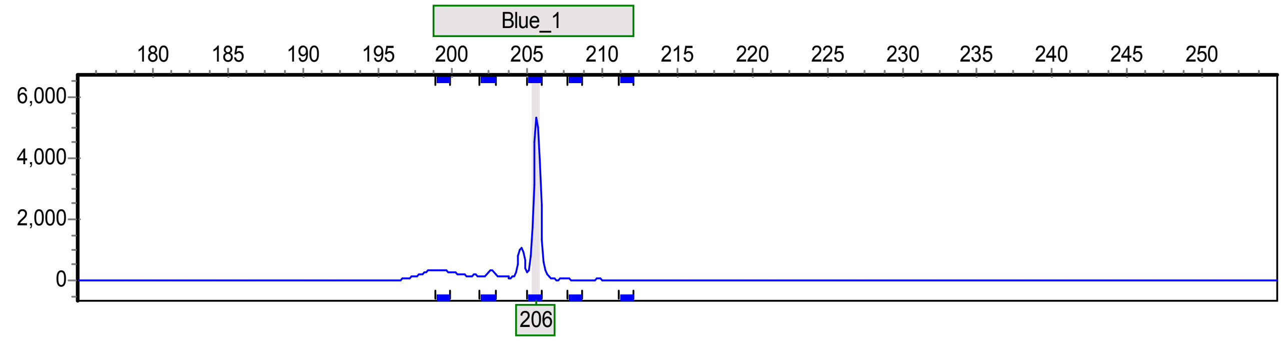

No	Size	Height	Area	Marker	Allele	Difference	Quality	Score	Allele Comments	Sample Comments
1	205.6	5314	27250	Blue_1	206	0.1	Pass	500.0		

Sample 39: Run date and time: 09/15/2020 - 18:52:27 -> 09/15/2020 - 19:38:19

Dye: Blue - 1 peaks - 44.fsa

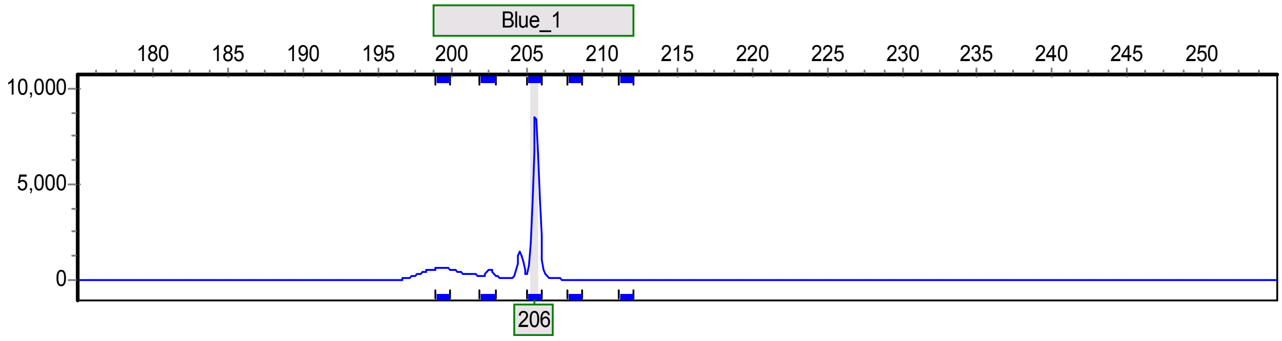

No	Size	Height	Area	Marker	Allele	Difference	Quality	Score	Allele Comments	Sample Comments
1	205.5	8449	41938	Blue_1	206	0.0	Pass	500.0		

Sample 40: Run date and time: 09/15/2020 - 18:52:27 -> 09/15/2020 - 19:38:19

Dye: Blue - 1 peaks - 45.fsa

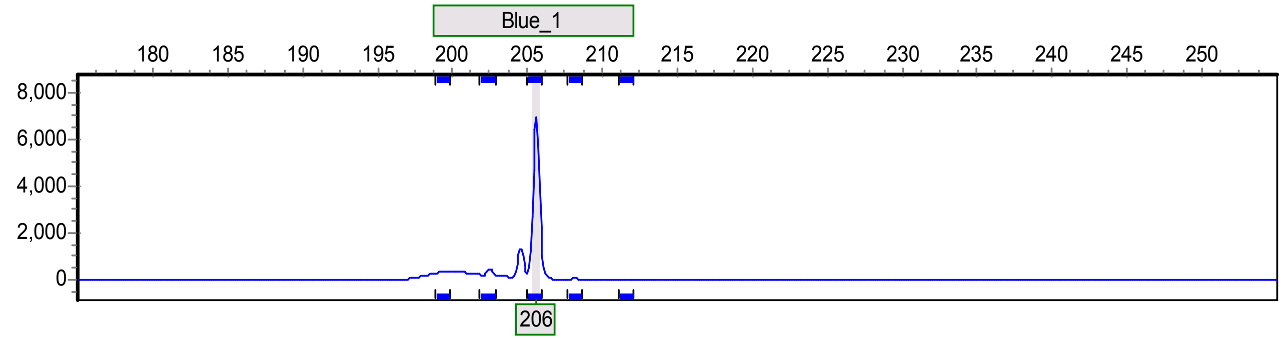

No	Size	Height	Area	Marker	Allele	Difference	Quality	Score	Allele Comments	Sample Comments
1	205.6	6921	33577	Blue_1	206	0.1	Pass	500.0		

Sample 41: Run date and time: 09/15/2020 - 18:52:27 -> 09/15/2020 - 19:38:19

Dye: Blue - 1 peaks - 46.fsa

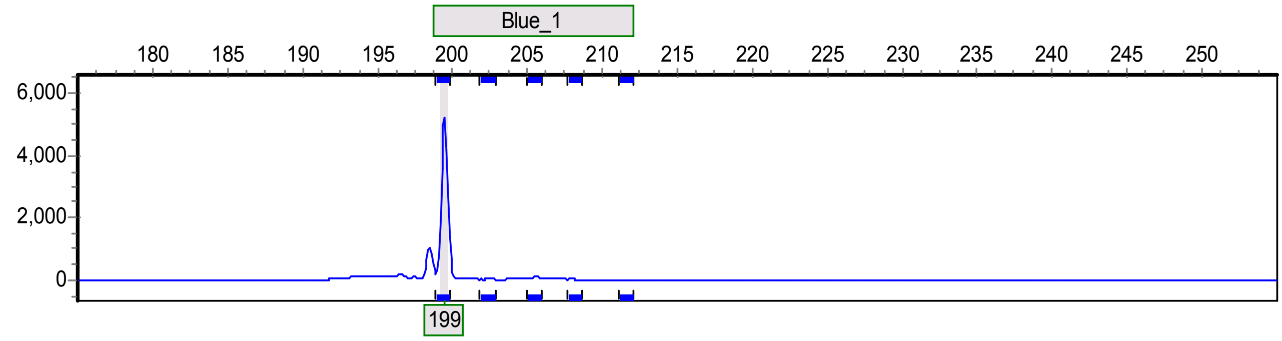

No	Size	Height	Area	Marker	Allele	Difference	Quality	Score	Allele Comments	Sample Comments
1	199.5	5181	23964	Blue_1	199	0.1	Pass	500.0		

Sample 42: Run date and time: 09/15/2020 - 18:52:27 -> 09/15/2020 - 19:38:19

Dye: Blue - 1 peaks - 47.fsa

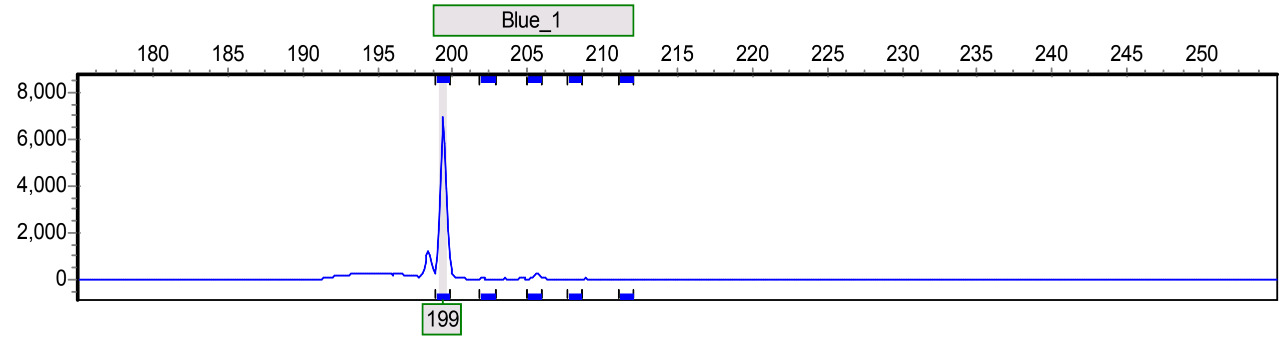

No	Size	Height	Area	Marker	Allele	Difference	Quality	Score	Allele Comments	Sample Comments
1	199.4	6943	32120	Blue_1	199	0.0	Pass	500.0		

Allele Report

Sample 43: Run date and time: 09/15/2020 - 18:52:27 -> 09/15/2020 - 19:38:19

Dye: Blue - 1 peaks - 48.fsa

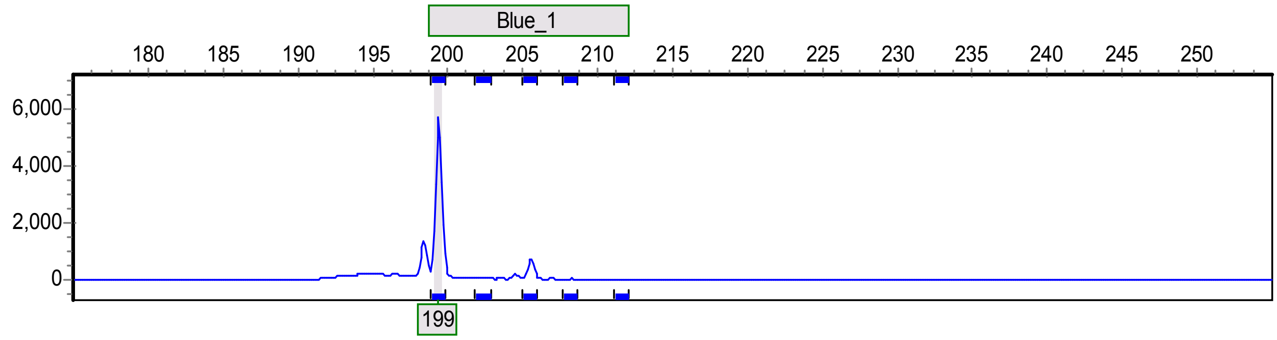

No	Size	Height	Area	Marker	Allele	Difference	Quality	Score	Allele Comments	Sample Comments
1	199.4	5709	26579	Blue_1	199	0.0	Pass	500.0		

Sample 44: Run date and time: 09/15/2020 - 18:52:27 -> 09/15/2020 - 19:38:19

Dye: Blue - 1 peaks - 49.fsa

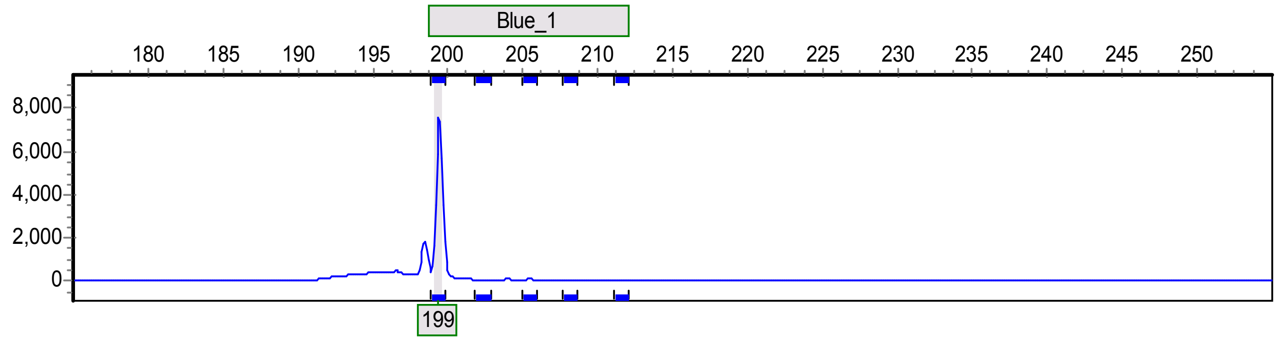

No	Size	Height	Area	Marker	Allele	Difference	Quality	Score	Allele Comments	Sample Comments
1	199.4	7515	35996	Blue_1	199	0.0	Pass	500.0		

Sample 45: Run date and time: 09/15/2020 - 18:52:27 -> 09/15/2020 - 19:38:19

Dye: Blue - 3 peaks - 5.fsa

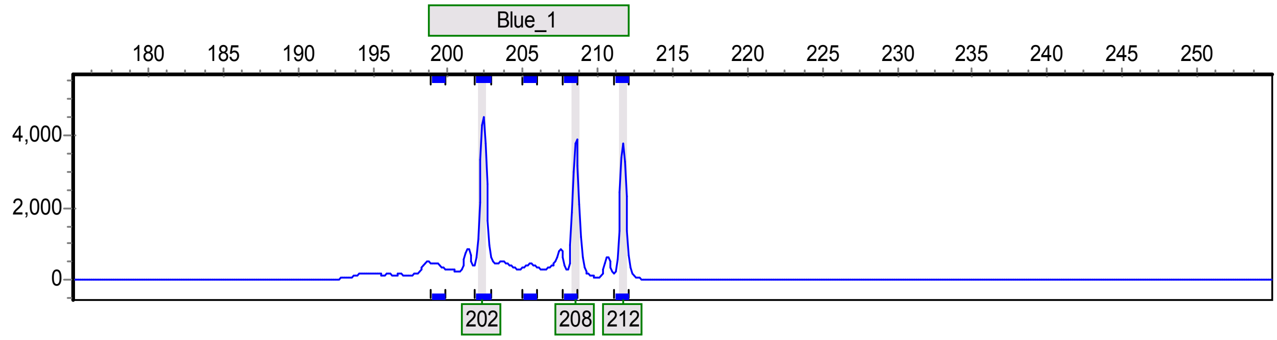

No	Size	Height	Area	Marker	Allele	Difference	Quality	Score	Allele Comments	Sample Comments
1	202.4	4497	23309	Blue_1	202	0.0	Pass	500.0		
2	208.6	3890	20002	Blue_1	208	0.4	Pass	500.0		
3	211.7	3763	18351	Blue_1	212	0.1	Pass	500.0		

Sample 46: Run date and time: 09/15/2020 - 18:52:27 -> 09/15/2020 - 19:38:19

Dye: Blue - 1 peaks - 50.fsa

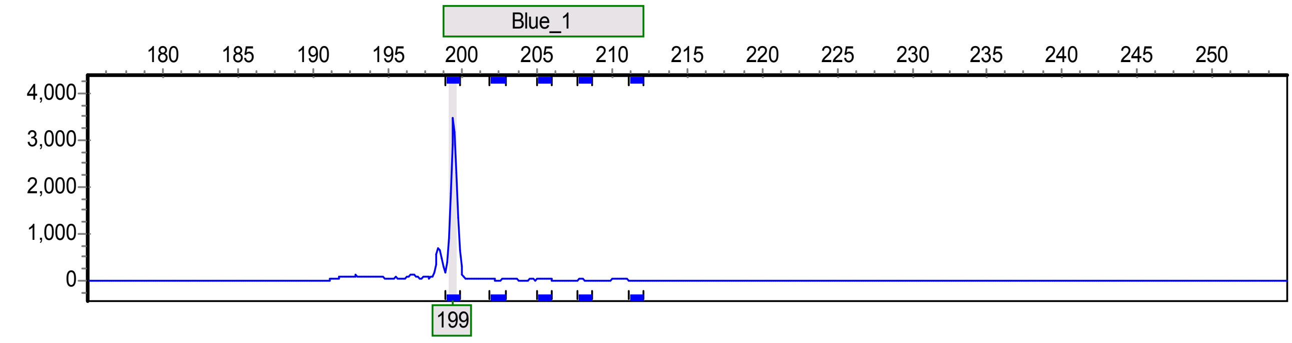

No	Size	Height	Area	Marker	Allele	Difference	Quality	Score	Allele Comments	Sample Comments
1	199.4	3475	16229	Blue_1	199	0.0	Pass	500.0		

Sample 47: Run date and time: 09/15/2020 - 18:52:27 -> 09/15/2020 - 19:38:19

Dye: Blue - 1 peaks - 51.fsa

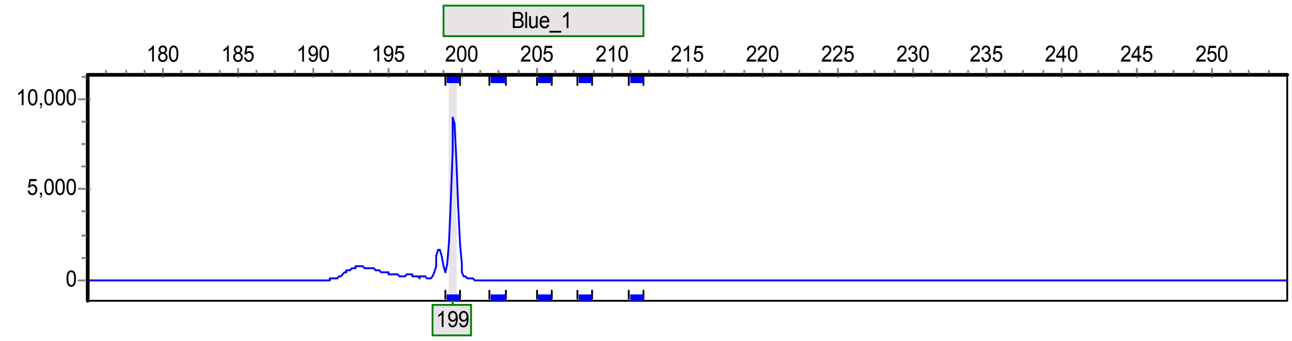

No	Size	Height	Area	Marker	Allele	Difference	Quality	Score	Allele Comments	Sample Comments
1	199.4	8891	42864	Blue_1	199	0.0	Pass	500.0		

Sample 48: Run date and time: 09/15/2020 - 18:52:27 -> 09/15/2020 - 19:38:19

Dye: Blue - 1 peaks - 52.fsa

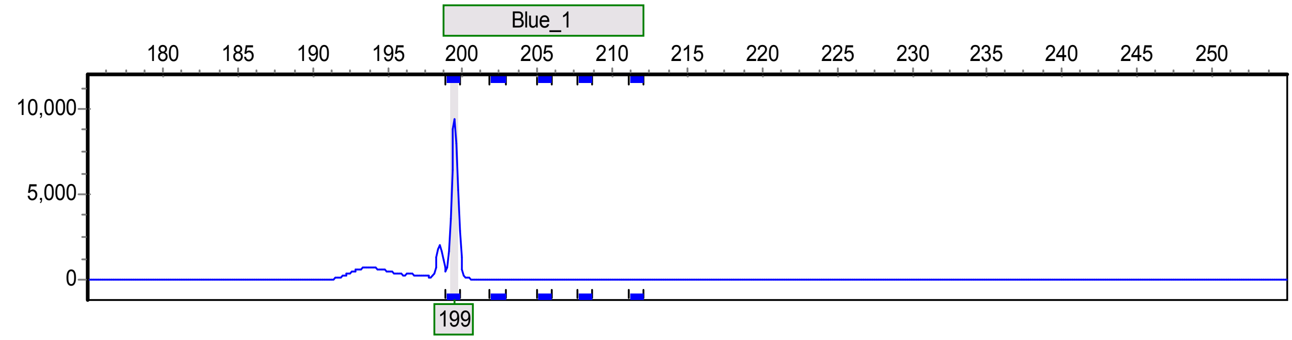

No	Size	Height	Area	Marker	Allele	Difference	Quality	Score	Allele Comments	Sample Comments
1	199.5	9412	44881	Blue_1	199	0.1	Pass	500.0		

Sample 49: Run date and time: 09/15/2020 - 18:52:27 -> 09/15/2020 - 19:38:19

Dye: Blue - 0 peaks - 53.fsa

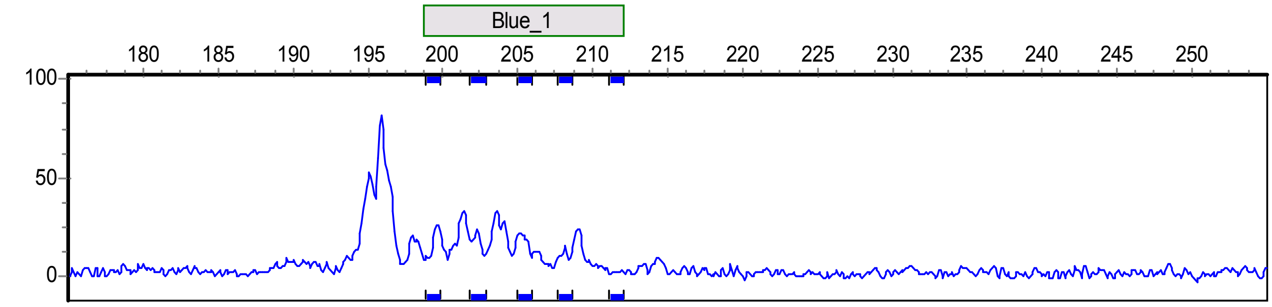

No	Size	Height	Area	Marker	Allele	Difference	Quality	Score	Allele Comments	Sample Comments

Sample 50: Run date and time: 09/15/2020 - 18:52:27 -> 09/15/2020 - 19:38:19

Dye: Blue - 2 peaks - 54.fsa

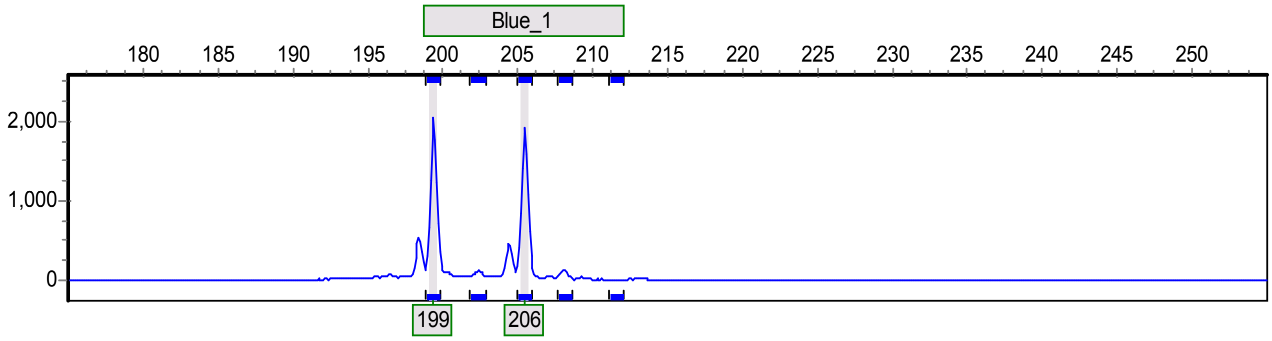

No	Size	Height	Area	Marker	Allele	Difference	Quality	Score	Allele Comments	Sample Comments
1	199.4	2034	9674	Blue_1	199	0.0	Pass	500.0		
2	205.5	1910	9587	Blue_1	206	0.0	Pass	500.0		

Sample 51: Run date and time: 09/15/2020 - 18:52:27 -> 09/15/2020 - 19:38:19

Dye: Blue - 1 peaks - 55.fsa

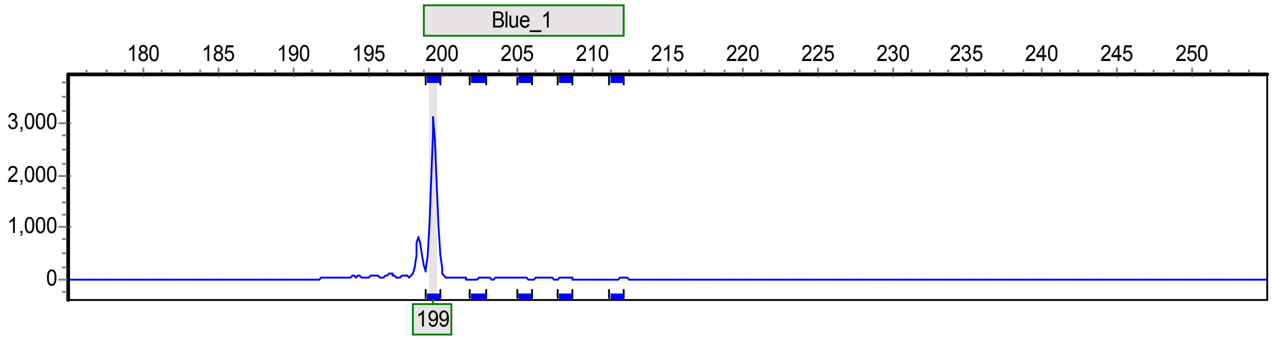

No	Size	Height	Area	Marker	Allele	Difference	Quality	Score	Allele Comments	Sample Comments
1	199.4	3090	14471	Blue_1	199	0.0	Pass	500.0		

Allele Report

Sample 52: Run date and time: 09/15/2020 - 18:52:27 -> 09/15/2020 - 19:38:19

Dye: Blue - 2 peaks - 56.fsa

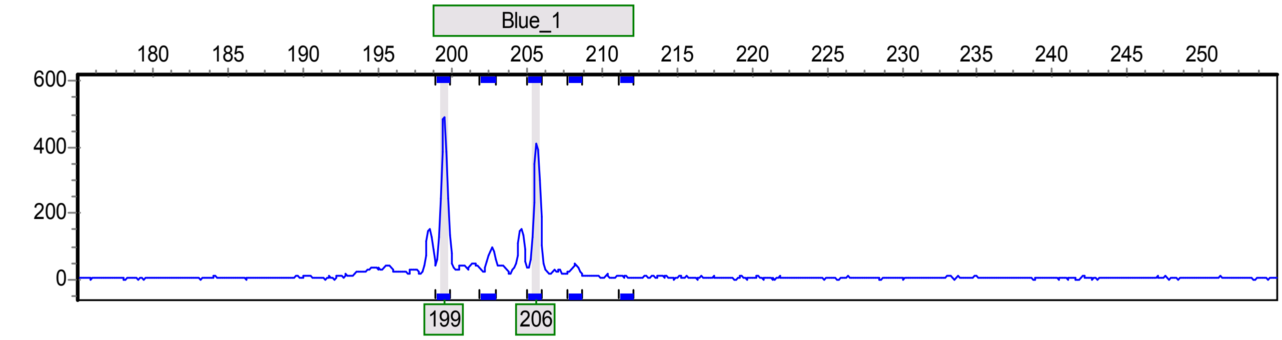

No	Size	Height	Area	Marker	Allele	Difference	Quality	Score	Allele Comments	Sample Comments
1	199.5	489	2494	Blue_1	199	0.1	Pass	68.0		
2	205.6	410	2088	Blue_1	206	0.1	Pass	51.6		

Sample 53: Run date and time: 09/15/2020 - 18:52:27 -> 09/15/2020 - 19:38:19

Dye: Blue - 1 peaks - 57.fsa

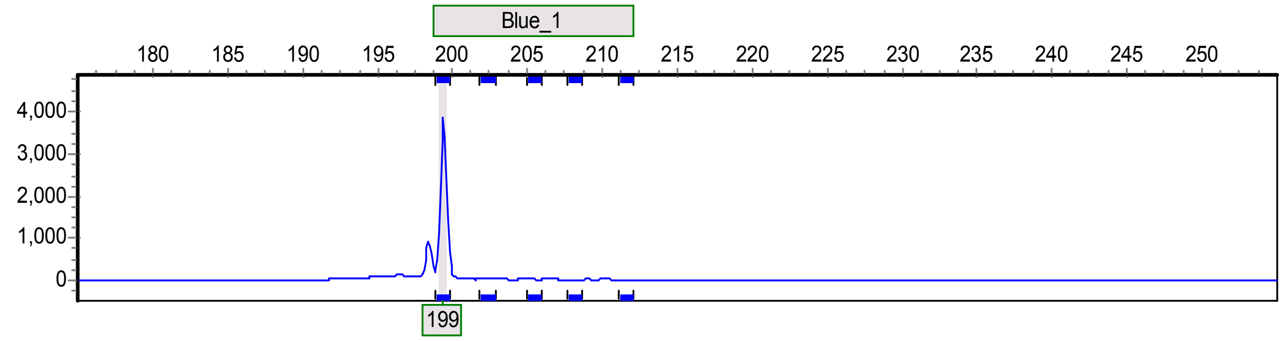

No	Size	Height	Area	Marker	Allele	Difference	Quality	Score	Allele Comments	Sample Comments
1	199.4	3848	17932	Blue_1	199	0.0	Pass	500.0		

Sample 54: Run date and time: 09/15/2020 - 18:52:27 -> 09/15/2020 - 19:38:19

Dye: Blue - 1 peaks - 58.fsa

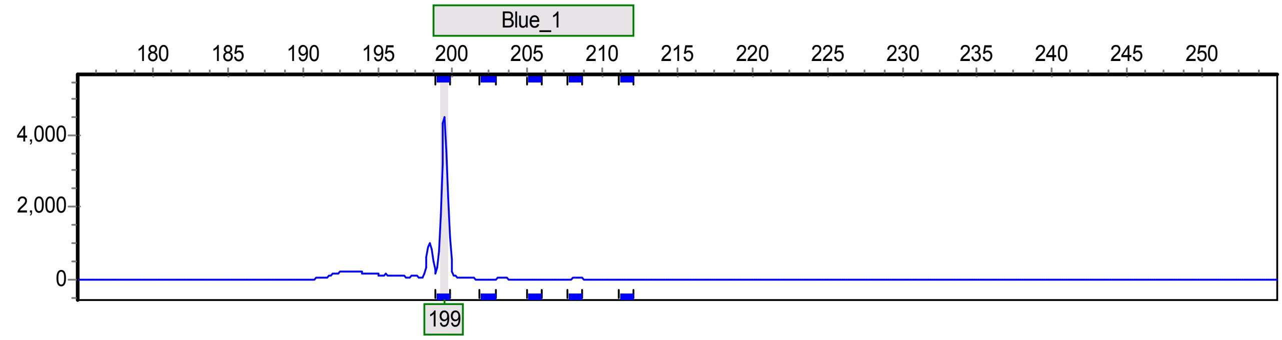

No	Size	Height	Area	Marker	Allele	Difference	Quality	Score	Allele Comments	Sample Comments
1	199.5	4453	20903	Blue_1	199	0.1	Pass	500.0		

Allele Report

Sample 55: Run date and time: 09/15/2020 - 18:52:27 -> 09/15/2020 - 19:38:19

Dye: Blue - 1 peaks - 59.fsa

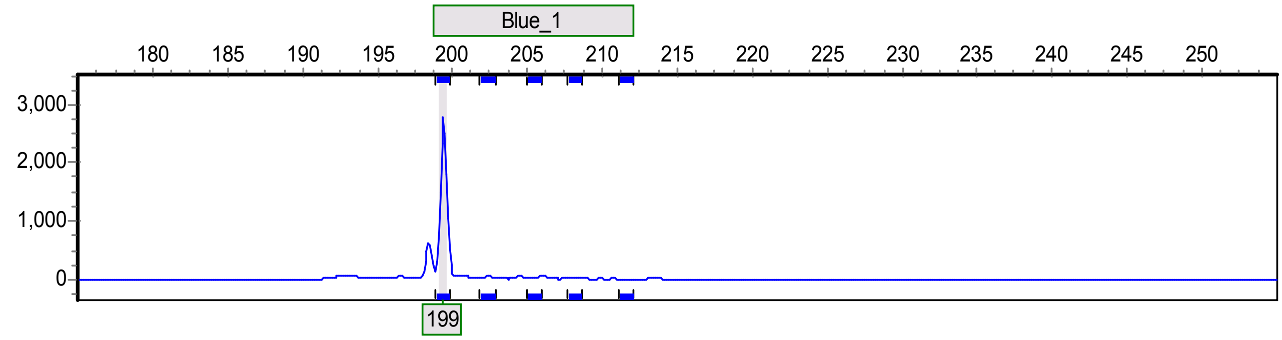

No	Size	Height	Area	Marker	Allele	Difference	Quality	Score	Allele Comments	Sample Comments
1	199.4	2758	12800	Blue_1	199	0.0	Pass	500.0		

Sample 56: Run date and time: 09/15/2020 - 18:52:27 -> 09/15/2020 - 19:38:19

Dye: Blue - 2 peaks - 6.fsa

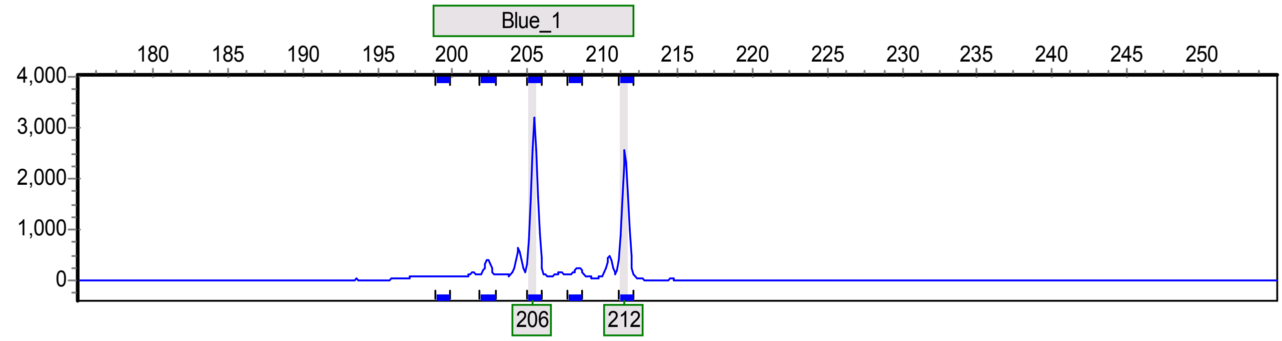

No	Size	Height	Area	Marker	Allele	Difference	Quality	Score	Allele Comments	Sample Comments
1	205.4	3192	16352	Blue_1	206	0.1	Pass	500.0		
2	211.5	2572	12653	Blue_1	212	0.1	Pass	500.0		

Sample 57: Run date and time: 09/15/2020 - 18:52:27 -> 09/15/2020 - 19:38:19

Dye: Blue - 0 peaks - 60.fsa

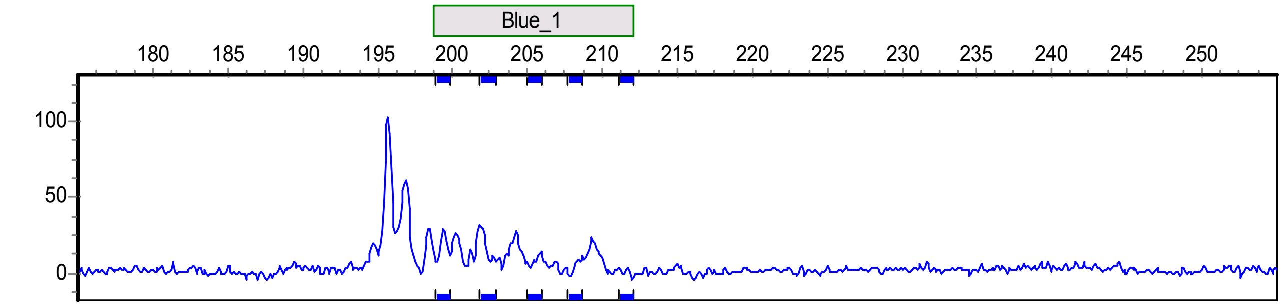

No	Size	Height	Area	Marker	Allele	Difference	Quality	Score	Allele Comments	Sample Comments

Sample 58: Run date and time: 09/15/2020 - 18:52:27 -> 09/15/2020 - 19:38:19

Dye: Blue - 1 peaks - 61.fsa

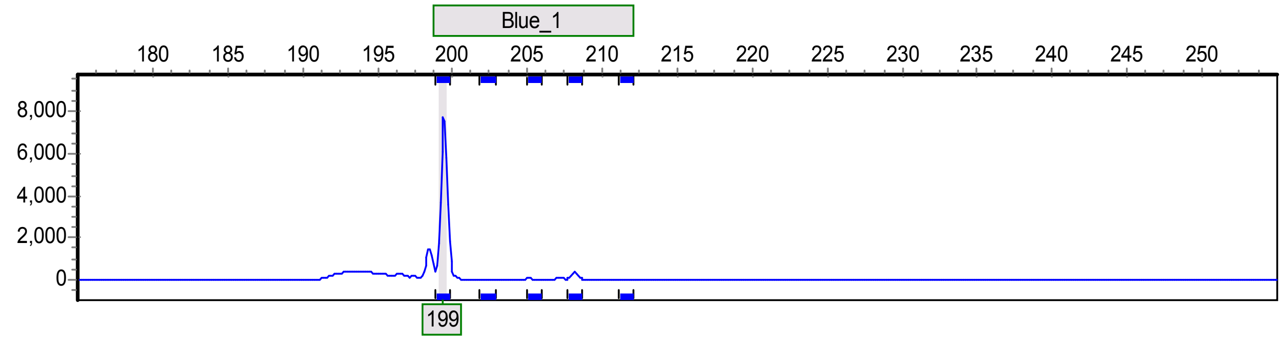

No	Size	Height	Area	Marker	Allele	Difference	Quality	Score	Allele Comments	Sample Comments
1	199.4	7698	36883	Blue_1	199	0.0	Pass	500.0		

Sample 59: Run date and time: 09/15/2020 - 18:52:27 -> 09/15/2020 - 19:38:19

Dye: Blue - 1 peaks - 62.fsa

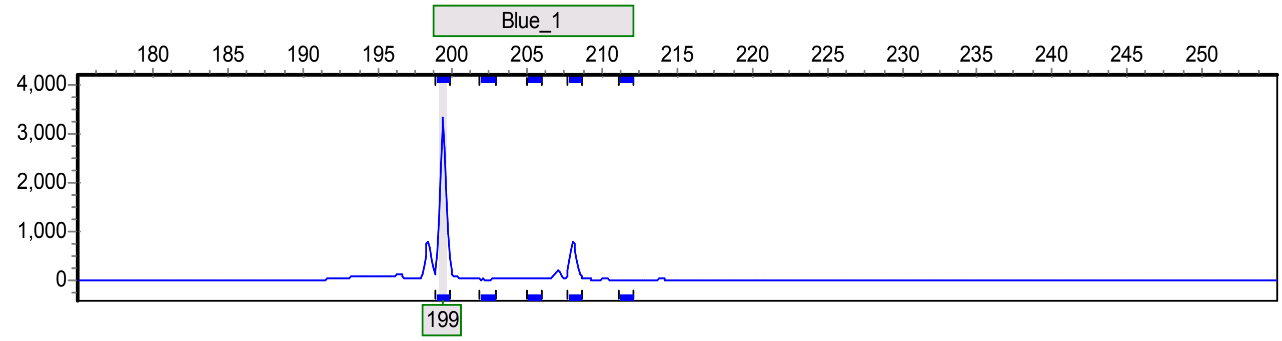

No	Size	Height	Area	Marker	Allele	Difference	Quality	Score	Allele Comments	Sample Comments
1	199.4	3300	15488	Blue_1	199	0.0	Pass	500.0		

Sample 60: Run date and time: 09/15/2020 - 18:52:27 -> 09/15/2020 - 19:38:19

Dye: Blue - 1 peaks - 63.fsa

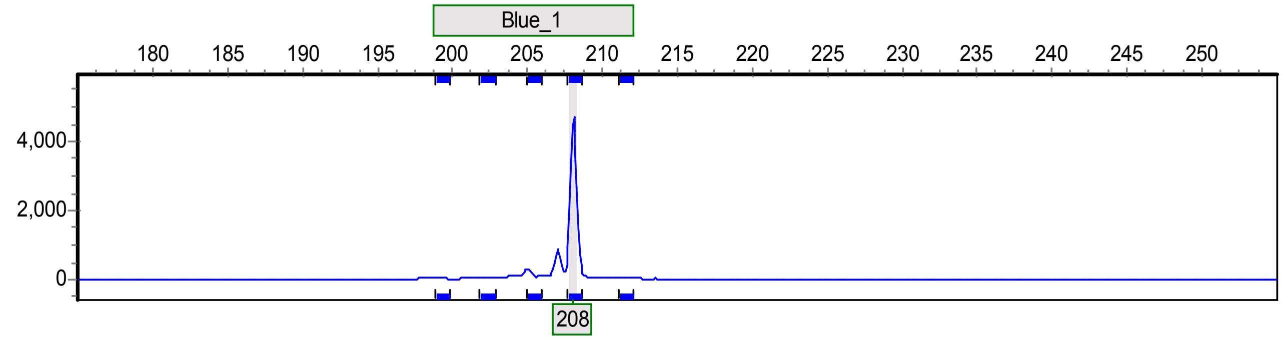

No	Size	Height	Area	Marker	Allele	Difference	Quality	Score	Allele Comments	Sample Comments
1	208.1	4707	23446	Blue_1	208	0.1	Pass	500.0		

Allele Report

Sample 61: Run date and time: 09/15/2020 - 18:52:27 -> 09/15/2020 - 19:38:19

Dye: Blue - 1 peaks - 64.fsa

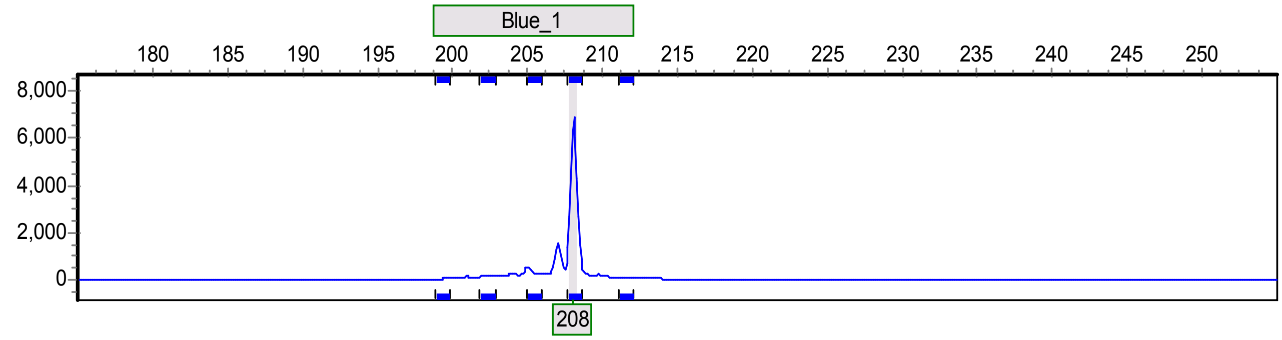

No	Size	Height	Area	Marker	Allele	Difference	Quality	Score	Allele Comments	Sample Comments
1	208.1	6847	34856	Blue_1	208	0.1	Pass	500.0		

Sample 62: Run date and time: 09/15/2020 - 18:52:27 -> 09/15/2020 - 19:38:19

Dye: Blue - 1 peaks - 65.fsa

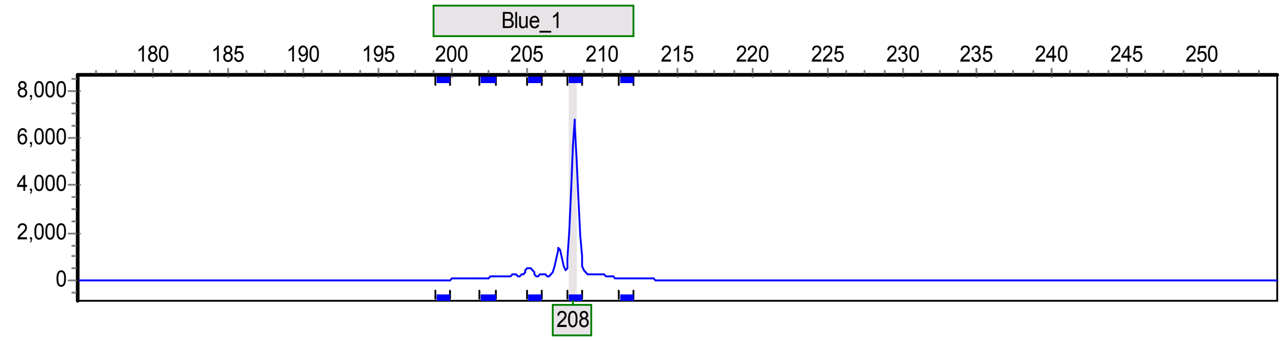

No	Size	Height	Area	Marker	Allele	Difference	Quality	Score	Allele Comments	Sample Comments
1	208.1	6826	35572	Blue_1	208	0.1	Pass	500.0		

Sample 63: Run date and time: 09/15/2020 - 18:52:27 -> 09/15/2020 - 19:38:19

Dye: Blue - 1 peaks - 66.fsa

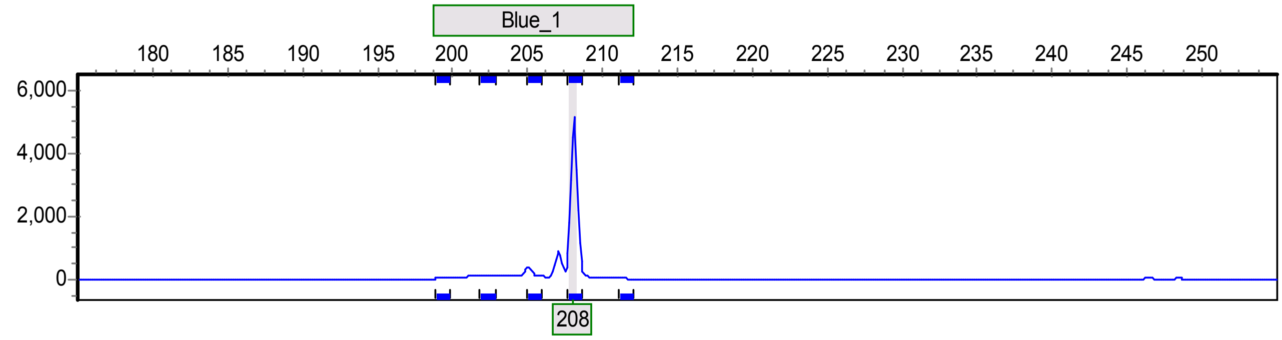

No	Size	Height	Area	Marker	Allele	Difference	Quality	Score	Allele Comments	Sample Comments
1	208.1	5128	26035	Blue_1	208	0.1	Pass	500.0		

Allele Report

Sample 64: Run date and time: 09/15/2020 - 18:52:27 -> 09/15/2020 - 19:38:19

Dye: Blue - 1 peaks - 67.fsa

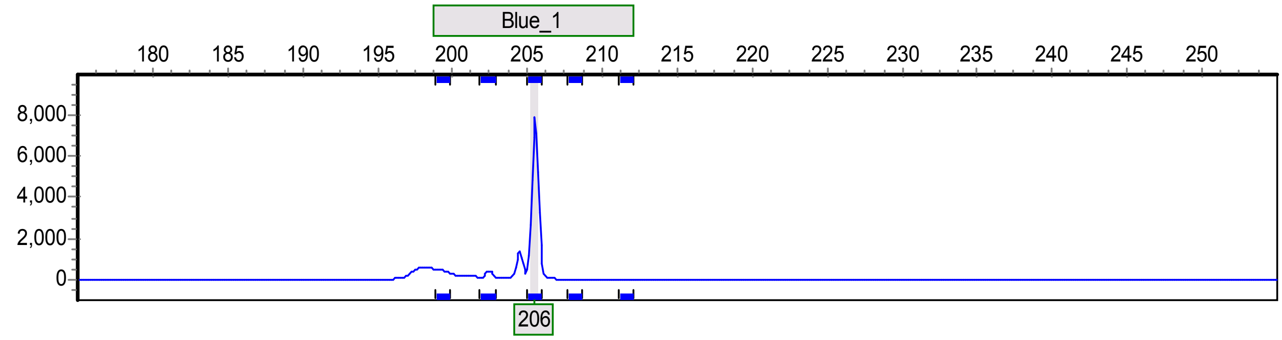

No	Size	Height	Area	Marker	Allele	Difference	Quality	Score	Allele Comments	Sample Comments
1	205.5	7820	38929	Blue_1	206	0.0	Pass	500.0		

Sample 65: Run date and time: 09/15/2020 - 18:52:27 -> 09/15/2020 - 19:38:19

Dye: Blue - 1 peaks - 68.fsa

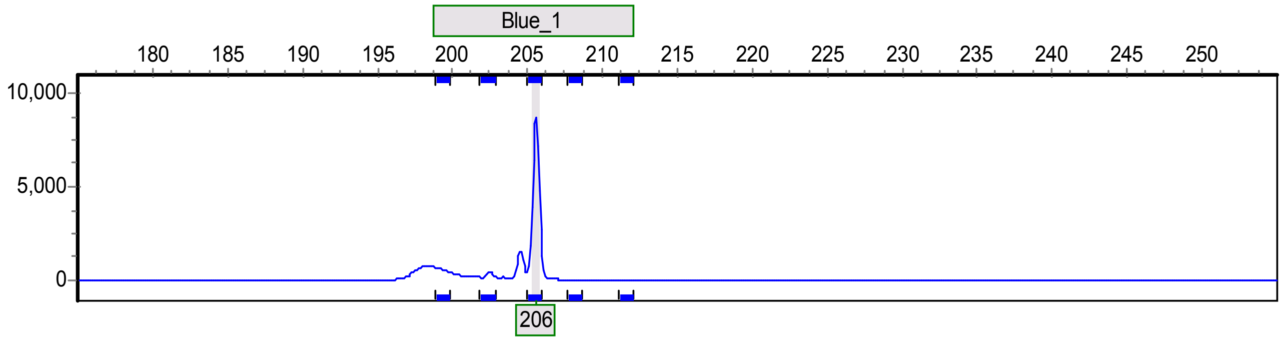

No	Size	Height	Area	Marker	Allele	Difference	Quality	Score	Allele Comments	Sample Comments
1	205.6	8608	43217	Blue_1	206	0.1	Pass	500.0		

Sample 66: Run date and time: 09/15/2020 - 18:52:27 -> 09/15/2020 - 19:38:19

Dye: Blue - 1 peaks - 69.fsa

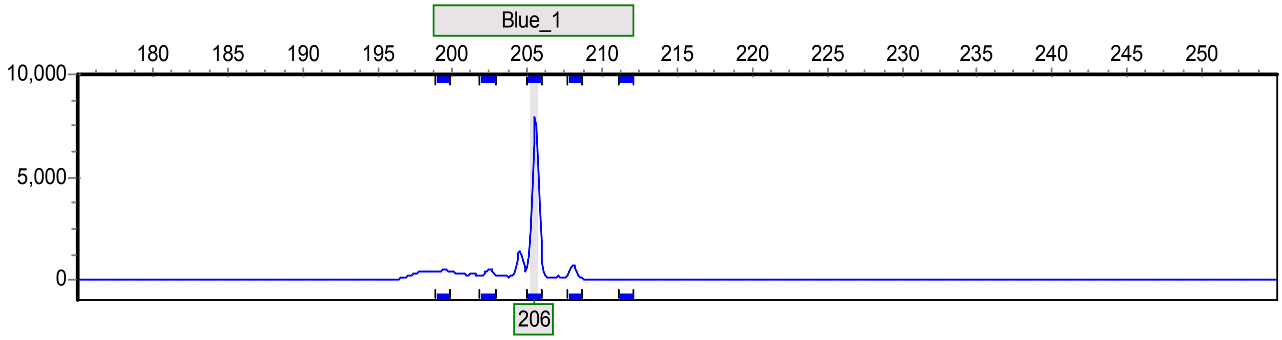

No	Size	Height	Area	Marker	Allele	Difference	Quality	Score	Allele Comments	Sample Comments
1	205.5	7890	40071	Blue_1	206	0.0	Pass	500.0		

Sample 67: Run date and time: 09/15/2020 - 18:52:27 -> 09/15/2020 - 19:38:19

Dye: Blue - 1 peaks - 7.fsa

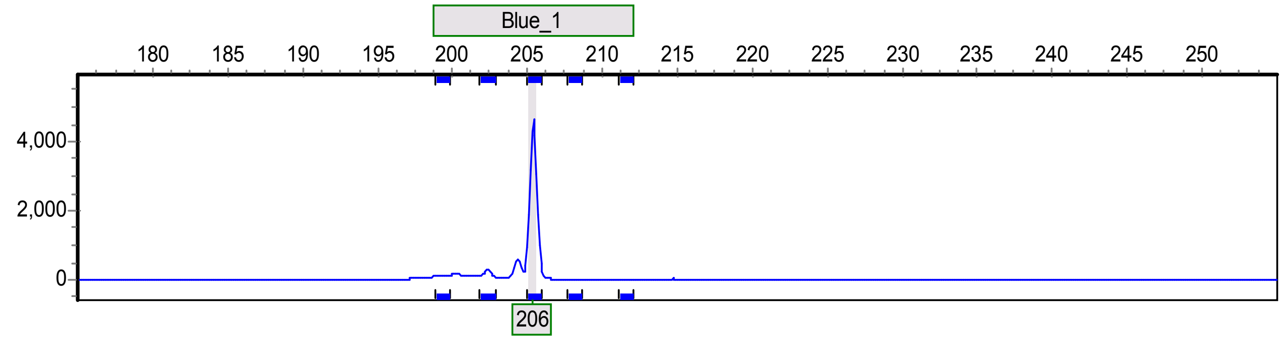

No	Size	Height	Area	Marker	Allele	Difference	Quality	Score	Allele Comments	Sample Comments
1	205.4	4661	24103	Blue_1	206	0.1	Pass	500.0		

Sample 68: Run date and time: 09/15/2020 - 18:52:27 -> 09/15/2020 - 19:38:19

Dye: Blue - 1 peaks - 70.fsa

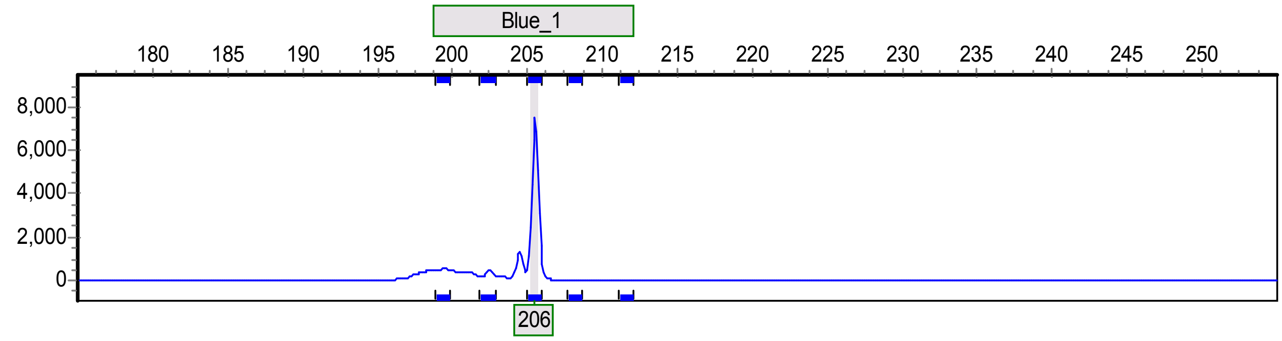

No	Size	Height	Area	Marker	Allele	Difference	Quality	Score	Allele Comments	Sample Comments
1	205.5	7446	36870	Blue_1	206	0.0	Pass	500.0		

Sample 69: Run date and time: 09/15/2020 - 18:52:27 -> 09/15/2020 - 19:38:19

Dye: Blue - 1 peaks - 71.fsa

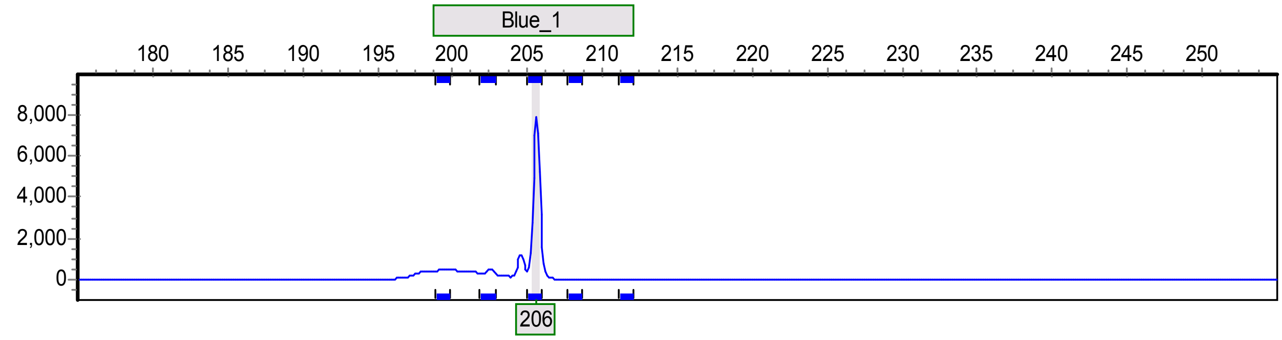

No	Size	Height	Area	Marker	Allele	Difference	Quality	Score	Allele Comments	Sample Comments
1	205.6	7839	39227	Blue_1	206	0.1	Pass	500.0		

Allele Report

Sample 70: Run date and time: 09/15/2020 - 18:52:27 -> 09/15/2020 - 19:38:19

Dye: Blue - 1 peaks - 72.fsa

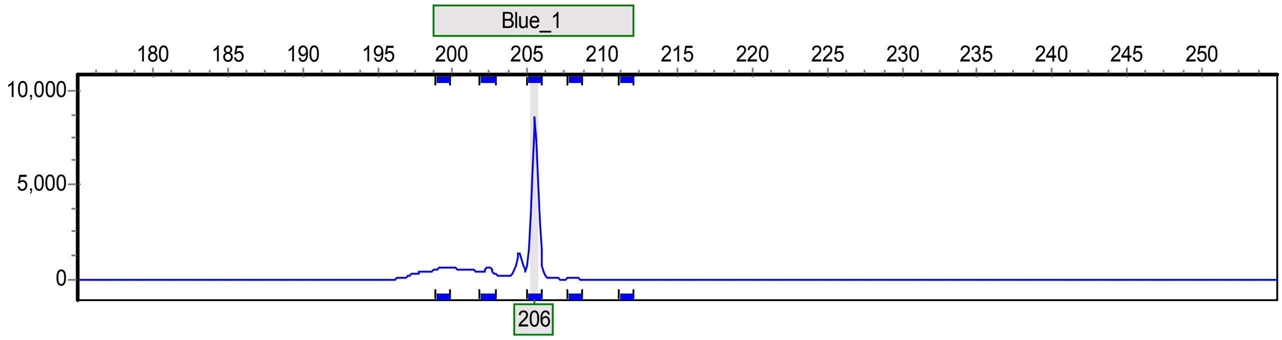

No	Size	Height	Area	Marker	Allele	Difference	Quality	Score	Allele Comments	Sample Comments
1	205.5	8538	43061	Blue_1	206	0.0	Pass	500.0		

Sample 71: Run date and time: 09/15/2020 - 18:52:27 -> 09/15/2020 - 19:38:19

Dye: Blue - 1 peaks - 73.fsa

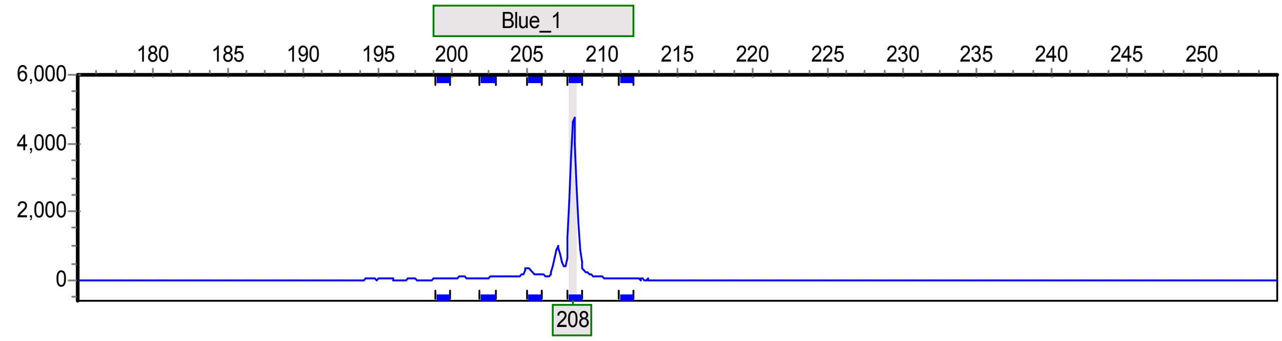

No	Size	Height	Area	Marker	Allele	Difference	Quality	Score	Allele Comments	Sample Comments
1	208.1	4736	25294	Blue_1	208	0.1	Pass	500.0		

Sample 72: Run date and time: 09/15/2020 - 18:52:27 -> 09/15/2020 - 19:38:19

Dye: Blue - 1 peaks - 74.fsa

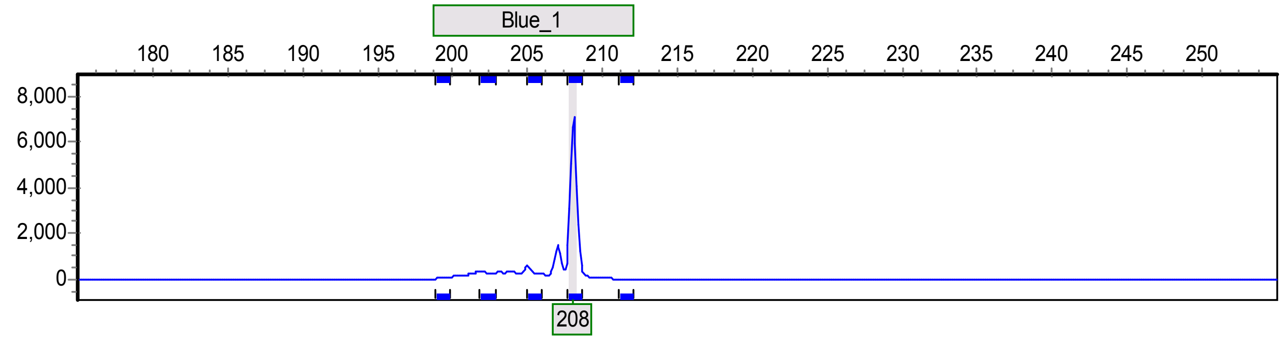

No	Size	Height	Area	Marker	Allele	Difference	Quality	Score	Allele Comments	Sample Comments
1	208.1	7038	35673	Blue_1	208	0.1	Pass	500.0		

Sample 73: Run date and time: 09/15/2020 - 18:52:27 -> 09/15/2020 - 19:38:19

Dye: Blue - 1 peaks - 75.fsa

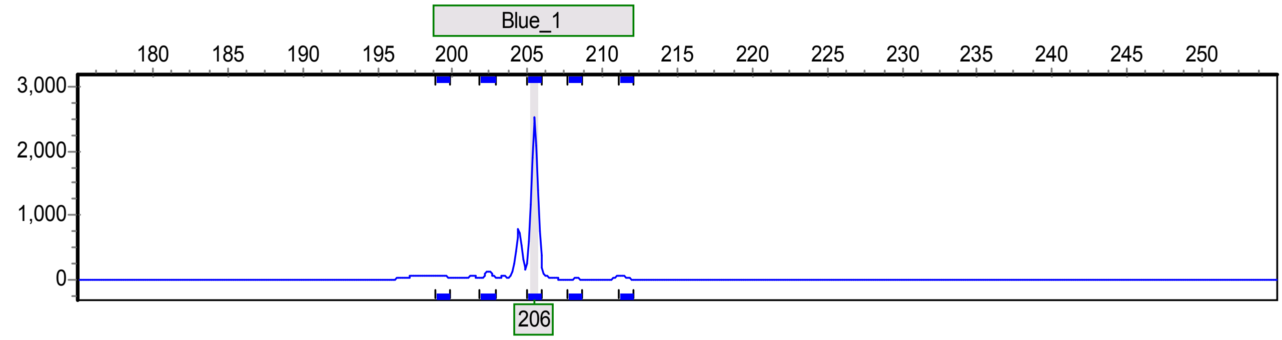

No	Size	Height	Area	Marker	Allele	Difference	Quality	Score	Allele Comments	Sample Comments
1	205.5	2518	12808	Blue_1	206	0.0	Pass	500.0		

Sample 74: Run date and time: 09/15/2020 - 18:52:27 -> 09/15/2020 - 19:38:19

Dye: Blue - 1 peaks - 76.fsa

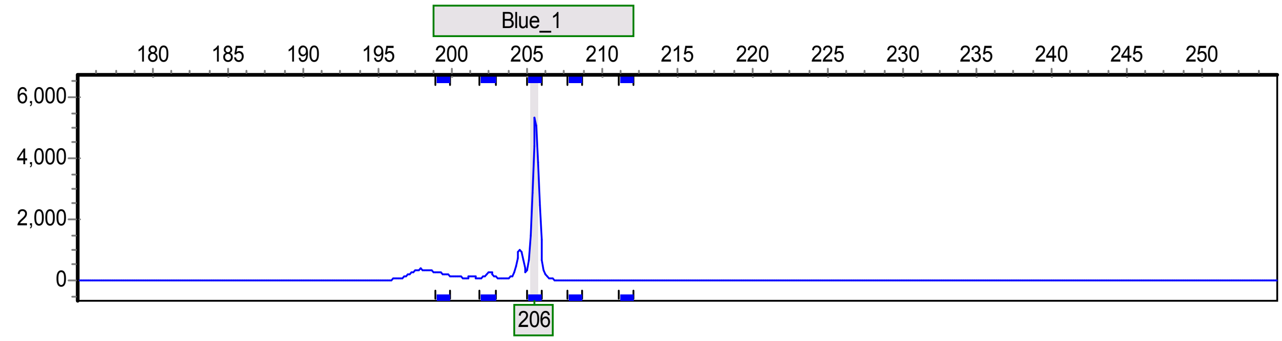

No	Size	Height	Area	Marker	Allele	Difference	Quality	Score	Allele Comments	Sample Comments
1	205.5	5299	26495	Blue_1	206	0.0	Pass	500.0		

Sample 75: Run date and time: 09/15/2020 - 18:52:27 -> 09/15/2020 - 19:38:19

Dye: Blue - 1 peaks - 77.fsa

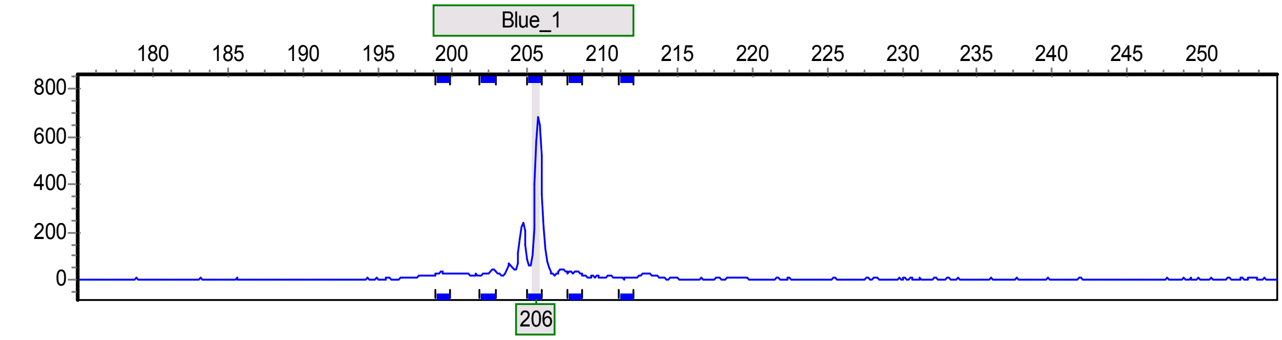

No	Size	Height	Area	Marker	Allele	Difference	Quality	Score	Allele Comments	Sample Comments
1	205.7	678	3708	Blue_1	206	0.2	Pass	103.6		

Sample 76: Run date and time: 09/15/2020 - 18:52:27 -> 09/15/2020 - 19:38:19

Dye: Blue - 2 peaks - 78.fsa

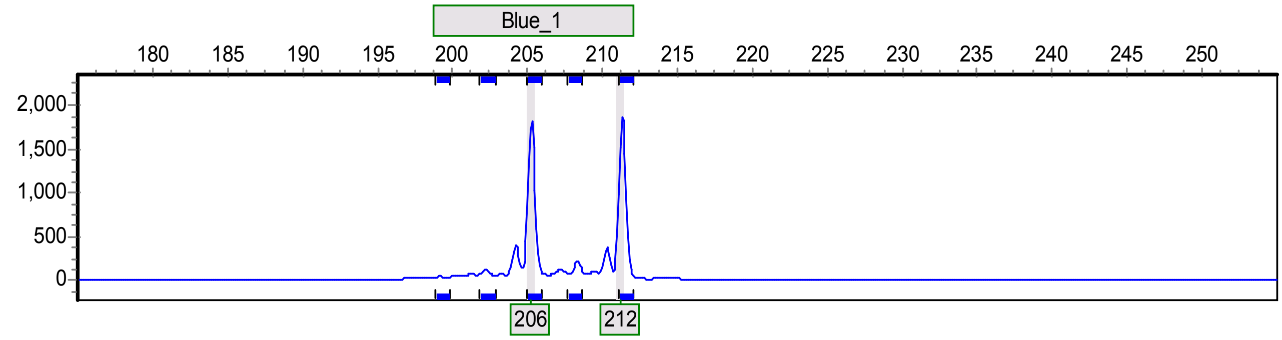

No	Size	Height	Area	Marker	Allele	Difference	Quality	Score	Allele Comments	Sample Comments
1	205.3	1817	9223	Blue_1	206	0.2	Pass	479.6		
2	211.3	1860	9542	Blue_1	212	0.3	Pass	491.0		

Sample 77: Run date and time: 09/15/2020 - 18:52:27 -> 09/15/2020 - 19:38:19

Dye: Blue - 1 peaks - 79.fsa

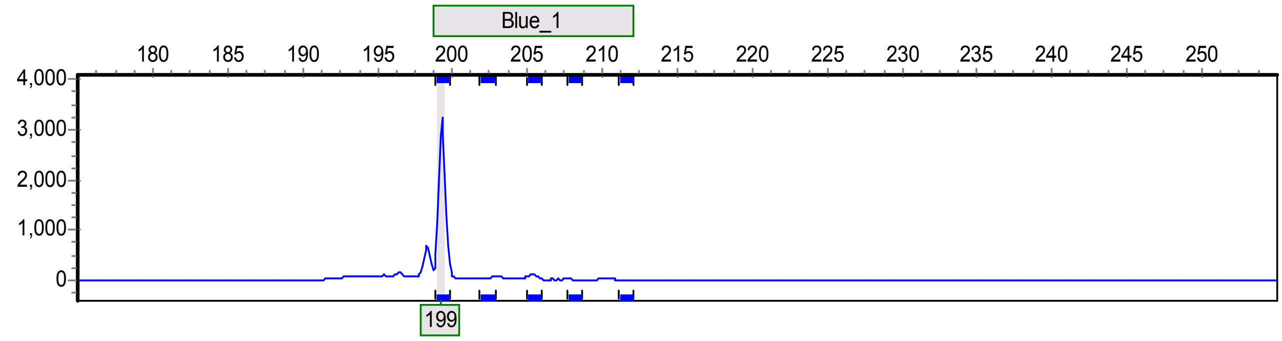

No	Size	Height	Area	Marker	Allele	Difference	Quality	Score	Allele Comments	Sample Comments
1	199.3	3225	16178	Blue_1	199	0.1	Pass	500.0		

Sample 78: Run date and time: 09/15/2020 - 18:52:27 -> 09/15/2020 - 19:38:19

Dye: Blue - 0 peaks - 8.fsa

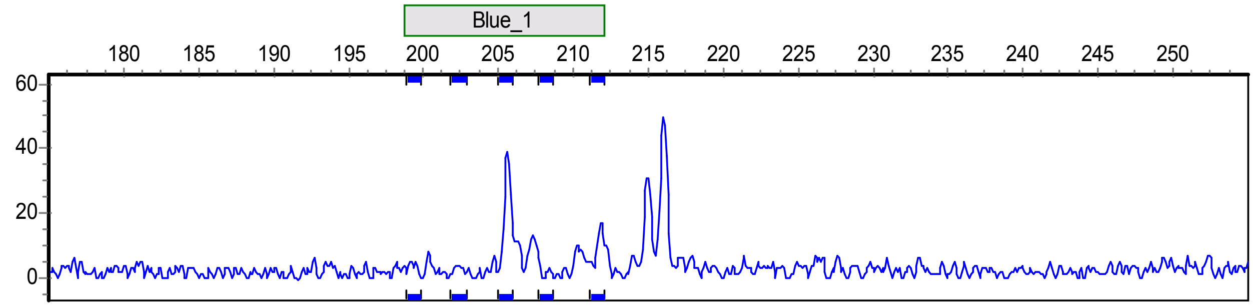

No	Size	Height	Area	Marker	Allele	Difference	Quality	Score	Allele Comments	Sample Comments

Sample 79: Run date and time: 09/15/2020 - 18:52:27 -> 09/15/2020 - 19:38:19

Dye: Blue - 2 peaks - 80.fsa

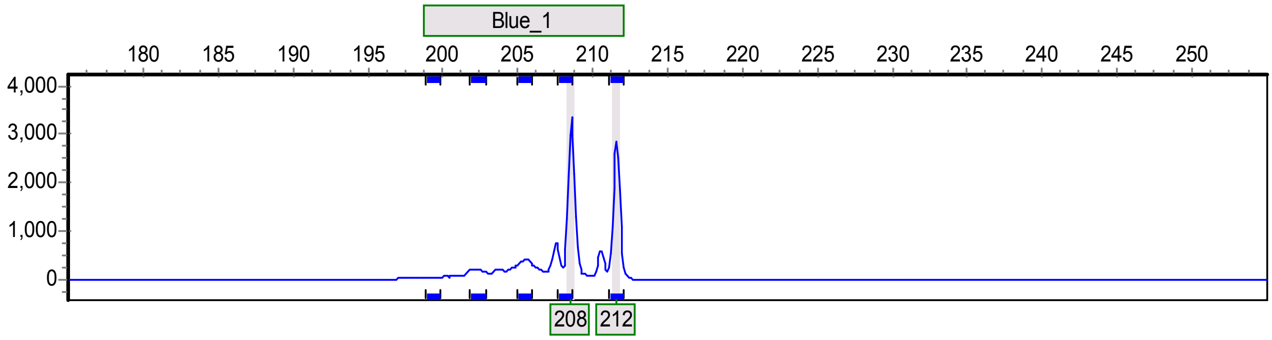

No	Size	Height	Area	Marker	Allele	Difference	Quality	Score	Allele Comments	Sample Comments
1	208.6	3332	16795	Blue_1	208	0.4	Pass	500.0		
2	211.6	2836	14341	Blue_1	212	0.0	Pass	500.0		

Sample 80: Run date and time: 09/15/2020 - 18:52:27 -> 09/15/2020 - 19:38:19

Dye: Blue - 1 peaks - 81.fsa

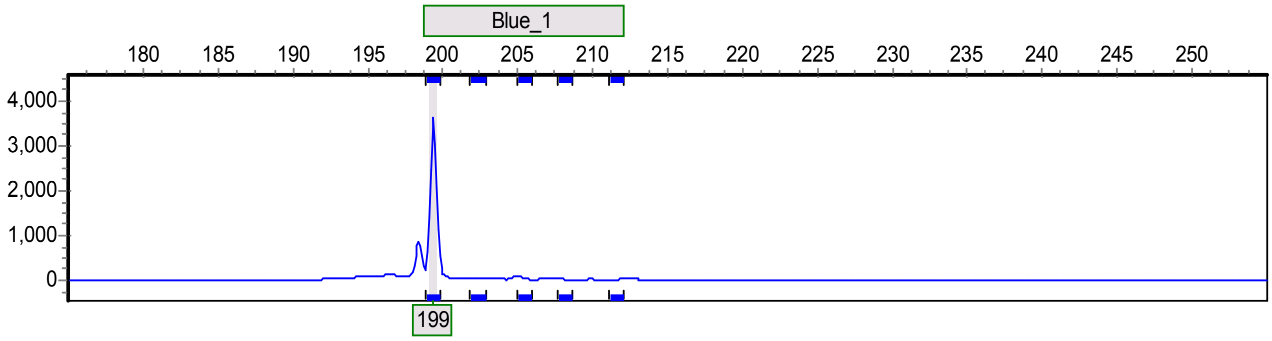

No	Size	Height	Area	Marker	Allele	Difference	Quality	Score	Allele Comments	Sample Comments
1	199.4	3616	17451	Blue_1	199	0.0	Pass	500.0		

Sample 81: Run date and time: 09/15/2020 - 18:52:27 -> 09/15/2020 - 19:38:19

Dye: Blue - 1 peaks - 82.fsa

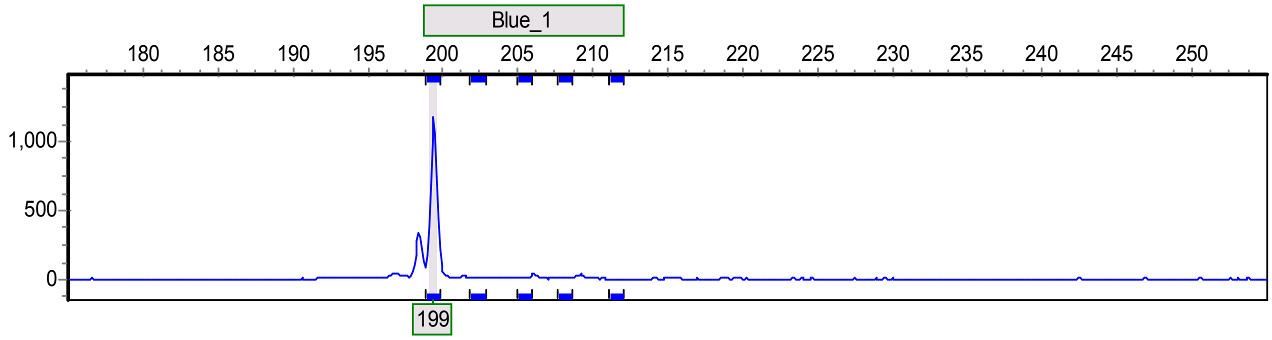

No	Size	Height	Area	Marker	Allele	Difference	Quality	Score	Allele Comments	Sample Comments
1	199.4	1171	5648	Blue_1	199	0.0	Pass	275.3		

Allele Report

Sample 82: Run date and time: 09/15/2020 - 18:52:27 -> 09/15/2020 - 19:38:19

Dye: Blue - 1 peaks - 83.fsa

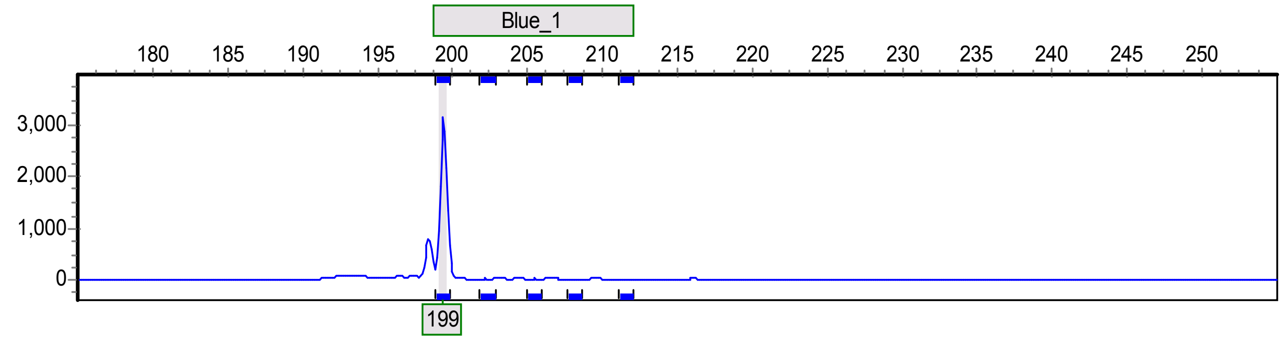

No	Size	Height	Area	Marker	Allele	Difference	Quality	Score	Allele Comments	Sample Comments
1	199.4	3133	15450	Blue_1	199	0.0	Pass	500.0		

Sample 83: Run date and time: 09/15/2020 - 18:52:27 -> 09/15/2020 - 19:38:19

Dye: Blue - 2 peaks - 84.fsa

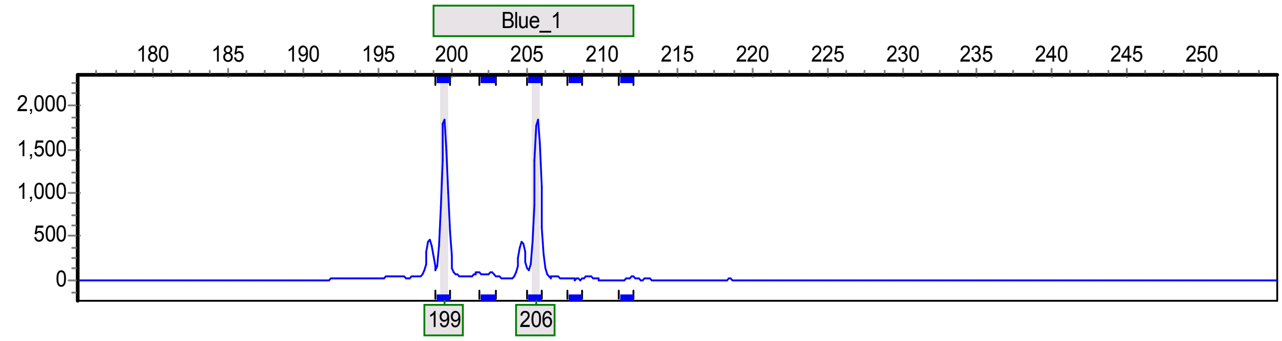

No	Size	Height	Area	Marker	Allele	Difference	Quality	Score	Allele Comments	Sample Comments
1	199.5	1835	9158	Blue_1	199	0.1	Pass	494.5		
2	205.7	1851	9497	Blue_1	206	0.2	Pass	482.9		

Sample 84: Run date and time: 09/15/2020 - 18:52:27 -> 09/15/2020 - 19:38:19

Dye: Blue - 1 peaks - 85.fsa

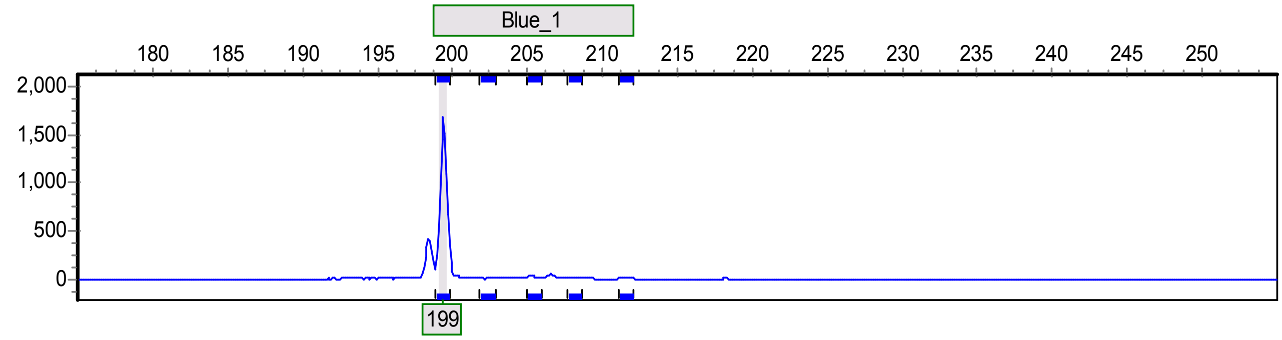

No	Size	Height	Area	Marker	Allele	Difference	Quality	Score	Allele Comments	Sample Comments
1	199.4	1672	8207	Blue_1	199	0.0	Pass	446.0		

Sample 85: Run date and time: 09/15/2020 - 18:52:27 -> 09/15/2020 - 19:38:19

Dye: Blue - 1 peaks - 86.fsa

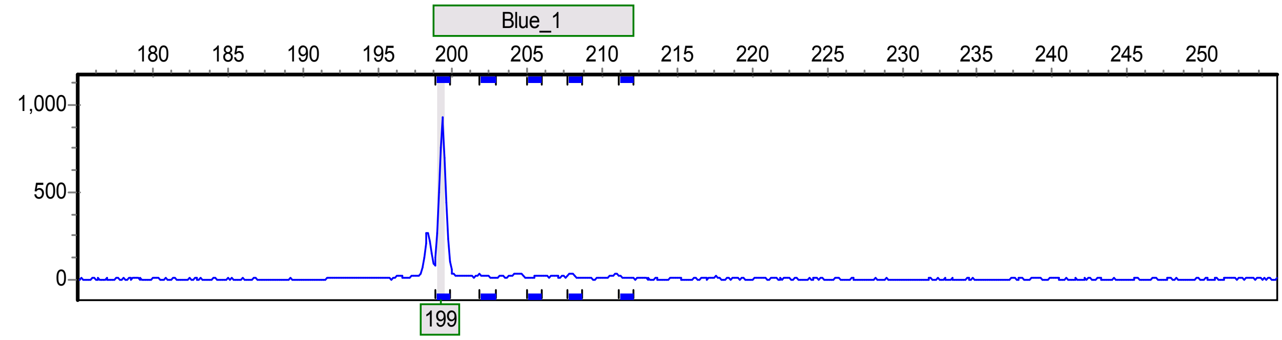

No	Size	Height	Area	Marker	Allele	Difference	Quality	Score	Allele Comments	Sample Comments
1	199.3	920	4637	Blue_1	199	0.1	Pass	182.5		

Sample 86: Run date and time: 09/15/2020 - 18:52:27 -> 09/15/2020 - 19:38:19

Dye: Blue - 1 peaks - 87.fsa

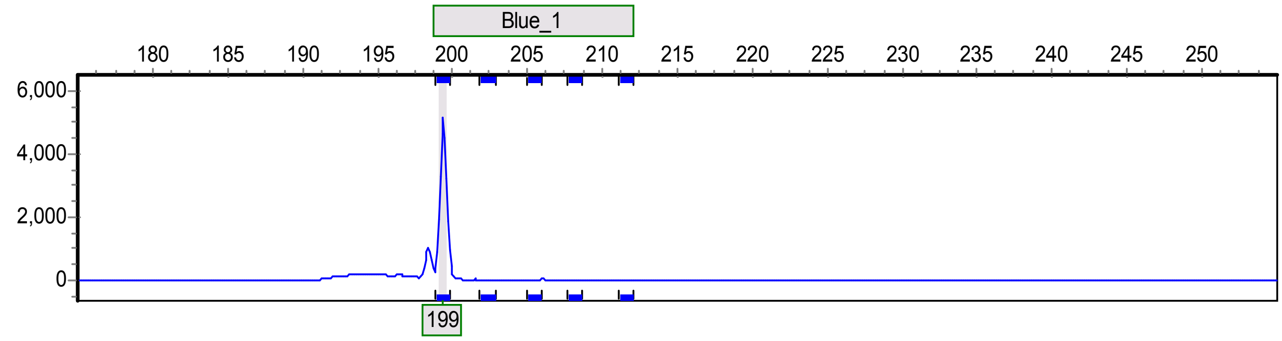

No	Size	Height	Area	Marker	Allele	Difference	Quality	Score	Allele Comments	Sample Comments
1	199.4	5153	25439	Blue_1	199	0.0	Pass	500.0		

Sample 87: Run date and time: 09/15/2020 - 18:52:27 -> 09/15/2020 - 19:38:19

Dye: Blue - 2 peaks - 88.fsa

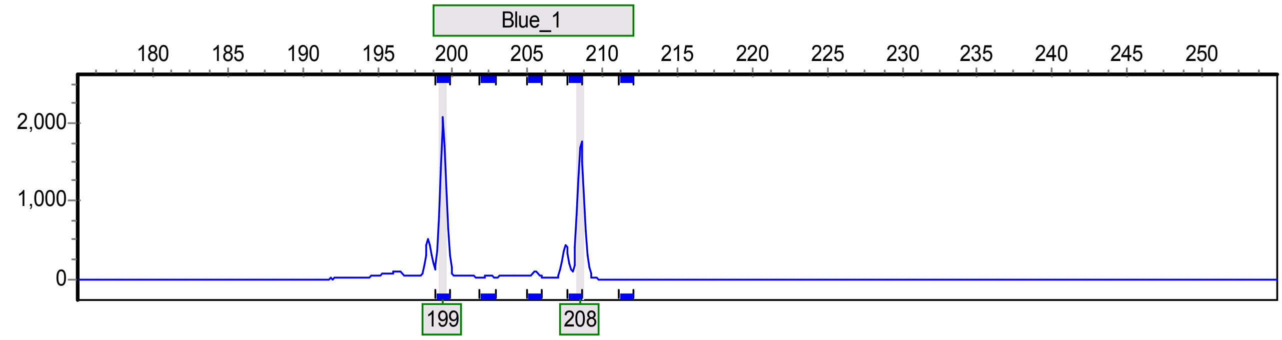

No	Size	Height	Area	Marker	Allele	Difference	Quality	Score	Allele Comments	Sample Comments
1	199.4	2056	9956	Blue_1	199	0.0	Pass	500.0		
2	208.6	1750	9134	Blue_1	208	0.4	Pass	436.8		

Sample 88: Run date and time: 09/15/2020 - 18:52:27 -> 09/15/2020 - 19:38:19

Dye: Blue - 1 peaks - 89.fsa

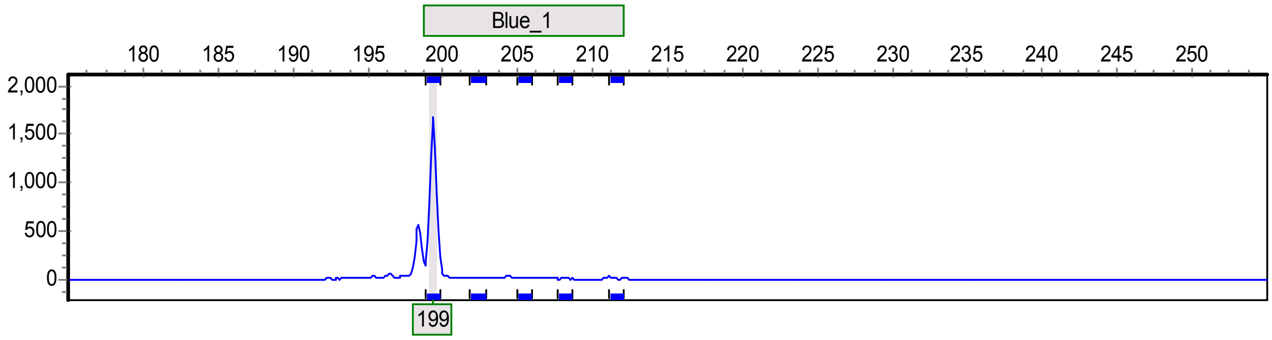

No	Size	Height	Area	Marker	Allele	Difference	Quality	Score	Allele Comments	Sample Comments
1	199.4	1667	8491	Blue_1	199	0.0	Pass	423.4		

Sample 89: Run date and time: 09/15/2020 - 18:52:27 -> 09/15/2020 - 19:38:19

Dye: Blue - 2 peaks - 9.fsa

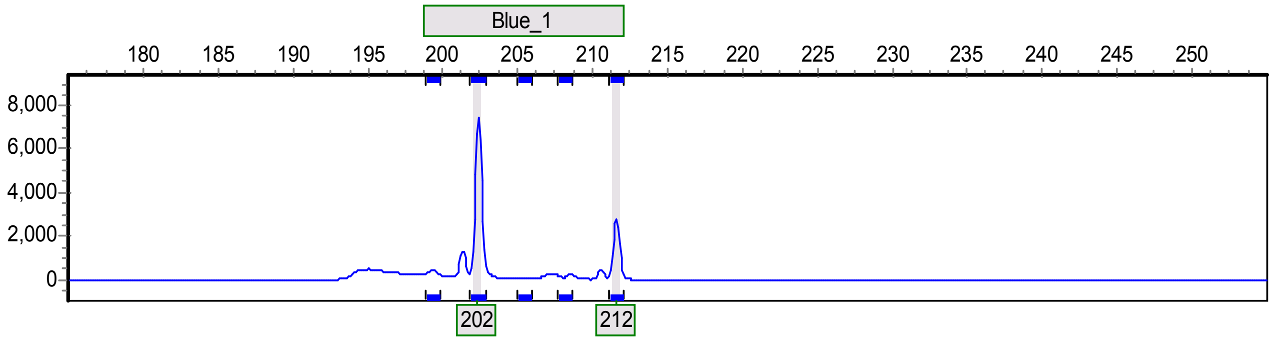

No	Size	Height	Area	Marker	Allele	Difference	Quality	Score	Allele Comments	Sample Comments
1	202.4	7357	36283	Blue_1	202	0.0	Pass	500.0		
2	211.6	2785	13796	Blue_1	212	0.0	Pass	500.0		

Sample 90: Run date and time: 09/15/2020 - 18:52:27 -> 09/15/2020 - 19:38:19

Dye: Blue - 1 peaks - 90.fsa

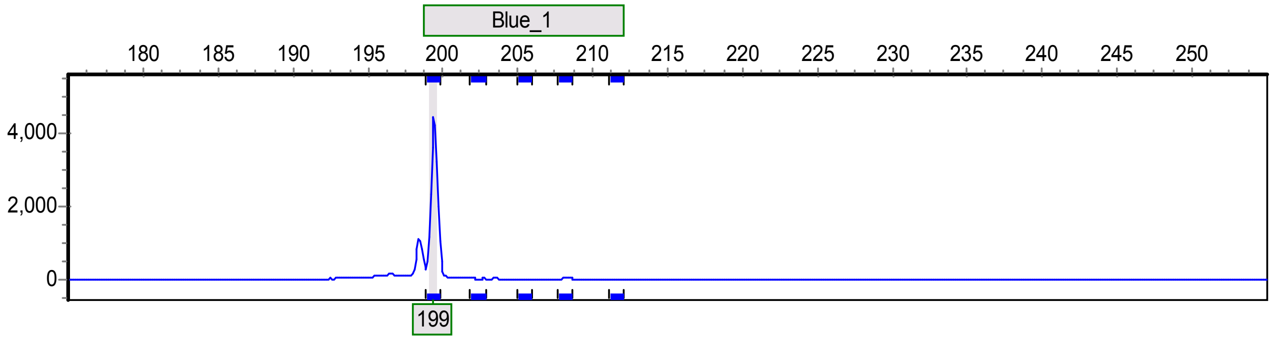

No	Size	Height	Area	Marker	Allele	Difference	Quality	Score	Allele Comments	Sample Comments
1	199.4	4429	21845	Blue_1	199	0.0	Pass	500.0		

Allele Report

Sample 91: Run date and time: 09/15/2020 - 18:52:27 -> 09/15/2020 - 19:38:19

Dye: Blue - 3 peaks - 91.fsa

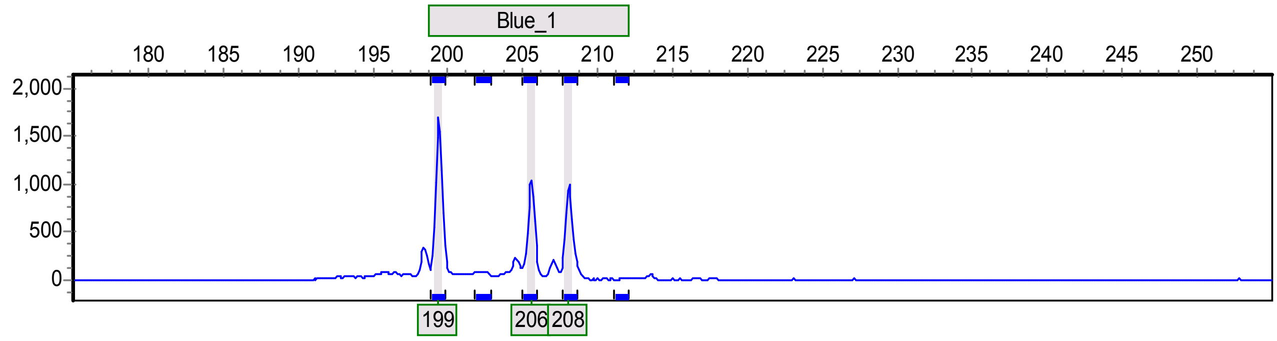

No	Size	Height	Area	Marker	Allele	Difference	Quality	Score	Allele Comments	Sample Comments
1	199.4	1687	8384	Blue_1	199	0.0	Pass	443.2		
2	205.6	1043	5504	Blue_1	206	0.1	Pass	207.3		
3	208.1	1001	5493	Blue_1	208	0.1	Pass	187.2		

Sample 92: Run date and time: 09/15/2020 - 18:52:27 -> 09/15/2020 - 19:38:19

Dye: Blue - 2 peaks - 92.fsa

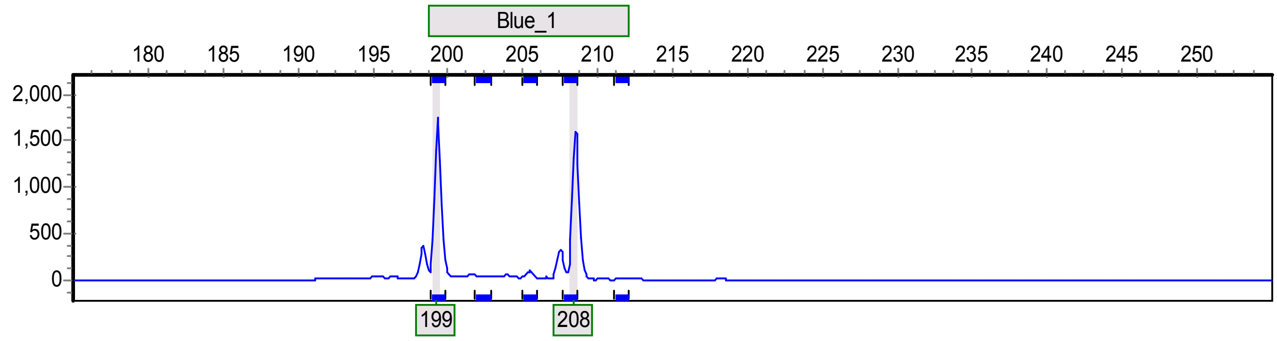

No	Size	Height	Area	Marker	Allele	Difference	Quality	Score	Allele Comments	Sample Comments
1	199.3	1741	8533	Blue_1	199	0.1	Pass	475.6		
2	208.5	1596	8254	Blue_1	208	0.3	Pass	393.5		

Sample 93: Run date and time: 09/15/2020 - 18:52:27 -> 09/15/2020 - 19:38:19

Dye: Blue - 2 peaks - 93.fsa

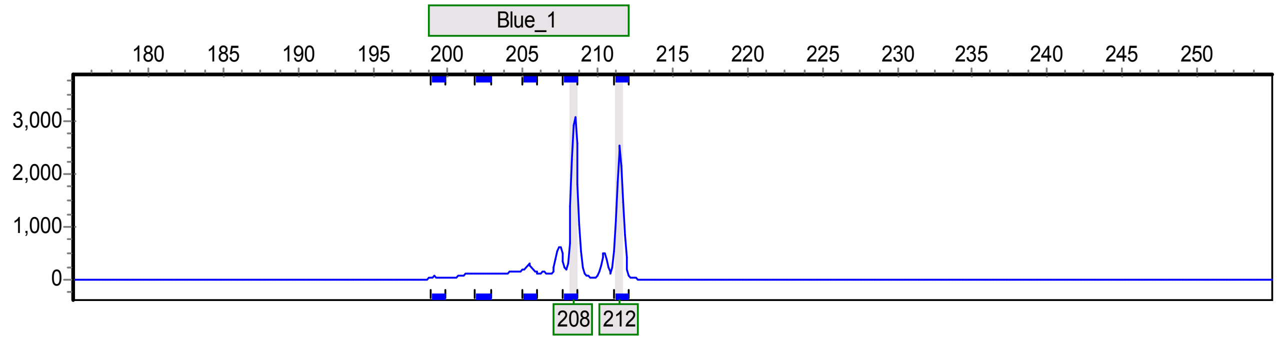

No	Size	Height	Area	Marker	Allele	Difference	Quality	Score	Allele Comments	Sample Comments
1	208.5	3063	15701	Blue_1	208	0.3	Pass	500.0		
2	211.5	2537	12888	Blue_1	212	0.1	Pass	500.0		

Allele Report

Sample 94: Run date and time: 09/15/2020 - 18:52:27 -> 09/15/2020 - 19:38:19

Dye: Blue - 2 peaks - 94.fsa

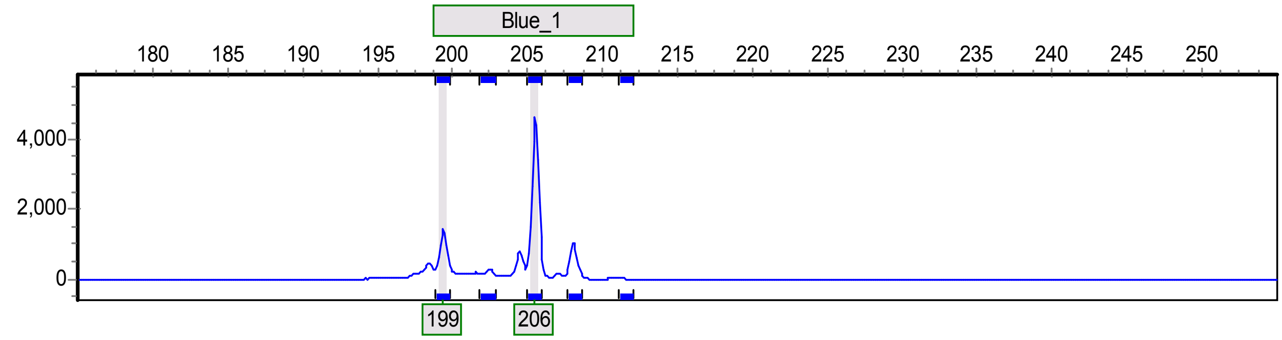

No	Size	Height	Area	Marker	Allele	Difference	Quality	Score	Allele Comments	Sample Comments
1	199.4	1455	8294	Blue_1	199	0.0	Pass	301.2		
2	205.5	4589	23950	Blue_1	206	0.0	Pass	500.0		

Sample 95: Run date and time: 09/15/2020 - 18:52:27 -> 09/15/2020 - 19:38:19

Dye: Blue - 3 peaks - 95.fsa

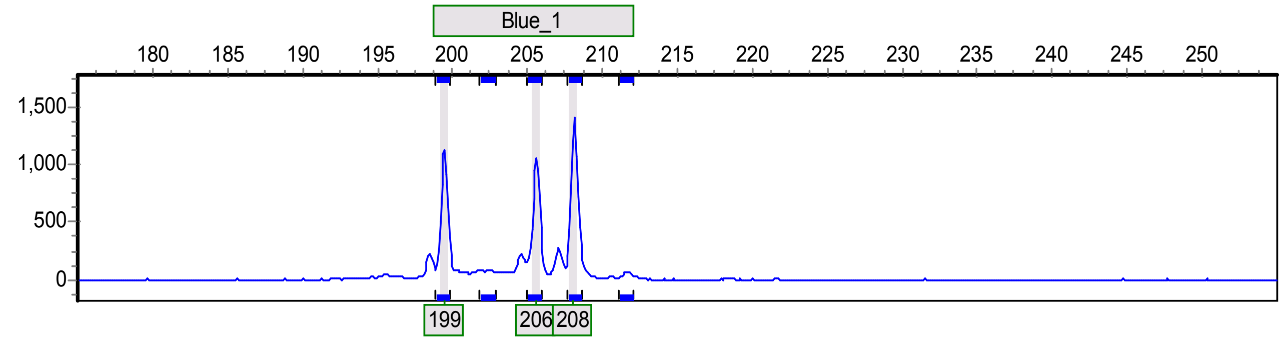

No	Size	Height	Area	Marker	Allele	Difference	Quality	Score	Allele Comments	Sample Comments
1	199.5	1119	5713	Blue_1	199	0.1	Pass	238.7		
2	205.6	1048	5791	Blue_1	206	0.1	Pass	197.8		
3	208.1	1397	7580	Blue_1	208	0.1	Pass	306.7		

Sample 96: Run date and time: 09/15/2020 - 18:52:27 -> 09/15/2020 - 19:38:19

Dye: Blue - 1 peaks - 96.fsa

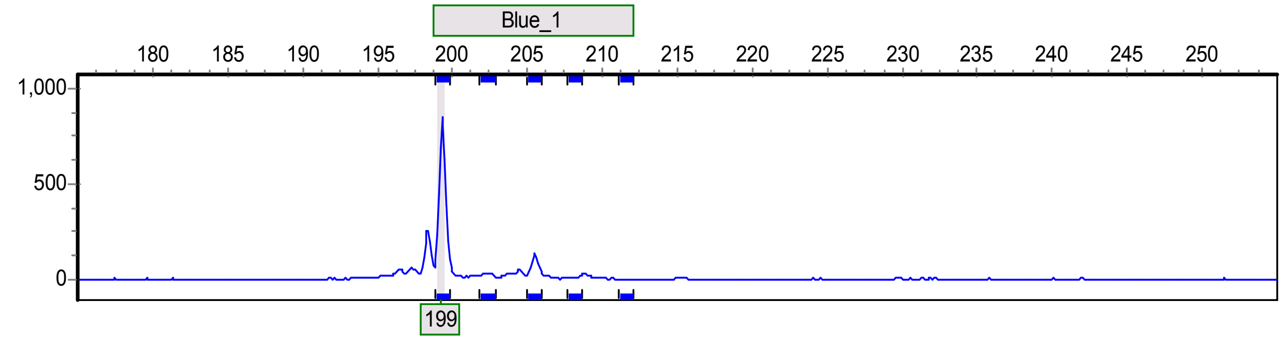

No	Size	Height	Area	Marker	Allele	Difference	Quality	Score	Allele Comments	Sample Comments
1	199.3	845	4207	Blue_1	199	0.1	Pass	165.2		
