# Supplementary material for: Genetic diversity and population structure of Polygonatum cyrtonema Hua in China using SSR markers
Source: PLoS One. 2023 Aug 31;18(8):e0290605. doi: 10.1371/journal.pone.0290605 (PMC10470896; doi:10.1371/journal.pone.0290605)
Supplement: S1 Data — (ZIP) [file pone.0290605.s002.zip › ╨┬╜¿╬─╝■╝╨ (2)/╥2╬∩21/yinwu21.pdf]

SoftGenetics

GeneMarker V2.2.0

Allele Report

2020/9/16 16:19:44

Sample 1: Run date and time: 09/11/2020 - 20:27:01 -> 09/11/2020 - 21:12:55

Dye: Blue - 3 peaks - 1.fsa

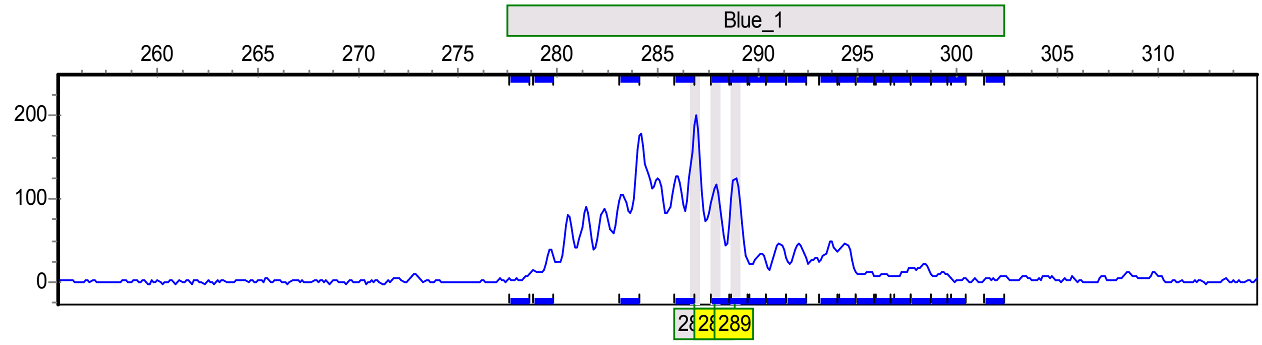

No	Size	Height	Area	Marker	Allele	Difference	Quality	Score	Allele Comments	Sample Comments
1	286.9	201	1295	Blue_1	286	0.5	Pass	7.7		
2	287.9	117	814	Blue_1	288	0.3	Check	2.0		
3	288.9	125	832	Blue_1	289	0.2	Check	3.6		

Sample 2: Run date and time: 09/11/2020 - 20:27:01 -> 09/11/2020 - 21:12:55

Dye: Blue - 2 peaks - 10.fsa

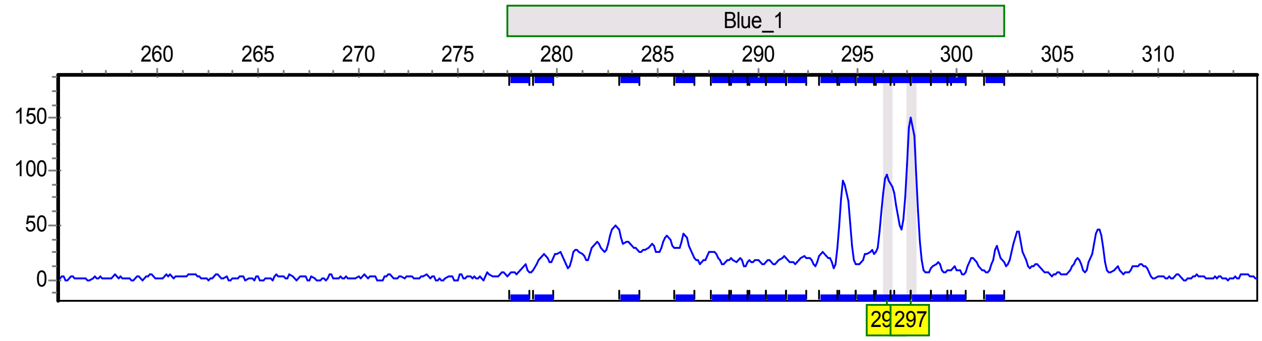

No	Size	Height	Area	Marker	Allele	Difference	Quality	Score	Allele Comments	Sample Comments
1	296.5	97	844	Blue_1	296	0.1	Check	1.2		
2	297.7	150	849	Blue_1	297	0.5	Check	7.4		

Sample 3: Run date and time: 09/11/2020 - 20:27:01 -> 09/11/2020 - 21:12:55

Dye: Blue - 2 peaks - 11.fsa

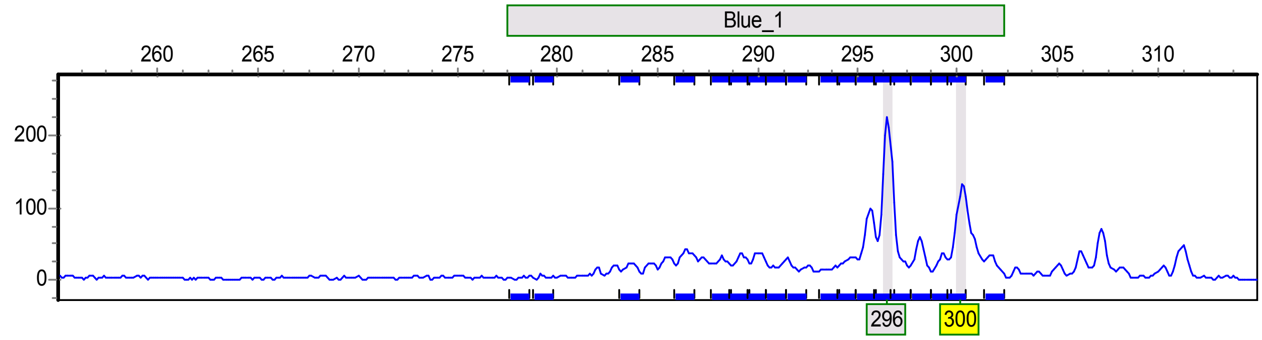

No	Size	Height	Area	Marker	Allele	Difference	Quality	Score	Allele Comments	Sample Comments
1	296.5	225	1204	Blue_1	296	0.1	Pass	15.8		
2	300.2	131	926	Blue_1	300	0.2	Check	3.4		

Sample 4: Run date and time: 09/11/2020 - 20:27:01 -> 09/11/2020 - 21:12:55

Dye: Blue - 0 peaks - 12.fsa

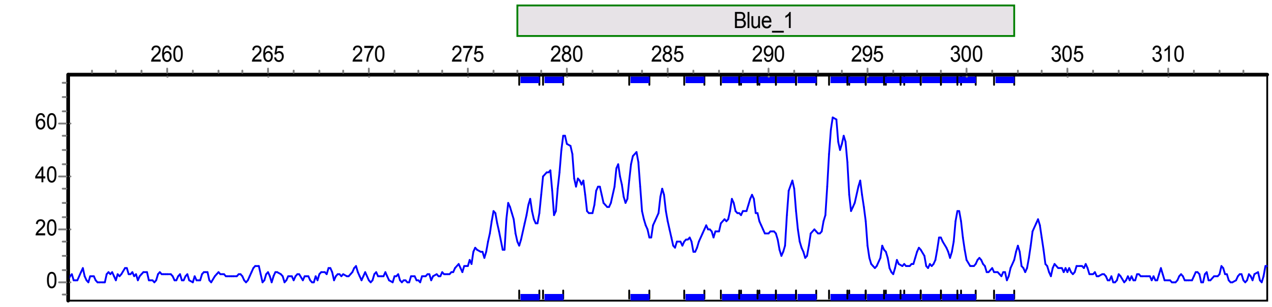

No	Size	Height	Area	Marker	Allele	Difference	Quality	Score	Allele Comments	Sample Comments

Sample 5: Run date and time: 09/11/2020 - 20:27:01 -> 09/11/2020 - 21:12:55

Dye: Blue - 3 peaks - 13.fsa

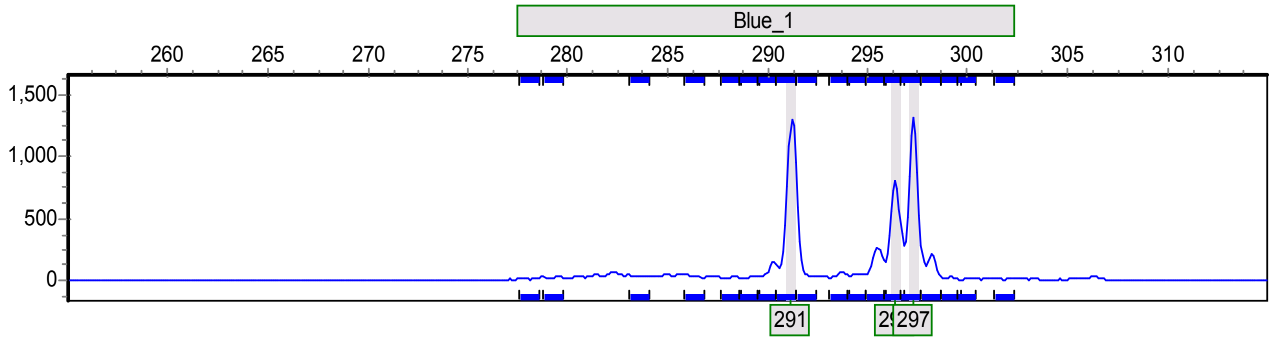

No	Size	Height	Area	Marker	Allele	Difference	Quality	Score	Allele Comments	Sample Comments
1	291.2	1294	6696	Blue_1	291	0.2	Pass	289.4		
2	296.4	805	4680	Blue_1	296	0.0	Pass	116.8		
3	297.3	1307	6865	Blue_1	297	0.1	Pass	287.6		

Sample 6: Run date and time: 09/11/2020 - 20:27:01 -> 09/11/2020 - 21:12:55

Dye: Blue - 1 peaks - 14.fsa

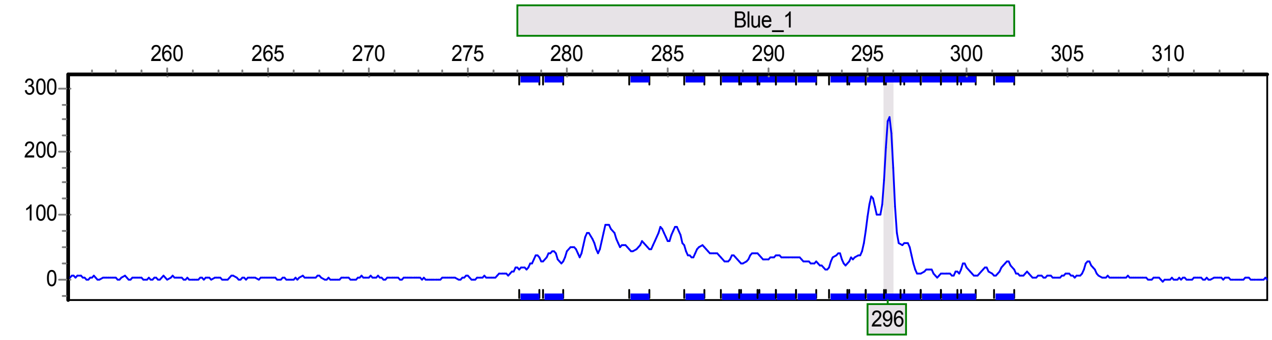

No	Size	Height	Area	Marker	Allele	Difference	Quality	Score	Allele Comments	Sample Comments
1	296.1	254	1561	Blue_1	296	0.3	Pass	14.6		

Sample 7: Run date and time: 09/11/2020 - 20:27:01 -> 09/11/2020 - 21:12:55

Dye: Blue - 2 peaks - 15.fsa

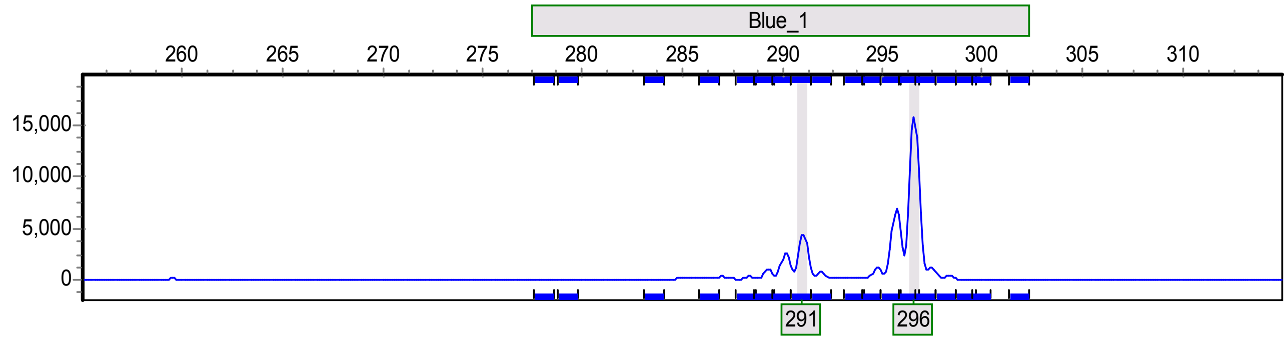

No	Size	Height	Area	Marker	Allele	Difference	Quality	Score	Allele Comments	Sample Comments
1	291.0	4416	23100	Blue_1	291	0.0	Pass	500.0		
2	296.6	15647	83074	Blue_1	296	0.2	Pass	500.0		

Sample 8: Run date and time: 09/11/2020 - 20:27:01 -> 09/11/2020 - 21:12:55

Dye: Blue - 2 peaks - 16.fsa

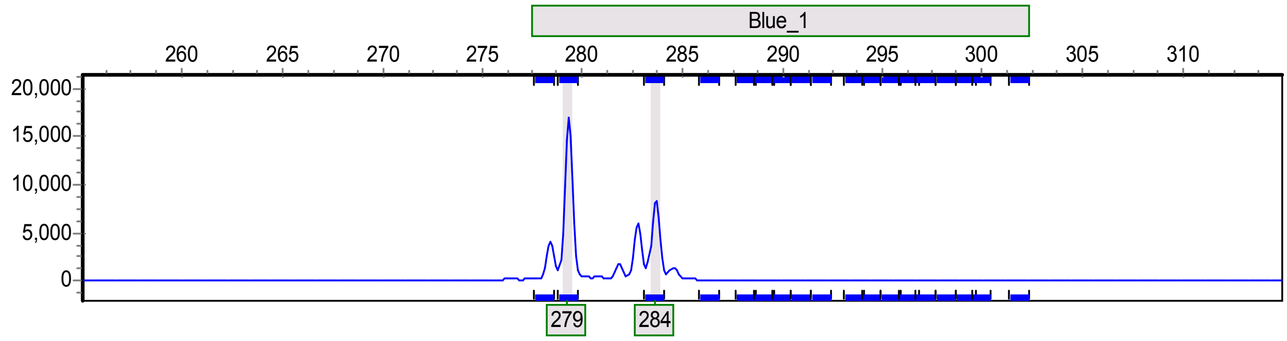

No	Size	Height	Area	Marker	Allele	Difference	Quality	Score	Allele Comments	Sample Comments
1	279.3	16895	78207	Blue_1	279	0.0	Pass	500.0		
2	283.7	8218	40612	Blue_1	284	0.1	Pass	500.0		

Sample 9: Run date and time: 09/11/2020 - 20:27:01 -> 09/11/2020 - 21:12:55

Dye: Blue - 4 peaks - 17.fsa

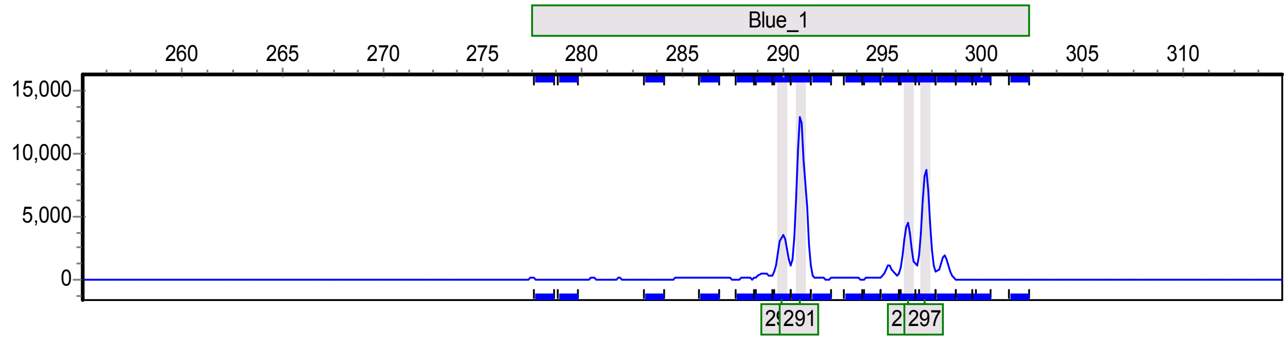

No	Size	Height	Area	Marker	Allele	Difference	Quality	Score	Allele Comments	Sample Comments
1	290.0	3515	18600	Blue_1	290	0.0	Pass	500.0		
2	290.9	12784	62252	Blue_1	291	0.1	Pass	500.0		
3	296.3	4463	22132	Blue_1	296	0.1	Pass	500.0		
4	297.2	8627	42072	Blue_1	297	0.0	Pass	500.0		

Allele Report

Sample 10: Run date and time: 09/11/2020 - 20:27:01 -> 09/11/2020 - 21:12:55

Dye: Blue - 6 peaks - 18.fsa

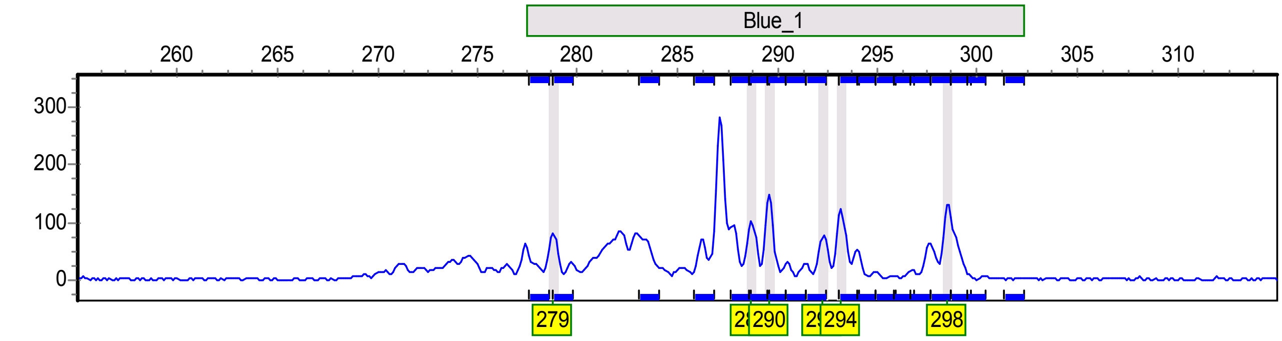

No	Size	Height	Area	Marker	Allele	Difference	Quality	Score	Allele Comments	Sample Comments
1	278.8	82	419	Blue_1	279	0.5	Check	3.1		
2	288.7	104	564	Blue_1	289	0.4	Check	4.2		
3	289.6	147	725	Blue_1	290	0.4	Check	9.3		
4	292.3	78	411	Blue_1	292	0.3	Check	2.6		
5	293.2	125	637	Blue_1	294	0.4	Check	6.5		
6	298.5	132	926	Blue_1	298	0.3	Check	5.0		

Sample 11: Run date and time: 09/11/2020 - 20:27:01 -> 09/11/2020 - 21:12:55

Dye: Blue - 1 peaks - 19.fsa

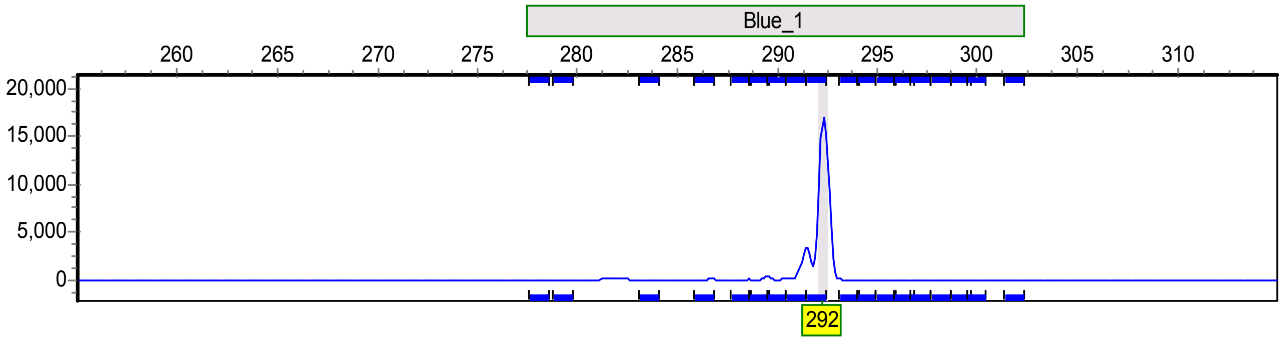

No	Size	Height	Area	Marker	Allele	Difference	Quality	Score	Allele Comments	Sample Comments
1	292.3	16885	88481	Blue_1	292	0.3	Check	500.0	[<SAT (Repaired)>]	

Sample 12: Run date and time: 09/11/2020 - 20:27:01 -> 09/11/2020 - 21:12:55

Dye: Blue - 2 peaks - 2.fsa

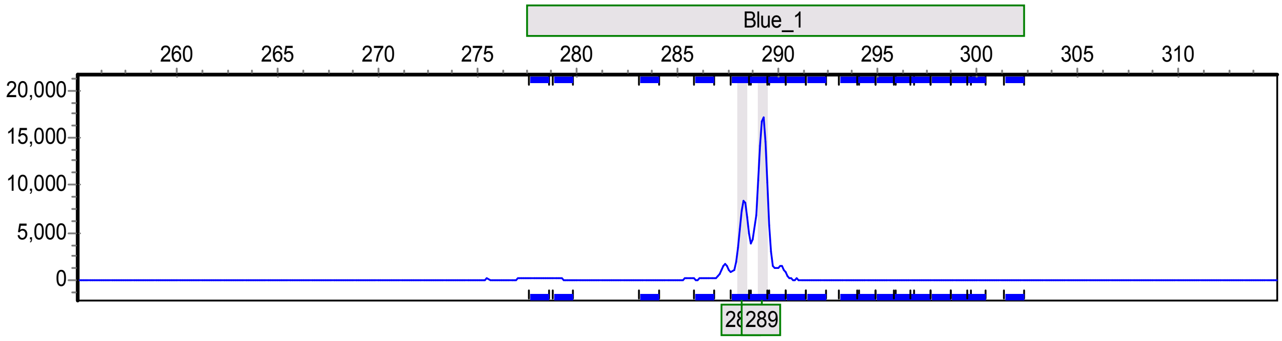

No	Size	Height	Area	Marker	Allele	Difference	Quality	Score	Allele Comments	Sample Comments
1	288.3	8451	49818	Blue_1	288	0.1	Pass	500.0		
2	289.3	17014	101145	Blue_1	289	0.2	Pass	500.0		

Sample 13: Run date and time: 09/11/2020 - 20:27:01 -> 09/11/2020 - 21:12:55

Dye: Blue - 2 peaks - 20.fsa

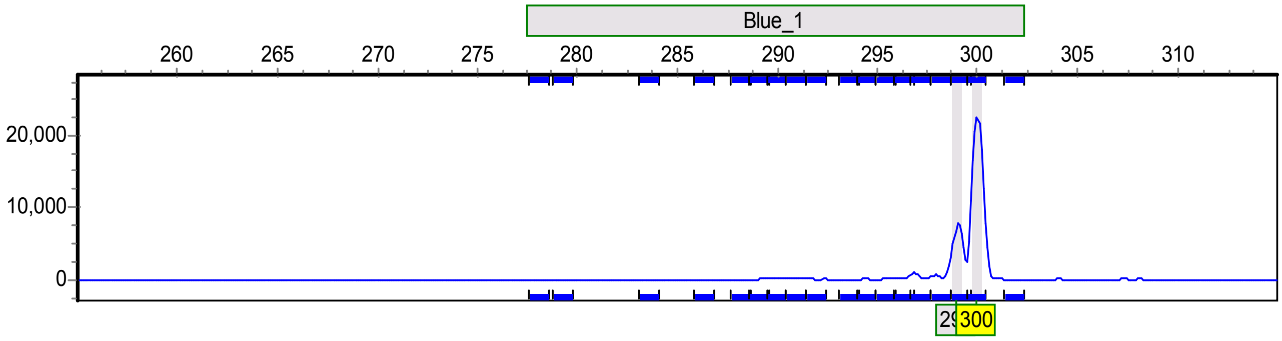

No	Size	Height	Area	Marker	Allele	Difference	Quality	Score	Allele Comments	Sample Comments
1	299.0	7845	45895	Blue_1	299	0.2	Pass	500.0		
2	300.0	22349	137492	Blue_1	300	0.0	Check	500.0	[<SAT (Repaired)>]	

Sample 14: Run date and time: 09/11/2020 - 20:27:01 -> 09/11/2020 - 21:12:55

Dye: Blue - 3 peaks - 21.fsa

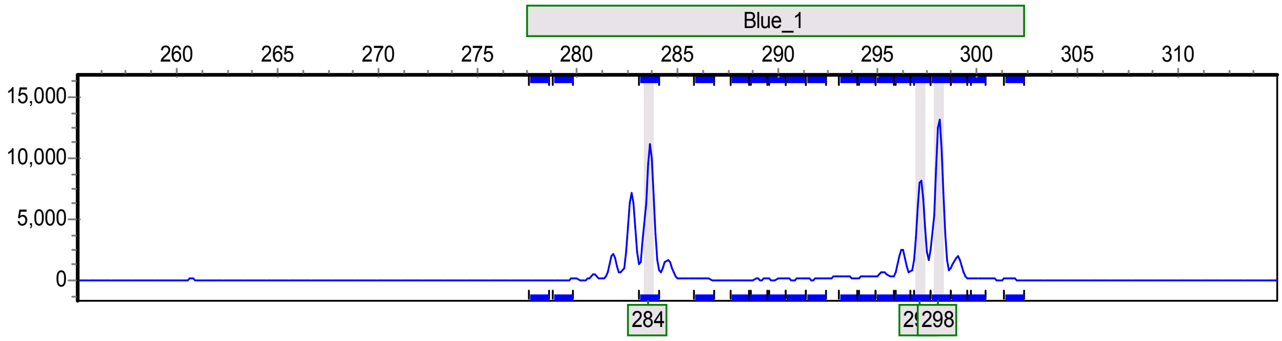

No	Size	Height	Area	Marker	Allele	Difference	Quality	Score	Allele Comments	Sample Comments
1	283.6	11211	51790	Blue_1	284	0.0	Pass	500.0		
2	297.2	8274	40261	Blue_1	297	0.0	Pass	500.0		
3	298.1	13307	63445	Blue_1	298	0.1	Pass	500.0		

Sample 15: Run date and time: 09/11/2020 - 20:27:01 -> 09/11/2020 - 21:12:55

Dye: Blue - 1 peaks - 22.fsa

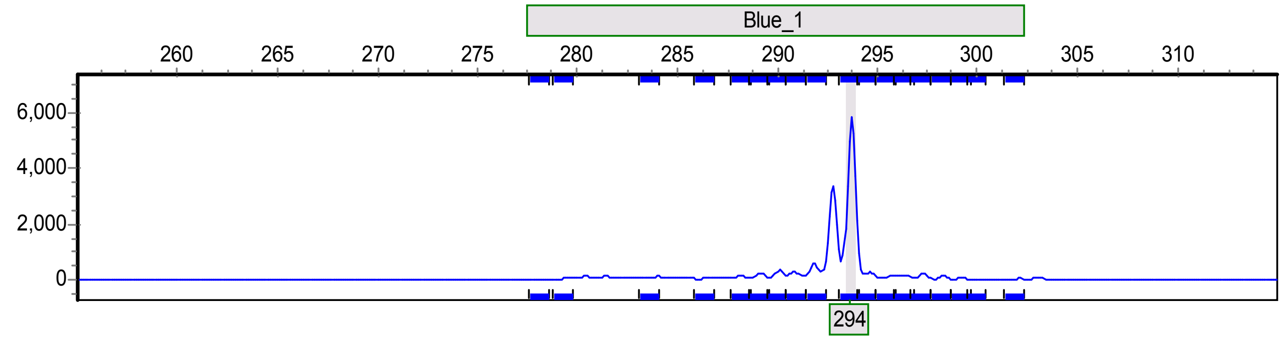

No	Size	Height	Area	Marker	Allele	Difference	Quality	Score	Allele Comments	Sample Comments
1	293.7	5818	27637	Blue_1	294	0.1	Pass	500.0		

Allele Report

Sample 16: Run date and time: 09/11/2020 - 20:27:01 -> 09/11/2020 - 21:12:55

Dye: Blue - 1 peaks - 23.fsa

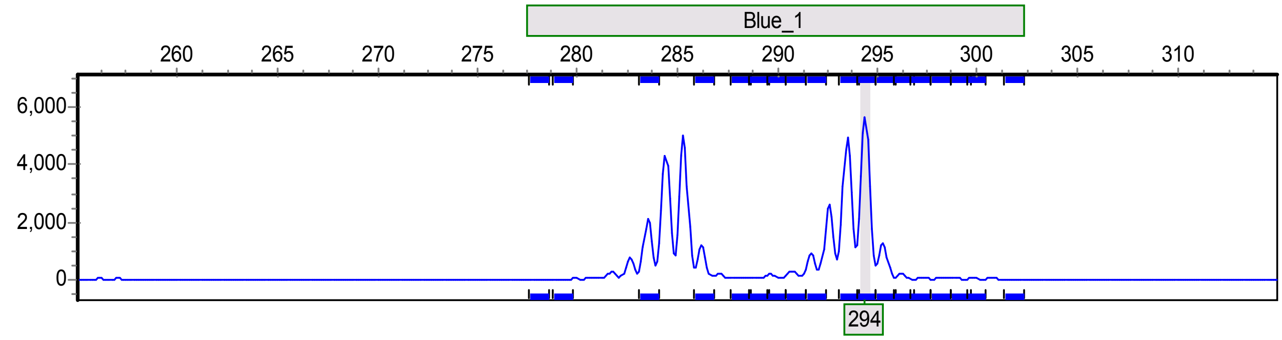

No	Size	Height	Area	Marker	Allele	Difference	Quality	Score	Allele Comments	Sample Comments
1	294.4	5607	28079	Blue_1	294	0.1	Pass	500.0		

Sample 17: Run date and time: 09/11/2020 - 20:27:01 -> 09/11/2020 - 21:12:55

Dye: Blue - 2 peaks - 24.fsa

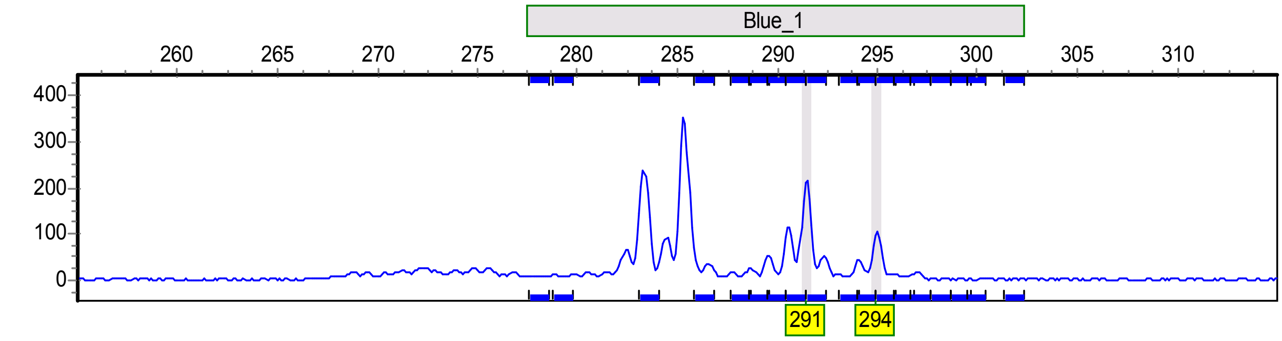

No	Size	Height	Area	Marker	Allele	Difference	Quality	Score	Allele Comments	Sample Comments
1	291.5	215	1175	Blue_1	291	0.5	Check	15.6		
2	295.0	107	588	Blue_1	294	0.5	Check	4.5		

Sample 18: Run date and time: 09/11/2020 - 20:27:01 -> 09/11/2020 - 21:12:55

Dye: Blue - 2 peaks - 25.fsa

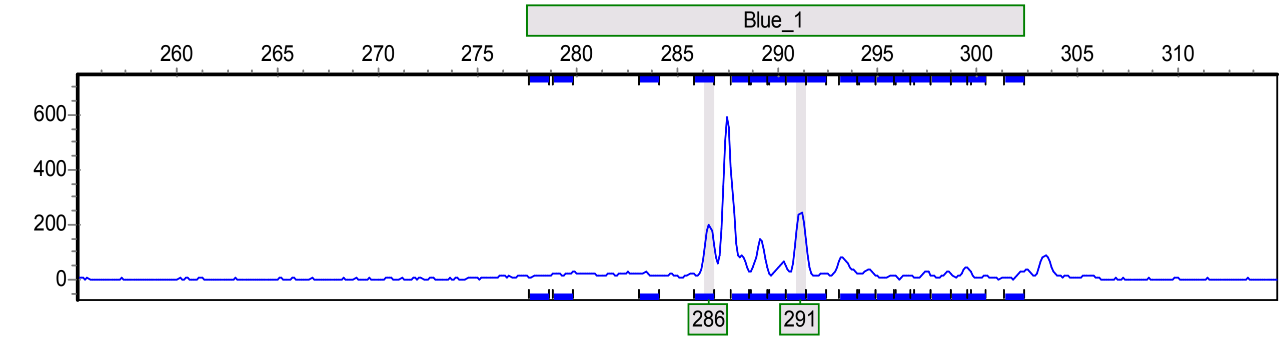

No	Size	Height	Area	Marker	Allele	Difference	Quality	Score	Allele Comments	Sample Comments
1	286.6	200	960	Blue_1	286	0.2	Pass	14.8		
2	291.2	248	1295	Blue_1	291	0.2	Pass	21.3		

Allele Report

Sample 19: Run date and time: 09/11/2020 - 20:27:01 -> 09/11/2020 - 21:12:55

Dye: Blue - 1 peaks - 26.fsa

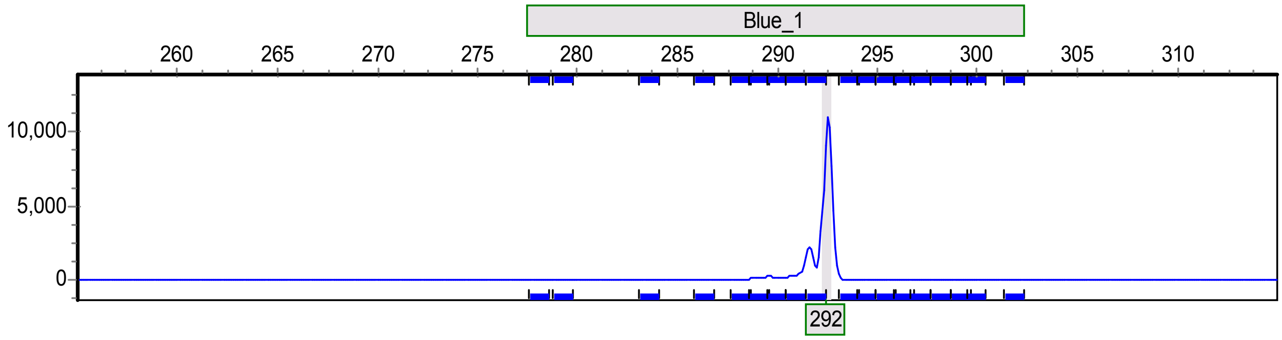

No	Size	Height	Area	Marker	Allele	Difference	Quality	Score	Allele Comments	Sample Comments
1	292.5	10897	53209	Blue_1	292	0.5	Pass	500.0		

Sample 20: Run date and time: 09/11/2020 - 20:27:01 -> 09/11/2020 - 21:12:55

Dye: Blue - 2 peaks - 27.fsa

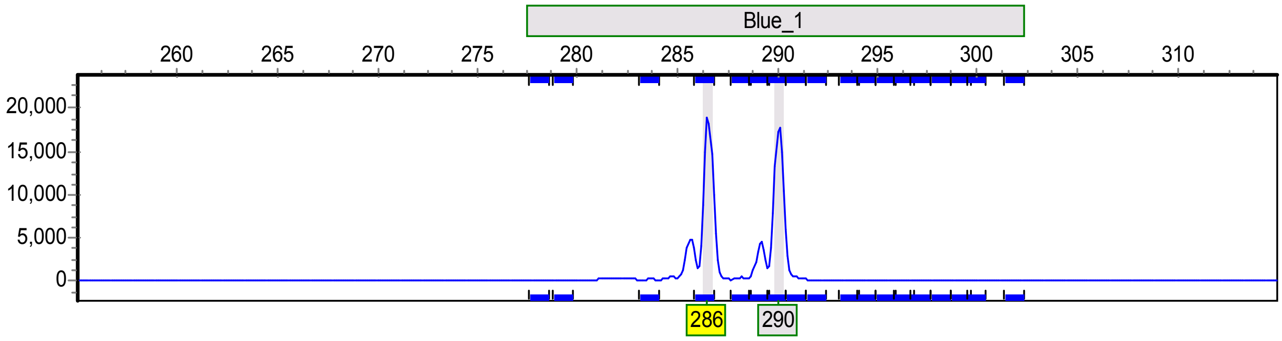

No	Size	Height	Area	Marker	Allele	Difference	Quality	Score	Allele Comments	Sample Comments
1	286.5	18799	92960	Blue_1	286	0.1	Check	500.0	[<SAT (Repaired)>]	
2	290.1	17757	90373	Blue_1	290	0.1	Pass	500.0		

Sample 21: Run date and time: 09/11/2020 - 20:27:01 -> 09/11/2020 - 21:12:55

Dye: Blue - 1 peaks - 28.fsa

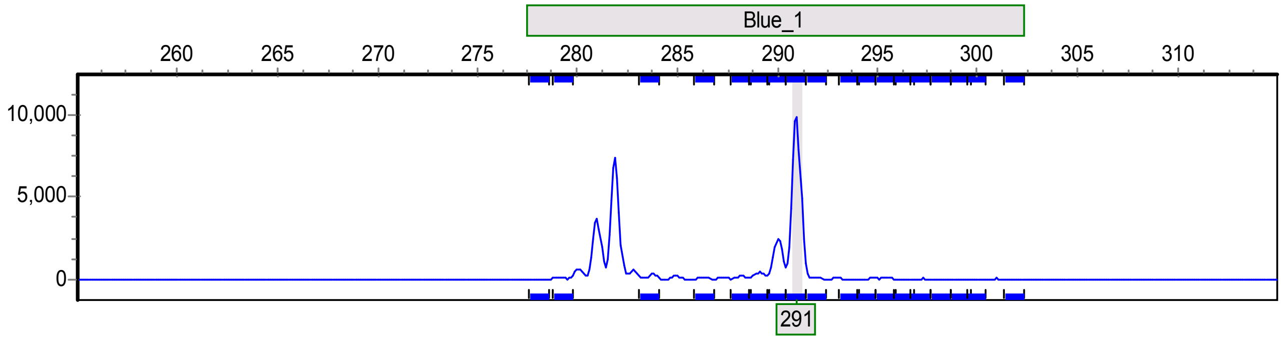

No	Size	Height	Area	Marker	Allele	Difference	Quality	Score	Allele Comments	Sample Comments
1	291.0	9764	46792	Blue_1	291	0.0	Pass	500.0		

Allele Report

Sample 22: Run date and time: 09/11/2020 - 20:27:01 -> 09/11/2020 - 21:12:55

Dye: Blue - 2 peaks - 29.fsa

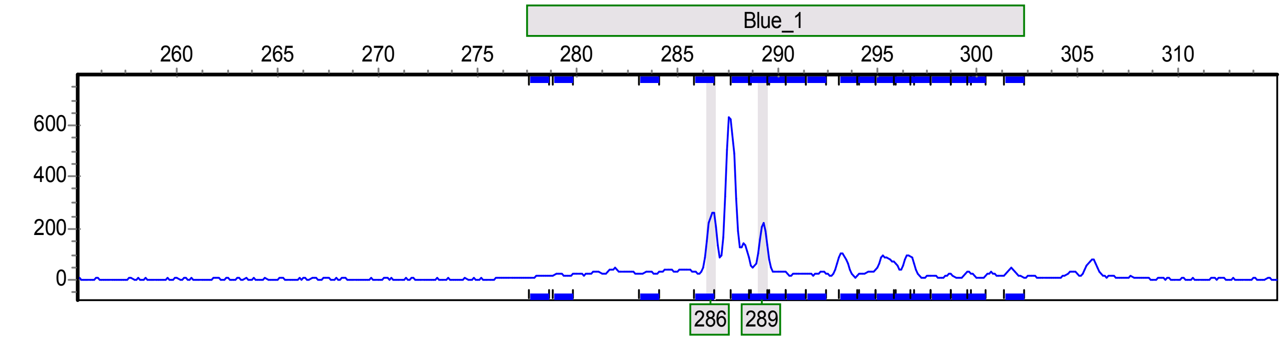

No	Size	Height	Area	Marker	Allele	Difference	Quality	Score	Allele Comments	Sample Comments
1	286.7	263	1407	Blue_1	286	0.3	Pass	21.9		
2	289.3	217	1157	Blue_1	289	0.2	Pass	16.1		

Sample 23: Run date and time: 09/11/2020 - 20:27:01 -> 09/11/2020 - 21:12:55

Dye: Blue - 4 peaks - 3.fsa

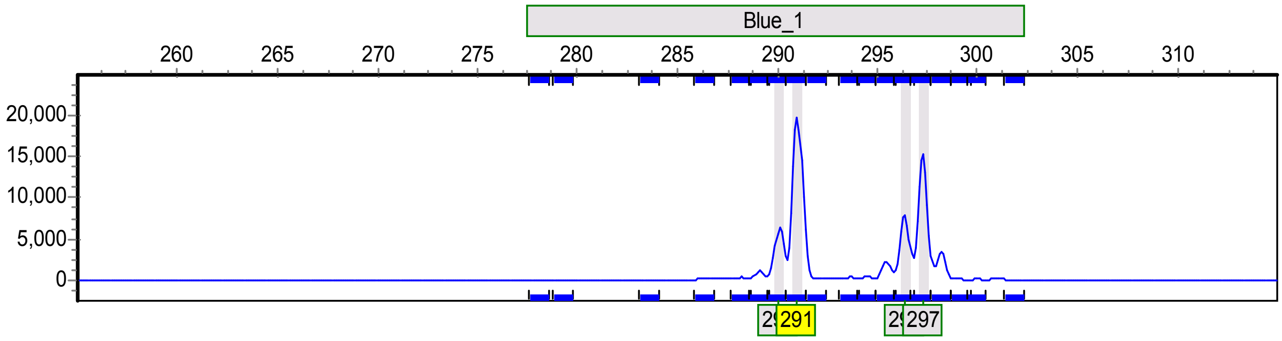

No	Size	Height	Area	Marker	Allele	Difference	Quality	Score	Allele Comments	Sample Comments
1	290.1	6329	33112	Blue_1	290	0.1	Pass	500.0		
2	291.0	19574	110814	Blue_1	291	0.0	Check	500.0	[<SAT (Repaired)>]	
3	296.4	7942	41821	Blue_1	296	0.0	Pass	500.0		
4	297.3	15145	81650	Blue_1	297	0.1	Pass	500.0		

Sample 24: Run date and time: 09/11/2020 - 20:27:01 -> 09/11/2020 - 21:12:55

Dye: Blue - 2 peaks - 30.fsa

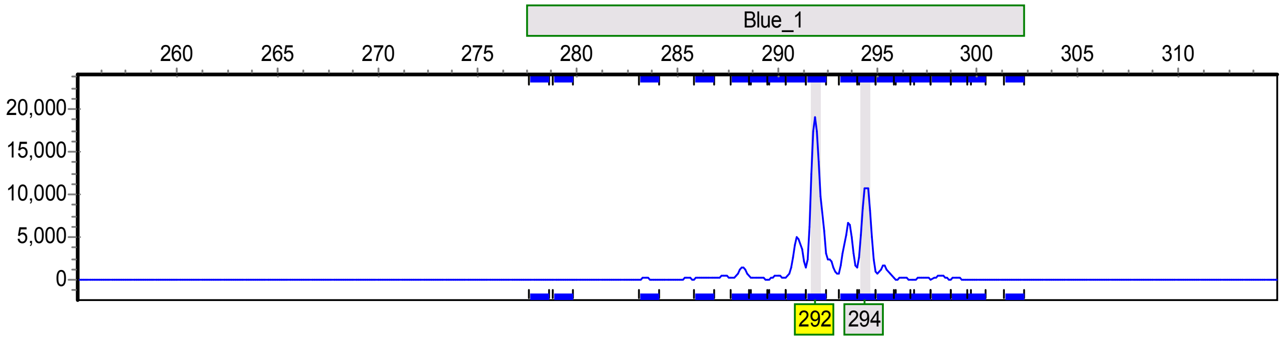

No	Size	Height	Area	Marker	Allele	Difference	Quality	Score	Allele Comments	Sample Comments
1	291.9	18939	102615	Blue_1	292	0.1	Check	500.0	[<SAT (Repaired)>]	
2	294.4	10759	52014	Blue_1	294	0.1	Pass	500.0		

Allele Report

Sample 25: Run date and time: 09/11/2020 - 20:27:01 -> 09/11/2020 - 21:12:55

Dye: Blue - 0 peaks - 31.fsa

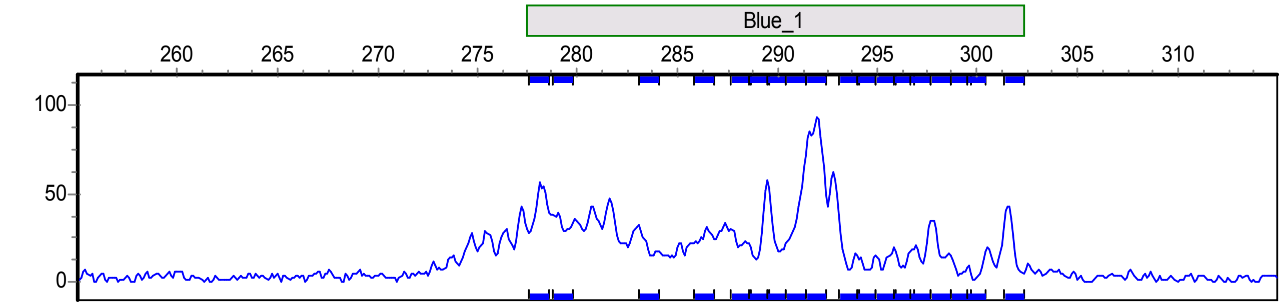

No	Size	Height	Area	Marker	Allele	Difference	Quality	Score	Allele Comments	Sample Comments

Sample 26: Run date and time: 09/11/2020 - 20:27:01 -> 09/11/2020 - 21:12:55

Dye: Blue - 3 peaks - 32.fsa

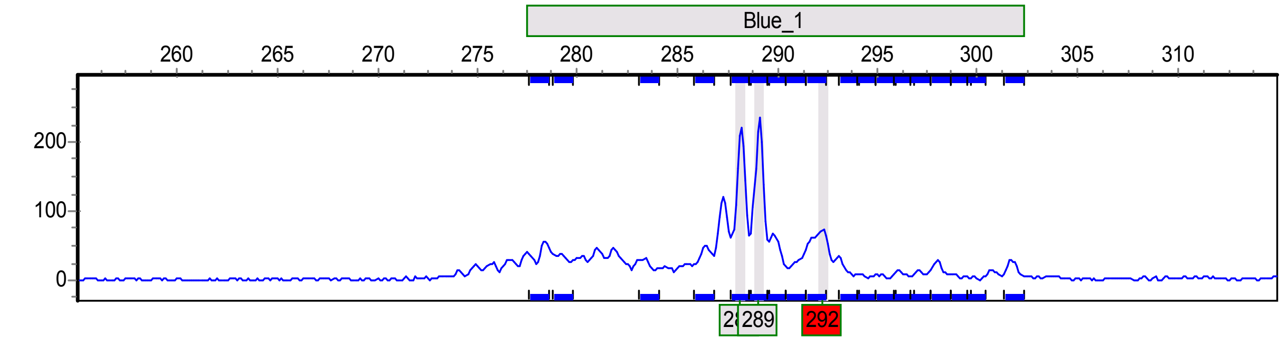

No	Size	Height	Area	Marker	Allele	Difference	Quality	Score	Allele Comments	Sample Comments
1	288.2	221	1200	Blue_1	288	0.0	Pass	14.1		
2	289.1	234	1202	Blue_1	289	0.0	Pass	17.9		
3	292.3	73	872	Blue_1	292	0.3	Undetermined	0.6		

Sample 27: Run date and time: 09/11/2020 - 20:27:01 -> 09/11/2020 - 21:12:55

Dye: Blue - 3 peaks - 33.fsa

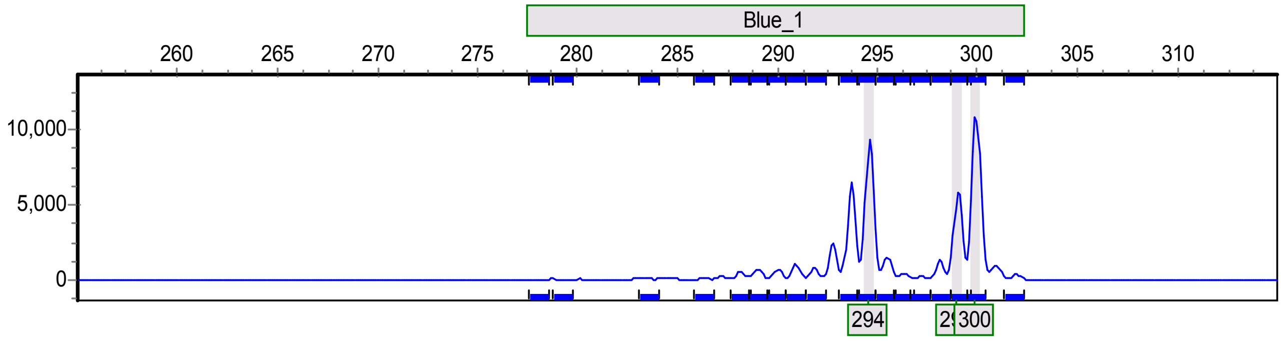

No	Size	Height	Area	Marker	Allele	Difference	Quality	Score	Allele Comments	Sample Comments
1	294.6	9228	42840	Blue_1	294	0.1	Pass	500.0		
2	299.0	5833	28793	Blue_1	299	0.2	Pass	500.0		
3	299.9	10691	53758	Blue_1	300	0.1	Pass	500.0		

Allele Report

Sample 28: Run date and time: 09/11/2020 - 20:27:01 -> 09/11/2020 - 21:12:55

Dye: Blue - 3 peaks - 34.fsa

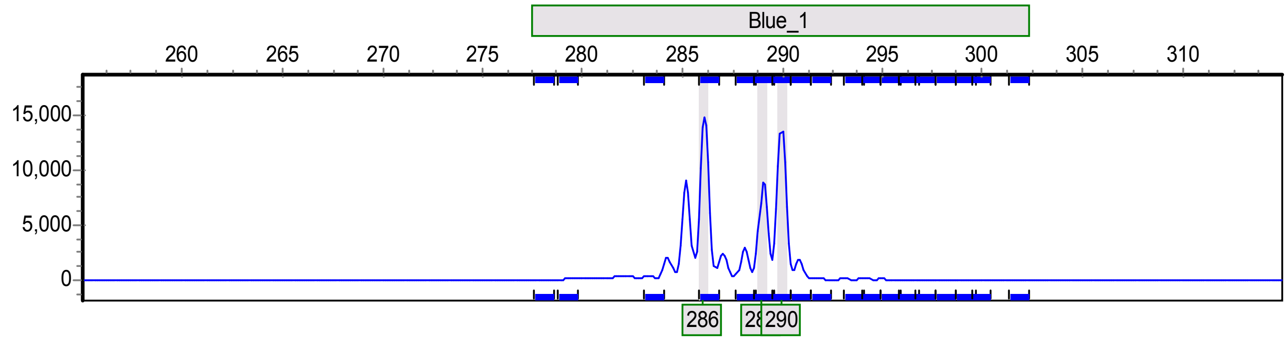

No	Size	Height	Area	Marker	Allele	Difference	Quality	Score	Allele Comments	Sample Comments
1	286.1	14778	77750	Blue_1	286	0.3	Pass	500.0		
2	289.0	8846	44445	Blue_1	289	0.1	Pass	500.0		
3	290.0	13580	66801	Blue_1	290	0.0	Pass	500.0		

Sample 29: Run date and time: 09/11/2020 - 20:27:01 -> 09/11/2020 - 21:12:55

Dye: Blue - 1 peaks - 35.fsa

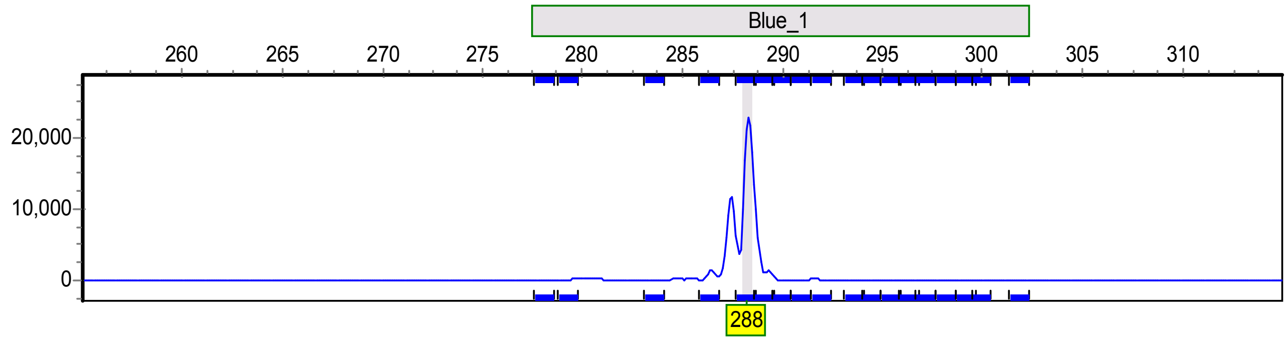

No	Size	Height	Area	Marker	Allele	Difference	Quality	Score	Allele Comments	Sample Comments
1	288.3	22805	141566	Blue_1	288	0.1	Check	500.0	[<SAT (Repaired)>]	

Sample 30: Run date and time: 09/11/2020 - 20:27:01 -> 09/11/2020 - 21:12:55

Dye: Blue - 3 peaks - 36.fsa

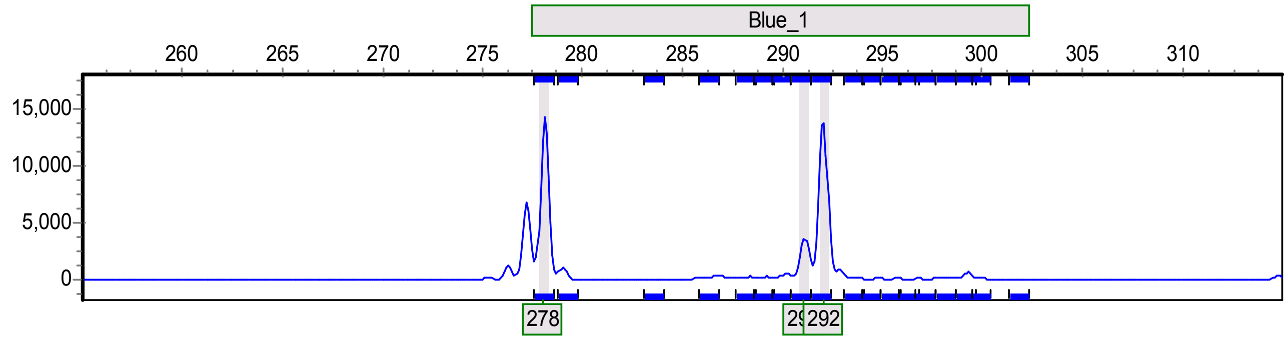

No	Size	Height	Area	Marker	Allele	Difference	Quality	Score	Allele Comments	Sample Comments
1	278.1	14151	66309	Blue_1	278	0.0	Pass	500.0		
2	291.1	3638	19059	Blue_1	291	0.1	Pass	500.0		
3	292.1	13633	67990	Blue_1	292	0.1	Pass	500.0		

Allele Report

Sample 31: Run date and time: 09/11/2020 - 20:27:01 -> 09/11/2020 - 21:12:55

Dye: Blue - 2 peaks - 37.fsa

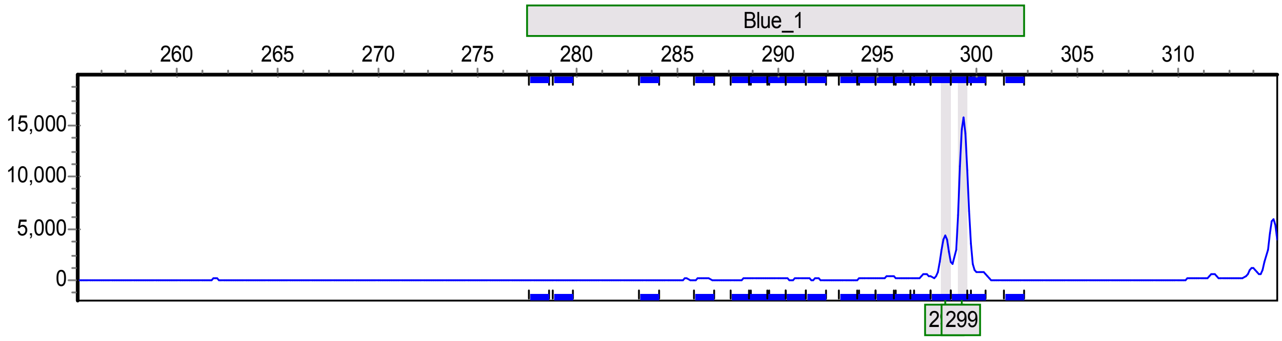

No	Size	Height	Area	Marker	Allele	Difference	Quality	Score	Allele Comments	Sample Comments
1	298.4	4379	21743	Blue_1	298	0.2	Pass	500.0		
2	299.3	15637	82531	Blue_1	299	0.1	Pass	500.0		

Sample 32: Run date and time: 09/11/2020 - 20:27:01 -> 09/11/2020 - 21:12:55

Dye: Blue - 2 peaks - 38.fsa

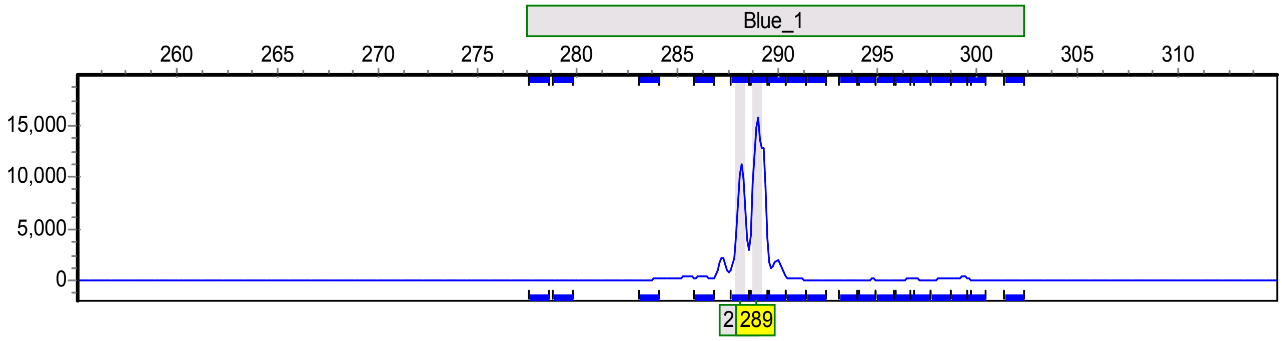

No	Size	Height	Area	Marker	Allele	Difference	Quality	Score	Allele Comments	Sample Comments
1	288.2	11215	54862	Blue_1	288	0.0	Pass	500.0		
2	289.0	15608	96914	Blue_1	289	0.1	Check	500.0	[<SAT (Repaired)>]	

Sample 33: Run date and time: 09/11/2020 - 20:27:01 -> 09/11/2020 - 21:12:55

Dye: Blue - 3 peaks - 39.fsa

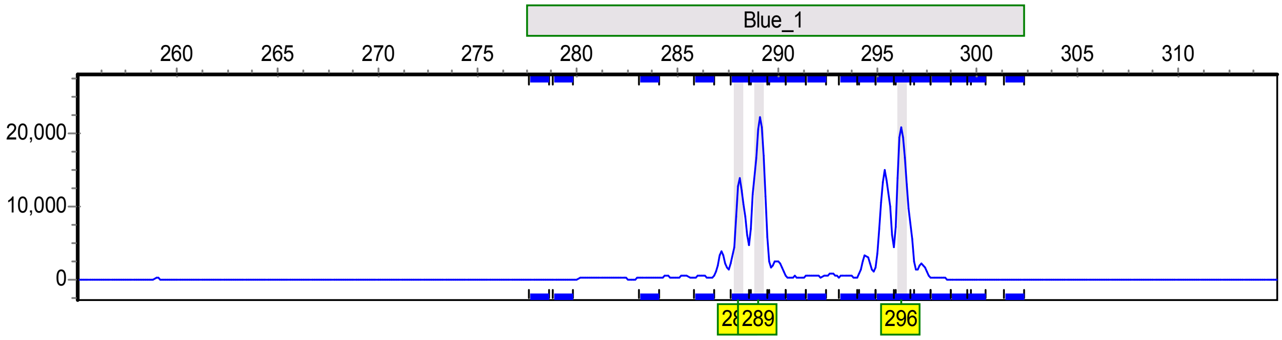

No	Size	Height	Area	Marker	Allele	Difference	Quality	Score	Allele Comments	Sample Comments
1	288.1	13907	77357	Blue_1	288	0.1	Check	500.0	[<SAT (Repaired)>]	
2	289.1	22086	131614	Blue_1	289	0.0	Check	500.0	[<SAT (Repaired)>]	
3	296.2	20741	125030	Blue_1	296	0.2	Check	500.0	[<SAT (Repaired)>]	

Allele Report

Sample 34: Run date and time: 09/11/2020 - 20:27:01 -> 09/11/2020 - 21:12:55

Dye: Blue - 1 peaks - 4.fsa

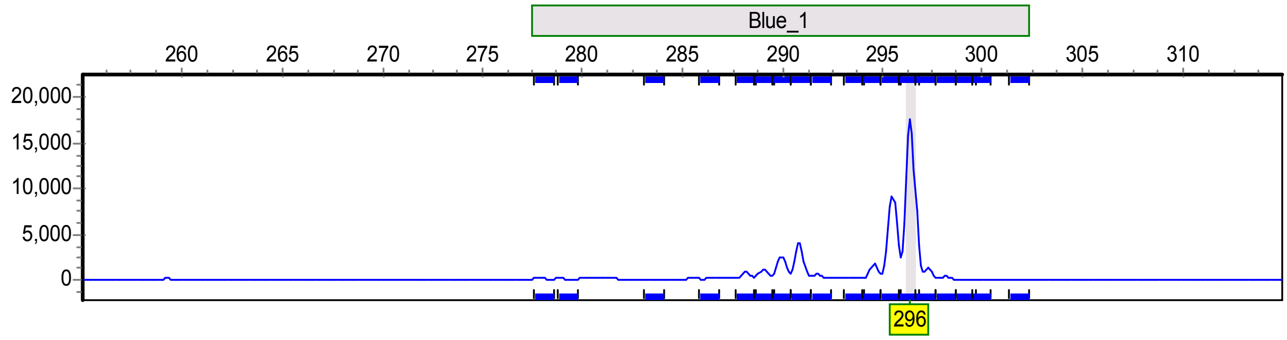

No	Size	Height	Area	Marker	Allele	Difference	Quality	Score	Allele Comments	Sample Comments
1	296.4	17750	90163	Blue_1	296	0.0	Check	500.0	[<SAT (Repaired)>]	

Sample 35: Run date and time: 09/11/2020 - 20:27:01 -> 09/11/2020 - 21:12:55

Dye: Blue - 3 peaks - 40.fsa

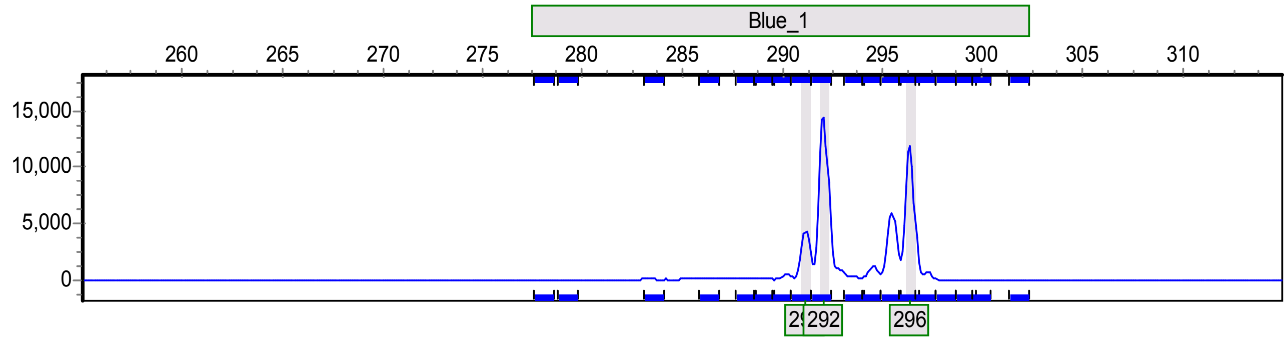

No	Size	Height	Area	Marker	Allele	Difference	Quality	Score	Allele Comments	Sample Comments
1	291.2	4292	21459	Blue_1	291	0.2	Pass	500.0		
2	292.1	14285	74362	Blue_1	292	0.1	Pass	500.0		
3	296.4	11939	59159	Blue_1	296	0.0	Pass	500.0		

Sample 36: Run date and time: 09/11/2020 - 20:27:01 -> 09/11/2020 - 21:12:55

Dye: Blue - 4 peaks - 41.fsa

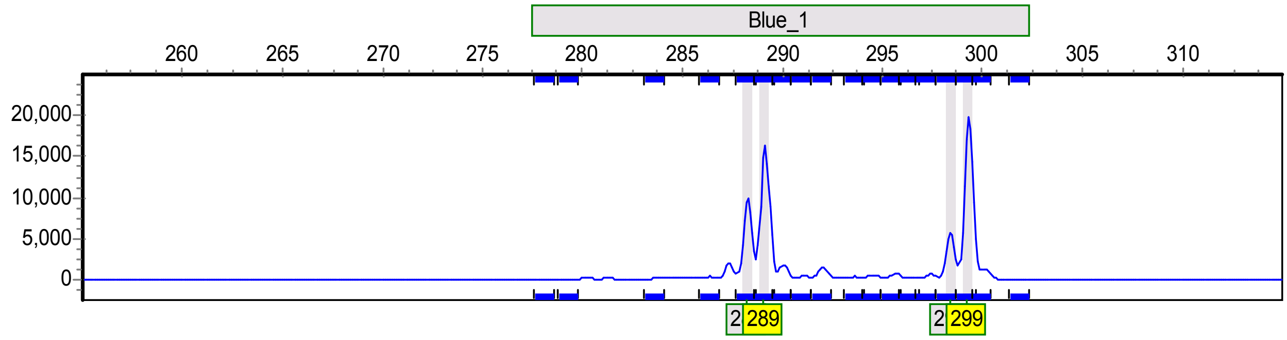

No	Size	Height	Area	Marker	Allele	Difference	Quality	Score	Allele Comments	Sample Comments
1	288.3	9782	48783	Blue_1	288	0.1	Pass	500.0		
2	289.1	16354	84514	Blue_1	289	0.0	Check	500.0	[<SAT (Repaired)>]	
3	298.4	5717	29947	Blue_1	298	0.2	Pass	500.0		
4	299.3	19654	100214	Blue_1	299	0.1	Check	500.0	[<SAT (Repaired)>]	

Sample 37: Run date and time: 09/11/2020 - 20:27:01 -> 09/11/2020 - 21:12:55

Dye: Blue - 3 peaks - 42.fsa

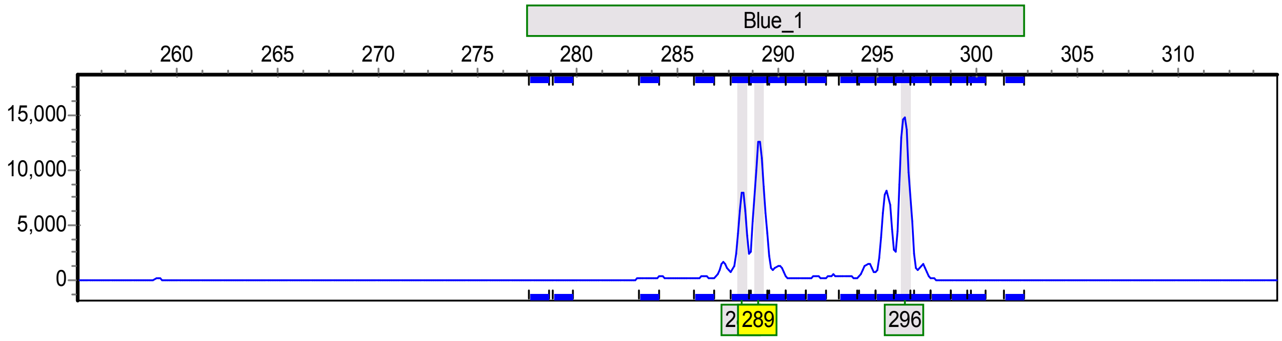

No	Size	Height	Area	Marker	Allele	Difference	Quality	Score	Allele Comments	Sample Comments
1	288.3	7879	41796	Blue_1	288	0.1	Pass	500.0		
2	289.1	12637	73561	Blue_1	289	0.0	Check	500.0	[<SAT (Repaired)>]	
3	296.4	14672	85477	Blue_1	296	0.0	Pass	500.0		

Sample 38: Run date and time: 09/11/2020 - 20:27:01 -> 09/11/2020 - 21:12:55

Dye: Blue - 4 peaks - 43.fsa

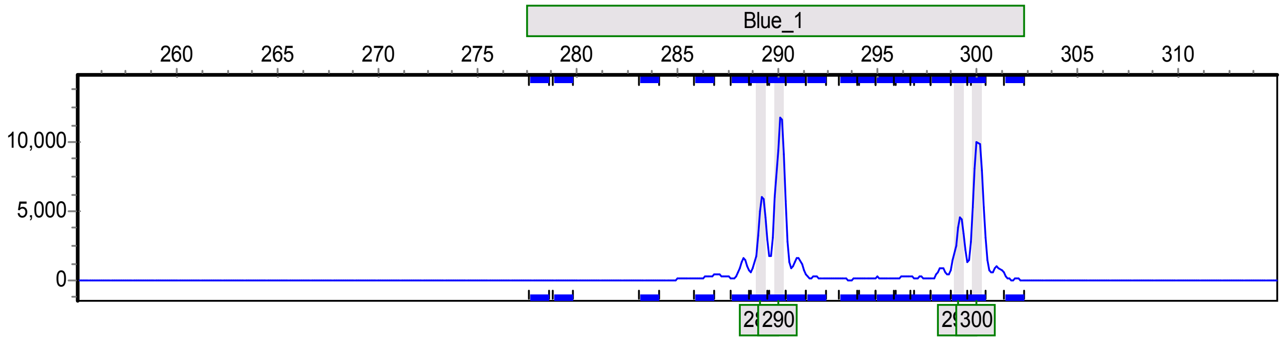

No	Size	Height	Area	Marker	Allele	Difference	Quality	Score	Allele Comments	Sample Comments
1	289.2	6093	31326	Blue_1	289	0.1	Pass	500.0		
2	290.1	11742	59331	Blue_1	290	0.1	Pass	500.0		
3	299.1	4628	23995	Blue_1	299	0.1	Pass	500.0		
4	300.0	10030	52885	Blue_1	300	0.0	Pass	500.0		

Sample 39: Run date and time: 09/11/2020 - 20:27:01 -> 09/11/2020 - 21:12:55

Dye: Blue - 2 peaks - 44.fsa

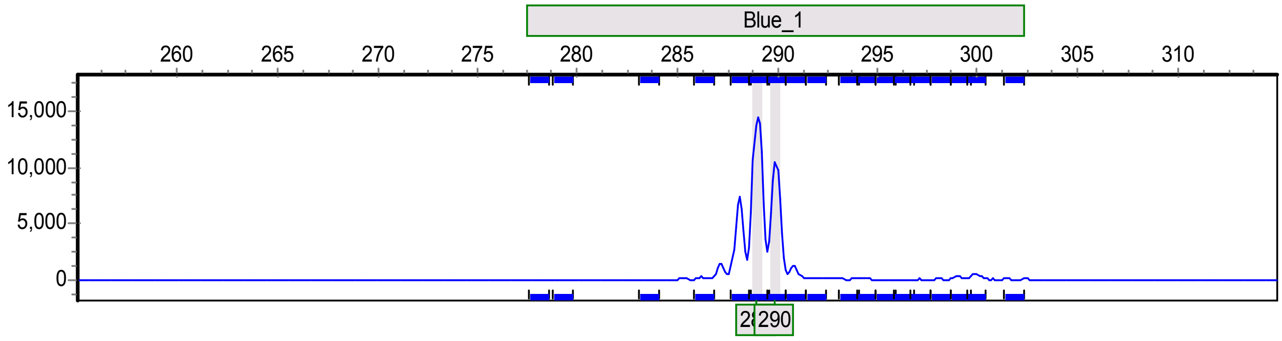

No	Size	Height	Area	Marker	Allele	Difference	Quality	Score	Allele Comments	Sample Comments
1	289.0	14418	82533	Blue_1	289	0.1	Pass	500.0		
2	289.9	10554	51187	Blue_1	290	0.1	Pass	500.0		

Allele Report

Sample 40: Run date and time: 09/11/2020 - 20:27:01 -> 09/11/2020 - 21:12:55

Dye: Blue - 2 peaks - 45.fsa

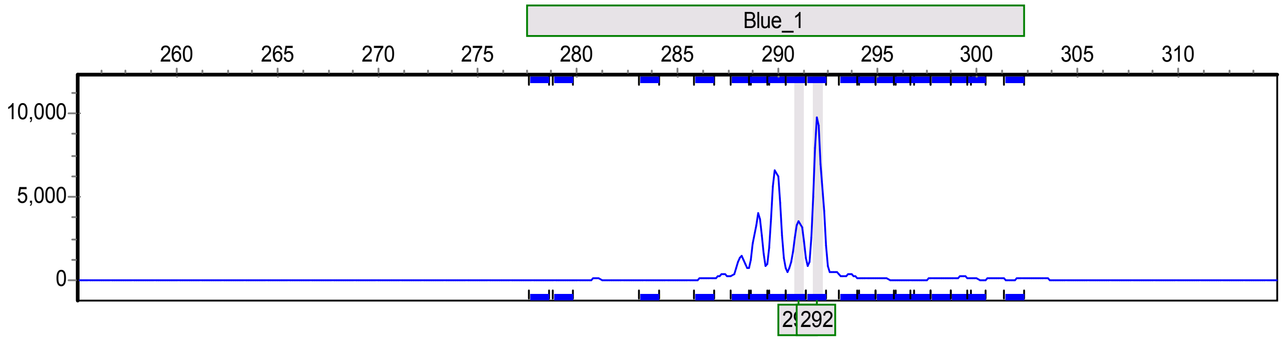

No	Size	Height	Area	Marker	Allele	Difference	Quality	Score	Allele Comments	Sample Comments
1	291.1	3555	20141	Blue_1	291	0.1	Pass	500.0		
2	292.0	9657	46302	Blue_1	292	0.0	Pass	500.0		

Sample 41: Run date and time: 09/11/2020 - 20:27:01 -> 09/11/2020 - 21:12:55

Dye: Blue - 3 peaks - 46.fsa

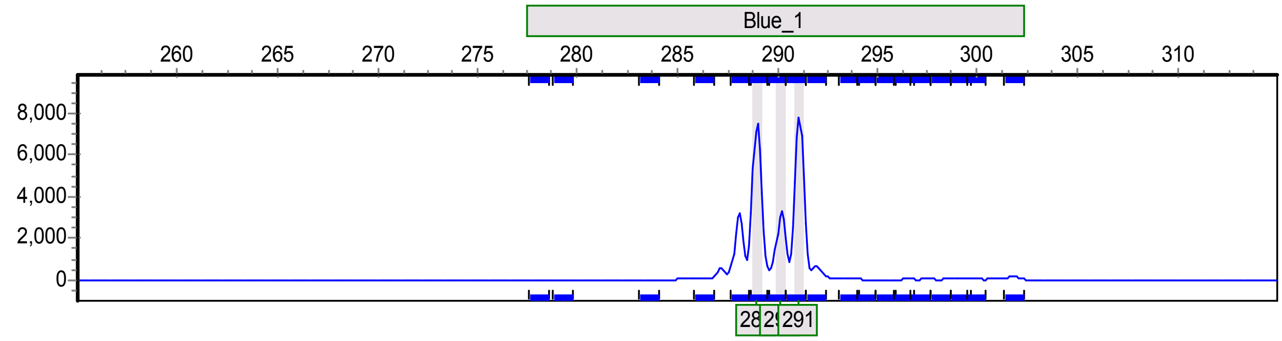

No	Size	Height	Area	Marker	Allele	Difference	Quality	Score	Allele Comments	Sample Comments
1	289.0	7491	37206	Blue_1	289	0.1	Pass	500.0		
2	290.2	3342	17316	Blue_1	290	0.2	Pass	500.0		
3	291.1	7722	36801	Blue_1	291	0.1	Pass	500.0		

Sample 42: Run date and time: 09/11/2020 - 20:27:01 -> 09/11/2020 - 21:12:55

Dye: Blue - 3 peaks - 47.fsa

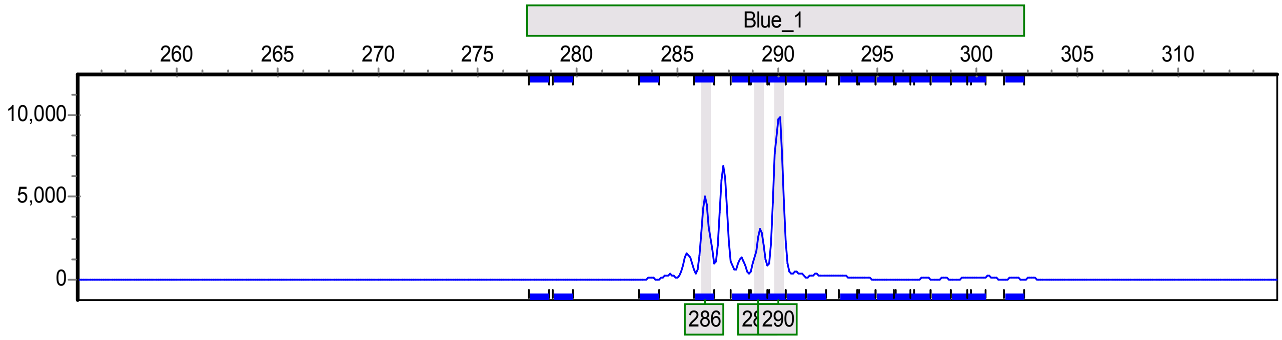

No	Size	Height	Area	Marker	Allele	Difference	Quality	Score	Allele Comments	Sample Comments
1	286.4	5012	24259	Blue_1	286	0.0	Pass	500.0		
2	289.1	3019	15687	Blue_1	289	0.0	Pass	500.0		
3	290.1	9769	47600	Blue_1	290	0.1	Pass	500.0		

Allele Report

Sample 43: Run date and time: 09/11/2020 - 20:27:01 -> 09/11/2020 - 21:12:55

Dye: Blue - 3 peaks - 48.fsa

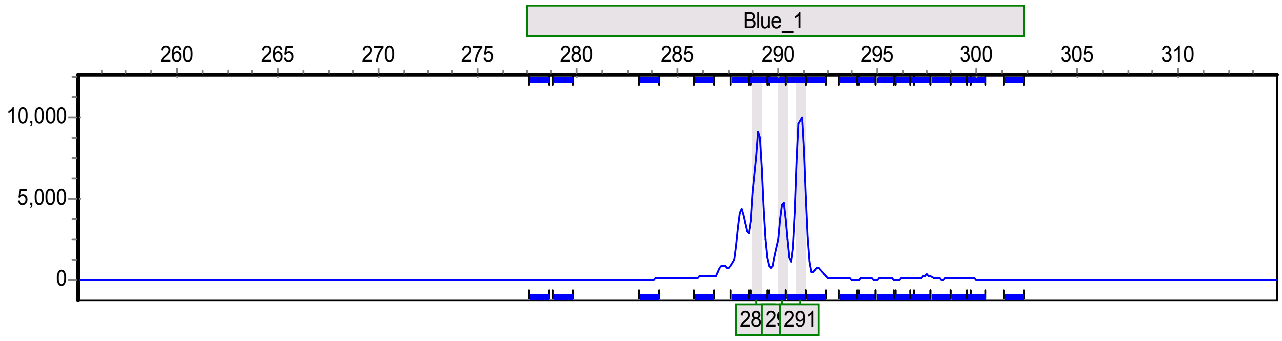

No	Size	Height	Area	Marker	Allele	Difference	Quality	Score	Allele Comments	Sample Comments
1	289.0	9109	47860	Blue_1	289	0.1	Pass	500.0		
2	290.3	4697	25069	Blue_1	290	0.3	Pass	500.0		
3	291.2	9904	47454	Blue_1	291	0.2	Pass	500.0		

Sample 44: Run date and time: 09/11/2020 - 20:27:01 -> 09/11/2020 - 21:12:55

Dye: Blue - 2 peaks - 49.fsa

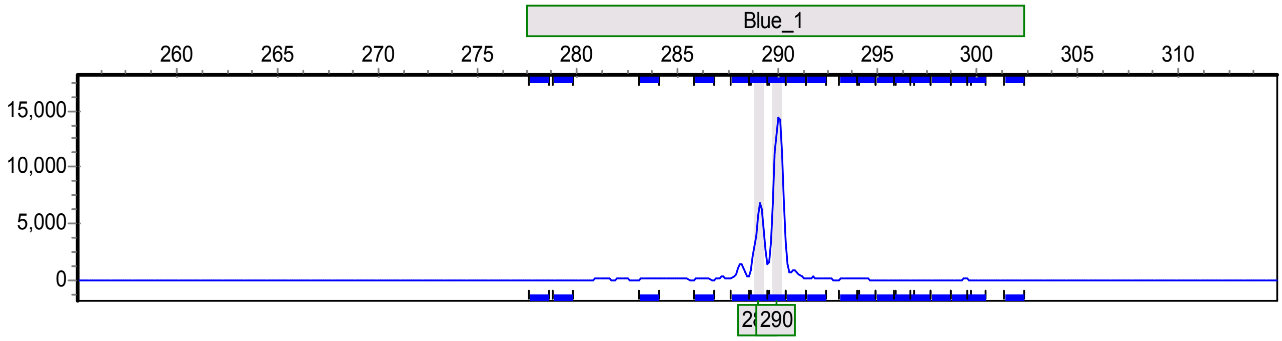

No	Size	Height	Area	Marker	Allele	Difference	Quality	Score	Allele Comments	Sample Comments
1	289.1	6787	33498	Blue_1	289	0.0	Pass	500.0		
2	290.0	14305	70456	Blue_1	290	0.0	Pass	500.0		

Sample 45: Run date and time: 09/11/2020 - 20:27:01 -> 09/11/2020 - 21:12:55

Dye: Blue - 4 peaks - 5.fsa

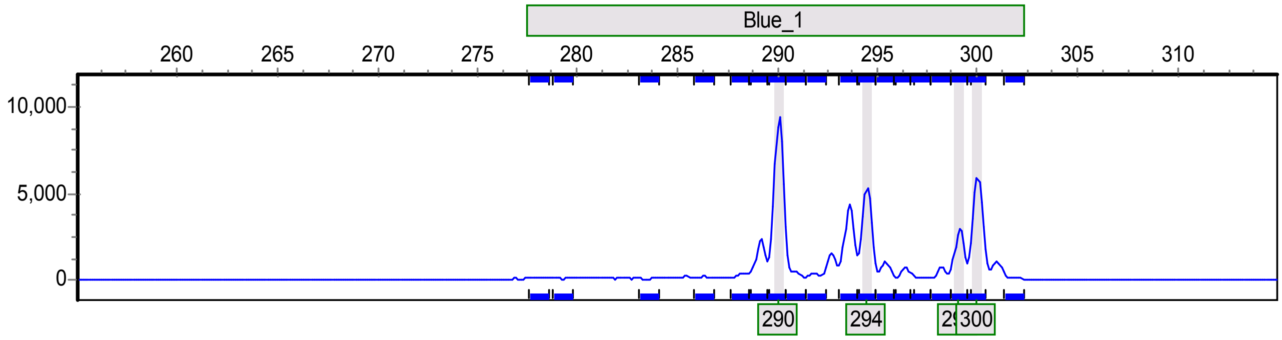

No	Size	Height	Area	Marker	Allele	Difference	Quality	Score	Allele Comments	Sample Comments
1	290.1	9377	48269	Blue_1	290	0.1	Pass	500.0		
2	294.5	5356	28053	Blue_1	294	0.0	Pass	500.0		
3	299.1	3007	16243	Blue_1	299	0.1	Pass	500.0		
4	300.0	5885	32790	Blue_1	300	0.0	Pass	500.0		

Allele Report

Sample 46: Run date and time: 09/11/2020 - 20:27:01 -> 09/11/2020 - 21:12:55

Dye: Blue - 2 peaks - 50.fsa

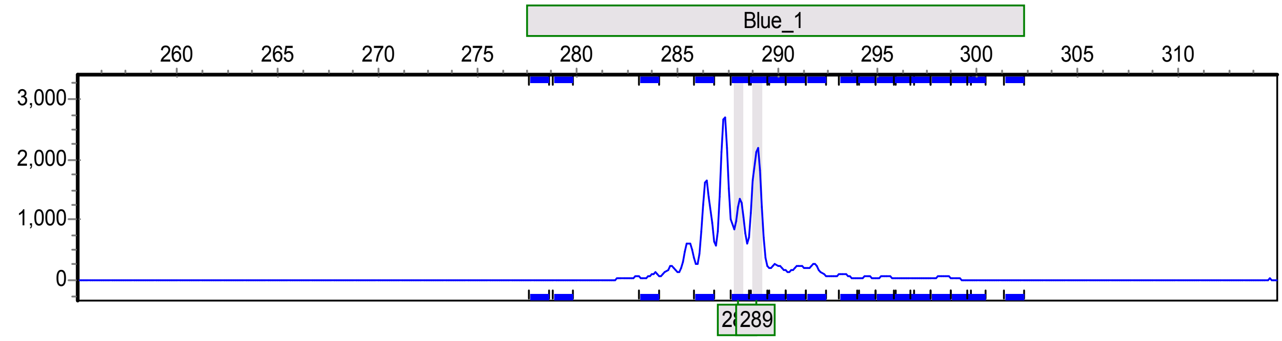

No	Size	Height	Area	Marker	Allele	Difference	Quality	Score	Allele Comments	Sample Comments
1	288.1	1344	8155	Blue_1	288	0.1	Pass	117.2		
2	289.0	2188	11609	Blue_1	289	0.1	Pass	500.0		

Sample 47: Run date and time: 09/11/2020 - 20:27:01 -> 09/11/2020 - 21:12:55

Dye: Blue - 2 peaks - 51.fsa

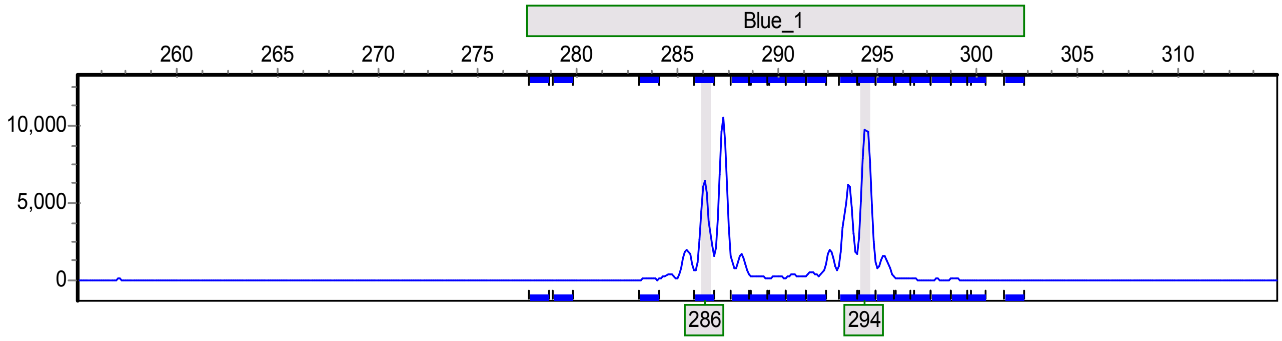

No	Size	Height	Area	Marker	Allele	Difference	Quality	Score	Allele Comments	Sample Comments
1	286.4	6550	31905	Blue_1	286	0.0	Pass	500.0		
2	294.4	9786	50586	Blue_1	294	0.1	Pass	500.0		

Sample 48: Run date and time: 09/11/2020 - 20:27:01 -> 09/11/2020 - 21:12:55

Dye: Blue - 2 peaks - 52.fsa

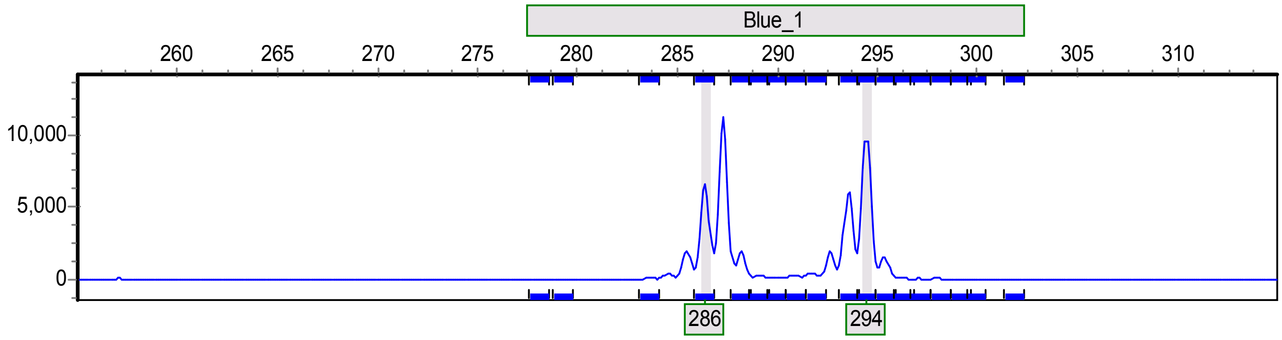

No	Size	Height	Area	Marker	Allele	Difference	Quality	Score	Allele Comments	Sample Comments
1	286.4	6607	33514	Blue_1	286	0.0	Pass	500.0		
2	294.5	9545	50703	Blue_1	294	0.0	Pass	500.0		

Sample 49: Run date and time: 09/11/2020 - 20:27:01 -> 09/11/2020 - 21:12:55

Dye: Blue - 5 peaks - 53.fsa

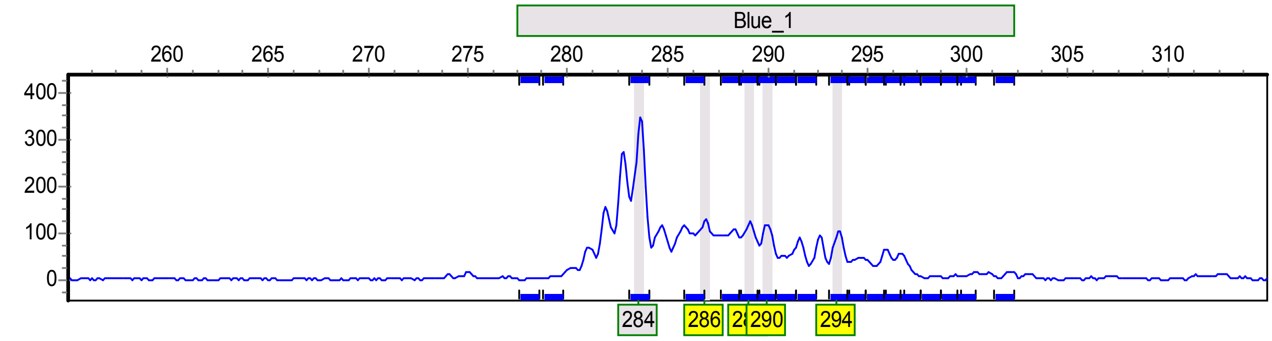

No	Size	Height	Area	Marker	Allele	Difference	Quality	Score	Allele Comments	Sample Comments
1	283.6	346	2137	Blue_1	284	0.0	Pass	20.7		
2	286.9	131	899	Blue_1	286	0.5	Check	1.1		
3	289.1	124	916	Blue_1	289	0.0	Check	1.2		
4	290.0	118	739	Blue_1	290	0.0	Check	2.0		
5	293.5	104	660	Blue_1	294	0.1	Check	2.6		

Sample 50: Run date and time: 09/11/2020 - 20:27:01 -> 09/11/2020 - 21:12:55

Dye: Blue - 3 peaks - 54.fsa

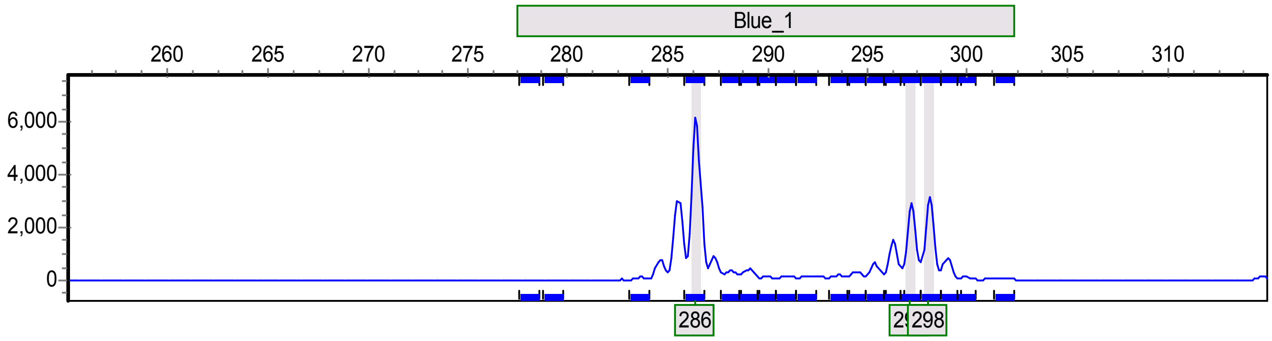

No	Size	Height	Area	Marker	Allele	Difference	Quality	Score	Allele Comments	Sample Comments
1	286.4	6103	30337	Blue_1	286	0.0	Pass	500.0		
2	297.2	2892	15269	Blue_1	297	0.0	Pass	500.0		
3	298.1	3158	16407	Blue_1	298	0.1	Pass	500.0		

Sample 51: Run date and time: 09/11/2020 - 20:27:01 -> 09/11/2020 - 21:12:55

Dye: Blue - 3 peaks - 55.fsa

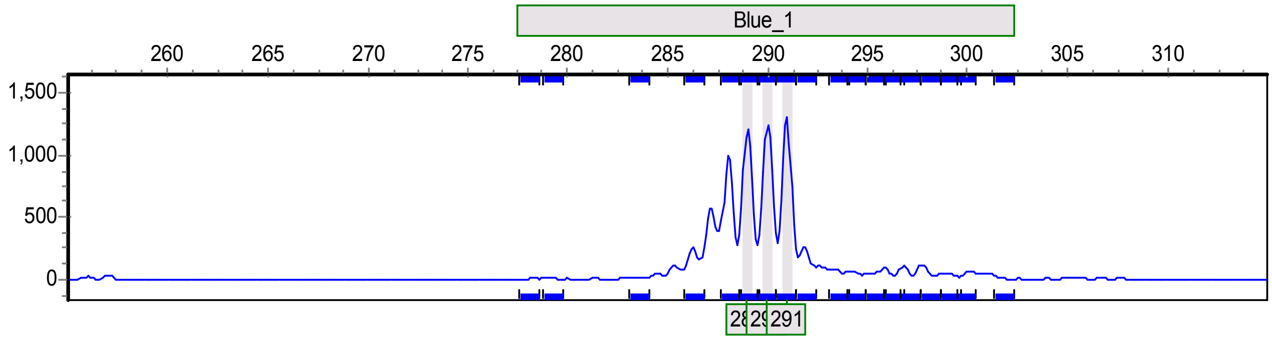

No	Size	Height	Area	Marker	Allele	Difference	Quality	Score	Allele Comments	Sample Comments
1	289.0	1209	6867	Blue_1	289	0.1	Pass	222.6		
2	290.0	1238	7114	Blue_1	290	0.0	Pass	224.3		
3	291.0	1297	6906	Blue_1	291	0.0	Pass	273.1		

Allele Report

Sample 52: Run date and time: 09/11/2020 - 20:27:01 -> 09/11/2020 - 21:12:55

Dye: Blue - 5 peaks - 56.fsa

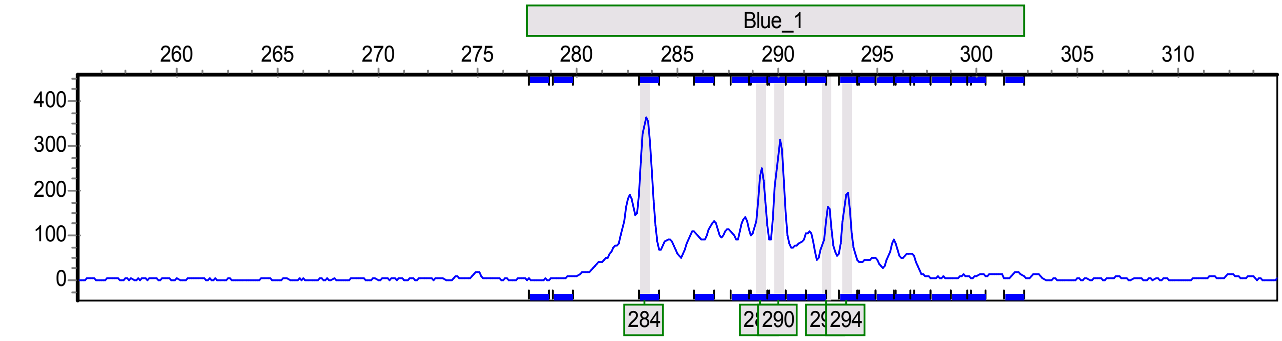

No	Size	Height	Area	Marker	Allele	Difference	Quality	Score	Allele Comments	Sample Comments
1	283.4	364	2620	Blue_1	284	0.2	Pass	21.2		
2	289.2	251	1513	Blue_1	289	0.1	Pass	14.6		
3	290.1	317	1798	Blue_1	290	0.1	Pass	26.9		
4	292.5	166	869	Blue_1	292	0.5	Pass	9.1		
5	293.5	198	999	Blue_1	294	0.1	Pass	14.2		

Sample 53: Run date and time: 09/11/2020 - 20:27:01 -> 09/11/2020 - 21:12:55

Dye: Blue - 2 peaks - 57.fsa

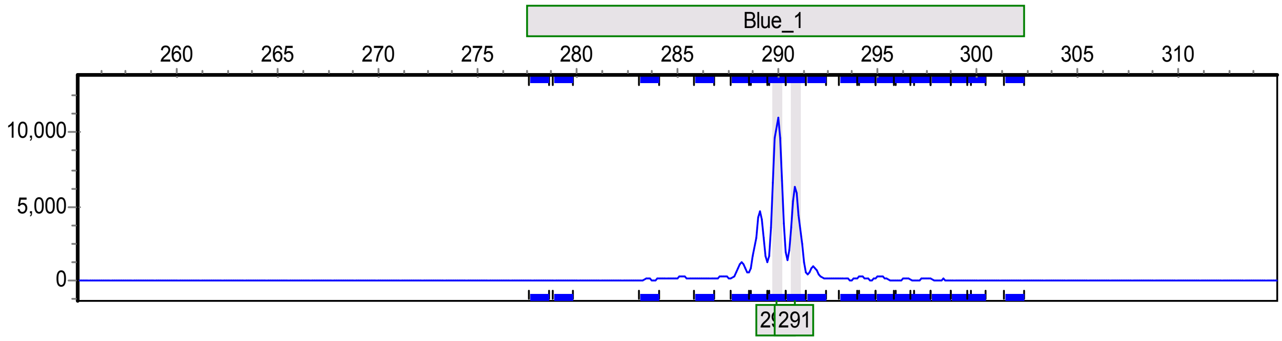

No	Size	Height	Area	Marker	Allele	Difference	Quality	Score	Allele Comments	Sample Comments
1	290.0	10955	52313	Blue_1	290	0.0	Pass	500.0		
2	290.9	6333	30409	Blue_1	291	0.1	Pass	500.0		

Sample 54: Run date and time: 09/11/2020 - 20:27:01 -> 09/11/2020 - 21:12:55

Dye: Blue - 2 peaks - 58.fsa

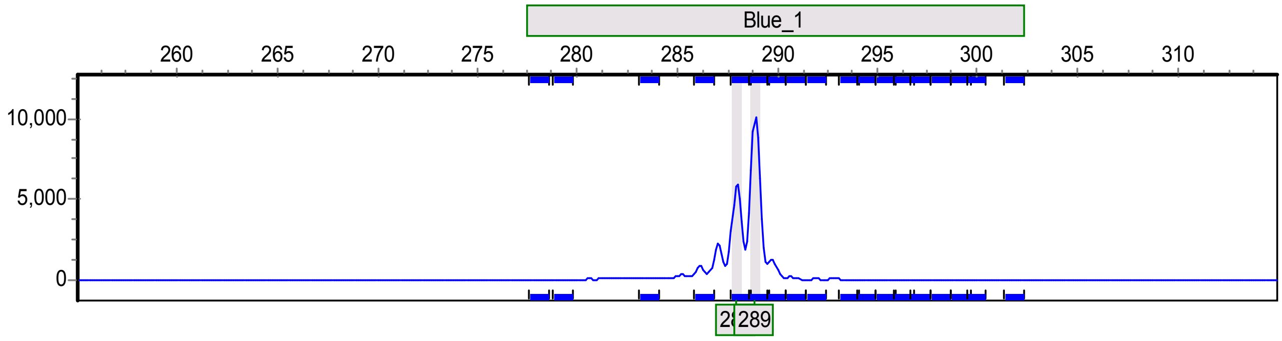

No	Size	Height	Area	Marker	Allele	Difference	Quality	Score	Allele Comments	Sample Comments
1	288.0	5982	32291	Blue_1	288	0.2	Pass	500.0		
2	288.9	10037	53154	Blue_1	289	0.2	Pass	500.0		

Allele Report

Sample 55: Run date and time: 09/11/2020 - 20:27:01 -> 09/11/2020 - 21:12:55

Dye: Blue - 2 peaks - 59.fsa

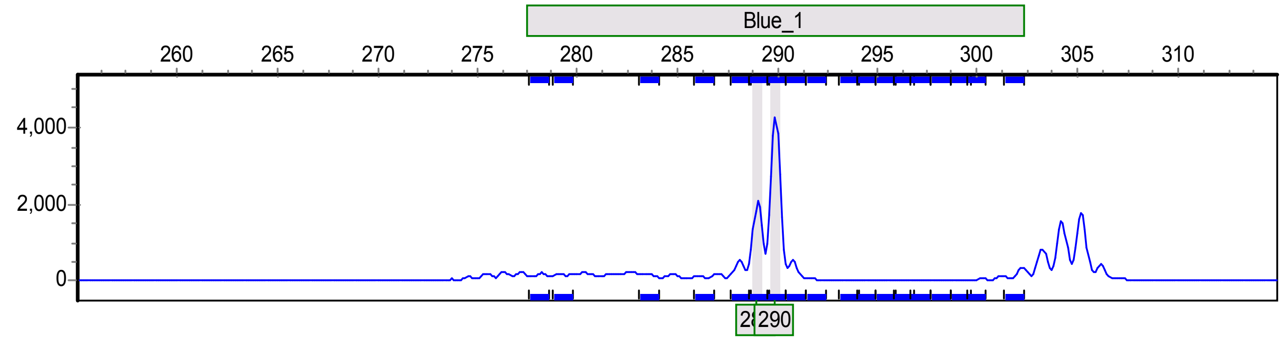

No	Size	Height	Area	Marker	Allele	Difference	Quality	Score	Allele Comments	Sample Comments
1	289.0	2081	10659	Blue_1	289	0.1	Pass	490.3		
2	289.9	4255	22074	Blue_1	290	0.1	Pass	500.0		

Sample 56: Run date and time: 09/11/2020 - 20:27:01 -> 09/11/2020 - 21:12:55

Dye: Blue - 1 peaks - 6.fsa

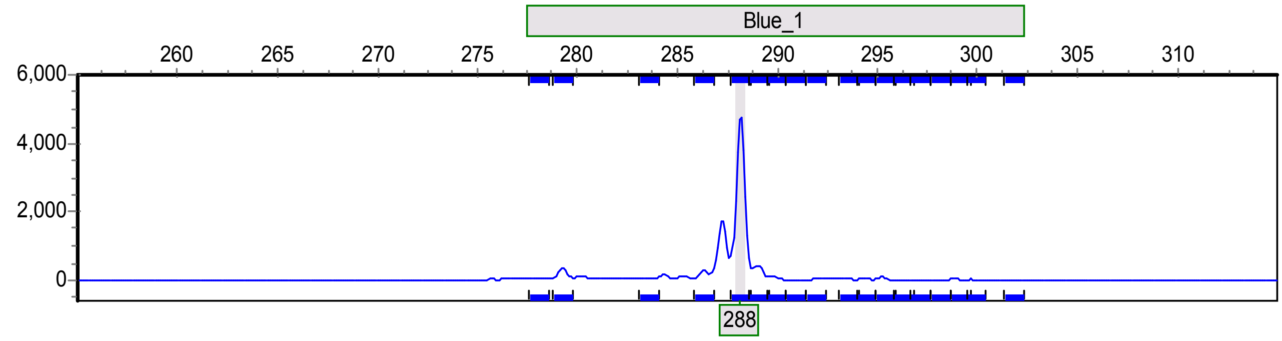

No	Size	Height	Area	Marker	Allele	Difference	Quality	Score	Allele Comments	Sample Comments
1	288.2	4725	24261	Blue_1	288	0.0	Pass	500.0		

Sample 57: Run date and time: 09/11/2020 - 20:27:01 -> 09/11/2020 - 21:12:55

Dye: Blue - 5 peaks - 60.fsa

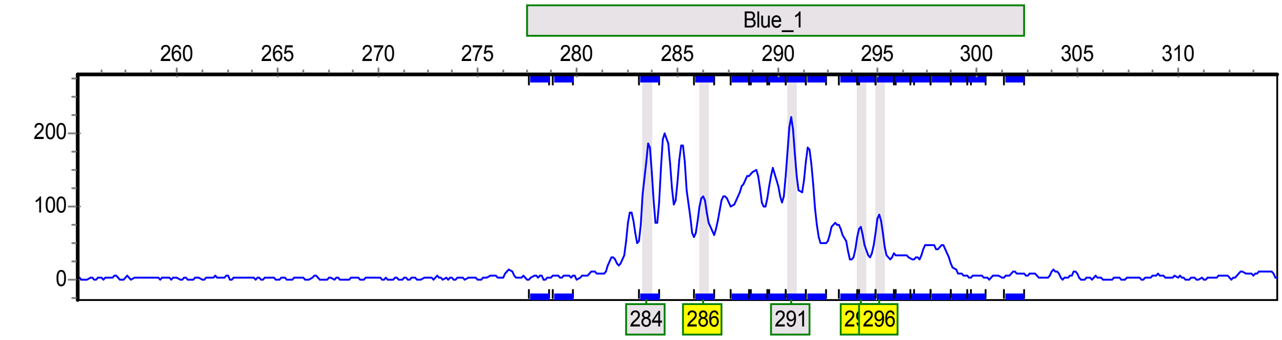

No	Size	Height	Area	Marker	Allele	Difference	Quality	Score	Allele Comments	Sample Comments
1	283.5	187	1049	Blue_1	284	0.1	Pass	9.4		
2	286.3	113	814	Blue_1	286	0.1	Check	1.6		
3	290.7	222	1503	Blue_1	291	0.3	Pass	7.2		
4	294.2	71	408	Blue_1	294	0.3	Check	1.4		
5	295.1	89	533	Blue_1	296	0.4	Check	2.4		

Allele Report

Sample 58: Run date and time: 09/11/2020 - 20:27:01 -> 09/11/2020 - 21:12:55

Dye: Blue - 2 peaks - 61.fsa

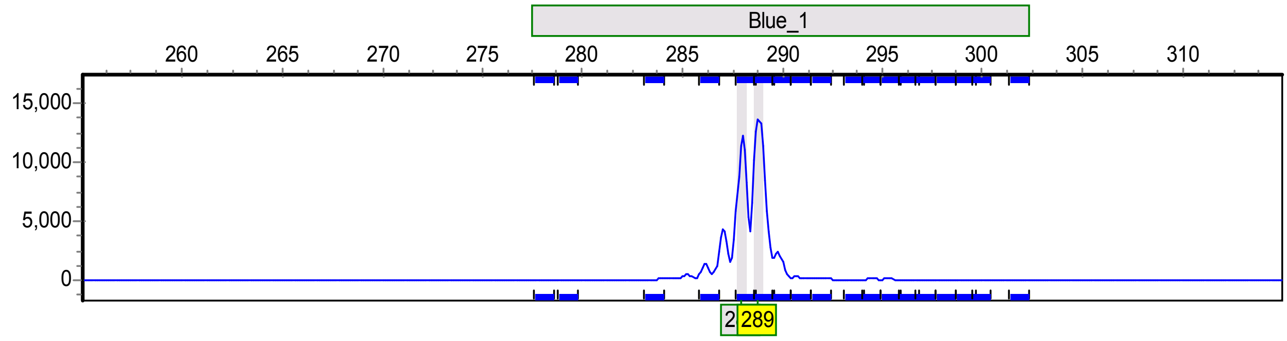

No	Size	Height	Area	Marker	Allele	Difference	Quality	Score	Allele Comments	Sample Comments
1	288.0	12257	66168	Blue_1	288	0.2	Pass	500.0		
2	288.8	13675	90031	Blue_1	289	0.3	Check	500.0	[<SAT (Repaired)>]	

Sample 59: Run date and time: 09/11/2020 - 20:27:01 -> 09/11/2020 - 21:12:55

Dye: Blue - 2 peaks - 62.fsa

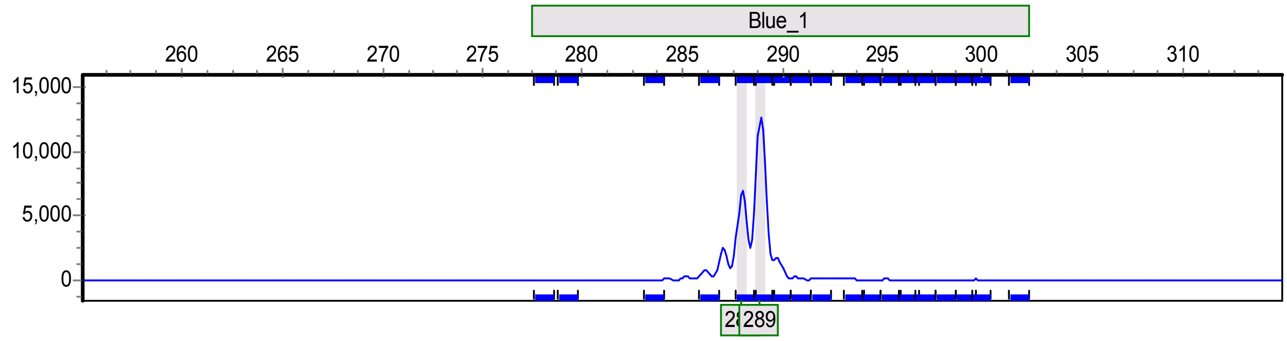

No	Size	Height	Area	Marker	Allele	Difference	Quality	Score	Allele Comments	Sample Comments
1	288.0	6891	37869	Blue_1	288	0.2	Pass	500.0		
2	288.9	12520	71175	Blue_1	289	0.2	Pass	500.0		

Sample 60: Run date and time: 09/11/2020 - 20:27:01 -> 09/11/2020 - 21:12:55

Dye: Blue - 2 peaks - 63.fsa

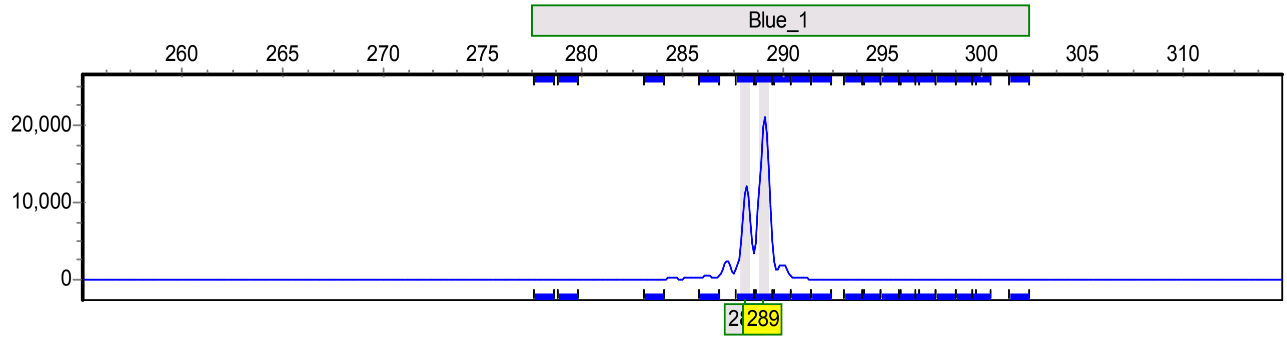

No	Size	Height	Area	Marker	Allele	Difference	Quality	Score	Allele Comments	Sample Comments
1	288.2	12116	60664	Blue_1	288	0.0	Pass	500.0		
2	289.1	20915	116246	Blue_1	289	0.0	Check	500.0	[<SAT (Repaired)>]	

Sample 61: Run date and time: 09/11/2020 - 20:27:01 -> 09/11/2020 - 21:12:55

Dye: Blue - 2 peaks - 64.fsa

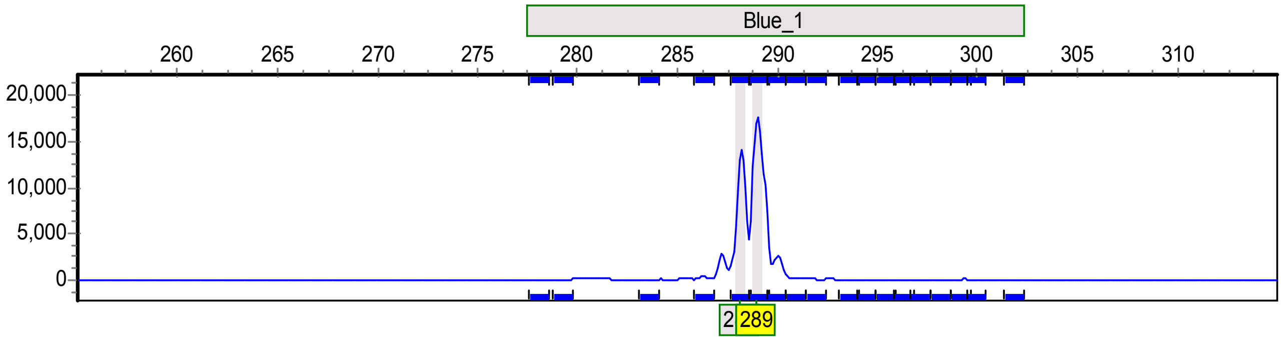

No	Size	Height	Area	Marker	Allele	Difference	Quality	Score	Allele Comments	Sample Comments
1	288.2	13978	78929	Blue_1	288	0.0	Pass	500.0		
2	289.0	17493	112982	Blue_1	289	0.1	Check	500.0	[<SAT (Repaired)>]	

Sample 62: Run date and time: 09/11/2020 - 20:27:01 -> 09/11/2020 - 21:12:55

Dye: Blue - 2 peaks - 65.fsa

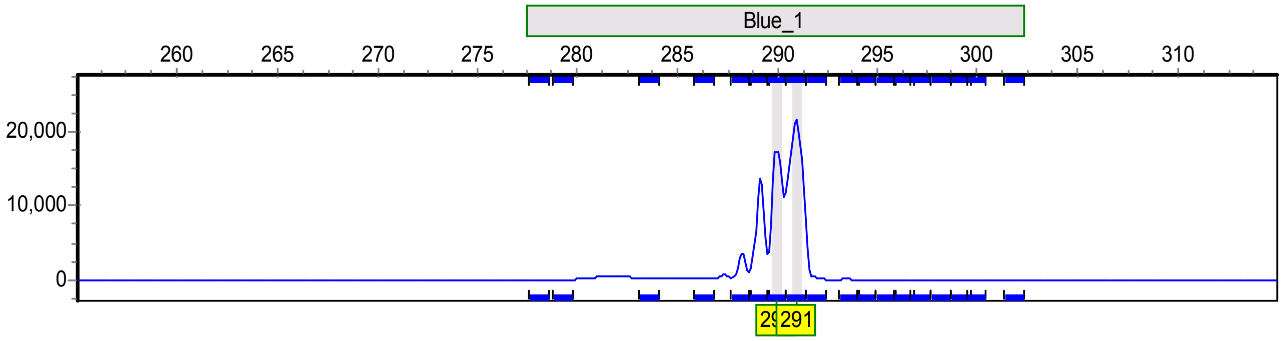

No	Size	Height	Area	Marker	Allele	Difference	Quality	Score	Allele Comments	Sample Comments
1	290.0	17345	99742	Blue_1	290	0.0	Check	500.0	[<SAT (Repaired)>]	
2	291.0	21745	177645	Blue_1	291	0.0	Check	500.0	[<SAT (Repaired)>]	

Sample 63: Run date and time: 09/11/2020 - 20:27:01 -> 09/11/2020 - 21:12:55

Dye: Blue - 2 peaks - 66.fsa

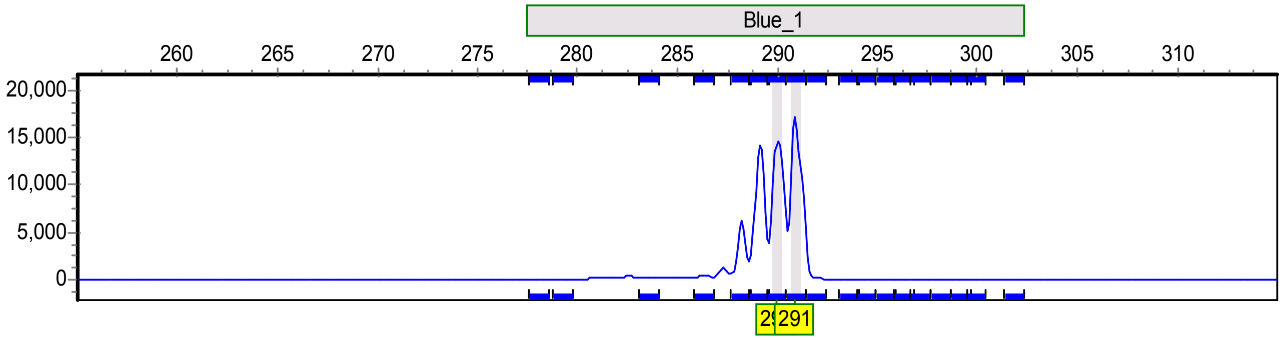

No	Size	Height	Area	Marker	Allele	Difference	Quality	Score	Allele Comments	Sample Comments
1	290.0	14636	98230	Blue_1	290	0.0	Check	500.0	[<SAT (Repaired)>]	
2	290.9	17051	104113	Blue_1	291	0.1	Check	500.0	[<SAT (Repaired)>]	

Allele Report

Sample 64: Run date and time: 09/11/2020 - 20:27:01 -> 09/11/2020 - 21:12:55

Dye: Blue - 3 peaks - 67.fsa

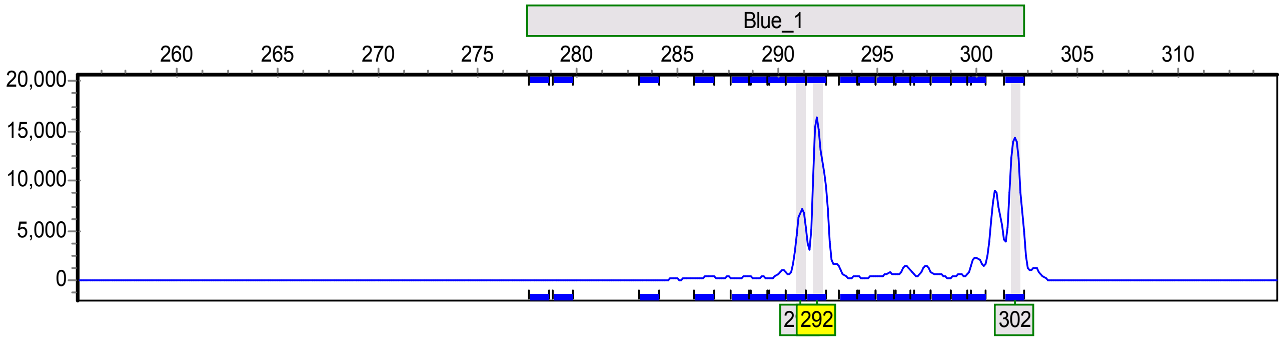

No	Size	Height	Area	Marker	Allele	Difference	Quality	Score	Allele Comments	Sample Comments
1	291.2	7147	37799	Blue_1	291	0.2	Pass	500.0		
2	292.0	16256	107100	Blue_1	292	0.0	Check	500.0	[<SAT (Repaired)>]	
3	301.9	14213	95105	Blue_1	302	0.0	Pass	500.0		

Sample 65: Run date and time: 09/11/2020 - 20:27:01 -> 09/11/2020 - 21:12:55

Dye: Blue - 3 peaks - 68.fsa

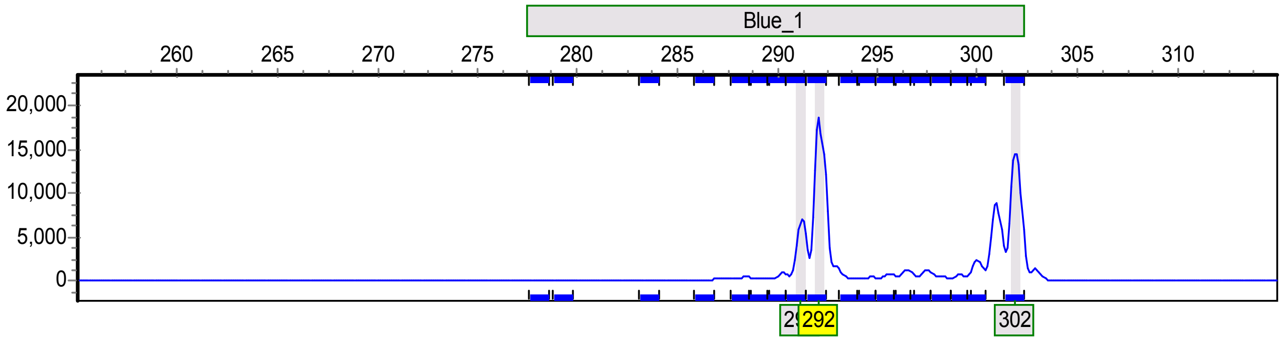

No	Size	Height	Area	Marker	Allele	Difference	Quality	Score	Allele Comments	Sample Comments
1	291.2	6955	37828	Blue_1	291	0.2	Pass	500.0		
2	292.1	18533	110764	Blue_1	292	0.1	Check	500.0	[<SAT (Repaired)>]	
3	301.9	14559	93864	Blue_1	302	0.0	Pass	500.0		

Sample 66: Run date and time: 09/11/2020 - 20:27:01 -> 09/11/2020 - 21:12:55

Dye: Blue - 4 peaks - 69.fsa

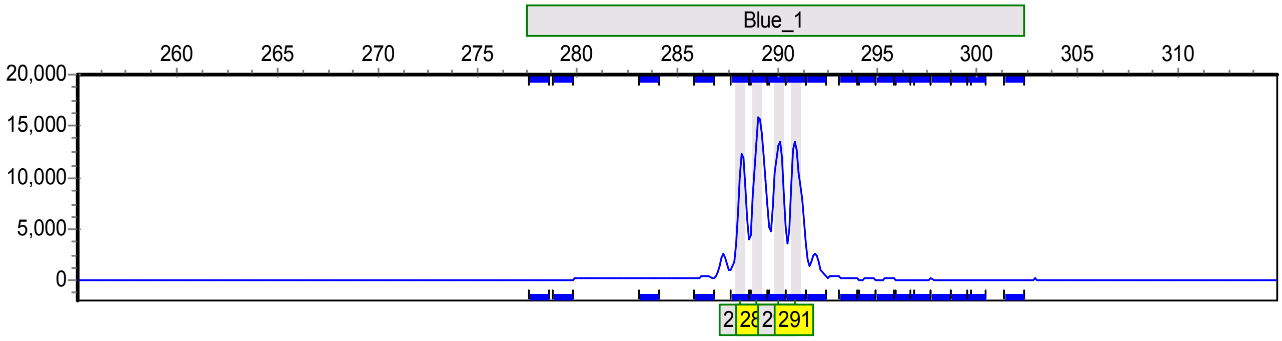

No	Size	Height	Area	Marker	Allele	Difference	Quality	Score	Allele Comments	Sample Comments
1	288.2	12313	63698	Blue_1	288	0.0	Pass	500.0		
2	289.0	15782	105884	Blue_1	289	0.1	Check	500.0	[<SAT (Repaired)>]	
3	290.1	13567	74321	Blue_1	290	0.1	Pass	500.0		
4	290.9	13580	80331	Blue_1	291	0.1	Check	500.0	[<SAT (Repaired)>]	

Allele Report

Sample 67: Run date and time: 09/11/2020 - 20:27:01 -> 09/11/2020 - 21:12:55

Dye: Blue - 1 peaks - 7.fsa

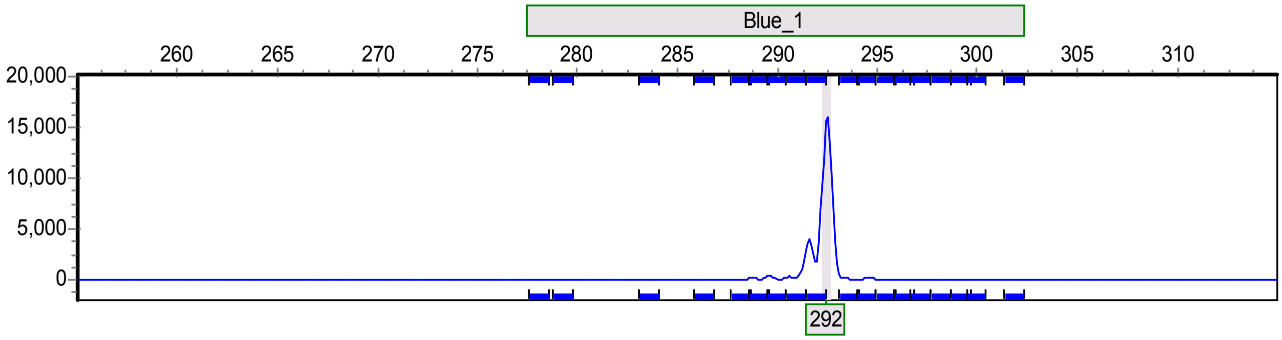

No	Size	Height	Area	Marker	Allele	Difference	Quality	Score	Allele Comments	Sample Comments
1	292.5	15955	87479	Blue_1	292	0.5	Pass	500.0		

Sample 68: Run date and time: 09/11/2020 - 20:27:01 -> 09/11/2020 - 21:12:55

Dye: Blue - 2 peaks - 70.fsa

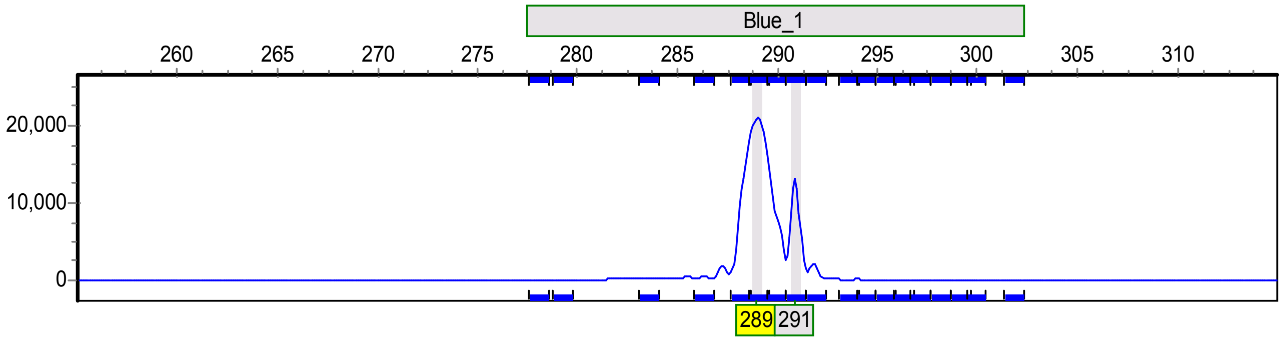

No	Size	Height	Area	Marker	Allele	Difference	Quality	Score	Allele Comments	Sample Comments
1	289.0	20812	334543	Blue_1	289	0.1	Check	500.0	[<SAT (Repaired)>]	
2	290.9	13021	69172	Blue_1	291	0.1	Pass	500.0		

Sample 69: Run date and time: 09/11/2020 - 20:27:01 -> 09/11/2020 - 21:12:55

Dye: Blue - 3 peaks - 71.fsa

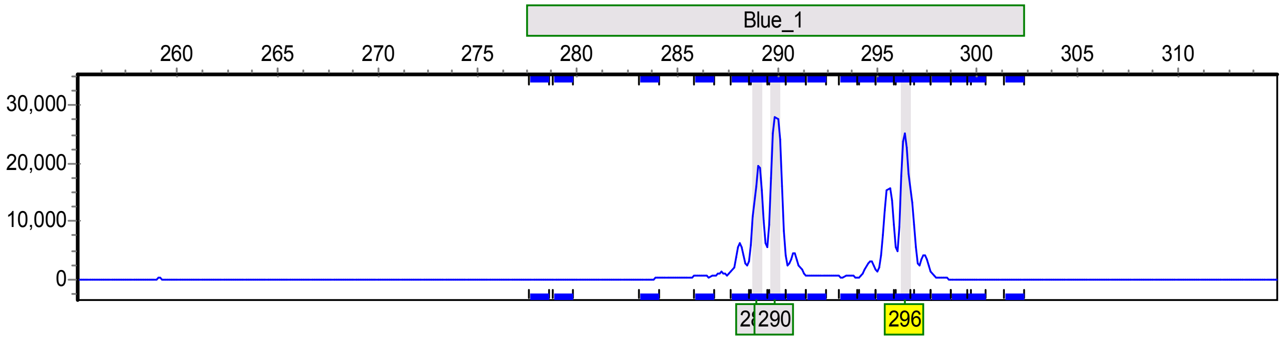

No	Size	Height	Area	Marker	Allele	Difference	Quality	Score	Allele Comments	Sample Comments
1	289.0	19571	103707	Blue_1	289	0.1	Pass	500.0		
2	289.9	27717	155488	Blue_1	290	0.1	Pass	500.0		
3	296.4	24956	145365	Blue_1	296	0.0	Check	500.0	[<SAT (Repaired)>]	

Sample 70: Run date and time: 09/11/2020 - 20:27:01 -> 09/11/2020 - 21:12:55

Dye: Blue - 3 peaks - 72.fsa

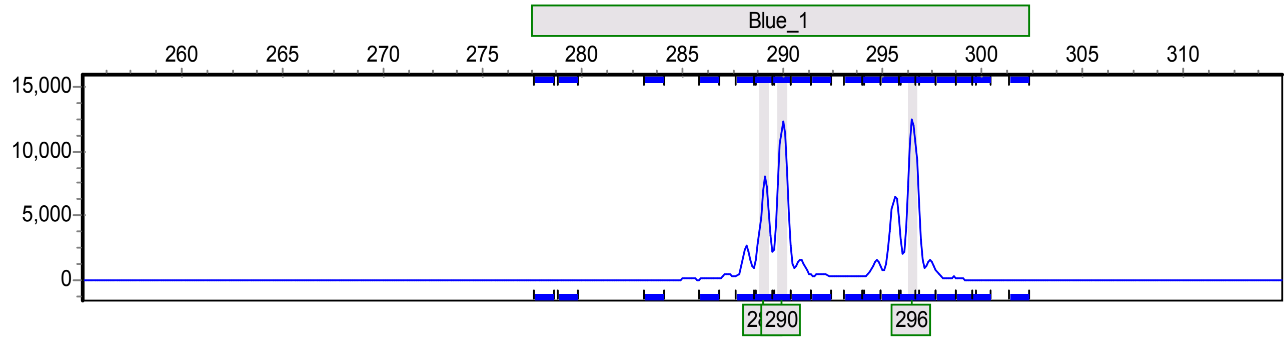

No	Size	Height	Area	Marker	Allele	Difference	Quality	Score	Allele Comments	Sample Comments
1	289.1	7980	41852	Blue_1	289	0.0	Pass	500.0		
2	290.0	12262	62098	Blue_1	290	0.0	Pass	500.0		
3	296.5	12521	65400	Blue_1	296	0.1	Pass	500.0		

Sample 71: Run date and time: 09/11/2020 - 20:27:01 -> 09/11/2020 - 21:12:55

Dye: Blue - 2 peaks - 73.fsa

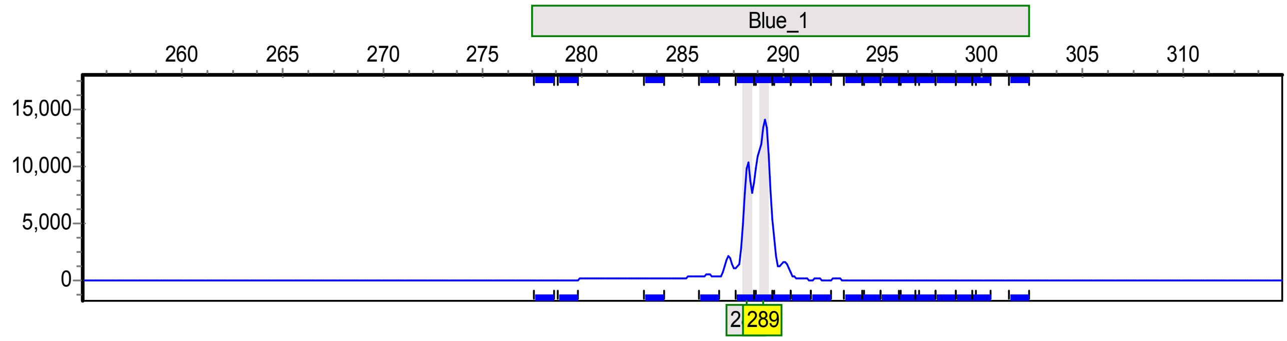

No	Size	Height	Area	Marker	Allele	Difference	Quality	Score	Allele Comments	Sample Comments
1	288.3	10263	60785	Blue_1	288	0.1	Pass	500.0		
2	289.1	14153	105407	Blue_1	289	0.0	Check	500.0	[<SAT (Repaired)>]	

Sample 72: Run date and time: 09/11/2020 - 20:27:01 -> 09/11/2020 - 21:12:55

Dye: Blue - 2 peaks - 74.fsa

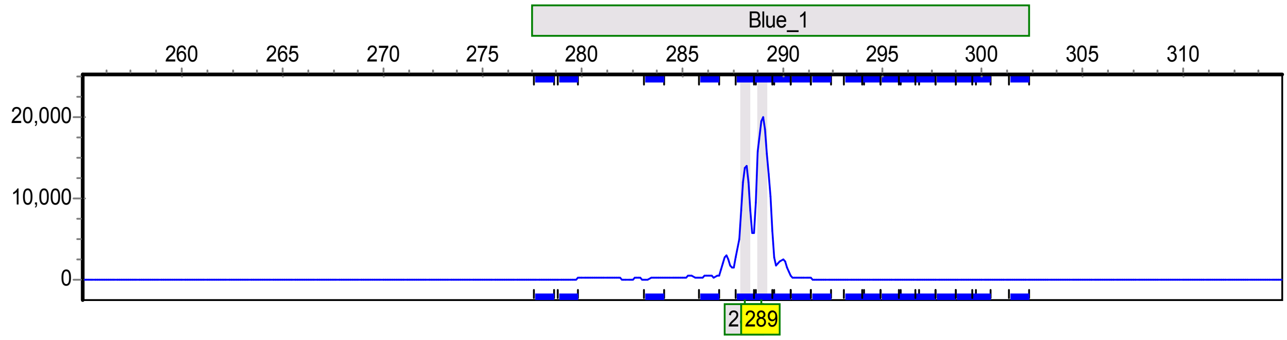

No	Size	Height	Area	Marker	Allele	Difference	Quality	Score	Allele Comments	Sample Comments
1	288.2	13903	80900	Blue_1	288	0.0	Pass	500.0		
2	289.0	19807	133430	Blue_1	289	0.1	Check	500.0	[<SAT (Repaired)>]	

Sample 73: Run date and time: 09/11/2020 - 20:27:01 -> 09/11/2020 - 21:12:55

Dye: Blue - 3 peaks - 75.fsa

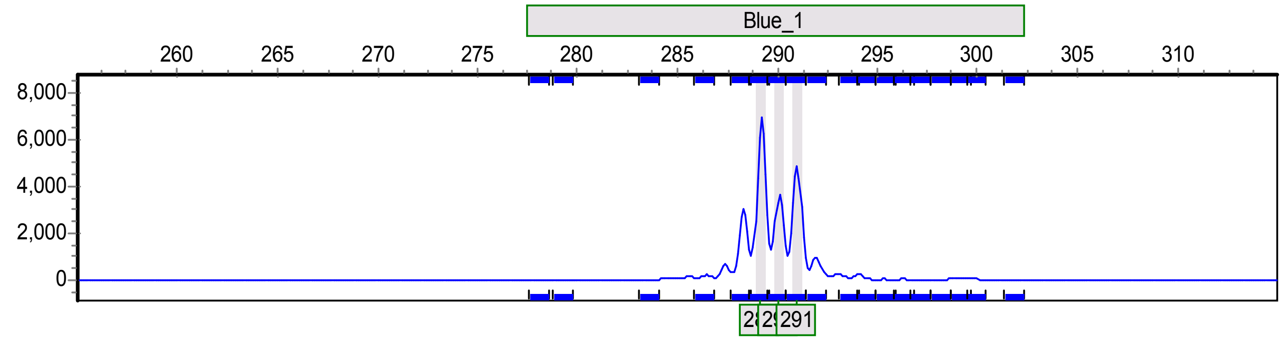

No	Size	Height	Area	Marker	Allele	Difference	Quality	Score	Allele Comments	Sample Comments
1	289.2	6944	36207	Blue_1	289	0.1	Pass	500.0		
2	290.1	3667	19608	Blue_1	290	0.1	Pass	500.0		
3	291.0	4930	25727	Blue_1	291	0.0	Pass	500.0		

Sample 74: Run date and time: 09/11/2020 - 20:27:01 -> 09/11/2020 - 21:12:55

Dye: Blue - 3 peaks - 76.fsa

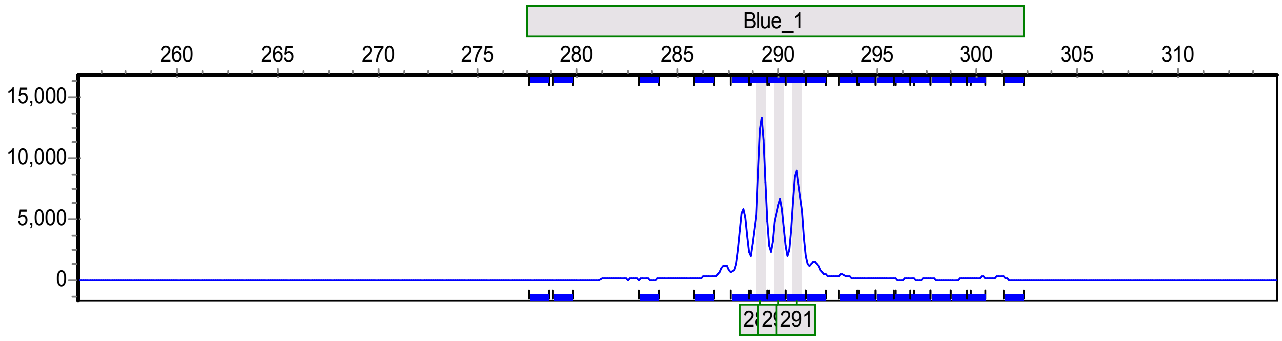

No	Size	Height	Area	Marker	Allele	Difference	Quality	Score	Allele Comments	Sample Comments
1	289.2	13241	68570	Blue_1	289	0.1	Pass	500.0		
2	290.1	6584	35838	Blue_1	290	0.1	Pass	500.0		
3	291.0	9049	48992	Blue_1	291	0.0	Pass	500.0		

Sample 75: Run date and time: 09/11/2020 - 20:27:01 -> 09/11/2020 - 21:12:55

Dye: Blue - 3 peaks - 77.fsa

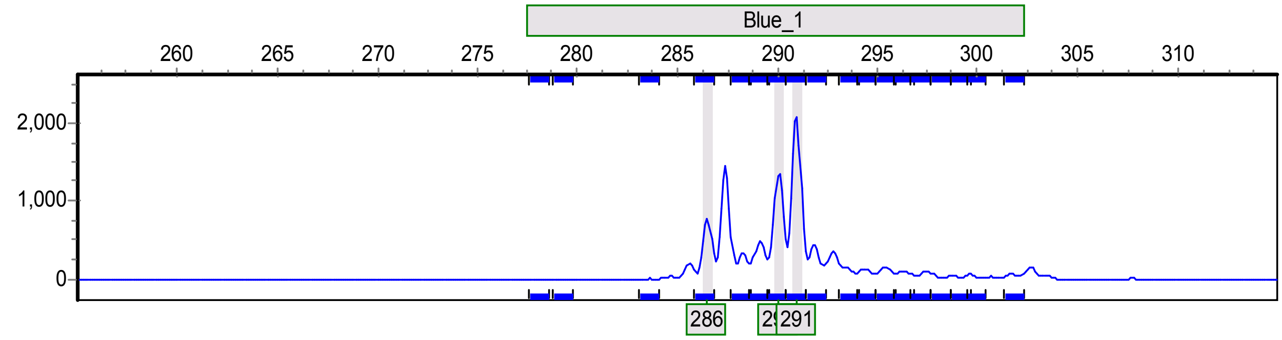

No	Size	Height	Area	Marker	Allele	Difference	Quality	Score	Allele Comments	Sample Comments
1	286.5	780	4131	Blue_1	286	0.1	Pass	128.3		
2	290.1	1351	7214	Blue_1	290	0.1	Pass	251.8		
3	291.0	2061	10955	Blue_1	291	0.0	Pass	500.0		

Sample 76: Run date and time: 09/11/2020 - 20:27:01 -> 09/11/2020 - 21:12:55

Dye: Blue - 5 peaks - 78.fsa

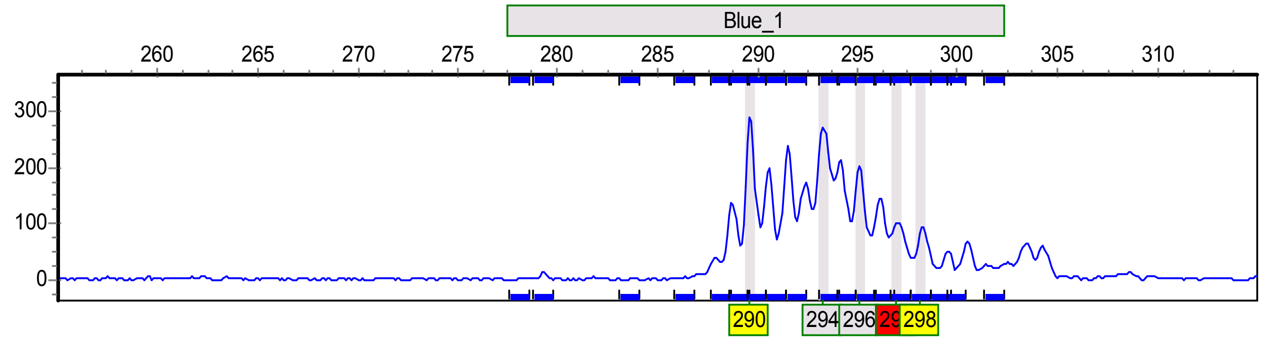

No	Size	Height	Area	Marker	Allele	Difference	Quality	Score	Allele Comments	Sample Comments
1	289.6	288	1564	Blue_1	290	0.4	Check	22.7		
2	293.3	271	1742	Blue_1	294	0.3	Pass	7.7		
3	295.1	202	1332	Blue_1	296	0.4	Pass	7.0		
4	297.0	102	903	Blue_1	297	0.2	Undetermined	0.6		
5	298.2	95	681	Blue_1	298	0.0	Check	1.9		

Sample 77: Run date and time: 09/11/2020 - 20:27:01 -> 09/11/2020 - 21:12:55

Dye: Blue - 2 peaks - 79.fsa

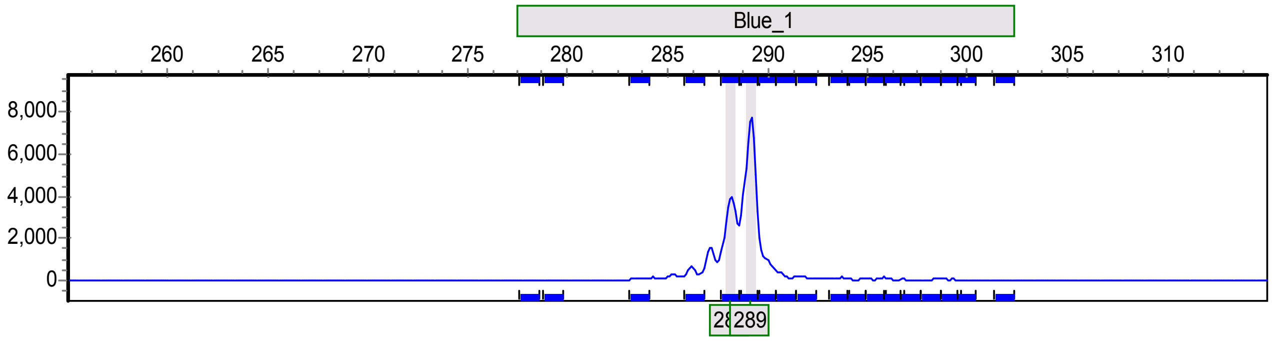

No	Size	Height	Area	Marker	Allele	Difference	Quality	Score	Allele Comments	Sample Comments
1	288.2	3921	28052	Blue_1	288	0.0	Pass	485.4		
2	289.2	7674	51522	Blue_1	289	0.1	Pass	500.0		

Sample 78: Run date and time: 09/11/2020 - 20:27:01 -> 09/11/2020 - 21:12:55

Dye: Blue - 4 peaks - 8.fsa

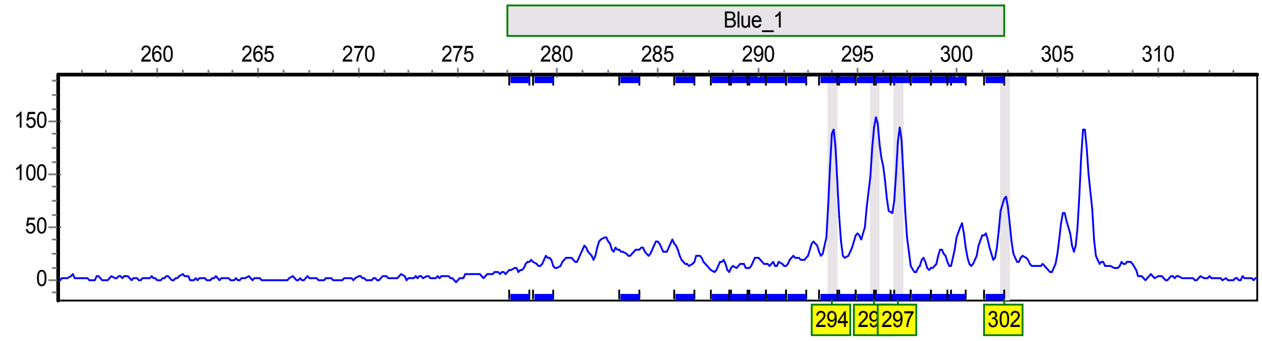

No	Size	Height	Area	Marker	Allele	Difference	Quality	Score	Allele Comments	Sample Comments
1	293.8	142	818	Blue_1	294	0.2	Check	6.9		
2	295.9	154	1366	Blue_1	296	0.4	Check	3.1		
3	297.1	144	789	Blue_1	297	0.1	Check	6.1		
4	302.4	78	498	Blue_1	302	0.5	Check	1.9		

Allele Report

Sample 79: Run date and time: 09/11/2020 - 20:27:01 -> 09/11/2020 - 21:12:55

Dye: Blue - 3 peaks - 80.fsa

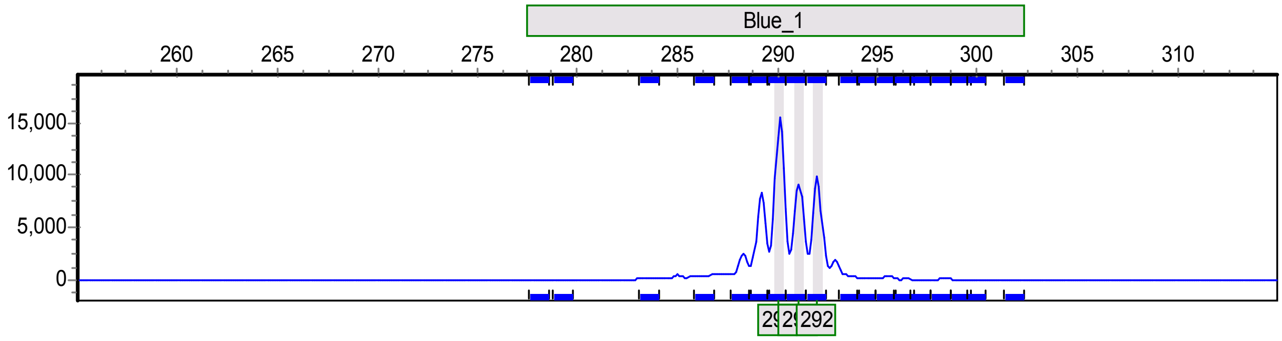

No	Size	Height	Area	Marker	Allele	Difference	Quality	Score	Allele Comments	Sample Comments
1	290.1	15394	82755	Blue_1	290	0.1	Pass	500.0		
2	291.1	9013	51115	Blue_1	291	0.1	Pass	500.0		
3	292.0	9781	53025	Blue_1	292	0.0	Pass	500.0		

Sample 80: Run date and time: 09/11/2020 - 20:27:01 -> 09/11/2020 - 21:12:55

Dye: Blue - 4 peaks - 81.fsa

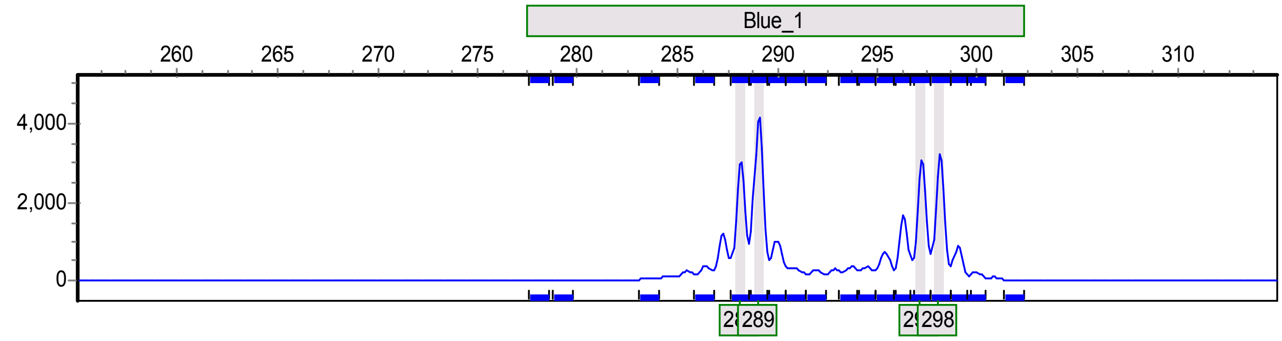

No	Size	Height	Area	Marker	Allele	Difference	Quality	Score	Allele Comments	Sample Comments
1	288.2	3023	16311	Blue_1	288	0.0	Pass	500.0		
2	289.1	4136	21827	Blue_1	289	0.0	Pass	500.0		
3	297.2	3077	16252	Blue_1	297	0.0	Pass	500.0		
4	298.1	3207	16148	Blue_1	298	0.1	Pass	500.0		

Sample 81: Run date and time: 09/11/2020 - 20:27:01 -> 09/11/2020 - 21:12:55

Dye: Blue - 2 peaks - 82.fsa

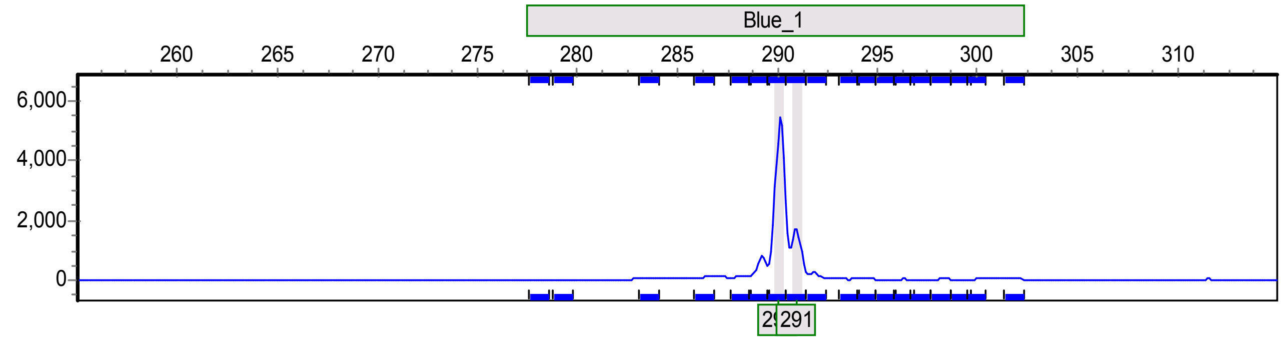

No	Size	Height	Area	Marker	Allele	Difference	Quality	Score	Allele Comments	Sample Comments
1	290.1	5401	29267	Blue_1	290	0.1	Pass	500.0		
2	291.0	1696	8877	Blue_1	291	0.0	Pass	287.9		

Sample 82: Run date and time: 09/11/2020 - 20:27:01 -> 09/11/2020 - 21:12:55

Dye: Blue - 3 peaks - 83.fsa

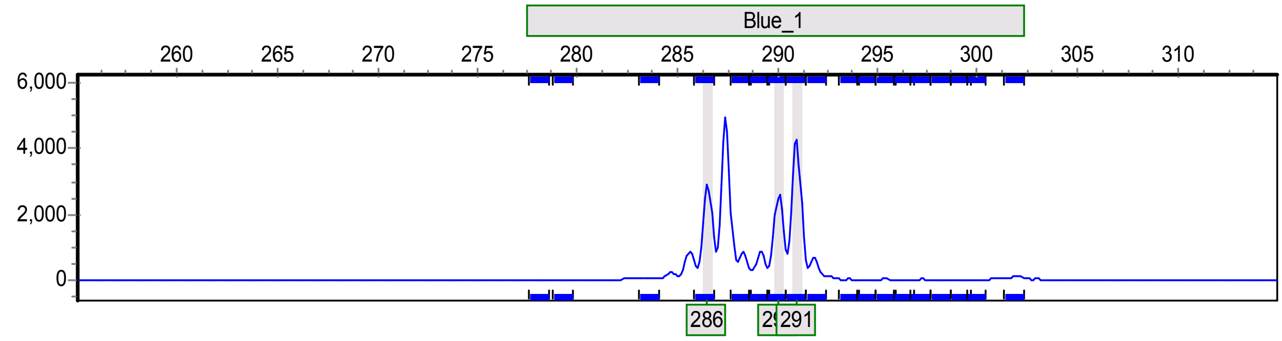

No	Size	Height	Area	Marker	Allele	Difference	Quality	Score	Allele Comments	Sample Comments
1	286.5	2894	15211	Blue_1	286	0.1	Pass	500.0		
2	290.1	2574	13694	Blue_1	290	0.1	Pass	500.0		
3	291.0	4273	22138	Blue_1	291	0.0	Pass	500.0		

Sample 83: Run date and time: 09/11/2020 - 20:27:01 -> 09/11/2020 - 21:12:55

Dye: Blue - 3 peaks - 84.fsa

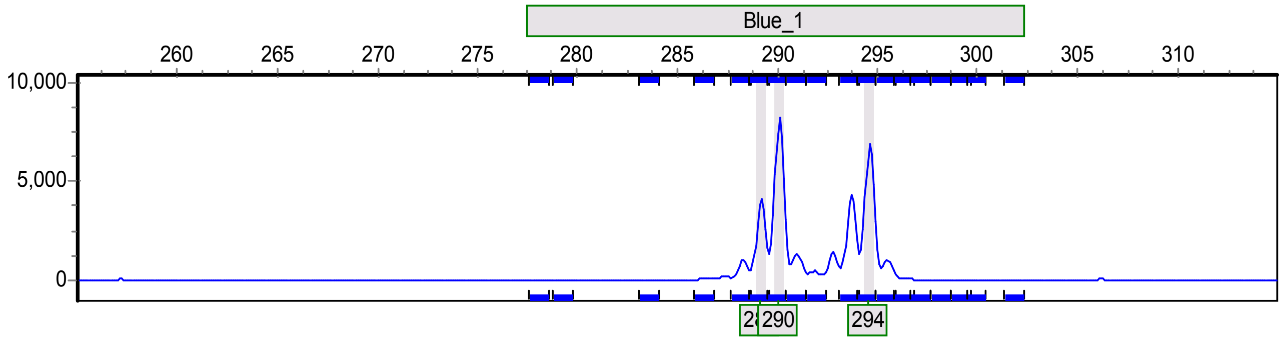

No	Size	Height	Area	Marker	Allele	Difference	Quality	Score	Allele Comments	Sample Comments
1	289.2	4077	20816	Blue_1	289	0.1	Pass	500.0		
2	290.1	8164	41776	Blue_1	290	0.1	Pass	500.0		
3	294.6	6836	36266	Blue_1	294	0.1	Pass	500.0		

Sample 84: Run date and time: 09/11/2020 - 20:27:01 -> 09/11/2020 - 21:12:55

Dye: Blue - 3 peaks - 85.fsa

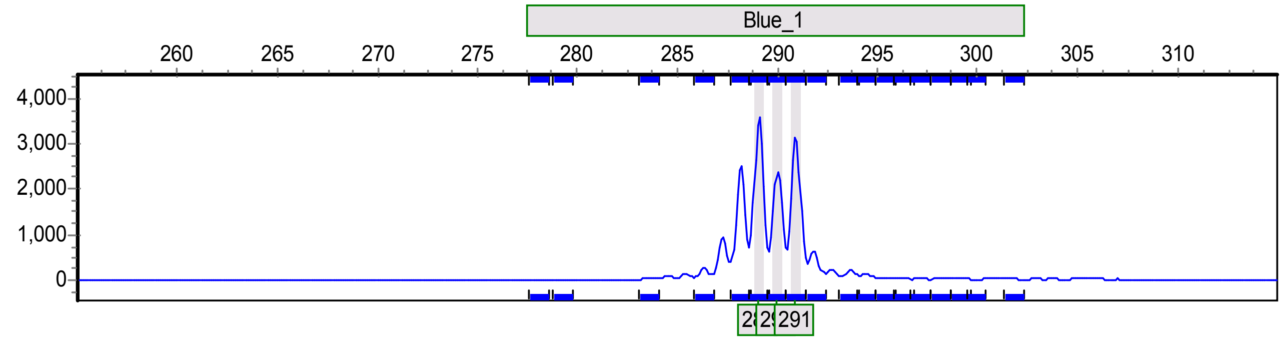

No	Size	Height	Area	Marker	Allele	Difference	Quality	Score	Allele Comments	Sample Comments
1	289.1	3558	18769	Blue_1	289	0.0	Pass	500.0		
2	290.0	2361	12540	Blue_1	290	0.0	Pass	500.0		
3	290.9	3133	16310	Blue_1	291	0.1	Pass	500.0		

Sample 85: Run date and time: 09/11/2020 - 20:27:01 -> 09/11/2020 - 21:12:55

Dye: Blue - 5 peaks - 86.fsa

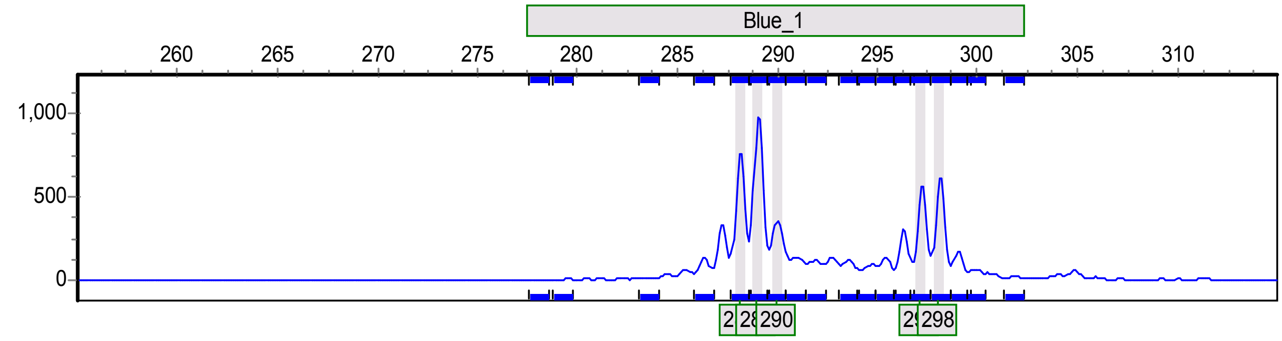

No	Size	Height	Area	Marker	Allele	Difference	Quality	Score	Allele Comments	Sample Comments
1	288.2	764	4191	Blue_1	288	0.0	Pass	108.4		
2	289.0	973	5452	Blue_1	289	0.1	Pass	164.7		
3	290.0	352	2355	Blue_1	290	0.0	Pass	17.2		
4	297.2	560	3045	Blue_1	297	0.0	Pass	73.5		
5	298.1	611	3246	Blue_1	298	0.1	Pass	84.6		

Sample 86: Run date and time: 09/11/2020 - 20:27:01 -> 09/11/2020 - 21:12:55

Dye: Blue - 3 peaks - 87.fsa

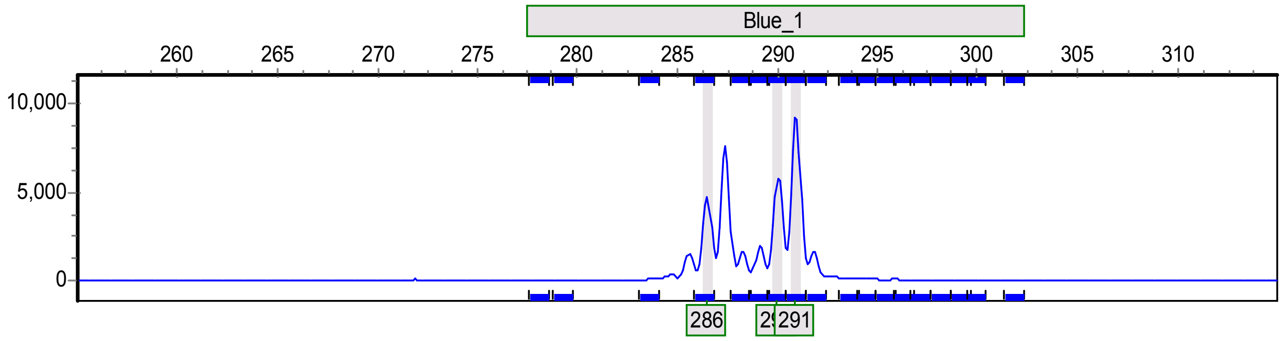

No	Size	Height	Area	Marker	Allele	Difference	Quality	Score	Allele Comments	Sample Comments
1	286.5	4726	23550	Blue_1	286	0.1	Pass	500.0		
2	290.0	5756	30644	Blue_1	290	0.0	Pass	500.0		
3	290.9	9164	48066	Blue_1	291	0.1	Pass	500.0		

Sample 87: Run date and time: 09/11/2020 - 20:27:01 -> 09/11/2020 - 21:12:55

Dye: Blue - 2 peaks - 88.fsa

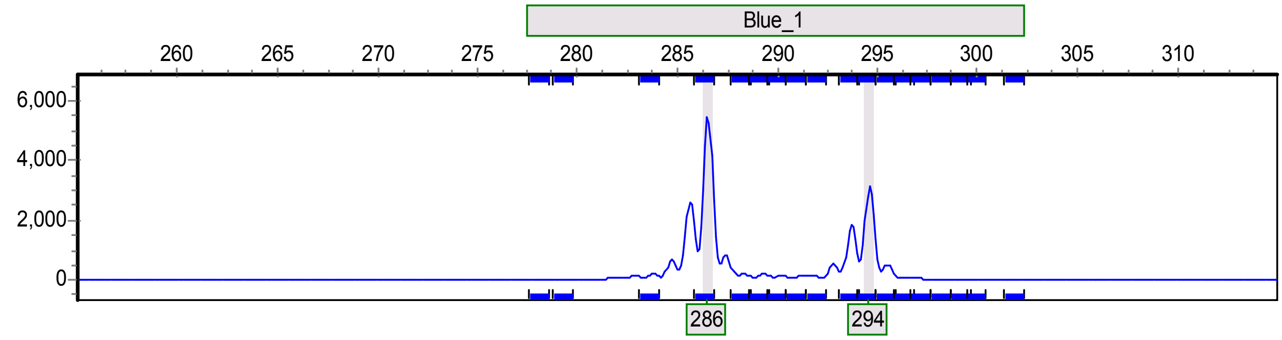

No	Size	Height	Area	Marker	Allele	Difference	Quality	Score	Allele Comments	Sample Comments
1	286.5	5411	28577	Blue_1	286	0.1	Pass	500.0		
2	294.6	3118	16555	Blue_1	294	0.1	Pass	500.0		

Allele Report

Sample 88: Run date and time: 09/11/2020 - 20:27:01 -> 09/11/2020 - 21:12:55

Dye: Blue - 3 peaks - 89.fsa

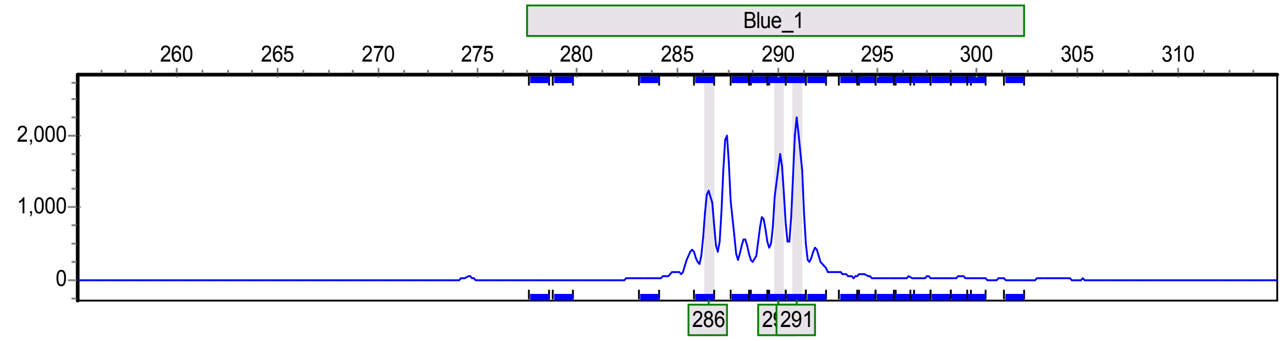

No	Size	Height	Area	Marker	Allele	Difference	Quality	Score	Allele Comments	Sample Comments
1	286.6	1238	6576	Blue_1	286	0.2	Pass	232.0		
2	290.1	1731	9762	Blue_1	290	0.1	Pass	370.0		
3	291.0	2233	11884	Blue_1	291	0.0	Pass	500.0		

Sample 89: Run date and time: 09/11/2020 - 20:27:01 -> 09/11/2020 - 21:12:55

Dye: Blue - 1 peaks - 9.fsa

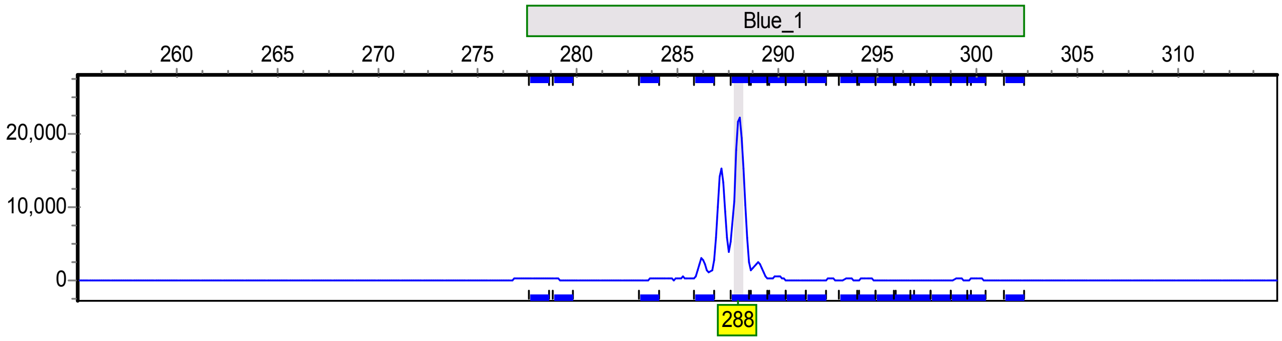

No	Size	Height	Area	Marker	Allele	Difference	Quality	Score	Allele Comments	Sample Comments
1	288.1	22113	128573	Blue_1	288	0.1	Check	500.0	[<SAT (Repaired)>]	

Sample 90: Run date and time: 09/11/2020 - 20:27:01 -> 09/11/2020 - 21:12:55

Dye: Blue - 2 peaks - 90.fsa

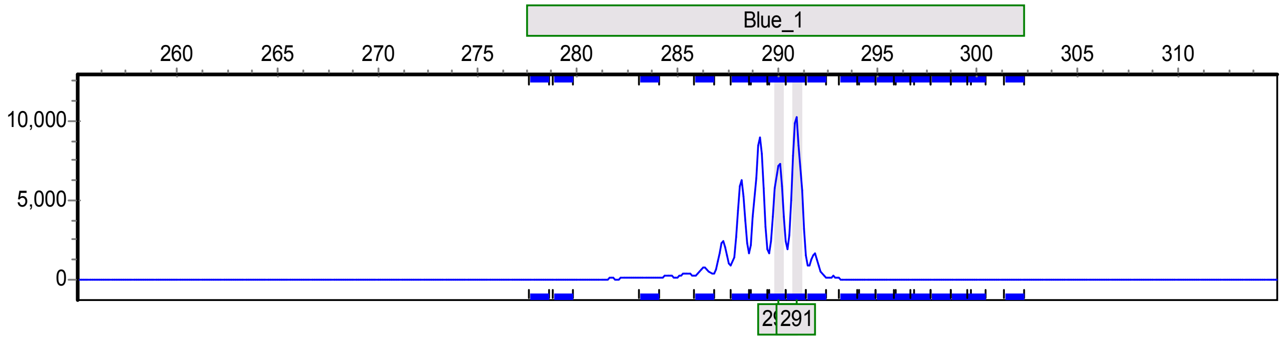

No	Size	Height	Area	Marker	Allele	Difference	Quality	Score	Allele Comments	Sample Comments
1	290.1	7232	40451	Blue_1	290	0.1	Pass	500.0		
2	291.0	10150	53905	Blue_1	291	0.0	Pass	500.0		

Allele Report

Sample 91: Run date and time: 09/11/2020 - 20:27:01 -> 09/11/2020 - 21:12:55

Dye: Blue - 5 peaks - 91.fsa

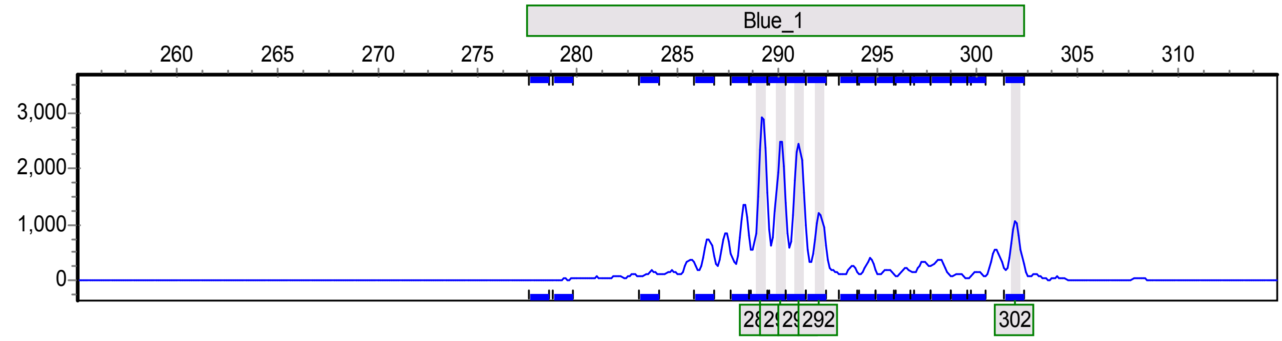

No	Size	Height	Area	Marker	Allele	Difference	Quality	Score	Allele Comments	Sample Comments
1	289.2	2892	16082	Blue_1	289	0.1	Pass	500.0		
2	290.2	2485	13013	Blue_1	290	0.2	Pass	500.0		
3	291.1	2442	13426	Blue_1	291	0.1	Pass	500.0		
4	292.1	1202	6643	Blue_1	292	0.1	Pass	215.5		
5	301.9	1045	5802	Blue_1	302	0.0	Pass	191.6		

Sample 92: Run date and time: 09/11/2020 - 20:27:01 -> 09/11/2020 - 21:12:55

Dye: Blue - 2 peaks - 92.fsa

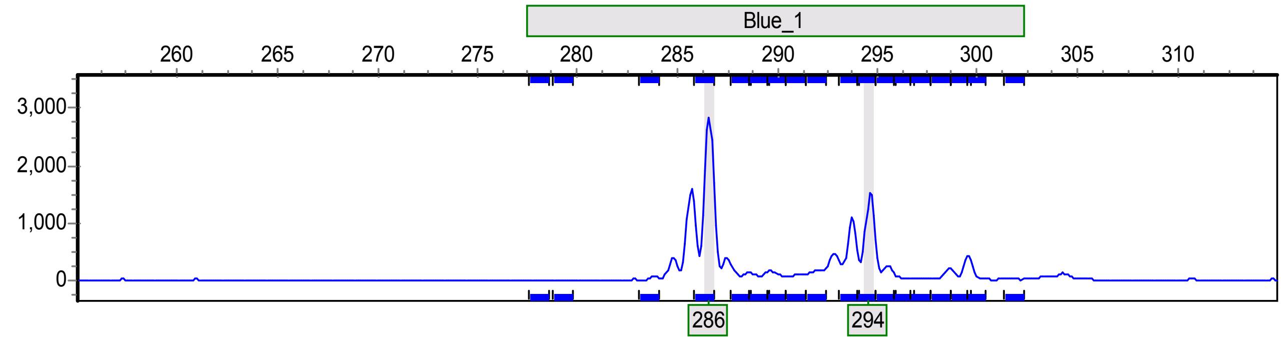

No	Size	Height	Area	Marker	Allele	Difference	Quality	Score	Allele Comments	Sample Comments
1	286.6	2815	14204	Blue_1	286	0.2	Pass	500.0		
2	294.6	1520	7749	Blue_1	294	0.1	Pass	352.2		

Sample 93: Run date and time: 09/11/2020 - 20:27:01 -> 09/11/2020 - 21:12:55

Dye: Blue - 3 peaks - 93.fsa

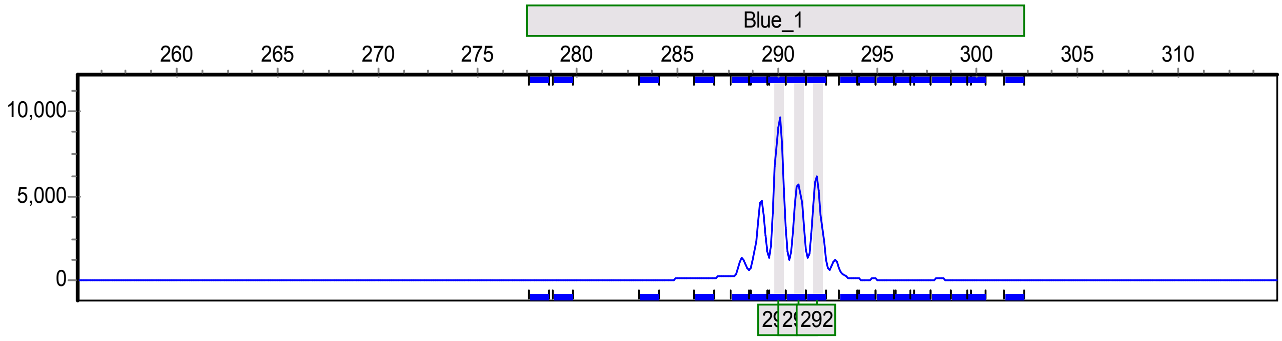

No	Size	Height	Area	Marker	Allele	Difference	Quality	Score	Allele Comments	Sample Comments
1	290.1	9575	48025	Blue_1	290	0.1	Pass	500.0		
2	291.1	5612	29609	Blue_1	291	0.1	Pass	500.0		
3	292.0	6159	32471	Blue_1	292	0.0	Pass	500.0		

Allele Report

Sample 94: Run date and time: 09/11/2020 - 20:27:01 -> 09/11/2020 - 21:12:55

Dye: Blue - 5 peaks - 94.fsa

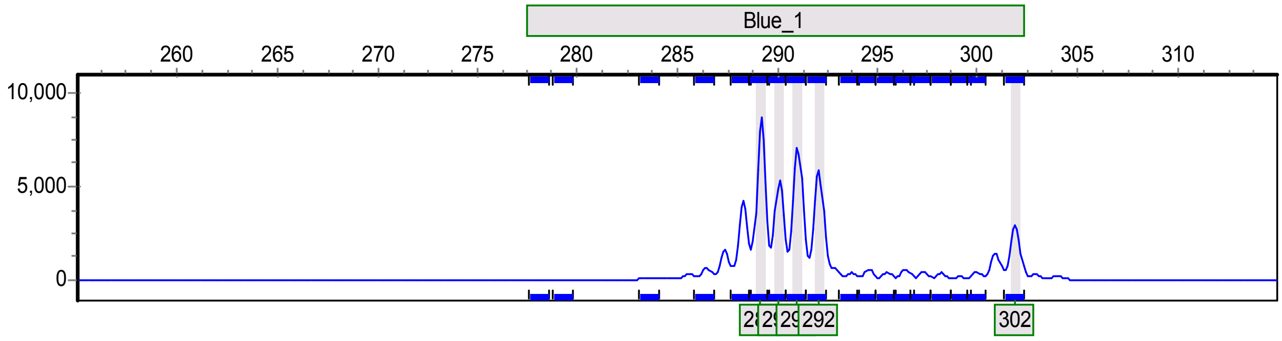

No	Size	Height	Area	Marker	Allele	Difference	Quality	Score	Allele Comments	Sample Comments
1	289.2	8685	47068	Blue_1	289	0.1	Pass	500.0		
2	290.1	5319	30013	Blue_1	290	0.1	Pass	500.0		
3	291.0	7067	38652	Blue_1	291	0.0	Pass	500.0		
4	292.1	5861	32809	Blue_1	292	0.1	Pass	500.0		
5	301.9	2972	17234	Blue_1	302	0.0	Pass	500.0		

Sample 95: Run date and time: 09/11/2020 - 20:27:01 -> 09/11/2020 - 21:12:55

Dye: Blue - 4 peaks - 95.fsa

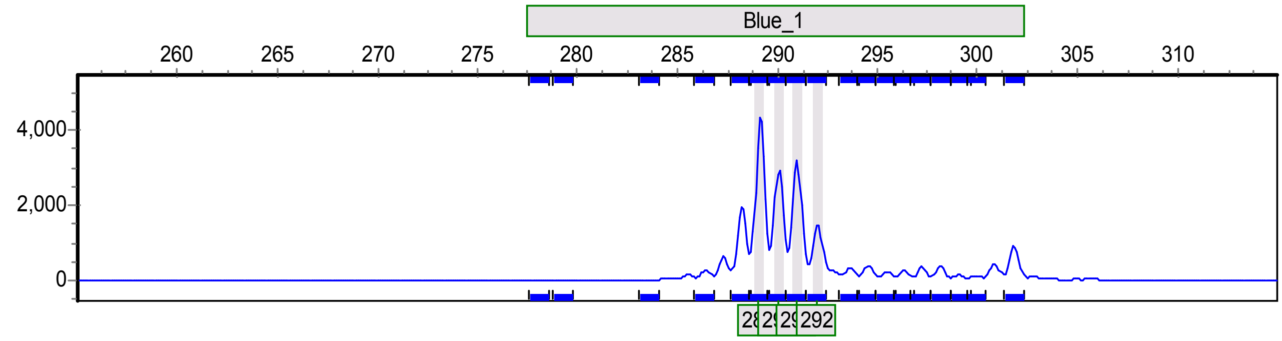

No	Size	Height	Area	Marker	Allele	Difference	Quality	Score	Allele Comments	Sample Comments
1	289.1	4288	22765	Blue_1	289	0.0	Pass	500.0		
2	290.1	2900	15386	Blue_1	290	0.1	Pass	500.0		
3	291.0	3161	16997	Blue_1	291	0.0	Pass	500.0		
4	292.0	1474	8505	Blue_1	292	0.0	Pass	279.0		

Sample 96: Run date and time: 09/11/2020 - 20:27:01 -> 09/11/2020 - 21:12:55

Dye: Blue - 3 peaks - 96.fsa

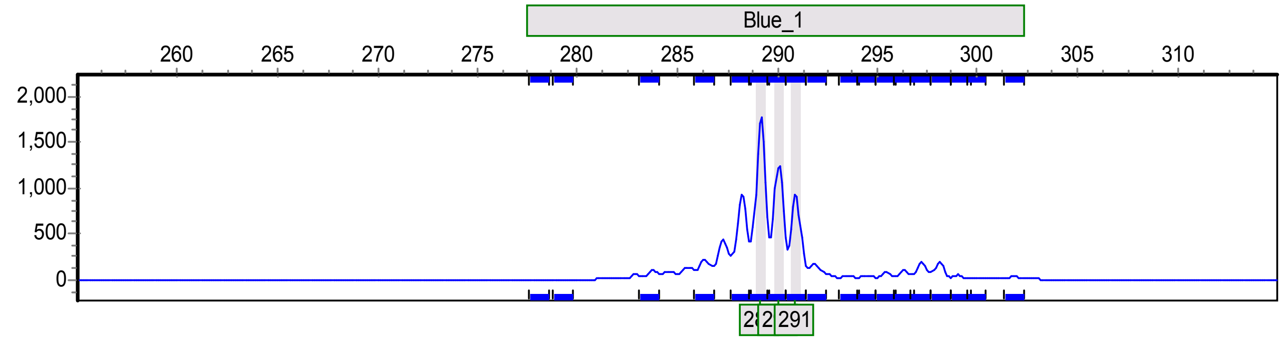

No	Size	Height	Area	Marker	Allele	Difference	Quality	Score	Allele Comments	Sample Comments
1	289.2	1764	9513	Blue_1	289	0.1	Pass	378.0		
2	290.1	1253	6859	Blue_1	290	0.1	Pass	218.7		
3	290.9	943	5103	Blue_1	291	0.1	Pass	145.9		
