# Supplementary material for: Genetic diversity and population structure of Polygonatum cyrtonema Hua in China using SSR markers
Source: PLoS One. 2023 Aug 31;18(8):e0290605. doi: 10.1371/journal.pone.0290605 (PMC10470896; doi:10.1371/journal.pone.0290605)
Supplement: S1 Data — (ZIP) [file pone.0290605.s002.zip › ╨┬╜¿╬─╝■╝╨ (2)/╥2╬∩24/yinwu24.pdf]

Allele Report

Sample 1: Run date and time: 09/15/2020 - 19:38:20 -> 09/15/2020 - 20:24:32

Dye: Blue - 0 peaks - 1.fsa

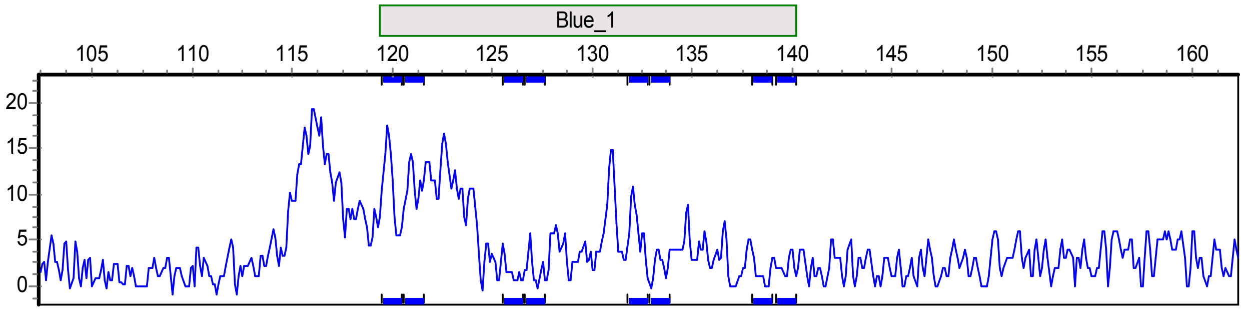

No	Size	Height	Area	Marker	Allele	Difference	Quality	Score	Allele Comments	Sample Comments

Sample 2: Run date and time: 09/15/2020 - 19:38:20 -> 09/15/2020 - 20:24:32

Dye: Blue - 0 peaks - 10.fsa

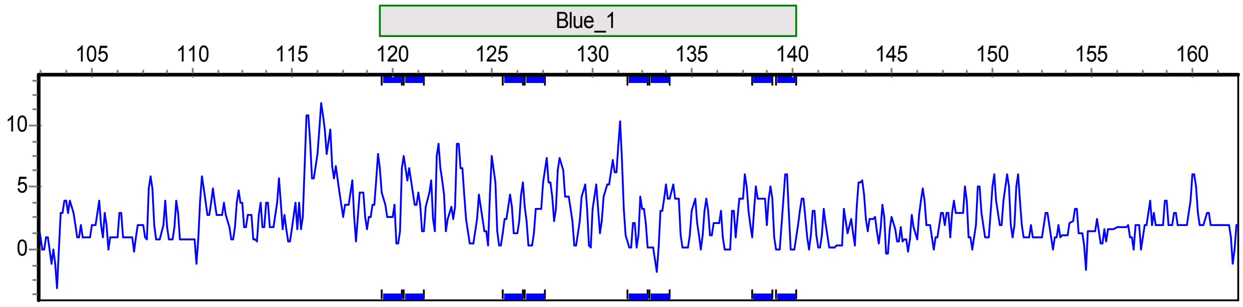

No	Size	Height	Area	Marker	Allele	Difference	Quality	Score	Allele Comments	Sample Comments

Sample 3: Run date and time: 09/15/2020 - 19:38:20 -> 09/15/2020 - 20:24:32

Dye: Blue - 0 peaks - 11.fsa

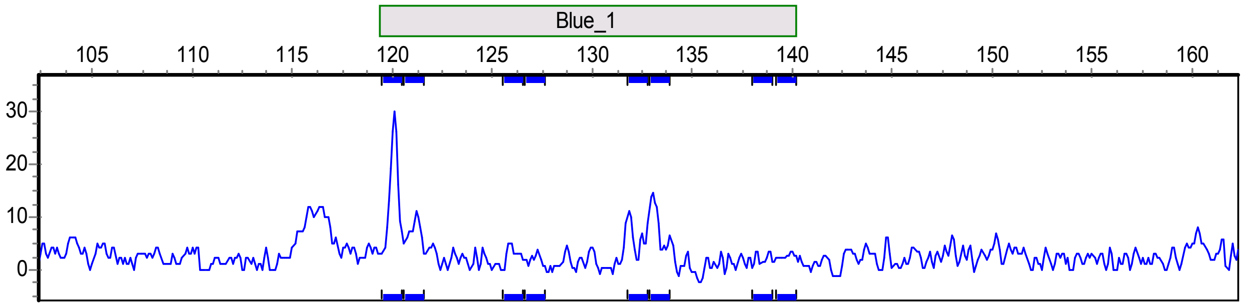

No	Size	Height	Area	Marker	Allele	Difference	Quality	Score	Allele Comments	Sample Comments

Sample 4: Run date and time: 09/15/2020 - 19:38:20 -> 09/15/2020 - 20:24:32

Dye: Blue - 0 peaks - 12.fsa

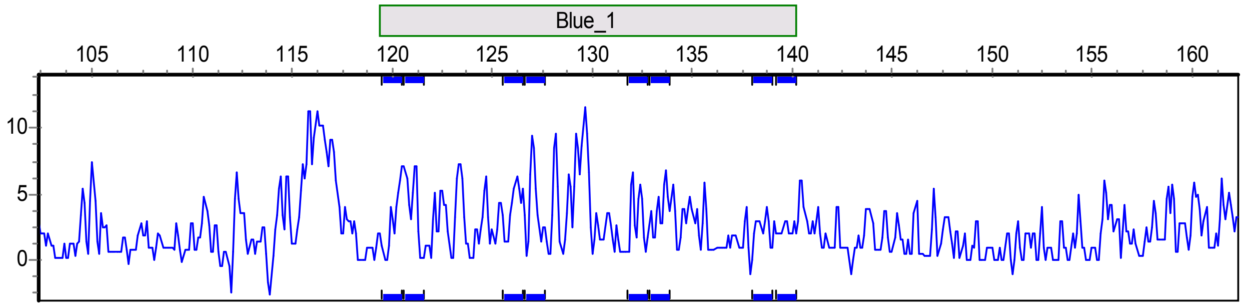

No	Size	Height	Area	Marker	Allele	Difference	Quality	Score	Allele Comments	Sample Comments

Sample 5: Run date and time: 09/15/2020 - 19:38:20 -> 09/15/2020 - 20:24:32

Dye: Blue - 0 peaks - 13.fsa

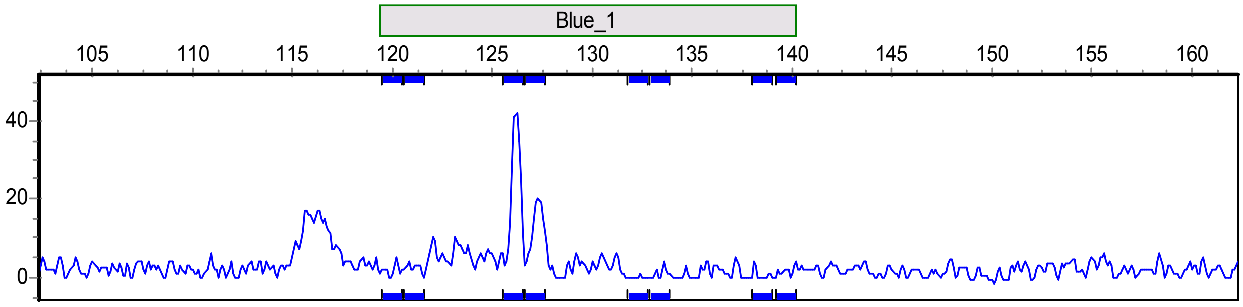

No	Size	Height	Area	Marker	Allele	Difference	Quality	Score	Allele Comments	Sample Comments

Sample 6: Run date and time: 09/15/2020 - 19:38:20 -> 09/15/2020 - 20:24:32

Dye: Blue - 0 peaks - 14.fsa

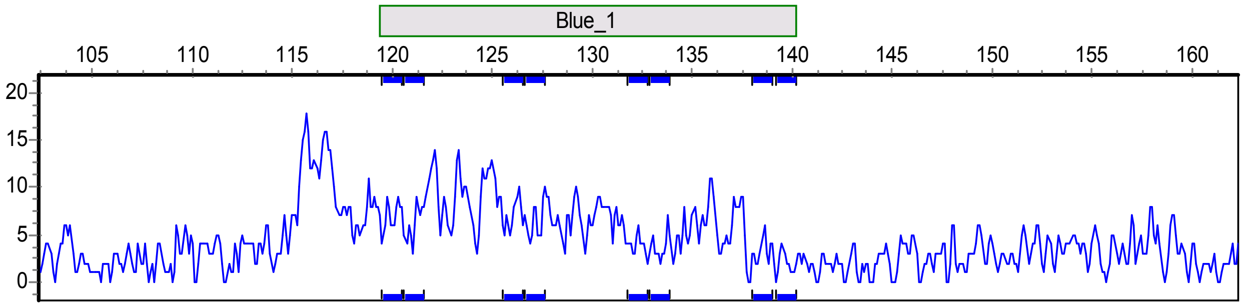

No	Size	Height	Area	Marker	Allele	Difference	Quality	Score	Allele Comments	Sample Comments

Sample 7: Run date and time: 09/15/2020 - 19:38:20 -> 09/15/2020 - 20:24:32

Dye: Blue - 2 peaks - 15.fsa

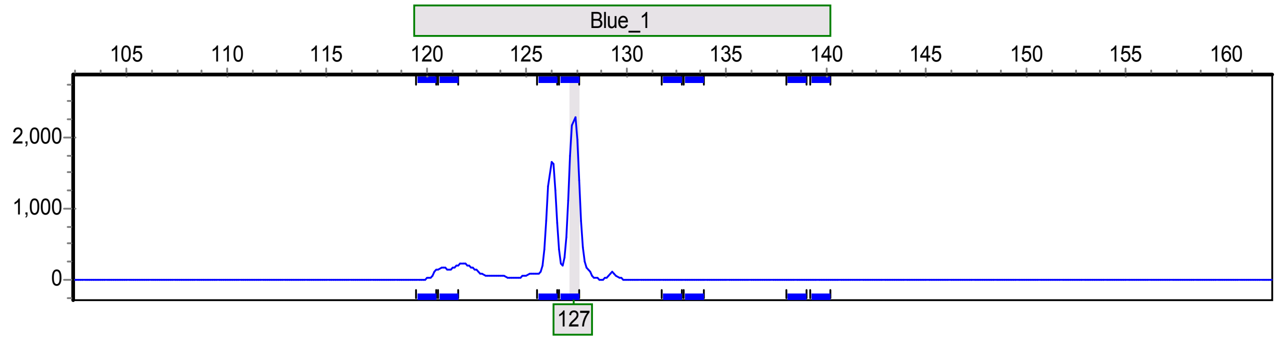

No	Size	Height	Area	Marker	Allele	Difference	Quality	Score	Allele Comments	Sample Comments
1	126.2	1641	8256	Blue_1	126	0.1	Pass	422.8	[<Deleted>]	
2	127.4	2259	12249	Blue_1	127	0.2	Pass	500.0		

Sample 8: Run date and time: 09/15/2020 - 19:38:20 -> 09/15/2020 - 20:24:32

Dye: Blue - 2 peaks - 16.fsa

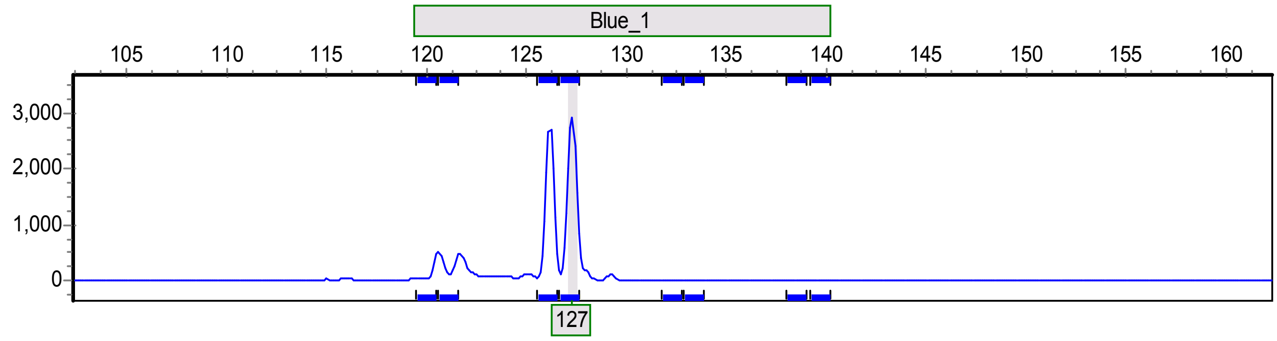

No	Size	Height	Area	Marker	Allele	Difference	Quality	Score	Allele Comments	Sample Comments
1	126.2	2693	11841	Blue_1	126	0.1	Pass	500.0	[<Deleted>]	
2	127.3	2909	13993	Blue_1	127	0.1	Pass	500.0		

Sample 9: Run date and time: 09/15/2020 - 19:38:20 -> 09/15/2020 - 20:24:32

Dye: Blue - 2 peaks - 17.fsa

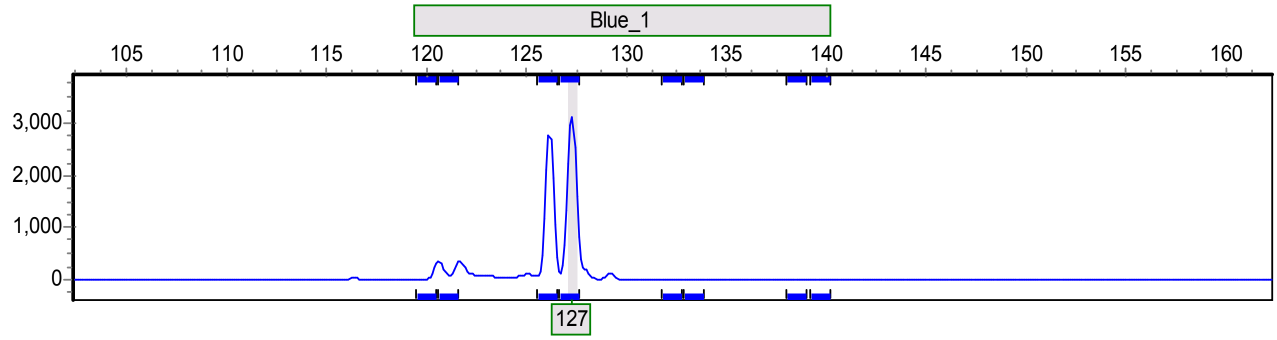

No	Size	Height	Area	Marker	Allele	Difference	Quality	Score	Allele Comments	Sample Comments
1	126.1	2745	12021	Blue_1	126	0.0	Pass	500.0	[<Deleted>]	
2	127.3	3089	14837	Blue_1	127	0.1	Pass	500.0		

Sample 10: Run date and time: 09/15/2020 - 19:38:20 -> 09/15/2020 - 20:24:32

Dye: Blue - 0 peaks - 18.fsa

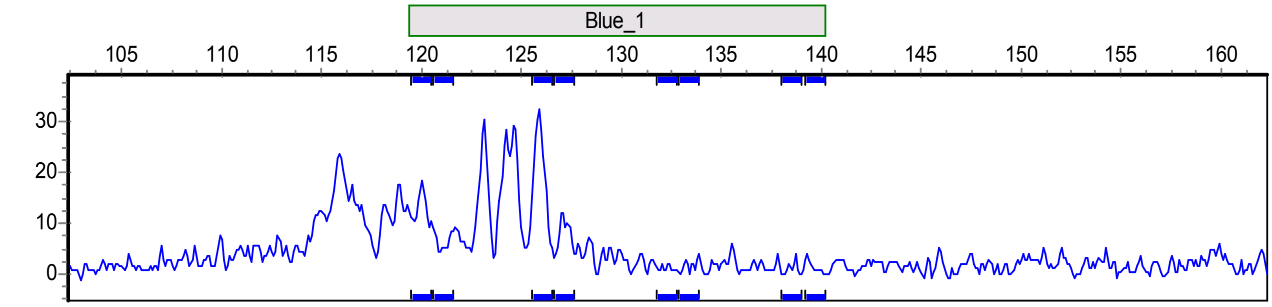

No	Size	Height	Area	Marker	Allele	Difference	Quality	Score	Allele Comments	Sample Comments

Sample 11: Run date and time: 09/15/2020 - 19:38:20 -> 09/15/2020 - 20:24:32

Dye: Blue - 2 peaks - 19.fsa

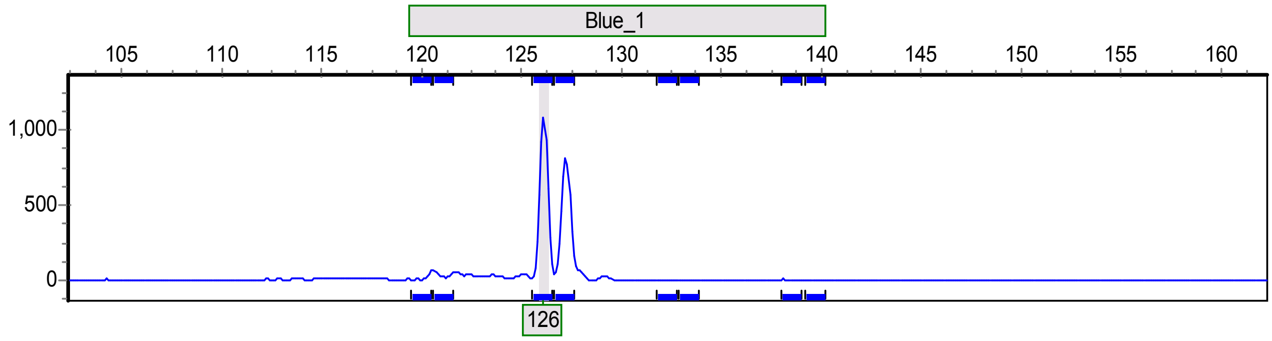

No	Size	Height	Area	Marker	Allele	Difference	Quality	Score	Allele Comments	Sample Comments
1	126.1	1076	4539	Blue_1	126	0.0	Pass	288.5		
2	127.2	818	3973	Blue_1	127	0.0	Pass	161.4	[<Deleted>]	

Sample 12: Run date and time: 09/15/2020 - 19:38:20 -> 09/15/2020 - 20:24:32

Dye: Blue - 4 peaks - 2.fsa

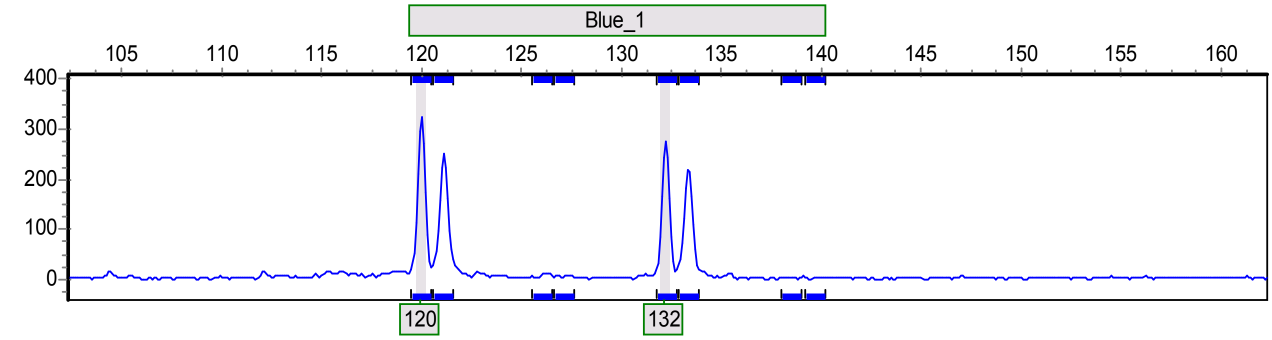

No	Size	Height	Area	Marker	Allele	Difference	Quality	Score	Allele Comments	Sample Comments
1	120.0	323	1479	Blue_1	120	0.0	Pass	41.1		
2	121.1	250	1296	Blue_1	121	0.0	Pass	22.4	[<Deleted>]	
3	132.2	277	1254	Blue_1	132	0.1	Pass	32.8		
4	133.3	219	1150	Blue_1	133	0.1	Pass	17.3	[<Deleted>]	

Sample 13: Run date and time: 09/15/2020 - 19:38:20 -> 09/15/2020 - 20:24:32

Dye: Blue - 2 peaks - 20.fsa

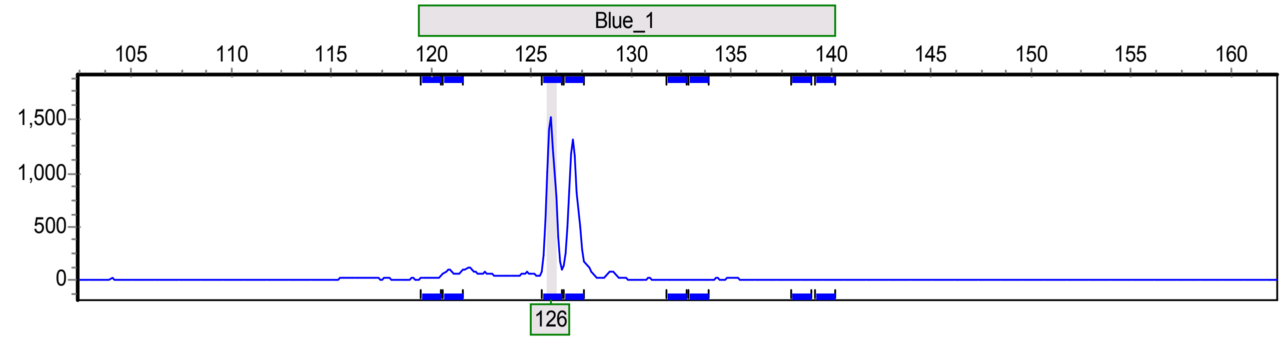

No	Size	Height	Area	Marker	Allele	Difference	Quality	Score	Allele Comments	Sample Comments
1	126.0	1519	6926	Blue_1	126	0.1	Pass	425.7		
2	127.1	1318	6623	Blue_1	127	0.1	Pass	308.0	[<Deleted>]	

Sample 14: Run date and time: 09/15/2020 - 19:38:20 -> 09/15/2020 - 20:24:32

Dye: Blue - 2 peaks - 21.fsa

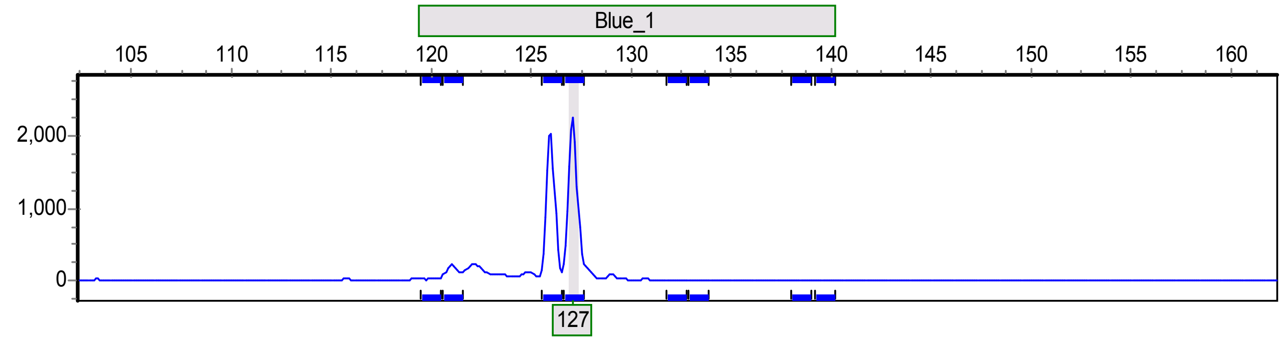

No	Size	Height	Area	Marker	Allele	Difference	Quality	Score	Allele Comments	Sample Comments
1	126.0	2028	9326	Blue_1	126	0.1	Pass	500.0	[<Deleted>]	
2	127.1	2244	11214	Blue_1	127	0.1	Pass	500.0		

Sample 15: Run date and time: 09/15/2020 - 19:38:20 -> 09/15/2020 - 20:24:32

Dye: Blue - 2 peaks - 22.fsa

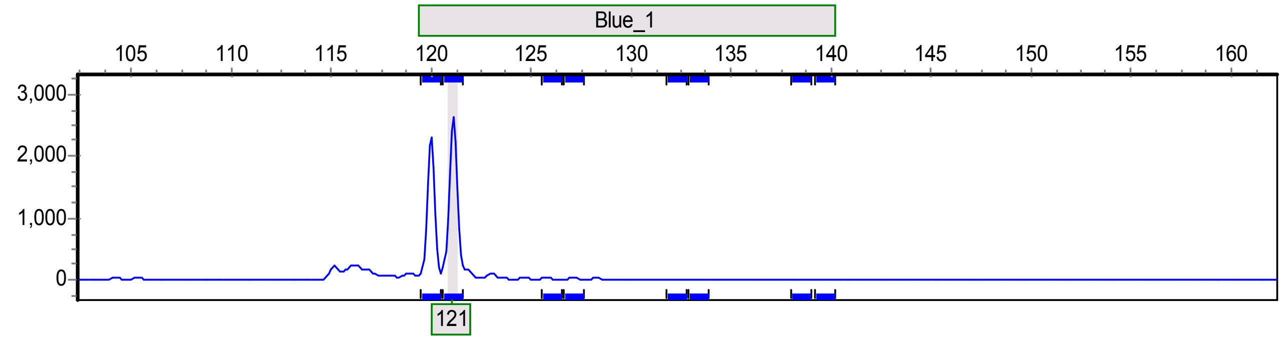

No	Size	Height	Area	Marker	Allele	Difference	Quality	Score	Allele Comments	Sample Comments
1	120.0	2296	10017	Blue_1	120	0.0	Pass	500.0	[<Deleted>]	
2	121.1	2620	12459	Blue_1	121	0.0	Pass	500.0		

Allele Report

Sample 16: Run date and time: 09/15/2020 - 19:38:20 -> 09/15/2020 - 20:24:32

Dye: Blue - 2 peaks - 23.fsa

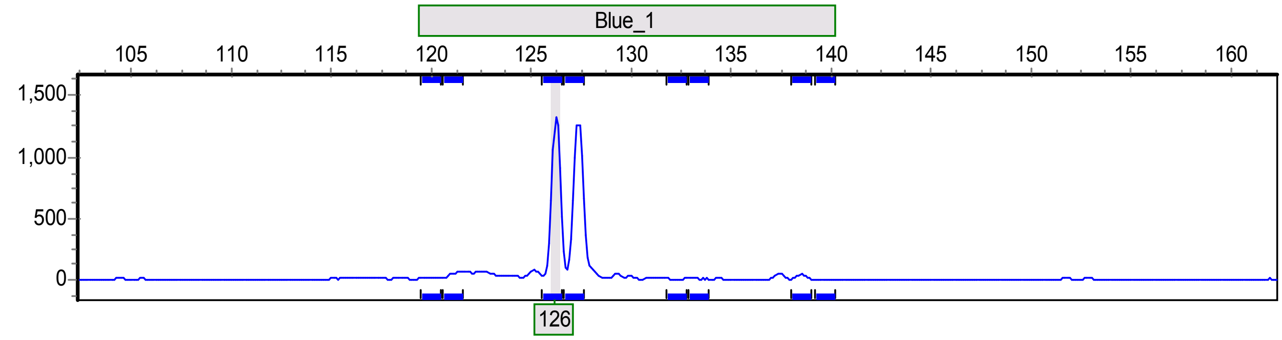

No	Size	Height	Area	Marker	Allele	Difference	Quality	Score	Allele Comments	Sample Comments
1	126.2	1317	5996	Blue_1	126	0.1	Pass	351.3		
2	127.4	1255	6500	Blue_1	127	0.2	Pass	276.3	[<Deleted>]	

Sample 17: Run date and time: 09/15/2020 - 19:38:20 -> 09/15/2020 - 20:24:32

Dye: Blue - 0 peaks - 24.fsa

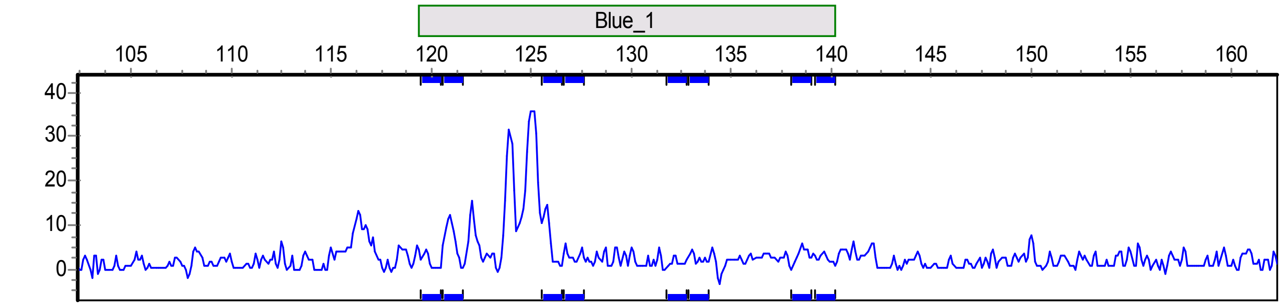

No	Size	Height	Area	Marker	Allele	Difference	Quality	Score	Allele Comments	Sample Comments

Sample 18: Run date and time: 09/15/2020 - 19:38:20 -> 09/15/2020 - 20:24:32

Dye: Blue - 0 peaks - 25.fsa

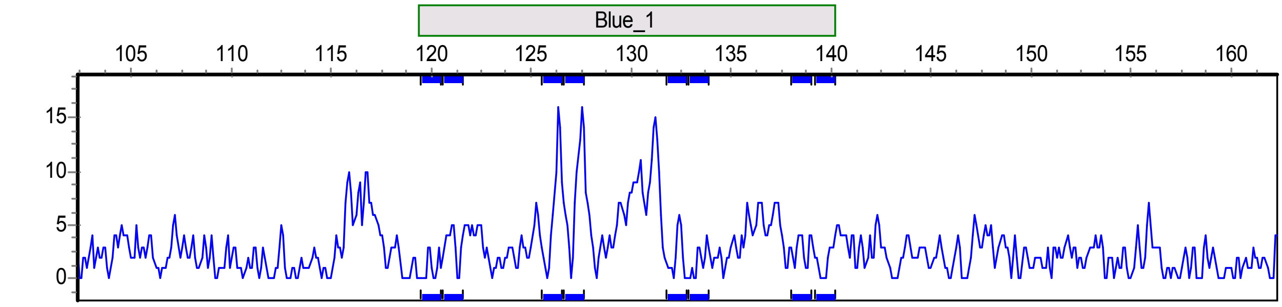

No	Size	Height	Area	Marker	Allele	Difference	Quality	Score	Allele Comments	Sample Comments

Sample 19: Run date and time: 09/15/2020 - 19:38:20 -> 09/15/2020 - 20:24:32

Dye: Blue - 2 peaks - 26.fsa

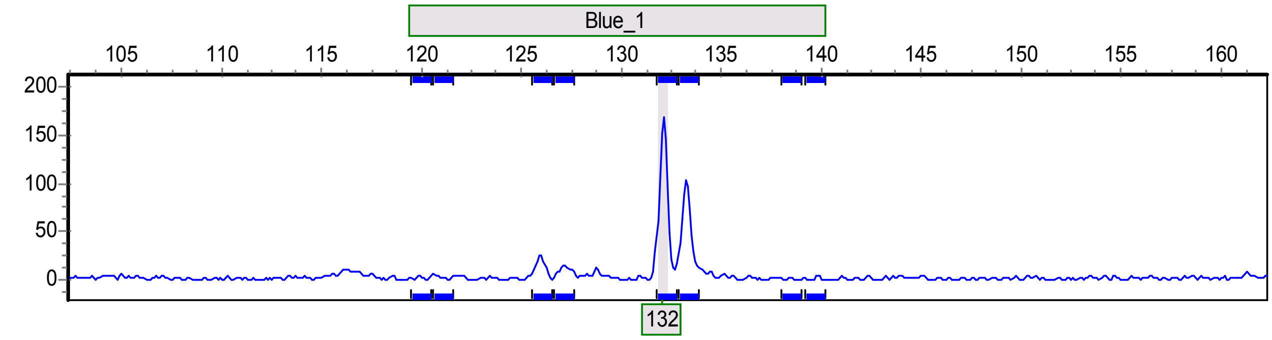

No	Size	Height	Area	Marker	Allele	Difference	Quality	Score	Allele Comments	Sample Comments
1	132.1	168	786	Blue_1	132	0.2	Pass	13.2		
2	133.2	103	552	Blue_1	133	0.2	Check	4.5	[<Deleted>]	

Sample 20: Run date and time: 09/15/2020 - 19:38:20 -> 09/15/2020 - 20:24:32

Dye: Blue - 2 peaks - 27.fsa

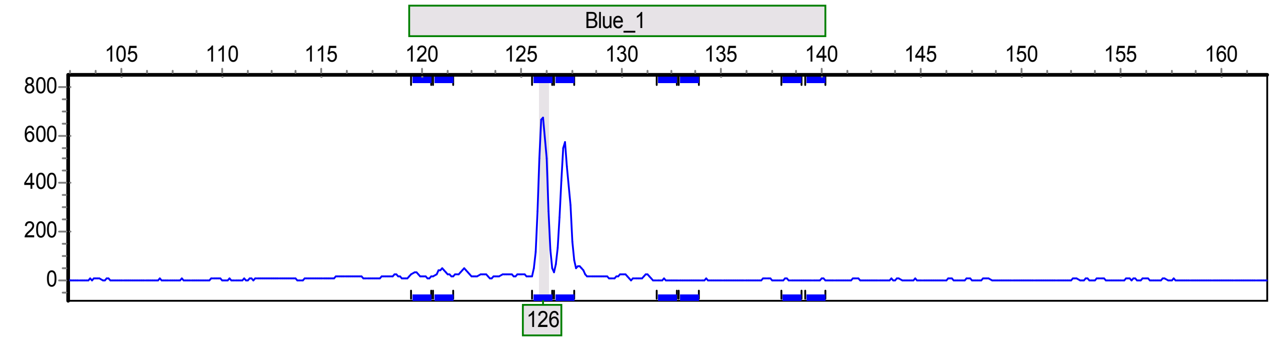

No	Size	Height	Area	Marker	Allele	Difference	Quality	Score	Allele Comments	Sample Comments
1	126.1	669	3006	Blue_1	126	0.0	Pass	132.6		
2	127.2	573	2828	Blue_1	127	0.0	Pass	92.7	[<Deleted>]	

Sample 21: Run date and time: 09/15/2020 - 19:38:20 -> 09/15/2020 - 20:24:32

Dye: Blue - 2 peaks - 28.fsa

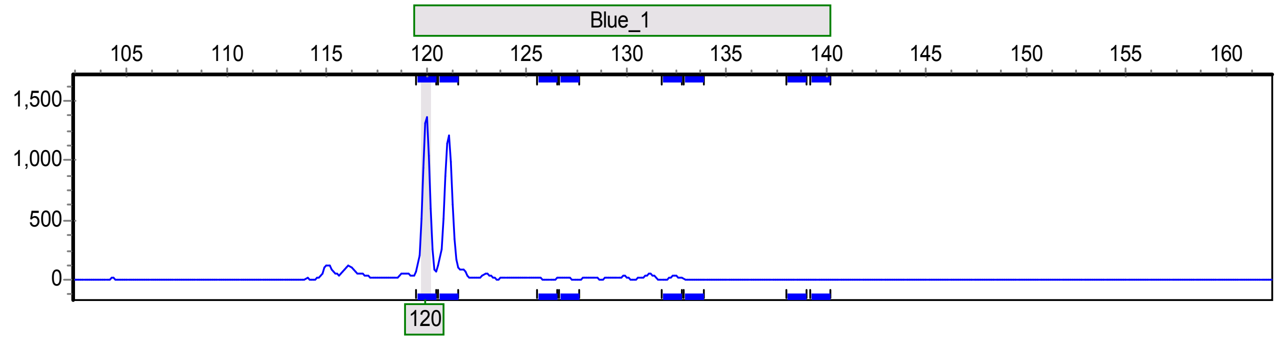

No	Size	Height	Area	Marker	Allele	Difference	Quality	Score	Allele Comments	Sample Comments
1	120.0	1349	5896	Blue_1	120	0.0	Pass	380.3		
2	121.1	1199	5827	Blue_1	121	0.0	Pass	280.8	[<Deleted>]	

Sample 22: Run date and time: 09/15/2020 - 19:38:20 -> 09/15/2020 - 20:24:32

Dye: Blue - 0 peaks - 29.fsa

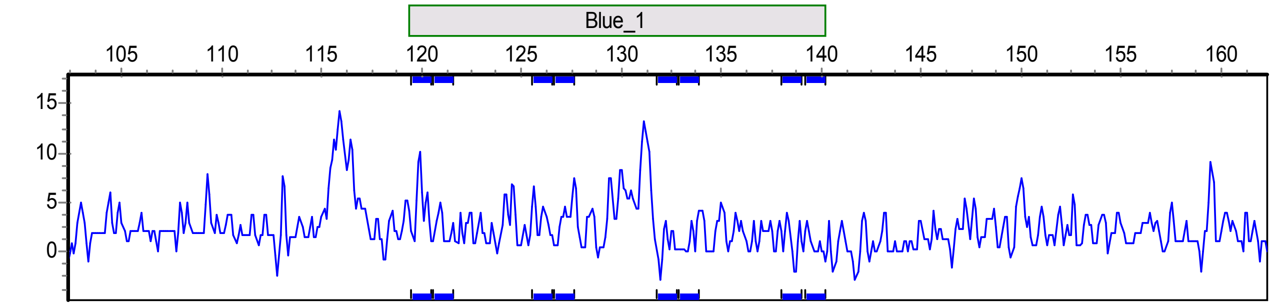

No	Size	Height	Area	Marker	Allele	Difference	Quality	Score	Allele Comments	Sample Comments

Sample 23: Run date and time: 09/15/2020 - 19:38:20 -> 09/15/2020 - 20:24:32

Dye: Blue - 2 peaks - 3.fsa

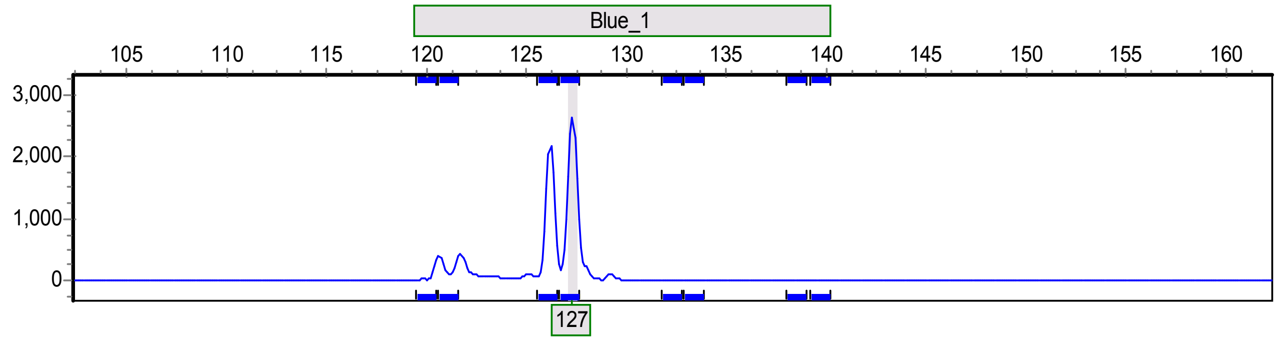

No	Size	Height	Area	Marker	Allele	Difference	Quality	Score	Allele Comments	Sample Comments
1	126.2	2175	9930	Blue_1	126	0.1	Pass	500.0	[<Deleted>]	
2	127.3	2624	13128	Blue_1	127	0.1	Pass	500.0		

Sample 24: Run date and time: 09/15/2020 - 19:38:20 -> 09/15/2020 - 20:24:32

Dye: Blue - 2 peaks - 30.fsa

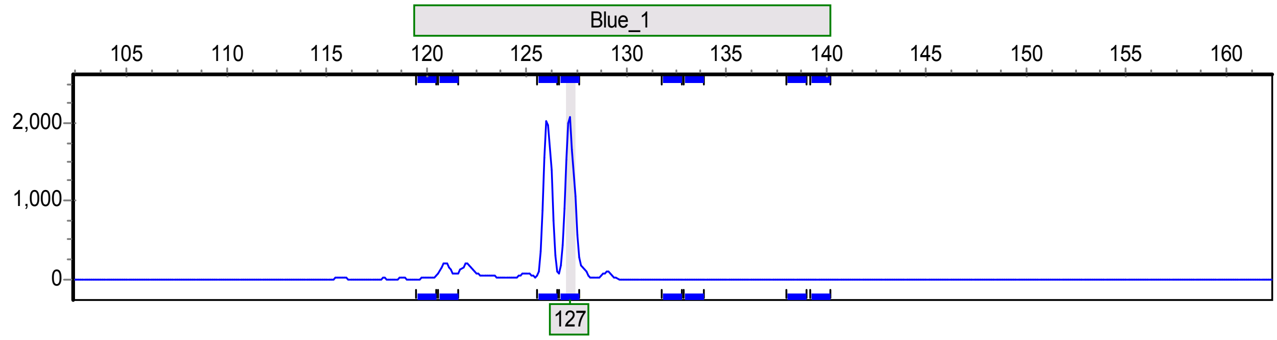

No	Size	Height	Area	Marker	Allele	Difference	Quality	Score	Allele Comments	Sample Comments
1	126.0	2009	8753	Blue_1	126	0.1	Pass	500.0	[<Deleted>]	
2	127.2	2059	10065	Blue_1	127	0.0	Pass	500.0		

Allele Report

Sample 25: Run date and time: 09/15/2020 - 19:38:20 -> 09/15/2020 - 20:24:32

Dye: Blue - 0 peaks - 31.fsa

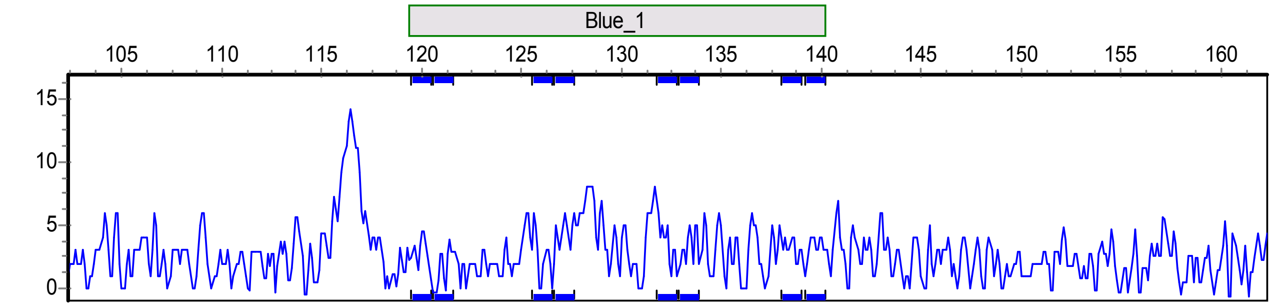

No	Size	Height	Area	Marker	Allele	Difference	Quality	Score	Allele Comments	Sample Comments

Sample 26: Run date and time: 09/15/2020 - 19:38:20 -> 09/15/2020 - 20:24:32

Dye: Blue - 0 peaks - 32.fsa

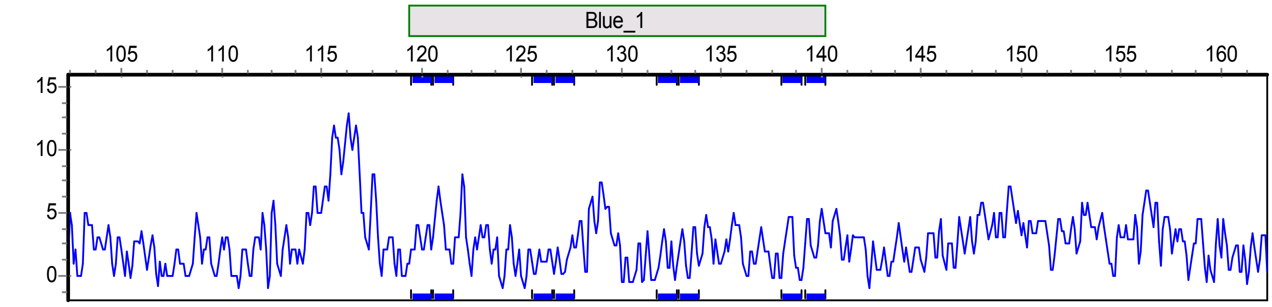

No	Size	Height	Area	Marker	Allele	Difference	Quality	Score	Allele Comments	Sample Comments

Sample 27: Run date and time: 09/15/2020 - 19:38:20 -> 09/15/2020 - 20:24:32

Dye: Blue - 3 peaks - 33.fsa

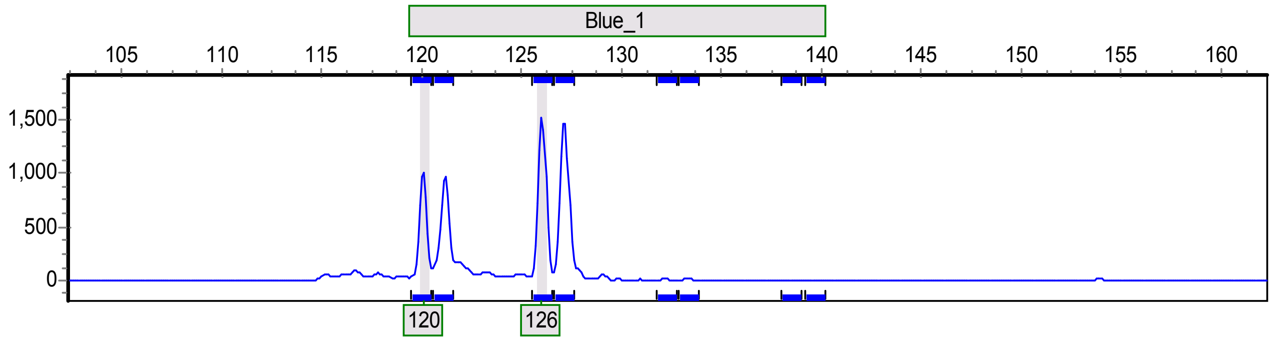

No	Size	Height	Area	Marker	Allele	Difference	Quality	Score	Allele Comments	Sample Comments
1	120.1	1001	4362	Blue_1	120	0.1	Pass	243.5		
2	126.0	1502	6496	Blue_1	126	0.1	Pass	448.5		
3	127.1	1455	7228	Blue_1	127	0.1	Pass	362.5	[<Deleted>]	

Allele Report

Sample 28: Run date and time: 09/15/2020 - 19:38:20 -> 09/15/2020 - 20:24:32

Dye: Blue - 3 peaks - 34.fsa

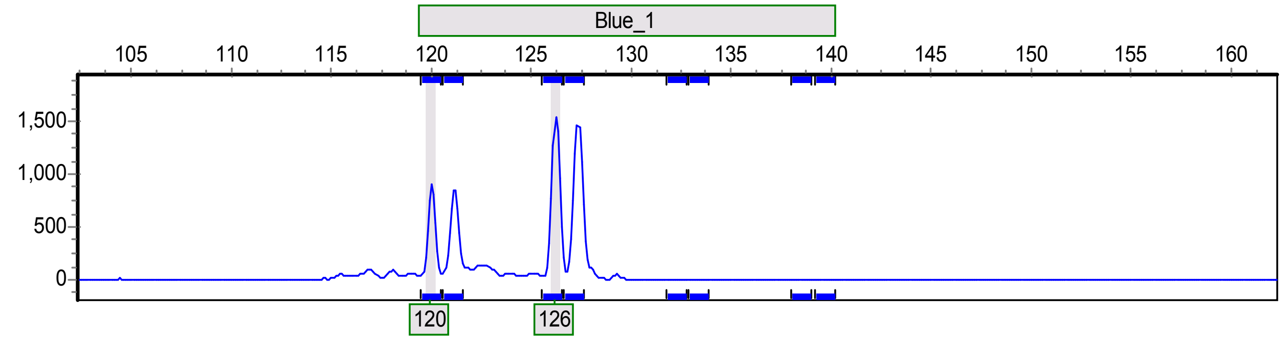

No	Size	Height	Area	Marker	Allele	Difference	Quality	Score	Allele Comments	Sample Comments
1	120.0	915	3958	Blue_1	120	0.0	Pass	220.7		
2	126.2	1538	6747	Blue_1	126	0.1	Pass	455.2		
3	127.3	1472	7381	Blue_1	127	0.1	Pass	365.1	[<Deleted>]	

Sample 29: Run date and time: 09/15/2020 - 19:38:20 -> 09/15/2020 - 20:24:32

Dye: Blue - 2 peaks - 35.fsa

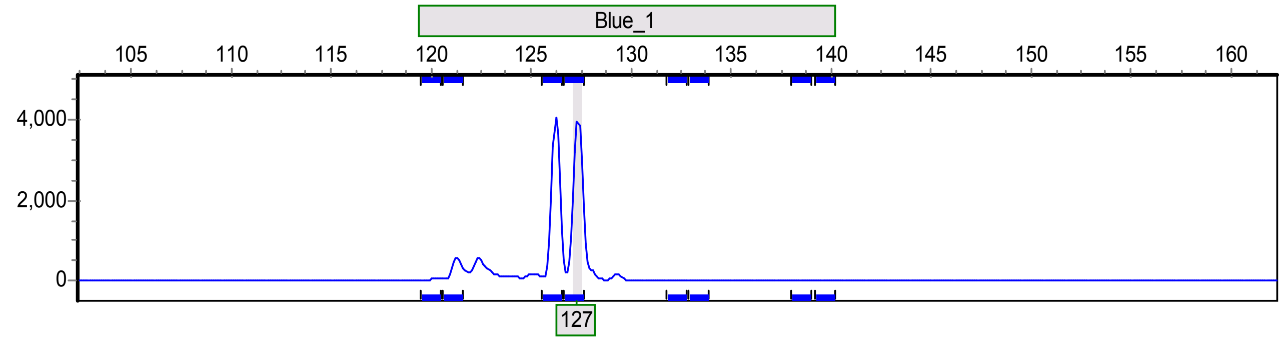

No	Size	Height	Area	Marker	Allele	Difference	Quality	Score	Allele Comments	Sample Comments
1	126.2	4045	17510	Blue_1	126	0.1	Pass	500.0	[<Deleted>]	
2	127.3	3975	19454	Blue_1	127	0.1	Pass	500.0		

Sample 30: Run date and time: 09/15/2020 - 19:38:20 -> 09/15/2020 - 20:24:32

Dye: Blue - 2 peaks - 36.fsa

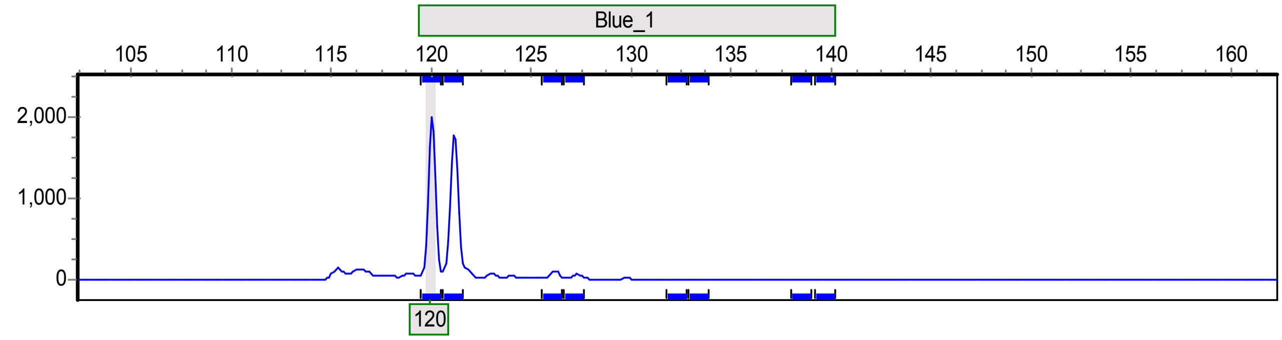

No	Size	Height	Area	Marker	Allele	Difference	Quality	Score	Allele Comments	Sample Comments
1	120.0	1986	8559	Blue_1	120	0.0	Pass	500.0		
2	121.1	1767	8686	Blue_1	121	0.0	Pass	480.4	[<Deleted>]	

Allele Report

Sample 31: Run date and time: 09/15/2020 - 19:38:20 -> 09/15/2020 - 20:24:32

Dye: Blue - 2 peaks - 37.fsa

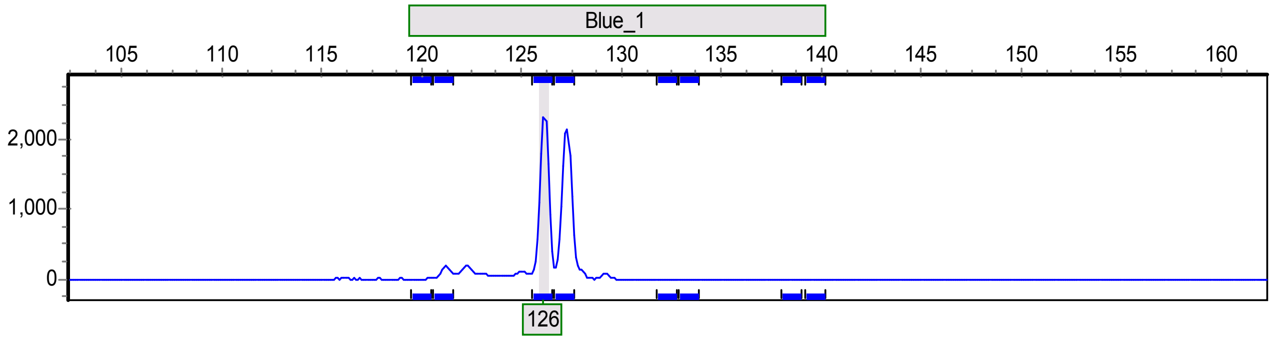

No	Size	Height	Area	Marker	Allele	Difference	Quality	Score	Allele Comments	Sample Comments
1	126.1	2303	10676	Blue_1	126	0.0	Pass	500.0		
2	127.3	2150	11112	Blue_1	127	0.1	Pass	500.0	[<Deleted>]	

Sample 32: Run date and time: 09/15/2020 - 19:38:20 -> 09/15/2020 - 20:24:32

Dye: Blue - 2 peaks - 38.fsa

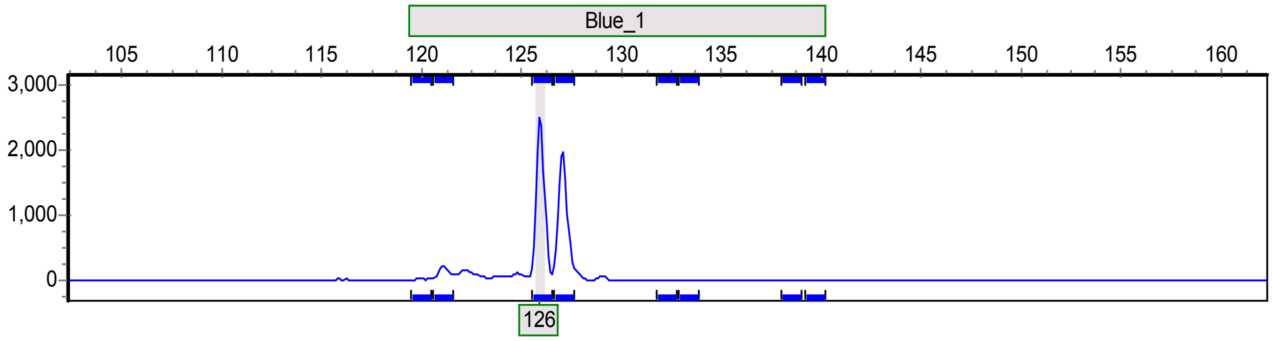

No	Size	Height	Area	Marker	Allele	Difference	Quality	Score	Allele Comments	Sample Comments
1	125.9	2473	10875	Blue_1	126	0.2	Pass	500.0		
2	127.1	1949	9783	Blue_1	127	0.1	Pass	500.0	[<Deleted>]	

Sample 33: Run date and time: 09/15/2020 - 19:38:20 -> 09/15/2020 - 20:24:32

Dye: Blue - 3 peaks - 39.fsa

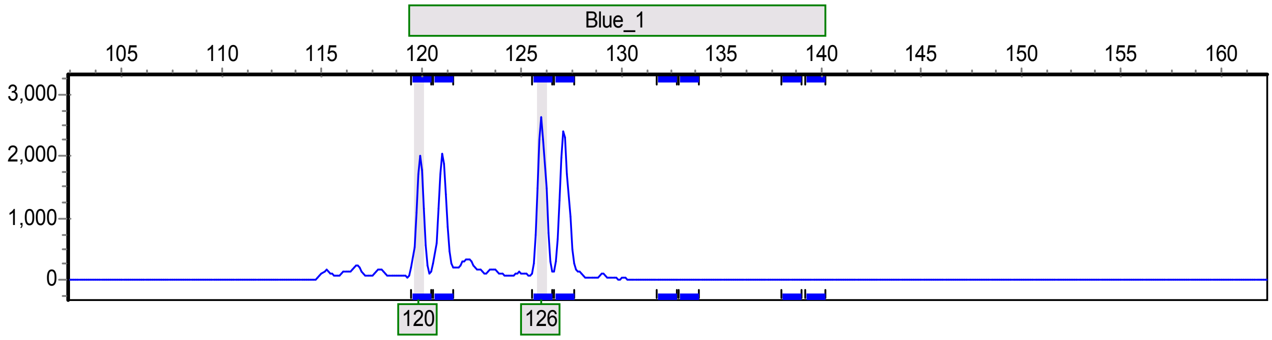

No	Size	Height	Area	Marker	Allele	Difference	Quality	Score	Allele Comments	Sample Comments
1	119.9	2015	8710	Blue_1	120	0.1	Pass	500.0		
2	126.0	2618	11507	Blue_1	126	0.1	Pass	500.0		
3	127.1	2390	11617	Blue_1	127	0.1	Pass	500.0	[<Deleted>]	

Allele Report

Sample 34: Run date and time: 09/15/2020 - 19:38:20 -> 09/15/2020 - 20:24:32

Dye: Blue - 2 peaks - 4.fsa

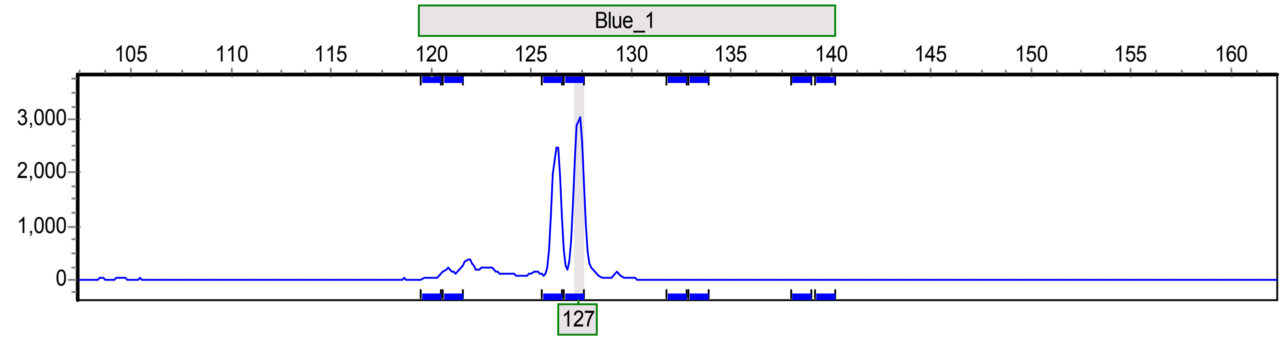

No	Size	Height	Area	Marker	Allele	Difference	Quality	Score	Allele Comments	Sample Comments
1	126.2	2485	12175	Blue_1	126	0.1	Pass	500.0	[<Deleted>]	
2	127.4	3028	15762	Blue_1	127	0.2	Pass	500.0		

Sample 35: Run date and time: 09/15/2020 - 19:38:20 -> 09/15/2020 - 20:24:32

Dye: Blue - 3 peaks - 40.fsa

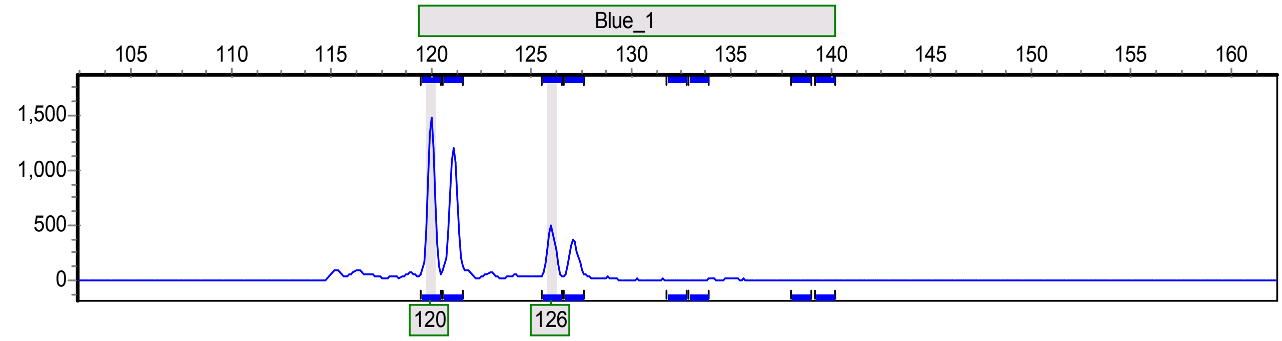

No	Size	Height	Area	Marker	Allele	Difference	Quality	Score	Allele Comments	Sample Comments
1	120.0	1471	6274	Blue_1	120	0.0	Pass	440.4		
2	121.1	1211	5903	Blue_1	121	0.0	Pass	284.6	[<Deleted>]	
3	126.0	496	2218	Blue_1	126	0.1	Pass	84.0		

Sample 36: Run date and time: 09/15/2020 - 19:38:20 -> 09/15/2020 - 20:24:32

Dye: Blue - 2 peaks - 41.fsa

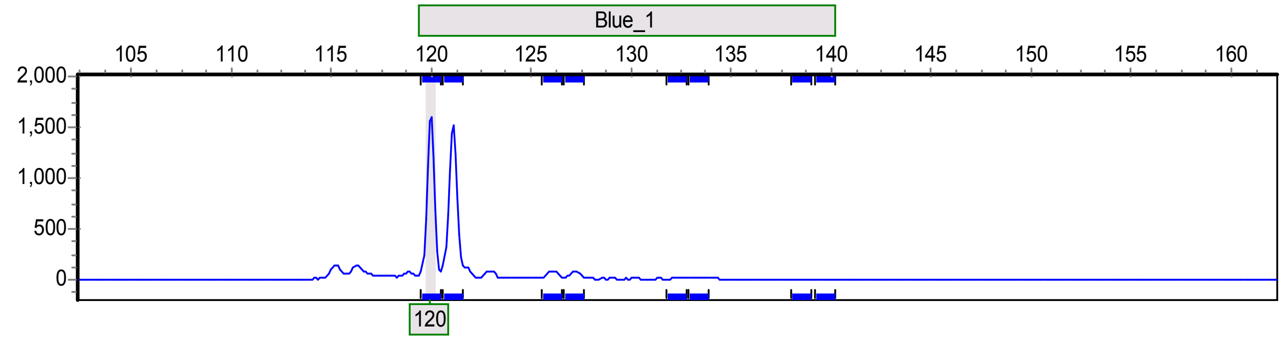

No	Size	Height	Area	Marker	Allele	Difference	Quality	Score	Allele Comments	Sample Comments
1	120.0	1587	6926	Blue_1	120	0.0	Pass	477.3		
2	121.1	1512	7309	Blue_1	121	0.0	Pass	393.7	[<Deleted>]	

Sample 37: Run date and time: 09/15/2020 - 19:38:20 -> 09/15/2020 - 20:24:32

Dye: Blue - 2 peaks - 42.fsa

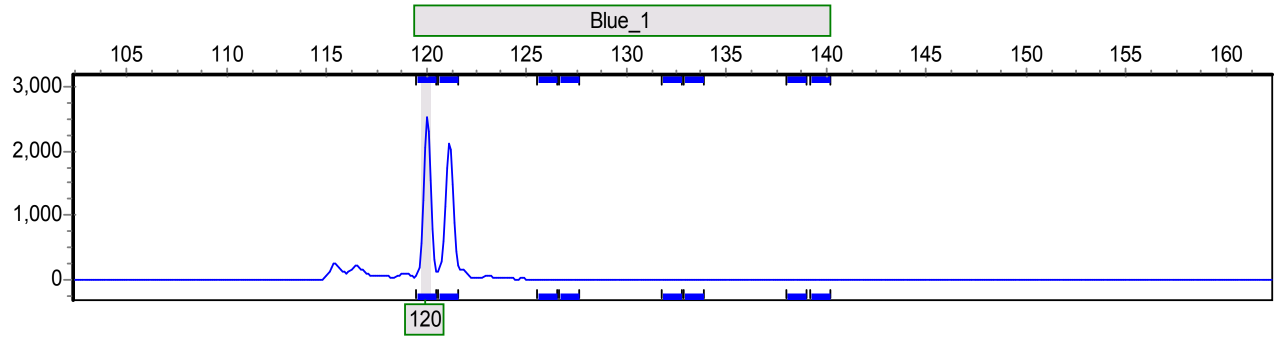

No	Size	Height	Area	Marker	Allele	Difference	Quality	Score	Allele Comments	Sample Comments
1	120.0	2509	10781	Blue_1	120	0.0	Pass	500.0		
2	121.1	2124	10262	Blue_1	121	0.0	Pass	500.0	[<Deleted>]	

Sample 38: Run date and time: 09/15/2020 - 19:38:20 -> 09/15/2020 - 20:24:32

Dye: Blue - 3 peaks - 43.fsa

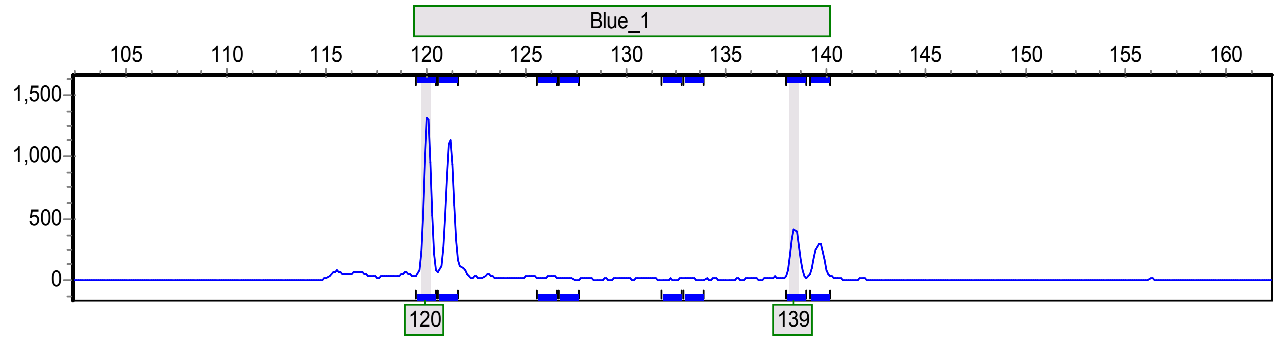

No	Size	Height	Area	Marker	Allele	Difference	Quality	Score	Allele Comments	Sample Comments
1	120.0	1311	5792	Blue_1	120	0.0	Pass	359.6		
2	121.2	1146	5688	Blue_1	121	0.1	Pass	256.1	[<Deleted>]	
3	138.4	408	1843	Blue_1	139	0.1	Pass	60.9		

Sample 39: Run date and time: 09/15/2020 - 19:38:20 -> 09/15/2020 - 20:24:32

Dye: Blue - 4 peaks - 44.fsa

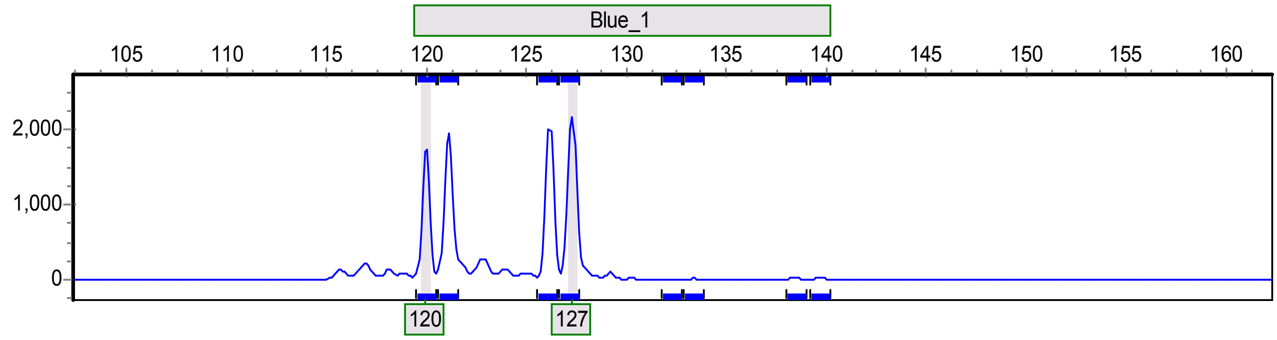

No	Size	Height	Area	Marker	Allele	Difference	Quality	Score	Allele Comments	Sample Comments
1	120.0	1747	7671	Blue_1	120	0.0	Pass	500.0		
2	121.1	1955	9374	Blue_1	121	0.0	Pass	500.0	[<Deleted>]	
3	126.1	1998	8814	Blue_1	126	0.0	Pass	500.0	[<Deleted>]	
4	127.3	2156	10355	Blue_1	127	0.1	Pass	500.0		

Allele Report

Sample 40: Run date and time: 09/15/2020 - 19:38:20 -> 09/15/2020 - 20:24:32

Dye: Blue - 3 peaks - 45.fsa

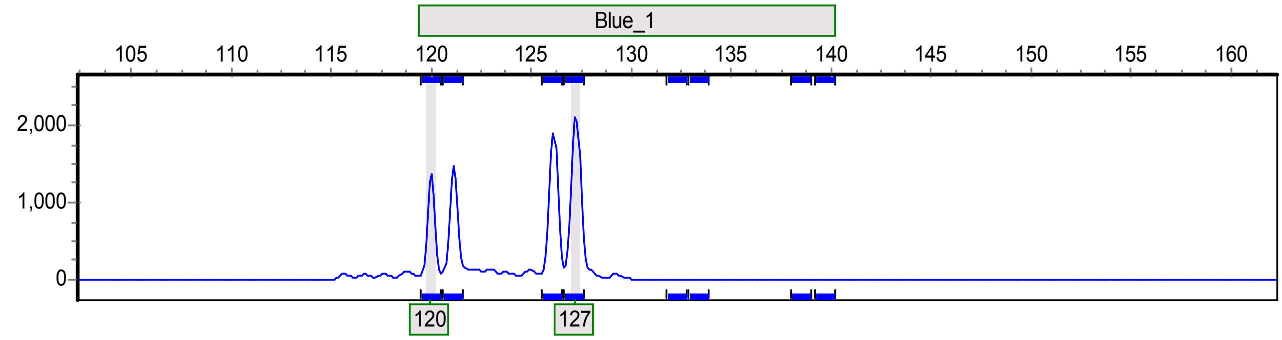

No	Size	Height	Area	Marker	Allele	Difference	Quality	Score	Allele Comments	Sample Comments
1	120.0	1368	5930	Blue_1	120	0.0	Pass	391.3		
2	126.1	1883	8865	Blue_1	126	0.0	Pass	500.0	[<Deleted>]	
3	127.2	2089	10915	Blue_1	127	0.0	Pass	500.0		

Sample 41: Run date and time: 09/15/2020 - 19:38:20 -> 09/15/2020 - 20:24:32

Dye: Blue - 2 peaks - 46.fsa

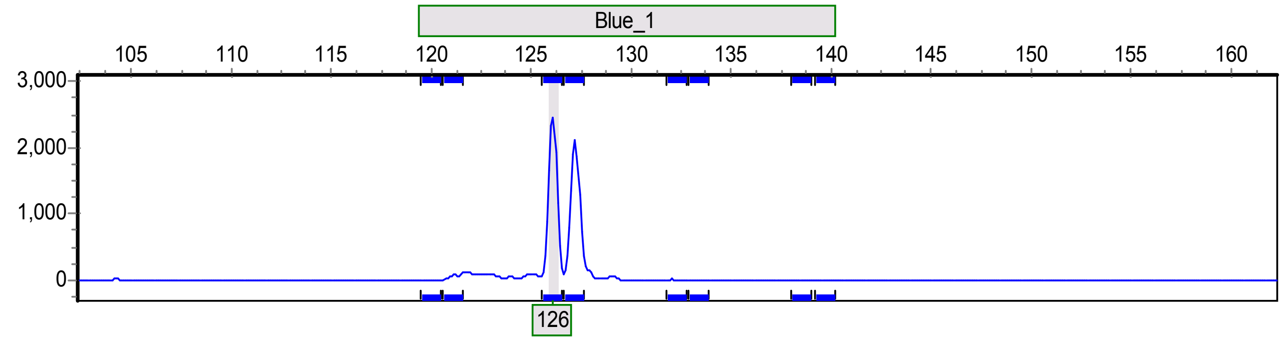

No	Size	Height	Area	Marker	Allele	Difference	Quality	Score	Allele Comments	Sample Comments
1	126.1	2438	10818	Blue_1	126	0.0	Pass	500.0		
2	127.2	2106	10312	Blue_1	127	0.0	Pass	500.0	[<Deleted>]	

Sample 42: Run date and time: 09/15/2020 - 19:38:20 -> 09/15/2020 - 20:24:32

Dye: Blue - 3 peaks - 47.fsa

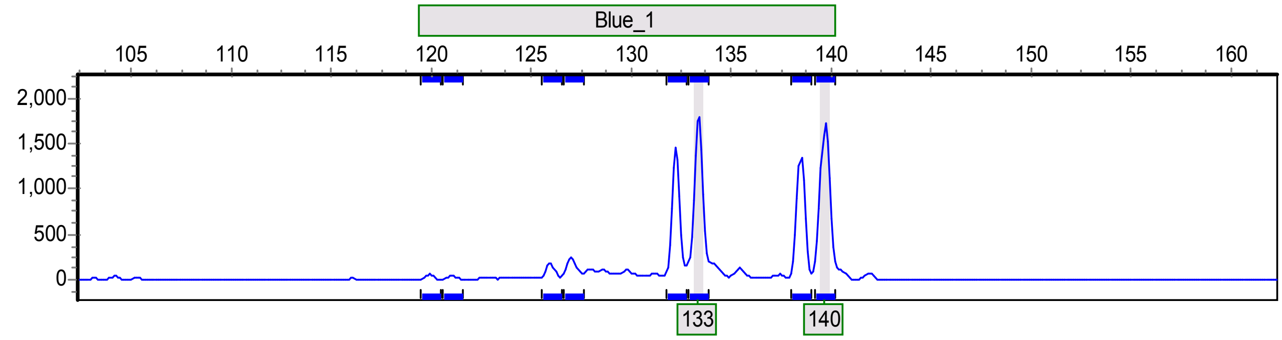

No	Size	Height	Area	Marker	Allele	Difference	Quality	Score	Allele Comments	Sample Comments
1	133.4	1782	9221	Blue_1	133	0.0	Pass	453.5		
2	138.5	1334	5999	Blue_1	139	0.0	Pass	363.4	[<Deleted>]	
3	139.7	1720	9205	Blue_1	140	0.0	Pass	411.6		

Sample 43: Run date and time: 09/15/2020 - 19:38:20 -> 09/15/2020 - 20:24:32

Dye: Blue - 2 peaks - 48.fsa

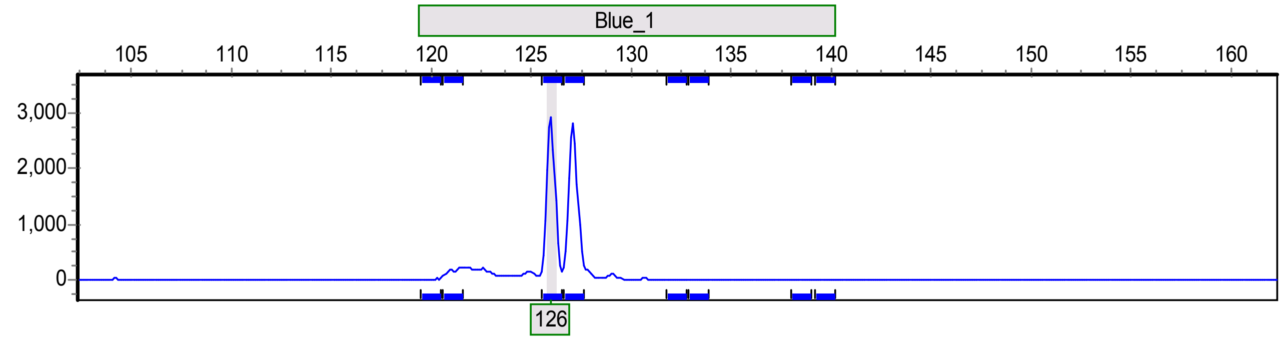

No	Size	Height	Area	Marker	Allele	Difference	Quality	Score	Allele Comments	Sample Comments
1	126.0	2904	12993	Blue_1	126	0.1	Pass	500.0		
2	127.1	2798	13804	Blue_1	127	0.1	Pass	500.0	[<Deleted>]	

Sample 44: Run date and time: 09/15/2020 - 19:38:20 -> 09/15/2020 - 20:24:32

Dye: Blue - 4 peaks - 49.fsa

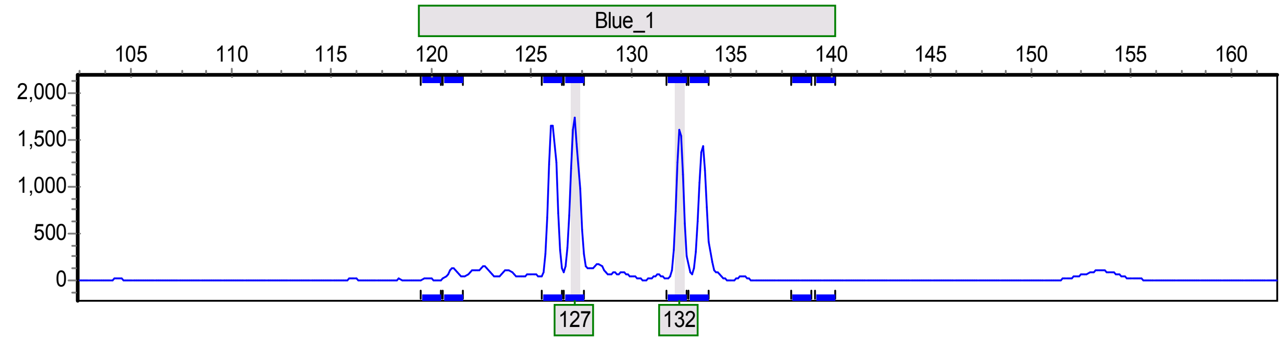

No	Size	Height	Area	Marker	Allele	Difference	Quality	Score	Allele Comments	Sample Comments
1	126.1	1652	7412	Blue_1	126	0.0	Pass	486.8	[<Deleted>]	
2	127.2	1722	8409	Blue_1	127	0.0	Pass	465.3		
3	132.4	1600	7073	Blue_1	132	0.1	Pass	478.4		
4	133.6	1426	7011	Blue_1	133	0.2	Pass	354.9	[<Deleted>]	

Sample 45: Run date and time: 09/15/2020 - 19:38:20 -> 09/15/2020 - 20:24:32

Dye: Blue - 4 peaks - 5.fsa

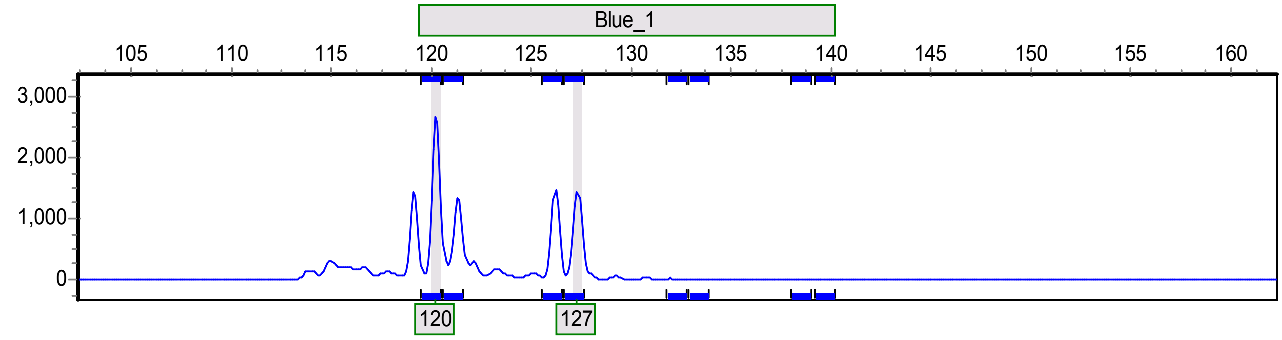

No	Size	Height	Area	Marker	Allele	Difference	Quality	Score	Allele Comments	Sample Comments
1	120.2	2642	12575	Blue_1	120	0.2	Pass	500.0		
2	121.3	1325	7037	Blue_1	121	0.2	Pass	278.8	[<Deleted>]	
3	126.2	1454	6322	Blue_1	126	0.1	Pass	425.1	[<Deleted>]	
4	127.3	1432	6836	Blue_1	127	0.1	Pass	370.6		

Allele Report

Sample 46: Run date and time: 09/15/2020 - 19:38:20 -> 09/15/2020 - 20:24:32

Dye: Blue - 2 peaks - 50.fsa

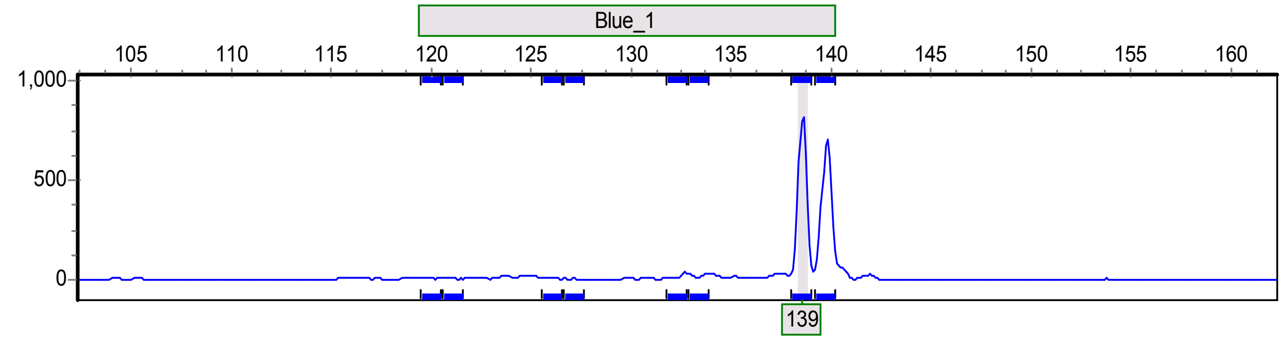

No	Size	Height	Area	Marker	Allele	Difference	Quality	Score	Allele Comments	Sample Comments
1	138.6	815	3737	Blue_1	139	0.1	Pass	172.4		
2	139.8	705	3936	Blue_1	140	0.1	Pass	104.6	[<Deleted>]	

Sample 47: Run date and time: 09/15/2020 - 19:38:20 -> 09/15/2020 - 20:24:32

Dye: Blue - 4 peaks - 51.fsa

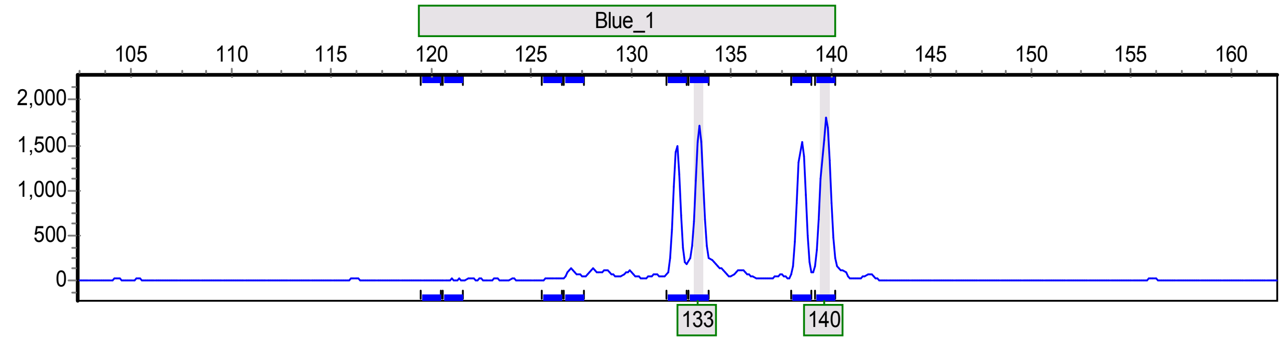

No	Size	Height	Area	Marker	Allele	Difference	Quality	Score	Allele Comments	Sample Comments
1	132.3	1487	6824	Blue_1	132	0.0	Pass	407.3	[<Deleted>]	
2	133.4	1707	8901	Blue_1	133	0.0	Pass	424.9		
3	138.5	1528	6886	Blue_1	139	0.0	Pass	438.1	[<Deleted>]	
4	139.7	1797	9649	Blue_1	140	0.0	Pass	436.6		

Sample 48: Run date and time: 09/15/2020 - 19:38:20 -> 09/15/2020 - 20:24:32

Dye: Blue - 4 peaks - 52.fsa

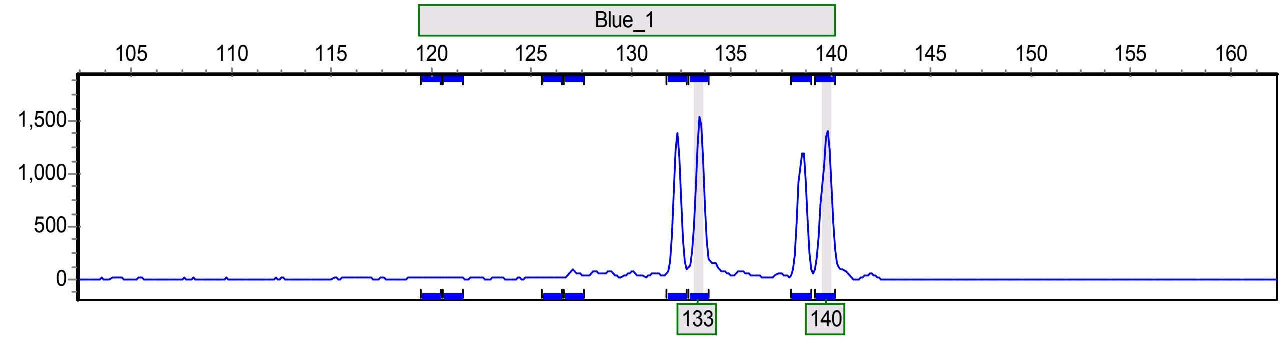

No	Size	Height	Area	Marker	Allele	Difference	Quality	Score	Allele Comments	Sample Comments
1	132.3	1378	6142	Blue_1	132	0.0	Pass	382.2	[<Deleted>]	
2	133.4	1525	7788	Blue_1	133	0.0	Pass	371.0		
3	138.5	1192	5551	Blue_1	139	0.0	Pass	295.4	[<Deleted>]	
4	139.8	1407	7737	Blue_1	140	0.1	Pass	299.2		

Allele Report

Sample 49: Run date and time: 09/15/2020 - 19:38:20 -> 09/15/2020 - 20:24:32

Dye: Blue - 2 peaks - 53.fsa

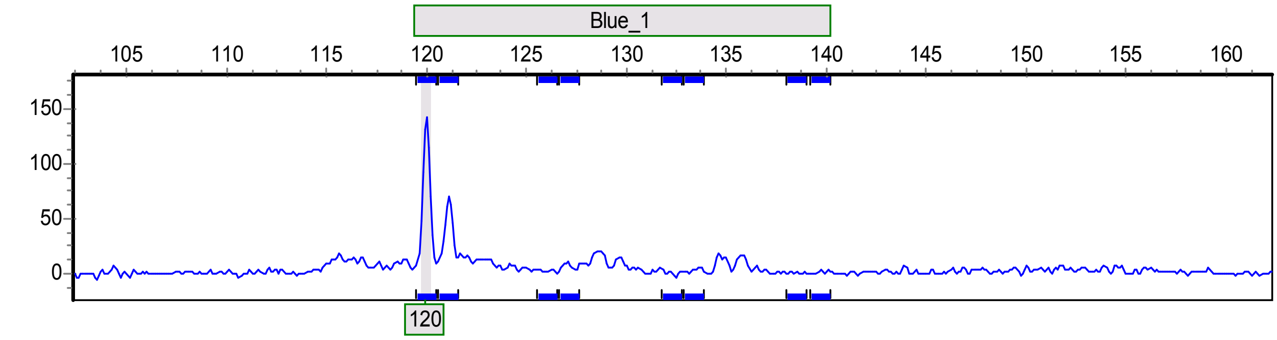

No	Size	Height	Area	Marker	Allele	Difference	Quality	Score	Allele Comments	Sample Comments
1	120.0	142	632	Blue_1	120	0.0	Pass	10.2		
2	121.1	70	382	Blue_1	121	0.0	Check	2.1	[<Deleted>]	

Sample 50: Run date and time: 09/15/2020 - 19:38:20 -> 09/15/2020 - 20:24:32

Dye: Blue - 4 peaks - 54.fsa

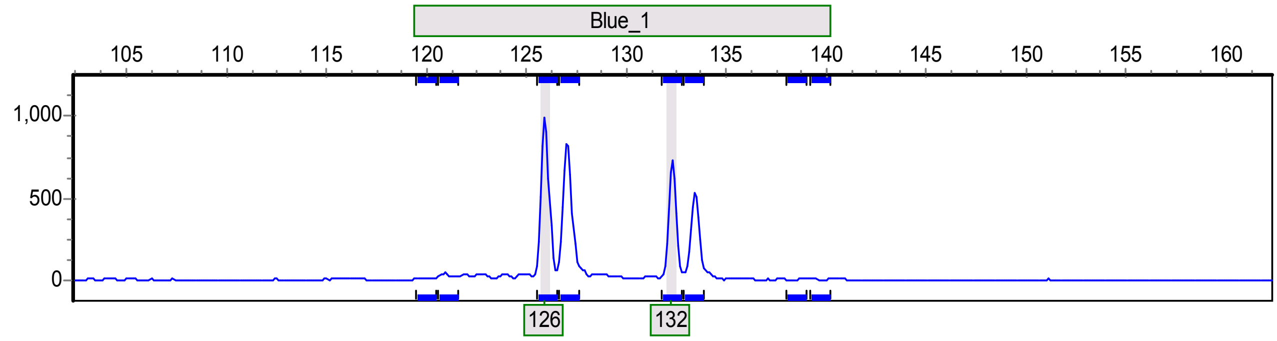

No	Size	Height	Area	Marker	Allele	Difference	Quality	Score	Allele Comments	Sample Comments
1	125.9	983	4349	Blue_1	126	0.2	Pass	239.3		
2	127.0	824	4237	Blue_1	127	0.2	Pass	151.3	[<Deleted>]	
3	132.3	732	3251	Blue_1	132	0.0	Pass	154.9		
4	133.4	533	2719	Blue_1	133	0.0	Pass	78.7	[<Deleted>]	

Sample 51: Run date and time: 09/15/2020 - 19:38:20 -> 09/15/2020 - 20:24:32

Dye: Blue - 2 peaks - 55.fsa

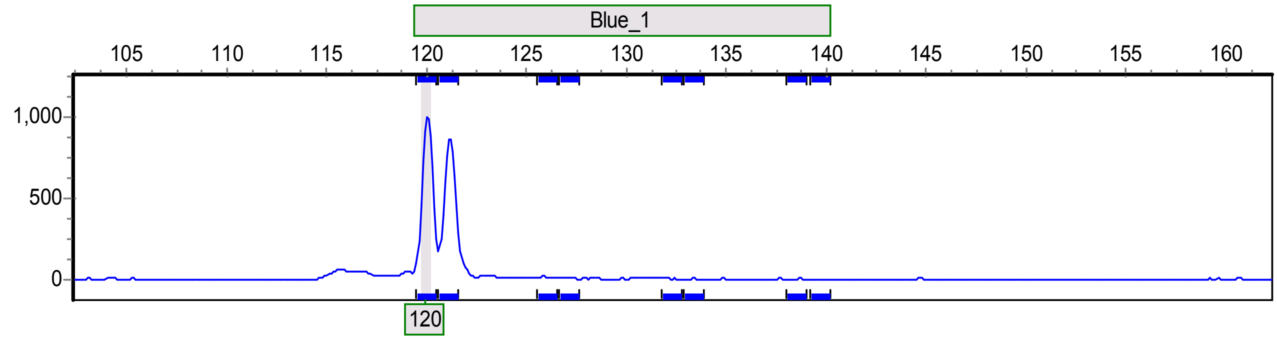

No	Size	Height	Area	Marker	Allele	Difference	Quality	Score	Allele Comments	Sample Comments
1	120.0	993	6546	Blue_1	120	0.0	Pass	131.0		
2	121.2	859	5948	Blue_1	121	0.1	Pass	93.2	[<Deleted>]	

Sample 52: Run date and time: 09/15/2020 - 19:38:20 -> 09/15/2020 - 20:24:32

Dye: Blue - 3 peaks - 56.fsa

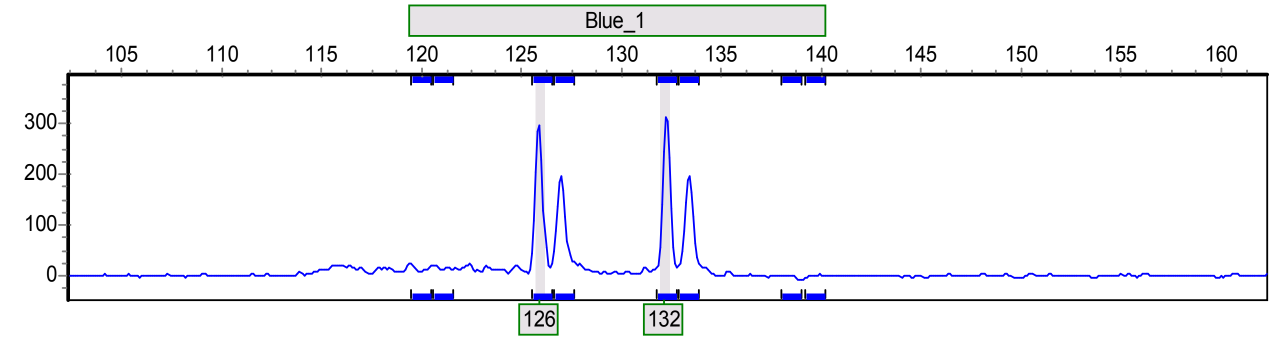

No	Size	Height	Area	Marker	Allele	Difference	Quality	Score	Allele Comments	Sample Comments
1	125.9	295	1291	Blue_1	126	0.2	Pass	37.5		
2	132.2	312	1419	Blue_1	132	0.1	Pass	39.3		
3	133.4	198	1025	Blue_1	133	0.0	Pass	14.9	[<Deleted>]	

Sample 53: Run date and time: 09/15/2020 - 19:38:20 -> 09/15/2020 - 20:24:32

Dye: Blue - 2 peaks - 57.fsa

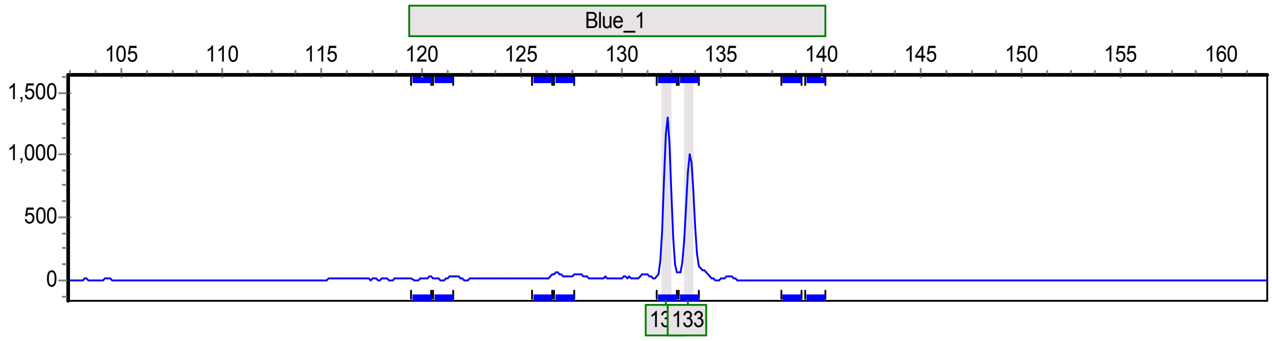

No	Size	Height	Area	Marker	Allele	Difference	Quality	Score	Allele Comments	Sample Comments
1	132.3	1291	5744	Blue_1	132	0.0	Pass	352.7		
2	133.4	1007	5001	Blue_1	133	0.0	Pass	213.2		

Sample 54: Run date and time: 09/15/2020 - 19:38:20 -> 09/15/2020 - 20:24:32

Dye: Blue - 4 peaks - 58.fsa

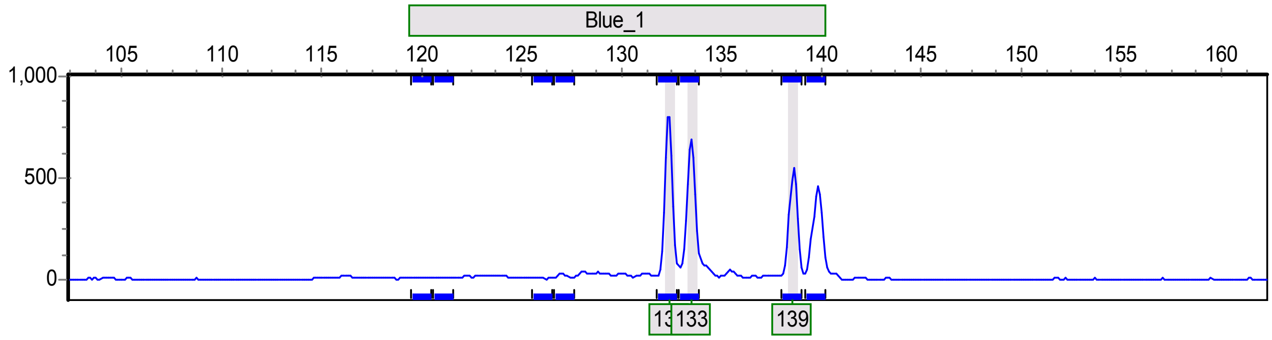

No	Size	Height	Area	Marker	Allele	Difference	Quality	Score	Allele Comments	Sample Comments
1	132.4	798	3644	Blue_1	132	0.1	Pass	167.7		
2	133.5	691	3518	Blue_1	133	0.1	Pass	116.5		
3	138.6	551	2410	Blue_1	139	0.1	Pass	102.4		
4	139.8	459	2522	Blue_1	140	0.1	Pass	55.3	[<Deleted>]	

Allele Report

Sample 55: Run date and time: 09/15/2020 - 19:38:20 -> 09/15/2020 - 20:24:32

Dye: Blue - 2 peaks - 59.fsa

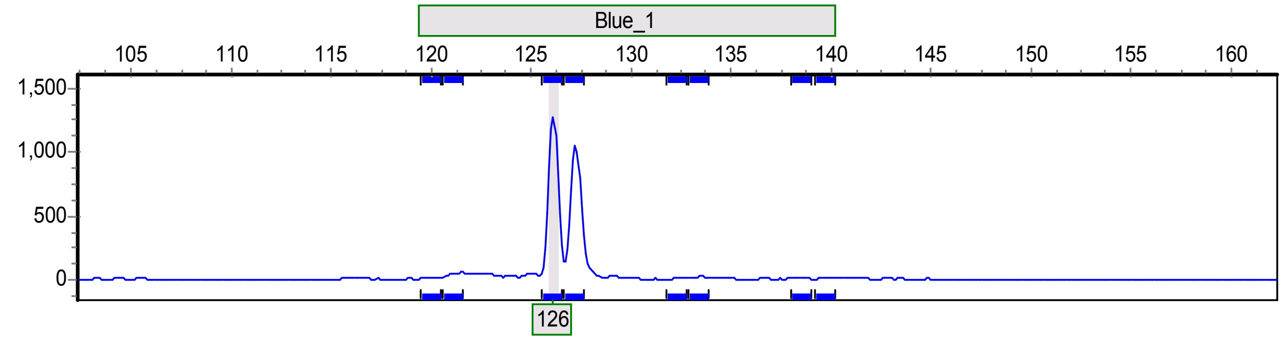

No	Size	Height	Area	Marker	Allele	Difference	Quality	Score	Allele Comments	Sample Comments
1	126.1	1269	6626	Blue_1	126	0.0	Pass	276.0		
2	127.2	1056	6127	Blue_1	127	0.0	Pass	182.5	[<Deleted>]	

Sample 56: Run date and time: 09/15/2020 - 19:38:20 -> 09/15/2020 - 20:24:32

Dye: Blue - 3 peaks - 6.fsa

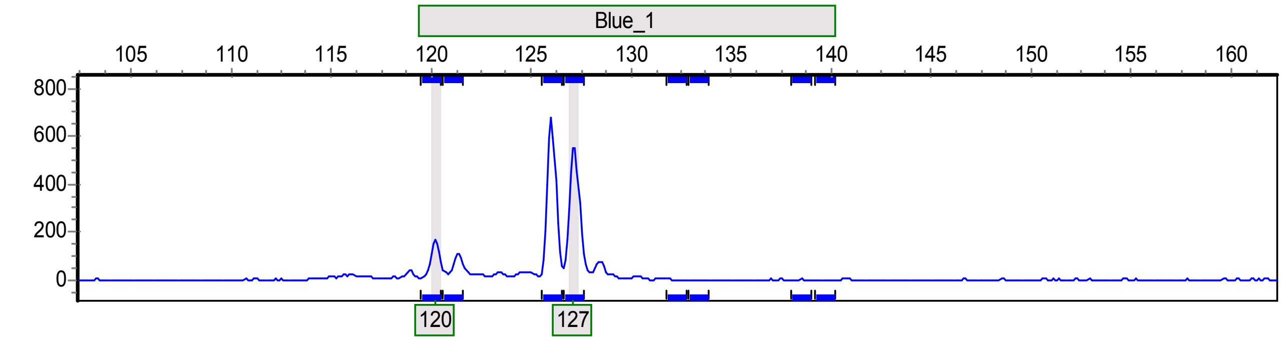

No	Size	Height	Area	Marker	Allele	Difference	Quality	Score	Allele Comments	Sample Comments
1	120.2	169	925	Blue_1	120	0.2	Pass	10.3		
2	126.0	675	3145	Blue_1	126	0.1	Pass	128.5	[<Deleted>]	
3	127.1	549	3057	Blue_1	127	0.1	Pass	72.5		

Sample 57: Run date and time: 09/15/2020 - 19:38:20 -> 09/15/2020 - 20:24:32

Dye: Blue - 0 peaks - 60.fsa

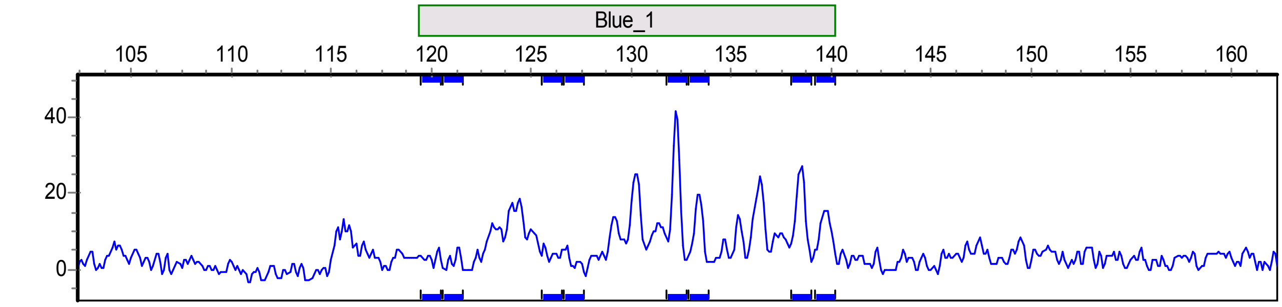

No	Size	Height	Area	Marker	Allele	Difference	Quality	Score	Allele Comments	Sample Comments

Sample 58: Run date and time: 09/15/2020 - 19:38:20 -> 09/15/2020 - 20:24:32

Dye: Blue - 4 peaks - 61.fsa

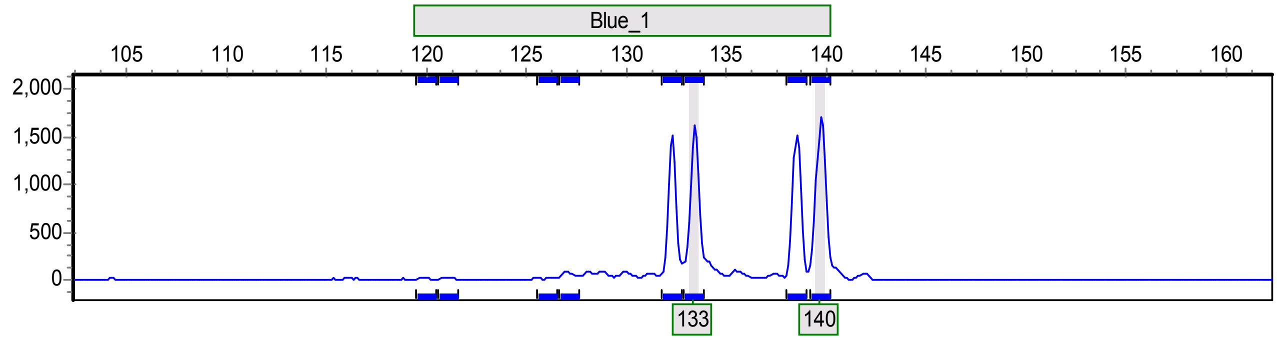

No	Size	Height	Area	Marker	Allele	Difference	Quality	Score	Allele Comments	Sample Comments
1	132.3	1513	6883	Blue_1	132	0.0	Pass	422.8	[<Deleted>]	
2	133.4	1610	8366	Blue_1	133	0.0	Pass	393.5		
3	138.5	1511	6785	Blue_1	139	0.0	Pass	434.0	[<Deleted>]	
4	139.7	1693	9112	Blue_1	140	0.0	Pass	400.2		

Sample 59: Run date and time: 09/15/2020 - 19:38:20 -> 09/15/2020 - 20:24:32

Dye: Blue - 4 peaks - 62.fsa

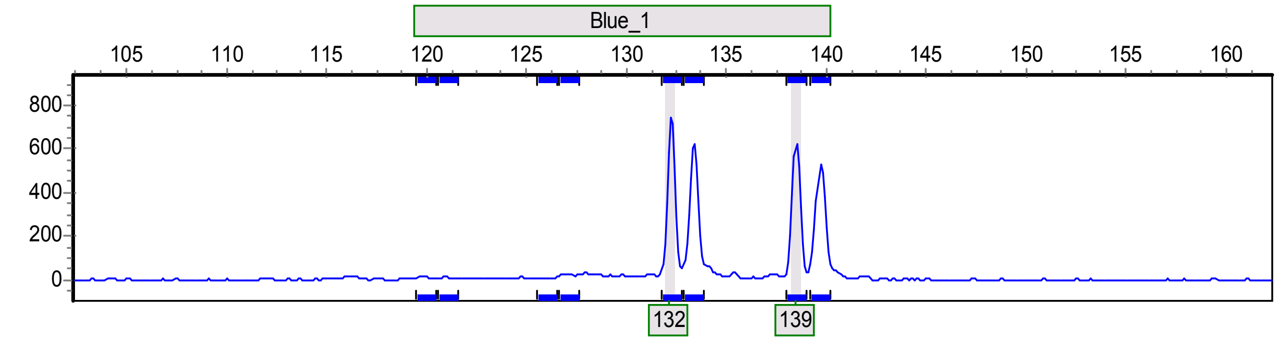

No	Size	Height	Area	Marker	Allele	Difference	Quality	Score	Allele Comments	Sample Comments
1	132.2	740	3377	Blue_1	132	0.1	Pass	151.1		
2	133.4	624	3285	Blue_1	133	0.0	Pass	95.5	[<Deleted>]	
3	138.5	620	2784	Blue_1	139	0.0	Pass	118.7		
4	139.7	531	2959	Blue_1	140	0.0	Pass	68.0	[<Deleted>]	

Sample 60: Run date and time: 09/15/2020 - 19:38:20 -> 09/15/2020 - 20:24:32

Dye: Blue - 4 peaks - 63.fsa

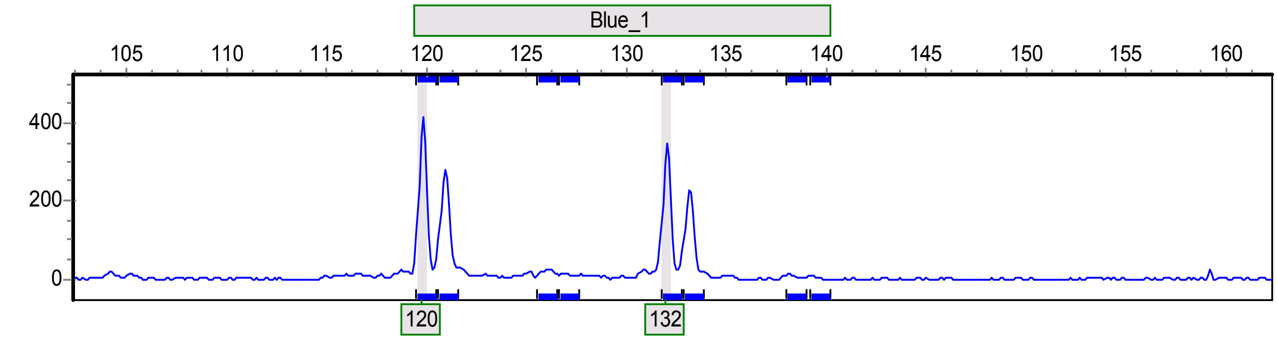

No	Size	Height	Area	Marker	Allele	Difference	Quality	Score	Allele Comments	Sample Comments
1	119.8	411	1790	Blue_1	120	0.2	Pass	65.5		
2	120.9	280	1429	Blue_1	121	0.2	Pass	27.3	[<Deleted>]	
3	132.0	345	1536	Blue_1	132	0.3	Pass	47.5		
4	133.1	227	1175	Blue_1	133	0.3	Pass	18.8	[<Deleted>]	

Sample 61: Run date and time: 09/15/2020 - 19:38:20 -> 09/15/2020 - 20:24:32

Dye: Blue - 4 peaks - 64.fsa

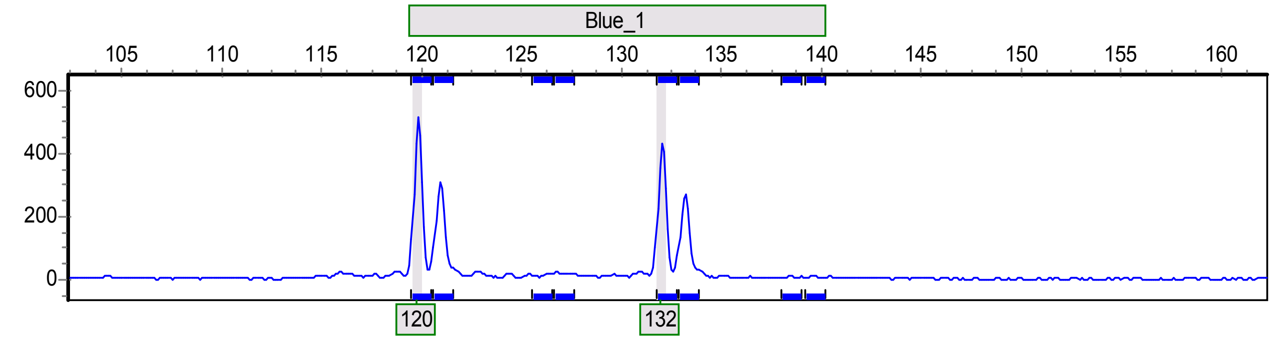

No	Size	Height	Area	Marker	Allele	Difference	Quality	Score	Allele Comments	Sample Comments
1	119.8	511	2245	Blue_1	120	0.2	Pass	91.3		
2	120.9	309	1584	Blue_1	121	0.2	Pass	32.4	[<Deleted>]	
3	132.0	431	1954	Blue_1	132	0.3	Pass	66.5		
4	133.2	271	1415	Blue_1	133	0.2	Pass	25.3	[<Deleted>]	

Sample 62: Run date and time: 09/15/2020 - 19:38:20 -> 09/15/2020 - 20:24:32

Dye: Blue - 2 peaks - 65.fsa

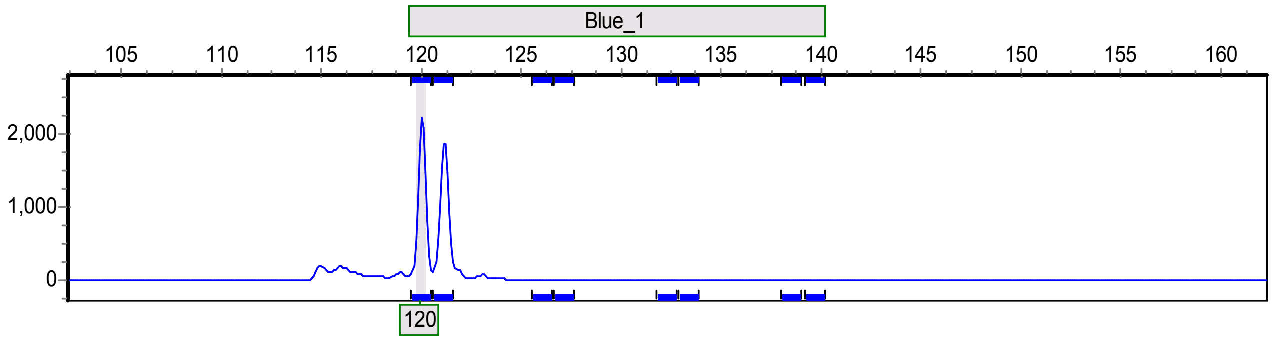

No	Size	Height	Area	Marker	Allele	Difference	Quality	Score	Allele Comments	Sample Comments
1	120.0	2199	9918	Blue_1	120	0.0	Pass	500.0		
2	121.1	1864	9617	Blue_1	121	0.0	Pass	486.8	[<Deleted>]	

Sample 63: Run date and time: 09/15/2020 - 19:38:20 -> 09/15/2020 - 20:24:32

Dye: Blue - 2 peaks - 66.fsa

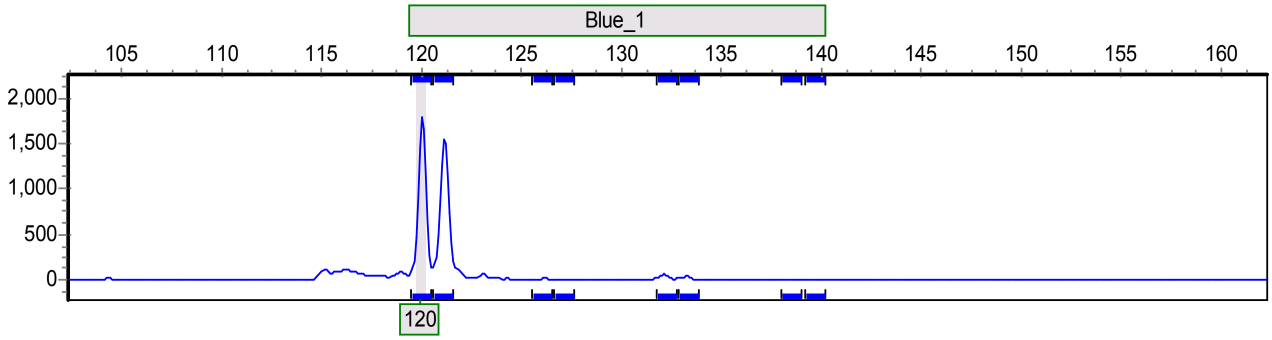

No	Size	Height	Area	Marker	Allele	Difference	Quality	Score	Allele Comments	Sample Comments
1	120.0	1782	8138	Blue_1	120	0.0	Pass	500.0		
2	121.1	1554	8041	Blue_1	121	0.0	Pass	376.0	[<Deleted>]	

Sample 64: Run date and time: 09/15/2020 - 19:38:20 -> 09/15/2020 - 20:24:32

Dye: Blue - 3 peaks - 67.fsa

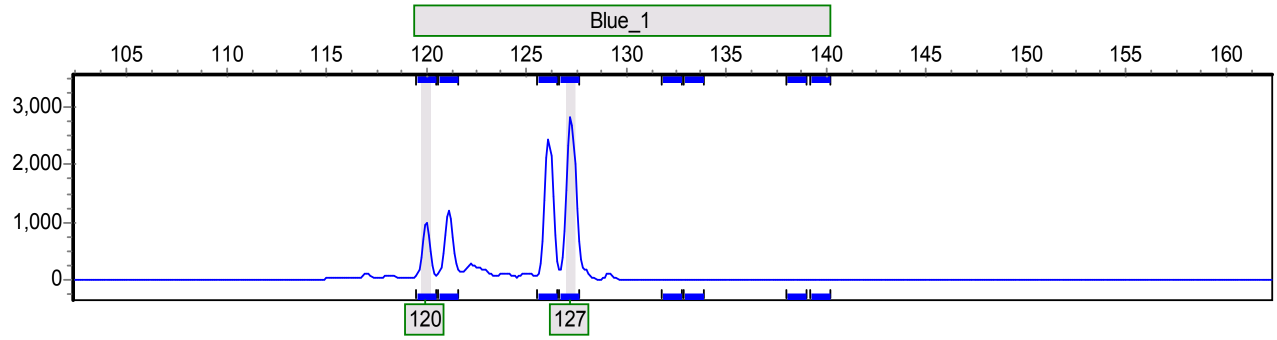

No	Size	Height	Area	Marker	Allele	Difference	Quality	Score	Allele Comments	Sample Comments
1	120.0	991	4532	Blue_1	120	0.0	Pass	230.1		
2	126.1	2438	10935	Blue_1	126	0.0	Pass	500.0	[<Deleted>]	
3	127.2	2798	13959	Blue_1	127	0.0	Pass	500.0		

Sample 65: Run date and time: 09/15/2020 - 19:38:20 -> 09/15/2020 - 20:24:32

Dye: Blue - 3 peaks - 68.fsa

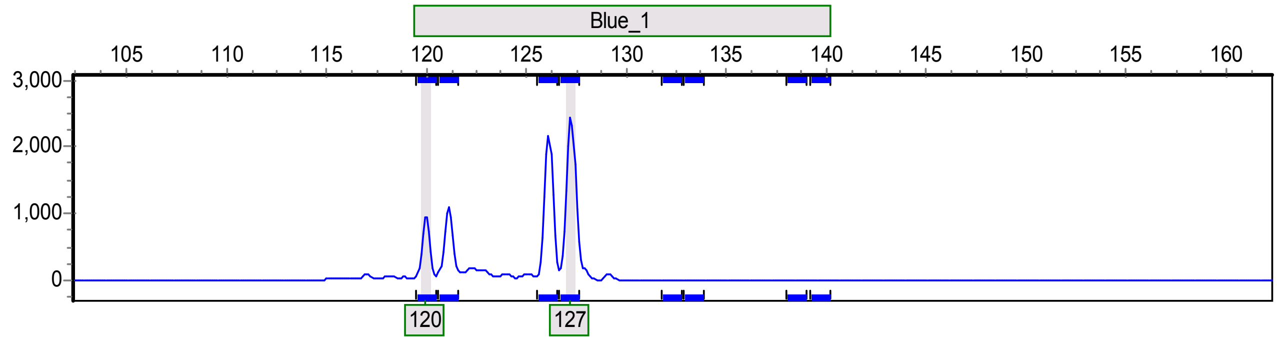

No	Size	Height	Area	Marker	Allele	Difference	Quality	Score	Allele Comments	Sample Comments
1	120.0	944	4308	Blue_1	120	0.0	Pass	214.2		
2	126.1	2167	9793	Blue_1	126	0.0	Pass	500.0	[<Deleted>]	
3	127.2	2428	12126	Blue_1	127	0.0	Pass	500.0		

Sample 66: Run date and time: 09/15/2020 - 19:38:20 -> 09/15/2020 - 20:24:32

Dye: Blue - 2 peaks - 69.fsa

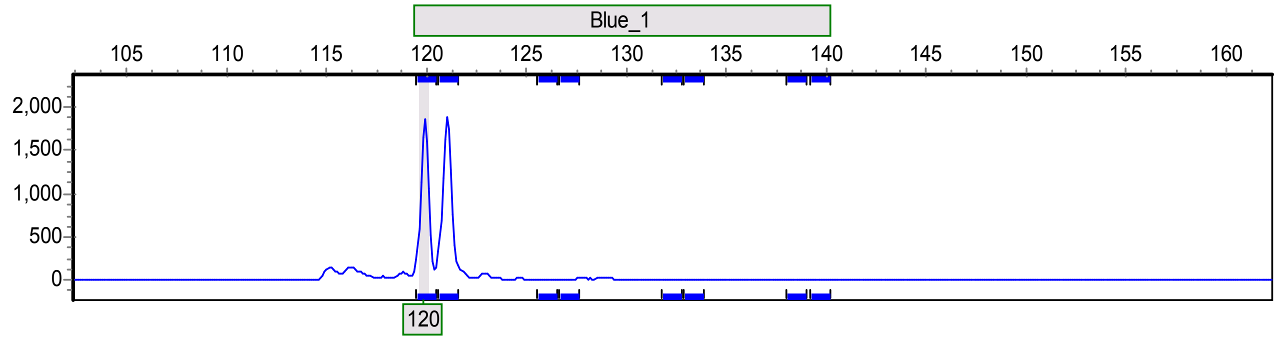

No	Size	Height	Area	Marker	Allele	Difference	Quality	Score	Allele Comments	Sample Comments
1	119.9	1867	8402	Blue_1	120	0.1	Pass	500.0		
2	121.0	1877	9540	Blue_1	121	0.1	Pass	497.3	[<Deleted>]	

Allele Report

Sample 67: Run date and time: 09/15/2020 - 19:38:20 -> 09/15/2020 - 20:24:32

Dye: Blue - 2 peaks - 7.fsa

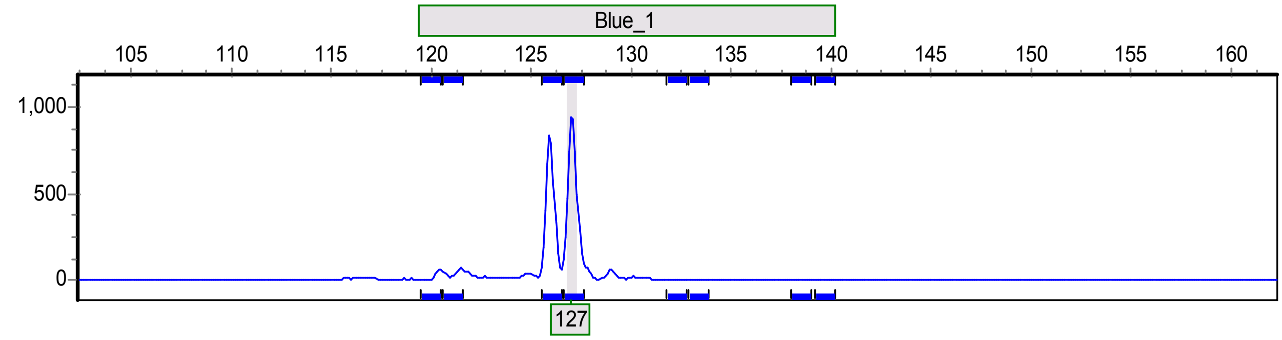

No	Size	Height	Area	Marker	Allele	Difference	Quality	Score	Allele Comments	Sample Comments
1	125.9	835	3794	Blue_1	126	0.2	Pass	181.4	[<Deleted>]	
2	127.0	942	4891	Blue_1	127	0.2	Pass	182.9		

Sample 68: Run date and time: 09/15/2020 - 19:38:20 -> 09/15/2020 - 20:24:32

Dye: Blue - 2 peaks - 70.fsa

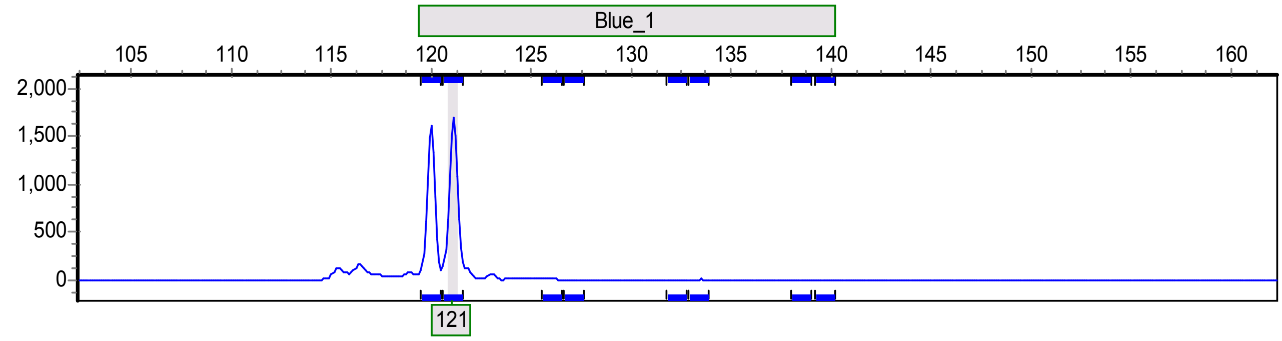

No	Size	Height	Area	Marker	Allele	Difference	Quality	Score	Allele Comments	Sample Comments
1	120.0	1604	7467	Blue_1	120	0.0	Pass	449.8	[<Deleted>]	
2	121.1	1689	8477	Blue_1	121	0.0	Pass	436.6		

Sample 69: Run date and time: 09/15/2020 - 19:38:20 -> 09/15/2020 - 20:24:32

Dye: Blue - 2 peaks - 71.fsa

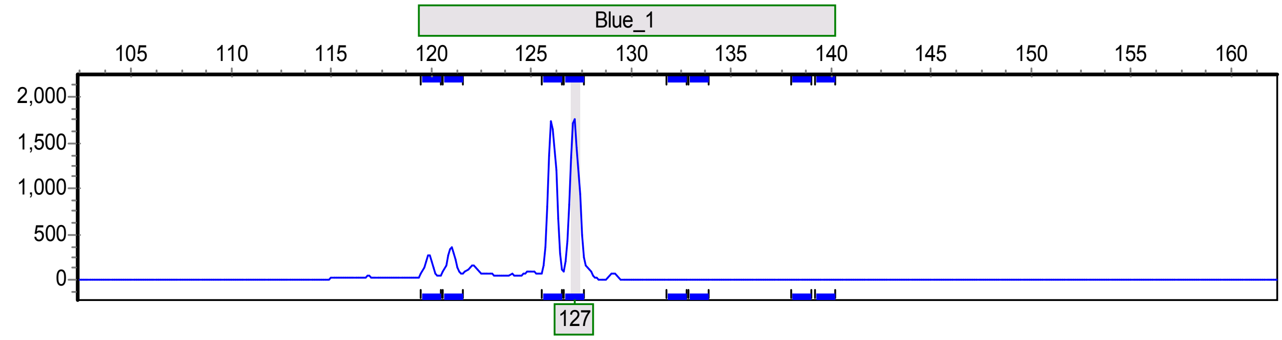

No	Size	Height	Area	Marker	Allele	Difference	Quality	Score	Allele Comments	Sample Comments
1	126.0	1735	7735	Blue_1	126	0.1	Pass	500.0	[<Deleted>]	
2	127.2	1774	8878	Blue_1	127	0.0	Pass	470.7		

Sample 70: Run date and time: 09/15/2020 - 19:38:20 -> 09/15/2020 - 20:24:32

Dye: Blue - 2 peaks - 72.fsa

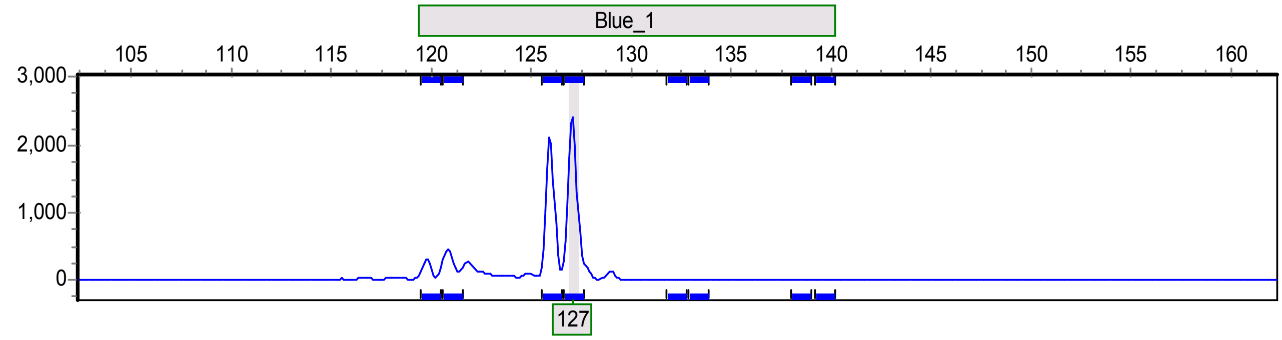

No	Size	Height	Area	Marker	Allele	Difference	Quality	Score	Allele Comments	Sample Comments
1	125.9	2096	9536	Blue_1	126	0.2	Pass	500.0	[<Deleted>]	
2	127.1	2394	12175	Blue_1	127	0.1	Pass	500.0		

Sample 71: Run date and time: 09/15/2020 - 19:38:20 -> 09/15/2020 - 20:24:32

Dye: Blue - 4 peaks - 73.fsa

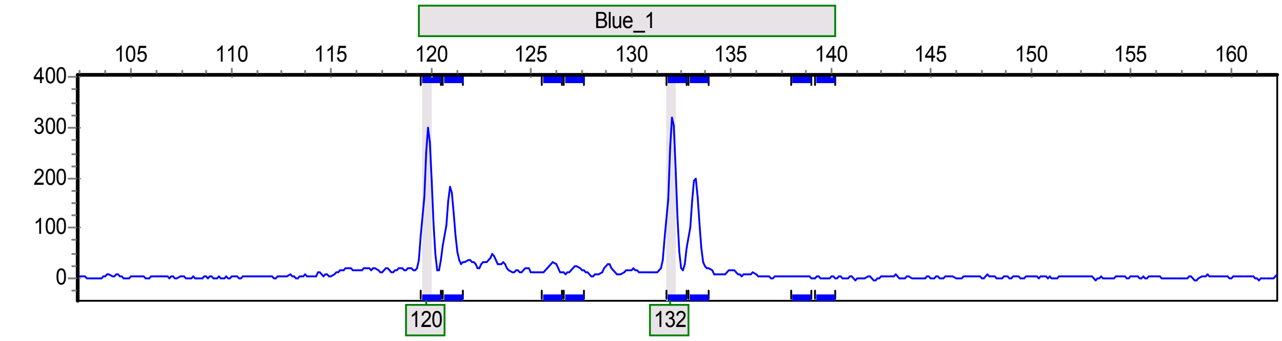

No	Size	Height	Area	Marker	Allele	Difference	Quality	Score	Allele Comments	Sample Comments
1	119.8	299	1372	Blue_1	120	0.2	Pass	36.0		
2	120.9	181	972	Blue_1	121	0.2	Pass	12.2	[<Deleted>]	
3	132.0	319	1461	Blue_1	132	0.3	Pass	40.4		
4	133.2	197	1043	Blue_1	133	0.2	Pass	14.2	[<Deleted>]	

Sample 72: Run date and time: 09/15/2020 - 19:38:20 -> 09/15/2020 - 20:24:32

Dye: Blue - 4 peaks - 74.fsa

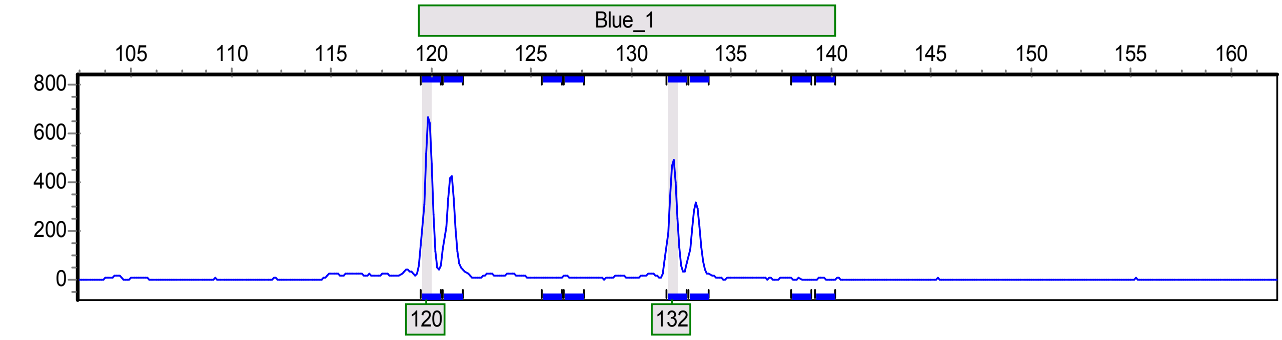

No	Size	Height	Area	Marker	Allele	Difference	Quality	Score	Allele Comments	Sample Comments
1	119.8	663	3007	Blue_1	120	0.2	Pass	128.9		
2	121.0	422	2199	Blue_1	121	0.1	Pass	52.3	[<Deleted>]	
3	132.1	491	2285	Blue_1	132	0.2	Pass	78.5		
4	133.2	316	1668	Blue_1	133	0.2	Pass	32.1	[<Deleted>]	

Allele Report

Sample 73: Run date and time: 09/15/2020 - 19:38:20 -> 09/15/2020 - 20:24:32

Dye: Blue - 3 peaks - 75.fsa

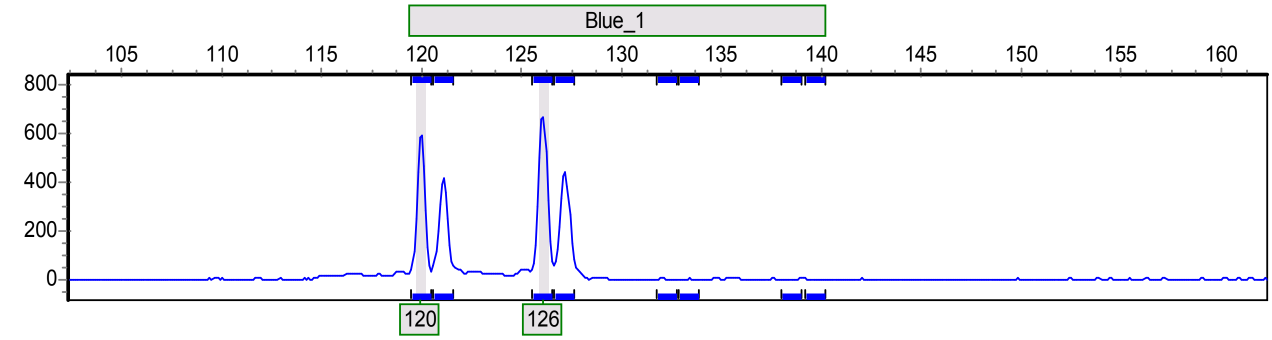

No	Size	Height	Area	Marker	Allele	Difference	Quality	Score	Allele Comments	Sample Comments
1	120.0	589	2750	Blue_1	120	0.0	Pass	103.4		
2	126.1	660	3165	Blue_1	126	0.0	Pass	118.1		
3	127.2	441	2371	Blue_1	127	0.0	Pass	53.7	[<Deleted>]	

Sample 74: Run date and time: 09/15/2020 - 19:38:20 -> 09/15/2020 - 20:24:32

Dye: Blue - 3 peaks - 76.fsa

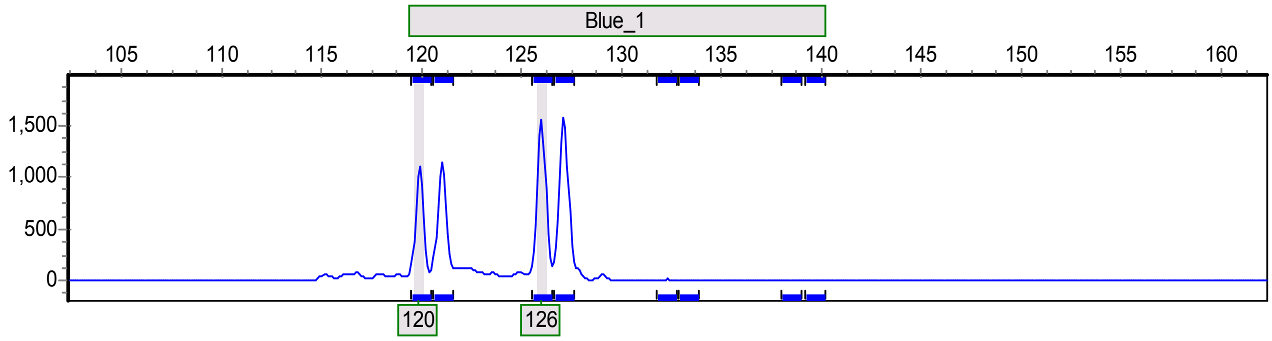

No	Size	Height	Area	Marker	Allele	Difference	Quality	Score	Allele Comments	Sample Comments
1	119.9	1093	4935	Blue_1	120	0.1	Pass	270.7		
2	126.0	1558	7221	Blue_1	126	0.1	Pass	431.0		
3	127.1	1565	8147	Blue_1	127	0.1	Pass	382.0	[<Deleted>]	

Sample 75: Run date and time: 09/15/2020 - 19:38:20 -> 09/15/2020 - 20:24:32

Dye: Blue - 2 peaks - 77.fsa

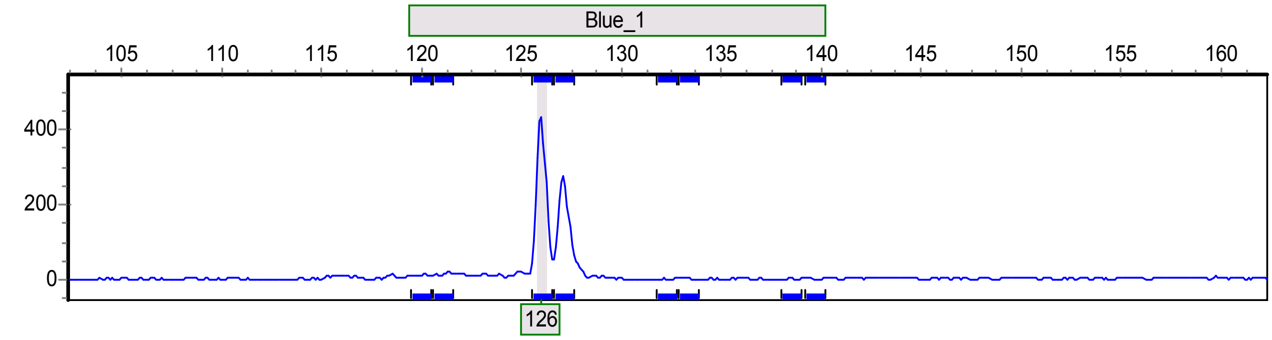

No	Size	Height	Area	Marker	Allele	Difference	Quality	Score	Allele Comments	Sample Comments
1	126.0	429	2257	Blue_1	126	0.1	Pass	52.5		
2	127.1	272	1658	Blue_1	127	0.1	Pass	18.4	[<Deleted>]	

Allele Report

Sample 76: Run date and time: 09/15/2020 - 19:38:20 -> 09/15/2020 - 20:24:32

Dye: Blue - 0 peaks - 78.fsa

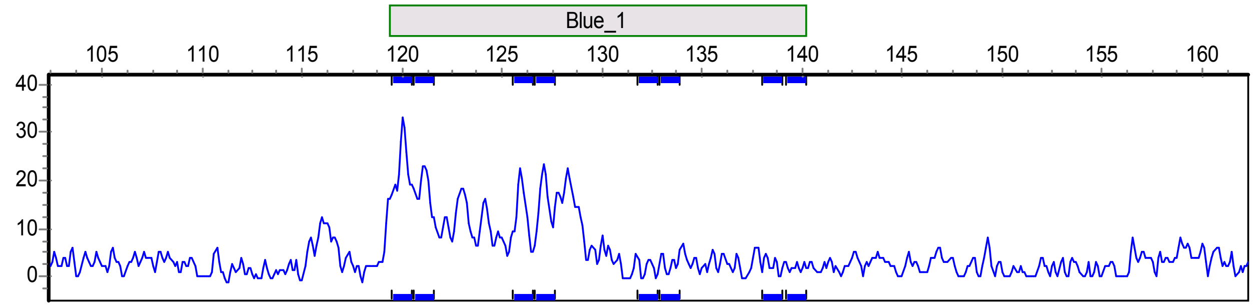

No	Size	Height	Area	Marker	Allele	Difference	Quality	Score	Allele Comments	Sample Comments

Sample 77: Run date and time: 09/15/2020 - 19:38:20 -> 09/15/2020 - 20:24:32

Dye: Blue - 4 peaks - 79.fsa

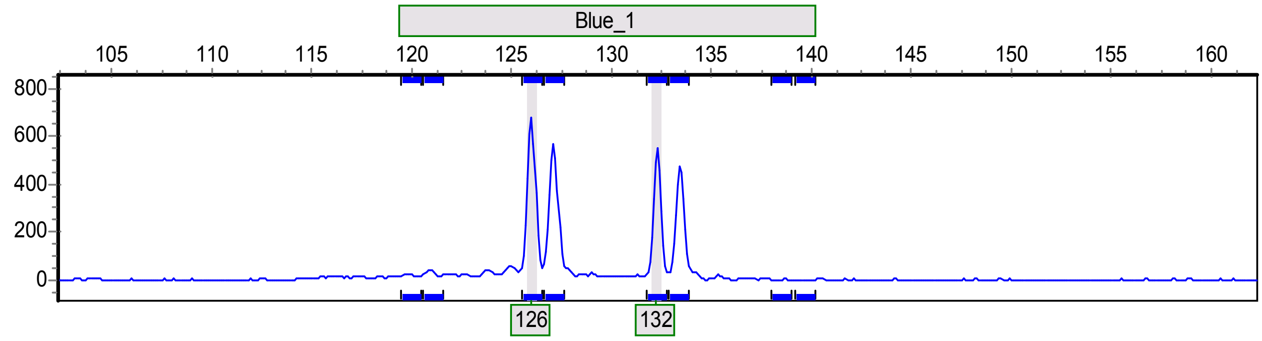

No	Size	Height	Area	Marker	Allele	Difference	Quality	Score	Allele Comments	Sample Comments
1	126.0	676	3060	Blue_1	126	0.1	Pass	133.3		
2	127.1	565	2907	Blue_1	127	0.1	Pass	86.0	[<Deleted>]	
3	132.3	549	2436	Blue_1	132	0.0	Pass	99.8		
4	133.4	472	2370	Blue_1	133	0.0	Pass	66.3	[<Deleted>]	

Sample 78: Run date and time: 09/15/2020 - 19:38:20 -> 09/15/2020 - 20:24:32

Dye: Blue - 0 peaks - 8.fsa

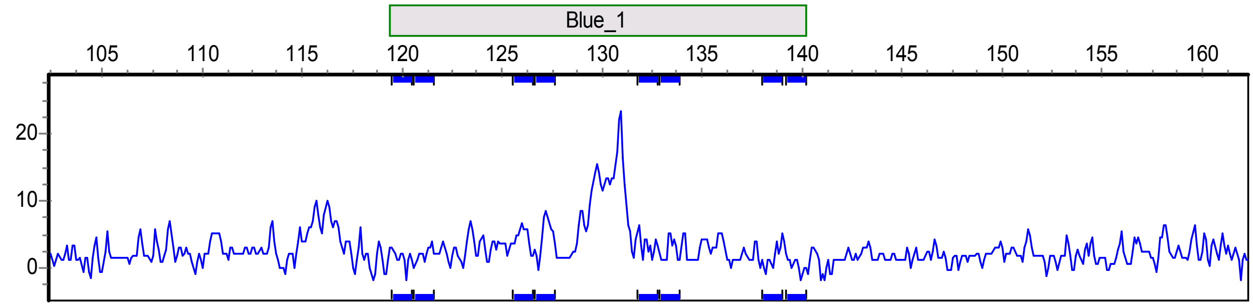

No	Size	Height	Area	Marker	Allele	Difference	Quality	Score	Allele Comments	Sample Comments

Sample 79: Run date and time: 09/15/2020 - 19:38:20 -> 09/15/2020 - 20:24:32

Dye: Blue - 2 peaks - 80.fsa

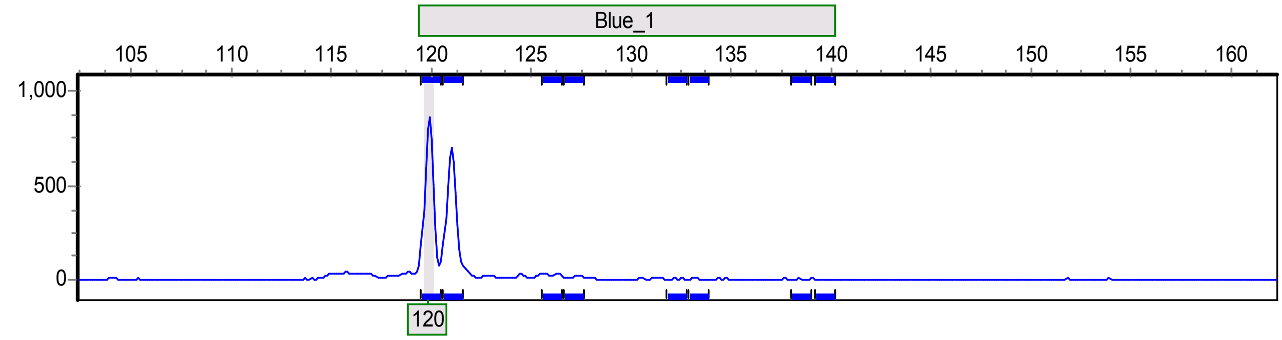

No	Size	Height	Area	Marker	Allele	Difference	Quality	Score	Allele Comments	Sample Comments
1	119.9	856	4213	Blue_1	120	0.1	Pass	168.9		
2	121.0	702	3820	Blue_1	121	0.1	Pass	109.4	[<Deleted>]	

Sample 80: Run date and time: 09/15/2020 - 19:38:20 -> 09/15/2020 - 20:24:32

Dye: Blue - 4 peaks - 81.fsa

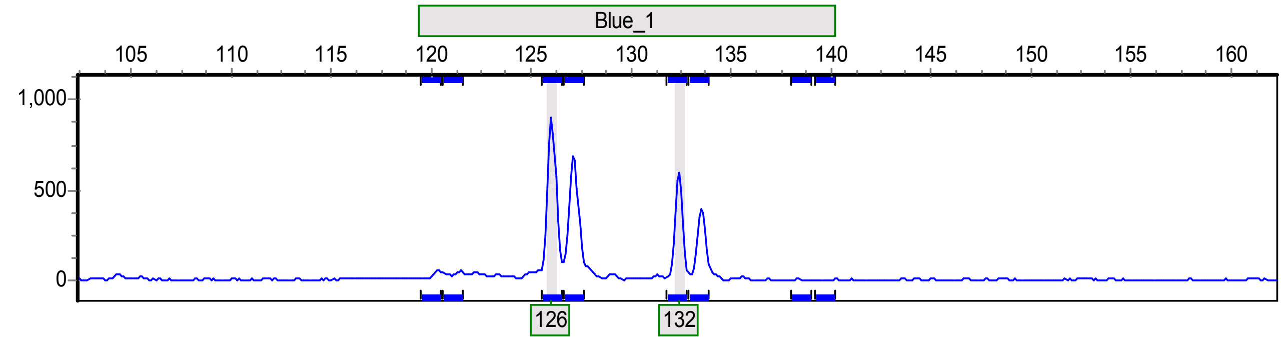

No	Size	Height	Area	Marker	Allele	Difference	Quality	Score	Allele Comments	Sample Comments
1	126.0	897	4161	Blue_1	126	0.1	Pass	195.3		
2	127.1	689	3531	Blue_1	127	0.1	Pass	111.1	[<Deleted>]	
3	132.4	596	2677	Blue_1	132	0.1	Pass	112.1		
4	133.5	398	2042	Blue_1	133	0.1	Pass	48.9	[<Deleted>]	

Sample 81: Run date and time: 09/15/2020 - 19:38:20 -> 09/15/2020 - 20:24:32

Dye: Blue - 4 peaks - 82.fsa

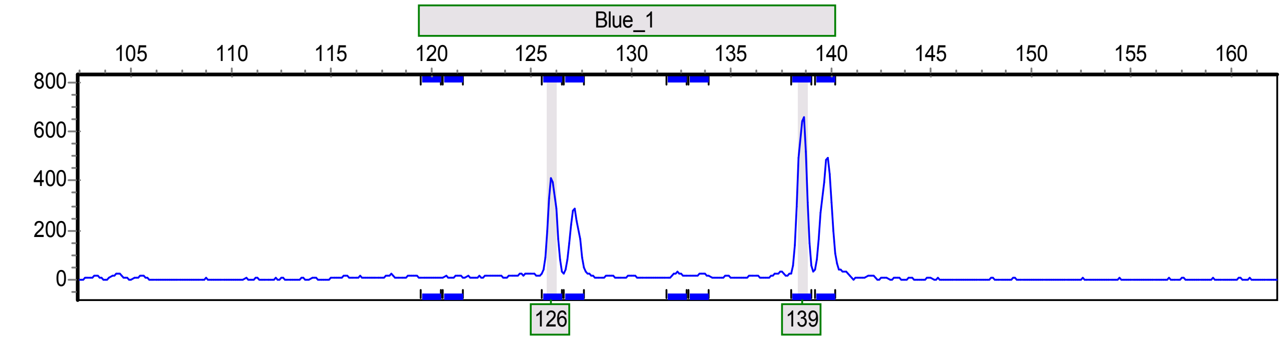

No	Size	Height	Area	Marker	Allele	Difference	Quality	Score	Allele Comments	Sample Comments
1	126.0	412	1876	Blue_1	126	0.1	Pass	60.9		
2	127.2	291	1529	Blue_1	127	0.0	Pass	28.1	[<Deleted>]	
3	138.6	655	3104	Blue_1	139	0.1	Pass	120.0		
4	139.8	497	2845	Blue_1	140	0.1	Pass	58.2	[<Deleted>]	

Sample 82: Run date and time: 09/15/2020 - 19:38:20 -> 09/15/2020 - 20:24:32

Dye: Blue - 4 peaks - 83.fsa

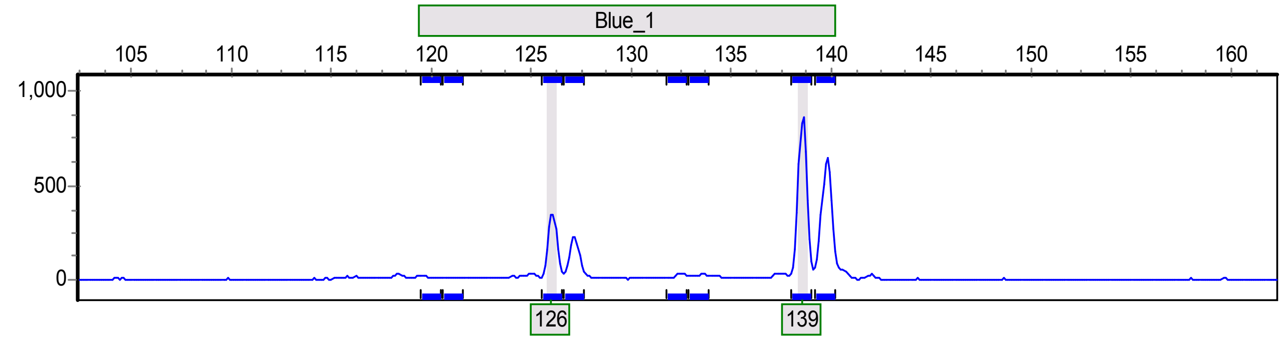

No	Size	Height	Area	Marker	Allele	Difference	Quality	Score	Allele Comments	Sample Comments
1	126.0	348	1669	Blue_1	126	0.1	Pass	43.4		
2	127.2	226	1257	Blue_1	127	0.0	Pass	16.5	[<Deleted>]	
3	138.6	858	4010	Blue_1	139	0.1	Pass	181.6		
4	139.8	647	3756	Blue_1	140	0.1	Pass	86.5	[<Deleted>]	

Sample 83: Run date and time: 09/15/2020 - 19:38:20 -> 09/15/2020 - 20:24:32

Dye: Blue - 3 peaks - 84.fsa

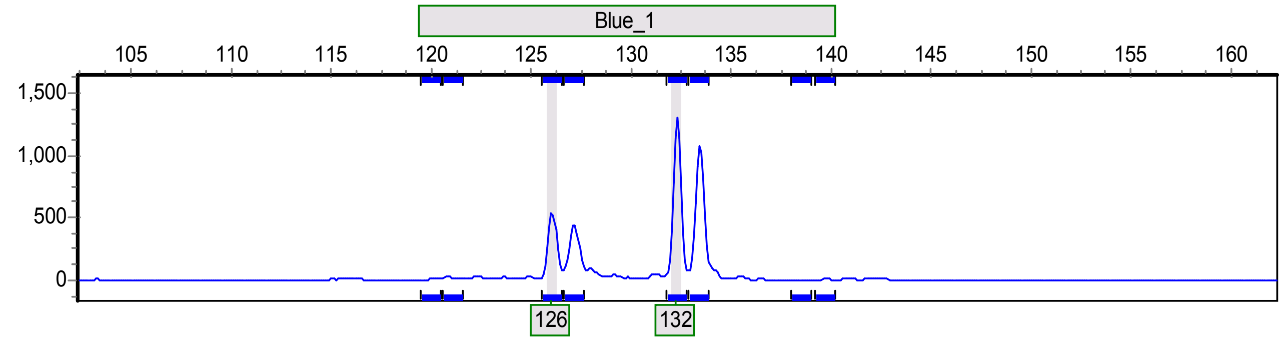

No	Size	Height	Area	Marker	Allele	Difference	Quality	Score	Allele Comments	Sample Comments
1	126.0	545	2604	Blue_1	126	0.1	Pass	90.1		
2	132.3	1303	5954	Blue_1	132	0.0	Pass	343.9		
3	133.4	1078	5660	Blue_1	133	0.0	Pass	217.9	[<Deleted>]	

Sample 84: Run date and time: 09/15/2020 - 19:38:20 -> 09/15/2020 - 20:24:32

Dye: Blue - 2 peaks - 85.fsa

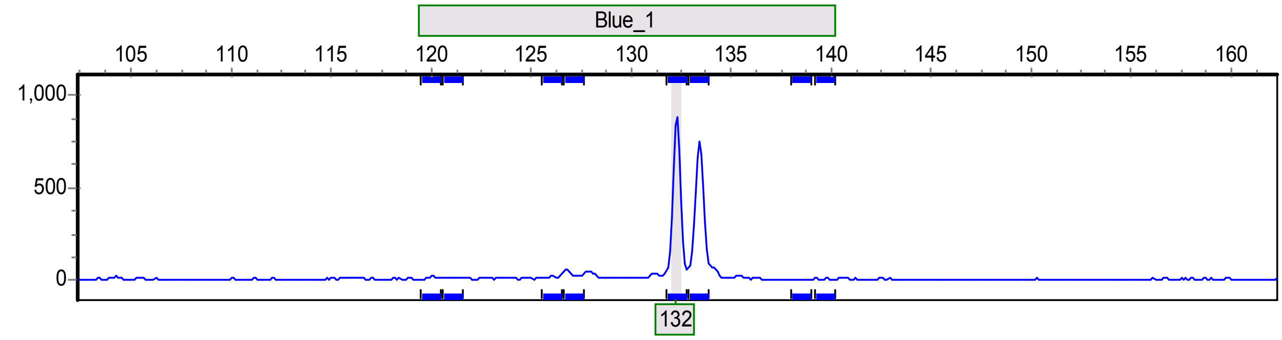

No	Size	Height	Area	Marker	Allele	Difference	Quality	Score	Allele Comments	Sample Comments
1	132.3	877	4014	Blue_1	132	0.0	Pass	192.9		
2	133.4	745	3868	Blue_1	133	0.0	Pass	127.2	[<Deleted>]	

Allele Report

Sample 85: Run date and time: 09/15/2020 - 19:38:20 -> 09/15/2020 - 20:24:32

Dye: Blue - 3 peaks - 86.fsa

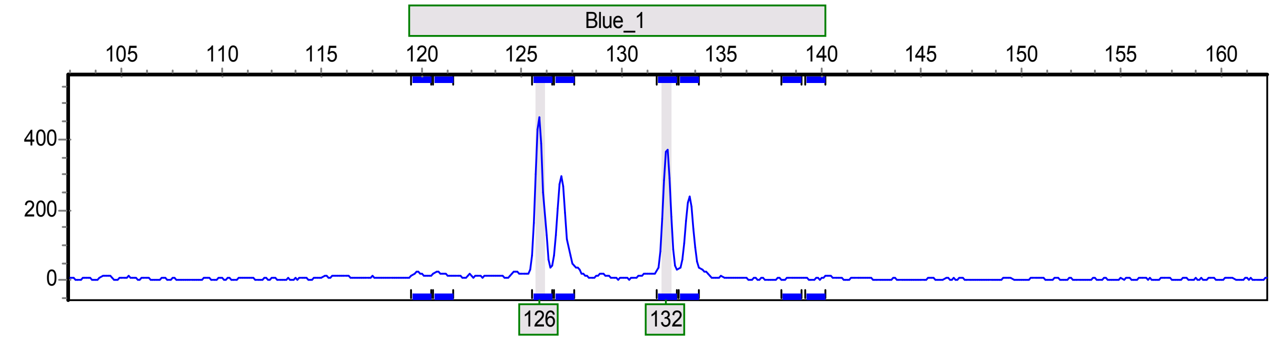

No	Size	Height	Area	Marker	Allele	Difference	Quality	Score	Allele Comments	Sample Comments
1	125.9	461	2119	Blue_1	126	0.2	Pass	72.5		
2	132.3	371	1785	Blue_1	132	0.0	Pass	48.0		
3	133.4	240	1270	Blue_1	133	0.0	Pass	20.2	[<Deleted>]	

Sample 86: Run date and time: 09/15/2020 - 19:38:20 -> 09/15/2020 - 20:24:32

Dye: Blue - 4 peaks - 87.fsa

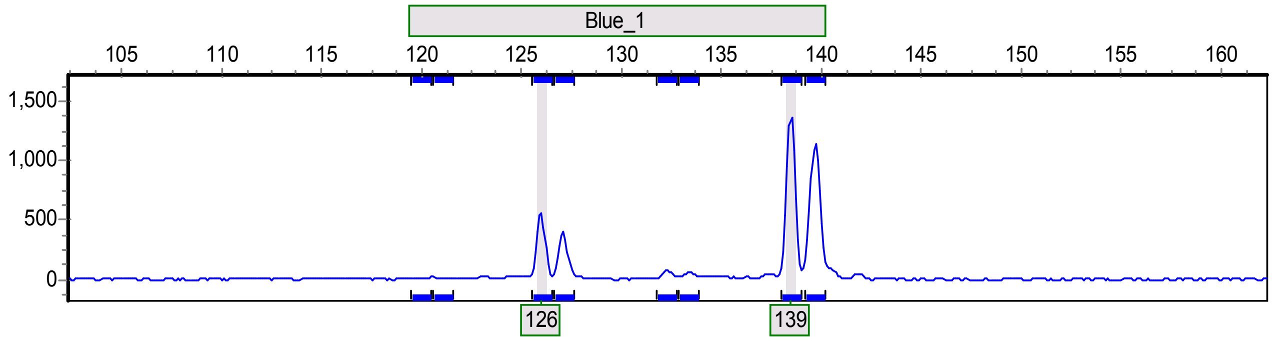

No	Size	Height	Area	Marker	Allele	Difference	Quality	Score	Allele Comments	Sample Comments
1	126.0	558	2554	Blue_1	126	0.1	Pass	97.5		
2	127.1	398	2010	Blue_1	127	0.1	Pass	49.7	[<Deleted>]	
3	138.5	1355	6290	Blue_1	139	0.0	Pass	355.4		
4	139.7	1143	6404	Blue_1	140	0.0	Pass	215.6	[<Deleted>]	

Sample 87: Run date and time: 09/15/2020 - 19:38:20 -> 09/15/2020 - 20:24:32

Dye: Blue - 2 peaks - 88.fsa

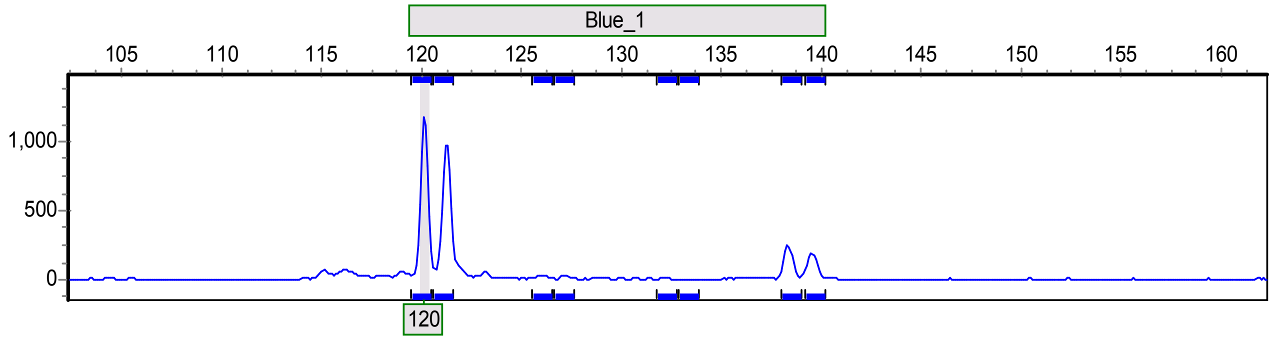

No	Size	Height	Area	Marker	Allele	Difference	Quality	Score	Allele Comments	Sample Comments
1	120.1	1174	5284	Blue_1	120	0.1	Pass	301.3		
2	121.3	980	5126	Blue_1	121	0.2	Pass	190.7	[<Deleted>]	

Sample 88: Run date and time: 09/15/2020 - 19:38:20 -> 09/15/2020 - 20:24:32

Dye: Blue - 4 peaks - 89.fsa

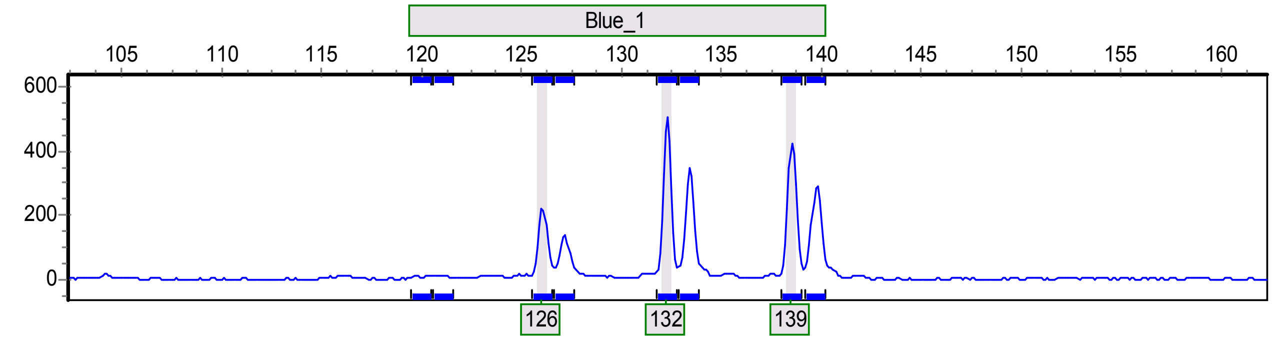

No	Size	Height	Area	Marker	Allele	Difference	Quality	Score	Allele Comments	Sample Comments
1	126.0	220	1142	Blue_1	126	0.1	Pass	19.1		
2	132.3	505	2355	Blue_1	132	0.0	Pass	82.4		
3	138.5	423	2013	Blue_1	139	0.0	Pass	60.4		
4	139.8	288	1699	Blue_1	140	0.1	Pass	22.4	[<Deleted>]	

Sample 89: Run date and time: 09/15/2020 - 19:38:20 -> 09/15/2020 - 20:24:32

Dye: Blue - 4 peaks - 9.fsa

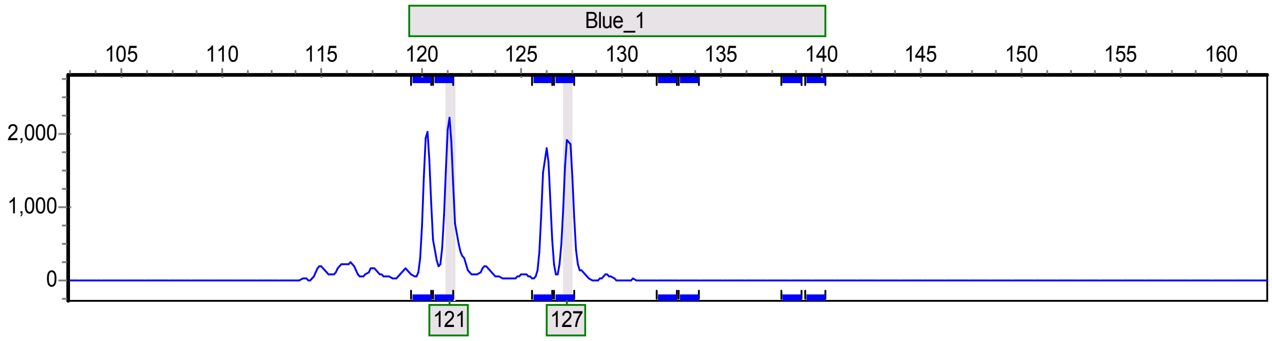

No	Size	Height	Area	Marker	Allele	Difference	Quality	Score	Allele Comments	Sample Comments
1	120.3	2022	9398	Blue_1	120	0.3	Pass	500.0	[<Deleted>]	
2	121.4	2196	10731	Blue_1	121	0.3	Pass	500.0		
3	126.2	1789	7619	Blue_1	126	0.1	Pass	500.0	[<Deleted>]	
4	127.3	1904	9286	Blue_1	127	0.1	Pass	500.0		

Sample 90: Run date and time: 09/15/2020 - 19:38:20 -> 09/15/2020 - 20:24:32

Dye: Blue - 2 peaks - 90.fsa

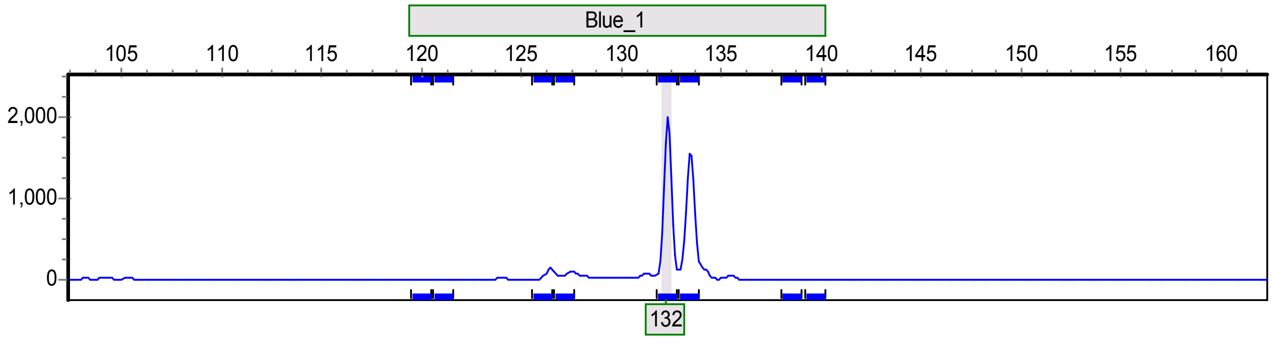

No	Size	Height	Area	Marker	Allele	Difference	Quality	Score	Allele Comments	Sample Comments
1	132.3	1996	9121	Blue_1	132	0.0	Pass	500.0		
2	133.4	1551	8150	Blue_1	133	0.0	Pass	367.8	[<Deleted>]	

Sample 91: Run date and time: 09/15/2020 - 19:38:20 -> 09/15/2020 - 20:24:32

Dye: Blue - 7 peaks - 91.fsa

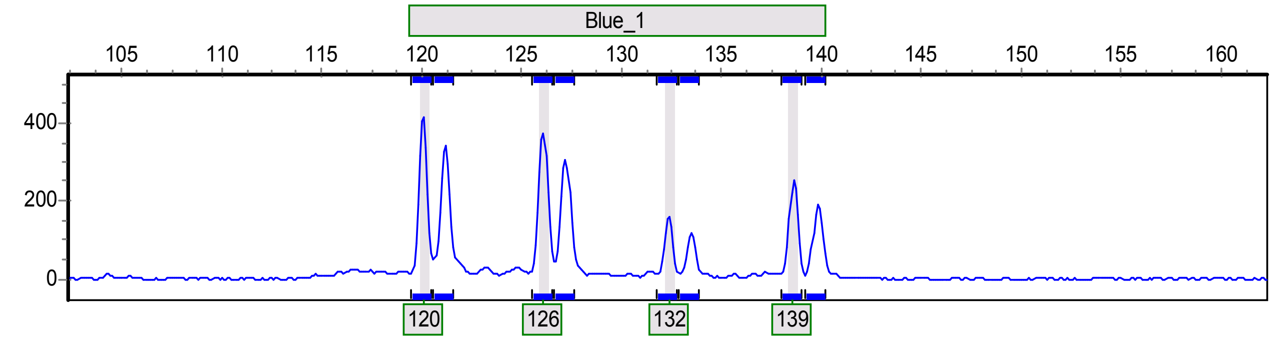

No	Size	Height	Area	Marker	Allele	Difference	Quality	Score	Allele Comments	Sample Comments
1	120.1	415	2081	Blue_1	120	0.1	Pass	53.7		
2	121.2	342	1945	Blue_1	121	0.1	Pass	32.9	[<Deleted>]	
3	126.1	375	1954	Blue_1	126	0.0	Pass	42.7		
4	127.2	308	1747	Blue_1	127	0.0	Pass	27.1	[<Deleted>]	
5	132.4	160	806	Blue_1	132	0.1	Pass	10.6		
6	138.6	254	1217	Blue_1	139	0.1	Pass	26.0		
7	139.8	189	1072	Blue_1	140	0.1	Pass	11.6	[<Deleted>]	

Sample 92: Run date and time: 09/15/2020 - 19:38:20 -> 09/15/2020 - 20:24:32

Dye: Blue - 2 peaks - 92.fsa

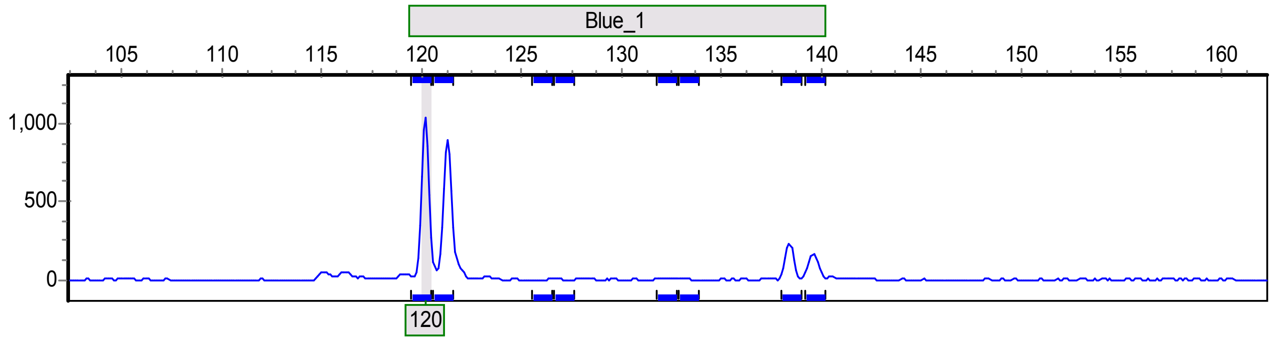

No	Size	Height	Area	Marker	Allele	Difference	Quality	Score	Allele Comments	Sample Comments
1	120.2	1028	4615	Blue_1	120	0.2	Pass	250.6		
2	121.3	896	4484	Blue_1	121	0.2	Pass	177.5	[<Deleted>]	

Sample 93: Run date and time: 09/15/2020 - 19:38:20 -> 09/15/2020 - 20:24:32

Dye: Blue - 2 peaks - 93.fsa

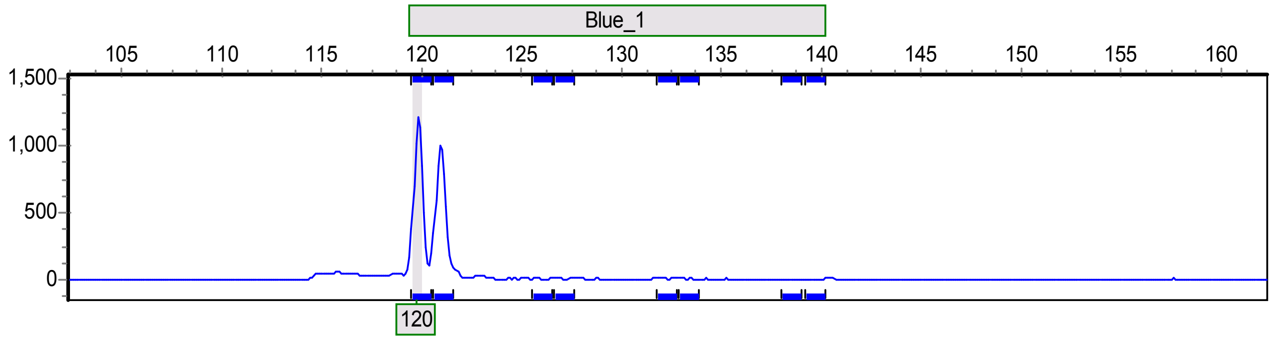

No	Size	Height	Area	Marker	Allele	Difference	Quality	Score	Allele Comments	Sample Comments
1	119.8	1211	5971	Blue_1	120	0.2	Pass	279.6		
2	120.9	1002	5607	Blue_1	121	0.2	Pass	178.7	[<Deleted>]	

Allele Report

Sample 94: Run date and time: 09/15/2020 - 19:38:20 -> 09/15/2020 - 20:24:32

Dye: Blue - 3 peaks - 94.fsa

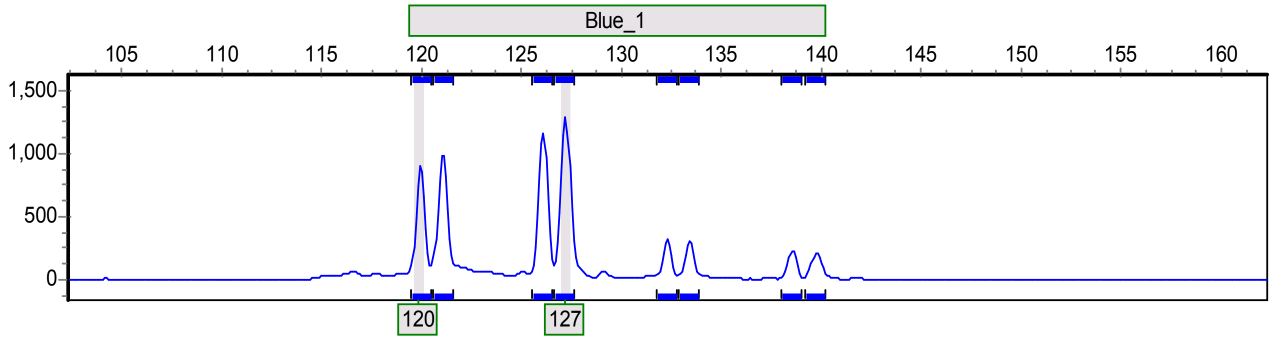

No	Size	Height	Area	Marker	Allele	Difference	Quality	Score	Allele Comments	Sample Comments
1	119.9	903	4482	Blue_1	120	0.1	Pass	181.6		
2	126.1	1160	5766	Blue_1	126	0.0	Pass	261.5	[<Deleted>]	
3	127.2	1288	6964	Blue_1	127	0.0	Pass	270.5		

Sample 95: Run date and time: 09/15/2020 - 19:38:20 -> 09/15/2020 - 20:24:32

Dye: Blue - 7 peaks - 95.fsa

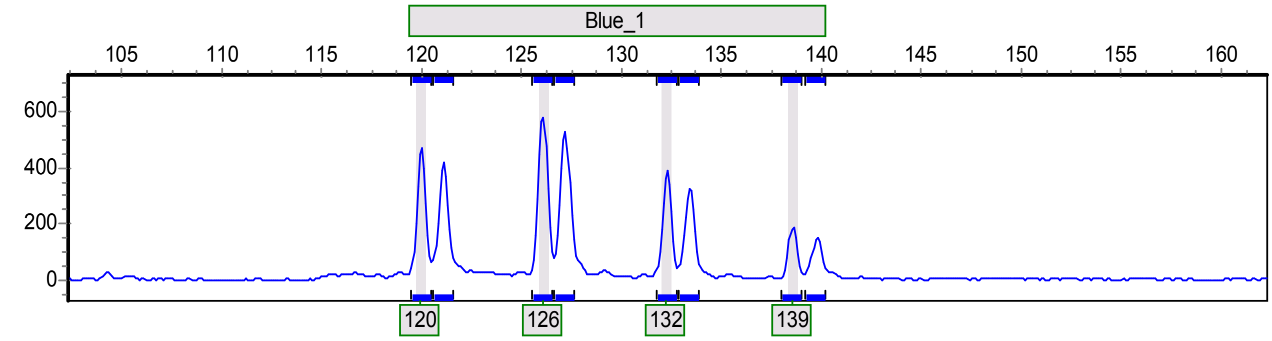

No	Size	Height	Area	Marker	Allele	Difference	Quality	Score	Allele Comments	Sample Comments
1	120.0	472	2371	Blue_1	120	0.0	Pass	65.8		
2	126.1	577	3047	Blue_1	126	0.0	Pass	83.9		
3	127.2	525	3067	Blue_1	127	0.0	Pass	62.5	[<Deleted>]	
4	132.3	388	2031	Blue_1	132	0.0	Pass	45.5		
5	133.4	326	1959	Blue_1	133	0.0	Pass	27.0	[<Deleted>]	
6	138.6	184	885	Blue_1	139	0.1	Pass	14.6		
7	139.8	152	910	Blue_1	140	0.1	Pass	7.5	[<Deleted>]	

Sample 96: Run date and time: 09/15/2020 - 19:38:20 -> 09/15/2020 - 20:24:32

Dye: Blue - 4 peaks - 96.fsa

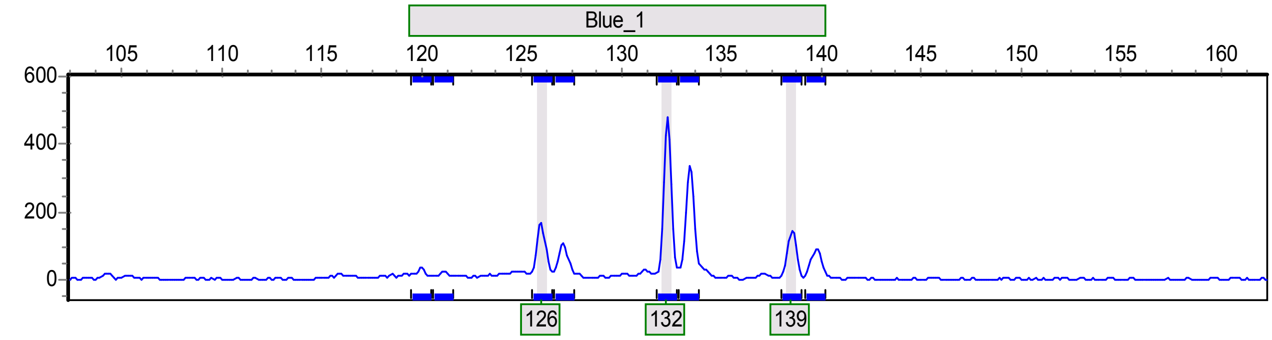

No	Size	Height	Area	Marker	Allele	Difference	Quality	Score	Allele Comments	Sample Comments
1	126.0	168	855	Blue_1	126	0.1	Pass	11.3		
2	132.3	478	2202	Blue_1	132	0.0	Pass	76.2		

3	133.4	337	1763	Blue_1	133	0.0	Pass	35.8	[<Deleted>]
4	138.5	143	707	Blue_1	139	0.0	Pass	9.1	
