# Supplementary material for: Genetic diversity and population structure of Polygonatum cyrtonema Hua in China using SSR markers
Source: PLoS One. 2023 Aug 31;18(8):e0290605. doi: 10.1371/journal.pone.0290605 (PMC10470896; doi:10.1371/journal.pone.0290605)
Supplement: S1 Data — (ZIP) [file pone.0290605.s002.zip › ╨┬╜¿╬─╝■╝╨ (2)/╥2╬∩26/yinwu26.pdf]

SoftGenetics

GeneMarker V2.2.0

Allele Report

Sample 1: Run date and time: 09/15/2020 - 20:24:33 -> 09/15/2020 - 21:10:18

Dye: Blue - 0 peaks - 1.fsa

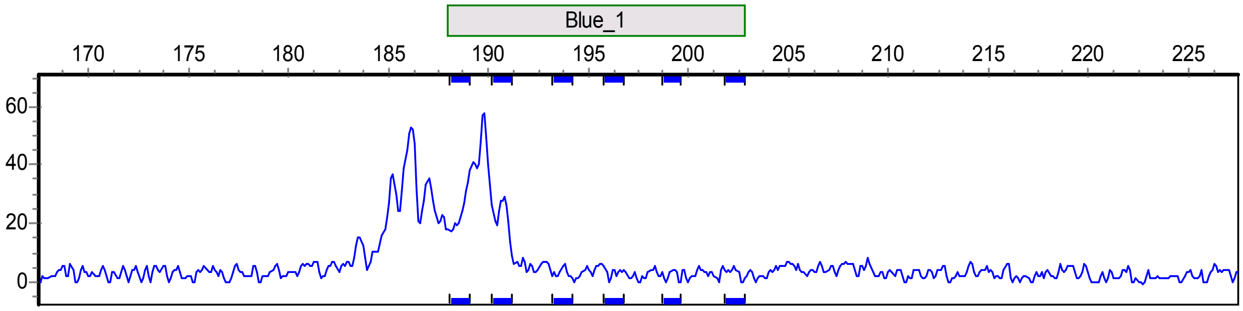

No	Size	Height	Area	Marker	Allele	Difference	Quality	Score	Allele Comments	Sample Comments

Sample 2: Run date and time: 09/15/2020 - 20:24:33 -> 09/15/2020 - 21:10:18

Dye: Blue - 0 peaks - 10.fsa

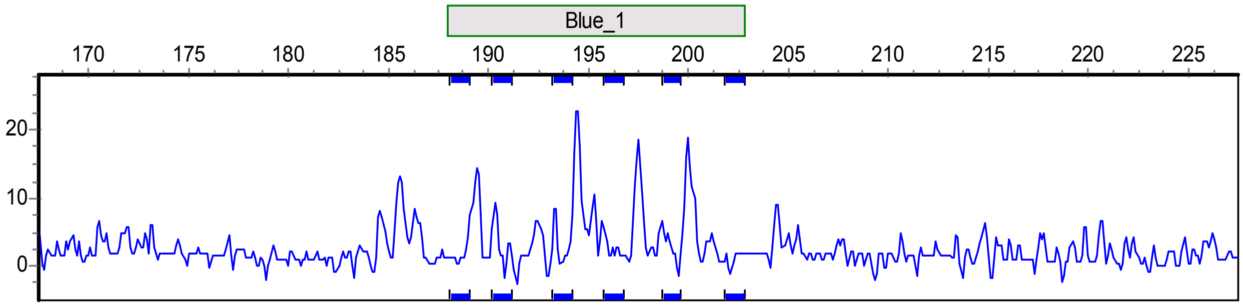

No	Size	Height	Area	Marker	Allele	Difference	Quality	Score	Allele Comments	Sample Comments

Sample 3: Run date and time: 09/15/2020 - 20:24:33 -> 09/15/2020 - 21:10:18

Dye: Blue - 0 peaks - 11.fsa

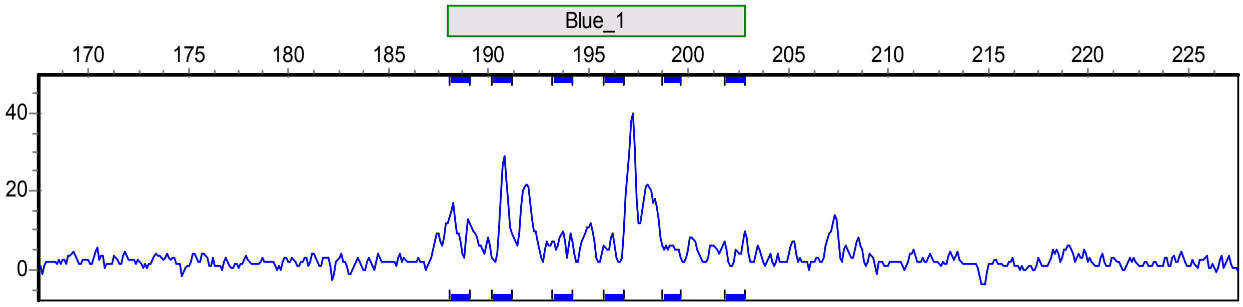

No	Size	Height	Area	Marker	Allele	Difference	Quality	Score	Allele Comments	Sample Comments

Sample 4: Run date and time: 09/15/2020 - 20:24:33 -> 09/15/2020 - 21:10:18

Dye: Blue - 0 peaks - 12.fsa

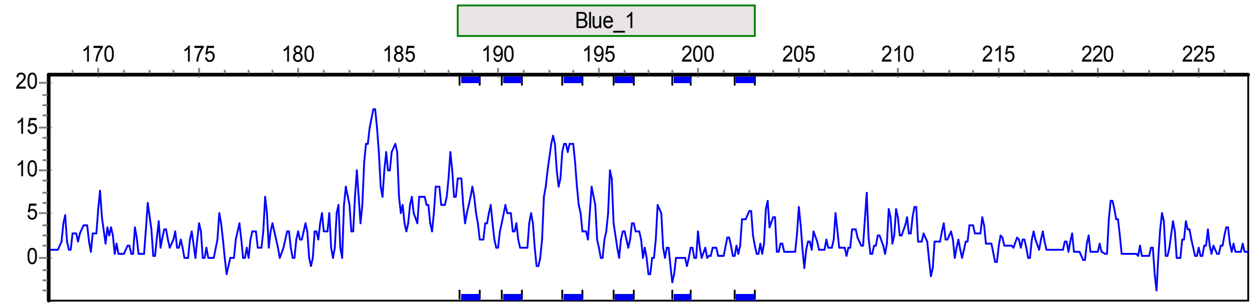

No	Size	Height	Area	Marker	Allele	Difference	Quality	Score	Allele Comments	Sample Comments

Sample 5: Run date and time: 09/15/2020 - 20:24:33 -> 09/15/2020 - 21:10:18

Dye: Blue - 1 peaks - 13.fsa

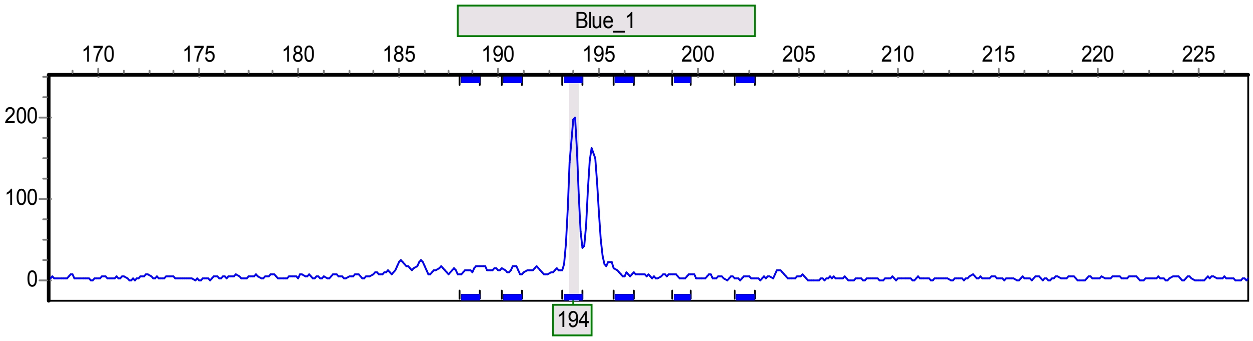

No	Size	Height	Area	Marker	Allele	Difference	Quality	Score	Allele Comments	Sample Comments
1	193.8	200	1009	Blue_1	194	0.1	Pass	15.5		

Sample 6: Run date and time: 09/15/2020 - 20:24:33 -> 09/15/2020 - 21:10:18

Dye: Blue - 0 peaks - 14.fsa

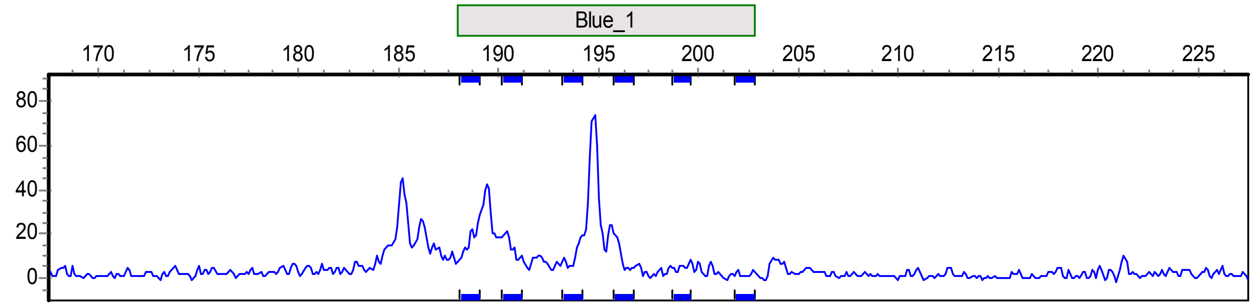

No	Size	Height	Area	Marker	Allele	Difference	Quality	Score	Allele Comments	Sample Comments

Sample 7: Run date and time: 09/15/2020 - 20:24:33 -> 09/15/2020 - 21:10:18

Dye: Blue - 1 peaks - 15.fsa

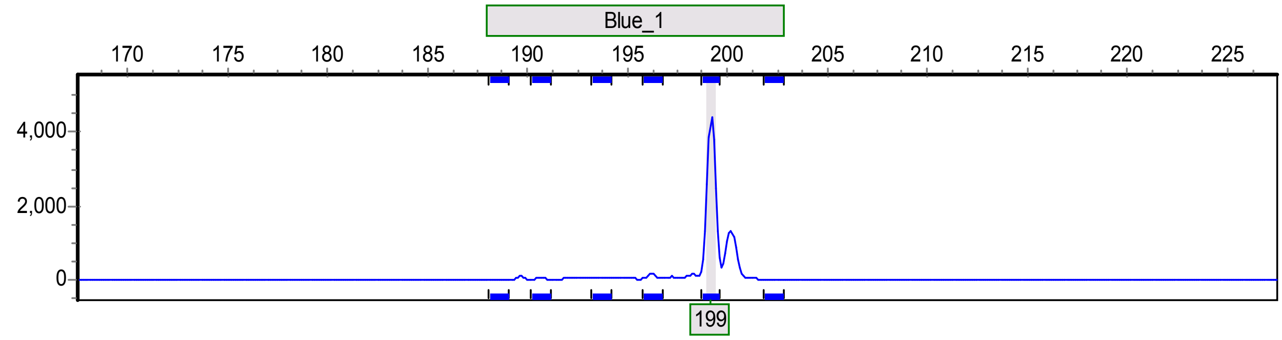

No	Size	Height	Area	Marker	Allele	Difference	Quality	Score	Allele Comments	Sample Comments
1	199.2	4367	19697	Blue_1	199	0.0	Pass	500.0		

Sample 8: Run date and time: 09/15/2020 - 20:24:33 -> 09/15/2020 - 21:10:18

Dye: Blue - 1 peaks - 16.fsa

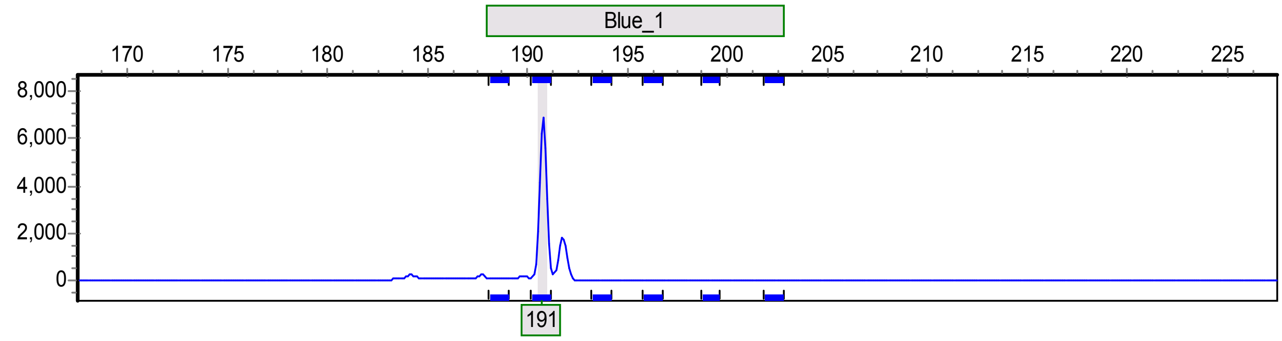

No	Size	Height	Area	Marker	Allele	Difference	Quality	Score	Allele Comments	Sample Comments
1	190.8	6860	29194	Blue_1	191	0.1	Pass	500.0		

Sample 9: Run date and time: 09/15/2020 - 20:24:33 -> 09/15/2020 - 21:10:18

Dye: Blue - 1 peaks - 17.fsa

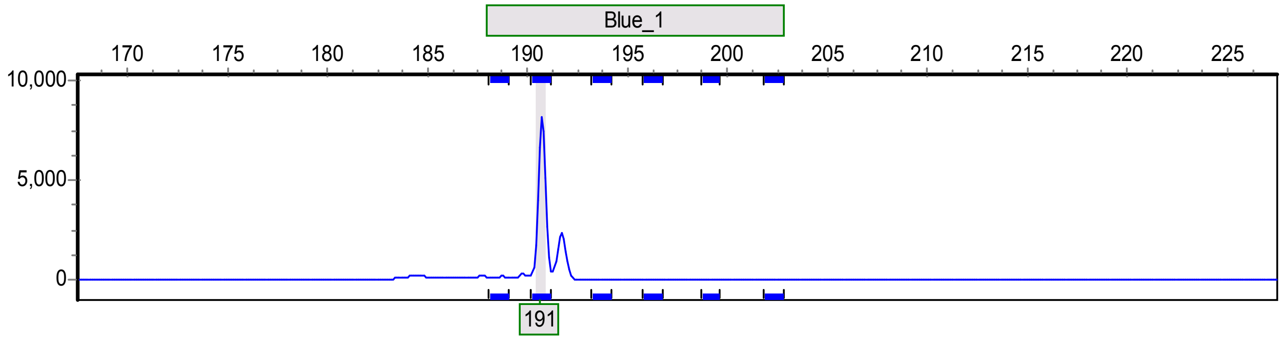

No	Size	Height	Area	Marker	Allele	Difference	Quality	Score	Allele Comments	Sample Comments
1	190.7	8153	35328	Blue_1	191	0.0	Pass	500.0		

Sample 10: Run date and time: 09/15/2020 - 20:24:33 -> 09/15/2020 - 21:10:18

Dye: Blue - 0 peaks - 18.fsa

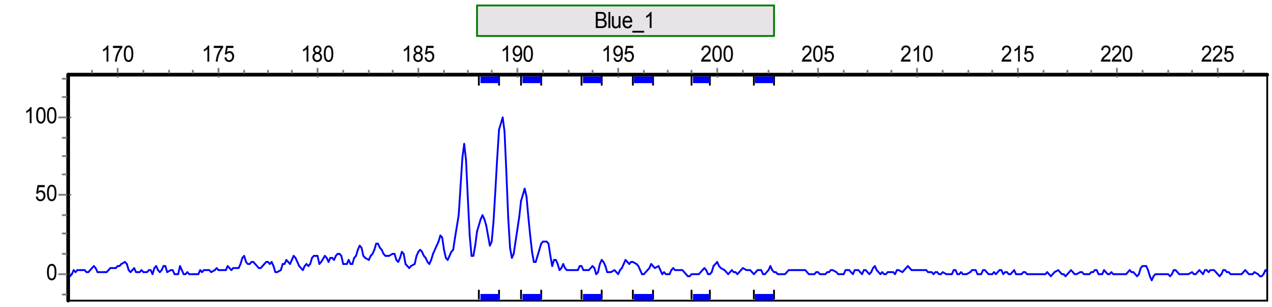

No	Size	Height	Area	Marker	Allele	Difference	Quality	Score	Allele Comments	Sample Comments

Sample 11: Run date and time: 09/15/2020 - 20:24:33 -> 09/15/2020 - 21:10:18

Dye: Blue - 1 peaks - 19.fsa

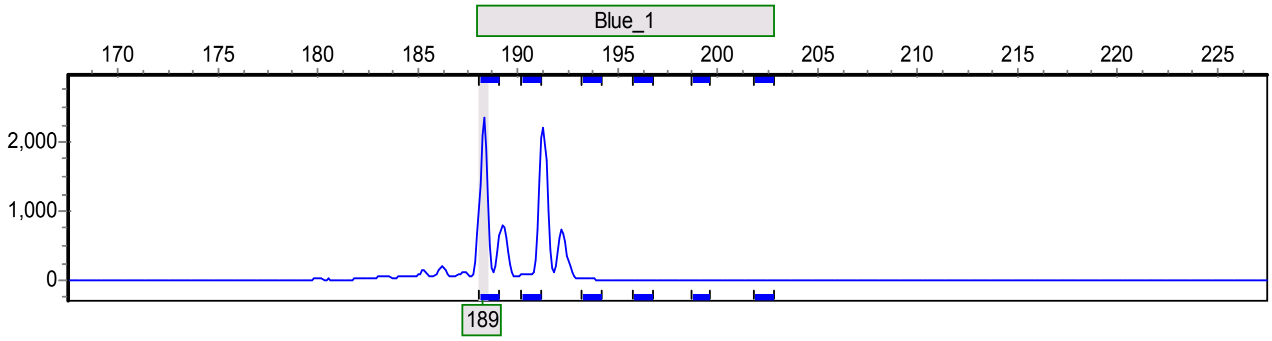

No	Size	Height	Area	Marker	Allele	Difference	Quality	Score	Allele Comments	Sample Comments
1	188.3	2325	9779	Blue_1	189	0.3	Pass	500.0		

Sample 12: Run date and time: 09/15/2020 - 20:24:33 -> 09/15/2020 - 21:10:18

Dye: Blue - 1 peaks - 2.fsa

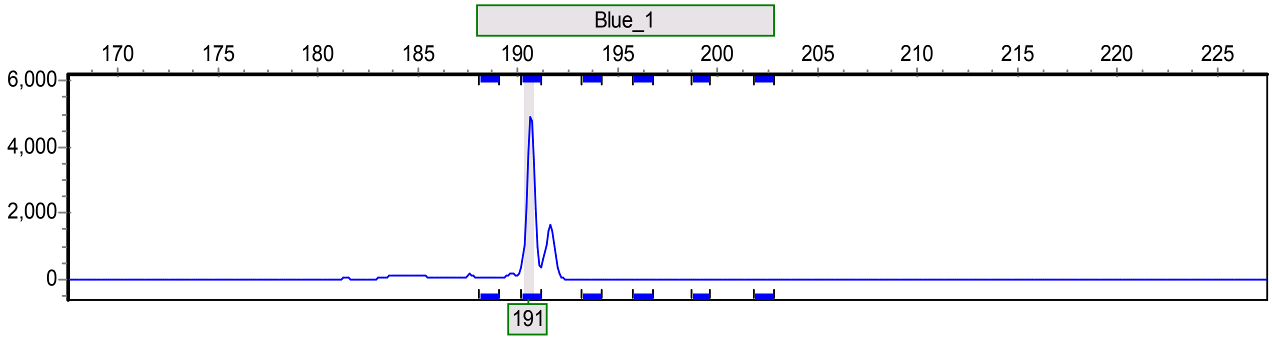

No	Size	Height	Area	Marker	Allele	Difference	Quality	Score	Allele Comments	Sample Comments
1	190.6	4891	22584	Blue_1	191	0.1	Pass	500.0		

Allele Report

Sample 13: Run date and time: 09/15/2020 - 20:24:33 -> 09/15/2020 - 21:10:18

Dye: Blue - 1 peaks - 20.fsa

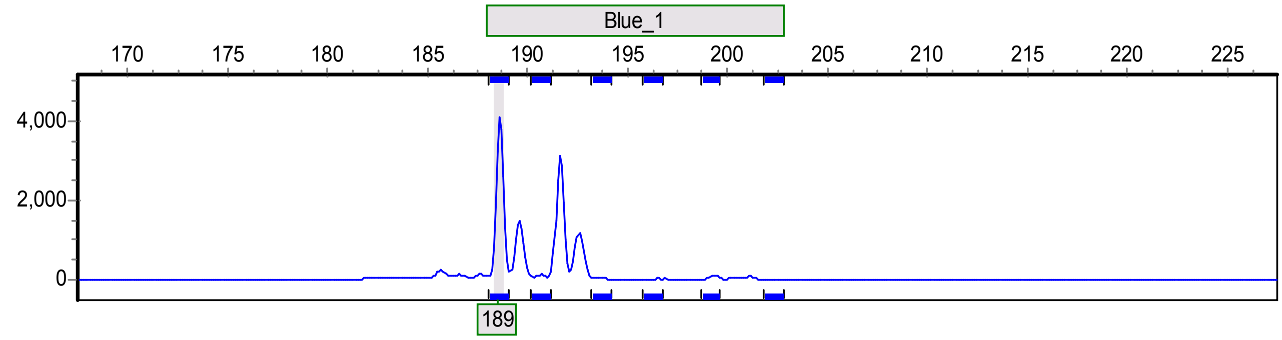

No	Size	Height	Area	Marker	Allele	Difference	Quality	Score	Allele Comments	Sample Comments
1	188.6	4078	17277	Blue_1	189	0.0	Pass	500.0		

Sample 14: Run date and time: 09/15/2020 - 20:24:33 -> 09/15/2020 - 21:10:18

Dye: Blue - 1 peaks - 21.fsa

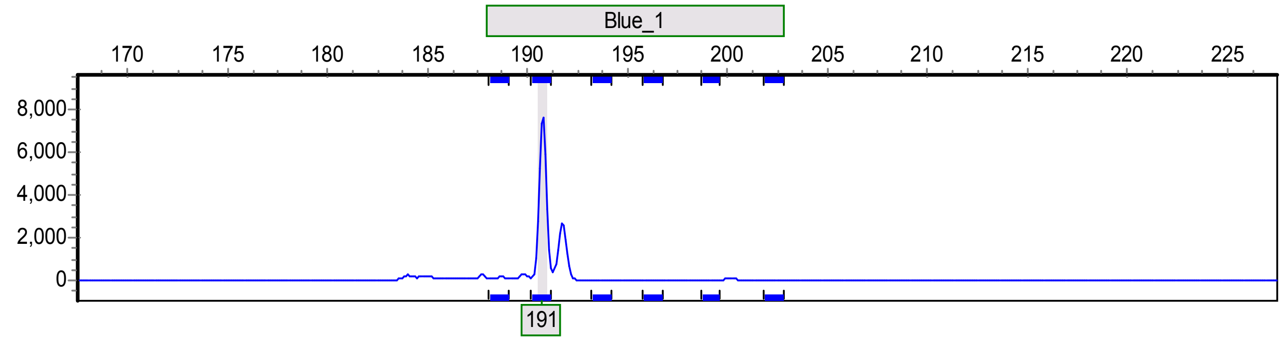

No	Size	Height	Area	Marker	Allele	Difference	Quality	Score	Allele Comments	Sample Comments
1	190.8	7613	33041	Blue_1	191	0.1	Pass	500.0		

Sample 15: Run date and time: 09/15/2020 - 20:24:33 -> 09/15/2020 - 21:10:18

Dye: Blue - 1 peaks - 22.fsa

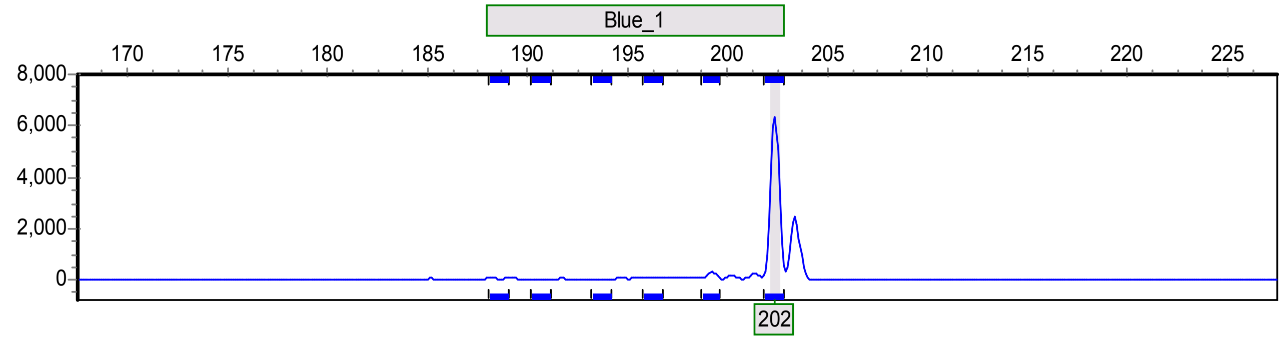

No	Size	Height	Area	Marker	Allele	Difference	Quality	Score	Allele Comments	Sample Comments
1	202.4	6315	28487	Blue_1	202	0.0	Pass	500.0		

Sample 16: Run date and time: 09/15/2020 - 20:24:33 -> 09/15/2020 - 21:10:18

Dye: Blue - 1 peaks - 23.fsa

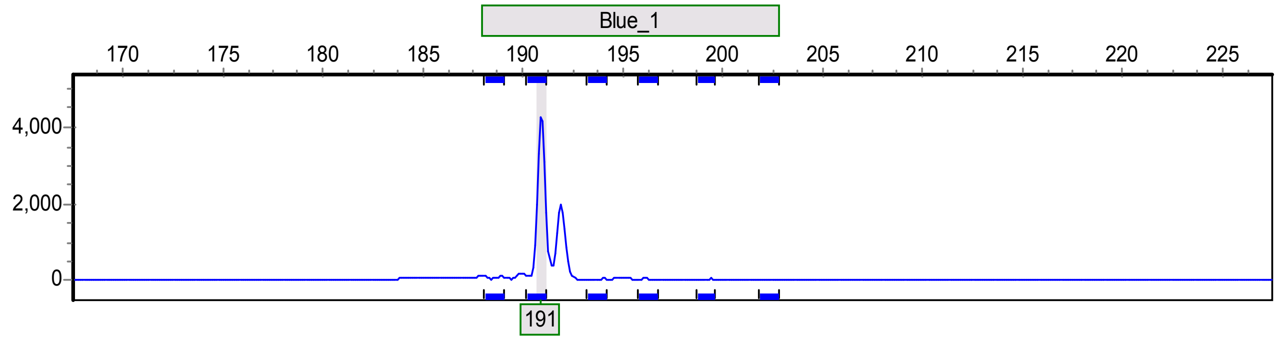

No	Size	Height	Area	Marker	Allele	Difference	Quality	Score	Allele Comments	Sample Comments
1	190.9	4267	19494	Blue_1	191	0.2	Pass	500.0		

Sample 17: Run date and time: 09/15/2020 - 20:24:33 -> 09/15/2020 - 21:10:18

Dye: Blue - 0 peaks - 24.fsa

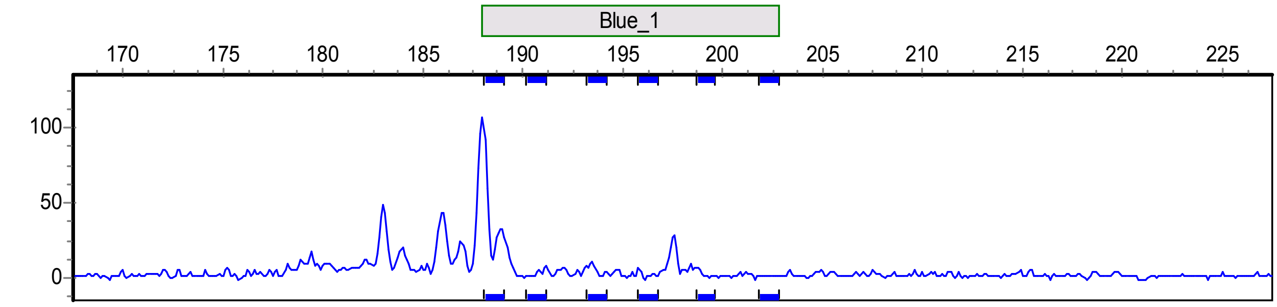

No	Size	Height	Area	Marker	Allele	Difference	Quality	Score	Allele Comments	Sample Comments

Sample 18: Run date and time: 09/15/2020 - 20:24:33 -> 09/15/2020 - 21:10:18

Dye: Blue - 0 peaks - 25.fsa

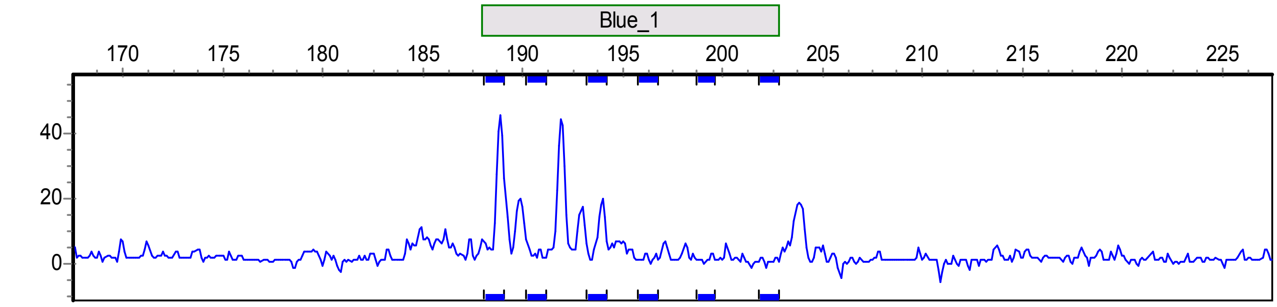

No	Size	Height	Area	Marker	Allele	Difference	Quality	Score	Allele Comments	Sample Comments

Allele Report

Sample 19: Run date and time: 09/15/2020 - 20:24:33 -> 09/15/2020 - 21:10:18

Dye: Blue - 0 peaks - 26.fsa

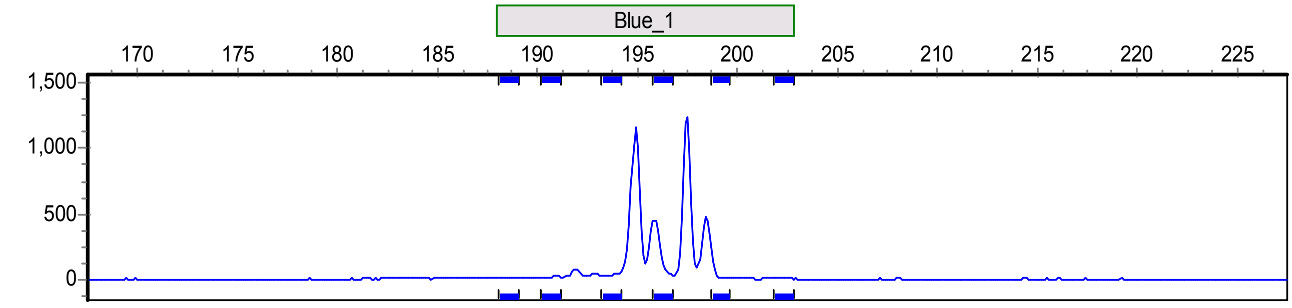

No	Size	Height	Area	Marker	Allele	Difference	Quality	Score	Allele Comments	Sample Comments

Sample 20: Run date and time: 09/15/2020 - 20:24:33 -> 09/15/2020 - 21:10:18

Dye: Blue - 1 peaks - 27.fsa

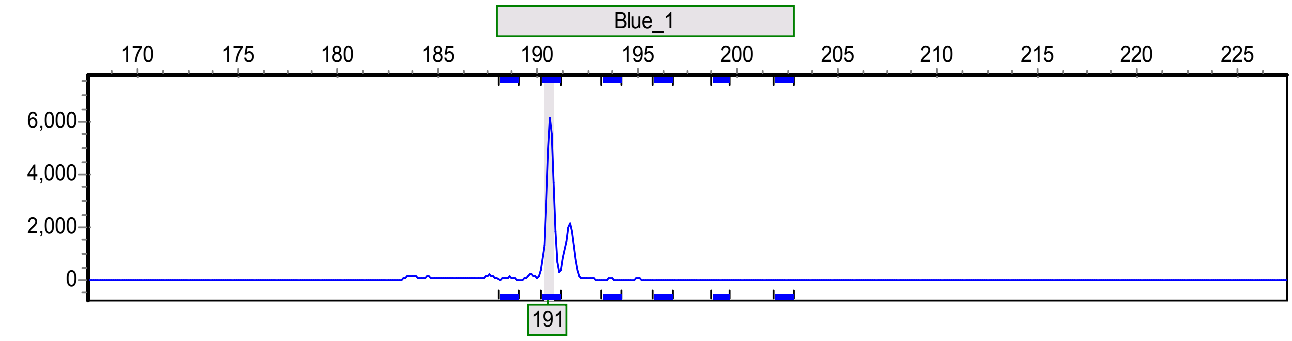

No	Size	Height	Area	Marker	Allele	Difference	Quality	Score	Allele Comments	Sample Comments
1	190.6	6149	25909	Blue_1	191	0.1	Pass	500.0		

Sample 21: Run date and time: 09/15/2020 - 20:24:33 -> 09/15/2020 - 21:10:18

Dye: Blue - 2 peaks - 28.fsa

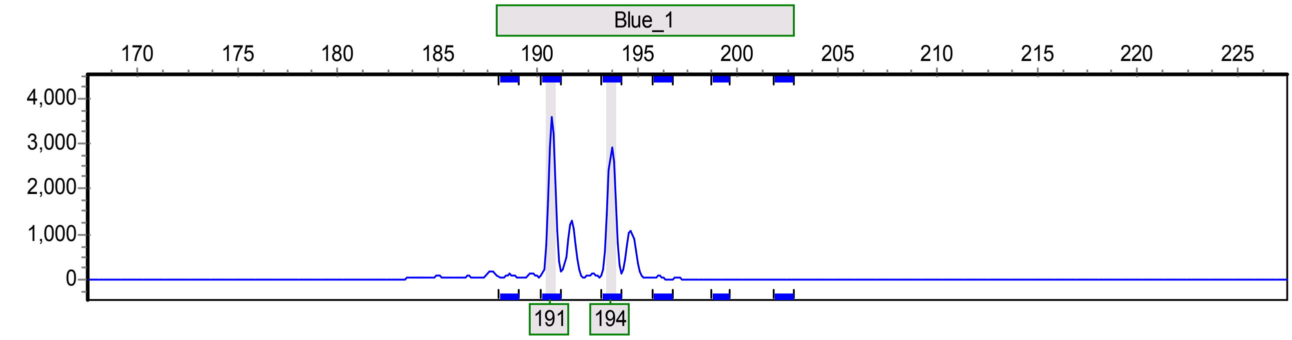

No	Size	Height	Area	Marker	Allele	Difference	Quality	Score	Allele Comments	Sample Comments
1	190.7	3557	15008	Blue_1	191	0.0	Pass	500.0		
2	193.7	2921	12211	Blue_1	194	0.0	Pass	500.0		

Sample 22: Run date and time: 09/15/2020 - 20:24:33 -> 09/15/2020 - 21:10:18

Dye: Blue - 2 peaks - 29.fsa

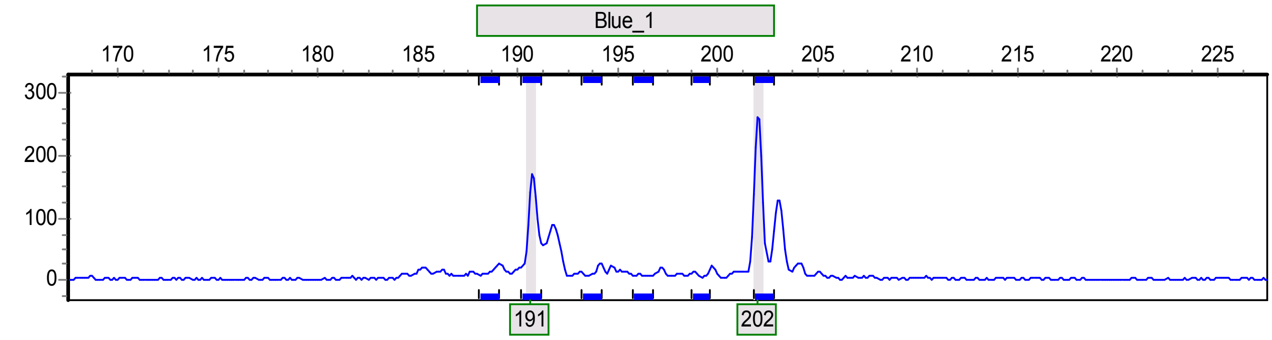

No	Size	Height	Area	Marker	Allele	Difference	Quality	Score	Allele Comments	Sample Comments
1	190.7	171	974	Blue_1	191	0.0	Pass	10.0		
2	202.0	261	1294	Blue_1	202	0.4	Pass	25.7		

Sample 23: Run date and time: 09/15/2020 - 20:24:33 -> 09/15/2020 - 21:10:18

Dye: Blue - 1 peaks - 3.fsa

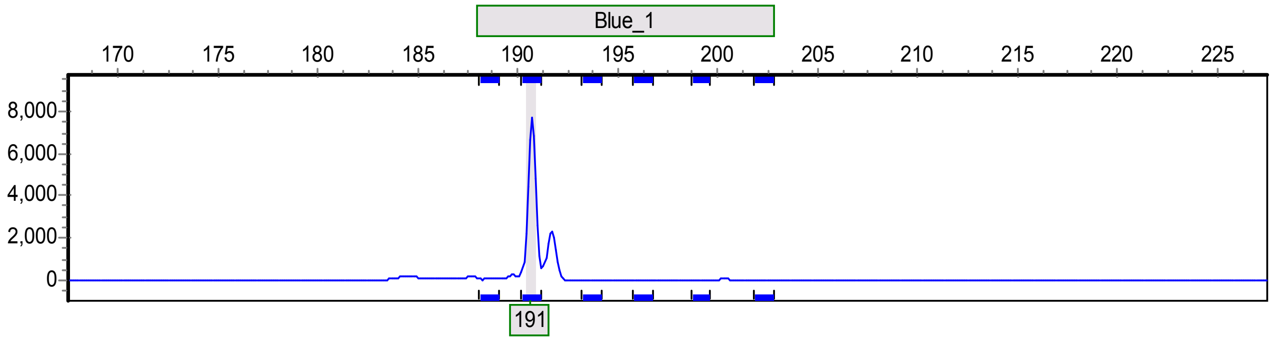

No	Size	Height	Area	Marker	Allele	Difference	Quality	Score	Allele Comments	Sample Comments
1	190.7	7651	35363	Blue_1	191	0.0	Pass	500.0		

Sample 24: Run date and time: 09/15/2020 - 20:24:33 -> 09/15/2020 - 21:10:18

Dye: Blue - 1 peaks - 30.fsa

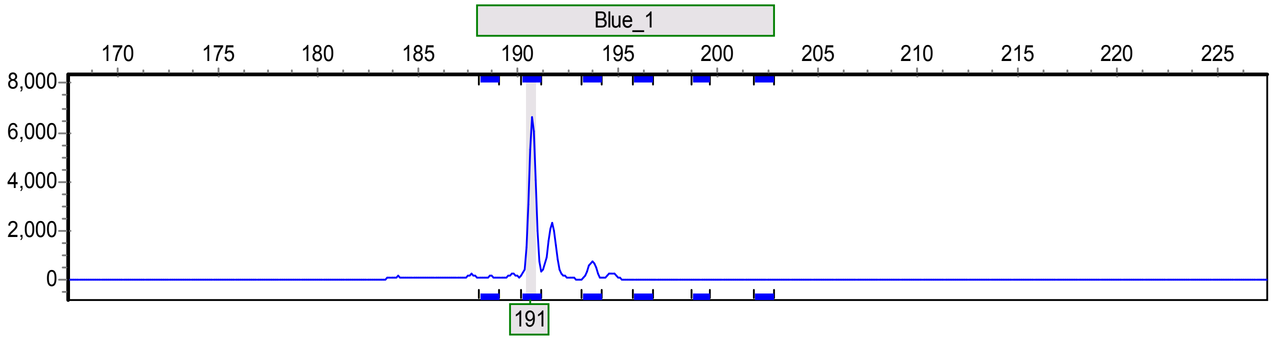

No	Size	Height	Area	Marker	Allele	Difference	Quality	Score	Allele Comments	Sample Comments
1	190.7	6588	27770	Blue_1	191	0.0	Pass	500.0		

Sample 25: Run date and time: 09/15/2020 - 20:24:33 -> 09/15/2020 - 21:10:18

Dye: Blue - 0 peaks - 31.fsa

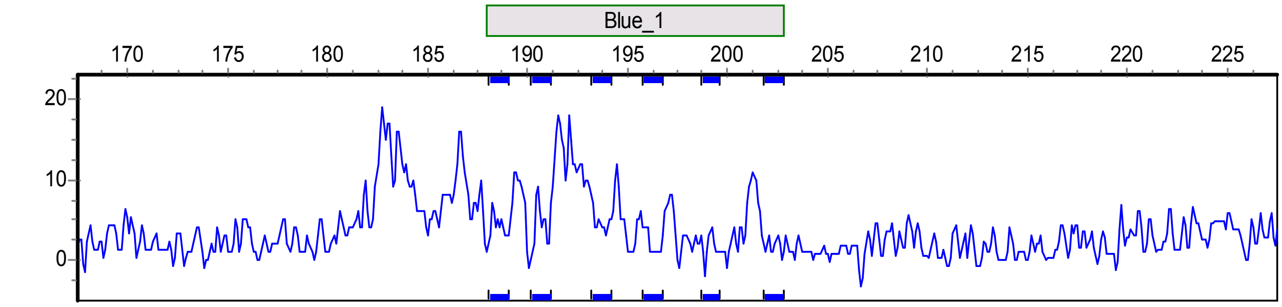

No	Size	Height	Area	Marker	Allele	Difference	Quality	Score	Allele Comments	Sample Comments

Sample 26: Run date and time: 09/15/2020 - 20:24:33 -> 09/15/2020 - 21:10:18

Dye: Blue - 0 peaks - 32.fsa

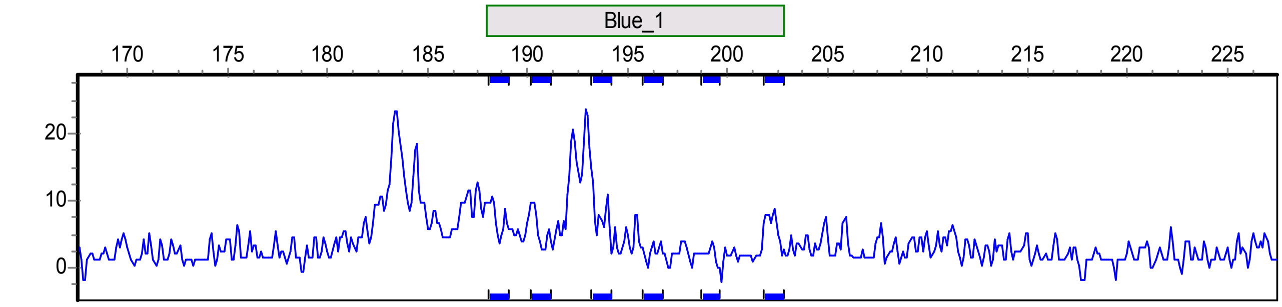

No	Size	Height	Area	Marker	Allele	Difference	Quality	Score	Allele Comments	Sample Comments

Sample 27: Run date and time: 09/15/2020 - 20:24:33 -> 09/15/2020 - 21:10:18

Dye: Blue - 1 peaks - 33.fsa

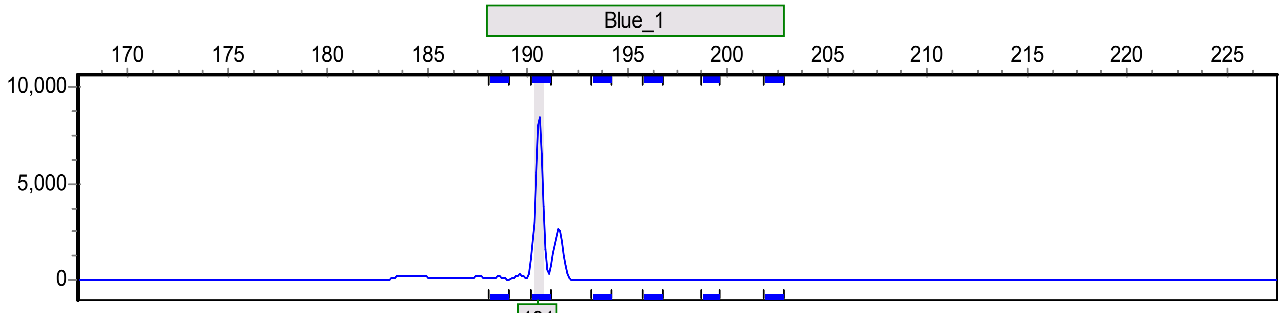

No	Size	Height	Area	Marker	Allele	Difference	Quality	Score	Allele Comments	Sample Comments
1	190.6	8374	36045	Blue_1	191	0.1	Pass	500.0		

Allele Report

Sample 28: Run date and time: 09/15/2020 - 20:24:33 -> 09/15/2020 - 21:10:18

Dye: Blue - 1 peaks - 34.fsa

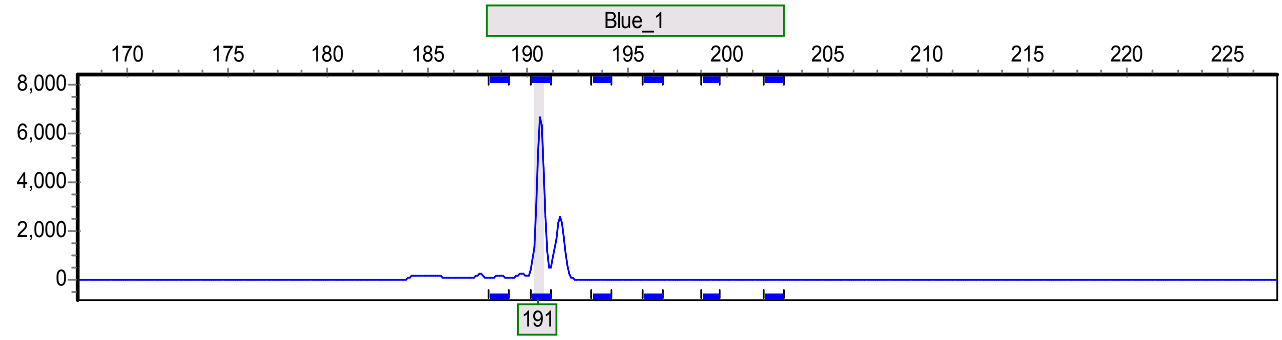

No	Size	Height	Area	Marker	Allele	Difference	Quality	Score	Allele Comments	Sample Comments
1	190.6	6646	29698	Blue_1	191	0.1	Pass	500.0		

Sample 29: Run date and time: 09/15/2020 - 20:24:33 -> 09/15/2020 - 21:10:18

Dye: Blue - 2 peaks - 35.fsa

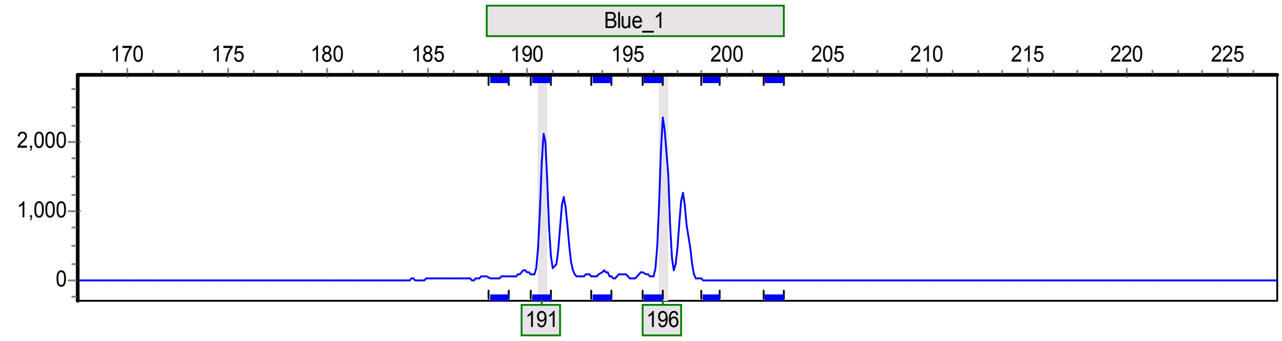

No	Size	Height	Area	Marker	Allele	Difference	Quality	Score	Allele Comments	Sample Comments
1	190.8	2108	9392	Blue_1	191	0.1	Pass	500.0		
2	196.8	2340	10144	Blue_1	196	0.5	Pass	500.0		

Sample 30: Run date and time: 09/15/2020 - 20:24:33 -> 09/15/2020 - 21:10:18

Dye: Blue - 1 peaks - 36.fsa

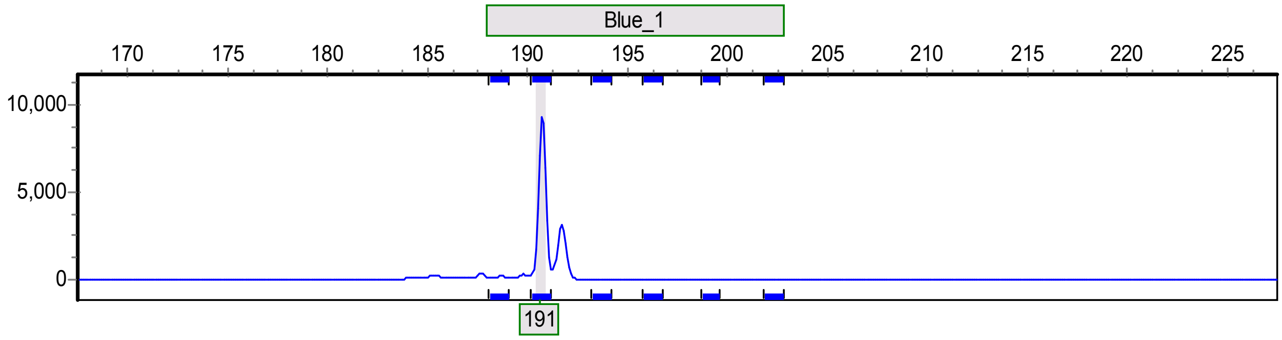

No	Size	Height	Area	Marker	Allele	Difference	Quality	Score	Allele Comments	Sample Comments
1	190.7	9272	40266	Blue_1	191	0.0	Pass	500.0		

Allele Report

Sample 31: Run date and time: 09/15/2020 - 20:24:33 -> 09/15/2020 - 21:10:18

Dye: Blue - 1 peaks - 37.fsa

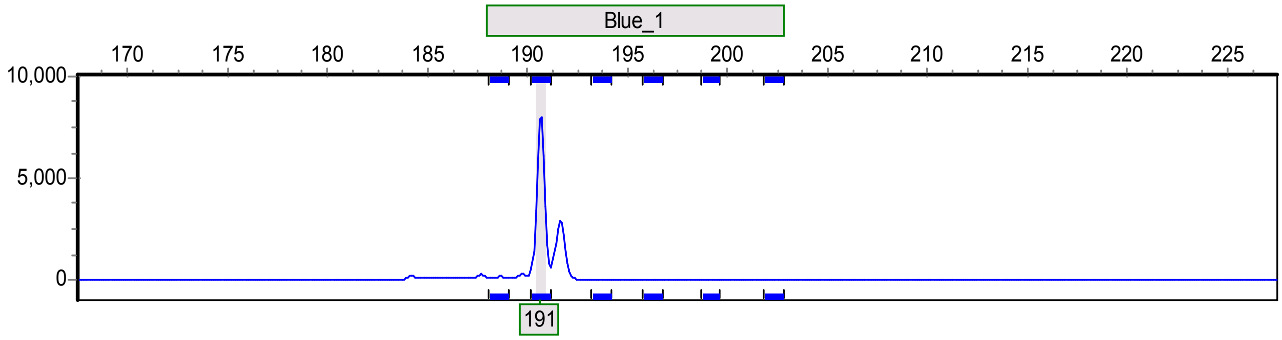

No	Size	Height	Area	Marker	Allele	Difference	Quality	Score	Allele Comments	Sample Comments
1	190.7	7976	36721	Blue_1	191	0.0	Pass	500.0		

Sample 32: Run date and time: 09/15/2020 - 20:24:33 -> 09/15/2020 - 21:10:18

Dye: Blue - 1 peaks - 38.fsa

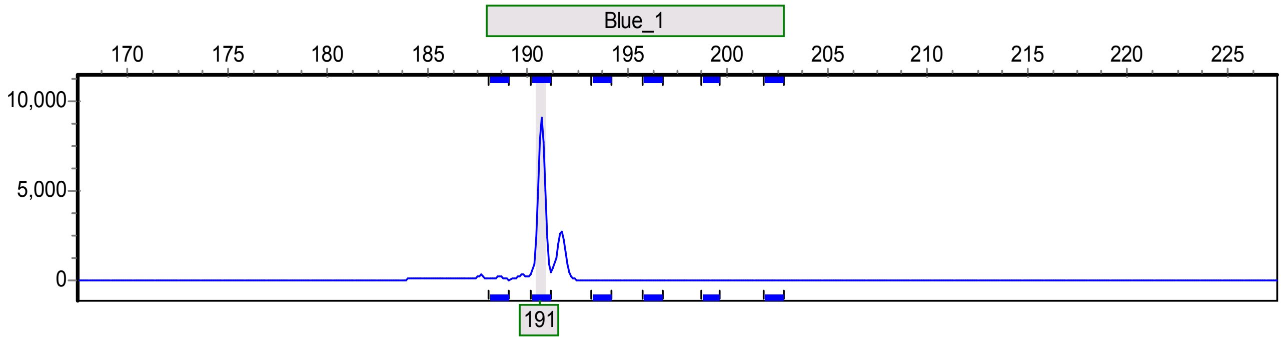

No	Size	Height	Area	Marker	Allele	Difference	Quality	Score	Allele Comments	Sample Comments
1	190.7	9081	38798	Blue_1	191	0.0	Pass	500.0		

Sample 33: Run date and time: 09/15/2020 - 20:24:33 -> 09/15/2020 - 21:10:18

Dye: Blue - 1 peaks - 39.fsa

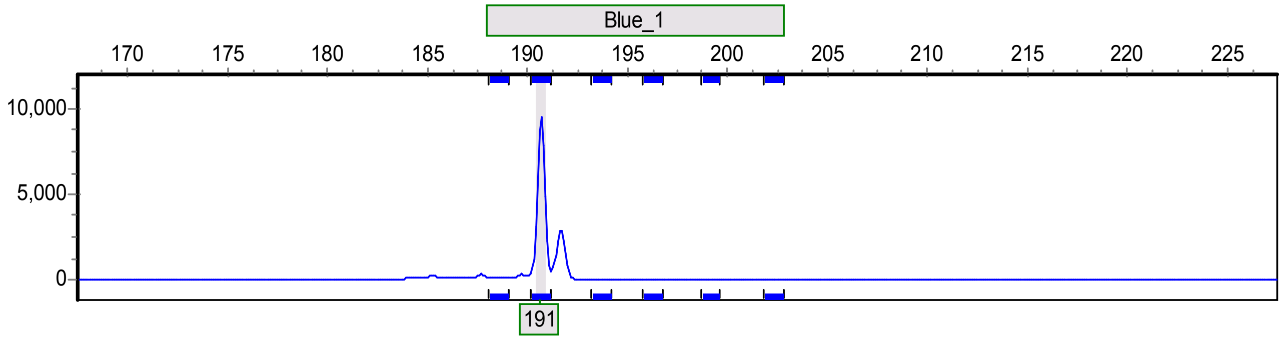

No	Size	Height	Area	Marker	Allele	Difference	Quality	Score	Allele Comments	Sample Comments
1	190.7	9473	41754	Blue_1	191	0.0	Pass	500.0		

Sample 34: Run date and time: 09/15/2020 - 20:24:33 -> 09/15/2020 - 21:10:18

Dye: Blue - 1 peaks - 4.fsa

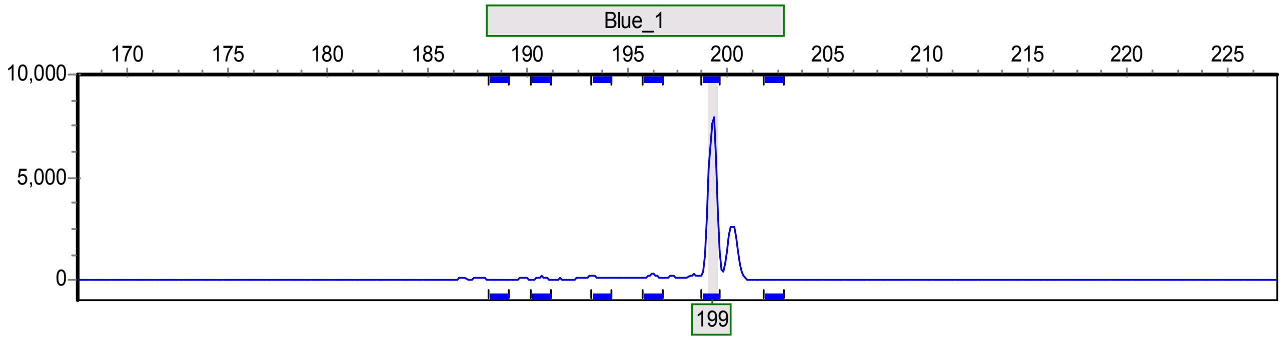

No	Size	Height	Area	Marker	Allele	Difference	Quality	Score	Allele Comments	Sample Comments
1	199.3	7903	34206	Blue_1	199	0.1	Pass	500.0		

Sample 35: Run date and time: 09/15/2020 - 20:24:33 -> 09/15/2020 - 21:10:18

Dye: Blue - 1 peaks - 40.fsa

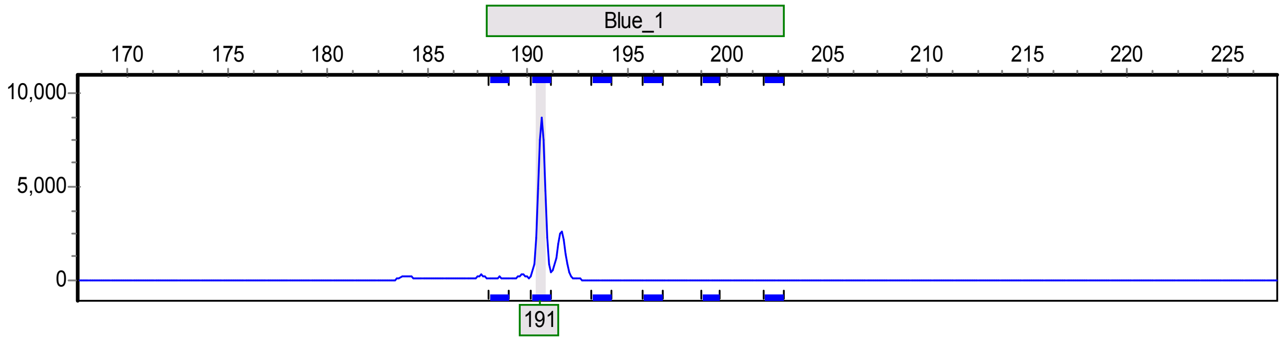

No	Size	Height	Area	Marker	Allele	Difference	Quality	Score	Allele Comments	Sample Comments
1	190.7	8639	37106	Blue_1	191	0.0	Pass	500.0		

Sample 36: Run date and time: 09/15/2020 - 20:24:33 -> 09/15/2020 - 21:10:18

Dye: Blue - 1 peaks - 41.fsa

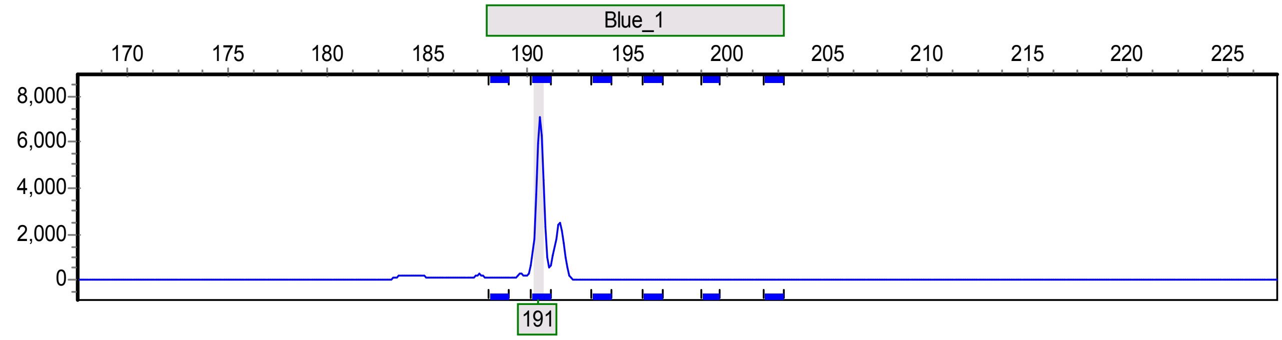

No	Size	Height	Area	Marker	Allele	Difference	Quality	Score	Allele Comments	Sample Comments
1	190.6	7065	31244	Blue_1	191	0.1	Pass	500.0		

Allele Report

Sample 37: Run date and time: 09/15/2020 - 20:24:33 -> 09/15/2020 - 21:10:18

Dye: Blue - 1 peaks - 42.fsa

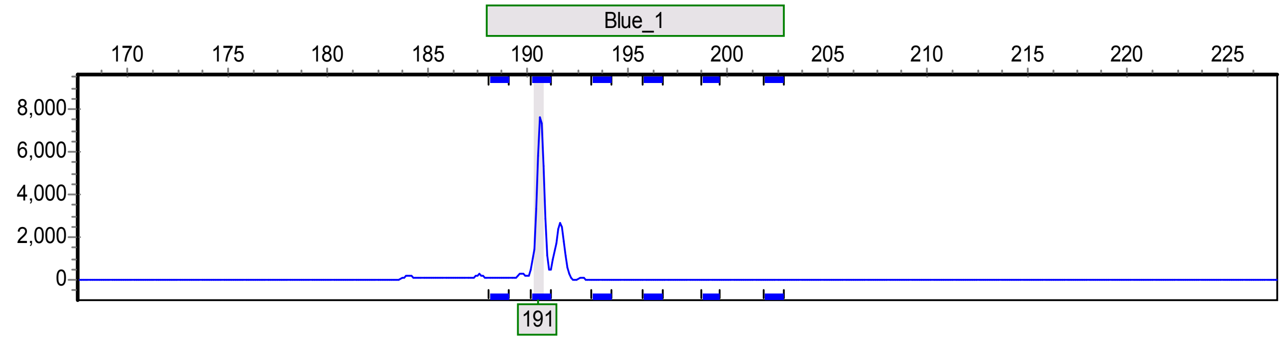

No	Size	Height	Area	Marker	Allele	Difference	Quality	Score	Allele Comments	Sample Comments
1	190.6	7611	33373	Blue_1	191	0.1	Pass	500.0		

Sample 38: Run date and time: 09/15/2020 - 20:24:33 -> 09/15/2020 - 21:10:18

Dye: Blue - 1 peaks - 43.fsa

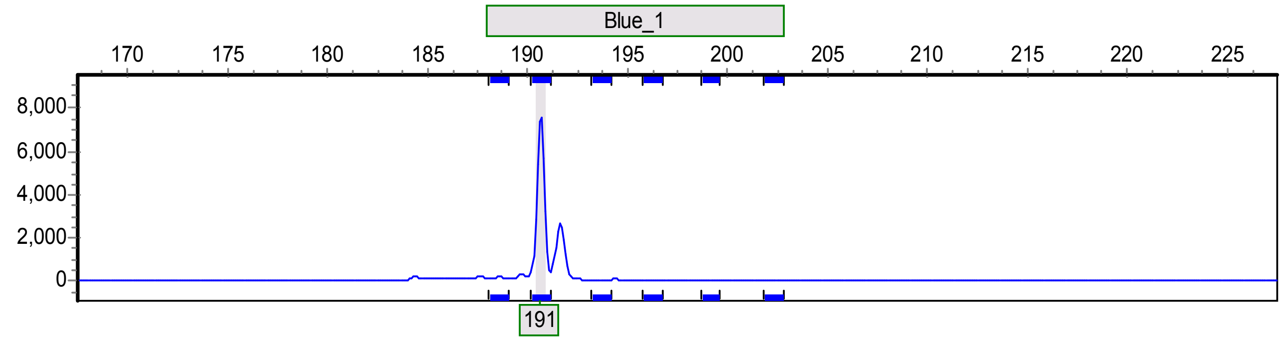

No	Size	Height	Area	Marker	Allele	Difference	Quality	Score	Allele Comments	Sample Comments
1	190.7	7523	32876	Blue_1	191	0.0	Pass	500.0		

Sample 39: Run date and time: 09/15/2020 - 20:24:33 -> 09/15/2020 - 21:10:18

Dye: Blue - 1 peaks - 44.fsa

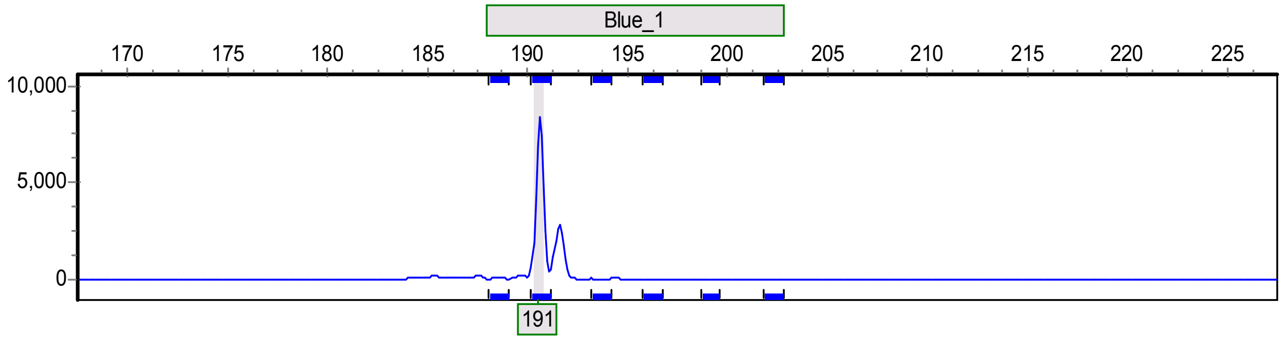

No	Size	Height	Area	Marker	Allele	Difference	Quality	Score	Allele Comments	Sample Comments
1	190.6	8339	35623	Blue_1	191	0.1	Pass	500.0		

Sample 40: Run date and time: 09/15/2020 - 20:24:33 -> 09/15/2020 - 21:10:18

Dye: Blue - 1 peaks - 45.fsa

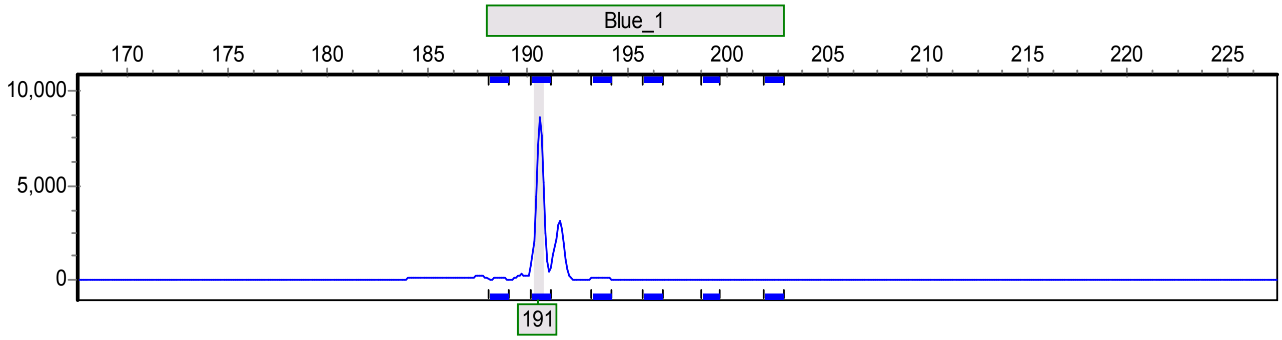

No	Size	Height	Area	Marker	Allele	Difference	Quality	Score	Allele Comments	Sample Comments
1	190.6	8580	36805	Blue_1	191	0.1	Pass	500.0		

Sample 41: Run date and time: 09/15/2020 - 20:24:33 -> 09/15/2020 - 21:10:18

Dye: Blue - 1 peaks - 46.fsa

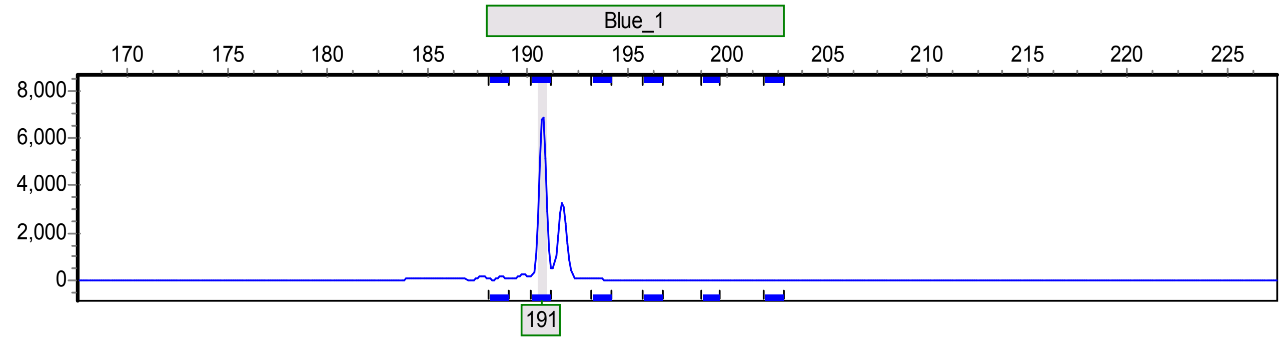

No	Size	Height	Area	Marker	Allele	Difference	Quality	Score	Allele Comments	Sample Comments
1	190.8	6827	30210	Blue_1	191	0.1	Pass	500.0		

Sample 42: Run date and time: 09/15/2020 - 20:24:33 -> 09/15/2020 - 21:10:18

Dye: Blue - 1 peaks - 47.fsa

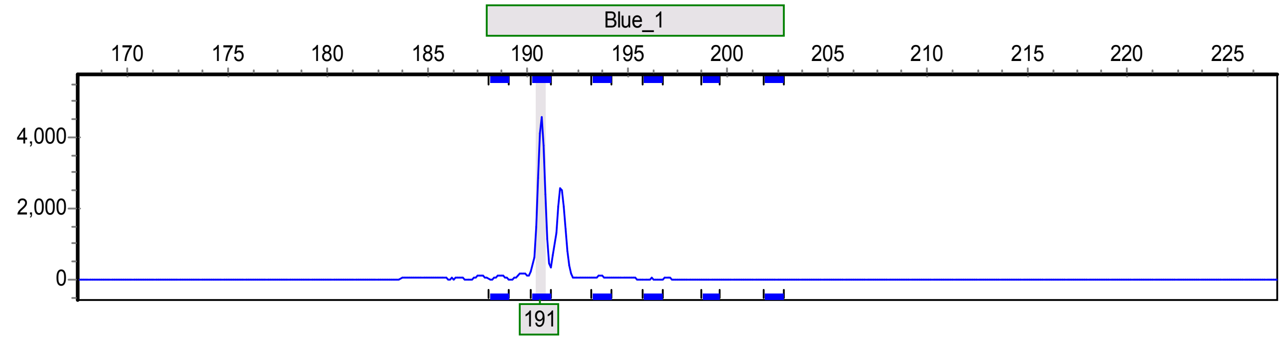

No	Size	Height	Area	Marker	Allele	Difference	Quality	Score	Allele Comments	Sample Comments
1	190.7	4540	20216	Blue_1	191	0.0	Pass	500.0		

Sample 43: Run date and time: 09/15/2020 - 20:24:33 -> 09/15/2020 - 21:10:18

Dye: Blue - 1 peaks - 48.fsa

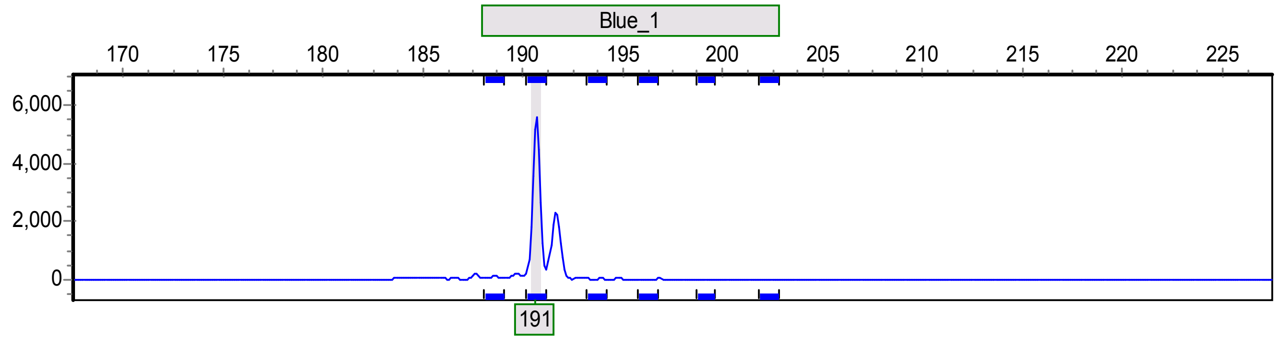

No	Size	Height	Area	Marker	Allele	Difference	Quality	Score	Allele Comments	Sample Comments
1	190.7	5571	24232	Blue_1	191	0.0	Pass	500.0		

Sample 44: Run date and time: 09/15/2020 - 20:24:33 -> 09/15/2020 - 21:10:18

Dye: Blue - 1 peaks - 49.fsa

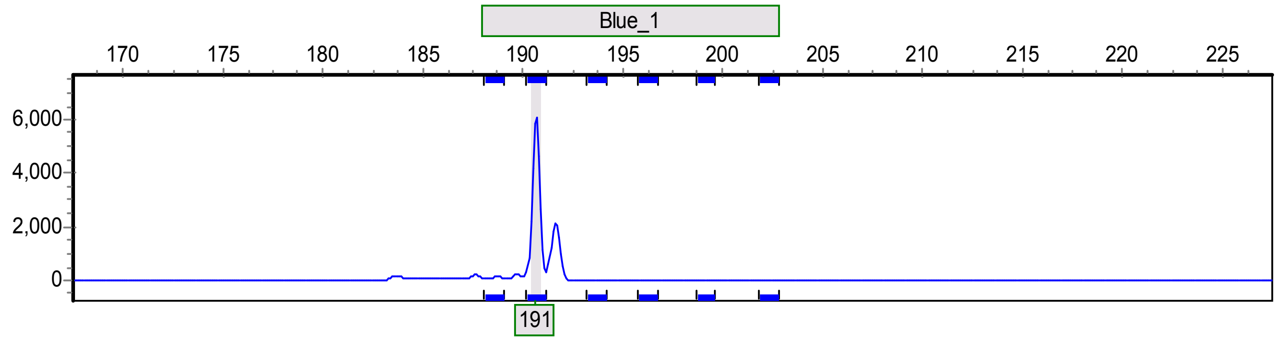

No	Size	Height	Area	Marker	Allele	Difference	Quality	Score	Allele Comments	Sample Comments
1	190.7	6028	26226	Blue_1	191	0.0	Pass	500.0		

Sample 45: Run date and time: 09/15/2020 - 20:24:33 -> 09/15/2020 - 21:10:18

Dye: Blue - 3 peaks - 5.fsa

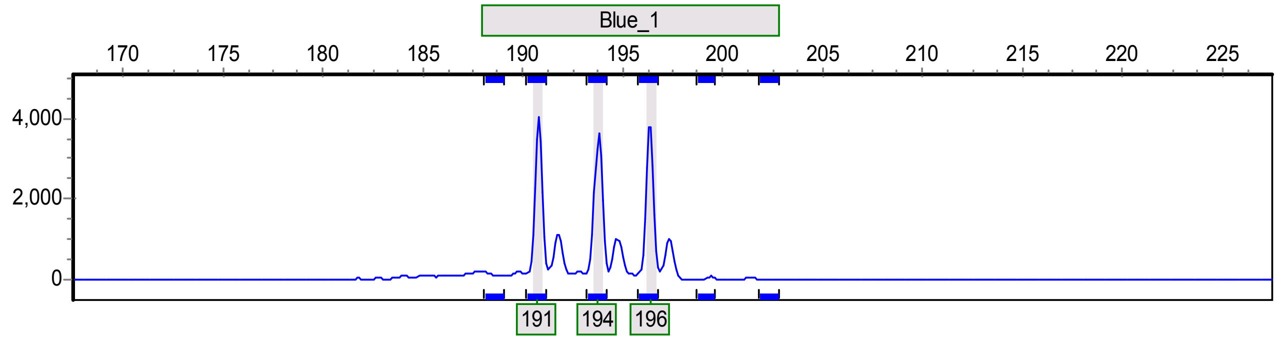

No	Size	Height	Area	Marker	Allele	Difference	Quality	Score	Allele Comments	Sample Comments
1	190.8	4015	17261	Blue_1	191	0.1	Pass	500.0		
2	193.8	3644	15997	Blue_1	194	0.1	Pass	500.0		
3	196.4	3796	16866	Blue_1	196	0.1	Pass	500.0		

Sample 46: Run date and time: 09/15/2020 - 20:24:33 -> 09/15/2020 - 21:10:18

Dye: Blue - 1 peaks - 50.fsa

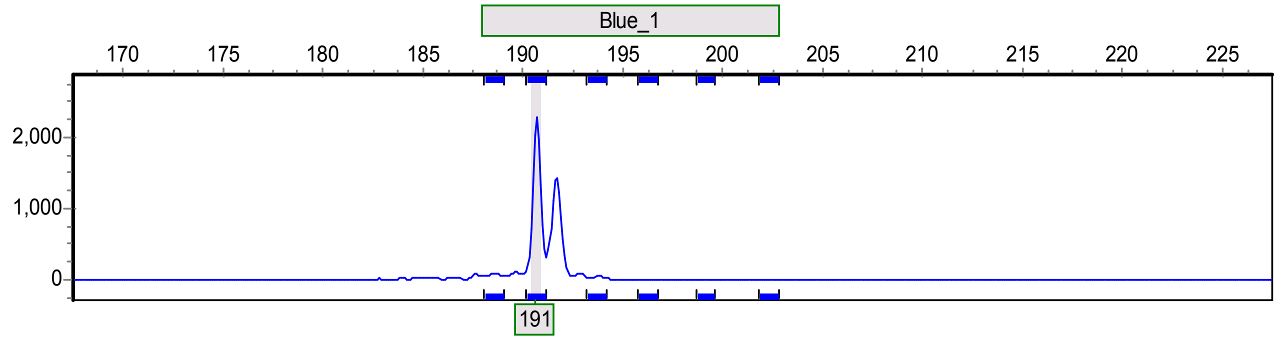

No	Size	Height	Area	Marker	Allele	Difference	Quality	Score	Allele Comments	Sample Comments
1	190.7	2274	10746	Blue_1	191	0.0	Pass	500.0		

Sample 47: Run date and time: 09/15/2020 - 20:24:33 -> 09/15/2020 - 21:10:18

Dye: Blue - 1 peaks - 51.fsa

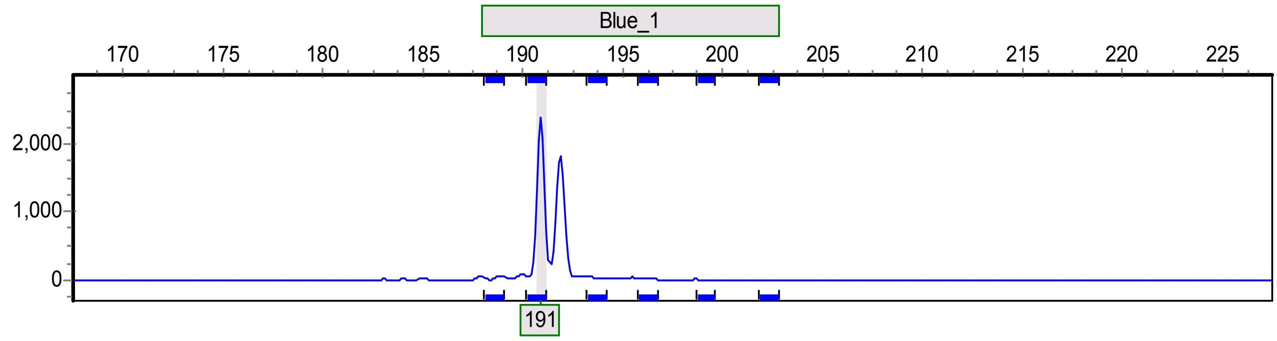

No	Size	Height	Area	Marker	Allele	Difference	Quality	Score	Allele Comments	Sample Comments
1	190.9	2359	10481	Blue_1	191	0.2	Pass	500.0		

Sample 48: Run date and time: 09/15/2020 - 20:24:33 -> 09/15/2020 - 21:10:18

Dye: Blue - 1 peaks - 52.fsa

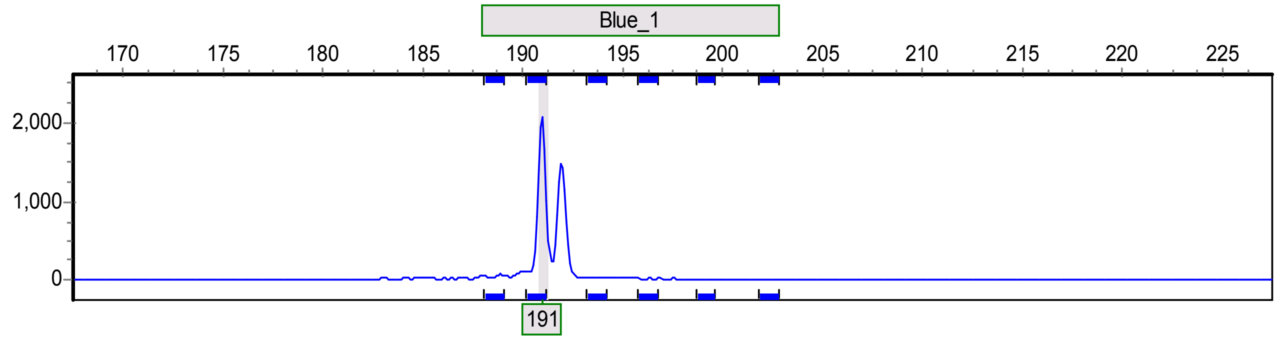

No	Size	Height	Area	Marker	Allele	Difference	Quality	Score	Allele Comments	Sample Comments
1	191.0	2075	9426	Blue_1	191	0.3	Pass	500.0		

Sample 49: Run date and time: 09/15/2020 - 20:24:33 -> 09/15/2020 - 21:10:18

Dye: Blue - 0 peaks - 53.fsa

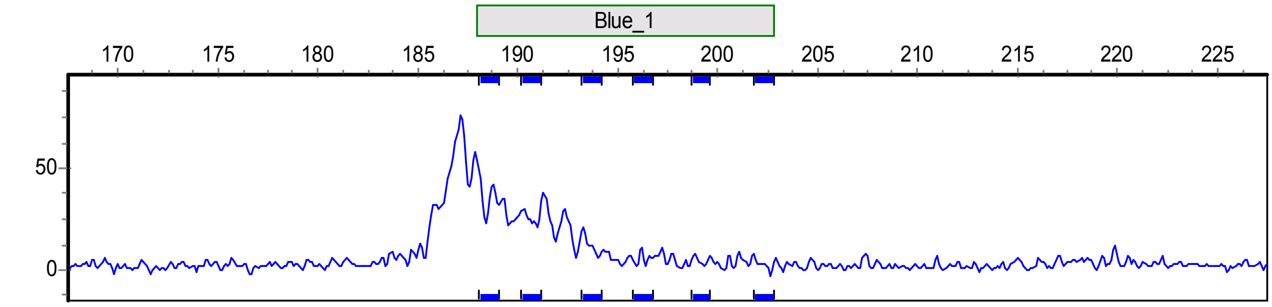

No	Size	Height	Area	Marker	Allele	Difference	Quality	Score	Allele Comments	Sample Comments

Sample 50: Run date and time: 09/15/2020 - 20:24:33 -> 09/15/2020 - 21:10:18

Dye: Blue - 1 peaks - 54.fsa

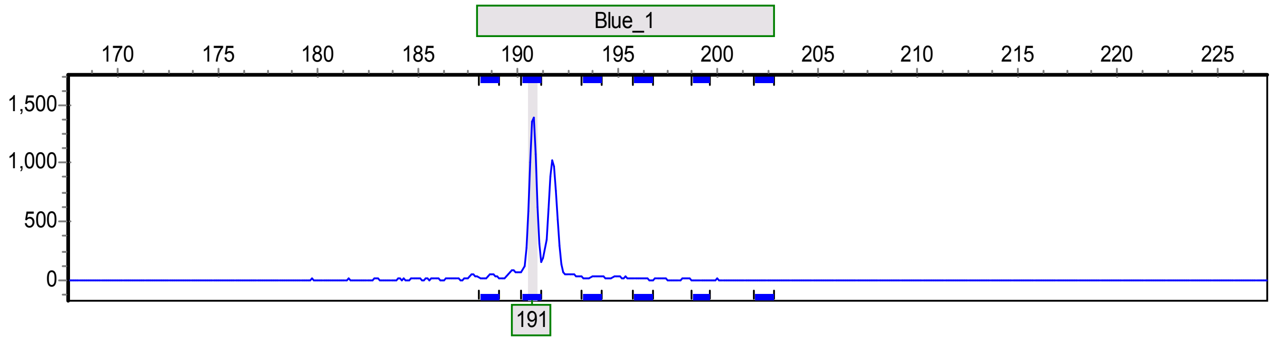

No	Size	Height	Area	Marker	Allele	Difference	Quality	Score	Allele Comments	Sample Comments
1	190.8	1379	6383	Blue_1	191	0.1	Pass	357.3		

Sample 51: Run date and time: 09/15/2020 - 20:24:33 -> 09/15/2020 - 21:10:18

Dye: Blue - 1 peaks - 55.fsa

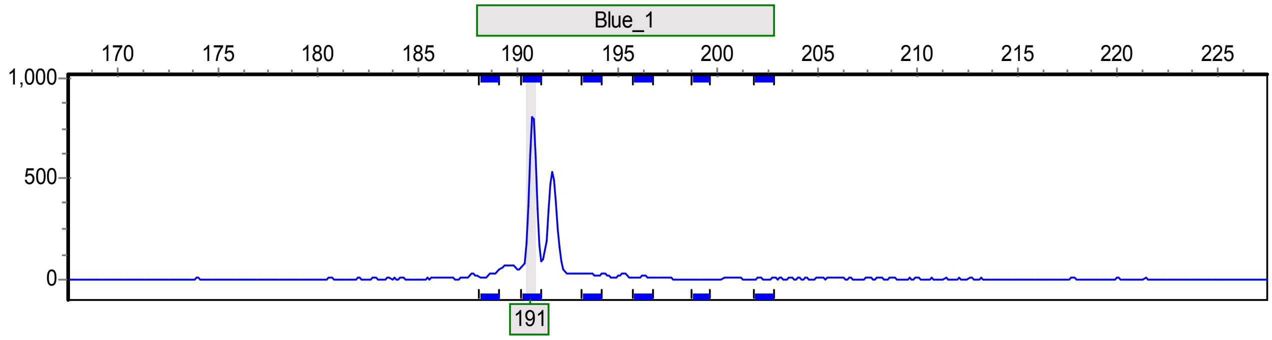

No	Size	Height	Area	Marker	Allele	Difference	Quality	Score	Allele Comments	Sample Comments
1	190.7	803	3750	Blue_1	191	0.0	Pass	164.5		

Sample 52: Run date and time: 09/15/2020 - 20:24:33 -> 09/15/2020 - 21:10:18

Dye: Blue - 1 peaks - 56.fsa

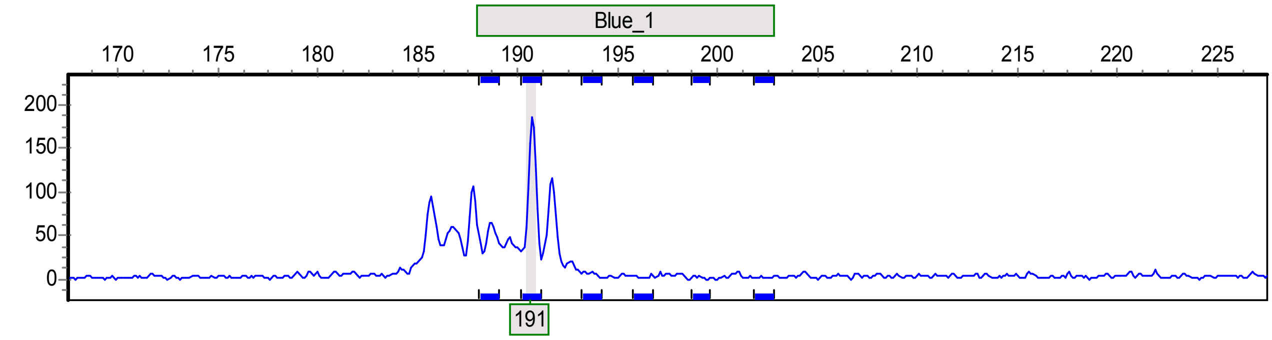

No	Size	Height	Area	Marker	Allele	Difference	Quality	Score	Allele Comments	Sample Comments
1	190.7	186	941	Blue_1	191	0.0	Pass	13.7		

Sample 53: Run date and time: 09/15/2020 - 20:24:33 -> 09/15/2020 - 21:10:18

Dye: Blue - 1 peaks - 57.fsa

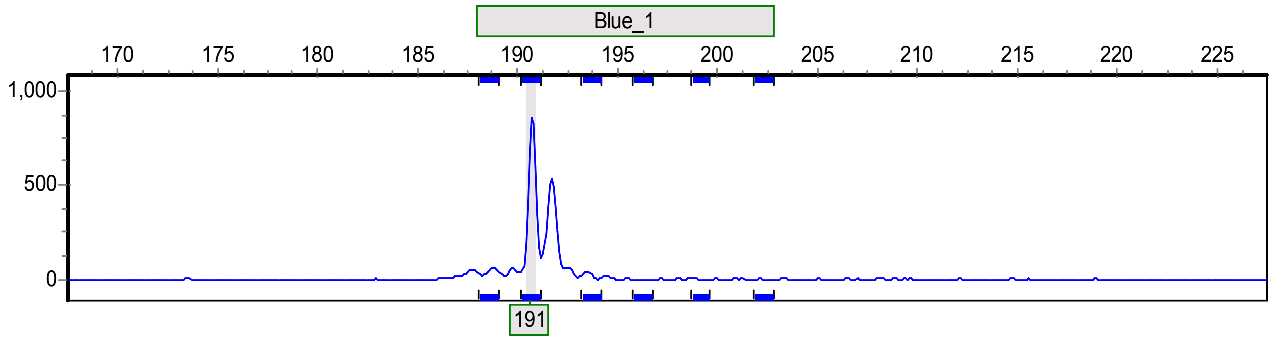

No	Size	Height	Area	Marker	Allele	Difference	Quality	Score	Allele Comments	Sample Comments
1	190.7	852	3934	Blue_1	191	0.0	Pass	182.1		

Sample 54: Run date and time: 09/15/2020 - 20:24:33 -> 09/15/2020 - 21:10:18

Dye: Blue - 1 peaks - 58.fsa

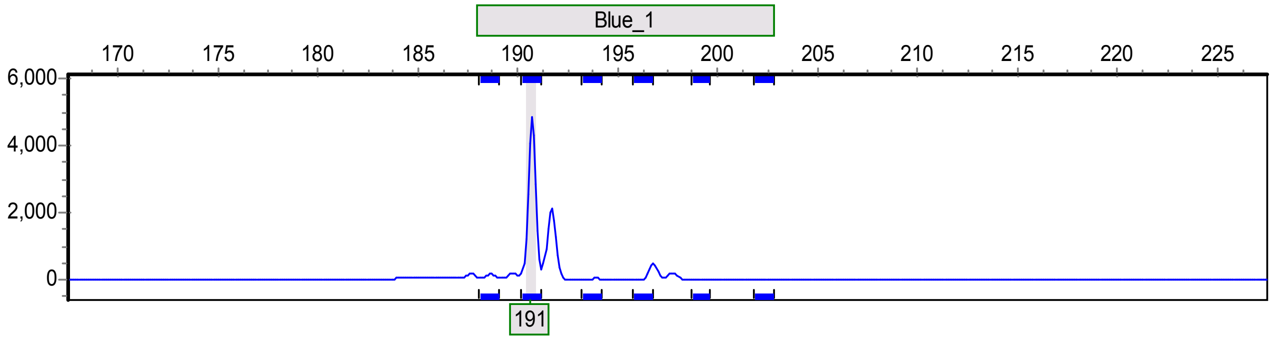

No	Size	Height	Area	Marker	Allele	Difference	Quality	Score	Allele Comments	Sample Comments
1	190.7	4812	20934	Blue_1	191	0.0	Pass	500.0		

Sample 55: Run date and time: 09/15/2020 - 20:24:33 -> 09/15/2020 - 21:10:18

Dye: Blue - 1 peaks - 59.fsa

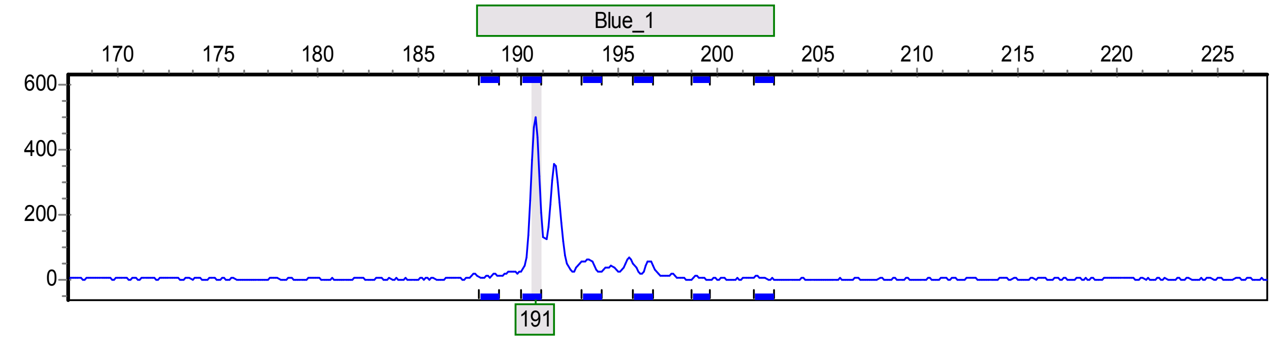

No	Size	Height	Area	Marker	Allele	Difference	Quality	Score	Allele Comments	Sample Comments
1	190.9	496	2790	Blue_1	191	0.2	Pass	59.4		

Sample 56: Run date and time: 09/15/2020 - 20:24:33 -> 09/15/2020 - 21:10:18

Dye: Blue - 1 peaks - 6.fsa

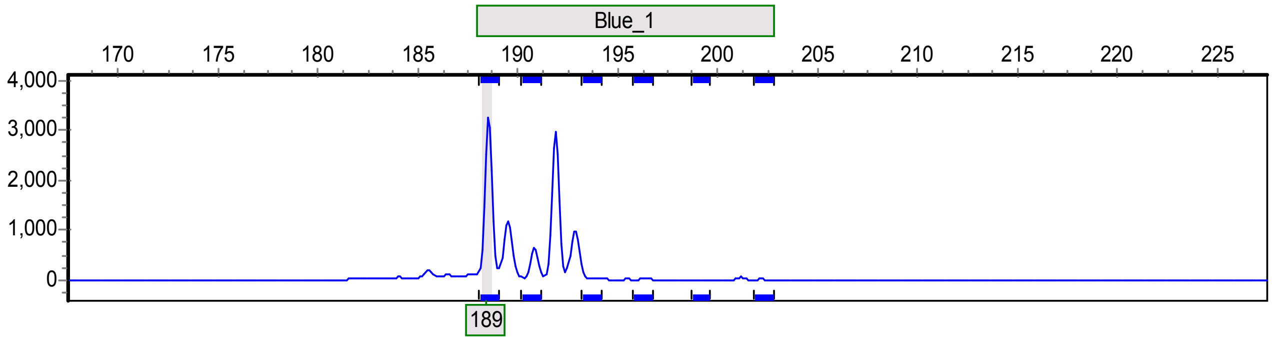

No	Size	Height	Area	Marker	Allele	Difference	Quality	Score	Allele Comments	Sample Comments
1	188.5	3241	14090	Blue_1	189	0.1	Pass	500.0		

Sample 57: Run date and time: 09/15/2020 - 20:24:33 -> 09/15/2020 - 21:10:18

Dye: Blue - 0 peaks - 60.fsa

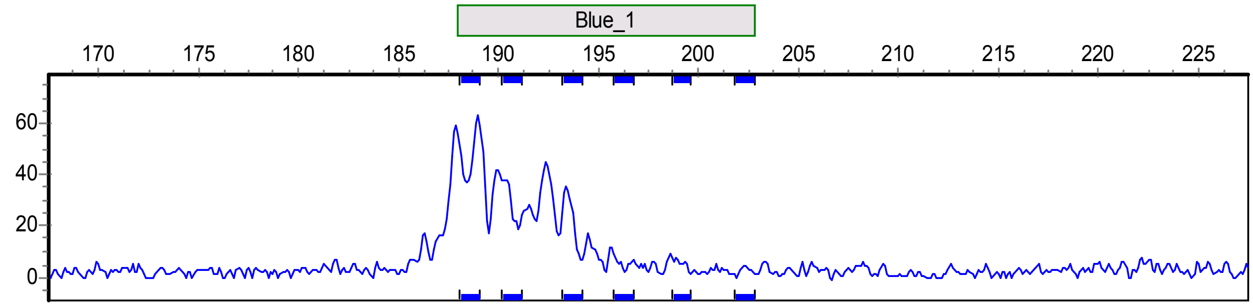

No	Size	Height	Area	Marker	Allele	Difference	Quality	Score	Allele Comments	Sample Comments

Allele Report

Sample 58: Run date and time: 09/15/2020 - 20:24:33 -> 09/15/2020 - 21:10:18

Dye: Blue - 1 peaks - 61.fsa

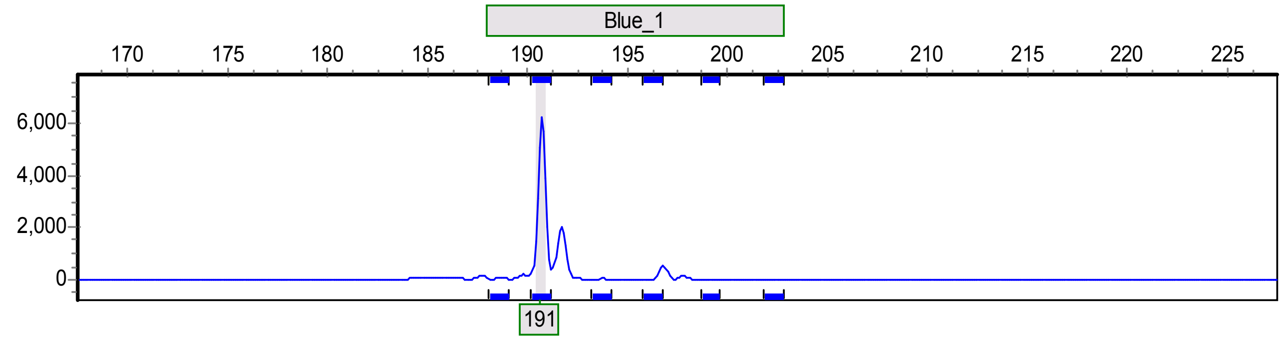

No	Size	Height	Area	Marker	Allele	Difference	Quality	Score	Allele Comments	Sample Comments
1	190.7	6181	27058	Blue_1	191	0.0	Pass	500.0		

Sample 59: Run date and time: 09/15/2020 - 20:24:33 -> 09/15/2020 - 21:10:18

Dye: Blue - 1 peaks - 62.fsa

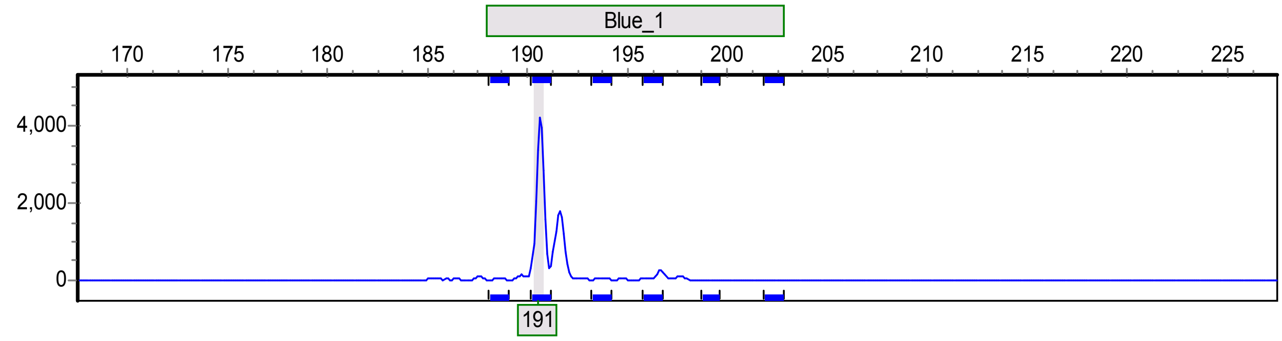

No	Size	Height	Area	Marker	Allele	Difference	Quality	Score	Allele Comments	Sample Comments
1	190.6	4168	18779	Blue_1	191	0.1	Pass	500.0		

Sample 60: Run date and time: 09/15/2020 - 20:24:33 -> 09/15/2020 - 21:10:18

Dye: Blue - 1 peaks - 63.fsa

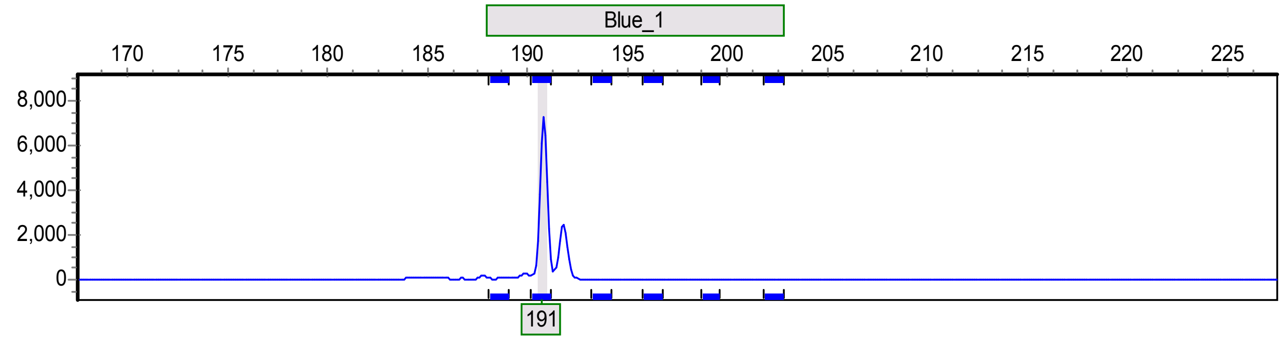

No	Size	Height	Area	Marker	Allele	Difference	Quality	Score	Allele Comments	Sample Comments
1	190.8	7263	31574	Blue_1	191	0.1	Pass	500.0		

Sample 61: Run date and time: 09/15/2020 - 20:24:33 -> 09/15/2020 - 21:10:18

Dye: Blue - 1 peaks - 64.fsa

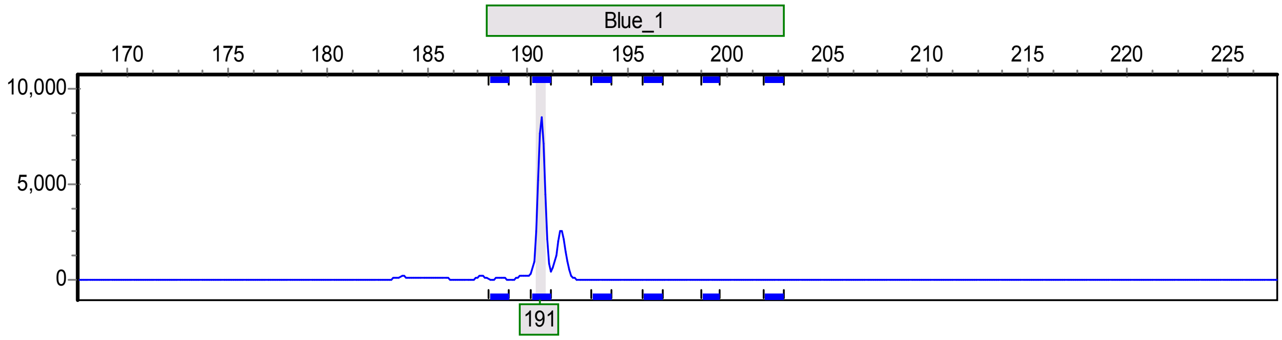

No	Size	Height	Area	Marker	Allele	Difference	Quality	Score	Allele Comments	Sample Comments
1	190.7	8501	37311	Blue_1	191	0.0	Pass	500.0		

Sample 62: Run date and time: 09/15/2020 - 20:24:33 -> 09/15/2020 - 21:10:18

Dye: Blue - 1 peaks - 65.fsa

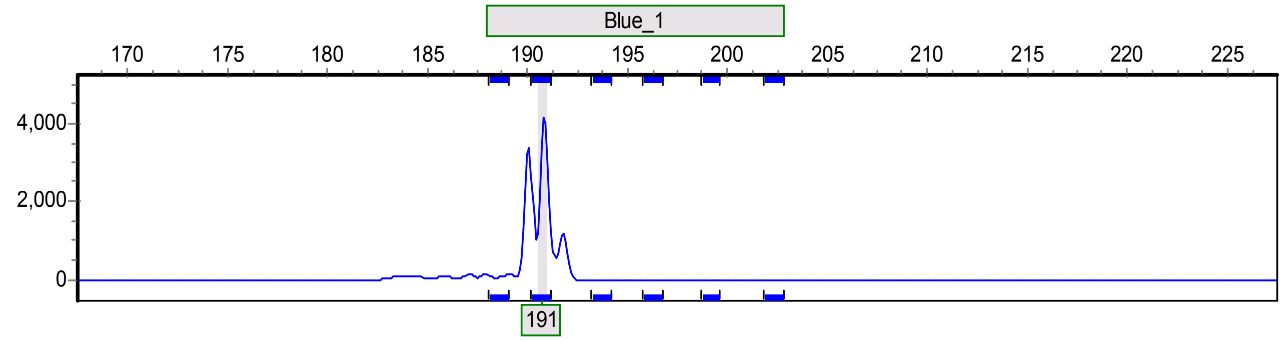

No	Size	Height	Area	Marker	Allele	Difference	Quality	Score	Allele Comments	Sample Comments
1	190.8	4118	21316	Blue_1	191	0.1	Pass	500.0		

Sample 63: Run date and time: 09/15/2020 - 20:24:33 -> 09/15/2020 - 21:10:18

Dye: Blue - 1 peaks - 66.fsa

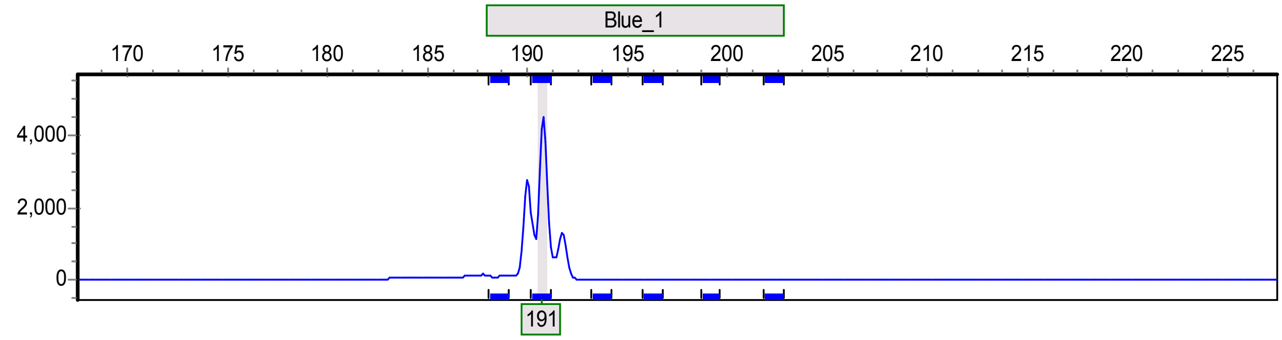

No	Size	Height	Area	Marker	Allele	Difference	Quality	Score	Allele Comments	Sample Comments
1	190.8	4492	23176	Blue_1	191	0.1	Pass	500.0		

Allele Report

Sample 64: Run date and time: 09/15/2020 - 20:24:33 -> 09/15/2020 - 21:10:18

Dye: Blue - 1 peaks - 67.fsa

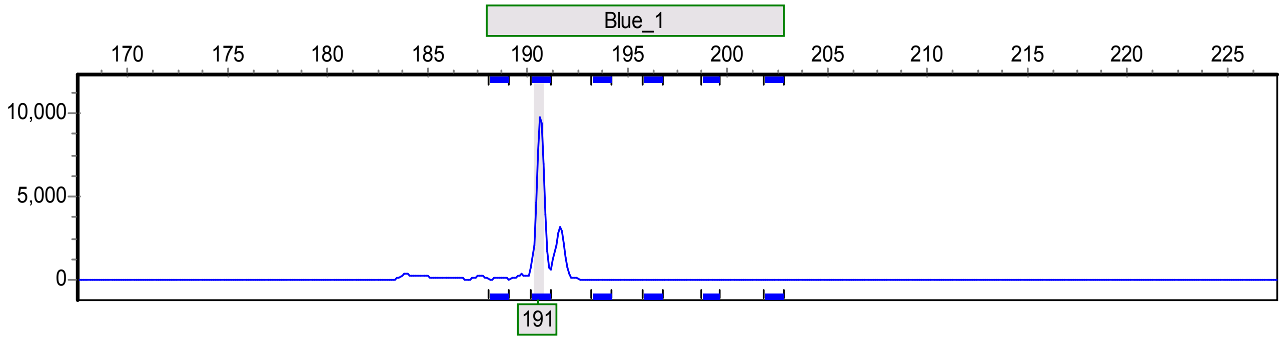

No	Size	Height	Area	Marker	Allele	Difference	Quality	Score	Allele Comments	Sample Comments
1	190.6	9653	43824	Blue_1	191	0.1	Pass	500.0		

Sample 65: Run date and time: 09/15/2020 - 20:24:33 -> 09/15/2020 - 21:10:18

Dye: Blue - 1 peaks - 68.fsa

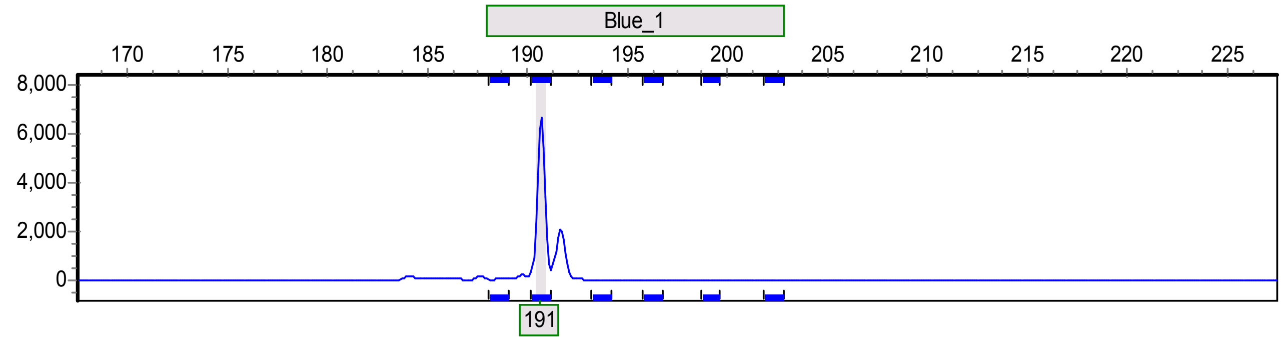

No	Size	Height	Area	Marker	Allele	Difference	Quality	Score	Allele Comments	Sample Comments
1	190.7	6625	29771	Blue_1	191	0.0	Pass	500.0		

Sample 66: Run date and time: 09/15/2020 - 20:24:33 -> 09/15/2020 - 21:10:18

Dye: Blue - 1 peaks - 69.fsa

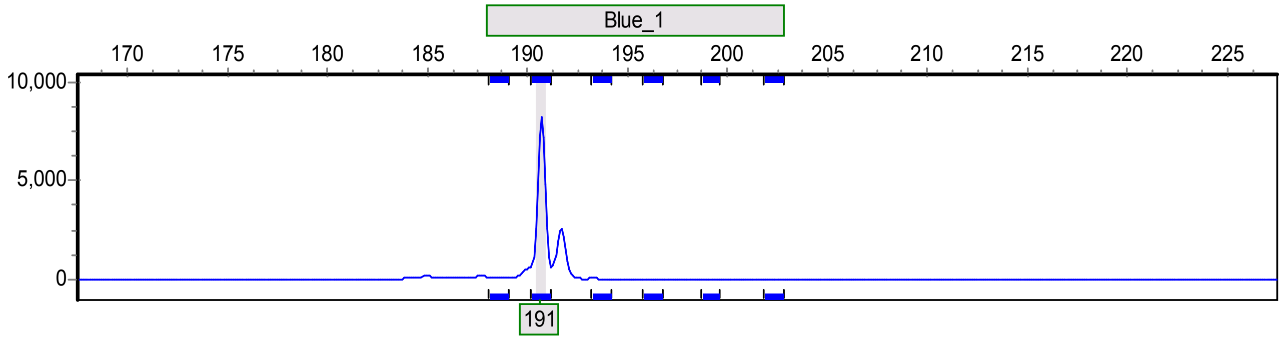

No	Size	Height	Area	Marker	Allele	Difference	Quality	Score	Allele Comments	Sample Comments
1	190.7	8188	37539	Blue_1	191	0.0	Pass	500.0		

Allele Report

Sample 67: Run date and time: 09/15/2020 - 20:24:33 -> 09/15/2020 - 21:10:18

Dye: Blue - 1 peaks - 7.fsa

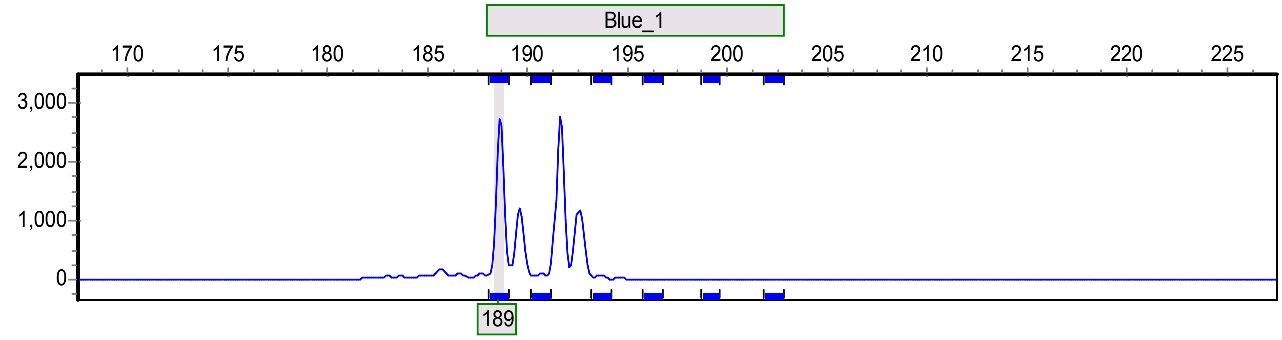

No	Size	Height	Area	Marker	Allele	Difference	Quality	Score	Allele Comments	Sample Comments
1	188.6	2739	12478	Blue_1	189	0.0	Pass	500.0		

Sample 68: Run date and time: 09/15/2020 - 20:24:33 -> 09/15/2020 - 21:10:18

Dye: Blue - 1 peaks - 70.fsa

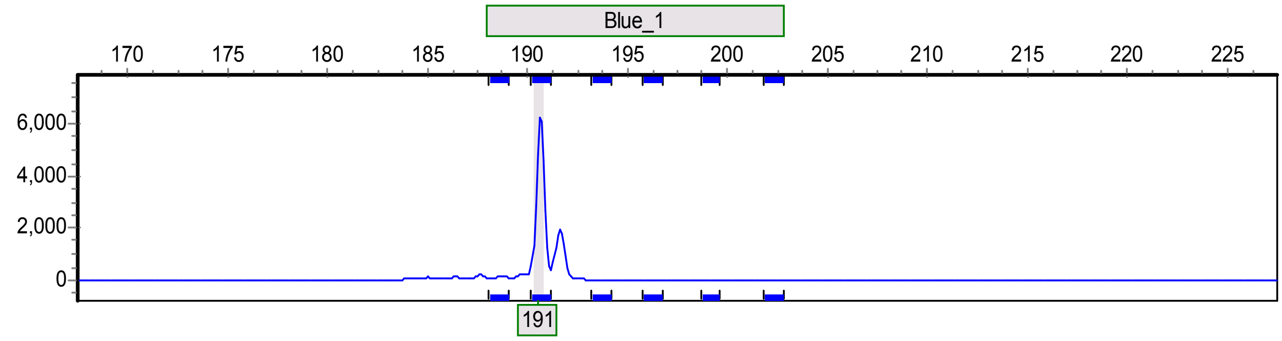

No	Size	Height	Area	Marker	Allele	Difference	Quality	Score	Allele Comments	Sample Comments
1	190.6	6174	28669	Blue_1	191	0.1	Pass	500.0		

Sample 69: Run date and time: 09/15/2020 - 20:24:33 -> 09/15/2020 - 21:10:18

Dye: Blue - 2 peaks - 71.fsa

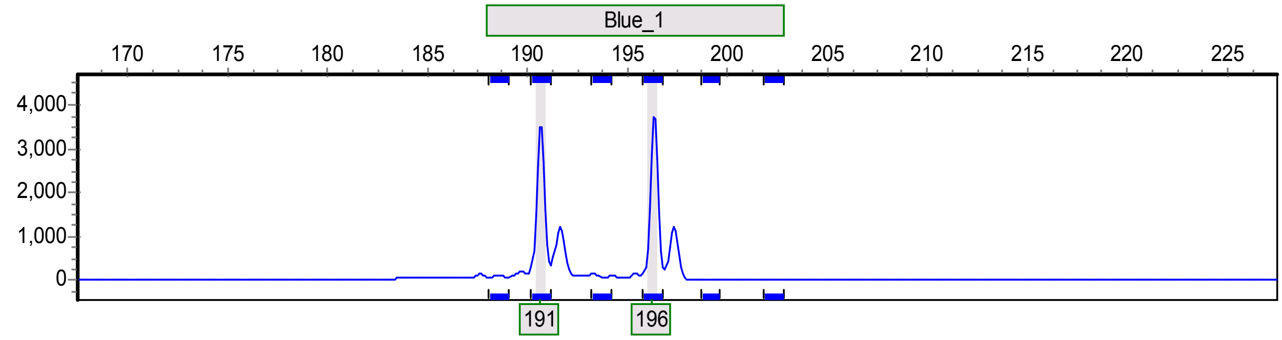

No	Size	Height	Area	Marker	Allele	Difference	Quality	Score	Allele Comments	Sample Comments
1	190.7	3522	16331	Blue_1	191	0.0	Pass	500.0		
2	196.3	3720	16802	Blue_1	196	0.0	Pass	500.0		

Allele Report

Sample 70: Run date and time: 09/15/2020 - 20:24:33 -> 09/15/2020 - 21:10:18

Dye: Blue - 2 peaks - 72.fsa

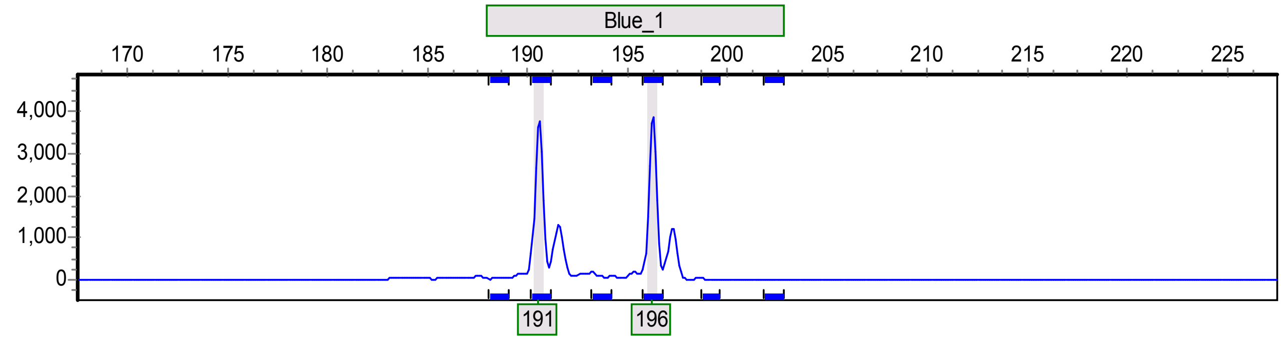

No	Size	Height	Area	Marker	Allele	Difference	Quality	Score	Allele Comments	Sample Comments
1	190.6	3775	17393	Blue_1	191	0.1	Pass	500.0		
2	196.3	3838	17525	Blue_1	196	0.0	Pass	500.0		

Sample 71: Run date and time: 09/15/2020 - 20:24:33 -> 09/15/2020 - 21:10:18

Dye: Blue - 1 peaks - 73.fsa

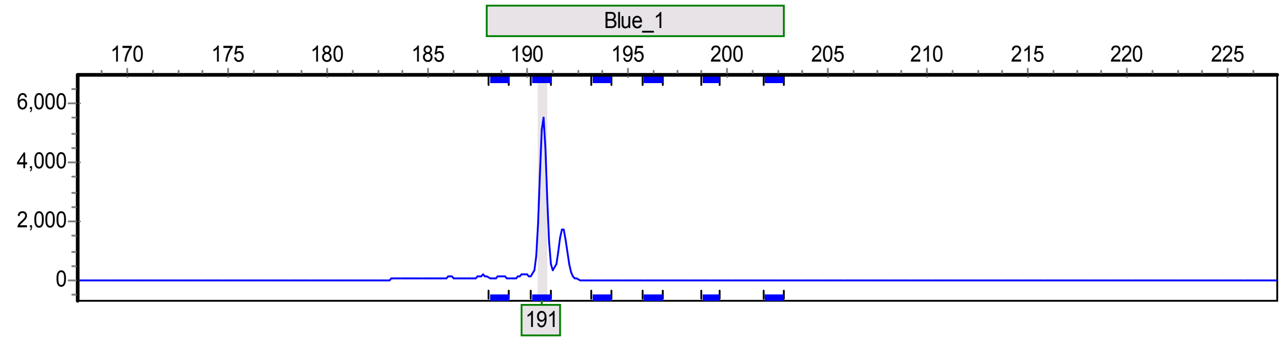

No	Size	Height	Area	Marker	Allele	Difference	Quality	Score	Allele Comments	Sample Comments
1	190.8	5495	24604	Blue_1	191	0.1	Pass	500.0		

Sample 72: Run date and time: 09/15/2020 - 20:24:33 -> 09/15/2020 - 21:10:18

Dye: Blue - 1 peaks - 74.fsa

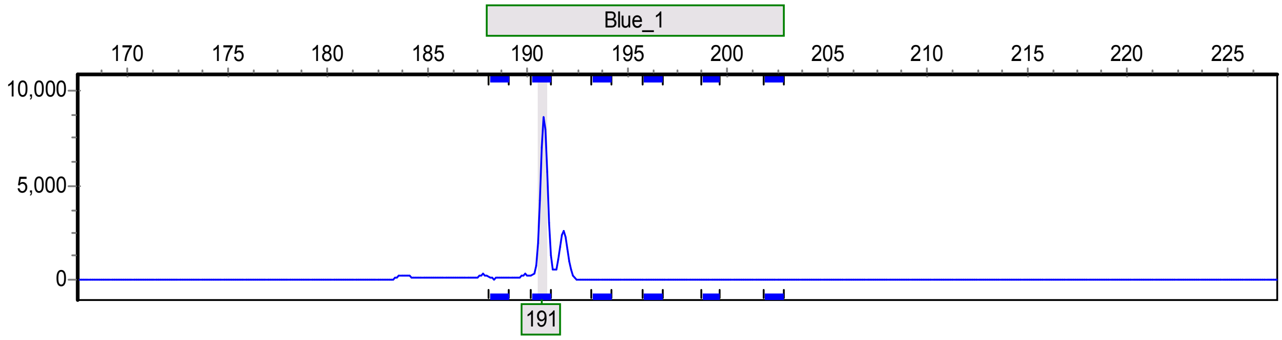

No	Size	Height	Area	Marker	Allele	Difference	Quality	Score	Allele Comments	Sample Comments
1	190.8	8591	38315	Blue_1	191	0.1	Pass	500.0		

Allele Report

Sample 73: Run date and time: 09/15/2020 - 20:24:33 -> 09/15/2020 - 21:10:18

Dye: Blue - 1 peaks - 75.fsa

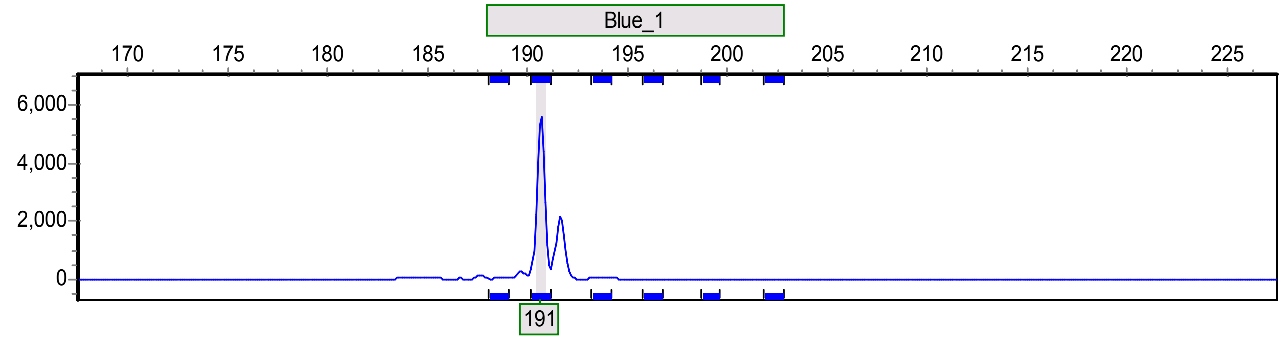

No	Size	Height	Area	Marker	Allele	Difference	Quality	Score	Allele Comments	Sample Comments
1	190.7	5539	25188	Blue_1	191	0.0	Pass	500.0		

Sample 74: Run date and time: 09/15/2020 - 20:24:33 -> 09/15/2020 - 21:10:18

Dye: Blue - 1 peaks - 76.fsa

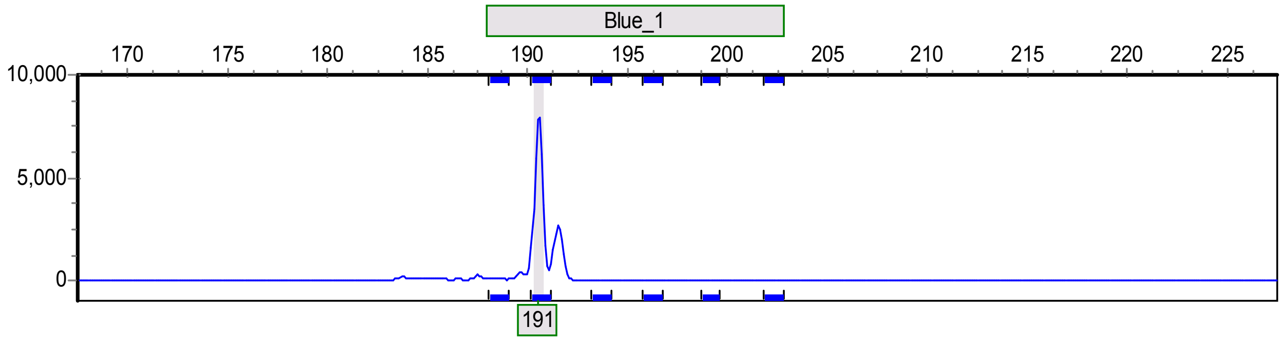

No	Size	Height	Area	Marker	Allele	Difference	Quality	Score	Allele Comments	Sample Comments
1	190.6	7883	36753	Blue_1	191	0.1	Pass	500.0		

Sample 75: Run date and time: 09/15/2020 - 20:24:33 -> 09/15/2020 - 21:10:18

Dye: Blue - 1 peaks - 77.fsa

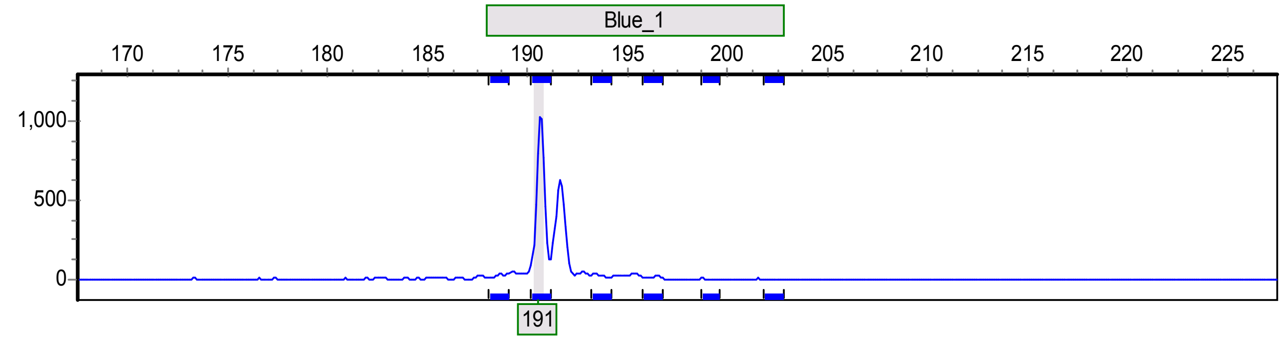

No	Size	Height	Area	Marker	Allele	Difference	Quality	Score	Allele Comments	Sample Comments
1	190.6	1014	4823	Blue_1	191	0.1	Pass	227.6		

Sample 76: Run date and time: 09/15/2020 - 20:24:33 -> 09/15/2020 - 21:10:18

Dye: Blue - 1 peaks - 78.fsa

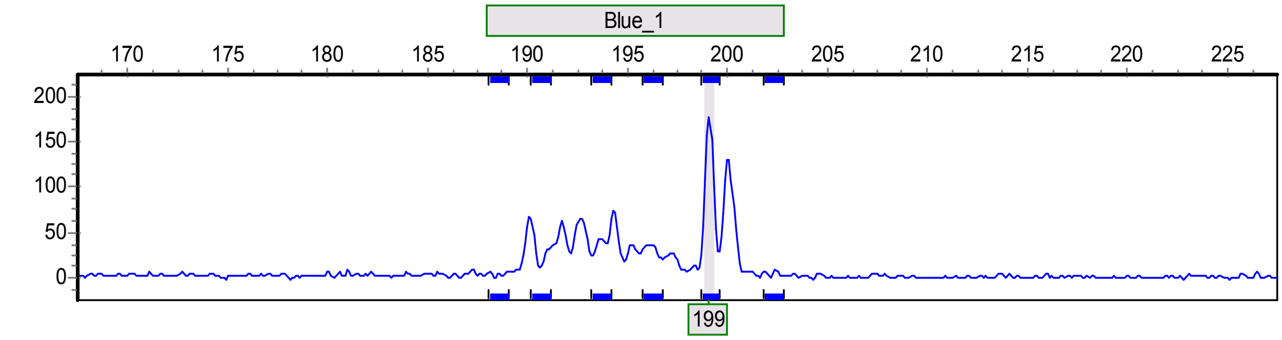

No	Size	Height	Area	Marker	Allele	Difference	Quality	Score	Allele Comments	Sample Comments
1	199.1	176	811	Blue_1	199	0.1	Pass	14.3		

Sample 77: Run date and time: 09/15/2020 - 20:24:33 -> 09/15/2020 - 21:10:18

Dye: Blue - 1 peaks - 79.fsa

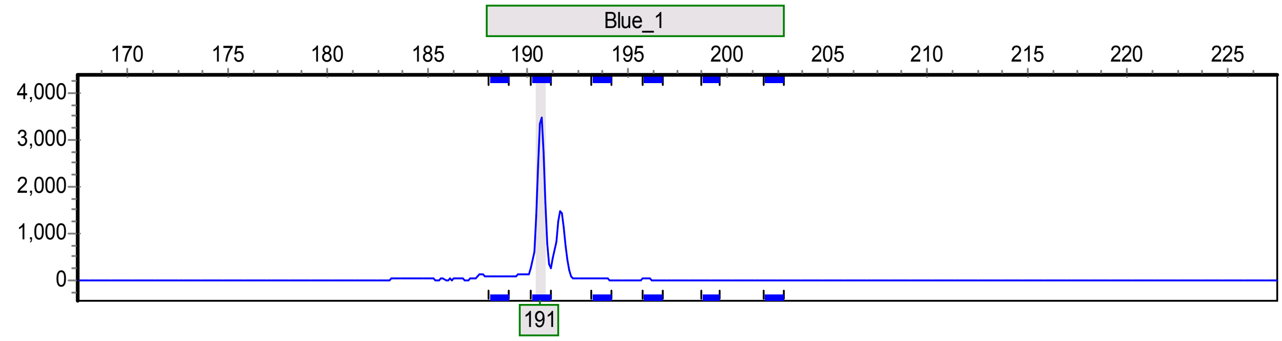

No	Size	Height	Area	Marker	Allele	Difference	Quality	Score	Allele Comments	Sample Comments
1	190.7	3455	15770	Blue_1	191	0.0	Pass	500.0		

Sample 78: Run date and time: 09/15/2020 - 20:24:33 -> 09/15/2020 - 21:10:18

Dye: Blue - 0 peaks - 8.fsa

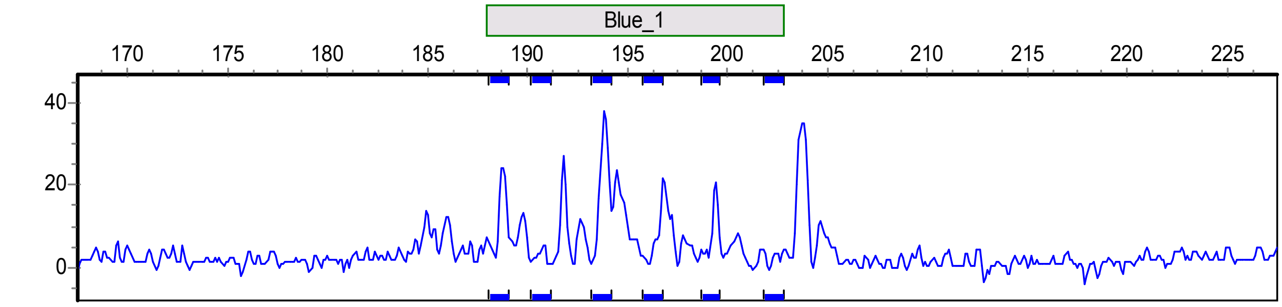

No	Size	Height	Area	Marker	Allele	Difference	Quality	Score	Allele Comments	Sample Comments

Sample 79: Run date and time: 09/15/2020 - 20:24:33 -> 09/15/2020 - 21:10:18

Dye: Blue - 1 peaks - 80.fsa

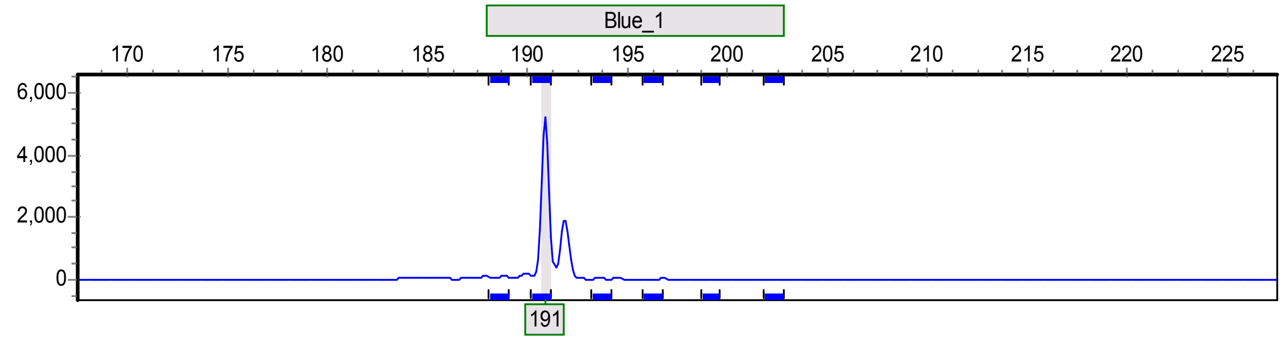

No	Size	Height	Area	Marker	Allele	Difference	Quality	Score	Allele Comments	Sample Comments
1	190.9	5198	23081	Blue_1	191	0.2	Pass	500.0		

Sample 80: Run date and time: 09/15/2020 - 20:24:33 -> 09/15/2020 - 21:10:18

Dye: Blue - 1 peaks - 81.fsa

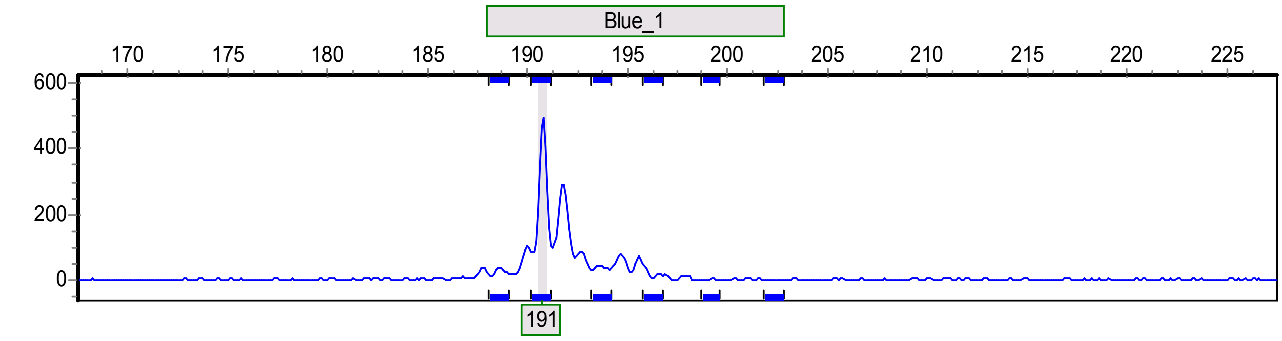

No	Size	Height	Area	Marker	Allele	Difference	Quality	Score	Allele Comments	Sample Comments
1	190.8	493	2459	Blue_1	191	0.1	Pass	68.6		

Sample 81: Run date and time: 09/15/2020 - 20:24:33 -> 09/15/2020 - 21:10:18

Dye: Blue - 1 peaks - 82.fsa

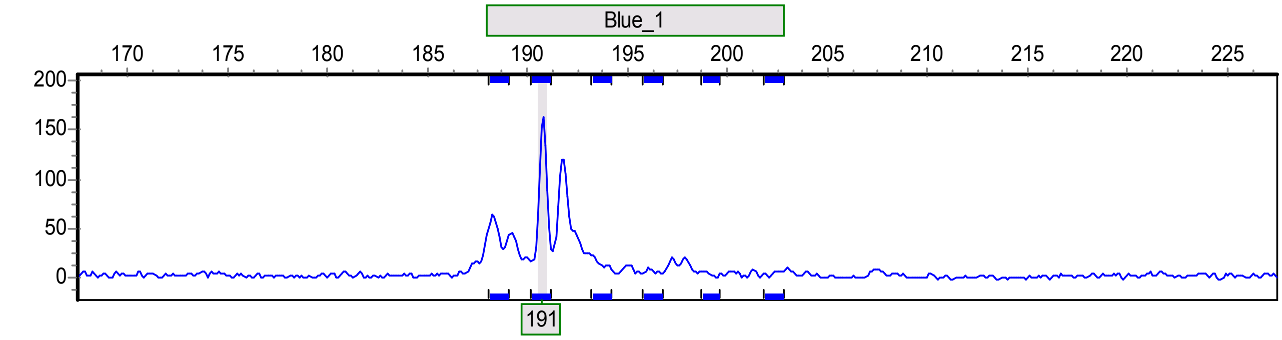

No	Size	Height	Area	Marker	Allele	Difference	Quality	Score	Allele Comments	Sample Comments
1	190.8	163	781	Blue_1	191	0.1	Pass	11.7		

Sample 82: Run date and time: 09/15/2020 - 20:24:33 -> 09/15/2020 - 21:10:18

Dye: Blue - 1 peaks - 83.fsa

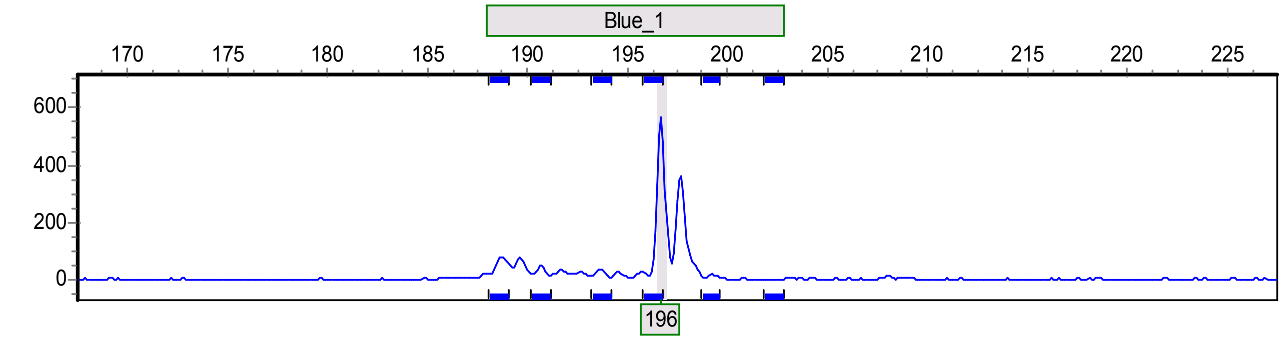

No	Size	Height	Area	Marker	Allele	Difference	Quality	Score	Allele Comments	Sample Comments
1	196.7	564	2498	Blue_1	196	0.4	Pass	103.6		

Sample 83: Run date and time: 09/15/2020 - 20:24:33 -> 09/15/2020 - 21:10:18

Dye: Blue - 1 peaks - 84.fsa

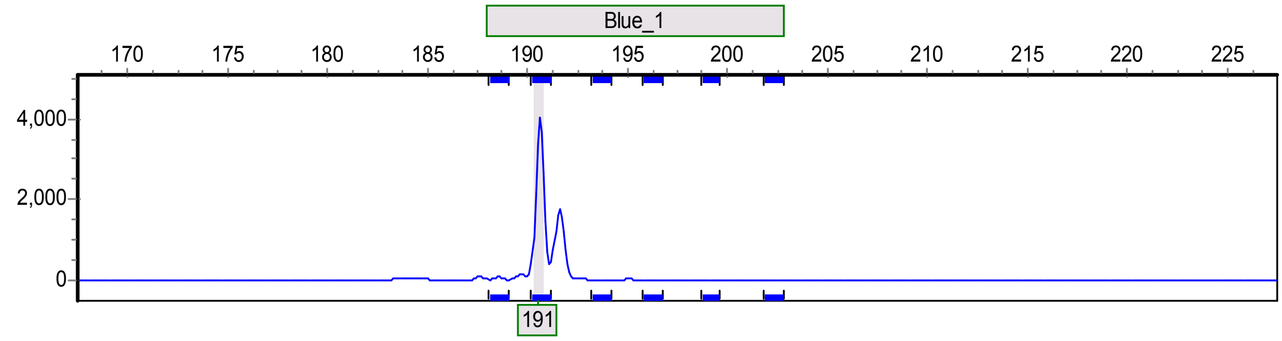

No	Size	Height	Area	Marker	Allele	Difference	Quality	Score	Allele Comments	Sample Comments
1	190.6	4012	18561	Blue_1	191	0.1	Pass	500.0		

Sample 84: Run date and time: 09/15/2020 - 20:24:33 -> 09/15/2020 - 21:10:18

Dye: Blue - 1 peaks - 85.fsa

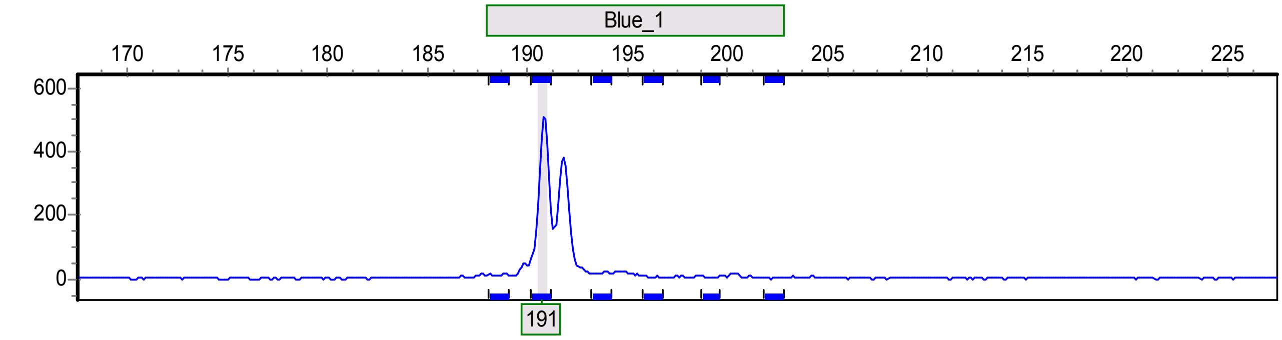

No	Size	Height	Area	Marker	Allele	Difference	Quality	Score	Allele Comments	Sample Comments
1	190.8	506	3319	Blue_1	191	0.1	Pass	48.6		

Allele Report

Sample 85: Run date and time: 09/15/2020 - 20:24:33 -> 09/15/2020 - 21:10:18

Dye: Blue - 1 peaks - 86.fsa

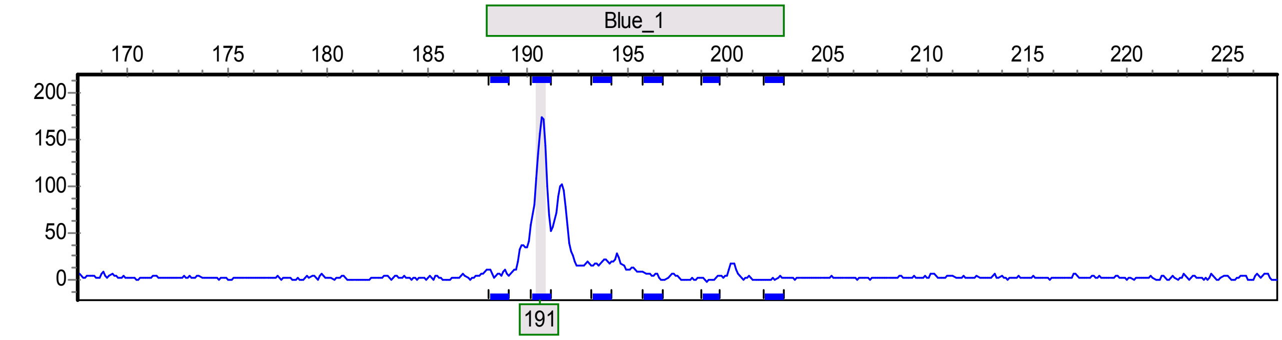

No	Size	Height	Area	Marker	Allele	Difference	Quality	Score	Allele Comments	Sample Comments
1	190.7	174	1184	Blue_1	191	0.0	Pass	6.0	[<Confirmed>]	

Sample 86: Run date and time: 09/15/2020 - 20:24:33 -> 09/15/2020 - 21:10:18

Dye: Blue - 1 peaks - 87.fsa

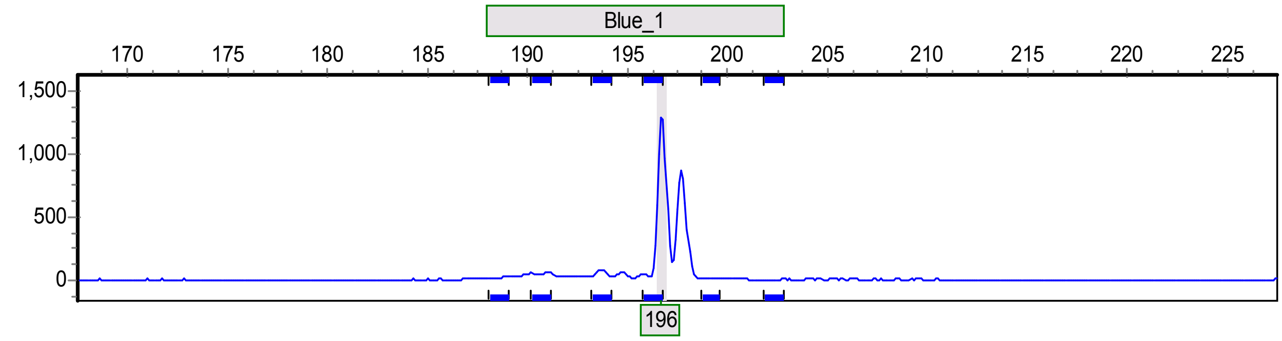

No	Size	Height	Area	Marker	Allele	Difference	Quality	Score	Allele Comments	Sample Comments
1	196.7	1278	5970	Blue_1	196	0.4	Pass	326.1		

Sample 87: Run date and time: 09/15/2020 - 20:24:33 -> 09/15/2020 - 21:10:18

Dye: Blue - 1 peaks - 88.fsa

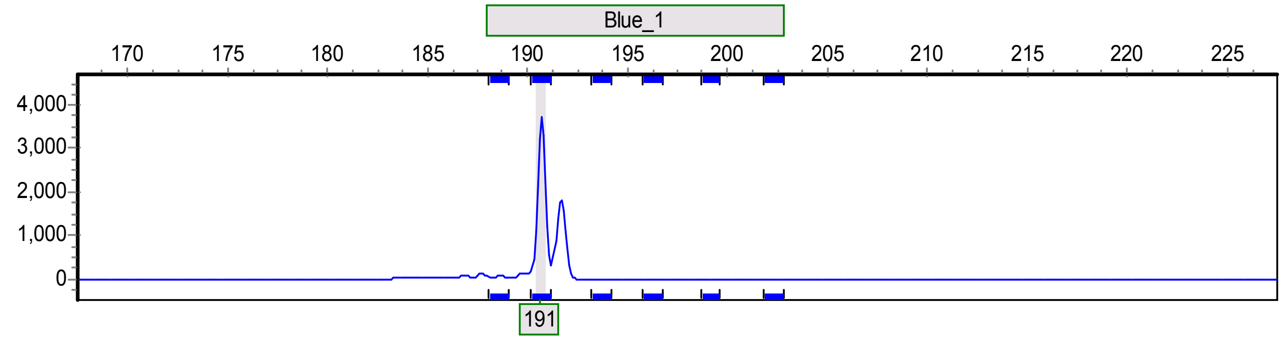

No	Size	Height	Area	Marker	Allele	Difference	Quality	Score	Allele Comments	Sample Comments
1	190.7	3680	16878	Blue_1	191	0.0	Pass	500.0		

Allele Report

Sample 88: Run date and time: 09/15/2020 - 20:24:33 -> 09/15/2020 - 21:10:18

Dye: Blue - 2 peaks - 89.fsa

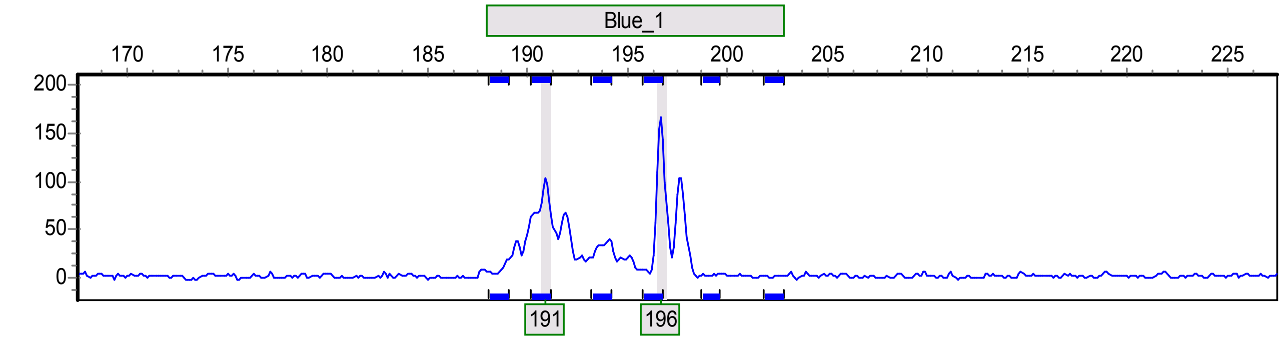

No	Size	Height	Area	Marker	Allele	Difference	Quality	Score	Allele Comments	Sample Comments
1	190.9	102	633	Blue_1	191	0.2	Pass	1.6	[<Confirmed>]	
2	196.7	166	801	Blue_1	196	0.4	Pass	12.1		

Sample 89: Run date and time: 09/15/2020 - 20:24:33 -> 09/15/2020 - 21:10:18

Dye: Blue - 1 peaks - 9.fsa

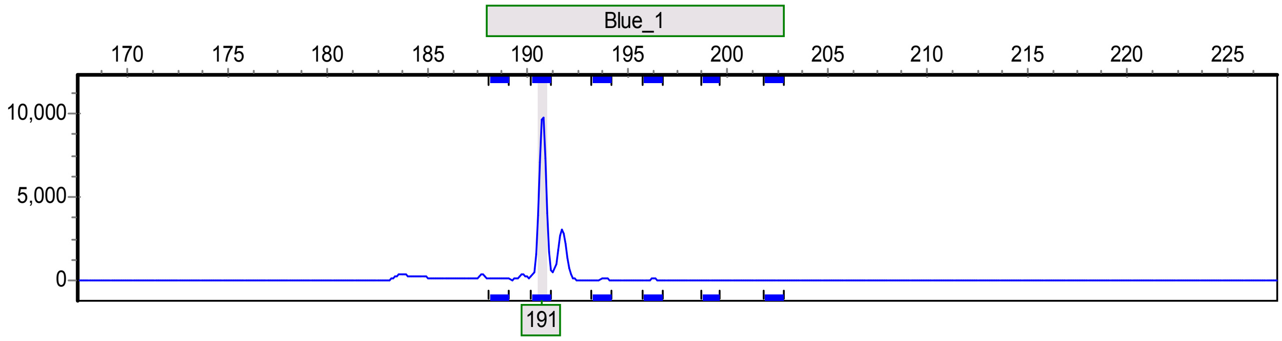

No	Size	Height	Area	Marker	Allele	Difference	Quality	Score	Allele Comments	Sample Comments
1	190.8	9699	42737	Blue_1	191	0.1	Pass	500.0		

Sample 90: Run date and time: 09/15/2020 - 20:24:33 -> 09/15/2020 - 21:10:18

Dye: Blue - 1 peaks - 90.fsa

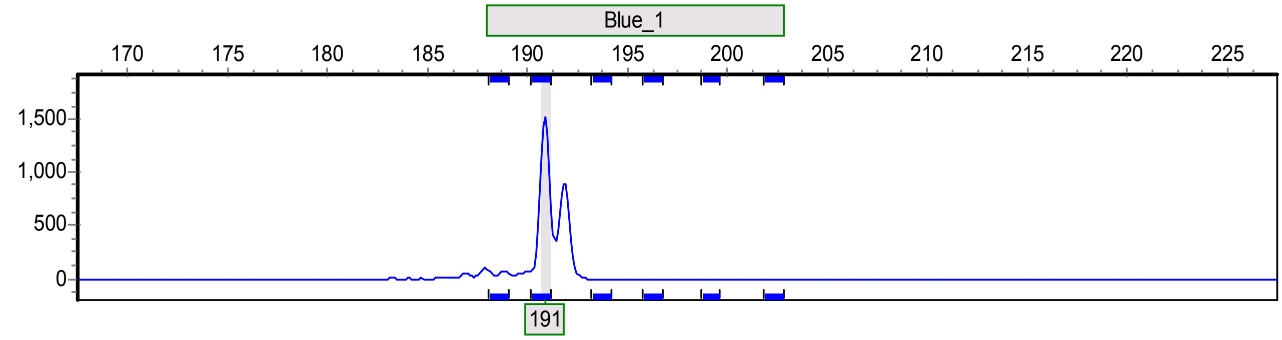

No	Size	Height	Area	Marker	Allele	Difference	Quality	Score	Allele Comments	Sample Comments
1	190.9	1501	8981	Blue_1	191	0.2	Pass	279.4		

Sample 91: Run date and time: 09/15/2020 - 20:24:33 -> 09/15/2020 - 21:10:18

Dye: Blue - 1 peaks - 91.fsa

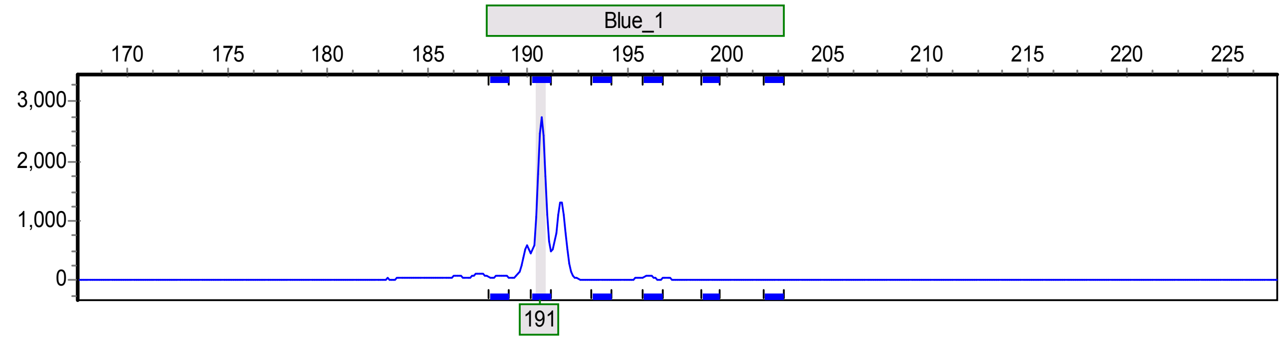

No	Size	Height	Area	Marker	Allele	Difference	Quality	Score	Allele Comments	Sample Comments
1	190.7	2724	14349	Blue_1	191	0.0	Pass	500.0		

Sample 92: Run date and time: 09/15/2020 - 20:24:33 -> 09/15/2020 - 21:10:18

Dye: Blue - 1 peaks - 92.fsa

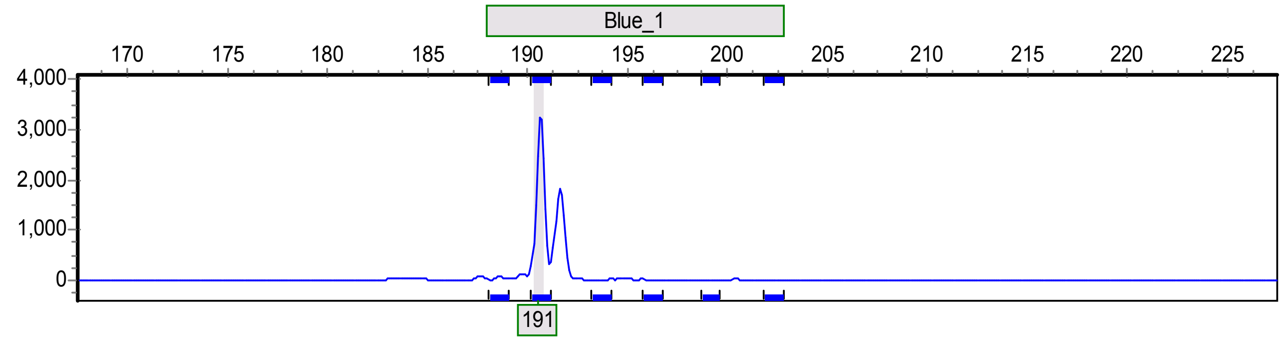

No	Size	Height	Area	Marker	Allele	Difference	Quality	Score	Allele Comments	Sample Comments
1	190.6	3230	15152	Blue_1	191	0.1	Pass	500.0		

Sample 93: Run date and time: 09/15/2020 - 20:24:33 -> 09/15/2020 - 21:10:18

Dye: Blue - 1 peaks - 93.fsa

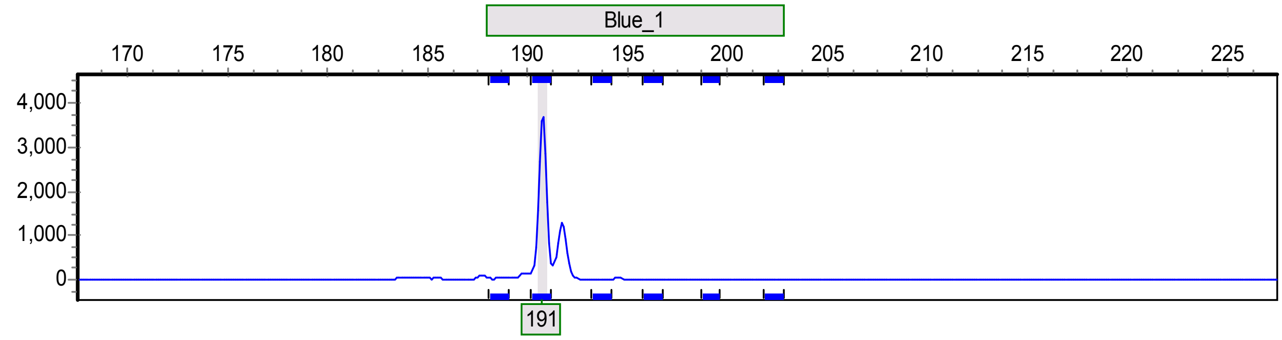

No	Size	Height	Area	Marker	Allele	Difference	Quality	Score	Allele Comments	Sample Comments
1	190.8	3675	17109	Blue_1	191	0.1	Pass	500.0		

Allele Report

Sample 94: Run date and time: 09/15/2020 - 20:24:33 -> 09/15/2020 - 21:10:18

Dye: Blue - 1 peaks - 94.fsa

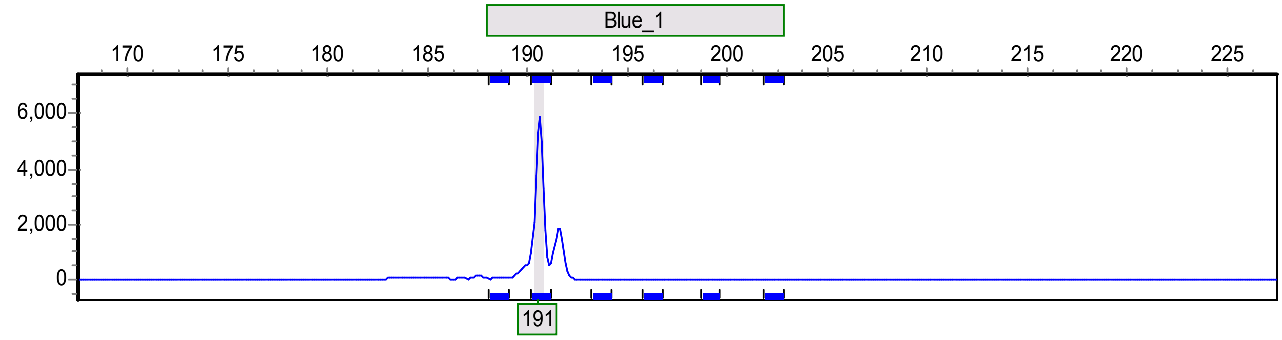

No	Size	Height	Area	Marker	Allele	Difference	Quality	Score	Allele Comments	Sample Comments
1	190.6	5835	27299	Blue_1	191	0.1	Pass	500.0		

Sample 95: Run date and time: 09/15/2020 - 20:24:33 -> 09/15/2020 - 21:10:18

Dye: Blue - 1 peaks - 95.fsa

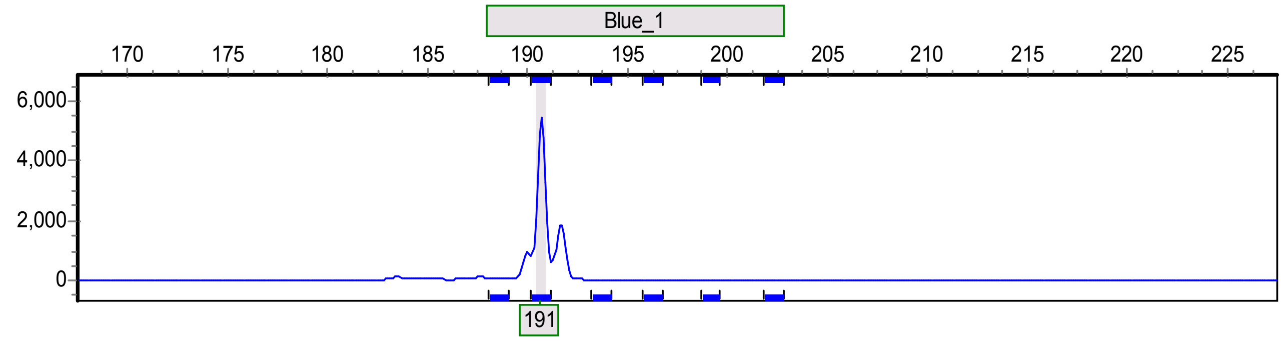

No	Size	Height	Area	Marker	Allele	Difference	Quality	Score	Allele Comments	Sample Comments
1	190.7	5411	26729	Blue_1	191	0.0	Pass	500.0		

Sample 96: Run date and time: 09/15/2020 - 20:24:33 -> 09/15/2020 - 21:10:18

Dye: Blue - 1 peaks - 96.fsa

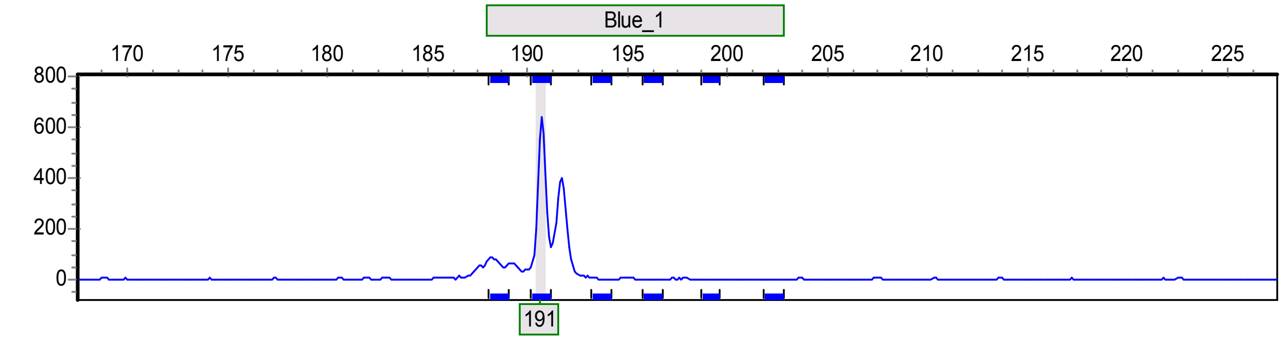

No	Size	Height	Area	Marker	Allele	Difference	Quality	Score	Allele Comments	Sample Comments
1	190.7	638	3278	Blue_1	191	0.0	Pass	105.9		
