# Supplementary material for: Genetic diversity and population structure of Polygonatum cyrtonema Hua in China using SSR markers
Source: PLoS One. 2023 Aug 31;18(8):e0290605. doi: 10.1371/journal.pone.0290605 (PMC10470896; doi:10.1371/journal.pone.0290605)
Supplement: S1 Data — (ZIP) [file pone.0290605.s002.zip › ╨┬╜¿╬─╝■╝╨ (2)/╥2╬∩34/yinwu34.pdf]

Allele Report

Sample 1: Run date and time: 09/15/2020 - 21:11:04 -> 09/15/2020 - 21:56:47

Dye: Blue - 2 peaks - 1.fsa

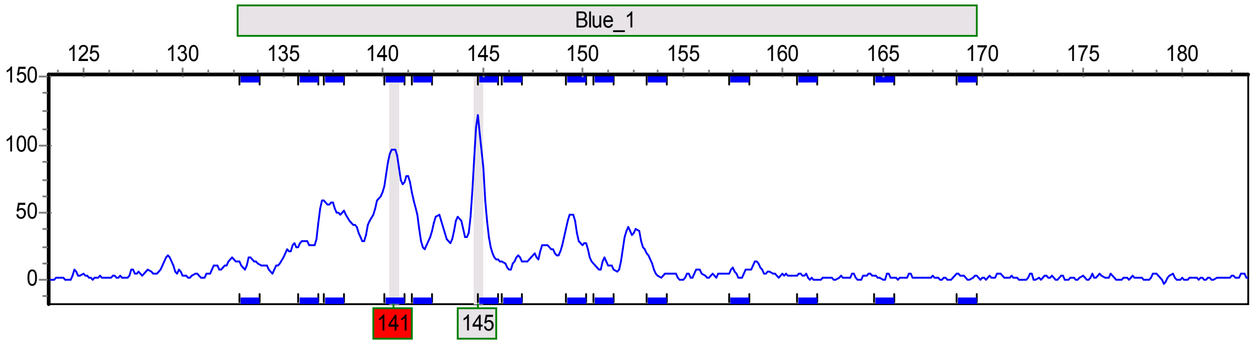

No	Size	Height	Area	Marker	Allele	Difference	Quality	Score	Allele Comments	Sample Comments
1	140.6	97	832	Blue_1	141	0.0	Undetermined	0.4		
2	144.8	122	725	Blue_1	145	0.5	Pass	4.4	[<Confirmed>]	

Sample 2: Run date and time: 09/15/2020 - 21:11:04 -> 09/15/2020 - 21:56:47

Dye: Blue - 0 peaks - 10.fsa

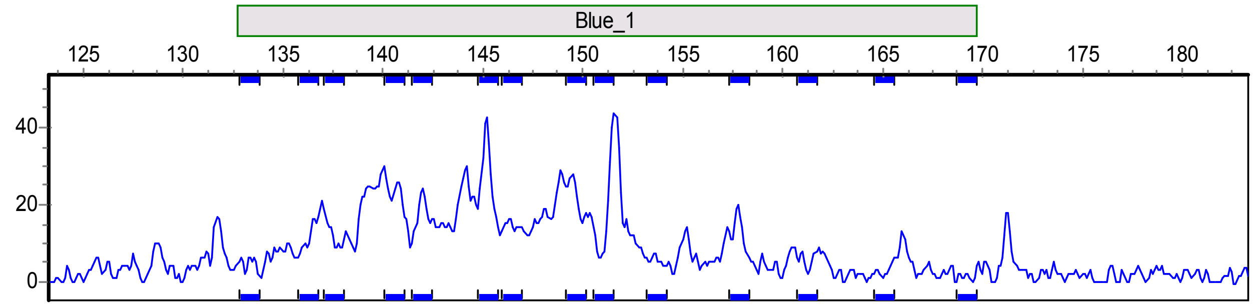

No	Size	Height	Area	Marker	Allele	Difference	Quality	Score	Allele Comments	Sample Comments

Sample 3: Run date and time: 09/15/2020 - 21:11:04 -> 09/15/2020 - 21:56:47

Dye: Blue - 0 peaks - 11.fsa

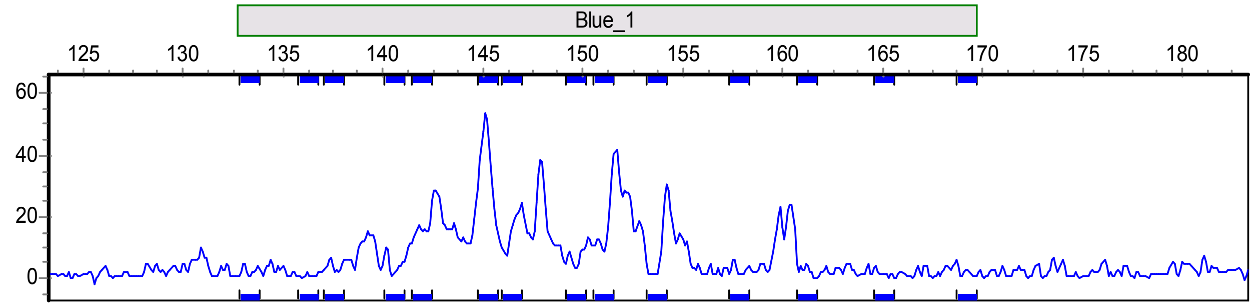

No	Size	Height	Area	Marker	Allele	Difference	Quality	Score	Allele Comments	Sample Comments

Sample 4: Run date and time: 09/15/2020 - 21:11:04 -> 09/15/2020 - 21:56:47

Dye: Blue - 0 peaks - 12.fsa

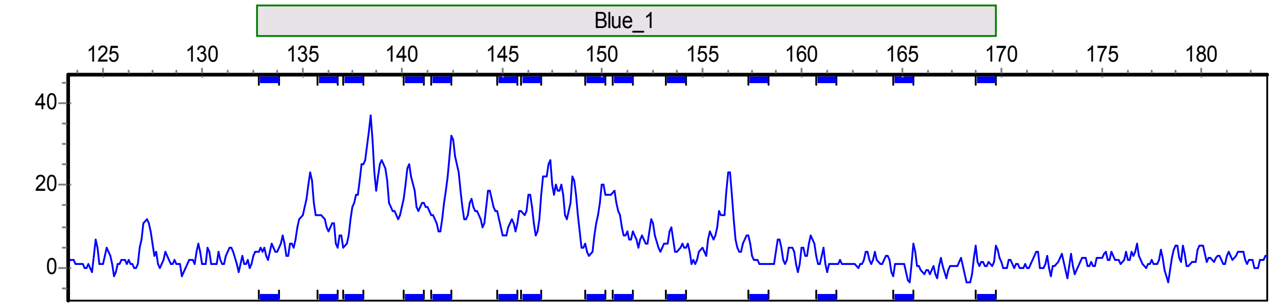

No	Size	Height	Area	Marker	Allele	Difference	Quality	Score	Allele Comments	Sample Comments

Sample 5: Run date and time: 09/15/2020 - 21:11:04 -> 09/15/2020 - 21:56:47

Dye: Blue - 1 peaks - 13.fsa

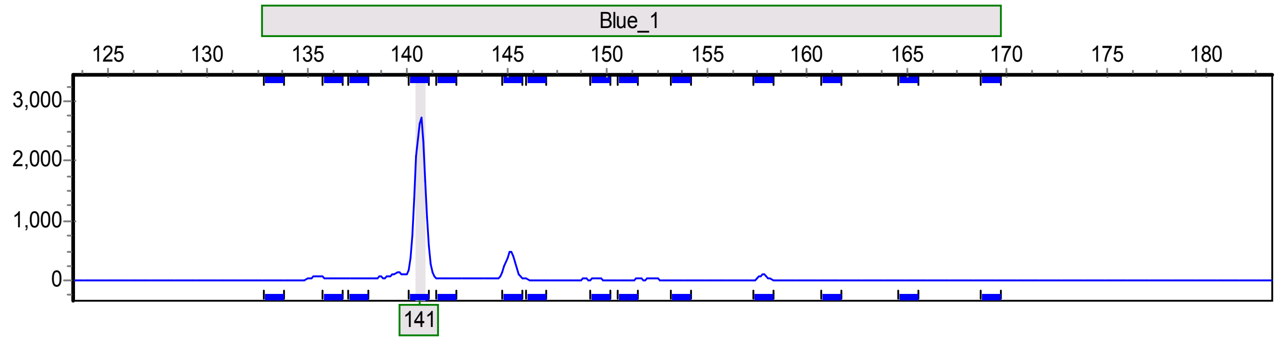

No	Size	Height	Area	Marker	Allele	Difference	Quality	Score	Allele Comments	Sample Comments
1	140.7	2699	14821	Blue_1	141	0.1	Pass	500.0		

Sample 6: Run date and time: 09/15/2020 - 21:11:04 -> 09/15/2020 - 21:56:47

Dye: Blue - 2 peaks - 14.fsa

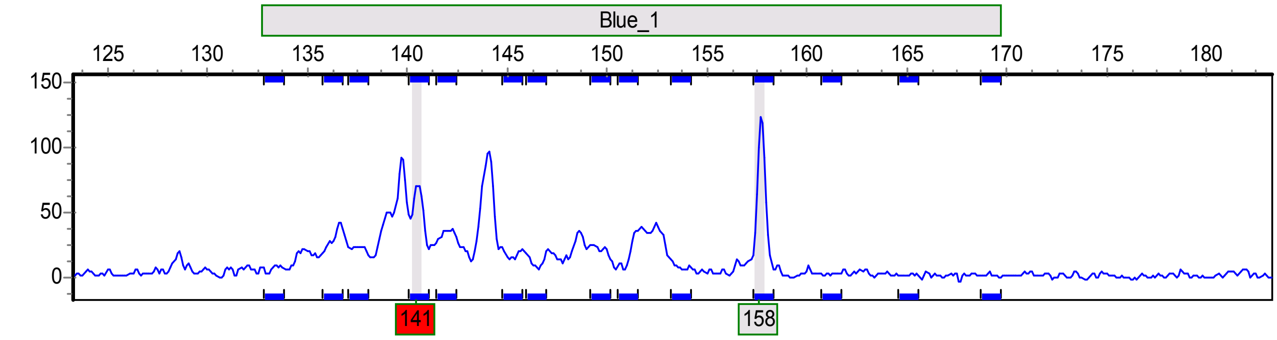

No	Size	Height	Area	Marker	Allele	Difference	Quality	Score	Allele Comments	Sample Comments
1	140.5	71	473	Blue_1	141	0.1	Undetermined	0.7		
2	157.7	124	625	Blue_1	158	0.2	Pass	6.6	[<Confirmed>]	

Sample 7: Run date and time: 09/15/2020 - 21:11:04 -> 09/15/2020 - 21:56:47

Dye: Blue - 1 peaks - 15.fsa

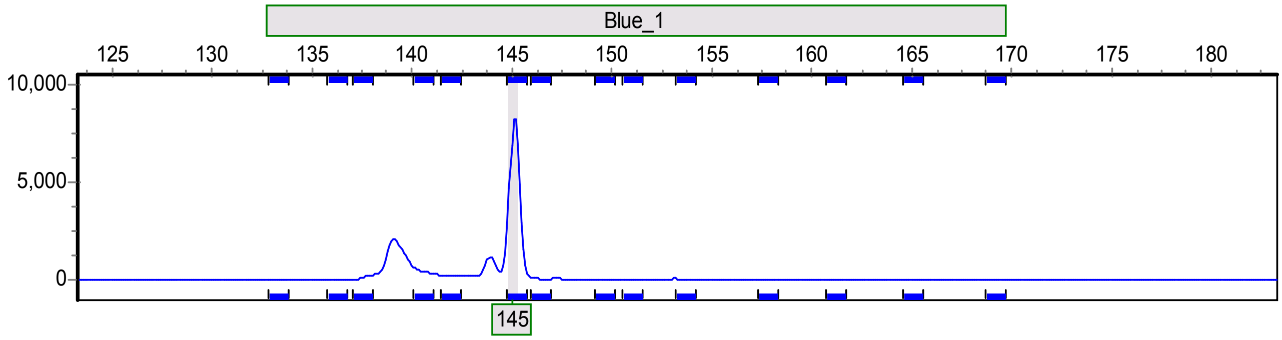

No	Size	Height	Area	Marker	Allele	Difference	Quality	Score	Allele Comments	Sample Comments
1	145.1	8281	46583	Blue_1	145	0.2	Pass	500.0		

Sample 8: Run date and time: 09/15/2020 - 21:11:04 -> 09/15/2020 - 21:56:47

Dye: Blue - 2 peaks - 16.fsa

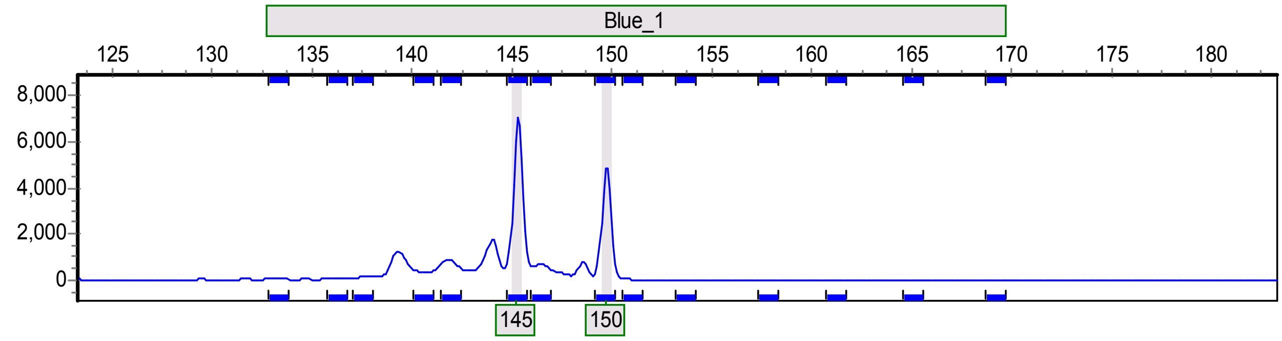

No	Size	Height	Area	Marker	Allele	Difference	Quality	Score	Allele Comments	Sample Comments
1	145.3	7016	38258	Blue_1	145	0.0	Pass	500.0		
2	149.8	4881	25486	Blue_1	150	0.1	Pass	500.0		

Sample 9: Run date and time: 09/15/2020 - 21:11:04 -> 09/15/2020 - 21:56:47

Dye: Blue - 2 peaks - 17.fsa

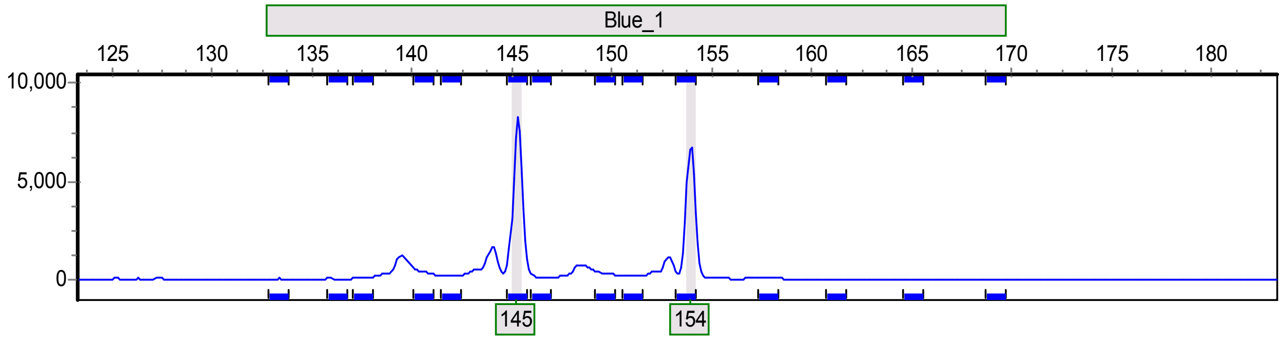

No	Size	Height	Area	Marker	Allele	Difference	Quality	Score	Allele Comments	Sample Comments
1	145.3	8196	42762	Blue_1	145	0.0	Pass	500.0		
2	154.0	6707	32462	Blue_1	154	0.2	Pass	500.0		

Allele Report

Sample 10: Run date and time: 09/15/2020 - 21:11:04 -> 09/15/2020 - 21:56:47

Dye: Blue - 1 peaks - 18.fsa

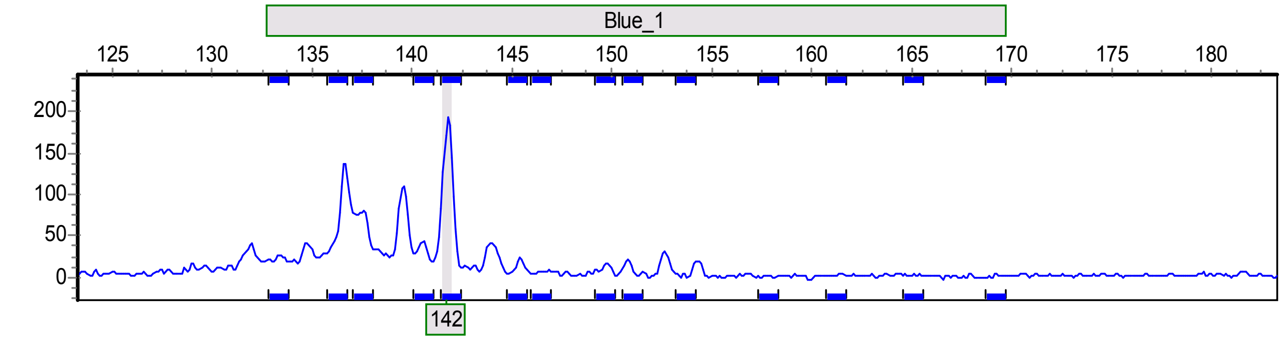

No	Size	Height	Area	Marker	Allele	Difference	Quality	Score	Allele Comments	Sample Comments
1	141.8	193	1106	Blue_1	142	0.2	Pass	11.9		

Sample 11: Run date and time: 09/15/2020 - 21:11:04 -> 09/15/2020 - 21:56:47

Dye: Blue - 1 peaks - 19.fsa

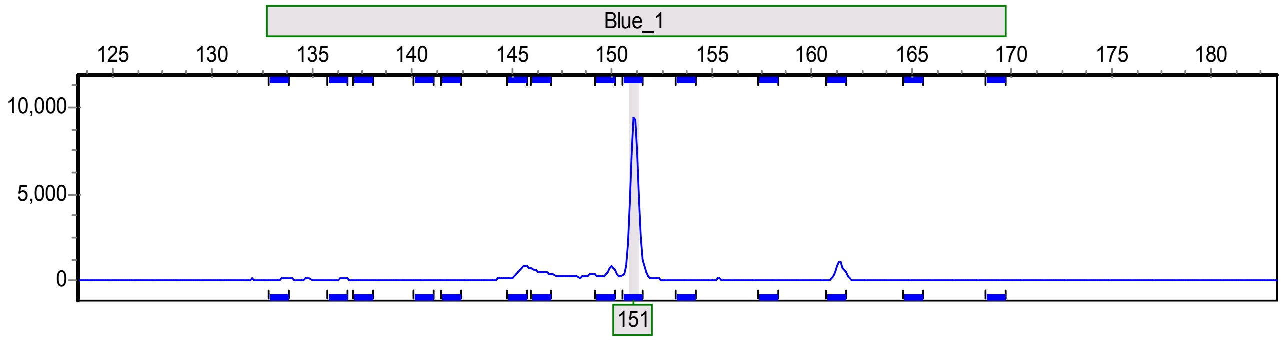

No	Size	Height	Area	Marker	Allele	Difference	Quality	Score	Allele Comments	Sample Comments
1	151.1	9373	46182	Blue_1	151	0.0	Pass	500.0		

Sample 12: Run date and time: 09/15/2020 - 21:11:04 -> 09/15/2020 - 21:56:47

Dye: Blue - 1 peaks - 2.fsa

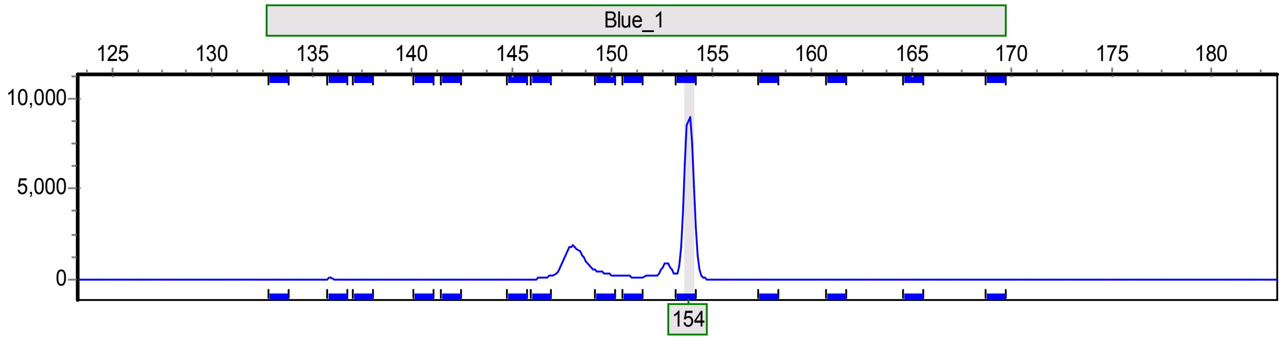

No	Size	Height	Area	Marker	Allele	Difference	Quality	Score	Allele Comments	Sample Comments
1	153.9	8886	43965	Blue_1	154	0.1	Pass	500.0		

Sample 13: Run date and time: 09/15/2020 - 21:11:04 -> 09/15/2020 - 21:56:47

Dye: Blue - 1 peaks - 20.fsa

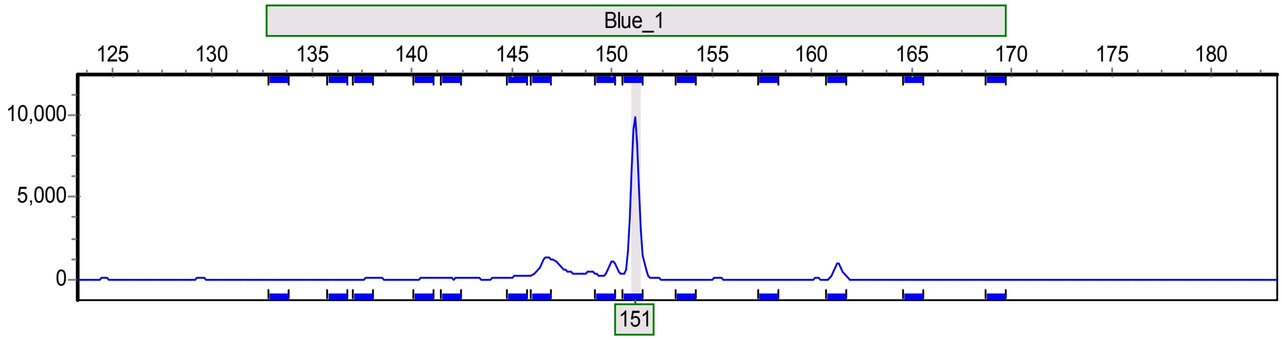

No	Size	Height	Area	Marker	Allele	Difference	Quality	Score	Allele Comments	Sample Comments
1	151.2	9784	47048	Blue_1	151	0.1	Pass	500.0		

Sample 14: Run date and time: 09/15/2020 - 21:11:04 -> 09/15/2020 - 21:56:47

Dye: Blue - 1 peaks - 21.fsa

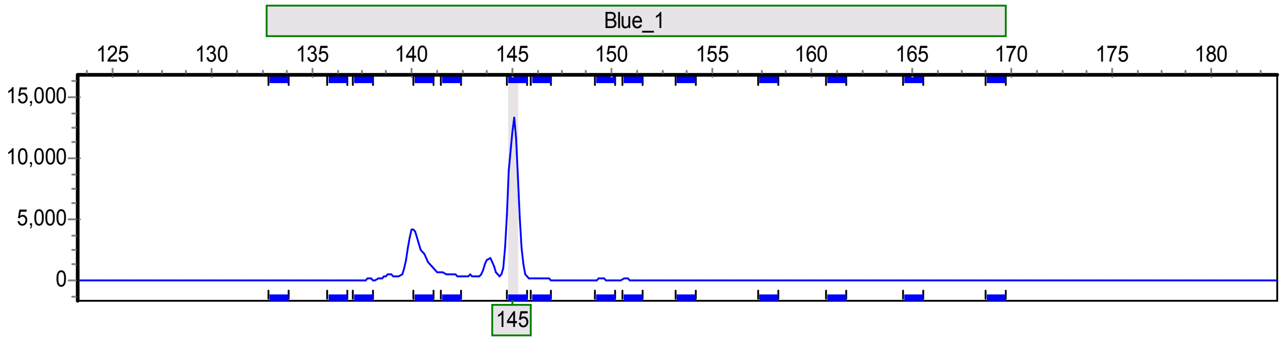

No	Size	Height	Area	Marker	Allele	Difference	Quality	Score	Allele Comments	Sample Comments
1	145.1	13237	67933	Blue_1	145	0.2	Pass	500.0		

Sample 15: Run date and time: 09/15/2020 - 21:11:04 -> 09/15/2020 - 21:56:47

Dye: Blue - 2 peaks - 22.fsa

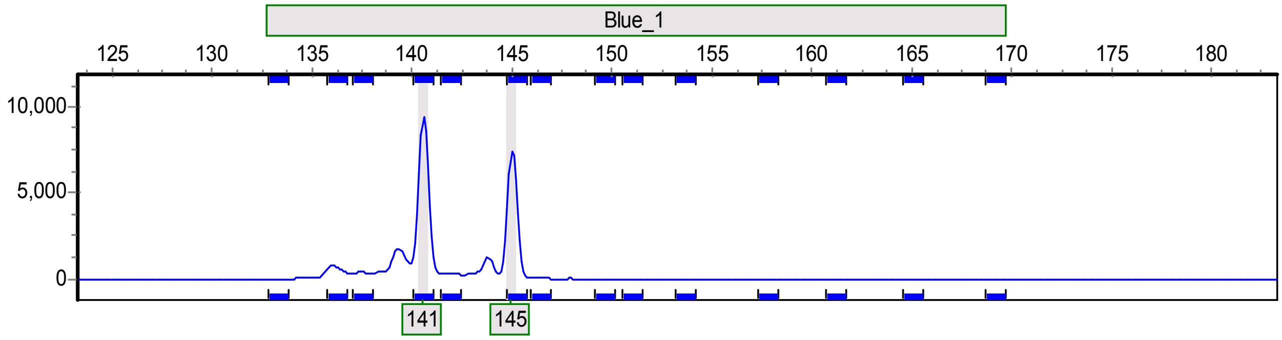

No	Size	Height	Area	Marker	Allele	Difference	Quality	Score	Allele Comments	Sample Comments
1	140.6	9320	50957	Blue_1	141	0.0	Pass	500.0		
2	145.0	7354	38880	Blue_1	145	0.3	Pass	500.0		

Sample 16: Run date and time: 09/15/2020 - 21:11:04 -> 09/15/2020 - 21:56:47

Dye: Blue - 1 peaks - 23.fsa

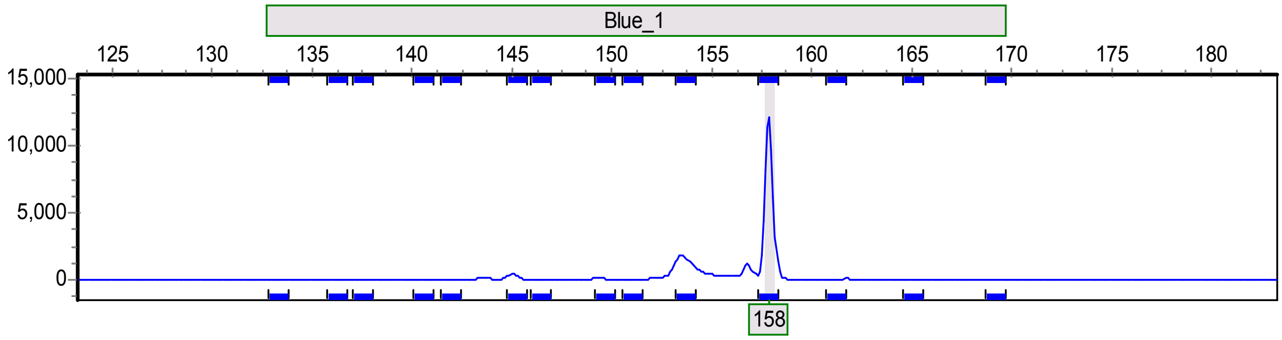

No	Size	Height	Area	Marker	Allele	Difference	Quality	Score	Allele Comments	Sample Comments
1	157.9	12003	54945	Blue_1	158	0.0	Pass	500.0		

Sample 17: Run date and time: 09/15/2020 - 21:11:04 -> 09/15/2020 - 21:56:47

Dye: Blue - 2 peaks - 24.fsa

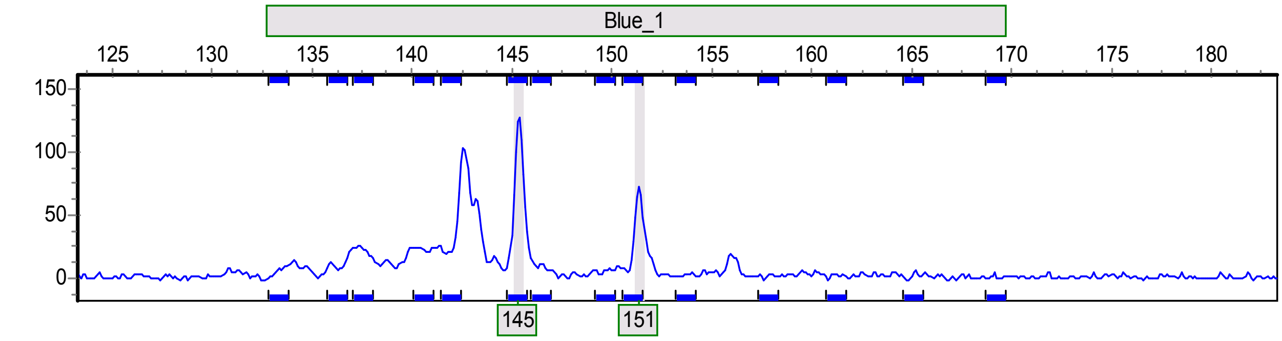

No	Size	Height	Area	Marker	Allele	Difference	Quality	Score	Allele Comments	Sample Comments
1	145.4	128	746	Blue_1	145	0.1	Pass	5.5	[<Confirmed>]	
2	151.4	73	387	Blue_1	151	0.3	Pass	2.3	[<Confirmed>]	

Sample 18: Run date and time: 09/15/2020 - 21:11:04 -> 09/15/2020 - 21:56:47

Dye: Blue - 2 peaks - 25.fsa

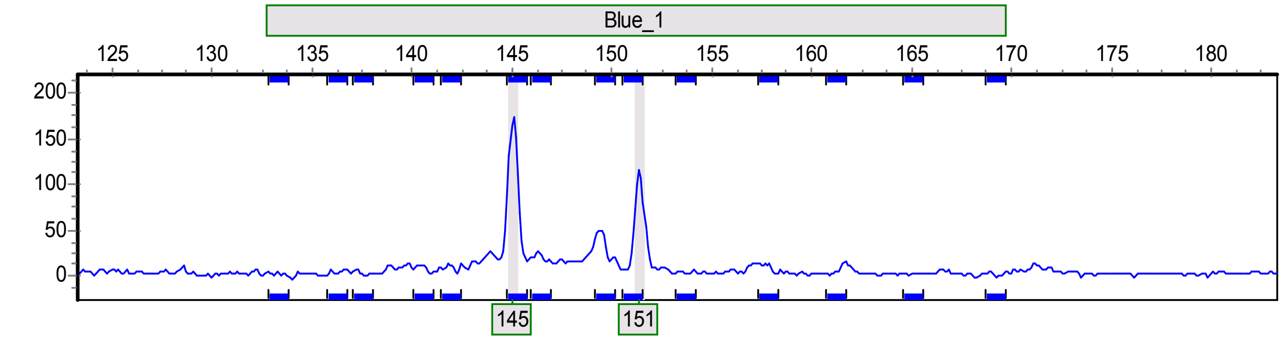

No	Size	Height	Area	Marker	Allele	Difference	Quality	Score	Allele Comments	Sample Comments
1	145.1	173	966	Blue_1	145	0.2	Pass	10.2		
2	151.4	116	619	Blue_1	151	0.3	Pass	5.5	[<Confirmed>]	

Sample 19: Run date and time: 09/15/2020 - 21:11:04 -> 09/15/2020 - 21:56:47

Dye: Blue - 2 peaks - 26.fsa

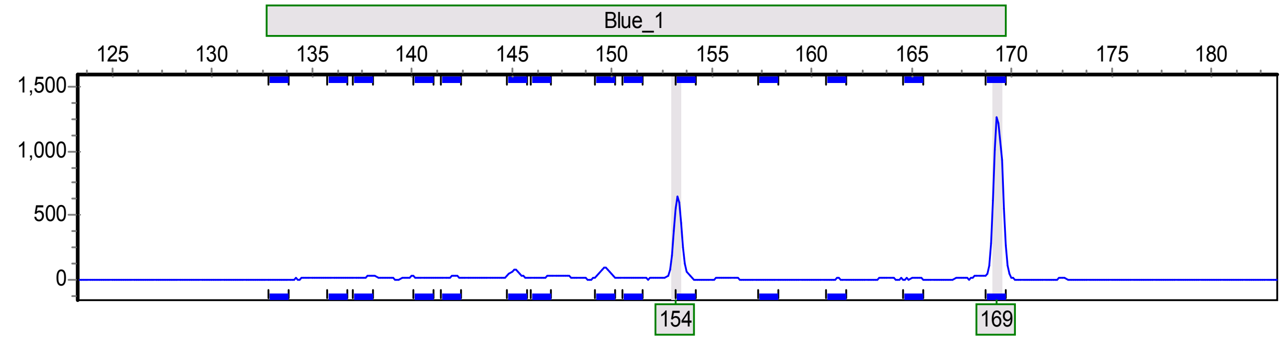

No	Size	Height	Area	Marker	Allele	Difference	Quality	Score	Allele Comments	Sample Comments
1	153.3	654	3154	Blue_1	154	0.5	Pass	116.7		
2	169.3	1259	5928	Blue_1	169	0.0	Pass	315.5		

Sample 20: Run date and time: 09/15/2020 - 21:11:04 -> 09/15/2020 - 21:56:47

Dye: Blue - 1 peaks - 27.fsa

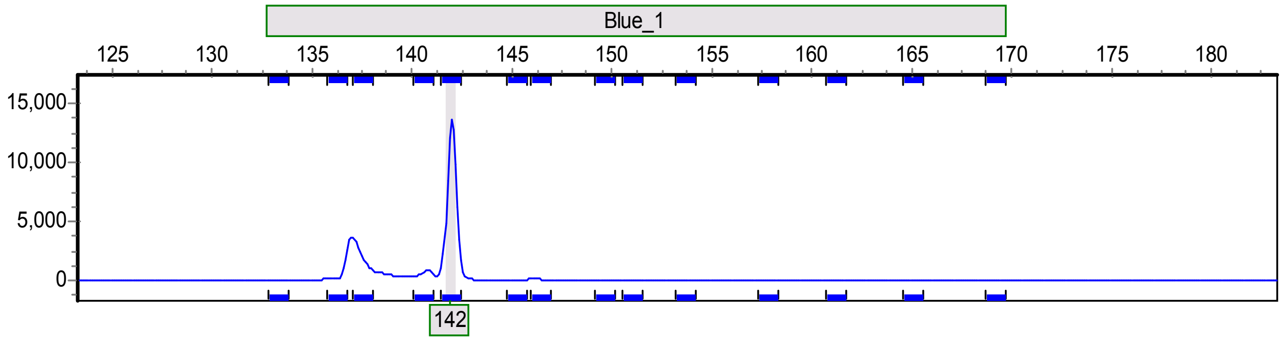

No	Size	Height	Area	Marker	Allele	Difference	Quality	Score	Allele Comments	Sample Comments
1	142.0	13760	71775	Blue_1	142	0.0	Pass	500.0		

Sample 21: Run date and time: 09/15/2020 - 21:11:04 -> 09/15/2020 - 21:56:47

Dye: Blue - 1 peaks - 28.fsa

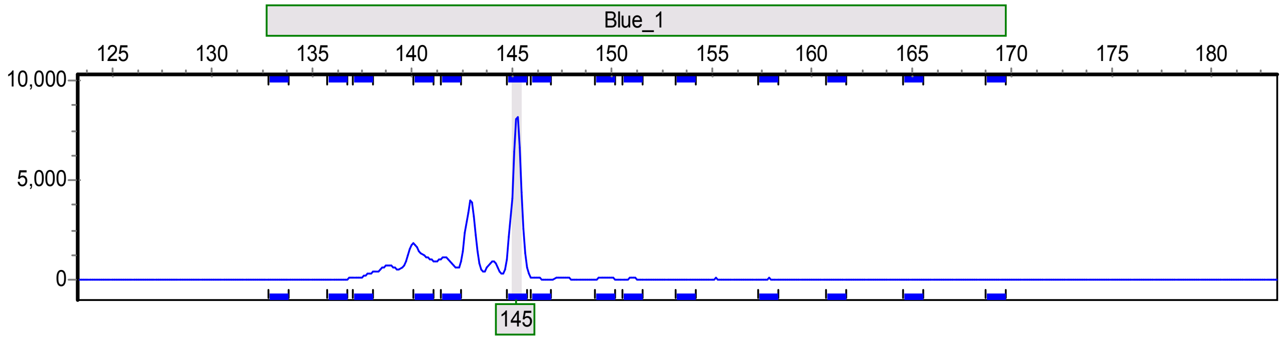

No	Size	Height	Area	Marker	Allele	Difference	Quality	Score	Allele Comments	Sample Comments
1	145.3	8105	42370	Blue_1	145	0.0	Pass	500.0		

Sample 22: Run date and time: 09/15/2020 - 21:11:04 -> 09/15/2020 - 21:56:47

Dye: Blue - 1 peaks - 29.fsa

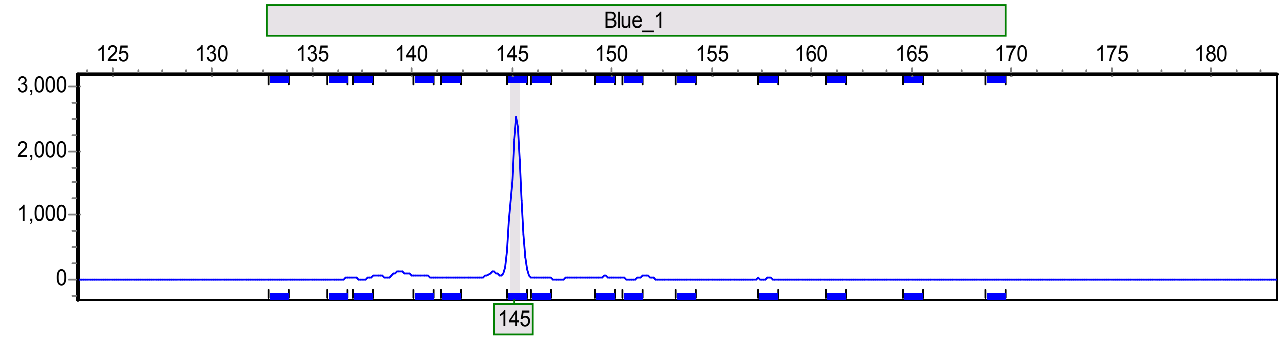

No	Size	Height	Area	Marker	Allele	Difference	Quality	Score	Allele Comments	Sample Comments
1	145.2	2510	13457	Blue_1	145	0.1	Pass	500.0		

Sample 23: Run date and time: 09/15/2020 - 21:11:04 -> 09/15/2020 - 21:56:47

Dye: Blue - 2 peaks - 3.fsa

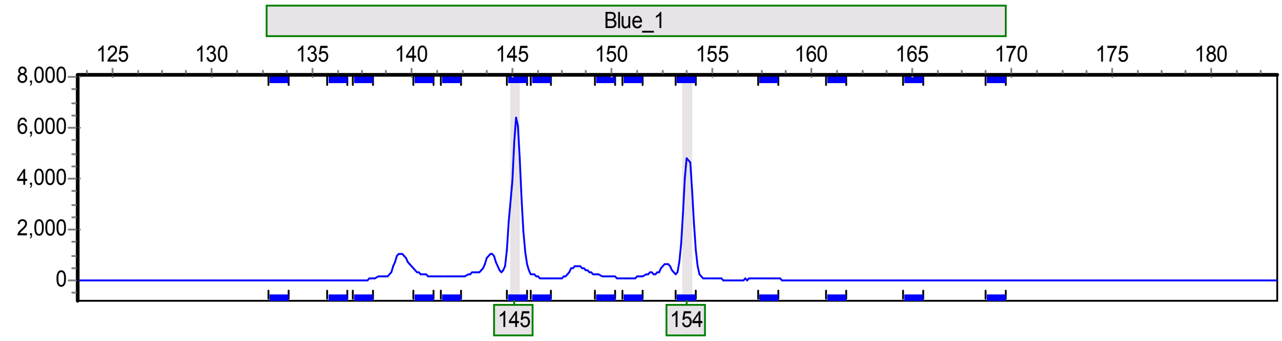

No	Size	Height	Area	Marker	Allele	Difference	Quality	Score	Allele Comments	Sample Comments
1	145.2	6371	34920	Blue_1	145	0.1	Pass	500.0		
2	153.8	4797	24383	Blue_1	154	0.0	Pass	500.0		

Sample 24: Run date and time: 09/15/2020 - 21:11:04 -> 09/15/2020 - 21:56:47

Dye: Blue - 1 peaks - 30.fsa

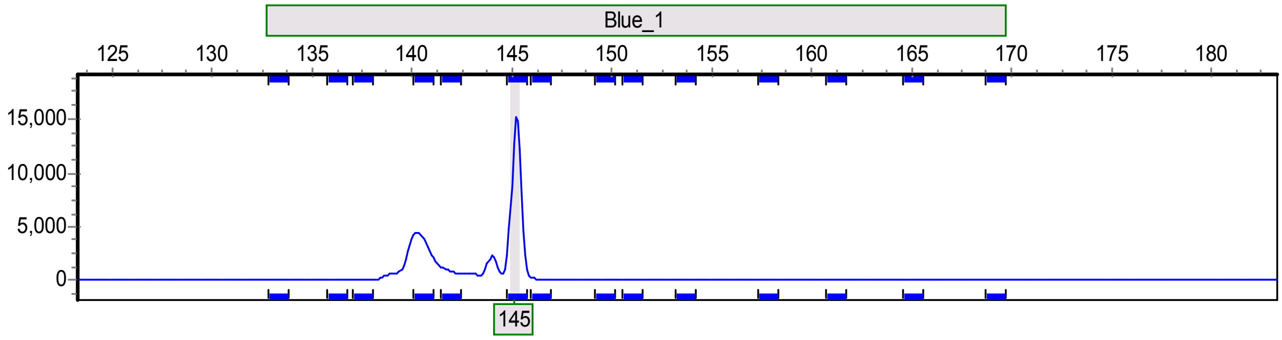

No	Size	Height	Area	Marker	Allele	Difference	Quality	Score	Allele Comments	Sample Comments
1	145.2	15149	81794	Blue_1	145	0.1	Pass	500.0		

Sample 25: Run date and time: 09/15/2020 - 21:11:04 -> 09/15/2020 - 21:56:47

Dye: Blue - 0 peaks - 31.fsa

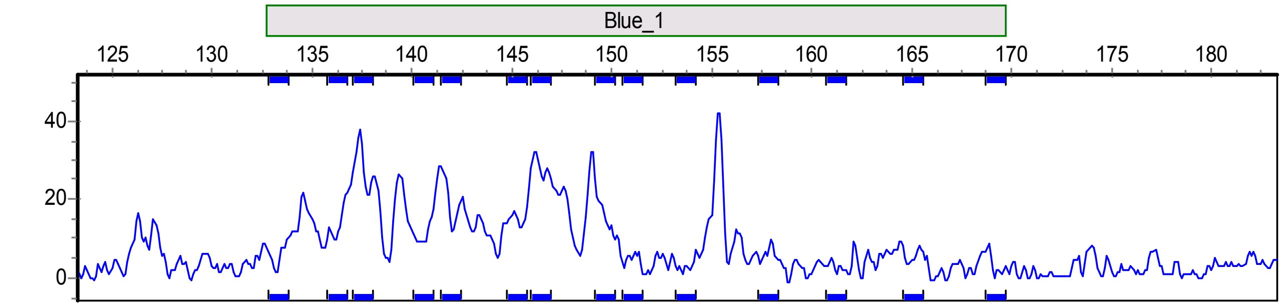

No	Size	Height	Area	Marker	Allele	Difference	Quality	Score	Allele Comments	Sample Comments

Sample 26: Run date and time: 09/15/2020 - 21:11:04 -> 09/15/2020 - 21:56:47

Dye: Blue - 1 peaks - 32.fsa

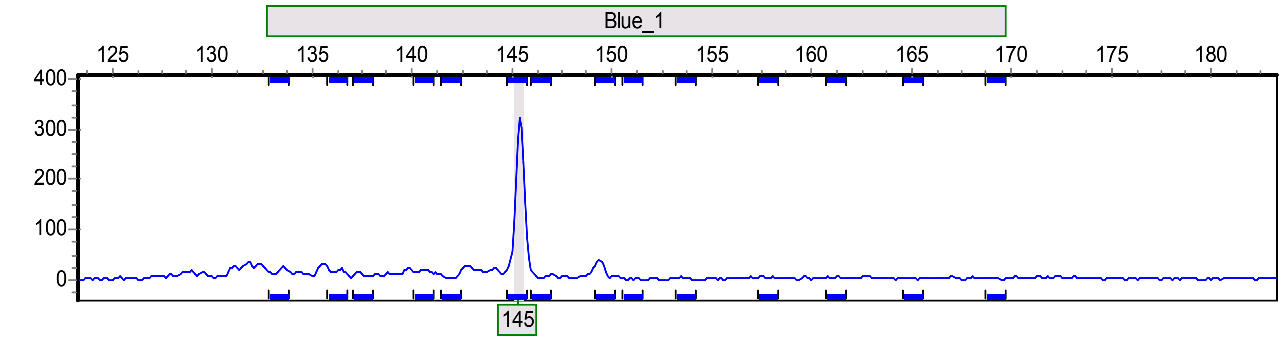

No	Size	Height	Area	Marker	Allele	Difference	Quality	Score	Allele Comments	Sample Comments
1	145.4	321	1701	Blue_1	145	0.1	Pass	32.9		

Sample 27: Run date and time: 09/15/2020 - 21:11:04 -> 09/15/2020 - 21:56:47

Dye: Blue - 2 peaks - 33.fsa

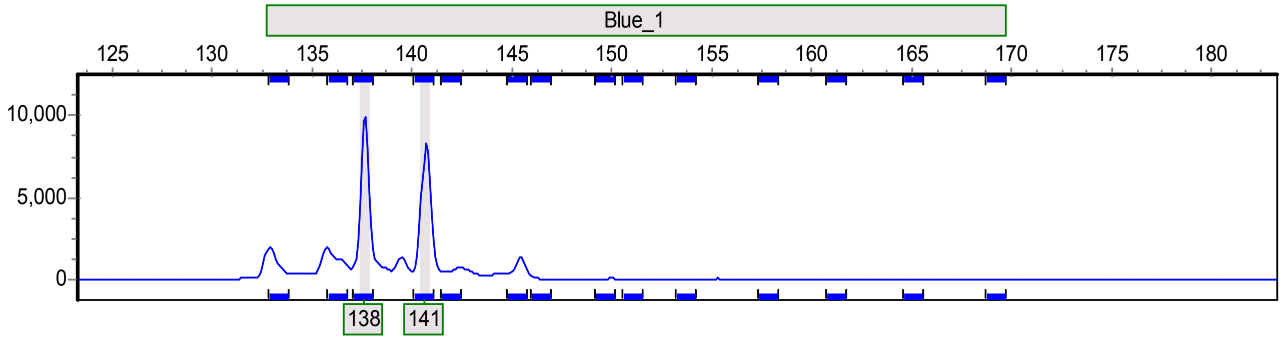

No	Size	Height	Area	Marker	Allele	Difference	Quality	Score	Allele Comments	Sample Comments
1	137.7	9820	50343	Blue_1	138	0.1	Pass	500.0		
2	140.7	8216	44436	Blue_1	141	0.1	Pass	500.0		

Allele Report

Sample 28: Run date and time: 09/15/2020 - 21:11:04 -> 09/15/2020 - 21:56:47

Dye: Blue - 2 peaks - 34.fsa

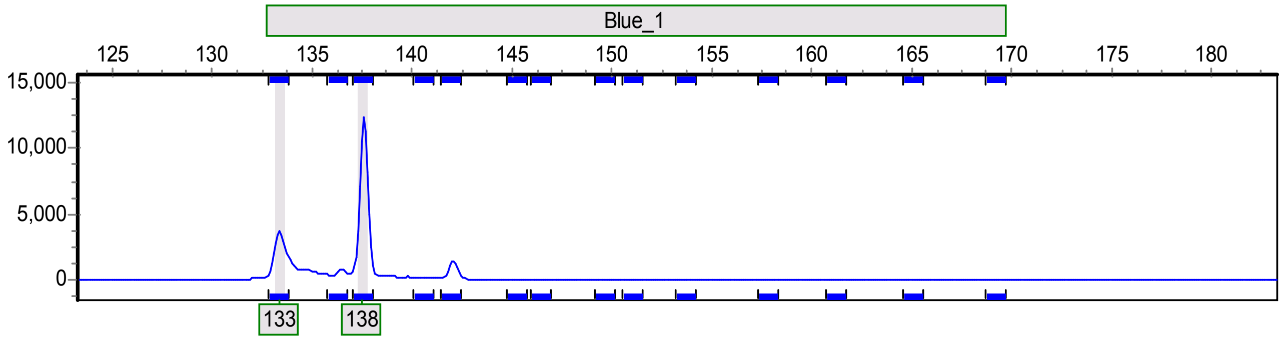

No	Size	Height	Area	Marker	Allele	Difference	Quality	Score	Allele Comments	Sample Comments
1	133.4	3652	25544	Blue_1	133	0.0	Pass	500.0		
2	137.6	12308	59472	Blue_1	138	0.0	Pass	500.0		

Sample 29: Run date and time: 09/15/2020 - 21:11:04 -> 09/15/2020 - 21:56:47

Dye: Blue - 2 peaks - 35.fsa

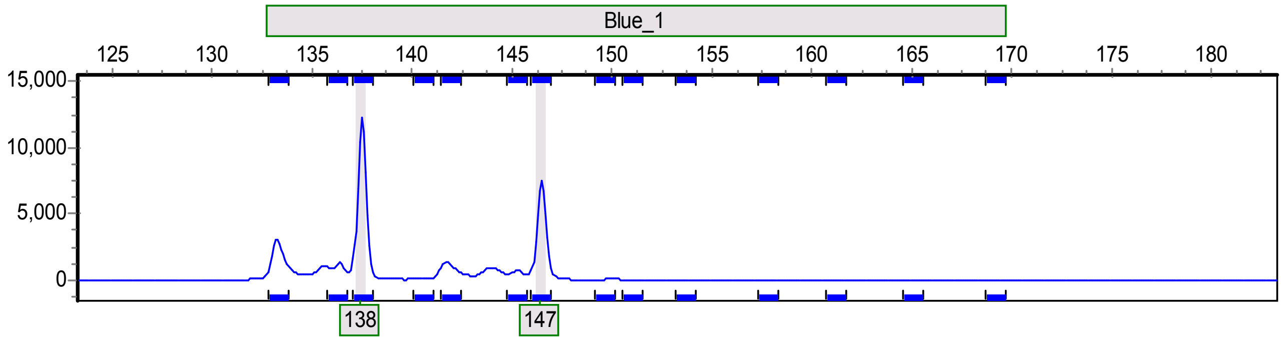

No	Size	Height	Area	Marker	Allele	Difference	Quality	Score	Allele Comments	Sample Comments
1	137.5	12139	58832	Blue_1	138	0.1	Pass	500.0		
2	146.5	7449	39030	Blue_1	147	0.0	Pass	500.0		

Sample 30: Run date and time: 09/15/2020 - 21:11:04 -> 09/15/2020 - 21:56:47

Dye: Blue - 1 peaks - 36.fsa

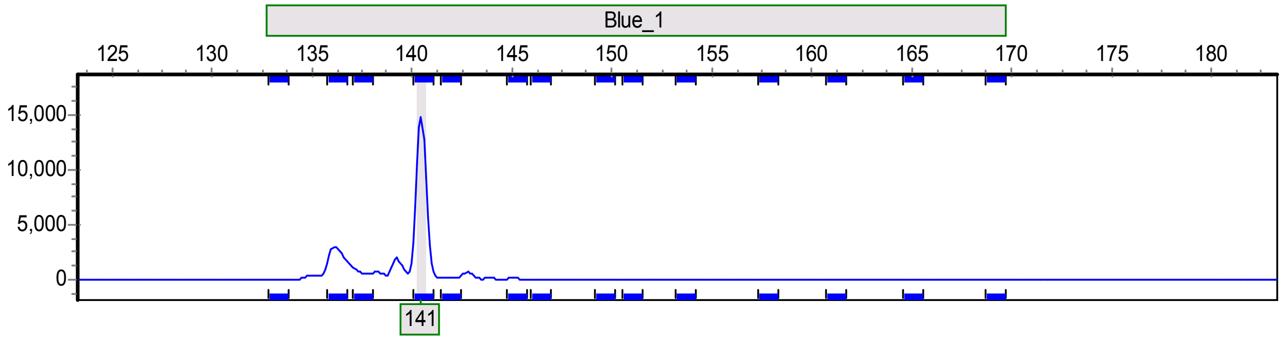

No	Size	Height	Area	Marker	Allele	Difference	Quality	Score	Allele Comments	Sample Comments
1	140.5	14672	77927	Blue_1	141	0.1	Pass	500.0		

Allele Report

Sample 31: Run date and time: 09/15/2020 - 21:11:04 -> 09/15/2020 - 21:56:47

Dye: Blue - 1 peaks - 37.fsa

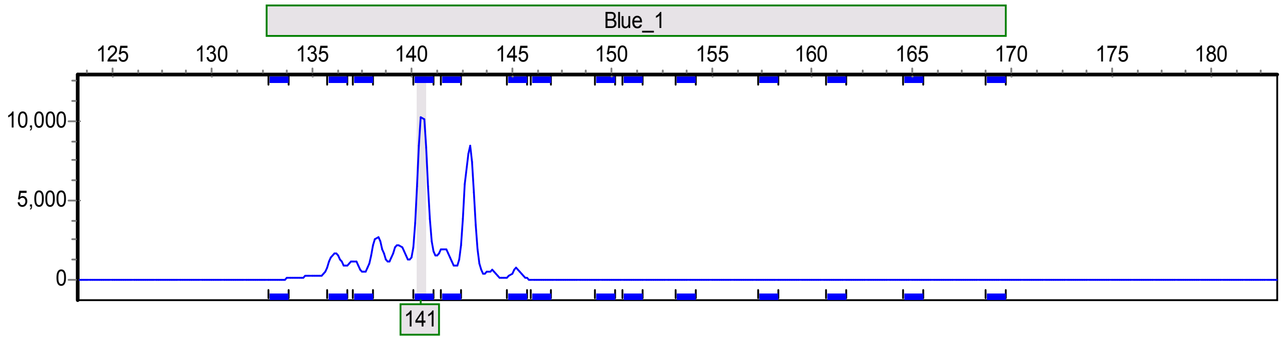

No	Size	Height	Area	Marker	Allele	Difference	Quality	Score	Allele Comments	Sample Comments
1	140.5	10249	59005	Blue_1	141	0.1	Pass	500.0		

Sample 32: Run date and time: 09/15/2020 - 21:11:04 -> 09/15/2020 - 21:56:47

Dye: Blue - 1 peaks - 38.fsa

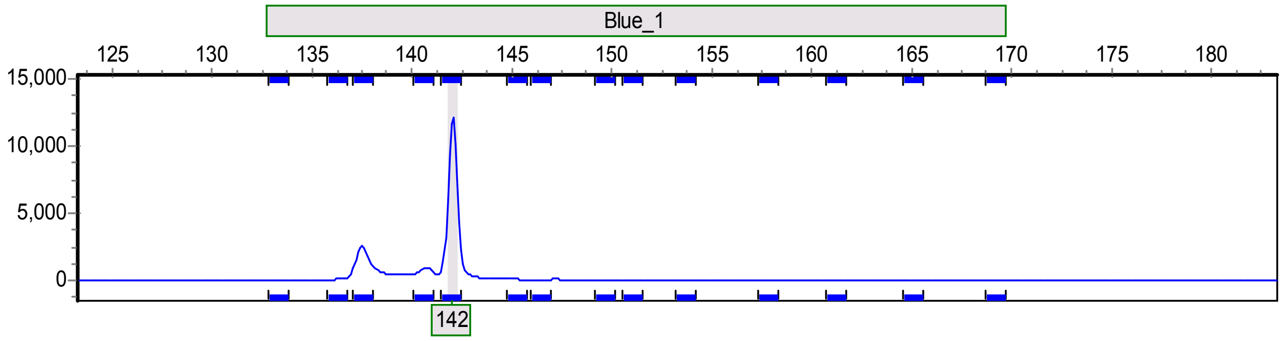

No	Size	Height	Area	Marker	Allele	Difference	Quality	Score	Allele Comments	Sample Comments
1	142.1	12004	64270	Blue_1	142	0.1	Pass	500.0		

Sample 33: Run date and time: 09/15/2020 - 21:11:04 -> 09/15/2020 - 21:56:47

Dye: Blue - 2 peaks - 39.fsa

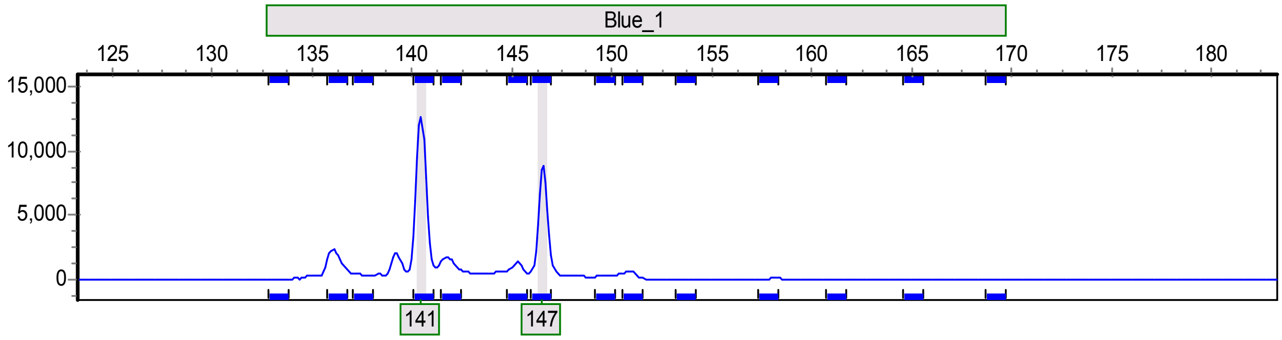

No	Size	Height	Area	Marker	Allele	Difference	Quality	Score	Allele Comments	Sample Comments
1	140.5	12598	69201	Blue_1	141	0.1	Pass	500.0		
2	146.6	8804	48098	Blue_1	147	0.1	Pass	500.0		

Sample 34: Run date and time: 09/15/2020 - 21:11:04 -> 09/15/2020 - 21:56:47

Dye: Blue - 1 peaks - 4.fsa

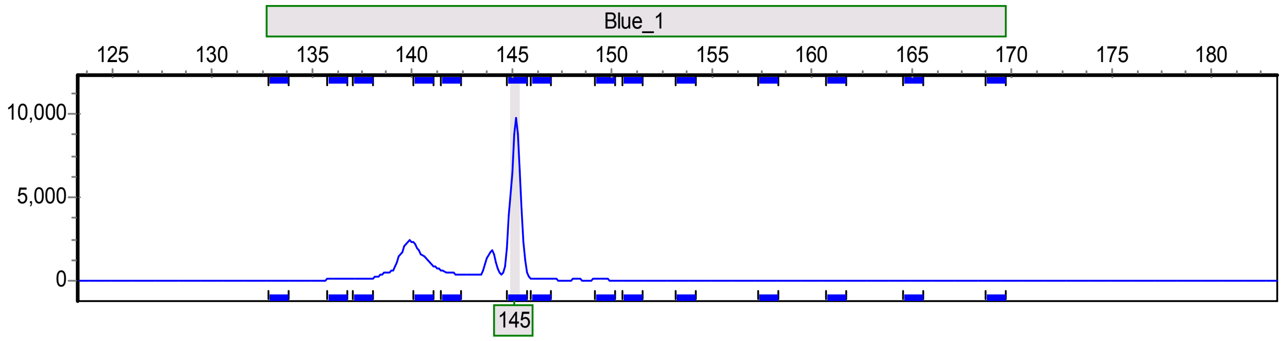

No	Size	Height	Area	Marker	Allele	Difference	Quality	Score	Allele Comments	Sample Comments
1	145.2	9743	51667	Blue_1	145	0.1	Pass	500.0		

Sample 35: Run date and time: 09/15/2020 - 21:11:04 -> 09/15/2020 - 21:56:47

Dye: Blue - 1 peaks - 40.fsa

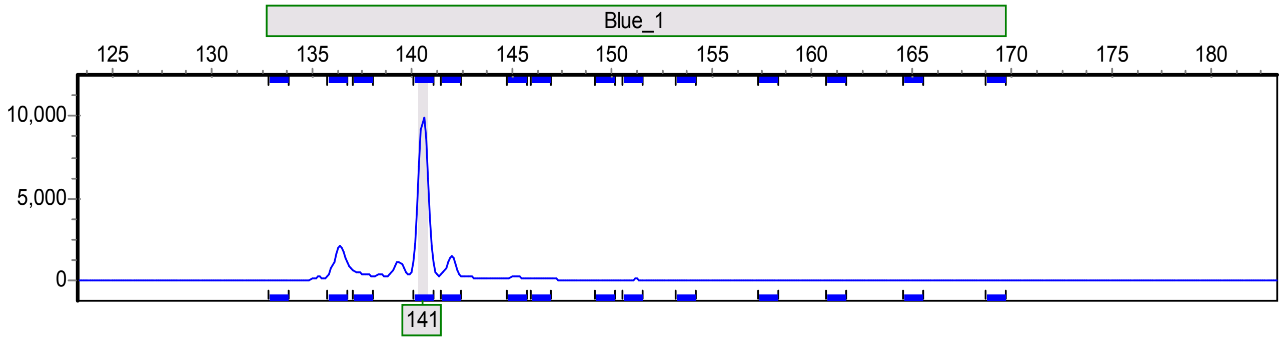

No	Size	Height	Area	Marker	Allele	Difference	Quality	Score	Allele Comments	Sample Comments
1	140.6	9839	51983	Blue_1	141	0.0	Pass	500.0		

Sample 36: Run date and time: 09/15/2020 - 21:11:04 -> 09/15/2020 - 21:56:47

Dye: Blue - 1 peaks - 41.fsa

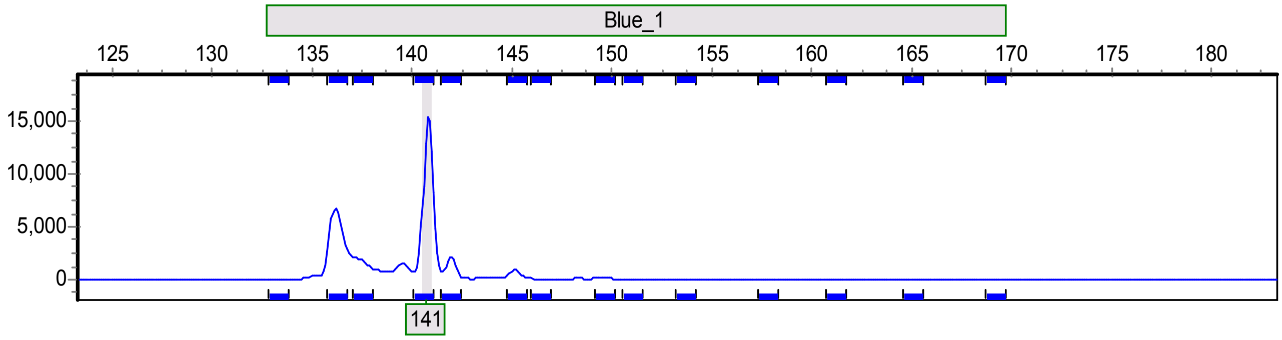

No	Size	Height	Area	Marker	Allele	Difference	Quality	Score	Allele Comments	Sample Comments
1	140.8	15264	83231	Blue_1	141	0.2	Pass	500.0		

Sample 37: Run date and time: 09/15/2020 - 21:11:04 -> 09/15/2020 - 21:56:47

Dye: Blue - 1 peaks - 42.fsa

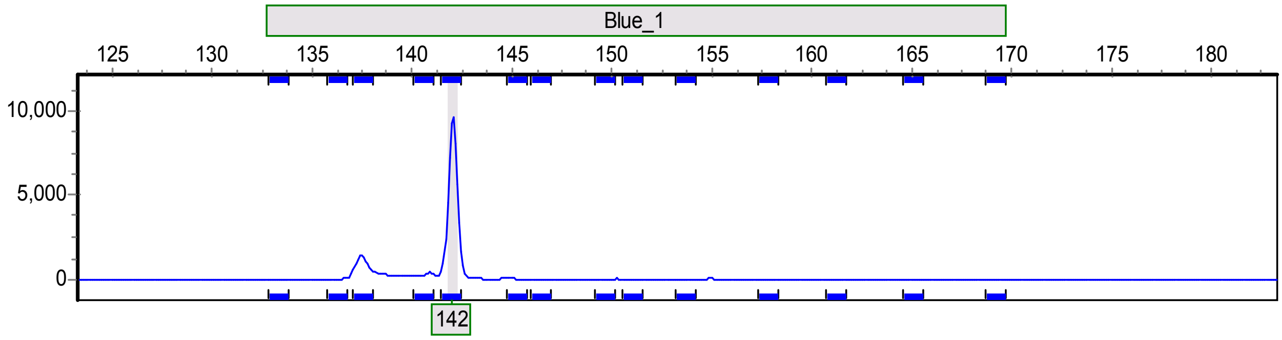

No	Size	Height	Area	Marker	Allele	Difference	Quality	Score	Allele Comments	Sample Comments
1	142.1	9553	50268	Blue_1	142	0.1	Pass	500.0		

Sample 38: Run date and time: 09/15/2020 - 21:11:04 -> 09/15/2020 - 21:56:47

Dye: Blue - 2 peaks - 43.fsa

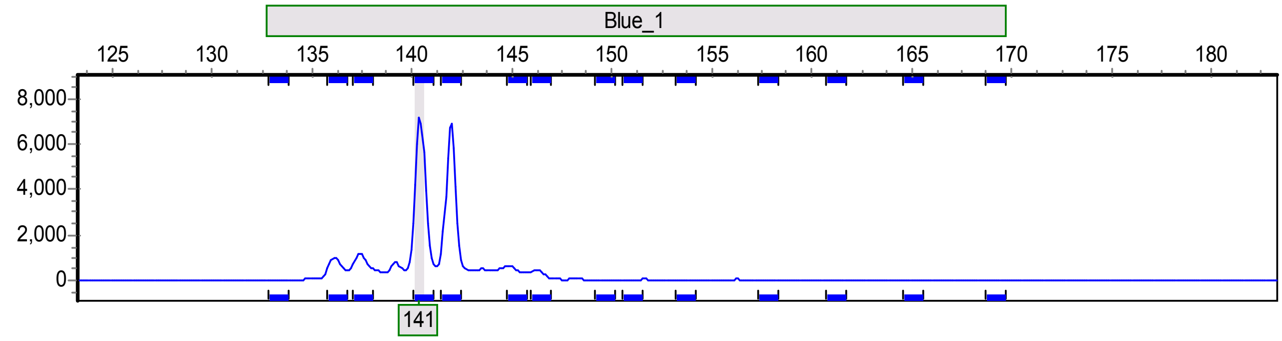

No	Size	Height	Area	Marker	Allele	Difference	Quality	Score	Allele Comments	Sample Comments
1	140.4	7144	40669	Blue_1	141	0.2	Pass	500.0		
2	142.0	6907	38701	Blue_1	142	0.0	Pass	500.0	[<Deleted>]	

Sample 39: Run date and time: 09/15/2020 - 21:11:04 -> 09/15/2020 - 21:56:47

Dye: Blue - 2 peaks - 44.fsa

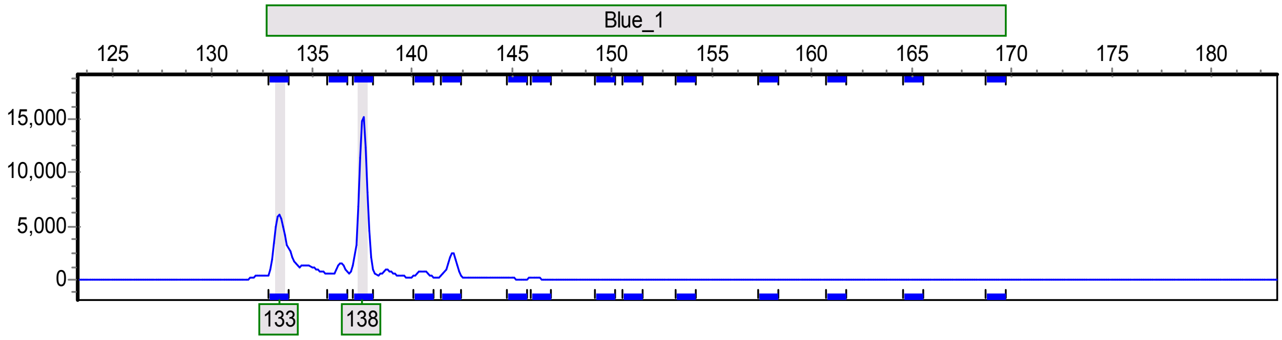

No	Size	Height	Area	Marker	Allele	Difference	Quality	Score	Allele Comments	Sample Comments
1	133.4	6105	42769	Blue_1	133	0.0	Pass	500.0		
2	137.6	15010	75010	Blue_1	138	0.0	Pass	500.0		

Allele Report

Sample 40: Run date and time: 09/15/2020 - 21:11:04 -> 09/15/2020 - 21:56:47

Dye: Blue - 2 peaks - 45.fsa

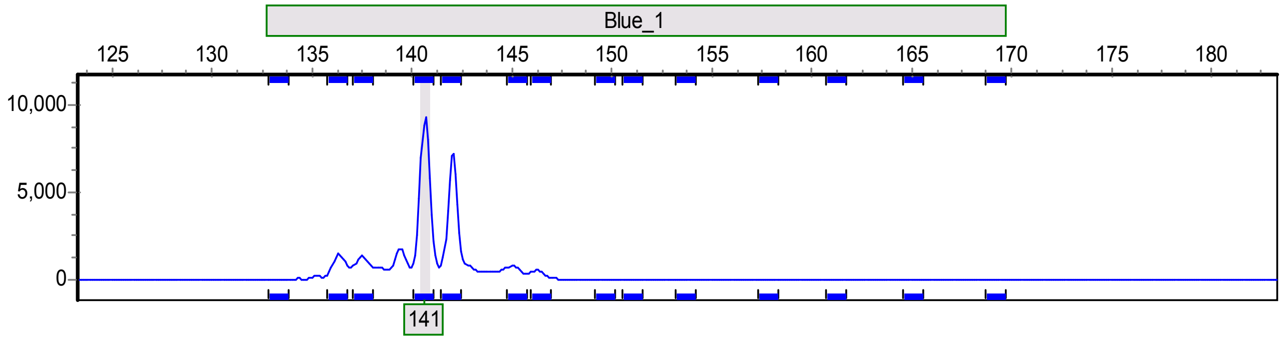

No	Size	Height	Area	Marker	Allele	Difference	Quality	Score	Allele Comments	Sample Comments
1	140.7	9200	51836	Blue_1	141	0.1	Pass	500.0		
2	142.1	7158	39193	Blue_1	142	0.1	Pass	500.0	[<Deleted>]	

Sample 41: Run date and time: 09/15/2020 - 21:11:04 -> 09/15/2020 - 21:56:47

Dye: Blue - 1 peaks - 46.fsa

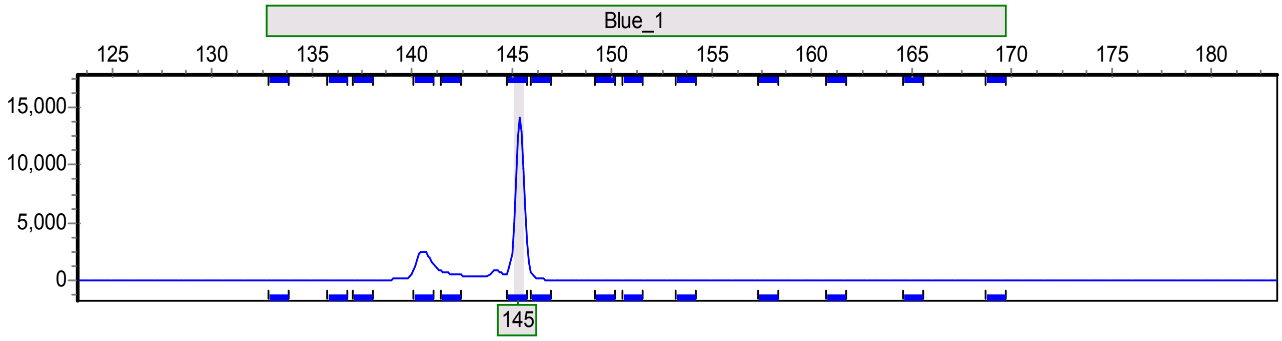

No	Size	Height	Area	Marker	Allele	Difference	Quality	Score	Allele Comments	Sample Comments
1	145.4	14063	72346	Blue_1	145	0.1	Pass	500.0		

Sample 42: Run date and time: 09/15/2020 - 21:11:04 -> 09/15/2020 - 21:56:47

Dye: Blue - 2 peaks - 47.fsa

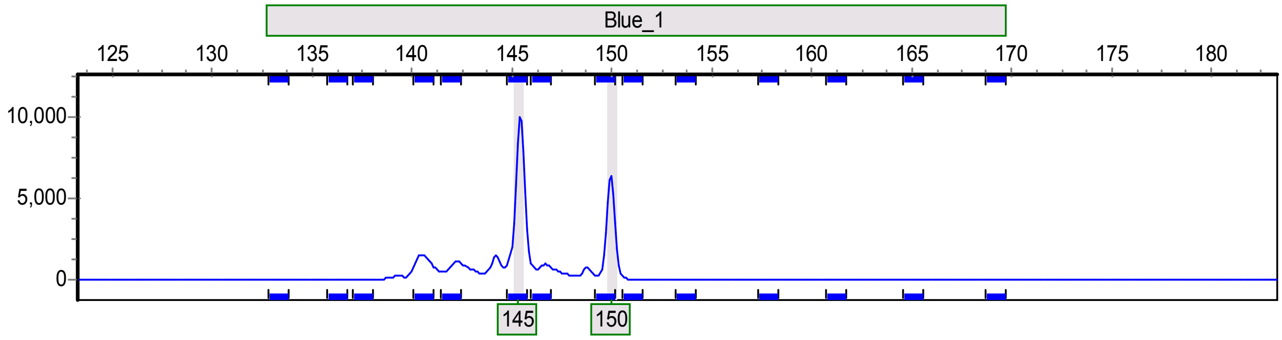

No	Size	Height	Area	Marker	Allele	Difference	Quality	Score	Allele Comments	Sample Comments
1	145.4	9897	55246	Blue_1	145	0.1	Pass	500.0		
2	150.0	6304	31829	Blue_1	150	0.3	Pass	500.0		

Allele Report

Sample 43: Run date and time: 09/15/2020 - 21:11:04 -> 09/15/2020 - 21:56:47

Dye: Blue - 1 peaks - 48.fsa

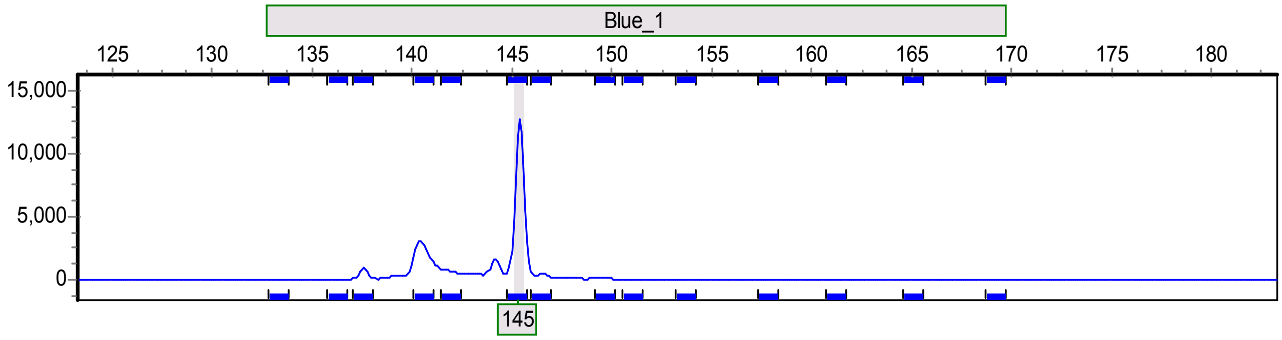

No	Size	Height	Area	Marker	Allele	Difference	Quality	Score	Allele Comments	Sample Comments
1	145.4	12762	65973	Blue_1	145	0.1	Pass	500.0		

Sample 44: Run date and time: 09/15/2020 - 21:11:04 -> 09/15/2020 - 21:56:47

Dye: Blue - 2 peaks - 49.fsa

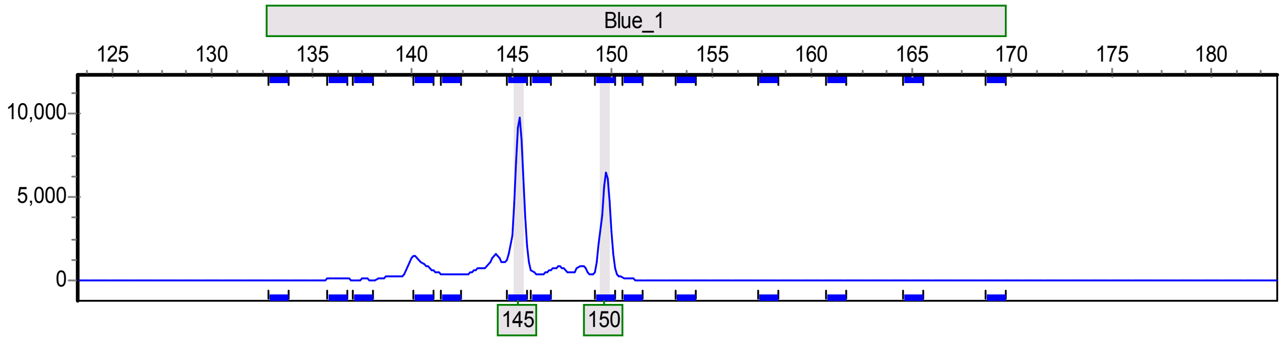

No	Size	Height	Area	Marker	Allele	Difference	Quality	Score	Allele Comments	Sample Comments
1	145.4	9720	53490	Blue_1	145	0.1	Pass	500.0		
2	149.7	6487	33621	Blue_1	150	0.0	Pass	500.0		

Sample 45: Run date and time: 09/15/2020 - 21:11:04 -> 09/15/2020 - 21:56:47

Dye: Blue - 1 peaks - 5.fsa

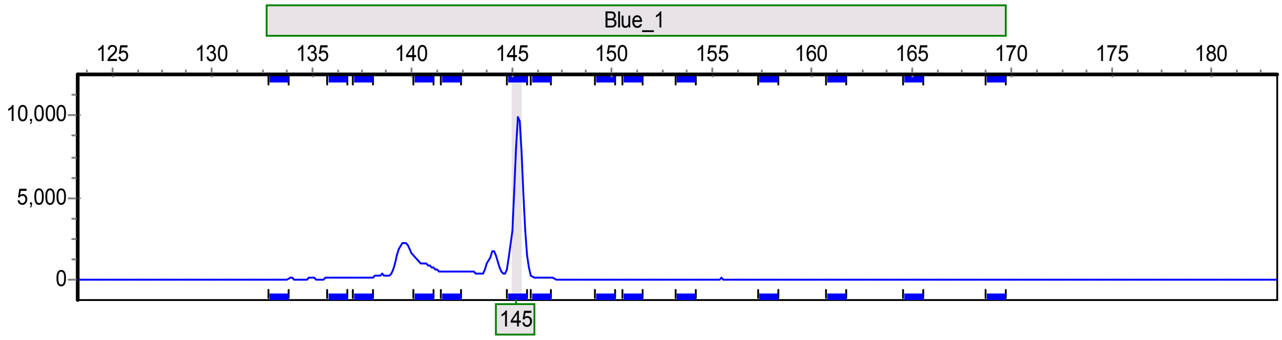

No	Size	Height	Area	Marker	Allele	Difference	Quality	Score	Allele Comments	Sample Comments
1	145.3	9817	52008	Blue_1	145	0.0	Pass	500.0		

Sample 46: Run date and time: 09/15/2020 - 21:11:04 -> 09/15/2020 - 21:56:47

Dye: Blue - 2 peaks - 50.fsa

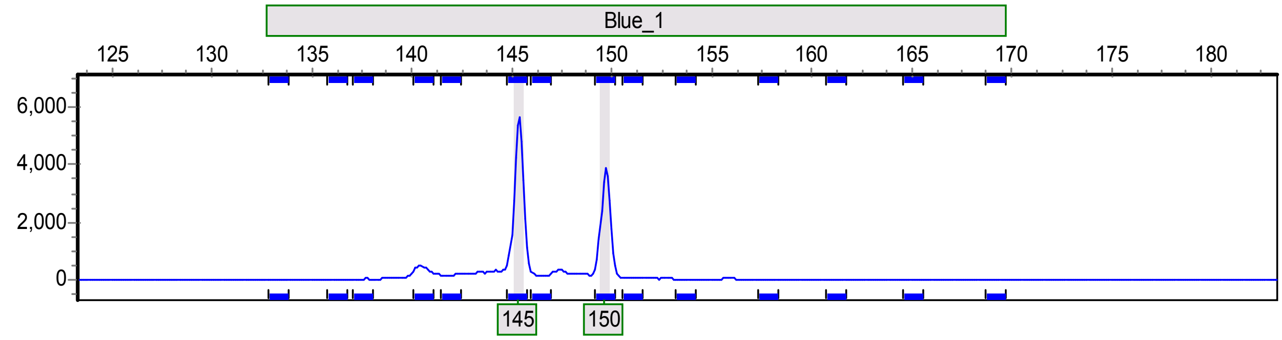

No	Size	Height	Area	Marker	Allele	Difference	Quality	Score	Allele Comments	Sample Comments
1	145.4	5621	30630	Blue_1	145	0.1	Pass	500.0		
2	149.7	3916	20401	Blue_1	150	0.0	Pass	500.0		

Sample 47: Run date and time: 09/15/2020 - 21:11:04 -> 09/15/2020 - 21:56:47

Dye: Blue - 2 peaks - 51.fsa

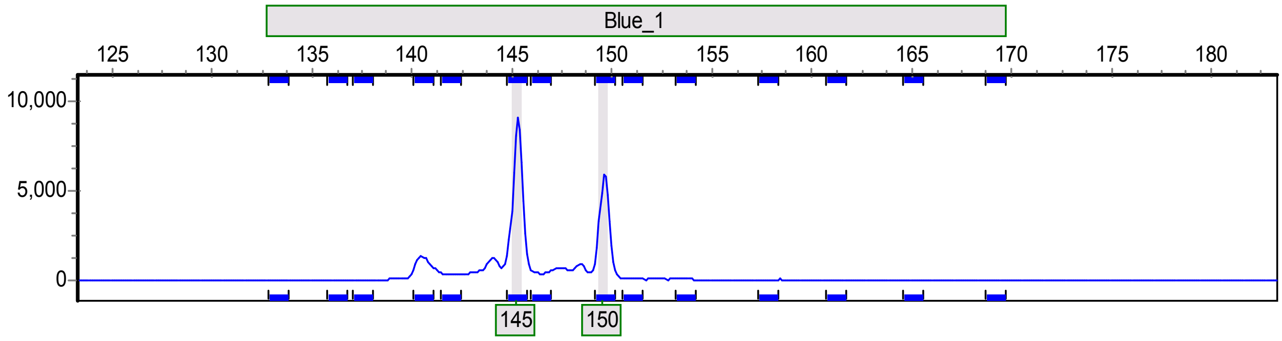

No	Size	Height	Area	Marker	Allele	Difference	Quality	Score	Allele Comments	Sample Comments
1	145.3	8987	51296	Blue_1	145	0.0	Pass	500.0		
2	149.6	5871	32132	Blue_1	150	0.1	Pass	500.0		

Sample 48: Run date and time: 09/15/2020 - 21:11:04 -> 09/15/2020 - 21:56:47

Dye: Blue - 2 peaks - 52.fsa

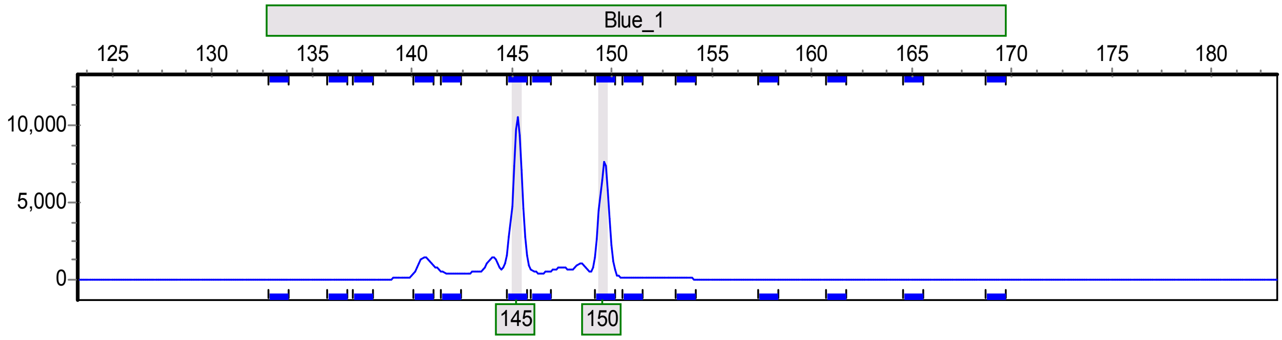

No	Size	Height	Area	Marker	Allele	Difference	Quality	Score	Allele Comments	Sample Comments
1	145.3	10435	58789	Blue_1	145	0.0	Pass	500.0		
2	149.6	7611	41232	Blue_1	150	0.1	Pass	500.0		

Allele Report

Sample 49: Run date and time: 09/15/2020 - 21:11:04 -> 09/15/2020 - 21:56:47

Dye: Blue - 3 peaks - 53.fsa

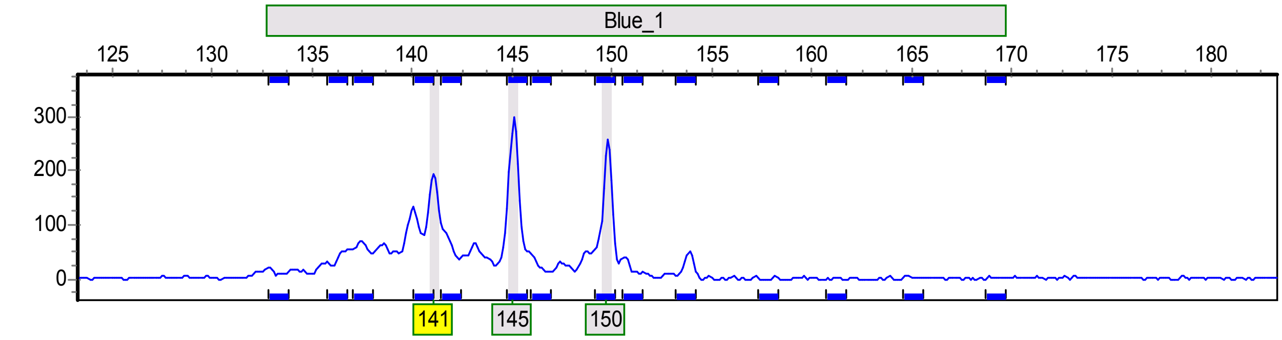

No	Size	Height	Area	Marker	Allele	Difference	Quality	Score	Allele Comments	Sample Comments
1	141.1	195	1315	Blue_1	141	0.5	Check	6.0		
2	145.1	298	1719	Blue_1	145	0.2	Pass	24.8		
3	149.8	257	1435	Blue_1	150	0.1	Pass	20.3		

Sample 50: Run date and time: 09/15/2020 - 21:11:04 -> 09/15/2020 - 21:56:47

Dye: Blue - 1 peaks - 54.fsa

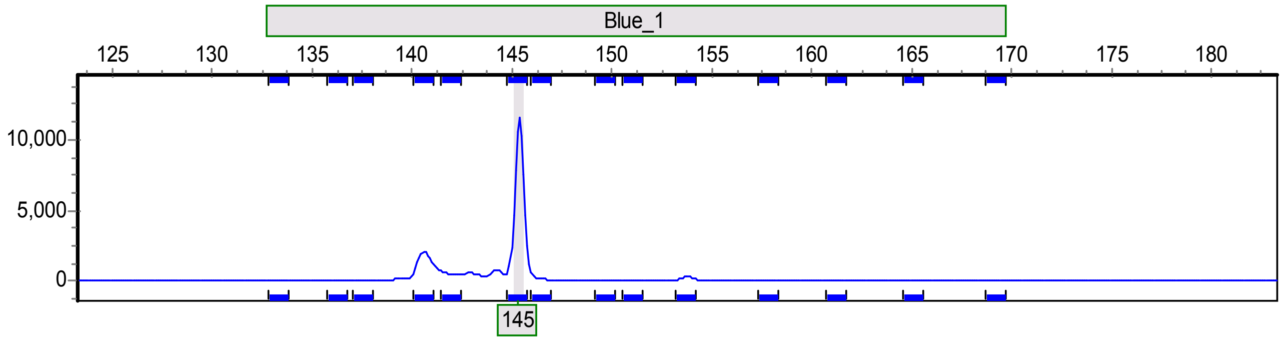

No	Size	Height	Area	Marker	Allele	Difference	Quality	Score	Allele Comments	Sample Comments
1	145.4	11590	59883	Blue_1	145	0.1	Pass	500.0		

Sample 51: Run date and time: 09/15/2020 - 21:11:04 -> 09/15/2020 - 21:56:47

Dye: Blue - 1 peaks - 55.fsa

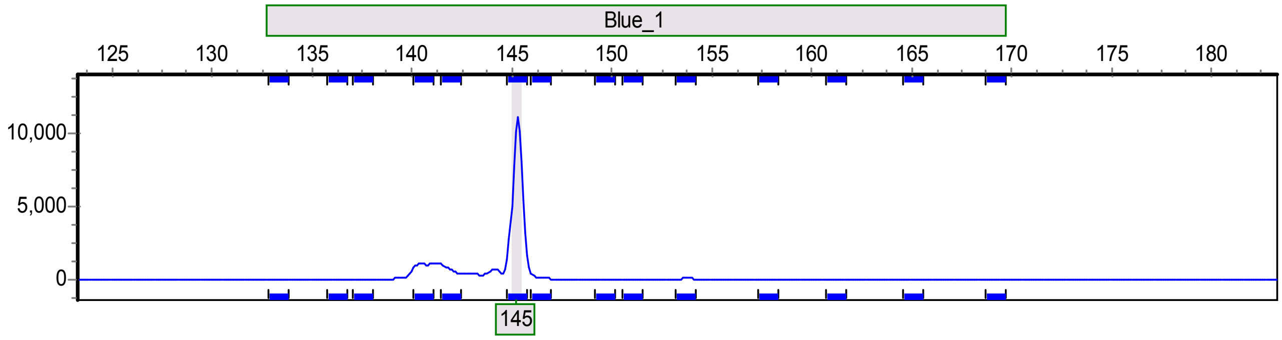

No	Size	Height	Area	Marker	Allele	Difference	Quality	Score	Allele Comments	Sample Comments
1	145.3	11050	63681	Blue_1	145	0.0	Pass	500.0		

Sample 52: Run date and time: 09/15/2020 - 21:11:04 -> 09/15/2020 - 21:56:47

Dye: Blue - 2 peaks - 56.fsa

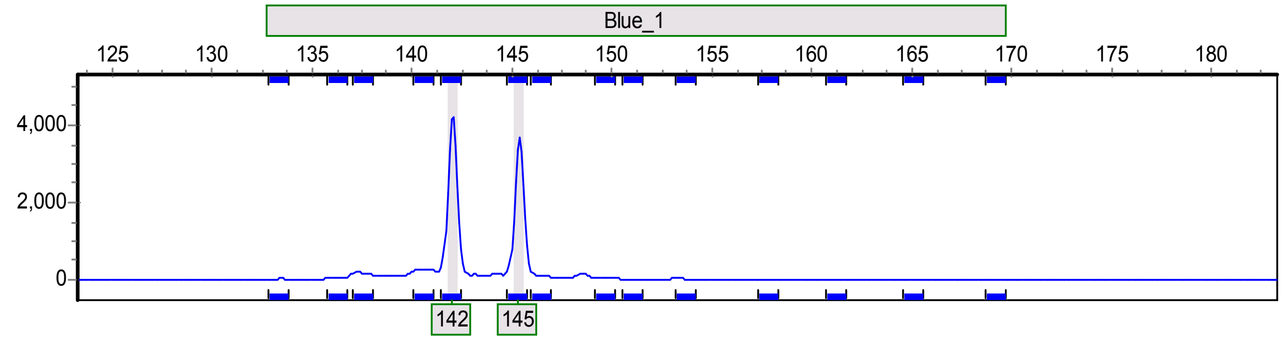

No	Size	Height	Area	Marker	Allele	Difference	Quality	Score	Allele Comments	Sample Comments
1	142.1	4177	22912	Blue_1	142	0.1	Pass	500.0		
2	145.4	3682	19629	Blue_1	145	0.1	Pass	500.0		

Sample 53: Run date and time: 09/15/2020 - 21:11:04 -> 09/15/2020 - 21:56:47

Dye: Blue - 1 peaks - 57.fsa

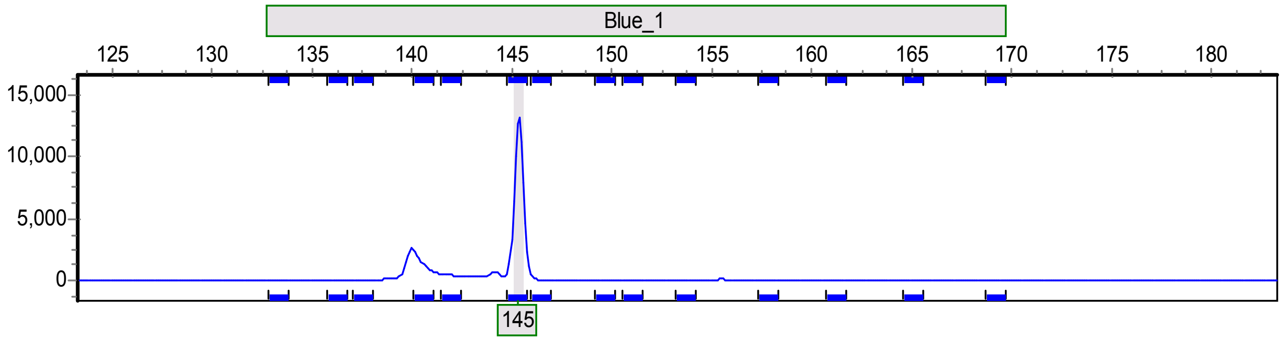

No	Size	Height	Area	Marker	Allele	Difference	Quality	Score	Allele Comments	Sample Comments
1	145.4	13103	69174	Blue_1	145	0.1	Pass	500.0		

Sample 54: Run date and time: 09/15/2020 - 21:11:04 -> 09/15/2020 - 21:56:47

Dye: Blue - 1 peaks - 58.fsa

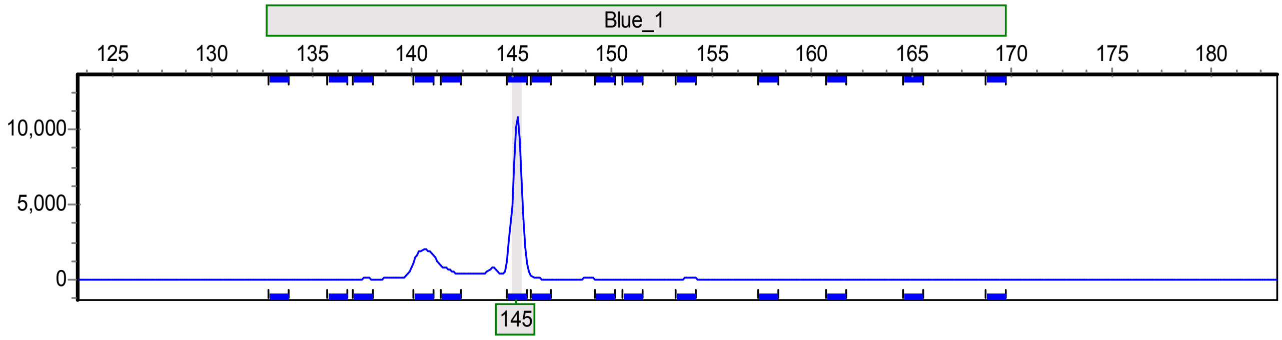

No	Size	Height	Area	Marker	Allele	Difference	Quality	Score	Allele Comments	Sample Comments
1	145.3	10672	56129	Blue_1	145	0.0	Pass	500.0		

Sample 55: Run date and time: 09/15/2020 - 21:11:04 -> 09/15/2020 - 21:56:47

Dye: Blue - 1 peaks - 59.fsa

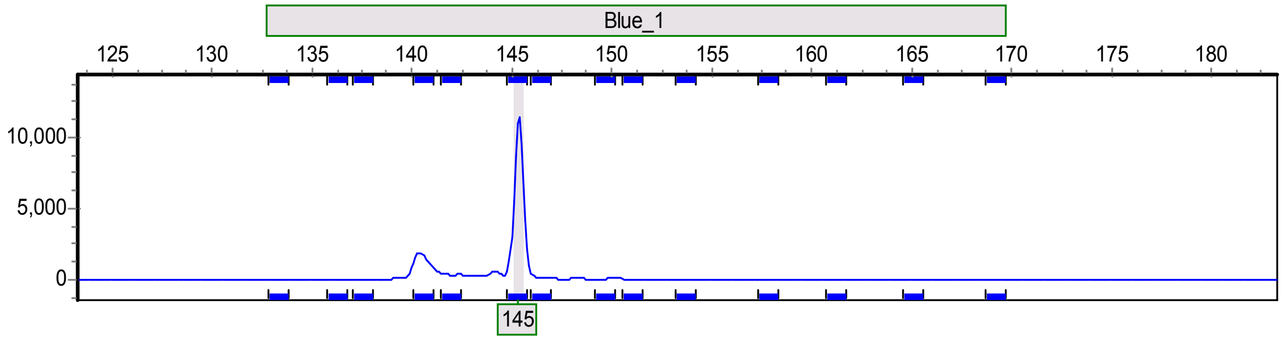

No	Size	Height	Area	Marker	Allele	Difference	Quality	Score	Allele Comments	Sample Comments
1	145.4	11363	60731	Blue_1	145	0.1	Pass	500.0		

Sample 56: Run date and time: 09/15/2020 - 21:11:04 -> 09/15/2020 - 21:56:47

Dye: Blue - 2 peaks - 6.fsa

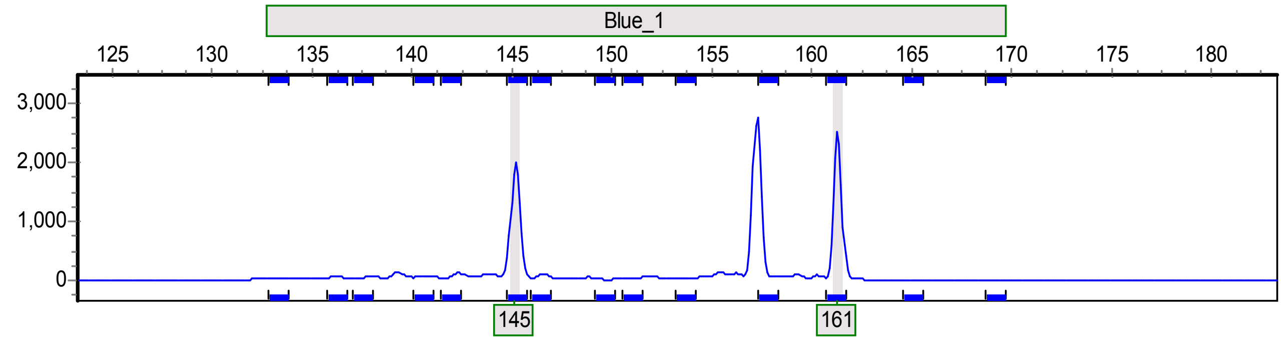

No	Size	Height	Area	Marker	Allele	Difference	Quality	Score	Allele Comments	Sample Comments
1	145.2	2008	10227	Blue_1	145	0.1	Pass	500.0		
2	161.3	2527	11288	Blue_1	161	0.0	Pass	500.0		

Sample 57: Run date and time: 09/15/2020 - 21:11:04 -> 09/15/2020 - 21:56:47

Dye: Blue - 1 peaks - 60.fsa

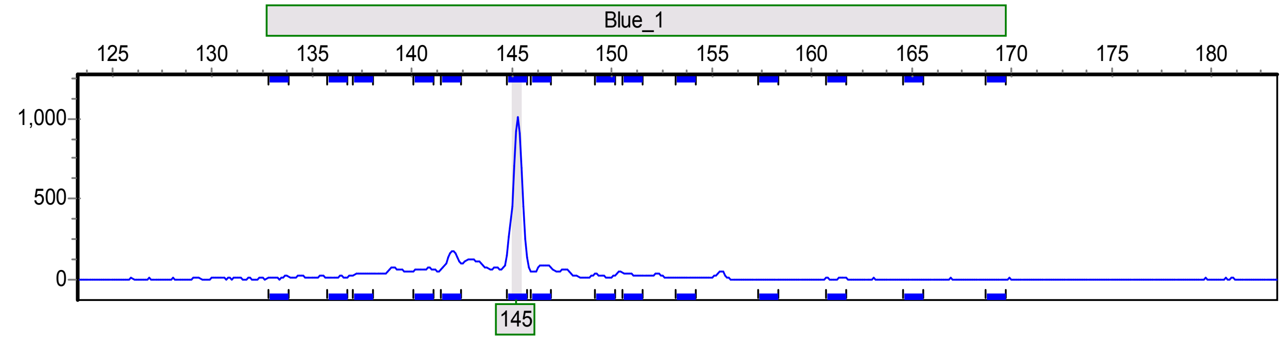

No	Size	Height	Area	Marker	Allele	Difference	Quality	Score	Allele Comments	Sample Comments
1	145.3	1006	5627	Blue_1	145	0.0	Pass	180.0		

Allele Report

Sample 58: Run date and time: 09/15/2020 - 21:11:04 -> 09/15/2020 - 21:56:47

Dye: Blue - 1 peaks - 61.fsa

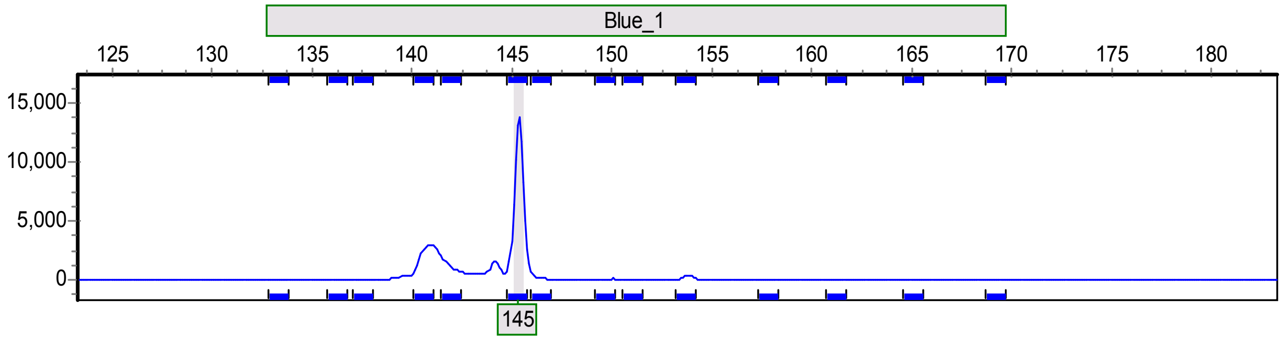

No	Size	Height	Area	Marker	Allele	Difference	Quality	Score	Allele Comments	Sample Comments
1	145.4	13753	72496	Blue_1	145	0.1	Pass	500.0		

Sample 59: Run date and time: 09/15/2020 - 21:11:04 -> 09/15/2020 - 21:56:47

Dye: Blue - 1 peaks - 62.fsa

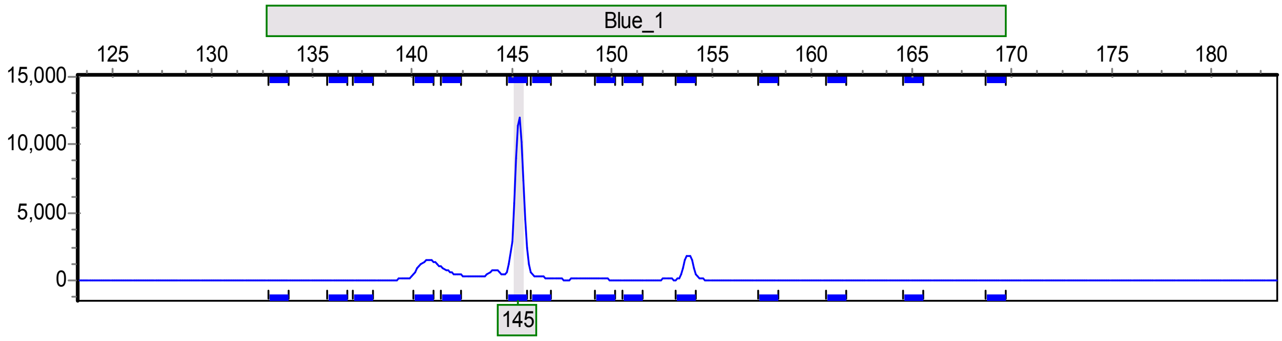

No	Size	Height	Area	Marker	Allele	Difference	Quality	Score	Allele Comments	Sample Comments
1	145.4	11914	63736	Blue_1	145	0.1	Pass	500.0		

Sample 60: Run date and time: 09/15/2020 - 21:11:04 -> 09/15/2020 - 21:56:47

Dye: Blue - 1 peaks - 63.fsa

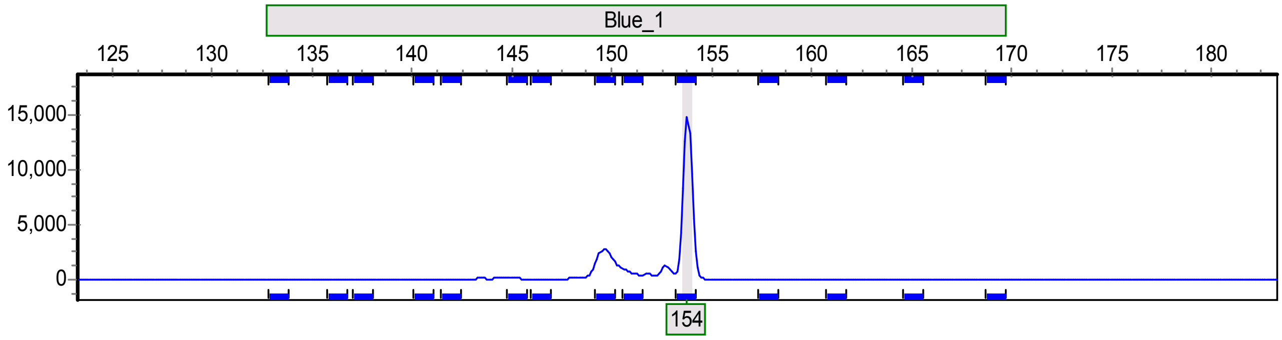

No	Size	Height	Area	Marker	Allele	Difference	Quality	Score	Allele Comments	Sample Comments
1	153.8	14726	69055	Blue_1	154	0.0	Pass	500.0		

Sample 61: Run date and time: 09/15/2020 - 21:11:04 -> 09/15/2020 - 21:56:47

Dye: Blue - 1 peaks - 64.fsa

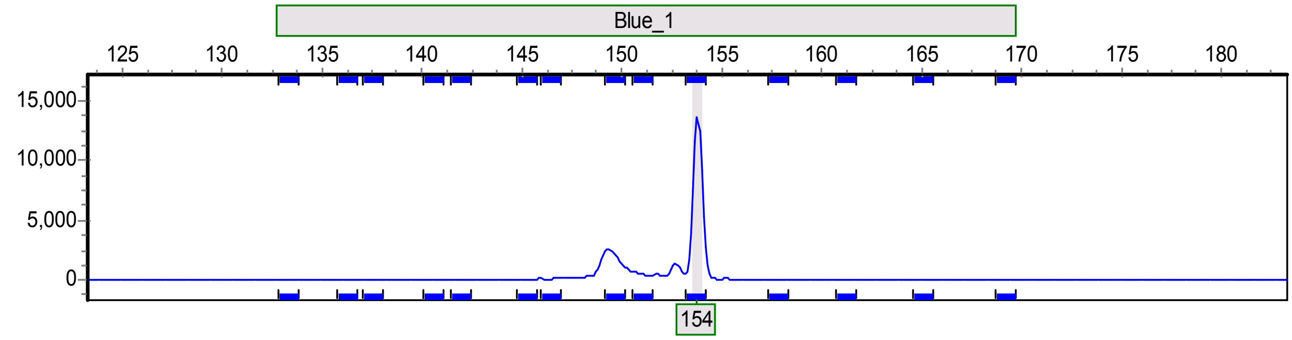

No	Size	Height	Area	Marker	Allele	Difference	Quality	Score	Allele Comments	Sample Comments
1	153.8	13513	64573	Blue_1	154	0.0	Pass	500.0		

Sample 62: Run date and time: 09/15/2020 - 21:11:04 -> 09/15/2020 - 21:56:47

Dye: Blue - 1 peaks - 65.fsa

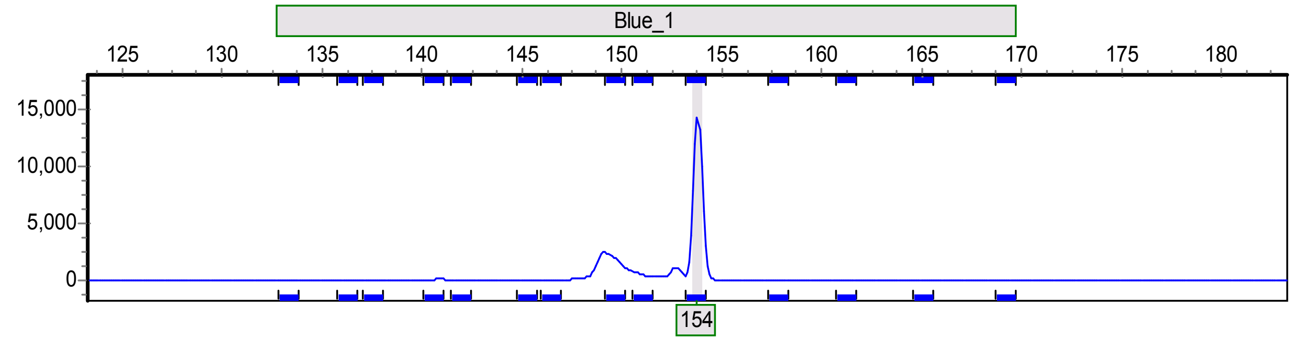

No	Size	Height	Area	Marker	Allele	Difference	Quality	Score	Allele Comments	Sample Comments
1	153.8	14279	69233	Blue_1	154	0.0	Pass	500.0		

Sample 63: Run date and time: 09/15/2020 - 21:11:04 -> 09/15/2020 - 21:56:47

Dye: Blue - 1 peaks - 66.fsa

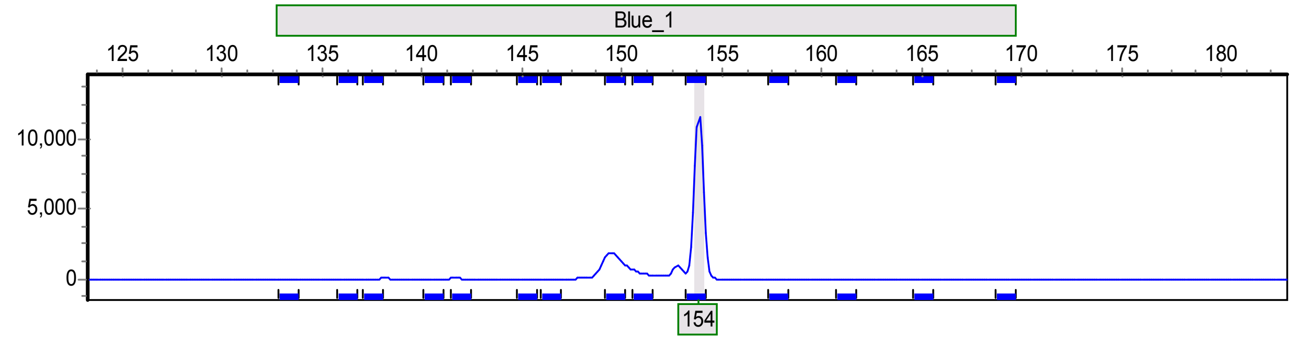

No	Size	Height	Area	Marker	Allele	Difference	Quality	Score	Allele Comments	Sample Comments
1	153.9	11457	55499	Blue_1	154	0.1	Pass	500.0		

Sample 64: Run date and time: 09/15/2020 - 21:11:04 -> 09/15/2020 - 21:56:47

Dye: Blue - 1 peaks - 67.fsa

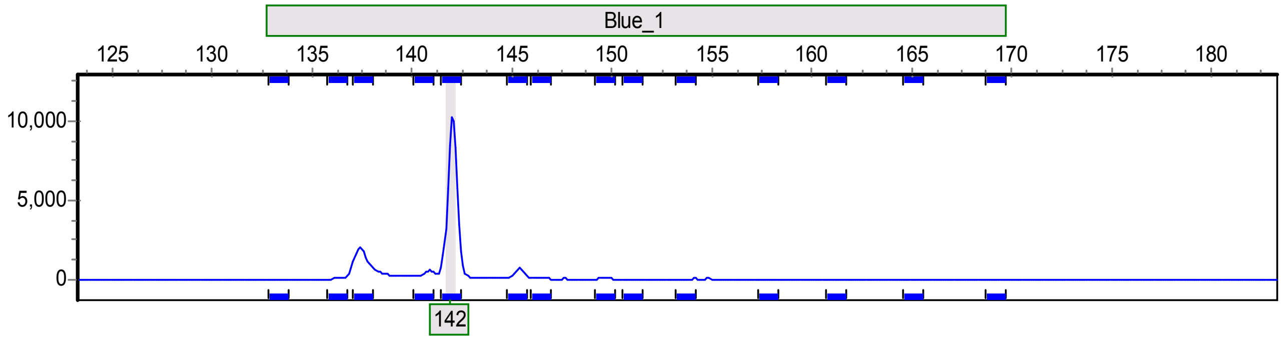

No	Size	Height	Area	Marker	Allele	Difference	Quality	Score	Allele Comments	Sample Comments
1	142.0	10170	55874	Blue_1	142	0.0	Pass	500.0		

Sample 65: Run date and time: 09/15/2020 - 21:11:04 -> 09/15/2020 - 21:56:47

Dye: Blue - 1 peaks - 68.fsa

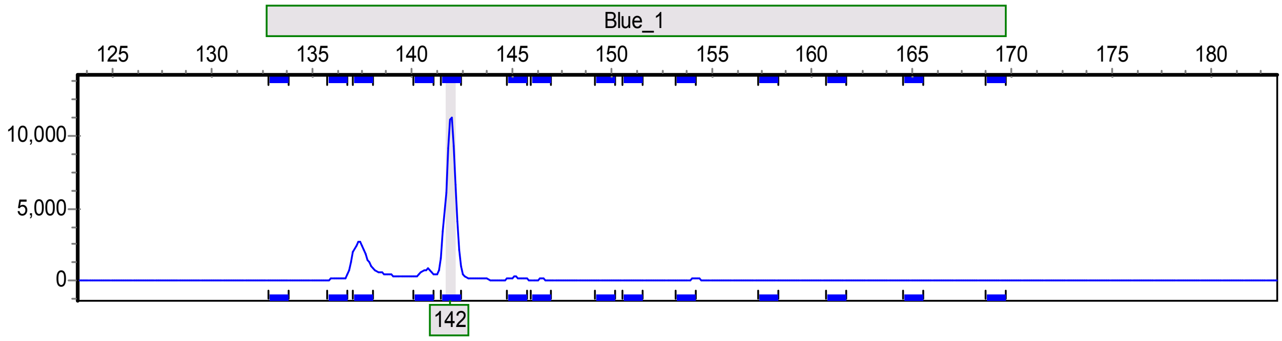

No	Size	Height	Area	Marker	Allele	Difference	Quality	Score	Allele Comments	Sample Comments
1	142.0	11223	62171	Blue_1	142	0.0	Pass	500.0		

Sample 66: Run date and time: 09/15/2020 - 21:11:04 -> 09/15/2020 - 21:56:47

Dye: Blue - 1 peaks - 69.fsa

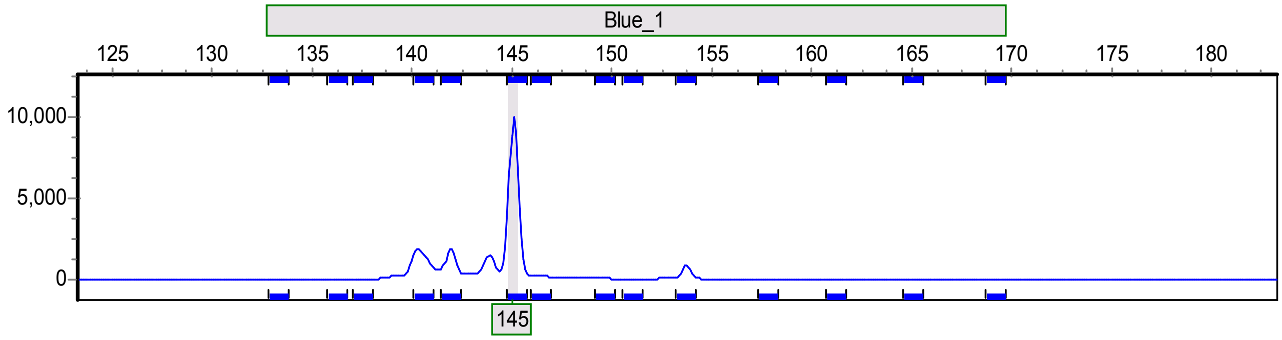

No	Size	Height	Area	Marker	Allele	Difference	Quality	Score	Allele Comments	Sample Comments
1	145.1	9930	52437	Blue_1	145	0.2	Pass	500.0		

Allele Report

Sample 67: Run date and time: 09/15/2020 - 21:11:04 -> 09/15/2020 - 21:56:47

Dye: Blue - 1 peaks - 7.fsa

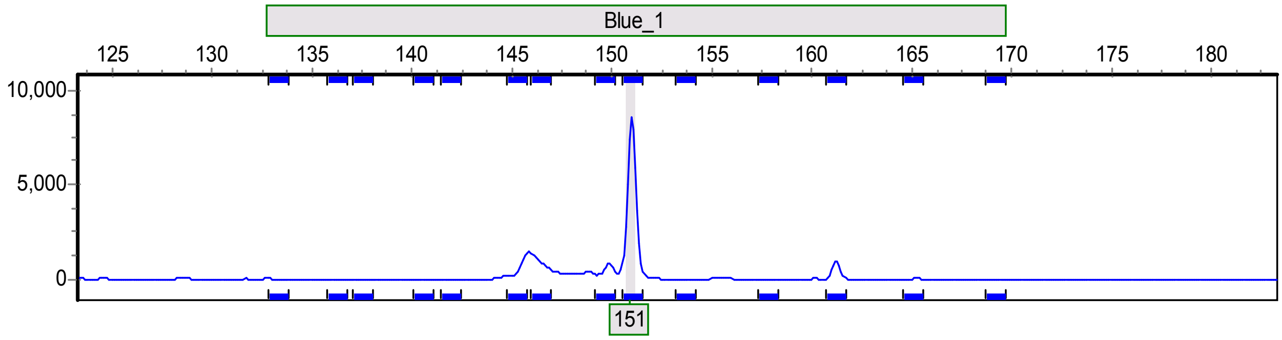

No	Size	Height	Area	Marker	Allele	Difference	Quality	Score	Allele Comments	Sample Comments
1	151.0	8508	42617	Blue_1	151	0.1	Pass	500.0		

Sample 68: Run date and time: 09/15/2020 - 21:11:04 -> 09/15/2020 - 21:56:47

Dye: Blue - 1 peaks - 70.fsa

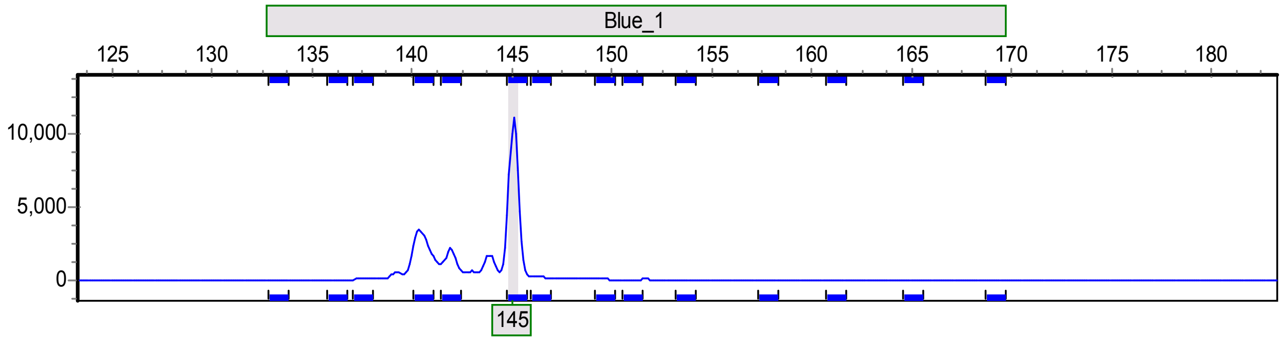

No	Size	Height	Area	Marker	Allele	Difference	Quality	Score	Allele Comments	Sample Comments
1	145.1	11015	58401	Blue_1	145	0.2	Pass	500.0		

Sample 69: Run date and time: 09/15/2020 - 21:11:04 -> 09/15/2020 - 21:56:47

Dye: Blue - 1 peaks - 71.fsa

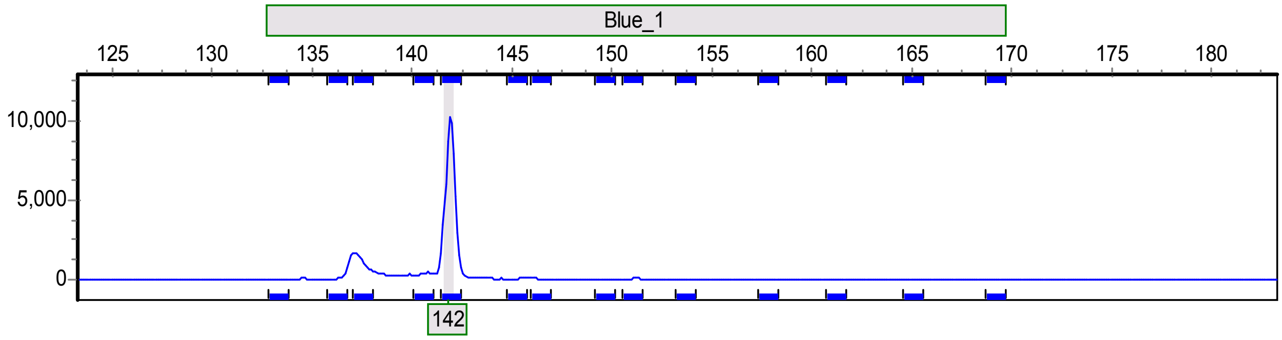

No	Size	Height	Area	Marker	Allele	Difference	Quality	Score	Allele Comments	Sample Comments
1	141.9	10195	54928	Blue_1	142	0.1	Pass	500.0		

Sample 70: Run date and time: 09/15/2020 - 21:11:04 -> 09/15/2020 - 21:56:47

Dye: Blue - 1 peaks - 72.fsa

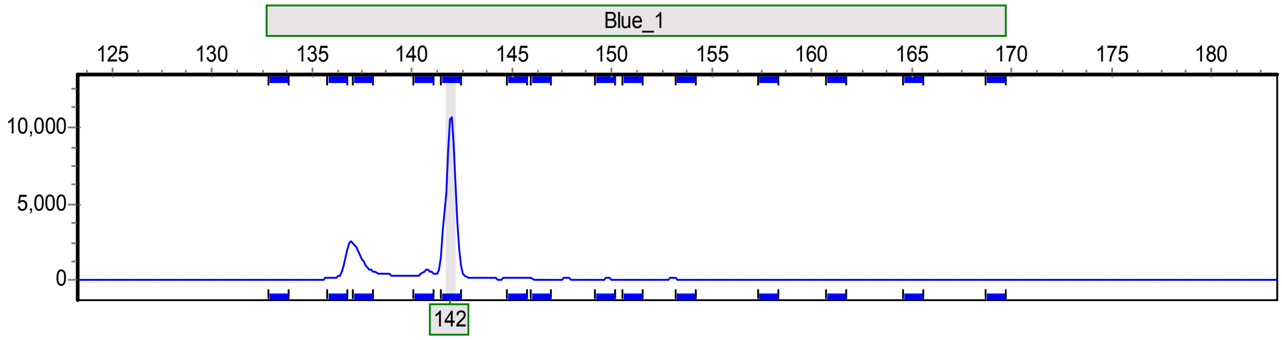

No	Size	Height	Area	Marker	Allele	Difference	Quality	Score	Allele Comments	Sample Comments
1	142.0	10654	58765	Blue_1	142	0.0	Pass	500.0		

Sample 71: Run date and time: 09/15/2020 - 21:11:04 -> 09/15/2020 - 21:56:47

Dye: Blue - 1 peaks - 73.fsa

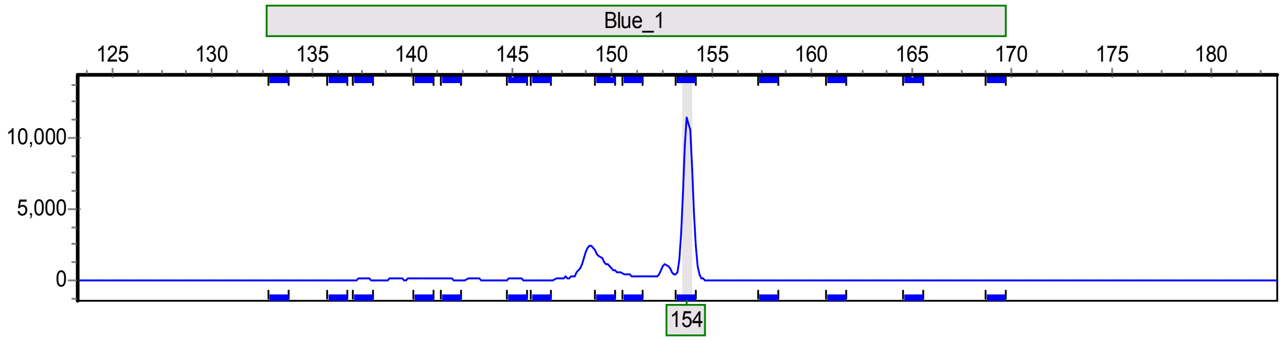

No	Size	Height	Area	Marker	Allele	Difference	Quality	Score	Allele Comments	Sample Comments
1	153.8	11391	54964	Blue_1	154	0.0	Pass	500.0		

Sample 72: Run date and time: 09/15/2020 - 21:11:04 -> 09/15/2020 - 21:56:47

Dye: Blue - 1 peaks - 74.fsa

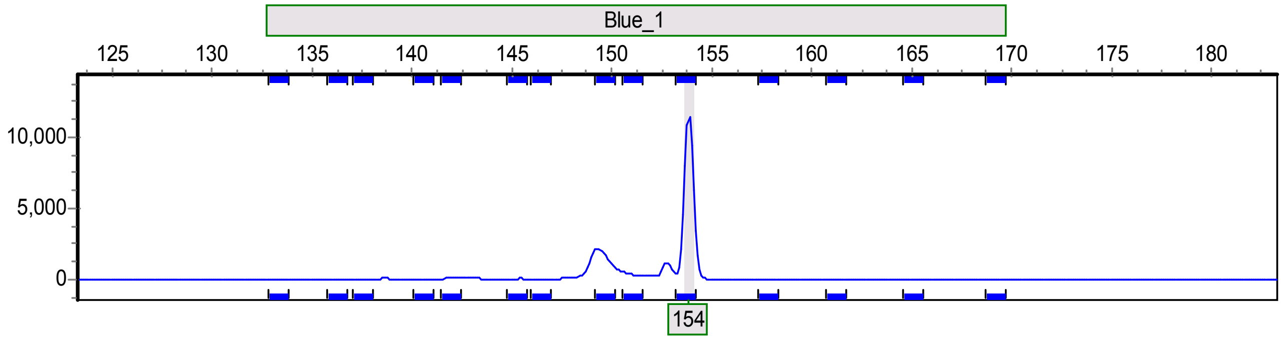

No	Size	Height	Area	Marker	Allele	Difference	Quality	Score	Allele Comments	Sample Comments
1	153.9	11413	55506	Blue_1	154	0.1	Pass	500.0		

Allele Report

Sample 73: Run date and time: 09/15/2020 - 21:11:04 -> 09/15/2020 - 21:56:47

Dye: Blue - 1 peaks - 75.fsa

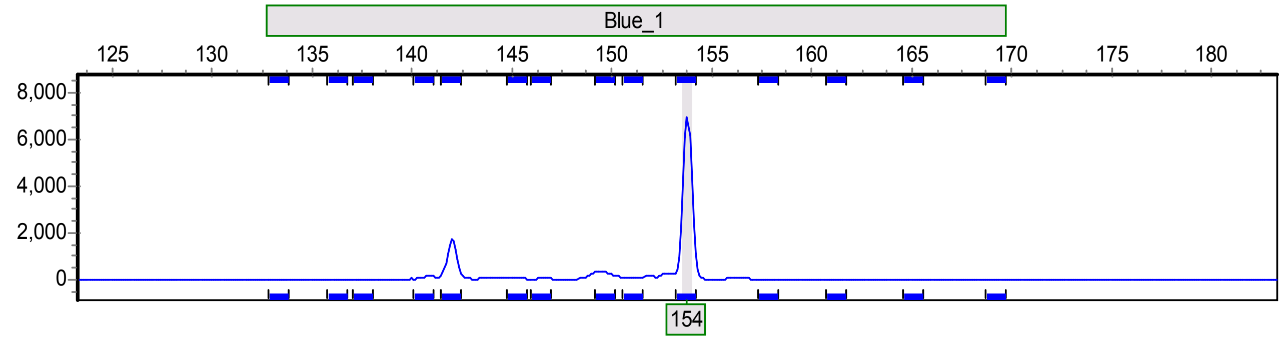

No	Size	Height	Area	Marker	Allele	Difference	Quality	Score	Allele Comments	Sample Comments
1	153.8	6951	33018	Blue_1	154	0.0	Pass	500.0		

Sample 74: Run date and time: 09/15/2020 - 21:11:04 -> 09/15/2020 - 21:56:47

Dye: Blue - 1 peaks - 76.fsa

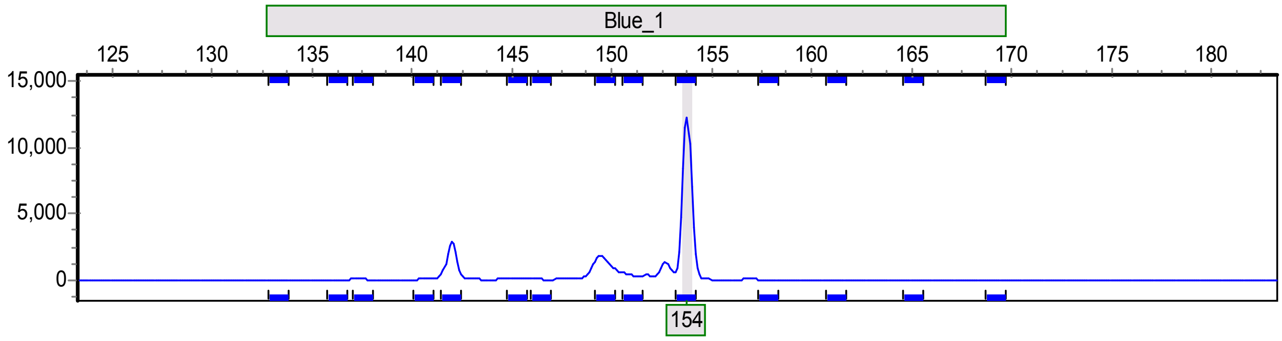

No	Size	Height	Area	Marker	Allele	Difference	Quality	Score	Allele Comments	Sample Comments
1	153.8	12178	59391	Blue_1	154	0.0	Pass	500.0		

Sample 75: Run date and time: 09/15/2020 - 21:11:04 -> 09/15/2020 - 21:56:47

Dye: Blue - 2 peaks - 77.fsa

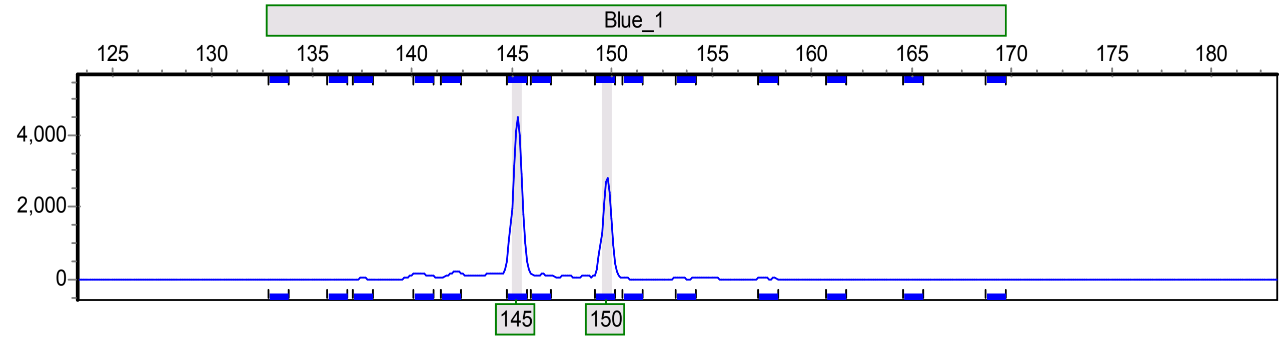

No	Size	Height	Area	Marker	Allele	Difference	Quality	Score	Allele Comments	Sample Comments
1	145.3	4469	23829	Blue_1	145	0.0	Pass	500.0		
2	149.8	2810	14579	Blue_1	150	0.1	Pass	500.0		

Sample 76: Run date and time: 09/15/2020 - 21:11:04 -> 09/15/2020 - 21:56:47

Dye: Blue - 1 peaks - 78.fsa

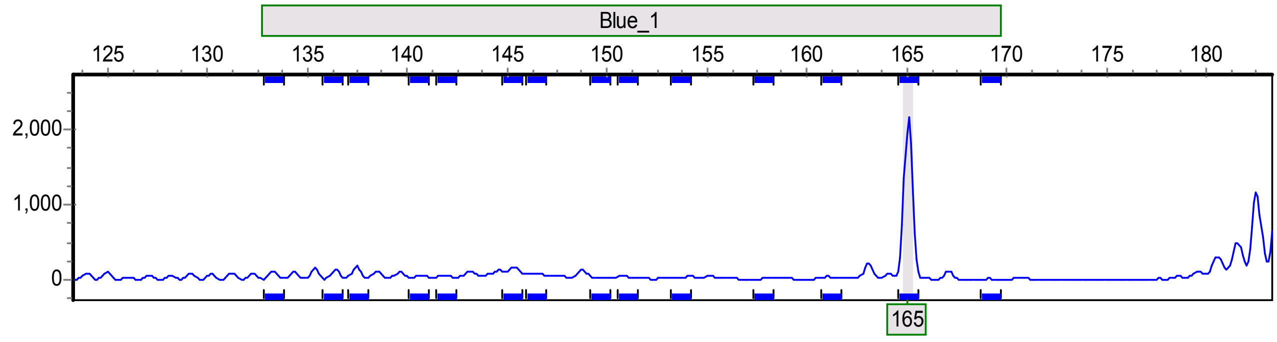

No	Size	Height	Area	Marker	Allele	Difference	Quality	Score	Allele Comments	Sample Comments
1	165.1	2148	9832	Blue_1	165	0.0	Pass	337.9		

Sample 77: Run date and time: 09/15/2020 - 21:11:04 -> 09/15/2020 - 21:56:47

Dye: Blue - 2 peaks - 79.fsa

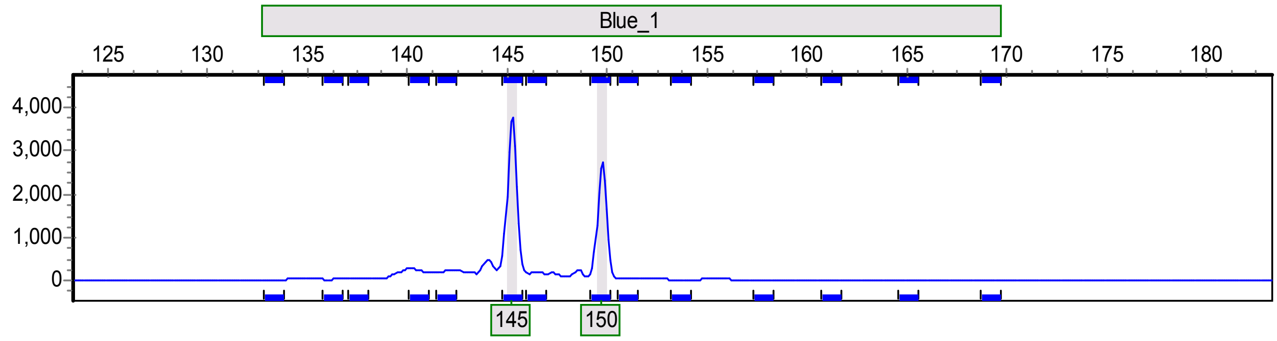

No	Size	Height	Area	Marker	Allele	Difference	Quality	Score	Allele Comments	Sample Comments
1	145.3	3747	20470	Blue_1	145	0.0	Pass	500.0		
2	149.8	2714	14198	Blue_1	150	0.1	Pass	500.0		

Sample 78: Run date and time: 09/15/2020 - 21:11:04 -> 09/15/2020 - 21:56:47

Dye: Blue - 0 peaks - 8.fsa

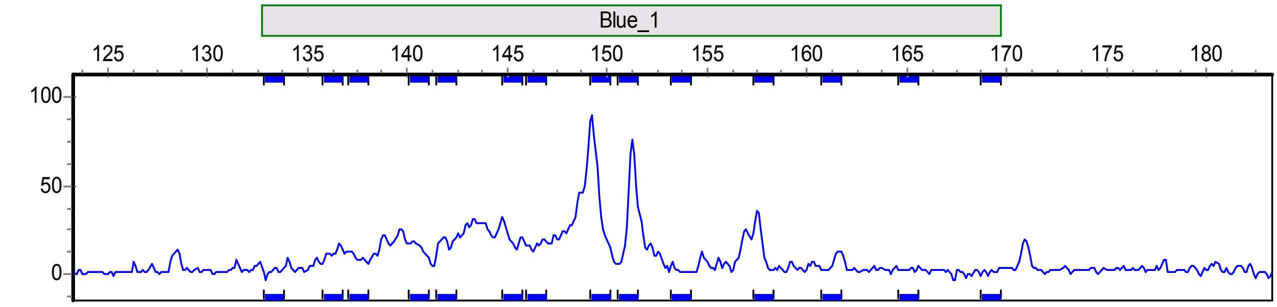

No	Size	Height	Area	Marker	Allele	Difference	Quality	Score	Allele Comments	Sample Comments

Sample 79: Run date and time: 09/15/2020 - 21:11:04 -> 09/15/2020 - 21:56:47

Dye: Blue - 2 peaks - 80.fsa

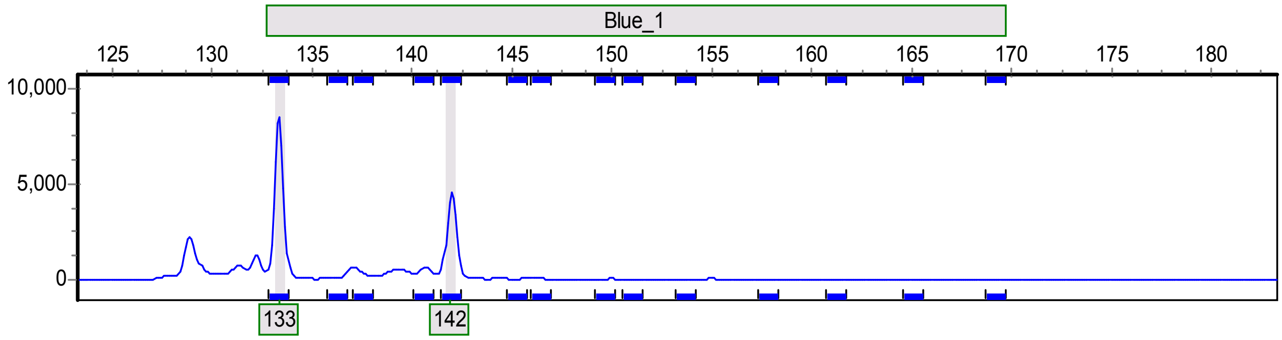

No	Size	Height	Area	Marker	Allele	Difference	Quality	Score	Allele Comments	Sample Comments
1	133.4	8484	42621	Blue_1	133	0.0	Pass	500.0		
2	142.0	4626	25595	Blue_1	142	0.0	Pass	500.0		

Sample 80: Run date and time: 09/15/2020 - 21:11:04 -> 09/15/2020 - 21:56:47

Dye: Blue - 1 peaks - 81.fsa

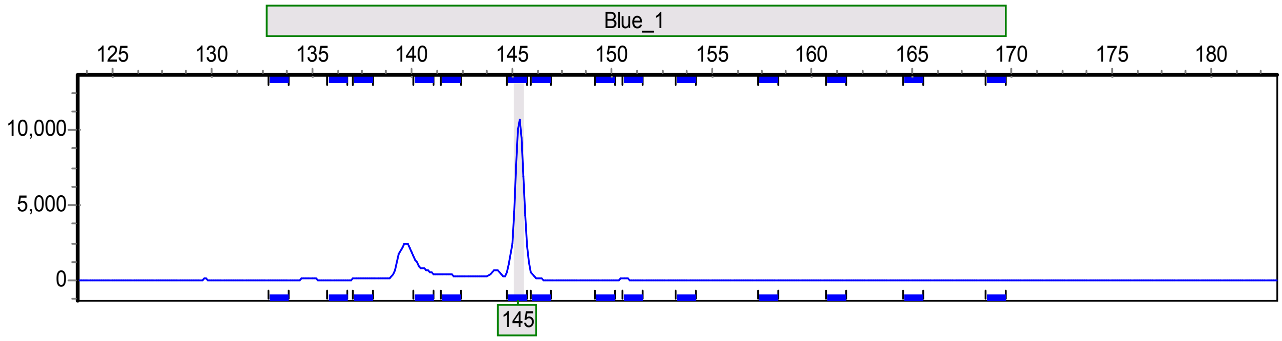

No	Size	Height	Area	Marker	Allele	Difference	Quality	Score	Allele Comments	Sample Comments
1	145.4	10767	57044	Blue_1	145	0.1	Pass	500.0		

Sample 81: Run date and time: 09/15/2020 - 21:11:04 -> 09/15/2020 - 21:56:47

Dye: Blue - 2 peaks - 82.fsa

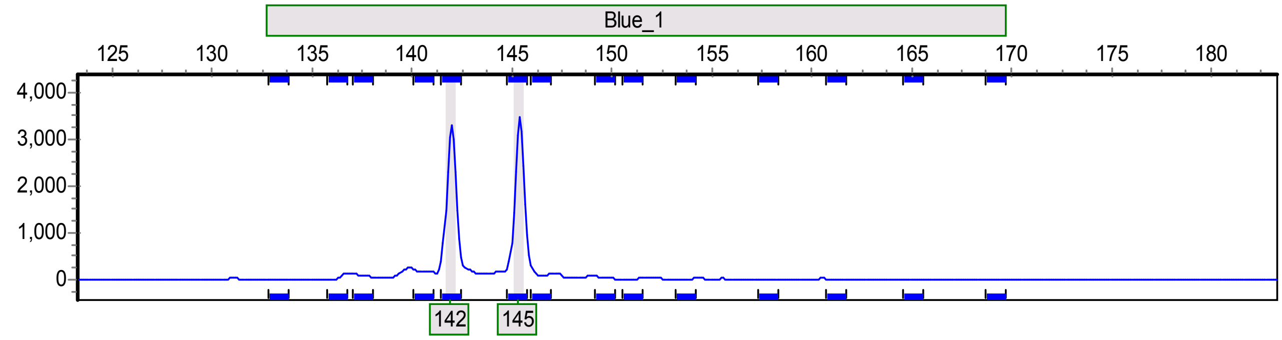

No	Size	Height	Area	Marker	Allele	Difference	Quality	Score	Allele Comments	Sample Comments
1	142.0	3295	18389	Blue_1	142	0.0	Pass	500.0		
2	145.4	3461	19029	Blue_1	145	0.1	Pass	500.0		

Sample 82: Run date and time: 09/15/2020 - 21:11:04 -> 09/15/2020 - 21:56:47

Dye: Blue - 1 peaks - 83.fsa

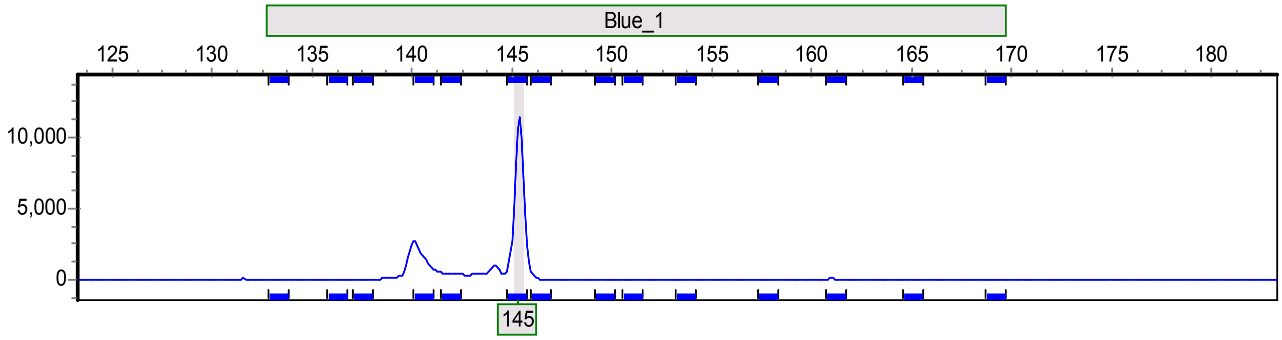

No	Size	Height	Area	Marker	Allele	Difference	Quality	Score	Allele Comments	Sample Comments
1	145.4	11319	60549	Blue_1	145	0.1	Pass	500.0		

Sample 83: Run date and time: 09/15/2020 - 21:11:04 -> 09/15/2020 - 21:56:47

Dye: Blue - 2 peaks - 84.fsa

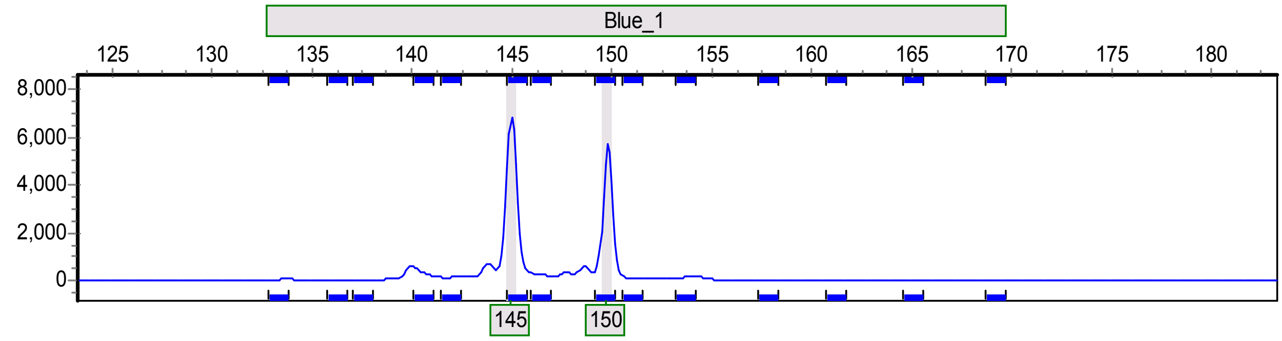

No	Size	Height	Area	Marker	Allele	Difference	Quality	Score	Allele Comments	Sample Comments
1	145.0	6787	39068	Blue_1	145	0.3	Pass	500.0		
2	149.8	5677	30446	Blue_1	150	0.1	Pass	500.0		

Sample 84: Run date and time: 09/15/2020 - 21:11:04 -> 09/15/2020 - 21:56:47

Dye: Blue - 2 peaks - 85.fsa

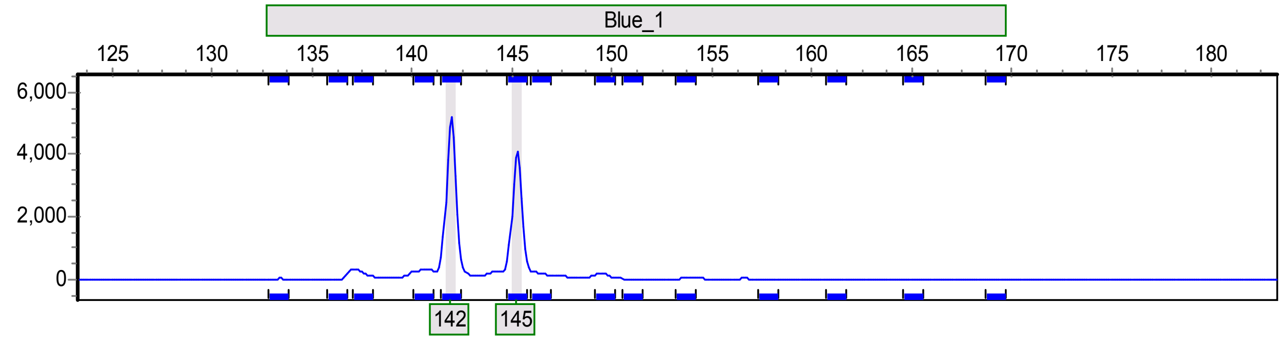

No	Size	Height	Area	Marker	Allele	Difference	Quality	Score	Allele Comments	Sample Comments
1	142.0	5158	28532	Blue_1	142	0.0	Pass	500.0		
2	145.3	4068	22718	Blue_1	145	0.0	Pass	500.0		

Sample 85: Run date and time: 09/15/2020 - 21:11:04 -> 09/15/2020 - 21:56:47

Dye: Blue - 1 peaks - 86.fsa

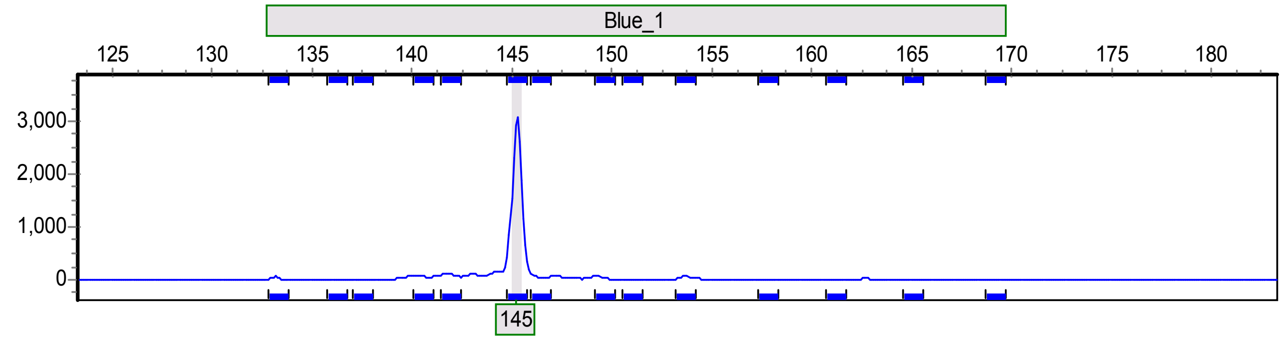

No	Size	Height	Area	Marker	Allele	Difference	Quality	Score	Allele Comments	Sample Comments
1	145.3	3066	16787	Blue_1	145	0.0	Pass	500.0		

Sample 86: Run date and time: 09/15/2020 - 21:11:04 -> 09/15/2020 - 21:56:47

Dye: Blue - 1 peaks - 87.fsa

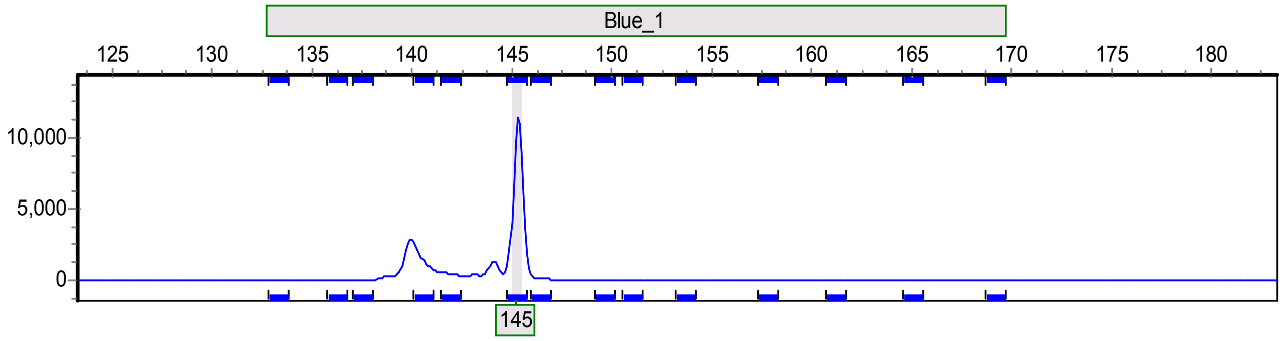

No	Size	Height	Area	Marker	Allele	Difference	Quality	Score	Allele Comments	Sample Comments
1	145.3	11305	62217	Blue_1	145	0.0	Pass	500.0		

Sample 87: Run date and time: 09/15/2020 - 21:11:04 -> 09/15/2020 - 21:56:47

Dye: Blue - 1 peaks - 88.fsa

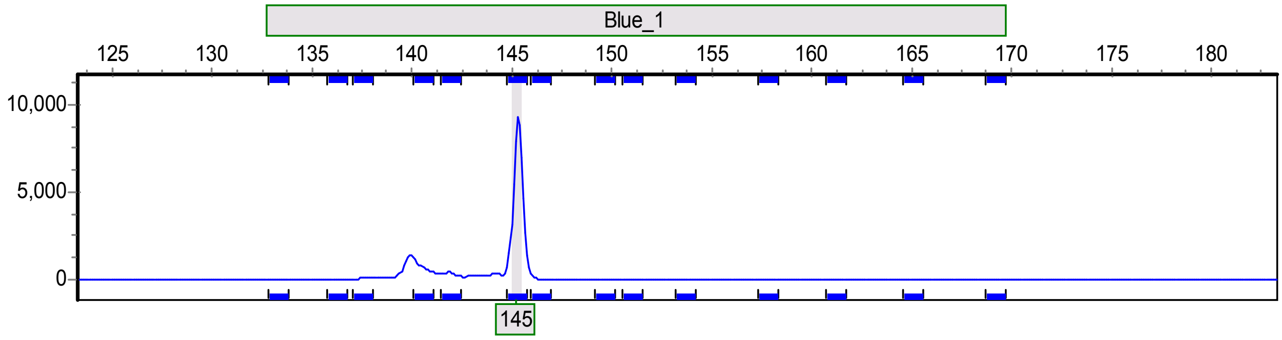

No	Size	Height	Area	Marker	Allele	Difference	Quality	Score	Allele Comments	Sample Comments
1	145.3	9285	49923	Blue_1	145	0.0	Pass	500.0		

Sample 88: Run date and time: 09/15/2020 - 21:11:04 -> 09/15/2020 - 21:56:47

Dye: Blue - 1 peaks - 89.fsa

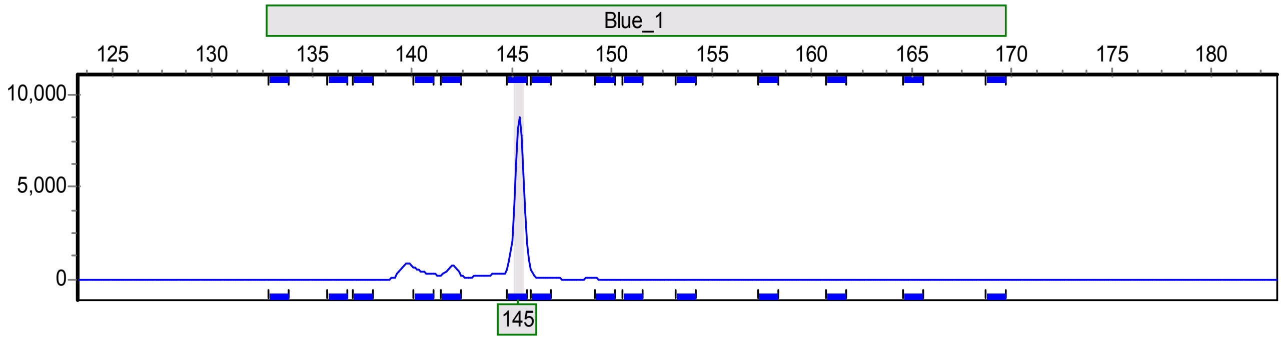

No	Size	Height	Area	Marker	Allele	Difference	Quality	Score	Allele Comments	Sample Comments
1	145.4	8731	47576	Blue_1	145	0.1	Pass	500.0		

Sample 89: Run date and time: 09/15/2020 - 21:11:04 -> 09/15/2020 - 21:56:47

Dye: Blue - 1 peaks - 9.fsa

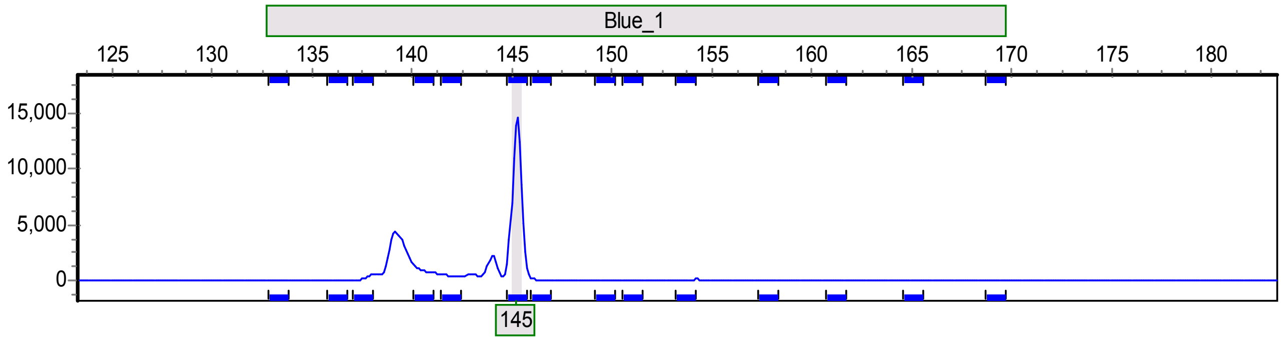

No	Size	Height	Area	Marker	Allele	Difference	Quality	Score	Allele Comments	Sample Comments
1	145.3	14474	76063	Blue_1	145	0.0	Pass	500.0		

Sample 90: Run date and time: 09/15/2020 - 21:11:04 -> 09/15/2020 - 21:56:47

Dye: Blue - 2 peaks - 90.fsa

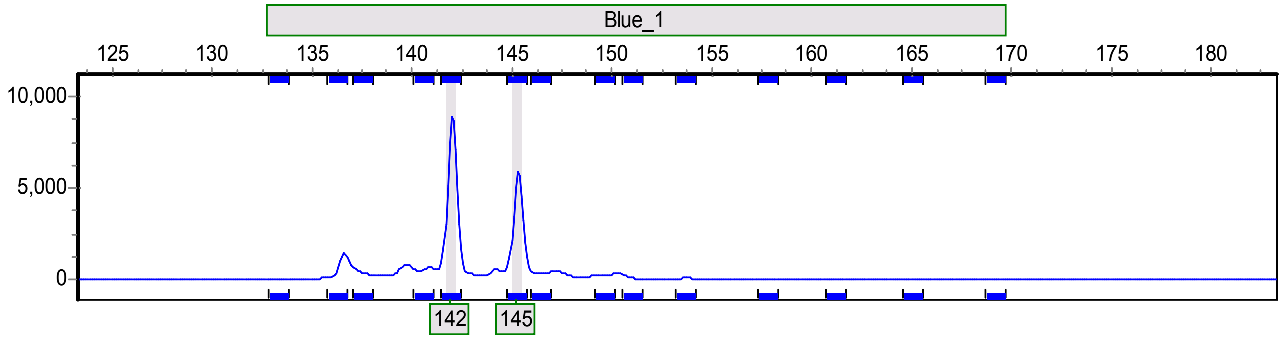

No	Size	Height	Area	Marker	Allele	Difference	Quality	Score	Allele Comments	Sample Comments
1	142.0	8863	49466	Blue_1	142	0.0	Pass	500.0		
2	145.3	5864	33019	Blue_1	145	0.0	Pass	500.0		

Sample 91: Run date and time: 09/15/2020 - 21:11:04 -> 09/15/2020 - 21:56:47

Dye: Blue - 2 peaks - 91.fsa

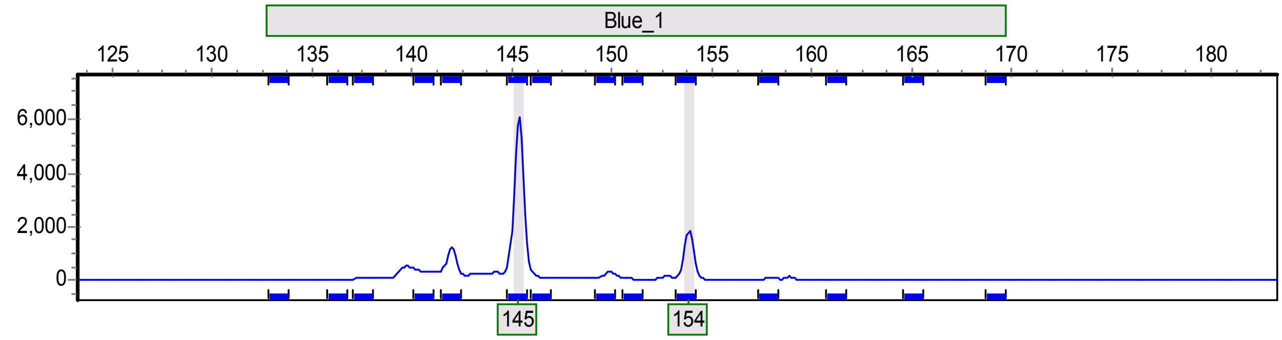

No	Size	Height	Area	Marker	Allele	Difference	Quality	Score	Allele Comments	Sample Comments
1	145.4	6072	34513	Blue_1	145	0.1	Pass	500.0		
2	153.9	1823	9164	Blue_1	154	0.1	Pass	485.9		

Sample 92: Run date and time: 09/15/2020 - 21:11:04 -> 09/15/2020 - 21:56:47

Dye: Blue - 1 peaks - 92.fsa

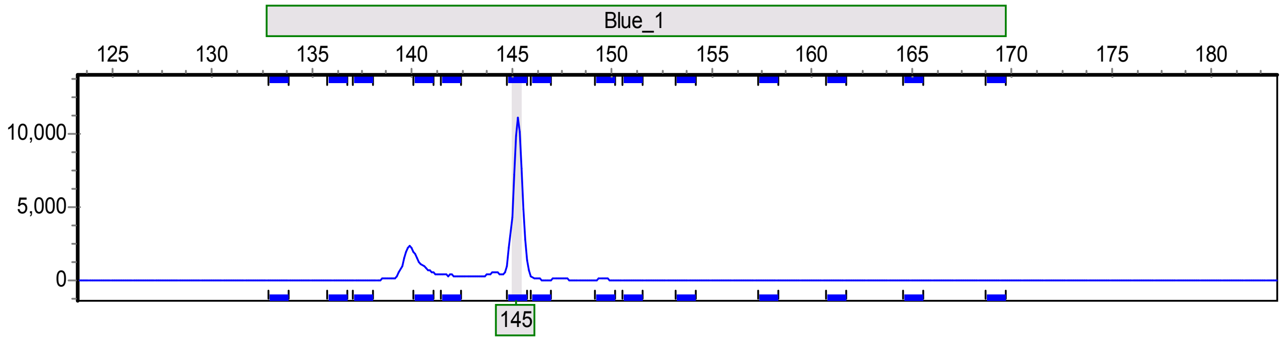

No	Size	Height	Area	Marker	Allele	Difference	Quality	Score	Allele Comments	Sample Comments
1	145.3	11025	58314	Blue_1	145	0.0	Pass	500.0		

Sample 93: Run date and time: 09/15/2020 - 21:11:04 -> 09/15/2020 - 21:56:47

Dye: Blue - 2 peaks - 93.fsa

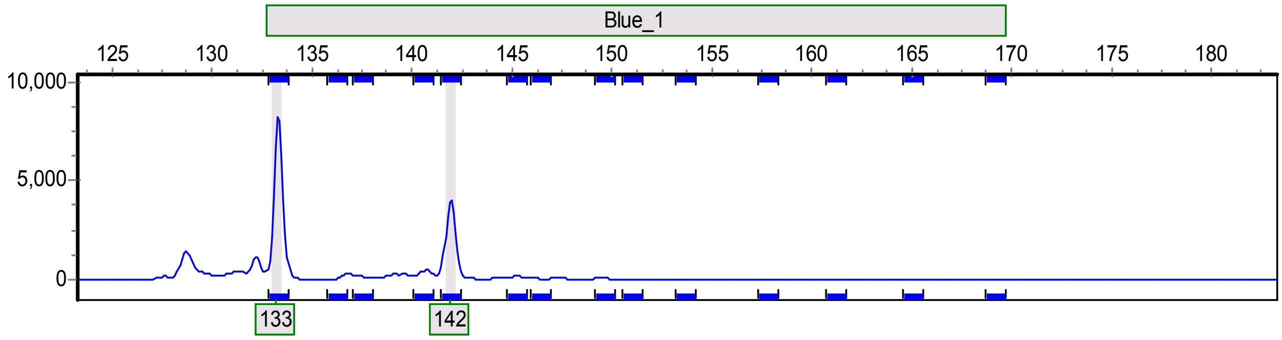

No	Size	Height	Area	Marker	Allele	Difference	Quality	Score	Allele Comments	Sample Comments
1	133.3	8186	41522	Blue_1	133	0.1	Pass	500.0		
2	142.0	3993	22201	Blue_1	142	0.0	Pass	500.0		

Sample 94: Run date and time: 09/15/2020 - 21:11:04 -> 09/15/2020 - 21:56:47

Dye: Blue - 3 peaks - 94.fsa

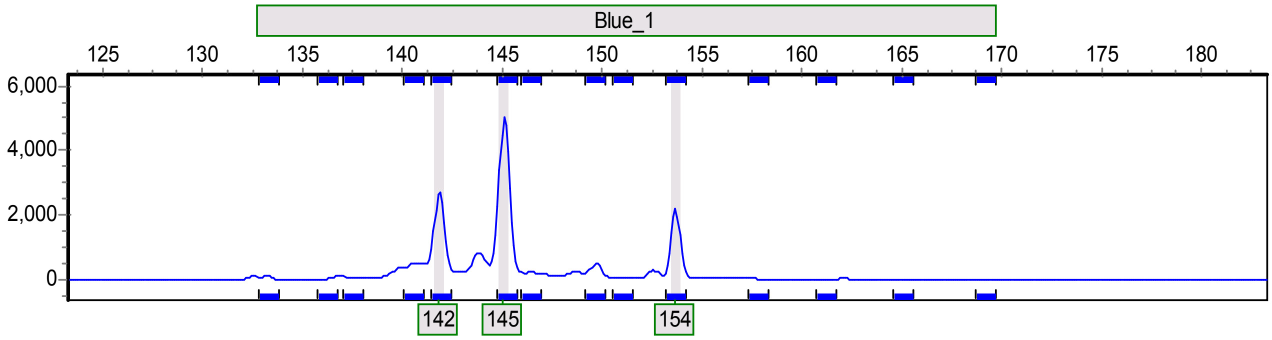

No	Size	Height	Area	Marker	Allele	Difference	Quality	Score	Allele Comments	Sample Comments
1	141.9	2709	17003	Blue_1	142	0.1	Pass	500.0		
2	145.1	5001	30630	Blue_1	145	0.2	Pass	500.0		
3	153.7	2181	10996	Blue_1	154	0.1	Pass	500.0		

Sample 95: Run date and time: 09/15/2020 - 21:11:04 -> 09/15/2020 - 21:56:47

Dye: Blue - 3 peaks - 95.fsa

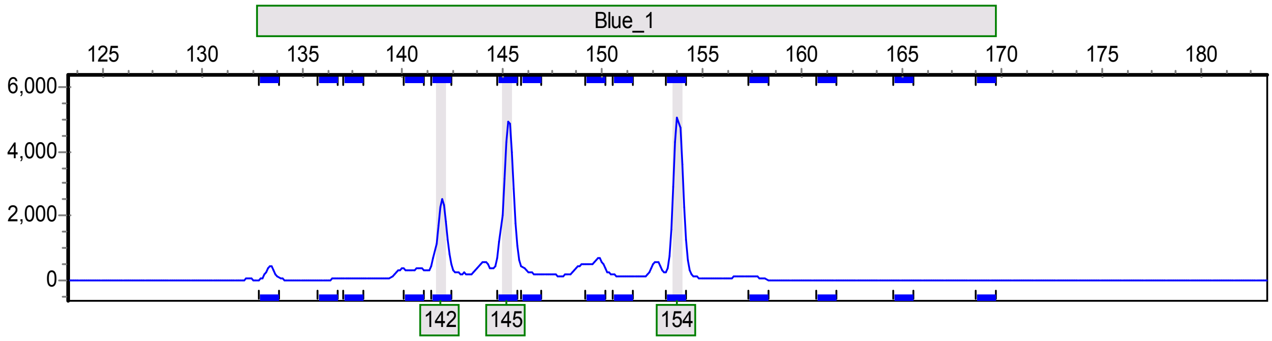

No	Size	Height	Area	Marker	Allele	Difference	Quality	Score	Allele Comments	Sample Comments
1	142.0	2508	14995	Blue_1	142	0.0	Pass	500.0		
2	145.3	4951	29711	Blue_1	145	0.0	Pass	500.0		
3	153.8	5020	25476	Blue_1	154	0.0	Pass	500.0		

Sample 96: Run date and time: 09/15/2020 - 21:11:04 -> 09/15/2020 - 21:56:47

Dye: Blue - 2 peaks - 96.fsa

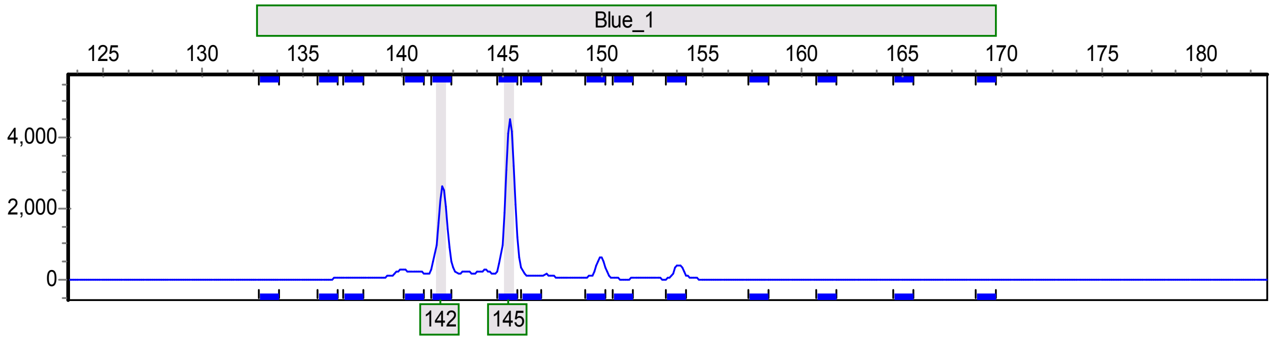

No	Size	Height	Area	Marker	Allele	Difference	Quality	Score	Allele Comments	Sample Comments
1	142.0	2619	14966	Blue_1	142	0.0	Pass	500.0		
2	145.4	4551	24908	Blue_1	145	0.1	Pass	500.0		
