# Supplementary material for: Genetic diversity and population structure of Polygonatum cyrtonema Hua in China using SSR markers
Source: PLoS One. 2023 Aug 31;18(8):e0290605. doi: 10.1371/journal.pone.0290605 (PMC10470896; doi:10.1371/journal.pone.0290605)
Supplement: S1 Data — (ZIP) [file pone.0290605.s002.zip › ╨┬╜¿╬─╝■╝╨ (2)/╥2╬∩38/yinwu38.pdf]

Allele Report

Sample 1: Run date and time: 09/15/2020 - 21:56:48 -> 09/15/2020 - 22:42:27

Dye: Blue - 0 peaks - 1.fsa

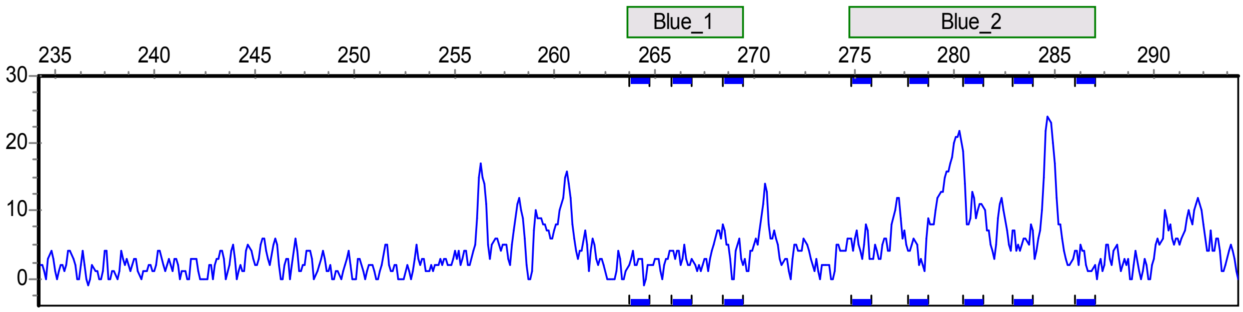

No	Size	Height	Area	Marker	Allele	Difference	Quality	Score	Allele Comments	Sample Comments

Sample 2: Run date and time: 09/15/2020 - 21:56:48 -> 09/15/2020 - 22:42:27

Dye: Blue - 0 peaks - 10.fsa

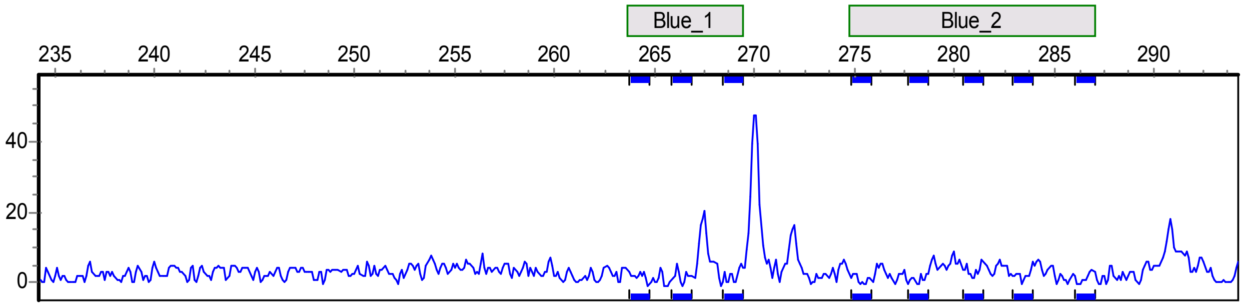

No	Size	Height	Area	Marker	Allele	Difference	Quality	Score	Allele Comments	Sample Comments

Sample 3: Run date and time: 09/15/2020 - 21:56:48 -> 09/15/2020 - 22:42:27

Dye: Blue - 0 peaks - 11.fsa

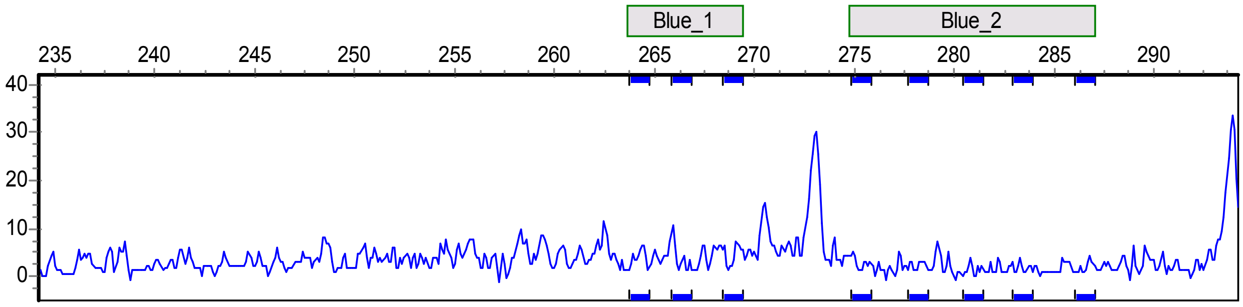

No	Size	Height	Area	Marker	Allele	Difference	Quality	Score	Allele Comments	Sample Comments

Allele Report

Sample 4: Run date and time: 09/15/2020 - 21:56:48 -> 09/15/2020 - 22:42:27

Dye: Blue - 0 peaks - 12.fsa

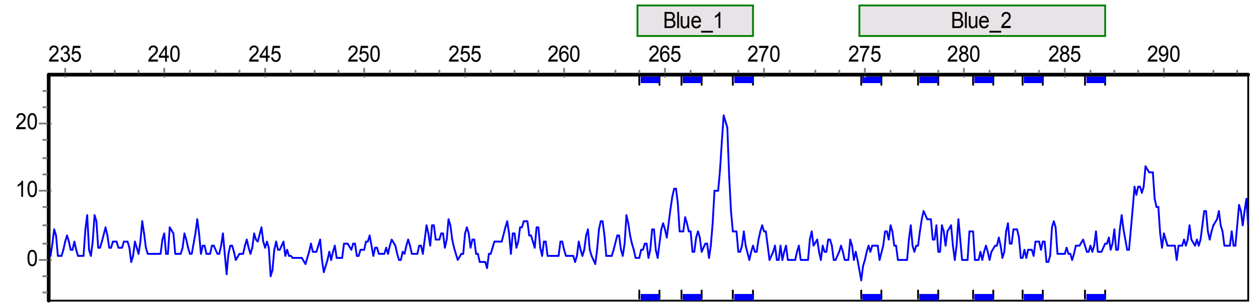

No	Size	Height	Area	Marker	Allele	Difference	Quality	Score	Allele Comments	Sample Comments

Sample 5: Run date and time: 09/15/2020 - 21:56:48 -> 09/15/2020 - 22:42:27

Dye: Blue - 0 peaks - 13.fsa

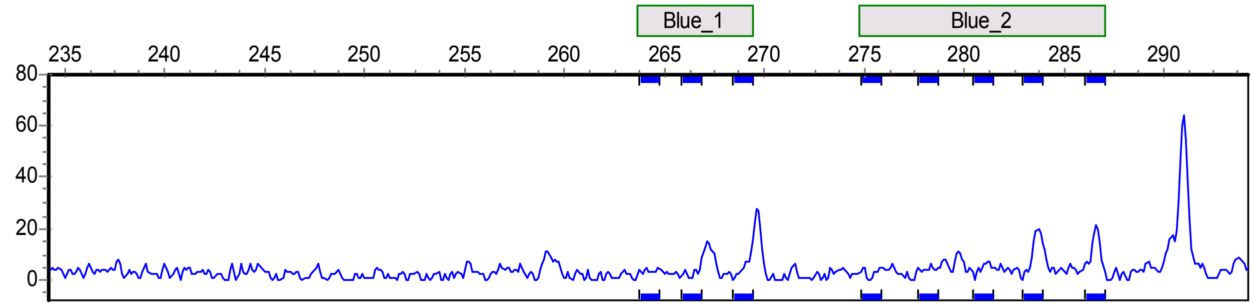

No	Size	Height	Area	Marker	Allele	Difference	Quality	Score	Allele Comments	Sample Comments

Sample 6: Run date and time: 09/15/2020 - 21:56:48 -> 09/15/2020 - 22:42:27

Dye: Blue - 0 peaks - 14.fsa

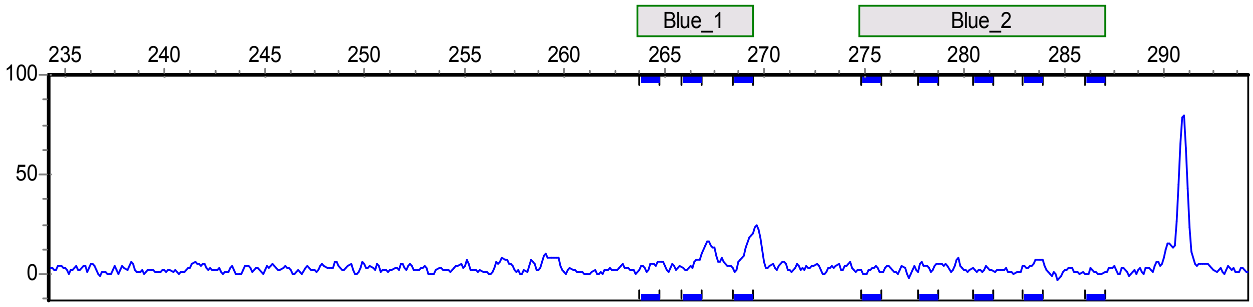

No	Size	Height	Area	Marker	Allele	Difference	Quality	Score	Allele Comments	Sample Comments

Allele Report

Sample 7: Run date and time: 09/15/2020 - 21:56:48 -> 09/15/2020 - 22:42:27

Dye: Blue - 0 peaks - 15.fsa

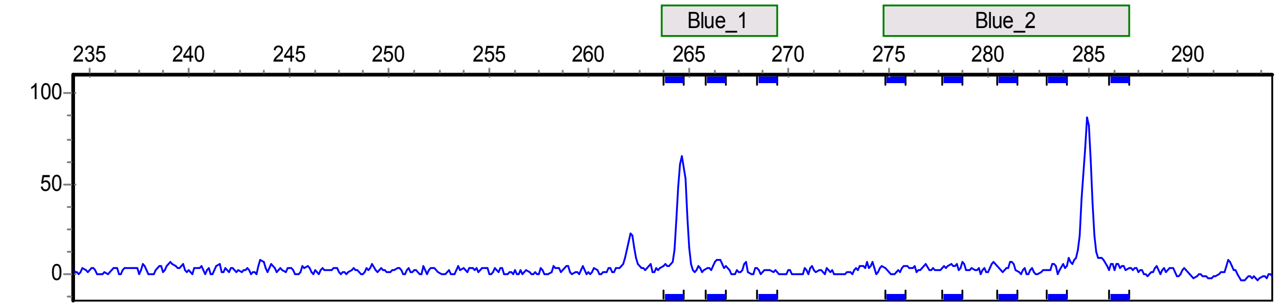

No	Size	Height	Area	Marker	Allele	Difference	Quality	Score	Allele Comments	Sample Comments

Sample 8: Run date and time: 09/15/2020 - 21:56:48 -> 09/15/2020 - 22:42:27

Dye: Blue - 1 peaks - 16.fsa

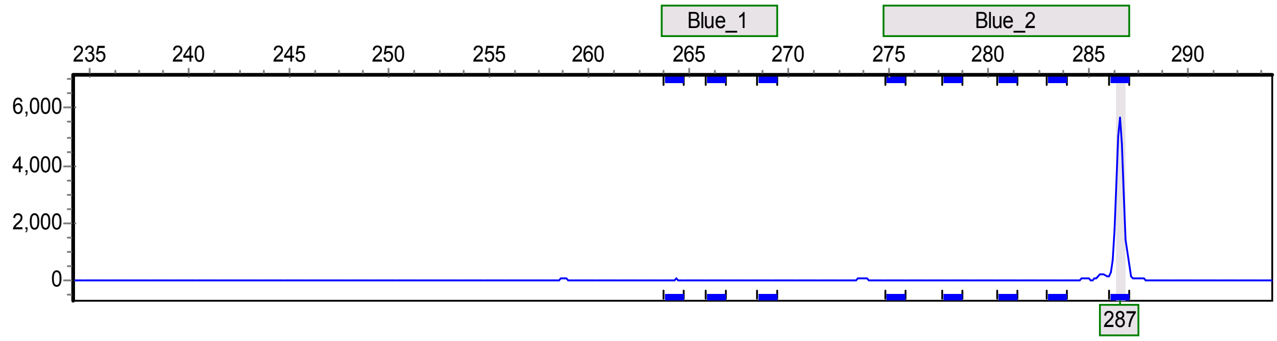

No	Size	Height	Area	Marker	Allele	Difference	Quality	Score	Allele Comments	Sample Comments
1	286.6	5641	24748	Blue_2	287	0.0	Pass	500.0		

Sample 9: Run date and time: 09/15/2020 - 21:56:48 -> 09/15/2020 - 22:42:27

Dye: Blue - 1 peaks - 17.fsa

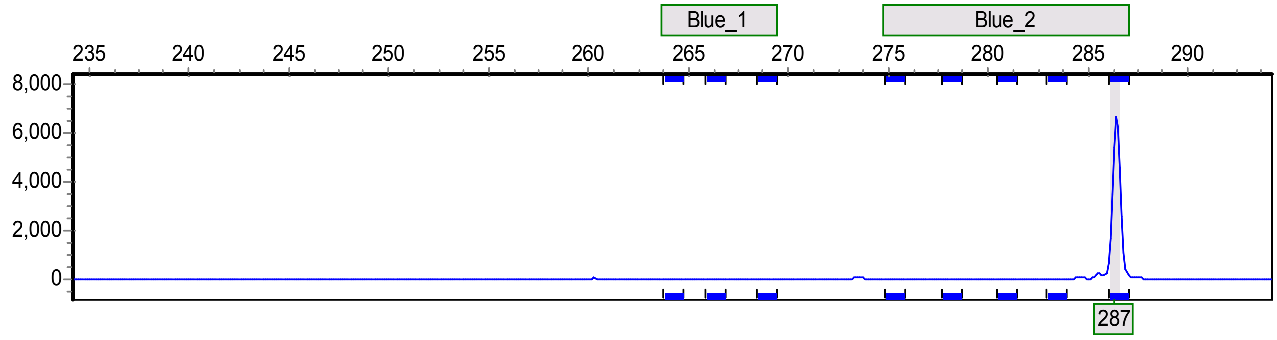

No	Size	Height	Area	Marker	Allele	Difference	Quality	Score	Allele Comments	Sample Comments
1	286.4	6659	30880	Blue_2	287	0.2	Pass	500.0		

Allele Report

Sample 10: Run date and time: 09/15/2020 - 21:56:48 -> 09/15/2020 - 22:42:27

Dye: Blue - 0 peaks - 18.fsa

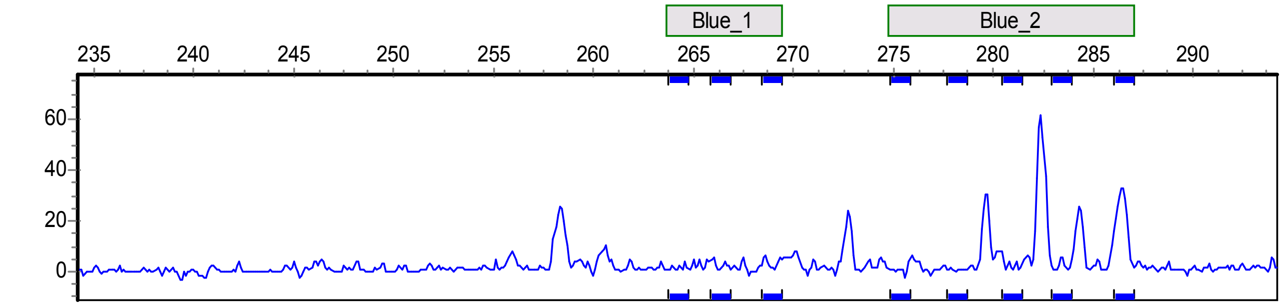

No	Size	Height	Area	Marker	Allele	Difference	Quality	Score	Allele Comments	Sample Comments

Sample 11: Run date and time: 09/15/2020 - 21:56:48 -> 09/15/2020 - 22:42:27

Dye: Blue - 2 peaks - 19.fsa

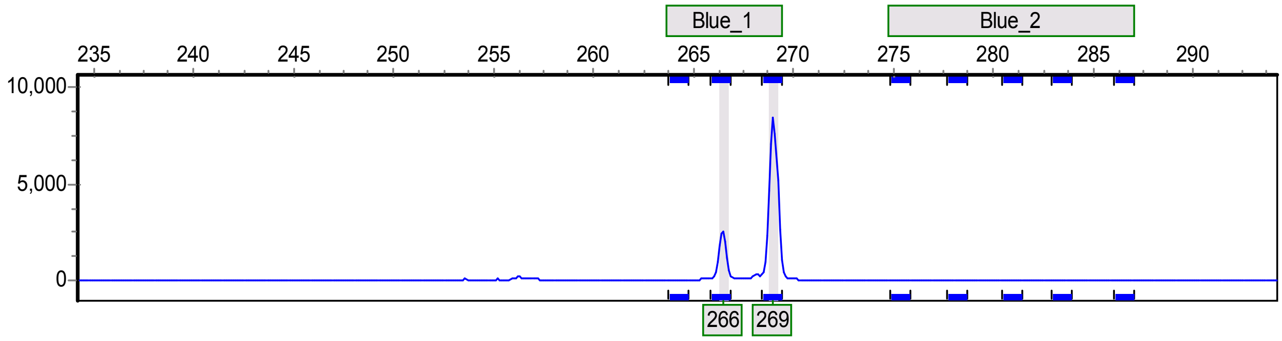

No	Size	Height	Area	Marker	Allele	Difference	Quality	Score	Allele Comments	Sample Comments
1	266.5	2487	11325	Blue_1	266	0.1	Pass	500.0		
2	269.0	8376	37511	Blue_1	269	0.0	Pass	500.0		

Sample 12: Run date and time: 09/15/2020 - 21:56:48 -> 09/15/2020 - 22:42:27

Dye: Blue - 1 peaks - 2.fsa

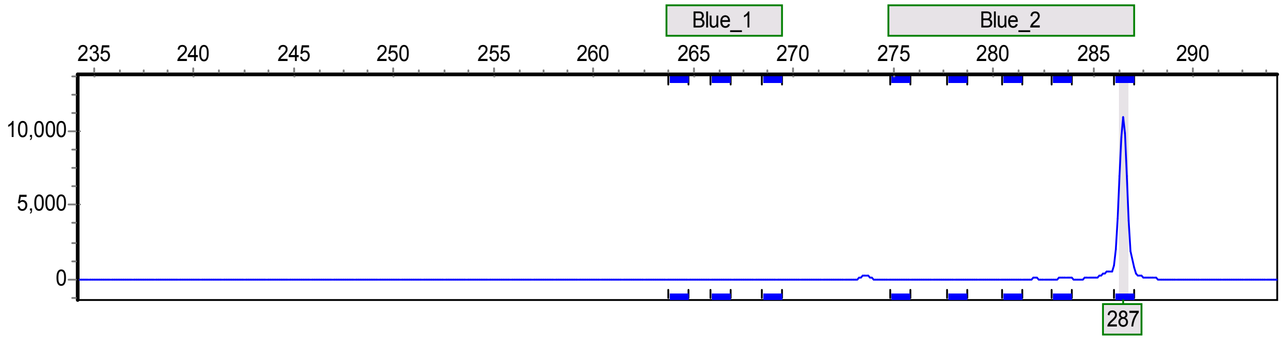

No	Size	Height	Area	Marker	Allele	Difference	Quality	Score	Allele Comments	Sample Comments
1	286.5	10860	53864	Blue_2	287	0.1	Pass	500.0		

Sample 13: Run date and time: 09/15/2020 - 21:56:48 -> 09/15/2020 - 22:42:27

Dye: Blue - 1 peaks - 20.fsa

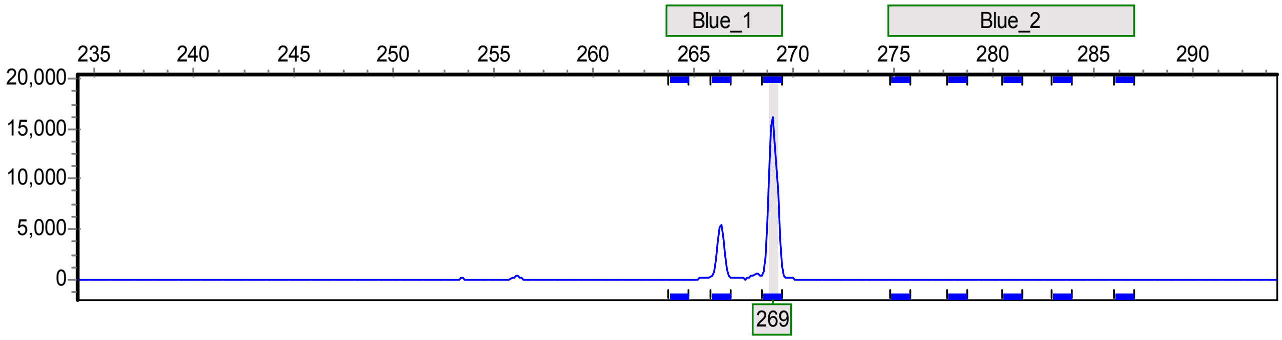

No	Size	Height	Area	Marker	Allele	Difference	Quality	Score	Allele Comments	Sample Comments
1	269.0	16015	73012	Blue_1	269	0.0	Pass	500.0		

Sample 14: Run date and time: 09/15/2020 - 21:56:48 -> 09/15/2020 - 22:42:27

Dye: Blue - 1 peaks - 21.fsa

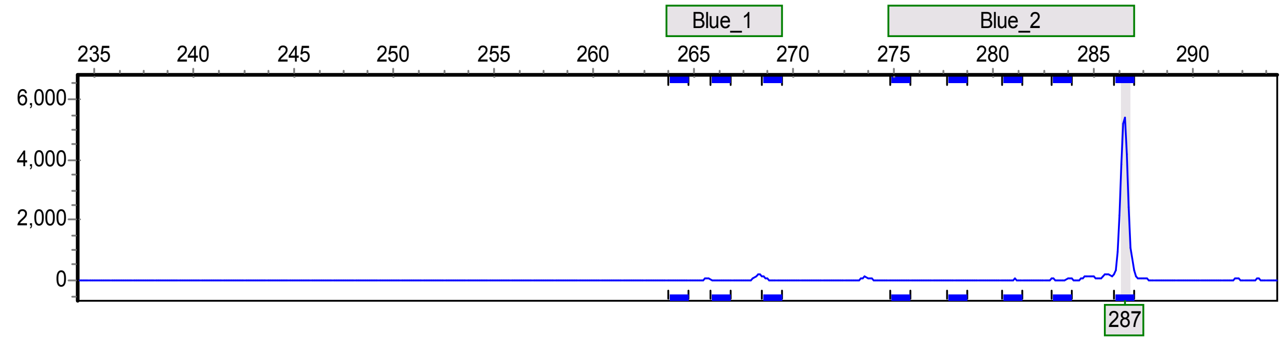

No	Size	Height	Area	Marker	Allele	Difference	Quality	Score	Allele Comments	Sample Comments
1	286.6	5363	23950	Blue_2	287	0.0	Pass	500.0		

Sample 15: Run date and time: 09/15/2020 - 21:56:48 -> 09/15/2020 - 22:42:27

Dye: Blue - 1 peaks - 22.fsa

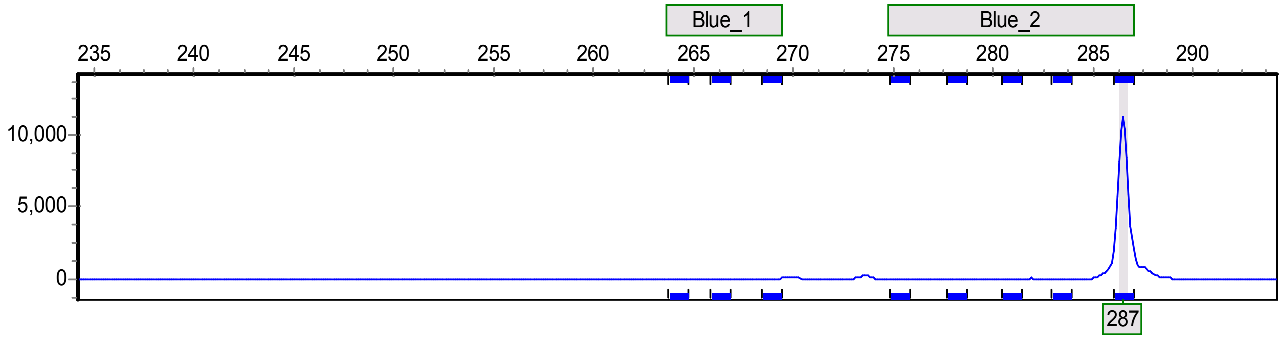

No	Size	Height	Area	Marker	Allele	Difference	Quality	Score	Allele Comments	Sample Comments
1	286.5	11158	68655	Blue_2	287	0.1	Pass	500.0		

Allele Report

Sample 16: Run date and time: 09/15/2020 - 21:56:48 -> 09/15/2020 - 22:42:27

Dye: Blue - 1 peaks - 23.fsa

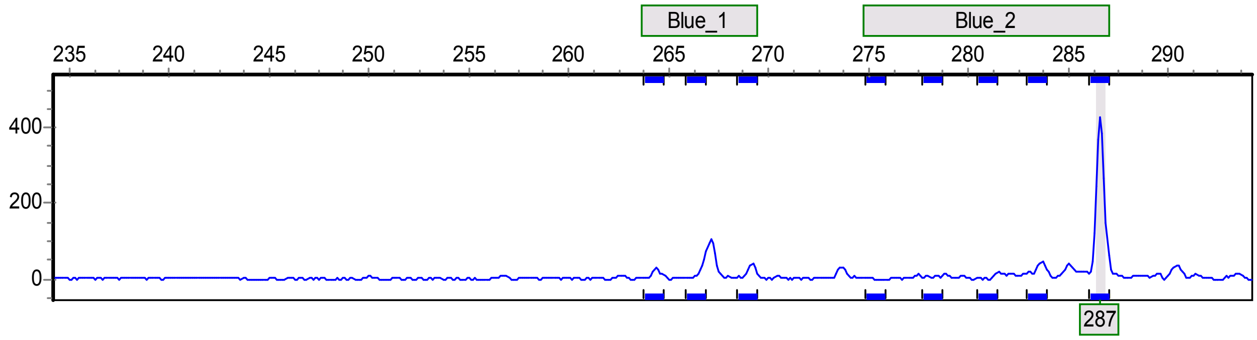

No	Size	Height	Area	Marker	Allele	Difference	Quality	Score	Allele Comments	Sample Comments
1	286.6	424	1960	Blue_2	287	0.0	Pass	63.4		

Sample 17: Run date and time: 09/15/2020 - 21:56:48 -> 09/15/2020 - 22:42:27

Dye: Blue - 0 peaks - 24.fsa

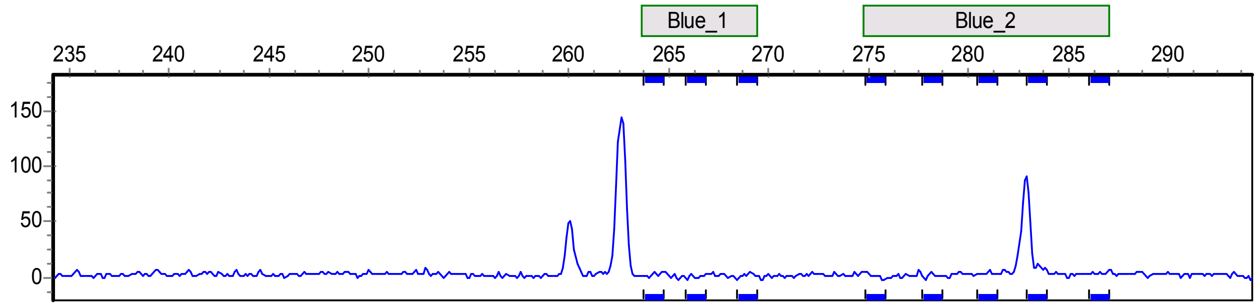

No	Size	Height	Area	Marker	Allele	Difference	Quality	Score	Allele Comments	Sample Comments

Sample 18: Run date and time: 09/15/2020 - 21:56:48 -> 09/15/2020 - 22:42:27

Dye: Blue - 1 peaks - 25.fsa

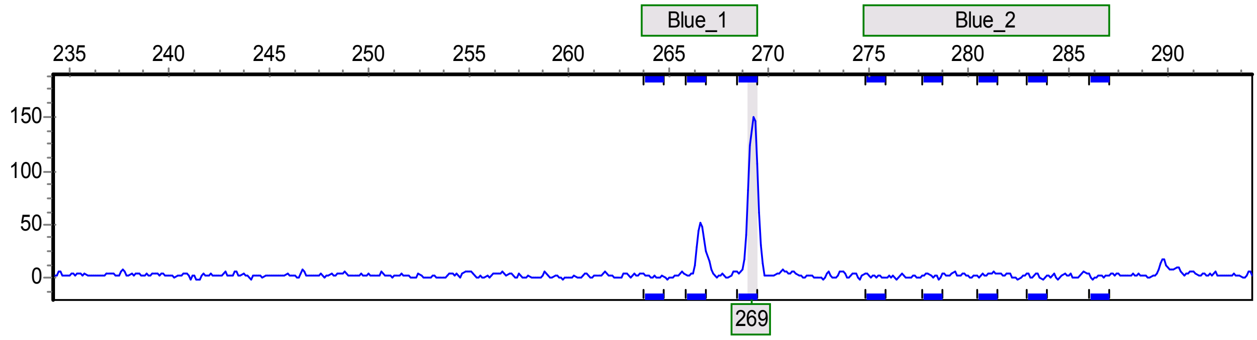

No	Size	Height	Area	Marker	Allele	Difference	Quality	Score	Allele Comments	Sample Comments
1	269.2	151	719	Blue_1	269	0.2	Pass	10.4		

Sample 19: Run date and time: 09/15/2020 - 21:56:48 -> 09/15/2020 - 22:42:27

Dye: Blue - 1 peaks - 26.fsa

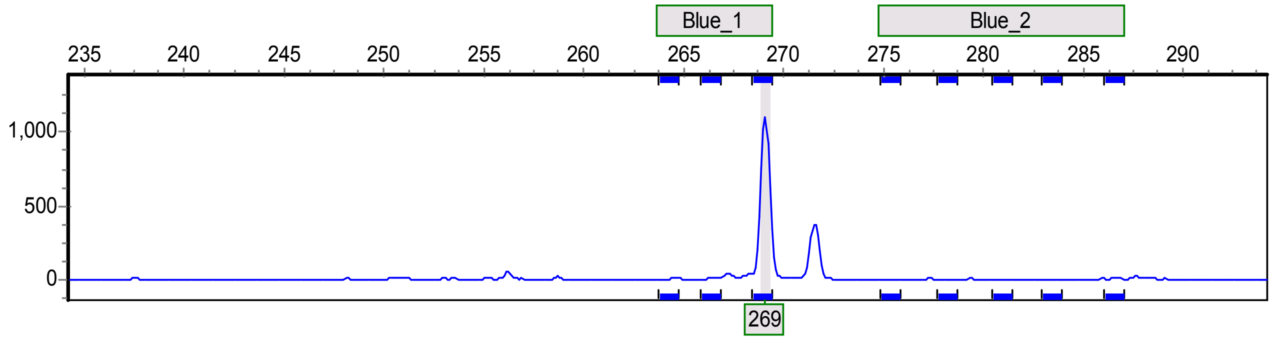

No	Size	Height	Area	Marker	Allele	Difference	Quality	Score	Allele Comments	Sample Comments
1	269.1	1094	5280	Blue_1	269	0.1	Pass	250.3		

Sample 20: Run date and time: 09/15/2020 - 21:56:48 -> 09/15/2020 - 22:42:27

Dye: Blue - 2 peaks - 27.fsa

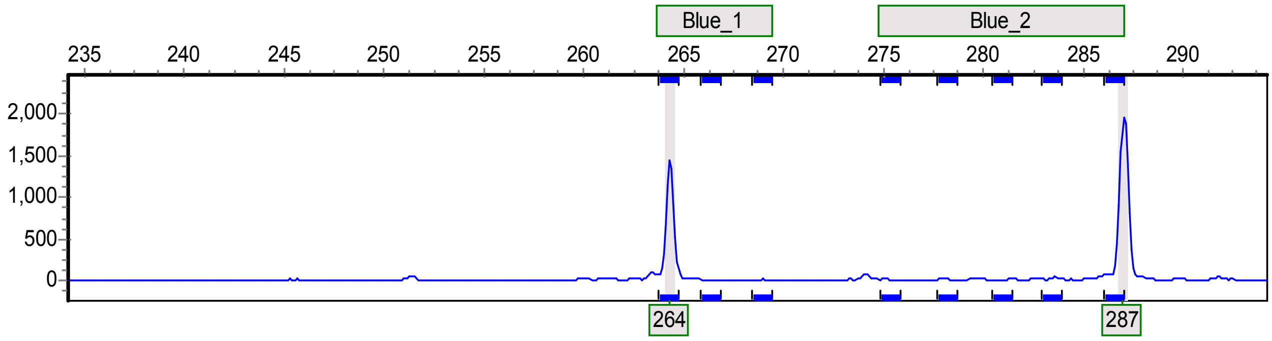

No	Size	Height	Area	Marker	Allele	Difference	Quality	Score	Allele Comments	Sample Comments
1	264.3	1441	6358	Blue_1	264	0.0	Pass	411.4		
2	287.0	1950	8933	Blue_2	287	0.4	Pass	500.0		

Sample 21: Run date and time: 09/15/2020 - 21:56:48 -> 09/15/2020 - 22:42:27

Dye: Blue - 2 peaks - 28.fsa

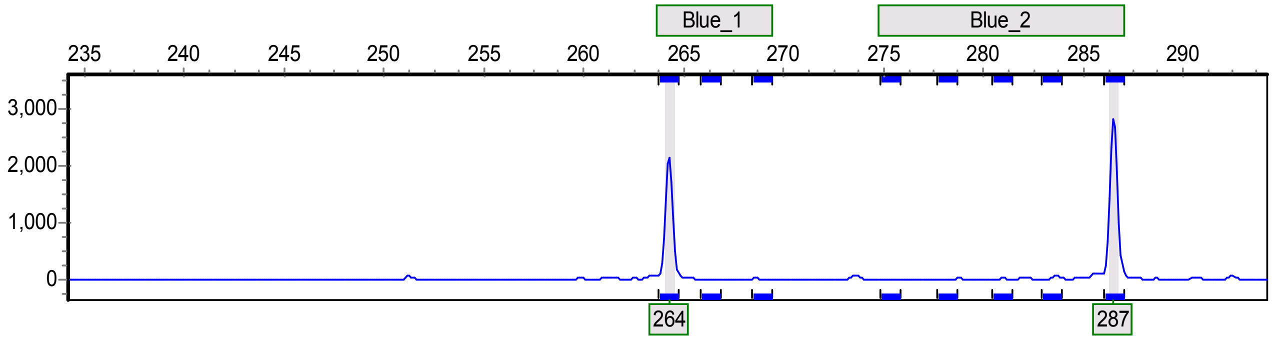

No	Size	Height	Area	Marker	Allele	Difference	Quality	Score	Allele Comments	Sample Comments
1	264.3	2144	9410	Blue_1	264	0.0	Pass	500.0		
2	286.5	2828	12593	Blue_2	287	0.1	Pass	500.0		

Sample 22: Run date and time: 09/15/2020 - 21:56:48 -> 09/15/2020 - 22:42:27

Dye: Blue - 0 peaks - 29.fsa

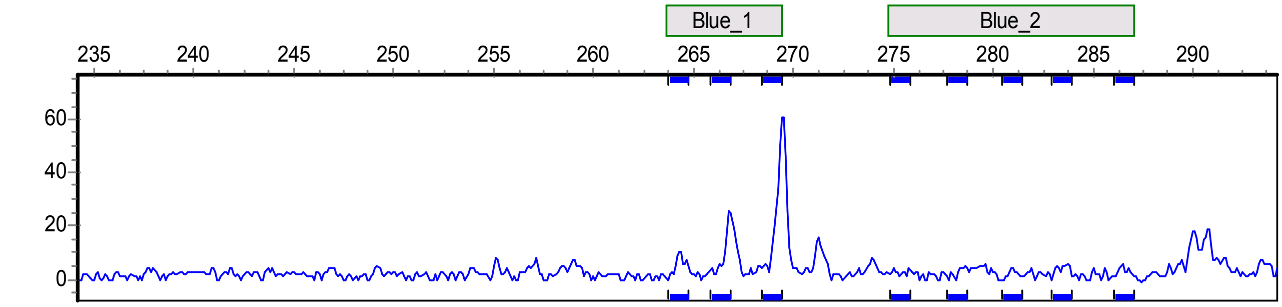

No	Size	Height	Area	Marker	Allele	Difference	Quality	Score	Allele Comments	Sample Comments

Sample 23: Run date and time: 09/15/2020 - 21:56:48 -> 09/15/2020 - 22:42:27

Dye: Blue - 1 peaks - 3.fsa

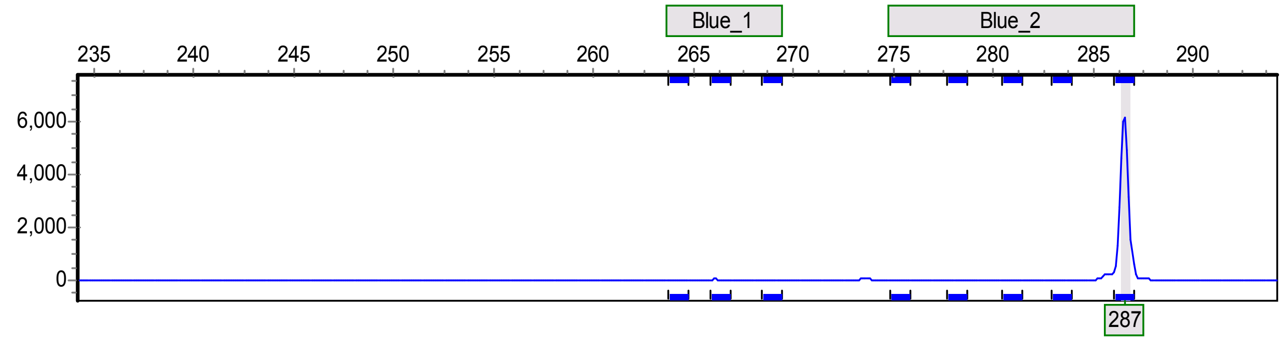

No	Size	Height	Area	Marker	Allele	Difference	Quality	Score	Allele Comments	Sample Comments
1	286.6	6132	29674	Blue_2	287	0.0	Pass	500.0		

Sample 24: Run date and time: 09/15/2020 - 21:56:48 -> 09/15/2020 - 22:42:27

Dye: Blue - 1 peaks - 30.fsa

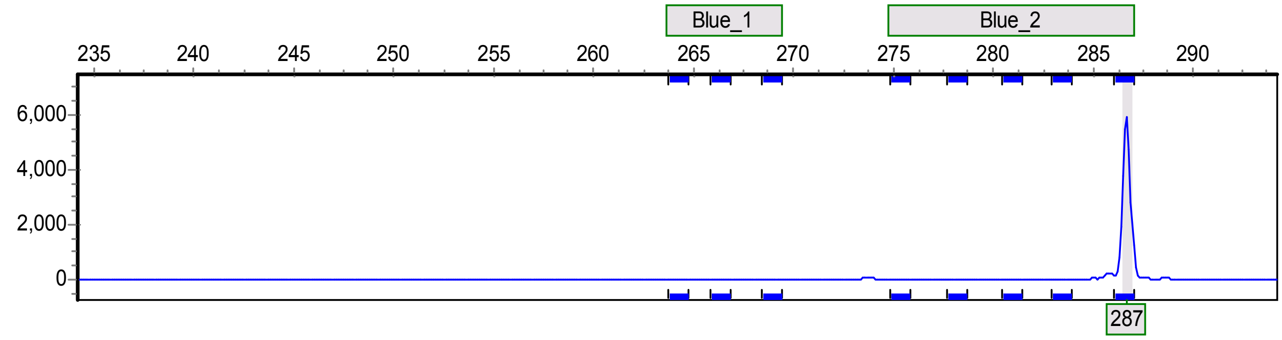

No	Size	Height	Area	Marker	Allele	Difference	Quality	Score	Allele Comments	Sample Comments
1	286.7	5905	25554	Blue_2	287	0.1	Pass	500.0		

Sample 25: Run date and time: 09/15/2020 - 21:56:48 -> 09/15/2020 - 22:42:27

Dye: Blue - 0 peaks - 31.fsa

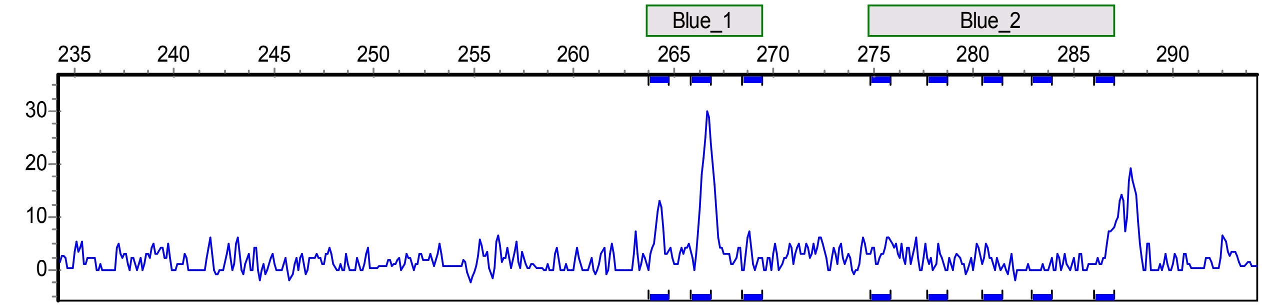

No	Size	Height	Area	Marker	Allele	Difference	Quality	Score	Allele Comments	Sample Comments

Sample 26: Run date and time: 09/15/2020 - 21:56:48 -> 09/15/2020 - 22:42:27

Dye: Blue - 0 peaks - 32.fsa

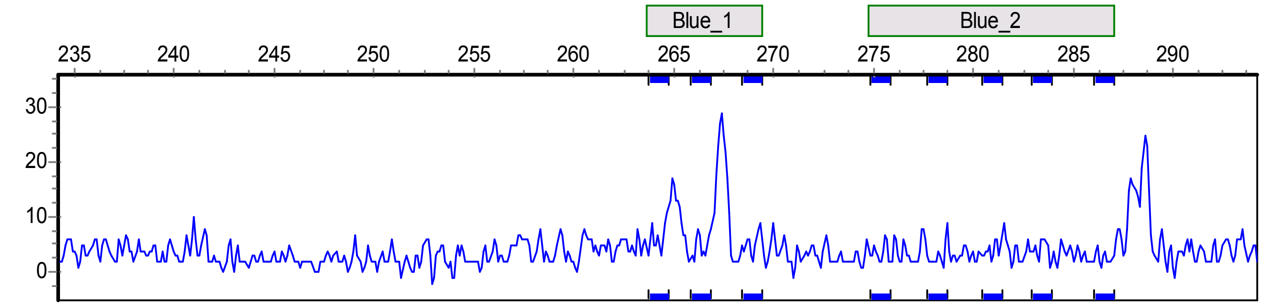

No	Size	Height	Area	Marker	Allele	Difference	Quality	Score	Allele Comments	Sample Comments

Sample 27: Run date and time: 09/15/2020 - 21:56:48 -> 09/15/2020 - 22:42:27

Dye: Blue - 1 peaks - 33.fsa

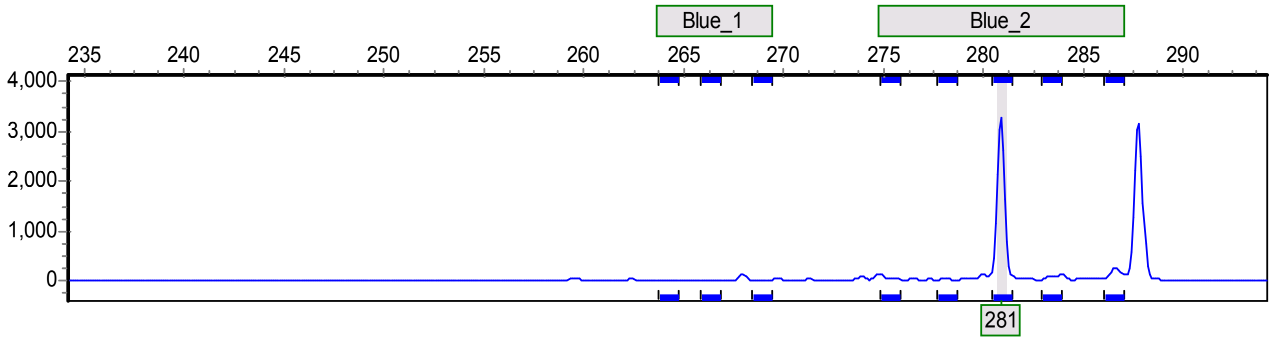

No	Size	Height	Area	Marker	Allele	Difference	Quality	Score	Allele Comments	Sample Comments
1	280.9	3251	14391	Blue_2	281	0.1	Pass	500.0		

Sample 28: Run date and time: 09/15/2020 - 21:56:48 -> 09/15/2020 - 22:42:27

Dye: Blue - 1 peaks - 34.fsa

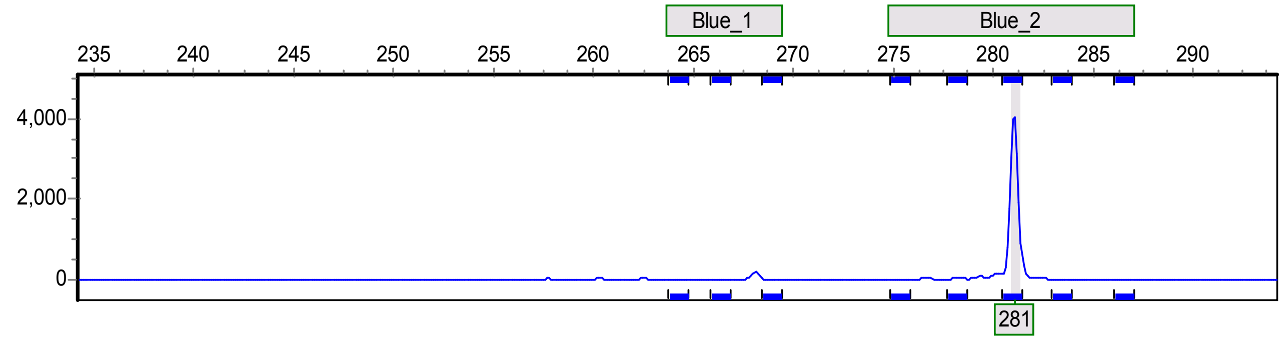

No	Size	Height	Area	Marker	Allele	Difference	Quality	Score	Allele Comments	Sample Comments
1	281.1	4023	18888	Blue_2	281	0.1	Pass	500.0		

Sample 29: Run date and time: 09/15/2020 - 21:56:48 -> 09/15/2020 - 22:42:27

Dye: Blue - 2 peaks - 35.fsa

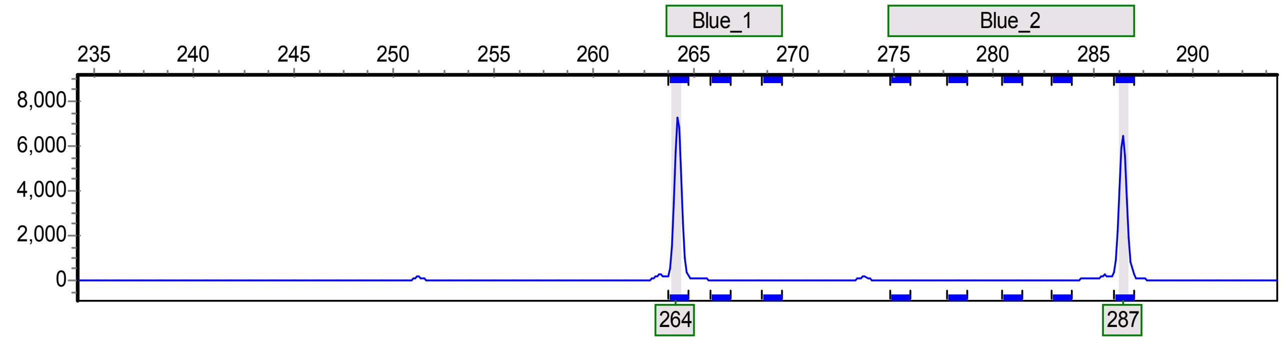

No	Size	Height	Area	Marker	Allele	Difference	Quality	Score	Allele Comments	Sample Comments
1	264.2	7251	31684	Blue_1	264	0.1	Pass	500.0		
2	286.5	6491	29840	Blue_2	287	0.1	Pass	500.0		

Sample 30: Run date and time: 09/15/2020 - 21:56:48 -> 09/15/2020 - 22:42:27

Dye: Blue - 2 peaks - 36.fsa

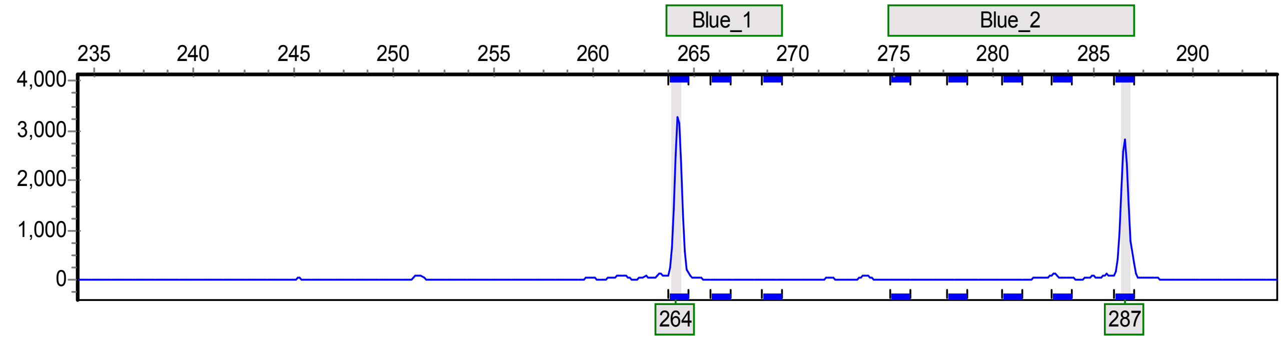

No	Size	Height	Area	Marker	Allele	Difference	Quality	Score	Allele Comments	Sample Comments
1	264.2	3250	14524	Blue_1	264	0.1	Pass	500.0		
2	286.6	2803	12758	Blue_2	287	0.0	Pass	500.0		

Allele Report

Sample 31: Run date and time: 09/15/2020 - 21:56:48 -> 09/15/2020 - 22:42:27

Dye: Blue - 1 peaks - 37.fsa

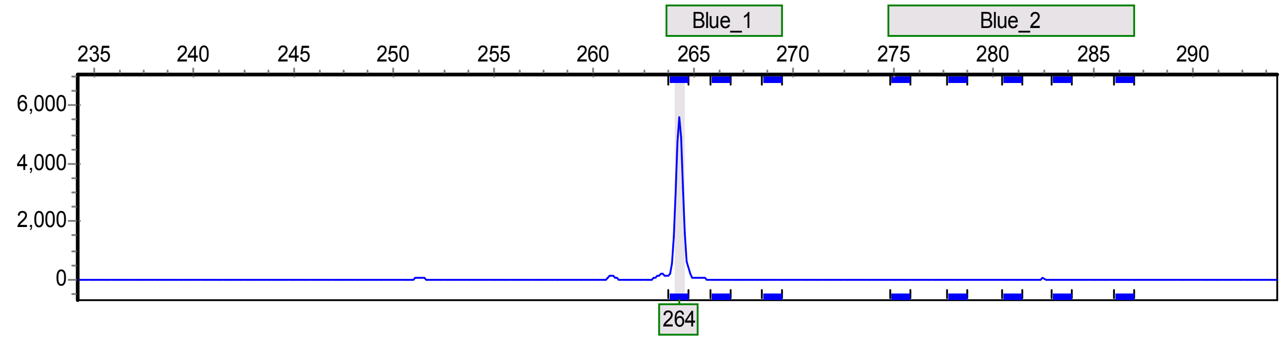

No	Size	Height	Area	Marker	Allele	Difference	Quality	Score	Allele Comments	Sample Comments
1	264.3	5546	24007	Blue_1	264	0.0	Pass	500.0		

Sample 32: Run date and time: 09/15/2020 - 21:56:48 -> 09/15/2020 - 22:42:27

Dye: Blue - 2 peaks - 38.fsa

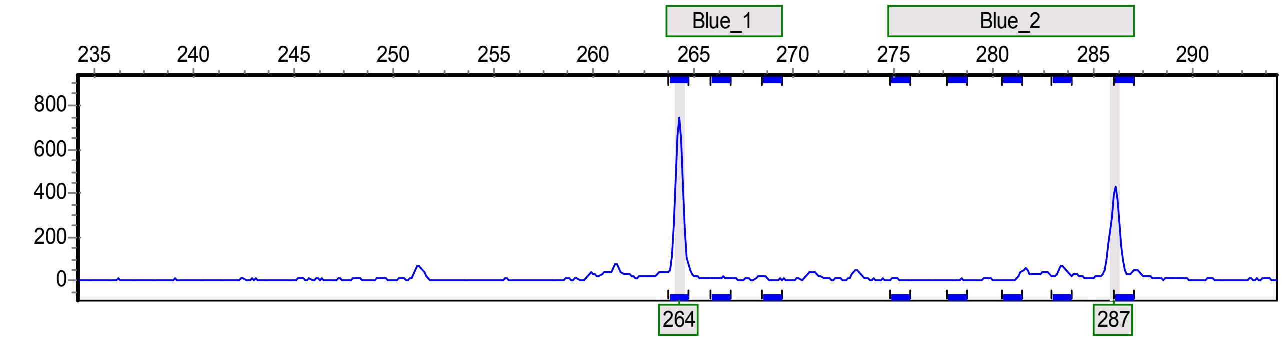

No	Size	Height	Area	Marker	Allele	Difference	Quality	Score	Allele Comments	Sample Comments
1	264.3	744	3462	Blue_1	264	0.0	Pass	148.6		
2	286.1	428	2187	Blue_2	287	0.5	Pass	55.1		

Sample 33: Run date and time: 09/15/2020 - 21:56:48 -> 09/15/2020 - 22:42:27

Dye: Blue - 2 peaks - 39.fsa

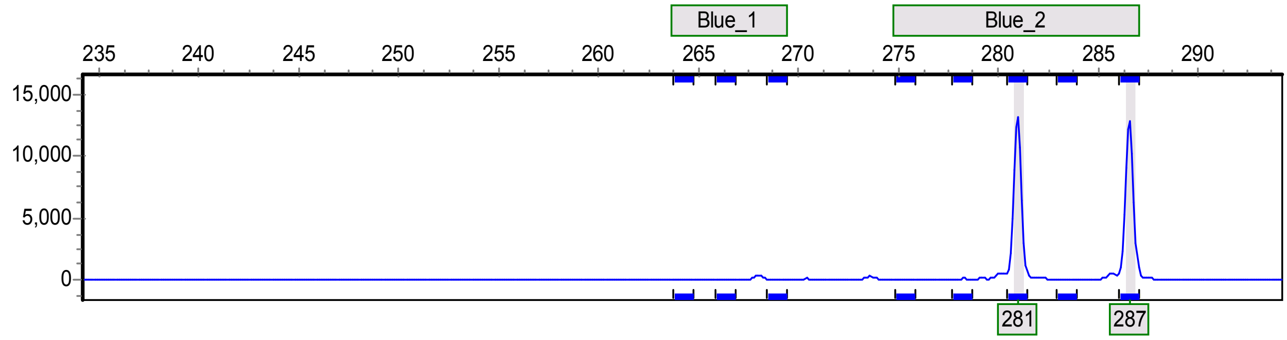

No	Size	Height	Area	Marker	Allele	Difference	Quality	Score	Allele Comments	Sample Comments
1	281.0	13132	59080	Blue_2	281	0.0	Pass	500.0		
2	286.6	12835	59125	Blue_2	287	0.0	Pass	500.0		

Sample 34: Run date and time: 09/15/2020 - 21:56:48 -> 09/15/2020 - 22:42:27

Dye: Blue - 0 peaks - 4.fsa

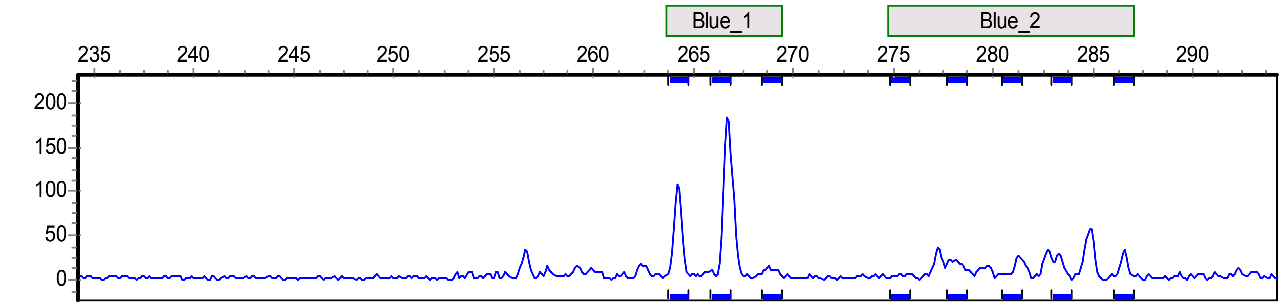

No	Size	Height	Area	Marker	Allele	Difference	Quality	Score	Allele Comments	Sample Comments

Sample 35: Run date and time: 09/15/2020 - 21:56:48 -> 09/15/2020 - 22:42:27

Dye: Blue - 2 peaks - 40.fsa

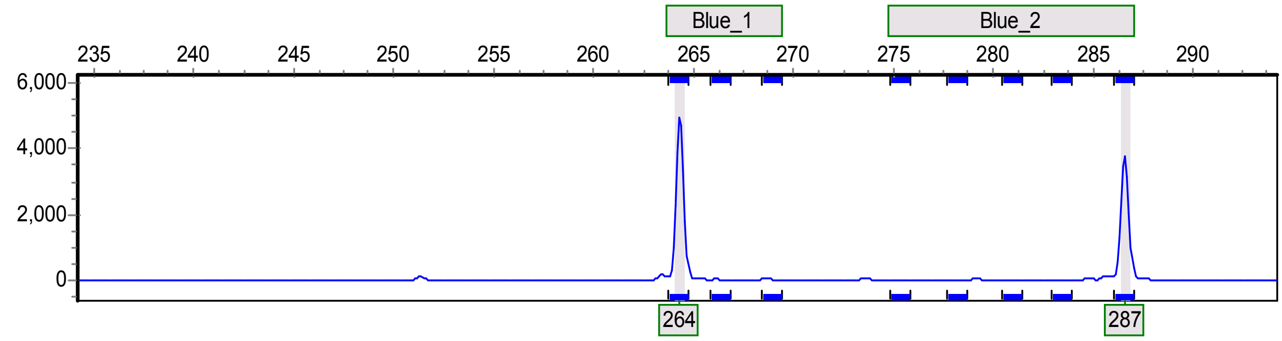

No	Size	Height	Area	Marker	Allele	Difference	Quality	Score	Allele Comments	Sample Comments
1	264.3	4917	21547	Blue_1	264	0.0	Pass	500.0		
2	286.6	3772	17096	Blue_2	287	0.0	Pass	500.0		

Sample 36: Run date and time: 09/15/2020 - 21:56:48 -> 09/15/2020 - 22:42:27

Dye: Blue - 1 peaks - 41.fsa

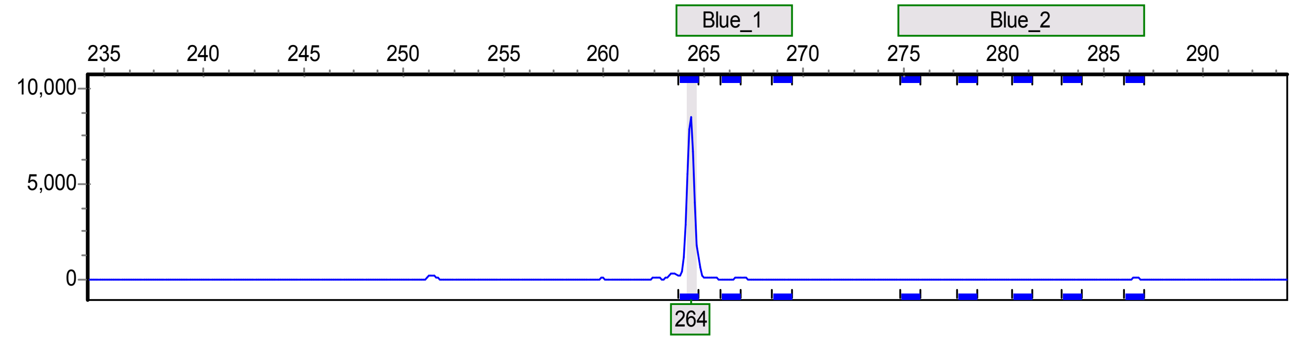

No	Size	Height	Area	Marker	Allele	Difference	Quality	Score	Allele Comments	Sample Comments
1	264.4	8459	36800	Blue_1	264	0.1	Pass	500.0		

Sample 37: Run date and time: 09/15/2020 - 21:56:48 -> 09/15/2020 - 22:42:27

Dye: Blue - 0 peaks - 42.fsa

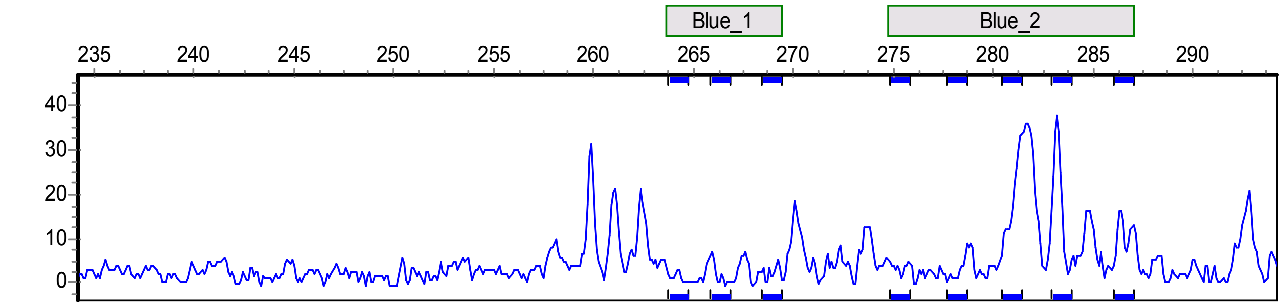

No	Size	Height	Area	Marker	Allele	Difference	Quality	Score	Allele Comments	Sample Comments

Sample 38: Run date and time: 09/15/2020 - 21:56:48 -> 09/15/2020 - 22:42:27

Dye: Blue - 2 peaks - 43.fsa

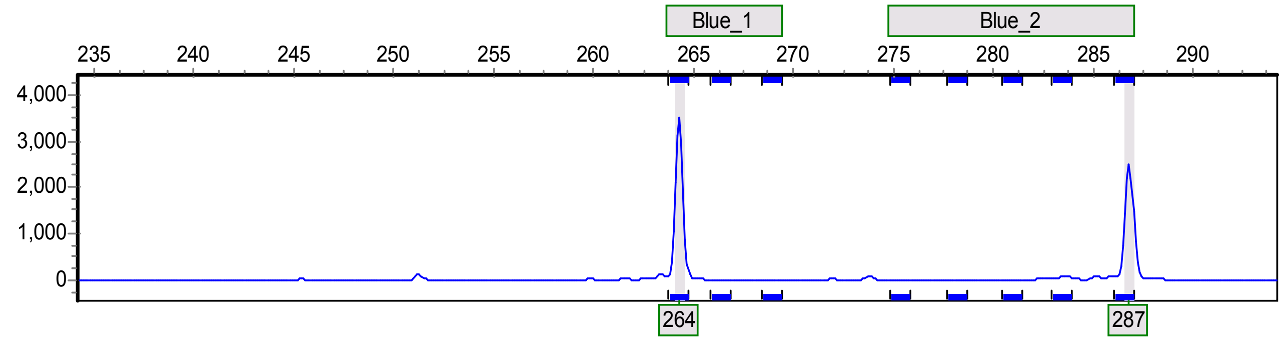

No	Size	Height	Area	Marker	Allele	Difference	Quality	Score	Allele Comments	Sample Comments
1	264.3	3484	15274	Blue_1	264	0.0	Pass	500.0		
2	286.8	2485	11383	Blue_2	287	0.2	Pass	500.0		

Sample 39: Run date and time: 09/15/2020 - 21:56:48 -> 09/15/2020 - 22:42:27

Dye: Blue - 2 peaks - 44.fsa

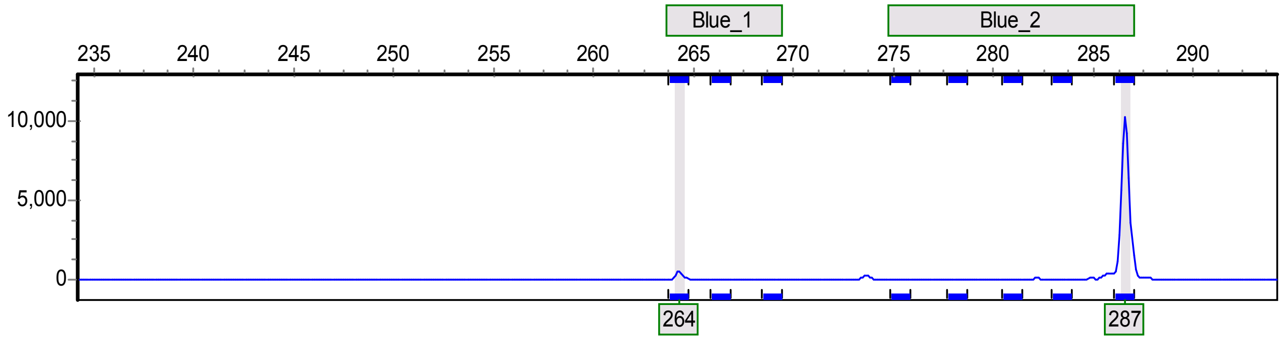

No	Size	Height	Area	Marker	Allele	Difference	Quality	Score	Allele Comments	Sample Comments
1	264.3	529	2360	Blue_1	264	0.0	Pass	93.4		
2	286.6	10167	46522	Blue_2	287	0.0	Pass	500.0		

Sample 40: Run date and time: 09/15/2020 - 21:56:48 -> 09/15/2020 - 22:42:27

Dye: Blue - 2 peaks - 45.fsa

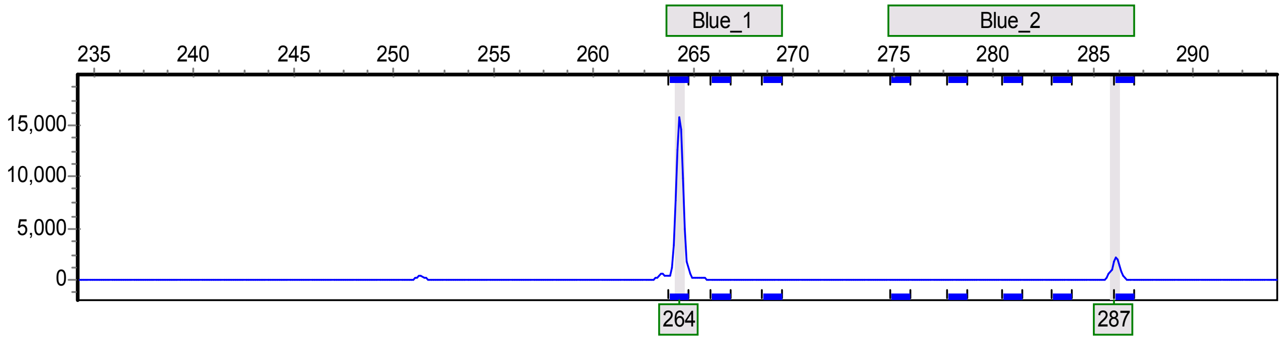

No	Size	Height	Area	Marker	Allele	Difference	Quality	Score	Allele Comments	Sample Comments
1	264.3	15601	66831	Blue_1	264	0.0	Pass	500.0		
2	286.1	2093	9536	Blue_2	287	0.5	Pass	500.0		

Sample 41: Run date and time: 09/15/2020 - 21:56:48 -> 09/15/2020 - 22:42:27

Dye: Blue - 1 peaks - 46.fsa

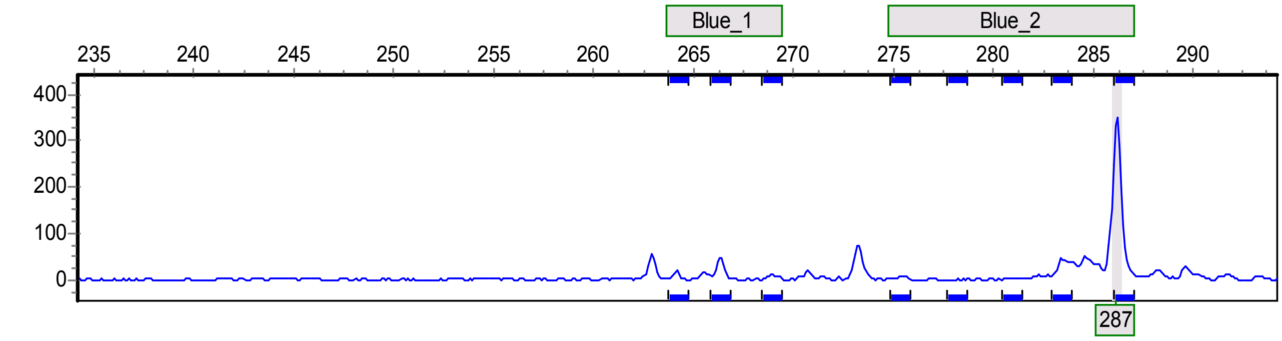

No	Size	Height	Area	Marker	Allele	Difference	Quality	Score	Allele Comments	Sample Comments
1	286.2	349	1763	Blue_2	287	0.4	Pass	40.1		

Sample 42: Run date and time: 09/15/2020 - 21:56:48 -> 09/15/2020 - 22:42:27

Dye: Blue - 1 peaks - 47.fsa

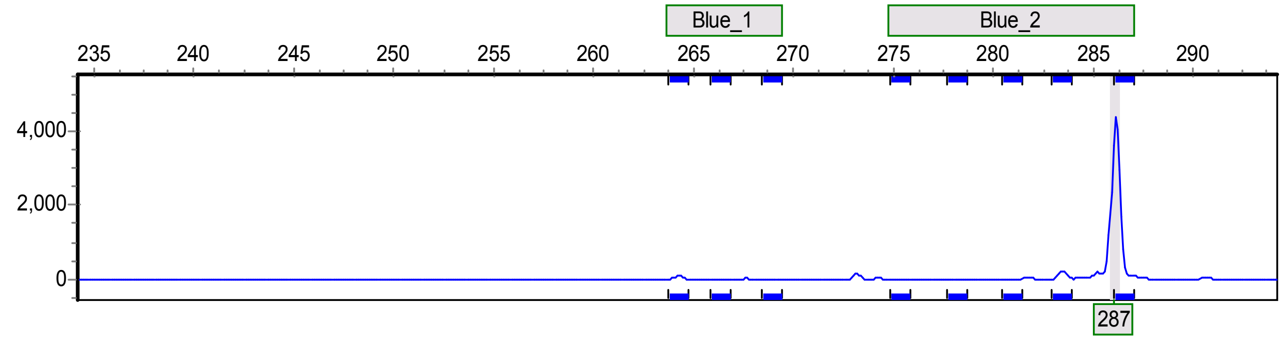

No	Size	Height	Area	Marker	Allele	Difference	Quality	Score	Allele Comments	Sample Comments
1	286.1	4340	20365	Blue_2	287	0.5	Pass	500.0		

Sample 43: Run date and time: 09/15/2020 - 21:56:48 -> 09/15/2020 - 22:42:27

Dye: Blue - 1 peaks - 48.fsa

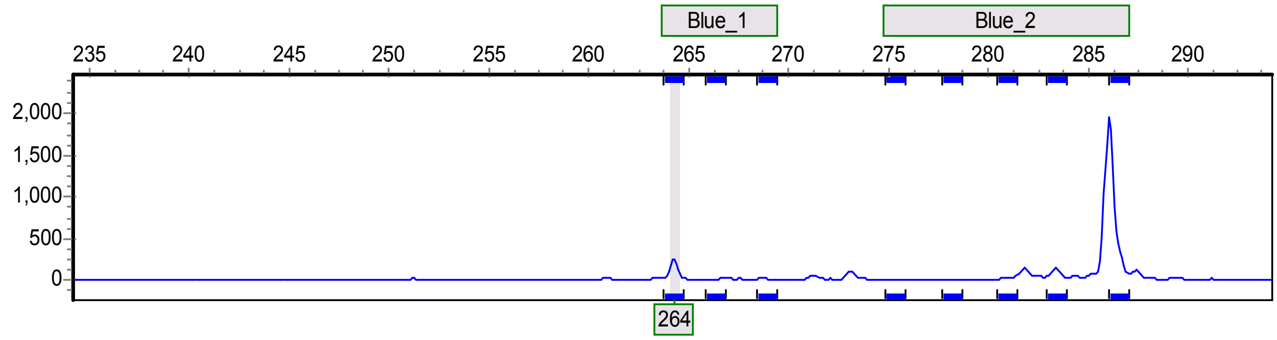

No	Size	Height	Area	Marker	Allele	Difference	Quality	Score	Allele Comments	Sample Comments
1	264.3	252	1184	Blue_1	264	0.0	Pass	26.3		

Sample 44: Run date and time: 09/15/2020 - 21:56:48 -> 09/15/2020 - 22:42:27

Dye: Blue - 1 peaks - 49.fsa

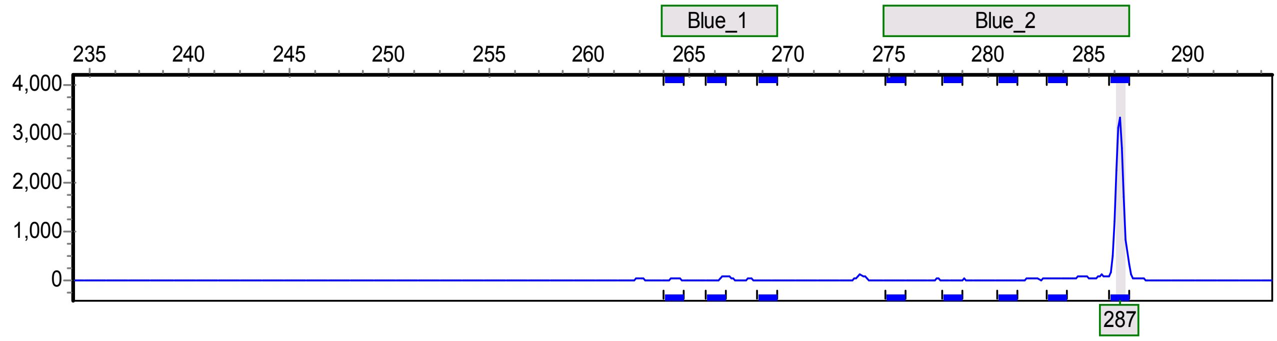

No	Size	Height	Area	Marker	Allele	Difference	Quality	Score	Allele Comments	Sample Comments
1	286.6	3324	15153	Blue_2	287	0.0	Pass	500.0		

Sample 45: Run date and time: 09/15/2020 - 21:56:48 -> 09/15/2020 - 22:42:27

Dye: Blue - 2 peaks - 5.fsa

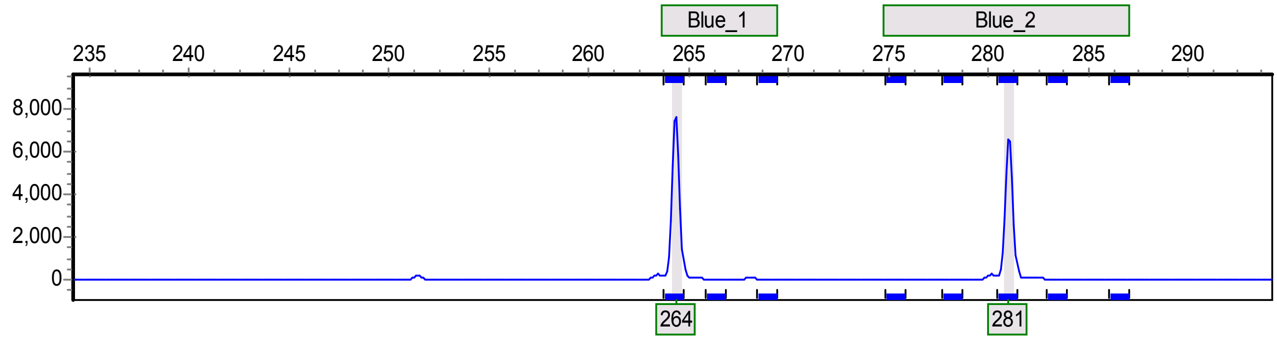

No	Size	Height	Area	Marker	Allele	Difference	Quality	Score	Allele Comments	Sample Comments
1	264.4	7612	33198	Blue_1	264	0.1	Pass	500.0		
2	281.0	6625	29289	Blue_2	281	0.0	Pass	500.0		

Sample 46: Run date and time: 09/15/2020 - 21:56:48 -> 09/15/2020 - 22:42:27

Dye: Blue - 0 peaks - 50.fsa

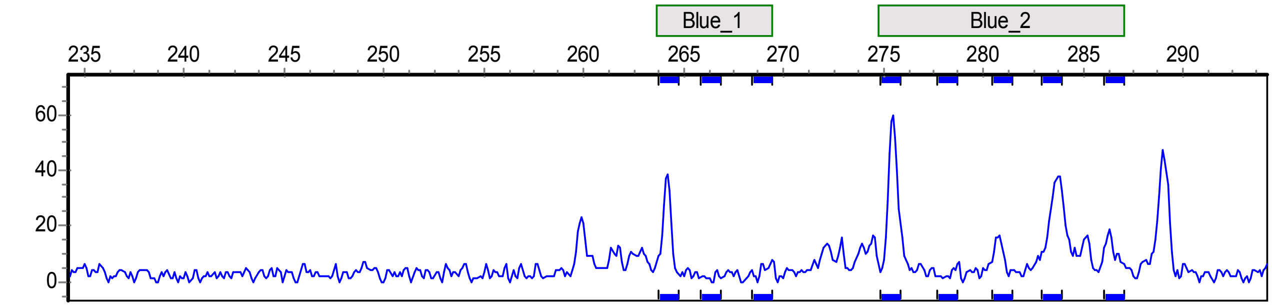

No	Size	Height	Area	Marker	Allele	Difference	Quality	Score	Allele Comments	Sample Comments

Sample 47: Run date and time: 09/15/2020 - 21:56:48 -> 09/15/2020 - 22:42:27

Dye: Blue - 2 peaks - 51.fsa

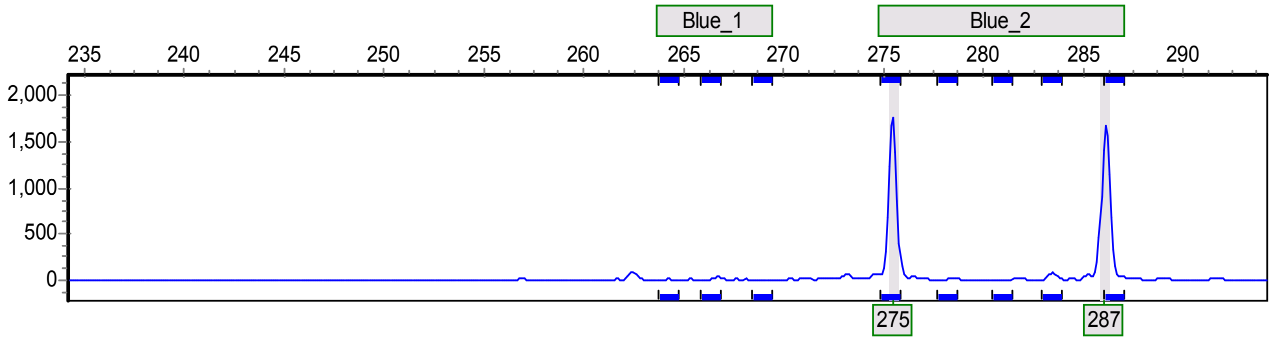

No	Size	Height	Area	Marker	Allele	Difference	Quality	Score	Allele Comments	Sample Comments
1	275.5	1756	8012	Blue_2	275	0.1	Pass	500.0		
2	286.1	1675	7949	Blue_2	287	0.5	Pass	465.8		

Sample 48: Run date and time: 09/15/2020 - 21:56:48 -> 09/15/2020 - 22:42:27

Dye: Blue - 2 peaks - 52.fsa

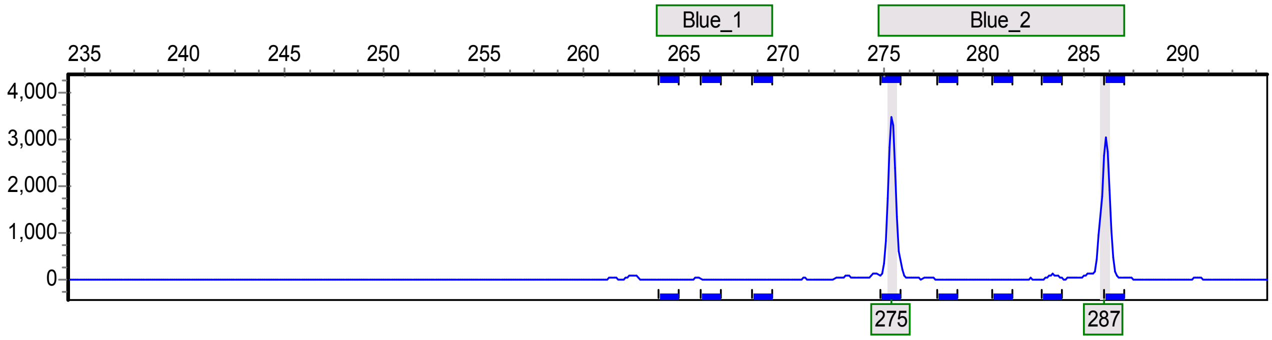

No	Size	Height	Area	Marker	Allele	Difference	Quality	Score	Allele Comments	Sample Comments
1	275.4	3469	15950	Blue_2	275	0.0	Pass	500.0		
2	286.1	3067	14361	Blue_2	287	0.5	Pass	500.0		

Allele Report

Sample 49: Run date and time: 09/15/2020 - 21:56:48 -> 09/15/2020 - 22:42:27

Dye: Blue - 0 peaks - 53.fsa

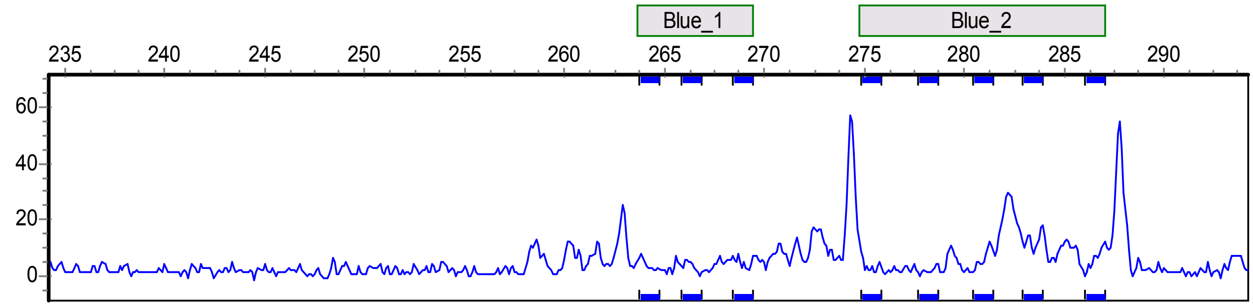

No	Size	Height	Area	Marker	Allele	Difference	Quality	Score	Allele Comments	Sample Comments

Sample 50: Run date and time: 09/15/2020 - 21:56:48 -> 09/15/2020 - 22:42:27

Dye: Blue - 1 peaks - 54.fsa

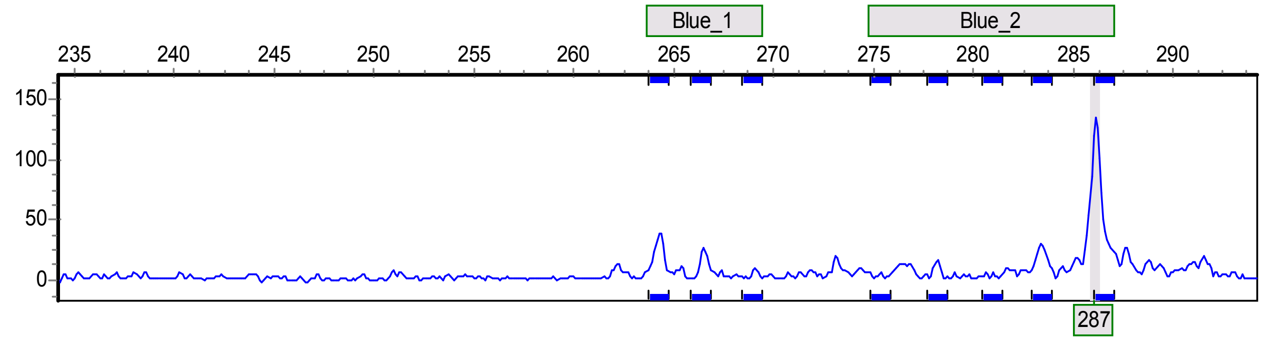

No	Size	Height	Area	Marker	Allele	Difference	Quality	Score	Allele Comments	Sample Comments
1	286.1	134	735	Blue_2	287	0.5	Pass	5.7	[<Confirmed>]	

Sample 51: Run date and time: 09/15/2020 - 21:56:48 -> 09/15/2020 - 22:42:27

Dye: Blue - 1 peaks - 55.fsa

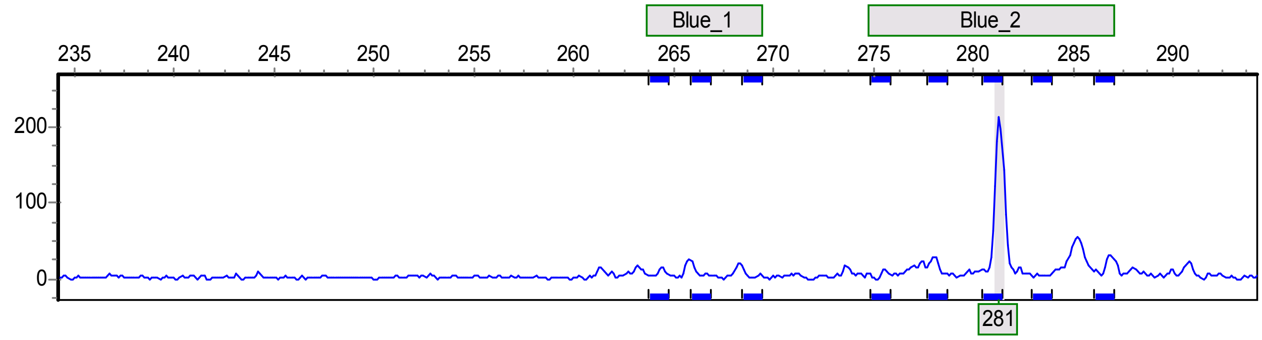

No	Size	Height	Area	Marker	Allele	Difference	Quality	Score	Allele Comments	Sample Comments
1	281.3	212	1026	Blue_2	281	0.3	Pass	18.7		

Sample 52: Run date and time: 09/15/2020 - 21:56:48 -> 09/15/2020 - 22:42:27

Dye: Blue - 0 peaks - 56.fsa

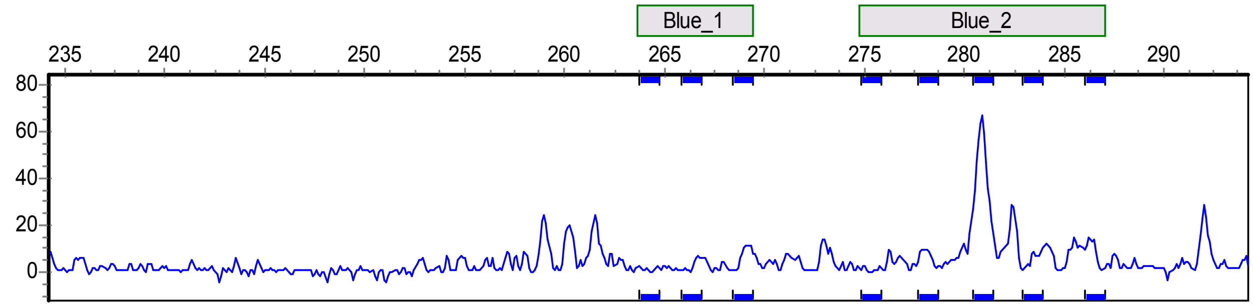

No	Size	Height	Area	Marker	Allele	Difference	Quality	Score	Allele Comments	Sample Comments

Sample 53: Run date and time: 09/15/2020 - 21:56:48 -> 09/15/2020 - 22:42:27

Dye: Blue - 0 peaks - 57.fsa

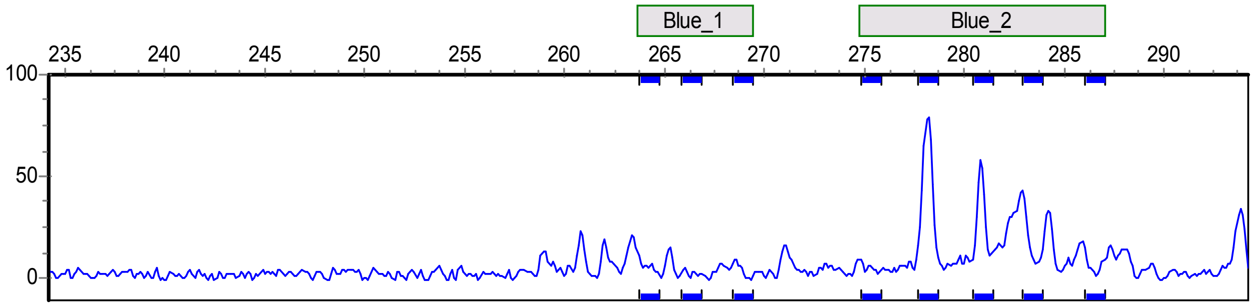

No	Size	Height	Area	Marker	Allele	Difference	Quality	Score	Allele Comments	Sample Comments

Sample 54: Run date and time: 09/15/2020 - 21:56:48 -> 09/15/2020 - 22:42:27

Dye: Blue - 1 peaks - 58.fsa

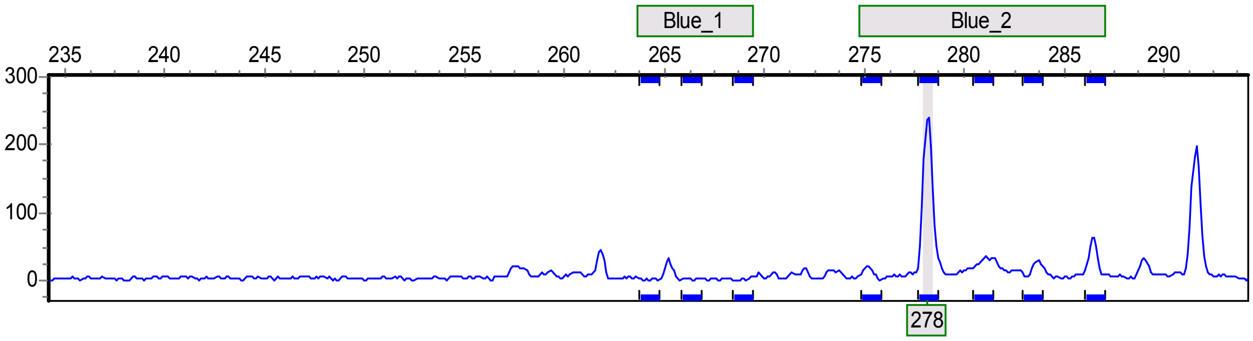

No	Size	Height	Area	Marker	Allele	Difference	Quality	Score	Allele Comments	Sample Comments
1	278.2	240	1191	Blue_2	278	0.0	Pass	20.9		

Allele Report

Sample 55: Run date and time: 09/15/2020 - 21:56:48 -> 09/15/2020 - 22:42:27

Dye: Blue - 0 peaks - 59.fsa

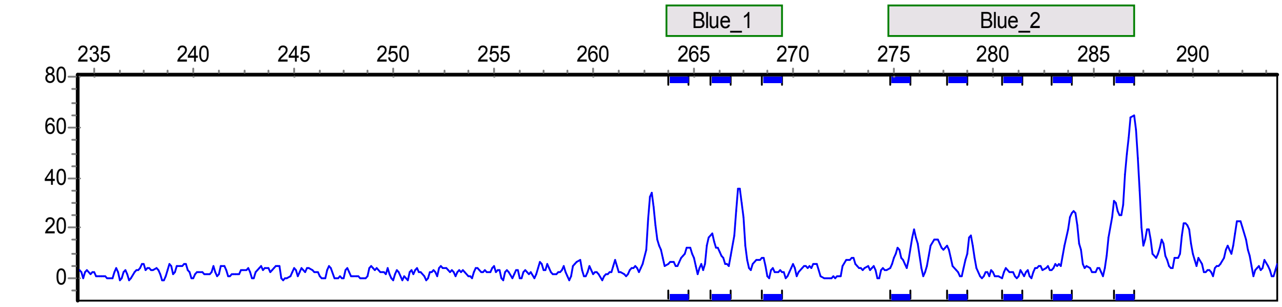

No	Size	Height	Area	Marker	Allele	Difference	Quality	Score	Allele Comments	Sample Comments

Sample 56: Run date and time: 09/15/2020 - 21:56:48 -> 09/15/2020 - 22:42:27

Dye: Blue - 2 peaks - 6.fsa

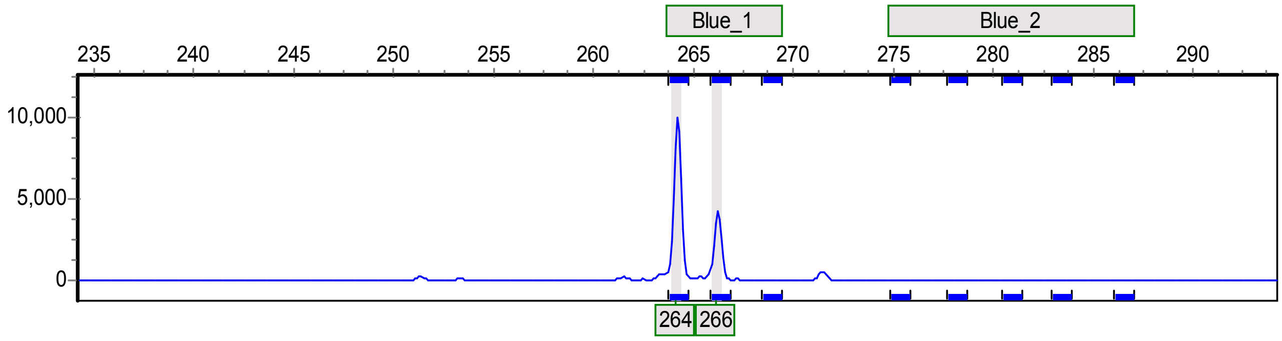

No	Size	Height	Area	Marker	Allele	Difference	Quality	Score	Allele Comments	Sample Comments
1	264.2	9915	43006	Blue_1	264	0.1	Pass	500.0		
2	266.2	4182	18398	Blue_1	266	0.2	Pass	500.0		

Sample 57: Run date and time: 09/15/2020 - 21:56:48 -> 09/15/2020 - 22:42:27

Dye: Blue - 0 peaks - 60.fsa

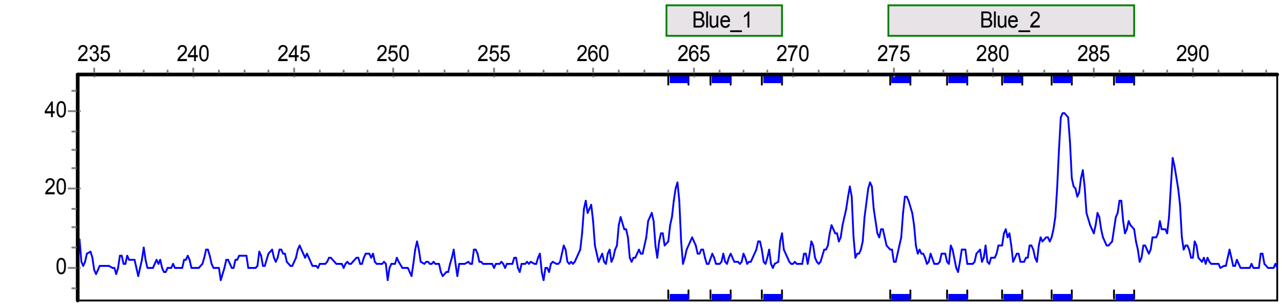

No	Size	Height	Area	Marker	Allele	Difference	Quality	Score	Allele Comments	Sample Comments

Sample 58: Run date and time: 09/15/2020 - 21:56:48 -> 09/15/2020 - 22:42:27

Dye: Blue - 1 peaks - 61.fsa

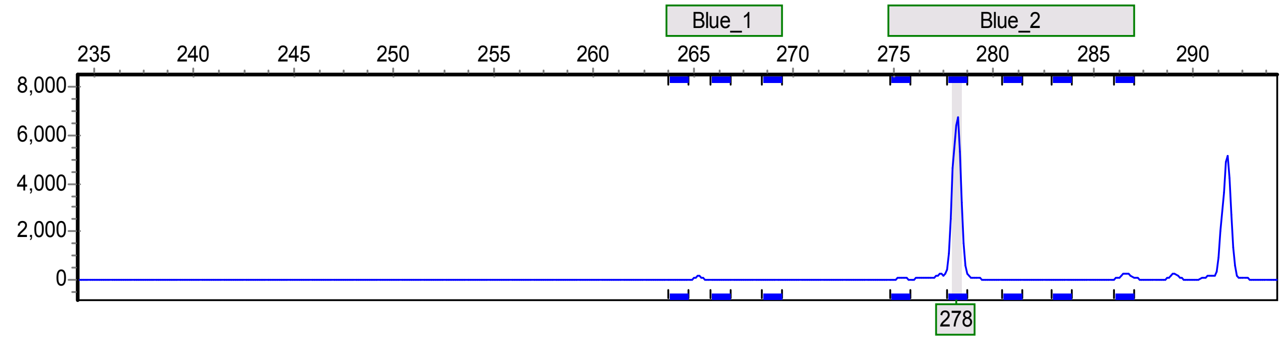

No	Size	Height	Area	Marker	Allele	Difference	Quality	Score	Allele Comments	Sample Comments
1	278.2	6716	30478	Blue_2	278	0.0	Pass	500.0		

Sample 59: Run date and time: 09/15/2020 - 21:56:48 -> 09/15/2020 - 22:42:27

Dye: Blue - 1 peaks - 62.fsa

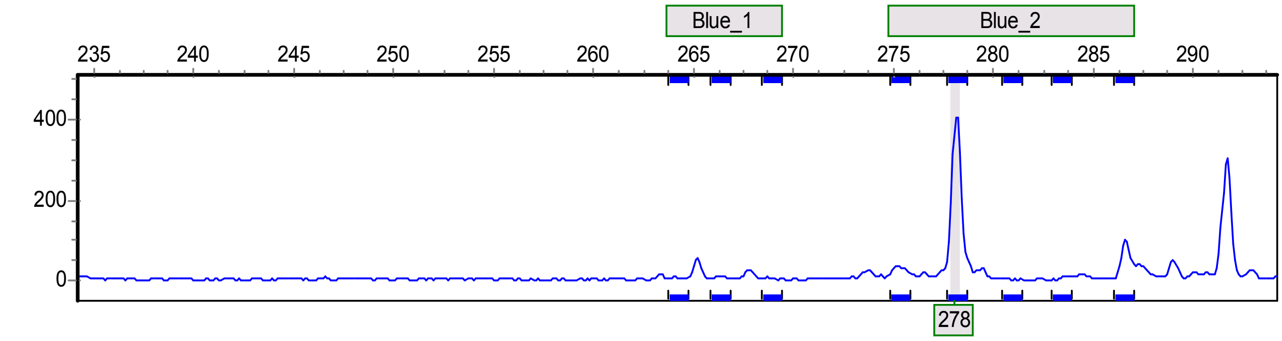

No	Size	Height	Area	Marker	Allele	Difference	Quality	Score	Allele Comments	Sample Comments
1	278.1	404	2052	Blue_2	278	0.1	Pass	51.3		

Sample 60: Run date and time: 09/15/2020 - 21:56:48 -> 09/15/2020 - 22:42:27

Dye: Blue - 1 peaks - 63.fsa

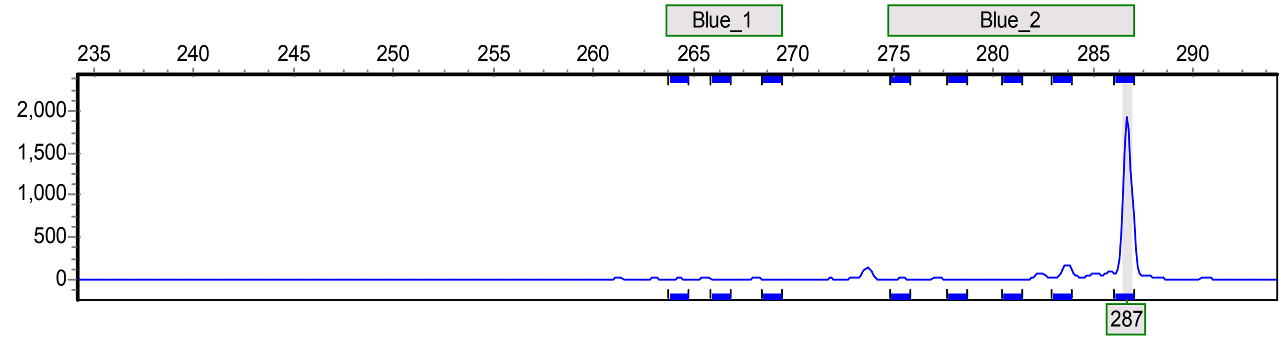

No	Size	Height	Area	Marker	Allele	Difference	Quality	Score	Allele Comments	Sample Comments
1	286.7	1909	9044	Blue_2	287	0.1	Pass	500.0		

Allele Report

Sample 61: Run date and time: 09/15/2020 - 21:56:48 -> 09/15/2020 - 22:42:27

Dye: Blue - 1 peaks - 64.fsa

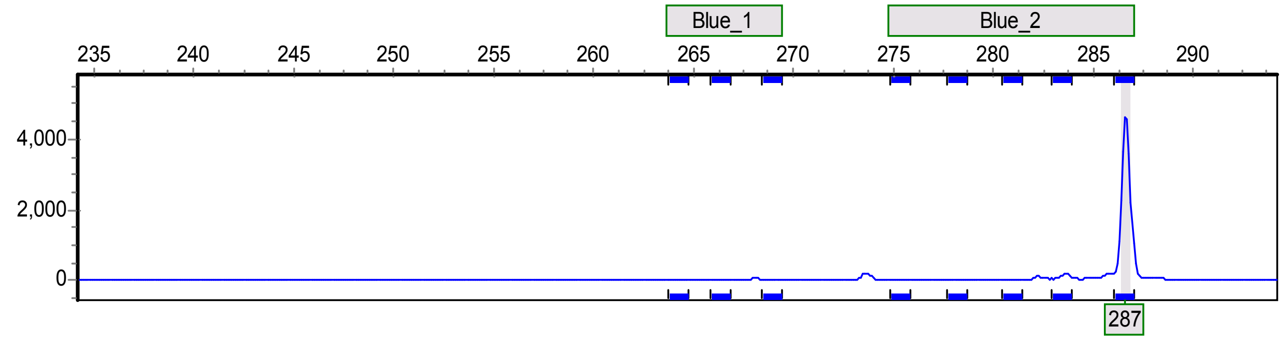

No	Size	Height	Area	Marker	Allele	Difference	Quality	Score	Allele Comments	Sample Comments
1	286.6	4617	22517	Blue_2	287	0.0	Pass	500.0		

Sample 62: Run date and time: 09/15/2020 - 21:56:48 -> 09/15/2020 - 22:42:27

Dye: Blue - 1 peaks - 65.fsa

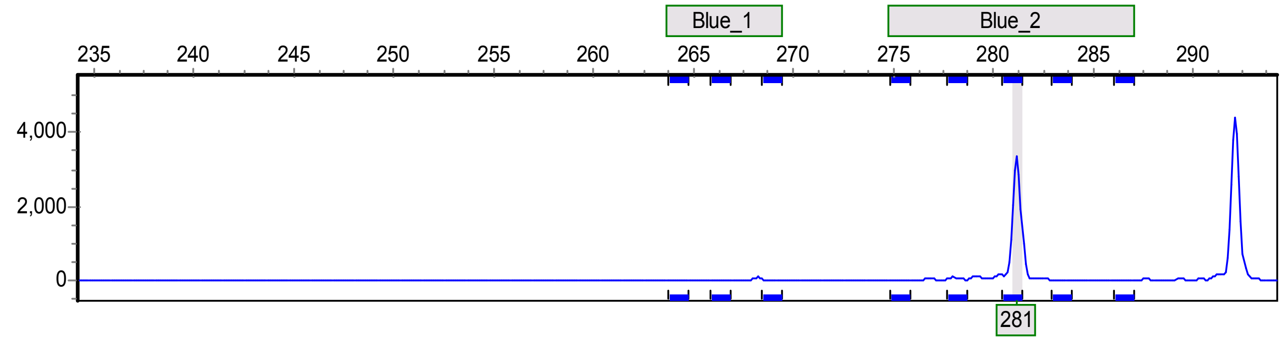

No	Size	Height	Area	Marker	Allele	Difference	Quality	Score	Allele Comments	Sample Comments
1	281.2	3349	15313	Blue_2	281	0.2	Pass	500.0		

Sample 63: Run date and time: 09/15/2020 - 21:56:48 -> 09/15/2020 - 22:42:27

Dye: Blue - 1 peaks - 66.fsa

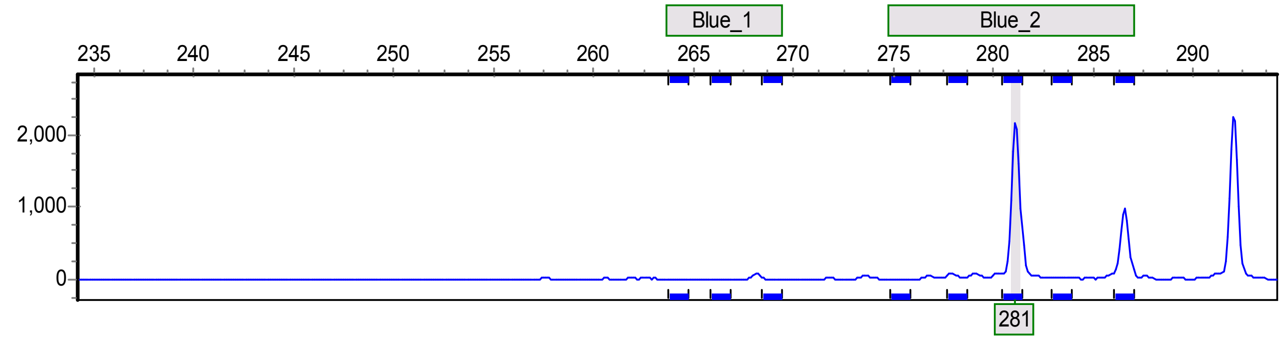

No	Size	Height	Area	Marker	Allele	Difference	Quality	Score	Allele Comments	Sample Comments
1	281.1	2160	10378	Blue_2	281	0.1	Pass	500.0		

Allele Report

Sample 64: Run date and time: 09/15/2020 - 21:56:48 -> 09/15/2020 - 22:42:27

Dye: Blue - 2 peaks - 67.fsa

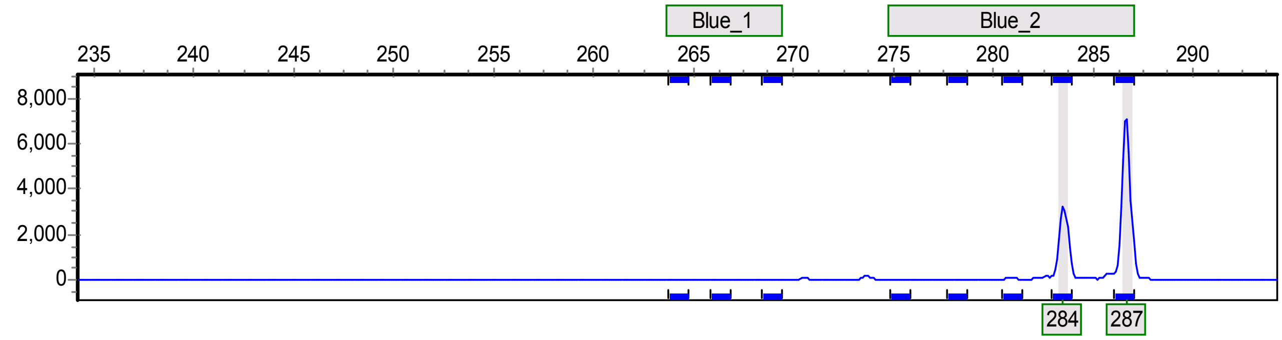

No	Size	Height	Area	Marker	Allele	Difference	Quality	Score	Allele Comments	Sample Comments
1	283.5	3265	15830	Blue_2	284	0.0	Pass	500.0		
2	286.7	7138	34159	Blue_2	287	0.1	Pass	500.0		

Sample 65: Run date and time: 09/15/2020 - 21:56:48 -> 09/15/2020 - 22:42:27

Dye: Blue - 2 peaks - 68.fsa

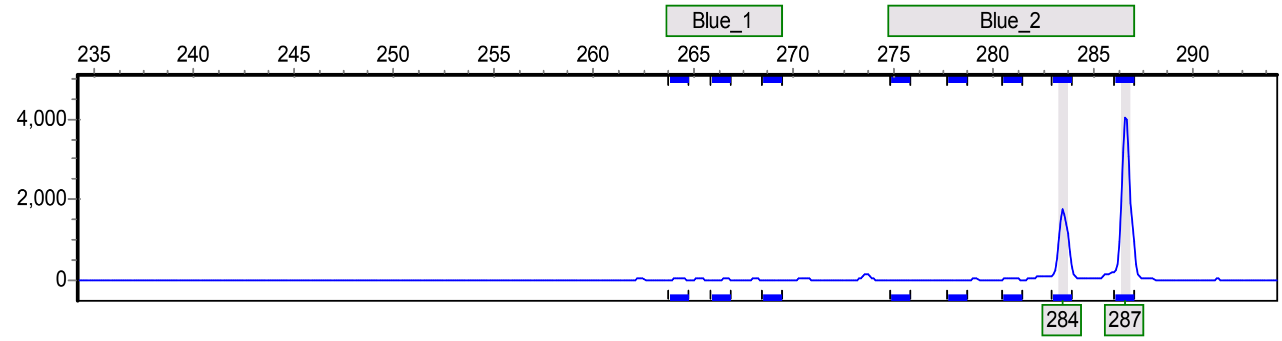

No	Size	Height	Area	Marker	Allele	Difference	Quality	Score	Allele Comments	Sample Comments
1	283.5	1761	8599	Blue_2	284	0.0	Pass	480.8		
2	286.6	4005	19601	Blue_2	287	0.0	Pass	500.0		

Sample 66: Run date and time: 09/15/2020 - 21:56:48 -> 09/15/2020 - 22:42:27

Dye: Blue - 1 peaks - 69.fsa

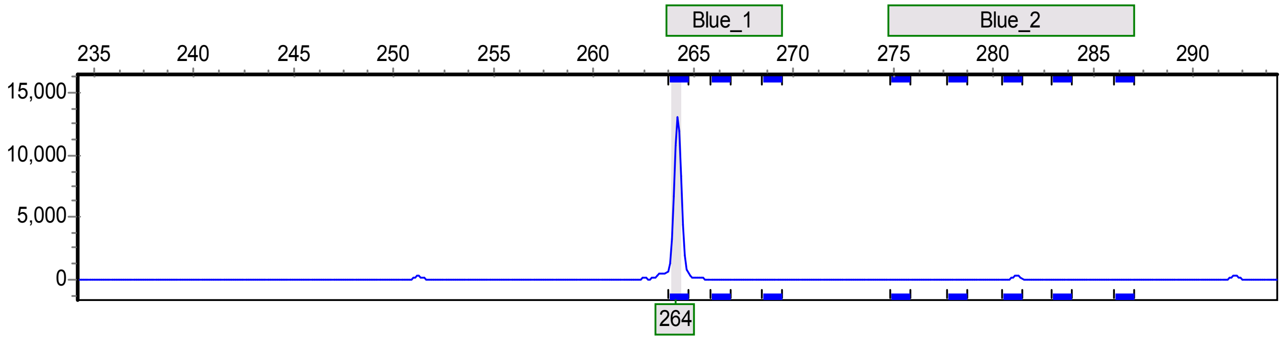

No	Size	Height	Area	Marker	Allele	Difference	Quality	Score	Allele Comments	Sample Comments
1	264.2	12973	58035	Blue_1	264	0.1	Pass	500.0		

Allele Report

Sample 67: Run date and time: 09/15/2020 - 21:56:48 -> 09/15/2020 - 22:42:27

Dye: Blue - 1 peaks - 7.fsa

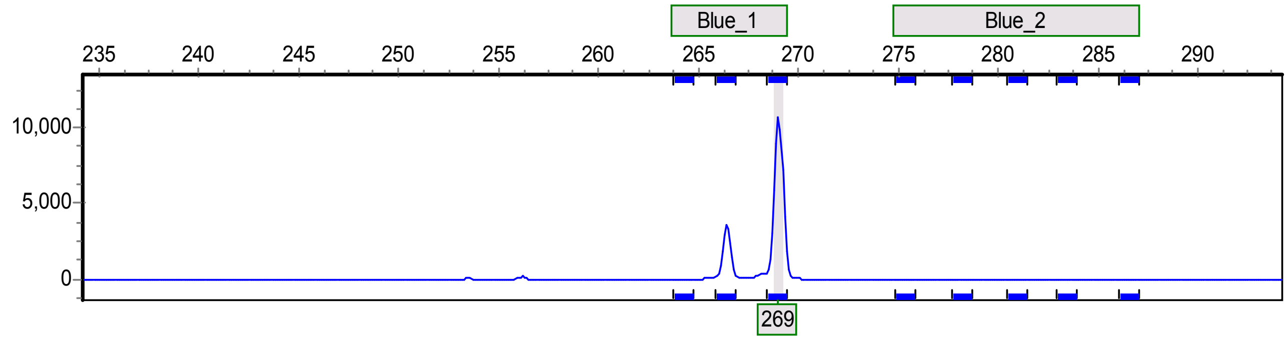

No	Size	Height	Area	Marker	Allele	Difference	Quality	Score	Allele Comments	Sample Comments
1	269.0	10579	50133	Blue_1	269	0.0	Pass	500.0		

Sample 68: Run date and time: 09/15/2020 - 21:56:48 -> 09/15/2020 - 22:42:27

Dye: Blue - 1 peaks - 70.fsa

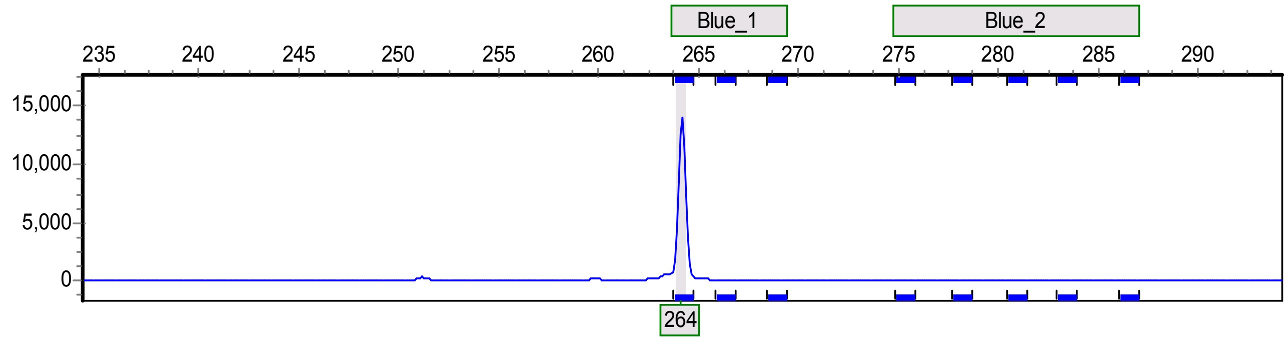

No	Size	Height	Area	Marker	Allele	Difference	Quality	Score	Allele Comments	Sample Comments
1	264.2	13943	61568	Blue_1	264	0.1	Pass	500.0		

Sample 69: Run date and time: 09/15/2020 - 21:56:48 -> 09/15/2020 - 22:42:27

Dye: Blue - 2 peaks - 71.fsa

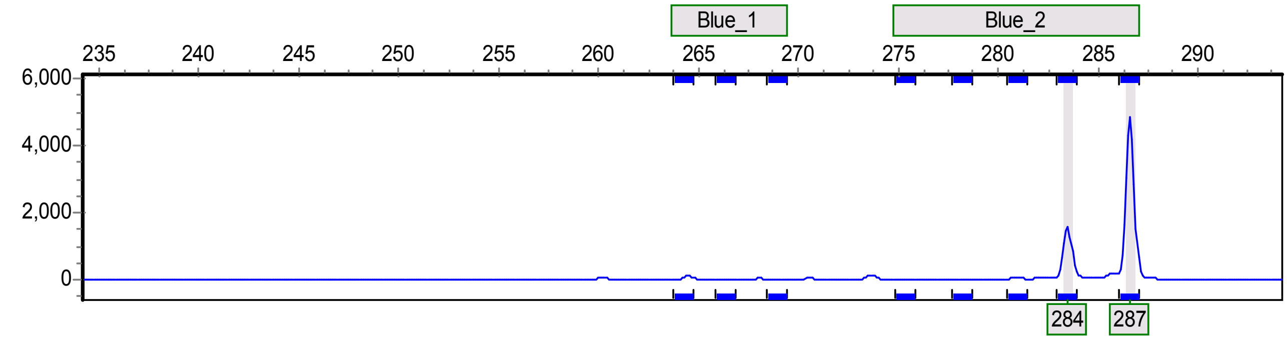

No	Size	Height	Area	Marker	Allele	Difference	Quality	Score	Allele Comments	Sample Comments
1	283.5	1581	7509	Blue_2	284	0.0	Pass	426.4		
2	286.6	4837	22600	Blue_2	287	0.0	Pass	500.0		

Allele Report

Sample 70: Run date and time: 09/15/2020 - 21:56:48 -> 09/15/2020 - 22:42:27

Dye: Blue - 2 peaks - 72.fsa

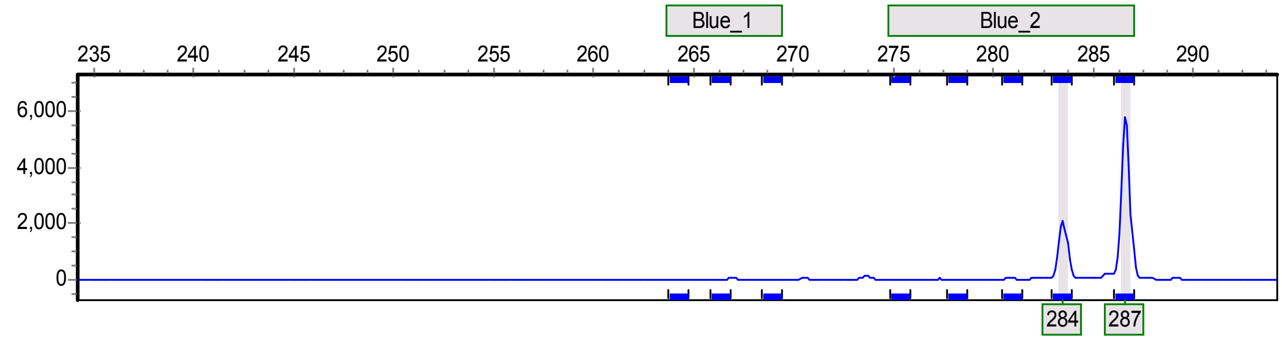

No	Size	Height	Area	Marker	Allele	Difference	Quality	Score	Allele Comments	Sample Comments
1	283.5	2100	10141	Blue_2	284	0.0	Pass	500.0		
2	286.6	5735	27592	Blue_2	287	0.0	Pass	500.0		

Sample 71: Run date and time: 09/15/2020 - 21:56:48 -> 09/15/2020 - 22:42:27

Dye: Blue - 1 peaks - 73.fsa

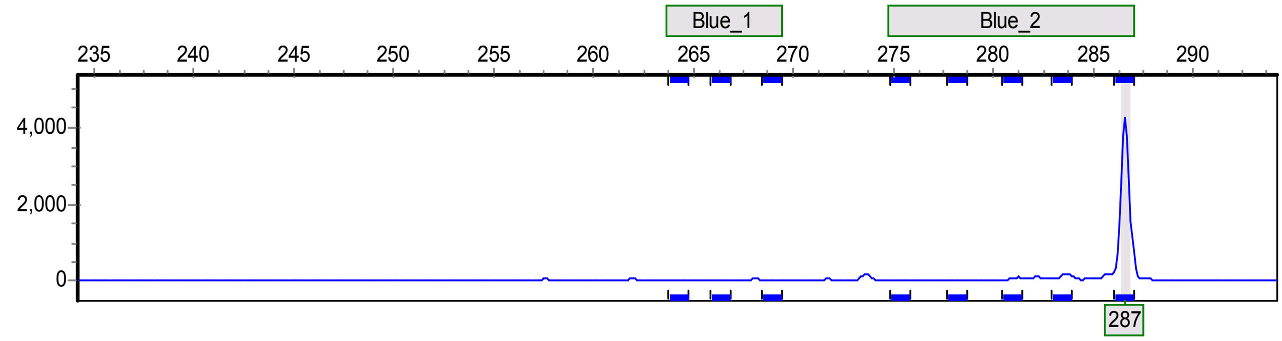

No	Size	Height	Area	Marker	Allele	Difference	Quality	Score	Allele Comments	Sample Comments
1	286.6	4269	20778	Blue_2	287	0.0	Pass	500.0		

Sample 72: Run date and time: 09/15/2020 - 21:56:48 -> 09/15/2020 - 22:42:27

Dye: Blue - 1 peaks - 74.fsa

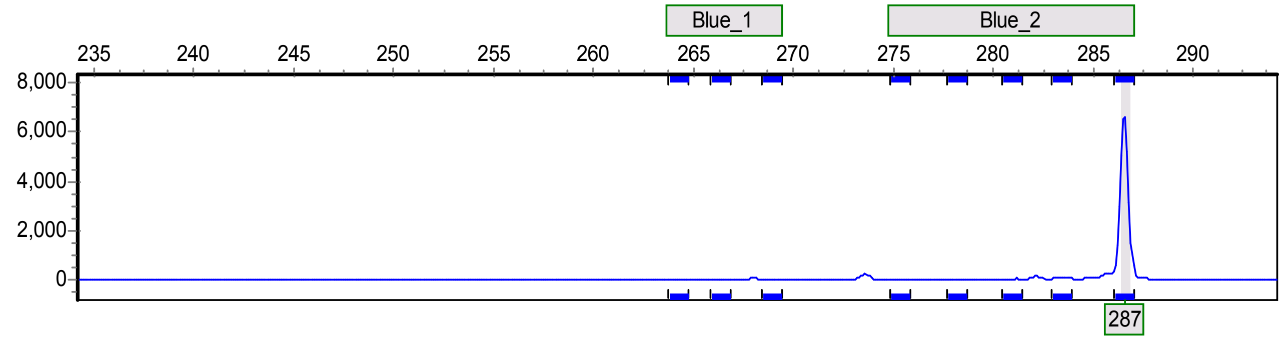

No	Size	Height	Area	Marker	Allele	Difference	Quality	Score	Allele Comments	Sample Comments
1	286.6	6576	31350	Blue_2	287	0.0	Pass	500.0		

Allele Report

Sample 73: Run date and time: 09/15/2020 - 21:56:48 -> 09/15/2020 - 22:42:27

Dye: Blue - 1 peaks - 75.fsa

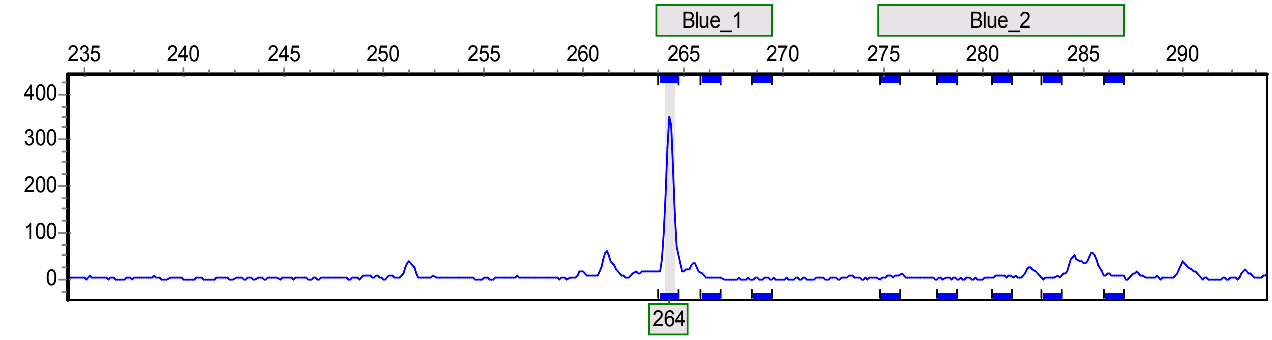

No	Size	Height	Area	Marker	Allele	Difference	Quality	Score	Allele Comments	Sample Comments
1	264.3	348	1653	Blue_1	264	0.0	Pass	44.2		

Sample 74: Run date and time: 09/15/2020 - 21:56:48 -> 09/15/2020 - 22:42:27

Dye: Blue - 1 peaks - 76.fsa

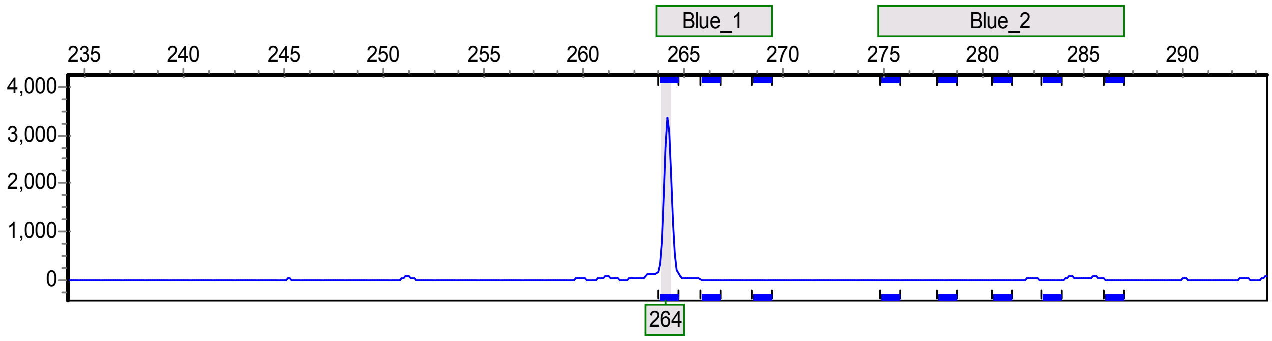

No	Size	Height	Area	Marker	Allele	Difference	Quality	Score	Allele Comments	Sample Comments
1	264.2	3338	15007	Blue_1	264	0.1	Pass	500.0		

Sample 75: Run date and time: 09/15/2020 - 21:56:48 -> 09/15/2020 - 22:42:27

Dye: Blue - 0 peaks - 77.fsa

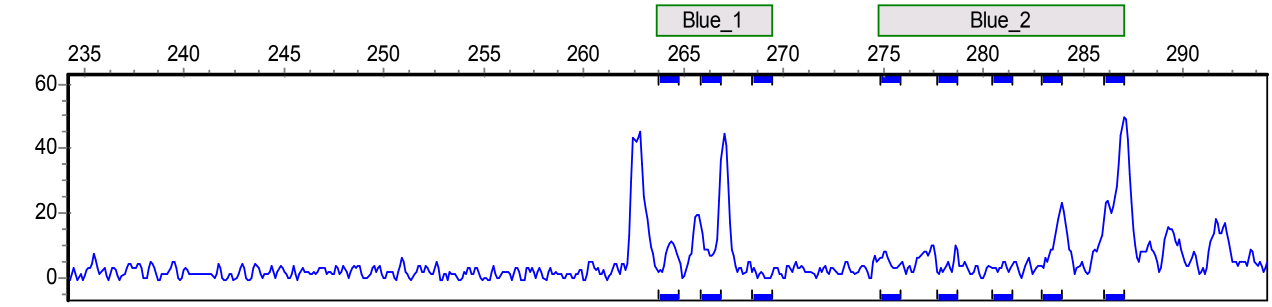

No	Size	Height	Area	Marker	Allele	Difference	Quality	Score	Allele Comments	Sample Comments

Allele Report

Sample 76: Run date and time: 09/15/2020 - 21:56:48 -> 09/15/2020 - 22:42:27

Dye: Blue - 1 peaks - 78.fsa

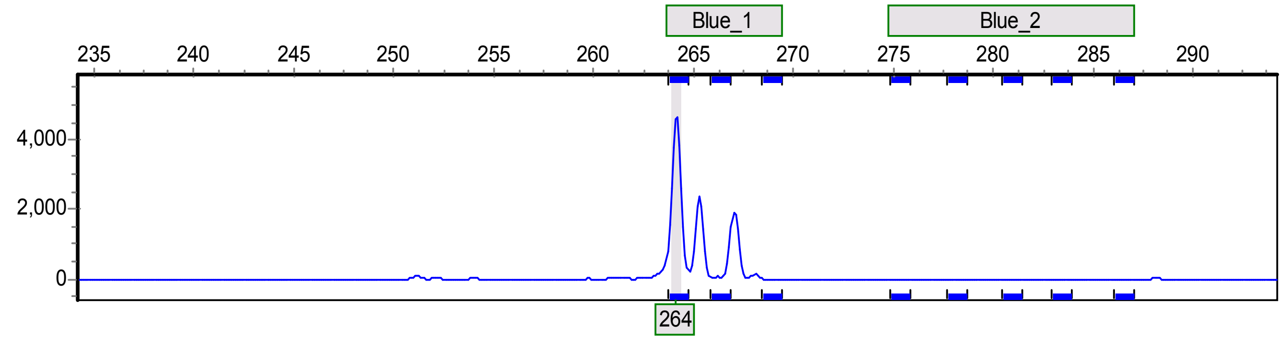

No	Size	Height	Area	Marker	Allele	Difference	Quality	Score	Allele Comments	Sample Comments
1	264.2	4584	25327	Blue_1	264	0.1	Pass	500.0		

Sample 77: Run date and time: 09/15/2020 - 21:56:48 -> 09/15/2020 - 22:42:27

Dye: Blue - 1 peaks - 79.fsa

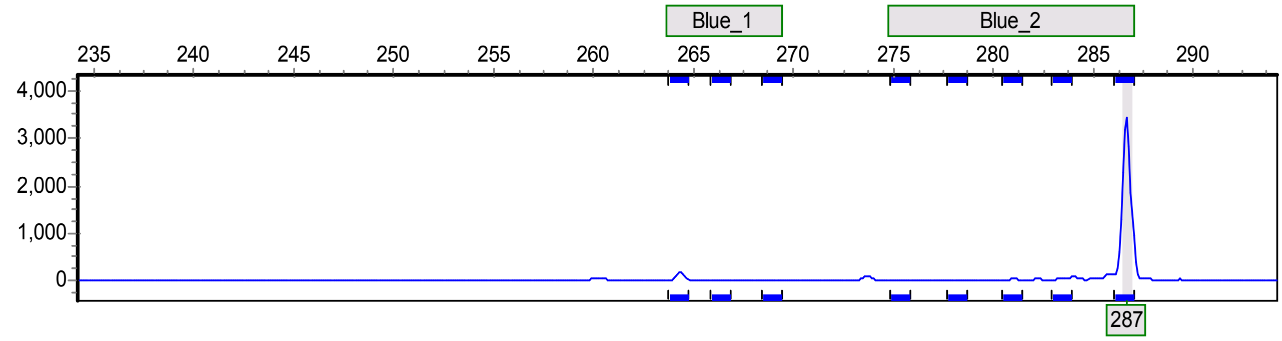

No	Size	Height	Area	Marker	Allele	Difference	Quality	Score	Allele Comments	Sample Comments
1	286.7	3420	15884	Blue_2	287	0.1	Pass	500.0		

Sample 78: Run date and time: 09/15/2020 - 21:56:48 -> 09/15/2020 - 22:42:27

Dye: Blue - 0 peaks - 8.fsa

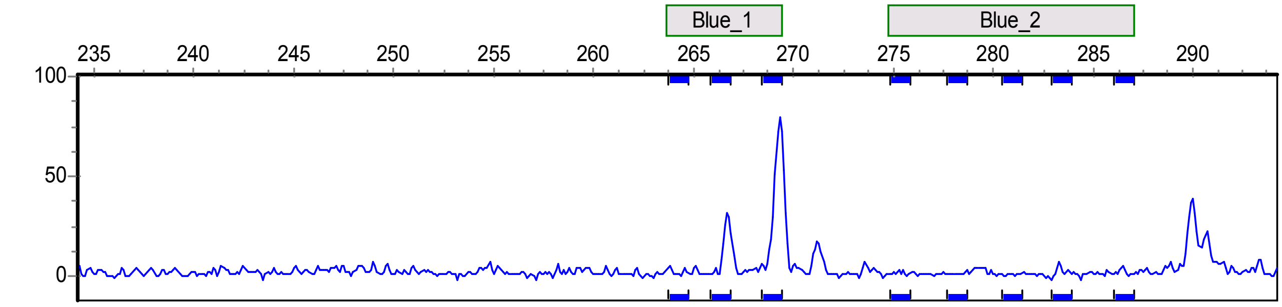

No	Size	Height	Area	Marker	Allele	Difference	Quality	Score	Allele Comments	Sample Comments

Allele Report

Sample 79: Run date and time: 09/15/2020 - 21:56:48 -> 09/15/2020 - 22:42:27

Dye: Blue - 2 peaks - 80.fsa

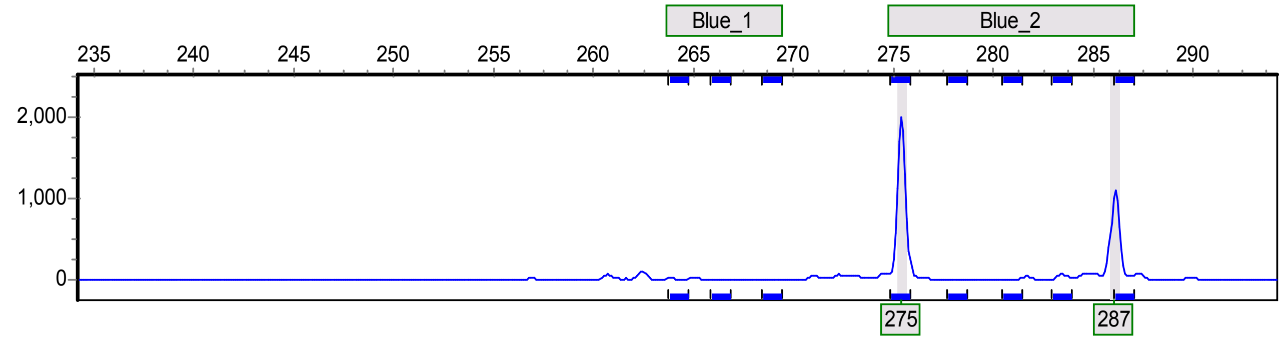

No	Size	Height	Area	Marker	Allele	Difference	Quality	Score	Allele Comments	Sample Comments
1	275.4	1976	9287	Blue_2	275	0.0	Pass	500.0		
2	286.1	1097	5258	Blue_2	287	0.5	Pass	252.5		

Sample 80: Run date and time: 09/15/2020 - 21:56:48 -> 09/15/2020 - 22:42:27

Dye: Blue - 1 peaks - 81.fsa

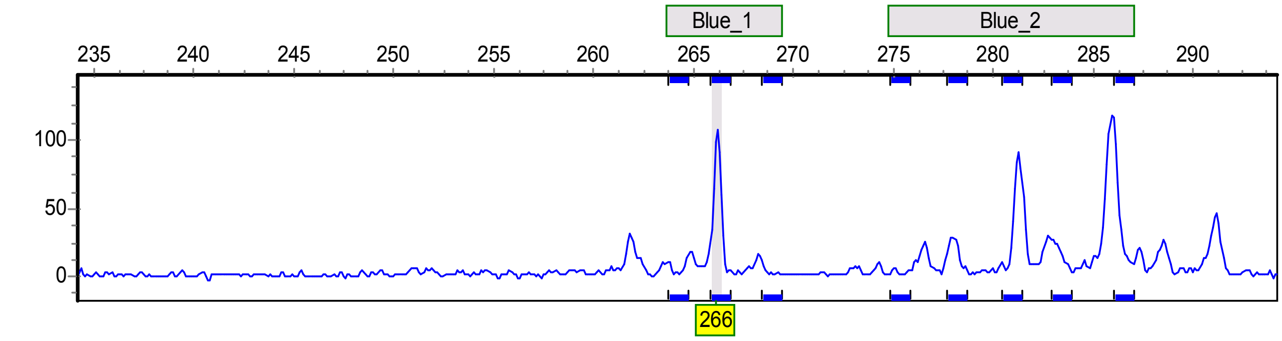

No	Size	Height	Area	Marker	Allele	Difference	Quality	Score	Allele Comments	Sample Comments
1	266.2	107	483	Blue_1	266	0.2	Check	6.1		

Sample 81: Run date and time: 09/15/2020 - 21:56:48 -> 09/15/2020 - 22:42:27

Dye: Blue - 0 peaks - 82.fsa

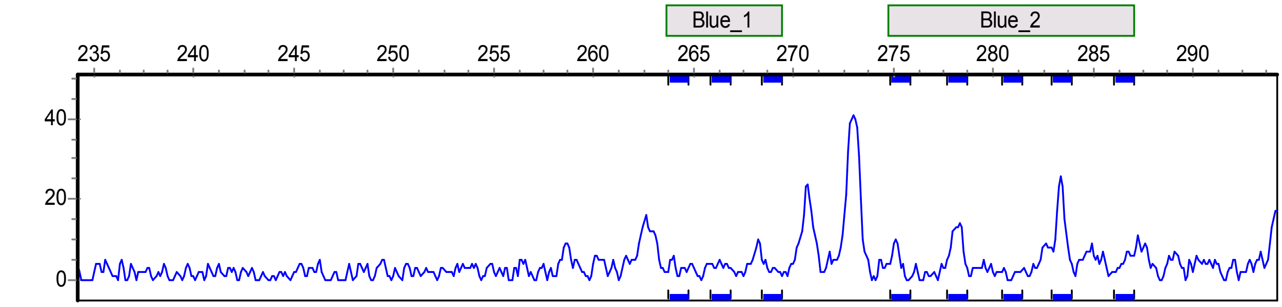

No	Size	Height	Area	Marker	Allele	Difference	Quality	Score	Allele Comments	Sample Comments

Allele Report

Sample 82: Run date and time: 09/15/2020 - 21:56:48 -> 09/15/2020 - 22:42:27

Dye: Blue - 2 peaks - 83.fsa

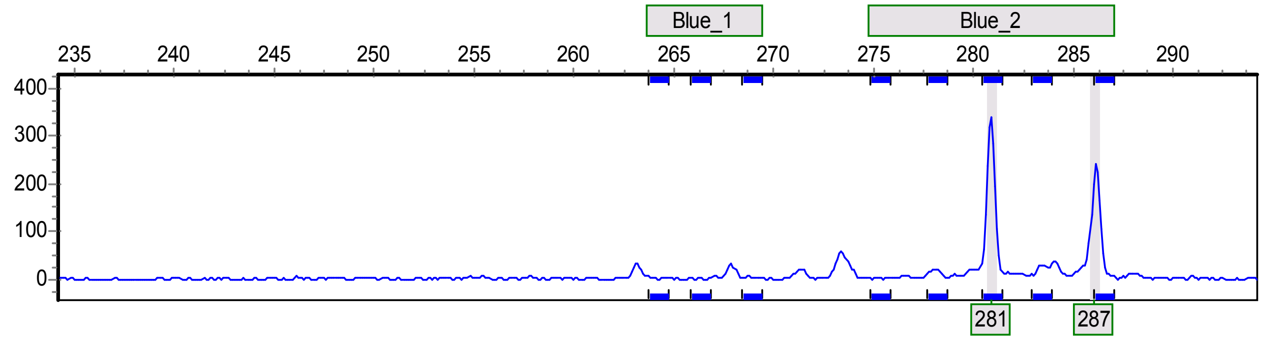

No	Size	Height	Area	Marker	Allele	Difference	Quality	Score	Allele Comments	Sample Comments
1	280.9	338	1644	Blue_2	281	0.1	Pass	40.6		
2	286.1	241	1203	Blue_2	287	0.5	Pass	21.9		

Sample 83: Run date and time: 09/15/2020 - 21:56:48 -> 09/15/2020 - 22:42:27

Dye: Blue - 1 peaks - 84.fsa

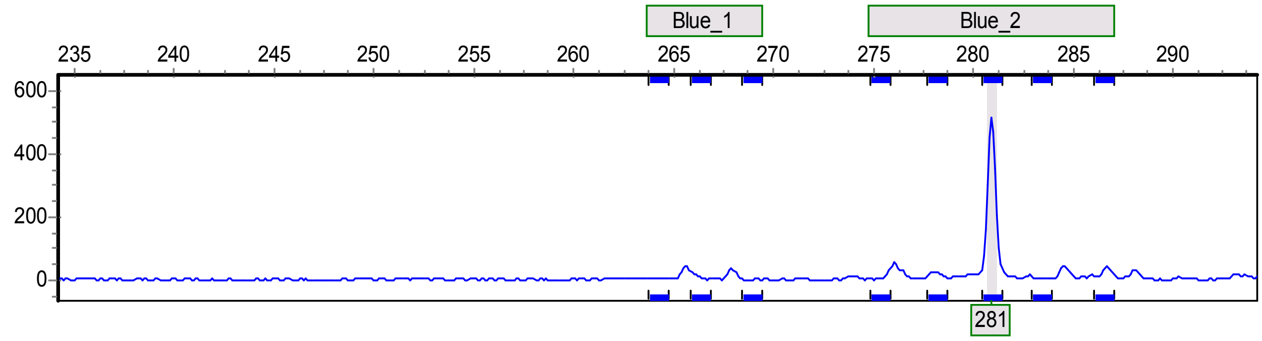

No	Size	Height	Area	Marker	Allele	Difference	Quality	Score	Allele Comments	Sample Comments
1	280.9	514	2532	Blue_2	281	0.1	Pass	78.0		

Sample 84: Run date and time: 09/15/2020 - 21:56:48 -> 09/15/2020 - 22:42:27

Dye: Blue - 1 peaks - 85.fsa

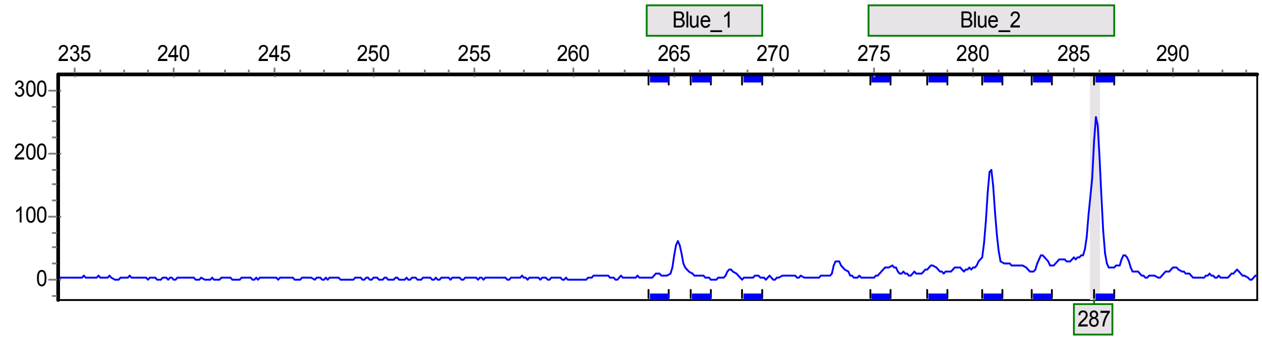

No	Size	Height	Area	Marker	Allele	Difference	Quality	Score	Allele Comments	Sample Comments
1	286.1	257	1486	Blue_2	287	0.5	Pass	19.9		

Sample 85: Run date and time: 09/15/2020 - 21:56:48 -> 09/15/2020 - 22:42:27

Dye: Blue - 0 peaks - 86.fsa

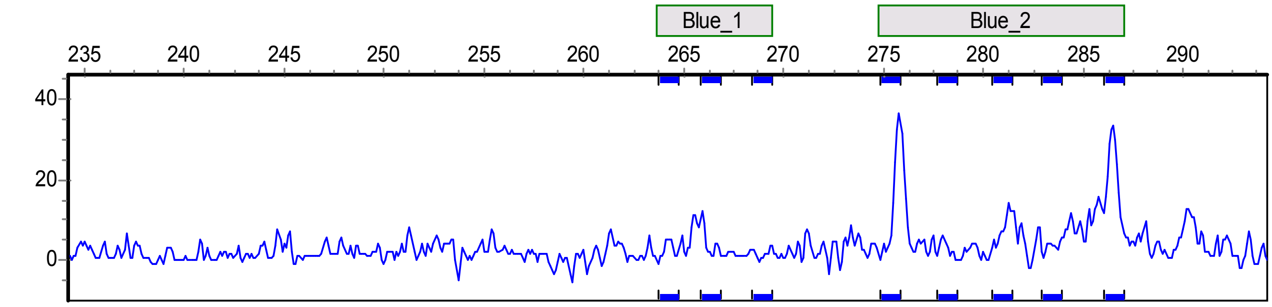

No	Size	Height	Area	Marker	Allele	Difference	Quality	Score	Allele Comments	Sample Comments

Sample 86: Run date and time: 09/15/2020 - 21:56:48 -> 09/15/2020 - 22:42:27

Dye: Blue - 2 peaks - 87.fsa

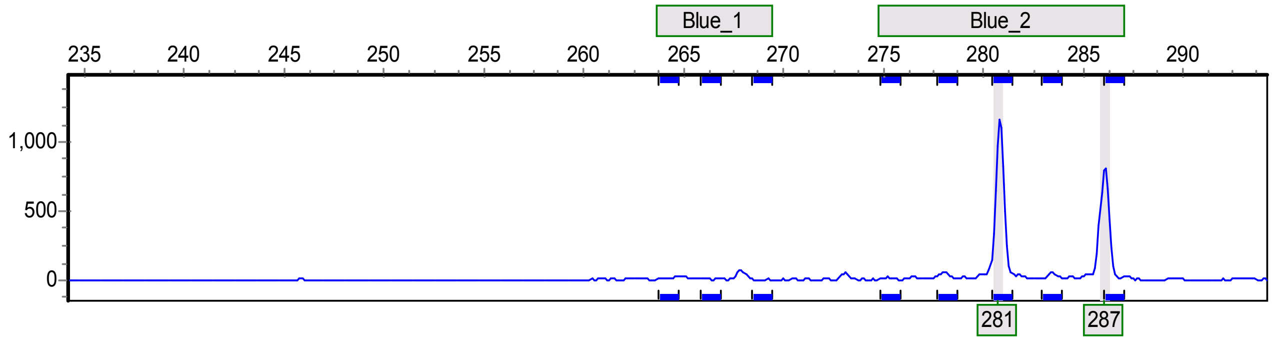

No	Size	Height	Area	Marker	Allele	Difference	Quality	Score	Allele Comments	Sample Comments
1	280.8	1165	5680	Blue_2	281	0.2	Pass	269.9		
2	286.1	805	4096	Blue_2	287	0.5	Pass	147.9		

Sample 87: Run date and time: 09/15/2020 - 21:56:48 -> 09/15/2020 - 22:42:27

Dye: Blue - 1 peaks - 88.fsa

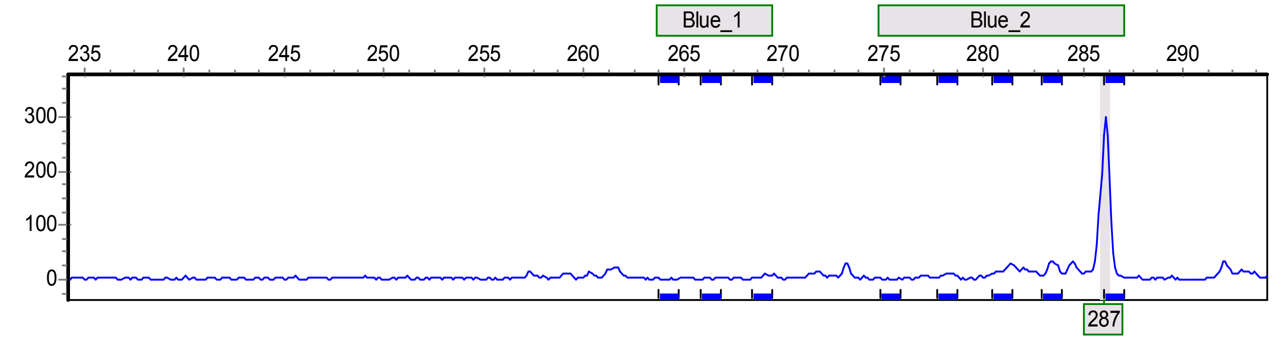

No	Size	Height	Area	Marker	Allele	Difference	Quality	Score	Allele Comments	Sample Comments
1	286.1	300	1495	Blue_2	287	0.5	Pass	32.4		

Sample 88: Run date and time: 09/15/2020 - 21:56:48 -> 09/15/2020 - 22:42:27

Dye: Blue - 0 peaks - 89.fsa

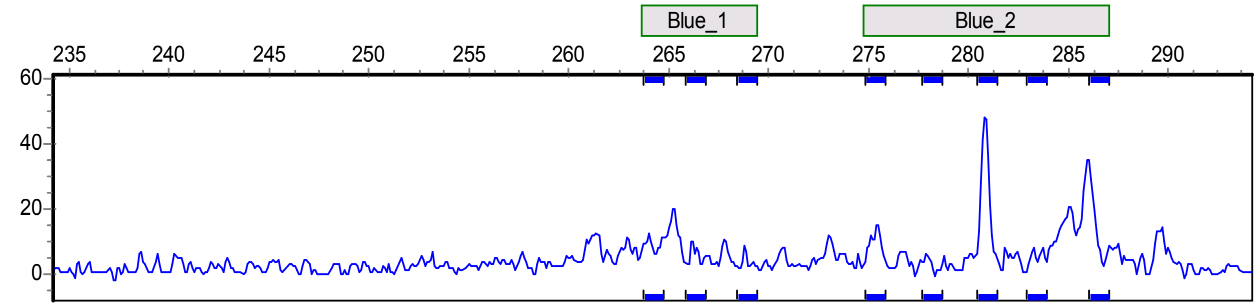

No	Size	Height	Area	Marker	Allele	Difference	Quality	Score	Allele Comments	Sample Comments

Sample 89: Run date and time: 09/15/2020 - 21:56:48 -> 09/15/2020 - 22:42:27

Dye: Blue - 2 peaks - 9.fsa

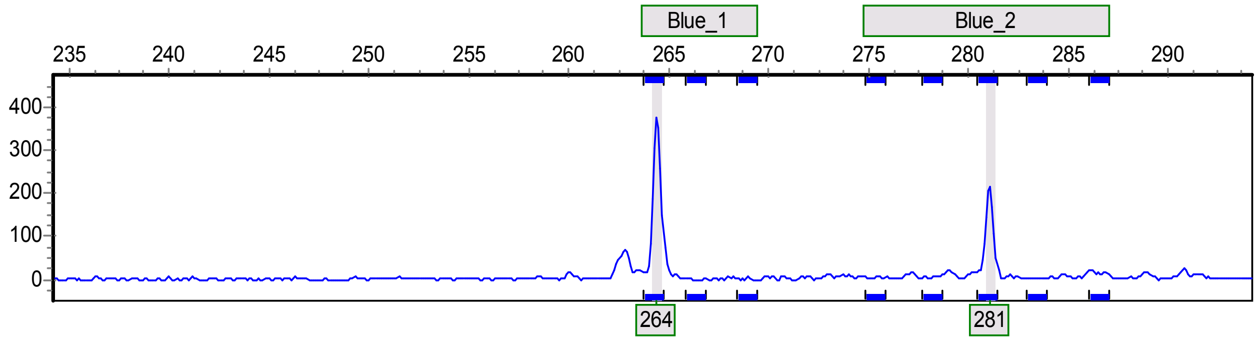

No	Size	Height	Area	Marker	Allele	Difference	Quality	Score	Allele Comments	Sample Comments
1	264.4	373	1703	Blue_1	264	0.1	Pass	51.8		
2	281.1	212	985	Blue_2	281	0.1	Pass	20.1		

Sample 90: Run date and time: 09/15/2020 - 21:56:48 -> 09/15/2020 - 22:42:27

Dye: Blue - 1 peaks - 90.fsa

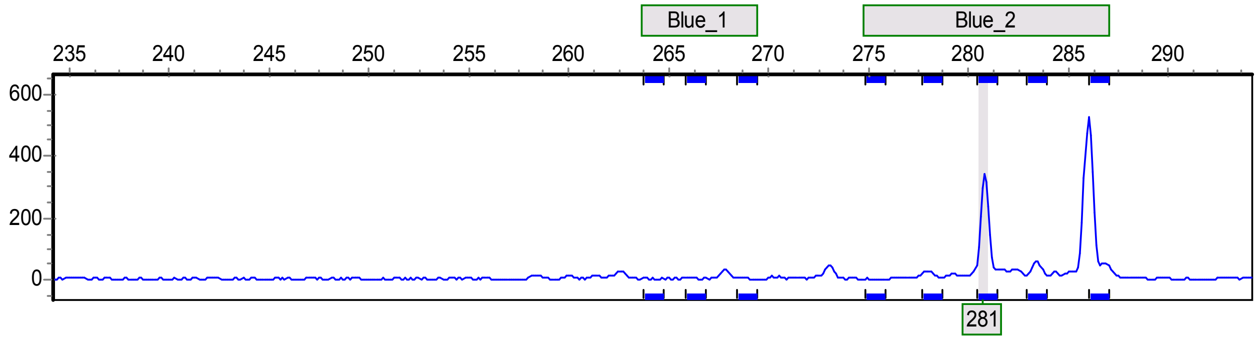

No	Size	Height	Area	Marker	Allele	Difference	Quality	Score	Allele Comments	Sample Comments
1	280.8	340	1695	Blue_2	281	1.0	Pass	39.5	[<Confirmed><Inserted>]	

Allele Report

Sample 91: Run date and time: 09/15/2020 - 21:56:48 -> 09/15/2020 - 22:42:27

Dye: Blue - 0 peaks - 91.fsa

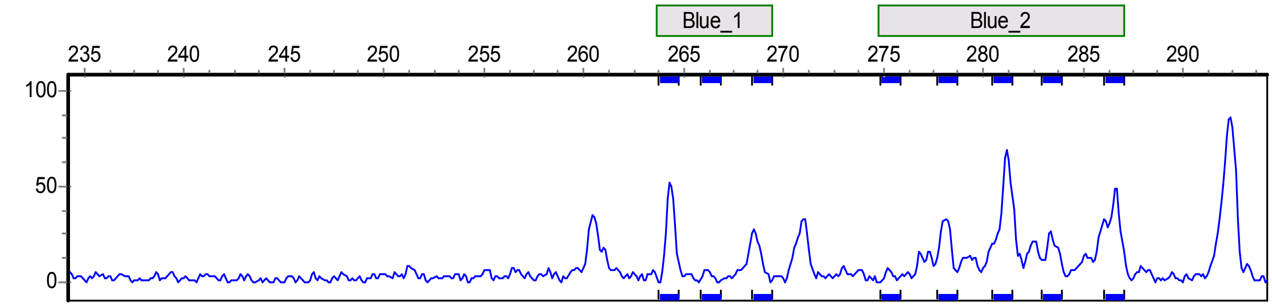

No	Size	Height	Area	Marker	Allele	Difference	Quality	Score	Allele Comments	Sample Comments

Sample 92: Run date and time: 09/15/2020 - 21:56:48 -> 09/15/2020 - 22:42:27

Dye: Blue - 1 peaks - 92.fsa

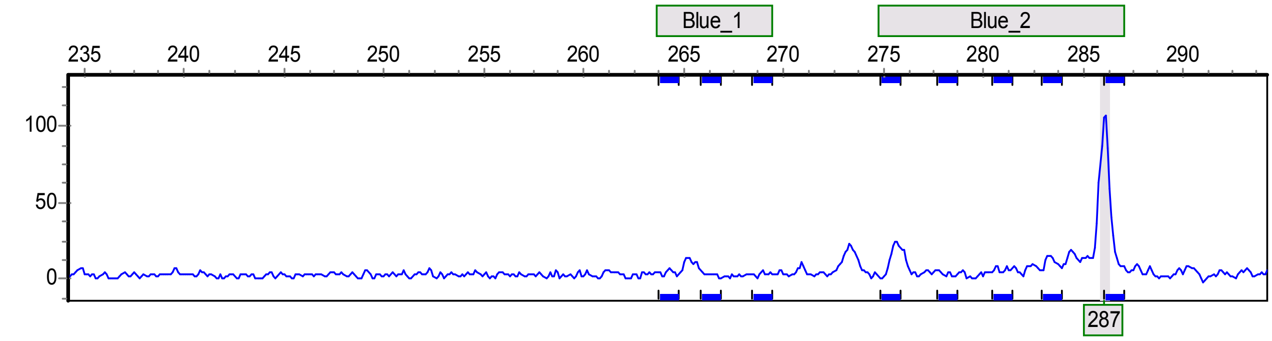

No	Size	Height	Area	Marker	Allele	Difference	Quality	Score	Allele Comments	Sample Comments
1	286.1	106	581	Blue_2	287	0.5	Pass	4.1	[<Confirmed>]	

Sample 93: Run date and time: 09/15/2020 - 21:56:48 -> 09/15/2020 - 22:42:27

Dye: Blue - 1 peaks - 93.fsa

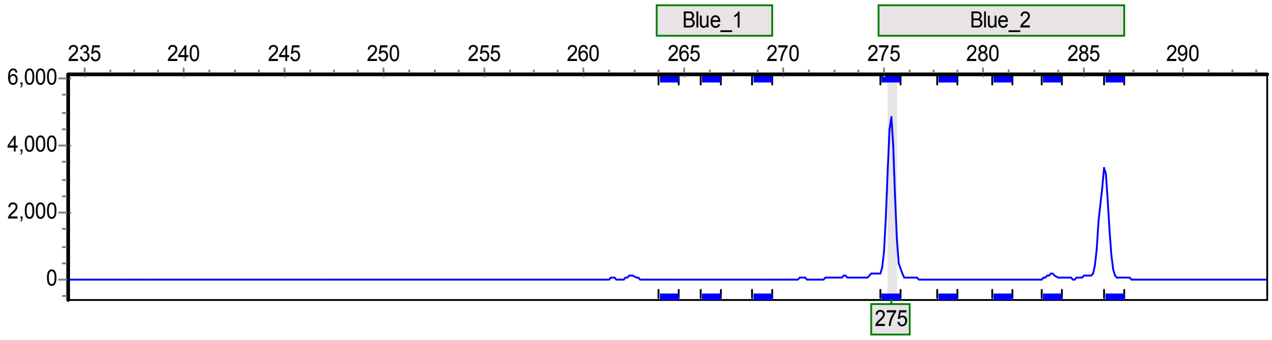

No	Size	Height	Area	Marker	Allele	Difference	Quality	Score	Allele Comments	Sample Comments
1	275.4	4804	22203	Blue_2	275	0.0	Pass	500.0		

Sample 94: Run date and time: 09/15/2020 - 21:56:48 -> 09/15/2020 - 22:42:27

Dye: Blue - 3 peaks - 94.fsa

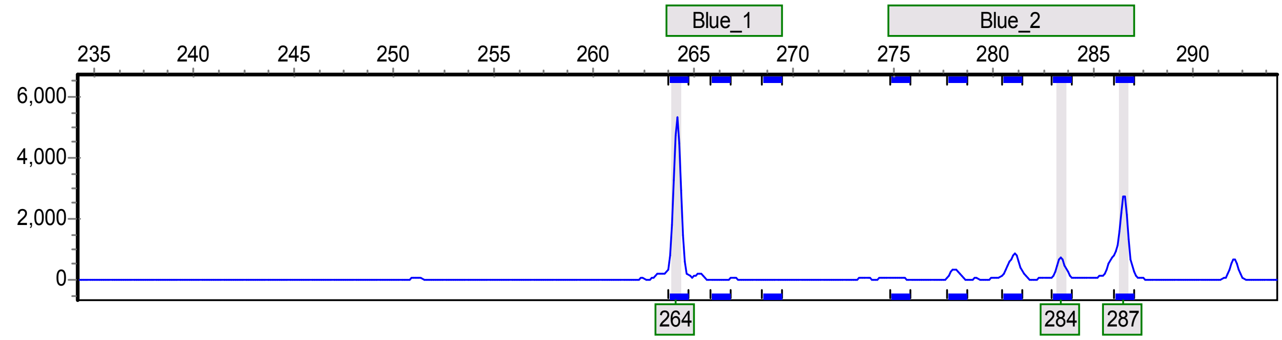

No	Size	Height	Area	Marker	Allele	Difference	Quality	Score	Allele Comments	Sample Comments
1	264.2	5330	24187	Blue_1	264	0.1	Pass	500.0		
2	283.4	755	3705	Blue_2	284	0.1	Pass	141.2		
3	286.5	2756	14447	Blue_2	287	0.1	Pass	500.0		

Sample 95: Run date and time: 09/15/2020 - 21:56:48 -> 09/15/2020 - 22:42:27

Dye: Blue - 2 peaks - 95.fsa

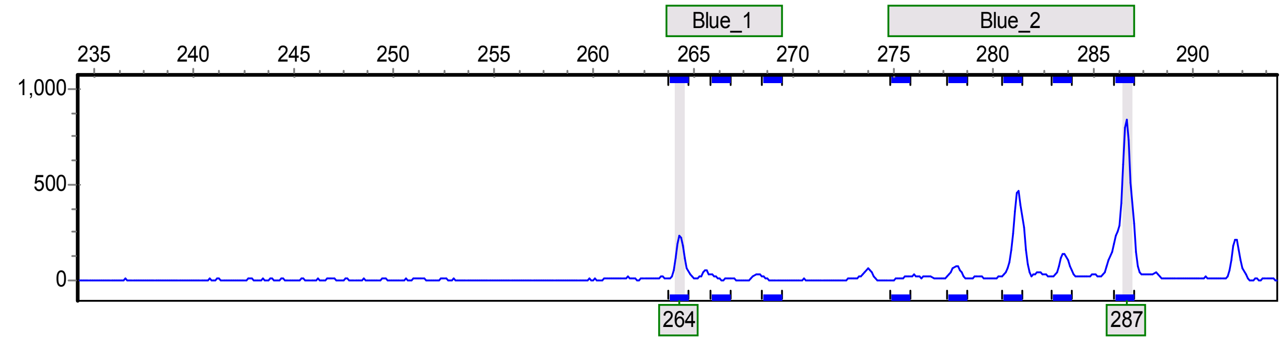

No	Size	Height	Area	Marker	Allele	Difference	Quality	Score	Allele Comments	Sample Comments
1	264.3	237	1158	Blue_1	264	0.0	Pass	22.3		
2	286.7	843	4504	Blue_2	287	0.1	Pass	144.5		

Sample 96: Run date and time: 09/15/2020 - 21:56:48 -> 09/15/2020 - 22:42:27

Dye: Blue - 0 peaks - 96.fsa

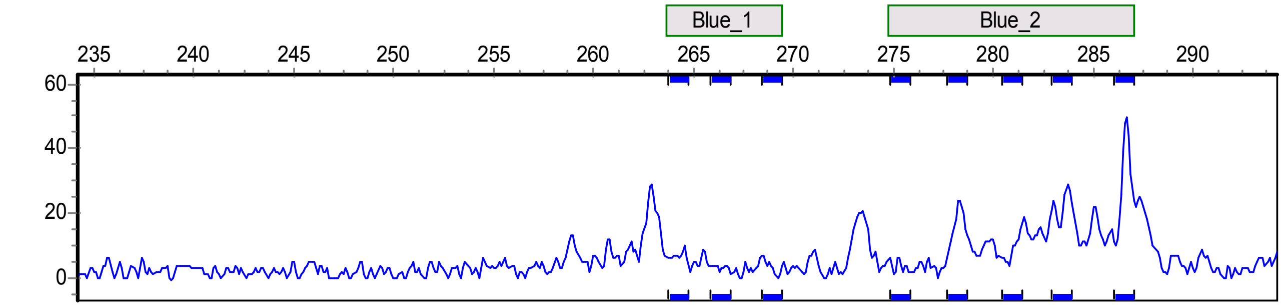

No	Size	Height	Area	Marker	Allele	Difference	Quality	Score	Allele Comments	Sample Comments
