# Supplementary material for: Genetic diversity and population structure of Polygonatum cyrtonema Hua in China using SSR markers
Source: PLoS One. 2023 Aug 31;18(8):e0290605. doi: 10.1371/journal.pone.0290605 (PMC10470896; doi:10.1371/journal.pone.0290605)
Supplement: S1 Data — (ZIP) [file pone.0290605.s002.zip › ╨┬╜¿╬─╝■╝╨ (2)/╥2╬∩40/yimwu40.pdf]

SoftGenetics | # Allele Report | 2020/9/16 17:53:01
GeneMarker V2.2.0 | |

Sample 1: Run date and time: 09/15/2020 - 22:42:28 -> 09/15/2020 - 23:28:27

Dye: Blue - 1 peaks - 1.fsa

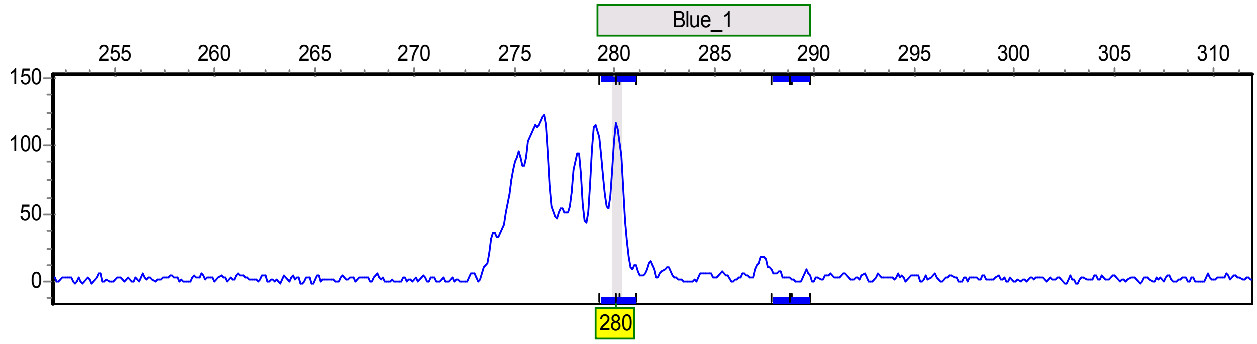

No	Size	Height	Area	Marker	Allele	Difference	Quality	Score	Allele Comments	Sample Comments
1	280.1	117	714	Blue_1	280	0.3	Check	3.1		

Sample 2: Run date and time: 09/15/2020 - 22:42:28 -> 09/15/2020 - 23:28:27

Dye: Blue - 0 peaks - 10.fsa

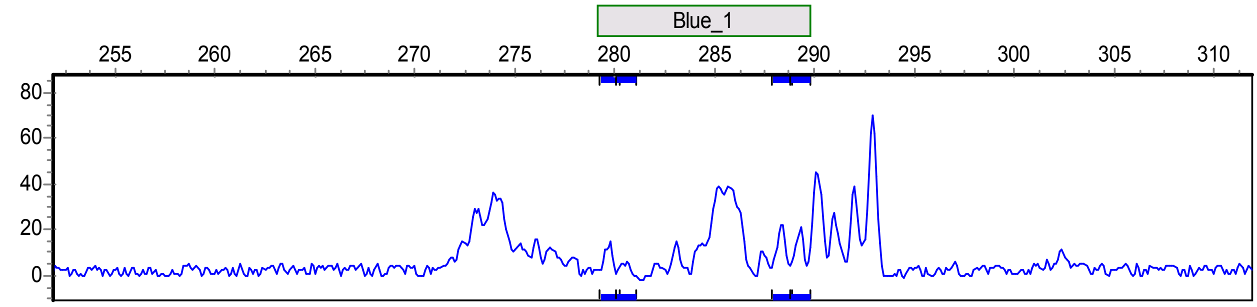

No	Size	Height	Area	Marker	Allele	Difference	Quality	Score	Allele Comments	Sample Comments

Sample 3: Run date and time: 09/15/2020 - 22:42:28 -> 09/15/2020 - 23:28:27

Dye: Blue - 0 peaks - 11.fsa

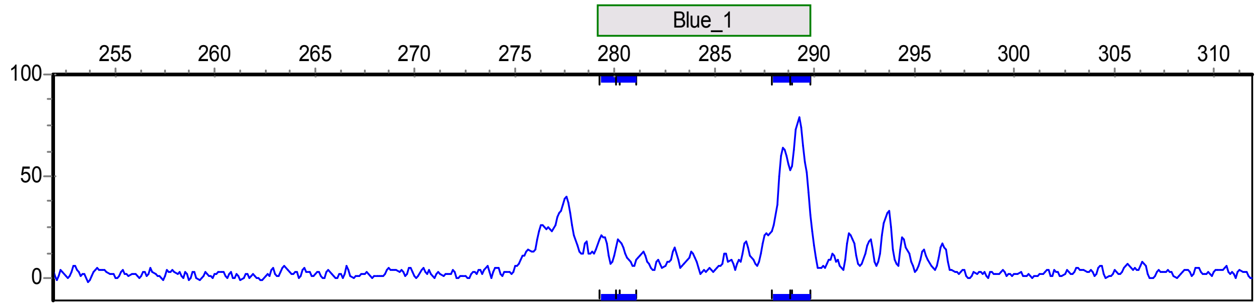

No	Size	Height	Area	Marker	Allele	Difference	Quality	Score	Allele Comments	Sample Comments

Allele Report

Sample 4: Run date and time: 09/15/2020 - 22:42:28 -> 09/15/2020 - 23:28:27

Dye: Blue - 0 peaks - 12.fsa

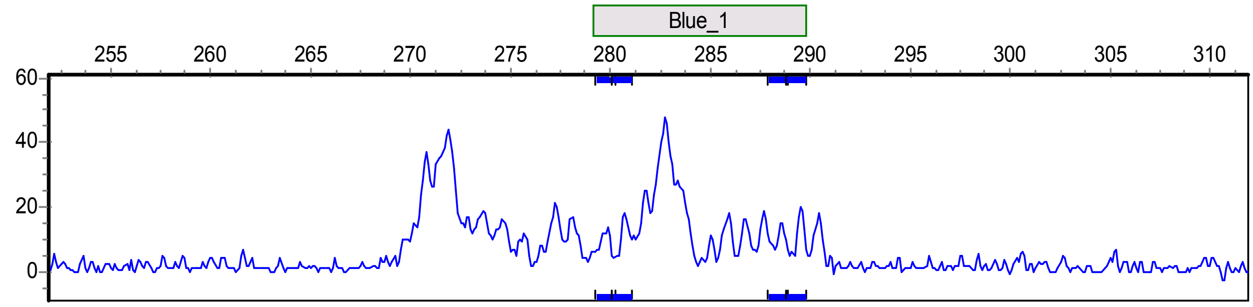

No	Size	Height	Area	Marker	Allele	Difference	Quality	Score	Allele Comments	Sample Comments

Sample 5: Run date and time: 09/15/2020 - 22:42:28 -> 09/15/2020 - 23:28:27

Dye: Blue - 1 peaks - 13.fsa

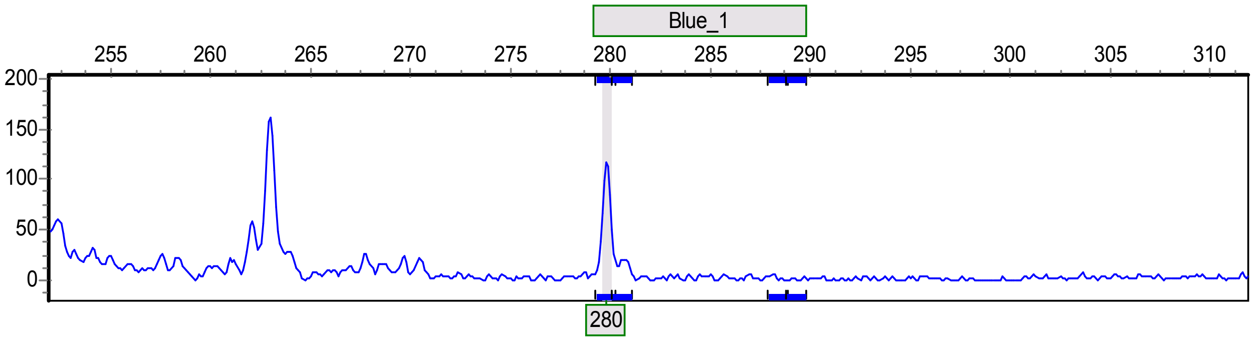

No	Size	Height	Area	Marker	Allele	Difference	Quality	Score	Allele Comments	Sample Comments
1	279.8	117	587	Blue_1	280	0.0	Pass	6.0	[<Confirmed>]	

Sample 6: Run date and time: 09/15/2020 - 22:42:28 -> 09/15/2020 - 23:28:27

Dye: Blue - 1 peaks - 14.fsa

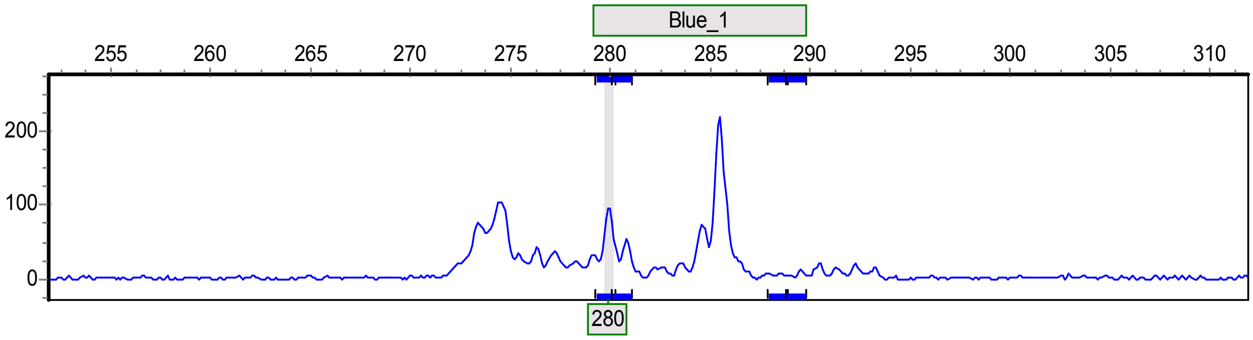

No	Size	Height	Area	Marker	Allele	Difference	Quality	Score	Allele Comments	Sample Comments
1	279.9	96	566	Blue_1	280	0.1	Pass	2.9	[<Confirmed>]	

Sample 7: Run date and time: 09/15/2020 - 22:42:28 -> 09/15/2020 - 23:28:27

Dye: Blue - 1 peaks - 15.fsa

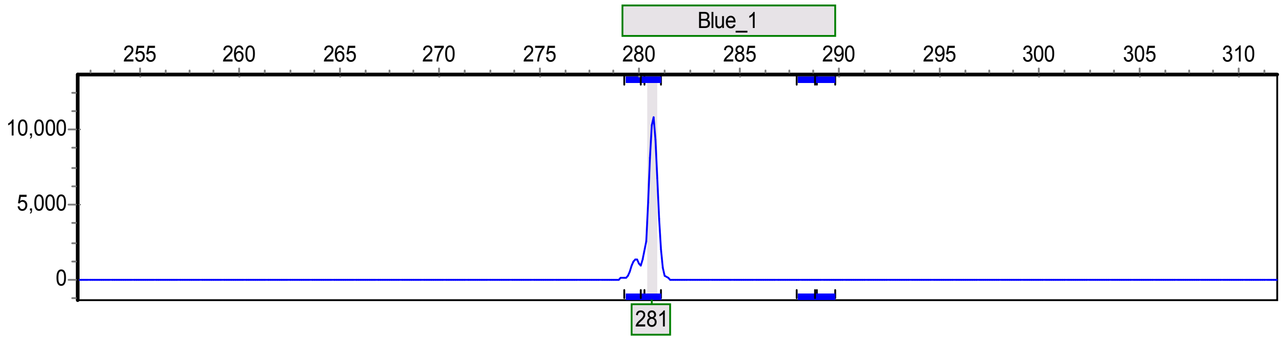

No	Size	Height	Area	Marker	Allele	Difference	Quality	Score	Allele Comments	Sample Comments
1	280.7	10708	57084	Blue_1	281	0.1	Pass	500.0		

Sample 8: Run date and time: 09/15/2020 - 22:42:28 -> 09/15/2020 - 23:28:27

Dye: Blue - 2 peaks - 16.fsa

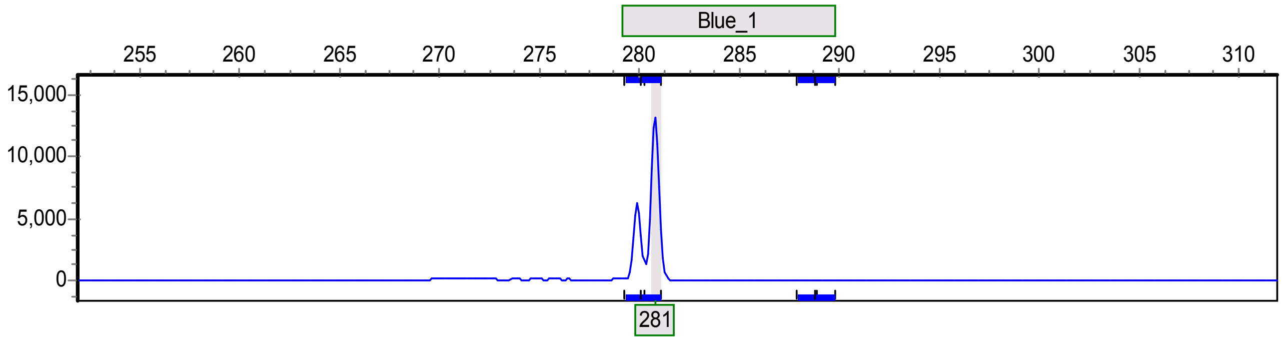

No	Size	Height	Area	Marker	Allele	Difference	Quality	Score	Allele Comments	Sample Comments
1	279.9	6217	27210	Blue_1	280	0.1	Pass	500.0	[<Deleted>]	
2	280.8	13111	62992	Blue_1	281	0.2	Pass	500.0		

Sample 9: Run date and time: 09/15/2020 - 22:42:28 -> 09/15/2020 - 23:28:27

Dye: Blue - 1 peaks - 17.fsa

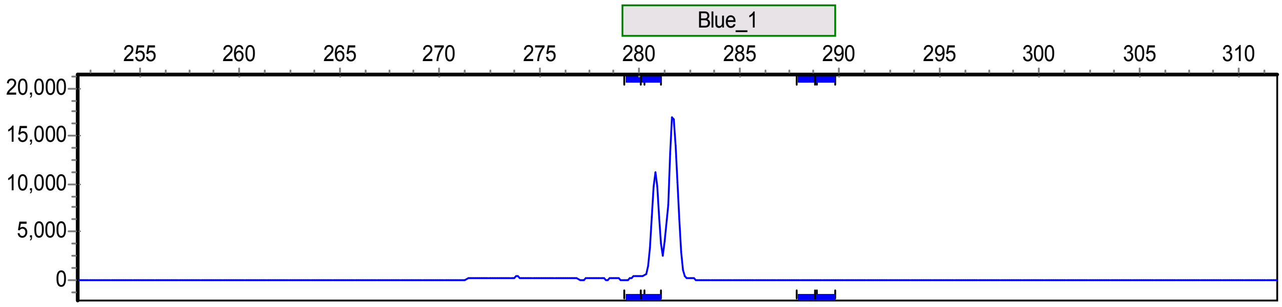

No	Size	Height	Area	Marker	Allele	Difference	Quality	Score	Allele Comments	Sample Comments
1	280.8	11188	50873	Blue_1	281	0.2	Pass	500.0	[<Deleted>]	

Sample 10: Run date and time: 09/15/2020 - 22:42:28 -> 09/15/2020 - 23:28:27

Dye: Blue - 1 peaks - 18.fsa

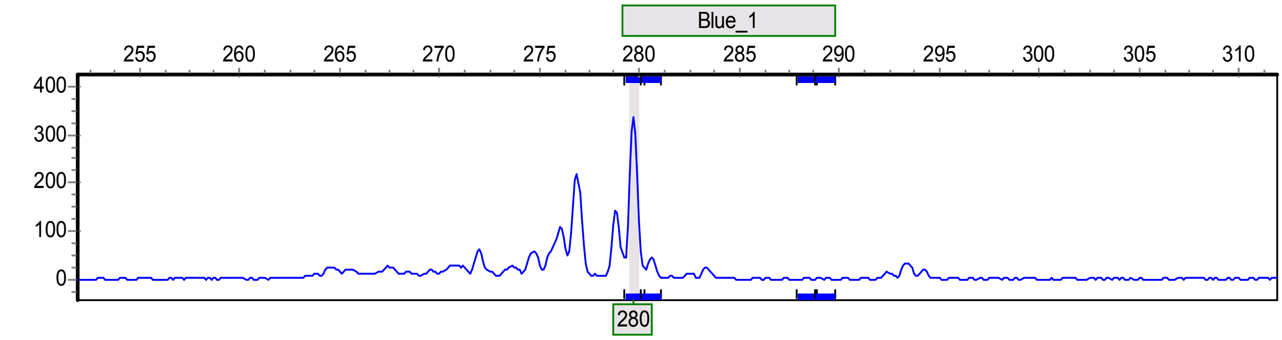

No	Size	Height	Area	Marker	Allele	Difference	Quality	Score	Allele Comments	Sample Comments
1	279.7	334	1639	Blue_1	280	0.1	Pass	38.9		

Sample 11: Run date and time: 09/15/2020 - 22:42:28 -> 09/15/2020 - 23:28:27

Dye: Blue - 1 peaks - 19.fsa

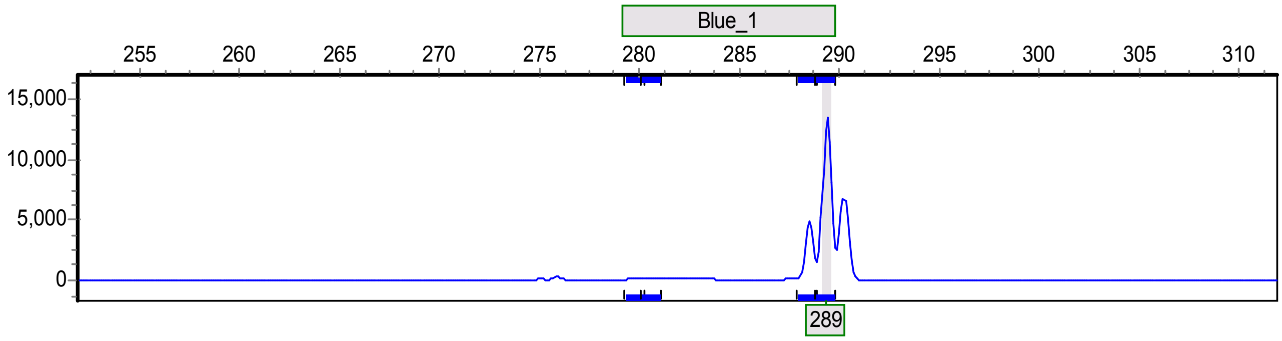

No	Size	Height	Area	Marker	Allele	Difference	Quality	Score	Allele Comments	Sample Comments
1	289.4	13405	64727	Blue_1	289	0.1	Pass	500.0		

Sample 12: Run date and time: 09/15/2020 - 22:42:28 -> 09/15/2020 - 23:28:27

Dye: Blue - 2 peaks - 2.fsa

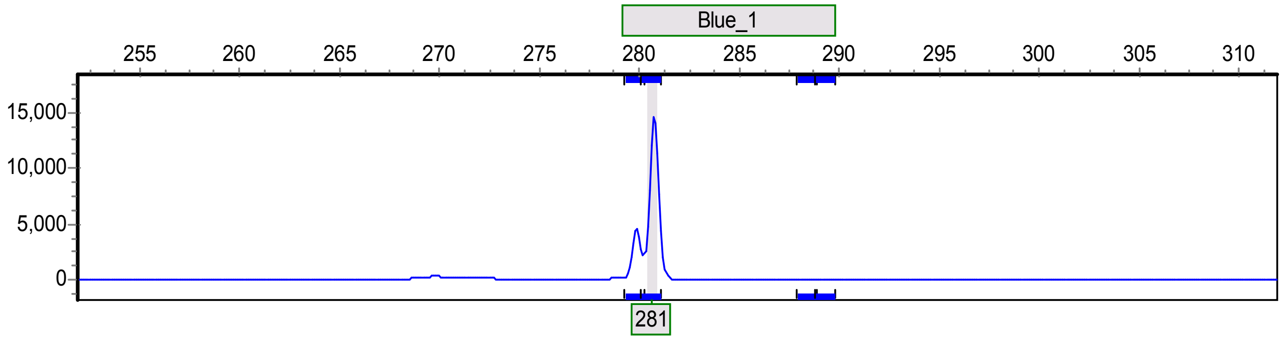

No	Size	Height	Area	Marker	Allele	Difference	Quality	Score	Allele Comments	Sample Comments
1	279.9	4524	24023	Blue_1	280	0.1	Pass	500.0	[<Deleted>]	
2	280.7	14463	77698	Blue_1	281	0.1	Pass	500.0		

Allele Report

Sample 13: Run date and time: 09/15/2020 - 22:42:28 -> 09/15/2020 - 23:28:27

Dye: Blue - 1 peaks - 20.fsa

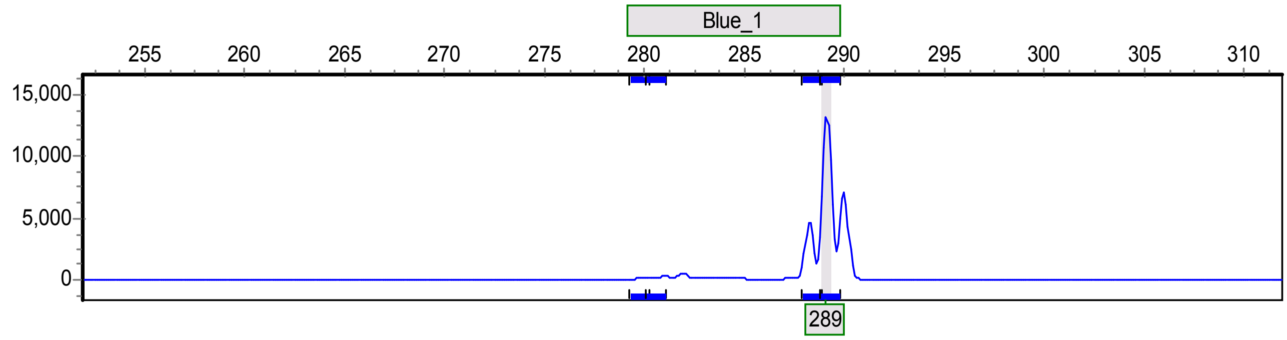

No	Size	Height	Area	Marker	Allele	Difference	Quality	Score	Allele Comments	Sample Comments
1	289.1	13135	65728	Blue_1	289	0.2	Pass	500.0		

Sample 14: Run date and time: 09/15/2020 - 22:42:28 -> 09/15/2020 - 23:28:27

Dye: Blue - 2 peaks - 21.fsa

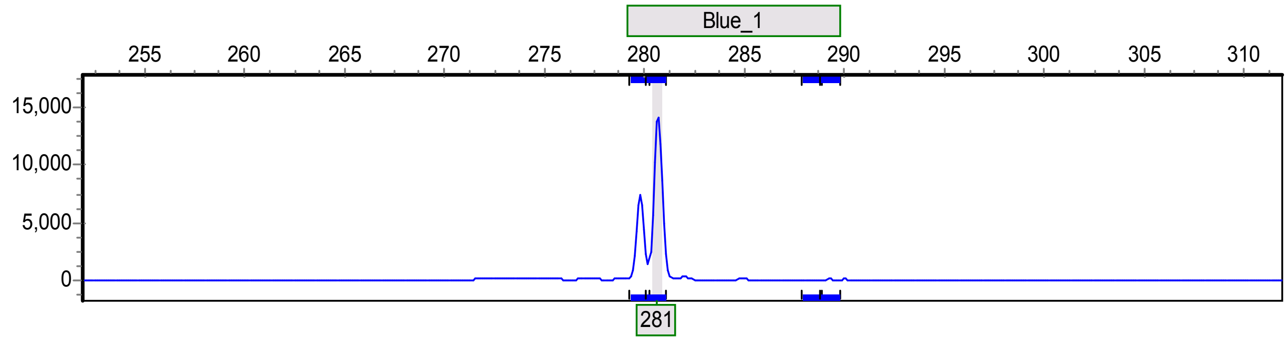

No	Size	Height	Area	Marker	Allele	Difference	Quality	Score	Allele Comments	Sample Comments
1	279.8	7481	33953	Blue_1	280	0.0	Pass	500.0	[<Deleted>]	
2	280.7	14027	70565	Blue_1	281	0.1	Pass	500.0		

Sample 15: Run date and time: 09/15/2020 - 22:42:28 -> 09/15/2020 - 23:28:27

Dye: Blue - 2 peaks - 22.fsa

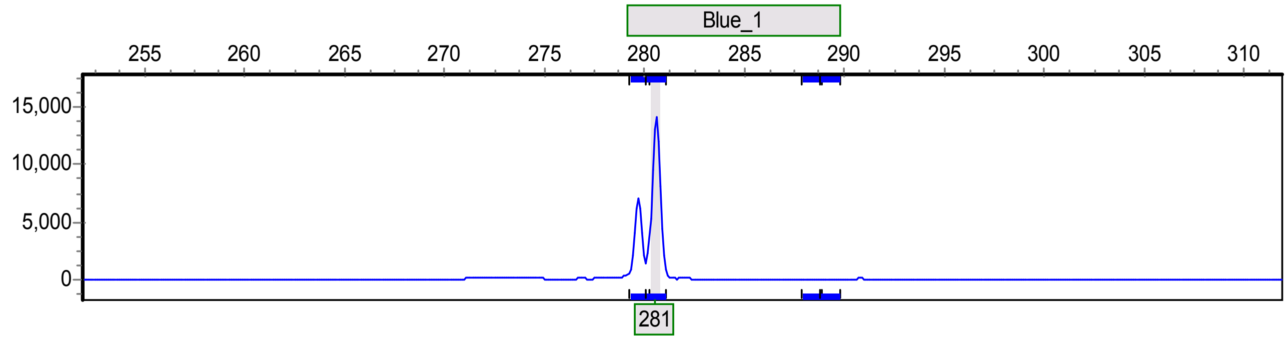

No	Size	Height	Area	Marker	Allele	Difference	Quality	Score	Allele Comments	Sample Comments
1	279.7	7110	31975	Blue_1	280	0.1	Pass	500.0	[<Deleted>]	
2	280.6	14052	67622	Blue_1	281	0.0	Pass	500.0		

Allele Report

Sample 16: Run date and time: 09/15/2020 - 22:42:28 -> 09/15/2020 - 23:28:27

Dye: Blue - 2 peaks - 23.fsa

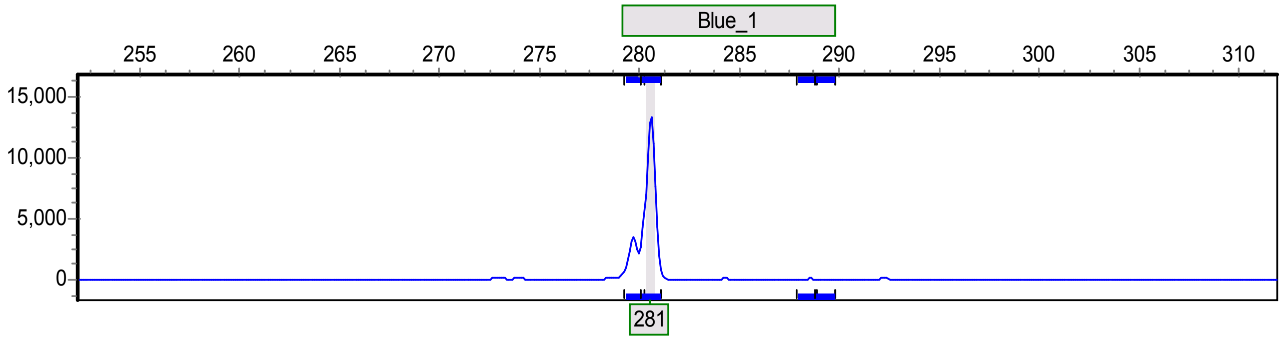

No	Size	Height	Area	Marker	Allele	Difference	Quality	Score	Allele Comments	Sample Comments
1	279.7	3532	20149	Blue_1	280	0.1	Pass	500.0	[<Deleted>]	
2	280.6	13265	73263	Blue_1	281	0.0	Pass	500.0		

Sample 17: Run date and time: 09/15/2020 - 22:42:28 -> 09/15/2020 - 23:28:27

Dye: Blue - 0 peaks - 24.fsa

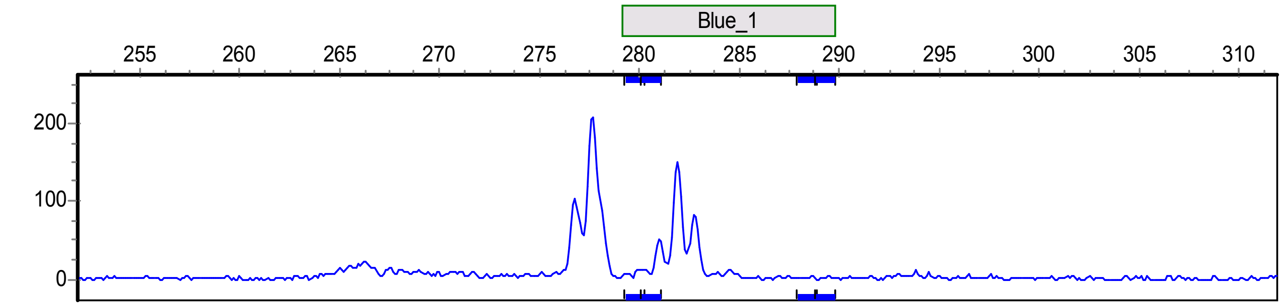

No	Size	Height	Area	Marker	Allele	Difference	Quality	Score	Allele Comments	Sample Comments

Sample 18: Run date and time: 09/15/2020 - 22:42:28 -> 09/15/2020 - 23:28:27

Dye: Blue - 1 peaks - 25.fsa

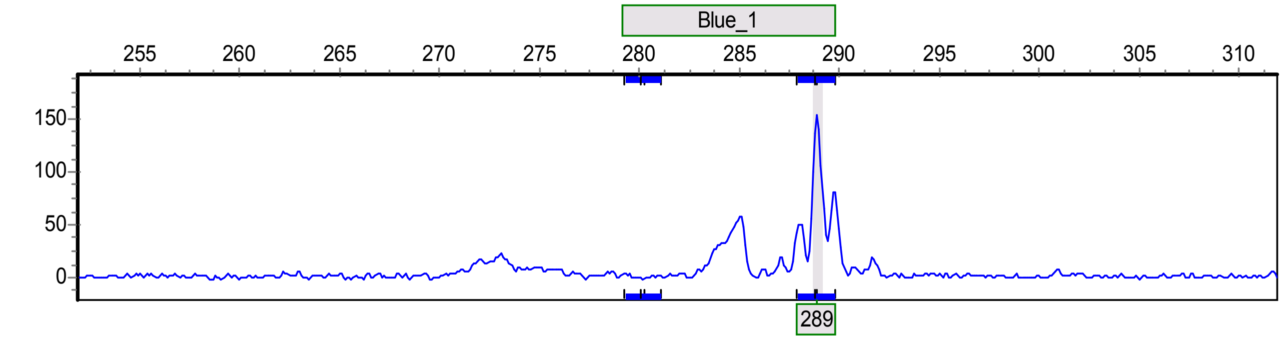

No	Size	Height	Area	Marker	Allele	Difference	Quality	Score	Allele Comments	Sample Comments
1	288.9	153	800	Blue_1	289	0.4	Pass	9.2	[<Confirmed>]	

Allele Report

Sample 19: Run date and time: 09/15/2020 - 22:42:28 -> 09/15/2020 - 23:28:27

Dye: Blue - 2 peaks - 26.fsa

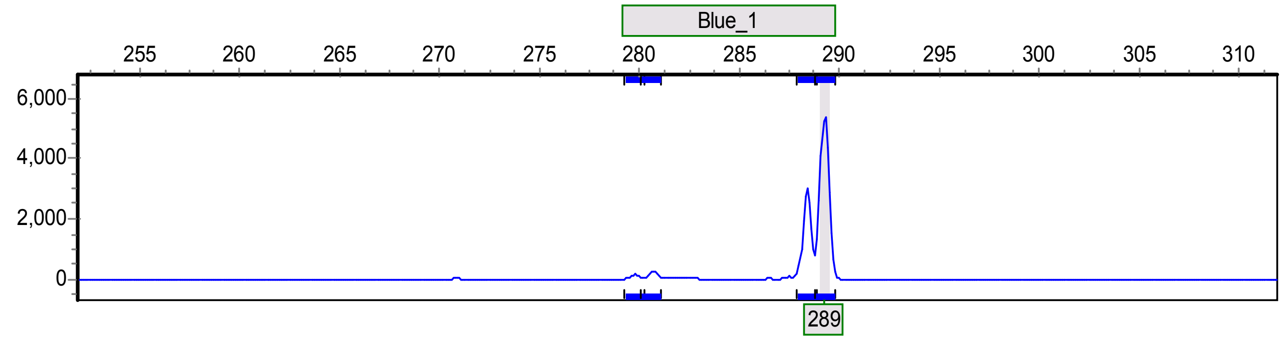

No	Size	Height	Area	Marker	Allele	Difference	Quality	Score	Allele Comments	Sample Comments
1	288.4	2999	13949	Blue_1	288	0.0	Pass	500.0	[<Deleted>]	
2	289.3	5337	27235	Blue_1	289	0.0	Pass	500.0		

Sample 20: Run date and time: 09/15/2020 - 22:42:28 -> 09/15/2020 - 23:28:27

Dye: Blue - 2 peaks - 27.fsa

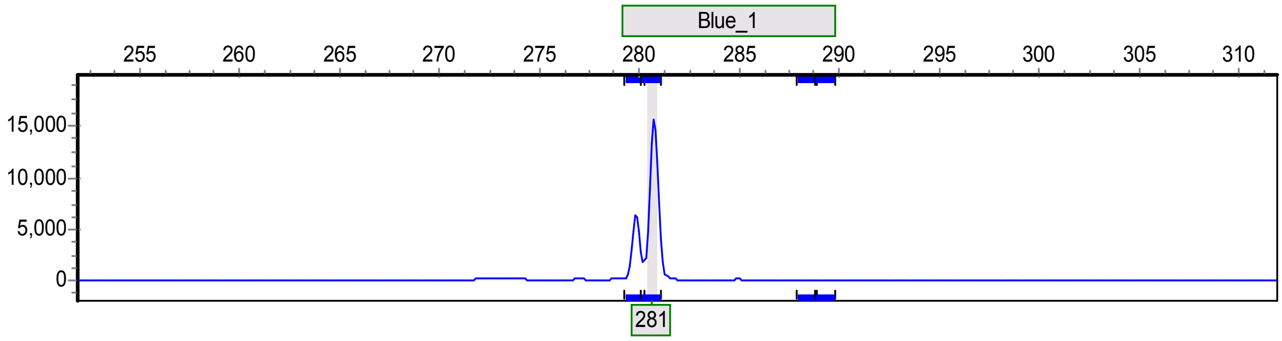

No	Size	Height	Area	Marker	Allele	Difference	Quality	Score	Allele Comments	Sample Comments
1	279.8	6255	30590	Blue_1	280	0.0	Pass	500.0	[<Deleted>]	
2	280.7	15741	79631	Blue_1	281	0.1	Pass	500.0	[<SAT (Repaired)><Confirmed>]	

Sample 21: Run date and time: 09/15/2020 - 22:42:28 -> 09/15/2020 - 23:28:27

Dye: Blue - 2 peaks - 28.fsa

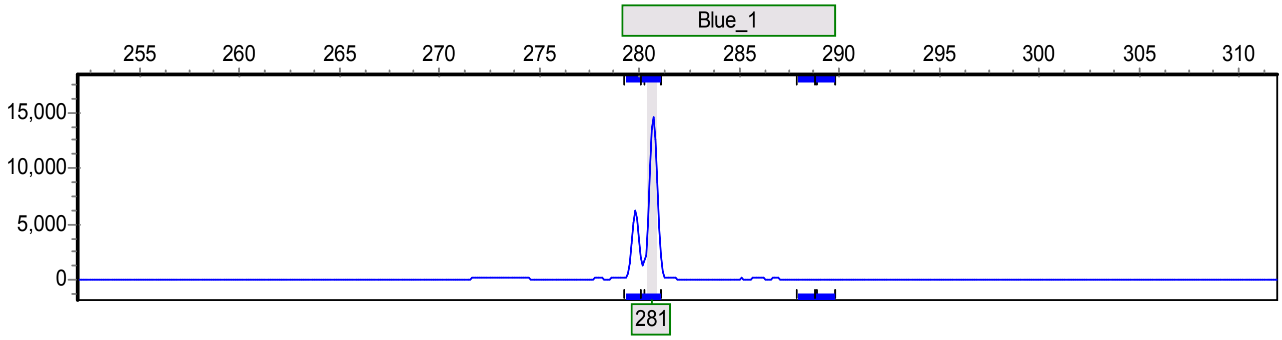

No	Size	Height	Area	Marker	Allele	Difference	Quality	Score	Allele Comments	Sample Comments
1	279.8	6214	26977	Blue_1	280	0.0	Pass	500.0	[<Deleted>]	
2	280.7	14493	70203	Blue_1	281	0.1	Pass	500.0		

Allele Report

Sample 22: Run date and time: 09/15/2020 - 22:42:28 -> 09/15/2020 - 23:28:27

Dye: Blue - 0 peaks - 29.fsa

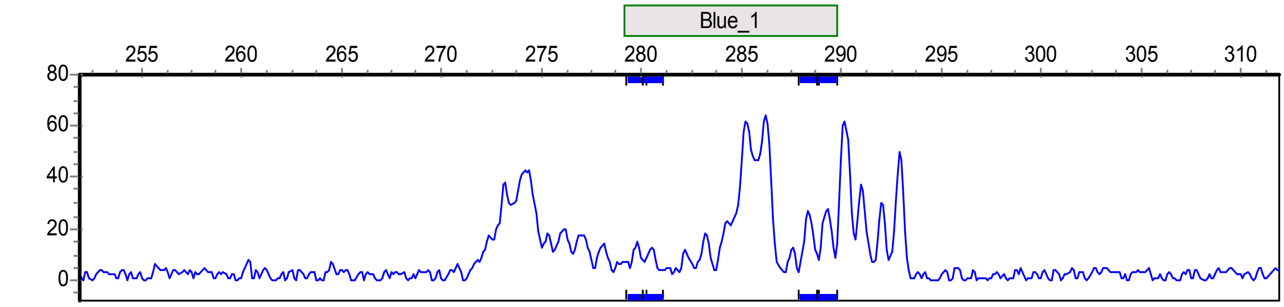

No	Size	Height	Area	Marker	Allele	Difference	Quality	Score	Allele Comments	Sample Comments

Sample 23: Run date and time: 09/15/2020 - 22:42:28 -> 09/15/2020 - 23:28:27

Dye: Blue - 1 peaks - 3.fsa

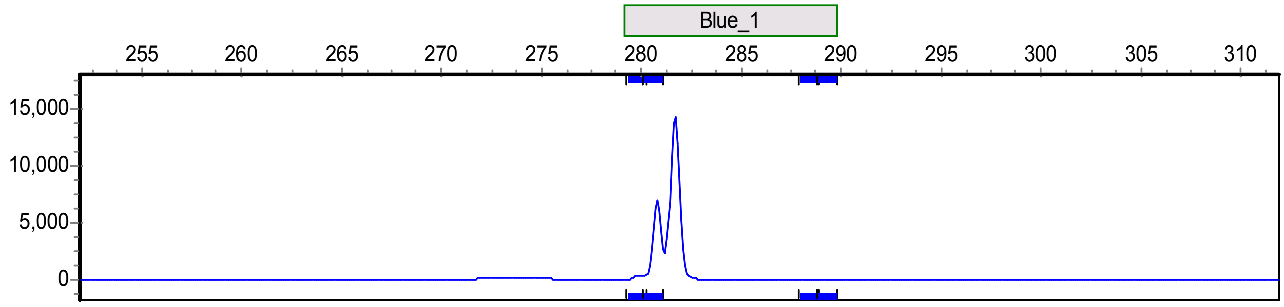

No	Size	Height	Area	Marker	Allele	Difference	Quality	Score	Allele Comments	Sample Comments
1	280.8	6976	33956	Blue_1	281	0.2	Pass	500.0	[<Deleted>]	

Sample 24: Run date and time: 09/15/2020 - 22:42:28 -> 09/15/2020 - 23:28:27

Dye: Blue - 2 peaks - 30.fsa

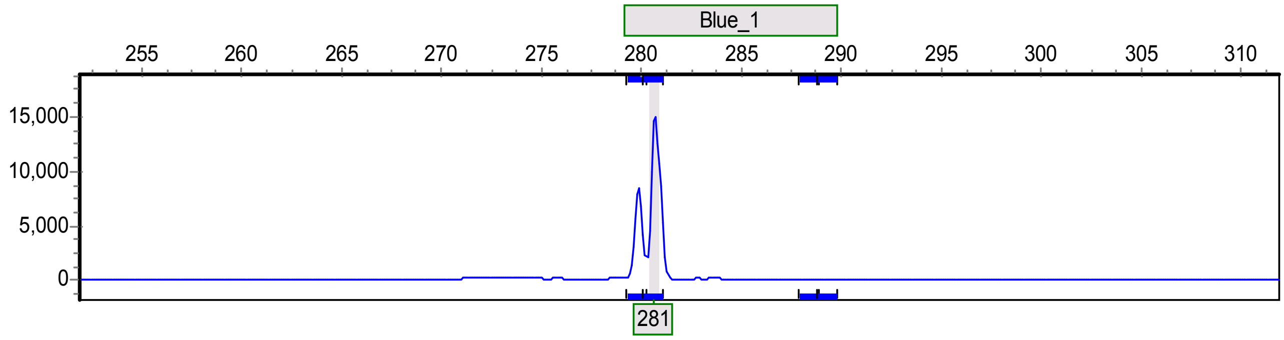

No	Size	Height	Area	Marker	Allele	Difference	Quality	Score	Allele Comments	Sample Comments
1	279.9	8398	38298	Blue_1	280	0.1	Pass	500.0	[<Deleted>]	
2	280.7	14982	81794	Blue_1	281	0.1	Pass	500.0		

Allele Report

Sample 25: Run date and time: 09/15/2020 - 22:42:28 -> 09/15/2020 - 23:28:27

Dye: Blue - 0 peaks - 31.fsa

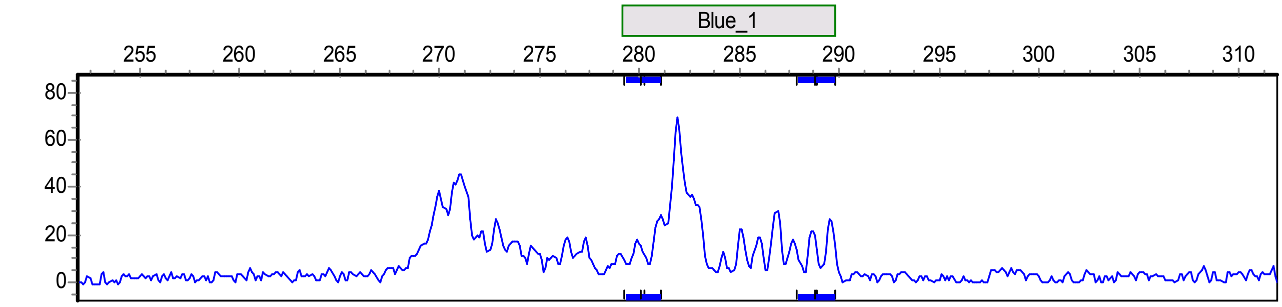

No	Size	Height	Area	Marker	Allele	Difference	Quality	Score	Allele Comments	Sample Comments

Sample 26: Run date and time: 09/15/2020 - 22:42:28 -> 09/15/2020 - 23:28:27

Dye: Blue - 0 peaks - 32.fsa

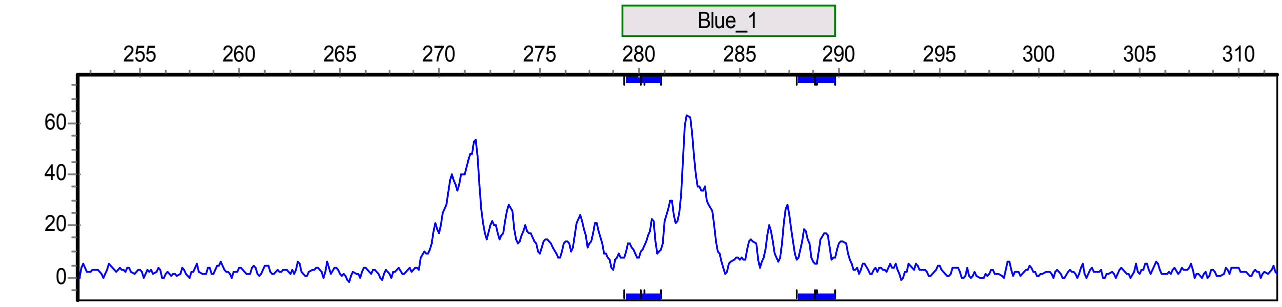

No	Size	Height	Area	Marker	Allele	Difference	Quality	Score	Allele Comments	Sample Comments

Sample 27: Run date and time: 09/15/2020 - 22:42:28 -> 09/15/2020 - 23:28:27

Dye: Blue - 2 peaks - 33.fsa

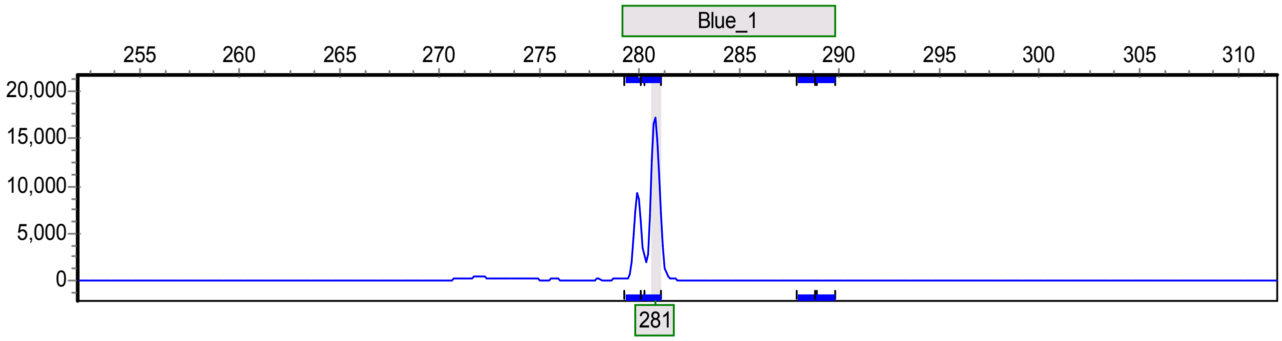

No	Size	Height	Area	Marker	Allele	Difference	Quality	Score	Allele Comments	Sample Comments
1	279.9	9219	42782	Blue_1	280	0.1	Pass	500.0	[<Deleted>]	
2	280.8	17109	89629	Blue_1	281	0.2	Pass	500.0	[<SAT (Repaired)><Confirmed>]	

Allele Report

Sample 28: Run date and time: 09/15/2020 - 22:42:28 -> 09/15/2020 - 23:28:27

Dye: Blue - 2 peaks - 34.fsa

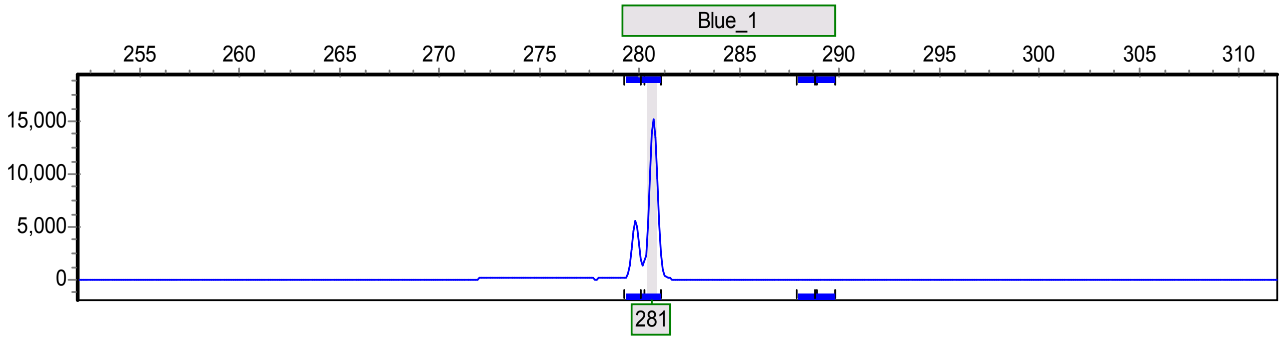

No	Size	Height	Area	Marker	Allele	Difference	Quality	Score	Allele Comments	Sample Comments
1	279.8	5476	24378	Blue_1	280	0.0	Pass	500.0	[<Deleted>]	
2	280.7	15206	73445	Blue_1	281	0.1	Pass	500.0		

Sample 29: Run date and time: 09/15/2020 - 22:42:28 -> 09/15/2020 - 23:28:27

Dye: Blue - 2 peaks - 35.fsa

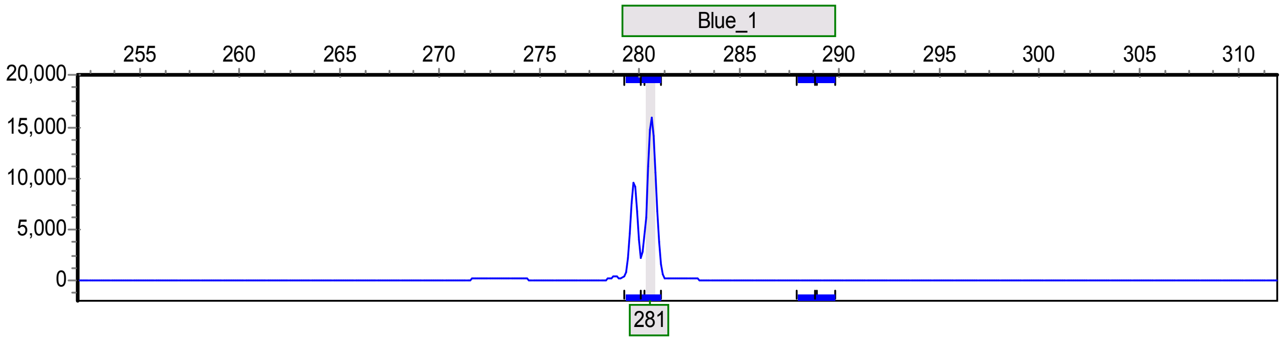

No	Size	Height	Area	Marker	Allele	Difference	Quality	Score	Allele Comments	Sample Comments
1	279.7	9457	45328	Blue_1	280	0.1	Pass	500.0	[<Deleted>]	
2	280.6	15802	83181	Blue_1	281	0.0	Pass	500.0		

Sample 30: Run date and time: 09/15/2020 - 22:42:28 -> 09/15/2020 - 23:28:27

Dye: Blue - 2 peaks - 36.fsa

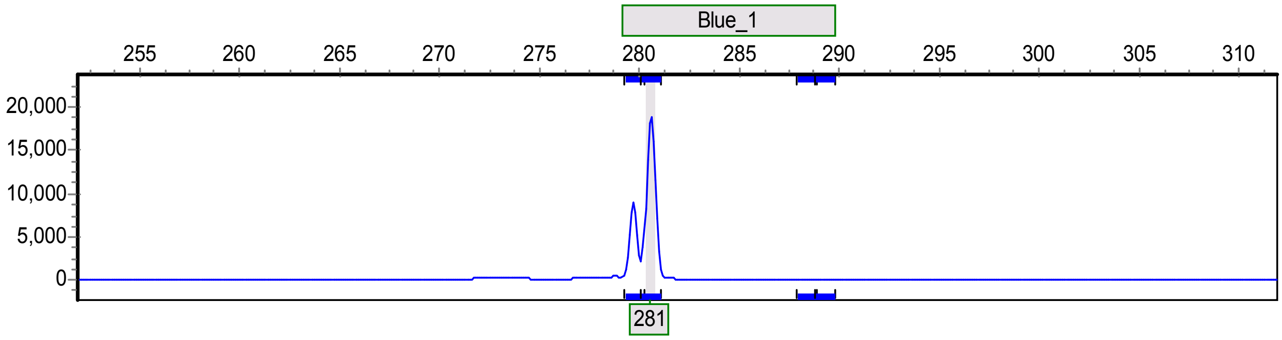

No	Size	Height	Area	Marker	Allele	Difference	Quality	Score	Allele Comments	Sample Comments
1	279.7	8946	39929	Blue_1	280	0.1	Pass	500.0	[<Deleted>]	
2	280.6	18742	96813	Blue_1	281	0.0	Pass	500.0	[<SAT (Repaired)><Confirmed>]	

Allele Report

Sample 31: Run date and time: 09/15/2020 - 22:42:28 -> 09/15/2020 - 23:28:27

Dye: Blue - 2 peaks - 37.fsa

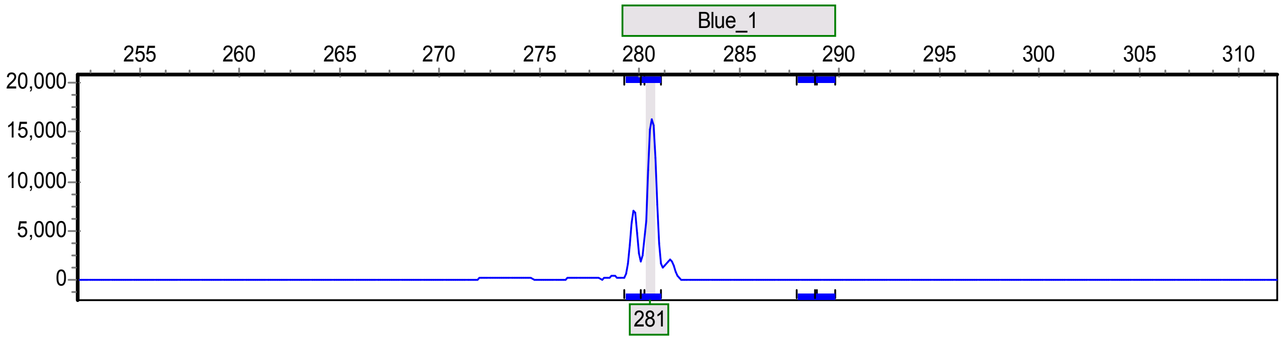

No	Size	Height	Area	Marker	Allele	Difference	Quality	Score	Allele Comments	Sample Comments
1	279.7	7115	34099	Blue_1	280	0.1	Pass	500.0	[<Deleted>]	
2	280.6	16428	88009	Blue_1	281	0.0	Pass	500.0		

Sample 32: Run date and time: 09/15/2020 - 22:42:28 -> 09/15/2020 - 23:28:27

Dye: Blue - 2 peaks - 38.fsa

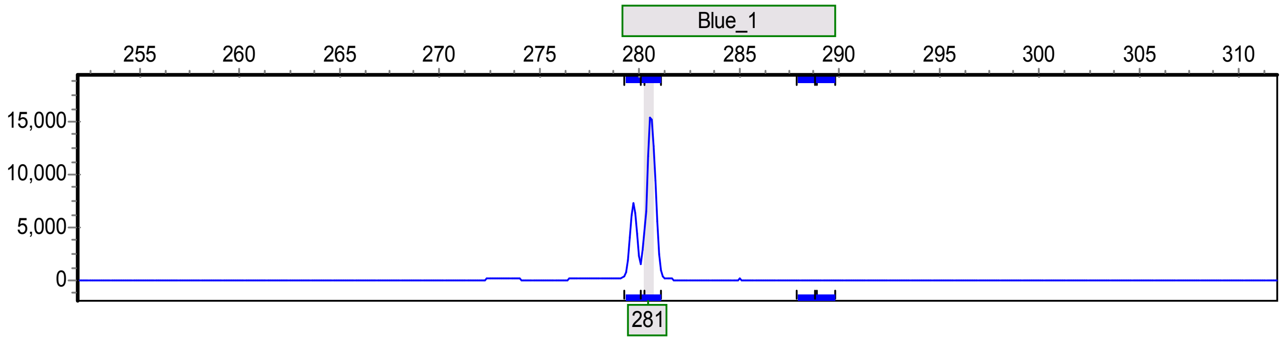

No	Size	Height	Area	Marker	Allele	Difference	Quality	Score	Allele Comments	Sample Comments
1	279.7	7282	32401	Blue_1	280	0.1	Pass	500.0	[<Deleted>]	
2	280.5	15335	78537	Blue_1	281	0.1	Pass	500.0		

Sample 33: Run date and time: 09/15/2020 - 22:42:28 -> 09/15/2020 - 23:28:27

Dye: Blue - 2 peaks - 39.fsa

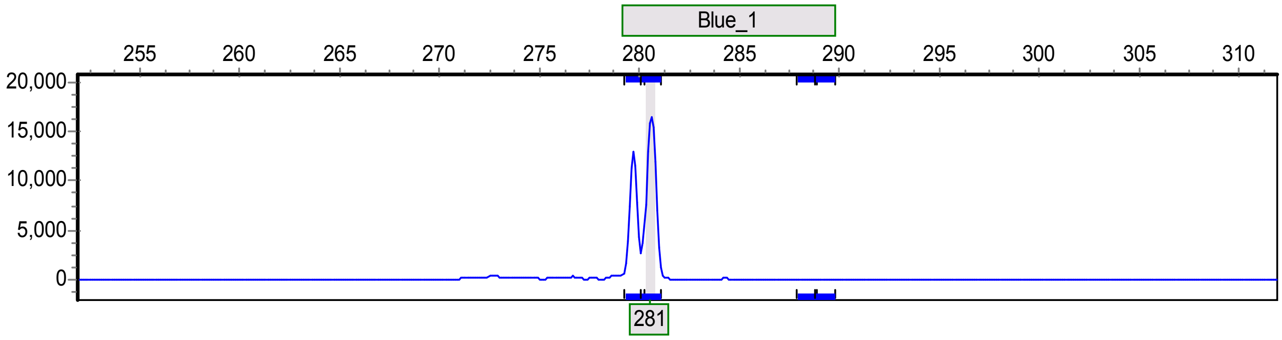

No	Size	Height	Area	Marker	Allele	Difference	Quality	Score	Allele Comments	Sample Comments
1	279.7	12955	60235	Blue_1	280	0.1	Pass	500.0	[<Deleted>]	
2	280.6	16353	92086	Blue_1	281	0.0	Pass	500.0		

Sample 34: Run date and time: 09/15/2020 - 22:42:28 -> 09/15/2020 - 23:28:27

Dye: Blue - 2 peaks - 4.fsa

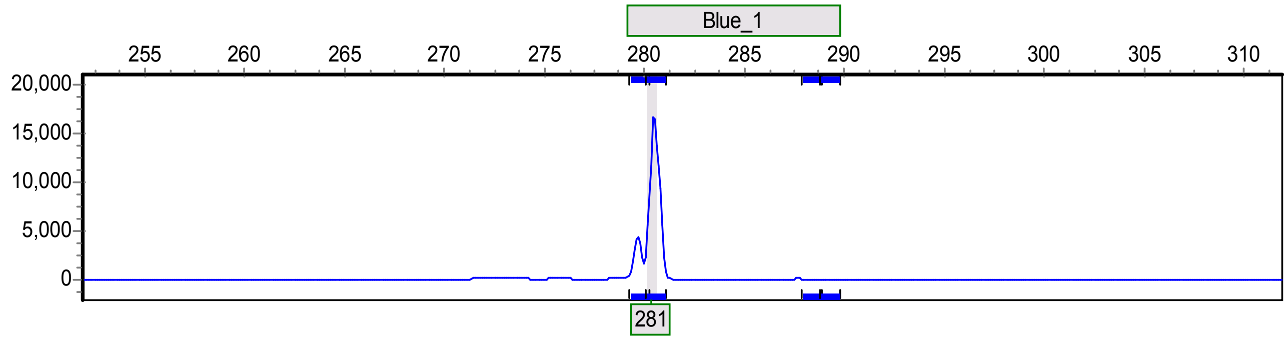

No	Size	Height	Area	Marker	Allele	Difference	Quality	Score	Allele Comments	Sample Comments
1	279.7	4486	22127	Blue_1	280	0.1	Pass	500.0	[<Deleted>]	
2	280.4	16599	91736	Blue_1	281	0.2	Pass	500.0	[<SAT (Repaired)><Confirmed>]	

Sample 35: Run date and time: 09/15/2020 - 22:42:28 -> 09/15/2020 - 23:28:27

Dye: Blue - 2 peaks - 40.fsa

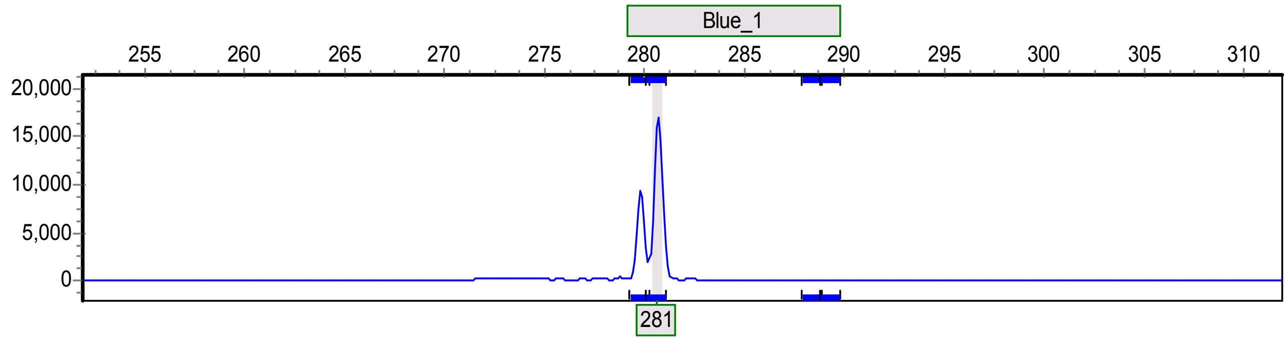

No	Size	Height	Area	Marker	Allele	Difference	Quality	Score	Allele Comments	Sample Comments
1	279.8	9305	43365	Blue_1	280	0.0	Pass	500.0	[<Deleted>]	
2	280.7	16892	85941	Blue_1	281	0.1	Pass	500.0		

Sample 36: Run date and time: 09/15/2020 - 22:42:28 -> 09/15/2020 - 23:28:27

Dye: Blue - 2 peaks - 41.fsa

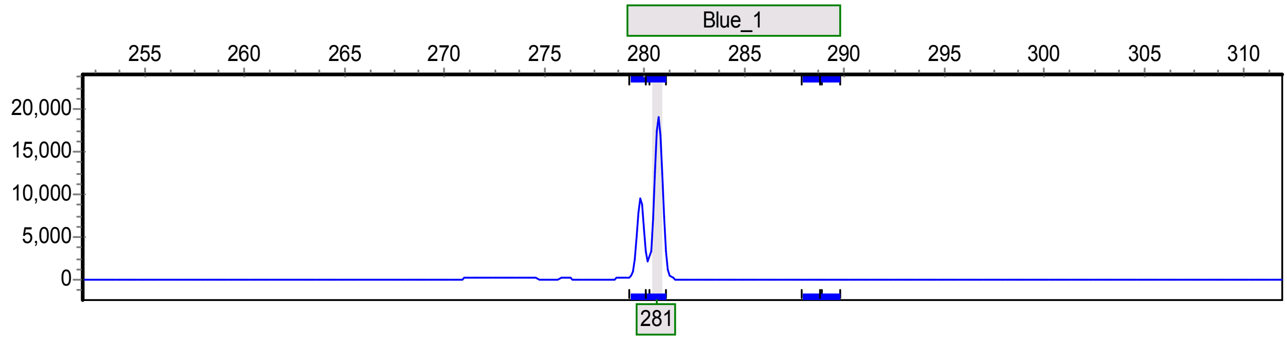

No	Size	Height	Area	Marker	Allele	Difference	Quality	Score	Allele Comments	Sample Comments
1	279.8	9621	45191	Blue_1	280	0.0	Pass	500.0	[<Deleted>]	
2	280.7	18958	95197	Blue_1	281	0.1	Pass	500.0		

Allele Report

Sample 37: Run date and time: 09/15/2020 - 22:42:28 -> 09/15/2020 - 23:28:27

Dye: Blue - 2 peaks - 42.fsa

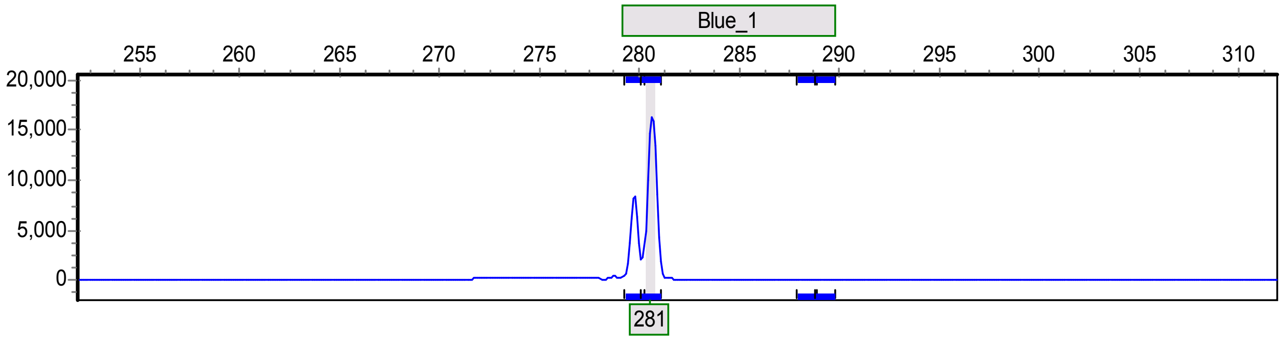

No	Size	Height	Area	Marker	Allele	Difference	Quality	Score	Allele Comments	Sample Comments
1	279.8	8329	39263	Blue_1	280	0.0	Pass	500.0	[<Deleted>]	
2	280.6	16220	87771	Blue_1	281	0.0	Pass	500.0		

Sample 38: Run date and time: 09/15/2020 - 22:42:28 -> 09/15/2020 - 23:28:27

Dye: Blue - 2 peaks - 43.fsa

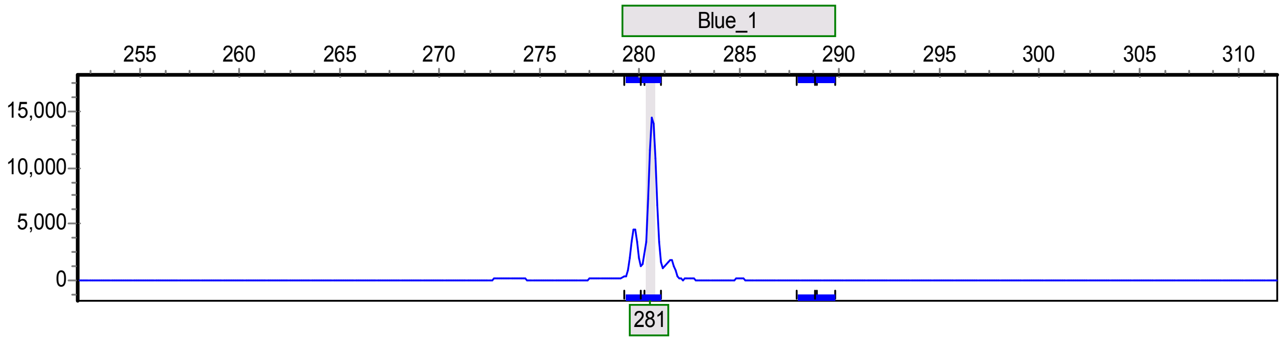

No	Size	Height	Area	Marker	Allele	Difference	Quality	Score	Allele Comments	Sample Comments
1	279.8	4499	21820	Blue_1	280	0.0	Pass	500.0	[<Deleted>]	
2	280.6	14345	70170	Blue_1	281	0.0	Pass	500.0		

Sample 39: Run date and time: 09/15/2020 - 22:42:28 -> 09/15/2020 - 23:28:27

Dye: Blue - 2 peaks - 44.fsa

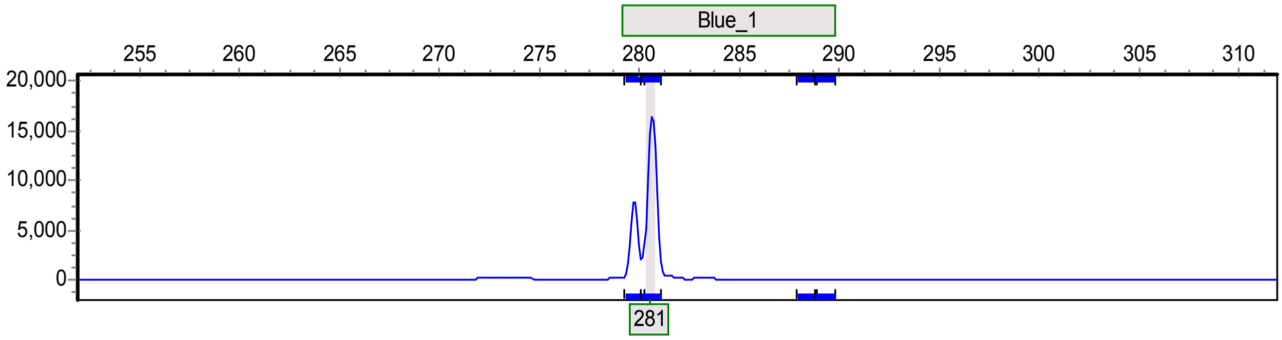

No	Size	Height	Area	Marker	Allele	Difference	Quality	Score	Allele Comments	Sample Comments
1	279.8	7873	37432	Blue_1	280	0.0	Pass	500.0	[<Deleted>]	
2	280.6	16270	88465	Blue_1	281	0.0	Pass	500.0		

Allele Report

Sample 40: Run date and time: 09/15/2020 - 22:42:28 -> 09/15/2020 - 23:28:27

Dye: Blue - 2 peaks - 45.fsa

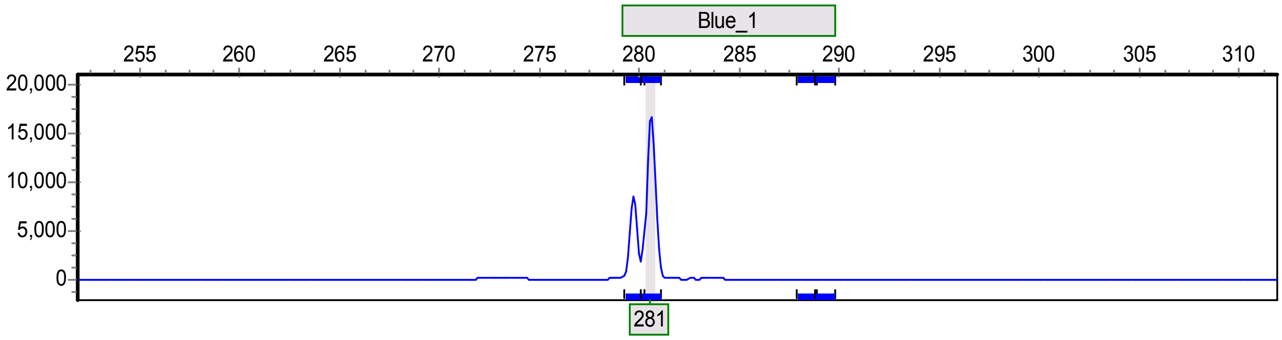

No	Size	Height	Area	Marker	Allele	Difference	Quality	Score	Allele Comments	Sample Comments
1	279.7	8659	38153	Blue_1	280	0.1	Pass	500.0	[<Deleted>]	
2	280.6	16601	85204	Blue_1	281	0.0	Pass	500.0		

Sample 41: Run date and time: 09/15/2020 - 22:42:28 -> 09/15/2020 - 23:28:27

Dye: Blue - 1 peaks - 46.fsa

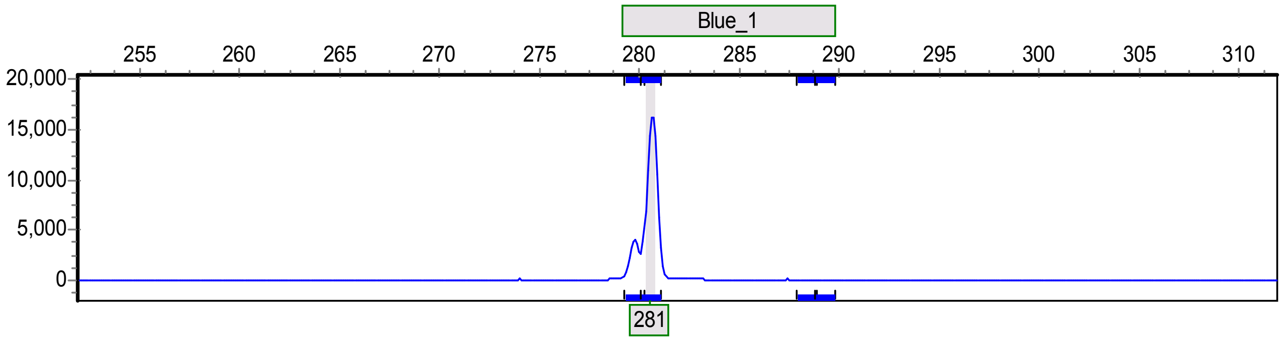

No	Size	Height	Area	Marker	Allele	Difference	Quality	Score	Allele Comments	Sample Comments
1	280.6	16088	100636	Blue_1	281	0.0	Pass	500.0		

Sample 42: Run date and time: 09/15/2020 - 22:42:28 -> 09/15/2020 - 23:28:27

Dye: Blue - 2 peaks - 47.fsa

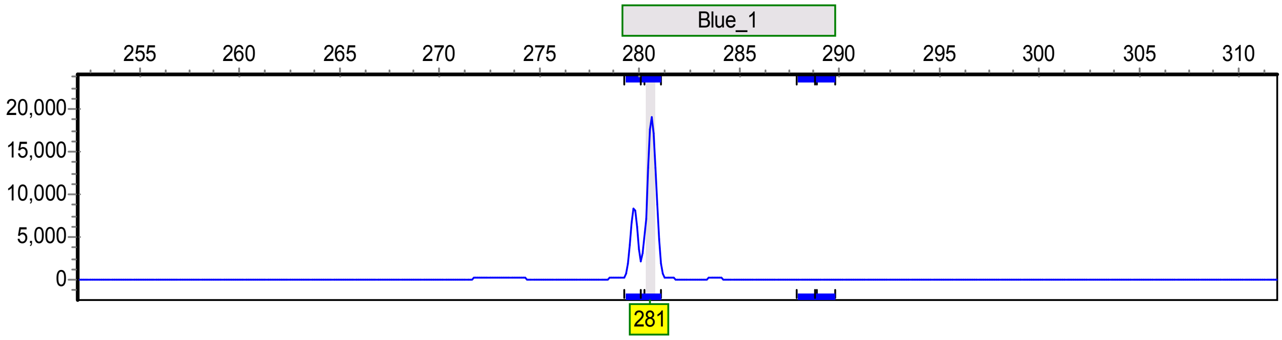

No	Size	Height	Area	Marker	Allele	Difference	Quality	Score	Allele Comments	Sample Comments
1	279.7	8433	40492	Blue_1	280	0.1	Pass	500.0	[<Deleted>]	
2	280.6	18923	100501	Blue_1	281	0.0	Check	500.0	[<SAT (Repaired)>]	

Allele Report

Sample 43: Run date and time: 09/15/2020 - 22:42:28 -> 09/15/2020 - 23:28:27

Dye: Blue - 2 peaks - 48.fsa

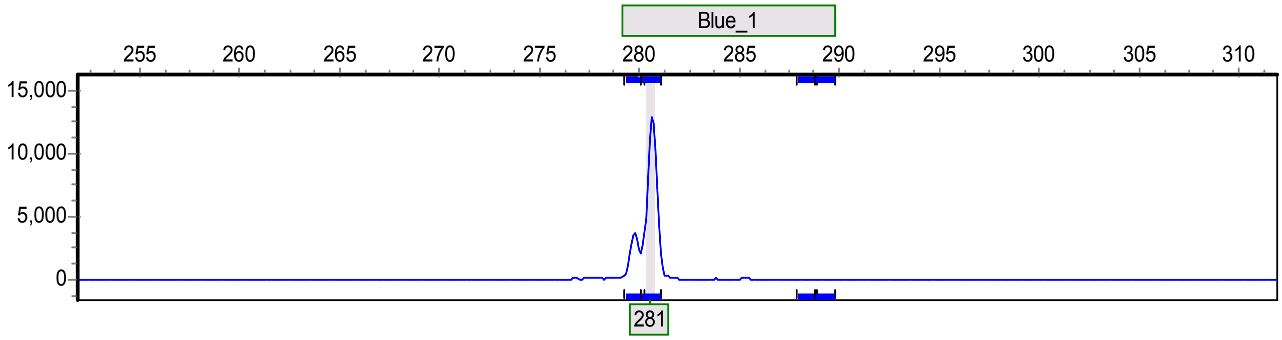

No	Size	Height	Area	Marker	Allele	Difference	Quality	Score	Allele Comments	Sample Comments
1	279.8	3710	21406	Blue_1	280	0.0	Pass	500.0	[<Deleted>]	
2	280.6	12808	73704	Blue_1	281	0.0	Pass	500.0		

Sample 44: Run date and time: 09/15/2020 - 22:42:28 -> 09/15/2020 - 23:28:27

Dye: Blue - 2 peaks - 49.fsa

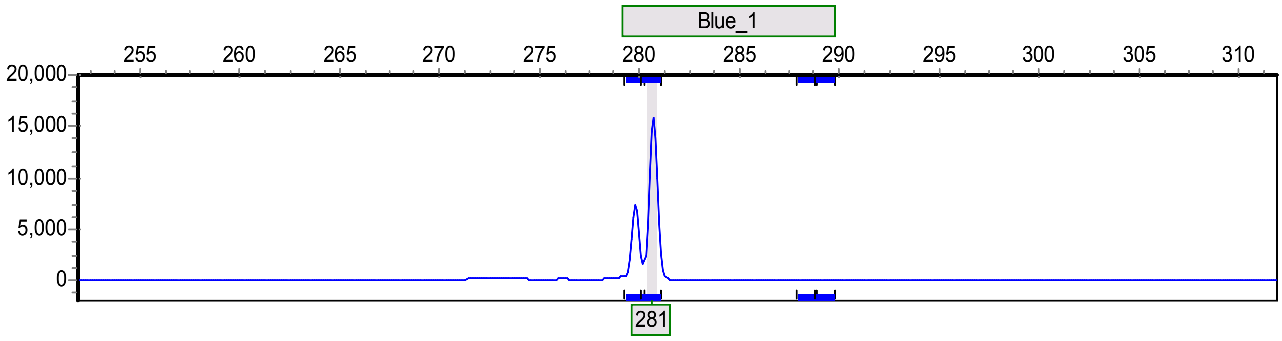

No	Size	Height	Area	Marker	Allele	Difference	Quality	Score	Allele Comments	Sample Comments
1	279.8	7430	34758	Blue_1	280	0.0	Pass	500.0	[<Deleted>]	
2	280.7	15775	76949	Blue_1	281	0.1	Pass	500.0		

Sample 45: Run date and time: 09/15/2020 - 22:42:28 -> 09/15/2020 - 23:28:27

Dye: Blue - 2 peaks - 5.fsa

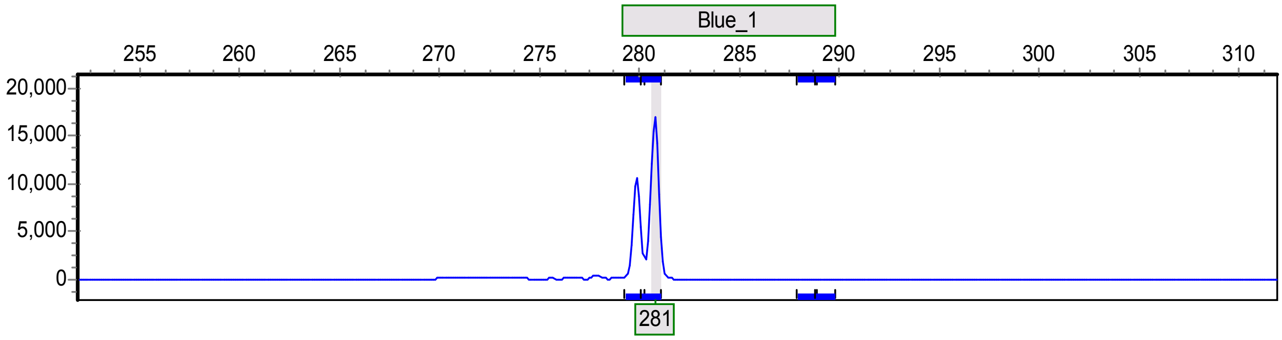

No	Size	Height	Area	Marker	Allele	Difference	Quality	Score	Allele Comments	Sample Comments
1	279.9	10638	49178	Blue_1	280	0.1	Pass	500.0	[<Deleted>]	
2	280.8	16873	82599	Blue_1	281	0.2	Pass	500.0		

Sample 46: Run date and time: 09/15/2020 - 22:42:28 -> 09/15/2020 - 23:28:27

Dye: Blue - 1 peaks - 50.fsa

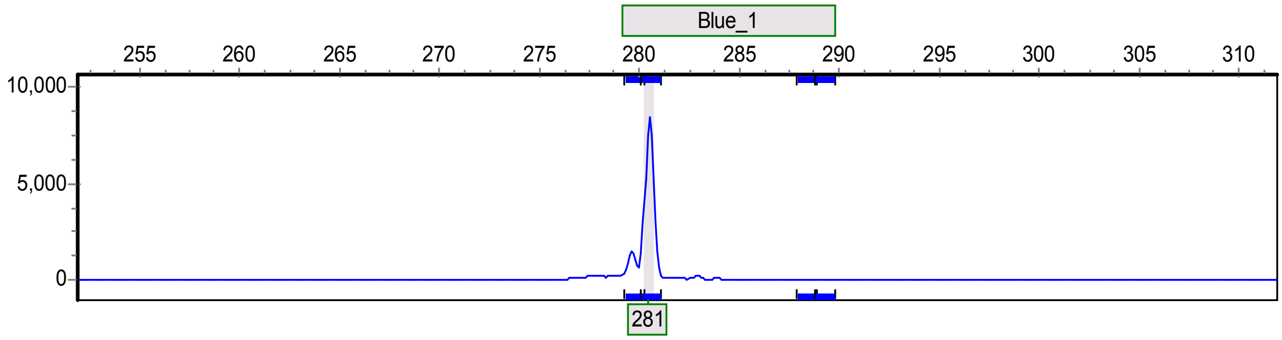

No	Size	Height	Area	Marker	Allele	Difference	Quality	Score	Allele Comments	Sample Comments
1	280.5	8387	40891	Blue_1	281	0.1	Pass	500.0		

Sample 47: Run date and time: 09/15/2020 - 22:42:28 -> 09/15/2020 - 23:28:27

Dye: Blue - 2 peaks - 51.fsa

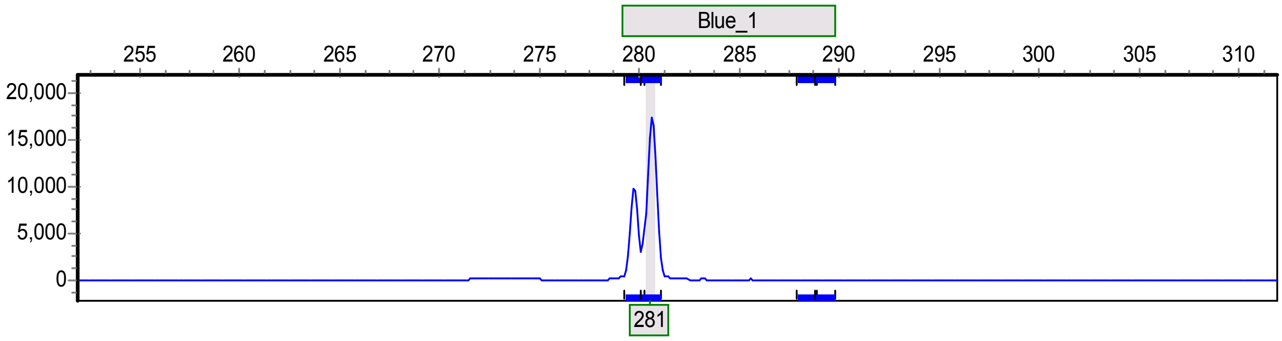

No	Size	Height	Area	Marker	Allele	Difference	Quality	Score	Allele Comments	Sample Comments
1	279.7	9699	50061	Blue_1	280	0.1	Pass	500.0	[<Deleted>]	
2	280.6	17242	99455	Blue_1	281	0.0	Pass	500.0		

Sample 48: Run date and time: 09/15/2020 - 22:42:28 -> 09/15/2020 - 23:28:27

Dye: Blue - 2 peaks - 52.fsa

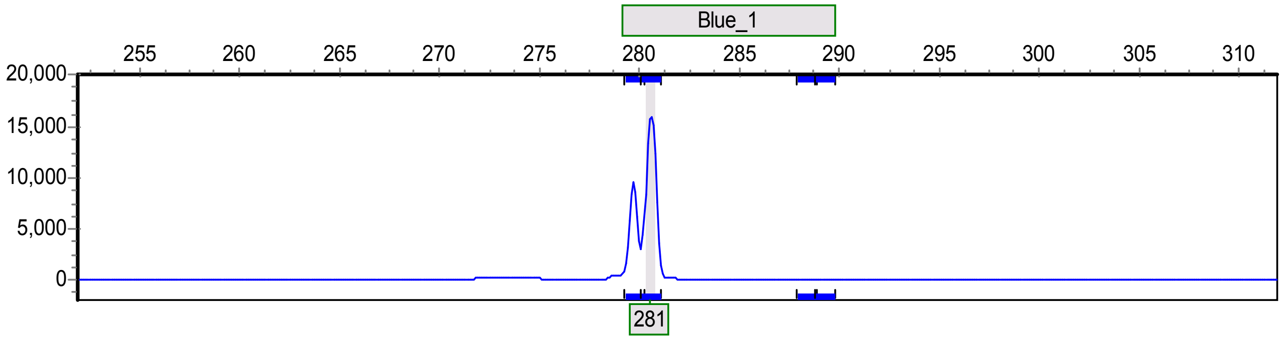

No	Size	Height	Area	Marker	Allele	Difference	Quality	Score	Allele Comments	Sample Comments
1	279.7	9555	45481	Blue_1	280	0.1	Pass	500.0	[<Deleted>]	
2	280.6	15814	96136	Blue_1	281	0.0	Pass	500.0		

Sample 49: Run date and time: 09/15/2020 - 22:42:28 -> 09/15/2020 - 23:28:27

Dye: Blue - 1 peaks - 53.fsa

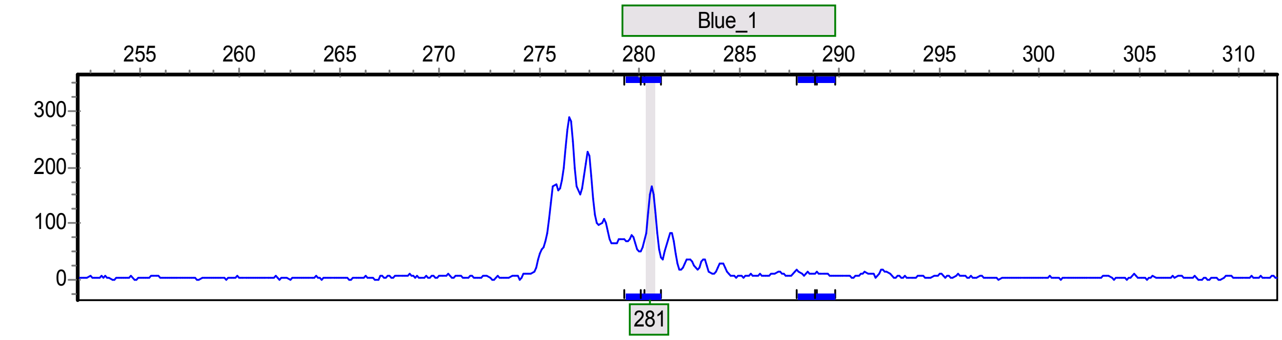

No	Size	Height	Area	Marker	Allele	Difference	Quality	Score	Allele Comments	Sample Comments
1	280.6	165	968	Blue_1	281	0.0	Pass	8.0		

Sample 50: Run date and time: 09/15/2020 - 22:42:28 -> 09/15/2020 - 23:28:27

Dye: Blue - 1 peaks - 54.fsa

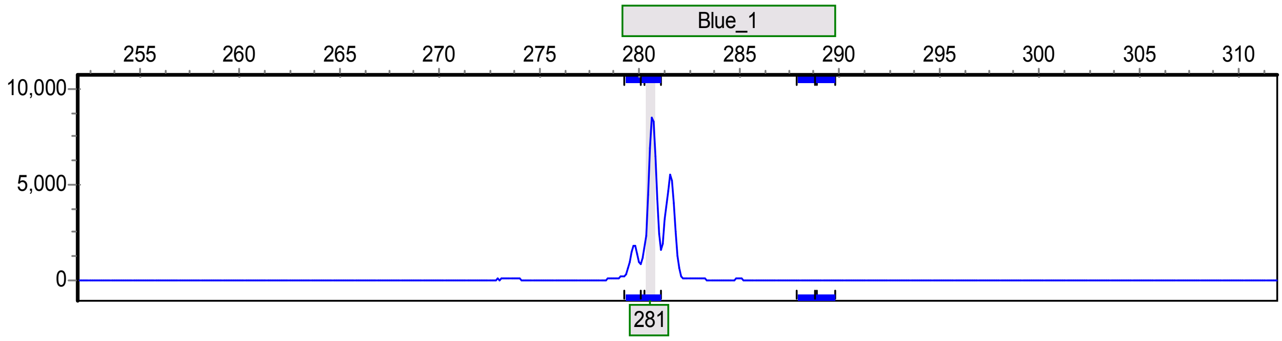

No	Size	Height	Area	Marker	Allele	Difference	Quality	Score	Allele Comments	Sample Comments
1	280.6	8466	44147	Blue_1	281	0.0	Pass	500.0		

Sample 51: Run date and time: 09/15/2020 - 22:42:28 -> 09/15/2020 - 23:28:27

Dye: Blue - 1 peaks - 55.fsa

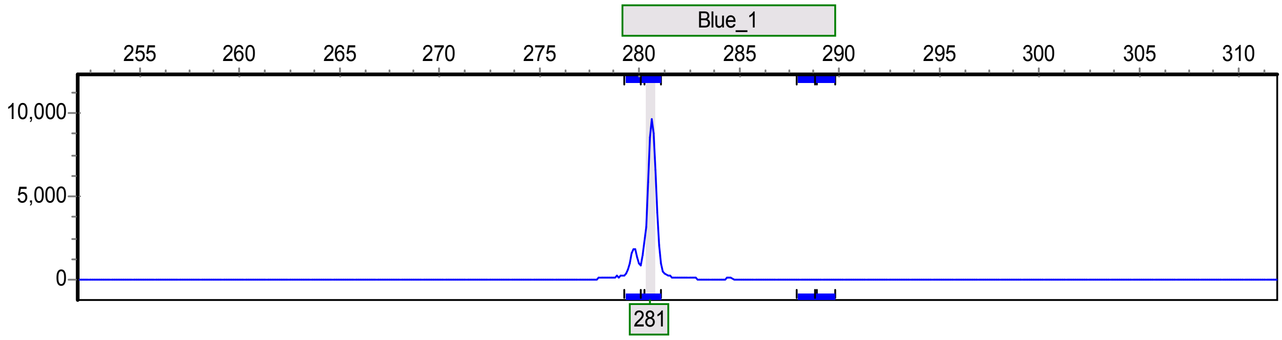

No	Size	Height	Area	Marker	Allele	Difference	Quality	Score	Allele Comments	Sample Comments
1	280.6	9690	48162	Blue_1	281	0.0	Pass	500.0		

Sample 52: Run date and time: 09/15/2020 - 22:42:28 -> 09/15/2020 - 23:28:27

Dye: Blue - 1 peaks - 56.fsa

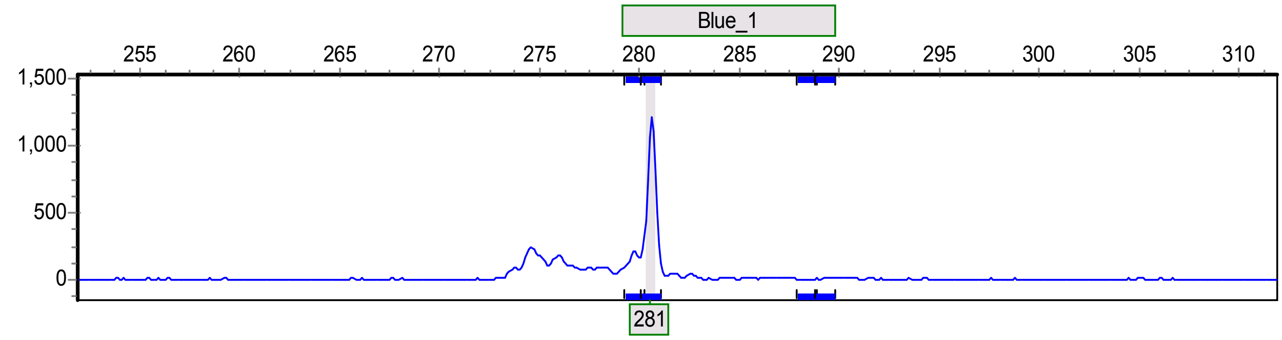

No	Size	Height	Area	Marker	Allele	Difference	Quality	Score	Allele Comments	Sample Comments
1	280.6	1201	6126	Blue_1	281	0.0	Pass	263.5		

Sample 53: Run date and time: 09/15/2020 - 22:42:28 -> 09/15/2020 - 23:28:27

Dye: Blue - 1 peaks - 57.fsa

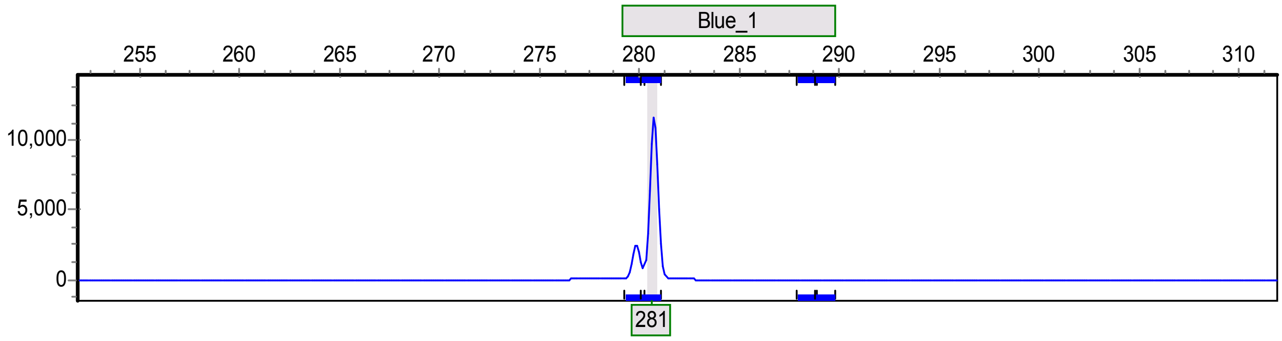

No	Size	Height	Area	Marker	Allele	Difference	Quality	Score	Allele Comments	Sample Comments
1	280.7	11447	56197	Blue_1	281	0.1	Pass	500.0		

Sample 54: Run date and time: 09/15/2020 - 22:42:28 -> 09/15/2020 - 23:28:27

Dye: Blue - 1 peaks - 58.fsa

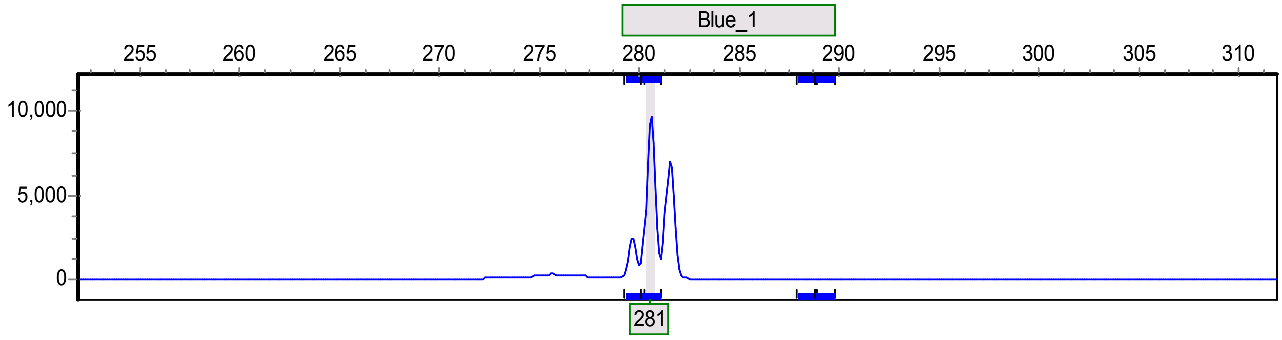

No	Size	Height	Area	Marker	Allele	Difference	Quality	Score	Allele Comments	Sample Comments
1	280.6	9634	48037	Blue_1	281	0.0	Pass	500.0		

Allele Report

Sample 55: Run date and time: 09/15/2020 - 22:42:28 -> 09/15/2020 - 23:28:27

Dye: Blue - 1 peaks - 59.fsa

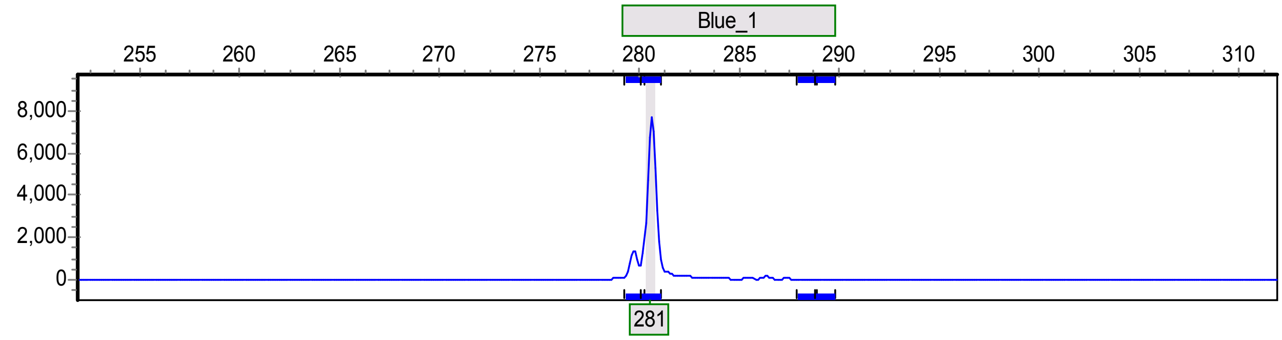

No	Size	Height	Area	Marker	Allele	Difference	Quality	Score	Allele Comments	Sample Comments
1	280.6	7656	39213	Blue_1	281	0.0	Pass	500.0		

Sample 56: Run date and time: 09/15/2020 - 22:42:28 -> 09/15/2020 - 23:28:27

Dye: Blue - 2 peaks - 6.fsa

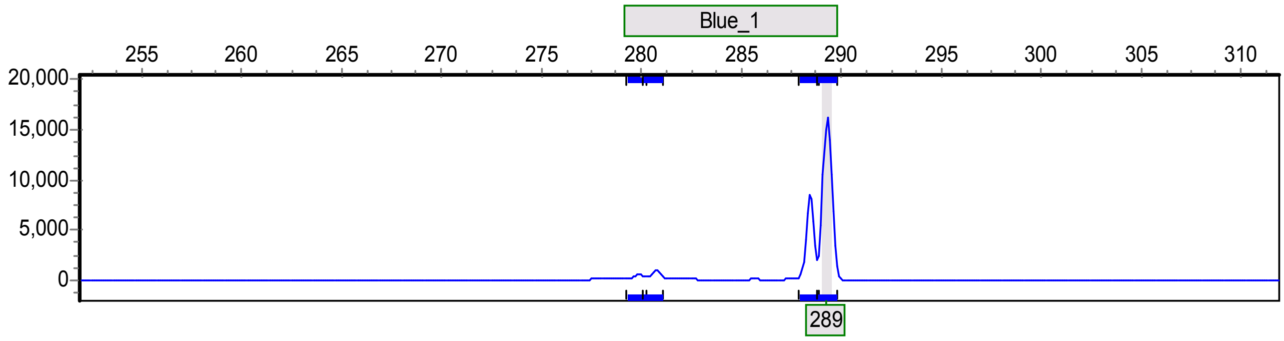

No	Size	Height	Area	Marker	Allele	Difference	Quality	Score	Allele Comments	Sample Comments
1	288.4	8563	39937	Blue_1	288	0.0	Pass	500.0	[<Deleted>]	
2	289.3	16127	81390	Blue_1	289	0.0	Pass	500.0		

Sample 57: Run date and time: 09/15/2020 - 22:42:28 -> 09/15/2020 - 23:28:27

Dye: Blue - 2 peaks - 60.fsa

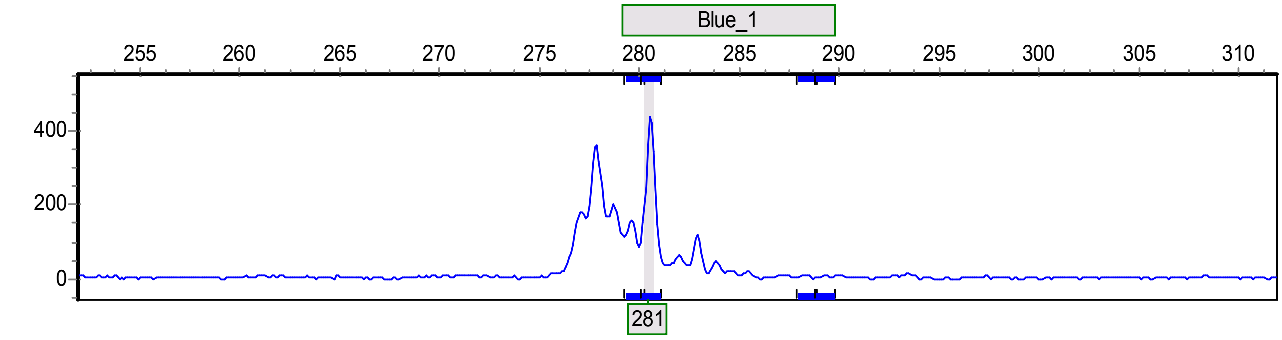

No	Size	Height	Area	Marker	Allele	Difference	Quality	Score	Allele Comments	Sample Comments
1	279.6	160	934	Blue_1	280	0.2	Check	2.7	[<Deleted>]	
2	280.5	439	2391	Blue_1	281	0.1	Pass	49.6		

Sample 58: Run date and time: 09/15/2020 - 22:42:28 -> 09/15/2020 - 23:28:27

Dye: Blue - 1 peaks - 61.fsa

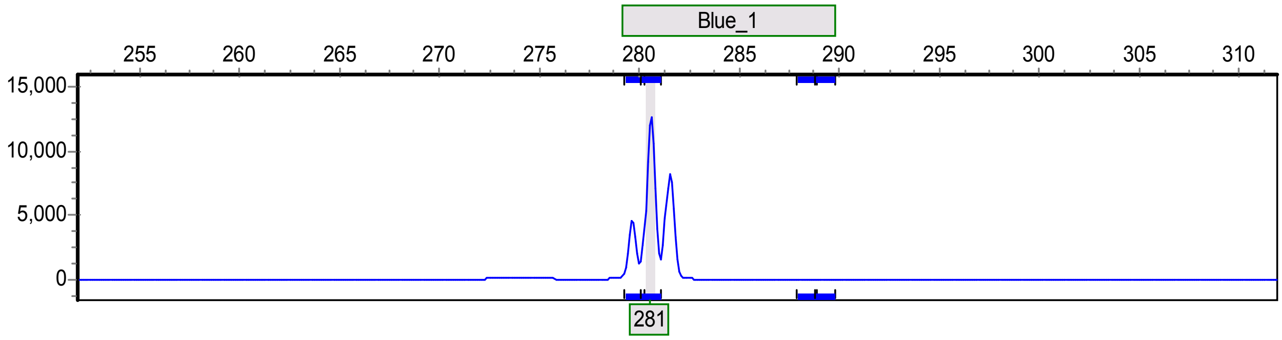

No	Size	Height	Area	Marker	Allele	Difference	Quality	Score	Allele Comments	Sample Comments
1	280.6	12541	62731	Blue_1	281	0.0	Pass	500.0		

Sample 59: Run date and time: 09/15/2020 - 22:42:28 -> 09/15/2020 - 23:28:27

Dye: Blue - 1 peaks - 62.fsa

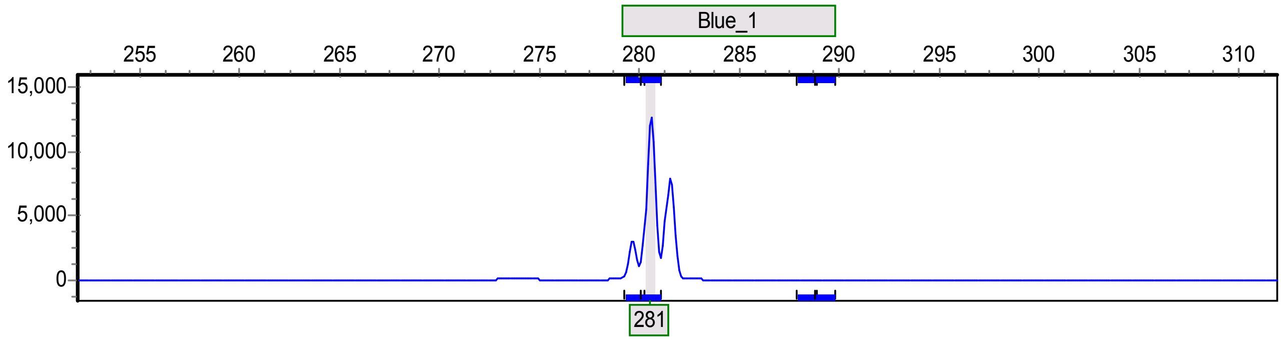

No	Size	Height	Area	Marker	Allele	Difference	Quality	Score	Allele Comments	Sample Comments
1	280.6	12584	64428	Blue_1	281	0.0	Pass	500.0		

Sample 60: Run date and time: 09/15/2020 - 22:42:28 -> 09/15/2020 - 23:28:27

Dye: Blue - 2 peaks - 63.fsa

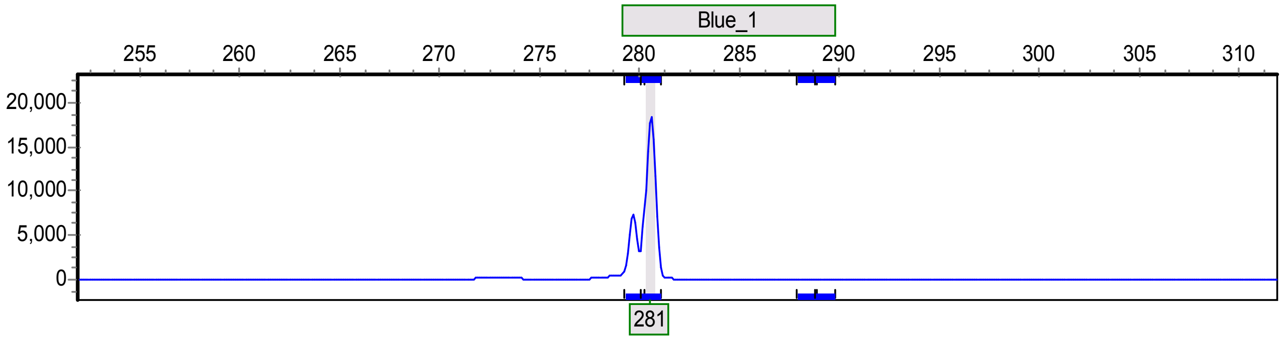

No	Size	Height	Area	Marker	Allele	Difference	Quality	Score	Allele Comments	Sample Comments
1	279.7	7384	37806	Blue_1	280	0.1	Pass	500.0	[<Deleted>]	
2	280.6	18246	103920	Blue_1	281	0.0	Pass	500.0		

Sample 61: Run date and time: 09/15/2020 - 22:42:28 -> 09/15/2020 - 23:28:27

Dye: Blue - 2 peaks - 64.fsa

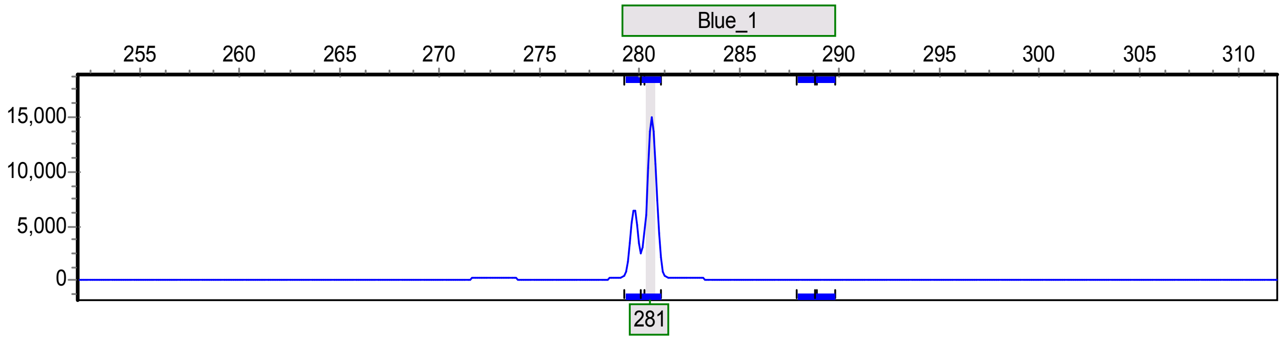

No	Size	Height	Area	Marker	Allele	Difference	Quality	Score	Allele Comments	Sample Comments
1	279.7	6508	34242	Blue_1	280	0.1	Pass	500.0	[<Deleted>]	
2	280.6	14996	82940	Blue_1	281	0.0	Pass	500.0		

Sample 62: Run date and time: 09/15/2020 - 22:42:28 -> 09/15/2020 - 23:28:27

Dye: Blue - 1 peaks - 65.fsa

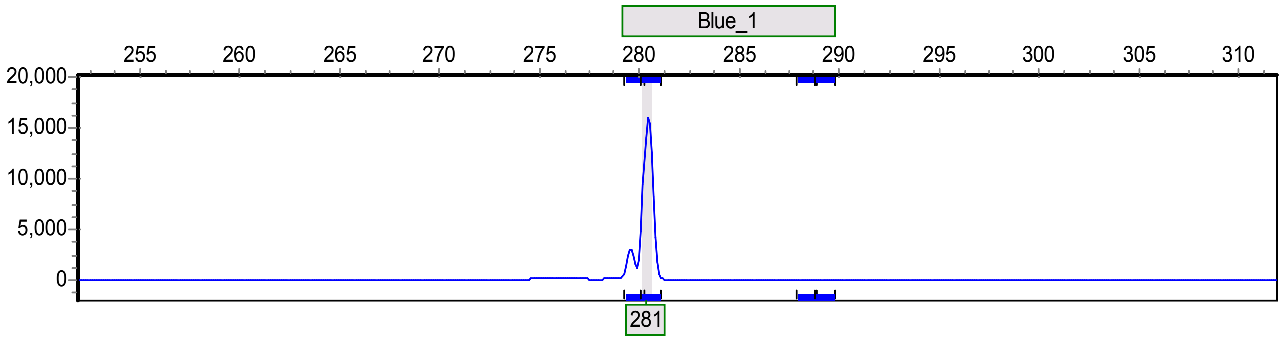

No	Size	Height	Area	Marker	Allele	Difference	Quality	Score	Allele Comments	Sample Comments
1	280.4	15943	84422	Blue_1	281	0.2	Pass	500.0		

Sample 63: Run date and time: 09/15/2020 - 22:42:28 -> 09/15/2020 - 23:28:27

Dye: Blue - 1 peaks - 66.fsa

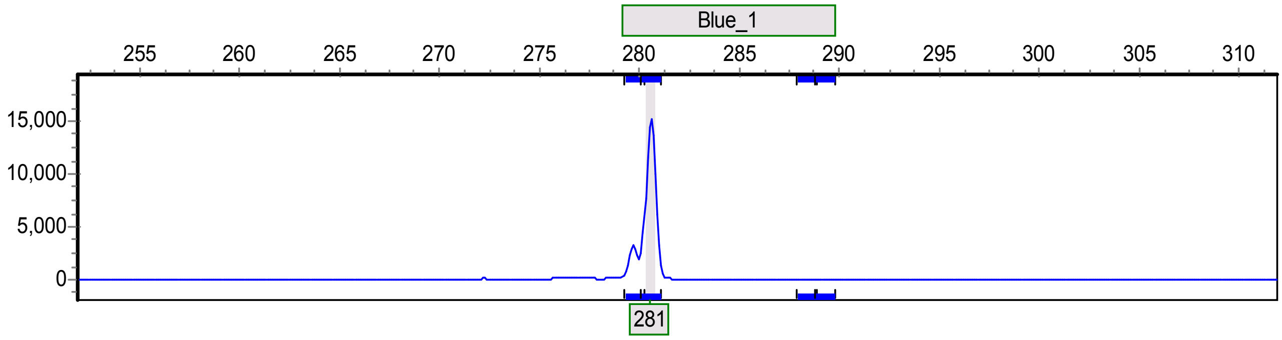

No	Size	Height	Area	Marker	Allele	Difference	Quality	Score	Allele Comments	Sample Comments
1	280.6	15375	86045	Blue_1	281	0.0	Pass	500.0		

Sample 64: Run date and time: 09/15/2020 - 22:42:28 -> 09/15/2020 - 23:28:27

Dye: Blue - 1 peaks - 67.fsa

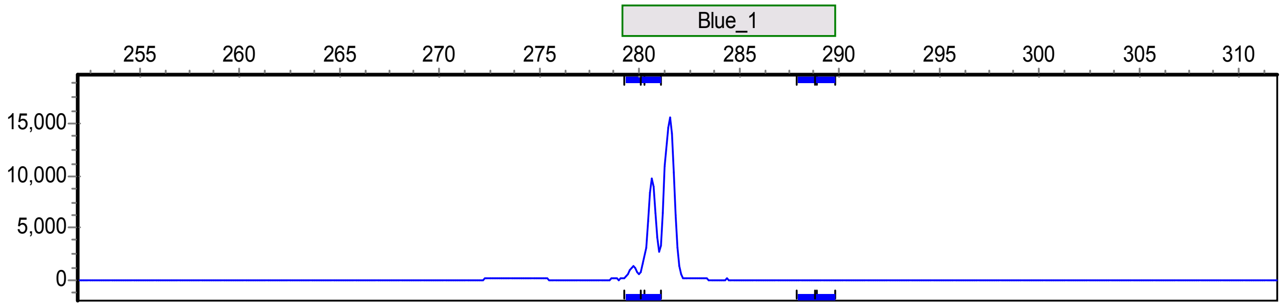

No	Size	Height	Area	Marker	Allele	Difference	Quality	Score	Allele Comments	Sample Comments
1	280.6	9681	49156	Blue_1	281	0.0	Pass	500.0	[<Deleted>]	

Sample 65: Run date and time: 09/15/2020 - 22:42:28 -> 09/15/2020 - 23:28:27

Dye: Blue - 1 peaks - 68.fsa

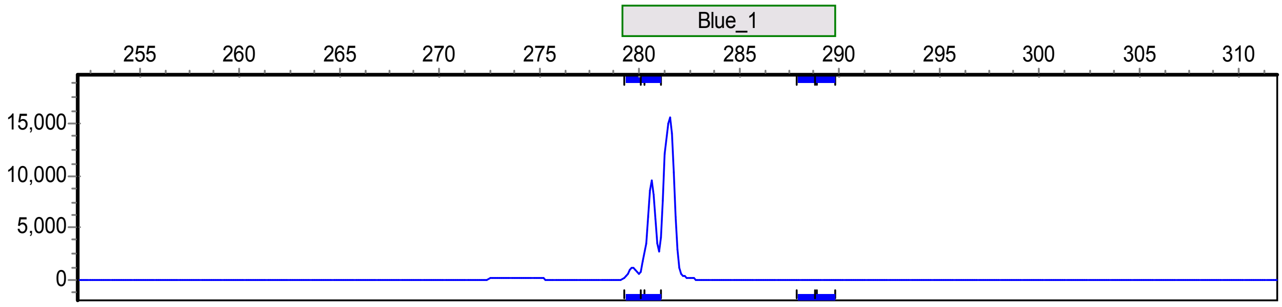

No	Size	Height	Area	Marker	Allele	Difference	Quality	Score	Allele Comments	Sample Comments
1	280.6	9535	45770	Blue_1	281	0.0	Pass	500.0	[<Deleted>]	

Sample 66: Run date and time: 09/15/2020 - 22:42:28 -> 09/15/2020 - 23:28:27

Dye: Blue - 1 peaks - 69.fsa

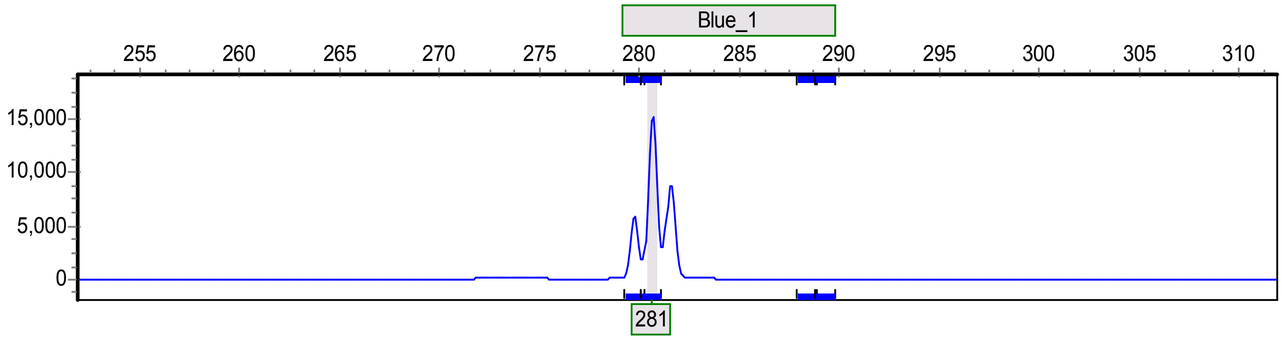

No	Size	Height	Area	Marker	Allele	Difference	Quality	Score	Allele Comments	Sample Comments
1	280.7	15007	79549	Blue_1	281	0.1	Pass	500.0		

Allele Report

Sample 67: Run date and time: 09/15/2020 - 22:42:28 -> 09/15/2020 - 23:28:27

Dye: Blue - 1 peaks - 7.fsa

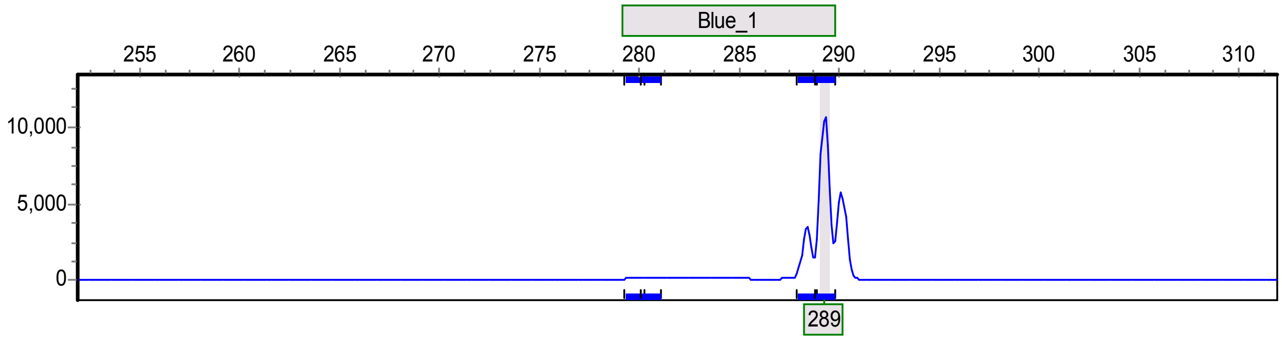

No	Size	Height	Area	Marker	Allele	Difference	Quality	Score	Allele Comments	Sample Comments
1	289.3	10631	55408	Blue_1	289	0.0	Pass	500.0		

Sample 68: Run date and time: 09/15/2020 - 22:42:28 -> 09/15/2020 - 23:28:27

Dye: Blue - 1 peaks - 70.fsa

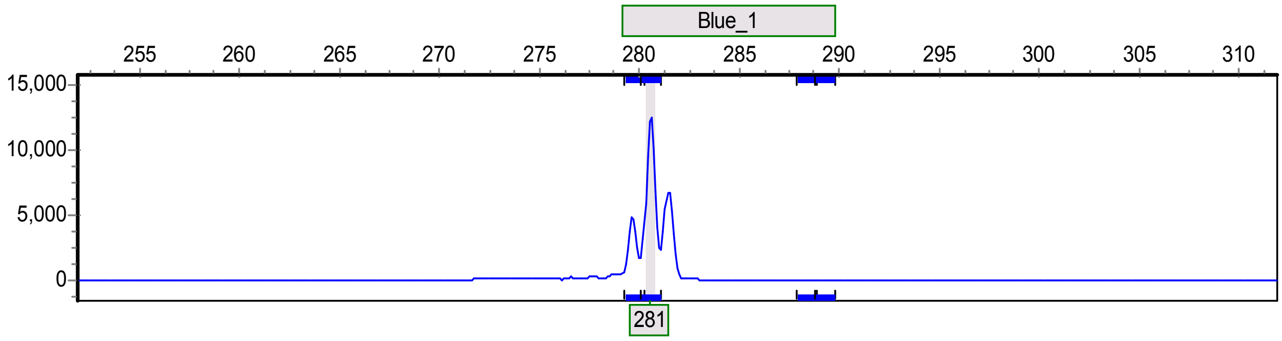

No	Size	Height	Area	Marker	Allele	Difference	Quality	Score	Allele Comments	Sample Comments
1	280.6	12412	65255	Blue_1	281	0.0	Pass	500.0		

Sample 69: Run date and time: 09/15/2020 - 22:42:28 -> 09/15/2020 - 23:28:27

Dye: Blue - 2 peaks - 71.fsa

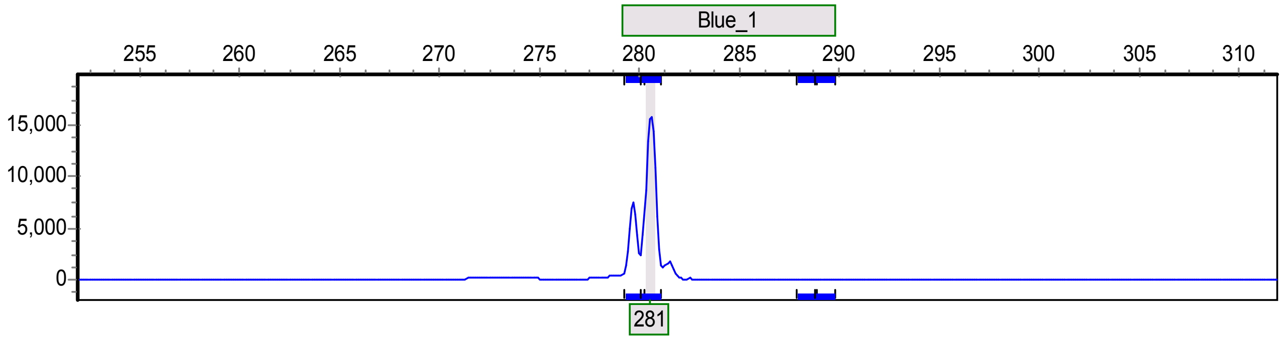

No	Size	Height	Area	Marker	Allele	Difference	Quality	Score	Allele Comments	Sample Comments
1	279.7	7479	35958	Blue_1	280	0.1	Pass	500.0	[<Deleted>]	
2	280.6	15728	90658	Blue_1	281	0.0	Pass	500.0		

Sample 70: Run date and time: 09/15/2020 - 22:42:28 -> 09/15/2020 - 23:28:27

Dye: Blue - 2 peaks - 72.fsa

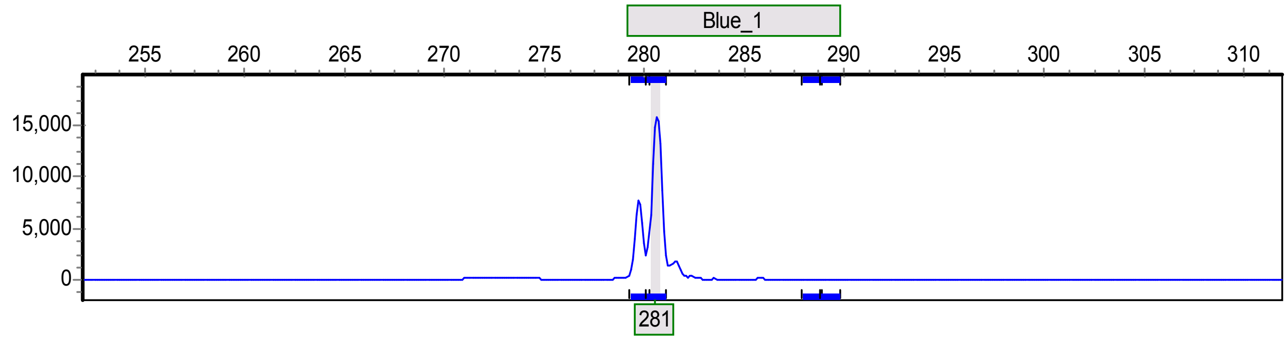

No	Size	Height	Area	Marker	Allele	Difference	Quality	Score	Allele Comments	Sample Comments
1	279.7	7587	38619	Blue_1	280	0.1	Pass	500.0	[<Deleted>]	
2	280.6	15622	91535	Blue_1	281	0.0	Pass	500.0		

Sample 71: Run date and time: 09/15/2020 - 22:42:28 -> 09/15/2020 - 23:28:27

Dye: Blue - 2 peaks - 73.fsa

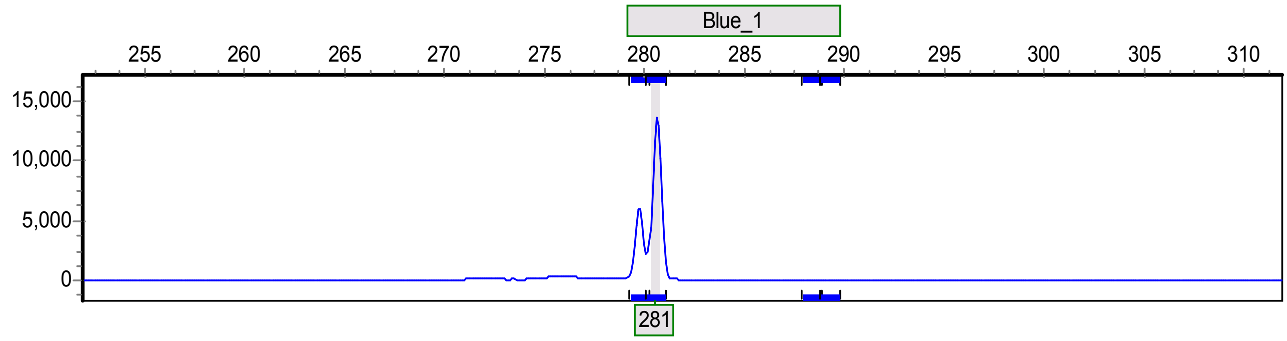

No	Size	Height	Area	Marker	Allele	Difference	Quality	Score	Allele Comments	Sample Comments
1	279.8	5957	30961	Blue_1	280	0.0	Pass	500.0	[<Deleted>]	
2	280.6	13540	70910	Blue_1	281	0.0	Pass	500.0		

Sample 72: Run date and time: 09/15/2020 - 22:42:28 -> 09/15/2020 - 23:28:27

Dye: Blue - 2 peaks - 74.fsa

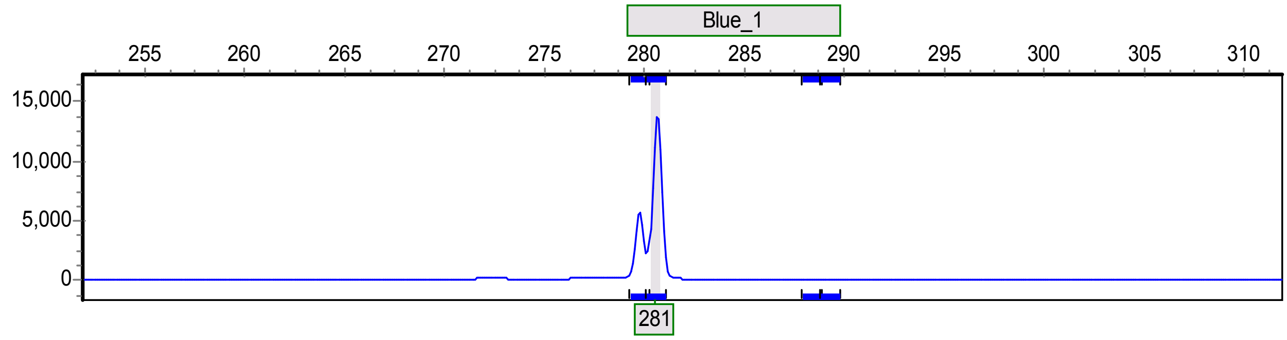

No	Size	Height	Area	Marker	Allele	Difference	Quality	Score	Allele Comments	Sample Comments
1	279.8	5663	29090	Blue_1	280	0.0	Pass	500.0	[<Deleted>]	
2	280.6	13593	73788	Blue_1	281	0.0	Pass	500.0		

Sample 73: Run date and time: 09/15/2020 - 22:42:28 -> 09/15/2020 - 23:28:27

Dye: Blue - 1 peaks - 75.fsa

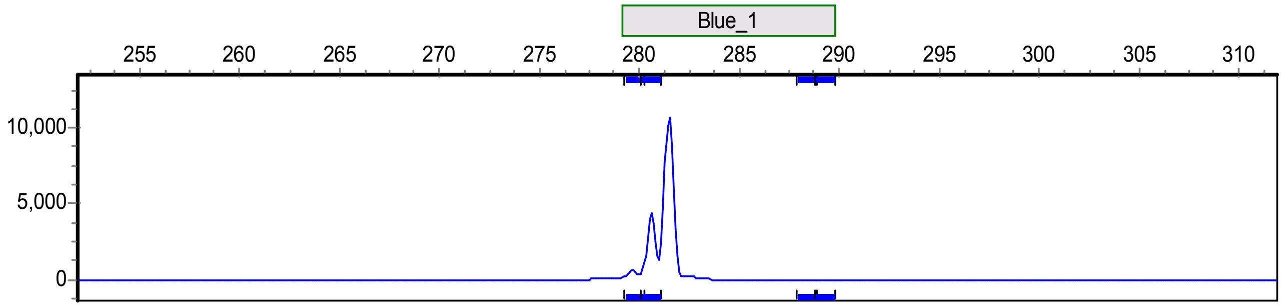

No	Size	Height	Area	Marker	Allele	Difference	Quality	Score	Allele Comments	Sample Comments
1	280.6	4351	20817	Blue_1	281	0.0	Pass	500.0	[<Deleted>]	

Sample 74: Run date and time: 09/15/2020 - 22:42:28 -> 09/15/2020 - 23:28:27

Dye: Blue - 1 peaks - 76.fsa

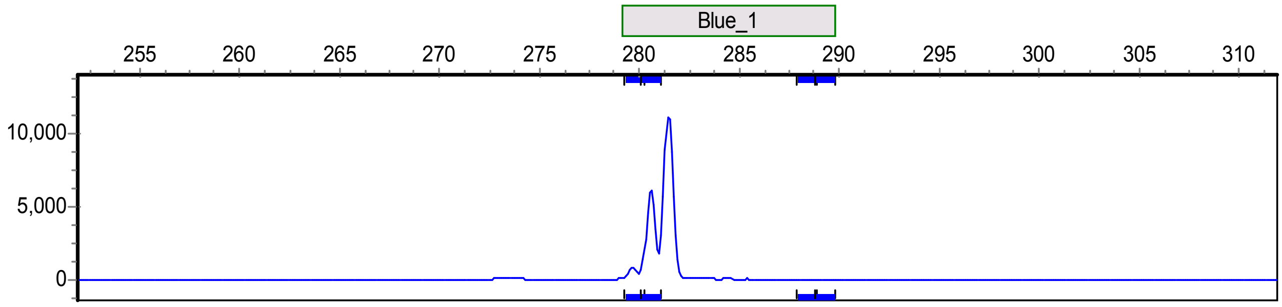

No	Size	Height	Area	Marker	Allele	Difference	Quality	Score	Allele Comments	Sample Comments
1	280.6	6185	31197	Blue_1	281	0.0	Pass	500.0	[<Deleted>]	

Sample 75: Run date and time: 09/15/2020 - 22:42:28 -> 09/15/2020 - 23:28:27

Dye: Blue - 1 peaks - 77.fsa

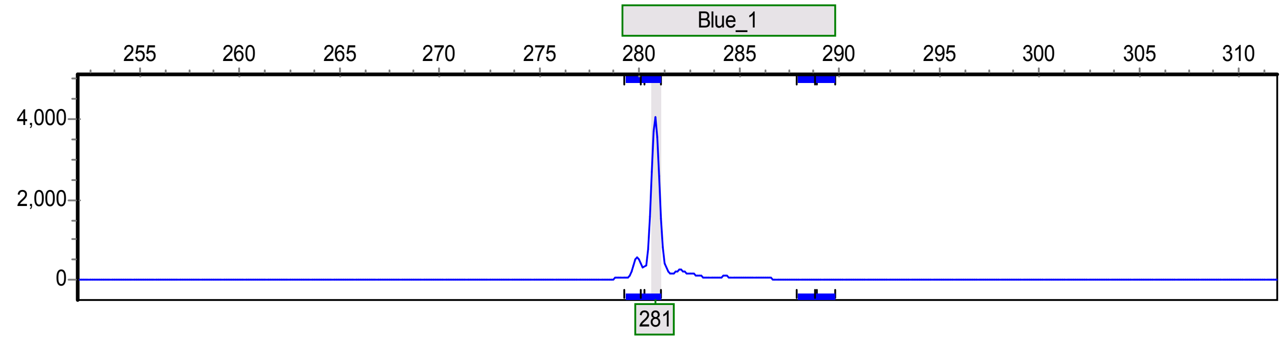

No	Size	Height	Area	Marker	Allele	Difference	Quality	Score	Allele Comments	Sample Comments
1	280.8	4035	20360	Blue_1	281	0.2	Pass	500.0		

Sample 76: Run date and time: 09/15/2020 - 22:42:28 -> 09/15/2020 - 23:28:27

Dye: Blue - 1 peaks - 78.fsa

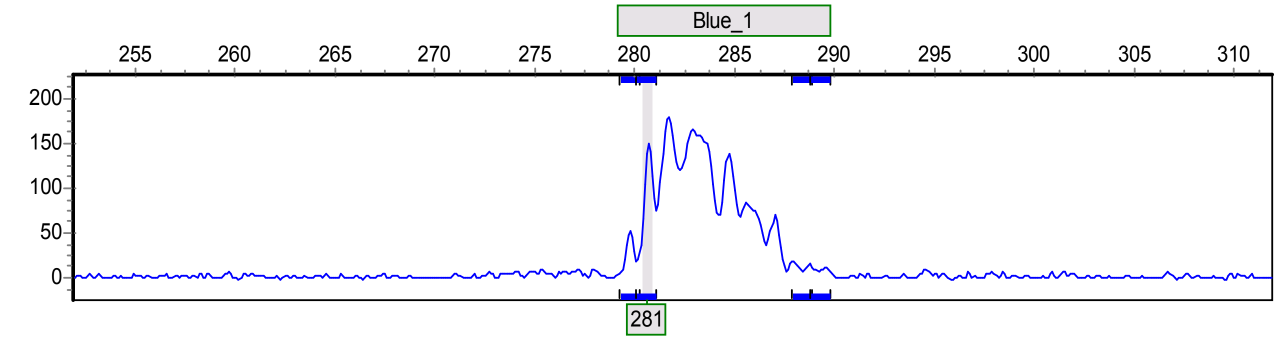

No	Size	Height	Area	Marker	Allele	Difference	Quality	Score	Allele Comments	Sample Comments
1	280.7	150	833	Blue_1	281	0.1	Pass	5.7	[<Confirmed>]	

Sample 77: Run date and time: 09/15/2020 - 22:42:28 -> 09/15/2020 - 23:28:27

Dye: Blue - 2 peaks - 79.fsa

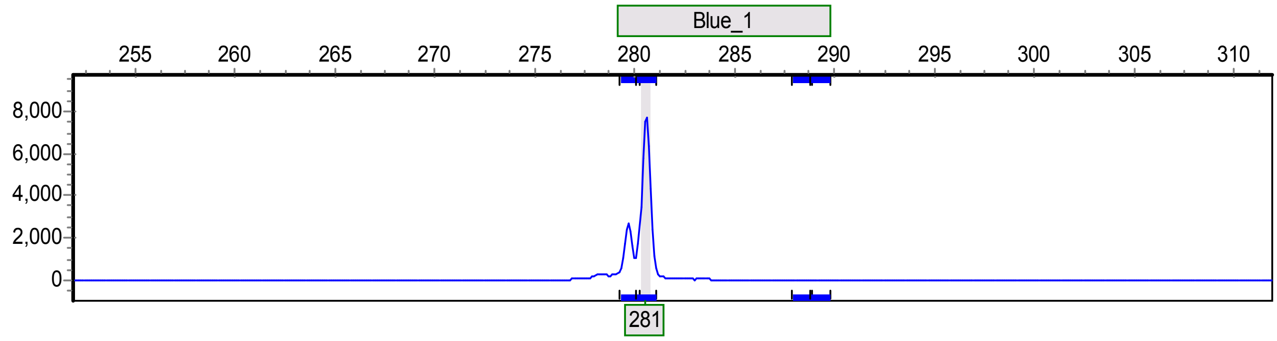

No	Size	Height	Area	Marker	Allele	Difference	Quality	Score	Allele Comments	Sample Comments
1	279.7	2672	13399	Blue_1	280	0.1	Pass	500.0	[<Deleted>]	
2	280.6	7644	38913	Blue_1	281	0.0	Pass	500.0		

Sample 78: Run date and time: 09/15/2020 - 22:42:28 -> 09/15/2020 - 23:28:27

Dye: Blue - 0 peaks - 8.fsa

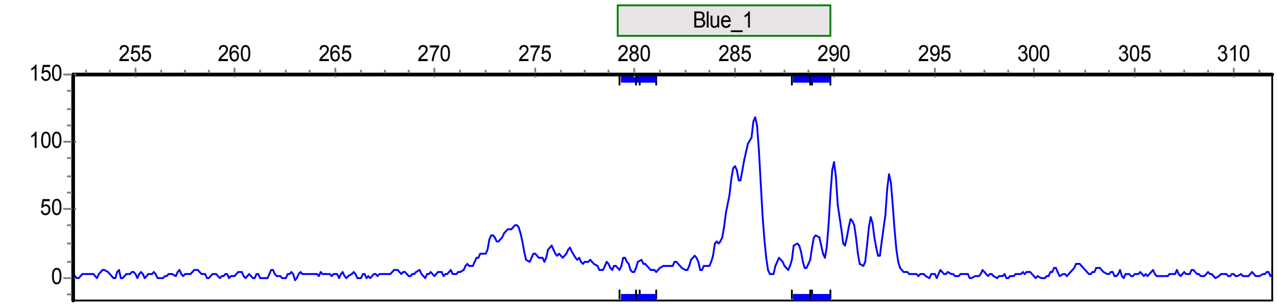

No	Size	Height	Area	Marker	Allele	Difference	Quality	Score	Allele Comments	Sample Comments

Sample 79: Run date and time: 09/15/2020 - 22:42:28 -> 09/15/2020 - 23:28:27

Dye: Blue - 2 peaks - 80.fsa

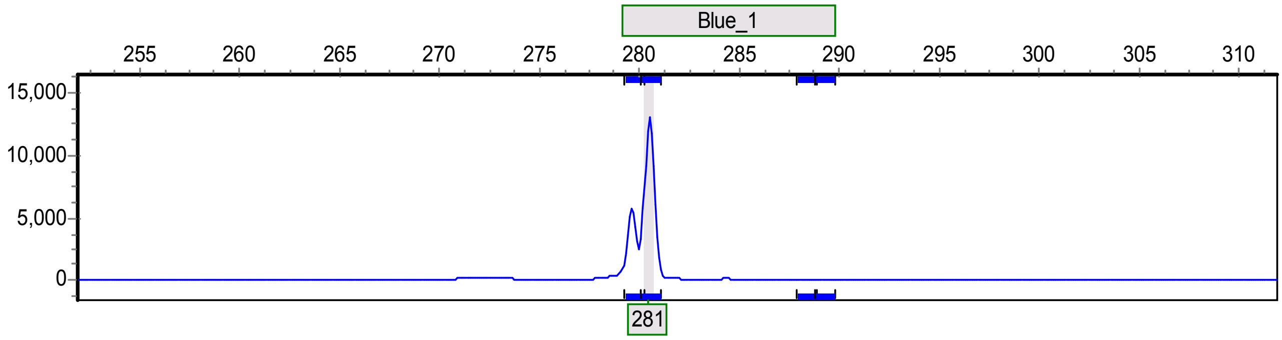

No	Size	Height	Area	Marker	Allele	Difference	Quality	Score	Allele Comments	Sample Comments
1	279.6	5729	32799	Blue_1	280	0.2	Pass	500.0	[<Deleted>]	
2	280.5	13034	74819	Blue_1	281	0.1	Pass	500.0		

Sample 80: Run date and time: 09/15/2020 - 22:42:28 -> 09/15/2020 - 23:28:27

Dye: Blue - 2 peaks - 81.fsa

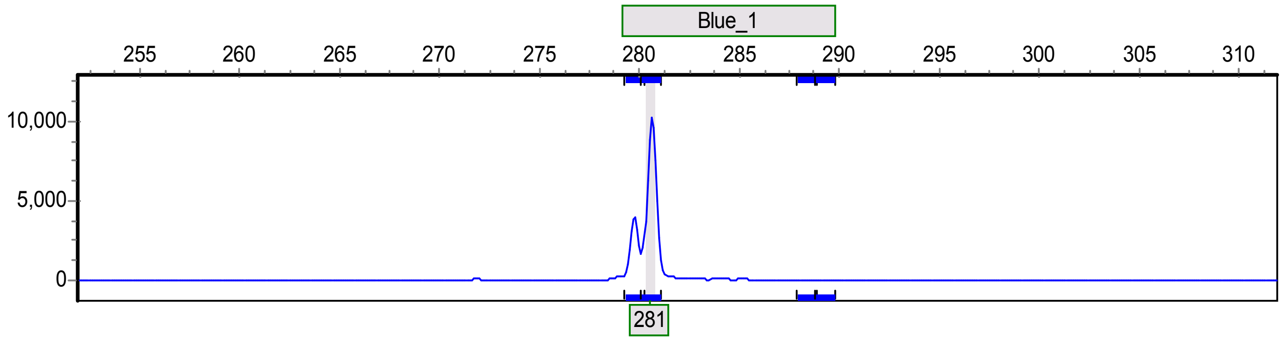

No	Size	Height	Area	Marker	Allele	Difference	Quality	Score	Allele Comments	Sample Comments
1	279.8	3940	21160	Blue_1	280	0.0	Pass	500.0	[<Deleted>]	
2	280.6	10221	54954	Blue_1	281	0.0	Pass	500.0		

Sample 81: Run date and time: 09/15/2020 - 22:42:28 -> 09/15/2020 - 23:28:27

Dye: Blue - 1 peaks - 82.fsa

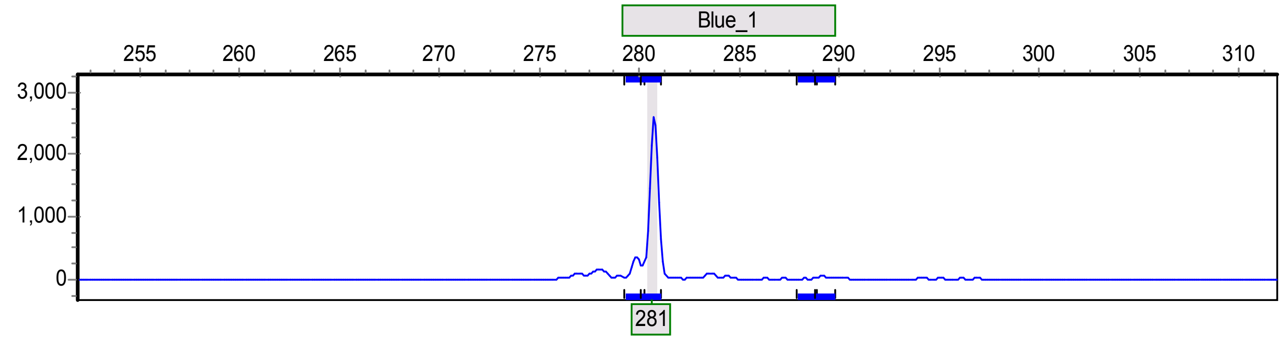

No	Size	Height	Area	Marker	Allele	Difference	Quality	Score	Allele Comments	Sample Comments
1	280.7	2583	13207	Blue_1	281	0.1	Pass	500.0		

Sample 82: Run date and time: 09/15/2020 - 22:42:28 -> 09/15/2020 - 23:28:27

Dye: Blue - 2 peaks - 83.fsa

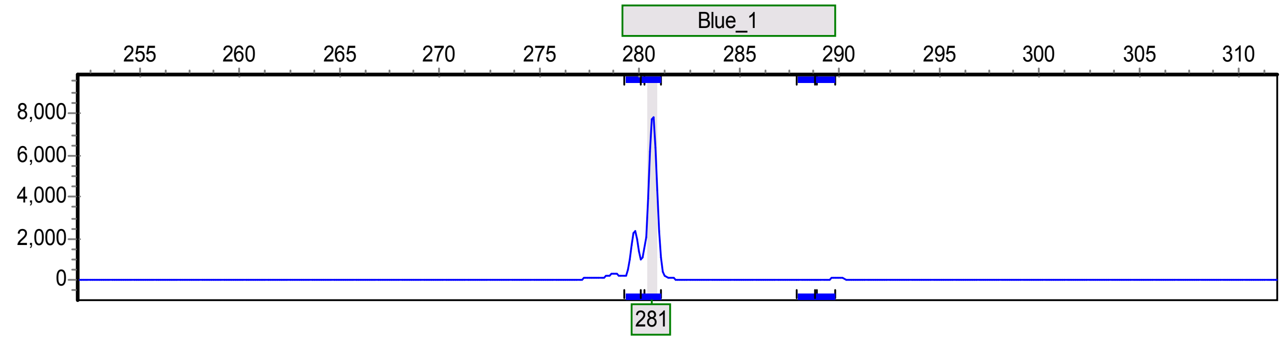

No	Size	Height	Area	Marker	Allele	Difference	Quality	Score	Allele Comments	Sample Comments
1	279.8	2345	11768	Blue_1	280	0.0	Pass	500.0	[<Deleted>]	
2	280.7	7783	40663	Blue_1	281	0.1	Pass	500.0		

Sample 83: Run date and time: 09/15/2020 - 22:42:28 -> 09/15/2020 - 23:28:27

Dye: Blue - 2 peaks - 84.fsa

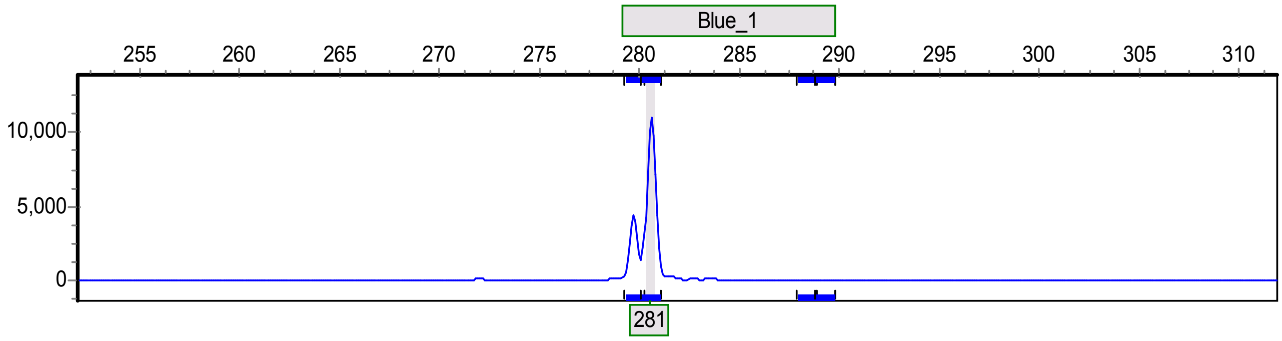

No	Size	Height	Area	Marker	Allele	Difference	Quality	Score	Allele Comments	Sample Comments
1	279.7	4325	20389	Blue_1	280	0.1	Pass	500.0	[<Deleted>]	
2	280.6	10908	55976	Blue_1	281	0.0	Pass	500.0		

Sample 84: Run date and time: 09/15/2020 - 22:42:28 -> 09/15/2020 - 23:28:27

Dye: Blue - 1 peaks - 85.fsa

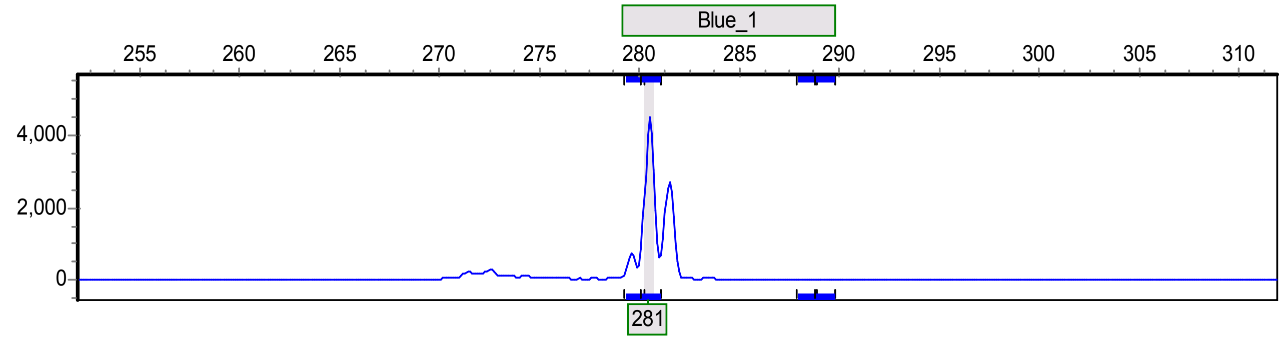

No	Size	Height	Area	Marker	Allele	Difference	Quality	Score	Allele Comments	Sample Comments
1	280.5	4503	23434	Blue_1	281	0.1	Pass	500.0		

Sample 85: Run date and time: 09/15/2020 - 22:42:28 -> 09/15/2020 - 23:28:27

Dye: Blue - 1 peaks - 86.fsa

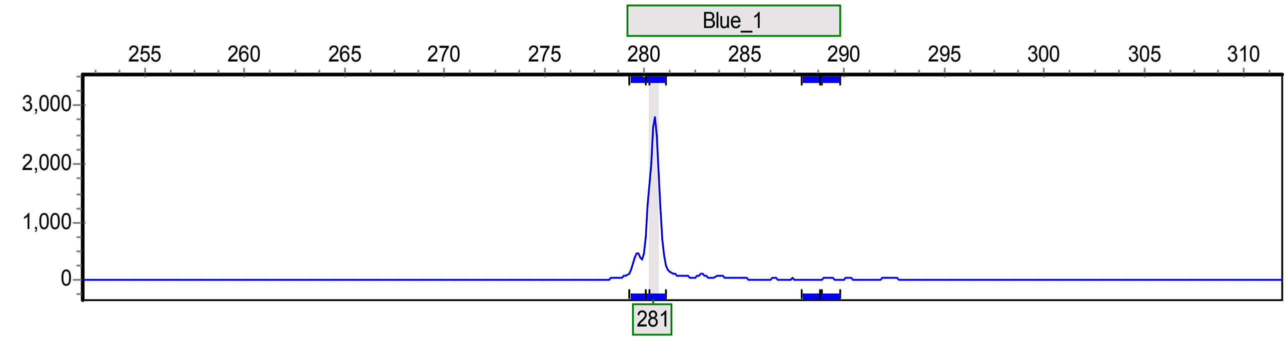

No	Size	Height	Area	Marker	Allele	Difference	Quality	Score	Allele Comments	Sample Comments
1	280.5	2790	15710	Blue_1	281	0.1	Pass	500.0		

Sample 86: Run date and time: 09/15/2020 - 22:42:28 -> 09/15/2020 - 23:28:27

Dye: Blue - 2 peaks - 87.fsa

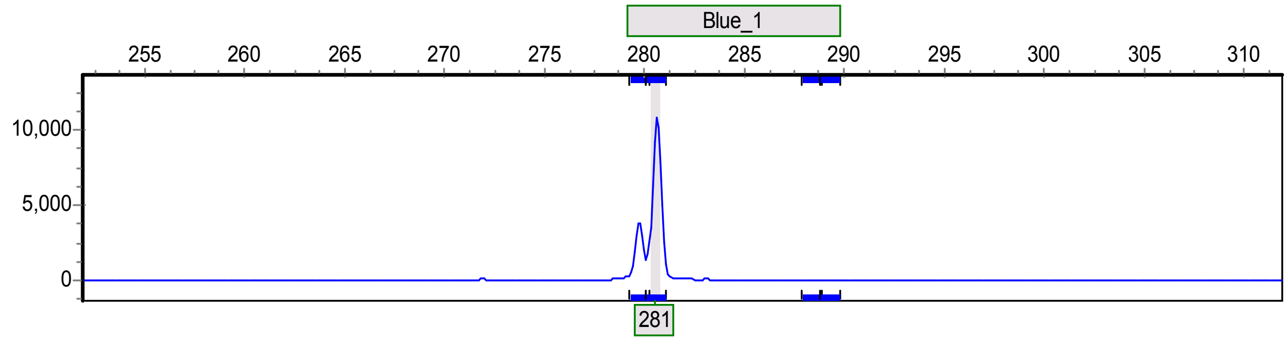

No	Size	Height	Area	Marker	Allele	Difference	Quality	Score	Allele Comments	Sample Comments
1	279.8	3816	19887	Blue_1	280	0.0	Pass	500.0	[<Deleted>]	
2	280.6	10735	55150	Blue_1	281	0.0	Pass	500.0		

Sample 87: Run date and time: 09/15/2020 - 22:42:28 -> 09/15/2020 - 23:28:27

Dye: Blue - 1 peaks - 88.fsa

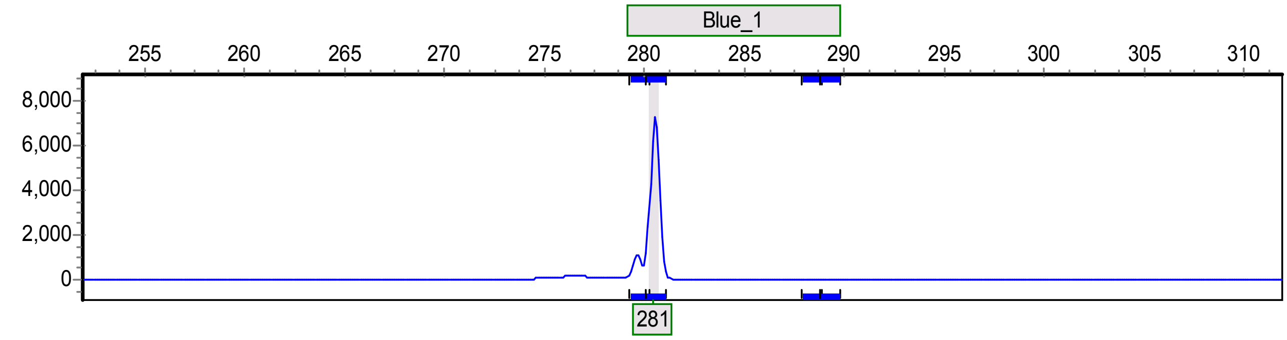

No	Size	Height	Area	Marker	Allele	Difference	Quality	Score	Allele Comments	Sample Comments
1	280.5	7202	37630	Blue_1	281	0.1	Pass	500.0		

Allele Report

Sample 88: Run date and time: 09/15/2020 - 22:42:28 -> 09/15/2020 - 23:28:27

Dye: Blue - 1 peaks - 89.fsa

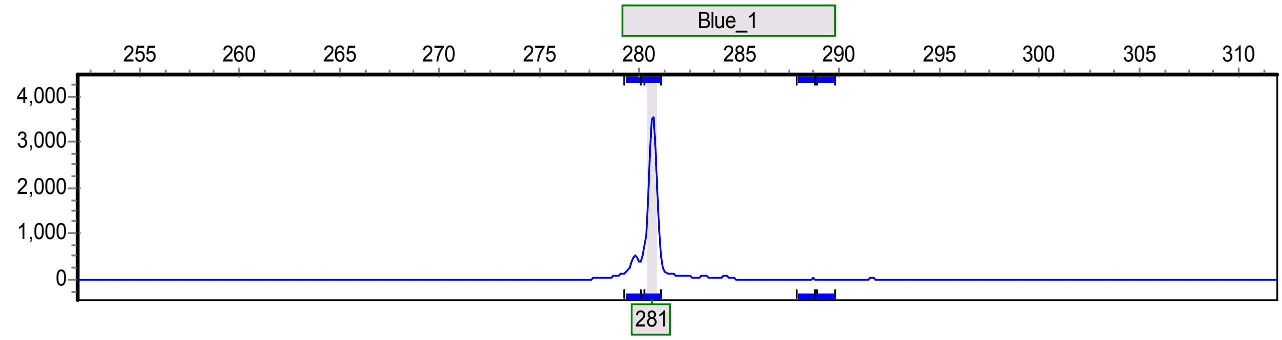

No	Size	Height	Area	Marker	Allele	Difference	Quality	Score	Allele Comments	Sample Comments
1	280.7	3522	18579	Blue_1	281	0.1	Pass	500.0		

Sample 89: Run date and time: 09/15/2020 - 22:42:28 -> 09/15/2020 - 23:28:27

Dye: Blue - 2 peaks - 9.fsa

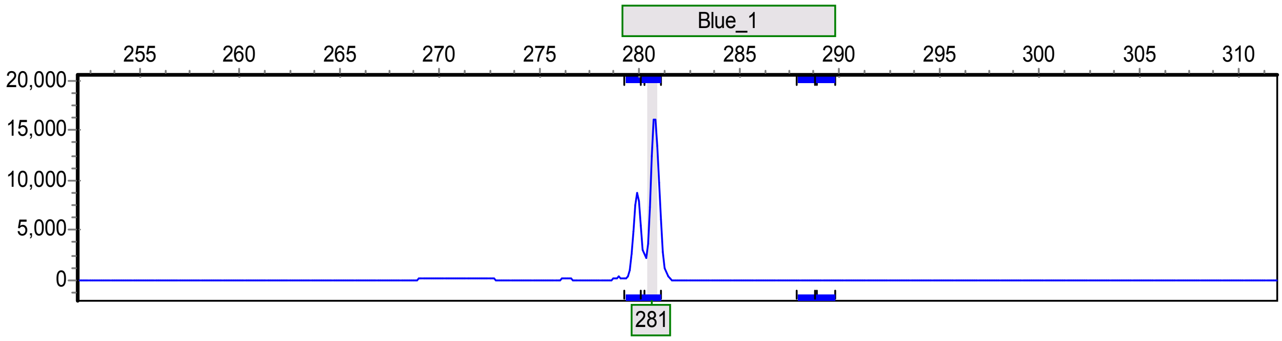

No	Size	Height	Area	Marker	Allele	Difference	Quality	Score	Allele Comments	Sample Comments
1	279.9	8790	40013	Blue_1	280	0.1	Pass	500.0	[<Deleted>]	
2	280.7	16184	85572	Blue_1	281	0.1	Pass	500.0		

Sample 90: Run date and time: 09/15/2020 - 22:42:28 -> 09/15/2020 - 23:28:27

Dye: Blue - 1 peaks - 90.fsa

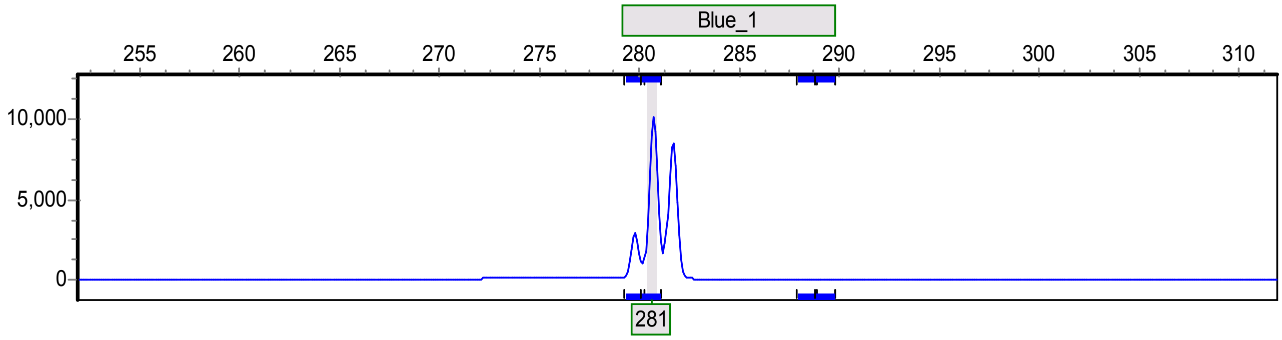

No	Size	Height	Area	Marker	Allele	Difference	Quality	Score	Allele Comments	Sample Comments
1	280.7	10118	52566	Blue_1	281	0.1	Pass	500.0		

Sample 91: Run date and time: 09/15/2020 - 22:42:28 -> 09/15/2020 - 23:28:27

Dye: Blue - 1 peaks - 91.fsa

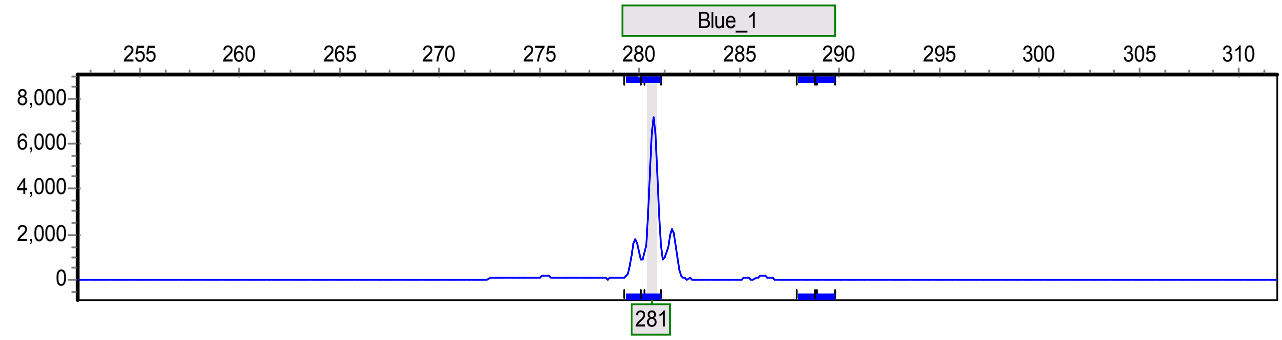

No	Size	Height	Area	Marker	Allele	Difference	Quality	Score	Allele Comments	Sample Comments
1	280.7	7139	37459	Blue_1	281	0.1	Pass	500.0		

Sample 92: Run date and time: 09/15/2020 - 22:42:28 -> 09/15/2020 - 23:28:27

Dye: Blue - 1 peaks - 92.fsa

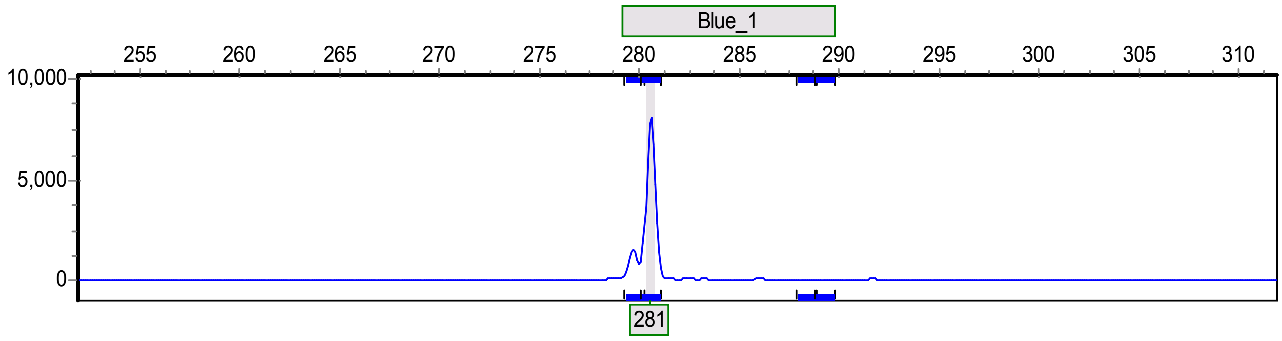

No	Size	Height	Area	Marker	Allele	Difference	Quality	Score	Allele Comments	Sample Comments
1	280.6	8040	41764	Blue_1	281	0.0	Pass	500.0		

Sample 93: Run date and time: 09/15/2020 - 22:42:28 -> 09/15/2020 - 23:28:27

Dye: Blue - 2 peaks - 93.fsa

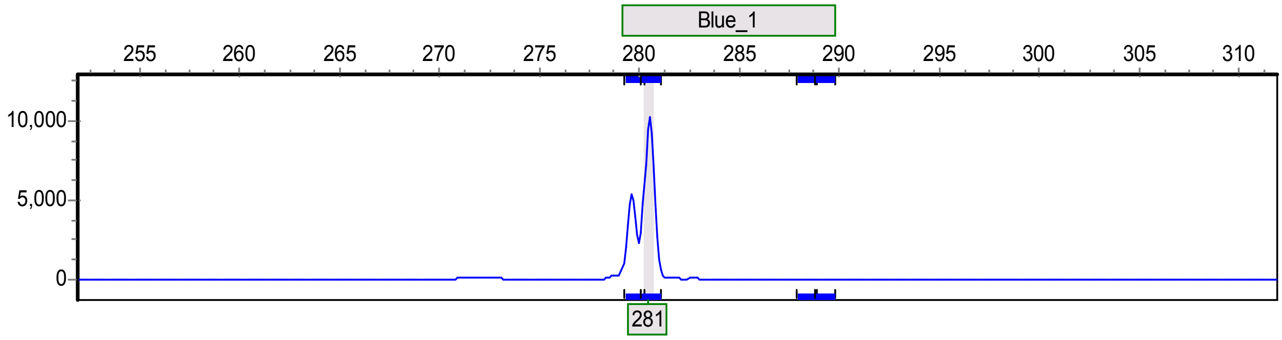

No	Size	Height	Area	Marker	Allele	Difference	Quality	Score	Allele Comments	Sample Comments
1	279.6	5355	30223	Blue_1	280	0.2	Pass	500.0	[<Deleted>]	
2	280.5	10139	57612	Blue_1	281	0.1	Pass	500.0		

Sample 94: Run date and time: 09/15/2020 - 22:42:28 -> 09/15/2020 - 23:28:27

Dye: Blue - 1 peaks - 94.fsa

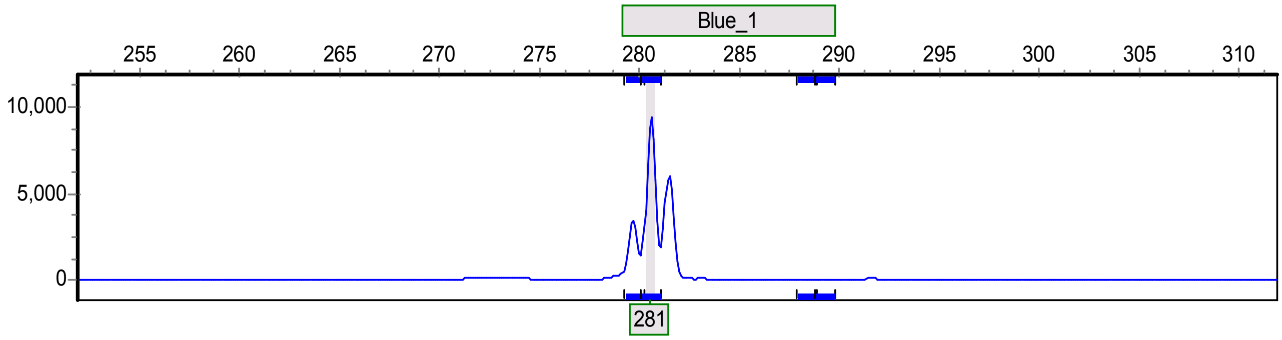

No	Size	Height	Area	Marker	Allele	Difference	Quality	Score	Allele Comments	Sample Comments
1	280.6	9372	50857	Blue_1	281	0.0	Pass	500.0		

Sample 95: Run date and time: 09/15/2020 - 22:42:28 -> 09/15/2020 - 23:28:27

Dye: Blue - 1 peaks - 95.fsa

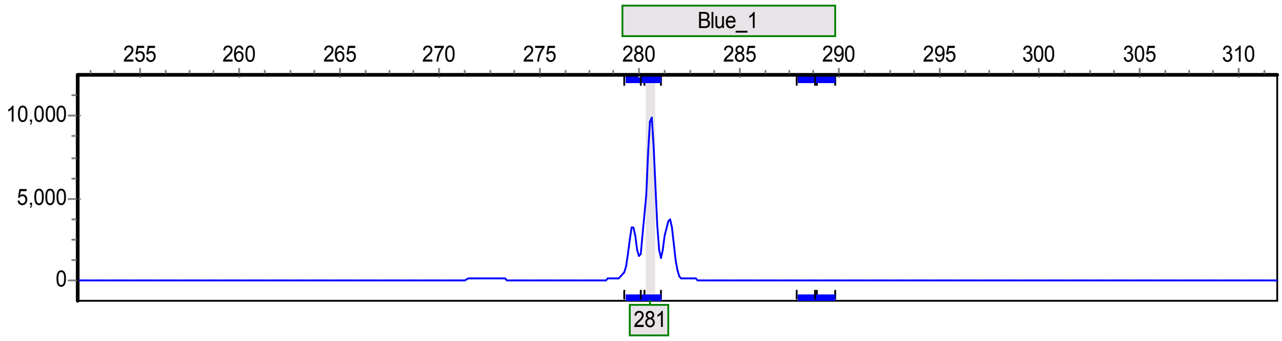

No	Size	Height	Area	Marker	Allele	Difference	Quality	Score	Allele Comments	Sample Comments
1	280.6	9820	53672	Blue_1	281	0.0	Pass	500.0		

Sample 96: Run date and time: 09/15/2020 - 22:42:28 -> 09/15/2020 - 23:28:27

Dye: Blue - 1 peaks - 96.fsa

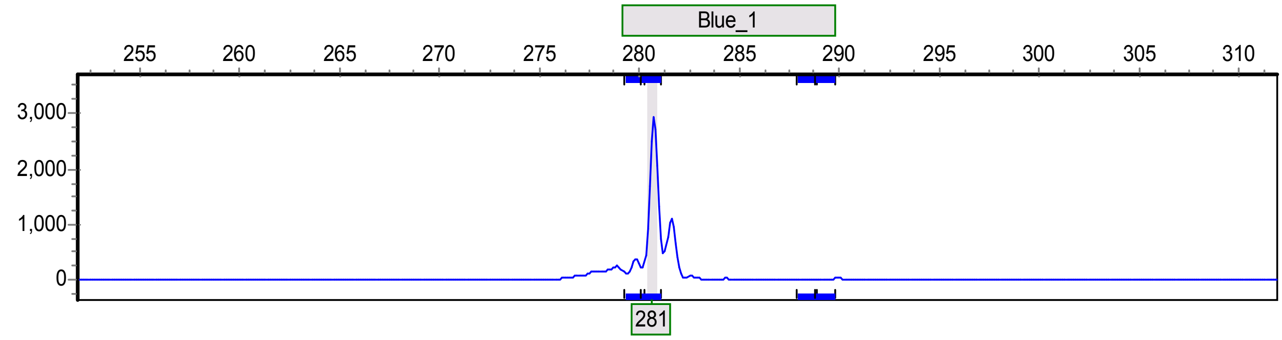

No	Size	Height	Area	Marker	Allele	Difference	Quality	Score	Allele Comments	Sample Comments
1	280.7	2927	15003	Blue_1	281	0.1	Pass	500.0		
